# Overexpression of *AtSHN1/WIN1* Provokes Unique Defense Responses

**DOI:** 10.1371/journal.pone.0070146

**Published:** 2013-07-29

**Authors:** Dikla Sela, Kobi Buxdorf, Jian Xin Shi, Ester Feldmesser, Lukas Schreiber, Asaph Aharoni, Maggie Levy

**Affiliations:** 1 Department of Plant Pathology and Microbiology, The Robert H. Smith Faculty of Agriculture, Food and Environment, The Hebrew University of Jerusalem, Rehovot, Israel; 2 Department of Plant Sciences and Bioinformatics Unit, Weizmann Institute of Science, Rehovot, Israel; 3 Institute of Cellular and Molecular Botany (IZMB), Department of Ecophysiology, Kirschallee, University of Bonn, Bonn, Germany; 4 National Center for Molecular Characterization of Genetically Modified Organisms, School of Life Science and Biotechnology, Shanghai Jiao Tong University, Shanghai, China; Volcani Center, Israel

## Abstract

The plant cell cuticle serves as the first barrier protecting plants from mechanical injury and invading pathogens. The cuticle can be breached by cutinase-producing pathogens and the degradation products may activate pathogenesis signals in the invading pathogens. Cuticle degradation products may also trigger the plant’s defense responses. *Botrytis cinerea* is an important plant pathogen, capable of attacking and causing disease in a wide range of plant species. *Arabidopsis thaliana shn1-1D* is a gain-of-function mutant, which has a modified cuticular lipid composition. We used this mutant to examine the effect of altering the whole-cuticle metabolic pathway on plant responses to *B. cinerea* attack. Following infection with *B. cinerea*, the *shn1-1D* mutant discolored more quickly, accumulated more H_2_O_2_, and showed accelerated cell death relative to wild-type (WT) plants. Whole transcriptome analysis of *B. cinerea*-inoculated *shn1-1D* vs. WT plants revealed marked upregulation of genes associated with senescence, oxidative stress and defense responses on the one hand, and genes involved in the magnitude of defense-response control on the other. We propose that altered cutin monomer content and composition of *shn1-1D* plants triggers excessive reactive oxygen species accumulation and release which leads to a strong, unique and uncontrollable defense response, resulting in plant sensitivity and death.

## Introduction

Plants encounter a wide range of pathogens and insects in their natural environment. Some of these are responsible for annual worldwide economic damage due to losses in important agricultural crops. Throughout their coevolution with pathogens, plants have developed both physical barriers and physiological responses, which aid them in coping with pathogen attacks. The first barrier protecting plants from pathogens is a physical one–the cuticle, which is defined as a noncellular waxy structure that covers the epidermal cells. The cuticular layer covers all of the aerial organs and plays multiple roles in plants, including regulation of epidermal permeability and nonstomatal water loss, and protection against insects, pathogens, UV light and frost [1]. The second barrier protecting plants from pathogenic attack is a set of biochemical reactions, which lead to hypersensitive and acquired immune responses. These constitutive and inducible defense events depend largely on the perception of signaling molecules [2]–[6], some of which can be activated by cuticle-degradation products.

It is generally accepted that the cuticle’s mechanical strength is provided by the cutin matrix, a polymer formed by three-dimensional crosslinking of covalent bonds [7]. Accordingly, it is assumed that one of the cuticle’s functions is to protect the plant surface from possible external mechanical damage caused by biting insects or growing fungal hyphae. Nevertheless, there is no conclusive evidence correlating cuticle thickness with plant resistance to different pathogens [8], [9]. In addition to the cuticle’s role as a physical barrier, there is growing evidence that its constituents may also act as pathogenesis signals for the invading pathogens and as triggers for the plant’s defense responses [10]–[14]. The cuticle has also been recently suggested to play an active role in systemic acquired resistance-related molecular signaling [15]. The precise set of events activated by cuticle components and degradation products in infected plant cells is still widely unexplored.

Since the cuticle serves as one of the first lines of defense against invading pathogens, its permeability may affect plant resistance. Indeed, cutinase-overexpressing transgenic *Arabidopsis* plants (designated CUTE plants) and various *Arabidopsis* mutants altered in key enzymes for cuticle formation and structure [e.g., *bodyguard* (*bdg*) and *long-chain-acyl-CoA* (*lacs2*)], have all been shown to possess full immunity to some necrotrophic pathogens but not others [16], [17]. This was attributed to possible defects in the integrity of their cuticular layer, which led to high permeability of their cuticle and to the release of fungitoxic compounds? [16]. The link between cuticle permeability and resistance to necrotrophic pathogens was further supported by observations in the *Arabidopsis* mutant *lacerate* (*lcr*). This mutant, with intermediate permeability as compared to *lacs2*, showed intermediate resistance to the necrotrophic fungal pathogen *Botrytis cinerea*, whereas the *hothead* (*hth*) mutant, with lower cuticle permeability, was as susceptible to *B. cinerea* as the wild type (WT) [18]. Cuticle permeability was also linked to accumulation of reactive oxygen species (ROS), *B. cinerea* resistance and induction of innate immunity [19], yet the recently characterized *resurrection 1*(*rst1*) mutant, which has elevated levels of cuticular lipids but normal cuticular permeability, exhibits enhanced resistance to *B. cinerea* but enhanced susceptibility to the biotrophic fungus *Erysiphe cichoracearum* [20]. The observed differences in the effects of permeable or altered cuticles on pathogenesis by diverse fungal pathogens suggest that other, yet to be discovered mechanisms may be involved in triggering the plant response and plant-induced resistance during cuticular disruption by invading pathogens.

SHINE1/WAX INDUCER1 (SHN1/WIN1) is a member of a clade of three proteins belonging to the plant-specific family of AP2/EREBP transcription factors. It is the first reported transcription factor to regulate surface lipid metabolism in plants [21]–[23]. Overexpression of all three *SHINE* clade members (*SHN1, SHN2* and *SHN*3) confers a typical brilliant, shiny green leaf surface phenotype similar to that of the original activation-tagged gain-of-function mutant *shine* (*shn1-1D*) [21], [22]. Biochemical analysis revealed that *shn1*-, *shn2-* and *shn3*-overexpressing plants, and the *shn1-1D* gain-of-function mutant, are all altered in wax and cutin composition [21], [22]. More recently, it was shown that along with its control of cuticular lipid metabolism, SHN1 modifies the epidermal cell wall by altering pectin metabolism and structural proteins [24].

To further investigate the cuticular role in plant–fungal interactions, we performed a genome-wide analysis of the defense response of *shn1-1D* following infection with *B. cinerea*. We discovered that *shn1-1D* plants generate excess ROS and exhibit strong activation of defense responses, yet these plants were more susceptible to the necrotrophic fungus *B. cinerea* than the WT. We propose that *shn1-1D* plants exhibit accelerated ROS generation, which leads to overstimulated activation of genes involved in the defense response that cannot be controlled, resulting in plant sensitivity and death.

## Results

### *shn1-1D* Plants are More Susceptible to *B. cinerea* than Wild-type Plants

**Figure 1:**
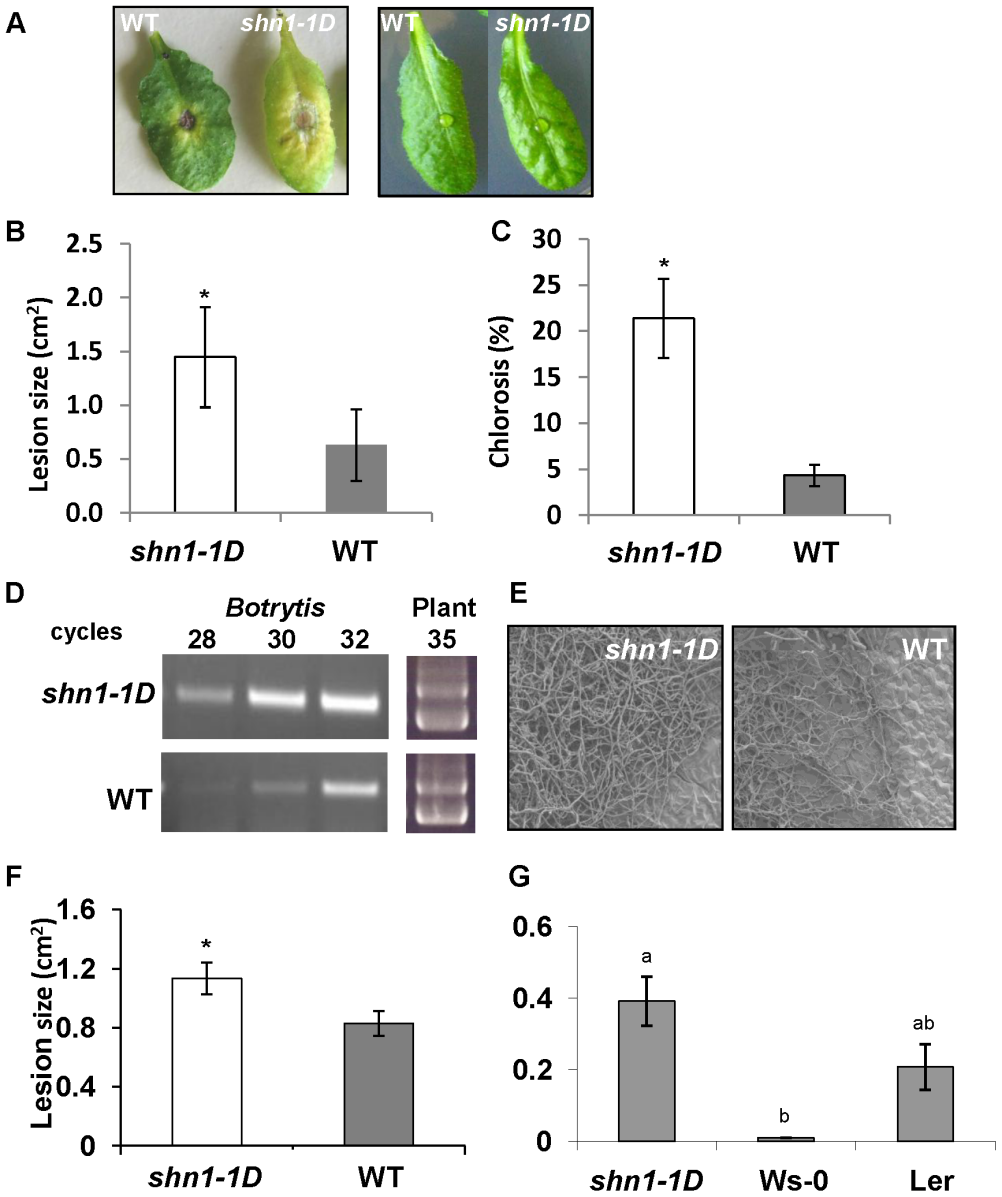
Disease symptoms on *shn1-1D* and WT leaves. **A,** Infected leaves 72 h post-inoculation with *B. cinerea* (left) and mock-treated leaves (right). **B,** Expanding lesion size 72 h post-inoculation. **C,** Chlorosis percentage 72 h post-inoculation. Bars represent mean±SD of 15 leaves. Asterisks denote significant differences (*P*<0.05) as determined by Student’s t-test. **D,** Quantification of fungal DNA from infected leaves using semi-quantitative PCR with β-tubulin primers of *B. cinerea* and *Arabidopsis* as a control. **E,** SEM of *shn1–1D* and WT leaves demonstrating *B. cinerea* hyphal density 72 h post-inoculation. **F,** Expanding lesion size 48 h post-inoculation with *S. sclerotiorum*. **G,** Expanding lesion size 144 h post-inoculation with *A. brassicicola*. Bars represent mean±SD of 15–18 leaves. Asterisks denote significant differences (*P*<0.05) as determined by Student’s t-test, different letters denote significant differences (*P*<0.05) as determined by Kruskal-Wallis ANOVA, Dunn’s Method.

**Figure 2:**
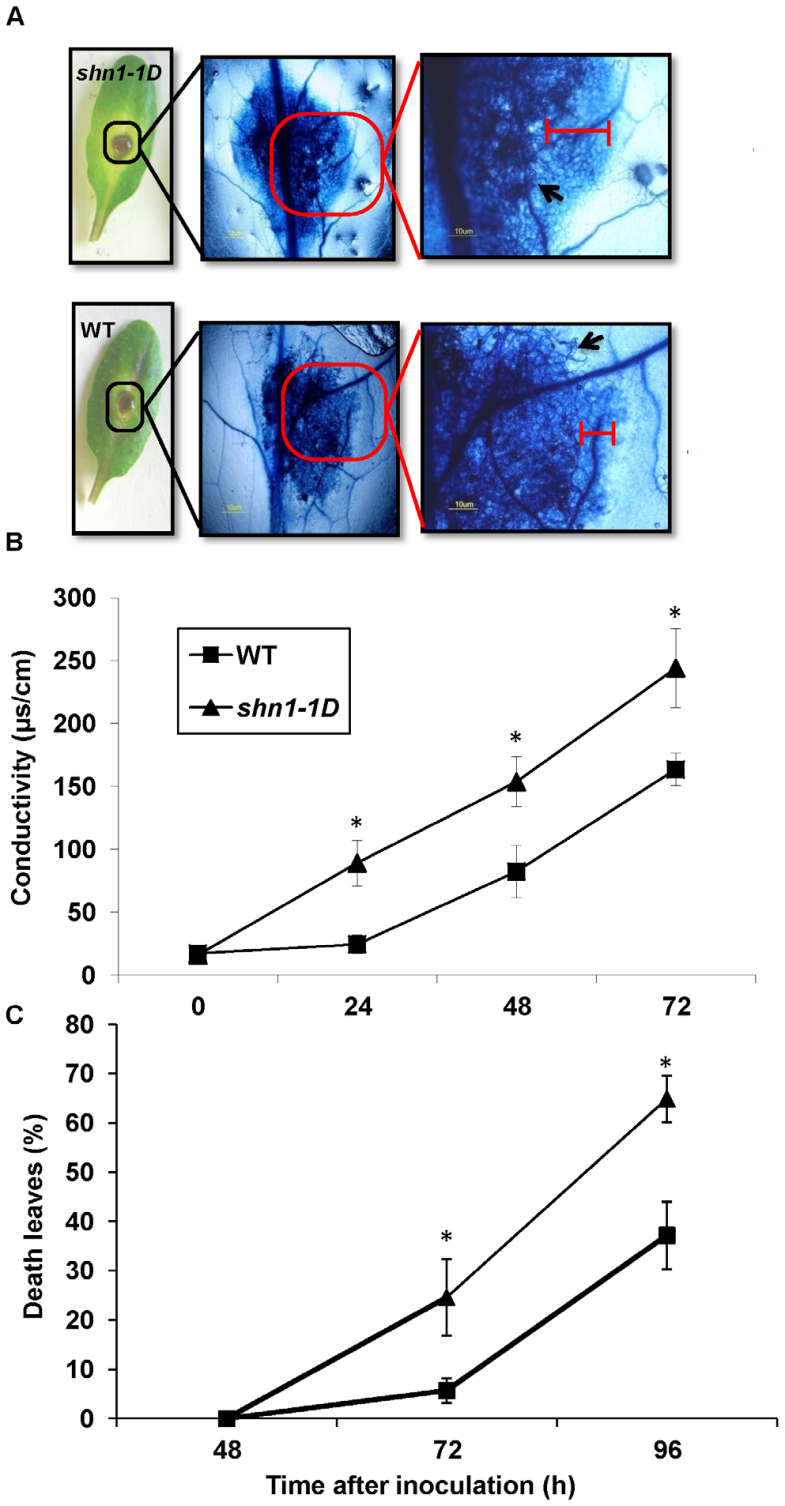
Cell death on *shn1-1D* and WT leaves. **A,** **** Infected leaves 72 h post-inoculation with *B. cinerea* (left). Infected leaves were stained with trypan blue: lesion area at 50X magnification (middle) and 100X magnification (right). Hyphae are marked with a black arrow, dead cell area is marked with a red line (scale bars: 10 µm). **B,** Ion leakage from *shn1–1D* and WT leaves. Electrical conductivity (EC) was measured (µS/cm) 0–72 h after inoculation with *B. cinerea*. Means±SD calculated from 10 leaves. Asterisks denote significant differences (*P*<0.05) between samples by Welch ANOVA test for each time point. **C,** Death progression in whole plants inoculated with *B. cinerea*. Percentage of dead leaves (leaves fully covered with lesions) per plant was calculated during disease development up to 96 h post-inoculation with *B. cinerea*. Means±SD of 10 plants are presented. Asterisk denotes statistical difference between WT and *shn1–1D* plants calculated for the specified time point by Student’s t-test (*P*<0.05). Shown is one representative experiment out of at least three more experiments with similar results.

Cuticular modifications have been shown to affect plant response to pathogen infection [2], [17]. We tested the response of *shn1-1D*, an activation-tagged gain-of-function *shine* mutant, which displays modified cuticle structure and composition [21], to *B. cinerea* infection. Inoculation of WT *Arabidopsis* leaves with a conidial suspension of *B. cinerea* resulted in disease symptoms 24 to 48 h post-inoculation, which appeared as necrotic spots under the inoculation droplets; 72 to 96 h post-inoculation, the size of the spots got larger, becoming water-soaked lesions (**Figure 1A**). Inoculation of *shn1-1D* leaves with *B. cinerea* lead to similar disease symptoms (**Figure 1A**), yet the lesions developed on *shn1-1D* leaves were 35 to 45% larger than those developed on infected WT leaves (**Figure 1B**). Furthermore, lesions on infected *shn1-1D* leaves exhibited larger chlorotic areas than those on infected WT leaves (**Figure 1C**). PCR and scanning electron microscopy (SEM) analyses of inoculated leaves revealed elevated levels of fungal DNA (**Figure 1D**) and denser fungal mycelium, respectively, in the lesion area (**Figure 1E**) of *shn1-1D* leaves, as compared to WT leaves. We further analyzed the spread of *B. cinerea*’s hyphae and plant cell viability in the chlorotic areas of infected leaves by vital stain using trypan blue. We did not observe any hyphae outside the expanding lesion, but a larger number of dead cells were identified around the lesions in inoculated *shn1-1D* leaves than in WT leaves (**Figure 2A**). The increased number of dead cells in *shn1-1D* leaves was further confirmed by ion-leakage analysis of cells located around the lesion; this revealed significantly higher ion leakage in *shn1-1D* vs. WT cells (**Figure 2B**). In addition, we observed that *shn1-1D* plants senesce and die faster than WT plants following inoculation with *B. cinerea*, as determined by counting the number of dead leaves 9 up to 96 h post-inoculation (**Figure 2C**).

We next tested the susceptibility of *shn1-1D* to other necrotrophic fungi. We analyzed the lesion size on infected leaves 48 and 144 h post-inoculation with *Sclerotinia sclerotiorum* (**Figure 1F**) and *Alternaria brassicicola* (**Figure 1G**), respectively. Our data showed that *shn1–1D* plants are generally more susceptible to necrotrophic pathogens than WT plants. Worth noting is that inoculation with *A. brassicicola*, which is a crucifer specialist that is usually not very aggressive on ecotype Ws-0 (the corresponding background of *shn1–1D*), resulted in quicker cell discoloration and death in *shn1–1D* vs. Ws-0 WT plants, similar to the susceptible ecotype Ler (**Figure 1G**).

### *shn1-1D* Plants Produce More H_2_O_2_ in Response to *B. cinerea* Infection and Abiotic Stress

**Figure 3:**
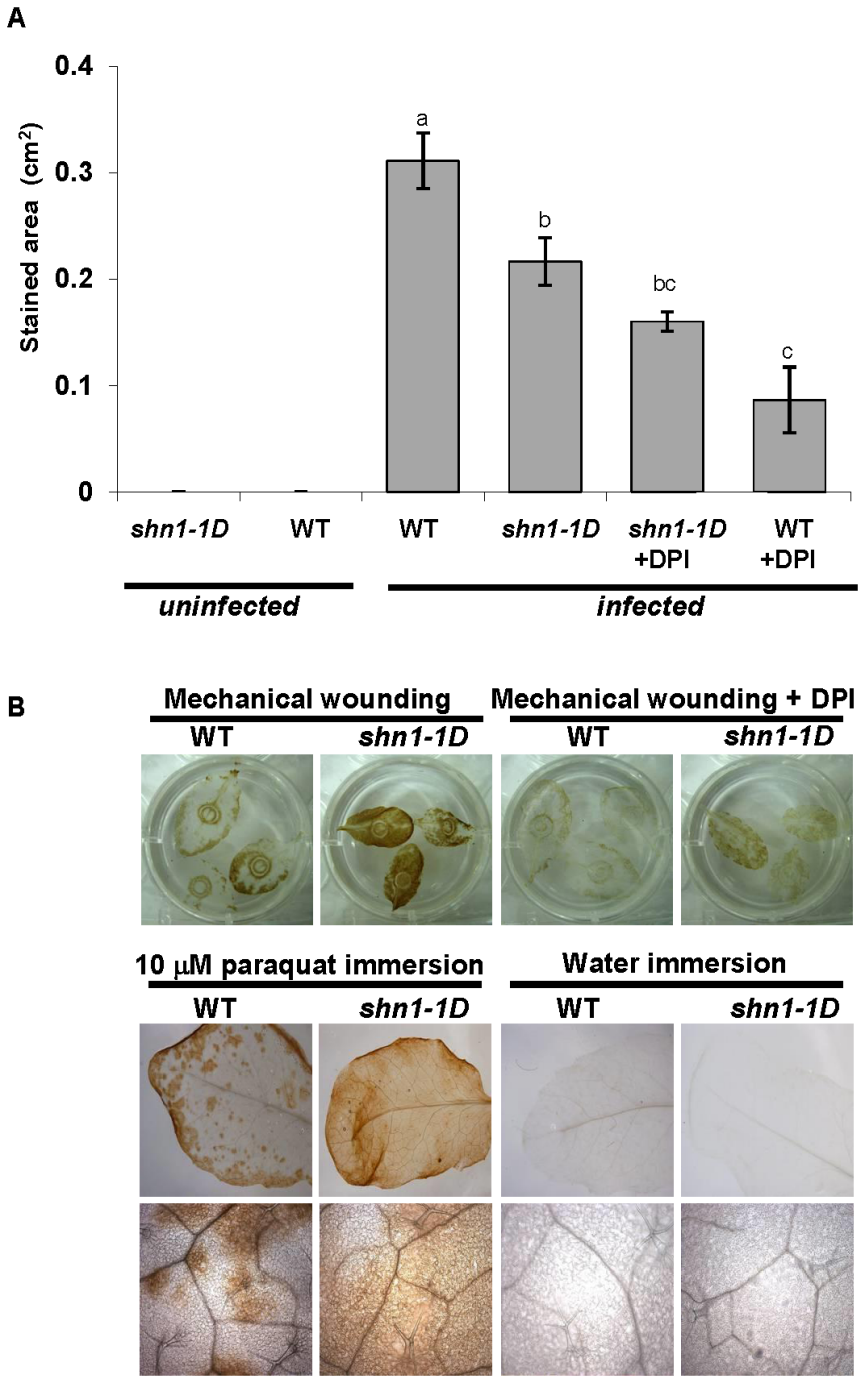
ROS accumulation in *shn1-1D*. **A,** **** ROS accumulation following biotic stress. H_2_O_2_ production was measured by staining *shn1–1D* and WT leaves with DAB 72 h post-inoculation with *B. cinerea*, and quantifying the DAB-stained area. Bars represent means±SD of 15 leaves. Different letters represent significant difference by Tukey-Kramer HSD test (*P*<0.0001). Shown is one representative experiment out of at least three more experiments with similar results. **B,** ROS accumulation following abiotic stress. H_2_O_2_ production was measured by staining *shn1–1D* and WT leaves with DAB after mechanical wounding; 100 µM DPI was added to control leaves (upper panel), and 8 h after immersion in 10 µM paraquat or water as a control (lower panel).

ROS production, which can be triggered by many signals, including cutin, is one of the plant’s defense responses against biotic and abiotic stresses [13], [14]. We tested ROS accumulation by measuring H_2_O_2_ accumulation using DAB staining of *shn1–1D* and WT plants in response to *B. cinerea* infection. **Figure 3A** shows that*shn1-1D* plants exhibited significantly higher levels of H_2_O_2_ following *B. cinerea* infection than the WT plants. *shn1–1D* plants also accumulated significantly more H_2_O_2_ following various abiotic stresses, which included mechanical wounding (**Figure 3B**) and paraquat treatment, irrespective of the treatment method (foliage immersion, filtration, spraying or drop application) (**Figure 3B** and **Figure S1A**). Note that with the paraquat treatment, higher H_2_O_2_ production in *shn1–1D* plants was correlated with their higher death rate: when 25 µM paraquat solution was sprayed on the foliage, 36.1% of the *shn1–1D* plants died 1 week after spraying, in contrast to only 0.48% of the WT plants. These results indicate that *shn1–1D* mutants produce more active H_2_O_2_ against various adverse environmental stresses, including *B. cinerea* infection. This might have elicited the observed massive cell death which, in turn, might have contributed to *shn1–1D* sensitivity to the necrotrophic fungus *B. cinerea*. Indeed, when diphenyleneiodonium (DPI), an inhibitor of NADP(H) oxidase, was added to the *B. cinerea* inoculation suspension, we observed inhibition of infection on both *shn1-1D* and WT leaves (**Figure S1B)**.

### Cell-death Induction in *shn1-1D* Plants does not Support *P. syringae* Proliferation

**Figure 4:**
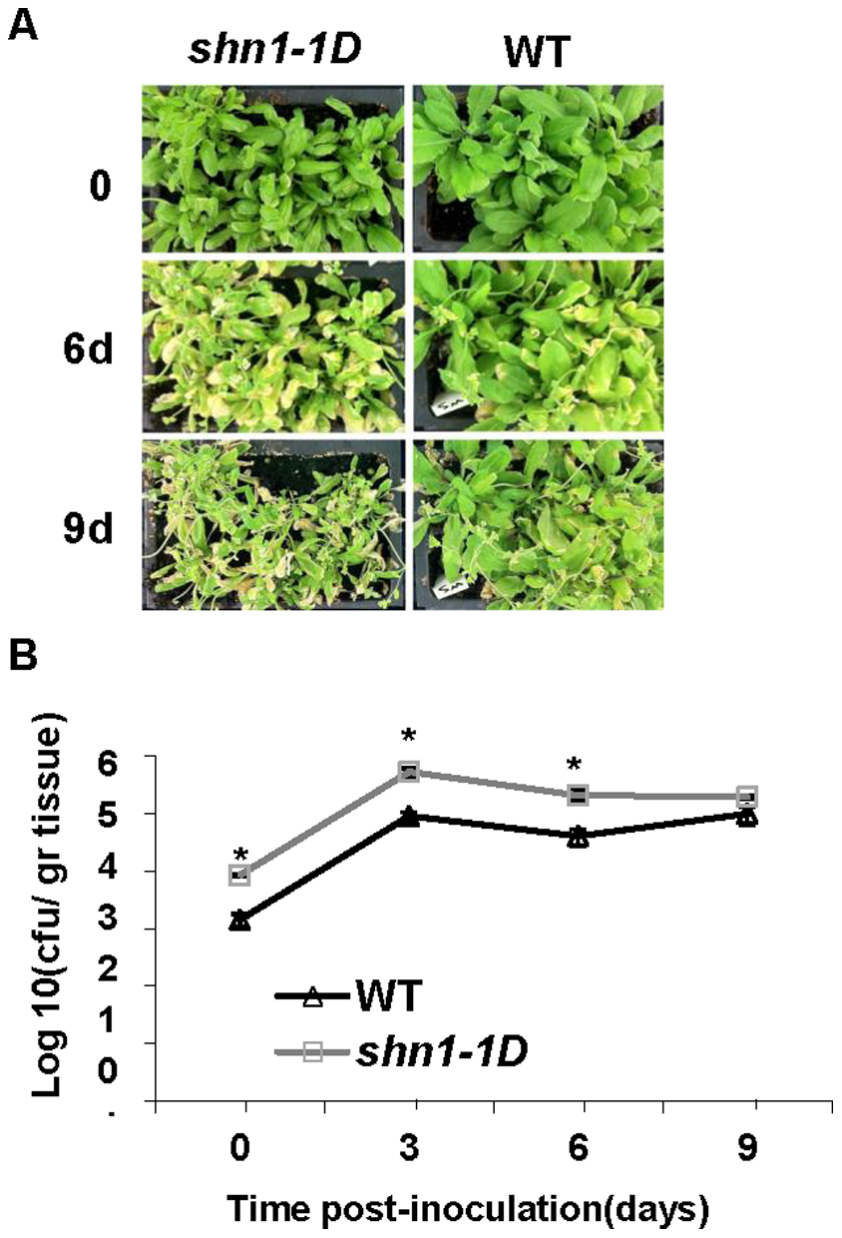
Bacterial proliferation on *shn1-1D* and WT plants. **A,** **** Infection phenotypes of representative Ws-0 wild-type and *shn1–1D* mutant plants at 0–9 days post-inoculation with *P. syringae* pv. *tomato* DC3000. **B,** Quantitative analysis of bacterial growth in WT and *shn1–1D* mutant plants is presented. Results represent means±SE (n = 6). Asterisk denotes statistical difference between WT and *shn1–1D* plants calculated for the specified time point by Student’s t-test (*P*<0.05).

Whereas massive cell death supports the growth of necrotrophs, it is expected to restrict the proliferation of biotrophic pathogens [25]. We examined the proliferation of the biotroph *P. syringae* pv. *tomato* DC3000 in *shn1–1D* plants relative to WT plants. We spray-inoculated leaves with virulent *P. syringae,* and extracted and counted colony-forming units 2 h (0) and 3, 6 and 9 days post-inoculation. Interestingly, while both *shn1–1D* and WT plants were inoculated with the same concentration of bacterial suspension, *shn1–1D* plants carried more bacterial colony-forming units at the time of infection (2 h post-inoculation) and later on at 3 and 6 days post-inoculation, most likely due to their permeable cuticle. However, 9 days post-inoculation, the number of *P. syringae* colony-forming units in the *shn1–1D* plants was similar to that in the WT plants (**Figure 4B**)**.** Nevertheless, *shn1–1D* plants exhibited a more chlorotic phenotype than the WT plants (**Figure 4A**). Similarly, no differences in the number of bacterial colony-forming units were observed between *shn1–1D* and WT leaves, which were spray-inoculated with *Xanthomonas campestris* pv. *campestris* (**Figure S2A**), yet the *shn1–1D* plants were chlorotic as compared to the WT (**Figure S2B**). Collectively, our data suggest that accelerated cell death in *shn1–1D* may contribute to high sensitivity to necrotrophic pathogens such as *B. cinerea*, *S. sclerotiorum* and *A. brassicicola* (**Figure 1**).

### *shn1-1D* Activates Defense and Redox-related Genes in Response to *B. cinerea* Infection

**Figure 5:**
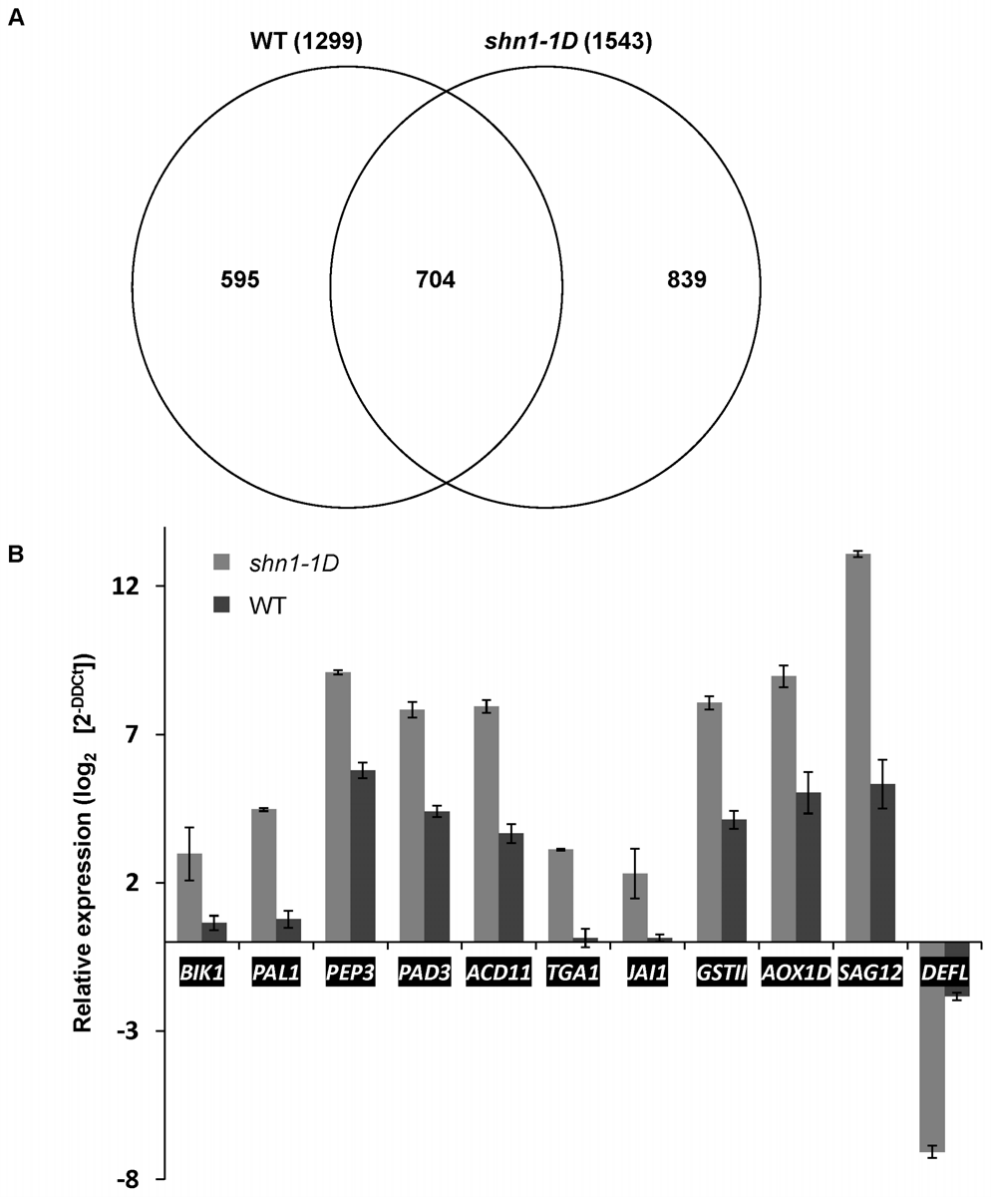
Differential gene regulation by *B. cinerea* in *shn1-1D* and WT. **A,** Venn diagram representing overlapping or non-overlapping gene sets differentially expressed in WT or *shn1–1D* plants 72 h after infection with *B. cinerea* and defined by FC >2 (*P*<0.05). **B,** Relative gene expression between inoculated and noninoculated *shn1–1D* and WT plants. Expression of selected genes from microarray data validated using qRT-PCR on cDNA extracted from *shn1–1D* or WT leaves 72 h after inoculation with *B. cinerea* relative to noninoculated leaves (mock).

For a comprehensive view of the transcriptomic changes in *shn1–1D* plants following infection with *B. cinerea,* their gene expression was analyzed using the Affymetrix ATH1 genome array and Partek statistical package. *shn1–1D* and WT leaves were harvested from mock-inoculated plants and from *B. cinerea*-inoculated plants for the microarray analysis. Our analysis revealed that 72 h post-inoculation with *B. cinerea*, the expression levels of 1,299 and 1,543 genes were changed at least twofold relative to noninoculated plants in *B. cinerea*-inoculated WT and *shn1-1D*, respectively. Among these genes, 839 (55%) were solely differentially expressed post-inoculation in *shn1–1D* and 595 (46%) of them were solely differentially expressed in the WT (**Figure 5A**). The overlapping set of genes, upregulated in both *shn1–1D* and the WT 72 h post-infection, included 704 genes, among them many of the pathogenesis-related genes that are typically activated upon *B. cinerea* infection (**Table S1**). We did not observe activation of pathogenesis-related genes in mock noninfected *shn1–1D* (**Table S2**).

Further analysis of genes that were differentially expressed in inoculated plants (marked by a threefold cutoff) revealed induction of 220 genes and repression of 131 genes in *shn1–1D* but not WT plants, whereas 85 genes were induced and 102 genes repressed in WT but not *shn1–1D* plants, 72 h post-inoculation with *B. cinerea* (**Tables S 3** and **S4**). Genes exhibiting greater than threefold differential expression post-inoculation (*P*<0.05) were assigned to functional categories using MapMan. The classification of genes expressed post-inoculation exclusively in *shn1–1D* or in WT plants is presented in **Figure S3**. The percentage of upregulated genes in the categories of cell-wall localization, energy and development did not differ between *shn1–1D* and the WT. However, in agreement with the higher ROS accumulation, many more oxidative stress, secondary metabolism, hormone regulation, cellular and fungal sensing, cell death, detoxification and stress response genes were upregulated post-inoculation with *B. cinerea* in *shn1–1D* vs. the WT (**Figure S3**). Stress-related genes that were differentially regulated post-inoculation in *shn1–1D* vs. WT plants are listed in **Table 1**.

**Table 1:** Genes significantly differentially expressed in shn1*–*1D plants 72 h post- inoculation with *B. cinerea* (FC >2; P<0.05).

ATG	FC		Gene Description	ATG	FC	Gene Description
**Hormone signaling**				**Redox state**		
**Auxin**				AT1G32350	62.7	AOX1D (Alternative oxidase 1)
AT4G37390	18.3		YDK1/YADOKARI1/GH32 encodes an IAA-amido synthas	AT2G29350	32.9	SAG13 (SENESCENCE-ASSOCIATED GENE 13)
AT3G25290	9.5		auxin-responsive family protein	AT1G28480	13.5	GRXC9 (glutaredoxin family protein )
AT1G28130	4.3		GH3.17 encodes an IAA-amido synthase	AT1G03850	8.6	GRXS13 (glutaredoxin family protein )
AT3G22850	3.6		similar to auxin down-regulated protein ARG10	AT3G28850	4.4	glutaredoxin family protein
AT5G13370	3.3		auxin-responsive family protein	AT3G19010	3.6	oxidoreductase,2OG-Fe(II)
AT2G37980	3.2		similar to Auxin induced axi 1	AT1G45145	2.9	ATTRX5 (Thioredoxin H-Type 5)
**Brasinosteroids**				**Peroxidases**		
AT1G74360	17		similar to BRL1(BRI1 LIKE)	AT5G05340	34.5	PER52 (Peroxidase52 precursor)
AT3G13380	7.1		BRL3(BRI1-LIKE3)	AT3G49120	8.9	PERX34(Peroxidase 34precursor)
ABA				AT5G06720	7.5	Identical to Peroxidase53 precursor (PER53)
AT1G02590	3.2		aldehyde oxidase	AT4G36430	7.2	PER49 (Peroxidase 49 precursor)
AT4G26080	3.1		ABI1 (ABA INSENSITIVE1)	AT5G19880	5.2	PER 58 (Peroxidase 58 precursor)
**Ethylene**				AT5G64100	3.8	PER69 (Peroxidase 69 precursor)
AT1G01480	19		ACS (ACC synthase gene family)	AT1G07890	3.8	APX1/MEEA6 (ASCORBATE PEROXIDASE1)
AT4G37150	16.7		esterase	AT4G37530	3.4	Identical to Peroxidase 51 precursor (PER51)
AT3G50260	4.8		AtERF11/CEJ1 (regulated by Ethylene and jasmonate)	**Glutathion-S-transferases**		
**Proteolysis**				AT1G69930	53.1	AtGSTU11(Glutathione S-transferase, class tau11)
AT3G50930		11.8	AAA-type ATPase family protein	AT2G29470	30.7	AtGSTU3 (GlutathioneS-transferase 21)
AT5G67340		8.6	armadillo/beta-catenin repeat family protein	AT2G29460	28.6	AtGSTU4 (GlutathioneS-transferase 22)
AT2G38860		7.6	YLS5 (yellow-leaf-specific gene5)	AT1G17180	23.3	AtGSTU25 (GlutathioneS-transferase, class tau 25)
AT2G42360		6.5	Zinc finger(C3HC4-type)family protein Identical to ATL2L	**β-glucanase**		
AT1G44130		6.2	nucellin protein, putative;aspartic-type endopeptidase	AT3G04010	3.9	glycosyl hydrolase family 17protein
AT5G63970		4.3	similar to copine-related	**PR proteins**		
AT5G45890		4.3	SAG12(SENESCENCE-ASSOCIATEDGENE 12	AT3G04320	14.1	endo peptidase inhibitor
AT5G41400		3.8	Zinc finger(C3HC4-type )family protein	AT1G02360	8.2	chitinase
AT5G57480		3.4	AAA-type ATPase family protein	AT3G23110	6.7	disease resistance family protein
AT1G08050		3.4	Zinc finger(C3HC4-type)family protein	AT1G55210	6.1	disease resistance response
AT1G76390		3.1	armadillo/beta-catenin repeat family protein	AT3G54420	5.7	ATEP3 (chitinase class IV)
AT2G45040		3.1	matrix metallo-proteinase	AT1G22900	3.8	disease resistance family protein
				AT1G71400	3.6	disease resistance family protein/LRR family protein
**Cell wall**				**Secondary metabolism**		
AT5G62150	22.9		peptidoglycan-binding LysM domain-containing protein			
AT3G09410	19		pectin acetyl esterase family protein	AT3G26830	67.6	PAD3 (PHYTOALEXIN DEFICIENT 3)(CYP450 71B15)
AT4G18990	6.5		AtXTH29 (xyloglucosyl transferase)	AT4G37990	8.6	ELI3*–*2(ELICITOR-ACTIVATED GENE3)
**Tabl1 cont.**						
**Cell wall cont.**				**Secondary metabolism cont.**		
AT1G67070	4.5		DIN9 (DARK INDUCIBLE9)	AT2G37040	8.1	PAL1(PHEAMMONIALYASE 1)
**Pathogen/pest attack signaling**				AT1G51680	5.9	4CL1(4-COUMARATECOA LIGASE 1)
AT5G64905	52		PROPEP3 (Elicitor peptide 3 precursor)	AT2G36800/AT2G36790	5.8	UGT73C6(UDP-glucosyl transferase73C6)
AT3G01830	23.9		calmodulin-related protein, putative	AT5G49690	5.4	UDP-glucosyl transferase family protein
AT5G11210	18.3		AtGLR2.5 (glutamate receptor 2.5)	AT3G51440	4.8	strictosidine synthase family protein
AT3G46280	12.1		protein kinase-related	AT5G63600	4.6	flavonol synthase, putative
AT5G01550	10.9		lectin protein kinase, putative	AT5G39050	4.6	transferase family protein
AT2G39200	9.6		MLO12 (MILDEW RESISTANCE LOCUS O12)	AT3G50280	4.4	transferase family protein
AT3G09010	7.4		Protein kinase family protein	AT2G18950	4.3	HPT1(HOMO GENTISATEPHYTYL TRANSFERASE 1)
AT5G25930	7.2		leucine-rich repeat family protein/protein kinase family	AT5G48180	4.3	NSP5 (nitrile-specifier protein)
AT5G26920	6.9		calmodulin binding protein	AT2G30490	3.8	ATC4H (CINNAMATE*–*4-HYDROXYLASE) (CYP450 73A5)
AT5G38250	6		serine/threonine protein kinase, putative	AT4G34230	3.7	CAD5 (CINNAMYL ALCOHOL DEHYDROGENASE 5)
AT4G23140	5.9		CRK6(CYSTEINE-RICH RLK6)	AT3G51430	3.1	YLS2(yellow-leaf-specific gene2)
AT1G70690	5.7		kinase-related	**Transcription factors**		
AT4G21380	4.9		ARK3(Arabidopsis Receptor Kinase3)	AT3G23250	15.6	AtMYB15/AtY19
AT5G06740	4.7		lectin protein kinase family	AT3G50260	4.7	ERF/AP2
AT4G23150	4.3		similsr to protein kinase family protein	AT1G48000	3.3	MYB112
AT3G54950	3.8		PLAIIIA/PLP7 (PATATIN-LIKE PROTEIN 7)	AT1G22190	3.2	AP2 domain-containing transcription factor, putative
AT1G66880	3.8		serine/threonine protein kinase family	AT5G65210	3	TGA1
AT4G28490	3.3		HAESA(RECEPTOR-LIKE PROTEIN KINASE 5)	AT1G32640	2.8	JAI1(MYC2)
AT3G47090	3.2		leucine-rich repeat transmembrane protein kinase, putative	**Abiotic stress**		
AT2G39660	3		BIK1 (botrytis induce kinase 1)	AT4G36990	7.2	HSF4 (HEAT SHOCK FACTOR 4)
AT2G25470	2.7		leucine-rich repeat family protein	AT2G21620	3.6	RD2 (RESPONSIVE TO DESSICATION 2)
AT3G20590	2.5		NDR1(NON RACE-SPECIFIC DISEASERESISTANCE1)			

Shown genes related to detoxification, secondary metabolism, and stress responses that were upregulated greater than twofold 72 h after *Botrytis cinerea* infection. Microarray data are derived from two biologically independent experiments; details are given in Supplemental Table 1and 2 online.

We further validated the expression levels of genes related to defense [phytoalexin deficient 3 (*PAD3*), *TGACG motif binding transcription factor 1* (*TGA1*), *phenylalanine ammonia-lyase 1* (*PAL1*), *Jasmonate Insensitive 1*(*JAI1*) and *elicitor peptide 3 precursor* (*PROPEP3*)], detoxification [(*Glutathione S-transferase 11*(*GST11*)], senescence and oxidative stress [*senescence-associated gene 12* (*SAG12*) and *alternative oxidase 1D* (*AOX1D*)], and programmed cell death (PCD) [(*accelerated cell death 11 (ACD11*)] by real-time quantitative RT-PCR (qRT-PCR) (**Figure 5B**). Gene expression was also validated in five independent biological experiments (**Figure S4**). Further analysis of the microarray data using the Limma statistical suite revealed similar results (**Table S5**). This activation pattern of the oxidative stress response genes was actually supported by ROS accumulation (**Figure 3**), indicating that the strong gene activation in *shn1–1D* is not due to a putative expression loop that does not support translation.

Taken together, our data indicate that in response to *B. cinerea* infection, the *shn1–1D* transcriptome changes differently from that of the WT, with stronger activation of defense-, stress-, senescence- and PCD-related genes. Interestingly, these genes’ activation was ineffective against *B. cinerea, A. brassicicola* and *S. sclerotiorum*, since the outcome was sensitivity of *shn1–1D* plants to those necrotrophic pathogens.

### *shn1-1D* Cutin Monomer Extract Affects Plant Sensitivity to *B. cinerea*

**Figure 6:**
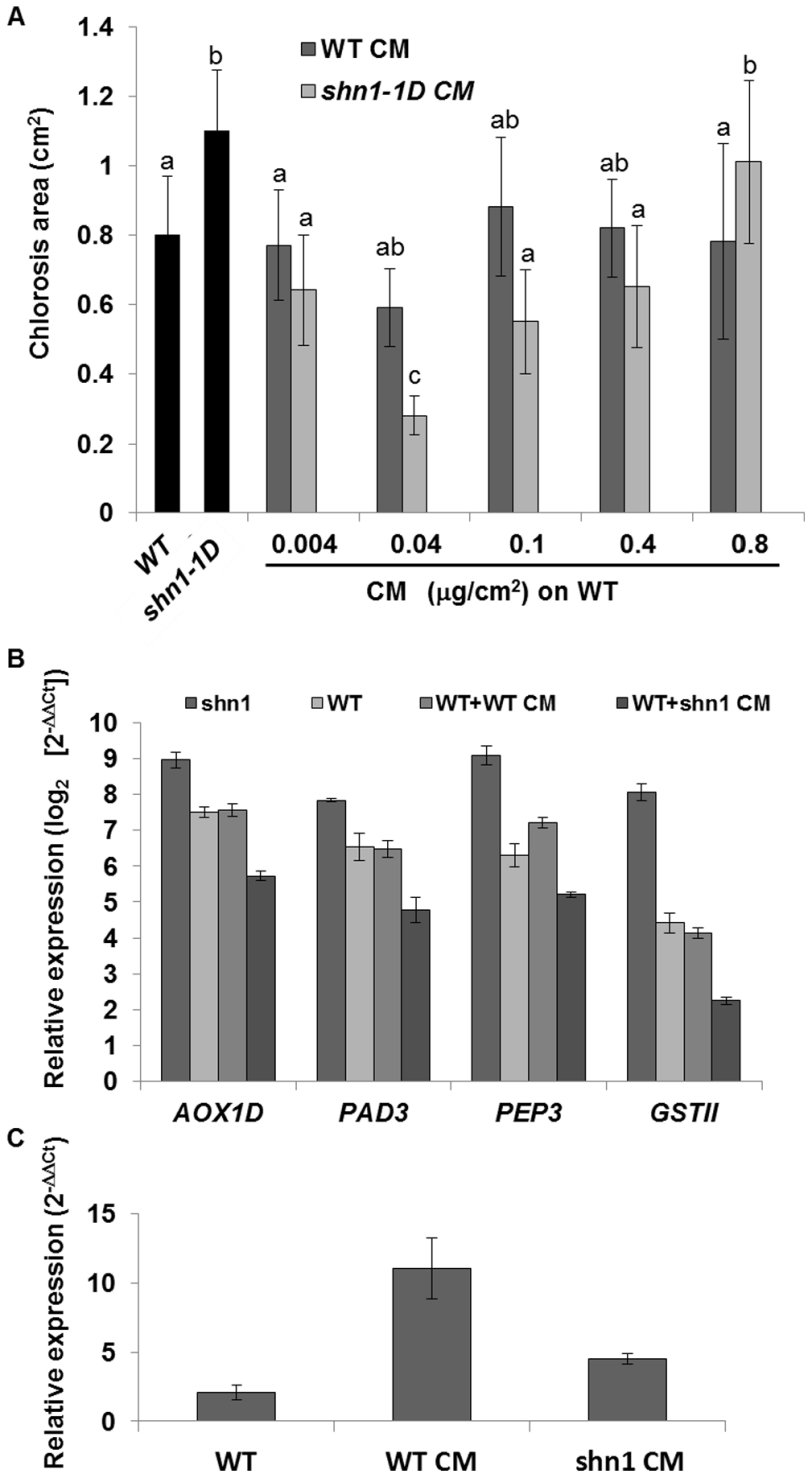
Effect of cutin monomers on disease symptoms and gene expression. **A,** WT leaves were inoculated with *B. cinerea* spores supplemented with 0.04, 0.4 or 0.8 µg/cm^2^ cutin monomers extracted from either *shn1–1D* (*shn1–1D*-CM) or the WT (WT-CM). As a control, we used WT and *shn1-1D* leaves inoculated with *B. cinerea* only. Presented are means±SD of chlorotic area of 15 leaves 72 h post-inoculation. Different letters represent significant difference by Tukey-Kramer HSD analysis (*P*<0.05). **B,** Expression of selected genes in WT leaves inoculated with *B. cinerea* spores supplemented with 0.04 µg/cm^2^ cutin monomers extracted from either *shn1–1D* (WT+*shn1–1D*-CM) or WT (WT+WT-CM). As a control, we used WT and *shn1–1D* leaves inoculated with *B. cinerea* only. **C,** PAL1 expression in WT leaves supplemented with 0.04 µg/cm^2^ cutin monomers extracted from either *shn1–1D* (WT+*shn1–1D* CM) or WT (WT+WT-CM).

The characteristic *SHN1/WIN1* overexpressor phenotype includes very high cutin content; the activation-tagged *shn1–1D* line used in this study was found to have a total of 28 times more cutin content than the WT (**Figure S5**). In comparison, overexpression of the *SHN1* gene under the constitutive 35S promoter leads to just a 3.5-fold increase in cutin levels as compared to WT plants [23]. Furthermore, not only was the total amount of cutin altered (**Figure S5A**), but its composition was as well (**Figure S5B**), and the fold change of each monomer ranged from 7 to 104 times more cutins in *shn1–1D* (**Figure S5C**). To determine if cutin monomer content and composition in *shn1–1D* plants are responsible for their susceptibility to *B. cinerea*, cutin monomers were extracted from *shn1–1D* and WT plants. They were then applied together with the *B. cinerea* conidial suspension to WT leaves. Interestingly, *B. cinerea* pathogenicity was inhibited when the inoculation suspension applied to WT leaves was supplemented with 0.04 µg/cm^2^ of *shn1–1D* cutin monomers as compare to WT plants inoculate with *B. cinerea* only (**Figure 6A**). We did not observe this activated-resistance effect when we added higher concentrations of cutin monomers (0.1, 0.4 or 0.8 µg/cm^2^ of either WT- or *shn1–1D*-extracted cutin monomers) or a lower concentration (0.004 µg/cm^2^) (**Figure 6A**), suggesting that the defense response is dependent on both cutin monomer dose and composition.

To check whether this effect is the result of direct toxicity of the extracted cutin monomers to the fungus, cutin monomer extracts were added to PDA plates to examine their effects on *B. cinerea* spore germination. Interestingly, *B. cinerea* spore germination on PDA plates containing cutin monomers from either *shn1–1D* or WT plants did not differ (**Figure S6A**). To test whether the observed susceptibility of *shn1–1D* to *B. cinerea* is due to higher carbon source availability in the thick *shn1–1D* cuticle we used a cutinase-deficient *B. cinerea* mutant [26] for pathogenicity assays. We found that *shn1–1D* is also more susceptible than the WT to this mutant (**Figure S6B**).

### *shn1-1D* Cutin Monomer Composition can Control Defense Activation

We quantified the transcripts of *PAD3*, *AOX1D* and *PROPEP3 (PEP3)* (genes that were strongly upregulated in the microarray analysis, **Table 1**) by qRT-PCR after coinoculation with *B. cinerea* and cutin monomers. In accordance with the resistance found when *shn1–1D* cutin monomers were supplemented to the inoculation suspension (**Figure 6A**), we found these genes to be moderately upregulated in WT plants 72 h after inoculation with *B. cinerea* suspension supplemented with *shn1–1D* cutin monomers, as compared to their expression in *shn1–1D* plants inoculated with *B. cinerea* alone, and even to their expression in inoculated WT plants (**Figure 6B**). Genes were upregulated, but to an appropriate level that supported resistance. Furthermore, when 0.04 µg of cutin monomers, extracted from either *shn1–1D* or the WT, were applied alone on WT leaves, we also observed that the *shn1–1D* cutin monomers cause moderate upregulation of *PAL1* as compared to WT cutin monomers (**Figure 6C**). These results suggested that the composition of *shn1–1D* cutin monomer may contribute to the magnitude of the defense activation.

## Discussion

We used *shn1–1D* mutants to characterize the cuticle’s role in defense responses against the necrotrophic fungus *B. cinerea*. Recent work has demonstrated that cuticular defects lead to full immunity to *B. cinerea* [16], [18], suggesting that increased permeability of the cuticle is involved in resistance due to an incremental release of fungitoxic compounds. Our data demonstrate that despite their more permeable cuticle [21], *shn1-1D* plants are more susceptible to *B. cinerea*, *S. sclerotiorum* and *A. brassicicola* than WT plants (**Figure 1**). However, *shn1-1D* plants inoculated with a *B. cinerea* isolate that is impaired in its cutinase gene did not demonstrate lower susceptibility relative to the WT, suggesting that the observed susceptibility is not due to availability of a carbon source in the thick *shn1-1D* cuticle (**Figure S6B**). Despite their increased susceptibility, *shn1-1D* plants exhibited an enhanced defense response that included elevated levels of ROS following both biotic and abiotic stresses, as well as enhanced PCD in the chlorotic area, which expanded beyond the lesion and its neighboring cells (**Figures 2** and **3**), supported by activation of the PCD marker *SAG13* [27]. This phenomenon has been previously shown in lesion-mimic mutants, in which cell death spreads uncontrollably into the uninfected surroundings of the site of the hypersensitive response [28], [29].

ROS accumulation is one of the earliest plant defense responses, which also activates associated cell death [30]; on the other hand, it can also be used by *B. cinerea*, as this necrotrophic pathogen uses ROS to kill host cells, thereby facilitating infection [31]-[33]. Even though we observed high H_2_O_2_ levels following abiotic stress as well (**Figure 3**), we cannot rule out the possibility that the pathogen is responsible for part of the accelerated generation of ROS following infection. We hypothesize that in *shn1–1D* plants, high levels of ROS are accumulated after infection, which activate a strong and unique defense response. This strong response ultimately leads to runaway hypersensitive response-like cell death and sensitivity. In support of this hypothesis, we found that even though the permeable *shn1–1D* plants contain more bacteria after inoculation with the biotrophic *P. syringae*, these bacteria do not proliferate to a higher concentration than in the WT, even though the plants are more chlorotic; this is probably due to plant cell death, which restricts biotrophic bacteria (**Figure 4**).

Genome-wide expression analysis of *shn1–1D* and WT plants following *B. cinerea* infection strengthened our hypothesis of strong and unique but inappropriate defense activation in the former. We observed a significant increase, as reflected by mean gene number and fold change, in the activation of defense-response genes in *shn1–1D* relative to the WT, but also of negative regulators of the defense machinery; we also found a set of genes that were only highly activated in *shn1–1D* (**Table 1** and **Table S3**).

Hyperinduction of the defense response can eventually cause plant suicide: this is why plants have evolved negative-feedback regulation to control this response’s magnitude [28], [29], [34]. Indeed, we found activation of negative regulators of the defense machinery and PCD, such as *accelerate cell death 11 (ACD11), mildew resistance locus O12* (*MLO12*) [35], [36], *glutaredoxin C9* (*GRX480*) [37] and MYC2/JIN1, which has been found to positively activate oxidative stress tolerance but also to act as a negative factor in the accumulation of tryptophan-derived secondary metabolites [38]. We suggest that the activation of genes that are negative regulators of defense and PCD in *shn1–1D* plants is part of the plant's unsuccessful attempt to control the magnitude of its defense response. On the other hand, we found downregulation of *jasmonate resistant 1* (*JAR1*) [39] in *shn1–1D* mutants after infection, which might be responsible for the increased sensitivity demonstrated against *B. cinerea*. However, it is likely that *JAR1* downregulation is also part of the plant’s attempt to lower the defense magnitude, since the downstream JA-responsive genes, such as *PDF1.2,* are activated. Furthermore, a large group of genes that were upregulated only in infected *shn1–1D* plants and are connected to the oxidative stress response were class III peroxides. This class of genes are involved in the defense response against pathogens and wounding by triggering oxidative burst [40] but also can be active as scavengers of ROS [41]–[43]. This may also reflect an attempt by *shn1–1D* plants to control the magnitude of the ROS accumulation and defense response. However, activation of the senescence-associated gene *SAG12* allows for the possibility that senescence is also part of the phenotype demonstrated in *shn1–1D*-infected plants. Alternatively, the activation of class III peroxidases might affect cell wall crosslinking that is dependent on H_2_O_2_, as previously demonstrated [44]. Since *SHN1* also acts to modify the epidermis cell wall by altering pectin metabolism and structural protein [24], and since it is well documented that cell wall integrity and ROS accumulation have an impact on plant–fungus interactions [45]–[48], it is likely that the cell wall modification in *shn1-1D* is part of the observed excess ROS accumulation and altered defense responses.

Based on the observed runaway cell death, we hypothesized that *shn1-1D* would be much more resistant to bacterial pathogens at first, due to strong PCD, which would stop the pathogen’s spread; eventually, however, the uncontrolled cell death activated by the plant defense machinery would kill the whole plant. Our data obtained using virulent bacterial pathogens support our hypothesis of uncontrolled cell death, preventing biotrophic pathogen proliferation in the dead tissue (**Figure 4** and **Figure S2**). However, we did not observe strong resistance of *shn1-1D* to biotrophic pathogens at the beginning of the infection. This might be due to the activation of genes involved in *shn1-1D*’s sensitivity to biotrophic fungi, such as the observed strong activation of *MLO12*. This gene is proposed to be a negative regulator of the defense mechanism and cell death in barley, as a loss-of-function mutation leads to resistance against biotrophic pathogens such as powdery mildews [35], [36]. Other MLO proteins have been suggested to act as negative regulators of cell-wall apposition formation during non-host resistance [49], [50]. Furthermore, MLO has been suggested to be a sensor and effector of cellular redox status [36]. Its strong activation further supports the notion of a strong defense response in *shn1-1D* via ROS accumulation, as well as our assumption of the plant’s unsuccessful attempts to lower the magnitude of the defense response.

Cutin monomers and surface wax constituents elicit H_2_O_2_ production in conditioned cucumber hypocotyl segments and enhance the activity of other H_2_O_2_ elicitors [13], [14], [51]. The high H_2_O_2_ accumulation in *shn1-1D* plants following biotic and abiotic stresses is compatible with the concurrent activation of oxidative stress genes such as *PROPEP3* or the alternative oxidase gene (*AOX1d*). *PROPEP3* suggested to be a ROS-dependent amplifier of both the ethylene/jasmonic acid (JA) and salicylic acid (SA) defense pathways [52], [53], and *AOX1d*, is regulated by stress and its expression has been found to be dependent on SA, ethylene/JA and ROS, and to be associated with leaf senescence as well [54], [55]. Furthermore, the brassinosteriod (BR) pathway, which was upregulated in infected *shn1-1D* plants, has been found to function in a broad range of resistance as well as tolerance to abiotic stresses that elicit ROS production and cell death [56]–[58].

We further demonstrated that the magnitude of activation of the defense-related genes is reduced when cutin monomers released after transesterification from *shn1-1D* leaves are administered to inoculated WT leaves, resulting in resistance to *B. cinerea*, which is cutin composition- and concentration-dependent (**Figure 6A, B**). This moderate activation was also found when uninfected leaves were treated only with *shn1-1D* cutin monomers, but not when supplemented with only WT cutin monomers (**Figure 6C**). WT cutin monomers at the same concentration, and even at a 20-fold higher concentration, had no such impact on plant defense, while a higher concentration of *shn1-1D* cutin monomers supplemented to *B. cinerea*-infected WT plants led to sensitivity of the latter resulting from runaway cell death, as in the *shn1-1D* plants. Since *shn1-1D* plants have a total of 28 times more cutin, but the composition of their cutin monomers is greatly altered–7 to 100 times more of each individual monomer (**Figure S5**)–our data indicate that the composition of *shn1-1D* cutin monomers might be involved, together with ROS induction, in the activation of a strong defense signal, resulting in more severe cell death, which enhances *B. cinerea* infection and plant sensitivity.

When the quantity of that monomer composition is adjusted, it can lead to fine-tuned defense activation that stops *B. cinerea* infection. Fatty acids of most free cutin monomers are probably activated in some form (eventually as CoA esters), are already linked to glycerol, or do not really occur in large amounts in planta at all. Nevertheless, it has been demonstrated that cutin monomers can be perceived by plant cells and can effectively elicit H_2_O_2_ in cucumber and rice [14], [59]–[61]. We therefore propose that the increased permeability of the *shn1-1D* cuticle facilitates excess ROS formation and release, as also demonstrated by L’Haridon and colleagues [19]. This is channeled after infection, together with release of the special *shn1-1D* cutin monomer content and composition, into a strong and inappropriate defense response, which eventually leads to sensitivity to *B. cinerea*. Although we cannot rule out the possibility that other compounds in our cutin monomer extract, changes in the wax, or even cell wall alterations in *shn1-1D* plants also play a role in defense responses, our results strongly suggest an important role for cutin monomer content and composition, rather than only cuticle structure and permeability, in plant–fungal interactions. The challenges are to reveal the exact composition or individual monomer, and/or their activated in-planta form that responsible for plant defense. Moreover to discover the molecular mechanisms that leads to excess ROS accumulation.

## Materials and Methods

### Plant Lines and Growth Conditions

*Arabidopsis thaliana* (L.) Heynh. accession Ws-0 (WT) and activation-tagged line *shn1-1D* [21] were used. All seeds were scarified on moist soil at 4°C for 2–3 days before placing them in a growth chamber. Plants were grown at 22°C and 60% relative humidity under illumination with fluorescent and incandescent light at a photofluency rate of approximately 120 µmol/m^2^·s; day length was 12 h unless otherwise specified.

### Fungal Strains, Growth and Inoculation Method

*B. cinerea* strain B05.10 (sequenced isolate obtained from Syngenta), *S. sclerotiorum* (sequenced isolate, 1980) and *A. brassicicola* (isolated in from infected *Brassica oleracea var. capitata*) were grown on potato dextrose agar (PDA; Difco, France). *B. cinerea* Δcutinase isolate [26] was grown on PDA supplemented with 100 mg/l hygromycin. Growth was in a controlled-environment chamber at 22°C under illumination with fluorescent and incandescent light at a photofluency rate of approximately 120 µmol/m^2^·s and 12 h day length. Conidia were harvested in sterile distilled water and filtered through four layers of sterile gauze to remove hyphae. For inoculation, the conidial suspension was adjusted to 3,000 conidia/µl in half-strength filtered (0.45 µm) grape juice (100% pure organic) for *B. cinerea* (mock was half-strength grape juice) and in water for *A. brassicicola* (mock was water). *S. sclerotiorum* inoculation was performed with 5-mm diameter mycelial plugs (mock was PDA plugs). Detached leaves from the different genotypes were layered on agar trays and inoculated with 5-µl droplets of conidial suspension or mycelial plugs. Lesion size, 3,3-diaminobenzidine (DAB)-stained area and chlorotic area or intensity were measured using ASSESS 2.0 image-analysis software for plant disease quantification (APS Press, USA).

### Bacterial Strain Growth and Inoculation Method

*Arabidopsis* plants were inoculated by spraying with 10^7^ cfu/ml virulent *Pseudomonas syringae* pv. *tomato* DC3000. Bacteria were extracted 2 h (0) and 3, 6 and 9 days post-inoculation and plated on nutrient agar medium. Data were expressed as log_10_ colony-forming units per gram tissue. Since we used spraying inoculation, symptoms started to develop 3–4 days after inoculation, unlike in vacuum infiltration symptoms begin to appear at 18–24 h. This is why we followed the bacterial growth for 9 days post-inoculation [62].

### Cutin Monomers Extraction

Rosette leaves (20–50) were exhaustively extracted with chloroform:methanol (1∶1, v/v) over a period of 2 weeks with a daily change of solvent. After air-drying, the leaves were flushed with nitrogen gas and kept for the depolymerization reaction.

Cutin monomers were released by transesterification of totally extracted leaves with 3 ml 1 N MeOH/HCl for 2 h at 80°C. After addition of 3 ml saturated NaCl/H_2_O, the hydrophobic monomers were extracted three times in 3 ml hexane. The combined extracts were dried over Na_2_SO_4_ (anhydrous) and then evaporated under a stream of nitrogen gas. Cutin monomers released by transesterification were then dissolved in 1 mg/ml dimethyl sulfoxide (DMSO) and used for inoculation and gene-activation experiments. A total of 0.8, 0.4 or 0.04 µg cutin monomers was used for each leaf, an equivalent DMSO concentration was added to the control inoculated leaves.

### Electron Microscopy

For SEM, leaves were collected and fixed with glutaraldehyde using standard protocols [63] and critical-point dried. Samples were mounted on aluminum stubs and sputter-coated with gold. SEM was performed using an XL30 ESEM FEG microscope (FEI, OR) at 5–10 kV.

### Trypan Blue Staining

To visualize the *B. cinerea* hyphae and dead plant cells, we stained inoculated leaves with a lactophenol-trypan blue solution [10 g phenol, 10 ml glycerol, 10 ml lactic acid, 10 ml water and 0.02 g trypan blue (Biological Industries, Beit Haemek, Israel)]. Leaves were boiled in this solution for 5 min and then washed for 3 days in 2.5 mg/ml chloral hydrate, replacing the washing solution every 24 h. Leaves were documented with a PowerShot S5 IS Canon digital camera and then visualized under an Axioscope light microscope (Carl Zeiss, Jena, Germany) and documented with a DXM1200F digital camera (Nikon, Tokyo, Japan).

### Ion-leakage Measurement

For conductivity measurements, 24, 48 and 72 h after infection with *B. cinerea*, leaf pieces (10 mm^2^) were cut from infected leaves with a scalpel, washed in distilled water and transferred to tubes containing 5 ml of distilled water for 6 h. Conductivity of the solution was determined with a conductivity meter (Mettler Toledo S30K, Germany) at the indicated time points. Means and standard errors were calculated from three replicate measurements per genotype per experiment after calibration with untreated leaves. For each measurement, we used seven pieces. The entire experiment was performed three times.

### Herbicide Treatment

Detached leaves were immersed in 10 µM paraquat solution or water in Petri dishes and kept in the growth room (20°C, 16 h light/8 h dark) for 8 h before analysis of ROS accumulation (see below). Alternatively, a 25 µM paraquat solution was used to spray the foliage thoroughly and then plants were kept in the growth room (20°C, 16 h light/8 h dark) for 1 week for death scoring.

### Mechanical Wounding

Fully expanded leaves from both WT and *shn1-1D* mutant plants were excised at the end of the petiole and wounded in the middle with a metal punch (0.5 cm in diameter). Leaves were then immediately subjected to ROS accumulation analysis (see below). DPI (100 µM) was added to control leaves.

### ROS Accumulation Analysis by DAB Staining

Production of H_2_O_2_ in plants was measured by staining plant tissues with DAB. Briefly, plant tissue was incubated in 1 mg/ml DAB solution (pH 3.8) for 40 min under vacuum, then rinsed with double-distilled water, cleared in boiling clearing solution (ethanol:acetic acid:glycerol, 4∶1:1, v/v) for 5 min, and kept in the same solution pending observation. For wounding treatment, tissues were incubated in a l mg/ml DAB solution for 3 h in the growth room in the light.

### Microarray Experiment and Data Analysis

Rosette leaves of 5-week-old *shn1-1D* mutant and WT plants were collected 72 h after inoculation with *B. cinerea* or mock inoculation with 0.5X grape juice. For each sample, we pooled leaves of three plants that showed leaves with clear infection as shown in **Figure 1A** but not dead leaves. Total RNA was extracted with TRI-Reagent (Invitrogen Corporation, CA) and then treated with DNase and cleaned on RNeasy columns (Qiagen, Valencia, CA). Labeled-copy RNA was prepared and hybridized to Affymetrix ATH1 GeneChips, according to the manufacturer’s guidelines [64]. Statistical analysis of the microarray data was performed using Partek® Genomics Suite (Partek Inc., St. Louis, MO) software. CEL files (containing raw expression measurements) were imported to Partek GS. The data were preprocessed and normalized using the RMA (Robust Multichip Average) algorithm [65]. The normalized data were processed by principal component analysis (PCA) and hierarchical clustering to detect batch or other random effects that may appear when the replicates are carried out sequentially (**Figure S7A, B**). Batch effects were not found. To identify differentially expressed genes, ANOVA was applied. False discovery rate (FDR) was used to correct for multiple comparisons [66]. Gene lists were created by filtering the genes based on: fold change and signal above background in at least one microarray. Upregulated genes were defined as those having an at least twofold linear intensity ratio. The Venny online resource (http://bioinfogp.cnb.csic.es/tools/venny/index.html) was used to create Venn diagrams. MapMan software (http://gabi.rzpd.de/projects/MapMan) was used to create MapMan overview diagrams of the microarray data [67].

### DNA Extraction and PCR Analysis

Leaf samples taken 72 h after inoculation with *B. cinerea* were ground in liquid nitrogen and extraction buffer was then added (1 M Tris HCl pH 7.5, 5 M NaCl, 0.5 M EDTA and 20% w/v SDS). Samples were incubated at 65°C for 15 min and then centrifuged for 15 min at 4°C, 1,880 *g* Supernatant was transferred to a new tube with isopropanol (1∶1, v/v), vortexed and incubated for 20 min at room temperature. Tubes were then centrifuged for 30 min at 4°C, 1,880 *g*. The supernatant was discarded and isopropanol was added to the pellet and centrifuged for 15 min at 4°C, 1,880 *g*. The pellet was then washed twice in 70% ethanol, dried at 37°C and resuspended in TE (10 mM Tris, 1 mM EDTA, pH 7.5). The extracted DNA (2 µl) was used for PCR analysis with two sets of β-tubulin primers, one for *B. cinerea* (BC1G_00122) (F: 3′ ATGATGGCCGCTTCCGATT 5′, R: 3′ CTCGCCCTCAATTGGGACCT 5′) and the second as a control for plants (β-tubulin 8; AT5G23860)(F: 3′ TTCTCGATGTTGTTCGTAAGGAAGC 5′, R: 3′ AGCTTTCGGAGGTCAGAGTTGAGTT 5′). PCR conditions were as follows: initial denaturation at 94°C for 2 min, then 35 cycles of 94°C for 20 s, annealing at 54°C for 15 s, extension at 70°C for 40 s followed by a final 10 min extension at 70°C.

### Quantitative Real-time RT-PCR (qRT-PCR) Analysis

qRT-PCR was performed with the SYBR Premix Ex Taq (TaKaRa, Otsu, Japan) in an ABI7000 real-time PCR machine (Applied Biosystems, Foster City, CA). The thermal cycling program was as follows: 95°C for 3 min; 45 cycles of 95°C for 15 s and 53°C for 30 s; a cycle of 95°C for 1 min, 53°C for 1 min, and 70°C for 10 s, and 50 cycles of 0.5°C increments for 10 s. Relative fold change of all gene normalized to *AtPTB1F* on samples from infected versus uninfected Arabidopsis leaves and was calculated by the comparative threshold cycle 2^−ΔΔCt^ method, an approximation method to determine relative gene expression. For primer sequences, see **Table S6**.

### Statistical Analysis

ANOVA tests were performed using Student t-test when equal variance test was passed. Otherwise Welch ANOVA test was performed. For multiple factors, Tukey-Kramer HSD tests were performed. Significance was accepted at *P*<0.05.

## Supporting Information

Figure S1 **Herbicide resistance and disease symptoms. A,** H_2_O_2_ accumulation after herbicide application. DAB staining of *shn1-1D* and WT leaves 8 h after foliage filtration (upper panel) or drop application (bottom panel) of 25 µM paraquat. **B,** Disease symptoms after *B. cinerea* infection with or without DPI. Infected leaves 72 h post-inoculation with *B. cinerea* with (white) and without (gray) 100 µM DPI. All bars represent mean±SE of 20–21 leaves. Bars with different letters denote significant differences (*P*<0.05) as determined by Kruskal-Wallis ANOVA, Dunn's Method.
(TIFF)

Figure S2 **Bacterial proliferation on** ***shn1-1D*** **and WT plants. A,** Quantitative analysis of*×. campestris* pv. *campestris* bacterial growth in WT and *shn1-1D* mutant plants is presented. **B,** Infection phenotypes of representative Ws-0 wild-type and *shn1-1D* mutant plants 7 days post-inoculation. Results represent means±SE (n = 6).
(TIFF)

Figure S3 **Figure S3. Regulation of gene expression in** ***shn1-1D*** **and WT after** ***B. cinerea*** **inoculation.** Classification of genes that were upregulated at least threefold 72 h post-inoculation exclusively in WT or *shn1-1D* leaves (*P*<0.05).
(TIFF)

Figure S4 **Differential gene regulation by** ***B. cinerea*** **in** ***shn1-1D*** **and WT.** Relative gene expression between inoculated and noninoculated *shn1-1D* and WT plants. Expression of selected genes from microarray data validated using qRT-PCR on cDNA extracted from *shn1-1D* or WT leaves 72 h after inoculation with *B. cinerea* relative to noninoculated leaves. Results represent means±SE obtained from five independent experiments.
(TIFF)

Figure S5 **Cutin content and composition. A,** Total cutin content in *shn1-1D* and WT leaves. **B,** cutin composition and **C,** Fold change of individual cutin monomers in *shn1-1D* as compared to the WT. FA, fatty acid; DA, α,ω-dicarboxylic FA; 2-HFA, dihydroxy FA; ω-HFA, ω-hydroxy FA; C16-9/10-OH-DA, C16-9/10-hydroxy DA. Values are means±SE (n = 3; *P*<0.05 by Student's t-test).
(TIFF)

Figure S6 **Spore** **germination and disease symptoms. A,** Spore germination *in vitro.* Percentage of *B. cinerea* spore germination on PDA, PDA with 1% DMSO and PDA supplemented with *shn1-1D*-CM or WT-CM. **B,** Disease symptoms after infection with *B. cinerea* Δcutinase mutant. Infected leaves 72 h post-inoculation with *B. cinerea* Δcutinase. All bars represent mean±SE of 20–21 leaves. Different letters above the columns indicate statistically significant differences (*P*<0.05) as determined by Kruskal-Wallis ANOVA, Dunn's Method.
(TIFF)

Figure S7 **Microarray data analysis. A,** Hierarchical clustering and **B,** PCA.
(TIFF)

Table S1 **Genes significantly differentially regulated in both** ***shn1-1D*** **plants and the WT 72 h post-inoculation with** ***B. cinerea*** **(** ***P*** **<0.05) with a fold change (FC) of at least 2.**
(XLS)

Table S2 **Genes significantly differentially regulated in** ***shn1-1D*** **plants as compared to WT plants without inoculation (** ***P*** **<0.05) with a fold change (FC) of at least 2.**
(XLS)

Table S3 **Genes significantly differentially regulated in** ***shn1-1D*** **plants 72 h post-inoculation with** ***B. cinerea*** **(** ***P*** **<0.05) with a fold change (FC) of at least 2.**
(XLS)

Table S4 **Genes significantly differentially regulated in WT plants 72 h post-inoculation with** ***B. cinerea*** **(** ***P*** **<0.05) with a fold change (FC) of at least 2.**
(XLS)

Table S5 **Genes significantly differentially regulated in** ***shn1-1D*** **and WT plants 72 h post-inoculation with** ***B. cinerea*** **(** ***P*** **<0.05) with a fold change (FC) of at least 2, using Limma statistical package.**
(PDF)

Table S6 **Primers sequences used for qRT-PCR.**
(DOC)
