# Supplementary figures and images for: Overexpression of AtSHN1/WIN1 Provokes Unique Defense Responses
Source: PLoS One. 2013 Jul 29;8(7):e70146. doi: 10.1371/journal.pone.0070146 (PMC3726498; doi:10.1371/journal.pone.0070146)

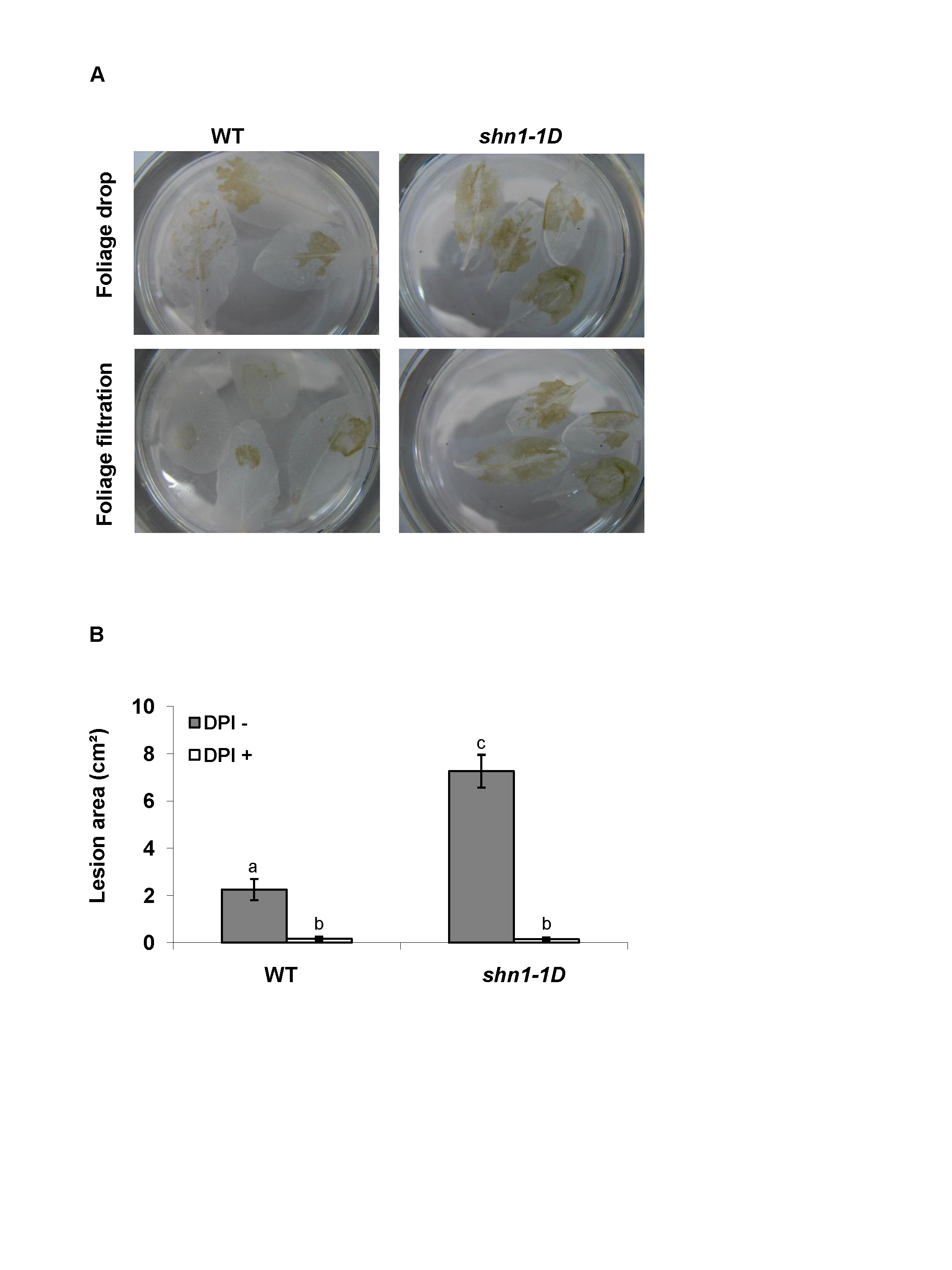

Supplement: Figure S1 — Herbicide resistance and disease symptoms. A, H2O2 accumulation after herbicide application. DAB staining of shn1-1D and WT leaves 8 h after foliage filtration (upper panel) or drop application (bottom panel) of 25 µM paraquat. B, Disease symptoms after B. cinerea infection with or without DPI. Infected leaves 72 h post-inoculation with B. cinerea with (white) and without (gray) 100 µM DPI. All bars represent mean±SE of 20–21 leaves. Bars with different letters denote significant differences (P<0.05) as determined by Kruskal-Wallis ANOVA, Dunn's Method. (TIFF) [file pone.0070146.s001.tiff]

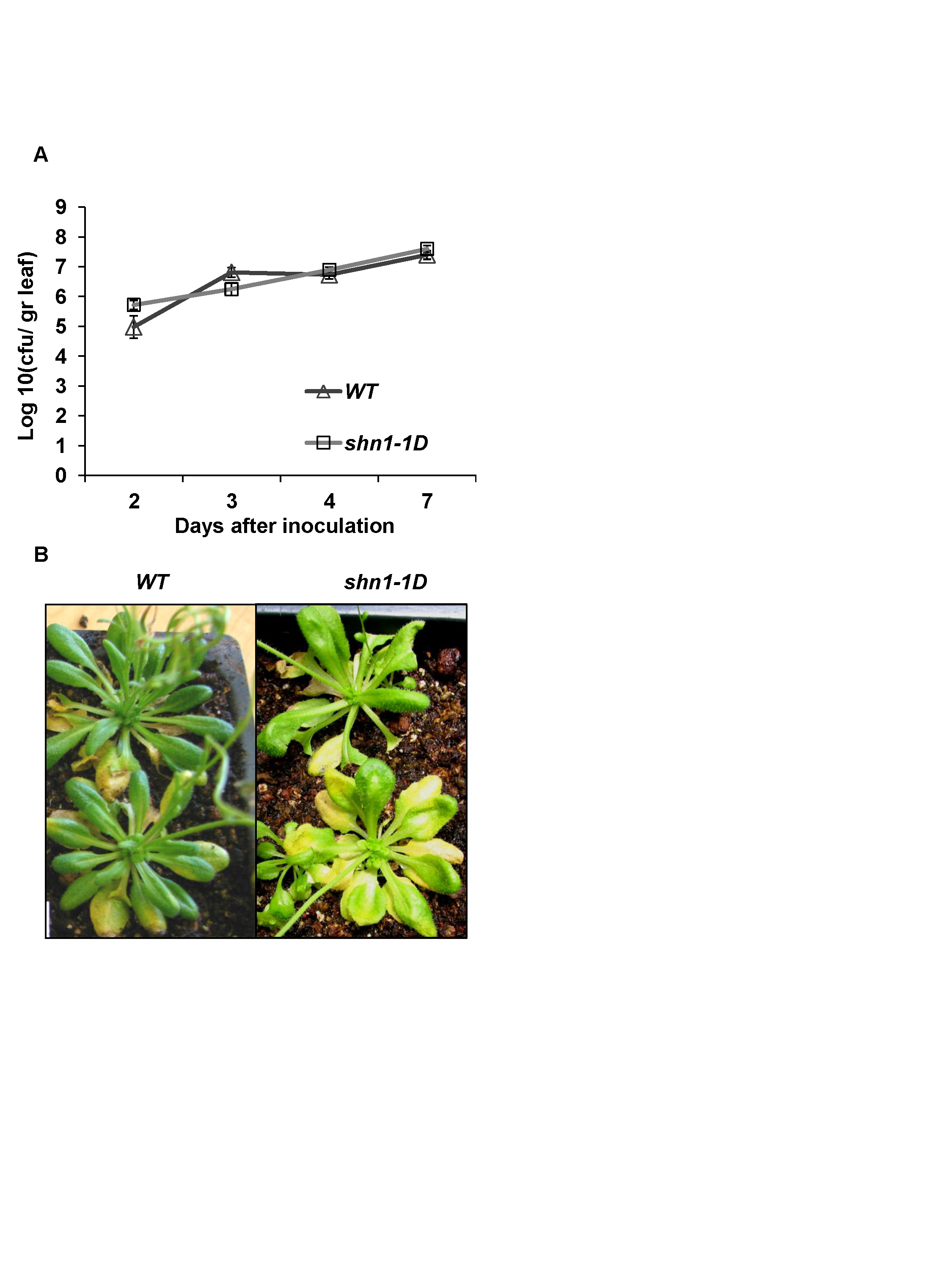

Supplement: Figure S2 — Bacterial proliferation on shn1-1D and WT plants. A, Quantitative analysis of×. campestris pv. campestris bacterial growth in WT and shn1-1D mutant plants is presented. B, Infection phenotypes of representative Ws-0 wild-type and shn1-1D mutant plants 7 days post-inoculation. Results represent means±SE (n = 6). (TIFF) [file pone.0070146.s002.tiff]

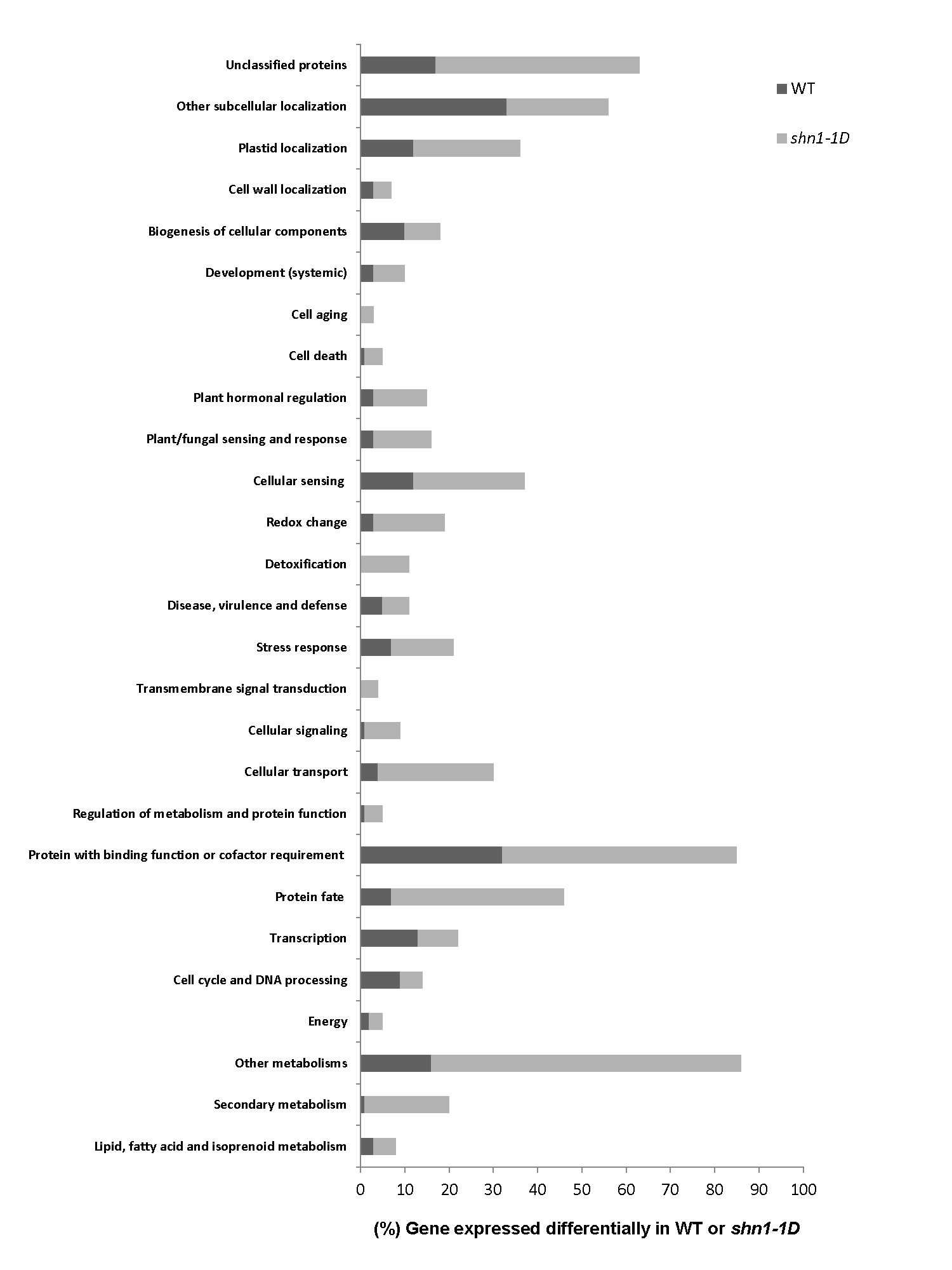

Supplement: Figure S3 — Regulation of gene expression in shn1-1D and WT after B. cinerea inoculation. Classification of genes that were upregulated at least threefold 72 h post-inoculation exclusively in WT or shn1-1D leaves (P<0.05). (TIFF) [file pone.0070146.s003.tiff]

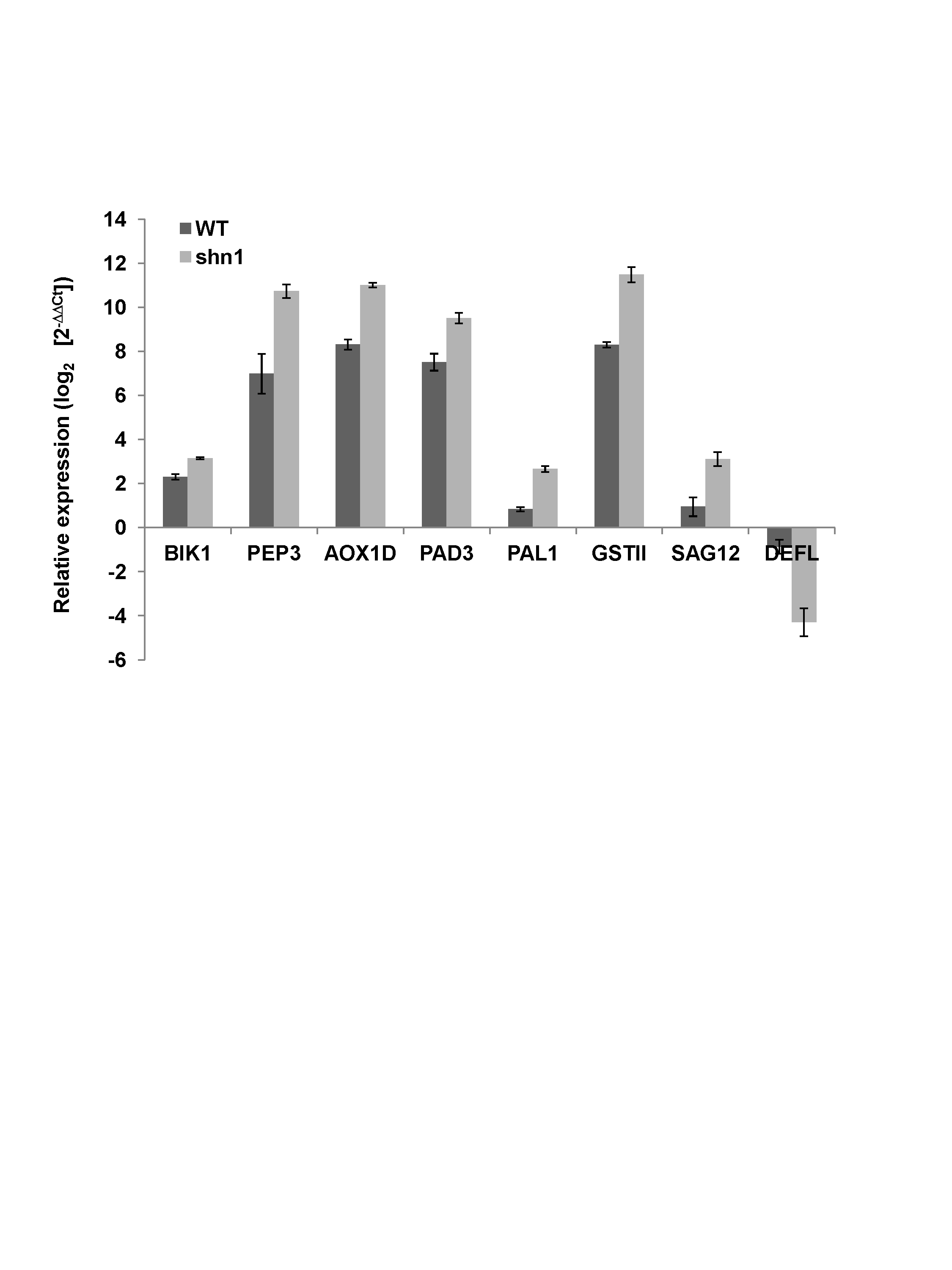

Supplement: Figure S4 — Differential gene regulation by B. cinerea in shn1-1D and WT. Relative gene expression between inoculated and noninoculated shn1-1D and WT plants. Expression of selected genes from microarray data validated using qRT-PCR on cDNA extracted from shn1-1D or WT leaves 72 h after inoculation with B. cinerea relative to noninoculated leaves. Results represent means±SE obtained from five independent experiments. (TIFF) [file pone.0070146.s004.tiff]

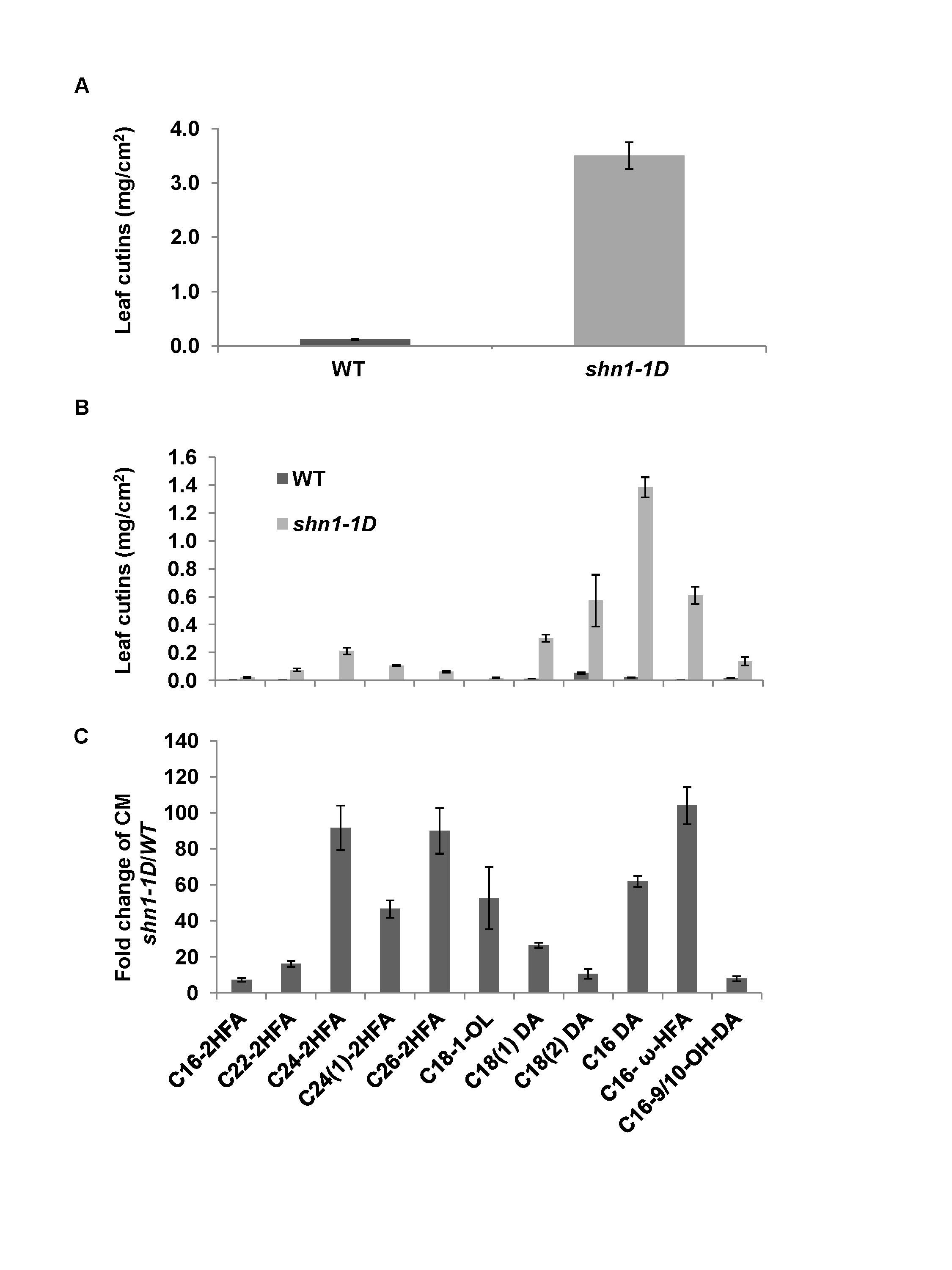

Supplement: Figure S5 — Cutin content and composition. A, Total cutin content in shn1-1D and WT leaves. B, cutin composition and C, Fold change of individual cutin monomers in shn1-1D as compared to the WT. FA, fatty acid; DA, α,ω-dicarboxylic FA; 2-HFA, dihydroxy FA; ω-HFA, ω-hydroxy FA; C16-9/10-OH-DA, C16-9/10-hydroxy DA. Values are means±SE (n = 3; P<0.05 by Student's t-test). (TIFF) [file pone.0070146.s005.tiff]

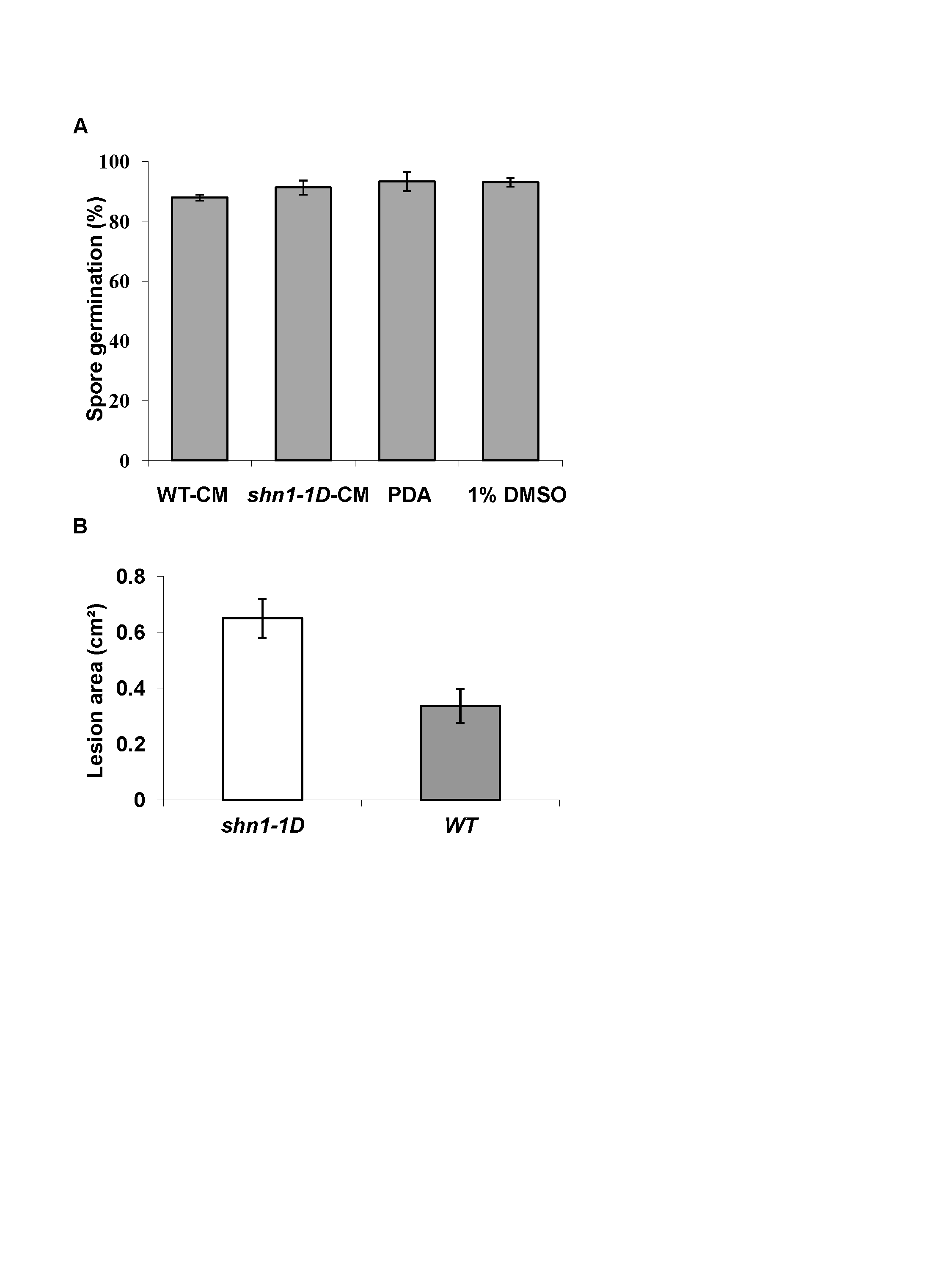

Supplement: Figure S6 — Spore germination and disease symptoms. A, Spore germination in vitro. Percentage of B. cinerea spore germination on PDA, PDA with 1% DMSO and PDA supplemented with shn1-1D-CM or WT-CM. B, Disease symptoms after infection with B. cinerea Δcutinase mutant. Infected leaves 72 h post-inoculation with B. cinerea Δcutinase. All bars represent mean±SE of 20–21 leaves. Different letters above the columns indicate statistically significant differences (P<0.05) as determined by Kruskal-Wallis ANOVA, Dunn's Method. (TIFF) [file pone.0070146.s006.tiff]

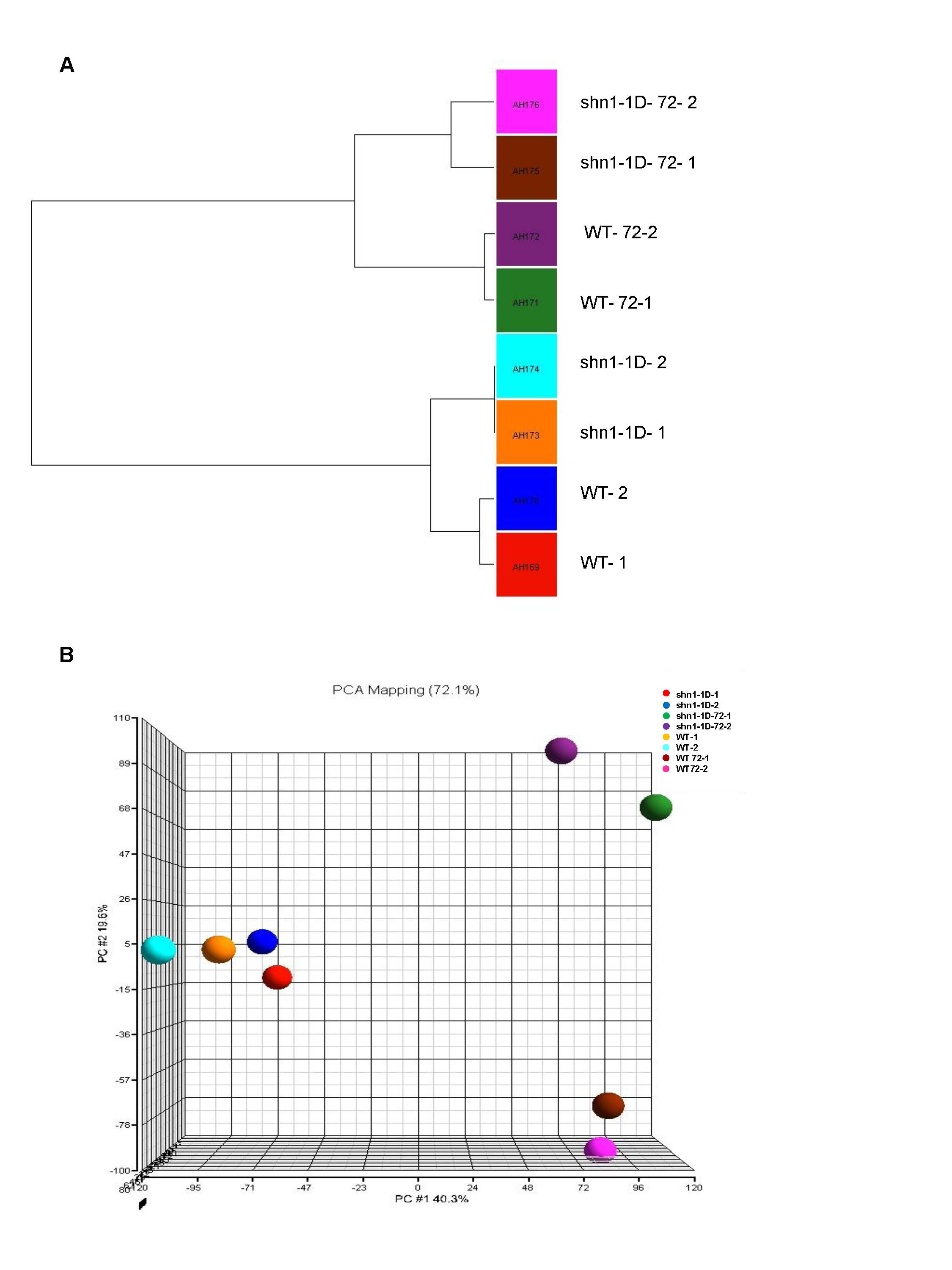

Supplement: Figure S7 — Microarray data analysis. A, Hierarchical clustering and B, PCA. (TIFF) [file pone.0070146.s007.tiff]
