# Supplementary material for: Overexpression of AtSHN1/WIN1 Provokes Unique Defense Responses
Source: PLoS One. 2013 Jul 29;8(7):e70146. doi: 10.1371/journal.pone.0070146 (PMC3726498; doi:10.1371/journal.pone.0070146)
Supplement: Table S6 — Primers sequences used for qRT-PCR. (DOC) [file pone.0070146.s013.doc]

| Tm (°C) | Product Length  (bp) | Sequence 5'-3' | ATG number | Gene  Name |
| --- | --- | --- | --- | --- |
| 59  60 | 81 | F: TCCCATGGAACAGCAGGAAC  R: CCGTCTTTGTATCTTATCGGGATG | AT5G65210 | *TGA1* |
| 58  58 | 81 | F: CAAAGCAGACATCTTCCACAATG  R: ACAGTCGAAGACGATTTCCGA | AT2G39660 | *BIK1* |
| 60  59 | 82 | F: CCTTGCTTAGCCCGTTCACA  R: AAGCGTTTGCAACGGGTAAC | AT1G32640 | *MYC2/JIN1/JAI1* |
| 59  60 | 85 | F: GGTGTCTCGGCATTATCTAGCC  R: CGGCAAAGCACCAGAAGAAA | AT2G37040 | *PAL1* |
| 59  60 | 81 | F: TGAAGTGTTCCGGTCTCGAAA  R: TCTCATCTTCCTCGCTGTGTGAT | AT5G64905 | *PROPEP3* |
| 60  60 | 81 | F: ATCGCCGCATTTATGTTCTCC  R:TGGGACGTCTATATCGATGAACACT | AT1G69930 | *GSTU11* |
| 58  59 | 81 | F: CTTGAAGCGAAGTCACCGAGT  R: GAGGTGGTCTAACACGTTTGGAA | AT1G62760 | *pectinestere inhibitor* |
| 60  60 | 84 | F: GGAGTCGCTGGCATAACACT  R: CCGAATCTCGTCTTGCACTT | AT3G26830 | *PAD3* |
| 58  59 | 81 | F: GTGTGTACCACGCAAGCAAGA  R: TTTCACCAGATCCGCGAGAT | AT4G39670 | *ACD11* |
| 60  60 | 80 | F: TCCGGAGAGATTTCAAGTGC  R: CCATGCGATGTTGCTAGAGA | AT1G32350 | *AOX1D* |
| 58  59 | 81 | F: ACGGCTGAGGCTAATTGTGATAC  R: TTGGGCTTCACAGTCTCTTTCC | AT3G05730 | *DEFL* |
| 60  60 | 84 | F: CGTGCCTGTGGTGTTTATTG  R: GGAACCAGAGAGCGATTGAG | AT4G15680 | *GRXS4* |
| 63  69 | 60 | F:GATCTGAATGTTAAGGCTTTTAGCG R:GGCTTAGATCAGGAAGTGTATAGTCTTG | AT3G01150 | *ATPTB1F* |

Table S5: Primers sequences used for qRT-PCR
