# Supplementary material for: Overexpression of AtSHN1/WIN1 Provokes Unique Defense Responses
Source: PLoS One. 2013 Jul 29;8(7):e70146. doi: 10.1371/journal.pone.0070146 (PMC3726498; doi:10.1371/journal.pone.0070146)
Supplement: Table S5 — Genes significantly differentially regulated in shn1-1D and WT plants 72 h post-inoculation with B. cinerea ( P <0.05) with a fold change (FC) of at least 2, using Limma statistical package. (PDF) [file pone.0070146.s012.pdf]

	wt.72 vs wt.0								
ID	Description	Gene Symb	AGI	FC	logFC	t	P.Value	adj.P.Val	B
257203_at	xyloglucan endotransglycosylase,	---	AT3G23730	-34.26	-5.10	-48.42	8.22E-10	1.87E-05	11.51
254889_at	osmotin precursor ;supported by fu	ATOSM34	AT4G11650	346.86	8.44	43.69	1.64E-09	1.87E-05	11.20
253505_at	Cytochrome P450-like protein flavo	CYP82C2	AT4G31970	45.74	5.52	35.09	7.12E-09	4.06E-05	10.41
245393_at	beta-1,3-glucanase class I precurs	---	AT4G16260	187.96	7.55	30.12	1.98E-08	9.02E-05	9.76
256964_at	unknown protein ;supported by full-	AGP12	AT3G13520	-12.54	-3.65	-29.02	2.54E-08	9.64E-05	9.58
267592_at	unknown protein similar to GP 2245	---	AT2G39710	5.15	2.37	24.71	7.42E-08	0.000167	8.78
260592_at	cellulose synthase catalytic subuni	CSLE1	AT1G55850	12.59	3.65	24.47	7.92E-08	0.000167	8.73
259150_at	unknown protein similar to unknown	---	AT3G10320	6.04	2.60	24.35	8.18E-08	0.000167	8.70
255360_at	putative protein ;supported by full-l	---	AT4G03960	6.87	2.78	24.21	8.50E-08	0.000167	8.67
254833_s_a	copper amine oxidase like protein (	---	AT4G12280	4.98	2.32	23.51	1.03E-07	0.000167	8.52
260101_at	putative trypsin inhibitor similar to tr	---	AT1G73260	15.06	3.91	23.12	1.15E-07	0.000167	8.43
249123_at	beta-ketoacyl-CoA synthase ; supp	KCS20	AT5G43760	-4.14	-2.05	-23.04	1.18E-07	0.000167	8.41
261728_at	pectinesterase, putative similar to p	sks5	AT1G76160	-23.08	-4.53	-22.92	1.22E-07	0.000167	8.38
267256_s_a	putative serine carboxypeptidase I	scpl10	AT2G23000	-10.07	-3.33	-22.81	1.27E-07	0.000167	8.35
263228_at	putative reticuline oxidase-like prot	---	AT1G30700	35.30	5.14	22.72	1.30E-07	0.000167	8.33
267200_at	hypothetical protein predicted by ge	---	AT2G31010	-8.06	-3.01	-22.51	1.38E-07	0.000167	8.28
264923_s_a	peroxiredoxin, putative similar to ty	---	AT1G60740	45.34	5.50	21.68	1.77E-07	0.000196	8.07
258975_at	putative WRKY-like transcriptional r	WRKY45	AT3G01970	7.65	2.93	21.62	1.81E-07	0.000196	8.05
258957_at	feebly-like protein contains similarity	DOX1	AT3G01420	11.34	3.50	21.04	2.17E-07	0.000206	7.90
267201_at	putative protein kinase contains a p	---	AT2G31010	-22.34	-4.48	-21.01	2.18E-07	0.000206	7.89
256598_at	cytochrome P450 homolog, putativ	BR6OX2	AT3G30180	-11.56	-3.53	-20.90	2.26E-07	0.000206	7.86
251323_at	delta-8 sphingolipid desaturase ;su	---	AT3G61580	-5.84	-2.55	-20.01	3.02E-07	0.000265	7.61
265305_at	putative tyrosine decarboxylase	---	AT2G20340	-7.51	-2.91	-19.67	3.38E-07	0.000277	7.51
260602_at	serine acetyltransferase identical to	ATSERAT2	AT1G55920	5.91	2.56	19.65	3.41E-07	0.000277	7.50
261020_at	hypothetical protein similar to reticu	---	AT1G26390	72.71	6.18	19.28	3.86E-07	0.000301	7.39
255885_at	sterol-C-methyltransferase identical	SMT2	AT1G20330	-4.45	-2.15	-18.91	4.39E-07	0.000301	7.27
263475_at	Expressed protein ; supported by fu	---	AT2G31945	5.72	2.52	18.90	4.40E-07	0.000301	7.27
257061_at	hypothetical protein predicted by ge	---	AT3G18250	15.09	3.92	18.79	4.58E-07	0.000301	7.23
245780_at	unknown protein ;supported by full-	---	AT1G45688	-5.65	-2.50	-18.76	4.62E-07	0.000301	7.23
259014_at	putative glucan endo-1-3-beta-gluc	---	AT3G07320	-10.57	-3.40	-18.70	4.72E-07	0.000301	7.21
252048_at	expressed protein supported by cD	---	AT3G52500	-8.10	-3.02	-18.58	4.93E-07	0.000301	7.17
257202_at	receptor-like kinase, putative simila	---	AT3G23750	-6.15	-2.62	-18.52	5.03E-07	0.000301	7.15
264271_at	beta-glucosidase, putative similar t	BGLU6	AT1G60270	-14.31	-3.84	-18.46	5.14E-07	0.000301	7.13

261785_at	hypothetical protein contains similar	---	AT1G08230	3.74	1.90	18.31	5.43E-07	0.00031	7.08
258483_at	putative mannose-6-phosphate isor	MEE31	AT3G02570	-9.09	-3.18	-17.99	6.09E-07	0.000312	6.97
245275_at	beta-amylase ; supported by cDNA:	BAM5	AT4G15210	-33.11	-5.05	-17.93	6.23E-07	0.000312	6.95
252414_at	putative protein sn-glycerol-3-phos	---	AT3G47420	8.97	3.17	17.91	6.29E-07	0.000312	6.95
267318_at	fatty acid hydroxylase (FAH1) ident	FAH1	AT2G34770	-4.96	-2.31	-17.88	6.36E-07	0.000312	6.94
245317_at	hypothetical protein ; supported by	---	AT4G15610	4.40	2.14	17.85	6.42E-07	0.000312	6.93
246200_at	putative protein ;supported by full-le	---	AT4G37240	-15.46	-3.95	-17.78	6.60E-07	0.000312	6.90
260686_at	unknown protein ; supported by cDI	---	AT1G17620	-22.26	-4.48	-17.72	6.75E-07	0.000312	6.88
260327_at	putative RING zinc finger protein P	---	AT1G63840	10.85	3.44	17.69	6.83E-07	0.000312	6.87
247183_at	putative protein strong similarity to	---	AT5G65440	-3.53	-1.82	-17.65	6.93E-07	0.000312	6.86
265233_s_a	hypothetical protein	---	AT2G07718	4.31	2.11	17.57	7.14E-07	0.000312	6.83
253382_at	putative protein AT.I.24, Arabidops	---	AT4G33040	6.05	2.60	17.50	7.33E-07	0.000312	6.80
260667_at	very-long-chain fatty acid condensi	KCS4	AT1G19440	-3.57	-1.84	-17.49	7.34E-07	0.000312	6.80
259958_at	leucine-rich repeat transmembrane	SRF6	AT1G53730	-4.39	-2.13	-17.48	7.37E-07	0.000312	6.80
256376_s_a	unknown protein	PXMT1	AT1G66690	82.21	6.36	17.48	7.38E-07	0.000312	6.80
265066_at	unknown protein location of ESTs	FLA9	AT1G03870	-10.58	-3.40	-17.39	7.62E-07	0.000313	6.77
263083_at	purple acid phosphatase precursor	PAP12	AT2G27190	3.93	1.97	17.37	7.69E-07	0.000313	6.76
261492_at	acid phosphatase, putative similar	SBH2	AT1G14290	-6.18	-2.63	-17.28	7.95E-07	0.000318	6.73
257071_at	unknown protein ; supported by cD	ATCSLC04	AT3G28180	-8.32	-3.06	-16.98	8.94E-07	0.000346	6.62
259072_at	unknown protein similar to unknowr	FLA18	AT3G11700	-6.60	-2.72	-16.92	9.16E-07	0.000348	6.60
265175_at	hypothetical protein similar to putati	ATCSLA03	AT1G23480	-9.58	-3.26	-16.83	9.49E-07	0.000355	6.56
264857_at	putative glycosyl transferase similar	LGT9	AT1G24170	-7.26	-2.86	-16.42	1.12E-06	0.000399	6.41
249093_at	putative protein similar to unknown	---	AT5G43880	-3.06	-1.61	-16.41	1.12E-06	0.000399	6.41
258250_at	putative delta 9 desaturase similar t	FAD5	AT3G15850	-3.07	-1.62	-16.38	1.13E-06	0.000399	6.40
249985_at	Ser/Thr specific protein kinase - lik	---	AT5G18500	-4.97	-2.31	-16.37	1.14E-06	0.000399	6.39
249309_at	homeotic protein BEL1 homolog ; s	BEL1	AT5G41410	4.69	2.23	16.24	1.20E-06	0.000414	6.34
260109_at	hypothetical protein predicted by ge	TET10	AT1G63260	-4.99	-2.32	-16.09	1.27E-06	0.000432	6.28
266184_s_a	phosphate transporter (AtPT2) iden	ATPT2	AT2G38940	15.63	3.97	16.07	1.29E-06	0.000432	6.27
253163_at	putative protein E1B 19K/Bcl-2-inte	---	AT4G35750	6.32	2.66	15.99	1.33E-06	0.000438	6.24
261926_at	unknown protein	PATL2	AT1G22530	-4.31	-2.11	-15.95	1.35E-06	0.000438	6.23
247794_at	phosphoinositide specific phospholi	PLC1	AT5G58670	-4.92	-2.30	-15.92	1.36E-06	0.000438	6.22
262733_s_a	lipase identical to lipase GB:AAA93	---	AT1G28660	4.36	2.12	15.82	1.43E-06	0.000442	6.17
253165_at	putative protein predicted protein, /	---	AT4G35320	-16.48	-4.04	-15.81	1.43E-06	0.000442	6.17
261722_at	acyl-(acyl carrier protein) thioestera	FATB	AT1G08510	-2.98	-1.58	-15.78	1.45E-06	0.000442	6.16
257985_at	hypothetical protein predicted by ge	---	AT3G20810	4.77	2.25	15.77	1.45E-06	0.000442	6.16

267364_at	unknown protein ;supported by full-	ELF4	AT2G40080	4.63	2.21	15.72	1.48E-06	0.000442	6.14
248419_at	putative protein similar to unknown	EXL3	AT5G51550	-9.83	-3.30	-15.67	1.51E-06	0.000442	6.12
253268_s_a	glucosyltransferase -like protein im	UGT73B2	AT4G34131	10.25	3.36	15.65	1.53E-06	0.000442	6.11
245262_at	predicted protein ; supported by cD	---	AT4G16563	-8.52	-3.09	-15.45	1.66E-06	0.000457	6.02
266296_at	putative glutathione S-transferase	ATGSTU7	AT2G29420	4.52	2.18	15.43	1.68E-06	0.000457	6.02
263662_at	ankyrin-like protein EST gb|ATTS09	---	AT1G04430	-8.15	-3.03	-15.42	1.68E-06	0.000457	6.01
253551_at	Expressed protein ; supported by cl	---	AT4G30996	-3.91	-1.97	-15.31	1.77E-06	0.000461	5.97
247597_at	GTP-binding protein - like GTP-bin	AtRABA1f	AT5G60860	-17.08	-4.09	-15.29	1.78E-06	0.000461	5.96
261851_at	hexokinase, putative similar to chlo	HKL1	AT1G50460	-10.38	-3.38	-15.28	1.79E-06	0.000461	5.95
245276_at	DNA-binding homeotic protein Athb	ATHB-2	AT4G16780	-4.29	-2.10	-15.27	1.80E-06	0.000461	5.95
260391_at	putative strictosidine synthase extre	SS2	AT1G74020	4.34	2.12	15.19	1.86E-06	0.00047	5.92
261292_at	hypothetical protein predicted by ge	---	AT1G36940	-4.11	-2.04	-15.08	1.95E-06	0.000479	5.87
261242_at	subtilisin-like serine protease conta	SBT3.3	AT1G32960	8.23	3.04	15.04	1.99E-06	0.000479	5.85
266295_at	tubulin beta-7 chain identical to GB:	TUB7	AT2G29550	-11.66	-3.54	-15.02	2.00E-06	0.000479	5.84
250102_at	receptor-like protein kinase	LRR1	AT5G16590	-4.10	-2.04	-14.98	2.04E-06	0.000479	5.83
260551_at	putative trypsin inhibitor ; supporte	ATTI1	AT2G43510	17.58	4.14	14.97	2.04E-06	0.000479	5.83
247186_at	putative protein contains similarity t	---	AT5G65470	-8.13	-3.02	-14.96	2.06E-06	0.000479	5.82
260431_at	putative zinc finger protein similar to	---	AT1G68190	-2.59	-1.37	-14.95	2.06E-06	0.000479	5.82
256243_at	basic chitinase identical to basic ch	ATHCHIB	AT3G12500	63.47	5.99	14.94	2.07E-06	0.000479	5.81
264449_at	hypothetical protein predicted by ge	NPGR1	AT1G27460	-8.54	-3.09	-14.93	2.08E-06	0.000479	5.81
258856_at	hypothetical protein predicted by ge	SRG3	AT3G02040	14.53	3.86	14.91	2.10E-06	0.00048	5.80
258676_at	hypothetical protein predicted by ge	---	AT3G08600	-3.99	-2.00	-14.81	2.19E-06	0.000491	5.75
257912_at	formin-like protein AHF1 identical to	AFH1	AT3G25500	-14.35	-3.84	-14.73	2.27E-06	0.000504	5.72
257299_at	unknown protein ;supported by full-	---	AT3G28050	3.74	1.90	14.69	2.31E-06	0.000507	5.70
253925_at	putative protein ; supported by cDN	SHV3	AT4G26690	-9.47	-3.24	-14.64	2.37E-06	0.000515	5.68
259611_at	GTP-binding protein RAB7D, putat	AtRABG3d	AT1G52280	-3.63	-1.86	-14.53	2.49E-06	0.000523	5.63
262603_at	hypothetical protein Is a member o	---	AT1G15380	15.94	3.99	14.52	2.50E-06	0.000523	5.63
258791_at	hevein-like protein precursor (PR-4	PR4	AT3G04720	23.75	4.57	14.48	2.54E-06	0.000527	5.61
262803_at	unknown protein Location of ESTs	---	AT1G21000	6.73	2.75	14.33	2.73E-06	0.000556	5.54
248060_at	putative protein contains similarity t	---	AT5G55560	3.16	1.66	14.25	2.82E-06	0.000556	5.51
255016_at	sucrose-phosphate synthase - like	ATSPS4F	AT4G10120	5.94	2.57	14.25	2.83E-06	0.000556	5.51
267497_at	putative glutaredoxin ;supported by	---	AT2G30540	-11.11	-3.47	-14.23	2.85E-06	0.000556	5.50
246932_at	ethylene-responsive element - like	---	AT5G25190	-14.94	-3.90	-14.23	2.85E-06	0.000556	5.50
247982_at	serine O-acetyltransferase (EC 2.3.	ATSERAT1	AT5G56760	-3.28	-1.71	-14.15	2.96E-06	0.000569	5.46
262930_at	hypothetical protein similar to hin1 (	---	AT1G65690	9.68	3.28	14.13	2.99E-06	0.000569	5.45

248704_at	putative protein pectinesterase-like;	sks3	AT5G48450	-4.65	-2.22	-14.07	3.07E-06	0.000576	5.42
253776_at	ADP,ATP carrier-like protein ADP,A	AAC3	AT4G28390	5.81	2.54	14.05	3.10E-06	0.000576	5.42
250110_at	putative protein many predicted pro	---	AT5G15350	-15.97	-4.00	-14.03	3.13E-06	0.000576	5.40
261346_at	putative aspartyl protease contains	---	AT1G79720	-3.29	-1.72	-14.03	3.13E-06	0.000576	5.40
261229_at	RAC-like GTP-binding protein ARAC	ROP2	AT1G20090	-2.52	-1.33	-13.99	3.18E-06	0.000581	5.39
258764_at	putative pectinesterase contains sir	---	AT3G10720	-11.69	-3.55	-13.97	3.22E-06	0.000582	5.38
247717_at	nonspecific lipid-transfer protein pr	LTP3	AT5G59320	-12.21	-3.61	-13.86	3.39E-06	0.000602	5.33
254759_at	short-chain alcohol dehydrogenase	---	AT4G13180	5.49	2.46	13.85	3.40E-06	0.000602	5.32
246682_at	putative protein EXOSTOSIN-1 (PU	XGD1	AT5G33290	3.61	1.85	13.80	3.49E-06	0.000602	5.30
255818_at	hypothetical protein predicted by ge	---	AT2G33570	-23.64	-4.56	-13.79	3.50E-06	0.000602	5.29
263680_at	unknown protein	---	AT1G26930	2.41	1.27	13.78	3.52E-06	0.000602	5.29
263989_at	putative MAP kinase ; supported by	ATMPK20	AT2G42880	-5.21	-2.38	-13.77	3.54E-06	0.000602	5.28
249071_at	putative protein similar to unknown	---	AT5G44050	-15.53	-3.96	-13.72	3.62E-06	0.000608	5.26
262137_at	transcription factor, putative similar	---	AT1G77920	2.85	1.51	13.71	3.64E-06	0.000608	5.25
263499_at	unknown protein ; supported by cD	TTL3	AT2G42580	-6.16	-2.62	-13.64	3.76E-06	0.000608	5.22
249101_at	unknown protein	---	AT5G43580	21.38	4.42	13.63	3.79E-06	0.000608	5.21
255411_at	putative ribonucleoprotein similarity	---	AT4G03110	-3.54	-1.82	-13.60	3.83E-06	0.000608	5.20
263981_at	unknown protein ;supported by full-	PAR1	AT2G42870	-36.61	-5.19	-13.59	3.85E-06	0.000608	5.20
251544_at	putative protein general stress prot	GAUT15	AT3G58790	-5.05	-2.34	-13.59	3.86E-06	0.000608	5.20
262452_at	hypothetical protein similar to hypot	---	AT1G11210	2.96	1.57	13.58	3.86E-06	0.000608	5.19
265414_at	nodulin-like protein	---	AT2G16660	-17.79	-4.15	-13.56	3.91E-06	0.000608	5.18
254363_at	pectinesterase like protein pectines	sks4	AT4G22010	-6.09	-2.61	-13.55	3.92E-06	0.000608	5.18
260581_at	MYB transcription factor (Atmyb2)	MYB2	AT2G47190	4.85	2.28	13.54	3.95E-06	0.000608	5.17
265610_at	unknown protein ;supported by full-	---	AT2G25430	-2.42	-1.27	-13.53	3.97E-06	0.000608	5.17
256293_at	pinhead-like protein similar to pinhe	AGO7	AT1G69440	5.05	2.34	13.44	4.15E-06	0.000618	5.12
257858_at	hypothetical protein predicted by ge	---	AT3G12920	-7.15	-2.84	-13.43	4.16E-06	0.000618	5.12
265444_s_a	aquaporin (plasma membrane intrin	RD28	AT2G37170	-8.79	-3.14	-13.42	4.19E-06	0.000618	5.11
249850_at	putative protein similar to unknown	---	AT5G23240	2.45	1.30	13.41	4.19E-06	0.000618	5.11
264209_at	RAS-related protein, RAB7 identica	RABG3B	AT1G22740	-3.65	-1.87	-13.40	4.22E-06	0.000618	5.11
267040_at	unknown protein	---	AT2G34300	-11.28	-3.50	-13.40	4.23E-06	0.000618	5.10
250657_at	steroid sulfotransferase-like protein	ST2B	AT5G07000	-9.69	-3.28	-13.38	4.26E-06	0.000619	5.10
267260_at	putative proline-rich protein ; suppo	AGP17	AT2G23130	-16.24	-4.02	-13.32	4.39E-06	0.000622	5.07
245025_at	ATPase I subunit	---	---	-2.20	-1.14	-13.31	4.42E-06	0.000622	5.06
248393_at	putative protein contains similarity t	ATBAG1	AT5G52060	-5.86	-2.55	-13.29	4.44E-06	0.000622	5.05
253405_at	transcription factor TINY homolog t	---	AT4G32800	-7.09	-2.82	-13.27	4.49E-06	0.000622	5.04

265771_at	unknown protein ;supported by full-	---	AT2G48030	-3.67	-1.88	-13.27	4.49E-06	0.000622	5.04
252102_at	dehydrin Xero2 ; supported by cDN	LTI30	AT3G50970	-24.86	-4.64	-13.26	4.53E-06	0.000622	5.03
245905_at	putative protein predicted proteins,	---	AT5G11090	3.04	1.61	13.23	4.59E-06	0.000622	5.02
252993_at	monooxygenase 2 (MO2)	---	AT4G38540	8.86	3.15	13.21	4.62E-06	0.000622	5.01
253407_at	putative protein	---	AT4G32920	3.09	1.63	13.20	4.64E-06	0.000622	5.01
265920_s_a	unknown protein	---	AT2G15120	14.20	3.83	13.20	4.67E-06	0.000622	5.00
252167_at	putative protein steroid dehydroger	---	AT3G50560	-3.31	-1.73	-13.18	4.70E-06	0.000622	5.00
267175_s_a	actin 3 identical to GB:U29480;supp	ACT1	AT2G37620	-4.49	-2.17	-13.18	4.71E-06	0.000622	5.00
259848_at	leucine-rich receptor-like protein ki	---	AT1G72180	-3.76	-1.91	-13.17	4.72E-06	0.000622	4.99
254815_at	putative pollen-specific protein poll	SKU5	AT4G12420	-6.91	-2.79	-13.17	4.72E-06	0.000622	4.99
254110_at	putative protein pectinesterase - Ci	---	AT4G25260	-13.84	-3.79	-13.16	4.75E-06	0.000623	4.99
251516_s_a	putative protein hypothetical protei	---	AT3G59310	-3.13	-1.65	-13.07	4.97E-06	0.000646	4.94
267162_s_a	putative phosphoribosylaminoimida	---	AT2G05140	4.99	2.32	13.06	4.99E-06	0.000646	4.94
258468_at	unknown protein predicted by gens	---	AT3G06070	-13.12	-3.71	-13.00	5.15E-06	0.000659	4.91
259598_at	sphingosine-1-phosphate lyase, pu	DPL1	AT1G27980	3.36	1.75	12.97	5.22E-06	0.000662	4.89
265057_at	unknown protein ;supported by full-	---	AT1G52140	-3.00	-1.58	-12.97	5.22E-06	0.000662	4.89
252265_at	putative protein SRG1 protein - Ara	DIN11	AT3G49620	69.59	6.12	12.91	5.39E-06	0.000672	4.86
246071_at	ids4-like protein ids-4 protein - Hor	SPX1	AT5G20150	16.08	4.01	12.89	5.42E-06	0.000672	4.85
260784_at	MYB-related protein identical to GE	ATMYB13	AT1G06180	8.39	3.07	12.89	5.42E-06	0.000672	4.85
263421_at	unknown protein ;supported by full-	EXL5	AT2G17230	-11.40	-3.51	-12.89	5.44E-06	0.000672	4.85
251791_at	expansin-like protein expansin At-E	ATEXPA16	AT3G55500	-11.84	-3.57	-12.88	5.45E-06	0.000672	4.85
266215_at	putative endoxyloglucan glycosyltra	EXGT-A1	AT2G06850	-23.00	-4.52	-12.80	5.69E-06	0.00069	4.80
257746_at	chorismate mutase identical to chor	CM1	AT3G29200	2.49	1.32	12.73	5.88E-06	0.000698	4.77
257196_at	AMP-binding protein, putative simil	---	AT3G23790	3.70	1.89	12.73	5.90E-06	0.000698	4.77
261727_at	S-adenosyl-methionine-sterol-C-m	SMT3	AT1G76090	-6.95	-2.80	-12.69	6.02E-06	0.0007	4.75
250505_at	cellulose synthase catalytic subunit	CESA5	AT5G09870	-4.34	-2.12	-12.68	6.05E-06	0.0007	4.74
247440_at	peptide transporter	---	AT5G62680	2.55	1.35	12.65	6.14E-06	0.000703	4.73
266962_at	hypothetical protein predicted by ge	---	AT2G39435	2.78	1.47	12.64	6.16E-06	0.000703	4.72
247360_at	cytochrome P450-like protein ;supp	CYP94B1	AT5G63450	-19.41	-4.28	-12.59	6.34E-06	0.000709	4.69
255064_at	putative phi-1-like phosphate-induc	EXO	AT4G08950	-50.23	-5.65	-12.58	6.37E-06	0.000709	4.69
262291_at	zinc finger and C2 domain protein,	---	AT1G70790	2.49	1.31	12.57	6.39E-06	0.000709	4.69
255714_at	contains weak similarity to S. cerev	---	AT4G00300	-3.09	-1.63	-12.57	6.41E-06	0.000709	4.68
256116_at	unknown protein contains similarity	CPuORF55	AT1G16858	-3.11	-1.64	-12.55	6.46E-06	0.000711	4.67
261005_at	hypothetical protein similar to reticu	---	AT1G26420	17.27	4.11	12.54	6.49E-06	0.000711	4.67
262970_at	unknown protein	---	AT1G75690	-2.72	-1.44	-12.49	6.68E-06	0.000729	4.64

252890_at	brassinosteroid insensitive 1 gene (	BRI1	AT4G39400	-2.53	-1.34	-12.47	6.73E-06	0.000731	4.63
251768_at	phosphoinositide-specific phospho	---	AT3G55940	-6.46	-2.69	-12.44	6.83E-06	0.000737	4.62
254667_at	glycine-rich cell wall protein-like gly	---	AT4G18280	-7.27	-2.86	-12.44	6.85E-06	0.000737	4.61
255433_at	putative xyloglucan endotransglyco	XTH9	AT4G03210	-9.91	-3.31	-12.42	6.92E-06	0.000738	4.60
250413_at	adenine phosphoribosyltransferase	APT5	AT5G11160	-4.40	-2.14	-12.41	6.96E-06	0.000738	4.60
263866_at	putative farnesylated protein ;supp	---	AT2G36950	4.98	2.32	12.40	6.99E-06	0.000738	4.59
264300_at	gamma glutamyl hydrolase, putativ	ATGGH3	AT1G78670	2.62	1.39	12.37	7.10E-06	0.000739	4.58
262229_at	unknown protein ; supported by cDI	---	AT1G68620	15.50	3.95	12.35	7.18E-06	0.000739	4.57
253879_s_a	UDP rhamnose--anthocyanidin-3-g	---	AT4G27560	-7.98	-3.00	-12.34	7.19E-06	0.000739	4.57
263867_at	putative aquaporin (tonoplast intrins	GAMMA-TI	AT2G36830	-7.41	-2.89	-12.34	7.19E-06	0.000739	4.56
261023_at	unknown protein similar to unknowr	---	AT1G12200	3.23	1.69	12.32	7.28E-06	0.000742	4.55
259132_at	putative auxin-independent growth	---	AT3G02250	-4.33	-2.11	-12.30	7.37E-06	0.000743	4.54
248466_at	putative protein similar to unknown	HVA22E	AT5G50720	-5.76	-2.53	-12.29	7.39E-06	0.000743	4.54
263948_at	similar to harpin-induced protein hir	YLS9	AT2G35980	16.76	4.07	12.29	7.41E-06	0.000743	4.53
266946_at	putative protein kinase contains a p	---	AT2G18890	-3.08	-1.62	-12.28	7.43E-06	0.000743	4.53
246595_at	formate dehydrogenase (FDH) ;su	FDH	AT5G14780	2.61	1.38	12.22	7.69E-06	0.000759	4.50
264770_at	unknown protein similar to unknowr	---	AT1G23030	-8.06	-3.01	-12.21	7.73E-06	0.00076	4.49
248721_at	putative protein strong similarity to u	GAUT4	AT5G47780	-2.08	-1.06	-12.16	7.92E-06	0.00077	4.47
267212_at	similar to late embryogenesis abun	---	AT2G44060	-2.37	-1.25	-12.11	8.14E-06	0.00078	4.44
251036_at	putative protein ; supported by cDN	---	AT5G02160	-2.95	-1.56	-12.11	8.14E-06	0.00078	4.44
248701_at	ligand-gated ion channel protein-like	GLR1.3	AT5G48410	2.70	1.43	12.03	8.52E-06	0.000802	4.39
260254_at	putative glycerophosphodiester pho	---	AT1G74210	2.53	1.34	12.01	8.58E-06	0.000802	4.38
265433_at	unknown protein	---	AT2G20950	3.66	1.87	12.01	8.59E-06	0.000802	4.38
256751_at	CLC-b chloride channel protein ide	CLC-B	AT3G27170	-7.25	-2.86	-11.98	8.74E-06	0.000804	4.36
263319_at	putative anion exchange protein ide	BOR1	AT2G47160	-6.67	-2.74	-11.97	8.78E-06	0.000804	4.36
251575_at	putative protein basic leucine zippe	BZIP61	AT3G58120	-14.36	-3.84	-11.96	8.81E-06	0.000804	4.36
261252_at	RAS-related protein ARA-1 identica	RABA5E	AT1G05810	-3.38	-1.76	-11.96	8.85E-06	0.000804	4.35
250180_at	early nodule-specific protein - like e	---	AT5G14450	-2.58	-1.37	-11.92	9.01E-06	0.000814	4.33
258386_at	hypothetical protein predicted by ge	---	AT3G15520	-4.00	-2.00	-11.90	9.14E-06	0.000821	4.32
247072_at	putative protein similar to unknown	---	AT5G66490	4.33	2.11	11.89	9.18E-06	0.000822	4.31
250665_at	unknown protein ;supported by full-	---	AT5G06980	-3.36	-1.75	-11.88	9.23E-06	0.000822	4.31
266424_at	unknown protein	---	AT2G41330	-4.93	-2.30	-11.86	9.32E-06	0.000822	4.30
250261_at	peptide transporter - like protein pe	---	AT5G13400	-7.58	-2.92	-11.85	9.37E-06	0.000822	4.29
260783_at	ethylene response factor, putative	ORA59	AT1G06160	18.53	4.21	11.83	9.49E-06	0.000823	4.28
245809_at	squalene monooxygenase, putative	XF1	AT1G58440	-3.08	-1.62	-11.77	9.77E-06	0.000838	4.25

247005_at	adenylylsulfate kinase-like protein	---	AT5G67520	-3.61	-1.85	-11.76	9.81E-06	0.000838	4.25
247161_at	putative protein similar to unknown	---	AT5G65810	-3.26	-1.70	-11.73	9.99E-06	0.000843	4.23
259410_at	hypothetical protein predicted by ge	---	AT1G13340	11.29	3.50	11.73	1.00E-05	0.000843	4.22
264471_at	hypothetical protein predicted by ge	---	AT1G67120	5.46	2.45	11.72	1.00E-05	0.000843	4.22
263876_at	putative RAS superfamily GTP-bind	ATRAB7A	AT2G21880	-3.72	-1.90	-11.72	1.00E-05	0.000843	4.22
245018_at	hypothetical protein	---	---	-2.24	-1.16	-11.72	1.01E-05	0.000843	4.22
245193_at	F12A21.6 hypothetical protein	SUFE2	AT1G67810	30.52	4.93	11.68	1.03E-05	0.000843	4.20
261006_at	unknown protein similar to reticuline	---	AT1G26410	10.01	3.32	11.67	1.03E-05	0.000843	4.19
249454_at	expressed protein predicted protein	---	AT5G39520	24.02	4.59	11.67	1.03E-05	0.000843	4.19
249377_at	putative protein predicted proteins,	---	AT5G40690	5.67	2.50	11.67	1.04E-05	0.000843	4.19
259232_at	unknown protein similar to hypothet	---	AT3G11420	-4.49	-2.17	-11.66	1.04E-05	0.000843	4.19
246995_at	formin-like protein	---	AT5G67470	-4.72	-2.24	-11.66	1.04E-05	0.000843	4.19
254785_at	putative pollen surface protein end	FLA2	AT4G12730	-53.32	-5.74	-11.64	1.05E-05	0.000844	4.17
246510_at	cyclic nucleotide-gated cation chan	DND1	AT5G15410	-2.85	-1.51	-11.64	1.05E-05	0.000844	4.17
259671_at	protein kinase, putative similar to G	---	AT1G52290	-9.09	-3.18	-11.63	1.05E-05	0.000844	4.17
244926_s_a	maturase	---	AT2G07711	3.07	1.62	11.60	1.07E-05	0.000845	4.15
246098_at	ethylene-forming-enzyme-like dioxy	---	AT5G20400	4.65	2.22	11.58	1.08E-05	0.000845	4.14
264772_at	unknown protein location of EST gl	---	AT1G22930	2.68	1.42	11.58	1.09E-05	0.000845	4.14
258750_at	putative pectinacetylesterase simila	---	AT3G05910	-9.47	-3.24	-11.57	1.09E-05	0.000845	4.13
246820_at	putative protein predicted proteins,	---	AT5G26910	-2.10	-1.07	-11.53	1.12E-05	0.000857	4.11
258158_at	acid phosphatase type 5 identical to	PAP17	AT3G17790	16.15	4.01	11.53	1.12E-05	0.000857	4.11
267127_at	putative acetone-cyanohydrin lyase	MES3	AT2G23610	-2.69	-1.43	-11.51	1.13E-05	0.00086	4.10
248046_at	receptor protein kinase-like protein	---	AT5G56040	-3.52	-1.81	-11.51	1.13E-05	0.00086	4.10
252940_at	receptor protein kinase - like protei	---	AT4G39270	4.67	2.22	11.49	1.14E-05	0.00086	4.09
266956_at	unknown protein	---	AT2G34510	-10.85	-3.44	-11.48	1.15E-05	0.000864	4.08
261762_at	hypothetical protein predicted by ge	---	AT1G15510	5.82	2.54	11.44	1.17E-05	0.000865	4.06
258467_at	unknown protein contains Pfam pro	---	AT3G06060	-2.87	-1.52	-11.44	1.18E-05	0.000865	4.06
254234_at	putative major latex protein major l	---	AT4G23680	15.71	3.97	11.42	1.19E-05	0.00087	4.05
267170_at	putative RING zinc finger protein	---	AT2G37585	-5.22	-2.39	-11.41	1.20E-05	0.000871	4.04
245947_at	spermine synthase (ACL5) ;suppor	ACL5	AT5G19530	-4.79	-2.26	-11.38	1.21E-05	0.00088	4.03
264313_at	carbonic anhydrase, putative simila	BCA4	AT1G70410	-2.10	-1.07	-11.37	1.22E-05	0.00088	4.02
249410_at	receptor-like protein kinase recepto	---	AT5G40380	-5.99	-2.58	-11.35	1.24E-05	0.000885	4.01
264479_at	hypothetical protein similar to recep	---	AT1G77280	2.82	1.50	11.35	1.24E-05	0.000885	4.01
247063_at	unknown protein	---	AT5G66820	3.97	1.99	11.34	1.25E-05	0.000888	4.00
261658_at	hypothetical protein predicted by ge	---	AT1G50040	-13.27	-3.73	-11.33	1.25E-05	0.00089	3.99

252000_at	hypothetical protein predicted prote	---	AT3G52710	3.11	1.64	11.30	1.27E-05	0.000897	3.98
248968_at	pectin acetylesterase ;supported by	---	AT5G45280	-6.35	-2.67	-11.28	1.29E-05	0.000903	3.96
261117_at	unknown protein	AUL1	AT1G75310	-4.34	-2.12	-11.27	1.30E-05	0.000908	3.96
266712_at	unknown protein	---	AT2G46750	3.02	1.59	11.21	1.34E-05	0.00092	3.92
250939_at	putative protein various predicted p	iqd2	AT5G03040	-4.15	-2.05	-11.20	1.34E-05	0.00092	3.92
255662_at	putative protein	GPAT8	AT4G00400	-6.24	-2.64	-11.20	1.35E-05	0.00092	3.92
244982_at	ribosomal protein L14	---	---	-2.84	-1.51	-11.20	1.35E-05	0.00092	3.92
251200_at	putative protein PrMC3, Pinus radia	GID1B	AT3G63010	3.24	1.69	11.19	1.35E-05	0.00092	3.91
259990_s_a	F-box protein FKF1/ADO3, AtFBX2	FKF1	AT1G68050	4.00	2.00	11.19	1.36E-05	0.00092	3.91
250125_at	biotin carboxyl carrier protein of ace	CAC1	AT5G16390	-2.48	-1.31	-11.19	1.36E-05	0.00092	3.91
254434_at	ethylene-regulated transcript 2 (ER	---	AT4G20880	2.28	1.19	11.18	1.36E-05	0.00092	3.91
245008_at	hypothetical protein	---	---	-2.20	-1.14	-11.17	1.37E-05	0.000921	3.90
261708_at	unknown protein similar to hypothet	---	AT1G32740	2.32	1.21	11.16	1.38E-05	0.000922	3.89
254484_at	hypothetical protein	---	AT4G20760	-2.29	-1.19	-11.16	1.38E-05	0.000922	3.89
248366_at	SCARECROW transcriptional regul	---	AT5G52510	2.88	1.52	11.15	1.39E-05	0.000922	3.89
248879_at	ornithine aminotransferase	DELTA-OA	AT5G46180	3.06	1.61	11.14	1.40E-05	0.000922	3.88
251141_at	Expressed protein ; supported by fu	---	AT5G01075	-5.37	-2.42	-11.11	1.42E-05	0.000924	3.87
265823_at	hypothetical protein predicted by ge	---	AT2G35760	-2.42	-1.27	-11.11	1.42E-05	0.000924	3.86
262868_at	unknown protein	---	AT1G64980	-4.06	-2.02	-11.10	1.43E-05	0.000924	3.86
247338_at	pyruvate kinase	---	AT5G63680	2.39	1.26	11.09	1.44E-05	0.000926	3.85
266682_at	putative disease resistance protein	---	AT2G19780	-4.83	-2.27	-11.07	1.45E-05	0.000931	3.84
262910_at	unknown protein ;supported by full-	---	AT1G59710	-3.38	-1.76	-11.05	1.47E-05	0.000939	3.83
253273_at	putative protein hypothetical protei	---	AT4G34180	2.21	1.14	11.05	1.47E-05	0.000939	3.83
250287_at	putative protein AP2 domain conta	Rap2.6L	AT5G13330	3.86	1.95	11.03	1.49E-05	0.000946	3.81
256441_at	unknown protein ;supported by full-	---	AT3G10940	-5.21	-2.38	-11.01	1.50E-05	0.000949	3.80
247979_at	pollen specific protein SF21 ;suppo	---	AT5G56750	-2.81	-1.49	-11.00	1.51E-05	0.000949	3.80
253489_at	monogalactosyldiacylglycerol synth	MGD1	AT4G31780	-3.69	-1.89	-11.00	1.51E-05	0.000949	3.80
249037_at	putative protein contains similarity t	FLA13	AT5G44130	-20.72	-4.37	-11.00	1.51E-05	0.000949	3.80
252365_at	Expressed protein ; supported by fu	---	AT3G48350	-8.54	-3.09	-10.99	1.52E-05	0.000949	3.79
250618_at	putative protein hypothetical protein	ATBAG3	AT5G07220	-2.25	-1.17	-10.95	1.56E-05	0.000963	3.77
253631_at	nucleotide sugar epimerase-like pr	GAE1	AT4G30440	-3.12	-1.64	-10.94	1.56E-05	0.000964	3.76
263606_at	putative beta-ketoacyl-CoA synthas	KCS9	AT2G16280	-3.72	-1.89	-10.93	1.58E-05	0.000966	3.75
245274_at	ankyrin like protein ; supported by c	---	AT4G14360	-2.68	-1.42	-10.93	1.58E-05	0.000966	3.75
259553_x_a	hypothetical protein	ATEXT3	AT1G21310	8.99	3.17	10.90	1.61E-05	0.000978	3.73
245528_at	pyruvate,orthophosphate dikinase	PPDK	AT4G15530	2.94	1.55	10.88	1.63E-05	0.00098	3.72

258264_at	unknown protein	MBD11	AT3G15790	-2.89	-1.53	-10.88	1.63E-05	0.00098	3.72
253891_at	putative protein ;supported by full-l	---	AT4G27720	-5.99	-2.58	-10.87	1.63E-05	0.00098	3.72
260914_at	beta-xylosidase, putative similar to	BXL2	AT1G02640	-9.61	-3.26	-10.85	1.65E-05	0.000986	3.71
254293_at	putative protein SF16 protein, Helia	IQD22	AT4G23060	-5.46	-2.45	-10.85	1.65E-05	0.000986	3.71
267319_at	ABC transporter (AtMRP2) identica	ATMRP2	AT2G34660	3.43	1.78	10.85	1.65E-05	0.000986	3.71
249765_at	putative protein contains similarity t	SLAH3	AT5G24030	-17.46	-4.13	-10.83	1.67E-05	0.000991	3.69
258751_at	low temperature and salt responsive	RCI2B	AT3G05890	-19.22	-4.26	-10.82	1.68E-05	0.000992	3.69
255110_at	peroxidase C2 precursor like prote	---	AT4G08770	75.94	6.25	10.81	1.69E-05	0.000994	3.68
266168_at	putative protease inhibitor ;support	---	AT2G38870	14.72	3.88	10.81	1.69E-05	0.000996	3.68
245859_at	serine (threonine) protein kinase - I	ATNEK3	AT5G28290	-4.07	-2.03	-10.80	1.70E-05	0.000996	3.67
245026_at	ATPase III subunit	---	---	-2.15	-1.11	-10.80	1.70E-05	0.000996	3.67
251864_at	pectate lyase - like protein pectate	PMR6	AT3G54920	-2.96	-1.56	-10.76	1.74E-05	0.001013	3.65
247461_at	putative protein various predicted p	ATBAG2	AT5G62100	-3.00	-1.58	-10.75	1.75E-05	0.001013	3.64
253994_at	protein phosphatase ABI1 ; suppor	ABI1	AT4G26080	2.41	1.27	10.75	1.76E-05	0.001014	3.64
249481_at	frnE protein - like frnE protein, Deir	---	AT5G38900	29.10	4.86	10.73	1.77E-05	0.001014	3.63
249008_at	putative protein contains similarity t	---	AT5G44680	-4.52	-2.18	-10.73	1.77E-05	0.001014	3.63
256578_at	peroxidase, putative similar to perc	---	AT3G28200	-5.43	-2.44	-10.73	1.78E-05	0.001015	3.63
261021_at	hypothetical protein similar to reticu	---	AT1G26380	52.43	5.71	10.70	1.81E-05	0.001028	3.61
266489_at	unknown protein ; supported by cD	NPSN11	AT2G35190	-4.20	-2.07	-10.68	1.83E-05	0.001029	3.60
245915_s_a	tubulin alpha-5 chain-like protein tu	TUA3	AT5G19770	-2.38	-1.25	-10.68	1.83E-05	0.001029	3.60
251413_at	bZIP protein ; supported by cDNA:	---	AT3G60320	-2.85	-1.51	-10.68	1.83E-05	0.001029	3.60
259010_at	unknown protein contains helix-loop	---	AT3G07340	-4.05	-2.02	-10.68	1.83E-05	0.001029	3.60
249383_at	putative protein putative DNA-bindi	PRE1	AT5G39860	-4.56	-2.19	-10.65	1.86E-05	0.001039	3.58
253811_at	hypothetical protein ;supported by	ULT1	AT4G28190	-5.61	-2.49	-10.62	1.90E-05	0.00105	3.56
255430_at	putative chloroplast protein import	tic20-IV	AT4G03320	5.47	2.45	10.60	1.91E-05	0.001054	3.55
246482_at	plant adhesion molecule 1 (PAM1)	PAM1	AT5G15930	-2.28	-1.19	-10.60	1.92E-05	0.001054	3.55
261017_at	UDP-glucose dehydrogenase, puta	UGD1	AT1G26570	-2.61	-1.38	-10.60	1.92E-05	0.001054	3.55
260856_at	TINY-like protein similar to TINY GE	---	AT1G21910	-33.68	-5.07	-10.58	1.94E-05	0.001059	3.54
259858_at	putative receptor kinase similar to r	---	AT1G68400	-4.88	-2.29	-10.56	1.96E-05	0.001066	3.52
253841_at	putative beta-glucosidase beta-glu	BGLU10	AT4G27830	3.45	1.78	10.56	1.97E-05	0.001067	3.52
254726_at	isoflavone reductase-like protein is	PRR2	AT4G13660	7.95	2.99	10.54	1.99E-05	0.001071	3.51
253224_at	invertase - like protein mRNA, Liliu	---	AT4G34860	3.71	1.89	10.52	2.02E-05	0.001082	3.50
261420_at	fatty acid elongase 3-ketoacyl-CoA	KCS3	AT1G07720	-6.29	-2.65	-10.51	2.03E-05	0.001082	3.49
261611_at	hypothetical protein predicted by ge	---	AT1G49730	-4.96	-2.31	-10.51	2.03E-05	0.001082	3.49
259642_at	hypothetical protein predicted by ge	---	AT1G69030	-3.58	-1.84	-10.50	2.03E-05	0.001082	3.49

259104_at	unknown protein ;supported by full-	LNG2	AT3G02170	-4.40	-2.14	-10.50	2.04E-05	0.001082	3.49
260000_at	unknown protein similar to C-termi	ATMAP70-	AT1G68060	-2.93	-1.55	-10.49	2.06E-05	0.001088	3.48
261563_at	polyphosphoinositide binding prote	---	AT1G01630	-2.52	-1.33	-10.45	2.10E-05	0.001106	3.45
248921_at	GDSL-motif lipase/hydrolase-like pr	---	AT5G45950	-9.30	-3.22	-10.44	2.11E-05	0.00111	3.45
251601_at	Expressed protein ; supported by fu	---	AT3G57800	-3.92	-1.97	-10.43	2.12E-05	0.001114	3.44
253253_at	putative protein auxin-regulated ge	---	AT4G34750	-2.27	-1.18	-10.42	2.13E-05	0.001116	3.44
254770_at	extensin-like protein extensin-like p	---	AT4G13340	-7.84	-2.97	-10.42	2.14E-05	0.001116	3.43
253911_at	putative receptor protein kinase re	---	AT4G27300	6.98	2.80	10.41	2.15E-05	0.001117	3.43
247123_at	unknown protein	---	AT5G66050	-3.38	-1.76	-10.39	2.18E-05	0.001119	3.41
246449_at	hypothetical protein	---	AT5G16810	-2.47	-1.31	-10.38	2.19E-05	0.001119	3.41
245740_at	amino acid permease, putative alm	AAP5	AT1G44100	2.92	1.54	10.38	2.19E-05	0.001119	3.41
253330_at	putative potassium transporter AtK	KUP5	AT4G33530	-2.19	-1.13	-10.37	2.20E-05	0.001119	3.40
263957_at	unknown protein ; supported by cD	---	AT2G35880	-2.10	-1.07	-10.34	2.24E-05	0.001129	3.38
264833_at	putative protein phosphatase simila	---	AT1G03590	-3.76	-1.91	-10.33	2.27E-05	0.001137	3.37
258515_at	ATP citrate lyase, putative similar t	ACLB-1	AT3G06650	-2.12	-1.09	-10.30	2.31E-05	0.001146	3.35
248179_at	receptor-protein kinase-like protein	THE1	AT5G54380	-6.97	-2.80	-10.29	2.32E-05	0.001146	3.35
259995_at	putative golgi transport complex pr	---	AT1G67930	-2.73	-1.45	-10.29	2.32E-05	0.001146	3.35
245816_at	hypothetical protein similar to hypot	---	AT1G26210	-6.24	-2.64	-10.27	2.34E-05	0.001146	3.34
257650_at	protein phosphatase, putative simil	---	AT3G16800	-3.04	-1.61	-10.27	2.34E-05	0.001146	3.34
264704_at	unknown protein similar to putative	LGT8	AT1G70090	-4.02	-2.01	-10.27	2.34E-05	0.001146	3.34
259766_at	unknown protein ;supported by full-	---	AT1G64360	4.45	2.15	10.27	2.35E-05	0.001146	3.33
258087_at	putative peroxiredoxin similar to pe	ATPRX Q	AT3G26060	-3.50	-1.81	-10.27	2.35E-05	0.001146	3.33
246968_at	RING finger-like protein RING-H2 f	---	AT5G24870	2.99	1.58	10.23	2.41E-05	0.001165	3.31
253496_at	glutathione peroxidase - like protei	ATGPX7	AT4G31870	-12.08	-3.59	-10.21	2.44E-05	0.001175	3.29
260015_at	putative S-adenosyl-L-methionine:t	CCOAMT	AT1G67980	25.96	4.70	10.19	2.46E-05	0.001179	3.29
256930_at	O-acetylserine(thiol) lyase, putative	OASA2	AT3G22460	2.70	1.43	10.19	2.46E-05	0.001179	3.29
255283_at	putative symbiosis-related protein ;	ATG8B	AT4G04620	3.41	1.77	10.17	2.50E-05	0.001192	3.27
253428_at	cellulose synthase catalytic subunit	CESA1	AT4G32410	-3.06	-1.61	-10.16	2.52E-05	0.001192	3.26
256787_at	beta-fructofuranosidase 1 identical	ATBFRUCT	AT3G13790	4.95	2.31	10.16	2.52E-05	0.001192	3.26
259915_at	unknown protein	---	AT1G72790	-7.44	-2.89	-10.16	2.52E-05	0.001192	3.26
248943_s_a	putative protein similar to unknown	---	AT5G45440	-6.24	-2.64	-10.15	2.53E-05	0.001195	3.26
250954_at	putative protein ;supported by full-l	ATRAB8C	AT5G03520	-2.91	-1.54	-10.14	2.55E-05	0.001197	3.25
257636_at	cytochrome P450, putative contain	CYP71B22	AT3G26200	6.93	2.79	10.14	2.55E-05	0.001197	3.25
246596_at	CARBONIC ANHYDRASE 2 ; sup	CA2	AT5G14740	-2.19	-1.13	-10.13	2.56E-05	0.001197	3.25
260877_at	unknown protein ; supported by full-	---	AT1G21500	-3.25	-1.70	-10.13	2.56E-05	0.001197	3.25

257772_at	unknown protein C-term similar to p	---	AT3G23080	-2.55	-1.35	-10.13	2.56E-05	0.001197	3.24
247954_at	beta-galactosidase (emb|CAB6474(	BGAL4	AT5G56870	2.67	1.42	10.08	2.64E-05	0.001218	3.21
246843_at	putative protein various predicted p	---	AT5G26740	-2.09	-1.07	-10.08	2.64E-05	0.001218	3.21
248082_at	fimbrin	---	AT5G55400	-4.26	-2.09	-10.07	2.65E-05	0.001221	3.21
253099_s_a	peroxidase - like protein peroxidase	---	AT4G37520	2.58	1.37	10.06	2.67E-05	0.001223	3.20
259523_at	hypothetical protein	---	AT1G12500	-4.09	-2.03	-10.06	2.68E-05	0.001223	3.20
256396_at	unknown protein	---	AT3G06150	-4.19	-2.07	-10.05	2.69E-05	0.001225	3.19
251342_at	putative protein SAUR-AC1, Arabic	---	AT3G60690	3.07	1.62	10.05	2.69E-05	0.001225	3.19
253050_at	putative protein probable arabinoga	AGP18	AT4G37450	-13.47	-3.75	-10.05	2.70E-05	0.001225	3.19
261191_at	starch synthase, putative similar to	---	AT1G32900	-5.02	-2.33	-10.04	2.72E-05	0.001225	3.18
253519_at	predicted protein red-1 gene, M.mu	---	AT4G31240	4.89	2.29	10.03	2.72E-05	0.001225	3.18
250936_at	putative protein ;supported by full-l	---	AT5G03120	-5.03	-2.33	-10.03	2.73E-05	0.001225	3.18
245671_at	purine permease identical to purine	PUP1	AT1G28230	2.11	1.08	10.03	2.73E-05	0.001225	3.18
259058_at	putative cytochrome P450 similar to	CYP89A9	AT3G03470	6.36	2.67	10.03	2.73E-05	0.001225	3.18
247596_at	putative protein predicted protein, I	---	AT5G60840	-4.36	-2.12	-10.02	2.75E-05	0.001226	3.17
246097_at	putative protein CGI-45 protein - H	HHP1	AT5G20270	-7.99	-3.00	-10.01	2.75E-05	0.001226	3.17
263131_at	ribosomal protein L13, putative sim	emb1473	AT1G78630	-2.21	-1.14	-10.01	2.76E-05	0.001226	3.17
255969_at	Expressed protein ; supported by fu	---	AT1G22330	-5.05	-2.34	-9.99	2.79E-05	0.001234	3.15
259911_at	putative cinnamyl-alcohol dehydrog	CAD1	AT1G72680	3.25	1.70	9.99	2.79E-05	0.001234	3.15
253590_at	putative protein various predicted p	HHP2	AT4G30850	-3.01	-1.59	-9.97	2.83E-05	0.001239	3.14
257194_at	serine acetyltransferase (Sat-1) ide	ATSERAT2	AT3G13110	2.32	1.22	9.97	2.83E-05	0.001239	3.14
244901_at	hypothetical protein	---	ATMG00640	2.45	1.29	9.97	2.84E-05	0.001239	3.14
251701_at	putative protein hypothetical protei	---	AT3G56650	-2.35	-1.23	-9.96	2.86E-05	0.001239	3.13
252885_at	glycine-rich protein (clone AtGRP8)	GR-RBP8	AT4G39260	3.37	1.75	9.95	2.86E-05	0.001239	3.13
265471_at	putative peroxidase ATP2a ;suppo	---	AT2G37130	7.13	2.83	9.95	2.87E-05	0.001239	3.13
255889_at	ABC transporter, putative similar to	WBC11	AT1G17840	-3.10	-1.63	-9.95	2.87E-05	0.001239	3.12
249693_at	histidine kinase-like protein ; suppo	AHK2	AT5G35750	-3.82	-1.94	-9.90	2.96E-05	0.001264	3.09
261150_at	floral nectary-specific protein, putat	JMT	AT1G19640	-16.83	-4.07	-9.90	2.96E-05	0.001264	3.09
254016_at	putative transcription factor Arabido	CGA1	AT4G26150	-4.28	-2.10	-9.88	3.00E-05	0.001277	3.08
260347_at	DHHC-type zinc finger protein, puta	---	AT1G69420	-3.60	-1.85	-9.87	3.02E-05	0.00128	3.07
253835_at	putative beta-glucosidase beta-glu	BGLU9	AT4G27820	2.44	1.29	9.86	3.03E-05	0.001283	3.07
247552_at	phytochelatin synthetase - like prot	COB	AT5G60920	-3.93	-1.98	-9.85	3.06E-05	0.001289	3.06
250569_at	myc-like protein myc-like regulatory	BIM1	AT5G08130	-4.63	-2.21	-9.85	3.07E-05	0.001291	3.05
252433_at	putative protein putative hydrolyse	---	AT3G47560	-3.33	-1.74	-9.84	3.07E-05	0.001291	3.05
263664_at	putative auxin-induced protein, IAA	AXR3	AT1G04250	-8.23	-3.04	-9.83	3.09E-05	0.001296	3.04

253974_at	receptor protein kinase - like protei	---	AT4G26540	-4.63	-2.21	-9.79	3.18E-05	0.00132	3.02
257074_at	unknown protein	---	AT3G19660	4.99	2.32	9.79	3.19E-05	0.00132	3.01
251402_at	SRG1 - like protein SRG1 protein,	---	AT3G60290	-6.90	-2.79	-9.79	3.19E-05	0.00132	3.01
245176_at	unknown protein similar to GP|2104	---	AT2G47440	-7.08	-2.82	-9.78	3.20E-05	0.001322	3.01
259741_at	unknown protein	---	AT1G71020	-2.98	-1.58	-9.78	3.21E-05	0.001322	3.01
251790_at	elicitor responsive/phloem -like pro	---	AT3G55470	2.45	1.29	9.77	3.22E-05	0.001322	3.00
255078_at	putative protein ascorbate peroxida	APX4	AT4G09010	-3.03	-1.60	-9.76	3.24E-05	0.00133	3.00
249120_at	unknown protein ;supported by full-	---	AT5G43750	-2.20	-1.13	-9.74	3.29E-05	0.001345	2.98
255298_at	putative protein similar to transcripti	ATMSRB6	AT4G04840	-16.86	-4.08	-9.74	3.29E-05	0.001345	2.98
267565_at	putative cytochrome P450	CYP71A12	AT2G30750	25.35	4.66	9.72	3.33E-05	0.001352	2.97
245362_at	homeobox-leucine zipper protein H/	HAT1	AT4G17460	-3.01	-1.59	-9.71	3.35E-05	0.001356	2.96
262396_at	unknown protein ;supported by full-	---	AT1G49470	5.24	2.39	9.70	3.38E-05	0.001365	2.95
254701_at	putative protein predicted protein, /	---	AT4G18030	-4.20	-2.07	-9.69	3.40E-05	0.001369	2.95
261564_at	NAC domain protein, putative simil	ATAF1	AT1G01720	3.01	1.59	9.68	3.42E-05	0.00137	2.94
250645_at	putative protein strong similarity to u	---	AT5G06700	-4.21	-2.07	-9.67	3.43E-05	0.00137	2.94
245698_at	phosphate/phosphoenolpyruvate tra	---	AT5G04160	-3.78	-1.92	-9.67	3.43E-05	0.00137	2.94
265824_at	putative glucosyltransferase ;suppo	ATCSLA07	AT2G35650	-2.87	-1.52	-9.67	3.44E-05	0.00137	2.93
264321_at	unknown protein Contains similarity	---	AT1G04200	-2.90	-1.54	-9.67	3.44E-05	0.00137	2.93
266963_at	unknown protein	MTP11	AT2G39450	-2.31	-1.21	-9.67	3.45E-05	0.00137	2.93
250408_at	serine/threonine protein kinase -lik	CIPK5	AT5G10930	3.92	1.97	9.66	3.47E-05	0.001377	2.92
249118_at	putative protein similar to unknown	---	AT5G43870	-2.63	-1.39	-9.64	3.50E-05	0.001381	2.91
256310_at	unknown protein ; supported by cDI	ERD4	AT1G30360	-3.20	-1.68	-9.64	3.51E-05	0.001381	2.91
259430_at	hypothetical protein similar to unkno	GPAT4	AT1G01610	-3.30	-1.72	-9.64	3.51E-05	0.001381	2.91
246371_at	unknown protein contains similarity	---	AT1G51940	-2.33	-1.22	-9.64	3.51E-05	0.001381	2.91
255590_at	cathepsin B-like cysteine protease,	---	AT4G01610	2.59	1.38	9.62	3.55E-05	0.001384	2.90
256417_s_a	omega-3 fatty acid desaturase, chl	FAD7	AT3G11170	-3.96	-1.99	-9.62	3.56E-05	0.001384	2.90
267078_at	unknown protein ;supported by full-	---	AT2G40960	-2.15	-1.10	-9.61	3.57E-05	0.001384	2.89
266884_at	phytocyanin identical to GB:U90428	UCC2	AT2G44790	8.63	3.11	9.59	3.62E-05	0.001395	2.88
252317_at	putative protein hypersensitivity-rel	---	AT3G48720	-17.99	-4.17	-9.57	3.67E-05	0.001408	2.86
263184_at	UDP-glucose:indole-3-acetate beta	UGT75B1	AT1G05560	3.08	1.62	9.56	3.69E-05	0.00141	2.86
250690_at	ABC transporter-like protein ; supp	---	AT5G06530	-3.32	-1.73	-9.55	3.73E-05	0.001419	2.85
263297_at	putative ADP-ribosylation factor ; s	ATARFB1A	AT2G15310	3.38	1.76	9.52	3.79E-05	0.001441	2.83
260900_s_a	branched-chain alpha keto-acid de	---	AT1G21400	4.28	2.10	9.51	3.82E-05	0.001441	2.82
267481_at	putative receptor-like protein kinase	---	AT2G02780	-6.82	-2.77	-9.51	3.83E-05	0.001441	2.82
261594_at	DNA-binding factor, putative simila	AT-GTL1	AT1G33240	-2.36	-1.24	-9.51	3.83E-05	0.001441	2.82

251996_at	beta-galactosidase precursor - like	BGAL2	AT3G52840	-2.46	-1.30	-9.50	3.84E-05	0.001441	2.82
257628_at	cytochrome P450, putative contain	CYP71B26	AT3G26290	5.25	2.39	9.49	3.86E-05	0.001443	2.81
253136_at	putative protein leucine-rich protein	---	AT4G35470	-2.24	-1.16	-9.49	3.88E-05	0.001443	2.81
252231_at	hypothetical protein ;supported by t	---	AT3G49720	-2.46	-1.30	-9.48	3.89E-05	0.001446	2.80
258357_at	putative leucine-rich repeat transme	SRF7	AT3G14350	-3.89	-1.96	-9.45	3.97E-05	0.00147	2.78
259057_at	unknown protein ; supported by cD	---	AT3G03310	2.72	1.44	9.45	3.99E-05	0.001475	2.77
267005_at	unknown protein ; supported by cDI	---	AT2G34460	-2.12	-1.08	-9.44	4.00E-05	0.001476	2.77
258921_at	unknown protein N-terminus similar	anac053	AT3G10500	2.67	1.42	9.42	4.05E-05	0.001488	2.76
249823_s_a	putative protein contains similarity t	---	AT5G23350	-3.03	-1.60	-9.41	4.07E-05	0.001493	2.75
254300_at	Translation factor EF-1 alpha - like	ACR7	AT4G22780	3.10	1.63	9.41	4.10E-05	0.001493	2.75
247949_at	cytochrome P450	CYP81F2	AT5G57220	34.41	5.10	9.41	4.10E-05	0.001493	2.75
255552_at	S-adenosylmethionine synthase 2	SAM-2	AT4G01850	-2.22	-1.15	-9.37	4.19E-05	0.001514	2.72
259008_at	metallothionein-like protein similar t	MT2A	AT3G09390	2.28	1.19	9.37	4.19E-05	0.001514	2.72
263953_at	hypothetical protein predicted by ge	OFP15	AT2G36050	-4.13	-2.05	-9.37	4.19E-05	0.001514	2.72
264960_at	hypothetical protein contains similar	ATEXT4	AT1G76930	37.44	5.23	9.37	4.19E-05	0.001514	2.72
264916_at	ATP citrate-lyase, putative similar t	ACLA-2	AT1G60810	-2.89	-1.53	-9.36	4.24E-05	0.00152	2.71
266791_at	unknown protein	---	AT2G02960	-2.02	-1.01	-9.35	4.24E-05	0.00152	2.71
266735_at	putative pectinesterase ; supported	---	AT2G46930	-2.61	-1.38	-9.35	4.26E-05	0.001524	2.71
256569_at	hypothetical protein ; supported by	---	AT3G19550	8.86	3.15	9.34	4.29E-05	0.001528	2.70
267008_at	putative ABC transporter	---	AT2G39350	-3.31	-1.73	-9.33	4.30E-05	0.001528	2.70
258123_at	putative MAP kinase similar to blast	MPK9	AT3G18040	-4.36	-2.12	-9.33	4.32E-05	0.001531	2.69
259161_at	carbonic anhydrase, chloroplast pr	CA1	AT3G01500	-7.72	-2.95	-9.31	4.36E-05	0.00154	2.68
256891_at	hypothetical protein contains similar	---	AT3G19030	2.70	1.44	9.30	4.40E-05	0.001546	2.67
259592_at	lipid transfer protein, putative conta	LTPG1	AT1G27950	-9.71	-3.28	-9.30	4.41E-05	0.001547	2.67
258736_at	unknown protein	---	AT3G05900	-3.23	-1.69	-9.29	4.42E-05	0.001548	2.66
253740_at	Expressed protein ; supported by cl	---	AT4G28706	-2.41	-1.27	-9.29	4.43E-05	0.001548	2.66
260222_at	putative alpha galactosyltransferase	XXT5	AT1G74380	-4.34	-2.12	-9.27	4.49E-05	0.001559	2.65
259645_at	putative DNA-binding protein predi	BIM2	AT1G69010	-2.70	-1.43	-9.27	4.49E-05	0.001559	2.65
247251_at	cellulose synthase catalytic subunit	CESA6	AT5G64740	-2.88	-1.53	-9.26	4.51E-05	0.001565	2.64
266899_at	hypothetical protein predicted by ge	---	AT2G34620	-6.47	-2.69	-9.23	4.63E-05	0.001592	2.62
265884_at	unknown protein	---	AT2G42320	-2.53	-1.34	-9.22	4.65E-05	0.001593	2.61
261131_at	unknown protein	---	AT1G19835	-3.76	-1.91	-9.22	4.65E-05	0.001593	2.61
247573_at	anthocyanin 5-aromatic acyltransfe	AACT1	AT5G61160	57.15	5.84	9.22	4.66E-05	0.001593	2.61
252255_at	pectinesterase - like protein pectine	---	AT3G49220	-5.79	-2.53	-9.21	4.68E-05	0.001597	2.60
265405_at	putative protein kinase contains a p	---	AT2G16750	-3.90	-1.96	-9.20	4.70E-05	0.001597	2.60

245629_at	hypothetical protein predicted by ge	---	AT1G56580	-2.65	-1.41	-9.19	4.73E-05	0.001601	2.59
258982_at	putative serine/threonine protein ki	---	AT3G08870	3.27	1.71	9.17	4.81E-05	0.001615	2.58
244950_at	cytochrome c oxidase subunit 2	---	---	3.24	1.70	9.17	4.82E-05	0.001615	2.57
254665_at	beta-1,3-glucanase-like protein stro	---	AT4G18340	-3.79	-1.92	-9.16	4.83E-05	0.001615	2.57
259106_at	unknown protein ;supported by full-	RALFL22	AT3G05490	-5.87	-2.55	-9.16	4.84E-05	0.001615	2.57
255709_at	putative YABBY3 axial regulator ; s	YAB3	AT4G00180	-2.37	-1.24	-9.16	4.84E-05	0.001615	2.57
257076_at	unknown protein	---	AT3G19680	-5.15	-2.36	-9.15	4.89E-05	0.001618	2.56
254709_at	Expressed protein ; supported by cI	AGD8	AT4G17890	-2.48	-1.31	-9.14	4.91E-05	0.001618	2.55
250062_at	BCS1 - like protein h-bcs1, Homo s	---	AT5G17760	3.52	1.82	9.14	4.92E-05	0.001618	2.55
245011_at	photosystem II G protein	---	---	-2.08	-1.06	-9.14	4.92E-05	0.001618	2.55
251584_at	putative protein At2g42580 - Arabic	TTL4	AT3G58620	-6.19	-2.63	-9.13	4.93E-05	0.001618	2.55
254453_at	amino acid transport protein AAT1	AAT1	AT4G21120	3.14	1.65	9.13	4.95E-05	0.001618	2.54
262729_at	rac-like GTP binding protein (ARAC	ARAC5	AT1G75840	-2.67	-1.42	-9.13	4.96E-05	0.001618	2.54
252997_at	putative pollen allergen pollen aller	ATEXLA2	AT4G38400	-7.33	-2.87	-9.12	4.98E-05	0.00162	2.54
247884_at	lipid transfer protein; glossy1 homol	CER3	AT5G57800	-6.95	-2.80	-9.11	5.03E-05	0.001622	2.53
255543_at	predicted protein of unknown functi	---	AT4G01870	7.50	2.91	9.10	5.03E-05	0.001622	2.53
260493_at	hypothetical protein predicted by ge	---	AT2G41830	-3.70	-1.89	-9.10	5.03E-05	0.001622	2.53
248138_at	pyruvate decarboxylase (gb|AAB16	PDC2	AT5G54960	2.74	1.45	9.10	5.03E-05	0.001622	2.53
258179_at	integral membrane protein, putative	---	AT3G21690	2.98	1.58	9.10	5.03E-05	0.001622	2.53
251192_at	alpha galactosyltransferase-like pro	XT1	AT3G62720	-6.04	-2.59	-9.08	5.11E-05	0.001641	2.51
259790_s_a	auxin-induced protein, putative sim	---	AT1G29430 ,	-9.62	-3.27	-9.08	5.13E-05	0.001643	2.51
245136_at	putative auxin-regulated protein	---	AT2G45210	6.19	2.63	9.06	5.19E-05	0.001652	2.50
266745_at	unknown protein ; supported by cD	PKS1	AT2G02950	-6.03	-2.59	-9.05	5.20E-05	0.001655	2.49
259586_at	unknown protein	---	AT1G28100	-2.00	-1.00	-9.05	5.24E-05	0.001662	2.48
266533_s_a	putative plasma membrane intrinsic	PIP2;8	AT2G16850 ,	-2.84	-1.50	-9.03	5.29E-05	0.001666	2.47
255850_at	putative chloroplast 50S ribosomal	---	AT2G33450	-2.31	-1.21	-9.03	5.29E-05	0.001666	2.47
257686_at	unknown protein similar to unknown	SDRB	AT3G12800	2.24	1.16	9.03	5.31E-05	0.001666	2.47
258371_at	hypothetical protein contains similar	---	AT3G14410	-2.20	-1.14	-9.01	5.36E-05	0.001673	2.46
246284_at	putative protein	---	AT4G36780	-2.18	-1.13	-9.00	5.40E-05	0.001682	2.45
256057_at	unknown protein contains similarity	NDA1	AT1G07180	-4.81	-2.27	-8.99	5.43E-05	0.001687	2.45
258239_at	putative chlorophyll A-B binding pro	LHCB2.3	AT3G27690	-29.66	-4.89	-8.98	5.47E-05	0.001693	2.44
244902_at	NADH dehydrogenase subunit 4L	---	ATMG00650	2.23	1.15	8.98	5.49E-05	0.001693	2.44
254764_at	putative protein ; supported by cDN	---	AT4G13250	4.92	2.30	8.97	5.51E-05	0.001699	2.43
256999_at	hypothetical protein predicted by ge	---	AT3G14200	-2.01	-1.01	-8.97	5.53E-05	0.001702	2.43
255626_at	coded for by A. thaliana cDNA T206	---	AT4G00780	2.30	1.20	8.97	5.54E-05	0.001702	2.43

260009_at	unknown protein ;supported by full-	---	AT1G67950	-2.99	-1.58	-8.96	5.55E-05	0.001704	2.42
261822_at	unknown protein ;supported by full-	---	AT1G11380	-3.87	-1.95	-8.92	5.70E-05	0.001729	2.39
259143_at	calmodulin-like protein similar to ca	---	AT3G10190	2.68	1.42	8.91	5.74E-05	0.001737	2.39
266938_at	hypothetical protein predicted by ge	HPT1	AT2G18950	2.40	1.26	8.88	5.87E-05	0.001758	2.36
254445_at	3-hydroxyisobutyrate dehydrogena	---	AT4G20930	2.09	1.07	8.88	5.88E-05	0.001758	2.36
264954_at	carboxyphosphonoenolpyruvate m	---	AT1G77060	-2.71	-1.44	-8.87	5.92E-05	0.001766	2.35
245987_at	NAM-like protein hypothetical prote	ANAC083	AT5G13180	2.17	1.12	8.83	6.10E-05	0.001791	2.32
258813_at	NAM-like protein (no apical meriste	anac046	AT3G04060	2.73	1.45	8.83	6.11E-05	0.001791	2.32
260225_at	putative glutathione S-transferase s	GSTU10	AT1G74590	22.22	4.47	8.83	6.11E-05	0.001791	2.32
254332_at	leucine rich repeat receptor kinase	---	AT4G22730	-2.84	-1.51	-8.82	6.12E-05	0.001791	2.32
255732_at	fatty acid condensing enzyme CUT	KCS5	AT1G25450	-6.80	-2.77	-8.81	6.18E-05	0.001802	2.31
256865_at	NAD dependent epimerase, putativ	GAE6	AT3G23820	-3.89	-1.96	-8.81	6.20E-05	0.001806	2.30
247480_at	KED - like protein KED, Nicotiana t	ATBAG7	AT5G62390	-2.48	-1.31	-8.80	6.22E-05	0.001809	2.30
264910_at	resistance protein, putative similar	---	AT1G61100	-4.43	-2.15	-8.80	6.22E-05	0.001809	2.30
251922_at	protein kinase -like protein protein	---	AT3G54030	-3.96	-1.99	-8.79	6.28E-05	0.001813	2.29
262482_at	SRG1-like protein Strong homology	SRG1	AT1G17020	11.65	3.54	8.78	6.30E-05	0.001813	2.29
247934_at	putative protein similar to unknown	---	AT5G57000	-2.04	-1.03	-8.78	6.32E-05	0.001813	2.28
254370_at	L1 specific homeobox gene ATML1	ATML1	AT4G21750	-2.49	-1.32	-8.77	6.34E-05	0.001818	2.28
260676_at	integral membrane protein, putative	---	AT1G19450	-3.91	-1.97	-8.77	6.38E-05	0.001822	2.27
267209_at	unknown protein ;supported by full-	---	AT2G30930	-2.46	-1.30	-8.75	6.43E-05	0.001827	2.27
257338_s_a	NADH dehydrogenase subunit 5 (na	---	AT2G07711	2.68	1.42	8.75	6.46E-05	0.001832	2.26
248914_at	Rab-type small GTP-binding protei	AtRABA1c	AT5G45750	-3.29	-1.72	-8.73	6.55E-05	0.001849	2.24
257216_at	4-methyl-5(b-hydroxyethyl)-thiazole	---	AT3G14990	4.68	2.23	8.73	6.56E-05	0.001849	2.24
263391_at	putative monogalactosyldiacylglyce	MGDC	AT2G11810	3.58	1.84	8.73	6.56E-05	0.001849	2.24
265817_at	histone H1 ;supported by full-length	HIS1-3	AT2G18050	6.56	2.71	8.72	6.59E-05	0.001852	2.24
256228_at	phosphoglycerate kinase, putative	---	AT1G56190	-2.48	-1.31	-8.72	6.60E-05	0.001852	2.24
245042_at	hypothetical protein predicted by ge	HEMD	AT2G26540	-3.51	-1.81	-8.69	6.73E-05	0.00187	2.22
251993_at	peroxiredoxin - like protein peroxire	---	AT3G52960	-2.27	-1.19	-8.69	6.74E-05	0.00187	2.21
266905_at	putative katanin ;supported by full-l	---	AT2G34560	-2.73	-1.45	-8.69	6.76E-05	0.00187	2.21
253394_at	putative protein predicted protein, S	VTE1	AT4G32770	-3.66	-1.87	-8.68	6.79E-05	0.001871	2.21
263382_at	putative anthranilate N-hydroxycinn	---	AT2G40230	-2.42	-1.27	-8.68	6.79E-05	0.001871	2.21
247069_at	pectinesterase like protein ;support	sks17	AT5G66920	-5.81	-2.54	-8.67	6.82E-05	0.001875	2.20
264307_at	hypothetical protein contains similar	---	AT1G61900	-2.30	-1.20	-8.66	6.88E-05	0.001886	2.19
263207_at	putative endoxyloglucan transferase	XTH33	AT1G10550	-17.37	-4.12	-8.66	6.89E-05	0.001887	2.19
261763_at	ABC transporter, putative similar to	PDR12	AT1G15520	20.13	4.33	8.65	6.91E-05	0.001888	2.19

254260_at	hypothetical protein	---	AT4G23440	-2.96	-1.56	-8.65	6.92E-05	0.001888	2.19
256456_at	unknown protein ; supported by full-	---	AT1G75180	-3.68	-1.88	-8.65	6.94E-05	0.001888	2.18
251855_at	sugar-phosphate isomerase - like p	---	AT3G54690	-4.10	-2.03	-8.64	6.97E-05	0.00189	2.18
246411_at	hypothetical protein contains similar	---	AT1G57770	-4.74	-2.24	-8.64	6.98E-05	0.00189	2.18
260746_at	glutathione transferase, putative si	ATGSTU19	AT1G78380	2.03	1.02	8.64	6.99E-05	0.00189	2.18
263851_at	putative retroelement pol polyprotei	---	---	6.22	2.64	8.62	7.06E-05	0.001901	2.17
265573_at	putative zinc-finger protein similar to	---	AT2G28200	4.23	2.08	8.62	7.07E-05	0.001902	2.16
254077_at	putative protein NorM, Vibrio parah	---	AT4G25640	2.34	1.23	8.62	7.10E-05	0.001908	2.16
253875_at	putative protein ENOD20 gene, Me	---	AT4G27520	-4.01	-2.00	-8.61	7.12E-05	0.001909	2.16
259020_at	unknown protein ; supported by cD	---	AT3G07470	-2.30	-1.20	-8.60	7.18E-05	0.001917	2.15
244937_at	NADH dehydrogenase 49KDa prote	---	---	-3.48	-1.80	-8.60	7.19E-05	0.001917	2.15
249493_at	acyltransferase -like protein Anthoc	---	AT5G39080	-3.07	-1.62	-8.60	7.19E-05	0.001917	2.14
249467_at	NAM / CUC2 - like protein CUC2, A	ATNAC6	AT5G39610	9.58	3.26	8.58	7.27E-05	0.001926	2.13
244953_s_a	NADH dehydrogenase subunit 6	---	AT2G07731	3.02	1.60	8.58	7.27E-05	0.001926	2.13
258565_at	unknown protein ; supported by cD	---	AT3G04350	2.86	1.52	8.58	7.30E-05	0.001927	2.13
261949_at	hypothetical protein predicted by ge	BDG1	AT1G64670	-5.67	-2.50	-8.58	7.30E-05	0.001927	2.13
247074_at	putative protein contains similarity t	---	AT5G66590	-2.64	-1.40	-8.57	7.32E-05	0.001931	2.13
256529_at	protein kinase, putative contains P	---	AT1G33260	-3.78	-1.92	-8.54	7.49E-05	0.001949	2.10
258033_at	unknown protein similar to MRP-like	MRP6	AT3G21250	3.51	1.81	8.54	7.50E-05	0.001949	2.10
265938_at	putative SF21 protein {Helianthus a	---	AT2G19620	-6.66	-2.74	-8.54	7.51E-05	0.001949	2.10
248487_at	ATP-dependent Clp protease ATP-	ERD1	AT5G51070	2.12	1.09	8.52	7.64E-05	0.001966	2.08
258632_s_a	putative MAP3K epsilon protein kin	MAPKKK7	AT3G07980	-2.55	-1.35	-8.51	7.66E-05	0.001969	2.08
246133_at	aldehyde oxidase AAO1 ; supporte	AAO1	AT5G20960	7.41	2.89	8.51	7.70E-05	0.001976	2.07
246368_at	light repressible receptor protein ki	---	AT1G51890	8.75	3.13	8.50	7.75E-05	0.00198	2.06
251661_at	putative protein NODULIN-26 - Gly	SIP2;1	AT3G56950	2.46	1.30	8.49	7.80E-05	0.001986	2.06
259237_at	putative 2-cys peroxiredoxin BAS1	---	AT3G11630	-2.59	-1.38	-8.48	7.87E-05	0.001988	2.05
258484_at	sterol-C5-desaturase identical to G	STE1	AT3G02580	-2.00	-1.00	-8.47	7.87E-05	0.001988	2.05
253492_at	enoyl-CoA hydratase - like protein	---	AT4G31810	5.34	2.42	8.47	7.88E-05	0.001988	2.05
255674_at	probable plasma membrane intrinsi	PIP1;4	AT4G00430	-3.73	-1.90	-8.47	7.89E-05	0.001988	2.05
252441_at	putative protein ; supported by cDN	PTAC16	AT3G46780	-2.86	-1.52	-8.46	7.97E-05	0.002	2.04
267335_s_a	putative beta-1,3-glucanase	---	AT1G64760	-2.96	-1.56	-8.45	8.01E-05	0.002002	2.03
254638_at	hypothetical protein	---	AT4G18740	-2.84	-1.51	-8.45	8.02E-05	0.002002	2.03
251803_at	putative protein predicted proteins,	---	AT3G55350	-2.40	-1.26	-8.43	8.13E-05	0.002023	2.01
249073_at	vegetative storage protein-like ;sup	---	AT5G44020	-6.04	-2.59	-8.43	8.16E-05	0.002026	2.01
258719_at	putative pectate lyase simliar to pec	---	AT3G09540	-4.75	-2.25	-8.40	8.32E-05	0.002058	1.99

266993_at	nodulin-like protein ; supported by	---	AT2G39210	3.41	1.77	8.39	8.35E-05	0.002063	1.99
263513_at	unknown protein	---	AT2G12400	-3.12	-1.64	-8.38	8.47E-05	0.00208	1.97
252563_at	putative protein cim1 induced aller	ATEXLA1	AT3G45970	-8.14	-3.03	-8.34	8.72E-05	0.002123	1.94
245339_at	hypothetical protein ;supported by f	COBL7	AT4G16120	-3.44	-1.78	-8.33	8.77E-05	0.00213	1.93
245757_at	phosphate-induced (phi-1) protein,	PHI-1	AT1G35140	-33.33	-5.06	-8.33	8.77E-05	0.00213	1.93
247038_at	anthranilate N-hydroxycinnamoyl/be	EPS1	AT5G67160	2.49	1.32	8.32	8.80E-05	0.002136	1.93
256848_at	hypothetical protein similar to hypot	---	AT3G27960	-7.74	-2.95	-8.32	8.81E-05	0.002136	1.93
253571_at	putative calmodulin-binding protein	---	AT4G31000	-8.43	-3.07	-8.31	8.89E-05	0.002145	1.92
263676_at	putative RNA-binding protein Ident	CRB	AT1G09340	-2.47	-1.31	-8.31	8.90E-05	0.002145	1.92
248273_at	putative protein contains similarity t	---	AT5G53500	-3.38	-1.76	-8.29	9.02E-05	0.002169	1.90
264037_at	putative steroid sulfotransferase ;s	---	AT2G03750	-2.23	-1.16	-8.29	9.03E-05	0.002169	1.90
249818_at	Expressed protein ; supported by c	TUB8	AT5G23860	-3.39	-1.76	-8.29	9.06E-05	0.002172	1.90
253215_at	putative protein AT.I.24-7, Arabido	---	AT4G34950	-5.79	-2.53	-8.27	9.13E-05	0.002183	1.89
256076_at	unknown protein ; supported by cD	---	AT1G18060	-2.99	-1.58	-8.27	9.14E-05	0.002183	1.89
247817_at	Expressed protein ; supported by fu	---	AT5G58375	2.20	1.13	8.26	9.20E-05	0.002187	1.88
246952_at	putative protein similar to unknown	OFP13	AT5G04820	-2.27	-1.18	-8.26	9.20E-05	0.002187	1.88
250327_at	putative serine rich protein predicte	---	AT5G12050	-6.87	-2.78	-8.26	9.21E-05	0.002187	1.88
263823_s_a	AP2 domain transcription factor	---	AT2G40340	4.22	2.08	8.25	9.30E-05	0.002202	1.87
253022_at	putative protein	---	AT4G38060	3.73	1.90	8.25	9.33E-05	0.002204	1.87
250983_at	putative protein In2, Zea mays, EM	---	AT5G02780	12.53	3.65	8.24	9.35E-05	0.002204	1.86
253309_at	male sterility 2-like protein male ste	CER4	AT4G33790	-4.24	-2.08	-8.24	9.35E-05	0.002204	1.86
252711_at	lipid-transfer protein-like protein no	---	AT3G43720	-9.20	-3.20	-8.24	9.35E-05	0.002204	1.86
253046_at	cytochrome P450 - like protein cyt	CYP81D8	AT4G37370	14.47	3.86	8.24	9.37E-05	0.002205	1.86
264280_at	beta-glucosidase, putative similar t	BGLU46	AT1G61820	4.15	2.05	8.24	9.39E-05	0.002205	1.86
248153_at	cyclic nucleotide and calmodulin-re	ATCNGC4	AT5G54250	2.06	1.04	8.21	9.60E-05	0.002229	1.83
252117_at	mucin -like protein hemomucin, Dr	YLS2	AT3G51430	2.96	1.56	8.21	9.60E-05	0.002229	1.83
257204_at	Expressed protein ; supported by fu	RALFL24	AT3G23805	-5.68	-2.51	-8.21	9.61E-05	0.002229	1.83
256725_at	hypothetical protein predicted by ge	---	AT2G34070	-4.22	-2.08	-8.20	9.64E-05	0.002231	1.83
253197_at	putative protein Cyanophora parad	---	AT4G35250	-2.62	-1.39	-8.20	9.65E-05	0.002231	1.83
263545_at	unknown protein	---	AT2G21560	4.92	2.30	8.20	9.65E-05	0.002231	1.83
263553_at	putative purple acid phosphatase p	PAP10	AT2G16430	2.21	1.14	8.20	9.68E-05	0.002233	1.83
258421_at	MtN3-like protein similar to MtN3 G	---	AT3G16690	-3.76	-1.91	-8.20	9.69E-05	0.002233	1.83
257766_at	auxin-inducible gene (IAA2) identic	IAA2	AT3G23030	-2.84	-1.51	-8.20	9.69E-05	0.002233	1.82
258528_at	unknown protein contains Pfam pro	---	AT3G06770	-4.78	-2.26	-8.19	9.76E-05	0.002245	1.82
262225_at	pectinesterase, putative similar to p	ATPME1	AT1G53840	-4.06	-2.02	-8.18	9.78E-05	0.002245	1.82

252945_at	putative protein ;supported by full-l	---	AT4G39140	2.10	1.07	8.18	9.84E-05	0.002253	1.81
262314_at	unknown protein contains similarity	---	AT1G70810	2.17	1.12	8.17	9.85E-05	0.002253	1.81
256617_at	unknown protein ; supported by ful	---	AT3G22240	5.13	2.36	8.16	9.98E-05	0.002269	1.79
267261_at	unknown protein ;supported by full-	---	AT2G23120	-3.43	-1.78	-8.15	9.99E-05	0.002269	1.79
244943_at	NADH dehydrogenase subunit 9	---	ATMG00070	2.42	1.27	8.15	0.0001	0.002269	1.79
251150_at	putative protein Sb34 (PREG-like p	CYCP1;1	AT3G63120	-2.07	-1.05	-8.15	0.0001	0.002269	1.79
255647_at	Ca2+-transporting ATPase - like pro	ECA2	AT4G00900	2.24	1.17	8.14	0.000101	0.002281	1.78
266650_at	unknown protein	---	AT2G25800	-2.24	-1.17	-8.14	0.000101	0.002281	1.78
253617_at	hypothetical protein	---	AT4G30410	-5.82	-2.54	-8.13	0.000102	0.00229	1.77
248608_at	ATP citrate lyase ; supported by cD	ACLB-2	AT5G49460	-2.16	-1.11	-8.13	0.000102	0.002291	1.77
262661_s_a	hypothetical protein	---	AT1G14230 ,	-12.70	-3.67	-8.13	0.000102	0.002291	1.77
259660_at	unknown protein ; supported by full-	---	AT1G55260	-11.11	-3.47	-8.13	0.000102	0.002291	1.77
247399_at	putative protein similar to unknown	---	AT5G62960	-3.33	-1.74	-8.12	0.000103	0.002302	1.76
250213_at	H-protein promoter binding factor-1	TBP1	AT5G13820	2.52	1.33	8.11	0.000103	0.002307	1.76
254174_at	putative protein various predicted p	YSL1	AT4G24120	2.79	1.48	8.10	0.000104	0.002327	1.75
250300_at	putative protein various predicted p	---	AT5G11890	-2.65	-1.41	-8.09	0.000105	0.002331	1.74
256305_at	glutathione S-conjugate transporting	MRP1	AT1G30400	2.62	1.39	8.08	0.000106	0.002333	1.73
266140_at	nodulin-like protein ; supported by c	---	AT2G28120	-2.44	-1.29	-8.07	0.000107	0.002352	1.72
258202_at	unknown protein	---	AT3G13940	2.98	1.57	8.07	0.000107	0.002354	1.72
257022_at	zinc finger protein, putative similar	AZF2	AT3G19580	2.30	1.20	8.06	0.000107	0.002357	1.71
254034_at	P-glycoprotein-2 (pgp2)	PGP2	AT4G25960	-2.58	-1.37	-8.06	0.000108	0.002357	1.71
261767_s_a	adenine nucleotide translocase, pu	NTT1	AT1G15500 ,	2.12	1.09	8.05	0.000108	0.002365	1.70
261055_at	chloroplast nucleoid DNA binding p	---	AT1G01300	-2.12	-1.08	-8.05	0.000108	0.002365	1.70
266418_at	putative annexin ;supported by full-	ANNAT4	AT2G38750	-6.86	-2.78	-8.05	0.000108	0.002365	1.70
245349_at	cyanohydrin lyase like protein ;supp	MES16	AT4G16690	4.30	2.10	8.04	0.000109	0.002367	1.70
262902_x_a	hypothetical protein similar to putati	---	AT1G59930	-2.89	-1.53	-8.04	0.000109	0.002372	1.69
252400_at	hypothetical protein	---	AT3G48020	3.97	1.99	8.02	0.000111	0.002391	1.68
258901_at	putative protein phosphatase-2C sir	---	AT3G05640	-19.30	-4.27	-8.02	0.000111	0.002392	1.68
251259_at	putative protein phosphoprotein ph	---	AT3G62260	2.85	1.51	8.01	0.000111	0.002396	1.68
260501_at	unknown protein ; supported by cD	---	AT2G41770	-2.44	-1.29	-8.01	0.000111	0.002396	1.67
263456_at	unknown protein	---	AT2G22125	-2.40	-1.26	-8.01	0.000111	0.002396	1.67
256855_at	30S ribosomal protein S20 contains	---	AT3G15190	-2.20	-1.13	-8.01	0.000112	0.002402	1.67
246792_at	putative protein predicted proteins,	---	AT5G27290	-4.01	-2.01	-8.00	0.000112	0.002402	1.67
258923_at	putative glucose acyltransferase sir	SCPL7	AT3G10450	4.10	2.04	8.00	0.000113	0.002411	1.66
259520_at	hypothetical protein	---	AT1G12320	2.99	1.58	7.98	0.000114	0.002425	1.65

259618_at	myb-related transcription factor (cp	MYB112	AT1G48000	2.51	1.33	7.98	0.000114	0.002427	1.65
248756_at	sodium-dicarboxylate cotransporter	TDT	AT5G47560	3.62	1.86	7.98	0.000114	0.002427	1.65
252866_at	putative protein	---	AT4G39840	-5.83	-2.54	-7.97	0.000115	0.002432	1.64
263947_at	unknown protein ;supported by full-	---	AT2G35820	3.20	1.68	7.95	0.000117	0.002457	1.62
246584_at	putative protein predicted protein, A	---	AT5G14730	10.01	3.32	7.95	0.000117	0.002457	1.62
251191_at	putative protein putative protein F1	---	AT3G62590	3.52	1.81	7.95	0.000117	0.002457	1.62
254805_at	pEARLI 1 ; supported by cDNA: gi_	pEARLI 1	AT4G12480	48.43	5.60	7.93	0.000118	0.002479	1.61
254189_at	putative protein cellulose synthase	CSLG2	AT4G24000	6.21	2.63	7.93	0.000118	0.002479	1.61
262665_at	xyloglucan fucosyltransferase, puta	FUT7	AT1G14070	5.47	2.45	7.92	0.000119	0.002493	1.60
250960_at	putative protein	---	AT5G02940	-3.31	-1.72	-7.92	0.00012	0.002497	1.60
251906_at	putative protein several hypothetica	ATCHX20	AT3G53720	2.34	1.22	7.92	0.00012	0.002497	1.59
259800_at	Expressed protein ; supported by fu	---	AT1G72175	-2.16	-1.11	-7.91	0.00012	0.0025	1.59
249941_at	putative protein similar to unknown	---	AT5G22270	2.32	1.22	7.91	0.000121	0.0025	1.59
251305_at	peptidylprolyl isomerase ROC4 ;su	ROC4	AT3G62030	-2.67	-1.42	-7.89	0.000122	0.002513	1.57
258395_at	putative jasmonic acid regulatory pr	ANAC055	AT3G15500	3.19	1.67	7.89	0.000123	0.002516	1.57
261758_at	hypothetical protein contains similar	ADT6	AT1G08250	2.82	1.50	7.89	0.000123	0.002516	1.57
261135_at	defensin AMP1, putative similar to	PDF1.4	AT1G19610	17.71	4.15	7.89	0.000123	0.002516	1.57
261240_at	subtilisin-like serine protease conta	SBT3.5	AT1G32940	5.30	2.41	7.87	0.000124	0.002533	1.56
249174_at	putative protein similar to unknown	---	AT5G42900	3.20	1.68	7.87	0.000124	0.002534	1.56
267279_at	hypothetical protein predicted by ge	---	AT2G19460	-3.95	-1.98	-7.87	0.000124	0.002534	1.56
257173_at	S-adenosyl-L-homocysteinas, puta	SAHH2	AT3G23810	-11.45	-3.52	-7.87	0.000124	0.002534	1.55
255794_at	putative NAM (no apical meristem)-	ANAC041	AT2G33480	4.22	2.08	7.84	0.000127	0.00256	1.53
251176_at	Ca2+-transporting ATPase -like pro	---	AT3G63380	9.99	3.32	7.84	0.000127	0.00256	1.53
256461_s_a	adenylosuccinate lyase - like protei	---	AT1G36280 ,	-6.49	-2.70	-7.84	0.000127	0.002564	1.53
265228_s_a	hypothetical protein	---	AT2G07698	2.15	1.10	7.83	0.000128	0.002579	1.52
245976_at	WRKY-like protein WRKY DNA bin	WRKY75	AT5G13080	15.69	3.97	7.83	0.000129	0.002582	1.52
249847_at	serine carboxypeptidase II-like prot	SCPL34	AT5G23210	-2.65	-1.41	-7.82	0.000129	0.002584	1.52
250827_at	cellulose synthase catalytic subunit	CEV1	AT5G05170	-2.42	-1.28	-7.82	0.00013	0.002588	1.51
245148_at	putative pectinesterase ; supportec	---	AT2G45220	16.55	4.05	7.82	0.00013	0.002588	1.51
245325_at	xyloglucan endotransglycosylase-re	XTR7	AT4G14130	-20.10	-4.33	-7.81	0.000131	0.002598	1.50
246446_at	putative protein hypothetical protei	---	AT5G17640	-2.19	-1.13	-7.80	0.000131	0.002602	1.50
252184_at	steroid 22-alpha-hydroxylase (DWF	DWF4	AT3G50660	-2.33	-1.22	-7.80	0.000132	0.002609	1.49
266000_at	putative cytochrome P450 identical	CYP71B6	AT2G24180	3.52	1.82	7.79	0.000132	0.002613	1.49
249807_at	pectinacetylesterase	---	AT5G23870	-2.52	-1.33	-7.79	0.000133	0.002622	1.48
255774_at	unknown protein	---	AT1G18620	-3.98	-1.99	-7.76	0.000136	0.002659	1.46

254085_at	abscisic acid-induced - like protein	HVA22D	AT4G24960	-3.71	-1.89	-7.74	0.000137	0.002679	1.45
258535_at	unknown protein ; supported by cD	---	AT3G06750	-3.23	-1.69	-7.74	0.000138	0.002682	1.44
246463_at	quinone oxidoreductase -like prote	AT-AER	AT5G16970	2.58	1.37	7.74	0.000138	0.002682	1.44
244983_at	ribosomal protein L16	---	---	-3.13	-1.64	-7.73	0.000138	0.002682	1.44
248683_at	putative protein similar to unknown	---	AT5G48490	-7.64	-2.93	-7.73	0.000138	0.002682	1.44
253281_at	predicted protein	UGT73B1	AT4G34138	3.89	1.96	7.73	0.000139	0.002682	1.44
254122_at	CER2 ;supported by full-length cD	CER2	AT4G24510	-35.22	-5.14	-7.73	0.000139	0.002682	1.44
266254_at	putative membrane transporter	NAT12	AT2G27810	-2.24	-1.17	-7.73	0.000139	0.002682	1.44
257793_at	unknown protein ;supported by full-	---	AT3G26960	-12.16	-3.60	-7.71	0.000141	0.0027	1.42
245692_at	myc - like protein	BHLH101	AT5G04150	-2.39	-1.26	-7.71	0.000141	0.002701	1.42
259530_at	hypothetical protein	---	AT1G12450	-3.21	-1.68	-7.71	0.000141	0.002701	1.42
254485_at	cell-cell signaling protein csgA - lik	---	AT4G20760	-2.25	-1.17	-7.71	0.000141	0.002704	1.42
249065_at	putative protein similar to unknown	---	AT5G44260	-3.25	-1.70	-7.70	0.000142	0.002709	1.41
261315_at	AP2 domain containing protein, pu	ERF8	AT1G53170	3.77	1.92	7.70	0.000143	0.002712	1.41
253975_at	putative protein proliferating-cell nu	---	AT4G26600	4.18	2.06	7.69	0.000143	0.002712	1.41
247679_at	1-aminocyclopropane-1-carboxylat	---	AT5G59540	3.66	1.87	7.68	0.000144	0.002722	1.40
256327_at	unknown protein ;supported by full-	GAUT9	AT3G02350	-2.15	-1.10	-7.68	0.000144	0.002722	1.40
263433_at	putative myo-inositol 1-phosphate s	MIPS2	AT2G22240	-2.58	-1.37	-7.68	0.000145	0.002726	1.39
247162_at	xyloglucan endo-transglycosylase-li	---	AT5G65730	-5.57	-2.48	-7.68	0.000145	0.002726	1.39
254520_at	potassium transporter-like protein p	---	AT4G19960	2.36	1.24	7.67	0.000145	0.002731	1.39
248311_at	beta-carotene hydroxylase	BETA-OHA	AT5G52570	-2.87	-1.52	-7.67	0.000146	0.002733	1.38
267635_at	rhodanese-like family protein ;supp	---	AT2G42220	-2.88	-1.53	-7.67	0.000146	0.002733	1.38
258368_at	subtilisin-like serine protease conta	---	AT3G14240	-3.88	-1.95	-7.66	0.000146	0.002735	1.38
244981_at	ribosomal protein S8	---	---	-2.08	-1.05	-7.64	0.000149	0.002765	1.36
260812_at	nodulin-like protein similar to MtN2	---	AT1G43650	-2.74	-1.46	-7.63	0.000151	0.002794	1.35
254563_at	putative protein several hypothetica	ERD3	AT4G19120	-2.29	-1.20	-7.62	0.000152	0.002804	1.34
251365_at	putative DNA-binding protein At2g3	---	AT3G61310	-2.20	-1.14	-7.61	0.000152	0.002813	1.34
253794_at	putative protein dimethylaniline mo	---	AT4G28720	-5.67	-2.50	-7.61	0.000153	0.002813	1.33
263497_at	cold-regulated protein cor15a precu	COR15A	AT2G42540	-27.93	-4.80	-7.58	0.000157	0.002868	1.30
247488_at	putative protein MtN19, Medicago t	---	AT5G61820	2.54	1.35	7.57	0.000157	0.002869	1.30
265042_at	unknown protein Similar to acid ph	---	AT1G04040	-6.37	-2.67	-7.56	0.000159	0.002877	1.29
253039_at	squalene epoxidase - like protein s	SQE3	AT4G37760	-3.45	-1.79	-7.56	0.000159	0.002878	1.29
253493_at	putative protein various predicted p	ENP	AT4G31820	-2.84	-1.51	-7.56	0.000159	0.002878	1.29
259729_at	hypothetical protein predicted by ge	---	AT1G77640	-5.80	-2.54	-7.56	0.000159	0.00288	1.29
263467_at	unknown protein	---	AT2G31730	-3.76	-1.91	-7.56	0.00016	0.00288	1.29

249045_at	berberine bridge enzyme-like protei	---	AT5G44380	8.12	3.02	7.55	0.00016	0.002886	1.28
259437_at	protein serine/threonine kinase, pu	---	AT1G01540	-3.25	-1.70	-7.54	0.000161	0.002896	1.27
264746_at	unknown protein similar to putative	WRKY6	AT1G62300	5.64	2.50	7.54	0.000161	0.002896	1.27
252411_at	putative protein PMP31 protein - C	PEX11B	AT3G47430	3.51	1.81	7.54	0.000161	0.002896	1.27
261768_at	gibberellin 3 beta-hydroxylase, puta	GA3OX1	AT1G15550	-2.04	-1.03	-7.54	0.000162	0.002898	1.27
264338_at	potassium transporter, putative sim	KUP6	AT1G70300	2.62	1.39	7.54	0.000162	0.0029	1.27
251796_at	synaptic glycoprotein SC2-like prot	---	AT3G55360	-2.74	-1.46	-7.53	0.000163	0.002912	1.26
260924_at	hypothetical protein similar to Pto ki	---	AT1G21590	2.51	1.33	7.52	0.000164	0.002918	1.26
255617_at	putative protein kinase	---	AT4G01330	-2.14	-1.09	-7.52	0.000164	0.002918	1.26
258997_at	hypothetical protein similar to unkno	---	AT3G01810	-2.86	-1.52	-7.52	0.000164	0.00292	1.25
253478_at	putative protein trichohyalin - huma	---	AT4G32350	-2.77	-1.47	-7.52	0.000165	0.00292	1.25
247126_at	protein phosphatase 2C-like protein	---	AT5G66080	-2.51	-1.33	-7.51	0.000165	0.002929	1.25
260898_at	plastid ribosomal protein L34 precu	---	AT1G29070	-2.23	-1.16	-7.51	0.000166	0.002929	1.24
248100_at	beta-1,3-glucanase-like protein ;su	---	AT5G55180	-5.99	-2.58	-7.51	0.000166	0.002933	1.24
255536_at	Expressed protein ; supported by fu	---	AT4G01575	-2.69	-1.43	-7.50	0.000168	0.002948	1.23
247819_at	MAP kinase ; supported by cDNA: g	WNK4	AT5G58350	2.45	1.29	7.49	0.000169	0.00296	1.23
258887_at	putative phospholipase D similar to	PLDP2	AT3G05630	2.41	1.27	7.49	0.000169	0.00296	1.23
257051_at	squamosa promoter binding protein	SPL5	AT3G15270	-6.35	-2.67	-7.48	0.00017	0.002972	1.22
267220_at	putative sugar nucleotide phosphor	ISPD	AT2G02500	-2.06	-1.04	-7.47	0.000171	0.00298	1.21
259664_at	unknown protein ; supported by full-	AGP21	AT1G55330	-38.51	-5.27	-7.47	0.000171	0.00298	1.21
259248_at	putative heat-shock protein similar t	---	AT3G07770	3.79	1.92	7.47	0.000172	0.002982	1.21
247925_at	TCH4 protein (gb|AAA92363.1) ; su	TCH4	AT5G57560	-12.58	-3.65	-7.46	0.000172	0.00299	1.20
253042_at	formamidase - like protein formami	---	AT4G37550	-2.76	-1.47	-7.45	0.000174	0.003005	1.19
247327_at	peroxidase (emb|CAA67551.1) ;su	---	AT5G64120	14.11	3.82	7.45	0.000174	0.003005	1.19
260302_at	putative sulfate transporter contains	---	AT1G80310	2.22	1.15	7.44	0.000176	0.00302	1.18
253424_at	putative protein ; supported by cDN	---	AT4G32330	-2.10	-1.07	-7.42	0.000179	0.003054	1.16
258684_at	putative protein kinase contains Pfa	---	AT3G08680	-2.20	-1.14	-7.41	0.000179	0.003059	1.16
261769_at	plastocyanin identical to plastocyan	PETE1	AT1G76100	-2.05	-1.04	-7.41	0.00018	0.003063	1.16
248460_at	Expressed protein ; supported by fu	---	AT5G50915	-8.57	-3.10	-7.40	0.000181	0.003066	1.15
248085_at	putative protein similar to unknown	SVL1	AT5G55480	-3.40	-1.77	-7.39	0.000183	0.003077	1.14
258225_at	unknown protein ; supported by cD	---	AT3G15630	-4.28	-2.10	-7.38	0.000184	0.003095	1.13
263411_at	putative C2H2-type zinc finger prote	---	AT2G28710	3.13	1.65	7.38	0.000185	0.003095	1.13
246408_at	hypothetical protein predicted by ge	---	AT1G57680	-2.14	-1.10	-7.37	0.000185	0.003097	1.13
267377_at	beta-ketoacyl-CoA synthase (FIDDL	KCS10	AT2G26250	-3.54	-1.82	-7.37	0.000185	0.003097	1.12
255904_at	lemir (miraculin), putative similar to	---	AT1G17860	2.14	1.10	7.37	0.000186	0.003098	1.12

263536_at	putative WRKY-type DNA binding p	WRKY60	AT2G25000	3.89	1.96	7.35	0.000189	0.003119	1.10
255479_at	coded for by A. thaliana cDNA T46	SAG21	AT4G02380	10.55	3.40	7.34	0.00019	0.003133	1.10
249052_at	antifungal protein-like (PDF1.2)	PDF1.2	AT5G44420	12.91	3.69	7.34	0.000191	0.00314	1.09
257157_at	syntaxin-like protein similar to synt	SYP32	AT3G24350	-2.26	-1.17	-7.33	0.000192	0.003159	1.08
251282_at	putative protein transcription factor	CRF6	AT3G61630	4.10	2.03	7.32	0.000193	0.003161	1.08
254210_at	putative protein zinc finger protein,	---	AT4G23450	3.19	1.67	7.32	0.000194	0.003161	1.08
254630_at	glycolate oxidase - like protein (S)-	---	AT4G18360	11.73	3.55	7.31	0.000195	0.003172	1.07
264078_at	putative beta-galactosidase	BGAL8	AT2G28470	-7.30	-2.87	-7.30	0.000197	0.0032	1.06
264788_at	putative DnaJ protein ;supported b	---	AT2G17880	-5.81	-2.54	-7.29	0.000198	0.003211	1.05
250668_at	unknown protein ; supported by ful	---	AT5G07020	-2.22	-1.15	-7.29	0.000198	0.003211	1.05
251105_at	putative protein unknown protein A	ATSCAR4	AT5G01730	-3.70	-1.89	-7.29	0.000199	0.003217	1.05
256981_at	brassinosteroid receptor kinase, pu	BRL3	AT3G13380	2.61	1.39	7.28	0.0002	0.003223	1.04
263809_at	putative GDSL-motif lipase/hydrolas	---	AT2G04570	-3.98	-1.99	-7.28	0.0002	0.003227	1.04
261639_at	tubulin alpha-2/alpha-4 chain, puta	TUA2	AT1G50010	-5.87	-2.55	-7.26	0.000203	0.003268	1.02
257219_at	uroporphyrinogen decarboxylase, p	HEME1	AT3G14930	-2.29	-1.19	-7.26	0.000203	0.003268	1.02
265649_at	putative ferredoxin ; supported by f	ATFD3	AT2G27510	3.04	1.60	7.25	0.000206	0.003285	1.01
258076_at	50S ribosomal protein L15, chlorop	RPL15	AT3G25920	-2.30	-1.20	-7.24	0.000206	0.003289	1.01
252549_at	protein kinase - like receptor-like p	---	AT3G45860	-4.32	-2.11	-7.24	0.000206	0.003289	1.01
253855_at	senescence-associated protein -lik	TET7	AT4G28050	-4.71	-2.23	-7.24	0.000207	0.003292	1.01
251428_at	beta-glucosidase-like protein severa	DIN2	AT3G60140	54.80	5.78	7.24	0.000207	0.003295	1.00
266995_at	putative cytochrome P450	CYP710A1	AT2G34500	12.55	3.65	7.23	0.000208	0.003311	1.00
260345_at	receptor protein kinase, putative si	RPK1	AT1G69270	2.41	1.27	7.23	0.000208	0.003311	1.00
260933_at	hypothetical protein contains non-c	---	AT1G02470	8.92	3.16	7.22	0.00021	0.003323	0.99
255692_at	putative protein	GPAT8	AT4G00400	-6.46	-2.69	-7.21	0.000211	0.003333	0.98
259185_at	putative phosphate/phosphoenolpy	PPT2	AT3G01550	-9.69	-3.28	-7.21	0.000211	0.003333	0.98
264990_at	unknown protein supported by cDN	---	AT1G27210	-3.13	-1.65	-7.21	0.000212	0.003333	0.98
258351_at	hypothetical protein contains simila	CNBT1	AT3G17700	2.47	1.30	7.21	0.000212	0.003335	0.98
250054_at	potassium-dependent sodium-calci	CAX7	AT5G17860	4.67	2.22	7.20	0.000213	0.003346	0.97
248040_at	putative protein contains similarity t	---	AT5G55970	3.52	1.82	7.20	0.000214	0.003355	0.97
259773_at	auxin-induced protein, putative sim	---	AT1G29500	-7.92	-2.99	-7.19	0.000216	0.003363	0.96
246777_at	RING-H2 zinc finger protein-like RI	---	AT5G27420	15.22	3.93	7.18	0.000218	0.003389	0.95
265913_at	Expressed protein ; supported by fu	---	AT2G25625	4.52	2.18	7.17	0.000219	0.003402	0.94
247427_at	putative protein similar to unknown	---	AT5G62580	-2.83	-1.50	-7.17	0.000219	0.003402	0.94
264521_at	unknown protein Location of EST g	---	AT1G10020	-3.95	-1.98	-7.17	0.000219	0.003402	0.94
247101_at	apospory-associated protein C-like	---	AT5G66530	-2.15	-1.11	-7.17	0.00022	0.003402	0.94

247175_at	G protein-coupled receptor-like prot	GCL1	AT5G65280	-4.87	-2.28	-7.17	0.00022	0.003402	0.94
256114_at	unknown protein ; supported by cDI	---	AT1G16850	-6.43	-2.68	-7.16	0.000221	0.003421	0.93
263628_at	unknown protein ;supported by full-	FLA7	AT2G04780	-8.21	-3.04	-7.16	0.000222	0.003421	0.93
264424_at	unknown protein similar to hypothet	---	AT1G61740	-2.05	-1.04	-7.16	0.000222	0.003422	0.93
261717_at	helix-loop-helix protein homolog, pu	BEE1	AT1G18400	-8.23	-3.04	-7.15	0.000222	0.003424	0.93
260005_at	unknown protein ;supported by full-	---	AT1G67920	6.78	2.76	7.14	0.000225	0.00344	0.92
265151_at	hypothetical protein similar to hypot	---	AT1G51340	2.05	1.04	7.14	0.000225	0.003447	0.91
255506_at	predicted glycosyl transferase simi	GATL6	AT4G02130	-3.21	-1.68	-7.13	0.000227	0.003462	0.91
251519_at	putative protein hypothetical protei	GUN4	AT3G59400	-2.12	-1.09	-7.13	0.000227	0.003462	0.90
248607_at	NaCl-inducible Ca2+-binding protei	ATCP1	AT5G49480	4.39	2.13	7.13	0.000227	0.003462	0.90
251503_at	ABC transporter-like protein putativ	ATMRP14	AT3G59140	2.77	1.47	7.13	0.000228	0.003464	0.90
258003_at	expansin At-EXP5 identical to expa	EXPA5	AT3G29030	-7.01	-2.81	-7.12	0.000228	0.003467	0.90
249027_at	ATP dependent copper transporter	RAN1	AT5G44790	2.06	1.04	7.12	0.00023	0.003479	0.89
250248_at	transporter-like protein	ZIF1	AT5G13740	-3.49	-1.80	-7.10	0.000232	0.0035	0.88
259788_at	lipase/hydrolase, putative contains	---	AT1G29670	-10.41	-3.38	-7.10	0.000233	0.003507	0.88
267339_at	unknown protein ; supported by cD	---	AT2G39870	-2.80	-1.48	-7.10	0.000234	0.003513	0.87
257339_s_a	ATP synthase subunit 9	---	AT2G07671	2.44	1.29	7.09	0.000234	0.003515	0.87
248812_at	palmitoyl-protein thioesterase precu	---	AT5G47330	-8.48	-3.08	-7.09	0.000235	0.003517	0.87
266071_at	unknown protein	---	AT2G18670	7.12	2.83	7.09	0.000235	0.003517	0.87
253412_at	putative protein (fragment) calcineu	CBL10	AT4G33000	-2.00	-1.00	-7.08	0.000237	0.003529	0.86
246724_at	putative protein CDPK substrate pr	---	AT5G29000	2.06	1.04	7.07	0.000238	0.003541	0.85
263509_s_a	hypothetical protein	---	AT2G07687	2.47	1.31	7.06	0.000241	0.003565	0.84
258472_at	unknown protein identical to unknov	---	AT3G06080	-2.54	-1.34	-7.05	0.000243	0.00357	0.83
249927_at	Glucose-1-phosphate adenylyltrans	APL1	AT5G19220	-2.55	-1.35	-7.05	0.000244	0.003578	0.83
248276_at	EspB-like protein	YSL3	AT5G53550	2.26	1.17	7.04	0.000245	0.003586	0.82
258338_at	putative L-asparaginase similar to L	---	AT3G16150	2.72	1.45	7.04	0.000245	0.003586	0.82
260685_at	Expressed protein ; supported by fu	GLYR2	AT1G17650	-2.28	-1.19	-7.03	0.000247	0.003604	0.81
251169_at	putative protein putative protein At2	MARD1	AT3G63210	3.53	1.82	7.00	0.000253	0.003649	0.79
256585_at	hypothetical protein	---	AT3G28760	-2.34	-1.23	-7.00	0.000253	0.003654	0.78
267116_at	unknown protein	---	AT2G32560	-2.97	-1.57	-6.99	0.000255	0.003665	0.78
253255_at	putative auxin-regulated protein au	---	AT4G34760	-8.97	-3.17	-6.99	0.000255	0.003665	0.78
266892_at	putative glycine dehydrogenase	AtGLDP2	AT2G26080	-2.06	-1.04	-6.99	0.000256	0.003674	0.77
253286_at	hypothetical protein	FUC95A	AT4G34260	-9.63	-3.27	-6.99	0.000257	0.003676	0.77
246911_at	transcription factor TINY	tny	AT5G25810	-3.94	-1.98	-6.98	0.000259	0.0037	0.76
246880_s_a	myrosinase TGG2 ; supported by c	TGG2	AT5G25980	-5.94	-2.57	-6.97	0.00026	0.003701	0.76

263591_at	unknown protein	ATMAP65-	AT2G01910	-6.92	-2.79	-6.97	0.000261	0.00371	0.75
257933_at	aminoalcoholphosphotransferase,	AAPT2	AT3G25585	2.54	1.35	6.96	0.000261	0.003714	0.75
248819_at	putative protein contains similarity t	---	AT5G47050	2.68	1.42	6.96	0.000262	0.003722	0.75
263348_at	cytoplasmic aconitate hydratase	---	AT2G05710	2.22	1.15	6.96	0.000263	0.003726	0.74
261192_at	NAM protein, putative similar to NA	ANAC13	AT1G32870	2.21	1.15	6.96	0.000263	0.003726	0.74
259892_at	germin-like protein similar to germin	GER1	AT1G72610	-12.28	-3.62	-6.95	0.000264	0.003735	0.74
265849_at	hypothetical protein predicted by ge	---	AT2G35736	2.21	1.15	6.95	0.000265	0.003737	0.74
248908_at	receptor kinase-like protein	MEE62	AT5G45800	5.05	2.34	6.93	0.00027	0.003794	0.71
250254_at	24-sterol C-methyltransferase ; sup	SMT1	AT5G13710	-2.25	-1.17	-6.92	0.000272	0.003807	0.71
261407_at	unknown protein ;supported by full-	---	AT1G18810	-2.44	-1.28	-6.92	0.000273	0.003814	0.71
250167_at	myb-related protein - like myb-relat	ATMYB16	AT5G15310	-2.28	-1.19	-6.91	0.000275	0.003829	0.70
259358_at	hypothetical protein similar to putati	GATL3	AT1G13250	-5.46	-2.45	-6.90	0.000276	0.003842	0.69
255462_at	hypothetical protein similar to A. th	---	AT4G02940	2.69	1.43	6.89	0.000279	0.003863	0.68
252515_at	heat shock protein 17 ; supported b	HSP17.4	AT3G46230	8.60	3.10	6.89	0.00028	0.003867	0.68
253688_at	putative protein ;supported by full-l	---	AT4G29590	-2.25	-1.17	-6.88	0.000281	0.003872	0.67
250351_at	heat shock protein 17.6A	AT-HSP17.	AT5G12030	4.66	2.22	6.88	0.000282	0.003881	0.67
249895_at	male sterility 2-like protein (emb|CA	FAR1	AT5G22500	-6.64	-2.73	-6.88	0.000282	0.003881	0.67
256793_at	unknown protein ;supported by full-	---	AT3G22160	3.58	1.84	6.87	0.000283	0.003881	0.66
257634_s_a	cytochrome P450, putative similar t	CYP71B19	AT3G26170 ,	3.02	1.60	6.86	0.000286	0.003901	0.65
248377_at	unknown protein ; supported by ful	---	AT5G51720	-3.14	-1.65	-6.86	0.000286	0.003901	0.65
251178_at	cytokinin oxidase -like protein cytok	CKX6	AT3G63440	-3.44	-1.78	-6.86	0.000287	0.003901	0.65
251406_at	putative protein various predicted p	---	AT3G60260	-2.72	-1.44	-6.85	0.000289	0.003921	0.64
258299_at	unknown protein contains GMC oxid	---	AT3G23410	-2.21	-1.14	-6.85	0.000289	0.003921	0.64
260820_at	receptor protein kinase, putative si	---	AT1G06840	2.79	1.48	6.84	0.00029	0.003937	0.64
264083_at	ethylene reponse factor-like AP2 do	ATERF15	AT2G31230	7.39	2.89	6.84	0.000292	0.003947	0.63
267611_at	unknown protein	SPX2	AT2G26660	2.70	1.43	6.84	0.000292	0.003947	0.63
246488_at	steroid 5alpha-reductase-like prote	---	AT5G16010	-2.75	-1.46	-6.83	0.000293	0.003955	0.63
256275_at	actin 11 (ACT11) identical to actin 1	ACT11	AT3G12110	-2.28	-1.19	-6.83	0.000293	0.003955	0.63
265716_at	unknown protein ;supported by full-	---	AT2G03350	-2.11	-1.08	-6.83	0.000295	0.003973	0.62
260362_at	putative protein kinase similar to C-	---	AT1G70530	3.63	1.86	6.82	0.000296	0.003977	0.62
261272_at	Expressed protein ; supported by cl	---	AT1G26665	3.05	1.61	6.82	0.000296	0.003977	0.61
256453_at	GSH-dependent dehydroascorbate	DHAR2	AT1G75270	3.03	1.60	6.82	0.000297	0.003987	0.61
252363_at	lipase - like protein lipase Arab-1, A	---	AT3G48460	-5.34	-2.42	-6.81	0.000298	0.003989	0.61
253949_at	grpE like protein ;supported by full-	AR192	AT4G26780	4.17	2.06	6.81	0.000299	0.003998	0.60
245277_at	glucosyltransferase like protein ; su	IAGLU	AT4G15550	3.05	1.61	6.81	0.0003	0.004002	0.60

252859_at	putative protein AP2 domain conta	---	AT4G39780	2.49	1.32	6.81	0.0003	0.004002	0.60
250006_at	putative protein 2'-hydroxyisoflavor	PCB2	AT5G18660	-4.28	-2.10	-6.81	0.0003	0.004003	0.60
253066_at	1-aminocyclopropane-1-carboxylat	ACS8	AT4G37770	-11.29	-3.50	-6.79	0.000303	0.004034	0.59
259431_at	plasma membrane intrinsic protein	PIP1C	AT1G01620	-8.66	-3.11	-6.79	0.000303	0.004037	0.59
247214_at	putative protein similar to unknown	---	AT5G64850	-4.18	-2.06	-6.79	0.000304	0.004037	0.59
266291_at	putative tropinone reductase ;supp	---	AT2G29320	2.84	1.51	6.79	0.000305	0.004048	0.58
261570_at	fatty acid elongase 3-ketoacyl-CoA	KCS1	AT1G01120	-11.91	-3.57	-6.78	0.000307	0.004055	0.58
247079_at	Expressed protein ; supported by cl	AKRP	AT5G66055	-2.20	-1.14	-6.78	0.000307	0.004056	0.58
265214_at	unkown protein ESTs gb|H36249, g	---	AT1G05000	3.55	1.83	6.78	0.000308	0.004061	0.57
246926_at	putative protein	---	AT5G25240	-2.62	-1.39	-6.78	0.000308	0.004062	0.57
265499_at	putative glucosyltransferase	UGT73B5	AT2G15480	7.71	2.95	6.77	0.000309	0.004066	0.57
257206_at	putative lectin similar to lectin SP:P	---	AT3G16530	14.40	3.85	6.77	0.00031	0.004066	0.57
251973_at	nodulin / glutamate-ammonia ligase	---	AT3G53180	-2.66	-1.41	-6.77	0.00031	0.004066	0.57
255232_at	putative protein phosphatidylinosito	AGD13	AT4G05330	-2.11	-1.08	-6.77	0.000311	0.004074	0.56
264266_at	putative protein phosphatase 2C sir	---	AT1G09160	-2.17	-1.12	-6.77	0.000311	0.004077	0.56
260386_at	putative strictosidine synthase simil	---	AT1G74010	6.72	2.75	6.76	0.000312	0.004084	0.56
261801_at	hypothetical protein similar to hypot	AAE14	AT1G30520	-2.10	-1.07	-6.76	0.000314	0.004095	0.55
245662_at	hypothetical protein predicted by ge	---	AT1G28190	7.76	2.96	6.75	0.000316	0.004105	0.55
249750_at	unknown protein ;supported by full-	---	AT5G24570	-2.55	-1.35	-6.75	0.000316	0.004105	0.55
266721_at	unknown protein	FT1	AT2G03220	-2.07	-1.05	-6.74	0.000318	0.004124	0.54
258505_at	hypothetical protein predicted by ge	---	AT3G06530	4.73	2.24	6.73	0.000321	0.004152	0.53
252291_s_a	peroxidase ;supported by full-lengt	PRXCB	AT3G49110	2.78	1.48	6.73	0.000322	0.004154	0.53
248252_at	putative protein similar to unknown	AGP22	AT5G53250	-4.73	-2.24	-6.72	0.000323	0.004162	0.52
262093_at	receptor protein kinase, putative co	---	AT1G56145	2.37	1.24	6.72	0.000324	0.004162	0.52
251743_at	Yippee-like protein Yippee protein,	---	AT3G55890	3.30	1.72	6.71	0.000325	0.004177	0.51
266014_s_a	hypothetical protein	---	AT2G07722	3.06	1.61	6.71	0.000328	0.004202	0.50
255690_at	probable cytochrome P450	CYP86A2	AT4G00360	-13.52	-3.76	-6.71	0.000328	0.004202	0.50
253109_at	putative protein predicted protein A	MCA1	AT4G35920	-2.69	-1.43	-6.70	0.000328	0.004207	0.50
254250_at	serine/threonine kinase - like prote	---	AT4G23290	-3.00	-1.58	-6.70	0.000329	0.004209	0.50
259771_at	unknown protein	---	AT1G29470	-2.20	-1.14	-6.70	0.00033	0.004216	0.50
250490_at	pectin methylesterase - like protein	---	AT5G09760	-3.31	-1.73	-6.70	0.00033	0.004216	0.50
259878_at	putative catechol O-methyltransfera	---	AT1G76790	-3.34	-1.74	-6.70	0.00033	0.004216	0.50
246389_at	amino acid carrier, putative similar	AAP3	AT1G77380	2.14	1.09	6.69	0.000332	0.004217	0.49
252740_at	pectinesterase -like protein PECTII	---	AT3G43270	-2.37	-1.24	-6.69	0.000334	0.004231	0.49
258809_at	NAM-like protein (no apical meriste	anac047	AT3G04070	3.92	1.97	6.69	0.000334	0.004231	0.49

261216_at	catechol O-methyltransferase, puta	---	AT1G33030	5.64	2.50	6.67	0.000339	0.00426	0.47
245398_at	hydroxyproline-rich glycoprotein ho	---	AT4G14900	2.42	1.27	6.65	0.000344	0.004301	0.45
258415_at	putative s-adenosylmethionine synt	MTO3	AT3G17390	-3.18	-1.67	-6.64	0.000348	0.004333	0.44
246984_at	cytochrome P450	CYP81G1	AT5G67310	6.59	2.72	6.64	0.000349	0.004336	0.44
248028_at	putative protein similar to unknown	---	AT5G55620	-3.75	-1.91	-6.62	0.000352	0.00436	0.43
262237_at	hypothetical protein predicted by ge	---	AT1G48320	2.70	1.43	6.62	0.000354	0.004371	0.42
266481_at	unknown protein ; supported by cD	TCP10	AT2G31070	-2.03	-1.02	-6.61	0.000357	0.004397	0.41
255575_at	predicted protein of unknown funct	---	AT4G01430	4.41	2.14	6.61	0.000358	0.004404	0.41
266873_at	putative PREG1-like negative regul	CYCP4;1	AT2G44740	-6.42	-2.68	-6.60	0.000359	0.004416	0.40
253871_at	protochlorophyllide reductase precu	PORB	AT4G27440	-2.26	-1.18	-6.59	0.000362	0.004436	0.40
264315_at	aromatic rich glycoprotein, putative	---	AT1G70370	-6.37	-2.67	-6.58	0.000367	0.004463	0.38
262174_at	putative GDP-mannose pyrophosph	---	AT1G74910	-2.00	-1.00	-6.57	0.000369	0.004476	0.38
245078_at	putative AP2 domain transcription fa	---	AT2G23340	-4.31	-2.11	-6.57	0.00037	0.004478	0.37
251494_at	protein kinase-like protein Pto kina	---	AT3G59350	-3.48	-1.80	-6.56	0.000373	0.004514	0.36
264708_at	putative ER6 protein similar to GB:	---	AT1G09740	2.06	1.04	6.55	0.000378	0.004553	0.35
254241_at	serine/threonine kinase - like prote	CRK11	AT4G23190	6.26	2.65	6.54	0.000379	0.004556	0.35
259661_at	Expressed protein ; supported by fu	---	AT1G55265	2.03	1.02	6.54	0.00038	0.004563	0.34
251017_at	protein phosphatase - like protein p	---	AT5G02760	-22.54	-4.49	-6.54	0.000382	0.004574	0.34
250656_at	putative protein contains similarity t	---	AT5G06970	-2.08	-1.06	-6.53	0.000385	0.004598	0.33
257129_at	cytochrome P450, putative contain	CYP705A1	AT3G20100	2.42	1.28	6.52	0.000389	0.004627	0.32
264837_at	unknown protein similar to ESTs er	---	AT1G03600	-2.53	-1.34	-6.51	0.00039	0.00463	0.32
253736_at	Proline-rich APG - like protein proli	---	AT4G28780	-2.39	-1.26	-6.51	0.000391	0.004632	0.31
264102_at	hypothetical protein predicted by ge	ECT8	AT1G79270	2.06	1.04	6.51	0.000391	0.004632	0.31
251157_at	mRNA binding protein precursor - l	CSP41A	AT3G63140	-2.13	-1.09	-6.50	0.000396	0.004672	0.30
251899_at	nucleoid DNA-binding - like protein	---	AT3G54400	-12.37	-3.63	-6.49	0.000399	0.004701	0.29
265260_at	NAM (no apical meristem)-like prote	anac042	AT2G43000	13.57	3.76	6.49	0.000399	0.004701	0.29
263656_at	putative auxin-induced protein AUX	SHY2	AT1G04240	-13.81	-3.79	-6.48	0.000401	0.004721	0.28
266155_at	cytochrome p450, putative similar t	CYP89A5	AT1G64950	3.36	1.75	6.47	0.000406	0.004755	0.27
258394_at	unknown protein ;supported by full-	---	AT3G15530	-2.73	-1.45	-6.46	0.000408	0.004769	0.27
247246_at	invertase inhibitor homolog (emb|C/	C/VIF2	AT5G64620	-2.53	-1.34	-6.45	0.000413	0.004803	0.25
249552_s_a	receptor serine/threonine protein ki	---	AT5G38240	2.56	1.36	6.45	0.000414	0.004804	0.25
256098_at	unknown protein similar to 6-phospl	---	AT1G13700	-2.78	-1.47	-6.43	0.000419	0.004842	0.24
250646_at	peroxidase (emb|CAA68212.1) ;su	---	AT5G06720	2.06	1.04	6.43	0.000419	0.004843	0.24
254543_at	putative chitinase chitinase (EC 3.2	---	AT4G19810	5.55	2.47	6.43	0.00042	0.004845	0.24
255230_at	ferredoxin--NADP+ reductase - like	ATRFNR1	AT4G05390	6.29	2.65	6.42	0.000423	0.004872	0.23

261356_at	predicted protein	---	AT1G79660	-2.11	-1.08	-6.42	0.000424	0.004884	0.22
254101_at	alpha-amylase - like protein alpha-	AMY1	AT4G25000	4.69	2.23	6.41	0.000428	0.00491	0.22
247037_at	putative protein similar to unknown	RALFL34	AT5G67070	-12.41	-3.63	-6.41	0.000429	0.00491	0.21
256655_at	hypothetical protein predicted by ge	---	AT3G18890	-2.23	-1.15	-6.40	0.00043	0.004915	0.21
248095_at	putative protein strong similarity to u	ATMAP65-	AT5G55230	-2.00	-1.00	-6.40	0.000431	0.004915	0.21
252320_at	endoxyloglucan transferase-like pro	---	AT3G48580	3.46	1.79	6.39	0.000434	0.004945	0.20
258708_at	putative oxidoreductase similar to p	---	AT3G09580	-2.22	-1.15	-6.39	0.000437	0.004959	0.19
260267_at	very-long-chain fatty acid condensi	KCS6	AT1G68530	-3.09	-1.63	-6.37	0.000445	0.005023	0.17
261109_at	cytokinin oxidase, putative similar t	CKX5	AT1G75450	-9.81	-3.29	-6.37	0.000446	0.005029	0.17
263942_at	unknown protein	FLA16	AT2G35860	-17.79	-4.15	-6.36	0.000446	0.005029	0.17
254417_at	putative protein	ATFMN/FH	AT4G21470	2.14	1.10	6.35	0.000452	0.005063	0.15
257506_at	auxin-induced protein, putative sim	---	AT1G29440	-6.36	-2.67	-6.35	0.000453	0.005066	0.15
264318_at	putative beta-ketoacyl-CoA synthas	KCS2	AT1G04220	-2.74	-1.46	-6.34	0.000455	0.005072	0.15
255676_at	putative beta-amylase similar to the	BAM2	AT4G00490	-3.31	-1.73	-6.34	0.000455	0.005072	0.15
264836_at	unknown protein similar to hypothet	---	AT1G03610	-2.30	-1.20	-6.34	0.000456	0.005072	0.14
255962_at	glycine-rich RNA-binding protein, p	---	AT1G22330	-6.29	-2.65	-6.33	0.000462	0.005102	0.13
254553_at	TMV resistance protein N - like TM	---	AT4G19530	-2.64	-1.40	-6.33	0.000462	0.005102	0.13
259707_at	thylakoid-bound ascorbate peroxida	TAPX	AT1G77490	-2.09	-1.06	-6.33	0.000462	0.005102	0.13
260939_at	hypothetical protein contains simila	---	AT1G45180	-3.65	-1.87	-6.32	0.000467	0.005127	0.12
257805_at	sugar transport, putative similar to	ATPLT5	AT3G18830	2.78	1.48	6.31	0.000468	0.005137	0.12
257365_x_a	putative antifungal protein	PDF1.2b	AT2G26020	13.44	3.75	6.31	0.000471	0.005162	0.11
246831_at	hexose transporter - like protein he	MSS1	AT5G26340	2.40	1.26	6.30	0.000474	0.005187	0.10
256385_at	60S ribosomal protein L10, putative	---	AT1G66580	2.41	1.27	6.29	0.000477	0.005205	0.10
262119_s_a	glutathione S-transferase, putative	ATGSTF6	AT1G02920	5.46	2.45	6.27	0.000485	0.005255	0.08
251109_at	ferritin 1 precursor ;supported by fu	FER1	AT5G01600	2.92	1.55	6.25	0.000496	0.00533	0.05
264195_at	putative gibberellin-regulated protei	---	AT1G22690	-2.42	-1.28	-6.24	0.000499	0.005355	0.05
258393_at	unknown protein similar to unknown	---	AT3G15480	-2.25	-1.17	-6.24	0.000503	0.005385	0.04
254328_at	adenine phosphoribosyltransferase	APT3	AT4G22570	-2.68	-1.42	-6.24	0.000503	0.005385	0.04
254137_at	hypothetical protein	---	AT4G24930	-2.43	-1.28	-6.23	0.000504	0.005396	0.04
259015_at	unknown protein similar to hypothet	---	AT3G07350	-3.61	-1.85	-6.23	0.000507	0.005416	0.03
255561_at	putative hexose transporter similar t	---	AT4G02050	2.15	1.11	6.23	0.000507	0.005418	0.03
250471_at	myo-inositol-1-phosphate synthase	MIPS3	AT5G10170	-2.20	-1.14	-6.22	0.00051	0.005431	0.02
258836_at	hypothetical protein predicted using	---	AT3G07210	-2.03	-1.03	-6.21	0.000515	0.005464	0.01
253044_at	hypothetical protein ;supported by f	---	AT4G37290	19.26	4.27	6.20	0.000518	0.005486	0.01
261618_at	unknown protein	---	AT1G33110	3.01	1.59	6.19	0.000524	0.005528	-0.01

252970_at	small auxin up RNA (SAUR-AC1) ;	SAUR15	AT4G38850	-2.31	-1.21	-6.19	0.000526	0.00554	-0.01
261309_at	phosphoethanolamine N-methyltra	CPuORF31	AT1G48598	-2.34	-1.23	-6.19	0.000527	0.005548	-0.01
245007_at	PSI P700 apoprotein A1	---	---	-2.46	-1.30	-6.18	0.000529	0.005557	-0.02
252852_at	hypothetical protein ;supported by	---	AT4G39900	-2.92	-1.55	-6.16	0.000538	0.005609	-0.04
263706_s_a	3-methyladenine DNA glycosylase,	---	AT1G31180	-2.29	-1.20	-6.16	0.000539	0.00561	-0.04
261395_at	unknown protein similar to putative	---	AT1G79700	-3.37	-1.75	-6.16	0.00054	0.005611	-0.04
262935_at	hypothetical protein similar to putati	AtOCT5	AT1G79410	4.12	2.04	6.16	0.00054	0.005611	-0.04
257623_at	cytochrome P450, putative contain	CYP71B23	AT3G26210	4.57	2.19	6.16	0.000541	0.005613	-0.04
265620_at	unknown protein	---	AT2G27310	4.69	2.23	6.15	0.000543	0.005628	-0.05
257113_at	cytochrome P450, putative contain	CYP705A2	AT3G20130	-2.78	-1.48	-6.15	0.000543	0.005629	-0.05
256756_at	ATPase II, putative similar to GB:A	---	AT3G25610	3.16	1.66	6.15	0.000548	0.005658	-0.05
250233_at	putative protein weak homology wi	IQD11	AT5G13460	-5.46	-2.45	-6.14	0.00055	0.00567	-0.06
261740_at	hypothetical protein similar to unkno	---	AT1G47740	-2.03	-1.02	-6.14	0.000551	0.005682	-0.06
259403_at	unknown protein ; supported by full-	PGDH	AT1G17745	7.25	2.86	6.14	0.000553	0.005692	-0.07
263962_at	putative protein kinase contains a p	---	AT2G36350	-2.03	-1.02	-6.14	0.000553	0.005692	-0.07
AFFX-Athal-	Arabidopsis thaliana /REF=M64116	GAPC1	---	2.15	1.11	6.13	0.000555	0.005698	-0.07
259791_at	unknown protein ; supported by cDI	---	AT1G29700	-2.44	-1.28	-6.13	0.000557	0.005717	-0.07
255571_at	hypothetical protein ; supported by	---	AT4G01220	-2.25	-1.17	-6.13	0.000558	0.005719	-0.07
260744_at	hypothetical protein predicted by ge	---	AT1G15010	-8.94	-3.16	-6.12	0.00056	0.005722	-0.08
257624_at	cytochrome P450 monooxygenase	CYP71B3	AT3G26220	6.45	2.69	6.12	0.000563	0.00574	-0.08
247189_at	unknown protein ;supported by full-	AGP7	AT5G65390	-4.17	-2.06	-6.11	0.000565	0.005756	-0.09
249932_at	unknown protein	---	AT5G22390	-3.88	-1.96	-6.11	0.000566	0.005763	-0.09
264394_at	aminomethyltransferase-like precu	---	AT1G11860	-2.12	-1.09	-6.11	0.000569	0.005786	-0.10
264574_at	putative zinc transporter Similar to	ZIP5	AT1G05300	2.78	1.47	6.10	0.000574	0.005821	-0.11
248656_at	fimbrin 2 (gb|AAB97844.1) ; suppo	---	AT5G48460	-4.03	-2.01	-6.10	0.000575	0.005822	-0.11
262744_at	anthranilate N-hydroxycinnamoyl/b	---	AT1G28680	2.38	1.25	6.09	0.000576	0.005828	-0.11
257556_at	hypothetical protein predicted by ge	---	AT3G28100	3.45	1.79	6.09	0.000579	0.005849	-0.12
254066_at	transcriptional activator CBF1-like	DREB1A	AT4G25480	4.01	2.00	6.08	0.000581	0.005853	-0.12
266163_at	hypothetical protein predicted by ge	---	AT2G28130	-2.16	-1.11	-6.08	0.000582	0.005858	-0.12
256883_at	unknown protein ; supported by cD	---	AT3G26440	4.41	2.14	6.08	0.000582	0.005859	-0.12
264611_at	putative pectate lyase A11 similar t	---	AT1G04680	-7.17	-2.84	-6.08	0.000585	0.005876	-0.13
254215_at	putative Na+/H+-exchanging protei	ATCHX17	AT4G23700	7.27	2.86	6.08	0.000585	0.005876	-0.13
261394_at	wall-associated kinase 2, putative	---	AT1G79680	12.10	3.60	6.07	0.000587	0.00589	-0.13
247351_at	putative protein contains similarity t	ANAC102	AT5G63790	3.57	1.84	6.07	0.000587	0.00589	-0.13
249550_at	protein kinase - like protein wall-as	---	AT5G38210	2.64	1.40	6.07	0.000589	0.005908	-0.13

258879_at	unknown protein ; supported by ful	---	AT3G03270	2.77	1.47	6.07	0.000591	0.005921	-0.14
253638_at	cinnamoyl-CoA reductase - like pro	---	AT4G30470	-3.85	-1.95	-6.06	0.000597	0.005966	-0.15
245399_at	membrane channel like protein ;sup	TIP2;2	AT4G17340	-2.53	-1.34	-6.05	0.0006	0.005986	-0.15
256451_s_a	unknown protein	---	AT1G75170	3.84	1.94	6.05	0.000601	0.005989	-0.15
264645_at	unknown protein Similar to Saccha	---	AT1G08940	4.41	2.14	6.05	0.000601	0.005991	-0.16
261366_at	unknown protein	---	AT1G53100	3.67	1.87	6.05	0.000601	0.005991	-0.16
260203_at	NAM-like protein similar to NAM (no	ANAC019	AT1G52890	2.42	1.27	6.04	0.000604	0.006006	-0.16
249057_at	putative protein contains similarity t	DUR	AT5G44480	5.42	2.44	6.04	0.000606	0.006015	-0.16
252876_at	putative protein ;supported by full-l	---	AT4G39970	-2.19	-1.13	-6.04	0.000607	0.006018	-0.17
265342_at	hypothetical protein predicted by ge	---	AT2G18300	-2.50	-1.32	-6.03	0.000612	0.00606	-0.18
253372_at	pectinesterase - like protein pectin	PME44	AT4G33220	-3.54	-1.82	-6.03	0.000613	0.006065	-0.18
261459_at	O-methyltransferase, putative simil	---	AT1G21100	12.01	3.59	6.01	0.000622	0.006129	-0.19
267057_at	hypothetical protein predicted by ge	---	AT2G32500	-3.25	-1.70	-6.01	0.000622	0.006129	-0.19
254601_at	putative protein DNA (cytosine-5-)-	CMT2	AT4G19020	-2.08	-1.05	-6.01	0.000625	0.006151	-0.20
253829_at	Medicago nodulin N21-like protein	---	AT4G28040	5.14	2.36	6.00	0.000629	0.006172	-0.20
253423_at	Expressed protein ; supported by cl	IAA29	AT4G32280	-10.89	-3.45	-6.00	0.00063	0.006182	-0.21
245213_at	photosystem II 22kDa protein, puta	NPQ4	AT1G44575	-3.45	-1.79	-5.98	0.000641	0.006257	-0.23
251479_at	serine/threonine-specific kinase lec	ATHLECRK	AT3G59700	3.59	1.84	5.97	0.000651	0.006316	-0.24
256446_at	RING zinc finger protein contains F	---	AT3G11110	-2.31	-1.21	-5.96	0.000656	0.006347	-0.25
254041_at	Expressed protein ; supported by fu	---	AT4G25830	-4.75	-2.25	-5.96	0.000657	0.006353	-0.25
264024_at	hypothetical protein predicted by ge	---	AT2G21180	2.55	1.35	5.95	0.000658	0.006355	-0.25
249469_at	UDP-glucose dehydrogenase-like p	---	AT5G39320	-3.13	-1.65	-5.95	0.000661	0.006374	-0.26
254040_at	possible apospory-associated like p	---	AT4G25900	2.21	1.15	5.94	0.000665	0.006389	-0.27
261861_at	hypothetical protein predicted by ge	---	AT1G50450	-2.74	-1.45	-5.94	0.000666	0.006399	-0.27
259787_at	auxin-induced protein, putative sim	---	AT1G29460	-4.34	-2.12	-5.94	0.000671	0.006424	-0.28
248198_at	putative protein contains similarity t	---	AT5G54200	-2.63	-1.40	-5.93	0.000673	0.006441	-0.28
257323_at	hypothetical protein	---	AT2G07698	3.59	1.84	5.92	0.00068	0.006492	-0.29
245385_at	hypothetical protein ;supported by f	---	AT4G14020	2.01	1.01	5.91	0.000686	0.006524	-0.30
262682_at	anter-specific proline-rich -like prote	---	AT1G75900	-4.80	-2.26	-5.91	0.000688	0.006537	-0.30
266044_s_a	hypothetical protein	---	AT2G07725	2.55	1.35	5.91	0.00069	0.00655	-0.31
262235_at	ribosomal protein L18, putative sim	---	AT1G48350	-2.02	-1.01	-5.90	0.000693	0.006567	-0.31
261455_at	unknown protein ;supported by full-	---	AT1G21070	-2.17	-1.12	-5.89	0.000702	0.00662	-0.32
264261_at	putative nicotianamine synthase sin	NAS3	AT1G09240	6.39	2.68	5.88	0.000706	0.006633	-0.33
263410_at	predicted protein ;supported by full-	---	AT2G04039	-2.21	-1.14	-5.87	0.000711	0.006672	-0.34
265877_at	unknown protein ; supported by cD	BZIP34	AT2G42380	-2.39	-1.26	-5.87	0.000715	0.006699	-0.34

245460_at	disease resistance RPP5 like protei	RLM3	AT4G16990	-2.53	-1.34	-5.86	0.000722	0.00675	-0.36
257314_at	integral membrane protein, putative	---	AT3G26590	3.24	1.69	5.85	0.000728	0.006795	-0.37
256411_at	ATP-dependent Clp protease protec	CLPP3	AT1G66670	2.14	1.10	5.82	0.000747	0.006922	-0.39
253040_at	endo-xyloglucan transferase - like	---	AT4G37800	-5.03	-2.33	-5.81	0.000761	0.007018	-0.41
254221_at	putative polygalacturonase polygal	---	AT4G23820	-6.22	-2.64	-5.80	0.000762	0.007018	-0.41
262047_at	hypothetical protein predicted by ge	---	AT1G80160	6.64	2.73	5.80	0.000766	0.007046	-0.42
252023_at	putative protein various predicted p	---	AT3G52920	3.15	1.66	5.79	0.000775	0.007108	-0.43
253550_at	putative protein kinase serine/threo	SIP3	AT4G30960	2.27	1.18	5.78	0.000779	0.007135	-0.44
246011_at	putative protein auxin-induced basi	---	AT5G08330	-2.59	-1.37	-5.78	0.000783	0.007154	-0.44
263517_at	unknown protein ;supported by full-	RD2	AT2G21620	2.03	1.02	5.77	0.000789	0.007177	-0.45
254500_at	vacuolar sorting receptor-like prote	---	AT4G20110	2.83	1.50	5.76	0.000794	0.007197	-0.46
262496_at	unknown protein ; supported by full-	---	AT1G21790	-2.40	-1.26	-5.76	0.000794	0.007197	-0.46
266327_at	homeodomain transcription factor (	ATHB-7	AT2G46680	2.65	1.41	5.76	0.000795	0.007197	-0.46
250337_at	putative protein SF21 protein, Helia	---	AT5G11790	-4.47	-2.16	-5.75	0.000802	0.007243	-0.47
266167_at	unknown protein ;supported by full-	YLS5	AT2G38860	4.86	2.28	5.75	0.000805	0.007261	-0.47
249188_at	N-hydroxycinnamoyl benzoyltransfe	---	AT5G42830	3.31	1.73	5.75	0.000808	0.007276	-0.48
262850_at	signal response protein (GAI) identi	GAI	AT1G14920	-2.47	-1.30	-5.75	0.000808	0.007277	-0.48
265411_at	unknown protein	---	AT2G16630	-5.04	-2.33	-5.74	0.000809	0.007282	-0.48
262920_at	2-dehydro-3-deoxyphosphooctonat	---	AT1G79500	-2.85	-1.51	-5.74	0.00081	0.007282	-0.48
257880_at	AMP-binding protein, putative cont	AAE7	AT3G16910	2.32	1.21	5.74	0.000811	0.007282	-0.48
261526_at	protein kinase identical to protein ki	APK2A	AT1G14370	2.28	1.19	5.74	0.000812	0.007282	-0.48
258203_at	hypothetical protein predicted by ge	---	AT3G13950	8.62	3.11	5.73	0.000817	0.007311	-0.49
265183_at	unknown protein ;supported by full-	---	AT1G23750	-2.18	-1.12	-5.73	0.00082	0.007323	-0.49
267503_at	unknown protein ; supported by ful	---	AT2G45600	-2.15	-1.10	-5.73	0.000821	0.007328	-0.50
262357_at	jacalin, putative similar to jacalin G	---	AT1G73040	4.22	2.08	5.72	0.000828	0.007368	-0.50
245252_at	ethylene responsive element bindin	ATERF-1	AT4G17500	4.18	2.06	5.72	0.00083	0.007381	-0.51
259783_at	auxin-induced protein, putative sim	SAUR68	AT1G29510	-8.69	-3.12	-5.71	0.000837	0.007407	-0.52
254299_at	hypothetical protein ;supported by	NYE1	AT4G22920	2.51	1.33	5.71	0.000841	0.007431	-0.52
248402_at	dihydrodipicolinate reductase-like p	crr1	AT5G52100	-2.17	-1.12	-5.71	0.000841	0.007431	-0.52
256713_at	putative peroxidase	---	AT2G34060	-2.15	-1.10	-5.70	0.000844	0.007447	-0.53
260547_at	putative trypsin inhibitor ;supportec	---	AT2G43550	-5.01	-2.33	-5.70	0.000844	0.007447	-0.53
257045_at	dynamin-like protein similar to phrag	ARC5	AT3G19720	-2.10	-1.07	-5.70	0.000847	0.007459	-0.53
254598_at	xyloglucan endo-transglycosylase -	---	AT4G18990	2.68	1.42	5.70	0.000849	0.007466	-0.53
248962_at	putative protein contains similarity t	FKBP13	AT5G45680	-2.14	-1.10	-5.69	0.000857	0.007516	-0.54
249306_at	RING zinc finger protein-like ;suppo	---	AT5G41400	3.23	1.69	5.67	0.000871	0.00759	-0.56

252834_at	putative protein RING-H2 zinc finge	---	AT4G40070	-2.53	-1.34	-5.67	0.000872	0.007599	-0.56
265450_at	hypothetical protein predicted by ge	---	AT2G46620	2.09	1.06	5.66	0.000879	0.007641	-0.57
253263_at	abscisic acid responsive elements-l	ABF3	AT4G34000	2.76	1.47	5.66	0.000881	0.007651	-0.57
246212_at	putative protein ; supported by cDN	SPT	AT4G36930	4.00	2.00	5.66	0.000884	0.007653	-0.58
258277_at	putative cytochrome P450 similar to	PAD3	AT3G26830	26.78	4.74	5.65	0.000885	0.007653	-0.58
267034_at	unknown protein ;supported by full-	---	AT2G38310	-3.94	-1.98	-5.64	0.000897	0.007719	-0.59
251281_at	putative protein hypothetical protei	AGP20	AT3G61640	-3.19	-1.67	-5.64	0.000899	0.007729	-0.59
260930_at	GTP-binding protein Sar1, putative	---	AT1G02620	-2.48	-1.31	-5.64	0.000901	0.007735	-0.60
259103_at	unknown protein ; supported by cD	---	AT3G11690	-2.97	-1.57	-5.64	0.000901	0.007735	-0.60
256407_at	sucrose-proton symporter, putative	ATSUC7	AT1G66570	4.89	2.29	5.64	0.000903	0.007745	-0.60
254673_at	membrane-bound small GTP-bindi	AtRABA1e	AT4G18430	3.80	1.93	5.63	0.000905	0.00776	-0.60
251096_at	receptor like protein kinase recepto	LECRKA4.:	AT5G01550	2.19	1.13	5.61	0.000927	0.007877	-0.63
259970_at	putative chlorophyll A-B binding pro	---	AT1G76570	-2.09	-1.06	-5.60	0.000932	0.007905	-0.63
267607_s_a	epoxide hydrolase (ATsEH) identica	ATSEH	AT2G26740 ,	-2.25	-1.17	-5.60	0.000933	0.007906	-0.64
247026_at	protein kinase-like protein	MAPKKK1¢	AT5G67080	11.32	3.50	5.60	0.000939	0.007947	-0.64
262980_at	endo-beta-1,4-glucanase, putative	AtGH9B7	AT1G75680	-6.86	-2.78	-5.59	0.000947	0.008002	-0.65
261025_at	Expressed protein ; supported by fu	---	AT1G01225	-2.26	-1.18	-5.57	0.000967	0.008115	-0.67
257264_at	hypothetical protein contains Pfam	---	AT3G22060	6.29	2.65	5.56	0.000972	0.008137	-0.68
257951_at	putative SGP1 monomeric G-protei	SGP2	AT3G21700	2.91	1.54	5.56	0.000977	0.008166	-0.69
246607_at	receptor-like protein kinase - like pr	---	AT5G35370	2.81	1.49	5.55	0.000983	0.008197	-0.69
245861_at	GTL1 - like protein GTL1, Arabidop	---	AT5G28300	-2.56	-1.35	-5.53	0.001007	0.008345	-0.72
261129_at	tubulin alpha-2/alpha-4 chain identi	TUA4	AT1G04820	-3.16	-1.66	-5.52	0.001013	0.008384	-0.73
261425_at	peptide transporter, putative simila	---	AT1G18880	2.62	1.39	5.51	0.001027	0.008483	-0.74
267645_at	putative beta-glucosidase	BGLU33	AT2G32860	-3.43	-1.78	-5.50	0.00103	0.00849	-0.74
247215_at	Expressed protein ; supported by fu	PROPEP3	AT5G64905	12.09	3.60	5.50	0.001032	0.008497	-0.75
250847_at	putative protein predicted protein, /	---	AT5G04480	-2.46	-1.30	-5.50	0.001033	0.008504	-0.75
261413_at	unknown protein ;supported by full-	PLL5	AT1G07630	2.89	1.53	5.48	0.001056	0.008631	-0.77
267549_at	hypothetical protein predicted by ge	---	AT2G32640	-3.39	-1.76	-5.47	0.00107	0.008693	-0.78
253747_at	serine threonine-specific kinase like	---	AT4G29050	2.33	1.22	5.47	0.001072	0.008702	-0.79
266971_at	unknown protein	---	AT2G39580	-2.46	-1.30	-5.45	0.001086	0.008763	-0.80
257983_at	unknown protein	---	AT3G20790	-3.17	-1.66	-5.45	0.001095	0.0088	-0.81
265067_at	hypothetical protein predicted by ge	---	AT1G03850	4.09	2.03	5.44	0.001101	0.008836	-0.82
254448_at	fatty acid hydroxylase - like protein	FAH2	AT4G20870	-3.54	-1.82	-5.44	0.001105	0.008857	-0.82
257093_at	hypothetical protein ;supported by t	---	AT3G20570	-2.10	-1.07	-5.43	0.001112	0.008881	-0.83
252624_at	Expressed protein ; supported by fu	PSK1	AT3G44735	-2.62	-1.39	-5.42	0.001124	0.008929	-0.84

264616_at	unknown protein	---	AT2G17740	12.63	3.66	5.41	0.001133	0.008953	-0.85
255719_at	unknown protein ;supported by full-	---	AT1G32080	-2.59	-1.38	-5.41	0.001134	0.008953	-0.85
251387_at	putative protein	---	AT3G60810	-2.04	-1.03	-5.41	0.001136	0.008964	-0.85
249677_at	DNA helicase-like	---	AT5G35970	-2.19	-1.13	-5.40	0.001146	0.009014	-0.86
248420_at	receptor-like protein kinase ;suppo	---	AT5G51560	-3.35	-1.74	-5.39	0.001157	0.009057	-0.87
264635_at	unknown protein ;supported by full-	---	AT1G65500	3.34	1.74	5.39	0.001158	0.009057	-0.87
264435_at	putative glutathione S-transferase T	ATGSTU18	AT1G10360	-2.16	-1.11	-5.39	0.001158	0.009057	-0.87
263126_at	hypothetical protein predicted by ge	---	AT1G78460	4.26	2.09	5.39	0.001163	0.009081	-0.88
253052_at	cytochrome P450-like protein ; supp	CYP81H1	AT4G37310	2.77	1.47	5.39	0.001164	0.009083	-0.88
259211_at	unknown protein identical to GB:AA	---	AT3G09020	4.71	2.24	5.38	0.001173	0.009124	-0.88
260542_at	putative FKBP type peptidyl-prolyl c	---	AT2G43560	-2.13	-1.09	-5.36	0.001192	0.00922	-0.90
261688_at	unknown protein contains similarity	---	AT1G47380	-3.56	-1.83	-5.35	0.001204	0.009259	-0.91
264324_at	myosin heavy chain MYA2 Strong s	XIB	AT1G04160	-2.32	-1.21	-5.35	0.001209	0.009285	-0.92
258815_at	putative short-chain type dehydroge	---	AT3G04000	2.18	1.12	5.34	0.001219	0.009331	-0.93
267169_at	putative oxidoreductase	---	AT2G37540	-2.48	-1.31	-5.34	0.001223	0.009345	-0.93
258662_at	unknown protein	---	AT3G02900	-2.48	-1.31	-5.34	0.001226	0.009363	-0.93
260438_at	putative bifunctional nuclease simila	ENDO 2	AT1G68290	-2.94	-1.56	-5.33	0.001229	0.009366	-0.94
252572_at	putative protein Mlo-h1 protein - Ho	MLO3	AT3G45290	2.11	1.08	5.33	0.00124	0.009419	-0.95
244995_at	ATPase a subunit	---	---	-2.26	-1.18	-5.32	0.001247	0.009453	-0.95
260490_at	ATP-dependent transmembrane tra	CER5	AT1G51500	-2.32	-1.21	-5.30	0.001269	0.009559	-0.97
251962_at	plasma membrane intrinsic protein	PIP2A	AT3G53420	-2.14	-1.10	-5.29	0.001289	0.009661	-0.99
252334_at	mitochondrial phosphate transporte	---	AT3G48850	6.93	2.79	5.29	0.001291	0.009661	-0.99
262263_at	auxin transport protein REH1, puta	PIN3	AT1G70940	-3.45	-1.79	-5.28	0.001295	0.00969	-0.99
250550_at	N-hydroxycinnamoyl benzoyltransf	---	AT5G07870	-2.49	-1.32	-5.28	0.001296	0.009697	-0.99
262796_at	cysteine proteinase XCP2 identical	XCP2	AT1G20850	-3.15	-1.66	-5.28	0.001298	0.009709	-1.00
267038_at	expressed protein supported by cD	---	AT2G38480	-2.86	-1.52	-5.28	0.001303	0.009734	-1.00
255177_at	strong similarity to 1-aminocyclopro	ACS11	AT4G08040	-3.78	-1.92	-5.28	0.001305	0.009737	-1.00
260371_at	unknown protein ; supported by cD	---	AT1G69690	-3.00	-1.59	-5.28	0.001306	0.009737	-1.00
250307_at	putative protein similar to unknown	---	AT5G12170	2.12	1.08	5.27	0.001313	0.009757	-1.01
245783_s_a	hypothetical protein similar to hypot	---	AT1G35170	-3.26	-1.70	-5.27	0.001314	0.00976	-1.01
251920_at	uracil phosphoribosyltransferase-lil	---	AT3G53900	-2.47	-1.30	-5.26	0.001326	0.009802	-1.02
251072_at	putative protein wound-inducible pr	---	AT5G01740	-2.40	-1.26	-5.26	0.001328	0.009805	-1.02
245657_at	unknown protein similar to receptor	---	AT1G56720	-3.07	-1.62	-5.26	0.001328	0.009805	-1.02
265740_at	RING-H2 finger protein RHA2b iden	RHA2B	AT2G01150	-2.64	-1.40	-5.24	0.001362	0.009973	-1.05
247199_at	DNA binding protein TGA1a homolo	TGA1	AT5G65210	2.31	1.21	5.23	0.001373	0.01003	-1.06

253987_at	pyrophosphate-dependent phosph	PFK3	AT4G26270	2.57	1.36	5.23	0.001378	0.010046	-1.06
251196_at	glutaredoxin -like protein glutaredo	---	AT3G62950	-3.62	-1.86	-5.23	0.001379	0.010046	-1.06
245352_at	indole-3-acetate beta-glucosyltrans	UGT84A3	AT4G15490	2.09	1.06	5.22	0.001385	0.010068	-1.07
258419_at	unknown protein	---	AT3G16670	-6.23	-2.64	-5.21	0.001395	0.010107	-1.07
265720_at	unknown protein	---	AT2G40110	2.41	1.27	5.21	0.001406	0.010167	-1.08
258682_at	putative ribosomal-protein S6 kinas	S6K2	AT3G08720	2.90	1.54	5.21	0.001407	0.010169	-1.08
250199_at	putative protein contains similarity t	MPL1	AT5G14180	4.23	2.08	5.21	0.001409	0.010179	-1.09
247166_at	unknown protein	---	AT5G65840	-2.25	-1.17	-5.20	0.00142	0.010216	-1.09
249361_at	protein kinase - like protein protein	---	AT5G40540	-2.35	-1.23	-5.20	0.001424	0.010231	-1.10
248162_at	1,4-benzoquinone reductase-like; T	FQR1	AT5G54500	2.04	1.03	5.19	0.001429	0.01026	-1.10
248687_at	ADPG pyrophosphorylase small sul	ADG1	AT5G48300	-2.33	-1.22	-5.19	0.001431	0.010263	-1.10
250611_at	gibberellin 20-oxidase	GA20OX3	AT5G07200	-2.00	-1.00	-5.19	0.001434	0.010276	-1.10
259516_at	hypothetical protein ; supported by	ERD10	AT1G20450	-2.35	-1.23	-5.19	0.001438	0.010283	-1.11
262280_at	unknown protein	---	AT1G68580	-2.24	-1.16	-5.19	0.001439	0.010284	-1.11
248335_at	putative protein strong similarity to	---	AT5G52450	2.05	1.04	5.18	0.001446	0.010326	-1.11
251100_at	aldose reductase-like protein aldos	---	AT5G01670	2.14	1.10	5.18	0.001448	0.010327	-1.12
266156_at	hypothetical protein predicted by ge	FRA8	AT2G28110	3.89	1.96	5.18	0.00145	0.01033	-1.12
260919_at	hypothetical protein predicted by ge	---	AT1G21520	4.69	2.23	5.16	0.001476	0.010462	-1.14
247478_at	DC1.2 homologue - like protein DC	---	AT5G62360	-3.64	-1.87	-5.16	0.00148	0.010478	-1.14
255926_at	AP2 domain containing protein RA	---	AT1G22190	2.43	1.28	5.15	0.00149	0.010539	-1.15
256112_at	guanine nucleotide regulatory prote	ATRABA1E	AT1G16920	-3.22	-1.69	-5.14	0.001509	0.010628	-1.16
267619_at	putative receptor-like protein kinase	---	AT2G26730	-2.15	-1.10	-5.14	0.00151	0.010636	-1.16
262309_at	phosphoglucomutase, putative sim	---	AT1G70820	-2.31	-1.21	-5.13	0.001522	0.01069	-1.17
250777_at	putative protein similar to unknown	---	AT5G05440	-5.42	-2.44	-5.13	0.00153	0.010724	-1.17
266368_at	putative embryo-abundant protein	---	AT2G41380	10.11	3.34	5.12	0.001546	0.010813	-1.19
261788_at	unknown protein ;supported by full-	NDF1	AT1G15980	-2.17	-1.12	-5.12	0.001546	0.010813	-1.19
265221_s_a	putative glutamate decarboxylase ;	GAD3	AT2G02000	4.59	2.20	5.11	0.001556	0.010858	-1.19
262195_at	allergen, putative similar to allerge	---	AT1G78040	-2.57	-1.36	-5.11	0.001566	0.0109	-1.20
256673_at	unknown protein	FLA15	AT3G52370	-5.10	-2.35	-5.10	0.001572	0.010917	-1.20
253207_at	putative protein small auxin up-reg	---	AT4G34770	-3.20	-1.68	-5.10	0.001578	0.010938	-1.21
260221_at	GAST1-like protein similar to GAST	---	AT1G74670	-7.40	-2.89	-5.10	0.001581	0.010952	-1.21
259297_at	putative disease resistance protein	AtRLP30	AT3G05360	2.85	1.51	5.09	0.001586	0.010963	-1.21
266292_at	putative tropinone reductase ;supp	SAG13	AT2G29350	13.90	3.80	5.09	0.001591	0.010984	-1.22
261032_at	unknown protein ;supported by full-	---	AT1G17430	-3.27	-1.71	-5.09	0.001594	0.010995	-1.22
254592_at	heat shock transcription factor - like	HSF A4A	AT4G18880	3.54	1.82	5.07	0.001623	0.011115	-1.24

253608_at	xyloglucan endo-1,4-beta-D-glucar	XTH19	AT4G30290	-3.80	-1.93	-5.07	0.00163	0.011136	-1.24
266856_at	putative ABC transporter related to	PDR4	AT2G26910	-2.20	-1.14	-5.04	0.001674	0.011359	-1.27
260982_at	chalcone isomerase, putative simila	---	AT1G53520	-3.66	-1.87	-5.04	0.001687	0.011412	-1.28
264400_at	glucose-6-phosphate/phosphate-tra	GPT2	AT1G61800	10.64	3.41	5.04	0.00169	0.011421	-1.28
266984_at	unknown protein ; supported by cD	---	AT2G39570	2.19	1.13	5.03	0.001693	0.011436	-1.29
251621_at	protein kinase - like protein wall-as	---	AT3G57700	2.08	1.06	5.03	0.001695	0.011436	-1.29
257333_at	cytochrome c oxidase subunit 1	---	---	2.24	1.16	5.02	0.001712	0.011496	-1.30
260153_at	putative lipase similar to monoglyce	---	AT1G52760	2.21	1.14	5.02	0.001725	0.011554	-1.31
252300_at	pyruvate kinase -like protein various	---	AT3G49160	2.12	1.08	5.02	0.001726	0.011555	-1.31
250012_x_a	auxin-induced protein-like	---	AT5G18060	-8.50	-3.09	-5.01	0.001735	0.011579	-1.31
266503_at	unknown protein	---	AT2G47780	-2.27	-1.19	-5.00	0.001758	0.011684	-1.33
246233_at	putative protein	---	AT4G36550	2.89	1.53	5.00	0.001763	0.011699	-1.33
250293_s_a	auxin reponsive - like protein Nt-gh	---	AT5G13360 ,	2.53	1.34	4.99	0.001768	0.011716	-1.33
248344_at	hyaluronan mediated motility recept	---	AT5G52280	-2.09	-1.06	-4.99	0.00178	0.011773	-1.34
263287_at	Expressed protein ; supported by fu	---	AT2G36145	-3.10	-1.63	-4.98	0.001793	0.011826	-1.35
260704_at	glycine cleavage system H protein	---	AT1G32470	-3.26	-1.70	-4.97	0.001811	0.011883	-1.36
254384_at	heat shock protein - like heat shock	---	AT4G21870	-3.33	-1.74	-4.97	0.00182	0.01191	-1.36
251665_at	responce reactor 4 ; supported by c	ARR9	AT3G57040	-2.88	-1.53	-4.96	0.001834	0.011955	-1.37
260522_x_a	hypothetical protein predicted by ge	---	AT2G41730	8.17	3.03	4.96	0.001835	0.011955	-1.37
258871_at	putative 26S proteosome regulatory	---	AT3G03060	2.51	1.33	4.96	0.001846	0.012005	-1.38
260884_at	unknown protein similar to unknown	---	AT1G29240	2.59	1.37	4.95	0.001847	0.012011	-1.38
245906_at	putative protein ;supported by full-l	---	AT5G11070	-2.49	-1.32	-4.94	0.001872	0.012113	-1.39
258000_at	AIG2-like protein similar to AIG2 pro	---	AT3G28940	2.08	1.05	4.94	0.001877	0.012128	-1.40
262210_at	hypothetical protein predicted by ge	IQD31	AT1G74690	-2.52	-1.33	-4.94	0.001883	0.01215	-1.40
247393_at	unknown protein	---	AT5G63130	5.57	2.48	4.92	0.001921	0.012315	-1.42
258470_at	Expressed protein ; supported by fu	---	AT3G06035	-7.04	-2.82	-4.92	0.001923	0.012315	-1.42
249610_at	putative protein	---	AT5G37360	-2.24	-1.17	-4.91	0.001932	0.012347	-1.43
257337_at	NADH dehydrogenase subunit 5 (na	---	---	2.35	1.23	4.90	0.001957	0.012455	-1.44
248794_at	ethylene responsive element bindin	ERF2	AT5G47220	6.64	2.73	4.89	0.001984	0.012582	-1.46
266097_at	unknown protein ; supported by cD	SOUL-1	AT2G37970	2.50	1.32	4.89	0.001986	0.012587	-1.46
264757_at	receptor protein kinase (IRK1), puta	---	AT1G61360	5.35	2.42	4.87	0.002033	0.012827	-1.49
258074_at	unknown protein	---	AT3G25890	2.88	1.53	4.86	0.002041	0.012843	-1.49
266172_at	putative aquaporin (water channel p	PIP2E	AT2G39010	-2.33	-1.22	-4.86	0.002048	0.012875	-1.49
262151_at	hypothetical protein predicted by ge	---	AT1G52510	-2.16	-1.11	-4.85	0.002066	0.012951	-1.50
266225_at	putative membrane channel protein	ATOEP16-	AT2G28900	-2.59	-1.37	-4.83	0.00212	0.013189	-1.53

258633_at	putative serine carboxypeptidase II	SCPL27	AT3G07990	-2.22	-1.15	-4.83	0.002122	0.013197	-1.53
264223_s_a	receptor kinase, putative similar to	---	AT1G67520	2.65	1.41	4.83	0.002131	0.013216	-1.54
258679_at	putative 2,3-bisphosphoglycerate-i	---	AT3G08590	2.13	1.09	4.82	0.00215	0.013311	-1.55
260179_at	unknown protein similar to hypothet	HWI1	AT1G70690	2.35	1.23	4.81	0.002161	0.013348	-1.55
267436_at	putative receptor-like protein kinase	FRK1	AT2G19190	3.61	1.85	4.81	0.002161	0.013348	-1.55
252214_at	putative protein EREBP-3 homolog	CEJ1	AT3G50260	2.59	1.37	4.81	0.002162	0.013348	-1.55
247314_at	3(2),5-bisphosphate nucleotidase (	SAL2	AT5G64000	2.83	1.50	4.81	0.002168	0.013376	-1.56
252366_at	Expressed protein ; supported by fu	---	AT3G48420	-2.46	-1.30	-4.81	0.002176	0.013403	-1.56
260028_at	unknown protein ; supported by cDI	---	AT1G29980	-4.66	-2.22	-4.81	0.002177	0.013405	-1.56
262518_at	putative glutathione transferase On	ATGSTU24	AT1G17170	20.64	4.37	4.80	0.002194	0.013483	-1.57
260882_at	DNA binding protein, putative simil	WRKY65	AT1G29280	2.11	1.08	4.78	0.002234	0.013662	-1.59
259730_at	unknown protein similar to hypothet	---	AT1G77660	2.80	1.49	4.78	0.002253	0.013729	-1.60
249996_at	glutaredoxin -like protein glutaredo	---	AT5G18600	-2.19	-1.13	-4.77	0.002268	0.013801	-1.60
258055_at	putative 2Fe-2S iron-sulfur cluster p	NDF4	AT3G16250	-2.99	-1.58	-4.77	0.002273	0.01382	-1.61
247912_at	putative protein contains similarity t	---	AT5G57480	2.26	1.17	4.77	0.002275	0.013824	-1.61
244934_at	NADH dehydrogenase ND6	---	---	-2.21	-1.15	-4.77	0.00228	0.013841	-1.61
267567_at	putative cytochrome P450	CYP71A13	AT2G30770	27.98	4.81	4.76	0.002282	0.013849	-1.61
262369_at	hypothetical protein predicted by ge	---	AT1G73010	5.12	2.36	4.76	0.002298	0.01392	-1.62
245760_s_a	receptor serine/threonine kinase PI	---	AT1G66910	2.45	1.29	4.76	0.002303	0.013937	-1.62
246280_at	hypothetical protein	---	AT4G36660	-2.26	-1.18	-4.75	0.002318	0.013991	-1.63
255856_at	unknown protein identical to unknov	---	AT1G66940	-4.84	-2.27	-4.74	0.002347	0.014115	-1.64
266831_at	putative squalene epoxidase	SQE2	AT2G22830	-3.09	-1.63	-4.73	0.002373	0.014221	-1.65
254343_at	PRH26 protein ;supported by full-le	אפר-03	AT4G21990	3.26	1.70	4.73	0.002381	0.014248	-1.66
249755_at	unknown protein ;supported by full-	---	AT5G24580	-3.15	-1.65	-4.72	0.002405	0.014342	-1.67
258183_at	unknown protein ;supported by full-	---	AT3G21550	-3.50	-1.81	-4.72	0.002412	0.014368	-1.67
264301_at	hypothetical protein contains similar	---	AT1G78780	2.60	1.38	4.71	0.002438	0.014469	-1.68
254232_at	tyrosine transaminase like protein t	CORI3	AT4G23600	-3.73	-1.90	-4.70	0.002462	0.014584	-1.69
255437_at	putative oxidoreductase similar to F	AOP2	AT4G03060	-3.05	-1.61	-4.69	0.002485	0.014686	-1.70
250090_at	glutamate decarboxylase 1 (GAD 1	GAD	AT5G17330	5.60	2.49	4.69	0.002486	0.014686	-1.70
259180_at	unknown protein	---	AT3G01680	-2.88	-1.53	-4.68	0.002511	0.014768	-1.71
262488_at	unknown protein EST gb|AA586241	---	AT1G21830	-2.09	-1.06	-4.67	0.002529	0.014842	-1.72
261421_at	hypothetical protein similar to unkno	IQD30	AT1G18840	-2.44	-1.29	-4.67	0.002551	0.014925	-1.73
246863_at	nodulin - like protein early nodulin I	---	AT5G25940	2.21	1.14	4.66	0.002558	0.014949	-1.73
259166_at	unknown protein ; supported by cD	---	AT3G01670	-3.16	-1.66	-4.66	0.002568	0.014991	-1.74
246487_at	putative protein with poly glutamic	---	AT5G16030	-2.06	-1.04	-4.66	0.002577	0.015035	-1.74

247881_at	putative protein similar to unknown	---	AT5G57700	-2.47	-1.30	-4.65	0.002585	0.015066	-1.75
251058_at	putative protein	---	AT5G01790	-3.69	-1.88	-4.65	0.00259	0.015085	-1.75
260804_at	hypothetical protein predicted by ge	---	AT1G78410	15.99	4.00	4.64	0.002614	0.015184	-1.76
256366_at	protein kinase, putative contains Pf	---	AT1G66880	2.24	1.16	4.64	0.002634	0.015278	-1.77
262414_at	acyl CoA synthetase, putative simil	LACS2	AT1G49430	-4.26	-2.09	-4.64	0.00264	0.015304	-1.77
249515_at	tryptophan synthase beta chain	---	AT5G38530	3.55	1.83	4.62	0.002688	0.015487	-1.79
260243_at	hypothetical protein similar to putati	---	AT1G63720	2.54	1.34	4.62	0.002699	0.015527	-1.79
253343_at	putative protein	---	AT4G33540	2.59	1.37	4.61	0.002715	0.015562	-1.80
258897_at	unknown protein	---	AT3G05730	-12.36	-3.63	-4.61	0.002733	0.015638	-1.81
260867_at	hypothetical protein predicted by ge	TED6	AT1G43790	-3.40	-1.77	-4.60	0.002751	0.015702	-1.81
261814_at	unknown protein	---	AT1G08310	5.66	2.50	4.60	0.002762	0.015756	-1.82
258588_s_a	glyceraldehyde-3-phosphate dehyd	GAPC1	AT3G04120	2.17	1.12	4.59	0.002789	0.015899	-1.83
247768_at	I-box binding factor - like protein I-b	---	AT5G58900	-3.03	-1.60	-4.58	0.002823	0.016048	-1.84
248327_at	putative protein similar to unknown	---	AT5G52750	8.40	3.07	4.57	0.002831	0.016077	-1.85
267178_at	unknown protein ; supported by ful	---	AT2G37750	4.29	2.10	4.57	0.002859	0.016178	-1.86
257325_at	hypothetical protein	---	AT2G07798	2.40	1.26	4.56	0.002866	0.016207	-1.86
262288_at	unknown protein identical to residue	CRR23	AT1G70760	-2.56	-1.35	-4.56	0.002869	0.016212	-1.86
258877_at	putative expansin precursor similar	ATEXPA13	AT3G03220	-2.31	-1.21	-4.56	0.002888	0.016264	-1.87
258929_at	unknown protein Pfam HMM hit: FK	---	AT3G10060	-2.64	-1.40	-4.55	0.002904	0.016325	-1.87
254331_s_a	cytochrome P450 - like protein flav	CYP706A2	AT4G22690	2.82	1.50	4.55	0.002905	0.016325	-1.87
264402_at	HSP100/ClpB, putative similar to H	CLPB4	AT2G25140	2.05	1.03	4.55	0.002909	0.016325	-1.87
251304_at	putative protein several O-methyltra	---	AT3G61990	3.44	1.78	4.55	0.002917	0.016349	-1.88
255817_at	unknown protein ;supported by full-	PDLP3	AT2G33330	-2.11	-1.08	-4.54	0.002934	0.01642	-1.88
259507_at	unknown protein	---	AT1G43910	2.75	1.46	4.54	0.00294	0.016451	-1.89
249983_at	putative protein S-receptor kinase	---	AT5G18470	10.93	3.45	4.54	0.002943	0.016451	-1.89
251272_at	homeobox-leucine zipper protein AT	ATHB-12	AT3G61890	2.82	1.50	4.53	0.002972	0.01656	-1.90
262634_at	unknown protein ; supported by cDI	---	AT1G06690	-2.53	-1.34	-4.53	0.002978	0.016568	-1.90
252950_at	putative protein phospholipase C (I	---	AT4G38690	-2.26	-1.18	-4.53	0.002979	0.016568	-1.90
266536_at	hypothetical protein predicted by ge	---	AT2G16900	2.93	1.55	4.53	0.00298	0.016568	-1.90
248448_at	putative protein contains similarity t	---	AT5G51190	5.35	2.42	4.53	0.002981	0.016568	-1.90
247213_at	unknown protein ; supported by ful	PROPEP1	AT5G64900	3.43	1.78	4.53	0.002989	0.016591	-1.90
245854_at	adenosine nucleotide translocator ;	AAC2	AT5G13490	6.12	2.61	4.50	0.003102	0.016985	-1.94
256186_at	4-coumarate:CoA ligase 1 identical	4CL1	AT1G51680	2.49	1.31	4.49	0.003107	0.017006	-1.95
250132_at	putative protein similar to unknown	KAN	AT5G16560	-2.03	-1.02	-4.49	0.003115	0.017038	-1.95
267610_at	K+ transporter, AKT1 identical to G	AKT1	AT2G26650	2.59	1.37	4.49	0.003135	0.017119	-1.96

246988_at	putative protein strong similarity to u	---	AT5G67340	2.45	1.29	4.48	0.003172	0.017258	-1.97
266017_at	unknown protein ; supported by cD	---	AT2G18690	2.74	1.46	4.47	0.003182	0.017286	-1.97
261913_at	flavin-containing monooxygenase F	FMO GS-O	AT1G65860	-2.30	-1.20	-4.47	0.00319	0.017307	-1.97
262871_at	hypothetical protein predicted by ge	---	AT1G65010	-2.98	-1.57	-4.46	0.00322	0.017393	-1.99
245793_at	unknown protein ;supported by full-	---	AT1G32220	-2.11	-1.08	-4.46	0.00323	0.017414	-1.99
247729_at	1-aminocyclopropane-1-carboxylat	---	AT5G59530	2.02	1.01	4.46	0.003245	0.017439	-1.99
250680_at	putative protein similar to unknown	---	AT5G06570	-2.21	-1.14	-4.46	0.003245	0.017439	-1.99
264777_at	unknown protein similar to L-allo-th	THA1	AT1G08630	4.26	2.09	4.45	0.003266	0.017519	-2.00
247487_at	putative protein predicted protein, A	---	AT5G62150	4.76	2.25	4.44	0.003295	0.017644	-2.01
251336_at	putative protein hypothetical protei	BAP1	AT3G61190	2.17	1.12	4.44	0.003305	0.017672	-2.01
246370_at	hypothetical protein predicted by ge	---	AT1G51920	4.50	2.17	4.44	0.003323	0.017728	-2.02
264524_at	tat-binding protein, putative Highly	ATBCAT-2	AT1G10070	4.45	2.16	4.44	0.003326	0.01774	-2.02
260754_at	unknown protein	---	AT1G49000	2.32	1.22	4.43	0.003343	0.017801	-2.03
251374_at	homeobox-leucine zipper protein HA	HAT3	AT3G60390	-2.09	-1.06	-4.43	0.003358	0.017865	-2.03
247908_at	GS1-like protein ;supported by full-l	GS1	AT5G57440	-2.22	-1.15	-4.43	0.003365	0.017889	-2.03
260239_at	putative receptor protein kinase sin	---	AT1G74360	5.16	2.37	4.42	0.003387	0.017977	-2.04
255842_at	putative serine carboxypeptidase II	scpl46	AT2G33530	-2.38	-1.25	-4.42	0.003394	0.017992	-2.04
248676_at	putative protein similar to unknown	ATSDI1	AT5G48850	-3.57	-1.84	-4.42	0.003394	0.017992	-2.04
260368_at	AtHVA22c identical to AtHVA22c G	ATHVA22C	AT1G69700	-3.07	-1.62	-4.42	0.003409	0.018051	-2.05
252133_at	hypothetical protein hypothetical pr	---	AT3G50900	3.13	1.64	4.40	0.003455	0.018224	-2.06
250892_at	putative protein beta-(1-3)-glucosyl	ATCSLA09	AT5G03760	-10.98	-3.46	-4.40	0.003458	0.018235	-2.06
246251_at	cold acclimation protein homolog	---	AT4G37220	-2.56	-1.35	-4.39	0.003493	0.01838	-2.07
255546_at	putative CHP-rich zinc finger protein	---	AT4G01910	2.31	1.21	4.39	0.003515	0.018449	-2.08
254737_at	fatty acid elongase - like protein (ce	---	AT4G13840	-2.61	-1.39	-4.39	0.003525	0.018487	-2.08
257923_at	hypothetical protein predicted by ge	---	AT3G23160	2.53	1.34	4.39	0.003531	0.01851	-2.09
248404_at	trehalose-6-phosphate phosphatase	ATTPPA	AT5G51460	-2.12	-1.08	-4.38	0.00355	0.018573	-2.09
260978_at	17.6 kDa heat shock protein (AA 1-	---	AT1G53540	2.29	1.20	4.37	0.003576	0.018667	-2.10
261480_at	phytochrome kinase substrate 1, p	PKS2	AT1G14280	-3.65	-1.87	-4.37	0.003578	0.018671	-2.10
264511_at	putative galactinol synthase similar	AtGolS3	AT1G09350	-3.15	-1.66	-4.36	0.003621	0.018837	-2.11
259979_at	unknown protein ;supported by full-	---	AT1G76600	2.19	1.13	4.36	0.003631	0.018866	-2.12
255065_s_a	putative arginase similar to arginas	---	AT4G08870	-3.39	-1.76	-4.36	0.003659	0.018982	-2.12
264147_at	receptor-like protein glossy1 (gl1),	CER1	AT1G02205	-13.77	-3.78	-4.35	0.003685	0.019084	-2.13
259244_at	unknown protein similar to zinc fing	COL9	AT3G07650	2.17	1.12	4.34	0.003705	0.019147	-2.14
250267_at	putative protein predicted proteins	---	AT5G12930	4.15	2.05	4.34	0.003722	0.019211	-2.14
252280_at	SF16 -like protein SF16 protein, po	iqd21	AT3G49260	-2.45	-1.29	-4.34	0.003731	0.019242	-2.15

248932_at	peptide transporter	PTR3	AT5G46050	3.62	1.86	4.34	0.003739	0.019268	-2.15
263924_at	enolase (2-phospho-D-glycerate hy	LOS2	AT2G36530	2.16	1.11	4.34	0.003746	0.019294	-2.15
249783_at	calcium sensor homolog (gb|AAC26	SOS3	AT5G24270	2.07	1.05	4.32	0.003802	0.019539	-2.17
259980_at	unknown protein ;supported by full-	---	AT1G76520	2.02	1.02	4.32	0.003808	0.019555	-2.17
265572_at	putative carbonic anhydrase	ATACA2	AT2G28210	8.70	3.12	4.32	0.003828	0.019623	-2.17
266775_at	putative SCARECROW gene regula	---	AT2G29060	2.04	1.03	4.31	0.003866	0.019778	-2.18
245336_at	Expressed protein ; supported by fu	---	AT4G16515	-4.36	-2.12	-4.31	0.003869	0.019787	-2.18
259033_at	putative pectinacetylesterase simila	---	AT3G09405	6.75	2.75	4.30	0.003893	0.01988	-2.19
255127_at	nodulin-like protein nodulin gene M	---	AT4G08300	3.43	1.78	4.28	0.003979	0.020196	-2.22
266132_at	hypothetical protein predicted by ge	SPX3	AT2G45130	2.14	1.10	4.27	0.004042	0.020446	-2.23
257938_at	cell elongation protein, Dwarf1 ider	DWF1	AT3G19820	-4.38	-2.13	-4.27	0.004047	0.020469	-2.23
248405_at	putative protein pectinesterase-like;	SKS2	AT5G51480	-5.07	-2.34	-4.27	0.004055	0.02049	-2.24
258362_at	unknown protein	---	AT3G14280	3.77	1.91	4.23	0.00423	0.021115	-2.28
254571_at	hypothetical protein	---	AT4G19370	3.79	1.92	4.22	0.004291	0.021291	-2.30
263565_at	unknown protein	FUT4	AT2G15390	3.10	1.63	4.22	0.004323	0.021388	-2.30
250443_at	Pto kinase interactor - like protein F	RBK1	AT5G10520	5.02	2.33	4.22	0.004326	0.021389	-2.31
247983_at	pyrophosphate-dependent phospho	PFK7	AT5G56630	2.45	1.29	4.21	0.004353	0.021488	-2.31
251183_at	putative protein putative protein At2	---	AT3G62630	-2.84	-1.51	-4.18	0.004516	0.022109	-2.35
263495_at	cold-regulated protein cor15b precu	COR15B	AT2G42530	-3.46	-1.79	-4.18	0.004537	0.022191	-2.36
245334_at	Expressed protein ; supported by fu	RALFL33	AT4G15800	-2.01	-1.01	-4.17	0.004548	0.022204	-2.36
253088_at	ferulate-5-hydroxylase (FAH1) ; su	FAH1	AT4G36220	2.10	1.07	4.17	0.004577	0.022294	-2.37
266516_at	putative glutaredoxin ;supported by	---	AT2G47880	-2.27	-1.18	-4.17	0.004583	0.02231	-2.37
266979_at	unknown protein ;supported by full-	PPL2	AT2G39470	-3.71	-1.89	-4.16	0.004599	0.022363	-2.37
249494_at	acyltransferase - like protein Antho	---	AT5G39050	2.23	1.16	4.16	0.004606	0.022382	-2.37
266150_s_a	hypothetical protein	---	AT2G12290	2.64	1.40	4.16	0.00463	0.022454	-2.38
258399_at	early auxin-induced protein, IAA19	IAA19	AT3G15540	-3.29	-1.72	-4.15	0.004653	0.022534	-2.38
264774_at	unknown protein	---	AT1G22890	2.32	1.22	4.15	0.004702	0.022729	-2.40
247444_at	putative protein strong similarity to u	HIPL2	AT5G62630	2.30	1.20	4.13	0.004792	0.023026	-2.42
259743_at	hypothetical protein predicted by ge	---	AT1G71140	3.65	1.87	4.12	0.004841	0.023212	-2.43
247816_at	similar to unknown protein (pir||S75	---	AT5G58260	-2.11	-1.08	-4.12	0.004851	0.023229	-2.43
257021_at	branched-chain amino acid aminot	BCAT4	AT3G19710	-2.07	-1.05	-4.12	0.004865	0.023271	-2.43
260477_at	Ser/Thr protein kinase isolog	---	AT1G11050	3.84	1.94	4.11	0.004915	0.023457	-2.44
261279_at	class I chitinase, putative similar to	POM1	AT1G05850	-4.76	-2.25	-4.11	0.004945	0.023534	-2.45
257058_at	Expressed protein ; supported by cl	COX17	AT3G15352	3.47	1.79	4.09	0.005061	0.023874	-2.48
253274_at	Phosphoglycerate dehydrogenase	EDA9	AT4G34200	6.48	2.70	4.08	0.005072	0.023907	-2.48

264501_at	putative lipase Similar to nodulins a	---	AT1G09390	-2.53	-1.34	-4.07	0.005137	0.02412	-2.49
255502_at	contains similarity to a protein kina	---	AT4G02410	3.68	1.88	4.07	0.005151	0.024153	-2.49
265656_at	putative nonspecific lipid-transfer pr	---	AT2G13820	-2.18	-1.13	-4.06	0.005222	0.024413	-2.51
260857_at	unknown protein contains Pfam pro	LYM1	AT1G21880	-2.15	-1.10	-4.06	0.005233	0.024457	-2.51
263182_at	Expressed protein ; supported by fu	---	AT1G05575	7.99	3.00	4.05	0.005299	0.024679	-2.53
253414_at	putative protein	EDA39	AT4G33050	4.91	2.30	4.05	0.005308	0.024707	-2.53
257547_at	hypothetical protein predicted by ge	---	AT3G13000	-2.40	-1.27	-4.04	0.005335	0.024796	-2.53
249109_at	auxin-induced protein AUX2-11 (sp	ATAUX2-1	AT5G43700	-3.09	-1.63	-4.04	0.00536	0.024877	-2.54
255111_at	peroxidase C2 precursor like prote	---	AT4G08780	10.86	3.44	4.03	0.005389	0.024964	-2.54
262942_at	hypothetical protein similar to unkno	ALIS5	AT1G79450	2.28	1.19	4.03	0.005416	0.025029	-2.55
265853_at	putative RING zinc finger protein	---	AT2G42360	2.91	1.54	4.03	0.005423	0.025057	-2.55
267028_at	putative WRKY-type DNA binding p	WRKY33	AT2G38470	5.41	2.43	4.03	0.005428	0.025075	-2.55
246516_at	putative protein auxin-independent	---	AT5G15740	-3.02	-1.59	-4.03	0.005449	0.025151	-2.56
256933_at	unknown protein contains Pfam pro	---	AT3G22600	10.82	3.44	4.03	0.005453	0.025156	-2.56
256015_at	PSI type II chlorophyll a/b-binding p	LHCA6	AT1G19150	-3.76	-1.91	-4.02	0.005506	0.025314	-2.57
249986_at	putative protein predicted proteins,	---	AT5G18460	-2.10	-1.07	-4.01	0.005566	0.025484	-2.58
245744_at	unknown protein	---	AT1G51110	-2.32	-1.22	-4.01	0.005591	0.025549	-2.58
262882_at	cytochrome p450, putative similar t	CYP89A2	AT1G64900	2.52	1.33	4.00	0.0056	0.025578	-2.59
261248_at	calreticulin, putative similar to GB:A	---	AT1G20030	-2.29	-1.19	-4.00	0.005616	0.025645	-2.59
254408_at	serine/threonine kinase - like prote	B120	AT4G21390	6.51	2.70	4.00	0.00565	0.025737	-2.59
246858_at	receptor-like protein kinase - like re	---	AT5G25930	2.89	1.53	3.99	0.005707	0.025936	-2.61
264433_at	beta-glucosidase, putative similar t	BGLU45	AT1G61810	4.61	2.21	3.99	0.005721	0.025969	-2.61
248895_at	receptor protein kinase	FLS2	AT5G46330	-2.06	-1.04	-3.97	0.00582	0.026285	-2.63
258282_at	unknown protein	---	AT3G26910	2.31	1.21	3.93	0.006125	0.027265	-2.68
255011_at	cytochrome c several plant cytochr	CYTC-2	AT4G10040	2.79	1.48	3.93	0.006176	0.027408	-2.69
266270_at	putative glutathione S-transferase	ATGSTU3	AT2G29470	7.92	2.98	3.91	0.006255	0.027647	-2.70
261247_at	hypothetical protein predicted by ge	---	AT1G20070	-3.39	-1.76	-3.90	0.00634	0.027922	-2.72
261451_at	unknown protein	---	AT1G21060	-2.55	-1.35	-3.90	0.006353	0.02795	-2.72
249485_at	receptor protein kinase - like protei	---	AT5G39020	2.43	1.28	3.90	0.006408	0.028125	-2.73
255923_at	hypothetical protein contains Pfam	---	AT1G22180	2.33	1.22	3.89	0.006423	0.028153	-2.73
252908_at	putative protein	---	AT4G39670	5.54	2.47	3.89	0.006452	0.028232	-2.74
258002_at	AIG2-like protein similar to AIG2 pro	AIG2	AT3G28930	4.71	2.24	3.89	0.006464	0.028269	-2.74
252178_at	putative protein various predicted p	---	AT3G50750	-2.11	-1.07	-3.87	0.006585	0.028658	-2.76
260706_at	oxidase, putative similar to oxidase	AOX1D	AT1G32350	10.36	3.37	3.85	0.00676	0.029176	-2.79
248970_at	urea active transporter-like protein	---	AT5G45380	2.10	1.07	3.85	0.006797	0.029298	-2.79

247754_at	putative protein	---	AT5G59080	-2.14	-1.10	-3.85	0.006799	0.0293	-2.79
262649_at	unknown protein contains similarity	---	AT1G14040	2.29	1.20	3.84	0.006875	0.029555	-2.81
253203_at	arginine decarboxylase SPE2 ; sup	ADC2	AT4G34710	-2.38	-1.25	-3.83	0.00694	0.029761	-2.82
246919_at	putative protein hypothetical protei	---	AT5G25460	-9.93	-3.31	-3.82	0.007046	0.030071	-2.83
248382_at	peroxidase	---	AT5G51890	-3.02	-1.60	-3.80	0.007217	0.030589	-2.86
256300_at	unknown protein similar to N-term h	NAP	AT1G69490	3.02	1.60	3.80	0.007225	0.030611	-2.86
266581_at	putative desiccation related protein	---	AT2G46140	4.11	2.04	3.79	0.00728	0.03076	-2.87
256583_at	hypothetical protein	---	AT3G28850	2.04	1.03	3.79	0.007282	0.03076	-2.87
262801_at	unknown protein ;supported by full-	---	AT1G21010	2.16	1.11	3.77	0.007496	0.031359	-2.90
266290_at	putative glutathione S-transferase ;	ATGSTU1	AT2G29490	5.18	2.37	3.77	0.007518	0.031406	-2.90
255879_at	receptor-like kinase, putative simila	---	AT1G67000	2.00	1.00	3.77	0.007522	0.031418	-2.90
256185_at	dof zinc finger protein identical to do	ADOF1	AT1G51700	2.47	1.30	3.77	0.007532	0.031435	-2.90
255340_at	putative receptor-like protein kinase	---	AT4G04490	3.02	1.60	3.77	0.007537	0.031435	-2.91
266752_at	putative ABC transporter related to	ABCB4	AT2G47000	5.41	2.44	3.75	0.007668	0.031854	-2.92
246275_at	putative protein ; supported by full-l	BEE2	AT4G36540	-4.63	-2.21	-3.75	0.007709	0.031965	-2.93
257008_at	myrosinase-associated protein, put	ESM1	AT3G14210	-4.02	-2.01	-3.75	0.007745	0.032091	-2.93
251395_at	unknown protein ;supported by full-	FLA8	AT2G45470	-7.96	-2.99	-3.73	0.00786	0.032415	-2.95
256589_at	cytochrome P450, putative contain	CYP81D1	AT3G28740	4.57	2.19	3.73	0.007869	0.032439	-2.95
252939_at	NAD(P)H oxidoreductase, isoflavor	---	AT4G39230	2.82	1.49	3.73	0.007907	0.032549	-2.96
258140_at	Expressed protein ; supported by cl	ALDH2C4	AT3G24503	2.19	1.13	3.73	0.007927	0.032586	-2.96
266834_s_a	putative protein phosphatase 2C	---	AT2G05050 /	4.41	2.14	3.72	0.00802	0.032844	-2.97
266992_at	similar to Mlo proteins from H. vulga	MLO12	AT2G39200	3.47	1.79	3.71	0.008083	0.032983	-2.98
258641_at	unknown protein ;supported by full-	---	AT3G08030	-2.80	-1.49	-3.71	0.008085	0.032986	-2.98
262926_s_a	receptor kinase, putative similar to	ARK1	AT1G65790 /	2.26	1.18	3.71	0.008145	0.033139	-2.99
262366_at	disease resistance protein, putative	---	AT1G72890	2.13	1.09	3.70	0.00825	0.033432	-3.00
255999_at	DNA-binding protein, putative simil	WRKY71	AT1G29860	2.08	1.06	3.69	0.008297	0.033519	-3.01
266294_at	putative small heat shock protein ;s	---	AT2G29500	3.91	1.97	3.69	0.008315	0.033581	-3.01
252338_at	putative progesterone-binding prote	MAPR3	AT3G48890	2.65	1.41	3.69	0.008339	0.033621	-3.01
245929_at	alcohol dehydrogenase - like prote	---	AT5G24760	2.46	1.30	3.67	0.008497	0.034049	-3.03
248719_at	respiratory burst oxidase protein ; s	RBOHD	AT5G47910	2.75	1.46	3.67	0.00852	0.034093	-3.04
259439_at	1-aminocyclopropane-1-carboxylate	ACS2	AT1G01480	4.76	2.25	3.66	0.0086	0.034301	-3.05
261363_at	pectinesterase, putative similar to C	SKS6	AT1G41830	-6.13	-2.62	-3.66	0.008702	0.034613	-3.06
255753_at	myb factor, putative similar to myb	MYB51	AT1G18570	5.06	2.34	3.65	0.008715	0.034655	-3.06
261653_at	subtilisin-like serine protease, puta	SBTI1.1	AT1G01900	-2.07	-1.05	-3.65	0.008737	0.03473	-3.06
266311_at	hypothetical protein predicted by ge	---	AT2G27130	-2.49	-1.32	-3.65	0.008778	0.034817	-3.07

261663_at	hypothetical protein similar to hypot	EPR1	AT1G18330	-2.42	-1.28	-3.64	0.008819	0.034893	-3.07
253373_at	lysine-ketoglutarate reductase/sacc	---	AT4G33150	2.32	1.21	3.64	0.008853	0.034979	-3.08
246821_at	calmodulin-binding - like protein ca	CBP60G	AT5G26920	3.11	1.64	3.64	0.008904	0.035128	-3.08
AFFX-r2-At-	Arabidopsis thaliana /REF=M6411(	---	---	2.09	1.06	3.63	0.008963	0.035292	-3.09
257466_at	hypothetical protein predicted by ge	---	AT1G62840	2.11	1.08	3.63	0.008985	0.035354	-3.09
261375_at	transcription factor, putative similar	SPL4	AT1G53160	-3.37	-1.75	-3.62	0.009124	0.03568	-3.11
264773_at	putative disease resistance respons	---	AT1G22900	2.26	1.17	3.61	0.00917	0.035806	-3.12
246949_at	cytochrome P450-like protein CYT(	CYP71B13	AT5G25140	-2.45	-1.29	-3.60	0.009335	0.036318	-3.14
266464_at	glutathione-conjugate transporter A	ATMRP4	AT2G47800	4.29	2.10	3.60	0.009359	0.036383	-3.14
245251_at	calcineurin B-like protein 1 ; suppor	CBL1	AT4G17615	2.33	1.22	3.59	0.009429	0.036577	-3.15
245318_at	Expressed protein ; supported by fu	---	AT4G16980	-5.66	-2.50	-3.58	0.009559	0.03694	-3.16
260405_at	putative glutathione transferase sim	ATGSTU11	AT1G69930	6.10	2.61	3.57	0.009687	0.03732	-3.18
250798_at	peroxidase	---	AT5G05340	5.57	2.48	3.57	0.009743	0.037484	-3.18
259757_at	putative thioredoxin similar to protei	ATPDIL1-2	AT1G77510	2.55	1.35	3.56	0.009793	0.037598	-3.19
254926_at	ACC synthase (AtACS-6) ; support	ACS6	AT4G11280	4.45	2.15	3.55	0.009929	0.037983	-3.20
249872_at	unknown protein	---	AT5G23130	-2.35	-1.23	-3.54	0.010065	0.038315	-3.22
260693_at	peptide transporter PTR2-B, putati	NRT1.5	AT1G32450	2.09	1.06	3.54	0.010077	0.038348	-3.22
253627_at	low temperature and salt responsiv	---	AT4G30650	-5.16	-2.37	-3.53	0.01028	0.038901	-3.24
257925_at	unknown protein ;supported by full-	---	AT3G23170	3.81	1.93	3.52	0.010408	0.039207	-3.25
252604_at	high-affinity nitrate transporter - like	NRT2.6	AT3G45060	-2.50	-1.32	-3.50	0.010579	0.039682	-3.27
263231_at	putative indole-3-acetate beta-gluco	---	AT1G05680	11.60	3.54	3.48	0.010885	0.040491	-3.30
254229_at	hypothetical protein ; supported by	---	AT4G23610	2.45	1.29	3.48	0.010934	0.040634	-3.30
256442_at	hypothetical protein predicted by ge	---	AT3G10930	4.09	2.03	3.48	0.010961	0.040711	-3.31
261648_at	salt-tolerance zinc finger protein ide	STZ	AT1G27730	3.19	1.68	3.48	0.01098	0.040744	-3.31
259479_at	Expressed protein ; supported by fu	---	AT1G19020	3.25	1.70	3.47	0.011071	0.040967	-3.32
245038_at	similar to latex allergen from Hevea	PLA2A	AT2G26560	2.52	1.34	3.47	0.011116	0.041109	-3.32
250916_at	monodehydroascorbate reductase	ATMDAR2	AT5G03630	2.37	1.25	3.45	0.011433	0.041985	-3.35
260745_at	2,4-D inducible glutathione S-trans	ATGSTU20	AT1G78370	-2.12	-1.08	-3.45	0.011434	0.041985	-3.35
246912_at	putative protein various predicted p	---	AT5G25820	2.03	1.02	3.44	0.011521	0.042238	-3.36
260568_at	endochitinase isolog	---	AT2G43570	8.36	3.06	3.43	0.011645	0.042518	-3.37
264756_at	receptor protein kinase (IRK1), put	---	AT1G61370	2.76	1.47	3.43	0.011704	0.042655	-3.38
250415_at	putative protein GLUR3 ligand-gate	GLR2.5	AT5G11210	2.39	1.26	3.42	0.011827	0.042965	-3.39
265028_at	hypothetical protein predicted by ge	---	AT1G24530	-2.49	-1.32	-3.42	0.011836	0.042975	-3.39
267559_at	putative cytochrome P450	CYP76C2	AT2G45570	3.34	1.74	3.41	0.011931	0.043251	-3.40
259213_at	putative receptor ser/thr protein kina	---	AT3G09010	2.69	1.43	3.41	0.011979	0.043371	-3.40

266865_at	omega-3 fatty acid desaturase iden	FAD3	AT2G29980	-9.98	-3.32	-3.41	0.011985	0.04338	-3.40
245250_at	ethylene responsive element bindin	ATERF6	AT4G17490	4.66	2.22	3.41	0.011985	0.04338	-3.40
266267_at	putative glutathione S-transferase	ATGSTU4	AT2G29460	3.81	1.93	3.40	0.012107	0.043676	-3.41
261431_at	Myb-related transcription factor mix	AtMYB47	AT1G18710	2.48	1.31	3.40	0.012119	0.043691	-3.41
257137_at	P-glycoprotein, putative similar to F	ABCB19	AT3G28860	-2.55	-1.35	-3.38	0.012399	0.044347	-3.44
251297_at	germin-like protein (GLP10)	GLP10	AT3G62020	-2.06	-1.04	-3.36	0.012746	0.045216	-3.47
258947_at	hypothetical protein similar to calmo	---	AT3G01830	4.24	2.08	3.36	0.012864	0.045485	-3.48
249928_at	CCR4-associated factor-like protein	---	AT5G22250	4.46	2.16	3.34	0.01324	0.046469	-3.51
256093_at	predicted protein ; supported by cD	---	AT1G20823	2.46	1.30	3.31	0.013726	0.04766	-3.55
245749_at	proline-rich protein, putative similar	---	AT1G51090	-2.25	-1.17	-3.31	0.013739	0.0477	-3.55
254954_at	hypothetical protein ;supported by	---	AT4G10910	-2.09	-1.06	-3.30	0.013797	0.047828	-3.55
248896_at	putative protein contains similarity t	WRKY8	AT5G46350	2.56	1.36	3.30	0.013925	0.048161	-3.56
255959_at	phosphatidylinositol-4-phosphate 5	ATPIP5K1	AT1G21980	-2.55	-1.35	-3.29	0.013983	0.048267	-3.57
264514_at	putative cinnamyl alcohol dehydrog	---	AT1G09500	3.81	1.93	3.29	0.014161	0.048682	-3.58
258787_at	hypothetical protein predicted by ge	PUB24	AT3G11840	3.39	1.76	3.28	0.014213	0.048829	-3.58
251975_at	CDC48 - like protein transitional en	---	AT3G53230	2.29	1.20	3.28	0.014234	0.048856	-3.59
245711_at	putative c2h2 zinc finger transcriptio	ZAT6	AT5G04340	2.55	1.35	3.28	0.014243	0.048861	-3.59
262381_at	virus resistance protein, putative si	---	AT1G72900	2.92	1.54	3.27	0.014464	0.049342	-3.60
264415_at	AP2 domain containing protein, pu	RAP2.6	AT1G43160	-48.25	-5.59	-38.70	3.70E-09	2.81E-05	10.79
259942_at	unknown protein	ATWHY2	AT1G71260	4.28	2.10	23.71	9.78E-08	0.000167	8.56
251142_at	Expressed protein ; supported by fu	---	AT5G01015	-6.78	-2.76	-22.49	1.39E-07	0.000167	8.27
247030_at	putative protein similar to unknown	---	AT5G67210	-3.88	-1.96	-21.19	2.06E-07	0.000206	7.94
260235_at	putative SET protein, phospatase 2	NRP1	AT1G74560	3.58	1.84	19.02	4.23E-07	0.000301	7.31
262811_at	unknown protein ESTs gb|R65381 a	---	AT1G11700	4.83	2.27	18.50	5.08E-07	0.000301	7.14
261086_at	hypothetical protein predicted by ge	TRFL3	AT1G17460	3.29	1.72	17.03	8.78E-07	0.000345	6.63
263412_at	putative histone H2B ;supported by	---	AT2G28720	3.42	1.77	15.65	1.53E-06	0.000442	6.11
255604_at	hypothetical protein	---	AT4G01080	-7.00	-2.81	-15.62	1.55E-06	0.000442	6.09
249839_at	Expressed protein ; supported by cl	---	AT5G23405	3.94	1.98	15.46	1.66E-06	0.000457	6.03
245060_at	GDP-mannose pyrophosphorylase	CYT1	AT2G39770	-2.85	-1.51	-15.34	1.75E-06	0.000461	5.98
260496_at	putative ABC transporter	---	AT2G41700	2.70	1.43	14.86	2.15E-06	0.000485	5.78
253358_at	gamma-VPE (vacuolar processing e	GAMMA-VI	AT4G32940	3.12	1.64	14.57	2.45E-06	0.000523	5.65
250194_at	putative protein various predicted p	---	AT5G14550	3.13	1.65	14.53	2.49E-06	0.000523	5.63
246809_s_a	SAR DNA-binding protein - like SA	---	AT5G27120	2.44	1.29	14.29	2.77E-06	0.000556	5.53
249970_at	dermal glycoprotein precursor -like	---	AT5G19100	-8.12	-3.02	-14.27	2.80E-06	0.000556	5.51
264657_at	putative 26S protease regulatory su	RPT5B	AT1G09100	2.92	1.55	14.12	2.99E-06	0.000569	5.45

252123_at	flavanone 3-hydroxylase (FH3) ;su	F3H	AT3G51240	-2.80	-1.49	-13.81	3.46E-06	0.000602	5.30
249090_at	Expressed protein ; supported by cl	---	AT5G43745	-4.85	-2.28	-13.79	3.50E-06	0.000602	5.29
261772_at	hypothetical protein predicted by ge	---	AT1G76240	-8.52	-3.09	-13.60	3.83E-06	0.000608	5.20
255264_at	putative protein ethylene responsiv	---	AT4G05170	-3.32	-1.73	-13.59	3.84E-06	0.000608	5.20
251472_at	putative protein nodule inception pr	---	AT3G59580	2.75	1.46	13.54	3.93E-06	0.000608	5.18
250545_at	serine/threonine protein kinase ;su	ATPK3	AT5G08160	-2.62	-1.39	-13.48	4.05E-06	0.000616	5.15
250663_at	putative protein prenylated rab acc	PRA1.B6	AT5G07110	-2.77	-1.47	-13.35	4.34E-06	0.000622	5.08
262996_at	hypothetical protein predicted by ge	---	AT1G54440	2.84	1.50	13.29	4.46E-06	0.000622	5.05
263929_at	hypothetical protein predicted by ge	URH1	AT2G36310	-3.21	-1.68	-13.02	5.08E-06	0.000655	4.92
257608_at	chaperonin, putative similar to CHA	HSP60-3A	AT3G13860	2.84	1.51	12.85	5.54E-06	0.000679	4.83
257164_at	mismatch binding protein, putative	MSH1	AT3G24320	2.88	1.52	12.80	5.68E-06	0.00069	4.81
257193_at	Expressed protein ; supported by fu	---	AT3G13160	2.42	1.28	12.78	5.75E-06	0.000694	4.79
245367_at	Expressed protein ; supported by fu	NRPB9B	AT4G16265	2.89	1.53	12.74	5.85E-06	0.000698	4.78
249354_at	nuclear pore protein -like nuclear p	EMB3012	AT5G40480	4.68	2.23	12.73	5.89E-06	0.000698	4.77
249540_at	4-coumarate--CoA ligase -like prote	---	AT5G38120	-8.44	-3.08	-12.70	5.99E-06	0.0007	4.75
264516_at	unknown protein	---	AT1G10090	-2.58	-1.37	-12.67	6.07E-06	0.0007	4.74
260605_at	hypothetical protein predicted by ge	---	AT2G43780	2.62	1.39	12.67	6.08E-06	0.0007	4.74
248753_at	acyl carrier protein-like	mtACP3	AT5G47630	2.59	1.38	12.61	6.26E-06	0.000709	4.71
266264_at	Expressed protein ; supported by fu	---	AT2G27775	2.52	1.33	12.59	6.34E-06	0.000709	4.69
261071_at	unknown protein	---	AT1G07380	3.38	1.76	12.40	6.97E-06	0.000738	4.60
261951_at	hypothetical protein predicted by ge	---	AT1G64490	2.73	1.45	12.38	7.05E-06	0.000739	4.59
252355_at	putative protein various predicted p	---	AT3G48250	2.62	1.39	12.36	7.15E-06	0.000739	4.57
258453_at	RNA polymerase I, II and III 24.3 k	NRPB5	AT3G22320	2.30	1.20	12.32	7.29E-06	0.000742	4.55
260364_at	putative alliinase similar to aliinase	TAA1	AT1G70560	4.12	2.04	12.27	7.47E-06	0.000745	4.53
245141_at	putative flavonol reductase	BEN1	AT2G45400	-3.06	-1.61	-12.24	7.60E-06	0.000753	4.51
263533_at	putative Rieske iron-sulfur protein ;	TIC55	AT2G24820	2.11	1.08	12.19	7.80E-06	0.000763	4.48
246603_at	copper amine oxidase, putative sim	---	AT1G31690	-4.03	-2.01	-12.16	7.93E-06	0.00077	4.46
255825_at	Expressed protein ; supported by fu	---	AT2G40475	-4.51	-2.17	-12.14	8.02E-06	0.000776	4.45
246057_at	putative protein predicted proteins,	---	AT5G08400	2.42	1.27	12.04	8.47E-06	0.000802	4.40
260500_at	Expressed protein ; supported by cl	---	AT2G41705	2.28	1.19	12.04	8.48E-06	0.000802	4.40
247434_at	Expressed protein ; supported by fu	---	AT5G62575	2.33	1.22	12.03	8.51E-06	0.000802	4.39
264257_at	unknown protein	---	AT1G09230	3.09	1.63	12.01	8.61E-06	0.000802	4.38
262379_at	hypothetical protein predicted by ge	---	AT1G73020	-2.35	-1.24	-11.98	8.74E-06	0.000804	4.36
254773_at	putative protein Cyclic beta-1-3-glu	ATCSLA15	AT4G13410	-12.05	-3.59	-11.96	8.81E-06	0.000804	4.36
253558_at	kinase binding protein - like Skb1 p	SKB1	AT4G31120	2.93	1.55	11.92	9.03E-06	0.000814	4.33

250598_at	transcription factor-like protein ; su	ATMYB29	AT5G07690	-4.41	-2.14	-11.86	9.31E-06	0.000822	4.30
256661_at	hypothetical protein similar to putati	---	AT3G11964	2.43	1.28	11.85	9.34E-06	0.000822	4.30
250529_at	RNA helicase-like protein	---	AT5G08610	3.81	1.93	11.83	9.45E-06	0.000823	4.28
257239_at	unknown protein	---	AT3G24100	2.67	1.42	11.83	9.48E-06	0.000823	4.28
257765_at	hypothetical protein contains Pfam	---	AT3G23020	2.29	1.20	11.79	9.69E-06	0.000837	4.26
246260_at	amino acid permease, putative con	---	AT1G31820	3.64	1.86	11.77	9.81E-06	0.000838	4.25
260089_at	putative ATPase contains Pfam pro	---	AT1G73170	2.16	1.11	11.69	1.02E-05	0.000843	4.20
265895_at	unknown protein ;supported by full-	---	AT2G15000	2.84	1.50	11.69	1.02E-05	0.000843	4.20
247386_at	putative protein similar to unknown	emb2746	AT5G63420	2.40	1.26	11.66	1.04E-05	0.000843	4.19
263679_at	RNA helicase, putative similar to R	---	AT1G59990	3.12	1.64	11.66	1.04E-05	0.000843	4.18
253976_at	putative protein kinase protein kina	D6PKL1	AT4G26610	-2.08	-1.05	-11.62	1.06E-05	0.000845	4.16
256489_at	unknown protein	---	AT1G31550	-2.10	-1.07	-11.60	1.07E-05	0.000845	4.15
257635_at	cytochrome P450 monooxygenase	CYP71B4	AT3G26280	3.09	1.63	11.60	1.08E-05	0.000845	4.15
261317_at	cytochrome C oxidase assembly pr	---	AT1G53030	2.55	1.35	11.60	1.08E-05	0.000845	4.15
248219_at	unknown protein ; supported by ful	---	AT5G53650	2.00	1.00	11.59	1.08E-05	0.000845	4.15
245323_at	cysteine proteinase inhibitor like pro	---	AT4G16500	-3.22	-1.69	-11.57	1.09E-05	0.000845	4.14
263786_at	putative auxin-responsive protein ;	JAR1	AT2G46370	-1.99	-0.99	-11.54	1.11E-05	0.000856	4.12
259759_at	hypothetical protein similar to GB:C	---	AT1G77550	2.51	1.33	11.50	1.14E-05	0.00086	4.09
264344_at	putative aspartic proteinase similar	---	AT1G11910	2.05	1.04	11.50	1.14E-05	0.00086	4.09
250196_at	polynucleotide phosphorylase	---	AT5G14580	3.06	1.61	11.47	1.16E-05	0.000864	4.08
250484_at	asparagine synthetase ASN3 ; sup	ASN3	AT5G10240	2.42	1.28	11.46	1.16E-05	0.000865	4.07
247575_at	RNA-binding protein - like RNA-bin	GR-RBP3	AT5G61030	3.40	1.76	11.45	1.17E-05	0.000865	4.07
261796_at	non-phototropic hypocotyl, putative	---	AT1G30440	-2.47	-1.30	-11.44	1.17E-05	0.000865	4.06
251951_s_a	putative protein predicted proteins,	---	AT1G55710 ,	2.54	1.35	11.42	1.19E-05	0.00087	4.05
267534_at	putative CCCH-type zinc finger prot	---	AT2G41900	2.33	1.22	11.39	1.21E-05	0.000879	4.03
263483_at	putative heat shock protein ; suppo	CR88	AT2G04030	2.59	1.38	11.37	1.22E-05	0.00088	4.02
258616_at	putative protein kinase contains Pfa	---	AT3G02880	-3.14	-1.65	-11.31	1.27E-05	0.000897	3.98
248878_at	50S ribosomal protein L14	---	AT5G46160	2.33	1.22	11.28	1.29E-05	0.000903	3.97
247453_at	putative protein similar to unknown	---	AT5G62440	3.21	1.68	11.23	1.32E-05	0.00092	3.94
261872_s_a	hypthetical protein contains similarit	---	AT1G11520 ,	2.60	1.38	11.22	1.33E-05	0.00092	3.93
266419_at	putative annexin ;supported by full-	ANNAT3	AT2G38760	-3.68	-1.88	-11.21	1.34E-05	0.00092	3.92
249115_at	PINHEAD (gb|AAD40098.1); transla	ZLL	AT5G43810	2.12	1.08	11.20	1.34E-05	0.00092	3.92
253422_at	putative protein ;supported by full-l	---	AT4G32240	2.55	1.35	11.15	1.39E-05	0.000922	3.89
255365_at	putative phosphofructokinase beta s	MEE51	AT4G04040	2.27	1.19	11.14	1.39E-05	0.000922	3.88
259422_at	unknown protein	---	AT1G13810	3.73	1.90	11.14	1.40E-05	0.000922	3.88

248739_at	DnaJ protein-like ;supported by full	GFA2	AT5G48030	2.22	1.15	11.13	1.40E-05	0.000922	3.88
264512_at	Expressed protein ; supported by c	---	AT1G09575	-2.71	-1.44	-11.12	1.41E-05	0.000924	3.87
255651_at	putative protein	---	AT4G00940	2.49	1.32	11.11	1.42E-05	0.000924	3.86
245108_at	putative cytokinin oxidase	CKX1	AT2G41510	2.31	1.21	11.10	1.43E-05	0.000924	3.85
262505_at	unknown protein ESTs gb|N96028,	---	AT1G21680	2.73	1.45	11.03	1.49E-05	0.000946	3.81
266841_at	putative heat shock transcription fa	ATHSFA2	AT2G26150	-4.26	-2.09	-10.99	1.52E-05	0.000949	3.79
256092_at	Expressed protein ; supported by fu	HMGB3	AT1G20696	2.00	1.00	10.99	1.52E-05	0.000949	3.79
266480_at	unknown protein ; supported by cD	---	AT2G31130	2.13	1.09	10.96	1.55E-05	0.000962	3.77
248984_at	DNA-directed RNA polymerase sub	NRPC2	AT5G45140	3.03	1.60	10.96	1.55E-05	0.000962	3.77
253562_at	putative protein ;supported by full-l	---	AT4G31130	2.03	1.02	10.90	1.60E-05	0.000978	3.74
261838_at	heat shock protein hsp70, putative	Hsp70b	AT1G16030	2.39	1.26	10.90	1.61E-05	0.000978	3.73
264239_at	hypothetical protein predicted by ge	---	AT1G54770	2.12	1.08	10.88	1.62E-05	0.00098	3.72
254999_at	putative protein ; supported by full-	---	AT4G09830	3.13	1.65	10.84	1.66E-05	0.00099	3.70
259515_at	hypothetical protein ; supported by	---	AT1G20430	2.08	1.06	10.83	1.67E-05	0.000991	3.69
251278_at	putative protein hypothetical protei	emb1703	AT3G61780	2.69	1.43	10.77	1.74E-05	0.001013	3.65
263375_s_a	putative prohibitin ;supported by fu	ATPHB6	AT2G20530	2.69	1.43	10.75	1.75E-05	0.001013	3.65
262618_at	unknown protein	---	AT1G06560	2.03	1.02	10.74	1.77E-05	0.001014	3.63
259819_at	unknown protein ; supported by cDI	ATMTK	AT1G49820	2.00	1.00	10.72	1.78E-05	0.001015	3.63
247247_at	50S ribosomal protein L17 ;suppor	---	AT5G64650	2.20	1.14	10.68	1.83E-05	0.001029	3.60
264922_s_a	U2 snRNP auxiliary factor, large su	---	AT1G60830	2.06	1.04	10.67	1.84E-05	0.001029	3.59
255428_at	putative LRR receptor-like protein	SRF3	AT4G03390	-2.29	-1.20	-10.65	1.86E-05	0.001039	3.58
256466_at	plastid protein, putative similar to G	---	AT1G32580	2.02	1.01	10.64	1.87E-05	0.00104	3.57
249971_at	dermal glycoprotein - like dermal g	---	AT5G19110	-4.23	-2.08	-10.63	1.89E-05	0.001047	3.57
248326_at	Notchless protein homolog	---	AT5G52820	3.03	1.60	10.61	1.90E-05	0.00105	3.56
245710_at	ferulate-5-hydroxylase	---	AT5G04330	-3.26	-1.71	-10.59	1.93E-05	0.001057	3.54
253973_at	Expressed protein ; supported by c	---	AT4G26555	-1.89	-0.92	-10.58	1.94E-05	0.001059	3.53
251015_at	putative protein predicted protein, A	---	AT5G02480	2.10	1.07	10.55	1.97E-05	0.001067	3.52
266643_s_a	putative flavonol 3-O-glucosyltransf	---	AT2G29710	-3.04	-1.60	-10.54	1.98E-05	0.00107	3.51
263371_at	unknown protein ;supported by full-	NOP10	AT2G20490	2.89	1.53	10.50	2.04E-05	0.001082	3.48
265511_at	putative glycine-rich protein ; supp	---	AT2G05540	3.50	1.81	10.47	2.07E-05	0.001092	3.47
260595_at	unknown protein	---	AT1G55890	2.60	1.38	10.42	2.14E-05	0.001116	3.43
263236_at	putative response regulator 3 simil	ARR4	AT1G10470	-4.07	-2.03	-10.40	2.17E-05	0.001119	3.42
264811_at	hypothetical protein predicted by ge	---	AT1G08640	2.02	1.02	10.39	2.17E-05	0.001119	3.42
265139_at	unknown protein	---	AT1G51310	2.68	1.42	10.39	2.18E-05	0.001119	3.41
258850_at	putative cellulose synthase catalyti	CSLD3	AT3G03050	-2.36	-1.24	-10.39	2.18E-05	0.001119	3.41

248362_at	fibrillarin 1 (AtFib1) identical to fibril	FIB1	AT5G52470	2.20	1.14	10.37	2.20E-05	0.001119	3.40
265147_at	hypothetical protein	---	AT1G51380	3.41	1.77	10.37	2.20E-05	0.001119	3.40
261927_at	RING-H2 zinc finger protein ATL5,	---	AT1G22500	6.71	2.75	10.37	2.21E-05	0.001122	3.40
262946_at	hypothetical protein predicted by ge	---	AT1G79390	2.32	1.22	10.35	2.23E-05	0.001127	3.39
261406_at	unknown protein contains similarity	NRP2	AT1G18800	3.69	1.88	10.35	2.24E-05	0.001129	3.39
252362_at	hypothetical protein	PDE312	AT3G48500	4.02	2.01	10.33	2.26E-05	0.001135	3.38
266466_at	hypothetical protein The first 3 exon	---	AT2G47790	2.23	1.16	10.32	2.28E-05	0.001139	3.37
266197_at	hypothetical protein predicted by ge	---	AT2G39120	2.19	1.13	10.31	2.28E-05	0.001139	3.37
254452_at	UV-damaged DNA-binding protein-	DDB1B	AT4G21100	2.87	1.52	10.30	2.30E-05	0.001146	3.36
257724_at	hypothetical protein predicted by ge	---	AT3G18510	2.60	1.38	10.29	2.31E-05	0.001146	3.35
249433_at	putative protein hypothetical protei	---	AT5G39940	3.13	1.65	10.27	2.34E-05	0.001146	3.34
264513_at	putative glucose-6-phosphate dehy	G6PD4	AT1G09420	2.27	1.18	10.26	2.36E-05	0.001148	3.33
256672_at	ABC transporter, putative contains	---	AT3G52310	-2.82	-1.50	-10.24	2.39E-05	0.001159	3.32
258059_at	NAM-like protein (No Apical Merist	ATNAC3	AT3G29035	3.69	1.88	10.23	2.41E-05	0.001165	3.31
250767_at	putative protein contains similarity t	ATNFXL2	AT5G05660	-2.04	-1.03	-10.22	2.42E-05	0.001169	3.30
246316_at	putative protein predicted proteins -	---	AT3G56890	1.89	0.92	10.17	2.50E-05	0.001192	3.27
255795_at	putative calcium-binding EF-hand p	RD20	AT2G33380	-7.38	-2.88	-10.17	2.50E-05	0.001192	3.27
255668_s_a	predicted protein ; supported by full	---	AT4G00238	2.79	1.48	10.11	2.60E-05	0.001209	3.23
253942_at	putative protein	---	AT4G27010	3.74	1.90	10.11	2.60E-05	0.001209	3.23
258009_at	unknown protein	---	AT3G19440	1.89	0.92	10.10	2.61E-05	0.001213	3.22
256091_at	Expressed protein ; supported by fu	HMGB2	AT1G20693	2.26	1.17	10.08	2.64E-05	0.001218	3.21
256064_at	Expressed protein ; supported by fu	---	AT1G07020	-2.22	-1.15	-10.08	2.64E-05	0.001218	3.21
261819_at	receptor-like kinase, putative simila	---	AT1G11410	2.16	1.11	10.06	2.67E-05	0.001223	3.20
261487_at	hypothetical protein predicted by ge	---	AT1G14340	2.12	1.09	10.03	2.73E-05	0.001225	3.18
248394_at	unknown protein	---	AT5G52070	2.11	1.08	10.03	2.73E-05	0.001225	3.18
247776_at	phosphoinositide-specific phospho	PLC4	AT5G58700	-2.75	-1.46	-10.01	2.76E-05	0.001226	3.17
260985_at	dTDP-D-glucose 4,6-dehydratase,	MUM4	AT1G53500	-2.42	-1.28	-10.00	2.78E-05	0.001232	3.16
258531_at	importin alpha identical to GB:AAC	IMPA-1	AT3G06720	2.01	1.00	9.99	2.81E-05	0.001237	3.15
256892_at	hypothetical protein contains simila	---	AT3G19000	4.18	2.06	9.98	2.81E-05	0.001237	3.15
264605_at	putative auxin-induced protein, IAA	IAA12	AT1G04550	2.30	1.20	9.98	2.82E-05	0.001239	3.14
267082_at	CPDK-related protein kinase CDPK	CRK1	AT2G41140	2.03	1.02	9.96	2.84E-05	0.001239	3.13
263200_at	hypothetical protein predicted by ge	---	AT1G05600	2.90	1.54	9.96	2.85E-05	0.001239	3.13
255008_at	putative protein hypothetical protei	---	AT4G10060	-2.24	-1.17	-9.95	2.87E-05	0.001239	3.12
250126_at	ubiquitin C-terminal hydrolase-like p	UCH1	AT5G16310	2.57	1.36	9.93	2.90E-05	0.001249	3.11
248510_at	Expressed protein ; supported by fu	---	---	1.91	0.93	9.91	2.94E-05	0.001261	3.10

253803_at	putative protein hypothetical protei	---	AT4G28200	2.13	1.09	9.90	2.96E-05	0.001264	3.09
253917_at	hypothetical protein ;supported by	---	AT4G27380	3.29	1.72	9.90	2.96E-05	0.001264	3.09
251699_at	cytochrome P450-like protein cytoc	CYP94D2	AT3G56630	2.30	1.20	9.88	3.01E-05	0.001277	3.08
254788_at	putative protein other predicted prot	---	AT4G12790	-2.00	-1.00	-9.85	3.05E-05	0.001289	3.06
260020_at	hydrophilic protein, putative similar	PDF6	AT1G29990	1.92	0.94	9.83	3.10E-05	0.001296	3.04
255749_at	p68 RNA helicase, putative similar	STRS1	AT1G31970	2.15	1.10	9.82	3.12E-05	0.001303	3.04
245019_at	hypothetical protein	---	---	-1.87	-0.90	-9.81	3.13E-05	0.001307	3.03
247132_at	putative protein strong similarity to	ARO2	AT5G66200	-2.89	-1.53	-9.79	3.18E-05	0.00132	3.02
251637_at	hypothetical protein	---	AT3G57570	2.33	1.22	9.78	3.21E-05	0.001322	3.01
267033_at	unknown protein ;supported by full-	---	AT2G38450	1.86	0.89	9.73	3.30E-05	0.001347	2.98
250513_at	protein kinase ;supported by full-le	---	AT5G09890	-2.14	-1.10	-9.72	3.32E-05	0.001352	2.97
260897_at	ER lumen protein retaining receptor	ERD2	AT1G29330	-2.16	-1.11	-9.72	3.33E-05	0.001353	2.97
254303_at	predicted protein	---	AT4G22830	-2.08	-1.06	-9.72	3.34E-05	0.001353	2.96
252942_at	putative protein ;supported by full-l	---	AT4G39300	2.43	1.28	9.68	3.41E-05	0.00137	2.94
261250_at	unknown protein	---	AT1G05890	2.09	1.06	9.67	3.44E-05	0.00137	2.93
267337_at	putative anthocyanin 5-aromatic ac	---	AT2G39980	-3.28	-1.71	-9.65	3.48E-05	0.001378	2.92
250863_at	F1F0-ATPase inhibitor - like protei	---	AT5G04750	1.89	0.92	9.64	3.52E-05	0.001381	2.91
260607_at	putative receptor protein kinase	---	AT2G43700	2.75	1.46	9.63	3.54E-05	0.001384	2.90
261102_at	unknown protein	---	AT1G62880	1.99	0.99	9.63	3.54E-05	0.001384	2.90
246457_at	WD40-repeat protein transducin ho	TOZ	AT5G16750	2.24	1.16	9.62	3.54E-05	0.001384	2.90
259347_at	putative GAR1 protein similar to GE	---	AT3G03920	2.07	1.05	9.62	3.56E-05	0.001384	2.90
258114_at	putative cytochrome P450 similar to	CYP72A13	AT3G14660	2.13	1.09	9.61	3.57E-05	0.001384	2.89
266127_s_a	putative mitochondrial translation el	---	AT1G45332	2.95	1.56	9.60	3.60E-05	0.001393	2.88
258812_at	unknown protein similar to GB:AAD	ECT1	AT3G03950	2.41	1.27	9.59	3.62E-05	0.001396	2.88
263774_at	unknown protein ; supported by cD	---	AT2G40280	-2.04	-1.03	-9.58	3.65E-05	0.001405	2.87
260688_at	hypothetical protein similar to dehyd	---	AT1G17665	2.28	1.19	9.57	3.68E-05	0.00141	2.86
262754_at	putative inosine-5-monophosphate	---	AT1G16350	2.42	1.28	9.56	3.69E-05	0.00141	2.86
246478_at	putative protein several hypothetica	---	AT5G15980	2.05	1.03	9.56	3.70E-05	0.001411	2.86
251478_at	putative protein SF16 protein, polle	IQD13	AT3G59690	1.91	0.93	9.52	3.80E-05	0.001441	2.83
250485_at	Expressed protein ; supported by fu	PROPEP5	AT5G09990	3.91	1.97	9.51	3.82E-05	0.001441	2.82
264142_at	hypothetical protein predicted by ge	---	AT1G78930	3.06	1.61	9.51	3.83E-05	0.001441	2.82
255573_at	hypothetical protein	---	AT4G01400	-1.81	-0.86	-9.50	3.84E-05	0.001441	2.82
248864_at	bHLH protein-like	---	AT5G46760	1.87	0.91	9.50	3.85E-05	0.001443	2.81
253926_at	hnRNP-like protein ; supported by	---	AT4G26650	-2.02	-1.02	-9.49	3.86E-05	0.001443	2.81
260696_at	unknown protein	---	AT1G32520	2.25	1.17	9.49	3.88E-05	0.001443	2.81

254026_at	putative calmodulin-binding protein	---	AT4G25800	1.85	0.89	9.48	3.91E-05	0.00145	2.80
256595_x_a	hypothetical protein	---	AT3G28530	2.42	1.27	9.44	4.01E-05	0.001476	2.77
254779_at	hypothetical protein	---	AT4G12760	1.95	0.96	9.43	4.03E-05	0.001483	2.76
248933_at	putative protein contains similarity t	---	AT5G46070	2.04	1.03	9.41	4.08E-05	0.001493	2.75
250314_at	putative protein contains similarity t	---	AT5G12190	1.93	0.95	9.41	4.09E-05	0.001493	2.75
266072_at	putative trehalose-6-phosphate syn	ATTPS11	AT2G18700	-3.66	-1.87	-9.40	4.12E-05	0.001499	2.74
262751_at	hypothetical protein predicted by ge	---	AT1G16310	1.94	0.96	9.38	4.18E-05	0.001514	2.73
266903_at	hypothetical protein predicted by ge	MEE21	AT2G34570	1.94	0.95	9.36	4.24E-05	0.00152	2.71
245483_at	cysteine proteinase contains similar	---	AT4G16190	1.93	0.95	9.36	4.24E-05	0.00152	2.71
262780_at	putative cytochrome P450 monoox	CYP71B28	AT1G13090	2.68	1.42	9.34	4.28E-05	0.001528	2.70
250356_at	clathrin binding protein - like Af10-p	---	AT5G11710	-1.76	-0.82	-9.34	4.29E-05	0.001528	2.70
258663_at	hypothetical protein	---	AT3G08670	-1.92	-0.94	-9.33	4.30E-05	0.001528	2.70
253386_at	sulfolipid biosynthesis protein SQD1	SQD1	AT4G33030	2.86	1.52	9.32	4.33E-05	0.001535	2.69
267123_at	putative acetone-cyanohydrin lyase	MES7	AT2G23560	-3.94	-1.98	-9.31	4.36E-05	0.00154	2.68
249335_at	putative protein contains similarity t	NRPB12	AT5G41010	2.11	1.08	9.31	4.37E-05	0.001541	2.68
247529_at	monosaccharide transporter STP3	---	AT5G61520	2.10	1.07	9.30	4.40E-05	0.001546	2.67
250424_at	bromodomain protein - like bromod	GTE2	AT5G10550	-1.90	-0.92	-9.30	4.40E-05	0.001546	2.67
254679_at	putative protein Mei2-like protein, A	AML3	AT4G18120	2.15	1.10	9.29	4.44E-05	0.001549	2.66
263711_at	putative protein phosphatase 2C ;s	---	AT2G20630	-2.13	-1.09	-9.27	4.48E-05	0.001559	2.65
249140_at	F-box protein family, AtFBX6 conta	---	AT5G43190	-2.82	-1.49	-9.25	4.54E-05	0.001572	2.64
255243_at	putative protein hypothetical protei	---	AT4G05590	2.04	1.03	9.25	4.55E-05	0.001572	2.64
255278_at	putative WD-repeat membrane prot	---	AT4G04940	3.21	1.68	9.24	4.59E-05	0.001583	2.63
250985_at	putative protein various predicted p	---	AT5G02830	1.93	0.95	9.23	4.61E-05	0.00159	2.62
256033_at	UDP-glucose glucosyltransferase,	UGT71C4	AT1G07250	-2.04	-1.03	-9.22	4.66E-05	0.001593	2.61
251320_at	3-methyl-2-oxobutanoate hydroxy-i	PANB2	AT3G61530	1.90	0.93	9.21	4.68E-05	0.001597	2.61
257697_at	hypothetical protein predicted by ge	---	AT3G12700	-3.10	-1.63	-9.20	4.70E-05	0.001597	2.60
259272_at	unknown protein similar to hypothet	---	AT3G01290	-2.43	-1.28	-9.20	4.70E-05	0.001597	2.60
245502_at	hypothetical protein	---	AT4G15640	2.50	1.32	9.20	4.73E-05	0.001601	2.59
261034_s_a	hypothetical protein predicted by ge	---	AT1G17450	2.66	1.41	9.19	4.75E-05	0.001605	2.59
259593_at	nuclear transport factor 2, putative	NTF2B	AT1G27970	1.81	0.86	9.19	4.76E-05	0.001605	2.59
263532_s_a	putative protein RIO1 protein, Sacc	---	AT2G24990	2.77	1.47	9.18	4.78E-05	0.001612	2.58
251011_at	putative protein histone H2A, garde	HTA12	AT5G02560	1.92	0.94	9.16	4.83E-05	0.001615	2.57
265946_s_a	putative WD-40 repeat protein, MS	FVE	AT2G19520	1.92	0.94	9.16	4.84E-05	0.001615	2.57
261187_at	beta-1,3-glucanase precursor, puta	---	AT1G32860	-3.49	-1.80	-9.15	4.86E-05	0.001618	2.56
262026_at	calcium-dependent protein kinase i	ATCDPK2	AT1G35670	-2.50	-1.32	-9.15	4.89E-05	0.001618	2.56

266951_at	putative salt-inducible protein ; sup	---	AT2G18940	1.85	0.89	9.14	4.91E-05	0.001618	2.55
255600_s_a	putative protein predicted protein, A	---	AT4G01020	1.86	0.89	9.14	4.91E-05	0.001618	2.55
262307_at	heat shock protein DnaJ, putative s	---	AT1G71000	-2.66	-1.41	-9.13	4.92E-05	0.001618	2.55
266002_at	hypothetical protein predicted by ge	---	AT2G37310	2.10	1.07	9.13	4.95E-05	0.001618	2.55
262957_s_a	RNA polymerase II subunit, putativ	NRPB8A	AT1G54250	1.73	0.79	9.13	4.96E-05	0.001618	2.54
252921_at	putative protein DNA damage-indu	EDS5	AT4G39030	-5.10	-2.35	-9.12	4.96E-05	0.001618	2.54
265451_at	unknown protein	---	AT2G46490	-1.82	-0.87	-9.12	4.98E-05	0.00162	2.54
257007_at	Expressed protein ; supported by cl	---	AT3G14205	-1.85	-0.89	-9.12	4.99E-05	0.00162	2.54
258687_at	unknown protein	---	AT3G07860	2.76	1.47	9.10	5.03E-05	0.001622	2.53
249315_at	unknown protein	---	AT5G41190	1.88	0.91	9.09	5.08E-05	0.001634	2.52
252293_at	4-coumarate-CoA ligase -like prote	---	AT3G48990	1.96	0.97	9.08	5.13E-05	0.001643	2.51
258120_at	hypothetical protein predicted by ge	---	AT3G14730	1.86	0.90	9.07	5.14E-05	0.001643	2.51
254045_at	pumilio-like protein abdominal segr	APUM6	AT4G25880	1.92	0.94	9.07	5.15E-05	0.001643	2.50
259285_at	hypothetical protein similar to putati	---	AT3G11460	1.99	1.00	9.07	5.15E-05	0.001643	2.50
260519_at	unknown protein	---	AT1G51540	-1.82	-0.86	-9.05	5.21E-05	0.001655	2.49
257825_at	protein kinase, putative contains Pf	---	AT3G26700	-2.14	-1.10	-9.04	5.25E-05	0.001664	2.48
246947_at	cytochrome P450-like protein CYT(	CYP71B11	AT5G25120	1.97	0.98	9.04	5.26E-05	0.001666	2.48
259426_at	hypothetical protein contains similar	LEA14	AT1G01470	-2.23	-1.16	-9.03	5.29E-05	0.001666	2.47
266259_at	unknown protein ;supported by full-	---	AT2G27830	2.04	1.03	9.03	5.30E-05	0.001666	2.47
258244_at	unknown protein ; supported by cD	---	AT3G27770	-1.96	-0.97	-9.03	5.31E-05	0.001666	2.47
259346_at	putative glutamate dehydrogenase	GDH3	AT3G03910	-1.88	-0.91	-9.02	5.35E-05	0.001673	2.46
252031_at	putative protein 150-kD protein clu/	---	AT3G52140	1.96	0.97	9.01	5.35E-05	0.001673	2.46
265025_at	Expressed protein ; supported by fu	---	AT1G24575	2.20	1.14	9.01	5.36E-05	0.001673	2.46
264047_at	hypothetical protein predicted by ge	---	AT2G22410	2.03	1.02	8.99	5.43E-05	0.001687	2.45
245569_at	RNA polymerase II fifth largest subu	NRPE7	AT4G14660	1.80	0.85	8.99	5.44E-05	0.001687	2.45
264515_at	hypothetical protein similar to hypot	---	AT1G10090	-1.72	-0.78	-8.98	5.48E-05	0.001693	2.44
254635_at	extensin-like protein extensin-like p	---	AT4G18670	-2.26	-1.17	-8.98	5.48E-05	0.001693	2.44
256480_at	unknown protein	SAR1	AT1G33410	2.40	1.26	8.95	5.61E-05	0.001719	2.41
250495_at	ribsomal protein - like chloroplast ri	---	AT5G09770	2.46	1.30	8.94	5.62E-05	0.001719	2.41
265258_at	hypothetical protein predicted by ge	---	AT2G20390	1.81	0.86	8.94	5.63E-05	0.001719	2.41
253396_at	putative protein RNA-binding prote	AtLa1	AT4G32720	2.02	1.01	8.94	5.63E-05	0.001719	2.41
264951_at	unknown protein contains similarity	---	AT1G76970	1.77	0.83	8.94	5.64E-05	0.001719	2.41
251221_at	putative protein ER6 protein - Lyco	---	AT3G62550	2.07	1.05	8.93	5.66E-05	0.001724	2.40
253480_at	putative protein ENOD16, Medicag	---	AT4G31840	-1.98	-0.99	-8.93	5.68E-05	0.001728	2.40
261073_at	hypothetical protein predicted by ge	---	AT1G07300	2.04	1.03	8.93	5.69E-05	0.001728	2.40

255305_at	putative ABC transporter similar to	ATABC1	AT4G04770	2.56	1.36	8.92	5.72E-05	0.001734	2.39
249727_at	putative protein similar to unknown	MRU1	AT5G35490	-1.88	-0.91	-8.91	5.76E-05	0.001737	2.38
266551_at	unknown protein ; supported by cD	---	AT2G35260	-1.72	-0.78	-8.91	5.76E-05	0.001737	2.38
261891_at	transcriptional regulator, putative s	---	AT1G80790	1.80	0.85	8.90	5.81E-05	0.001752	2.37
252980_at	putative protein Human multiple ex	---	AT4G38040	-1.80	-0.85	-8.89	5.85E-05	0.001758	2.37
266229_at	putative RING zinc finger ankyrin pr	---	AT2G28840	1.91	0.93	8.89	5.86E-05	0.001758	2.37
257451_at	hypothetical protein predicted by ge	BT3	AT1G05690	2.07	1.05	8.88	5.87E-05	0.001758	2.36
249581_at	glutamate--ammonia ligase ; suppo	GSR 1	AT5G37600	1.98	0.98	8.88	5.87E-05	0.001758	2.36
250987_at	putative protein crp1 protein, Zea n	---	AT5G02860	2.25	1.17	8.88	5.89E-05	0.001758	2.36
258512_at	beta-glucosidase, putative similar t	---	AT3G06510	1.72	0.78	8.86	5.96E-05	0.001776	2.35
259835_at	unknown protein	TRZ3	AT1G52160	1.96	0.97	8.86	5.98E-05	0.001776	2.34
260294_at	GMP synthase similar to GMP synth	---	AT1G63660	1.90	0.93	8.86	5.98E-05	0.001776	2.34
256797_at	DEAD box helicase protein, putativ	---	AT3G18600	3.04	1.60	8.85	6.00E-05	0.001781	2.34
244906_at	hypothetical protein	---	ATMG00690	1.96	0.97	8.84	6.04E-05	0.001789	2.33
250978_at	putative protein contains transmem	---	AT5G03080	-1.78	-0.83	-8.84	6.06E-05	0.00179	2.33
249475_at	putative protein CGI-108 protein, H	---	AT5G38890	2.56	1.36	8.84	6.06E-05	0.00179	2.33
256745_at	UDP-glucose dehydrogenase, puta	---	AT3G29360	-2.35	-1.24	-8.84	6.07E-05	0.00179	2.33
260292_at	putative UDP-N-acetylmuramoylala	MURE	AT1G63680	1.73	0.79	8.83	6.07E-05	0.00179	2.33
251686_at	putative protein hypothetical protein	ATATG18D	AT3G56440	-1.90	-0.93	-8.83	6.09E-05	0.001791	2.32
261576_at	unknown protein ;supported by full-	---	AT1G01070	5.12	2.35	8.82	6.13E-05	0.001791	2.32
257122_at	RNA-binding protein, putative conta	APUM5	AT3G20250	3.01	1.59	8.82	6.13E-05	0.001791	2.32
255132_at	inositol 1,3,4-trisphosphate 5/6-kin	---	AT4G08170	-2.38	-1.25	-8.80	6.24E-05	0.00181	2.30
264689_at	hypothetical protein predicted by ge	---	AT1G09900	2.79	1.48	8.79	6.25E-05	0.001812	2.30
260733_at	RNA-binding protein, putative simil	---	AT1G17640	2.35	1.23	8.79	6.28E-05	0.001813	2.29
248292_at	putative protein similar to unknown	---	AT5G53030	2.93	1.55	8.79	6.29E-05	0.001813	2.29
251740_at	peptidylprolyl isomerase	ROC2	AT3G56070	4.17	2.06	8.78	6.29E-05	0.001813	2.29
246623_at	nuM1 protein, putative similar to nu	ATNUC-L1	AT1G48920	2.59	1.37	8.78	6.30E-05	0.001813	2.29
264099_at	replicase, putative similar to replica	---	AT1G79050	2.08	1.06	8.78	6.32E-05	0.001813	2.28
260824_at	unknown protein	---	AT1G06720	4.02	2.01	8.77	6.37E-05	0.001822	2.28
261070_at	disease resistance protein, putative	AtRLP1	AT1G07390	2.42	1.28	8.76	6.41E-05	0.001827	2.27
259881_at	Expressed protein ; supported by cI	---	AT1G76820	2.86	1.52	8.76	6.42E-05	0.001827	2.27
249253_at	putative protein similar to unknown	---	AT5G42060	2.18	1.13	8.76	6.42E-05	0.001827	2.27
263049_at	unknown protein ;supported by full-	---	AT2G05260	-2.04	-1.03	-8.75	6.43E-05	0.001827	2.26
265826_at	hypothetical protein predicted by ge	---	AT2G35795	1.83	0.87	8.74	6.51E-05	0.001843	2.25
259393_at	trehalose-6-phosphate synthase, p	ATTPS7	AT1G06410	-2.08	-1.06	-8.73	6.52E-05	0.001846	2.25

259884_at	hypothetical protein predicted by ge	---	AT1G76390	1.88	0.91	8.72	6.58E-05	0.001852	2.24
248710_at	unknown protein ;supported by full-	---	AT5G48480	2.50	1.32	8.72	6.60E-05	0.001852	2.24
256570_at	unknown protein ; supported by cD	---	AT3G19540	-1.78	-0.84	-8.71	6.62E-05	0.001856	2.23
263122_at	prenyltransferase, putative similar t	SPS1	AT1G78510	2.32	1.21	8.71	6.66E-05	0.001864	2.23
248192_at	IAA-amino acid hydrolase homolog	ILL3	AT5G54140	1.93	0.95	8.70	6.67E-05	0.001864	2.23
261839_at	unknown protein	---	AT1G16040	2.14	1.09	8.70	6.69E-05	0.001867	2.22
261303_at	unknown protein contains DNA-bind	HON5	AT1G48620	1.74	0.80	8.69	6.74E-05	0.00187	2.21
266824_at	homeodomain transcription factor (I	HAT9	AT2G22800	1.90	0.92	8.69	6.74E-05	0.00187	2.21
261319_at	phytochrome A supressor spa1, pu	SPA4	AT1G53090	1.68	0.75	8.69	6.75E-05	0.00187	2.21
255176_s_a	putative DNA-directed RNA polyme	---	AT1G01210	1.95	0.97	8.69	6.76E-05	0.00187	2.21
267051_at	hypothetical protein predicted by ge	---	AT2G38500	-2.72	-1.44	-8.68	6.76E-05	0.00187	2.21
263104_at	unknown protein	---	AT2G05120	2.14	1.10	8.68	6.79E-05	0.001871	2.21
253393_at	Expressed protein ; supported by cl	GLB3	AT4G32690	2.10	1.07	8.67	6.82E-05	0.001875	2.20
249375_at	putative protein predicted proteins,	AGP24	AT5G40730	-2.03	-1.02	-8.66	6.88E-05	0.001886	2.19
266194_at	unknown protein	---	AT2G39090	-1.69	-0.75	-8.66	6.90E-05	0.001887	2.19
263127_at	hypothetical protein predicted by ge	MSL6	AT1G78610	-2.24	-1.16	-8.65	6.94E-05	0.001888	2.18
252281_at	putative protein various predicted p	---	AT3G49320	2.58	1.37	8.65	6.95E-05	0.00189	2.18
246808_at	putative protein many predicted pro	---	AT5G27110	1.97	0.98	8.64	6.98E-05	0.00189	2.18
260486_at	F-box protein ZEITLUPE/FKF/LKP/	---	AT1G51550	2.03	1.02	8.64	6.99E-05	0.00189	2.18
265605_at	putative cellulose synthase catalytic	CESA10	AT2G25540	-1.69	-0.76	-8.63	7.04E-05	0.001897	2.17
250197_at	isocitrate dehydrogenase - like pro	---	AT5G14590	1.84	0.88	8.63	7.04E-05	0.001897	2.17
252868_at	putative protein tubulin-folding cofa	POR	AT4G39920	-1.81	-0.85	-8.61	7.12E-05	0.001909	2.16
247833_at	putative protein	---	AT5G58575	1.80	0.85	8.61	7.14E-05	0.00191	2.15
261044_at	molybdopterin biosynthesis CNX3	CNX3	AT1G01290	2.09	1.07	8.61	7.14E-05	0.00191	2.15
262656_at	unknown protein location of EST T/	---	AT1G14200	-2.04	-1.03	-8.59	7.23E-05	0.001924	2.14
252619_at	putative protein several hypothetica	---	AT3G45210	3.66	1.87	8.59	7.26E-05	0.001926	2.14
267344_at	hypothetical protein predicted by ge	---	AT2G44230	-4.75	-2.25	-8.58	7.27E-05	0.001926	2.13
259551_at	hypothetical protein	---	AT1G21190	1.94	0.96	8.58	7.30E-05	0.001927	2.13
248307_at	selenium-binding protein-like	---	AT5G52850	2.16	1.11	8.56	7.38E-05	0.001943	2.12
265961_at	unknown protein ;supported by full-	---	AT2G37400	2.94	1.55	8.56	7.39E-05	0.001943	2.12
252677_at	nitrilase 3 ;supported by full-length	NIT3	AT3G44320	-3.38	-1.76	-8.56	7.39E-05	0.001943	2.12
254147_at	putative protein similarity Human m	---	AT4G24270	3.03	1.60	8.56	7.41E-05	0.001945	2.11
252824_at	histone H3.3 ; supported by cDNA:	---	AT4G40030	2.09	1.06	8.55	7.43E-05	0.001946	2.11
265326_at	unknown protein predicted by gens	---	AT2G18220	2.59	1.37	8.55	7.43E-05	0.001946	2.11
257477_at	unknown protein	---	AT1G10660	2.46	1.30	8.55	7.45E-05	0.001946	2.11

265011_at	putative chloroplast membrane pro	ALB4	AT1G24490	1.71	0.78	8.55	7.45E-05	0.001946	2.11
253061_at	putative protein SPOP, Homo sapie	BT5	AT4G37610	-3.49	-1.80	-8.54	7.48E-05	0.001949	2.10
250217_at	nodulin-like protein	---	AT5G14120	-2.55	-1.35	-8.54	7.49E-05	0.001949	2.10
264169_at	hypothetical protein predicted by ge	---	AT1G02020	1.64	0.72	8.54	7.51E-05	0.001949	2.10
250679_at	putative protein contains similarity t	---	AT5G06550	2.82	1.50	8.54	7.53E-05	0.001952	2.10
262769_at	actin-like protein strong similarity to	DIS1	AT1G13180	-2.24	-1.16	-8.53	7.56E-05	0.001958	2.09
253294_at	hypothetical protein	---	AT4G33740	2.02	1.01	8.53	7.57E-05	0.001959	2.09
256808_at	unknown protein	ALY3	AT3G21430	1.77	0.82	8.52	7.60E-05	0.001961	2.09
251961_at	inorganic pyrophosphatase -like pr	AtPPa4	AT3G53620	-3.61	-1.85	-8.52	7.61E-05	0.001961	2.08
253249_at	GATA transcription factor 3	---	AT4G34680	2.46	1.30	8.52	7.61E-05	0.001961	2.08
247818_at	contains similarity to GTP-binding p	---	AT5G58370	1.97	0.98	8.50	7.70E-05	0.001976	2.07
254133_at	putative protein various predicted A	---	AT4G24810	1.78	0.83	8.50	7.73E-05	0.001978	2.07
263249_at	delta 9 desaturase ALMOST identic	ADS2	AT2G31360	-1.83	-0.87	-8.50	7.73E-05	0.001978	2.07
260407_at	putative protein kinase Pfam HMM	---	AT1G69910	-1.78	-0.83	-8.50	7.74E-05	0.001978	2.07
267099_at	DNA-(apurinic or apyrimidinic site) l	ARP	AT2G41460	2.96	1.57	8.49	7.79E-05	0.001986	2.06
248573_at	cellulase homolog OR16pep precur	ATGH9A1	AT5G49720	-2.16	-1.11	-8.49	7.80E-05	0.001986	2.06
262943_at	inosine-5'-monophosphate dehydro	---	AT1G79470	1.92	0.94	8.48	7.84E-05	0.001988	2.05
265029_at	zinc finger protein 7, ZFP7 ; suppo	ZFP7	AT1G24625	2.58	1.37	8.48	7.85E-05	0.001988	2.05
252183_at	UTP-glucose glucosyltransferase -	UGT72E1	AT3G50740	2.39	1.26	8.48	7.86E-05	0.001988	2.05
245269_at	hypothetical protein ; supported by	---	AT4G14500	-2.05	-1.03	-8.48	7.86E-05	0.001988	2.05
260390_at	unknown protein ; supported by ful	---	AT1G73940	2.13	1.09	8.47	7.89E-05	0.001988	2.05
259174_at	hypothetical protein similar to putat	UNE7	AT3G03690	-1.67	-0.74	-8.47	7.91E-05	0.00199	2.04
255870_at	hypothetical protein predicted by ge	---	AT2G30280	1.90	0.92	8.47	7.91E-05	0.00199	2.04
266366_at	unknown protein	---	AT2G41420	-2.32	-1.22	-8.46	7.95E-05	0.001996	2.04
245388_at	hypothetical protein ;supported by f	---	AT4G16410	1.80	0.84	8.45	8.01E-05	0.002002	2.03
265712_s_a	unknown protein	---	AT2G03330	1.95	0.97	8.45	8.01E-05	0.002002	2.03
257300_at	unknown protein ; supported by ful	---	AT3G28080	3.41	1.77	8.45	8.03E-05	0.002002	2.03
251087_at	putative protein unknown protein T	---	AT5G01460	-1.82	-0.87	-8.45	8.03E-05	0.002002	2.03
261979_at	nitrate reductase, putative similar t	NIA2	AT1G37130	3.26	1.71	8.44	8.09E-05	0.002015	2.02
255670_at	Expressed protein ; supported by fu	RHB1A	AT4G00335	1.76	0.81	8.43	8.15E-05	0.002024	2.01
246152_at	IPP transferase - like protein tRNA	IPT9	AT5G20040	1.91	0.93	8.40	8.30E-05	0.002057	1.99
251267_at	putative protein hypothetical protei	---	AT3G62330	-2.19	-1.13	-8.40	8.32E-05	0.002058	1.99
248315_at	selenium-binding protein-like	MEF1	AT5G52630	2.01	1.01	8.39	8.37E-05	0.002067	1.98
250463_at	transcription factor OBF4	TGA4	AT5G10030	2.19	1.13	8.39	8.39E-05	0.002069	1.98
246789_at	long-chain-fatty-acid--CoA ligase-li	LACS7	AT5G27600	1.84	0.88	8.38	8.42E-05	0.002073	1.98

250721_at	putative protein contains similarity t	---	AT5G06210	1.85	0.88	8.38	8.43E-05	0.002073	1.98
254778_at	putative protein	---	AT4G12750	2.34	1.23	8.38	8.45E-05	0.002076	1.97
247459_at	putative protein embryo-specific pr	---	AT5G62200	1.97	0.98	8.37	8.48E-05	0.002081	1.97
267562_at	unknown protein similar to hypothet	---	AT2G39670	1.84	0.88	8.37	8.49E-05	0.002081	1.97
253585_at	putative protein hypothetical protei	---	AT4G30720	1.85	0.89	8.36	8.54E-05	0.002091	1.96
262354_at	H+-transporting ATPase protein, pu	VHA-E3	AT1G64200	-4.60	-2.20	-8.36	8.59E-05	0.002099	1.96
246124_at	putative protein acyl-protein thioest	---	AT5G20060	-1.67	-0.74	-8.35	8.61E-05	0.002102	1.95
261409_at	zinc finger protein OBP2 identical to	OBP2	AT1G07640	2.02	1.02	8.35	8.63E-05	0.002105	1.95
262168_at	unknown protein ;supported by full-	---	AT1G74730	-1.98	-0.99	-8.34	8.67E-05	0.002112	1.95
252901_at	putative protein various predicted p	---	AT4G39550	1.86	0.89	8.32	8.82E-05	0.002136	1.93
258245_at	hypothetical protein	---	AT3G29075	-1.94	-0.96	-8.32	8.86E-05	0.002142	1.92
266005_at	unknown protein	RSZ33	AT2G37340	2.42	1.27	8.31	8.87E-05	0.002142	1.92
266957_at	unknown protein	PTAC12	AT2G34640	2.20	1.14	8.31	8.92E-05	0.002149	1.91
254955_at	putative protein RNA polymerase II	KELP	AT4G10920	1.85	0.88	8.30	8.93E-05	0.00215	1.91
247550_at	putative protein many predicted pro	---	AT5G61370	1.74	0.80	8.28	9.07E-05	0.002172	1.90
245465_at	cellulose synthase like protein	ATCSLA01	AT4G16590	-13.42	-3.75	-8.27	9.15E-05	0.002183	1.89
247187_at	putative protein similar to unknown	---	AT5G65490	1.90	0.93	8.27	9.15E-05	0.002183	1.89
253996_at	nucleosome assembly protein I-like	NAP1;1	AT4G26110	2.11	1.07	8.27	9.16E-05	0.002184	1.89
262947_at	unknown protein ;supported by full-	GASA1	AT1G75750	-1.67	-0.74	-8.26	9.23E-05	0.002188	1.88
247355_at	unknown protein ; supported by ful	SPT42	AT5G63670	1.84	0.88	8.26	9.23E-05	0.002188	1.88
247155_at	2-oxoglutarate dehydrogenase, E1	---	AT5G65750	2.63	1.40	8.24	9.35E-05	0.002204	1.86
263449_at	unknown protein	AtRABA5d	AT2G31670	-1.85	-0.89	-8.24	9.39E-05	0.002205	1.86
253538_at	putative protein ribosomal protein Y	---	AT4G31460	1.75	0.81	8.23	9.41E-05	0.002205	1.86
266288_s_a	putative pumilio/Mpt5 family RNA-b	APUM1	AT2G29140	2.16	1.11	8.23	9.41E-05	0.002205	1.86
252875_at	GTP-binding protein GB3 ;supporte	RABA4B	AT4G39990	-1.80	-0.85	-8.22	9.49E-05	0.002222	1.85
267405_at	putative related to microbial divalen	CUTA	AT2G33740	1.80	0.84	8.22	9.50E-05	0.002222	1.85
253344_at	putative protein predicted protein, A	---	AT4G33550	-3.65	-1.87	-8.21	9.56E-05	0.002229	1.84
248197_at	NADPH:protochlorophyllide oxidore	PORA	AT5G54190	-1.90	-0.93	-8.21	9.56E-05	0.002229	1.84
266715_at	putative RNA-binding protein	---	AT2G46780	-2.14	-1.10	-8.21	9.56E-05	0.002229	1.84
265828_at	hypothetical protein predicted by ge	---	AT2G14520	1.79	0.84	8.21	9.57E-05	0.002229	1.84
248378_at	unknown protein ;supported by full-	---	AT5G51840	-1.82	-0.86	-8.21	9.58E-05	0.002229	1.84
257149_at	prohibitin, putative similar to prohib	ATPHB4	AT3G27280	3.93	1.97	8.21	9.62E-05	0.002229	1.83
245244_at	IAA-amino acid hydrolase, putative	ILL6	AT1G44350	-2.77	-1.47	-8.19	9.70E-05	0.002233	1.82
262094_at	SAR DNA binding protein, putative	NOP56	AT1G56110	4.16	2.06	8.18	9.78E-05	0.002245	1.81
256002_at	carbamoylphosphate synthetase, p	CARB	AT1G29900	1.97	0.98	8.18	9.80E-05	0.002246	1.81

259081_at	putative sodium proton exchanger s	NHX2	AT3G05030	2.24	1.16	8.17	9.91E-05	0.002265	1.80
261535_at	Expressed protein ; supported by fu	---	AT1G01725	2.21	1.15	8.16	9.95E-05	0.002269	1.80
247406_at	response regulator 6 (ARR6) ; supp	ARR6	AT5G62920	-5.37	-2.43	-8.16	9.95E-05	0.002269	1.80
250112_at	low density lipoprotein B-like protein	---	AT5G16300	-1.80	-0.85	-8.16	9.96E-05	0.002269	1.79
266557_at	unknown protein ; supported by cD	---	AT2G46080	1.64	0.71	8.16	9.97E-05	0.002269	1.79
267511_at	unknown protein	---	AT2G45670	-1.88	-0.91	-8.15	0.0001	0.002276	1.79
247641_at	imidazoleglycerol-phosphate synth	PDX2	AT5G60540	1.96	0.97	8.14	0.000101	0.002281	1.78
266589_at	hypothetical protein predicted by ge	---	AT2G46250	2.45	1.29	8.14	0.000101	0.002285	1.78
251927_at	hypothetical protein ER6 protein - L	---	AT3G53990	-1.80	-0.84	-8.13	0.000102	0.002291	1.77
266570_at	putative chloroplast ribosomal prote	---	AT2G24090	-1.82	-0.87	-8.13	0.000102	0.002291	1.77
253672_at	mRNA cleavage factor subunit - lik	CFIM-25	AT4G29820	2.27	1.18	8.12	0.000103	0.002302	1.76
256890_at	glycine-rich RNA binding protein, p	GRP4	AT3G23830	4.17	2.06	8.11	0.000103	0.002306	1.76
249821_at	putative protein contains similarity t	---	AT5G23690	3.09	1.63	8.11	0.000103	0.002307	1.76
259344_at	putative polynucleotide phosphoryl	RIF10	AT3G03710	2.19	1.13	8.09	0.000105	0.002329	1.74
257218_at	unknown protein similar to DAG pro	---	AT3G15000	1.82	0.86	8.09	0.000105	0.002329	1.74
254079_at	putative protein pmt2 methyltransfe	---	AT4G25730	3.42	1.77	8.09	0.000105	0.002329	1.74
253004_at	putative protein	---	AT4G38280	2.46	1.30	8.09	0.000105	0.002331	1.74
251678_at	putative protein conserved hypothe	EDA7	AT3G56990	1.78	0.83	8.09	0.000105	0.002331	1.74
254593_s_a	putative protein IEF SSP 9502, Ho	---	AT4G18900	2.16	1.11	8.08	0.000106	0.002333	1.73
252613_at	putative protein hypothetical protein	---	AT3G45190	1.70	0.77	8.08	0.000106	0.002333	1.73
256253_at	60S acidic ribosomal protein, putat	---	AT3G11250	2.02	1.02	8.08	0.000106	0.002342	1.73
254919_at	RING-H2 finger protein RHA1b ;su	RHA1B	AT4G11360	2.88	1.53	8.07	0.000106	0.002344	1.73
246173_s_a	putative protein hypothetical protein	---	AT3G61520	2.49	1.32	8.06	0.000107	0.002357	1.71
257674_at	translational inhibitor protein, putat	---	AT3G20390	-1.82	-0.87	-8.06	0.000107	0.002357	1.71
254187_at	putative protein hypothetical protein	---	AT4G23890	1.73	0.79	8.06	0.000107	0.002357	1.71
250329_at	putative potassium transport protei	KEA6	AT5G11800	-1.95	-0.96	-8.06	0.000108	0.002359	1.71
251256_at	putative protein hypothetical protein	---	AT3G62300	2.25	1.17	8.05	0.000108	0.002365	1.70
247907_at	unknown protein	---	AT5G57460	-1.75	-0.81	-8.04	0.000109	0.002367	1.70
252723_at	hypothetical protein ; supported by	---	AT3G43520	1.71	0.77	8.04	0.000109	0.002367	1.70
253388_at	putative protein ; supported by cDN	---	AT4G32910	2.59	1.38	8.04	0.000109	0.002367	1.70
256458_at	integral membrane protein, putative	---	AT1G75220	-2.96	-1.57	-8.04	0.000109	0.00237	1.70
262595_at	putative ethylene responsive eleme	SHN1	AT1G15360	-1.91	-0.93	-8.03	0.00011	0.002372	1.69
259846_at	peptide transporter PTR2-B, putativ	---	AT1G72140	-2.04	-1.03	-8.03	0.00011	0.002387	1.69
265272_at	unknown protein	ARF10	AT2G28350	2.52	1.34	8.02	0.00011	0.002388	1.68
250510_at	15.9 kDa subunit of RNA polymeras	NRPB4	AT5G09920	1.80	0.85	8.02	0.000111	0.002393	1.68

255686_at	putative potassium/H+ antiporter	KEA2	AT4G00630	2.20	1.14	8.01	0.000112	0.0024	1.67
247763_at	RNA polymerase II	NRPB7	AT5G59180	1.89	0.92	8.00	0.000112	0.002407	1.67
250004_at	putative protein predicted proteins -	---	AT5G18750	2.37	1.24	7.99	0.000114	0.002425	1.65
263863_at	3-oxoacyl carrier protein synthase	---	AT2G04540	1.67	0.74	7.99	0.000114	0.002425	1.65
250743_at	phosphate/phosphoenolpyruvate tra	---	AT5G05820	-2.02	-1.02	-7.98	0.000114	0.002425	1.65
249785_at	soluble starch synthase	SSI1	AT5G24300	-1.98	-0.99	-7.98	0.000114	0.002425	1.65
266117_at	hypothetical protein ; supported by	---	AT2G02170	-1.78	-0.83	-7.98	0.000114	0.002425	1.65
259932_at	zinc finger protein, putative similar	STOP1	AT1G34370	1.95	0.96	7.98	0.000114	0.002425	1.65
251987_at	CYTOCHROME P450 71B5 ; supp	CYP71B5	AT3G53280	2.10	1.07	7.98	0.000114	0.002427	1.65
255338_at	PEROXISOMAL MEMBRANE PRO	PMP22	AT4G04470	1.72	0.79	7.97	0.000115	0.002436	1.64
263637_at	hypothetical protein predicted by ge	---	AT2G11890	1.78	0.83	7.97	0.000115	0.002436	1.64
265475_at	ferredoxin--nitrite reductase ; supp	NIR1	AT2G15620	2.62	1.39	7.97	0.000115	0.002439	1.64
249359_at	putative protein unnamed ORF, Ho	---	AT5G40470	2.18	1.13	7.96	0.000116	0.00245	1.63
265472_at	putative RING-H2 zinc finger protei	---	AT2G15580	2.17	1.12	7.96	0.000116	0.002453	1.63
247735_at	thymidylate kinase - like protein thy	ZEU1	AT5G59440	3.68	1.88	7.95	0.000117	0.002457	1.62
250753_at	glucuronosyl transferase-like protei	UGT76C2	AT5G05860	-2.29	-1.19	-7.94	0.000118	0.002472	1.61
258485_at	putative stearoyl-acyl carrier protein	---	AT3G02630	1.61	0.69	7.94	0.000118	0.002479	1.61
264924_at	RNA polymerase subunit identical t	ATRPAC43	AT1G60620	1.77	0.83	7.93	0.000119	0.002489	1.60
267044_at	nodulin-like protein	---	AT2G34357	3.05	1.61	7.92	0.00012	0.002497	1.60
252058_at	putative protein hin1 protein -Nicoti	---	AT3G52470	-2.82	-1.50	-7.92	0.00012	0.002497	1.60
262402_at	unknown protein ; supported by cDI	TOM6	AT1G49410	2.29	1.19	7.92	0.00012	0.002497	1.59
252093_at	putative protein predicted protein, (	---	AT3G51500	1.96	0.97	7.91	0.00012	0.0025	1.59
255465_at	hypothetical protein similar to A. th	---	AT4G02990	2.16	1.11	7.91	0.000121	0.0025	1.59
262846_at	endomembrane protein, putative si	---	AT1G14670	-1.97	-0.97	-7.91	0.000121	0.0025	1.59
250028_at	putative protein similar to unknown	---	AT5G18130	1.88	0.91	7.91	0.000121	0.0025	1.59
253322_at	putative protein ;supported by full-l	---	AT4G33980	2.29	1.19	7.90	0.000121	0.002501	1.58
261803_at	transcription factor, putative similar	NF-YA7	AT1G30500	2.43	1.28	7.90	0.000121	0.002501	1.58
257211_at	exonuclease, putative contains Pfa	---	AT3G15080	2.32	1.21	7.90	0.000121	0.002505	1.58
257369_at	hypothetical protein predicted by ge	BPC7	AT2G35550	1.99	0.99	7.90	0.000122	0.00251	1.58
255819_s_a	40S ribosomal protein S26	---	AT2G40510	1.87	0.90	7.89	0.000122	0.002512	1.58
261954_at	plastid ribosomal protein S6, putati	---	AT1G64510	-1.89	-0.92	-7.89	0.000123	0.002516	1.57
265678_at	putative RAD50 DNA repair protein	RAD50	AT2G31970	2.75	1.46	7.89	0.000123	0.002516	1.57
248369_at	arginine/serine-rich splicing factor F	ATRSP41	AT5G52040	2.29	1.19	7.88	0.000123	0.002516	1.57
262714_s_a	unknown protein similar to gb|AJ22	---	AT1G07950	1.78	0.83	7.88	0.000124	0.002527	1.56
258953_at	hypothetical protein predicted by ge	---	AT3G01430	-2.10	-1.07	-7.87	0.000125	0.002534	1.55

248039_at	putative protein similar to unknown	---	AT5G55950	-1.68	-0.75	-7.87	0.000125	0.002537	1.55
247836_at	putative protein similar to unknown	---	AT5G57860	2.05	1.04	7.86	0.000125	0.002537	1.55
254763_at	ribosomal protein L13a like protein	---	AT4G13170	2.24	1.16	7.86	0.000125	0.002542	1.55
247261_at	ZW10-like protein ; supported by c	---	AT5G64460	-1.82	-0.87	-7.85	0.000126	0.002556	1.54
264436_at	putative glutathione S-transferase T	ERD9	AT1G10370	-10.54	-3.40	-7.85	0.000126	0.002557	1.54
260160_at	putative RNA-binding protein conta	---	AT1G79880	1.79	0.84	7.85	0.000127	0.00256	1.54
246856_at	nucleic acid binding protein - like n	AL4	AT5G26210	1.68	0.75	7.84	0.000127	0.00256	1.53
259955_s_a	unknown protein ;supported by full-	BZR1	AT1G19350	1.98	0.99	7.84	0.000127	0.00256	1.53
266108_at	putative peptide/amino acid transpo	---	AT2G37900	-2.11	-1.08	-7.84	0.000127	0.00256	1.53
256979_at	Expressed protein ; supported by fu	PSBTN	AT3G09550	-1.99	-0.99	-7.84	0.000127	0.00256	1.53
246253_at	myb-related protein	MYB73	AT4G37260	-2.39	-1.26	-7.83	0.000129	0.002582	1.52
256675_at	putative protein DNA-binding prote	---	AT3G52170	3.34	1.74	7.82	0.000129	0.002584	1.52
256337_at	unknown protein	---	AT1G72060	6.28	2.65	7.82	0.000129	0.002584	1.52
247029_at	TINY-like protein	---	AT5G67190	-2.30	-1.20	-7.82	0.00013	0.002588	1.51
251355_at	putative protein hypothetical protei	---	AT3G61100	2.23	1.16	7.82	0.00013	0.002588	1.51
259859_at	putative protein phosphatase simila	---	AT1G68410	-4.74	-2.25	-7.81	0.000131	0.002598	1.50
257891_at	hypothetical protein predicted by ge	RFC3	AT3G17170	2.69	1.43	7.81	0.000131	0.002598	1.50
261547_at	protein kinase, putative contains P	---	AT1G63500	-1.90	-0.93	-7.80	0.000131	0.002605	1.50
257756_at	uridylate kinase, putative similar to	---	AT3G18680	2.03	1.02	7.80	0.000131	0.002606	1.50
252508_at	putative protein tRNA nucleotidyltra	---	AT3G46210	2.74	1.45	7.80	0.000131	0.002606	1.50
244980_at	ribosomal protein L36	---	---	-1.87	-0.90	-7.80	0.000132	0.002609	1.49
264633_at	step II splicing factor, putative simil	SMP1	AT1G65660	1.90	0.93	7.79	0.000132	0.002615	1.49
258079_at	hypothetical protein predicted by ge	---	AT3G25940	1.88	0.91	7.78	0.000133	0.002624	1.48
260995_at	unknown protein	---	AT1G12120	-1.85	-0.88	-7.77	0.000134	0.002643	1.47
265900_at	unknown protein	---	AT2G25730	1.59	0.67	7.77	0.000134	0.002643	1.47
250316_at	cystatin (emb|CAA03929.1) ; suppo	ATCYS1	AT5G12140	-1.82	-0.86	-7.77	0.000135	0.002649	1.47
255481_at	T14P8.6 ; supported by cDNA: gi_	PMS1	AT4G02460	2.25	1.17	7.77	0.000135	0.002649	1.47
266822_at	60S ribosomal protein L30 ;suppor	---	AT2G44860	2.48	1.31	7.76	0.000135	0.002657	1.46
256279_at	protein phosphatase 2C, putative c	---	AT3G12620	-1.60	-0.68	-7.76	0.000135	0.002657	1.46
257948_at	hypothetical protein predicted by ge	APO4	AT3G21740	1.96	0.97	7.75	0.000137	0.002673	1.45
265722_at	putative chlorophyll a/b binding prot	LHCB4.3	AT2G40100	-2.87	-1.52	-7.75	0.000137	0.002674	1.45
262636_at	DEIH-box RNA/DNA helicase identi	NIH	AT1G06670	2.06	1.04	7.75	0.000137	0.002674	1.45
260915_at	hypothetical protein similar to hypot	---	AT1G02660	-4.33	-2.11	-7.75	0.000137	0.002674	1.45
260129_at	hypothetical protein similar to vanill	---	AT1G36380	1.73	0.79	7.74	0.000137	0.002679	1.45
255446_at	hypothetical protein similar to A. th	---	AT4G02750	1.98	0.98	7.74	0.000138	0.002682	1.45

245797_at	defective chloroplasts and leaves (	---	AT1G45230	1.58	0.66	7.73	0.000138	0.002682	1.44
267596_s_a	putative leucine-rich repeat disease	AtRLP26	AT2G33050	4.92	2.30	7.73	0.000139	0.002682	1.44
252990_at	putative protein	---	AT4G38440	3.56	1.83	7.73	0.000139	0.002682	1.44
250034_at	apyrase (gb|AAF00612.1) ; suppor	ATAPY2	AT5G18280	-1.99	-0.99	-7.73	0.000139	0.002682	1.44
263548_at	glycine-rich RNA binding protein 7	CCR2	AT2G21660	1.92	0.94	7.72	0.000139	0.002687	1.43
267036_at	Expressed protein ; supported by fu	---	AT2G38465	3.07	1.62	7.72	0.00014	0.002687	1.43
266888_s_a	putative thiamin pyrophosphokinase	TPK1	AT1G02880	1.79	0.84	7.72	0.00014	0.002687	1.43
246398_at	auxin-induced basic helix-loop-heli	---	AT1G58100	-1.61	-0.69	-7.72	0.00014	0.002691	1.43
247572_at	putative protein ; supported by full-	---	AT5G61220	1.73	0.79	7.72	0.00014	0.002692	1.43
253490_at	methyltransferase - like protein me	---	AT4G31790	3.45	1.79	7.72	0.00014	0.002692	1.43
249641_at	DegP protease contains similarity to	DegP10	AT5G36950	1.97	0.98	7.72	0.00014	0.002692	1.43
257016_at	protein kinase, putative similar to P	---	AT3G28690	1.99	0.99	7.70	0.000142	0.002709	1.41
264835_at	unknown protein similar to unknown	---	AT1G03550	1.60	0.68	7.70	0.000142	0.002709	1.41
251642_at	imbibition protein homolog probabl	AtSIP2	AT3G57520	-1.94	-0.96	-7.70	0.000142	0.002709	1.41
257154_at	unknown protein ; supported by cD	---	AT3G27210	2.21	1.15	7.70	0.000142	0.002711	1.41
258553_at	hypothetical protein ; supported by	PDE320	AT3G06960	1.74	0.80	7.69	0.000143	0.002712	1.41
247277_at	putative protein similar to unknown	---	AT5G64420	1.88	0.91	7.69	0.000143	0.002712	1.41
267081_at	putative phosphatidylinositol-4-phos	PIP5K5	AT2G41210	1.82	0.87	7.69	0.000143	0.002714	1.40
264953_at	alcohol dehydrogenase identical to	ADH1	AT1G77120	-2.30	-1.20	-7.69	0.000143	0.002714	1.40
256511_at	RNA helicase, putative similar to G	---	AT1G33390	1.75	0.81	7.69	0.000144	0.002717	1.40
256789_at	seven in absentia-like protein simila	---	AT3G13672	3.66	1.87	7.69	0.000144	0.00272	1.40
250148_at	putative protein various predicted p	---	AT5G14530	1.96	0.97	7.68	0.000144	0.002722	1.40
264094_at	unknown protein ;supported by full-	---	AT1G79200	1.96	0.97	7.68	0.000145	0.002726	1.39
266579_at	putative small nuclear ribonucleopro	SNRNP-G	AT2G23930	2.13	1.09	7.68	0.000145	0.002726	1.39
263912_at	starch branching enzyme II	SBE2.1	AT2G36390	2.23	1.16	7.67	0.000145	0.00273	1.39
261306_at	regulatory protein HAL3B similar to	---	AT1G48610	-1.86	-0.89	-7.67	0.000146	0.002735	1.38
248549_at	putative protein contains similarity t	---	AT5G50310	1.81	0.85	7.66	0.000146	0.002735	1.38
261062_at	transcription factor scarecrow-like 1	SCL14	AT1G07530	1.82	0.86	7.66	0.000146	0.002735	1.38
262590_at	putative RING-H2 zinc finger prote	RHA2A	AT1G15100	1.98	0.99	7.66	0.000147	0.00274	1.38
255010_at	lipase-like protein monoglyceride li	---	AT4G10050	1.75	0.81	7.66	0.000147	0.00274	1.38
259340_at	hypothetical protein predicted using	PYK10	AT3G03870	2.02	1.01	7.66	0.000147	0.00274	1.37
251959_at	putative protein KIAA0544 protein,	---	AT3G53410	1.78	0.83	7.65	0.000148	0.002756	1.37
259643_at	putative menaquinone biosynthesis	---	AT1G68890	-1.71	-0.77	-7.65	0.000149	0.002763	1.36
249330_at	putative protein similar to unknown	---	AT5G40970	1.86	0.90	7.64	0.000149	0.002769	1.36
257730_at	unknown protein contains Pfam pro	---	AT3G18420	2.38	1.25	7.63	0.00015	0.002785	1.35

262001_at	myrosinase binding protein, putativ	---	AT1G33790	-1.99	-0.99	-7.63	0.000151	0.002793	1.35
261069_at	small G protein, putative similar to	ATRABA2E	AT1G07410	-1.64	-0.71	-7.62	0.000151	0.002796	1.35
266284_at	putative pumilio/Mpt5 family RNA-b	APUM2	AT2G29190	1.94	0.95	7.62	0.000151	0.002797	1.34
256229_at	unknown protein	MSL3	AT1G58200	-2.26	-1.18	-7.61	0.000152	0.002813	1.34
254526_at	putative protein cyclin C homolog 1	CYCT1;4	AT4G19600	1.75	0.81	7.61	0.000153	0.002813	1.33
255572_at	hypothetical protein ; supported by	---	AT4G01050	-1.60	-0.68	-7.61	0.000153	0.002813	1.33
250627_at	putative amidase	---	AT5G07360	2.05	1.04	7.61	0.000153	0.002813	1.33
252548_at	kinesin-related protein - like kinesin	---	AT3G45850	-1.97	-0.98	-7.61	0.000153	0.002813	1.33
249997_at	chromatin remodelling complex AT	CHR17	AT5G18620	1.98	0.98	7.61	0.000153	0.002813	1.33
259130_at	putative ribosomal protein L39 simil	---	AT3G02190	1.98	0.99	7.61	0.000153	0.002813	1.33
250283_at	putative protein many hypothetical	---	AT5G13270	1.91	0.93	7.60	0.000154	0.002817	1.33
246943_at	putative protein kinase serine/threo	---	AT5G25440	-2.66	-1.41	-7.59	0.000155	0.002843	1.32
249947_at	FVT1 - like protein follicular lympho	---	AT5G19200	-1.68	-0.75	-7.58	0.000156	0.002854	1.31
260373_at	unknown protein	---	AT1G73970	1.81	0.86	7.58	0.000156	0.002854	1.31
247632_at	protein transport protein subunit - li	---	AT5G60460	2.00	1.00	7.58	0.000157	0.002863	1.31
251429_at	dynamin-like protein 4 (ADL4) ; sup	ADL1E	AT3G60190	1.91	0.94	7.57	0.000157	0.002868	1.30
266422_at	hypothetical protein predicted by ge	GAUT7	AT2G38650	-1.78	-0.84	-7.57	0.000158	0.002876	1.30
262931_at	small nuclear ribonucleoprotein, pu	---	AT1G65700	1.87	0.90	7.57	0.000158	0.002877	1.30
266490_at	unknown protein	---	AT2G07000	2.08	1.06	7.57	0.000158	0.002877	1.29
256204_at	DNA polymerase A family protein,	POLGAMM	AT1G50840	2.17	1.12	7.57	0.000158	0.002877	1.29
253964_at	putative protein qkI-7, Mus muscul	---	AT4G26480	-1.82	-0.86	-7.56	0.000159	0.002877	1.29
265768_at	putative sugar transporter ;support	---	AT2G48020	-1.74	-0.80	-7.56	0.000159	0.002878	1.29
257131_at	mitochondrial carrier protein, putati	---	AT3G20240	1.68	0.75	7.56	0.000159	0.002878	1.29
251421_at	enoyl-CoA-hydratase - like protein	---	AT3G60510	1.83	0.87	7.56	0.00016	0.00288	1.29
258489_at	14-3-3 protein GF14nu (grf7) identic	GRF7	AT3G02520	1.68	0.75	7.55	0.00016	0.002885	1.28
250087_at	putative protein contains similarity t	---	AT5G17270	2.86	1.51	7.55	0.00016	0.002885	1.28
246619_at	transmembrane protein FT27/PFT2	---	AT5G36290	-1.66	-0.73	-7.55	0.00016	0.002886	1.28
260289_at	CAD ATPase (AAA1) identical to C	ERH3	AT1G80350	-1.57	-0.65	-7.54	0.000161	0.002896	1.27
262678_at	hypothetical protein ;supported by f	---	AT1G75810	2.28	1.19	7.54	0.000162	0.002898	1.27
260705_at	unknown protein ;supported by full-	TOM2A	AT1G32400	-1.71	-0.77	-7.54	0.000162	0.0029	1.27
253557_at	putative protein wall-associated kin	---	AT4G31110	1.65	0.72	7.53	0.000163	0.002903	1.27
245479_at	extensin like protein	---	AT4G16140	-1.86	-0.90	-7.53	0.000163	0.002905	1.27
264120_at	latex-abundant protein, putative sir	AtMC4	AT1G79340	-1.64	-0.72	-7.53	0.000163	0.002908	1.26
265483_at	hypothetical protein ; supported by	SQN	AT2G15790	2.38	1.25	7.52	0.000164	0.002918	1.26
259793_at	AP2-containing DNA-binding protei	---	AT1G64380	-5.53	-2.47	-7.52	0.000165	0.002921	1.25

252113_at	putative protein predicted protein, A	---	AT3G51620	1.67	0.74	7.52	0.000165	0.002921	1.25
261524_at	hypothetical protein predicted by ge	---	AT1G14300	1.97	0.98	7.51	0.000165	0.002925	1.25
254038_at	nitrogen fixation like protein nitroge	NFU3	AT4G25910	1.70	0.77	7.51	0.000166	0.002929	1.25
266986_at	putative dolichyl-phosphate beta-glu	---	AT2G39630	1.67	0.74	7.51	0.000166	0.002932	1.24
262231_at	putative receptor protein similar to	---	AT1G68740	4.20	2.07	7.50	0.000167	0.00294	1.24
255829_at	putative potassium transporter ; su	KT2	AT2G40540	-1.65	-0.72	-7.50	0.000167	0.002943	1.24
263064_at	putative bZIP transcription factor co	CPuORF1	AT2G18160	2.34	1.23	7.50	0.000168	0.002948	1.23
252625_at	putative histone deacetylase similar	HDA3	AT3G44750	5.29	2.40	7.49	0.000169	0.00296	1.23
249977_at	chaperonin 60 alpha chain - like pr	EMB3007	AT5G18820	2.25	1.17	7.49	0.000169	0.00296	1.23
255685_s_a	putative tetrahydrofolate synthase s	---	AT4G00600	2.62	1.39	7.49	0.000169	0.002961	1.22
246594_at	pyrroline-5-carboxylate reductase ;	P5CR	AT5G14800	2.13	1.09	7.48	0.000169	0.002966	1.22
255482_at	putative chloroplast outer envelope	TOC159	AT4G02510	1.76	0.82	7.48	0.00017	0.002967	1.22
251427_at	beta-glucosidase-like protein severa	BGLU16	AT3G60130	-2.37	-1.25	-7.48	0.00017	0.002975	1.22
249864_at	putative protein similar to unknown	ATMGT10	AT5G22830	-1.63	-0.71	-7.47	0.000171	0.002977	1.21
250787_at	meiotic check point regulator-like pr	EMB2771	AT5G05560	1.73	0.79	7.47	0.000171	0.00298	1.21
257799_at	protein kinase, putative similar to s	---	AT3G15890	1.85	0.89	7.47	0.000171	0.002982	1.21
251447_at	U6 snRNA-associated Sm-like prot	---	AT3G59810	1.63	0.71	7.46	0.000172	0.002992	1.20
246479_at	hypothetical protein	---	AT5G16060	1.83	0.87	7.46	0.000173	0.002999	1.20
248942_at	putative protein similar to unknown	---	AT5G45480	-1.98	-0.98	-7.46	0.000173	0.003	1.20
248920_at	magnesium chelatase subunit of pr	CHLI2	AT5G45930	-1.88	-0.91	-7.45	0.000173	0.003005	1.20
258861_at	putative helicase similar to C-termir	---	AT3G02060	2.30	1.20	7.45	0.000174	0.003005	1.19
254076_at	putative protein immunophilin FKB	---	AT4G25340	3.04	1.60	7.45	0.000174	0.003005	1.19
259945_at	unknown protein	---	AT1G71460	1.98	0.99	7.45	0.000174	0.003005	1.19
266338_at	putative ligand-gated ion channel su	GLR5	AT2G32400	2.11	1.08	7.45	0.000175	0.003009	1.19
253952_at	ubiquitin-like protein ubiquitin-like p	SUMO1	AT4G26840	1.79	0.84	7.44	0.000175	0.003011	1.19
263212_at	unknown protein similar to ribonucle	emb2004	AT1G10510	1.81	0.85	7.44	0.000175	0.003011	1.19
261124_at	hypothetical protein predicted by ge	---	AT1G04900	1.79	0.84	7.44	0.000175	0.003015	1.18
258999_at	putative D-ribulose-5-phosphate 3-e	---	AT3G01850	1.81	0.85	7.43	0.000176	0.003024	1.18
259838_at	unknown protein ;supported by full-	---	AT1G52220	-1.67	-0.74	-7.43	0.000176	0.003024	1.18
248515_at	putative protein strong similarity to u	---	AT5G50530	-1.95	-0.96	-7.43	0.000176	0.003024	1.18
246282_at	ATPase-like protein	---	AT4G36580	2.69	1.43	7.43	0.000177	0.003041	1.17
245106_at	unknown protein	---	AT2G41650	2.03	1.02	7.42	0.000178	0.003041	1.17
245981_at	putative protein ;supported by full-l	---	AT5G13100	-1.95	-0.96	-7.42	0.000178	0.003044	1.17
265484_at	unknown protein ; supported by cD	OTP51	AT2G15820	1.86	0.90	7.42	0.000178	0.003045	1.17
251593_at	DNA-directed RNA polymerase I 19	NRPA1	AT3G57660	2.71	1.44	7.42	0.000178	0.003045	1.17

266716_at	unknown protein ;supported by full-	PSI-P	AT2G46820	-1.85	-0.89	-7.42	0.000179	0.003054	1.16
261166_s_a	Expressed protein ; supported by fu	---	AT1G34570 ,	2.21	1.14	7.41	0.00018	0.003065	1.16
252923_at	kinesin like protein kinesin motor p	---	AT4G39050	-2.26	-1.18	-7.41	0.00018	0.003065	1.15
249081_at	protein phosphatase 2A 62 kDa B r	---	AT5G44090	1.79	0.84	7.40	0.000181	0.003066	1.15
248492_at	putative protein similar to unknown	---	AT5G51040	1.68	0.75	7.40	0.000181	0.003066	1.15
265594_at	50S ribosomal protein L4 ;supporte	---	AT2G20060	2.62	1.39	7.40	0.000181	0.003066	1.15
248820_at	putative protein similar to unknown	---	AT5G47060	2.23	1.16	7.40	0.000181	0.003073	1.15
259882_at	unknown protein similar to hypothet	---	AT1G76670	-1.89	-0.92	-7.40	0.000181	0.003073	1.15
246052_at	putative protein predicted proteins,	---	AT5G08310	2.11	1.08	7.40	0.000182	0.003073	1.15
256603_at	unknown protein similar to At14a pr	---	AT3G28270	-4.03	-2.01	-7.40	0.000182	0.003073	1.15
258519_at	unknown protein	---	AT3G06760	-2.11	-1.08	-7.39	0.000182	0.003076	1.14
266055_at	hypothetical protein predicted by ge	---	AT2G40650	1.98	0.98	7.39	0.000182	0.003076	1.14
248442_at	DEAD-box protein abstrakt	---	AT5G51280	2.03	1.02	7.39	0.000183	0.003077	1.14
251892_at	putative protein	WRI1	AT3G54320	2.30	1.20	7.39	0.000183	0.003077	1.14
264928_at	auxin-induced protein, putative sim	ATB2	AT1G60710	-1.81	-0.86	-7.39	0.000183	0.003084	1.14
262207_at	hypothetical protein predicted by ge	PTAC2	AT1G74850	2.12	1.09	7.39	0.000183	0.003084	1.14
253900_at	translation initiation factor ; suppor	---	AT4G27130	1.78	0.83	7.38	0.000184	0.003091	1.13
249826_at	iron superoxide dismutase 3 (gb|AA	FSD3	AT5G23310	2.30	1.20	7.38	0.000184	0.003095	1.13
247748_at	putative protein ;supported by full-l	---	AT5G58920	1.82	0.86	7.38	0.000185	0.003096	1.13
258800_at	unknown protein predicted by gens	---	AT3G04550	1.98	0.99	7.38	0.000185	0.003096	1.13
249703_at	putative protein strong similarity to u	---	AT5G35560	1.66	0.73	7.37	0.000186	0.003098	1.12
260250_at	putative phosphoribosylformylglycin	PUR4	AT1G74260	2.04	1.03	7.37	0.000186	0.003098	1.12
250896_at	putative protein ; supported by cDN	---	AT5G03560	2.34	1.23	7.37	0.000186	0.003099	1.12
256741_at	hypothetical protein predicted by ge	---	AT3G29375	2.35	1.23	7.37	0.000186	0.003101	1.12
261664_s_a	hypothetical protein contains similar	---	AT1G18320 ,	2.37	1.24	7.37	0.000186	0.003101	1.12
247866_at	endoxyloglucan transferase (gb|AA	XTR3	AT5G57550	-6.82	-2.77	-7.36	0.000187	0.003101	1.12
245337_at	Expressed protein ; supported by fu	---	AT4G16566	-1.70	-0.77	-7.36	0.000187	0.003101	1.12
252602_at	putative protein KIAA1094 protein,	---	AT3G45040	-1.93	-0.95	-7.36	0.000187	0.003101	1.11
247525_at	pseudo-response regulator 1 ; sup	TOC1	AT5G61380	1.59	0.67	7.36	0.000187	0.003101	1.11
259082_at	unknown protein similar to hypothet	---	AT3G04820	2.69	1.43	7.36	0.000187	0.003101	1.11
266732_at	unknown protein	---	AT2G03240	-2.40	-1.26	-7.36	0.000187	0.003101	1.11
249933_at	rac GTPase activating protein	---	AT5G22400	-1.69	-0.76	-7.36	0.000187	0.003101	1.11
252593_at	putative protein tRNA intron endon	ATSEN1	AT3G45590	2.07	1.05	7.36	0.000187	0.003101	1.11
261750_at	hypothetical protein contains similar	---	AT1G76120	2.04	1.03	7.36	0.000188	0.003105	1.11
266705_at	40S ribosomal protein S30 ; suppo	---	AT2G19750	2.44	1.29	7.36	0.000188	0.003105	1.11

261223_at	hypothetical protein predicted by ge	HVA22H	AT1G19950	-1.58	-0.66	-7.35	0.00019	0.003126	1.10
260491_at	hypothetical protein predicted by ge	---	AT1G51440	-2.36	-1.24	-7.35	0.00019	0.003126	1.10
260062_at	hypothetical protein contains Pfam	---	AT1G73710	2.01	1.01	7.34	0.00019	0.003133	1.10
261335_at	nodulin protein, putative similar to (	---	AT1G44800	1.59	0.66	7.34	0.000191	0.00314	1.09
251039_at	putative protein hypothetical protei	---	AT5G02020	4.16	2.06	7.33	0.000192	0.003159	1.08
267068_at	unknown protein	---	AT2G41020	2.04	1.03	7.33	0.000193	0.003161	1.08
246249_at	salt-inducible like protein	---	AT4G36680	2.08	1.06	7.32	0.000193	0.003161	1.08
267129_at	curly leaf protein (polycomb-group)	CLF	AT2G23380	-1.70	-0.77	-7.32	0.000193	0.003161	1.08
262937_at	FtsH protease, putative contains si	FTSH12	AT1G79560	1.67	0.74	7.32	0.000193	0.003161	1.08
246064_at	DNA binding protein ACBF - like D	---	AT5G19350	1.80	0.85	7.32	0.000193	0.003161	1.08
258876_at	putative ADP-ribosylation factor sim	ATARFB1C	AT3G03120	1.65	0.72	7.32	0.000194	0.003161	1.08
246622_at	protein phosphatase-2C PP2C-like	---	AT5G36250	-1.63	-0.71	-7.32	0.000194	0.003168	1.07
262706_at	putative ATP-dependent RNA helica	---	AT1G16280	2.19	1.13	7.31	0.000195	0.003182	1.07
249201_at	putative protein similar to unknown	---	AT5G42370	2.16	1.11	7.31	0.000196	0.003186	1.06
248433_at	putative protein strong similarity to u	---	AT5G51400	-1.69	-0.76	-7.31	0.000196	0.003186	1.06
247506_at	Mei2-like protein Mei2-like protein,	AML1	AT5G61960	1.63	0.71	7.31	0.000196	0.003186	1.06
264506_at	germin-like protein Identical to Aral	GLP5	AT1G09560	1.97	0.98	7.30	0.000196	0.003188	1.06
260100_at	Expressed protein ; supported by fu	BNS	AT1G73177	1.64	0.72	7.30	0.000197	0.0032	1.06
262456_at	glucose transporter almost identical	STP1	AT1G11260	-3.07	-1.62	-7.30	0.000197	0.0032	1.06
247918_at	putative protein contains similarity t	---	AT5G57610	2.10	1.07	7.29	0.000199	0.003217	1.05
261588_at	hypothetical protein predicted by ge	---	AT1G01670	1.60	0.67	7.29	0.000199	0.003217	1.05
259862_at	hypothetical protein ;supported by f	TRFL6	AT1G72650	2.04	1.03	7.28	0.0002	0.003225	1.04
261984_at	TINY-like protein similar to TINY GE	---	AT1G33760	-2.12	-1.09	-7.28	0.0002	0.003227	1.04
248299_at	unknown protein ;supported by full-	---	AT5G53080	1.80	0.85	7.27	0.000201	0.003237	1.04
265314_at	hypothetical protein predicted by ge	---	AT2G20210	1.88	0.91	7.26	0.000204	0.003269	1.02
256401_at	putative guanylate kinase similar to	---	AT3G06200	1.55	0.63	7.26	0.000204	0.003269	1.02
246555_at	putative protein 68 kDa protein - Ci	GAUT14	AT5G15470	-2.44	-1.29	-7.26	0.000204	0.003272	1.02
255611_at	hypothetical protein	---	AT4G01210	-1.68	-0.75	-7.26	0.000204	0.003274	1.02
245712_at	pullulanase-like protein (starch debr	ATLDA	AT5G04360	-1.72	-0.78	-7.25	0.000205	0.003277	1.02
267492_at	histone H1 ;supported by full-length	---	AT2G30620	2.18	1.13	7.25	0.000205	0.003277	1.02
266471_at	putative GTP-binding protein	---	AT2G31060	2.61	1.39	7.25	0.000205	0.003285	1.01
254263_at	Expressed protein ; supported by fu	---	AT4G23493	1.71	0.77	7.25	0.000206	0.003289	1.01
253605_at	putative protein probable membran	---	AT4G30990	2.23	1.16	7.24	0.000207	0.003295	1.00
261076_at	unknown protein ;supported by full-	SMO2-1	AT1G07420	-1.66	-0.73	-7.23	0.000208	0.003311	1.00
251184_at	small nuclear ribonucleoprotein-like	---	AT3G62840	1.59	0.67	7.23	0.000209	0.003312	1.00

264107_s_a	putative receptor-like protein kinase	SERK5	AT2G13790	-1.88	-0.91	-7.23	0.000209	0.003316	0.99
263934_at	hypothetical protein predicted by ge	EDA12	AT2G35950	1.59	0.67	7.22	0.00021	0.00333	0.99
245612_at	carnitine racemase like protein	HCD1	AT4G14440	-6.40	-2.68	-7.22	0.000211	0.00333	0.99
265330_at	unknown protein	GUT15	---	1.92	0.94	7.22	0.000211	0.00333	0.99
246850_at	Lon protease homolog 2 precursor	LON1	AT5G26860	3.52	1.82	7.22	0.000211	0.00333	0.98
250822_at	unknown protein	---	AT5G05230	1.66	0.73	7.22	0.000211	0.00333	0.98
245422_at	putative protein	---	AT4G17470	-6.78	-2.76	-7.22	0.000211	0.003333	0.98
258755_at	hypothetical protein identical to hyp	---	AT3G11945	-1.93	-0.95	-7.21	0.000212	0.003335	0.98
253238_at	putative protein (fragment)	---	AT4G34480	-1.95	-0.97	-7.21	0.000213	0.003346	0.97
246302_at	Ca2+/H+-exchanging protein-like A	CAX3	AT3G51860	6.33	2.66	7.20	0.000213	0.003346	0.97
262653_at	dioxygenase-like protein very simila	---	AT1G14130	1.61	0.69	7.20	0.000213	0.003346	0.97
250512_at	Expressed protein ; supported by fu	---	AT5G09995	-1.93	-0.95	-7.20	0.000213	0.003346	0.97
251291_at	putative protein auxin-induced prot	---	AT3G61900	1.73	0.79	7.20	0.000214	0.003355	0.97
256436_at	hypothetical protein predicted by ge	---	AT3G11150	1.80	0.85	7.20	0.000214	0.003355	0.97
266718_at	putative zinc transporter ;supported	ZAT	AT2G46800	-1.80	-0.85	-7.19	0.000215	0.003361	0.96
263117_at	putative lipoamide dehydrogenase	---	AT1G03040	1.61	0.69	7.19	0.000215	0.003361	0.96
249403_at	putative protein intracellular protein	---	AT5G40270	2.09	1.06	7.19	0.000216	0.003363	0.96
250733_at	2-cys peroxiredoxin-like protein ;su	2-Cys Prx E	AT5G06290	-2.00	-1.00	-7.19	0.000216	0.003363	0.96
263636_at	unknown protein ; supported by ful	---	AT2G11910	1.87	0.90	7.19	0.000216	0.003363	0.96
256991_at	unknown protein	---	AT3G28600	-1.64	-0.72	-7.18	0.000218	0.003389	0.95
255631_at	hypothetical protein	BSK3	AT4G00710	-1.60	-0.68	-7.17	0.00022	0.003402	0.94
259505_at	hypothetical protein contains similar	---	AT1G15810	2.13	1.09	7.17	0.00022	0.003402	0.94
262056_at	Expressed protein ; supported by fu	---	AT1G80245	1.83	0.87	7.16	0.000221	0.003421	0.93
249089_at	ATPase, calcium-transporting	---	AT5G44240	1.56	0.64	7.16	0.000221	0.003421	0.93
254848_at	putative protein hypothetical protei	PGRL1B	AT4G11960	1.78	0.83	7.16	0.000222	0.003421	0.93
254951_at	putative protein various kinesin light	---	AT4G10840	-1.65	-0.72	-7.16	0.000222	0.003421	0.93
260805_at	glutathione transferase, putative si	ATGSTU23	AT1G78320	1.62	0.70	7.15	0.000222	0.003423	0.93
249307_s_a	DNA excision repair cross-complem	XPB1	AT5G41360	1.78	0.83	7.15	0.000223	0.003425	0.93
254418_at	glucose transporter glucose transp	---	AT4G21480	-1.79	-0.84	-7.15	0.000223	0.003429	0.92
261544_at	unknown protein	---	AT1G63540	2.17	1.12	7.15	0.000223	0.00343	0.92
258161_at	hypothetical protein predicted by ge	---	AT3G17930	1.88	0.91	7.15	0.000224	0.00343	0.92
249979_s_a	putative protein	---	AT5G18860	-3.98	-1.99	-7.15	0.000224	0.00343	0.92
250883_at	putative protein predicted proteins,	---	AT5G03970	2.12	1.09	7.14	0.000224	0.003435	0.92
245893_at	hypothetical protein ;supported by	---	AT5G09270	1.74	0.80	7.14	0.000225	0.00344	0.92
250079_at	putative protein similar to unknown	---	AT5G16650	-1.95	-0.96	-7.13	0.000227	0.003462	0.91

257265_at	PHD-finger protein, putative contai	---	AT3G14980	-1.87	-0.90	-7.13	0.000227	0.003462	0.90
251786_at	phosphatase - like protein DUAL S	MKP1	AT3G55270	1.68	0.74	7.13	0.000227	0.003462	0.90
265425_at	hypothetical protein	GCL2	AT2G20770	-2.04	-1.03	-7.13	0.000227	0.003462	0.90
249901_at	histone deacetylase-like protein no	HD2B	AT5G22650	2.26	1.17	7.12	0.000228	0.003467	0.90
266246_at	putative cytochrome P450	CYP94C1	AT2G27690	-5.79	-2.53	-7.12	0.000229	0.003473	0.90
259192_at	unknown protein ;supported by full-	---	AT3G01740	1.97	0.98	7.12	0.000229	0.003477	0.89
250139_at	putative protein de novo DNA meth	DRM2	AT5G14620	1.57	0.65	7.12	0.000229	0.003479	0.89
247800_at	Expressed protein ; supported by fu	---	AT5G58570	9.77	3.29	7.11	0.000231	0.003495	0.89
248956_at	unknown protein	---	AT5G45610	1.80	0.85	7.11	0.000231	0.003496	0.89
248093_at	putative protein similar to unknown	---	AT5G55210	1.92	0.94	7.11	0.000231	0.003496	0.88
262891_at	ent-kaurene synthase, putative sim	GA2	AT1G79460	-2.53	-1.34	-7.11	0.000232	0.003499	0.88
249886_at	unknown protein ; supported by cD	---	AT5G22320	2.39	1.26	7.11	0.000232	0.0035	0.88
252246_at	putative protein various predicted p	---	AT3G49725	1.82	0.87	7.10	0.000232	0.0035	0.88
261112_at	unknown protein	---	AT1G75420	1.94	0.95	7.10	0.000232	0.003505	0.88
267300_at	putative glucosyltransferase	---	AT2G30140	1.68	0.75	7.10	0.000233	0.003509	0.88
263875_at	unknown protein ;supported by full-	טפס-02	AT2G21970	2.43	1.28	7.10	0.000234	0.003513	0.87
246906_at	Expressed protein ; supported by fu	---	AT5G25475	1.73	0.79	7.09	0.000234	0.003513	0.87
260413_at	hypothetical protein predicted by ge	---	AT1G69800	2.18	1.13	7.09	0.000234	0.003515	0.87
256240_at	unknown protein ; supported by cDI	atnudt16	AT3G12600	-1.79	-0.84	-7.09	0.000234	0.003515	0.87
246958_at	putative protein similar to unknown	---	AT5G24690	2.18	1.12	7.09	0.000235	0.003517	0.87
253399_at	putative poly(A) polymerase polynu	nPAP	AT4G32850	1.66	0.73	7.09	0.000235	0.003517	0.87
246453_at	syntaxin homologue ;supported by	SYP21	AT5G16830	-1.85	-0.88	-7.09	0.000236	0.00352	0.86
260989_at	unknown protein ; supported by cDI	---	AT1G53450	2.11	1.08	7.09	0.000236	0.00352	0.86
252688_at	putative protein	---	AT3G44020	1.87	0.90	7.08	0.000236	0.00352	0.86
263142_at	unknown protein	---	AT1G65230	-1.85	-0.89	-7.08	0.000236	0.003521	0.86
252319_at	putative protein putative protein - A	---	AT3G48710	2.22	1.15	7.08	0.000236	0.003521	0.86
246614_at	serine/threonine protein kinase SOS	SOS2	AT5G35410	1.72	0.78	7.08	0.000237	0.003524	0.86
253703_at	putative protein origin recognition c	ORC5	AT4G29910	1.84	0.88	7.07	0.000238	0.003543	0.85
247668_at	pseudo-response regulator - like ps	APRR3	AT5G60100	1.59	0.67	7.07	0.000238	0.003543	0.85
250382_at	putative protein rjs protein, Mus mu	---	AT5G11580	1.56	0.64	7.07	0.000239	0.003546	0.85
259253_at	hypothetical protein predicted by ge	---	AT3G07640	1.56	0.64	7.07	0.00024	0.003554	0.85
255378_at	putative glucan synthase compone	ATGSL05	AT4G03550	-1.69	-0.76	-7.07	0.00024	0.003556	0.84
256528_at	C2H2-type zinc finger protein, puta	ZFP4	AT1G66140	2.37	1.24	7.06	0.00024	0.003556	0.84
260800_at	unknown protein	TSD2	AT1G78240	-2.26	-1.18	-7.06	0.00024	0.003556	0.84
249938_at	Ruv DNA-helicase-like protein ;sup	RIN1	AT5G22330	1.69	0.76	7.06	0.000241	0.003565	0.84

255799_at	Deetiolated1 (DET1) light signal tra	DET1	AT4G10180	1.83	0.87	7.06	0.000241	0.003565	0.84
253549_at	Expressed protein ; supported by fu	NFD1	AT4G30930	2.55	1.35	7.06	0.000242	0.003565	0.84
262610_at	putative mitochondrial uncoupling p	---	AT1G14140	-1.55	-0.63	-7.06	0.000242	0.003565	0.84
267545_at	unknown protein	GRP23	AT2G32690	-2.25	-1.17	-7.06	0.000242	0.003565	0.84
248065_at	putative protein similar to unknown	---	AT5G55580	2.15	1.10	7.06	0.000242	0.003565	0.84
256383_at	hypothetical protein ; supported by	---	AT1G66820	1.92	0.94	7.05	0.000242	0.003565	0.83
260343_at	fructokinase (Frk1), putative similar	---	AT1G69200	2.24	1.16	7.05	0.000242	0.003569	0.83
246083_at	zinc finger protein - like probable zi	AL5	AT5G20510	1.67	0.74	7.05	0.000243	0.00357	0.83
260774_at	serine-threonine protein kinase, pu	---	AT1G78290	4.67	2.22	7.05	0.000243	0.00357	0.83
260936_at	hypothetical protein predicted by ge	---	AT1G45150	1.81	0.85	7.05	0.000244	0.003577	0.83
245152_at	putative mitochondrial carrier protei	---	AT2G47490	-1.77	-0.82	-7.04	0.000244	0.003579	0.83
251611_at	putative protein several hypothetica	---	AT3G57940	2.21	1.15	7.04	0.000245	0.003586	0.82
249125_at	1-aminocyclopropane-1-carboxylate	---	AT5G43450	2.47	1.30	7.04	0.000245	0.003586	0.82
262756_at	putative transport protein may be a	אוק-06	AT1G16370	-7.36	-2.88	-7.04	0.000245	0.003586	0.82
258559_at	putative transcriptional regulator sin	ATCHR12	AT3G06010	2.01	1.01	7.04	0.000246	0.003588	0.82
264372_at	lactoylglutathione lyase-like protein	ATGLX1	AT1G11840	-2.36	-1.24	-7.03	0.000246	0.003597	0.82
249575_at	low-molecular-weight heat shock p	---	AT5G37670	2.35	1.23	7.03	0.000247	0.003598	0.81
261931_at	alcohol dehydrogenase ADH, putat	---	AT1G22430	-1.63	-0.71	-7.03	0.000247	0.003605	0.81
248177_at	putative protein strong similarity to u	---	AT5G54630	-1.68	-0.75	-7.03	0.000248	0.00361	0.81
251967_at	putative protein DMR-N9 PROTEIN	---	AT3G53390	1.65	0.72	7.02	0.000248	0.003613	0.81
257700_at	unknown protein similar to unknown	ALIS1	AT3G12740	1.93	0.95	7.02	0.000248	0.003613	0.81
249975_s_a	ribosomal protein L33 - like ribosor	---	AT3G06320	2.70	1.43	7.02	0.000249	0.003614	0.81
253462_at	synaptobrevin-like protein synaptol	ATVAMP71	AT4G32150	1.95	0.96	7.02	0.00025	0.003625	0.80
247586_at	mipC protein - like (aquaporin) mip	PIP2;4	AT5G60660	-1.76	-0.81	-7.02	0.00025	0.003628	0.80
257175_s_a	cyclopropane-fatty-acyl-phospholip	---	AT3G23470	-1.81	-0.85	-7.02	0.00025	0.003628	0.80
253897_at	putative protein	---	AT4G27120	1.69	0.75	7.02	0.00025	0.003628	0.80
250825_at	putative protein similar to unknown	---	AT5G05210	1.70	0.77	7.01	0.00025	0.003629	0.80
265909_at	unknown protein ;supported by full-	---	AT2G25720	1.83	0.87	7.01	0.000251	0.003629	0.80
260938_at	hypothetical protein contains similar	---	AT1G45170	1.96	0.97	7.01	0.000251	0.003629	0.80
261909_at	hypothetical protein predicted by ge	SAR3	AT1G80680	1.99	1.00	7.01	0.000251	0.003629	0.80
247464_at	putative protein various predicted p	IQD23	AT5G62070	-2.61	-1.38	-7.01	0.000251	0.003635	0.79
262592_at	unknown protein ESTs gb|H37295 a	---	AT1G15400	2.21	1.15	7.00	0.000253	0.003649	0.79
254102_at	acyl carrier - like protein acyl carrie	ACP4	AT4G25050	-1.94	-0.95	-7.00	0.000253	0.003649	0.79
245939_at	oxoglutarate/malate translocator-lik	---	AT5G19760	-1.76	-0.82	-6.99	0.000255	0.003665	0.78
255650_s_a	hypothetical protein	CIP4.1	AT4G00930	1.93	0.95	6.99	0.000255	0.003665	0.78

250504_at	putative protein similar to unknown	---	AT5G09840	2.00	1.00	6.99	0.000255	0.003665	0.78
262493_at	auxilin-like protein similar to GB:CA	---	AT1G21660	-1.69	-0.76	-6.99	0.000256	0.003676	0.77
261259_at	unknown protein	---	AT1G26660	1.80	0.85	6.99	0.000257	0.003676	0.77
264335_s_a	hypothetical protein contains simila	UPL1	AT1G55860	1.57	0.65	6.99	0.000257	0.003676	0.77
260157_at	unknown protein ; supported by cD	---	AT1G52930	3.46	1.79	6.98	0.000257	0.003681	0.77
267309_at	Expressed protein ; supported by fu	---	AT2G19385	2.29	1.19	6.98	0.000258	0.003699	0.76
250970_at	putative protein ; supported by cDN	---	AT5G02770	1.66	0.73	6.98	0.000259	0.0037	0.76
265270_at	putative ribose phosphate pyrophos	---	AT2G42910	1.71	0.77	6.97	0.000259	0.003701	0.76
265172_at	putative spermidine synthase	SPDS1	AT1G23820	1.60	0.68	6.97	0.000259	0.003701	0.76
262700_at	unknown protein	---	AT1G76020	-2.05	-1.03	-6.97	0.00026	0.003701	0.76
252479_at	small zinc finger-like protein TIM9 ;	TIM9	AT3G46560	2.25	1.17	6.97	0.00026	0.003705	0.76
262800_at	putative ATP-dependent RNA helic	emb1507	AT1G20960	1.79	0.84	6.97	0.000261	0.00371	0.75
261197_at	putative calcium-binding protein, ca	GAPA-2	AT1G12900	-1.85	-0.89	-6.97	0.000261	0.003712	0.75
266782_at	putative ligand-gated ion channel p	ATGLR2.7	AT2G29120	-1.69	-0.76	-6.96	0.000262	0.003714	0.75
260871_at	hypothetical protein predicted by ge	---	AT1G29040	-1.57	-0.65	-6.96	0.000262	0.003714	0.75
246494_at	putative protein putative cellulose s	CSLA11	AT5G16190	-1.88	-0.91	-6.95	0.000264	0.003733	0.74
256792_at	hypothetical protein predicted by ge	---	AT3G22150	3.43	1.78	6.95	0.000264	0.003735	0.74
263766_at	hypothetical protein predicted by ge	---	AT2G21440	2.09	1.06	6.95	0.000264	0.003735	0.74
255279_at	putative thioredoxin ; supported by	---	AT4G04950	2.42	1.28	6.95	0.000265	0.003737	0.74
248678_at	U6 snRNA-associated Sm-like prote	SAD1	AT5G48870	1.81	0.86	6.95	0.000265	0.003737	0.74
246295_at	calmodulin-binding protein ; suppo	CIP111	AT3G56690	2.08	1.06	6.95	0.000265	0.00374	0.73
247734_at	putative protein predicted protein, A	---	AT5G59400	1.82	0.86	6.95	0.000266	0.003745	0.73
250669_at	polygalacturonase inhibiting protein	PGIP2	AT5G06870	-5.20	-2.38	-6.94	0.000266	0.003746	0.73
255614_at	putative myb-related DNA-binding p	---	AT4G01280	1.66	0.74	6.94	0.000266	0.003749	0.73
245761_at	hypothetical protein predicted by ge	---	AT1G66890	1.76	0.81	6.94	0.000267	0.003757	0.73
251980_at	putative protein endopeptidase Clp	---	AT3G53270	1.58	0.66	6.93	0.000269	0.00378	0.72
265521_at	hypothetical protein predicted by ge	ORG4	AT2G06010	1.93	0.95	6.93	0.000269	0.003781	0.72
251771_at	putative protein cellulose synthase	CSLA14	AT3G56000	-1.82	-0.86	-6.93	0.00027	0.003793	0.72
252328_at	Expressed protein ; supported by cl	---	AT3G48570	-1.70	-0.77	-6.92	0.000271	0.003803	0.71
256406_at	unknown protein	---	AT1G50710	-1.83	-0.87	-6.92	0.000272	0.003805	0.71
266365_at	ferredoxin-dependent glutamate sy	GLU2	AT2G41220	2.15	1.10	6.92	0.000273	0.003814	0.71
250969_at	putative protein ;supported by full-l	---	AT5G02740	1.68	0.74	6.91	0.000273	0.003818	0.70
250711_at	cell division related protein-like	---	AT5G06110	1.71	0.78	6.91	0.000274	0.003822	0.70
250454_at	unknown protein ; supported by ful	---	AT5G09830	1.52	0.60	6.91	0.000274	0.003829	0.70
266482_at	putative small nuclear ribonucleopro	---	AT2G47640	2.03	1.02	6.91	0.000275	0.003829	0.70

248605_at	putative protein similar to unknown	---	AT5G49410	2.45	1.30	6.91	0.000275	0.003829	0.70
254879_at	hypothetical protein	---	AT4G11670	1.76	0.82	6.90	0.000275	0.003832	0.70
251853_at	putative protein various Arabidopsis	---	AT3G54790	1.66	0.73	6.90	0.000276	0.003842	0.69
246131_at	MOLYBDOPTERIN BIOSYNTHESI	B73	AT5G20990	1.60	0.68	6.90	0.000276	0.003842	0.69
250613_at	putative protein SF16 protein, polle	IQD24	AT5G07240	-1.96	-0.97	-6.90	0.000277	0.003842	0.69
254624_at	hypothetical protein ;supported by	---	AT4G18580	1.73	0.79	6.90	0.000277	0.003843	0.69
248815_at	putative protein contains similarity t	---	AT5G46920	2.26	1.18	6.89	0.000278	0.003851	0.69
255619_s_a	putative CHP-rich zinc finger protei	---	AT4G01350	-2.11	-1.08	-6.89	0.000278	0.003851	0.69
264360_at	putative isoamylase Similar to gi|16	DBE1	AT1G03310	1.98	0.99	6.89	0.000279	0.003863	0.68
257587_at	3'-5' exonuclease, putative contain	---	AT1G56310	1.66	0.73	6.89	0.00028	0.003867	0.68
259666_at	unknown protein ; supported by cDI	SR33	AT1G55310	1.72	0.78	6.88	0.00028	0.00387	0.68
255681_at	predicted protein of unknown functi	DGD2	AT4G00550	1.91	0.93	6.88	0.00028	0.00387	0.68
247497_at	Peter Pan - like protein Peter Pan,	PPAN	AT5G61770	2.31	1.21	6.88	0.00028	0.00387	0.68
247891_at	GTP binding protein-like	---	AT5G57960	1.72	0.78	6.88	0.000281	0.003872	0.67
245161_at	putative myrosinase binding protein	NSP2	AT2G33070	-1.98	-0.98	-6.88	0.000281	0.003874	0.67
262162_at	unknown protein ;supported by full-	---	AT1G78020	2.09	1.07	6.88	0.000282	0.003881	0.67
259908_at	RNA polymerase subunit identical t	ATRPAC42	AT1G60850	2.17	1.11	6.87	0.000282	0.003881	0.67
256889_at	PHD-finger protein, putative contai	ING1	AT3G24010	1.57	0.65	6.87	0.000282	0.003881	0.67
264211_at	putative gigantea protein strong sim	GI	AT1G22770	1.59	0.66	6.87	0.000283	0.003881	0.67
255594_at	putative ABC transporter	ATABC1	AT4G01660	-1.69	-0.76	-6.87	0.000283	0.003881	0.66
245130_at	putative receptor-like protein kinase	---	AT2G45340	1.60	0.68	6.87	0.000283	0.003881	0.66
250119_at	putative protein similar to unknown	---	AT5G16470	1.65	0.72	6.87	0.000283	0.003881	0.66
258193_at	putative pectinesterase similar to pe	PME31	AT3G29090	1.62	0.69	6.87	0.000284	0.003882	0.66
250207_at	chalcone synthase (naringenin-chal	TT4	AT5G13930	-5.11	-2.35	-6.87	0.000284	0.003882	0.66
256860_at	hypothetical protein similar to acety	---	AT3G23840	-1.76	-0.81	-6.87	0.000284	0.003889	0.66
250477_at	putative protein CIS,CIS-MUCONA	---	AT5G10190	1.60	0.68	6.86	0.000285	0.003901	0.66
257932_at	unknown protein contains Pfam pro	HCF107	AT3G17040	-1.87	-0.91	-6.86	0.000286	0.003901	0.65
266521_at	unknown protein ; supported by cD	---	AT2G24020	-1.62	-0.69	-6.86	0.000286	0.003901	0.65
248508_at	histone acetyltransferase ;supporte	ELO3	AT5G50320	2.08	1.06	6.86	0.000286	0.003901	0.65
263082_at	putative nucleotide-binding protein	---	AT2G27200	1.55	0.63	6.86	0.000286	0.003901	0.65
255542_at	hypothetical protein	---	AT4G01860	1.90	0.93	6.86	0.000287	0.003903	0.65
267602_at	unknown protein ; supported by cD	---	AT2G32970	-2.11	-1.08	-6.85	0.000289	0.003921	0.64
262966_at	ER lumen protein retaining recepto	---	AT1G75760	-1.71	-0.78	-6.84	0.00029	0.003936	0.64
246070_at	ribosomal protein L7Ae-like NHP2/	---	AT5G20160	2.34	1.22	6.84	0.00029	0.003937	0.64
251583_at	putative protein several hypothetica	---	AT3G58590	2.23	1.16	6.84	0.000291	0.00394	0.63

254732_at	hypothetical protein	---	AT4G13750	2.23	1.16	6.84	0.000291	0.003942	0.63
247834_at	cinnamoyl-CoA reductase - like pro	---	AT5G58490	1.61	0.69	6.83	0.000292	0.003952	0.63
265510_at	putative microtubule-associated pro	ATG8D	AT2G05630	1.72	0.78	6.83	0.000293	0.003952	0.63
250141_at	protein kinase MSK-3 - like protein	SK13	AT5G14640	1.93	0.95	6.83	0.000294	0.003965	0.62
AFFX-Athal-	Arabidopsis thaliana /REF=M64116	GAPC1	---	2.11	1.08	6.82	0.000296	0.003977	0.62
245377_at	hypothetical protein	---	AT4G17700	-1.73	-0.79	-6.82	0.000296	0.003977	0.62
263939_at	hypothetical protein predicted by ge	ATTIM44-2	AT2G36070	1.71	0.77	6.82	0.000296	0.003977	0.61
250589_at	transcription factor (gb|AAD53097.1	MYB76	AT5G07700	-4.54	-2.18	-6.82	0.000296	0.003977	0.61
255451_at	putative protein similar to T5J8.17	---	AT4G02860	1.90	0.92	6.82	0.000296	0.003977	0.61
258104_at	hypothetical protein predicted by ge	---	AT3G23620	2.44	1.29	6.82	0.000298	0.003987	0.61
261919_at	type 2 peroxiredoxin, putative simil	TPX1	AT1G65980	1.88	0.91	6.81	0.000298	0.003987	0.61
251368_at	putative protein hypothetical protei	---	AT3G61380	-2.53	-1.34	-6.81	0.000298	0.003989	0.61
262433_s_a	DNA binding protein, putative simil	ATRBP47C	AT1G47490	1.63	0.71	6.81	0.0003	0.004003	0.60
258094_at	putative cytochrome P450 similar to	CYP72A15	AT3G14690	1.78	0.83	6.80	0.000303	0.004033	0.59
247696_at	MYB27 protein - like MYB27 protei	MYB59	AT5G59780	3.55	1.83	6.80	0.000303	0.004034	0.59
260290_at	hypothetical protein predicted by g	---	AT1G80510	-1.77	-0.82	-6.79	0.000304	0.004045	0.59
258390_at	unknown protein similar to leucine-r	---	AT3G15410	-1.69	-0.76	-6.79	0.000305	0.004048	0.58
263298_at	unknown protein ; supported by cD	TIC21	AT2G15290	-1.60	-0.68	-6.79	0.000305	0.004048	0.58
256455_at	unknown protein ;supported by full-	---	AT1G75190	-1.70	-0.77	-6.78	0.000306	0.004051	0.58
251760_at	Expressed protein ; supported by fu	---	AT3G55605	2.73	1.45	6.78	0.000306	0.004051	0.58
248102_at	putative protein contains similarity t	---	AT5G55140	2.14	1.10	6.78	0.000306	0.004053	0.58
259420_at	hypothetical protein similar to putati	---	AT1G13900	-1.49	-0.57	-6.78	0.000306	0.004053	0.58
266441_at	hypothetical protein predicted by ge	---	AT2G43250	1.70	0.77	6.78	0.000306	0.004053	0.58
259318_at	unknown protein similar to HYP1 G	HYP1	AT3G01100	1.99	1.00	6.78	0.000307	0.004055	0.58
265118_at	beta-fructosidase nearly identical to	---	AT1G62660	-2.50	-1.32	-6.78	0.000307	0.004056	0.58
245122_at	putative dimethyladenosine transfer	---	AT2G47420	1.81	0.86	6.78	0.000308	0.004061	0.57
267419_at	putative thioredoxin	ATO1	AT2G35010	1.74	0.80	6.77	0.000309	0.004066	0.57
266351_at	unknown protein ;supported by full-	---	AT2G01490	1.85	0.89	6.77	0.00031	0.004066	0.57
264467_at	unknown protein similar to EST gb|A	---	AT1G10140	1.80	0.85	6.77	0.00031	0.004066	0.57
249991_at	zinc finger -like protein zinc finger p	---	AT5G18550	-1.70	-0.76	-6.77	0.00031	0.004066	0.57
263180_at	unknown protein ;supported by full-	URH2	AT1G05620	-1.77	-0.82	-6.76	0.000311	0.004078	0.56
253096_at	cytochrome P450-like protein	CYP81D4	AT4G37330	1.82	0.87	6.76	0.000312	0.004083	0.56
256269_at	transcription factor HBP-1B-like nea	TGA6	AT3G12250	1.63	0.70	6.76	0.000312	0.004085	0.56
262708_at	UV-sensitive rad23, putative simila	---	AT1G16190	-1.57	-0.65	-6.76	0.000313	0.004092	0.56
261833_at	ATP citrate-lyase, putative similar t	ACLA-1	AT1G10670	-1.71	-0.77	-6.76	0.000314	0.004095	0.55

265453_at	putative cellular apoptosis susceptil	---	AT2G46520	-1.92	-0.94	-6.75	0.000314	0.004095	0.55
247027_at	subtilisin-like serine protease conta	---	AT5G67090	-1.57	-0.65	-6.75	0.000314	0.004098	0.55
251772_at	cyclophilin-like protein ROC7, cyclo	---	AT3G55920	-1.94	-0.95	-6.75	0.000315	0.004105	0.55
251242_at	ARGININE/SERINE-RICH SPLICIN	RSP31	AT3G61860	1.61	0.69	6.75	0.000315	0.004105	0.55
261322_at	hypothetical protein predicted by ge	ATPUP11	AT1G44750	1.81	0.86	6.75	0.000315	0.004105	0.55
266371_at	calmodulin-like protein identical to C	---	AT2G41410	3.06	1.61	6.75	0.000316	0.00411	0.54
262637_at	oxidoreductase, putative contains F	---	AT1G06640	-12.47	-3.64	-6.74	0.000317	0.004112	0.54
262351_at	beta-galactosidase (BGAL), putativ	BGAL17	AT1G72990	1.56	0.64	6.74	0.000318	0.004124	0.54
253410_at	putative protein various predicted p	---	AT4G32970	2.03	1.02	6.74	0.000318	0.004124	0.54
260873_at	hypothetical protein predicted by ge	---	AT1G21580	-1.55	-0.63	-6.74	0.000318	0.004127	0.54
262345_at	hypothetical protein similar to hypot	---	AT1G64180	1.79	0.84	6.74	0.000319	0.004129	0.54
263870_at	unknown protein	RKP	AT2G22010	1.78	0.83	6.74	0.000319	0.004129	0.53
253912_at	putative protein met-10+ protein, N	---	AT4G27340	1.89	0.92	6.73	0.00032	0.004139	0.53
246076_at	sucrose-phosphate synthase-like p	ATSPS1F	AT5G20280	1.75	0.81	6.73	0.000321	0.004151	0.53
266271_at	putative glutathione S-transferase	ATGSTU6	AT2G29440	-6.38	-2.67	-6.73	0.000322	0.004154	0.52
255317_at	putative vesicle transfer ATPase	---	AT4G04180	2.61	1.39	6.73	0.000322	0.004155	0.52
258673_at	unknown protein	---	AT3G08620	2.18	1.13	6.72	0.000323	0.004162	0.52
266461_at	glutathione S-transferase (GST6) i	ATGSTF8	AT2G47730	-2.61	-1.38	-6.72	0.000323	0.004162	0.52
252946_at	Expressed protein ; supported by fu	---	AT4G39235	2.38	1.25	6.72	0.000323	0.004162	0.52
249325_at	urophorphyrin III methylase (gb|AAI	UPM1	AT5G40850	2.00	1.00	6.72	0.000324	0.004162	0.52
251025_at	putative protein CND41, chloroplas	PCS1	AT5G02190	-2.00	-1.00	-6.72	0.000324	0.004163	0.52
258609_at	unknown protein ;supported by full-	---	AT3G02910	1.84	0.88	6.71	0.000327	0.004201	0.51
265187_at	putative ADP-ribosylation factor ; s	---	AT1G23490	-1.49	-0.57	-6.71	0.000327	0.004201	0.51
259034_at	putative pectinacetylesterase simila	---	AT3G09410	2.34	1.22	6.70	0.000329	0.004209	0.50
258766_at	galactokinase-like protein similar to	---	AT3G10700	-1.63	-0.70	-6.70	0.00033	0.004214	0.50
266414_at	mevalonate diphosphate decarboxy	MVD1	AT2G38700	-1.76	-0.82	-6.70	0.00033	0.004216	0.50
266977_at	putative phospholipase	---	AT2G39420	-3.79	-1.92	-6.70	0.000331	0.004216	0.50
255764_at	unknown protein	HCF173	AT1G16720	-1.96	-0.97	-6.69	0.000331	0.004217	0.49
250013_at	putative protein similar to unknown	---	AT5G18040	1.63	0.71	6.69	0.000331	0.004217	0.49
255535_at	putative RNaseP-associated protei	---	AT4G01790	2.18	1.12	6.69	0.000332	0.004217	0.49
246861_at	putative protein phytochrome-asso	IAA28	AT5G25890	1.73	0.79	6.69	0.000332	0.004217	0.49
253005_at	hypothetical protein ;supported by	---	AT4G38100	2.33	1.22	6.69	0.000332	0.004217	0.49
257227_at	monodehydroascorbate reductase s	MDAR4	AT3G27820	1.74	0.80	6.69	0.000332	0.004219	0.49
249427_at	40S ribosomal protein S9-like 40S	---	AT5G39850	4.20	2.07	6.69	0.000333	0.004224	0.49
265593_s_a	unknown protein ;supported by full-	LCV1	AT2G20120	-1.62	-0.69	-6.68	0.000334	0.004231	0.48

247972_at	histone acetyltransferase HAT B	HAG2	AT5G56740	2.12	1.08	6.68	0.000334	0.004231	0.48
266641_at	Expressed protein ; supported by fu	---	AT2G35605	1.73	0.79	6.68	0.000334	0.004231	0.48
256262_at	unknown protein	---	AT3G12150	-1.95	-0.97	-6.68	0.000334	0.004231	0.48
250358_at	putative protein gibberellin-respons	AGP15	AT5G11740	-2.02	-1.02	-6.68	0.000335	0.004232	0.48
262837_at	dynamin, putative similar to dynam	ADL1C	AT1G14830	-1.75	-0.81	-6.68	0.000335	0.004236	0.48
250718_at	unknown protein	emb2735	AT5G06240	1.69	0.76	6.68	0.000335	0.004237	0.48
265366_at	putative N-acetylglucosaminyltransf	---	AT2G13290	1.75	0.81	6.68	0.000336	0.00424	0.48
248418_at	cleavage and polyadenylation spec	CPSF160	AT5G51660	1.76	0.81	6.67	0.000337	0.004251	0.47
267174_at	60S ribosomal protein L36 ;suppor	---	AT2G37600	2.62	1.39	6.67	0.000337	0.004253	0.47
246708_at	putative protein various predicted p	---	AT5G28150	-1.98	-0.98	-6.67	0.000338	0.004257	0.47
257783_at	unknown protein	---	AT3G26950	-1.69	-0.76	-6.67	0.000338	0.004257	0.47
250704_at	Expressed protein ; supported by fu	---	AT5G06265	1.95	0.96	6.67	0.000339	0.00426	0.47
250647_at	putative protein contains similarity t	---	AT5G06770	-1.72	-0.79	-6.67	0.000339	0.00426	0.47
250246_at	polyamine oxidase	ATPAO1	AT5G13700	-1.55	-0.63	-6.67	0.000339	0.00426	0.47
252114_at	mucin -like protein hemomucin, Dro	---	AT3G51450	-4.99	-2.32	-6.66	0.00034	0.00427	0.47
247446_at	unknown protein ; supported by cD	---	AT5G62650	1.59	0.66	6.66	0.00034	0.00427	0.46
260775_at	14-3-3 protein GF14omega (grf2) id	GRF2	AT1G78300	-1.98	-0.98	-6.66	0.000341	0.004274	0.46
256107_at	hypothetical protein predicted by ge	---	AT1G16830	1.60	0.67	6.66	0.000342	0.004288	0.46
260058_at	unknown protein ; supported by cD	---	AT1G78100	-1.78	-0.84	-6.66	0.000342	0.004288	0.46
259444_at	hypothetical protein predicted by ge	---	AT1G02370	3.39	1.76	6.66	0.000342	0.004288	0.46
264675_at	putative phosphoribosylglycinamide	---	AT1G09830	1.64	0.71	6.65	0.000343	0.004291	0.46
261334_at	unknown protein	---	AT1G44960	1.74	0.80	6.65	0.000343	0.004291	0.46
252986_at	putative protein T19C21.18 Arabido	---	AT4G38380	1.50	0.58	6.65	0.000345	0.004308	0.45
250784_at	unknown protein ; supported by cDI	---	AT5G05480	1.91	0.94	6.65	0.000345	0.004315	0.45
258868_at	putative exportin1 (XPO1) protein s	XPO1B	AT3G03110	1.78	0.83	6.64	0.000347	0.004333	0.44
256145_at	lipid transfer protein, putative simila	---	AT1G48750	-3.64	-1.86	-6.64	0.000347	0.004333	0.44
265596_at	hypothetical protein predicted by ge	CAF1	AT2G20020	3.05	1.61	6.64	0.000348	0.004333	0.44
259690_at	replication factor, putative similar to	---	AT1G63160	2.34	1.22	6.64	0.000348	0.004333	0.44
253775_at	putative protein T01B08.14 gene, E	---	AT4G28440	1.57	0.65	6.64	0.000348	0.004333	0.44
261700_at	hypothetical protein similar to hypot	---	AT1G32690	1.97	0.98	6.64	0.000348	0.004333	0.44
266774_at	hypothetical protein predicted by ge	ATRBL1	AT2G29050	-1.96	-0.97	-6.64	0.000349	0.004336	0.44
250662_at	steroid sulfotransferase-like protein	ST2A	AT5G07010	-3.37	-1.75	-6.63	0.000349	0.004339	0.44
261018_at	unknown protein contains similarity	---	AT1G26550	1.49	0.58	6.63	0.000349	0.004339	0.44
256255_at	MYB-family transcription factor, pu	---	AT3G11280	-1.52	-0.60	-6.63	0.00035	0.004342	0.43
266704_at	putative N-acetyl-gamma-glutamyl-p	---	AT2G19940	-1.90	-0.93	-6.63	0.00035	0.004342	0.43

251733_at	copper homeostasis factor ;suppor	CCH	AT3G56240	1.77	0.82	6.63	0.00035	0.004342	0.43
246829_at	putative protein pyruvate water diki	ATGWD3	AT5G26570	1.62	0.69	6.63	0.000351	0.004351	0.43
251432_at	putative protein leucine zipper-EF-l	---	AT3G59820	2.25	1.17	6.63	0.000351	0.004352	0.43
246593_at	putative protein predicted protein, A	---	AT5G14790	-2.12	-1.09	-6.62	0.000352	0.00436	0.43
256852_at	unknown protein contains Pfam pro	ATRANGAI	AT3G18610	2.12	1.09	6.62	0.000353	0.004369	0.42
263824_at	putative WD-40 repeat protein	---	AT2G40360	2.55	1.35	6.62	0.000355	0.004385	0.42
254503_at	hypothetical protein ;supported by f	PTAC9	AT4G20010	1.77	0.82	6.61	0.000356	0.00439	0.42
249264_s_a	disease resistance protein-like	---	AT5G41740	-2.67	-1.42	-6.61	0.000356	0.004395	0.41
259411_at	hypothetical protein contains Pfam	---	AT1G13410	1.69	0.76	6.61	0.000357	0.0044	0.41
263729_at	hypothetical protein predicted by ge	---	AT1G60080	1.52	0.61	6.61	0.000357	0.0044	0.41
264201_at	unknown protein ;supported by full-	---	AT1G22630	2.26	1.18	6.61	0.000358	0.004405	0.41
259105_at	stress related protein, putative simi	---	AT3G05500	-2.09	-1.06	-6.61	0.000358	0.004405	0.41
257207_at	hypothetical protein predicted by ge	---	AT3G14900	1.85	0.89	6.60	0.00036	0.004422	0.40
264712_at	unknown protein	ECT11	AT1G09810	2.06	1.04	6.60	0.00036	0.004424	0.40
258992_at	unknown protein similar to unknowr	---	AT3G08930	-1.51	-0.59	-6.60	0.000361	0.004433	0.40
253121_at	putative protein predicted protein, (	---	AT4G35890	1.49	0.58	6.60	0.000361	0.004433	0.40
247345_at	putative protein contains similarity t	---	AT5G63760	1.84	0.88	6.59	0.000362	0.004436	0.40
262566_at	auxin response factor 1, putative si	ARF12	AT1G34310	-2.19	-1.13	-6.59	0.000362	0.004436	0.40
266065_at	phytochrome B Identical to GB:X17	PHYB	AT2G18790	-1.83	-0.88	-6.59	0.000363	0.004442	0.39
259154_at	unknown protein similar to unknowr	---	AT3G10260	-1.68	-0.75	-6.59	0.000363	0.004445	0.39
258728_at	unknown protein ; supported by cD	---	AT3G11800	-1.60	-0.68	-6.59	0.000364	0.004453	0.39
246953_at	unknown protein ;supported by full-	VPS60.2	AT5G04850	1.66	0.73	6.59	0.000365	0.004456	0.39
265931_at	unknown protein ;supported by full-	---	AT2G18520	1.99	0.99	6.58	0.000365	0.004456	0.39
245592_at	CCAAT-binding transcription factor	NF-YB3	AT4G14540	2.09	1.06	6.58	0.000365	0.004456	0.39
250938_at	putative protein various predicted p	---	AT5G03180	2.08	1.06	6.58	0.000365	0.004456	0.39
254758_at	putative protein dimethylaniline mo	YUC2	AT4G13260	-2.85	-1.51	-6.58	0.000366	0.004462	0.38
260041_at	unknown protein similar to hypothet	---	AT1G68780	-1.83	-0.87	-6.58	0.000366	0.004463	0.38
261695_at	Mg-chelatase, putative similar to M	CHLD	AT1G08520	-1.84	-0.88	-6.58	0.000367	0.004463	0.38
249843_at	putative protein similar to unknown	SGS3	AT5G23570	2.20	1.14	6.58	0.000367	0.004466	0.38
248785_at	unknown protein	---	AT5G47400	1.52	0.61	6.58	0.000367	0.004466	0.38
252529_at	putative protein several oxidases, r	---	AT3G46490	-4.77	-2.25	-6.58	0.000368	0.004472	0.38
267601_at	hypothetical protein predicted by ge	---	AT2G32980	-1.73	-0.79	-6.57	0.000368	0.004472	0.38
259295_at	hypothetical protein predicted by ge	---	AT3G05340	1.94	0.96	6.57	0.000369	0.004472	0.38
259708_at	lysophospholipase isolog, putative	---	AT1G77420	-1.98	-0.98	-6.57	0.000369	0.004472	0.38
259724_at	putative serine/threonine-protein kir	SNRK2.10	AT1G60940	2.00	1.00	6.57	0.000369	0.004476	0.38

246521_at	N2,N2-dimethylguanine tRNA meth	---	AT5G15810	1.97	0.98	6.57	0.00037	0.004484	0.37
259616_at	hypothetical protein contains similar	C/VIF1	AT1G47960	2.35	1.23	6.56	0.000372	0.0045	0.37
263574_at	putative tetracycline transporter pro	---	AT2G16990	-1.60	-0.67	-6.56	0.000373	0.004508	0.37
255716_at	coded for by A. thaliana cDNA R30	CRCK2	AT4G00330	-1.48	-0.57	-6.56	0.000375	0.004525	0.36
251260_at	putative protein ISOPENICILLIN N	---	AT3G62130	2.37	1.24	6.55	0.000376	0.004535	0.36
266280_at	putative tropinone reductase	---	AT2G29260	1.53	0.61	6.55	0.000376	0.00454	0.35
247311_at	DNA polymerase III catalytic subun	EMB2780	AT5G63960	1.74	0.80	6.55	0.000376	0.00454	0.35
263106_at	hypothetical protein predicted by ge	---	AT2G05160	-1.91	-0.93	-6.55	0.000377	0.004542	0.35
264219_at	unknown protein contains similarity	---	AT1G60420	2.19	1.13	6.55	0.000377	0.004542	0.35
247089_at	dimethyladenosine transferase-like	---	AT5G66360	1.80	0.85	6.55	0.000378	0.004555	0.35
258847_at	unknown protein ; supported by ful	---	AT3G03100	1.56	0.65	6.54	0.000379	0.004556	0.35
252081_at	putative heat shock transcription fa	HSFA7A	AT3G51910	3.28	1.71	6.54	0.000379	0.004556	0.35
255046_at	H+-transporting ATP synthase-like	ATPD	AT4G09650	-1.81	-0.86	-6.54	0.000379	0.004557	0.35
264100_at	lupeol synthase identical to lupeol s	LUP1	AT1G78970	-5.40	-2.43	-6.54	0.000381	0.004572	0.34
254051_at	putative protein other Arabidopsis p	---	AT4G25270	-1.68	-0.75	-6.54	0.000382	0.004574	0.34
257000_at	hypothetical protein predicted by ge	---	AT3G14120	2.28	1.19	6.54	0.000382	0.004574	0.34
257056_at	unknown protein similar to hypothet	---	AT3G15350	-1.73	-0.79	-6.53	0.000383	0.00458	0.34
266205_s_a	putative dTDP-glucose 4-6-dehydra	AXS2	AT1G08200	-1.83	-0.87	-6.53	0.000383	0.004581	0.34
250523_at	putative protein strong similarity to u	---	AT5G08510	1.53	0.61	6.53	0.000383	0.004581	0.34
266808_at	Expressed protein ; supported by fu	---	AT2G29995	-1.98	-0.99	-6.53	0.000385	0.004604	0.33
266513_at	unknown protein	---	AT2G47700	-1.93	-0.95	-6.52	0.000386	0.004607	0.33
245821_at	hypothetical protein similar to putati	---	AT1G26270	-1.93	-0.95	-6.52	0.000386	0.004608	0.33
256207_at	GTP-binding protein, putative simil	---	AT1G50920	1.97	0.98	6.52	0.000386	0.004608	0.33
254911_at	putative protein ;supported by full-l	---	AT4G11100	-1.63	-0.70	-6.52	0.000386	0.004608	0.33
261136_at	ribokinase, putative similar to GB:A	---	AT1G19600	1.60	0.68	6.52	0.000387	0.004612	0.32
262941_at	hypothetical protein predicted by ge	EMB2217	AT1G79490	2.17	1.12	6.52	0.000387	0.004612	0.32
249167_at	putative protein similar to unknown	---	AT5G42860	-3.74	-1.90	-6.52	0.000388	0.004616	0.32
261893_at	unknown protein ;supported by full-	---	AT1G80690	-1.68	-0.75	-6.51	0.000389	0.004627	0.32
263259_at	putative zinc-binding protein similar	PUB18	AT1G10560	-2.65	-1.41	-6.51	0.000389	0.004629	0.32
263521_at	hypothetical protein predicted by ge	---	AT2G24960	2.07	1.05	6.51	0.00039	0.004632	0.31
246020_at	putative protein ; supported by cDN	---	AT5G10710	1.66	0.73	6.51	0.00039	0.004632	0.31
265575_at	proline iminopeptidase identical to (	PIP	AT2G14260	1.61	0.69	6.51	0.000391	0.004632	0.31
252305_at	putative protein CRP1 protein, Zea	emb1796	AT3G49240	2.17	1.11	6.51	0.000391	0.004632	0.31
253438_at	glycine hydroxymethyltransferase (	SHM3	AT4G32520	1.98	0.99	6.51	0.000392	0.004635	0.31
262118_at	beta-glucosidase, putative similar t	BGLU11	AT1G02850	5.35	2.42	6.51	0.000392	0.004636	0.31

263039_at	mak16-like protein-related similar t	---	AT1G23280	2.14	1.10	6.51	0.000392	0.004636	0.31
248217_at	cytochrome b5 (dbj|BAA74839.1) ;	CB5-E	AT5G53560	1.87	0.90	6.50	0.000394	0.004653	0.31
265100_at	hypothetical protein predicted by ge	OSB4	AT1G31010	2.11	1.08	6.49	0.000396	0.004678	0.30
254427_at	putative protein predicted protein, A	emb1417	AT4G21190	2.46	1.30	6.49	0.000397	0.004683	0.30
252136_at	calmodulin-like protein flagellar cal	---	AT3G50770	16.83	4.07	6.49	0.000398	0.004698	0.29
256281_at	myb-family transcription factor, puta	TRFL9	AT3G12560	-1.89	-0.91	-6.48	0.000401	0.004716	0.29
259562_at	hypothetical protein ; supported by	---	AT1G21200	1.70	0.77	6.48	0.000401	0.00472	0.29
261053_at	hypothetical protein predicted by ge	---	AT1G01320	2.02	1.01	6.48	0.000402	0.004721	0.28
257019_at	unknown protein	---	AT3G19640	1.49	0.58	6.47	0.000403	0.004742	0.28
259746_at	hypothetical protein predicted by ge	---	AT1G71060	2.07	1.05	6.47	0.000404	0.004742	0.28
260453_s_a	unknown protein	---	AT1G72510	1.99	1.00	6.47	0.000404	0.004742	0.28
253175_at	WD-40 repeat protein (MSI3) ; sup	MSI3	AT4G35050	2.44	1.28	6.47	0.000404	0.004742	0.28
266166_at	putative glucosyltransferase ; supp	---	AT2G28080	-2.15	-1.10	-6.47	0.000405	0.004752	0.27
267411_at	putative disease resistance protein	---	AT2G34930	-16.62	-4.05	-6.47	0.000406	0.004761	0.27
259179_at	hypothetical protein similar to putati	---	AT3G01660	1.98	0.98	6.46	0.000407	0.004769	0.27
266934_at	unknown protein	---	AT2G18900	1.79	0.84	6.46	0.000408	0.004769	0.27
254213_at	tyrosine transaminase like protein t	---	AT4G23590	-1.63	-0.70	-6.46	0.000408	0.004769	0.27
261277_at	hypothetical protein predicted by ge	---	AT1G20230	1.84	0.88	6.46	0.000409	0.004774	0.27
255788_at	auxin regulated protein (IAA13) GB	IAA13	AT2G33310	2.03	1.02	6.46	0.000411	0.004792	0.26
255960_at	unknown protein ; supported by cDI	---	AT1G22140	1.94	0.96	6.46	0.000411	0.004792	0.26
252303_at	putative protein hypothetical protei	---	AT3G49210	1.60	0.68	6.46	0.000411	0.004792	0.26
260792_at	unknown protein ;supported by full-	---	AT1G06270	1.73	0.79	6.45	0.000411	0.004796	0.26
265192_at	hypothetical protein predicted by ge	---	AT1G05060	1.92	0.94	6.45	0.000412	0.004799	0.26
260069_at	unknown protein	---	AT1G73820	1.63	0.71	6.45	0.000412	0.004799	0.25
267406_at	unknown protein	MEE22	AT2G34780	1.83	0.87	6.45	0.000413	0.004799	0.25
259246_s_a	hypothetical protein predicted by ge	---	AT3G07730	1.96	0.97	6.45	0.000413	0.004799	0.25
248173_at	putative protein contains similarity t	---	AT5G54580	1.62	0.70	6.45	0.000413	0.004799	0.25
249443_at	putative protein ; supported by cDN	---	AT5G39600	1.99	1.00	6.45	0.000413	0.004803	0.25
264487_at	vacuolar protein sorting homolog al	VPS45	AT1G77140	-1.56	-0.64	-6.45	0.000414	0.004806	0.25
250856_at	putative protein salt-inducible prote	---	AT5G04810	1.91	0.93	6.44	0.000415	0.004811	0.25
256932_at	unknown protein	---	AT3G22520	1.61	0.69	6.44	0.000416	0.004825	0.25
258937_at	putative ribosomal protein S28 simi	---	AT3G10090	2.26	1.17	6.44	0.000417	0.004828	0.24
246681_at	chloride channel-like protein chloric	---	AT5G33280	1.55	0.63	6.44	0.000417	0.004832	0.24
247855_at	similar to unknown protein (sp|P232	---	AT5G58210	1.98	0.98	6.44	0.000417	0.004832	0.24
260815_at	chloroplast inner envelope protein,	TIC110	AT1G06950	2.37	1.25	6.44	0.000418	0.004839	0.24

258215_at	phosphoprotein phosphatase, puta	atToc64-III	AT3G17970	2.14	1.10	6.43	0.000419	0.004845	0.24
256286_at	unknown protein	---	AT3G12180	1.74	0.80	6.43	0.000421	0.004859	0.23
259675_at	unknown protein similar to hypothet	---	AT1G77710	1.62	0.69	6.43	0.000421	0.00486	0.23
266410_at	unknown protein	EMB2765	AT2G38770	1.59	0.67	6.43	0.000422	0.004868	0.23
254852_at	putative disease resistance protein	WRKY19	AT4G12020	-1.50	-0.59	-6.42	0.000423	0.004872	0.23
248021_at	RuBisCO subunit binding-protein b	---	AT5G56500	2.63	1.39	6.42	0.000425	0.004888	0.22
262189_at	GTPase, putative similar to GTPas	---	AT1G78010	1.69	0.76	6.42	0.000426	0.004894	0.22
267429_at	putative UDP-galactose-4-epimeras	MEE25	AT2G34850	-2.14	-1.10	-6.42	0.000426	0.004894	0.22
253520_at	hypothetical protein ;supported by f	---	AT4G31410	1.56	0.64	6.41	0.000427	0.00491	0.22
253583_at	translation initiation factor-like prote	---	AT4G30680	-1.91	-0.93	-6.41	0.000428	0.00491	0.21
249400_at	putative protein MtN24 gene, Medi	---	AT5G40300	1.92	0.94	6.41	0.000428	0.00491	0.21
258993_at	putative chlorophyll a/b-binding pro	LHCB4.2	AT3G08940	-1.81	-0.86	-6.41	0.000429	0.00491	0.21
260258_at	putative ribosomal protein similar to	---	AT1G74270	2.03	1.02	6.41	0.000429	0.00491	0.21
252115_at	non-specific lipid transfer protein ;s	LTP5	AT3G51600	-1.87	-0.90	-6.41	0.000429	0.00491	0.21
258434_at	AP2 domain containing protein RAF	ATEBP	AT3G16770	2.18	1.12	6.41	0.000429	0.00491	0.21
267553_s_a	hypothetical protein predicted by ge	---	AT2G32180	2.18	1.12	6.41	0.000429	0.00491	0.21
263018_at	putative non-LTR retroelement reve	---	---	-1.48	-0.57	-6.41	0.00043	0.004914	0.21
247942_at	putative protein similar to unknown	---	AT5G57120	2.10	1.07	6.41	0.00043	0.004914	0.21
254827_at	putative protein KIAA0255 gene, H	---	AT4G12650	-1.71	-0.78	-6.40	0.000431	0.004916	0.21
246058_at	putative protein predicted proteins,	---	AT5G08430	1.73	0.79	6.40	0.000433	0.004935	0.20
259131_at	unknown protein similar to hypothet	SP1L3	AT3G02180	-1.63	-0.71	-6.40	0.000433	0.004939	0.20
250538_at	RNA helicase (emb|CAA09212.1) ;	STRS2	AT5G08620	2.36	1.24	6.39	0.000434	0.004945	0.20
258474_at	hypothetical protein predicted by ge	---	AT3G02650	2.18	1.13	6.39	0.000435	0.004945	0.20
251865_at	protein phosphatase 2A regulatory	---	AT3G54930	-1.55	-0.63	-6.39	0.000435	0.004945	0.20
262648_at	putative ribulose-1,5 bisphosphate	---	AT1G14030	1.64	0.71	6.39	0.000435	0.004945	0.20
263609_at	putative LRR receptor protein kinas	---	AT2G16250	-1.61	-0.68	-6.39	0.000435	0.004945	0.20
245314_at	Expressed protein ; supported by fu	---	AT4G16745	-1.69	-0.76	-6.39	0.000435	0.004945	0.20
263689_at	ribonuclease, RNS3 identical to rib	RNS3	AT1G26820	-2.42	-1.27	-6.39	0.000437	0.004959	0.19
265190_at	unknown protein ; supported by cD	---	AT1G23780	1.53	0.62	6.39	0.000438	0.004964	0.19
248795_at	Myb-related transcription activator-l	---	AT5G47390	-1.54	-0.62	-6.39	0.000438	0.004964	0.19
256972_at	unknown protein	---	AT3G21140	1.68	0.75	6.38	0.000439	0.004976	0.19
256223_at	unknown protein ;supported by full-	emb1303	AT1G56200	1.78	0.83	6.38	0.00044	0.004987	0.18
248938_at	receptor-like protein kinase	---	AT5G45780	1.57	0.65	6.38	0.000442	0.005001	0.18
256113_at	unknown protein ;supported by full-	---	AT1G16810	1.77	0.83	6.37	0.000442	0.005004	0.18
263692_at	unknown protein Location of ESTs	---	AT1G26850	-1.66	-0.73	-6.37	0.000443	0.005006	0.18

259673_at	putative phorbol ester / diacylglycer	---	AT1G77800	1.97	0.97	6.37	0.000444	0.005022	0.17
248603_at	WD-40 repeat protein-like	---	AT5G49430	1.54	0.62	6.37	0.000446	0.005029	0.17
264806_at	hypothetical protein predicted by ge	---	AT1G08610	2.73	1.45	6.37	0.000446	0.005029	0.17
264389_at	unknown protein similar to hypothet	---	AT1G11960	-2.38	-1.25	-6.37	0.000446	0.005029	0.17
261222_at	anter-specific proline-rich protein A	---	AT1G20120	1.59	0.67	6.36	0.000446	0.005029	0.17
260370_at	putative aminolevulinate dehydratas	HEMB1	AT1G69740	-1.64	-0.71	-6.36	0.000447	0.005035	0.17
249049_at	tubulin beta-4 chain (sp|P24636) ; s	TUB4	AT5G44340	-2.12	-1.08	-6.36	0.000448	0.005043	0.16
252693_s_a	ribosomal protein S29 - like ribosor	---	AT3G43980 ;	1.61	0.69	6.36	0.000448	0.005043	0.16
258526_at	DAG protein, putative similar to DA	---	AT3G06790	1.69	0.76	6.36	0.000449	0.005043	0.16
246188_at	putative protein	---	AT5G21050	-1.51	-0.60	-6.36	0.000449	0.005044	0.16
267580_at	unknown protein	---	AT2G41990	-1.82	-0.86	-6.36	0.000449	0.005045	0.16
258621_at	zinc finger protein 1 (zfn1) identical	ZFN1	AT3G02830	-1.60	-0.68	-6.36	0.00045	0.005046	0.16
246496_at	putative protein KIAA1468 - Homo	---	AT5G16210	-1.73	-0.79	-6.36	0.00045	0.005046	0.16
254153_at	putative protein hypothetical protei	PWD	AT4G24450	2.68	1.42	6.36	0.00045	0.005046	0.16
253254_at	predicted protein	SQS2	AT4G34650	-2.80	-1.49	-6.35	0.000451	0.005056	0.16
249794_at	putative protein contains similarity t	AtCXE18	AT5G23530	-1.51	-0.60	-6.35	0.000452	0.00506	0.16
256894_at	PREG-like protein, putative similar	CYCP2;1	AT3G21870	1.51	0.60	6.35	0.000452	0.005063	0.15
265283_at	unknown protein predicted by gene	MUR3	AT2G20370	-2.14	-1.10	-6.35	0.000453	0.005066	0.15
250861_at	putative protein predicted proteins,	---	AT5G04740	1.79	0.84	6.35	0.000453	0.005066	0.15
247106_at	WD repeat protein-like ;supported b	---	AT5G66240	1.73	0.79	6.34	0.000455	0.005072	0.15
257403_at	hypothetical protein similar to hypot	---	AT1G62520	-1.67	-0.74	-6.34	0.000455	0.005072	0.15
260682_at	hypothetical protein predicted by ge	---	AT1G17510	1.65	0.72	6.34	0.000455	0.005072	0.15
252357_at	putative protein predicted proteins,	---	AT3G48410	2.13	1.09	6.34	0.000455	0.005072	0.15
249470_at	putative protein predicted proteins,	---	AT5G39350	1.85	0.88	6.34	0.000456	0.005072	0.15
253440_at	putative protein ;supported by full-le	TIFY8	AT4G32570	1.47	0.56	6.34	0.000456	0.005072	0.15
254306_at	putative protein ; supported by cDN	ATCES1	AT4G22330	-1.51	-0.60	-6.34	0.000456	0.005072	0.15
251206_at	putative protein various predicted p	---	AT3G63090	2.16	1.11	6.34	0.000456	0.005072	0.14
263707_at	hypothetical protein Similar to gi|15	---	AT1G09300	2.16	1.11	6.34	0.000456	0.005072	0.14
256887_at	hypothetical protein predicted by ge	---	AT3G15150	1.77	0.83	6.34	0.000457	0.005074	0.14
248579_at	mitotic checkpoint protein-like	---	AT5G49880	2.08	1.06	6.34	0.000457	0.005076	0.14
247449_at	putative protein swelling-induced c	---	AT5G62290	2.42	1.28	6.34	0.000458	0.005081	0.14
254527_at	putative protein KIAA0682 protein,	---	AT4G19610	1.49	0.58	6.34	0.000458	0.005081	0.14
246824_at	drought-induced protein - like non-c	---	AT5G26990	-1.56	-0.64	-6.34	0.000459	0.005081	0.14
248765_at	MutT domain protein-like ; supporte	ATNUDT2	AT5G47650	-1.57	-0.65	-6.33	0.000459	0.005081	0.14
261743_s_a	protein serine/threonine phosphata	BSL2	AT1G08420 ;	1.62	0.70	6.33	0.000459	0.005081	0.14

258097_at	transport protein, putative similar to	---	AT3G23660	-1.53	-0.61	-6.33	0.00046	0.005093	0.14
254493_at	DAG-like protein DAG precursor, A	---	AT4G20020	2.45	1.29	6.33	0.000461	0.005095	0.13
249862_at	PGPD14 protein ;supported by full-	---	AT5G22920	-2.94	-1.56	-6.33	0.000461	0.005098	0.13
255510_at	polycomb group - like protein simila	SWN	AT4G02020	1.99	0.99	6.33	0.000462	0.005102	0.13
251713_at	putative protein several hypothetica	---	AT3G56080	1.94	0.95	6.33	0.000463	0.005103	0.13
265206_at	putative protease belongs to eukary	---	AT2G36670	1.81	0.86	6.32	0.000463	0.005105	0.13
259899_at	hypothetical protein predicted by ge	---	AT1G71210	1.98	0.98	6.32	0.000463	0.005105	0.13
248951_at	putative protein similar to unknown	---	AT5G45550	-1.86	-0.89	-6.32	0.000464	0.005105	0.13
256542_at	unknown protein	PMI1	AT1G42550	1.92	0.94	6.32	0.000464	0.005105	0.13
251233_at	putative protein hypothetical protei	DRB4	AT3G62800	1.66	0.73	6.32	0.000465	0.005114	0.12
260087_at	hypothetical protein predicted by ge	---	AT1G73200	-1.99	-0.99	-6.32	0.000465	0.005114	0.12
260678_at	hypothetical protein predicted by ge	---	AT1G07970	1.49	0.58	6.32	0.000467	0.005127	0.12
257092_at	unknown protein	DDL	AT3G20550	1.93	0.95	6.31	0.000468	0.005137	0.12
259392_at	hypothetical protein similar to hypot	---	AT1G06380	1.57	0.65	6.31	0.000468	0.005137	0.12
258555_at	fatty acid multifunctional protein (At	MFP2	AT3G06860	1.76	0.82	6.31	0.000469	0.00514	0.12
267064_at	calmodulin (cam2)	CAM2	AT2G41110	-2.77	-1.47	-6.31	0.00047	0.005153	0.11
261422_at	unknown protein	NDF6	AT1G18730	-1.83	-0.88	-6.30	0.000472	0.00517	0.11
255061_at	putative protein disulfide isomerase	ATAPRL6	AT4G08930	-1.53	-0.61	-6.30	0.000473	0.005176	0.11
255891_at	hypothetical protein predicted by ge	EGY3	AT1G17870	-1.81	-0.86	-6.30	0.000475	0.005189	0.10
267211_at	Expressed protein ; supported by fu	---	AT2G44065	1.51	0.59	6.30	0.000475	0.005194	0.10
258538_at	putative tRNA pseudouridine syntha	---	AT3G06950	1.59	0.67	6.30	0.000475	0.005194	0.10
262074_at	glutathione S-transferase, putative	ATGSTU15	AT1G59670	2.45	1.29	6.29	0.000476	0.005202	0.10
247779_at	putative protein damage-specific D	DDB2	AT5G58760	1.84	0.88	6.29	0.000477	0.005205	0.10
266563_at	nodulin-like protein	---	AT2G23990	-1.62	-0.69	-6.29	0.000478	0.005214	0.09
260954_at	glyoxalase II isozyme, putative sim	GLX2-4	AT1G06130	1.81	0.85	6.29	0.00048	0.005227	0.09
265087_at	hypothetical protein predicted by ge	---	AT1G03760	2.47	1.31	6.29	0.00048	0.005227	0.09
261935_at	phosphoribosylanthranilate transfe	---	AT1G22610	-1.55	-0.63	-6.29	0.00048	0.005228	0.09
263222_at	putative protein kinase Strong simila	---	AT1G30640	-2.17	-1.12	-6.28	0.000481	0.005231	0.09
253659_at	hypothetical protein	---	AT4G30150	1.66	0.73	6.28	0.000481	0.005231	0.09
258925_at	unknown protein similar to hypothe	---	AT3G10420	1.82	0.87	6.28	0.000482	0.005236	0.09
251667_at	putative protein DYSKERIN (NUCL	NAP57	AT3G57150	3.23	1.69	6.28	0.000482	0.00524	0.08
250549_at	N-hydroxycinnamoyl benzoyltransf	---	AT5G07860	-2.21	-1.15	-6.28	0.000482	0.00524	0.08
256230_at	FKBP-type peptidyl-prolyl cis-trans	---	AT3G12340	1.89	0.92	6.28	0.000483	0.005242	0.08
265101_at	unknown protein similar to ESTs gb	---	AT1G30880	2.32	1.21	6.28	0.000483	0.005242	0.08
254353_s_a	putative protein probable membran	---	AT4G22285	2.02	1.01	6.28	0.000483	0.005242	0.08

261336_at	unknown protein ;supported by full-	---	AT1G44790	-2.17	-1.12	-6.28	0.000484	0.005243	0.08
263448_at	putative importin (nuclear transport	SAD2	AT2G31660	1.82	0.86	6.28	0.000484	0.005244	0.08
264621_at	putative protein kinase contains a p	---	AT2G17700	1.75	0.80	6.28	0.000484	0.005246	0.08
251879_at	putative protein hin1 protein, Nicoti	---	AT3G54200	-2.72	-1.44	-6.27	0.000487	0.005271	0.07
244936_at	NADH dehydrogenase ND1	---	---	-1.63	-0.70	-6.27	0.000488	0.005278	0.07
252374_at	response reactor 2 (ATRR2) ; supp	ARR5	AT3G48100	-2.41	-1.27	-6.27	0.000488	0.005278	0.07
256723_at	unknown protein ;supported by full-	---	AT2G34160	1.50	0.59	6.27	0.000489	0.005285	0.07
245926_at	VPS13 - like protein VPS13, Sacch	---	AT5G24740	1.68	0.74	6.26	0.00049	0.005288	0.07
262852_at	hypothetical protein predicted by ge	---	AT1G20910	1.61	0.69	6.26	0.000492	0.005308	0.06
265991_at	chloroplast single subunit DNA-dep	SCA3	AT2G24120	2.43	1.28	6.26	0.000492	0.005308	0.06
266045_s_a	hypothetical protein	---	AT2G07727	1.99	0.99	6.26	0.000493	0.005319	0.06
267147_at	putative anthocyanidin synthase ;s	---	AT2G38240	-8.39	-3.07	-6.26	0.000494	0.005319	0.06
250330_at	putative protein	---	AT5G11810	-1.73	-0.79	-6.26	0.000494	0.005319	0.06
265947_at	putative WD-40 repeat protein ; su	---	AT2G19540	2.30	1.20	6.26	0.000494	0.00532	0.06
246308_at	chlorophyll synthetase ; supported	G4	AT3G51820	-1.87	-0.90	-6.25	0.000495	0.005324	0.06
266076_at	ATP-dependent RNA helicase iden	---	AT2G40700	2.50	1.32	6.25	0.000495	0.005324	0.06
254784_at	growth factor like protein antisense	NUDT7	AT4G12720	-3.30	-1.72	-6.25	0.000498	0.005349	0.05
261591_at	protein kinase, putative similar to p	---	AT1G01740	1.96	0.97	6.25	0.000498	0.005349	0.05
249283_at	amino acid permease-like protein; p	---	AT5G41800	-2.04	-1.03	-6.25	0.000498	0.00535	0.05
262326_at	hypothetical protein similar to putati	---	AT1G64140	1.55	0.63	6.25	0.000498	0.00535	0.05
258845_at	unknown protein ; supported by ful	---	AT3G03150	-1.75	-0.81	-6.24	0.000502	0.005385	0.04
264920_at	hypothetical protein contains simila	ECHID	AT1G60550	-1.77	-0.82	-6.24	0.000503	0.005385	0.04
259794_at	unknown protein	---	AT1G64330	-1.73	-0.79	-6.24	0.000503	0.005385	0.04
266104_at	putative phosphatidate cytidylyltran	---	AT2G45150	1.73	0.79	6.23	0.000506	0.005412	0.03
255028_at	putative protein Arabidopsis thaliar	PMP	AT3G24160	2.86	1.51	6.23	0.000508	0.005421	0.03
247173_at	poly(A)-binding protein II-like ; sup	---	AT5G65260	1.79	0.84	6.23	0.000508	0.005421	0.03
261016_at	beta-glucosidase, putative similar t	BGLU40	AT1G26560	-5.99	-2.58	-6.23	0.000508	0.005421	0.03
247032_at	putative protein strong similarity to u	SDN3	AT5G67240	1.60	0.68	6.22	0.000509	0.005424	0.03
259278_at	hypothetical protein predicted by ge	---	AT3G01160	1.76	0.82	6.22	0.000512	0.005455	0.02
257125_at	t-complex polypeptide 1 homologue	ATTCP-1	AT3G20050	1.80	0.85	6.22	0.000512	0.005455	0.02
249960_at	squamosa promoter binding protein	SPL7	AT5G18830	1.63	0.70	6.22	0.000513	0.005455	0.02
254382_at	hypothetical protein	---	AT4G21890	-2.05	-1.04	-6.21	0.000513	0.005457	0.02
251130_at	oligopeptide transporter - like prote	PTR5	AT5G01180	-1.54	-0.62	-6.21	0.000513	0.005457	0.02
246262_at	hypothetical protein	---	AT1G31790	2.03	1.02	6.21	0.000514	0.005464	0.01
258380_at	PP1/PP2A phosphatases pleiotropi	---	AT3G16650	1.69	0.76	6.21	0.000514	0.005464	0.01

246490_at	S-adenosylmethionine decarboxyla	CPuORF10	AT5G15948	1.98	0.99	6.21	0.000515	0.005464	0.01
253208_at	putative protein	---	AT4G34830	1.59	0.67	6.21	0.000515	0.005467	0.01
247207_at	putative protein similar to unknown	---	AT5G65000	1.48	0.56	6.21	0.000516	0.005468	0.01
257892_at	unknown protein similar to ER6 pro	---	AT3G17020	1.64	0.71	6.21	0.000516	0.005474	0.01
254734_at	methionyl-tRNA synthetase - like p	---	AT4G13780	1.70	0.77	6.21	0.000517	0.005474	0.01
263693_at	hypothetical protein predicted by ge	ATPP2-A9	AT1G31200	3.50	1.81	6.21	0.000517	0.005474	0.01
249964_at	MAP kinase -like protein ATMPK9,	MPK16	AT5G19010	-1.62	-0.70	-6.21	0.000517	0.005474	0.01
265383_at	putative WD-40 repeat protein, MS	MSI2	AT2G16780	2.02	1.02	6.20	0.000519	0.005491	0.00
263599_at	putative histidine kinase ; supporte	WOL	AT2G01830	-2.19	-1.13	-6.20	0.000521	0.005507	0.00
247312_at	putative protein strong similarity to u	---	AT5G63970	1.83	0.87	6.20	0.000522	0.005518	0.00
255580_at	putative water channel protein	TIP1;3	AT4G01470	-1.84	-0.88	-6.19	0.000523	0.005526	0.00
248495_at	putative protein strong similarity to u	---	AT5G50780	1.77	0.83	6.19	0.000523	0.005526	-0.01
259172_at	O-acetylserine (thiol) lyase identica	CS26	AT3G03630	2.68	1.42	6.19	0.000525	0.00554	-0.01
264053_at	hypothetical protein predicted by ge	---	AT2G22560	-1.93	-0.95	-6.19	0.000526	0.005543	-0.01
247053_at	protein kinase ATN1-like protein	---	AT5G66710	-1.49	-0.57	-6.19	0.000527	0.005548	-0.01
248680_at	pantoate-beta-alanine ligase ;supp	PANC	AT5G48840	1.74	0.80	6.18	0.000528	0.005557	-0.01
253443_at	putative protein beta transducin-like	LUG	AT4G32551	1.45	0.54	6.18	0.000529	0.005557	-0.02
248058_at	putative protein similar to unknown	---	AT5G55530	1.68	0.75	6.18	0.000529	0.005557	-0.02
253622_at	cyclic nucleotide and calmodulin-re	ATCNGC9	AT4G30560	-1.61	-0.69	-6.18	0.00053	0.005559	-0.02
262967_at	hypothetical protein predicted by ge	---	AT1G75730	-1.70	-0.76	-6.18	0.00053	0.005559	-0.02
260337_at	unknown protein	WRKY57	AT1G69310	1.78	0.83	6.18	0.00053	0.005559	-0.02
258716_at	unknown protein similar to a region	---	AT3G09700	2.22	1.15	6.18	0.000531	0.005565	-0.02
267628_at	unknown protein similar to GP|2262	---	AT2G42280	1.71	0.77	6.18	0.000531	0.005565	-0.02
246384_at	glutaredoxin, putative similar to glu	---	AT1G77370	1.53	0.61	6.18	0.000533	0.005577	-0.02
258292_at	hypothetical protein predicted by ge	---	AT3G23330	1.80	0.85	6.18	0.000533	0.005577	-0.02
265865_at	hypothetical protein predicted by ge	---	AT2G01740	1.52	0.60	6.18	0.000533	0.005577	-0.02
253980_at	sucrase-like protein sucrose cleava	---	AT4G26620	2.25	1.17	6.17	0.000534	0.005581	-0.03
245050_at	PSII K protein	---	---	-1.46	-0.55	-6.17	0.000534	0.005581	-0.03
260229_at	putative RING zinc finger protein c	---	AT1G74370	-1.88	-0.91	-6.17	0.000534	0.005581	-0.03
247625_at	zinc finger protein - like dof6 zinc fi	---	AT5G60200	-1.95	-0.97	-6.17	0.000536	0.005596	-0.03
250440_at	40S ribsomal protein S6 ; supporte	EMB3010	AT5G10360	2.01	1.01	6.17	0.000537	0.005604	-0.03
263880_at	unknown protein ;supported by full-	---	AT2G21960	-2.00	-1.00	-6.17	0.000537	0.005608	-0.03
245685_at	Expressed protein ; supported by cl	E2F1	AT5G22220	-1.50	-0.59	-6.17	0.000538	0.005608	-0.03
255742_at	DNA-binding protein RAV2, putativ	TEM1	AT1G25560	-1.57	-0.65	-6.17	0.000538	0.005608	-0.03
259489_at	unknown protein	---	AT1G15790	2.45	1.29	6.16	0.000538	0.005609	-0.04

252573_at	zinc finger protein zinc finger prote	---	AT3G45260	-1.64	-0.72	-6.16	0.000539	0.005609	-0.04
261190_at	ribosomal protein L11, putative sim	PRPL11	AT1G32990	-1.69	-0.75	-6.16	0.00054	0.005611	-0.04
259756_at	unknown protein ; supported by cD	---	AT1G71080	-1.56	-0.65	-6.16	0.00054	0.005611	-0.04
246045_at	putative protein predicted proteins,	---	AT5G19430	1.90	0.93	6.16	0.00054	0.005611	-0.04
249070_at	cellulose synthase catalytic subunit	CESA4	AT5G44030	-1.83	-0.87	-6.16	0.000542	0.005628	-0.04
248757_at	putative protein contains similarity t	---	AT5G47680	1.54	0.62	6.16	0.000543	0.005628	-0.04
246134_at	protein kinase tousled ; supported	TSL	AT5G20930	1.84	0.88	6.16	0.000543	0.005628	-0.04
249026_at	Expressed protein ; supported by fu	OSB3	AT5G44785	1.88	0.91	6.15	0.000545	0.005639	-0.05
247495_at	putative protein predicted protein, A	IPK2BETA	AT5G61760	1.81	0.85	6.15	0.000545	0.005642	-0.05
255443_at	sulfate transporter protein similar to	SULTR3;2	AT4G02700	-1.77	-0.82	-6.15	0.000546	0.005649	-0.05
250402_at	putative protein unnamed ORF cDI	---	AT5G10940	1.57	0.65	6.15	0.000546	0.00565	-0.05
247418_at	glutaredoxin-like protein ; supporte	---	AT5G63030	1.91	0.93	6.15	0.000547	0.005656	-0.05
246148_at	glycine-rich RNA-binding protein - I	---	AT5G19960	1.74	0.80	6.14	0.000548	0.005663	-0.06
258965_at	hypothetical protein similar to hypo	---	AT3G10530	2.66	1.41	6.14	0.000549	0.00567	-0.06
254943_at	putative protein predicted proteins,	---	AT4G10890	1.62	0.69	6.14	0.00055	0.00567	-0.06
260766_at	unknown protein similar to hypothet	---	AT1G48960	-1.67	-0.74	-6.14	0.000551	0.005682	-0.06
254398_at	photosystem II oxygen-evolving co	PSBQA	AT4G21280	-1.70	-0.76	-6.14	0.000552	0.005682	-0.06
266889_at	hypothetical protein predicted by ge	---	AT2G44640	1.71	0.78	6.13	0.000554	0.005693	-0.07
266932_s_a	putative RNA helicase	---	AT1G63250	2.20	1.14	6.13	0.000555	0.005698	-0.07
263978_at	unknown protein ;supported by full-	MBF1A	AT2G42680	1.56	0.64	6.13	0.000555	0.005701	-0.07
251859_at	putative protein proteophosphoglyc	---	AT3G54680	1.68	0.75	6.13	0.000556	0.005712	-0.07
250672_at	DnaJ homologue (gb|AAB91418.1|)	ATJ6	AT5G06910	1.48	0.57	6.13	0.000558	0.005719	-0.07
260425_at	hypothetical protein similar to CCA	EDA25	AT1G72440	3.22	1.69	6.13	0.000559	0.005719	-0.08
253958_at	putative protein RING zinc finger p	---	AT4G26400	-1.69	-0.76	-6.13	0.000559	0.005719	-0.08
256483_at	hypothetical protein	---	AT1G31410	-1.84	-0.88	-6.13	0.000559	0.005719	-0.08
246830_at	chaperonin gamma chain - like pro	---	AT5G26360	2.49	1.32	6.12	0.000559	0.005722	-0.08
265150_at	unknown protein ; supported by cD	---	AT1G51200	1.84	0.88	6.12	0.00056	0.005722	-0.08
248766_at	putative protein strong similarity to u	---	AT5G47580	2.79	1.48	6.12	0.00056	0.005722	-0.08
263472_at	molybdopterin synthase (CNX2) ;su	CNX2	AT2G31955	1.85	0.89	6.12	0.00056	0.005723	-0.08
245405_at	hypothetical protein	---	AT4G17150	-1.49	-0.57	-6.12	0.000561	0.00573	-0.08
261398_at	hypothetical protein contains similar	NHX6	AT1G79610	1.64	0.72	6.12	0.000561	0.00573	-0.08
250757_at	tetrahydrofolylpolyglutamate syntha	ATDFB	AT5G05980	1.64	0.71	6.11	0.000564	0.005753	-0.09
249817_at	putative protein similar to unknown	---	AT5G23820	-13.09	-3.71	-6.11	0.000566	0.005763	-0.09
247718_at	nonspecific lipid-transfer protein pr	LTP4	AT5G59310	-1.94	-0.96	-6.11	0.000567	0.005768	-0.09
249128_at	1-aminocyclopropane-1-carboxylate	---	AT5G43440	1.60	0.68	6.11	0.000569	0.005786	-0.10

245702_at	calcium lipid binding protein - like G	SYTC	AT5G04220	2.04	1.03	6.10	0.00057	0.005789	-0.10
262044_s_a	hypothetical protein predicted by ge	---	AT1G80210	1.63	0.70	6.10	0.00057	0.005791	-0.10
249753_at	putative protein similar to unknown	---	AT5G24610	-1.48	-0.56	-6.10	0.000571	0.005791	-0.10
254190_at	Expressed protein ; supported by fu	---	AT4G23885	2.19	1.13	6.10	0.000571	0.005791	-0.10
260622_at	hypothetical protein predicted by ge	NF-YC10	AT1G07980	1.56	0.64	6.10	0.000571	0.005791	-0.10
257291_at	DNA-binding protein, putative simil	---	AT3G15590	3.00	1.58	6.10	0.000571	0.005792	-0.10
267268_at	hypothetical protein predicted by ge	---	AT2G02570	1.64	0.72	6.10	0.000574	0.005821	-0.11
258811_at	unknown protein similar to putative	---	AT3G03990	-2.16	-1.11	-6.09	0.000575	0.005825	-0.11
258445_at	putative lipoxygenase similar to lipo	LOX5	AT3G22400	-2.33	-1.22	-6.09	0.000577	0.005841	-0.11
264072_at	hypothetical protein	---	---	1.55	0.63	6.09	0.000578	0.005842	-0.11
251029_at	putative protein gene product of su	---	AT5G02050	2.75	1.46	6.09	0.000579	0.005849	-0.11
266939_at	plasma membrane proton ATPase	AHA1	AT2G18960	1.48	0.56	6.09	0.000579	0.005849	-0.12
267084_at	unknown protein ; supported by cD	---	AT2G41180	6.37	2.67	6.09	0.00058	0.005849	-0.12
259610_at	bZIP protein, putative similar to GI:	---	AT1G52320	1.52	0.61	6.09	0.00058	0.005851	-0.12
265662_at	putative C2H2-type zinc finger prote	FZF	AT2G24500	1.91	0.93	6.08	0.00058	0.005853	-0.12
261826_at	pectin methylesterase, putative sim	PMEPCRA	AT1G11580	-2.94	-1.55	-6.08	0.000581	0.005853	-0.12
261844_at	hypothetical protein predicted by ge	---	AT1G15940	2.30	1.20	6.08	0.000582	0.005859	-0.12
263843_at	translin-like protein ; supported by	---	AT2G37020	1.59	0.67	6.08	0.000583	0.005862	-0.12
261483_at	hypothetical protein predicted by ge	---	AT1G14270	-1.72	-0.78	-6.08	0.000583	0.005865	-0.12
256770_at	hypothetical protein predicted by ge	---	AT3G13700	-1.89	-0.92	-6.07	0.000588	0.0059	-0.13
259273_s_a	serine/threonine specific protein kir	---	AT3G01300	1.61	0.69	6.07	0.000589	0.005908	-0.13
256205_at	hypothetical protein	---	AT1G50890	-1.59	-0.67	-6.06	0.000594	0.005945	-0.14
248063_at	RNA-binding protein-like ; supporte	---	AT5G55550	-1.57	-0.65	-6.06	0.000594	0.005947	-0.14
246400_at	hypothetical protein contains similar	---	AT1G57540	1.52	0.61	6.06	0.000595	0.005954	-0.15
249345_at	putative protein ; supported by cDN	---	AT5G40740	-1.68	-0.75	-6.06	0.000595	0.005954	-0.15
265340_at	putative AAA-type ATPase similar t	---	AT2G18330	2.45	1.29	6.05	0.000598	0.00597	-0.15
246286_at	unknown protein	---	AT1G31910	1.55	0.63	6.05	0.000599	0.005978	-0.15
259163_at	putative protein kinase similar to AT	---	AT3G01490	-1.86	-0.89	-6.05	0.0006	0.005989	-0.15
254389_s_a	putative protein various predicted p	---	AT4G21903	-3.29	-1.72	-6.05	0.000601	0.005991	-0.16
245723_at	hypothetical protein predicted by ge	---	AT1G73400	1.86	0.90	6.05	0.000602	0.005997	-0.16
262341_at	E2, ubiquitin-conjugating enzyme,	UBC28	AT1G64230	1.76	0.81	6.05	0.000603	0.005997	-0.16
247886_at	branched-chain amino acid aminotr	---	AT5G57850	1.98	0.99	6.05	0.000603	0.005997	-0.16
259080_at	putative mitogen activated protein k	WNK1	AT3G04910	-1.77	-0.83	-6.04	0.000604	0.006006	-0.16
251935_at	fructokinase - like protein fructokina	---	AT3G54090	2.03	1.02	6.04	0.000605	0.006013	-0.16
253421_at	putative protein predicted proteins,	---	AT4G32340	1.88	0.91	6.04	0.000606	0.006015	-0.16

261337_at	unknown protein ; supported by cDI	---	AT1G44810	2.41	1.27	6.04	0.000606	0.006015	-0.17
259948_at	unknown protein similar to LIGATIN	---	AT1G71350	2.08	1.06	6.04	0.000608	0.006024	-0.17
250222_at	putative protein similar to unknown	---	AT5G14050	2.22	1.15	6.04	0.000608	0.006025	-0.17
252512_at	receptor protein kinase -like recept	---	AT3G46290	-1.60	-0.68	-6.03	0.000609	0.006035	-0.17
263920_at	unknown protein ;supported by full-	---	AT2G36410	-1.83	-0.87	-6.03	0.000613	0.006065	-0.18
253919_at	putative protein ; supported by full-	---	AT4G27350	-2.67	-1.42	-6.02	0.000617	0.0061	-0.18
251936_at	putative protein crp1 protein, Zea n	MEE40	AT3G53700	1.82	0.86	6.02	0.000617	0.006102	-0.19
245557_at	phosphatase like protein	PUX5	AT4G15410	-1.45	-0.54	-6.02	0.000618	0.006103	-0.19
258461_at	hypothetical protein predicted by ge	---	AT3G17340	-1.52	-0.60	-6.02	0.000618	0.006103	-0.19
245180_at	putative protein similarity to proteins	---	AT5G12410	2.07	1.05	6.02	0.000619	0.006103	-0.19
256424_at	unknown protein ;supported by full-	---	AT1G33490	1.61	0.68	6.02	0.000619	0.006103	-0.19
245668_at	dormancy-associated protein, puta	DYL1	AT1G28330	1.90	0.92	6.01	0.000621	0.006127	-0.19
250602_s_a	unknown protein	---	AT5G07940	1.60	0.68	6.01	0.000623	0.006137	-0.20
259138_s_a	putative DNA gyrase subunit B simi	---	AT3G10270	1.66	0.73	6.01	0.000624	0.006142	-0.20
251265_at	ATP-dependent RNA helicase-like	---	AT3G62310	1.88	0.91	6.01	0.000625	0.006148	-0.20
251663_at	putative protein hypothetical protei	---	AT3G57070	-1.62	-0.69	-6.01	0.000626	0.006152	-0.20
246090_at	putative protein BEM46 bem1/bud5	WAV2	AT5G20520	2.47	1.31	6.01	0.000626	0.006152	-0.20
253715_at	RIBOSOMAL PROTEIN S30 homo	---	AT4G29390	1.69	0.76	6.00	0.000627	0.006161	-0.20
252886_at	cellulose synthase catalytic subunit	CESA2	AT4G39350	-1.72	-0.78	-6.00	0.000631	0.006191	-0.21
264818_at	hypothetical protein similar to hypot	NAF1	AT1G03530	2.85	1.51	5.99	0.000633	0.00621	-0.21
256225_at	unknown protein ; supported by full-	---	AT1G56220	1.98	0.99	5.99	0.000635	0.006222	-0.22
259665_at	unknown protein ; supported by cDI	---	AT1G55160	-1.87	-0.91	-5.99	0.000636	0.00623	-0.22
262525_at	putative cytochrome P450 41% ider	CYP72C1	AT1G17060	-2.30	-1.20	-5.99	0.000636	0.00623	-0.22
266253_at	unknown protein contains non-cons	HDT4	AT2G27840	2.58	1.37	5.99	0.000637	0.006235	-0.22
253445_at	putative protein pAFD103 mRNA, M	---	AT4G32605	1.71	0.77	5.99	0.000638	0.006248	-0.22
262932_at	glutathione-s-transferase, putative	---	AT1G65820	1.92	0.94	5.98	0.00064	0.006255	-0.22
246322_at	S-locus protein, putative similar to	---	AT1G16680	1.55	0.64	5.98	0.00064	0.006255	-0.22
249585_at	5-oxoprolinase -like protein 5-oxop	OXP1	AT5G37830	1.58	0.66	5.98	0.00064	0.006255	-0.22
258001_at	AIG2-like protein similar to AIG2 pro	---	AT3G28950	1.57	0.65	5.98	0.00064	0.006255	-0.22
253857_at	putative protein hypothetical chloro	---	AT4G27990	1.64	0.71	5.98	0.000641	0.006259	-0.23
265939_at	putative Ta11-like non-LTR retroele	---	AT2G19650	2.08	1.05	5.98	0.000642	0.00626	-0.23
252032_at	putative protein RNA-binding prote	---	AT3G52150	-1.50	-0.59	-5.98	0.000644	0.00628	-0.23
264322_at	unknown protein Contains similarity	---	AT1G04190	1.72	0.78	5.97	0.000646	0.006296	-0.23
259825_at	RNA and export factor binding prot	---	AT1G66260	-1.83	-0.87	-5.97	0.000646	0.006297	-0.23
264356_at	putative DNA repair protein Strong	UVH6	AT1G03190	1.59	0.67	5.97	0.000648	0.006305	-0.24

257685_at	unknown protein	---	AT3G12770	1.76	0.82	5.97	0.000648	0.006305	-0.24
252143_at	putative protein centromere protein	---	AT3G51150	-1.58	-0.66	-5.97	0.000648	0.006307	-0.24
264790_at	putative histidine kinase histidine ki	ATHK1	AT2G17820	1.80	0.85	5.97	0.000649	0.006314	-0.24
257771_at	SNF1 related protein kinase (ATSR	CIPK7	AT3G23000	4.43	2.15	5.97	0.00065	0.006316	-0.24
262385_at	root hair defective, putative similar	---	AT1G72960	-1.47	-0.55	-5.97	0.00065	0.006316	-0.24
251243_at	putative protein hypothetical protei	---	AT3G61870	-1.96	-0.97	-5.97	0.00065	0.006316	-0.24
259361_at	glyceraldehyde-3-phosphate dehyd	GAPC2	AT1G13440	1.69	0.76	5.97	0.000651	0.006316	-0.24
253808_at	predicted proline-rich protein hydro	---	AT4G28300	-1.83	-0.87	-5.97	0.000651	0.006316	-0.24
265776_at	hypothetical protein predicted by ge	---	AT2G07340	1.52	0.60	5.97	0.000651	0.006316	-0.24
248905_at	putative protein contains similarity t	---	AT5G46250	1.59	0.67	5.96	0.000653	0.006329	-0.25
245886_at	ribosomal protein S15-like ribosom	---	AT5G09510	1.48	0.57	5.96	0.000653	0.006329	-0.25
257044_at	hypothetical protein predicted by ge	ARC5	AT3G19720	-1.80	-0.85	-5.96	0.000654	0.006337	-0.25
250409_at	putative protein 110K5.11, unknow	---	AT5G10860	1.60	0.68	5.96	0.000656	0.006347	-0.25
249636_at	beta-glucosidase -like protein prun	BGLU42	AT5G36890	-1.63	-0.70	-5.96	0.000656	0.006347	-0.25
258396_at	unknown protein similar to unknown	---	AT3G15460	2.53	1.34	5.96	0.000656	0.006347	-0.25
267388_at	putative beta-glucosidase	BGLU15	AT2G44450	-2.55	-1.35	-5.95	0.000659	0.00636	-0.26
256982_at	unknown protein	ECT2	AT3G13460	1.60	0.68	5.95	0.00066	0.006374	-0.26
254329_at	putative protein oxysterol-binding p	ORP2A	AT4G22540	-1.67	-0.74	-5.95	0.000661	0.006374	-0.26
254750_at	putative protein	---	AT4G13150	1.83	0.87	5.95	0.000661	0.006374	-0.26
262259_s_a	hypothetical protein predicted by ge	---	AT1G53870	2.13	1.09	5.95	0.000661	0.006374	-0.26
264076_at	unknown protein	---	AT2G28540	1.46	0.55	5.95	0.000662	0.006374	-0.26
257970_at	putative sucrose cleavage protein s	---	AT3G27570	1.67	0.74	5.95	0.000662	0.006374	-0.26
258647_at	unknown protein ; supported by cD	---	AT3G07870	1.66	0.73	5.95	0.000662	0.006374	-0.26
267208_at	putative shaggy-like protein kinase	ASKdZeta	AT2G30980	-1.46	-0.55	-5.95	0.000663	0.006374	-0.26
265718_at	putative WRKY DNA-binding protei	WRKY3	AT2G03340	1.67	0.74	5.95	0.000664	0.006382	-0.26
263480_at	putative root iron transporter protein	ZIP7	AT2G04032	-1.72	-0.78	-5.95	0.000664	0.006382	-0.26
264377_at	similar to early nodulins	---	AT2G25060	-1.51	-0.59	-5.94	0.000667	0.0064	-0.27
251943_at	splicing factor - like protein splicing	RSZ32	AT3G53500	1.47	0.55	5.94	0.000668	0.006413	-0.27
248748_at	putative protein contains similarity t	AMK2	AT5G47840	-1.62	-0.70	-5.94	0.000669	0.006418	-0.27
247593_at	ABC transporter homolog PnATH -	GCN1	AT5G60790	1.66	0.73	5.94	0.00067	0.006423	-0.27
260498_at	putative AP2 domain transcription fa	---	AT2G41710	1.81	0.85	5.94	0.00067	0.006424	-0.27
245865_at	hypothetical protein contains similar	---	AT1G58025	1.57	0.65	5.93	0.000672	0.00643	-0.28
262824_at	putative DNA binding protein simila	RBP45B	AT1G11650	1.46	0.55	5.93	0.000672	0.006432	-0.28
248287_at	putative protein similar to unknown	---	AT5G52970	-1.99	-0.99	-5.93	0.000674	0.006449	-0.28
254301_at	predicted protein predicted protein,	---	AT4G22790	2.06	1.05	5.93	0.000675	0.006455	-0.28

249204_at	unknown protein ;supported by full-	---	AT5G42570	-1.57	-0.65	-5.93	0.000676	0.006458	-0.28
264597_at	unknown protein	---	AT1G04620	1.89	0.92	5.92	0.000678	0.006479	-0.29
266089_at	hypothetical protein predicted by ge	---	AT2G38010	-2.58	-1.37	-5.92	0.000679	0.006479	-0.29
264054_at	unknown protein	SVP	AT2G22540	1.72	0.78	5.92	0.000679	0.006479	-0.29
264258_at	hypothetical protein predicted by ge	---	AT1G09220	1.66	0.73	5.92	0.000681	0.006492	-0.29
246838_at	flap endonuclease - like protein Os	---	AT5G26680	1.62	0.70	5.92	0.000682	0.006499	-0.29
246432_at	RGA-like protein putative member	RGL3	AT5G17490	-2.31	-1.21	-5.92	0.000683	0.006505	-0.29
254457_at	putative protein various predicted p	---	AT4G21170	1.86	0.90	5.91	0.000685	0.006515	-0.30
253629_at	glycine-rich protein glycine-rich cel	---	AT4G30450	-1.78	-0.83	-5.91	0.000685	0.006515	-0.30
251094_at	putative protein ;supported by full-l	---	AT5G01350	1.68	0.75	5.91	0.000685	0.006515	-0.30
264254_at	unknown protein	---	AT1G09150	1.46	0.55	5.91	0.000687	0.006533	-0.30
259434_at	unknown protein	---	AT1G01490	1.55	0.63	5.91	0.000689	0.006542	-0.30
261011_at	cytochrome b5 similar to cytochrom	CB5-A	AT1G26340	1.70	0.77	5.90	0.000691	0.006561	-0.31
253698_at	nucleotide pyrophosphatase - like p	---	AT4G29680	1.57	0.65	5.90	0.000692	0.006562	-0.31
266874_at	unknown protein	---	AT2G44760	1.47	0.56	5.90	0.000692	0.006563	-0.31
265940_at	putative nucleosome assembly prot	NAP1;2	AT2G19480	1.59	0.67	5.90	0.000694	0.006575	-0.31
266348_at	putative MAP kinase	ATMPK17	AT2G01450	-1.83	-0.87	-5.90	0.000694	0.006575	-0.31
264152_at	Mago Nashi-like protein similar to M	MAGO	AT1G02140	1.62	0.70	5.90	0.000695	0.006583	-0.31
255017_at	hypothetical protein ;supported by f	CNX7	AT4G10100	1.61	0.69	5.90	0.000697	0.00659	-0.32
246311_at	high mobility group protein 2-like ; s	HMGB1	AT3G51880	1.68	0.75	5.90	0.000697	0.00659	-0.32
251605_at	receptor-like protein kinase several	---	AT3G57830	-1.74	-0.80	-5.90	0.000697	0.00659	-0.32
260268_at	unknown protein ;supported by full-	---	AT1G68490	1.55	0.63	5.89	0.000698	0.006601	-0.32
255947_at	hypothetical protein contains Pfam	---	AT1G22040	1.59	0.67	5.89	0.000699	0.006603	-0.32
254144_at	putative protein	---	AT4G24690	1.62	0.69	5.89	0.0007	0.006612	-0.32
265896_at	unknown protein	emb2410	AT2G25660	1.62	0.70	5.89	0.000701	0.00662	-0.32
248709_at	unknown protein ;supported by full-	---	AT5G48470	2.28	1.19	5.89	0.000702	0.00662	-0.32
257209_at	hypothetical protein	---	AT3G14920	1.46	0.55	5.89	0.000702	0.006622	-0.33
246602_at	copper amine oxidase, putative sim	---	AT1G31710	-2.49	-1.32	-5.89	0.000703	0.006622	-0.33
253184_at	putative protein protein kinase APK	BSK1	AT4G35230	1.64	0.71	5.89	0.000703	0.006622	-0.33
250678_at	selenium-binding protein-like	---	AT5G06540	1.50	0.58	5.89	0.000703	0.006622	-0.33
261168_at	unknown protein	---	AT1G04940 /	2.35	1.23	5.88	0.000704	0.006624	-0.33
256324_at	MATE efflux family protein, putative	---	AT1G66760	-6.98	-2.80	-5.88	0.000704	0.006624	-0.33
251646_at	putative protein	---	AT3G57780	-5.35	-2.42	-5.88	0.000704	0.006624	-0.33
248700_at	ligand-gated ion channel protein-like	ATGLR1.2	AT5G48400	1.74	0.80	5.88	0.000706	0.006639	-0.33
265431_at	(1-4)-beta-mannan endohydrolase	---	AT2G20680	-1.57	-0.65	-5.88	0.000708	0.006654	-0.33

262791_at	unknown protein EST gb|F13926 co	---	AT1G10830	-1.75	-0.80	-5.88	0.00071	0.006666	-0.34
257062_at	zinc finger protein, putative contain	EMB2454	AT3G18290	-2.85	-1.51	-5.88	0.000711	0.006669	-0.34
245178_at	putative protein similarity to cDNA c	FIS1B	AT5G12390	1.52	0.60	5.88	0.000711	0.006669	-0.34
256336_at	unknown protein	---	AT1G72030	1.86	0.89	5.87	0.000713	0.006681	-0.34
247210_at	annexin ;supported by full-length c	ANNAT2	AT5G65020	-2.64	-1.40	-5.87	0.000715	0.006702	-0.35
256284_at	unknown protein	PSF2	AT3G12530	2.06	1.04	5.87	0.000717	0.006712	-0.35
263674_at	hypothetical protein predicted by ge	---	AT2G04790	1.66	0.73	5.86	0.000721	0.006746	-0.35
246191_at	T-complex protein 1, beta subunit T	---	AT5G20890	1.77	0.83	5.86	0.000721	0.006746	-0.35
246230_at	SCARECROW-like protein	---	AT4G36710	1.56	0.64	5.86	0.000722	0.006753	-0.36
262629_at	heat shock protein, putative contair	ACD32.1	AT1G06460	1.52	0.61	5.86	0.000723	0.006759	-0.36
252941_at	phenylalanyl-trna synthetase - like	---	AT4G39280	1.50	0.59	5.86	0.000724	0.006767	-0.36
262818_at	hypothetical protein predicted by ge	---	AT1G11755 /	1.59	0.67	5.86	0.000725	0.006767	-0.36
262964_at	hypothetical protein predicted by ge	---	AT1G54380	2.03	1.02	5.86	0.000725	0.006767	-0.36
266279_at	putative tropinone reductase	---	AT2G29290	-1.85	-0.88	-5.85	0.000729	0.006799	-0.37
258067_at	putative mitotic checkpoint protein	---	AT3G25980	-1.57	-0.65	-5.85	0.000729	0.006799	-0.37
266839_at	unknown protein	ELF3	AT2G25930	1.52	0.60	5.84	0.000735	0.006853	-0.38
257288_at	anthocyanin 5-aromatic acyltransfe	---	AT3G29670	-1.56	-0.64	-5.84	0.000737	0.006863	-0.38
253537_at	putative protein ; supported by full-	HCF153	AT4G31560	1.66	0.73	5.84	0.000739	0.006879	-0.38
265729_at	putative glucan synthase	ATGSL03	AT2G31960	1.83	0.87	5.84	0.000739	0.006879	-0.38
253035_at	glycosyltransferase like protein alp	CGL1	AT4G38240	1.52	0.61	5.84	0.000739	0.006879	-0.38
254286_at	putative MADS Box / AGL protein M	AGL19	AT4G22950	2.27	1.18	5.83	0.000741	0.006886	-0.38
252667_at	protein kinase-like protein MSTK2S	NEK6	AT3G44200	-1.86	-0.90	-5.83	0.000741	0.006886	-0.38
263799_at	unknown protein ; supported by cD	---	AT2G24550	-2.04	-1.03	-5.83	0.000741	0.006888	-0.38
251257_at	ADP-ribosylation factor-like protein	ATARFA1E	AT3G62290	1.93	0.95	5.83	0.000742	0.006891	-0.39
262058_at	Expressed protein ; supported by fu	---	AT1G79990	-1.44	-0.53	-5.83	0.000743	0.006893	-0.39
246476_at	putative protein predicted proteins -	---	AT5G16730	-1.96	-0.97	-5.83	0.000744	0.006907	-0.39
258054_at	delta tonoplast integral protein (del	DELTA-TIP	AT3G16240	-19.48	-4.28	-5.83	0.000746	0.006919	-0.39
265907_at	unknown protein ;supported by full-	---	AT2G25650	1.71	0.78	5.83	0.000746	0.006919	-0.39
245290_at	hypothetical protein ; supported by c	---	AT4G16490	-1.94	-0.96	-5.82	0.000747	0.006922	-0.39
247996_at	putative protein contains similarity t	---	AT5G56170	-2.26	-1.18	-5.82	0.000748	0.006927	-0.39
251066_at	putative protein RING-H2 finger pro	---	AT5G01880	2.16	1.11	5.82	0.00075	0.006939	-0.40
261649_at	putative clathrin-coat assembly prot	---	AT1G27700	-1.57	-0.65	-5.82	0.000751	0.006945	-0.40
249778_at	Expressed protein ; supported by fu	---	AT5G24165	1.54	0.63	5.82	0.000751	0.006945	-0.40
260996_at	NBS/LRR disease resistance protei	RFL1	AT1G12210	1.86	0.90	5.81	0.000755	0.006977	-0.40
258689_at	putative GTPase activating protein c	---	AT3G07940	-1.51	-0.59	-5.81	0.000755	0.006981	-0.41

260412_at	putative alpha-amylase similar to al	AMY3	AT1G69830	-1.46	-0.54	-5.81	0.000756	0.006981	-0.41
257875_at	hypothetical protein	---	AT3G17120	-1.62	-0.70	-5.81	0.000756	0.006985	-0.41
265831_at	unknown protein	---	AT2G14460	2.83	1.50	5.81	0.000758	0.006997	-0.41
245742_at	aldehyde dehydrogenase, putative	ALDH3H1	AT1G44170	2.08	1.06	5.81	0.000759	0.007	-0.41
254664_at	initiation factor-2Bepsilon-like prote	---	AT4G18300	1.67	0.74	5.81	0.00076	0.007011	-0.41
252426_at	putative protein proline transport he	---	AT3G47630	1.71	0.78	5.81	0.000762	0.007018	-0.41
251800_at	putative protein various predicted p	RBL	AT3G55510	2.57	1.36	5.80	0.000763	0.007024	-0.42
254650_at	hypothetical protein	---	---	-1.49	-0.58	-5.80	0.000764	0.007034	-0.42
259534_at	hypothetical protein	---	AT1G12290	1.73	0.79	5.80	0.000765	0.007038	-0.42
245868_at	hypothetical protein	CAT2	AT1G58030	1.74	0.80	5.80	0.000766	0.007046	-0.42
255455_at	mitochondrial elongation factor Tu	---	AT4G02930	2.59	1.37	5.80	0.000768	0.007058	-0.42
251240_at	putative protein	---	AT3G62450	1.52	0.60	5.79	0.000774	0.007108	-0.43
260886_at	hypothetical protein contains similar	---	AT1G29200	-1.48	-0.56	-5.79	0.000774	0.007108	-0.43
253174_at	catalase ; supported by cDNA: gi_1	CAT2	AT4G35090	-1.73	-0.79	-5.79	0.000775	0.007108	-0.43
246948_at	cytochrome P450-like protein CYT	CYP71B12	AT5G25130	-1.48	-0.56	-5.79	0.000776	0.00712	-0.43
255977_at	ribosomal protein S18, putative sim	---	AT1G34030	1.87	0.90	5.79	0.000777	0.007122	-0.44
258804_at	hypothetical protein similar to hypot	---	AT3G04760	1.89	0.92	5.78	0.000779	0.007135	-0.44
263226_at	unknown protein Contains the PF|0	---	AT1G30690	-1.46	-0.54	-5.78	0.000779	0.007135	-0.44
245512_at	hypothetical protein	---	AT4G15770	2.09	1.06	5.78	0.000781	0.007145	-0.44
261428_at	isochorismate synthase, putative s	ICS2	AT1G18870	-2.13	-1.09	-5.78	0.000781	0.007145	-0.44
252559_at	RNA binding protein -like COLD-IN	---	AT3G46020	1.47	0.55	5.78	0.000781	0.007145	-0.44
266635_at	unknown protein ;supported by full-	---	AT2G35470	-1.79	-0.84	-5.78	0.000782	0.007147	-0.44
250604_at	putative protein heparanase precur	PIP1B	AT2G45960	2.04	1.03	5.78	0.000782	0.007147	-0.44
257174_at	uracil phosphoribosyltransferase, p	---	AT3G27190	1.83	0.87	5.78	0.000783	0.00715	-0.44
253090_at	beta-galactosidase like protein ; sup	BGAL3	AT4G36360	-7.11	-2.83	-5.78	0.000784	0.007155	-0.45
250811_at	cysteine proteinase inhibitor-like pro	---	AT5G05110	2.36	1.24	5.77	0.000785	0.007163	-0.45
258783_at	unknown protein	---	AT3G11760	-1.65	-0.73	-5.77	0.000785	0.007163	-0.45
256908_at	hypothetical protein similar to mucin	---	AT3G24040	-1.64	-0.72	-5.77	0.000786	0.007167	-0.45
253992_at	putative protein ;supported by full-l	---	AT4G26060	1.63	0.70	5.77	0.000787	0.007167	-0.45
254649_at	pherophorin - like protein pheropho	---	AT4G18570	-1.66	-0.73	-5.77	0.000787	0.007167	-0.45
261418_at	mitochondrial ribosomal protein, pu	---	AT1G07830	2.01	1.01	5.77	0.000787	0.007168	-0.45
265984_at	putative limonene cyclase	TPS10	AT2G24210	-1.54	-0.62	-5.77	0.000788	0.007168	-0.45
261301_at	hypothetical protein predicted by ge	---	AT1G48570	2.99	1.58	5.77	0.000788	0.007168	-0.45
252066_at	receptor-protein kinase-like protein	FER	AT3G51550	-2.02	-1.01	-5.77	0.000789	0.007177	-0.45
266983_at	putative phospholipase ; supported	---	AT2G39400	2.05	1.04	5.77	0.00079	0.007183	-0.45

264894_at	unknown protein Location of EST g	---	AT1G23040	1.73	0.79	5.77	0.000791	0.007183	-0.45
262825_at	putative chloroplast prephenate deh	ADT1	AT1G11790	-2.27	-1.18	-5.76	0.000792	0.007195	-0.46
258276_at	putative signal peptidase 21kDa sul	---	AT3G15710	-1.74	-0.80	-5.76	0.000793	0.007195	-0.46
254228_at	putative protein ; supported by full-	---	AT4G23620	1.57	0.65	5.76	0.000793	0.007195	-0.46
251551_at	transcriptional coactivator - like pro	MBF1B	AT3G58680	1.43	0.51	5.76	0.000794	0.007197	-0.46
249672_at	nucleolar protein-like	---	AT5G35910	1.78	0.83	5.76	0.000795	0.007197	-0.46
247284_at	Isp4-like protein ; supported by full-	OPT4	AT5G64410	-2.84	-1.51	-5.76	0.000796	0.007201	-0.46
245291_at	Expressed protein ; supported by cl	---	AT4G16155	-1.58	-0.66	-5.76	0.000796	0.007203	-0.46
261900_at	unknown protein ;supported by full-	---	AT1G80940	1.58	0.66	5.76	0.000796	0.007203	-0.46
260830_at	histone H1, putative similar to histo	---	AT1G06760	2.25	1.17	5.76	0.000797	0.007203	-0.46
261435_at	GTP-binding protein, putative simil	---	AT1G07615	2.19	1.13	5.76	0.000799	0.007218	-0.47
267371_at	Expressed protein ; supported by cl	---	AT2G44510	2.08	1.06	5.76	0.0008	0.007229	-0.47
258333_at	myosin heavy chain-like protein sim	MFP1	AT3G16000	-1.60	-0.67	-5.75	0.000801	0.007237	-0.47
267314_at	putative myosin heavy chain	---	AT2G34730	1.87	0.90	5.75	0.000803	0.007247	-0.47
257017_at	beta-xylosidase, putative similar to	---	AT3G19620	-1.55	-0.63	-5.75	0.000807	0.007276	-0.48
259732_at	putative replication factor C similar	---	AT1G77470	1.64	0.71	5.75	0.000808	0.007276	-0.48
263712_at	Expressed protein ; supported by fu	NFD6	AT2G20585	1.98	0.98	5.74	0.000809	0.007282	-0.48
246747_at	ribosomal protein S21 - like ribosor	---	AT5G27700	1.96	0.97	5.74	0.00081	0.007282	-0.48
261534_at	unknown protein ;supported by full-	PEX11C	AT1G01820	1.48	0.56	5.74	0.000811	0.007282	-0.48
246892_at	putative protein predicted protein, (	---	AT5G25500	1.62	0.70	5.74	0.000811	0.007282	-0.48
249210_at	unknown protein	---	AT5G42670	1.49	0.58	5.74	0.000811	0.007282	-0.48
259077_s_a	reversibly glycosylated polypeptide-	RGP1	AT3G02230	-2.44	-1.29	-5.74	0.000814	0.007299	-0.49
264052_at	putative cytochrome P450	CYP79B3	AT2G22330	-2.50	-1.32	-5.74	0.000815	0.007306	-0.49
258501_at	hypothetical protein predicted by ge	---	AT3G06780	-1.99	-1.00	-5.74	0.000815	0.007307	-0.49
261038_at	unknown protein ; supported by full-	---	AT1G17490	1.74	0.80	5.74	0.000816	0.007308	-0.49
249015_at	Dreg-2 like protein	---	AT5G44730	1.60	0.68	5.74	0.000816	0.007311	-0.49
262299_at	unknown protein Similar to gb|U04	---	AT1G27520	1.56	0.64	5.73	0.000817	0.007312	-0.49
260316_at	hypothetical protein similar to hypot	---	AT1G63810	1.79	0.84	5.73	0.000818	0.007315	-0.49
250897_at	putative protein ; supported by cDN	---	AT5G03430	1.57	0.65	5.73	0.000818	0.007315	-0.49
253826_s_a	E2, ubiquitin-conjugating enzyme 9	UBC9	AT4G27960	1.45	0.53	5.73	0.000819	0.007319	-0.49
253789_at	putative protein predicted proteins,	---	AT4G28570	1.76	0.82	5.73	0.00082	0.007323	-0.49
246559_at	putative protein YTM1 - Homo sapi	---	AT5G15550	2.11	1.08	5.73	0.00082	0.007323	-0.49
255957_at	unknown protein ;supported by full-	---	AT1G22160	3.33	1.74	5.73	0.000821	0.007325	-0.50
255231_at	F-box protein family, AtFBL20 cont	---	AT4G05460	1.51	0.59	5.73	0.000823	0.007342	-0.50
253920_at	histone H2A- like protein histone H	HTA2	AT4G27230	2.00	1.00	5.72	0.000826	0.007364	-0.50

266534_at	putative splicing factor ;supported k	---	AT2G16940	1.49	0.57	5.72	0.000827	0.007366	-0.50
263708_at	unknown protein	---	AT1G09320	1.56	0.64	5.72	0.000827	0.007368	-0.50
260398_at	putative RNA-binding domain conta	APUM23	AT1G72320	2.02	1.01	5.72	0.000829	0.007377	-0.51
261944_at	unknown protein	---	AT1G64650	-1.84	-0.88	-5.72	0.00083	0.007381	-0.51
256400_at	putative RING zinc finger protein c	---	AT3G06140	-1.83	-0.87	-5.72	0.00083	0.007381	-0.51
254010_at	putative protein ;supported by full-l	---	AT1G72125	1.53	0.61	5.71	0.000833	0.007405	-0.51
256786_at	unknown protein ;supported by full-	---	AT3G13740	-1.79	-0.84	-5.71	0.000834	0.007405	-0.51
259597_at	ATP-dependent RNA helicase, put	---	AT1G27900	1.67	0.74	5.71	0.000834	0.007405	-0.51
267278_at	unknown protein ; supported by ful	---	AT2G19350	1.41	0.49	5.71	0.000834	0.007405	-0.51
263098_at	Expressed protein ; supported by fu	---	AT2G16005	-1.49	-0.58	-5.71	0.000835	0.007405	-0.51
262419_at	hypothetical protein identical to hyp	---	AT1G50380	1.86	0.90	5.71	0.000835	0.007407	-0.51
251968_at	putative protein proline-rich protein	---	AT3G53100	-1.86	-0.90	-5.71	0.000836	0.007407	-0.52
265868_at	unknown protein ;supported by full-	PUX2	AT2G01650	1.86	0.90	5.71	0.000836	0.007407	-0.52
262565_at	hypothetical protein predicted by ge	---	AT1G34320	1.59	0.67	5.71	0.000837	0.007407	-0.52
249066_at	WD-repeat protein-like	---	AT5G43920	1.45	0.53	5.71	0.000837	0.007407	-0.52
256160_at	pyruvate dehydrogenase E1 beta s	PDH-E1 BE	AT1G30120	-1.90	-0.93	-5.71	0.000838	0.007412	-0.52
258356_at	unknown protein	---	AT3G14340	-1.72	-0.78	-5.71	0.000839	0.007417	-0.52
254956_at	putative protein various predicted p	---	AT4G10850	-1.49	-0.57	-5.70	0.000843	0.007445	-0.52
264188_at	histone H2A, putative similar to his	GAMMA-H2	AT1G54690	2.58	1.37	5.70	0.000843	0.007447	-0.53
256648_at	ribosomal protein, putative similar t	---	AT3G13580	1.90	0.93	5.70	0.000845	0.007447	-0.53
260323_at	putative U3 small nucleolar ribonuc	IMP4	AT1G63780	2.44	1.29	5.70	0.000845	0.007447	-0.53
263471_at	hypothetical protein predicted by ge	RAP	AT2G31890	1.66	0.73	5.70	0.000845	0.007447	-0.53
252245_at	putative protein various predicted p	---	AT3G49710	2.16	1.11	5.70	0.000845	0.007449	-0.53
252932_at	putative proton pump vacuolar prot	VHA-A3	AT4G39080	-1.58	-0.66	-5.70	0.000847	0.007459	-0.53
249786_at	putative protein strong similarity to u	ABIL3	AT5G24310	2.40	1.26	5.70	0.000849	0.007466	-0.53
254116_at	hypothetical protein	---	AT4G24730	-1.52	-0.60	-5.70	0.000849	0.007466	-0.53
266325_at	putative extensin	---	AT2G46630	-4.58	-2.20	-5.69	0.000851	0.007483	-0.54
255081_at	MLH1 protein ; supported by cDNA	MLH1	AT4G09140	2.23	1.16	5.69	0.000852	0.00749	-0.54
267163_at	unknown protein	---	AT2G37520	1.82	0.86	5.69	0.000855	0.007508	-0.54
247801_at	putative protein ; supported by full-l	---	AT5G58560	-1.59	-0.67	-5.69	0.000855	0.007508	-0.54
252156_at	BCS1 protein-like protein BCS1 pro	---	AT3G50940	1.76	0.82	5.69	0.000855	0.007508	-0.54
252213_at	flavonol synthase - like protein SRC	---	AT3G50210	1.75	0.81	5.69	0.000858	0.007522	-0.54
262953_at	hypothetical protein predicted by ge	---	AT1G75670	2.21	1.14	5.69	0.000858	0.007524	-0.54
264210_at	putative myb-related transcription f	MYB3	AT1G22640	1.98	0.98	5.68	0.00086	0.007535	-0.55
245428_at	putative protein	---	AT4G17570	1.58	0.66	5.68	0.00086	0.007535	-0.55

257495_at	unknown protein	ATPDIL5-1	AT1G07960	1.67	0.74	5.68	0.000861	0.007538	-0.55
251997_at	60S ribosomal protein - like 60S rib	STV1	AT3G53020	1.86	0.90	5.68	0.000861	0.00754	-0.55
260049_at	DNA-directed RNA polymerase sub	NRPA2	AT1G29940	2.43	1.28	5.68	0.000862	0.00754	-0.55
246214_at	heat shock transcription factor HSF	HSF4	AT4G36988	1.87	0.90	5.68	0.000863	0.007549	-0.55
258696_at	hypothetical protein predicted by ge	HCF152	AT3G09650	2.08	1.06	5.68	0.000865	0.007566	-0.55
248473_at	small zinc finger-like protein ;suppo	TIM8	AT5G50810	1.75	0.81	5.68	0.000866	0.007566	-0.55
265315_at	hypothetical protein predicted by ge	CLASP	AT2G20190	-1.56	-0.64	-5.68	0.000867	0.007571	-0.55
255452_at	hypothetical protein similar to A. th	---	AT4G02880	1.50	0.59	5.67	0.000869	0.007585	-0.56
252468_at	starch phosphorylase H (cytosolic f	PHS2	AT3G46970	1.83	0.87	5.67	0.000869	0.007585	-0.56
248854_at	putative protein contains similarity t	---	AT5G46580	1.71	0.77	5.67	0.000869	0.007585	-0.56
261824_at	DAG protein, putative similar to DA	---	AT1G11430	1.47	0.56	5.67	0.00087	0.007587	-0.56
245574_at	hypothetical protein	IQD19	AT4G14750	-1.93	-0.95	-5.67	0.000871	0.00759	-0.56
265088_at	hypothetical protein similar to DNA	SWI2	AT1G03750	1.44	0.52	5.67	0.000871	0.00759	-0.56
249121_at	aluminum-induced protein-like ; su	---	AT5G43830	-1.59	-0.67	-5.67	0.000873	0.007601	-0.56
261952_at	unknown protein	---	AT1G64430	1.43	0.51	5.67	0.000874	0.007603	-0.56
248624_at	unknown protein ; supported by cD	---	AT5G48790	-1.53	-0.61	-5.67	0.000874	0.007603	-0.56
247720_at	dTDP-glucose 4-6-dehydratase - li	UXS3	AT5G59290	-1.69	-0.75	-5.66	0.000876	0.00762	-0.57
260372_at	unknown protein contains a region s	---	AT1G73980	1.54	0.62	5.66	0.000877	0.007624	-0.57
245822_at	unknown protein ; supported by cDI	---	AT1G26110	1.72	0.78	5.66	0.000881	0.007653	-0.57
252913_at	AtRer1A ; supported by full-length	ATRER1A	AT4G39220	-1.55	-0.63	-5.66	0.000882	0.007653	-0.57
244979_at	ribosomal protein S11	---	---	-1.43	-0.52	-5.66	0.000882	0.007653	-0.57
266268_at	unknown protein predicted by gene	---	AT2G29510	-1.50	-0.59	-5.66	0.000883	0.007653	-0.58
262890_at	nucleoporin, putative similar to nuc	NUP155	AT1G14850	1.99	1.00	5.66	0.000883	0.007653	-0.58
247465_at	RNA helicase ; supported by cDNA	PRH75	AT5G62190	2.15	1.10	5.66	0.000884	0.007653	-0.58
267004_at	hypothetical protein predicted by ge	---	AT2G34260	4.02	2.01	5.66	0.000884	0.007653	-0.58
250555_at	putative protein ferredoxin (2Fe-2S	---	AT5G07950	1.91	0.94	5.65	0.000885	0.007653	-0.58
260620_at	unknown protein	---	AT1G08070	1.57	0.65	5.65	0.000885	0.007653	-0.58
250735_at	putative protein similar to unknown	---	AT5G06280	1.58	0.66	5.65	0.000885	0.007653	-0.58
250339_at	NADP dependent malic enzyme - l	ATNADP-M	AT5G11670	1.83	0.87	5.65	0.000886	0.007661	-0.58
251285_at	putative protein several hypothetica	---	AT3G61770	1.56	0.65	5.65	0.000888	0.007668	-0.58
265908_at	contains similarity to S. cerevisiae A	---	AT4G00270	1.95	0.96	5.65	0.000888	0.007668	-0.58
261119_at	chloroplast 50S ribosomal protein L	emb2184	AT1G75350	-1.39	-0.48	-5.65	0.000889	0.007673	-0.58
250326_at	putative protein strong similarity to u	MSL10	AT5G12080	1.86	0.90	5.65	0.00089	0.007683	-0.58
267102_at	putative small nuclear ribonucleopro	LIS	AT2G41500	1.63	0.70	5.65	0.000891	0.007684	-0.58
254357_at	nifU-like protein nifU protein homol	ISU1	AT4G22220	1.40	0.49	5.65	0.000892	0.00769	-0.59

249257_at	unknown protein	---	AT5G41640	-1.41	-0.50	-5.65	0.000892	0.00769	-0.59
265675_at	70kD heat shock protein ;supported	HSP70T-2	AT2G32120	1.86	0.90	5.64	0.000897	0.007719	-0.59
264476_at	hypothetical protein contains similar	PGSIP2	AT1G77130	1.68	0.75	5.64	0.000897	0.007719	-0.59
263332_at	putative snRNP splicing factor ;sup	---	AT2G03870	1.51	0.60	5.64	0.000897	0.007719	-0.59
259305_at	unknown protein ;supported by full-	---	AT3G05070	1.57	0.65	5.64	0.000897	0.007719	-0.59
261922_at	amp-binding protein, putative simila	AAE12	AT1G65890	-1.87	-0.90	-5.64	0.000899	0.007729	-0.59
247600_at	Myb transcription factor homolog (A	MYB34	AT5G60890	-3.28	-1.71	-5.64	0.000901	0.007735	-0.60
266302_at	ethylene-insensitive3-like1 (EIL1) ic	EIL1	AT2G27050	-1.75	-0.81	-5.64	0.000902	0.007744	-0.60
245983_at	5-formyltetrahydrofolate cyclo-ligas	5-FCL	AT5G13050	1.55	0.63	5.63	0.000908	0.007783	-0.61
258426_at	hypothetical protein predicted by ge	PLDP1	AT3G16785	-1.55	-0.64	-5.63	0.000909	0.00779	-0.61
245632_at	unknown protein contains similarity	ATRBL10	AT1G25290	-1.58	-0.66	-5.63	0.00091	0.007794	-0.61
263810_at	putative translation initiation factor e	---	AT2G04520	1.46	0.54	5.63	0.00091	0.007796	-0.61
255344_s_a	putative receptor-like protein kinase	---	AT4G04540 ;	-3.46	-1.79	-5.63	0.000911	0.007796	-0.61
263132_at	unknown protein ;supported by full-	---	AT1G78560	-1.46	-0.54	-5.63	0.000911	0.007799	-0.61
253464_at	putative protein ; supported by full-	---	AT4G32030	-1.80	-0.85	-5.62	0.000913	0.007809	-0.61
248107_at	small Ras-like GTP-binding protein	RAN3	AT5G55190	1.46	0.54	5.62	0.000913	0.007809	-0.61
245933_at	putative protein KIAA1521 - Homo	VPS9B	AT5G09320	1.49	0.57	5.62	0.000913	0.007809	-0.61
262559_at	unknown protein similar to hypothet	---	AT1G31300	-1.64	-0.72	-5.62	0.000914	0.007813	-0.61
258298_at	unknown protein ; supported by cD	---	AT3G23300	-1.84	-0.88	-5.62	0.000915	0.007813	-0.61
262362_at	disease resistance protein, putative	---	AT1G72840	1.52	0.61	5.62	0.000915	0.007816	-0.61
258939_at	unknown protein predicted by gene	---	AT3G10020	-2.51	-1.33	-5.62	0.000917	0.007832	-0.62
262584_at	hypothetical protein Strong similari	---	AT1G15440	2.71	1.44	5.62	0.000918	0.007835	-0.62
264591_at	hypothetical protein contains similar	---	AT2G17670	1.58	0.66	5.62	0.000919	0.007837	-0.62
260981_at	hypothetical protein predicted by ge	---	AT1G53460	1.72	0.79	5.62	0.00092	0.007844	-0.62
266407_at	putative elongation factor	TFIIS	AT2G38560	1.54	0.62	5.62	0.00092	0.007844	-0.62
251915_at	putative protein peroxisomal Ca-de	---	AT3G53940	1.78	0.83	5.62	0.000921	0.007847	-0.62
251630_at	putative protein unknown protein -	---	AT3G57420	-1.48	-0.57	-5.61	0.000923	0.007863	-0.62
256062_at	unknown protein ;supported by full-	LSH6	AT1G07090	1.49	0.58	5.61	0.000925	0.007874	-0.63
263831_at	putative ferritin	ATFER4	AT2G40300	2.05	1.03	5.61	0.000925	0.007874	-0.63
250776_at	monooxygenase ;supported by full-	---	AT5G05320	1.47	0.55	5.61	0.000926	0.007874	-0.63
266298_at	unknown protein ;supported by full-	---	AT2G29590	1.70	0.76	5.61	0.000926	0.007874	-0.63
245745_at	unknown protein contains similarity	---	AT1G51110	-1.96	-0.97	-5.61	0.000927	0.007877	-0.63
248767_at	putative protein contains similarity t	---	AT5G47710	1.66	0.74	5.61	0.000927	0.007878	-0.63
259164_at	hypothetical protein contains brom	ATBET10	AT3G01770	1.86	0.90	5.61	0.000929	0.00789	-0.63
251117_at	putative protein predicted protein, (	---	AT3G63390	1.73	0.79	5.61	0.000929	0.00789	-0.63

246012_at	Pspzf zinc finger protein - like DNA	---	AT5G10650	1.97	0.98	5.60	0.000931	0.007901	-0.63
255567_at	hypothetical protein ; supported by	---	AT4G01150	-1.57	-0.65	-5.60	0.000933	0.007906	-0.63
251018_at	60S ribosomal protein - like 60S rib	---	AT5G02450	1.91	0.94	5.60	0.000934	0.007915	-0.64
260236_at	geranylgeranyl reductase identical	---	AT1G74470	-1.45	-0.53	-5.60	0.000937	0.00794	-0.64
245521_at	Expressed protein ;supported by cD	ESD4	AT4G15880	1.51	0.60	5.59	0.000941	0.007965	-0.64
247000_at	casein kinase II alpha subunit ; sup	CKA1	AT5G67380	1.70	0.77	5.59	0.000943	0.007979	-0.65
262051_at	putative coatomer protein complex	---	AT1G79990	-1.72	-0.78	-5.59	0.000944	0.007985	-0.65
255623_at	putative L5 ribosomal protein ;supp	---	AT4G01310	-1.52	-0.60	-5.59	0.000945	0.007988	-0.65
246378_at	integral membrane protein, putative	---	AT1G57620	-1.49	-0.57	-5.59	0.000947	0.008002	-0.65
266510_at	unknown protein	SWA1	AT2G47990	2.54	1.34	5.59	0.000949	0.008016	-0.65
256934_at	unknown protein contains Pfam pro	---	AT3G22530	1.68	0.75	5.58	0.000949	0.008016	-0.65
246613_at	acetyl-CoA carboxylase ;supported	CAC2	AT5G35360	-1.77	-0.83	-5.58	0.00095	0.00802	-0.66
247403_at	putative protein hypersensitive-indu	---	AT5G62740	1.55	0.63	5.58	0.000957	0.008073	-0.66
250297_at	putative protein predicted protein, [	---	AT5G11980	-1.66	-0.73	-5.58	0.000958	0.008074	-0.66
263482_at	putative GDSL-motif lipase/hydrolas	---	AT2G03980	-8.09	-3.02	-5.58	0.000958	0.008077	-0.66
258651_at	putative phosphatidylinositol-4-phos	PIP5K9	AT3G09920	-1.68	-0.75	-5.58	0.000959	0.008081	-0.67
250559_at	putative protein condensin subunit	---	AT5G08010	1.67	0.74	5.57	0.00096	0.008087	-0.67
263598_at	putative xyloglucan-specific glucana	EXGT-A3	AT2G01850	-1.62	-0.69	-5.57	0.000961	0.008087	-0.67
247075_at	putative protein contains similarity t	PLP3b	AT5G66410	-1.67	-0.74	-5.57	0.000961	0.008087	-0.67
249965_at	putative protein predicted proteins,	---	AT5G19020	1.40	0.48	5.57	0.000961	0.008087	-0.67
245137_at	unknown protein	---	AT2G45460	1.80	0.85	5.57	0.000962	0.008087	-0.67
260360_at	chorismate mutase, putative contai	CM3	AT1G69370	-1.86	-0.89	-5.57	0.000962	0.008091	-0.67
256249_at	26S proteasome regulatory subuni	MEE34	AT3G11270	1.59	0.67	5.57	0.000963	0.008091	-0.67
252496_at	putative protein several hypothetica	CRR2	AT3G46790	2.01	1.01	5.57	0.000963	0.008093	-0.67
249177_at	unknown protein ;supported by full-	---	AT5G42850	1.53	0.62	5.57	0.000966	0.008112	-0.67
252968_at	putative protein unknown mRNA, H	---	AT4G38890	1.94	0.96	5.57	0.000967	0.008115	-0.67
261149_s_a	GSH-dependent dehydroascorbate	DHAR1	AT1G19550	-2.14	-1.10	-5.57	0.000968	0.008118	-0.68
262940_at	unknown protein	---	AT1G79520	1.99	0.99	5.56	0.000969	0.008126	-0.68
249222_at	putative protein similar to unknown	---	AT5G42450	1.43	0.51	5.56	0.00097	0.008127	-0.68
264849_at	putative ribosomal protein S4 conta	---	AT2G17360	1.71	0.77	5.56	0.00097	0.008128	-0.68
261266_at	expansin 10 identical to GB:AAF61	ATEXPA10	AT1G26770	-1.89	-0.91	-5.56	0.00097	0.008128	-0.68
255789_at	60S ribosomal protein L23 ;suppor	---	AT2G33370	3.12	1.64	5.56	0.000973	0.008143	-0.68
254639_at	putative protein various predicted p	DOT4	AT4G18750	1.60	0.68	5.56	0.000974	0.008146	-0.68
265760_at	unknown protein	---	AT2G01220	1.58	0.66	5.56	0.000974	0.008146	-0.68
260819_at	b-Zip DNA binding protein, putative	AtbZIP52	AT1G06850	-2.33	-1.22	-5.56	0.000977	0.008166	-0.69

261972_at	hypothetical protein predicted by ge	---	AT1G64600	2.13	1.09	5.55	0.000979	0.008181	-0.69
264031_at	unknown protein	---	AT2G03780	1.70	0.77	5.55	0.000979	0.008181	-0.69
248289_at	unknown protein ; supported by cD	---	AT5G52880	1.51	0.59	5.55	0.00098	0.008184	-0.69
256398_at	putative major intrinsic protein conta	NIP7;1	AT3G06100	-1.42	-0.51	-5.55	0.000982	0.008192	-0.69
260852_at	transmembrane protein, putative si	---	AT1G21900	1.47	0.56	5.55	0.000983	0.008197	-0.69
246235_at	putative protein	---	AT4G36830	-1.70	-0.76	-5.55	0.000986	0.008213	-0.70
264086_at	unknown protein ;supported by full-	---	AT2G31190	1.77	0.82	5.55	0.000986	0.008213	-0.70
248049_at	putative protein contains similarity t	COX15	AT5G56090	2.66	1.41	5.55	0.000986	0.008213	-0.70
250244_at	putative protein similar to unknown	ABO1	AT5G13680	2.12	1.08	5.55	0.000988	0.008227	-0.70
247342_at	ARI-like RING zinc finger protein-lik	---	AT5G63730	-1.52	-0.61	-5.54	0.00099	0.008241	-0.70
244916_at	hypothetical protein	ORF187	ATMG00880	-1.49	-0.58	-5.54	0.000991	0.008248	-0.70
256430_at	DREB2B transcription factor identic	DREB2B	AT3G11020	1.53	0.62	5.54	0.000992	0.008252	-0.70
250818_at	Expressed protein ; supported by cl	ALA1	AT5G04930	1.84	0.88	5.54	0.000992	0.008252	-0.70
260760_at	hypothetical protein predicted by ge	---	AT1G49170	1.75	0.81	5.54	0.000993	0.008252	-0.70
267179_at	putative ADP ribosylation factor 1 G	AGD7	AT2G37550	-1.91	-0.93	-5.54	0.000996	0.008274	-0.71
256482_at	protein kinase, putative contains Pf	FEI1	AT1G31420	-1.55	-0.63	-5.54	0.000998	0.008287	-0.71
252633_x_a	putative protein HISTONE DEACET	hda17	AT3G44490	1.42	0.50	5.54	0.000999	0.008294	-0.71
264264_at	unknown protein ESTs gb|T04610,	---	AT1G09250	-2.67	-1.41	-5.53	0.001	0.008301	-0.71
264828_at	hypothetical protein predicted by ge	ATATG18G	AT1G03380	1.86	0.89	5.53	0.001001	0.008303	-0.71
260440_at	hypothetical protein predicted by ge	---	AT1G68160	1.52	0.60	5.53	0.001005	0.008332	-0.72
251676_at	hypothetical protein	---	AT3G57320	-1.92	-0.94	-5.53	0.001006	0.008341	-0.72
250832_at	nicotianamine synthase (dbj|BAA74	NAS1	AT5G04950	5.45	2.45	5.53	0.001008	0.008354	-0.72
261765_at	cyclin, putative similar to cyclin GI:	CYCA2;3	AT1G15570	-1.61	-0.69	-5.52	0.00101	0.008368	-0.72
245249_at	acyl-CoA oxidase like protein ; supp	ACX1	AT4G16760	-1.88	-0.91	-5.52	0.001011	0.008369	-0.72
261415_at	globulin-like protein similar to GB:7	---	AT1G07750	-2.33	-1.22	-5.52	0.001014	0.008384	-0.73
257984_at	putative cell differentiation protein s	---	AT3G20800	1.80	0.85	5.52	0.001014	0.008384	-0.73
262633_at	Expressed protein ; supported by fu	---	AT1G06630	-1.48	-0.57	-5.52	0.001016	0.008396	-0.73
251945_at	dTDP-glucose 4-6-dehydratase -lik	UXS1	AT3G53520	-1.54	-0.62	-5.52	0.001019	0.008422	-0.73
253561_at	aspartate--tRNA ligase - like protei	---	AT4G31180	1.49	0.57	5.51	0.001028	0.008488	-0.74
257619_at	unknown protein	ICK3	AT3G24810	1.65	0.72	5.51	0.001029	0.00849	-0.74
254476_at	putative protein gamma-SNAP prot	GSNAP	AT4G20410	-1.50	-0.59	-5.51	0.001029	0.00849	-0.74
260523_at	putative salt-inducible protein	EMB2654	AT2G41720	1.88	0.91	5.51	0.00103	0.00849	-0.74
254333_at	predicted protein predicted protein	SMO1-3	AT4G22753	-3.77	-1.92	-5.51	0.00103	0.00849	-0.74
262049_at	unknown protein ;supported by full-	---	AT1G80180	1.63	0.70	5.51	0.00103	0.00849	-0.74
264484_at	hypothetical protein similar to ankyr	---	AT1G77260	-1.49	-0.58	-5.50	0.001031	0.00849	-0.74

250857_at	unknown protein	---	AT5G04790	-1.89	-0.92	-5.50	0.001034	0.008506	-0.75
261613_at	abscisic acid responsive elements-I	ABF1	AT1G49720	1.97	0.98	5.50	0.001035	0.008516	-0.75
261296_at	unknown protein	---	AT1G48460	2.28	1.19	5.50	0.001037	0.008526	-0.75
251469_at	putative protein strictosidine syntha	---	AT3G59530	-1.48	-0.57	-5.50	0.001037	0.008526	-0.75
258747_at	unknown protein ; supported by ful	---	AT3G05810	1.82	0.87	5.50	0.001038	0.008532	-0.75
265872_at	unknown protein ;supported by full-	atnudt17	AT2G01670	1.92	0.94	5.50	0.001039	0.008532	-0.75
258810_at	hypothetical protein similar to hypot	---	AT3G03970	-1.63	-0.71	-5.50	0.00104	0.008539	-0.75
247048_at	photoreceptor-interacting protein-lik	---	AT5G66560	-2.55	-1.35	-5.50	0.001041	0.008539	-0.75
253843_at	putative protein MLL protein, Gallu	SDG16	AT4G27910	-1.44	-0.53	-5.49	0.001041	0.008539	-0.75
252971_at	extensin - like protein proline-rich p	PRP4	AT4G38770	-16.95	-4.08	-5.49	0.001041	0.00854	-0.76
249047_at	berberine bridge enzyme-like protei	---	AT5G44410	1.62	0.69	5.49	0.001043	0.008554	-0.76
251683_at	putative protein several hypothetica	---	AT3G57120	-2.59	-1.37	-5.49	0.001046	0.008575	-0.76
263010_at	T76725 come from this gene.	---	AT1G23330	1.48	0.56	5.49	0.001048	0.008582	-0.76
247498_at	peroxisomal Ca-dependent solute (	---	AT5G61810	-1.77	-0.82	-5.49	0.001048	0.008582	-0.76
258355_at	hypothetical protein predicted by ge	---	AT3G14330	1.89	0.92	5.49	0.001048	0.008582	-0.76
260383_s_a	putative 60S ribosomal protein L6 s	---	AT1G74050	1.84	0.88	5.49	0.00105	0.008589	-0.76
260430_at	putative zinc finger protein similar to	---	AT1G68200	-1.45	-0.54	-5.49	0.00105	0.008589	-0.76
252737_at	putative protein KIAA0274 protein,	---	AT3G43220	-1.49	-0.58	-5.49	0.001051	0.008595	-0.77
255077_at	putative protein	---	AT4G09150	-2.00	-1.00	-5.48	0.001056	0.00863	-0.77
267515_at	putative PCF2-like DNA binding pro	---	AT2G45680	-1.70	-0.77	-5.48	0.001057	0.008639	-0.77
251860_at	Gluthatione reductase, chloroplast	GR	AT3G54660	-1.42	-0.50	-5.48	0.001059	0.008651	-0.77
256785_at	unknown protein ;supported by full-	PRA8	AT3G13720	-1.86	-0.89	-5.48	0.00106	0.008653	-0.77
259225_at	putative small nuclear ribonucleopro	---	AT3G07590	3.08	1.62	5.48	0.001061	0.008657	-0.78
251888_at	putative protein GTP-binding regula	---	AT3G54190	1.45	0.54	5.48	0.001062	0.008662	-0.78
266728_at	similar to late embryogenesis abund	---	AT2G03140	1.53	0.61	5.48	0.001062	0.008663	-0.78
265968_at	putative protein translocase ; suppo	ATTIM17-2	AT2G37410	1.98	0.98	5.47	0.001063	0.008666	-0.78
256140_at	hypothetical protein contains similar	---	AT1G48650	1.92	0.94	5.47	0.001063	0.008666	-0.78
249016_at	putative protein contains similarity t	REV1	AT5G44750	1.76	0.82	5.47	0.001065	0.008672	-0.78
253777_at	SOF1 protein-like protein SOF1 (inv	---	AT4G28450	3.00	1.58	5.47	0.001065	0.008672	-0.78
251309_at	putative protein carbonyl reductase	---	AT3G61220	1.67	0.74	5.47	0.001066	0.008672	-0.78
261854_at	unknown protein	---	AT1G50670	-1.41	-0.50	-5.47	0.001066	0.008672	-0.78
246028_at	AKIN beta1 ; supported by full-leng	---	AT5G21170	-2.00	-1.00	-5.47	0.001067	0.008678	-0.78
265078_at	hypothetical protein contains similar	ECT4	AT1G55500	2.30	1.20	5.47	0.001067	0.008678	-0.78
250192_at	pescadillo - like protein embryonic	---	AT5G14520	2.00	1.00	5.47	0.001068	0.008678	-0.78
252095_at	epoxide hydrolase-like protein epox	---	AT3G51000	1.72	0.78	5.47	0.001071	0.008697	-0.79

263419_at	putative protein kinase contains a p	---	AT2G17220	1.90	0.92	5.47	0.001072	0.008701	-0.79
265735_at	putative aldolase ;supported by full	---	AT2G01140	1.92	0.94	5.47	0.001072	0.008702	-0.79
247249_at	putative protein similar to unknown	---	AT5G64670	1.86	0.90	5.46	0.001074	0.008709	-0.79
246196_at	hypothetical protein ;supported by f	---	AT4G37090	1.87	0.91	5.46	0.001074	0.00871	-0.79
265398_at	unknown protein	ATMAK10	AT2G11000	1.55	0.64	5.46	0.001075	0.008716	-0.79
251143_at	putative protein SqdX protein, Syne	SQD2	AT5G01220	2.37	1.24	5.46	0.001078	0.008732	-0.79
251617_at	putative protein hypothetical protei	---	AT3G58000	-1.49	-0.58	-5.46	0.001078	0.008732	-0.79
253668_at	snRNP Sm protein F - like Sm pro	RUXF	AT4G30220	1.66	0.73	5.46	0.00108	0.008741	-0.79
245002_at	PSII D2 protein	---	---	-1.72	-0.78	-5.46	0.00108	0.008743	-0.80
247398_at	putative protein predicted protein, A	---	AT5G62950	1.67	0.74	5.46	0.001082	0.008752	-0.80
253397_at	putative protein kinase protein kina	---	AT4G32710	-1.46	-0.54	-5.46	0.001082	0.008752	-0.80
255887_at	hypothetical protein contains Pfam	---	AT1G20370	1.45	0.54	5.46	0.001083	0.008752	-0.80
261973_at	hypothetical protein predicted by ge	---	AT1G64610	1.93	0.95	5.46	0.001083	0.008752	-0.80
267013_at	putative protein kinase contains a p	CCR2	AT2G39180	-1.57	-0.65	-5.46	0.001083	0.008755	-0.80
259912_at	hypothetical protein	iqd8	AT1G72670	-1.69	-0.76	-5.46	0.001084	0.008755	-0.80
260729_at	regulatory protein, putative contain	KU80	AT1G48050	1.44	0.53	5.45	0.001085	0.008763	-0.80
257515_at	zinc finger protein, putative similar	DOT5	AT1G13290	-1.49	-0.57	-5.45	0.001088	0.008777	-0.80
258478_at	unknown protein	---	AT3G02710	1.54	0.62	5.45	0.001088	0.008777	-0.80
265987_at	unknown protein	---	AT2G24240	1.98	0.99	5.45	0.00109	0.008784	-0.80
251391_at	putative protein weak homology wi	---	AT3G60910	1.68	0.75	5.45	0.00109	0.008784	-0.80
256853_at	hypothetical protein predicted by ge	---	AT3G18640	1.49	0.57	5.45	0.001092	0.0088	-0.81
253946_at	putative APG protein proline-rich p	---	AT4G26790	-1.99	-0.99	-5.45	0.001093	0.0088	-0.81
256568_at	hypothetical protein ;supported by	---	AT3G19520	1.51	0.59	5.45	0.001093	0.0088	-0.81
248237_at	receptor protein kinase-like protein	---	AT5G53890	1.73	0.79	5.45	0.001094	0.0088	-0.81
263621_at	putative DNA-directed RNA polyme	NRPB6B	AT2G04630	1.58	0.66	5.45	0.001094	0.0088	-0.81
265474_at	Expressed protein ; supported by cl	---	AT2G15690	2.09	1.07	5.45	0.001094	0.0088	-0.81
253679_at	cytidine deaminase 6 (CDA6)	---	AT4G29610	-2.88	-1.53	-5.45	0.001094	0.0088	-0.81
262534_at	unknown protein contains Pfam pro	SHA	AT1G17040	1.81	0.85	5.44	0.001096	0.008807	-0.81
255734_at	hypothetical protein similar to putati	---	AT1G25550	1.87	0.90	5.44	0.001097	0.008809	-0.81
264676_at	unknown protein EST gb|R64758 co	---	AT1G09870	1.45	0.53	5.44	0.001098	0.008816	-0.81
265344_at	unknown protein predicted by gene	---	AT2G22660	1.87	0.90	5.44	0.001101	0.008836	-0.82
245695_at	rec - like protein	---	AT5G04270	-1.57	-0.65	-5.44	0.001105	0.008857	-0.82
265325_at	putative integral membrane protein	---	AT2G18240	1.75	0.80	5.44	0.001105	0.008857	-0.82
261268_at	unknown protein similar to 50S ribo	---	AT1G26740	1.65	0.72	5.44	0.001105	0.008857	-0.82
248974_at	disease resistance protein-like	---	AT5G45060	1.50	0.59	5.43	0.001107	0.008868	-0.82

249067_at	putative protein similar to unknown	---	AT5G43960	1.55	0.63	5.43	0.001108	0.008871	-0.82
248625_at	3-keto-acyl-CoA thiolase 2 (gb|AAC	PKT2	AT5G48880	-2.43	-1.28	-5.43	0.001109	0.008876	-0.82
256397_at	putative dual-specificity protein pho	MKP2	AT3G06110	1.50	0.58	5.43	0.001109	0.008876	-0.82
255020_at	isoleucine-tRNA ligase - like protei	---	AT4G10320	1.40	0.48	5.43	0.00111	0.008876	-0.83
258323_at	unknown protein similar to putative	---	AT3G22750	1.84	0.88	5.43	0.00111	0.008876	-0.83
245486_x_a	glycine-rich protein homolog	---	AT4G16240	-1.54	-0.62	-5.43	0.001112	0.008881	-0.83
246034_at	putative protein FH protein interact	---	AT5G08350	-2.11	-1.07	-5.43	0.001112	0.008881	-0.83
264701_at	hypothetical protein similar to hypot	---	AT1G70160	1.59	0.67	5.43	0.001112	0.008881	-0.83
267219_at	putative transport protein ;supporte	---	AT2G02590	-1.82	-0.87	-5.43	0.001113	0.008886	-0.83
253143_at	putative protein	---	AT4G35530	1.72	0.78	5.43	0.001115	0.008897	-0.83
259193_at	putative thylakoid lumen rotamase s	CYP38	AT3G01480	-1.87	-0.91	-5.43	0.001116	0.008897	-0.83
256117_at	unknown protein ; supported by cDI	---	AT1G16840	-1.46	-0.55	-5.43	0.001117	0.008901	-0.83
257444_at	hypothetical protein predicted by ge	---	AT2G12550	1.68	0.75	5.43	0.001117	0.008901	-0.83
252615_at	serine/proline-rich protein	---	AT3G45230	-1.98	-0.99	-5.43	0.001117	0.008901	-0.83
258568_at	unknown protein contains Pfam pro	---	AT3G04470	-1.54	-0.62	-5.43	0.001118	0.008905	-0.83
253733_at	hypothetical protein	ATMND1	AT4G29170	1.66	0.73	5.42	0.00112	0.008914	-0.83
264205_at	hypothetical protein identical to hyp	---	AT1G22790	1.46	0.54	5.42	0.00112	0.008917	-0.84
260656_at	hypothetical protein predicted by ge	---	AT1G19380	-5.21	-2.38	-5.42	0.001122	0.00892	-0.84
266359_at	putative phospholipid cytidylyltransf	CCT1	AT2G32260	1.49	0.57	5.42	0.001122	0.00892	-0.84
266661_at	TINY-like AP2 domain transcription	---	AT2G25820	-1.66	-0.73	-5.42	0.001122	0.00892	-0.84
264183_at	unknown protein	CLV2	AT1G65380	1.61	0.69	5.42	0.001123	0.008927	-0.84
262737_at	hypothetical protein	SRD2	AT1G28560	1.65	0.72	5.42	0.001125	0.008931	-0.84
262173_at	betaine aldehyde dehydrogenase,	ALDH10A8	AT1G74920	-1.59	-0.67	-5.42	0.001125	0.008931	-0.84
250353_at	putative protein predicted proteins,	---	AT5G11630	1.64	0.71	5.42	0.001125	0.008931	-0.84
250381_at	putative protein various predicted p	---	AT5G11610	2.26	1.18	5.42	0.001126	0.008935	-0.84
257835_at	cytochrome P450 monooxygenase	CYP82G1	AT3G25180	-3.83	-1.94	-5.42	0.001127	0.008935	-0.84
251174_at	putative protein latex protein allerg	PLP9	AT3G63200	-2.45	-1.29	-5.42	0.001128	0.00894	-0.84
255089_at	nucleoside-diphosphate kinase ;su	NDPK1	AT4G09320	1.88	0.91	5.42	0.001128	0.00894	-0.84
250470_at	(3R)-hydroxymyristoyl-[acyl carrier	---	AT5G10160	-1.71	-0.77	-5.42	0.001128	0.00894	-0.84
265724_at	hypothetical protein predicted by ge	OFP16	AT2G32100	-1.94	-0.96	-5.42	0.001129	0.008943	-0.84
252649_at	putative protein histone deacetylas	HDA9	AT3G44680	1.43	0.51	5.41	0.00113	0.008946	-0.84
262112_at	unknown protein ;supported by full-	---	AT1G02870	1.80	0.85	5.41	0.00113	0.008946	-0.84
261377_at	unknown protein ;supported by full-	---	AT1G18850	2.50	1.32	5.41	0.001132	0.008953	-0.85
253222_at	chalcone synthase - like protein ch	---	AT4G34850	-1.41	-0.49	-5.41	0.001132	0.008953	-0.85
264175_at	unknown protein Similar to rice cha	---	AT1G02050	1.70	0.76	5.41	0.001132	0.008953	-0.85

255527_at	putative protein	---	AT4G02360	-5.20	-2.38	-5.41	0.001134	0.008953	-0.85
260245_at	putative RNA-binding protein simila	GR-RBP5	AT1G74230	1.44	0.52	5.41	0.001134	0.008953	-0.85
249014_at	putative protein similar to unknown	---	AT5G44710	1.71	0.78	5.41	0.001134	0.008953	-0.85
257712_at	hypothetical protein predicted by ge	---	AT3G27420	-1.40	-0.49	-5.41	0.001135	0.008958	-0.85
259782_at	unknown protein	---	AT1G29680	-1.44	-0.52	-5.41	0.001138	0.008972	-0.85
253876_at	COP1-interacting protein 7 (CIP7)	CIP7	AT4G27430	-1.99	-1.00	-5.41	0.001138	0.008972	-0.85
255872_at	putative protein kinase contains a p	SIP4	AT2G30360	-3.03	-1.60	-5.41	0.001139	0.008976	-0.85
249164_at	U2 snRNP auxiliary factor, small su	U2AF35B	AT5G42820	1.66	0.73	5.41	0.00114	0.00898	-0.85
258814_at	putative short-chain type dehydroge	---	AT3G03980	-1.63	-0.71	-5.41	0.001141	0.008987	-0.86
245144_at	putative methionine aminopeptidase	MAP1A	AT2G45240	1.58	0.66	5.40	0.001144	0.009003	-0.86
262064_at	elongation factor, putative similar to	LOS1	AT1G56070	1.63	0.71	5.40	0.001144	0.009003	-0.86
265539_at	unknown protein	---	AT2G15830	3.04	1.60	5.40	0.001146	0.009014	-0.86
264027_at	putative AAA-type ATPase similar t	CDC48B	AT2G03670	1.79	0.84	5.40	0.00115	0.009042	-0.86
261392_at	hypothetical protein predicted by ge	---	AT1G79780	-1.46	-0.55	-5.40	0.001151	0.009044	-0.86
260665_at	unknown protein ;supported by full-	---	AT1G19360	1.59	0.67	5.40	0.001152	0.009044	-0.87
247325_at	putative protein contains similarity t	SC35	AT5G64200	1.47	0.56	5.40	0.001152	0.009044	-0.87
250518_at	Expressed protein ; supported by cl	---	AT5G08139	1.44	0.53	5.40	0.001152	0.009044	-0.87
262313_at	unknown protein ;supported by full-	---	AT1G70900	1.88	0.91	5.40	0.001153	0.009051	-0.87
264908_at	unknown protein	---	AT2G17440	1.53	0.62	5.39	0.001157	0.009057	-0.87
258134_at	rubisco expression protein, putative	---	AT3G24530	-1.84	-0.88	-5.39	0.001157	0.009057	-0.87
260545_at	putative glutathione peroxidase ;su	ATGPX3	AT2G43350	1.58	0.66	5.39	0.001157	0.009057	-0.87
248006_at	unknown protein	HAP8	AT5G56250	-1.52	-0.61	-5.39	0.001157	0.009057	-0.87
255087_at	putative protein proteins of this bac	---	AT4G09340	1.46	0.55	5.39	0.001158	0.009057	-0.87
250546_at	nhp2-like protein high mobility group	---	AT5G08180	2.39	1.26	5.39	0.001158	0.009057	-0.87
249318_at	uridine kinase-like protein	AtUK/UPRT	AT5G40870	1.53	0.62	5.39	0.001161	0.009078	-0.87
259763_at	unknown protein similar to GB:AAB	---	AT1G77630	-1.94	-0.96	-5.39	0.001161	0.009078	-0.87
248386_at	DNA-directed RNA polymerase II su	NRPB6A	AT5G51940	1.57	0.65	5.39	0.001162	0.009081	-0.88
263251_at	unknown protein ; supported by cD	---	AT2G31410	2.38	1.25	5.39	0.001163	0.009083	-0.88
258883_at	unknown protein predicted by gens	---	AT3G09970	1.61	0.69	5.39	0.001164	0.009083	-0.88
261782_at	unknown protein	---	AT1G76110	-1.74	-0.80	-5.39	0.001165	0.009087	-0.88
267096_at	unknown protein ; supported by cD	---	AT2G38180	2.30	1.20	5.38	0.001168	0.009102	-0.88
250666_at	SPF1-like protein SPF1 protein - Ip	WRKY26	AT5G07100	2.66	1.41	5.38	0.001168	0.009102	-0.88
249235_at	beta-1,3-glucanase-like protein ;su	BG_PPAP	AT5G42100	-1.38	-0.46	-5.38	0.001169	0.009111	-0.88
267378_at	hypothetical protein	---	AT2G26200	1.44	0.52	5.38	0.001169	0.009111	-0.88
258253_at	putative short chain alcohol dehydro	---	AT3G26760	-1.48	-0.56	-5.38	0.00117	0.009114	-0.88

259981_at	unknown protein ; supported by ful	---	AT1G76450	-1.67	-0.74	-5.38	0.001171	0.009118	-0.88
251536_at	ketol-acid reductoisomerase ; supp	---	AT3G58610	1.58	0.66	5.38	0.001173	0.009124	-0.89
258093_at	unknown protein ; supported by cD	---	AT3G14750	1.67	0.74	5.38	0.001173	0.009124	-0.89
247558_at	histone deacetylase - like histone c	HDA05	AT5G61060	1.54	0.62	5.38	0.001174	0.009124	-0.89
245010_at	NADH dehydrogenase subunit	---	---	-1.64	-0.72	-5.38	0.001174	0.009124	-0.89
255116_at	receptor protein kinase-like protein	---	AT4G08850	1.84	0.88	5.38	0.001174	0.009124	-0.89
258545_at	putative GTPase contains Pfam pro	---	AT3G07050	2.06	1.04	5.38	0.001175	0.009126	-0.89
258295_at	unknown protein ;supported by full-	---	AT3G23400	-1.91	-0.93	-5.38	0.001177	0.009136	-0.89
262807_at	unknown protein strong similarity to	---	AT1G11740	-1.75	-0.81	-5.38	0.001178	0.009144	-0.89
256268_at	retinoblastoma-related protein, puta	RBR1	AT3G12280	1.75	0.80	5.37	0.00118	0.009154	-0.89
252969_at	putative protein vsf-1 protein, Lyco	---	AT4G38900	-1.49	-0.58	-5.37	0.001181	0.009163	-0.89
246566_at	oligopeptide transporter -like protei	---	AT5G14940	2.25	1.17	5.37	0.001182	0.009164	-0.89
265825_at	ubiquitin-like (UBQ7), putative simi	UBQ7	AT2G35635	1.58	0.66	5.37	0.001186	0.009189	-0.90
258988_at	unknown protein similar to unknowr	---	AT3G08890	-1.56	-0.64	-5.37	0.001186	0.009189	-0.90
264299_s_a	hypothetical protein predicted by ge	---	AT1G78850	-2.76	-1.46	-5.37	0.001187	0.009189	-0.90
251124_s_a	laccase -like protein lac110 laccase	LAC8	AT5G01040	-7.86	-2.97	-5.37	0.001187	0.009189	-0.90
246041_at	phospholipase - like protein various	---	AT5G19290	-1.42	-0.51	-5.37	0.001189	0.009201	-0.90
244944_s_a	ribosomal protein L16	---	---	1.86	0.90	5.36	0.001192	0.00922	-0.90
267338_at	26S proteasome regulatory subunit	EIF2	AT2G39990	1.64	0.71	5.36	0.001193	0.009224	-0.90
246976_s_a	putative protein UBIQUINONE BIO	---	AT5G24810	-1.98	-0.99	-5.36	0.001193	0.009224	-0.90
253578_at	putative protein diacylglycerol kina	ATDGK7	AT4G30340	-1.91	-0.93	-5.36	0.001194	0.009224	-0.90
258260_at	unknown protein ; supported by cD	---	AT3G26850	1.46	0.55	5.36	0.001195	0.009229	-0.91
253640_at	putative protein ;supported by full-l	---	AT4G30630	1.45	0.53	5.36	0.001196	0.009233	-0.91
260806_at	RNA recognition motif-containing pr	---	AT1G78260	-1.41	-0.50	-5.36	0.001197	0.009233	-0.91
252130_at	putative protein 1 photosystem II o	PSBO2	AT3G50820	-1.54	-0.63	-5.36	0.001197	0.009233	-0.91
249741_at	putative protein contains similarity t	APRR5	AT5G24470	1.39	0.47	5.36	0.001197	0.009233	-0.91
257072_at	myrosinase-associated protein, put	---	AT3G14220	-2.31	-1.21	-5.36	0.001197	0.009233	-0.91
258919_at	Expressed protein ; supported by fu	---	AT3G10525	-1.75	-0.80	-5.36	0.001198	0.009233	-0.91
259768_at	expressed protein supported by cDI	COR314-TI	AT1G29390	-1.39	-0.47	-5.36	0.001198	0.009233	-0.91
250866_at	Expressed protein ; supported by fu	---	AT5G03905	1.57	0.65	5.36	0.001199	0.009237	-0.91
261655_at	hypothetical protein predicted by ge	---	AT1G01940	1.83	0.87	5.36	0.001199	0.009237	-0.91
248659_at	cyclin C-like protein	---	AT5G48640	1.63	0.71	5.36	0.001201	0.009238	-0.91
257794_at	hypothetical protein predicted by ge	---	AT3G27050	-1.75	-0.81	-5.36	0.001201	0.009238	-0.91
264327_at	putative translation initiation factor	EIF2 GAMM	AT1G04170	1.48	0.56	5.36	0.001201	0.009238	-0.91
266818_at	unknown protein	HUB1	AT2G44950	1.66	0.73	5.35	0.001207	0.009282	-0.92

247208_at	nodulin-like ;supported by full-lengt	---	AT5G64870	-1.60	-0.68	-5.35	0.001208	0.009285	-0.92
256524_at	glutamine synthetase, putative sim	ATGSR2	AT1G66200	1.58	0.66	5.35	0.001209	0.009285	-0.92
253889_at	forever young gene (FEY)	FEY	AT4G27760	-1.44	-0.53	-5.35	0.00121	0.009286	-0.92
246117_at	CAF-like protein CAF protein - Aral	DCL4	AT5G20320	1.89	0.92	5.35	0.00121	0.009286	-0.92
257271_at	Expressed protein ; supported by fu	---	AT3G28007	-3.90	-1.96	-5.35	0.00121	0.009286	-0.92
253909_at	putative protein LEDI-3 protein, Lith	---	AT4G27270	-1.67	-0.74	-5.35	0.001213	0.009305	-0.92
261154_at	unknown protein ;supported by full-	---	AT1G04970	1.76	0.82	5.35	0.001215	0.009316	-0.92
262539_at	unknown protein Location of est 13(	---	AT1G17200	-1.86	-0.90	-5.34	0.001218	0.00933	-0.93
256803_at	cytochrome P450, putative similar t	CYP705A3	AT3G20960	1.45	0.54	5.34	0.001218	0.00933	-0.93
265332_at	hypothetical protein predicted by ge	---	AT2G18410	1.66	0.73	5.34	0.001218	0.00933	-0.93
261585_at	NAC domain protein, putative simil	ANAC001	AT1G01010	2.05	1.04	5.34	0.001219	0.009332	-0.93
251688_at	putative protein hypothetical protei	---	AT3G56480	-2.55	-1.35	-5.34	0.00122	0.009334	-0.93
253247_at	Homeodomain - like protein similar	BLH6	AT4G34610	-2.11	-1.08	-5.34	0.00122	0.009334	-0.93
256118_at	hypothetical protein predicted by ge	KU70	AT1G16970	1.71	0.78	5.34	0.001222	0.009345	-0.93
251647_at	protein kinase - like protein wall-as	---	AT3G57770	1.65	0.73	5.34	0.001223	0.009345	-0.93
262853_at	unknown protein Location of ESTs	---	AT1G20890	1.56	0.64	5.34	0.001226	0.009363	-0.93
258910_at	putative RNA helicase similar to RN	---	AT3G06480	1.47	0.56	5.34	0.001227	0.009363	-0.93
254831_at	Ribosomal protein L7Ae -like variou	---	AT4G12600	2.54	1.34	5.34	0.001227	0.009366	-0.93
252075_at	putative protein saposin precursor	---	AT3G51730	1.80	0.85	5.34	0.001228	0.009366	-0.93
247357_at	receptor-like protein kinase ; suppo	---	AT5G63710	1.55	0.64	5.33	0.001229	0.009366	-0.94
253479_at	ferredoxin-NADP+ reductase - like	---	AT4G32360	1.45	0.54	5.33	0.001229	0.009366	-0.94
260291_at	putative protein kinase similar to MA	YDA	AT1G63700	-1.40	-0.48	-5.33	0.001232	0.009381	-0.94
248385_at	putative protein contains similarity t	---	AT5G51910	-1.69	-0.76	-5.33	0.001232	0.009381	-0.94
258649_at	putative transitional endoplasmic re	CDC48	AT3G09840	1.41	0.49	5.33	0.001232	0.009381	-0.94
261725_at	small nuclear ribonucleoprotein, pu	SmD3	AT1G76300	1.79	0.84	5.33	0.001234	0.009389	-0.94
262117_at	ribosomal protein L19, putative sim	emb2386	AT1G02780	1.71	0.77	5.33	0.001234	0.009389	-0.94
261019_at	unknown protein ; supported by cDI	---	AT1G26470	1.58	0.66	5.33	0.001236	0.009398	-0.94
258742_at	hypothetical protein predicted by ge	---	AT3G05800	-2.31	-1.21	-5.33	0.001238	0.009412	-0.94
245168_at	3-ketoacyl-CoA thiolase ;supported	PKT3	AT2G33150	1.69	0.76	5.33	0.00124	0.009419	-0.95
248798_at	putative protein contains similarity t	---	AT5G47190	-1.57	-0.65	-5.33	0.00124	0.009419	-0.95
257988_at	unknown protein ; supported by cD	---	AT3G20870	1.54	0.62	5.33	0.00124	0.009419	-0.95
266116_at	unknown protein ;supported by full-	TOM3	AT2G02180	-1.76	-0.82	-5.32	0.001243	0.009433	-0.95
247709_at	D-xylose-H+ symporter - like protei	---	AT5G59250	-1.69	-0.76	-5.32	0.001244	0.009436	-0.95
245145_at	putative dihydrodipicolinate synthas	DHDPS2	AT2G45440	2.07	1.05	5.32	0.001246	0.009452	-0.95
245693_at	thioredoxin - like protein	WCRKC2	AT5G04260	1.60	0.68	5.32	0.001247	0.009453	-0.95

265702_at	putative purple acid phosphatase co	PAP9	AT2G03450	1.60	0.68	5.32	0.001248	0.009454	-0.95
263995_at	putative MADS-box protein	SVP	AT2G22540	1.67	0.74	5.32	0.00125	0.00947	-0.95
247405_at	Arac10 ; supported by cDNA: gi_37	ARAC10	AT5G62880	-1.56	-0.65	-5.32	0.001251	0.009475	-0.96
257883_at	calmodulin-binding protein, putative	---	AT3G16940	1.75	0.80	5.32	0.001252	0.009479	-0.96
265953_at	unknown protein	---	AT2G37478	1.49	0.57	5.32	0.001253	0.009479	-0.96
266916_at	hypothetical protein predicted by ge	---	AT2G45860	1.95	0.96	5.32	0.001254	0.00949	-0.96
258132_at	protein kinase, putative similar to G	ATPERK1	AT3G24550	-1.87	-0.90	-5.31	0.001255	0.009493	-0.96
267134_at	putative protein kinase contains a p	---	AT2G23450	1.38	0.46	5.31	0.001256	0.009493	-0.96
266109_at	putative mitochondrial carrier protei	---	AT2G37890	2.29	1.19	5.31	0.001257	0.009503	-0.96
257982_at	unknown protein similar to TYPE II	ATTOP6B	AT3G20780	-1.71	-0.77	-5.31	0.00126	0.009516	-0.96
257680_at	aspartate carbamoyltransferase pr	---	AT3G20330	1.75	0.81	5.31	0.00126	0.009516	-0.96
252056_at	ubiquitin extension protein (UBQ1)	UBQ1	AT3G52590	1.50	0.58	5.31	0.00126	0.009516	-0.96
266592_at	putative fatty acid desaturase/cytoc	---	AT2G46210	-1.50	-0.58	-5.31	0.001261	0.009518	-0.96
256747_at	unknown protein similar to CW14 G	---	AT3G29180	1.59	0.67	5.31	0.001264	0.009534	-0.97
258060_at	protein phosphatase 2A regulatory	ATB' DELT	AT3G26030	-1.44	-0.53	-5.31	0.001264	0.009538	-0.97
266767_at	Expressed protein ; supported by fu	---	AT2G46910	-1.75	-0.80	-5.31	0.001265	0.00954	-0.97
248403_at	arginine-aspartate-rich RNA binding	---	AT5G51410	1.54	0.62	5.31	0.001266	0.00954	-0.97
252647_at	protein-tyrosine-phosphatase-like p	---	AT3G44620	-1.99	-0.99	-5.30	0.001269	0.009559	-0.97
251706_at	nodulin-like protein MtN21 - Medica	---	AT3G56620	1.64	0.72	5.30	0.00127	0.009566	-0.97
251598_at	AP2 transcription factor - like prote	---	AT3G57600	-1.46	-0.54	-5.30	0.001274	0.009588	-0.97
250671_at	transcription factor HBP-1b homolo	AHBP-1B	AT5G06950	1.47	0.56	5.30	0.001274	0.009588	-0.98
255636_at	homeodomain protein AHDP ; supp	ANL2	AT4G00730	1.65	0.72	5.30	0.001275	0.009595	-0.98
252464_at	RNA-binding protein-like protein va	---	AT3G47160	1.75	0.81	5.30	0.001276	0.009596	-0.98
248172_at	unknown protein ;supported by full-	---	AT5G54660	2.17	1.12	5.30	0.001278	0.009609	-0.98
245781_at	S-ribonuclease binding protein SBP	SBP1	AT1G45976	1.66	0.73	5.30	0.001279	0.009611	-0.98
245052_at	putative pectinesterase	---	AT2G26440	-1.72	-0.78	-5.29	0.001282	0.009628	-0.98
247268_at	putative protein contains similarity t	---	AT5G64080	-1.72	-0.79	-5.29	0.001282	0.00963	-0.98
250851_at	putative protein rngB protein, Dicty	---	AT5G04420	-1.67	-0.74	-5.29	0.001283	0.00963	-0.98
251356_at	putative protein hypothetical protein	AtPP2-A13	AT3G61060	1.68	0.74	5.29	0.001286	0.00965	-0.99
251837_s_a	spliceosomal - like protein spliceos	---	AT3G55200	1.66	0.73	5.29	0.001288	0.009661	-0.99
247627_at	cysteine proteinase AALP identical	AALP	AT5G60360	1.45	0.53	5.29	0.001289	0.009661	-0.99
254834_at	flavonoid 3,5-hydroxylase -like prot	CYP706A4	AT4G12300	1.73	0.79	5.29	0.001289	0.009661	-0.99
258934_at	putative RecA protein similar to Rec	RECA3	AT3G10140	1.59	0.67	5.29	0.001289	0.009661	-0.99
260664_at	myb-related protein, putative simila	ATRL5	AT1G19510	2.45	1.29	5.29	0.00129	0.009661	-0.99
247156_at	lysosomal Pro-X carboxypeptidase	---	AT5G65760	1.55	0.63	5.29	0.001291	0.009661	-0.99

262888_at	RNA-directed RNA polymerase, pu	RDR1	AT1G14790	2.79	1.48	5.28	0.0013	0.009718	-1.00
256715_at	hypothetical protein predicted by g	MEE18	AT2G34090	-1.90	-0.93	-5.28	0.001301	0.009723	-1.00
251225_at	putative protein glycosyl transferas	GATL7	AT3G62660	-1.83	-0.87	-5.28	0.001303	0.009734	-1.00
252353_at	putative protein KIAA0226 gene pr	---	AT3G48200	-1.54	-0.63	-5.28	0.001304	0.009737	-1.00
245112_at	glycerol-3-phosphate dehydrogenas	GPDHC1	AT2G41540	-3.39	-1.76	-5.28	0.001304	0.009737	-1.00
261559_at	LIM domain protein, putative simila	---	AT1G01780	1.60	0.68	5.28	0.001306	0.009737	-1.00
249360_at	putative protein KIAA0409 protein,	---	AT5G40530	1.51	0.59	5.28	0.001307	0.009737	-1.00
265419_at	putative secretory carrier-associate	---	AT2G20840	-1.67	-0.74	-5.28	0.001307	0.009737	-1.00
246925_at	cytochrome P450 71B1 - like prote	CYP71B14	AT5G25180	1.48	0.57	5.28	0.001307	0.009737	-1.00
261569_at	DNA-binding protein, putative simil	LHY	AT1G01060	-1.62	-0.70	-5.28	0.001308	0.009742	-1.00
257294_at	non-phototropic hypocotyl protein,	---	AT3G15570	-1.95	-0.96	-5.27	0.001309	0.009745	-1.01
263922_s_a	putative pyruvate kinase ;supporte	---	AT2G36580	1.44	0.53	5.27	0.001309	0.009745	-1.01
255440_at	predicted protein of unknown functi	---	AT4G02530	-1.64	-0.72	-5.27	0.00131	0.009748	-1.01
247865_at	Expressed protein ; supported by fu	---	AT5G57815	1.48	0.56	5.27	0.001313	0.009757	-1.01
255821_at	putative initiator tRNA phosphoribos	---	AT2G40570	1.54	0.63	5.27	0.001313	0.009757	-1.01
255041_at	putative protein hypothetical protei	---	AT4G09620	1.66	0.73	5.27	0.001314	0.00976	-1.01
258966_at	putative DNA gyrase subunit A simi	---	AT3G10690	2.19	1.13	5.27	0.001314	0.00976	-1.01
267101_at	putative peroxidase	---	AT2G41480	1.80	0.85	5.27	0.001315	0.00976	-1.01
265742_at	putative ribose 5-phosphate isomer	---	AT2G01290	1.87	0.90	5.27	0.001316	0.009763	-1.01
266552_at	unknown protein ; supported by cDI	AGP16	AT2G46330	-1.86	-0.90	-5.27	0.001316	0.009765	-1.01
252271_s_a	putative protein CGI-141 protein- H	---	AT3G49420	1.42	0.51	5.27	0.001317	0.009768	-1.01
264323_at	putative dimethylaniline monooxyge	---	AT1G04180	-4.52	-2.18	-5.27	0.001318	0.009768	-1.01
245644_at	unknown protein similar to putative	---	AT1G25320	-1.64	-0.71	-5.27	0.001319	0.009772	-1.01
254073_at	splicing factor At-SRp40 contains E	RSP35	AT4G25500	1.56	0.64	5.27	0.001319	0.009772	-1.01
263785_at	unknown protein ; supported by cD	---	AT2G46390	1.56	0.64	5.26	0.001324	0.009802	-1.02
262558_at	Expressed protein ; supported by fu	---	AT1G31335	-1.59	-0.67	-5.26	0.001324	0.009802	-1.02
262531_at	putative leucine-rich receptor protei	---	AT1G17230	-1.78	-0.84	-5.26	0.001325	0.009802	-1.02
255557_at	hypothetical protein ; supported by	---	AT4G01990	1.96	0.97	5.26	0.001325	0.009802	-1.02
254002_at	steroid sulfotransferase - like prote	---	AT4G26280	-1.39	-0.48	-5.26	0.001326	0.009802	-1.02
256016_at	hypothetical protein predicted by ge	---	AT1G19240	1.63	0.71	5.26	0.001326	0.009802	-1.02
245730_at	unknown protein ;supported by full-	---	AT1G73470	-1.47	-0.56	-5.26	0.001326	0.009802	-1.02
258064_at	putative cytochrome P450 similar to	CYP72A14	AT3G14680	3.12	1.64	5.26	0.001327	0.009803	-1.02
253359_at	putative protein kinase protein kina	---	AT4G33080	1.67	0.74	5.26	0.001331	0.00982	-1.02
248594_at	unknown protein ; supported by ful	---	AT5G49210	1.90	0.93	5.26	0.001331	0.00982	-1.02
264517_at	hypothetical protein predicted by ge	---	AT1G10120	-1.77	-0.82	-5.26	0.001333	0.009827	-1.02

266845_at	unknown protein	---	AT2G26110	-2.01	-1.01	-5.26	0.001334	0.009831	-1.03
248008_at	transcription factor NtWRKY4-like ;	WRKY2	AT5G56270	1.86	0.90	5.26	0.001335	0.009842	-1.03
265446_at	unknown protein ;supported by full-	---	AT2G37110	-1.54	-0.62	-5.26	0.001336	0.009842	-1.03
259047_at	unknown protein	DegP7	AT3G03380	1.40	0.49	5.25	0.001342	0.009884	-1.03
261230_at	beta tubulin 1, putative similar to G	TUB5	AT1G20010	-1.47	-0.56	-5.25	0.001345	0.009899	-1.03
245599_at	RING-H2 finger protein RHF1a	RHF1A	AT4G14220	1.93	0.95	5.25	0.001347	0.009911	-1.04
245084_at	putative MYB family transcription fa	AtMYB70	AT2G23290	-2.08	-1.06	-5.25	0.001348	0.009916	-1.04
260912_at	ATP-dependent Clp protease prote	CLPP5	AT1G02560	1.46	0.55	5.25	0.00135	0.009927	-1.04
245147_at	putative RAD51C-like DNA repair p	ATRAD51C	AT2G45280	2.25	1.17	5.24	0.001351	0.009931	-1.04
267523_at	unknown protein	---	AT2G30600	-1.57	-0.65	-5.24	0.001351	0.009931	-1.04
267620_at	putative beta-1,3-glucanase	---	AT2G39640	-1.46	-0.55	-5.24	0.001352	0.009931	-1.04
253954_at	putative aconitase Pumpkin mRNA	---	AT4G26970	1.96	0.97	5.24	0.001352	0.009931	-1.04
259984_at	putative RNA-binding protein conta	---	AT1G76460	1.61	0.69	5.24	0.001353	0.009935	-1.04
255743_at	unknown protein ; supported by cDI	---	AT1G25375	-1.53	-0.61	-5.24	0.001356	0.009953	-1.04
252925_at	ES43 like protein ES43 protein - ba	SHL1	AT4G39100	1.70	0.76	5.24	0.001357	0.009958	-1.04
253459_at	hypothetical protein	---	AT4G32080	-1.41	-0.49	-5.24	0.001358	0.009958	-1.04
250174_at	agp6	AGP6	AT5G14380	-1.45	-0.54	-5.24	0.001358	0.009958	-1.04
250829_at	disease resistance - like protein rp	ADR1-L2	AT5G04720	1.64	0.71	5.24	0.00136	0.009964	-1.05
260738_at	hypothetical protein predicted by ge	---	AT1G14990	1.48	0.56	5.24	0.00136	0.009964	-1.05
245986_at	protein kinase-like protein kinase 1	PBS1	AT5G13160	-1.42	-0.51	-5.24	0.00136	0.009964	-1.05
260548_at	biotin synthase (Bio B) ;supported	BIO2	AT2G43360	1.95	0.96	5.24	0.00136	0.009964	-1.05
254923_at	protein phosphatase type 1 PP1BG	TOPP7	AT4G11240	1.69	0.76	5.24	0.001363	0.009978	-1.05
252084_at	wax synthase-like protein wax synt	AtSAT1	AT3G51970	-1.68	-0.75	-5.24	0.001364	0.009979	-1.05
260895_at	unknown protein similar to unknowr	---	AT1G29250	2.29	1.20	5.24	0.001364	0.00998	-1.05
249268_at	Expressed protein ; supported by cl	---	AT5G41685	1.46	0.55	5.23	0.001367	0.009994	-1.05
254080_at	fibrillarin 2 (AtFib2) identical to fibril	FIB2	AT4G25630	3.31	1.73	5.23	0.001369	0.010002	-1.05
245477_at	hypothetical protein	ARR2	AT4G16110	-1.54	-0.63	-5.23	0.001369	0.010002	-1.05
249529_at	putative protein predicted proteins,	---	AT5G38730	1.87	0.90	5.23	0.001369	0.010002	-1.05
245618_at	hypothetical protein	---	AT4G14510	1.64	0.71	5.23	0.001374	0.01003	-1.06
256149_at	zinc finger protein, putative similar	AtIDD7	AT1G55110	1.80	0.85	5.23	0.001374	0.010031	-1.06
249178_at	putative protein similar to unknown	SCP2	AT5G42890	1.47	0.56	5.23	0.001376	0.010043	-1.06
265816_s_a	putative elongation factor beta-1 ;s	---	AT1G30230	1.75	0.81	5.23	0.001377	0.010046	-1.06
262749_at	lipase, putative contains Pfam prof	---	AT1G28580	1.51	0.60	5.23	0.001379	0.010046	-1.06
250826_at	unknown protein ; supported by cDI	---	AT5G05220	-1.56	-0.64	-5.23	0.001379	0.010046	-1.06
257087_at	purple acid phosphatase, putative s	PAP18	AT3G20500	1.63	0.70	5.23	0.001379	0.010046	-1.06

255469_at	predicted OR23 protein of unknown	---	AT4G03030	1.55	0.63	5.22	0.001381	0.010056	-1.06
248985_at	GTP-binding protein-like; root hair c	---	AT5G45160	1.45	0.53	5.22	0.001382	0.010059	-1.06
266261_at	putative zinc finger protein ;suppor	---	AT2G27580	-1.39	-0.48	-5.22	0.001383	0.010059	-1.06
264803_at	hypothetical protein predicted by ge	---	AT1G08580	1.82	0.87	5.22	0.001383	0.010059	-1.06
245063_at	Expressed protein ; supported by fu	---	AT2G39795	2.69	1.43	5.22	0.001386	0.010074	-1.07
250928_at	putative protein	EIN2	AT5G03280	1.47	0.55	5.22	0.001386	0.010074	-1.07
258126_at	hypothetical protein predicted by ge	---	AT3G24490	1.58	0.66	5.22	0.001388	0.01008	-1.07
263136_at	trehalose-6-phosphate synthase, p	ATTPS1	AT1G78580	-1.53	-0.62	-5.22	0.00139	0.010093	-1.07
255933_at	membrane protein, putative similar	ATRBL6	AT1G12750	-1.95	-0.97	-5.22	0.001391	0.010093	-1.07
259895_at	unknown protein ;supported by full-	PFI	AT1G71440	-1.45	-0.53	-5.22	0.001391	0.010093	-1.07
247361_at	unknown protein ;supported by full-	---	AT5G63480	-1.72	-0.78	-5.22	0.001393	0.010107	-1.07
266561_at	unknown protein	---	AT2G23960	-1.43	-0.51	-5.22	0.001394	0.010107	-1.07
250554_at	unknown protein ;supported by full-	---	AT5G07900	1.52	0.61	5.21	0.001395	0.010107	-1.07
253019_at	putative protein crp1-hcf111 allele,	---	AT4G38010	1.49	0.58	5.21	0.001395	0.010107	-1.07
247885_at	putative protein similar to unknown	---	AT5G57830	-1.98	-0.99	-5.21	0.001398	0.010123	-1.08
246153_s_a	RAN1 small Ras-like GTP-binding r	RAN-1	AT5G20010	2.02	1.01	5.21	0.001399	0.010127	-1.08
255878_at	putative bZIP transcription factor co	---	AT2G40620	-1.88	-0.91	-5.21	0.001399	0.010127	-1.08
254087_at	brefeldin A-sensitive Golgi protein -	---	AT4G24840	-1.41	-0.50	-5.21	0.001403	0.010154	-1.08
256870_at	cytochrome P450, putative contain	CYP71B34	AT3G26300	2.19	1.13	5.21	0.001404	0.010158	-1.08
261566_at	unknown protein ;supported by full-	---	AT1G33230	-1.70	-0.77	-5.21	0.001409	0.010179	-1.08
261201_at	unknown protein ;supported by full-	---	AT1G12850	-1.54	-0.63	-5.20	0.00141	0.010179	-1.09
264248_at	hypothetical protein predicted by ge	---	AT1G78700	-1.78	-0.83	-5.20	0.00141	0.010179	-1.09
265149_at	unknown protein ; supported by full-	---	AT1G51400	-1.60	-0.67	-5.20	0.00141	0.010179	-1.09
246865_s_a	eukaryotic translation initiation fact	TIF3B1	AT5G25780	1.38	0.47	5.20	0.001411	0.010184	-1.09
259688_at	membrane protein, putative similar	ATRBL2	AT1G63120	-1.89	-0.92	-5.20	0.001412	0.010186	-1.09
246632_at	hypothetical protein similar to putati	---	AT1G29710	1.55	0.64	5.20	0.001415	0.010205	-1.09
255586_at	hypothetical protein ;supported by f	MEE49	AT4G01560	2.05	1.03	5.20	0.001416	0.010205	-1.09
250769_at	putative protein similar to unknown	---	AT5G05680	2.12	1.08	5.20	0.001416	0.010206	-1.09
245242_at	chlorophyll a oxygenase identical to	CH1	AT1G44446	-1.73	-0.79	-5.20	0.001418	0.010213	-1.09
246447_at	putative protein SART-1 protein - H	DOT2	AT5G16780	1.58	0.66	5.20	0.001419	0.010213	-1.09
250891_at	fatty acid elongase - like protein KC	KCS19	AT5G04530	-1.67	-0.74	-5.20	0.001419	0.010213	-1.09
263930_at	hypothetical protein predicted by ge	---	AT2G36300	-1.52	-0.60	-5.20	0.001419	0.010213	-1.09
253812_at	putative wound induced protein wo	---	AT4G28240	-1.63	-0.70	-5.20	0.001421	0.01022	-1.09
259840_at	photosystem I subunit VI precursor	PSAH2	AT1G52230	-1.68	-0.75	-5.20	0.001423	0.01023	-1.10
258773_at	hypothetical protein predicted by ge	---	AT3G10730	-1.37	-0.46	-5.20	0.001423	0.01023	-1.10

262297_at	hypothetical protein similar to hypot	---	AT1G27600	-1.41	-0.50	-5.19	0.001425	0.010238	-1.10
259245_at	unknown protein predicted by gene	---	AT3G07660	1.62	0.69	5.19	0.001429	0.01026	-1.10
247549_at	putative transcription factor MYB28	MYB28	AT5G61420	1.97	0.98	5.19	0.00143	0.010263	-1.10
262770_at	hypothetical protein predicted by ge	---	AT1G13190	-1.92	-0.94	-5.19	0.00143	0.010263	-1.10
266228_at	unknown protein ; supported by ful	CXIP4	AT2G28910	1.64	0.71	5.19	0.001432	0.010265	-1.10
256873_at	cytochrome P450, putative contain	CYP71B34	AT3G26300	1.56	0.64	5.19	0.001434	0.010276	-1.10
253612_at	putative protein D-ribulokinase - Kl	---	AT4G30310	-1.47	-0.56	-5.19	0.001434	0.010276	-1.10
261628_at	hypothetical protein predicted by ge	---	AT1G50000	1.43	0.52	5.19	0.001435	0.010276	-1.11
248669_at	putative protein contains similarity t	---	AT5G48730	1.85	0.89	5.19	0.001436	0.010283	-1.11
254594_at	putative protein	---	AT4G18930	1.96	0.97	5.19	0.001437	0.010283	-1.11
256516_at	receptor protein kinase (TMK1), pu	TMK1	AT1G66150	-1.98	-0.99	-5.19	0.001438	0.010283	-1.11
260823_at	hypothetical protein predicted by g	DRIP1	AT1G06770	1.49	0.57	5.19	0.001438	0.010283	-1.11
259257_at	unknown protein ;supported by full-	---	AT3G07760	1.60	0.68	5.18	0.001443	0.010313	-1.11
259545_at	hypothetical protein ; supported by	AAE1	AT1G20560	1.53	0.62	5.18	0.001446	0.010326	-1.11
262055_at	unknown protein ; supported by full-	---	AT1G79970	1.45	0.53	5.18	0.001447	0.010326	-1.11
258379_at	putative decarboxilase similar to 5-	---	AT3G16700	-1.83	-0.87	-5.18	0.001447	0.010326	-1.11
255505_at	SAR1/GTP-binding secretory factor	ATSAR2	AT4G02080	1.50	0.59	5.18	0.001447	0.010327	-1.11
245639_at	unknown protein contains similarity	---	AT1G25260	2.11	1.08	5.18	0.001448	0.010327	-1.12
260968_at	hypothetical protein predicted by ge	---	AT1G12250	-1.63	-0.70	-5.18	0.001449	0.010327	-1.12
250355_at	putative protein predicted protein, A	---	AT5G11700	-1.63	-0.71	-5.18	0.001453	0.010353	-1.12
246120_at	tetratricopeptide repeat protein tetr	---	AT5G20360	1.65	0.72	5.18	0.001454	0.010353	-1.12
248255_at	ATP-dependent Clp protease ATP-l	CLPX	AT5G53350	1.59	0.67	5.17	0.001455	0.010355	-1.12
260444_at	unknown protein ;supported by full-	---	AT1G68300	1.57	0.65	5.17	0.001455	0.010355	-1.12
257141_at	60S ribosomal protein L34, putative	---	AT3G28900	2.16	1.11	5.17	0.001456	0.010355	-1.12
267315_at	putative CCAAT-binding transcriptic	NF-YA4	AT2G34720	1.91	0.93	5.17	0.001456	0.010355	-1.12
257780_at	hypothetical protein predicted by ge	---	AT3G27100	1.64	0.71	5.17	0.001456	0.010355	-1.12
258656_at	putative Ras-like GTP-binding prote	ATRABE1E	AT3G09900	1.80	0.85	5.17	0.001458	0.01036	-1.12
255474_at	coded for by A. thaliana cDNA T75	---	AT4G02480	1.51	0.60	5.17	0.00146	0.010374	-1.12
253814_at	putative protein ;supported by full-l	---	AT4G28290	-1.91	-0.94	-5.17	0.00146	0.010374	-1.12
253782_at	hypothetical protein	---	AT4G28590	1.40	0.49	5.17	0.001463	0.010389	-1.13
258701_at	putative RNA helicase similar to RI	---	AT3G09720	2.04	1.03	5.17	0.001464	0.010397	-1.13
260283_at	hypothetical protein predicted by g	PTAC17	AT1G80480	1.39	0.47	5.17	0.001467	0.010412	-1.13
250421_at	putative protein	OCP3	AT5G11270	1.68	0.75	5.16	0.001471	0.010437	-1.13
256379_at	hypothetical protein predicted by ge	---	AT1G66840	1.80	0.85	5.16	0.001473	0.010445	-1.13
259735_at	Expressed protein ; supported by fu	---	AT1G64405	2.02	1.02	5.16	0.001476	0.010462	-1.14

247130_at	putative protein contains similarity t	---	AT5G66180	2.24	1.16	5.16	0.001477	0.010469	-1.14
266656_at	putative CCCH-type zinc finger prot	ATCTH	AT2G25900	-1.80	-0.85	-5.16	0.001478	0.010473	-1.14
259471_at	hypothetical protein contains similar	---	AT1G19040	-1.38	-0.47	-5.16	0.001482	0.010492	-1.14
255070_at	isoamylase-like protein probable is	ISA3	AT4G09020	1.45	0.54	5.16	0.001485	0.010513	-1.14
260441_at	unknown protein ;supported by full-	---	AT1G68260	-2.59	-1.37	-5.15	0.001488	0.010527	-1.14
256753_at	unknown protein contains Pfam pro	GHS1	AT3G27160	-1.66	-0.73	-5.15	0.001492	0.010549	-1.15
265530_at	12-oxophytodienoate-10,11-reduct	OPR3	AT2G06050	-3.09	-1.63	-5.15	0.001494	0.010563	-1.15
249700_at	40S ribosomal protein S3 ; support	---	AT5G35530	1.71	0.77	5.15	0.001495	0.010564	-1.15
266152_s_a	hypothetical protein predicted by ge	---	AT2G12050	-1.44	-0.52	-5.15	0.001495	0.010564	-1.15
258425_at	unknown protein	---	AT3G16760	-1.39	-0.47	-5.15	0.001497	0.010572	-1.15
251801_at	cytosolic triosephosphatisomerase	TPI	AT3G55440	-1.37	-0.46	-5.15	0.001498	0.010579	-1.15
259896_at	unknown protein ;supported by full-	---	AT1G71500	-1.56	-0.64	-5.15	0.0015	0.010585	-1.15
249466_at	ribosomal protein L5 - like ribosom	RPL5B	AT5G39740	1.93	0.95	5.15	0.001501	0.010589	-1.15
260566_at	cysteine synthase (cpACS1) ;supp	OASB	AT2G43750	-1.66	-0.73	-5.14	0.001502	0.010596	-1.16
254081_at	putative protein various predicted p	---	AT4G25660	-1.63	-0.70	-5.14	0.001503	0.010597	-1.16
260311_at	hypothetical protein predicted by ge	---	AT1G70570	1.38	0.47	5.14	0.001505	0.010608	-1.16
264946_at	hypothetical protein predicted by ge	---	AT1G77010	1.64	0.72	5.14	0.001508	0.010625	-1.16
262272_at	unknown protein ; supported by cDI	---	AT1G42440	1.39	0.48	5.14	0.001514	0.010658	-1.16
249553_at	receptor serine/threonine protein ki	---	AT5G38260	1.79	0.84	5.14	0.001515	0.010663	-1.16
248103_at	ubiquitin-like protein SMT3-like ; su	SUMO2	AT5G55160	1.85	0.89	5.14	0.001516	0.010663	-1.16
261490_at	tumor suppressor, putative similar t	SAC52	AT1G14320	1.75	0.81	5.14	0.001516	0.010663	-1.16
256513_at	unknown protein similar to phospho	SGR2	AT1G31480	1.47	0.56	5.14	0.001516	0.010663	-1.17
256404_at	unknown protein similar to far-red ir	FRS7	AT3G06250	1.76	0.81	5.13	0.00152	0.010683	-1.17
265141_at	hypothetical protein predicted by ge	---	AT1G51350	1.52	0.61	5.13	0.001522	0.01069	-1.17
250858_at	I-box binding factor - like protein I-b	---	AT5G04760	1.84	0.88	5.13	0.001522	0.010692	-1.17
247648_at	laccase - like protein laccase LAC2	LAC17	AT5G60020	-1.59	-0.67	-5.13	0.001523	0.010692	-1.17
247015_at	protease-like	---	AT5G66960	1.72	0.78	5.13	0.001524	0.010694	-1.17
265154_at	putative GTP-binding protein, ERG	---	AT1G30960	2.21	1.15	5.13	0.001525	0.010696	-1.17
260587_at	unknown protein ; supported by cDI	---	AT1G53210	-1.54	-0.62	-5.13	0.001525	0.010696	-1.17
266554_s_a	eukaryotic translation initiation facto	---	AT2G46280	1.67	0.74	5.13	0.001529	0.010721	-1.17
245123_at	unknown protein ; supported by cD	CAO	AT2G47450	-1.59	-0.67	-5.13	0.001531	0.010729	-1.18
264045_at	putative GTP cyclohydrolase	---	AT2G22450	1.46	0.55	5.13	0.001532	0.010731	-1.18
251327_at	prolyl aminopeptidase-like protein p	---	AT3G61540	1.38	0.47	5.12	0.00154	0.010782	-1.18
245747_at	hypothetical protein predicted by ge	---	AT1G51100	1.77	0.83	5.12	0.001542	0.010797	-1.18
246436_at	putative protein cisplatin resistance	---	AT5G17440	1.42	0.50	5.12	0.001543	0.010799	-1.18

264878_at	unknown protein	---	AT2G17410	1.41	0.50	5.12	0.001544	0.010805	-1.19
246699_at	putative protein predicted proteins,	---	AT5G27990	1.80	0.85	5.12	0.001548	0.010815	-1.19
253867_at	putative protein tRNA nucleotidyltra	---	AT4G27490	1.97	0.98	5.12	0.001548	0.010815	-1.19
247013_at	putative protein strong similarity to u	BT4	AT5G67480	-2.07	-1.05	-5.12	0.001548	0.010815	-1.19
261864_s_a	10-formyltetrahydrofolate synthetas	THFS	AT1G50480	1.54	0.63	5.12	0.00155	0.010821	-1.19
257487_at	hypothetical protein predicted by ge	---	AT1G71850	2.17	1.12	5.12	0.00155	0.010821	-1.19
266237_at	DNA-directed RNA polymerase 14	ATRPC14	AT2G29540	1.93	0.95	5.11	0.001557	0.010862	-1.19
253717_at	putative protein putative suppresso	---	AT4G29440	-1.49	-0.58	-5.11	0.001558	0.01087	-1.20
255660_at	Expressed protein ; supported by fu	---	AT4G00755	1.58	0.66	5.11	0.001559	0.010872	-1.20
250716_at	sucrose transporter protein	ATSUC9	AT5G06170	-1.46	-0.54	-5.11	0.00156	0.010872	-1.20
253336_at	putative protein Caenorhabditis ele	EIF3K	AT4G33250	1.55	0.63	5.11	0.00156	0.010872	-1.20
248718_at	farnesyl diphosphate synthase pred	FPS1	AT5G47770	-1.93	-0.95	-5.11	0.001563	0.010891	-1.20
257816_at	glycosyl transferase, putative conta	QUA1	AT3G25140	-1.56	-0.65	-5.11	0.001564	0.010892	-1.20
258192_at	putative terpene synthase-related p	---	AT3G29110	-1.41	-0.49	-5.11	0.001565	0.010898	-1.20
251476_at	putative protein hypothetical protei	---	AT3G59670	1.99	0.99	5.11	0.001566	0.010898	-1.20
249811_at	unknown protein ;supported by full-	---	AT5G23760	1.42	0.51	5.11	0.001568	0.010908	-1.20
251037_at	putative protein oxysterol-binding p	UNE18	AT5G02100	1.88	0.91	5.10	0.00157	0.010914	-1.20
250609_at	peptide methionine sulfoxide reduct	PMSR3	AT5G07470	1.44	0.53	5.10	0.00157	0.010914	-1.20
261434_at	receptor-like serine/threonine kinas	---	AT1G07650	-1.85	-0.89	-5.10	0.001571	0.010917	-1.20
263351_at	hypothetical protein predicted by gr	---	AT2G22120	1.37	0.46	5.10	0.001571	0.010917	-1.20
245255_at	COP9 protein ; supported by cDNA	COP9	AT4G14110	1.52	0.61	5.10	0.001572	0.010917	-1.20
257832_at	light regulated protein, putative sim	CCL	AT3G26740	1.47	0.55	5.10	0.001573	0.010917	-1.21
266396_at	unknown protein	---	AT2G38790	1.82	0.87	5.10	0.001574	0.010922	-1.21
258455_at	unknown protein ;supported by full-	---	AT3G22440	1.82	0.87	5.10	0.001574	0.010922	-1.21
261088_at	DNA-binding protein, putative simil	---	AT1G07590	1.63	0.70	5.10	0.001576	0.010931	-1.21
250041_at	putative protein p53 inducible prote	PIR121	AT5G18410	-1.38	-0.47	-5.10	0.001578	0.010938	-1.21
266813_at	unknown protein	---	AT2G44920	-1.65	-0.72	-5.10	0.001581	0.010952	-1.21
255767_at	ribosomal protein L20, putative sim	---	AT1G16740	1.50	0.58	5.10	0.001582	0.010952	-1.21
261513_at	unknown protein contains Pfam pro	---	AT1G71840	1.57	0.65	5.10	0.001582	0.010953	-1.21
247335_at	RNA helicase	---	AT5G63630	1.52	0.60	5.10	0.001583	0.010957	-1.21
267506_at	unknown protein ;supported by full-	---	AT2G45520	1.44	0.53	5.10	0.001585	0.010963	-1.21
245773_at	hypothetical protein predicted by ge	---	AT1G30290	1.51	0.59	5.09	0.001585	0.010963	-1.21
262848_at	unknown protein ; supported by cDI	BPC2	AT1G14685	-1.52	-0.60	-5.09	0.001586	0.010963	-1.21
265295_at	putative DNA replication licensing fa	MCM9	AT2G14050	1.63	0.71	5.09	0.001587	0.010966	-1.21
255688_at	putative RNA helicase similar to AT	---	AT4G00660	1.49	0.57	5.09	0.001589	0.010975	-1.22

259889_at	predicted protein ; supported by cD	---	AT1G76405	1.79	0.84	5.09	0.001589	0.010975	-1.22
267011_at	unknown protein	---	AT2G39230	1.73	0.79	5.09	0.001591	0.010984	-1.22
257802_at	unknown protein	---	AT3G18770	1.71	0.77	5.09	0.001592	0.010985	-1.22
261578_at	acidic ribosomal protein, putative s	---	AT1G01100	1.71	0.77	5.09	0.001593	0.010986	-1.22
264913_at	hypothetical protein predicted by ge	---	AT1G60770	1.99	0.99	5.09	0.001596	0.010999	-1.22
264887_at	unknown protein similar to ripening	---	AT1G23120	1.87	0.91	5.09	0.001596	0.010999	-1.22
263334_at	putative nonsense-mediated mRNA	---	AT2G03820	1.50	0.59	5.09	0.001597	0.011004	-1.22
251658_at	putative protein strictosidine syntha	---	AT3G57020	-2.65	-1.41	-5.09	0.001599	0.011007	-1.22
252312_at	hypothetical protein SF16 protein, l	iqd15	AT3G49380	-1.62	-0.69	-5.09	0.001599	0.011007	-1.22
249658_s_a	N-glyceraldehyde-2-phosphotransfe	---	AT5G36700	-1.79	-0.84	-5.08	0.001603	0.01103	-1.23
250457_at	splicing factor-like protein ; support	---	AT5G09880	1.54	0.63	5.08	0.001603	0.01103	-1.23
264568_at	putative O-GlcNAc transferase simi	---	AT1G05150	-1.67	-0.74	-5.08	0.001604	0.01103	-1.23
247206_at	putative protein similar to unknown	---	AT5G64950	1.35	0.43	5.08	0.001604	0.011031	-1.23
263438_at	unknown protein	---	AT2G28660	1.68	0.75	5.08	0.001606	0.011042	-1.23
245796_at	unknown protein similar to unknown	RCD1	AT1G32230	1.51	0.59	5.08	0.001607	0.011046	-1.23
263431_at	unknown protein ;supported by full-	---	AT2G22170	-13.63	-3.77	-5.08	0.001608	0.011048	-1.23
248747_at	ribosomal protein S27 ;supported b	---	AT5G47930	1.76	0.81	5.08	0.001609	0.011054	-1.23
267259_at	Expressed protein ; supported by fu	---	AT2G23090	1.54	0.62	5.08	0.001612	0.011067	-1.23
259056_at	hypothetical protein similar to Ku70	---	AT3G03420	1.64	0.72	5.08	0.001612	0.011067	-1.23
250714_at	sorting nexin-like protein	SNX1	AT5G06140	1.58	0.66	5.08	0.001613	0.011068	-1.23
256094_at	hypothetical protein predicted by ge	SAUL1	AT1G20780	-1.50	-0.59	-5.08	0.001614	0.011068	-1.23
251858_at	aquaporin MIP - like protein aquap	PIP2;5	AT3G54820	-1.97	-0.98	-5.08	0.001614	0.011068	-1.23
245794_at	endoxyloglucan transferase, putativ	XTR4	AT1G32170	-1.69	-0.76	-5.08	0.001614	0.011068	-1.23
251518_at	putative protein protein CG15643 -	---	AT3G59390	1.52	0.61	5.08	0.001616	0.011073	-1.23
258229_at	unknown protein contains Pfam pro	---	AT3G27700	1.44	0.53	5.08	0.001619	0.011095	-1.24
262703_at	putative auxin-induced protein simil	---	AT1G16510	1.85	0.89	5.07	0.001624	0.011119	-1.24
258726_at	Expressed protein ; supported by fu	---	AT3G11745	1.44	0.52	5.07	0.001625	0.011122	-1.24
249582_at	calmodulin 1 (CAM1) ; supported b	CAM1	AT5G37780	1.63	0.71	5.07	0.001626	0.011127	-1.24
250649_at	thioredoxin-like ; supported by cDN	WCRKC1	AT5G06690	-1.59	-0.67	-5.07	0.001626	0.011127	-1.24
261532_at	amino acid permease, putative sim	---	AT1G71680	-1.45	-0.54	-5.07	0.001627	0.011129	-1.24
251209_at	putative protein several hypothetica	---	AT3G62890	1.65	0.73	5.07	0.001628	0.011134	-1.24
255948_at	hypothetical protein predicted by ge	---	AT1G22060	-1.40	-0.48	-5.07	0.001629	0.011135	-1.24
250038_at	disease resistance protein -like dis	---	AT5G18360	-1.80	-0.85	-5.07	0.001632	0.011146	-1.25
266806_at	unknown protein ;supported by full-	---	AT2G30000	1.66	0.73	5.06	0.001643	0.011219	-1.25
265316_at	unknown protein	---	AT2G20400	1.82	0.86	5.06	0.001645	0.011232	-1.25

249999_at	triacylglycerol lipase-like protein tri	---	AT5G18640	2.14	1.10	5.06	0.001647	0.011242	-1.26
253275_at	Expressed protein ; supported by fu	---	AT4G34215	-1.57	-0.65	-5.06	0.001648	0.011246	-1.26
262701_at	hypothetical protein predicted by ge	---	AT1G16480	1.60	0.68	5.06	0.001649	0.011251	-1.26
257267_at	unknown protein similar to TCP3 G	TCP4	AT3G15030	-1.56	-0.64	-5.06	0.00165	0.011252	-1.26
251360_at	putative protein embryonic abunda	---	AT3G61210	-1.47	-0.55	-5.06	0.001651	0.011252	-1.26
263242_at	unknown protein	---	AT2G31400	1.54	0.62	5.06	0.001652	0.011261	-1.26
262068_at	splicing factor Prp8, putative simila	SUS2	AT1G80070	1.77	0.83	5.06	0.001654	0.011271	-1.26
258392_at	anther development protein, ATA2	ATA20	AT3G15400	-1.70	-0.76	-5.05	0.001656	0.011274	-1.26
255929_at	hypothetical protein predicted by ge	---	AT1G12650	1.59	0.67	5.05	0.001656	0.011274	-1.26
252385_at	putative protein Vps29, Schizosac	MAG1	AT3G47810	1.44	0.52	5.05	0.001656	0.011274	-1.26
266812_at	putative protein kinase contains a p	---	AT2G44830	-1.72	-0.78	-5.05	0.001657	0.011275	-1.26
245738_at	nucellin, putative similar to nucellin	---	AT1G44130	1.98	0.98	5.05	0.001658	0.011281	-1.26
246514_at	DNA-directed RNA polymerase (mi	---	AT5G15700	1.89	0.92	5.05	0.001659	0.011283	-1.26
246794_s_a	putative protein predicted proteins,	---	AT5G06350	2.20	1.14	5.05	0.00166	0.011283	-1.26
256173_at	unknown protein	---	AT1G51730	1.42	0.50	5.05	0.001666	0.011322	-1.27
259769_at	RNA-binding protein MEI2, putative	AML5	AT1G29400	1.89	0.92	5.05	0.001668	0.011334	-1.27
263534_at	putative steroid binding protein ;su	AtMAPR2	AT2G24940	1.57	0.65	5.05	0.001671	0.011352	-1.27
261684_at	hypothetical protein predicted by ge	---	AT1G47400	-4.11	-2.04	-5.04	0.001674	0.011359	-1.27
256008_s_a	allinase, putative similar to allinase	---	AT1G34040	1.50	0.59	5.04	0.001674	0.011359	-1.27
245899_at	putative protein	ALF4	AT5G11030	1.51	0.59	5.04	0.001675	0.011359	-1.27
256948_at	unknown protein	---	AT3G18930	1.45	0.54	5.04	0.001675	0.011359	-1.27
247265_at	putative protein strong similarity to	CPuORF22	AT5G64550	2.45	1.30	5.04	0.001675	0.011359	-1.27
261720_at	hypothetical protein similar to hypot	HDA08	AT1G08460	1.99	1.00	5.04	0.001681	0.011395	-1.28
259700_at	unknown protein	---	AT1G68980	1.36	0.45	5.04	0.001684	0.01141	-1.28
256728_at	glutamyl-tRNA amidotransferase s	---	AT3G25660	-1.77	-0.83	-5.04	0.001685	0.01141	-1.28
252379_at	ABC-type transport protein-like pro	ATH1	AT3G47730	1.53	0.61	5.04	0.001685	0.01141	-1.28
253524_at	predicted protein ; supported by cD	---	AT4G31430	1.54	0.62	5.04	0.001686	0.011412	-1.28
257310_at	ATP-dependent RNA helicase, put	---	AT3G26560	1.56	0.64	5.04	0.001687	0.011412	-1.28
258381_at	kinesin-like protein similar to mitotic	KINESIN-1	AT3G16630	-1.56	-0.64	-5.04	0.001688	0.011412	-1.28
250426_at	ovule development protein - like ov	AIL6	AT5G10510	-1.43	-0.52	-5.04	0.001688	0.011412	-1.28
264996_at	nuclear matrix constituent protein 1	LINC1	AT1G67230	-1.61	-0.68	-5.04	0.001688	0.011412	-1.28
256125_at	pathogenesis-related group 5 prote	ATLP-1	AT1G18250	-1.79	-0.84	-5.04	0.00169	0.011421	-1.28
263847_at	putative glucosyltransferase ; supp	---	AT2G36970	4.44	2.15	5.03	0.001695	0.011436	-1.29
250190_at	ribosomal protein precursor - like ri	---	AT5G14320	-1.46	-0.54	-5.03	0.001695	0.011436	-1.29
265739_at	unknown protein ; supported by cD	AtQSOX2	AT2G01270	1.37	0.45	5.03	0.001695	0.011436	-1.29

248146_at	translation initiation factor-like prote	---	AT5G54940	1.48	0.57	5.03	0.001696	0.011436	-1.29
264295_at	hypothetical protein predicted by ge	---	AT1G78740	-1.38	-0.46	-5.03	0.001698	0.011446	-1.29
258143_at	hypothetical protein	---	AT3G18170	-1.89	-0.92	-5.03	0.001701	0.011462	-1.29
267130_at	hypothetical protein predicted by ge	---	AT2G23390	1.77	0.83	5.03	0.001701	0.011462	-1.29
247328_at	unknown protein ;supported by full-	---	AT5G64130	-1.88	-0.91	-5.03	0.001702	0.011462	-1.29
254549_at	putative protein various predicted p	---	AT4G19880	1.78	0.83	5.03	0.001702	0.011462	-1.29
262261_at	CLE17, putative CLAVATA3/ESR-I	CLE17	AT1G70895	-1.61	-0.69	-5.03	0.001703	0.011462	-1.29
250935_at	polyubiquitin (UBQ3) identical to GI	UBQ3	AT5G03240	1.97	0.98	5.03	0.001704	0.011466	-1.29
263255_at	unknown protein similar to steroid r	---	AT1G10490	1.74	0.80	5.03	0.001704	0.011467	-1.29
264014_at	putative auxin-regulated protein	---	AT2G21210	3.67	1.87	5.03	0.001705	0.011467	-1.29
262625_at	hypothetical protein predicted by ge	---	AT1G06440	1.40	0.49	5.03	0.00171	0.011496	-1.30
250981_at	receptor like protein kinase recepto	---	AT5G03140	-1.75	-0.81	-5.02	0.001711	0.011496	-1.30
258285_at	photosystem I subunit VI precursor	PSAH-1	AT3G16140	-1.41	-0.50	-5.02	0.001711	0.011496	-1.30
264019_at	cyclophilin (CYP2) Same as GB: AF	---	AT2G21130	1.64	0.71	5.02	0.001712	0.011496	-1.30
247771_at	Ran binding protein 1 homolog - lik	RANBP1	AT5G58590	1.57	0.65	5.02	0.001712	0.011496	-1.30
251372_at	putative protein ;supported by full-l	---	AT3G60520	1.67	0.74	5.02	0.001713	0.011496	-1.30
266814_at	homeodomain transcription factor (A	ATHB4	AT2G44910	-1.36	-0.44	-5.02	0.001713	0.011496	-1.30
246923_at	putative multispanning membrane	---	AT5G25100	-1.63	-0.71	-5.02	0.001713	0.011496	-1.30
261286_at	unknown protein ; supported by cDI	---	AT1G35780	-1.68	-0.75	-5.02	0.001714	0.011498	-1.30
249028_at	DNA-damage-inducible protein P ;	POLH	AT5G44740	1.66	0.74	5.02	0.001718	0.01152	-1.30
261930_at	alcohol dehydrogenase ADH, putat	---	AT1G22440	1.79	0.84	5.02	0.00172	0.01153	-1.30
260029_at	endoplasmic reticulum alpha-mann	---	AT1G30000	-1.42	-0.51	-5.02	0.001724	0.01155	-1.30
258254_at	unknown protein contains Pfam pro	---	AT3G26780	1.72	0.78	5.02	0.001725	0.011554	-1.31
251007_at	ribosomal protein L35 - like riboson	---	AT5G02610	1.62	0.70	5.02	0.001726	0.011554	-1.31
247585_at	putative protein predicted proteins,	---	AT5G60680	-1.69	-0.76	-5.02	0.001727	0.011556	-1.31
261986_s_a	cytochrome P450, putative similar t	CYP76C5	AT1G33720	-3.01	-1.59	-5.01	0.001729	0.011562	-1.31
262161_at	signal peptidase subunit, putative s	---	AT1G52600	1.45	0.53	5.01	0.001729	0.011562	-1.31
251500_at	receptor-like protein kinase recepto	---	AT3G59110	-1.46	-0.55	-5.01	0.001729	0.011562	-1.31
251270_at	putative protein	---	AT3G62370	-1.83	-0.87	-5.01	0.001731	0.011564	-1.31
260644_at	hypothetical protein contains similar	---	AT1G53290	-1.66	-0.73	-5.01	0.001731	0.011564	-1.31
266628_at	unknown protein	---	AT2G35330	1.40	0.49	5.01	0.001732	0.011565	-1.31
257144_at	glucose-6-phosphate 1-dehydroger	G6PD5	AT3G27300	1.45	0.54	5.01	0.001734	0.011577	-1.31
258979_at	heat-shock protein (At-hsc70-3) ide	---	AT3G09440	-2.22	-1.15	-5.01	0.001735	0.011579	-1.31
254145_at	hypothetical protein	---	AT4G24700	2.36	1.24	5.01	0.001739	0.011599	-1.31
247520_at	cytochrome c oxidase subunit - like	---	AT5G61310	2.07	1.05	5.01	0.00174	0.011604	-1.32

258139_at	heat shock transcription factor HSF	HSFC1	AT3G24520	1.68	0.75	5.01	0.001741	0.011608	-1.32
245307_at	gibberellin oxidase-like protein non-	---	AT4G16770	-1.59	-0.67	-5.01	0.001744	0.011621	-1.32
255759_at	hypothetical protein predicted by ge	---	AT1G16790	1.51	0.60	5.01	0.001744	0.011621	-1.32
261278_at	hypothetical protein predicted by ge	DGL	AT1G05800	-1.45	-0.53	-5.01	0.001745	0.011627	-1.32
248132_at	SGP1 monomeric G-protein (emb|C	SGP1	AT5G54840	1.85	0.88	5.01	0.001746	0.011629	-1.32
254711_at	putative protein	---	AT4G18060	-1.57	-0.65	-5.01	0.001747	0.011629	-1.32
254218_at	putative receptor kinase receptor-li	---	AT4G23740	-1.53	-0.61	-5.00	0.001751	0.011656	-1.32
261204_s_a	Eukaryotic peptide chain release fa	ERF1-2	AT1G12920	1.36	0.45	5.00	0.001755	0.011678	-1.32
262581_at	unknown protein ESTs gb|T22508,	---	AT1G15370	-1.38	-0.46	-5.00	0.001756	0.011683	-1.33
265974_at	unknown protein	---	---	-1.98	-0.98	-5.00	0.001757	0.011683	-1.33
262699_at	unknown protein	---	AT1G75980	1.51	0.59	5.00	0.001758	0.011684	-1.33
245438_at	growth regulator like protein	---	AT4G16650	1.47	0.55	5.00	0.001758	0.011684	-1.33
255603_at	hypothetical protein	---	AT4G01040	-1.42	-0.50	-5.00	0.001759	0.011684	-1.33
251308_at	putative protein several hypothetica	GAUT1	AT3G61130	-1.46	-0.55	-5.00	0.001761	0.011693	-1.33
264906_at	putative mitochondrial phosphate tr	---	AT2G17270	1.74	0.80	5.00	0.001761	0.011693	-1.33
252077_at	putative protein several putative my	---	AT3G51720	1.61	0.69	5.00	0.001764	0.011704	-1.33
250921_at	Expressed protein ; supported by fu	---	AT5G03460	1.69	0.76	5.00	0.001765	0.011704	-1.33
247612_at	ATPase - like protein ATPase, Sola	---	AT5G60730	1.53	0.62	5.00	0.001766	0.011709	-1.33
247775_at	phosphoinositide-specific phospho	PLC5	AT5G58690	-2.02	-1.02	-5.00	0.001767	0.011714	-1.33
250622_at	putative transcription factor AP2 do	---	AT5G07310	-1.43	-0.51	-4.99	0.001771	0.011731	-1.33
256419_at	RNA-binding protein similar to GI:82	---	AT1G33470	-1.55	-0.63	-4.99	0.001772	0.011733	-1.34
250083_at	glutathione S-transferase-like prote	ATGSTF12	AT5G17220	-1.57	-0.65	-4.99	0.001772	0.011733	-1.34
247004_at	putative protein similar to unknown	DG1	AT5G67570	1.54	0.62	4.99	0.001773	0.011734	-1.34
267524_at	unknown protein	---	AT2G30600	-1.81	-0.86	-4.99	0.001774	0.011739	-1.34
250268_s_a	putative protein putative secreted p	---	AT5G12950	1.74	0.80	4.99	0.001781	0.011778	-1.34
259051_at	unknown protein similar to CGI-86	---	AT3G03330	1.52	0.61	4.99	0.001782	0.011785	-1.34
258542_at	unknown protein	---	AT3G07030	1.60	0.68	4.99	0.001785	0.011796	-1.34
246199_at	putative protein ;supported by full-le	---	AT4G36530	-1.83	-0.87	-4.99	0.001785	0.011796	-1.34
261911_at	ribosomal protein L7, putative simil	---	AT1G80750	2.19	1.13	4.98	0.001787	0.011805	-1.34
257459_at	unknown protein	---	AT1G24040	1.49	0.57	4.98	0.001788	0.011807	-1.34
261208_at	unknown protein ; supported by cDI	---	AT1G12930	1.70	0.77	4.98	0.001789	0.01181	-1.35
245870_at	hypothetical protein similar to hypot	---	AT1G26300	-1.44	-0.53	-4.98	0.001791	0.011816	-1.35
246573_at	copper amine oxidase, putative sin	---	AT1G31690	-1.74	-0.80	-4.98	0.001791	0.011816	-1.35
245708_at	pectate lyase-like protein	---	AT5G04310	-1.61	-0.69	-4.98	0.001793	0.011826	-1.35
256146_at	delta-adaptin, putative similar to de	delta-ADR	AT1G48760	-1.44	-0.53	-4.98	0.001796	0.011838	-1.35

247124_at	prolyl 4-hydroxylase, alpha subunit	---	AT5G66060	-1.43	-0.51	-4.98	0.001796	0.011838	-1.35
264702_at	hypothetical protein contains similar	---	AT1G70190	1.56	0.64	4.98	0.001797	0.011838	-1.35
252377_at	putative peptide transporter peptide	---	AT3G47960	-1.68	-0.75	-4.98	0.001798	0.011846	-1.35
256220_at	unknown protein	---	AT1G56230	1.65	0.72	4.98	0.0018	0.011855	-1.35
265253_at	putative peptide/amino acid transpo	---	AT2G02020	-1.42	-0.50	-4.98	0.001802	0.011862	-1.35
250060_at	chromo domain protein polycomb-l	TFL2	AT5G17690	1.60	0.68	4.98	0.001803	0.011863	-1.35
260928_at	hypothetical protein similar to putati	GATL5	AT1G02720	-1.50	-0.58	-4.98	0.001803	0.011863	-1.35
250011_at	putative protein crp1 protein, maize	---	AT5G18475	-1.42	-0.51	-4.98	0.001804	0.011863	-1.35
255901_at	GDP-L-fucose synthetase, putative	GER2	AT1G17890	-1.85	-0.89	-4.98	0.001804	0.011863	-1.35
262298_at	unknown protein Similar to gb|AF15	---	AT1G27530	1.39	0.48	4.98	0.001806	0.011867	-1.36
254011_at	putative protein transcription termir	---	AT4G26370	1.55	0.63	4.98	0.001806	0.011867	-1.36
255000_at	S18.A ribosomal protein ;supportec	RPS18C	AT4G09800	1.82	0.87	4.97	0.001807	0.011867	-1.36
267519_at	putative VP1/ABI3 family regulatory	HSI2	AT2G30470	-1.75	-0.80	-4.97	0.001807	0.011867	-1.36
247114_at	unknown protein ; supported by cD	---	AT5G65910	-1.58	-0.66	-4.97	0.001807	0.011867	-1.36
253820_at	hypothetical protein	---	AT4G28370	1.47	0.55	4.97	0.001809	0.011873	-1.36
266846_at	unknown protein	---	AT2G25970	1.42	0.51	4.97	0.00181	0.011877	-1.36
249426_at	putative RNA helicase putative ATI	---	AT5G39840	2.53	1.34	4.97	0.001814	0.0119	-1.36
256041_at	unknown protein	---	AT1G07230	1.68	0.75	4.97	0.001816	0.011906	-1.36
256622_at	unknown protein	AtHB34	AT3G28920	1.38	0.47	4.97	0.001819	0.01191	-1.36
250803_at	putative protein contains similarity t	---	AT5G04980	1.88	0.91	4.97	0.001819	0.01191	-1.36
247380_at	4-coumarate-CoA ligase-like protein	---	AT5G63380	-1.69	-0.75	-4.97	0.001819	0.01191	-1.36
249829_at	c-myc binding protein MM-1-like pro	PDF5	AT5G23290	1.47	0.56	4.97	0.001819	0.01191	-1.36
259110_at	unknown protein	---	AT3G05570	1.75	0.81	4.97	0.001819	0.01191	-1.36
249146_at	putative protein contains similarity t	---	AT5G43310	-1.45	-0.54	-4.97	0.001821	0.01191	-1.36
267161_at	hypothetical protein predicted by ge	---	AT2G37680	-1.42	-0.50	-4.97	0.001821	0.01191	-1.36
246193_at	Expressed protein ; supported by fu	---	---	1.47	0.55	4.97	0.001823	0.011916	-1.37
250418_at	putative protein predicted proteins,	---	AT5G11240	2.19	1.13	4.97	0.001823	0.011916	-1.37
256919_at	hypothetical protein predicted by ge	---	AT3G18970	1.55	0.63	4.97	0.001823	0.011916	-1.37
245597_at	salt-inducible protein homolog	---	AT4G14190	1.47	0.56	4.97	0.001824	0.011916	-1.37
258184_at	two-component phosphorelay med	AHP1	AT3G21510	-1.74	-0.80	-4.96	0.001828	0.011941	-1.37
261582_at	unknown protein ; supported by cDI	GIF2	AT1G01160	1.44	0.53	4.96	0.001829	0.011941	-1.37
260747_at	unknown protein ; supported by cDI	---	AT1G78420	1.40	0.48	4.96	0.001831	0.011955	-1.37
260619_at	glyoxalase I, putative similar to GB	---	AT1G08110	1.67	0.74	4.96	0.001833	0.011955	-1.37
251670_at	putative protein translation releasir	---	AT3G57190	1.64	0.72	4.96	0.001833	0.011955	-1.37
248952_at	putative protein similar to unknown	---	AT5G45410	1.56	0.64	4.96	0.001833	0.011955	-1.37

253956_at	fimbrin-like protein (ATFIM1) ; supp	FIM1	AT4G26700	-1.98	-0.98	-4.96	0.001834	0.011955	-1.37
248461_s_a	fumarate hydratase ; supported by	FUM1	AT2G47510	-1.58	-0.66	-4.96	0.001835	0.011955	-1.37
257797_at	hypothetical protein contains Pfam	---	AT3G15940	-1.55	-0.63	-4.96	0.001835	0.011955	-1.37
250406_at	enhancer of rudimentary ; supporte	ER	AT5G10810	1.76	0.82	4.96	0.001838	0.011968	-1.37
263761_at	putative fructose bisphosphate aldo	---	AT2G21330	-1.73	-0.79	-4.96	0.001839	0.01197	-1.38
254845_at	hydroxymethylglutaryl-CoA synthas	MVA1	AT4G11820	-1.70	-0.76	-4.96	0.001839	0.011971	-1.38
256071_at	ubiquitin, putative similar to ubiquit	---	AT1G13640	1.84	0.88	4.96	0.00184	0.011973	-1.38
251008_at	putative protein ;supported by full-le	---	AT5G02710	1.61	0.68	4.95	0.001849	0.01202	-1.38
251794_at	mannose-1-phosphate guanylyltrar	---	AT3G55590	-1.42	-0.50	-4.95	0.001851	0.012026	-1.38
251098_at	light-inducible protein ATLS1	---	AT5G01650	1.38	0.47	4.95	0.001851	0.012026	-1.38
260946_at	hypothetical protein predicted by ge	---	AT1G06010	1.58	0.66	4.95	0.001854	0.012044	-1.38
267347_at	hypothetical protein predicted by ge	---	AT2G39950	1.44	0.52	4.95	0.001856	0.012049	-1.39
266587_at	unknown protein ; supported by ful	---	AT2G14880	1.57	0.66	4.95	0.00186	0.012073	-1.39
267214_at	unknown protein ; supported by cD	---	AT2G43970	1.70	0.76	4.95	0.001864	0.012095	-1.39
251078_at	putative protein hypothetical protein	---	AT5G01990	1.87	0.91	4.95	0.001865	0.012098	-1.39
257786_at	hypothetical protein predicted by ge	---	AT3G26990	1.71	0.77	4.95	0.001866	0.012099	-1.39
259816_at	RING-H2 finger protein RHY1a ide	---	AT1G49850	1.50	0.58	4.94	0.001867	0.012102	-1.39
260201_at	F12A21.27 hypothetical protein;sup	---	AT1G67600	2.28	1.19	4.94	0.001867	0.012102	-1.39
258270_at	putative lysophospholipase similar t	---	AT3G15650	-2.48	-1.31	-4.94	0.001868	0.012103	-1.39
258994_at	unknown protein similar to putative	RAPTOR1	AT3G08850	1.42	0.50	4.94	0.00187	0.012111	-1.39
267304_at	putative Fe(II) transport protein	ZIP6	AT2G30080	1.66	0.73	4.94	0.001871	0.012113	-1.39
257395_at	putative salt-inducible protein	---	AT2G15630	1.70	0.76	4.94	0.001872	0.012113	-1.39
250045_at	putative protein mRNA (orf04), Ara	---	AT5G17700	-1.86	-0.90	-4.94	0.001872	0.012113	-1.40
254965_at	putative protein other hypothetical p	---	AT4G11090	-1.41	-0.49	-4.94	0.001872	0.012113	-1.40
263320_at	putative galactinol synthase ;suppo	AtGolS1	AT2G47180	-3.61	-1.85	-4.94	0.001873	0.012114	-1.40
248410_at	putative protein similar to unknown	---	AT5G51570	1.46	0.55	4.94	0.001874	0.012116	-1.40
245195_at	F12A21.13 putative photosystem II	PSBY	AT1G67740	-1.47	-0.55	-4.94	0.001875	0.012116	-1.40
246021_at	ascorbate oxidase-like protein asc	---	AT5G21100	1.89	0.92	4.94	0.001876	0.01212	-1.40
254349_at	hypothetical protein	---	AT4G22250	1.56	0.64	4.94	0.00188	0.012141	-1.40
248969_at	unknown protein ; supported by cD	---	AT5G45310	2.38	1.25	4.94	0.001881	0.012141	-1.40
267298_at	putative homeodomain transcription	BLH4	AT2G23760	-1.75	-0.81	-4.94	0.001886	0.012168	-1.40
247065_s_a	disease resistance protein-like	---	AT5G66900	1.67	0.74	4.94	0.001886	0.012168	-1.40
267199_at	hypothetical protein predicted by ge	---	AT2G30990	-3.55	-1.83	-4.93	0.001888	0.012176	-1.40
248336_at	unknown protein ; supported by cD	---	AT5G52420	1.91	0.94	4.93	0.001889	0.012176	-1.40
255830_at	putative PRP19-like spliceosomal p	---	AT2G33340	2.12	1.08	4.93	0.00189	0.012176	-1.41

248797_at	putative protein similar to unknown	---	AT5G47210	1.57	0.65	4.93	0.00189	0.012176	-1.41
247541_at	structural protein - like glycine-rich	---	AT5G61660	-1.58	-0.66	-4.93	0.00189	0.012176	-1.41
264130_at	hypothetical protein predicted by ge	---	AT1G79160	1.64	0.71	4.93	0.001898	0.012226	-1.41
248746_at	NADH dehydrogenase 10.5K chain	---	AT5G47890	1.91	0.93	4.93	0.001899	0.012226	-1.41
250021_at	casein kinase-like protein	---	AT5G18190	-1.35	-0.43	-4.93	0.001907	0.012274	-1.42
251930_at	putative protein several hypothetica	ATRBL4	AT3G53780	1.62	0.69	4.92	0.00191	0.012284	-1.42
253092_at	putative protein various predicted p	---	AT4G37380	1.99	0.99	4.92	0.00191	0.012284	-1.42
250920_at	putative protein ;supported by full-l	---	AT5G03390	-2.03	-1.02	-4.92	0.00191	0.012284	-1.42
260359_at	hypothetical protein predicted by ge	---	AT1G69210	1.70	0.77	4.92	0.001911	0.012285	-1.42
247138_at	3-dehydroquinate synthase-like pro	---	AT5G66120	1.83	0.87	4.92	0.001911	0.012285	-1.42
251024_at	putative protein hypothetical protein	---	AT5G02180	-1.62	-0.69	-4.92	0.001915	0.012304	-1.42
252770_at	putative protein cellular nucleic aci	---	AT3G42860	1.55	0.63	4.92	0.001915	0.012304	-1.42
250522_at	cleft lip and palate associated trans	---	AT5G08500	1.44	0.52	4.92	0.001916	0.012304	-1.42
259152_at	hypothetical protein similar to putati	---	AT3G10210	1.37	0.45	4.92	0.001917	0.012309	-1.42
250852_at	putative protein BRL protein, Homo	---	AT5G04670	1.39	0.48	4.92	0.001918	0.012309	-1.42
267609_at	unknown protein similar to GP|5221	---	AT2G26780	1.33	0.42	4.92	0.001919	0.012309	-1.42
261077_at	protein phosphatase 2C, putative s	---	AT1G07430	1.87	0.90	4.92	0.001919	0.012309	-1.42
251177_at	cyclophylin -like protein CDC28/cd	---	AT3G63400	1.61	0.69	4.92	0.001921	0.012315	-1.42
257900_at	hypothetical protein predicted by ge	---	AT3G28420	-1.41	-0.50	-4.92	0.001922	0.012315	-1.42
257868_at	unknown protein	RIN4	AT3G25070	1.68	0.75	4.92	0.001922	0.012315	-1.42
264128_at	unknown protein	---	AT1G79190	1.46	0.55	4.92	0.001923	0.012315	-1.42
258449_s_a	putative RNA helicase similar to RN	PMH1	AT3G22310	1.80	0.84	4.92	0.001924	0.012315	-1.42
260176_at	unknown protein similar to Pi starva	---	AT1G71950	-1.52	-0.60	-4.92	0.001926	0.012328	-1.43
264351_at	unknown protein Contains similarity	---	AT1G03370	-1.51	-0.59	-4.92	0.001927	0.012328	-1.43
257509_at	unknown protein	---	AT1G63190	1.52	0.61	4.92	0.001929	0.012341	-1.43
264736_at	oligopeptide transporter, putative s	---	AT1G62200	1.70	0.76	4.91	0.001931	0.012345	-1.43
255946_at	glycine hydroxymethyltransferase,	SHM6	AT1G22020	1.35	0.43	4.91	0.001932	0.012347	-1.43
255635_at	Shaggy related protein kinase tetha	ATSK32	AT4G00720	1.54	0.62	4.91	0.001933	0.012349	-1.43
255216_s_a	putative peptidase	---	AT4G07670	2.12	1.09	4.91	0.001937	0.012374	-1.43
259761_at	putative acyl-CoA synthetase simila	LACS9	AT1G77590	-1.79	-0.84	-4.91	0.001939	0.012382	-1.43
259041_at	unknown protein similar to putative	ATCSLC6	AT3G07330	-1.79	-0.84	-4.91	0.00194	0.012385	-1.43
253762_at	putative protein conserved hypothe	---	AT4G28830	1.50	0.58	4.91	0.001945	0.012411	-1.44
255128_at	putative protein glutamic acid-rich p	---	AT4G08310	1.65	0.72	4.91	0.001946	0.012415	-1.44
260679_at	unknown protein	---	AT1G07990	1.42	0.50	4.91	0.001947	0.012415	-1.44
267346_at	coronatine-insensitive 1 (COI1), At	COI1	AT2G39940	1.68	0.75	4.91	0.001947	0.012415	-1.44

259872_at	putative translation initiation factor I	---	AT1G76810	1.43	0.52	4.91	0.001947	0.012415	-1.44
247280_at	phi-1-like protein ;supported by full-	EXL2	AT5G64260	-2.31	-1.21	-4.91	0.001949	0.012424	-1.44
263108_at	hypothetical protein similar to putati	---	AT1G65240	-3.60	-1.85	-4.90	0.001952	0.012436	-1.44
264131_at	unknown protein	---	AT1G79150	1.81	0.86	4.90	0.001956	0.012455	-1.44
264084_at	putative kinesin light chain	---	AT2G31240	1.48	0.57	4.90	0.001956	0.012455	-1.44
260190_at	putative lipoxygenase similar to 13-	---	AT1G67560	-1.96	-0.97	-4.90	0.001956	0.012455	-1.44
262172_at	ribosomal protein S9, putative simi	RPS9	AT1G74970	-1.47	-0.56	-4.90	0.001959	0.012467	-1.44
253001_at	Expressed protein ; supported by cl	---	AT4G38490	1.36	0.44	4.90	0.001961	0.012471	-1.45
262372_at	hypothetical protein predicted by ge	---	AT1G73130	1.95	0.96	4.90	0.001962	0.012471	-1.45
246435_at	hypothetical protein ; supported by	---	AT5G17460	1.39	0.47	4.90	0.001962	0.012471	-1.45
250808_at	putative protein contains similarity t	ATG18E	AT5G05150	-1.61	-0.68	-4.90	0.001962	0.012471	-1.45
266320_at	hypothetical protein predicted by ge	---	AT2G46640	-1.94	-0.96	-4.90	0.001963	0.012473	-1.45
260086_at	hypothetical protein predicted by ge	---	AT1G63240	-1.73	-0.79	-4.90	0.001965	0.012484	-1.45
250762_at	putative protein similar to unknown	---	AT5G05990	2.36	1.24	4.89	0.001975	0.012537	-1.45
263156_at	myrosinase-associated protein, put	---	AT1G54030	-2.20	-1.14	-4.89	0.001975	0.012537	-1.45
263759_at	hypothetical protein predicted by ge	---	AT2G21290	1.51	0.59	4.89	0.001978	0.012554	-1.45
261896_at	mRNA export protein, putative simi	---	AT1G80670	1.60	0.68	4.89	0.001983	0.012582	-1.46
261183_at	hypothetical protein predicted by ge	EMB2756	AT1G34550	-1.61	-0.69	-4.89	0.001984	0.012583	-1.46
249845_at	putative protein similar to unknown	---	AT5G23610	1.44	0.52	4.89	0.001985	0.012585	-1.46
264594_at	hypothetical protein ; supported by	ATSERAT3	AT2G17640	1.51	0.59	4.89	0.001989	0.012602	-1.46
245714_at	RNA-binding protein-like	---	AT5G04280	1.47	0.56	4.89	0.001991	0.012614	-1.46
253683_at	cytidine deaminase 4 (CDA4)	---	AT4G29650	-1.45	-0.53	-4.88	0.001996	0.012643	-1.47
248453_at	unknown protein	---	AT5G51340	1.46	0.55	4.88	0.001999	0.012656	-1.47
254350_at	putative protein hypothetical protei	---	AT4G22280	1.38	0.46	4.88	0.002003	0.012677	-1.47
245118_at	putative thioredoxin reductase The	NTRC	AT2G41680	-1.44	-0.53	-4.88	0.002004	0.012677	-1.47
248398_at	sorbitol dehydrogenase-like protein	---	AT5G51970	1.57	0.65	4.88	0.002005	0.012684	-1.47
260655_at	pathogenesis-related protein 5 pre	---	AT1G19320	-2.27	-1.18	-4.88	0.002013	0.012728	-1.47
266188_at	unknown protein	---	AT2G39000	1.56	0.64	4.88	0.002013	0.012728	-1.47
260423_at	unknown protein	ATEXO70D	AT1G72470	-2.50	-1.32	-4.88	0.002015	0.012734	-1.48
265306_at	unknown protein	---	AT2G20320	1.79	0.84	4.88	0.002016	0.012741	-1.48
246017_at	putative protein predicted proteins f	---	AT5G10730	1.41	0.50	4.87	0.002018	0.012748	-1.48
262417_at	hypothetical protein similar to hypot	ATSIRB	AT1G50170	1.36	0.45	4.87	0.002024	0.012783	-1.48
263076_at	putative protein kinase/endoribonuc	IRE1A	AT2G17520	-1.46	-0.55	-4.87	0.002025	0.012783	-1.48
247854_at	contains similarity to phosphoestera	---	AT5G58200	1.53	0.61	4.87	0.002026	0.012786	-1.48
256386_at	cytochrome P450, putative contain	---	AT1G66540	-1.39	-0.48	-4.87	0.002028	0.012798	-1.48

263193_at	acetyl-CoA carboxylase, putative s	---	AT1G36050	-1.44	-0.53	-4.87	0.002034	0.012828	-1.49
260103_at	unknown protein	---	AT1G35430	-1.46	-0.54	-4.87	0.002035	0.012831	-1.49
255094_at	putative zinc finger protein	ORTHL	AT4G08590	-1.91	-0.93	-4.87	0.002037	0.012838	-1.49
257504_at	dynein light chain, putative similar t	ROPGEF1	AT1G52240	-2.18	-1.12	-4.87	0.002038	0.012839	-1.49
253027_at	putative protein leaf protein (PnC4	---	AT4G38150	1.89	0.92	4.87	0.002039	0.012843	-1.49
262088_at	unknown protein	---	AT1G56020	-1.44	-0.53	-4.86	0.002039	0.012843	-1.49
265310_at	hypothetical protein predicted by ge	---	AT2G20280	1.44	0.53	4.86	0.002041	0.012843	-1.49
245304_at	hypothetical protein ; supported by	---	AT4G15630	-3.14	-1.65	-4.86	0.002041	0.012843	-1.49
252473_s_a	hypothetical protein several hypothe	---	AT3G46610	1.81	0.85	4.86	0.002043	0.012847	-1.49
265015_at	putative Ta11-1 reverse transcripta	---	---	-1.35	-0.43	-4.86	0.002043	0.012847	-1.49
254433_at	putative protein tripeptidyl-peptidas	TPP2	AT4G20850	1.38	0.47	4.86	0.002049	0.012877	-1.49
261858_at	Expressed protein ; supported by fu	---	AT1G50570	1.40	0.49	4.86	0.002052	0.012892	-1.49
262741_at	hypothetical protein	---	AT1G28760	1.42	0.50	4.86	0.002054	0.012905	-1.50
261701_at	hypothetical protein similar to hypot	HAF01	AT1G32750	1.35	0.43	4.86	0.002056	0.01291	-1.50
255992_at	unknown protein	---	AT1G29790	-1.55	-0.63	-4.86	0.002057	0.012917	-1.50
264200_at	putative invertase Similar to LIM17	---	AT1G22650	-1.89	-0.92	-4.86	0.002059	0.012919	-1.50
260060_at	feebly-like protein similar to feebly p	---	AT1G73680	1.63	0.71	4.86	0.002059	0.012919	-1.50
247933_at	putative protein non-consensus CG	---	AT5G56980	-2.78	-1.47	-4.86	0.002062	0.012935	-1.50
253691_at	putative protein predicted proteins,	EMB2752	AT4G29660	1.36	0.45	4.85	0.002064	0.012942	-1.50
256512_at	Expressed protein ; supported by fu	---	AT1G33265	1.59	0.67	4.85	0.002066	0.012951	-1.50
254181_at	FtsH protease, putative contains si	---	AT4G23940	-1.39	-0.47	-4.85	0.002068	0.012961	-1.50
258524_at	acetyl-coA dehydrogenase, putativ	IBR3	AT3G06810	1.56	0.64	4.85	0.002071	0.012972	-1.50
261254_at	Expressed protein ; supported by fu	---	AT1G05805	-1.51	-0.59	-4.85	0.002073	0.012982	-1.51
252475_s_a	putative protein several hypothetica	PCL1	AT3G46640	-1.63	-0.70	-4.85	0.002073	0.012982	-1.51
267396_at	hypothetical protein predicted by gr	---	AT2G44270	1.61	0.69	4.85	0.002078	0.013002	-1.51
255615_at	hypothetical protein	---	AT4G01290	1.34	0.42	4.85	0.002078	0.013002	-1.51
247659_at	DNA-directed RNA polymerase - lik	NRPC1	AT5G60040	2.56	1.36	4.85	0.002079	0.013005	-1.51
266363_at	hypothetical protein predicted by ge	---	AT2G41250	2.03	1.02	4.85	0.002079	0.013005	-1.51
253319_at	putative protein various predicted p	EMB2758	AT4G33990	1.82	0.87	4.85	0.002083	0.013024	-1.51
259535_at	hypothetical protein	---	AT1G12280	1.77	0.82	4.84	0.002086	0.01304	-1.51
252507_at	putative protein predicted protein, S	aTNUDT9	AT3G46200	-1.78	-0.83	-4.84	0.002091	0.013067	-1.52
262960_at	unknown protein ; supported by cDI	---	AT1G54320	1.48	0.56	4.84	0.002092	0.013068	-1.52
255826_at	putative uroporphyrinogen decarbo	HEME2	AT2G40490	-1.50	-0.58	-4.84	0.002094	0.013078	-1.52
250703_at	putative protein similar to unknown	---	AT5G06360	1.64	0.72	4.84	0.002097	0.013094	-1.52
249694_at	glucose-6-phosphate dehydrogenas	G6PD1	AT5G35790	-1.37	-0.45	-4.84	0.002098	0.013094	-1.52

263105_at	putative N-acetylglucosaminyltransf	---	AT2G05320	1.40	0.49	4.84	0.002098	0.013094	-1.52
266668_at	hypothetical protein predicted by gr	---	AT2G29760	2.18	1.13	4.84	0.002101	0.013108	-1.52
259928_at	DNA polymerase type I, putative si	---	AT1G34380	1.93	0.95	4.84	0.002107	0.013141	-1.52
247856_at	receptor-like protein kinase	---	AT5G58300	-1.71	-0.78	-4.84	0.002107	0.013141	-1.52
248555_at	DNA repair protein-like	---	AT5G50340	1.49	0.58	4.84	0.002108	0.013143	-1.52
252277_at	alpha NAC-like protein Nascent po	NACA2	AT3G49470	-1.36	-0.44	-4.83	0.00211	0.01315	-1.53
255724_at	hypothetical protein	BRCA2(IV)	AT4G00020	-1.41	-0.50	-4.83	0.002111	0.013155	-1.53
259227_at	putative 3 exoribonuclease contain	---	AT3G07750	2.81	1.49	4.83	0.002113	0.013158	-1.53
264705_at	putative leucyl-tRNA synthetase Str	---	AT1G09620	1.54	0.63	4.83	0.002113	0.013158	-1.53
247295_at	putative protein similar to unknown	---	AT5G64180	1.50	0.59	4.83	0.002115	0.013165	-1.53
250923_at	putative protein ; supported by full-	CDC25	AT5G03455	1.60	0.68	4.83	0.002118	0.013181	-1.53
248850_at	putative protein contains similarity t	---	AT5G46550	1.49	0.58	4.83	0.002126	0.013216	-1.53
246461_at	putative protein AAA family protein	---	AT5G16930	2.68	1.42	4.83	0.002126	0.013216	-1.53
247385_at	putative protein similar to unknown	emb2746	AT5G63420	1.74	0.80	4.83	0.002128	0.013216	-1.53
265647_at	hypothetical protein predicted by ge	---	AT2G27410	-1.46	-0.54	-4.83	0.002129	0.013216	-1.53
245729_at	unknown protein	---	AT1G73490	1.60	0.67	4.83	0.002129	0.013216	-1.54
253813_at	putative protein hypothetical protei	---	AT4G28150	-1.52	-0.60	-4.83	0.002129	0.013216	-1.54
262136_at	auxin response factor, putative sim	ARF17	AT1G77850	1.43	0.52	4.83	0.00213	0.013216	-1.54
253837_at	putative proline-rich protein extens	---	AT4G27850	-1.43	-0.51	-4.83	0.00213	0.013216	-1.54
259210_at	hypothetical protein nearly identical	HY2	AT3G09150	1.67	0.74	4.83	0.002131	0.013216	-1.54
267218_at	Expressed protein ; supported by fu	---	AT2G02515	-1.45	-0.54	-4.83	0.002131	0.013216	-1.54
248147_at	putative protein contains similarity t	ATRBP45A	AT5G54900	1.48	0.57	4.82	0.002137	0.01325	-1.54
263143_at	hypothetical protein Contains PF|00	---	AT1G03100	1.55	0.63	4.82	0.002138	0.013254	-1.54
245758_at	unknown protein contains similarity	KAN2	AT1G32240	1.84	0.88	4.82	0.002142	0.013271	-1.54
257618_at	protein kinase, putative similar to T	---	AT3G24715	-1.35	-0.43	-4.82	0.002145	0.013291	-1.54
263114_at	putative photosystem I reaction cer	PSAD-2	AT1G03130	-1.51	-0.59	-4.82	0.002147	0.013292	-1.54
246882_at	putative protein predicted proteins,	---	AT5G26180	1.39	0.48	4.82	0.002147	0.013292	-1.54
250404_at	CCR4-ASSOCIATED FACTOR -li	---	AT5G10960	1.38	0.46	4.82	0.002155	0.013335	-1.55
257407_at	unknown protein	---	AT1G27100	-1.45	-0.54	-4.81	0.002156	0.013341	-1.55
251833_at	putative protein various predicted p	---	AT3G55160	1.43	0.52	4.81	0.002157	0.013341	-1.55
254035_at	putative phosphatidylserine decarb	PSD3	AT4G25970	1.46	0.54	4.81	0.002159	0.013348	-1.55
252823_at	Expressed protein ; supported by fu	---	AT4G40045	-1.49	-0.58	-4.81	0.00216	0.013348	-1.55
256248_at	hypothetical protein	---	AT3G66652	1.77	0.83	4.81	0.00216	0.013348	-1.55
255133_at	putative protein (fragment) glutami	---	AT4G08320	1.55	0.64	4.81	0.002163	0.013353	-1.55
262164_at	unknown protein ; supported by cDI	---	AT1G78070	1.68	0.74	4.81	0.002164	0.013357	-1.55

259967_at	putative DNA-binding protein contai	---	AT1G76510	1.39	0.48	4.81	0.002166	0.013366	-1.55
251273_at	serine/threonine-protein kinase-like	---	AT3G61960	1.81	0.86	4.81	0.002169	0.013377	-1.56
263502_s_a	hypothetical protein	---	AT2G07675	1.86	0.89	4.81	0.002172	0.013393	-1.56
253280_at	poly(A)-binding protein non-conser	PAB2	AT4G34110	1.39	0.48	4.81	0.002174	0.013401	-1.56
255702_at	subtilisin-like serine protease XSP1	XSP1	AT4G00230	-1.78	-0.83	-4.81	0.002176	0.013403	-1.56
248690_at	acetyl-CoA C-acetyltransferase ; su	ACAT2	AT5G48230	1.38	0.46	4.81	0.002177	0.013405	-1.56
245289_at	Expressed protein ; supported by cl	---	AT4G15470	-1.36	-0.45	-4.80	0.002181	0.013422	-1.56
266347_at	putative ATP-dependent DNA helica	---	AT2G01440	1.66	0.73	4.80	0.00219	0.013473	-1.57
267187_s_a	putative methylenetetrahydrofolate	MTHFR2	AT2G44160	-1.75	-0.80	-4.80	0.002191	0.013477	-1.57
250526_at	pyruvate kinase	---	AT5G08570	1.45	0.54	4.80	0.002191	0.013477	-1.57
245575_at	centromere protein homolog	---	AT4G14760	-1.46	-0.54	-4.80	0.002193	0.013483	-1.57
253276_at	caffeoyl-CoA O-methyltransferase	---	AT4G34050	1.78	0.83	4.80	0.002201	0.013516	-1.57
253477_at	L-ascorbate peroxidase - like protei	APX6	AT4G32320	2.03	1.02	4.80	0.002201	0.013516	-1.57
262509_at	hypothetical protein similar to hypot	CRR22	AT1G11290	1.53	0.62	4.80	0.002201	0.013516	-1.57
265911_at	unknown protein ;supported by full-	---	AT2G25670	1.39	0.47	4.80	0.002202	0.013516	-1.57
261089_at	protein kinase APK1A identical to S	APK1A	AT1G07570	-1.83	-0.87	-4.80	0.002202	0.013516	-1.57
265356_at	putative TRAP protein	---	AT2G16595	1.65	0.72	4.80	0.002203	0.013518	-1.57
262686_at	proteasome regulatory subunit S3,	---	AT1G75990	-1.54	-0.63	-4.80	0.002204	0.013519	-1.57
263035_at	splicing factor, putative similar to R	RSZP21	AT1G23860	1.65	0.72	4.79	0.002205	0.013523	-1.57
245706_at	putative protein	---	AT5G04310	-1.47	-0.55	-4.79	0.002206	0.013526	-1.57
259071_at	unknown protein similar to hin1 GB:	NHL2	AT3G11650	-1.92	-0.94	-4.79	0.002214	0.01357	-1.58
253674_at	UDP-N-acetylglucosamine O-acyltr	---	AT4G29540	1.46	0.55	4.79	0.002215	0.013575	-1.58
254694_at	putative protein	---	AT4G17900	1.71	0.77	4.79	0.002216	0.013575	-1.58
253873_at	hypothetical protein ;supported by f	PRA1.H	AT4G27540	1.44	0.52	4.79	0.002218	0.013586	-1.58
266599_at	unknown protein	---	AT2G46100	-1.41	-0.50	-4.79	0.002223	0.013615	-1.58
246290_at	calmodulin-3 ;supported by full-leng	CAM3	AT3G56800	-1.40	-0.49	-4.79	0.002225	0.013623	-1.58
266299_at	glutathione S-transferase identical t	ATGSTU5	AT2G29450	-2.26	-1.18	-4.78	0.00223	0.013651	-1.59
263356_at	putative RNA-binding protein ; supp	UBA1A	AT2G22090	-1.39	-0.48	-4.78	0.002233	0.013657	-1.59
255784_at	hypothetical protein predicted by ge	---	AT1G19860	1.44	0.52	4.78	0.002233	0.013657	-1.59
258062_at	unknown protein ; supported by cD	---	AT3G26000	1.69	0.76	4.78	0.002235	0.013662	-1.59
248689_at	SCARECROW gene regulator-like	PAT1	AT5G48150	1.58	0.66	4.78	0.002236	0.013668	-1.59
250188_at	putative protein COP9 complex sub	COP13	AT5G14250	1.45	0.54	4.78	0.002239	0.01368	-1.59
248789_at	putative protein strong similarity to u	---	AT5G47440	-1.75	-0.81	-4.78	0.00224	0.013683	-1.59
253468_at	putative protein predicted gene T10	---	AT4G32160	1.60	0.67	4.78	0.002241	0.013683	-1.59
250792_at	unknown protein ;supported by full-	---	AT5G05520	1.47	0.56	4.78	0.002244	0.013699	-1.59

247375_at	Expressed protein ; supported by fu	---	AT5G63135	1.34	0.42	4.78	0.002244	0.0137	-1.59
260949_at	F-box protein family contains simila	SKIP16	AT1G06110	1.47	0.56	4.78	0.002247	0.013712	-1.59
257215_at	RING zinc finger protein, putative c	---	AT3G15070	-1.46	-0.54	-4.78	0.002248	0.013714	-1.59
245330_at	hypothetical protein ;supported by f	---	AT4G14930	1.60	0.68	4.78	0.002252	0.013729	-1.60
261507_at	RIBOSOMAL PROTEIN, putative s	---	AT1G71720	-1.44	-0.53	-4.78	0.002253	0.013729	-1.60
264396_at	fumarylacetoacetate hydrolase-like	---	AT1G12050	1.40	0.48	4.78	0.002253	0.013729	-1.60
264583_at	putative AVR9 elicitor response pro	---	AT1G05170	1.62	0.70	4.77	0.002256	0.013746	-1.60
246469_at	putative protein predicted protein, [	---	AT5G17070	-1.43	-0.52	-4.77	0.00226	0.013765	-1.60
258700_at	putative SF16 protein similar to SF1	IQD1	AT3G09710	-1.45	-0.54	-4.77	0.002261	0.013765	-1.60
262029_at	50S ribosomal protein L21 chloropla	---	AT1G35680	-1.65	-0.72	-4.77	0.002264	0.013784	-1.60
262921_at	transfactor, putative similar to trans	APL	AT1G79430	1.46	0.55	4.77	0.002271	0.013812	-1.61
255568_at	putative DNA-binding protein ; sup	WRKY22	AT4G01250	-3.82	-1.93	-4.77	0.002271	0.013812	-1.61
259487_at	hypothetical protein contains similar	TRFL5	AT1G15720	-1.34	-0.43	-4.77	0.002275	0.013824	-1.61
264118_at	unknown protein	---	AT1G79150	2.40	1.26	4.77	0.002275	0.013824	-1.61
258964_at	putative 3-phosphoinositide-depend	---	AT3G10540	-1.47	-0.56	-4.77	0.002278	0.013837	-1.61
248477_at	putative protein similar to unknown	---	AT5G50900	-1.61	-0.69	-4.77	0.00228	0.013841	-1.61
267500_s_a	putative cytochrome P450	CYP704A1	AT2G44890	1.86	0.90	4.76	0.002281	0.013846	-1.61
256238_at	unknown protein ; supported by full-	ELC	AT3G12400	-1.65	-0.72	-4.76	0.002283	0.01385	-1.61
259603_at	Expressed protein ; supported by fu	---	AT1G56500	-1.80	-0.84	-4.76	0.002284	0.01385	-1.61
264551_at	unknown protein similar to beta-1,3	---	AT1G09460	-2.08	-1.05	-4.76	0.002286	0.013861	-1.61
265188_at	putative aldehyde dehydrogenase	ALDH2B7	AT1G23800	-1.66	-0.73	-4.76	0.002291	0.013885	-1.61
256274_at	GTPase, putative contains Pfam pr	emb2738	AT3G12080	1.41	0.50	4.76	0.002295	0.013907	-1.62
249441_at	avirulence induced gene (AIG) - lik	---	AT5G39730	-1.63	-0.70	-4.76	0.002298	0.01392	-1.62
248224_at	thylakoid lumenal 17.4 kD protein,	---	AT5G53490	-1.72	-0.79	-4.76	0.002299	0.01392	-1.62
261439_at	Expressed protein ; supported by fu	---	AT1G28395	2.09	1.06	4.76	0.0023	0.013922	-1.62
261857_at	hypothetical protein predicted by ge	---	AT1G50620	2.37	1.25	4.76	0.002304	0.013938	-1.62
255607_at	putative acetyltransferase similar to	---	AT4G01130	-1.93	-0.95	-4.76	0.002306	0.013942	-1.62
254980_at	putative ribosomal protein L9, cytos	---	AT4G10450	2.23	1.16	4.76	0.002306	0.013942	-1.62
249685_at	putative protein similar to unknown	CCB3	AT5G36120	-1.39	-0.47	-4.75	0.002314	0.013989	-1.63
263699_at	putative potassium transporter simil	KUP10	AT1G31120	1.77	0.83	4.75	0.002316	0.013989	-1.63
255695_at	putative protein	UNE11	AT4G00080	-1.40	-0.49	-4.75	0.002316	0.013989	-1.63
267089_at	unknown protein	---	AT2G38300	1.66	0.73	4.75	0.002316	0.013989	-1.63
250104_at	unknown protein	---	AT5G16610	1.71	0.77	4.75	0.002316	0.013989	-1.63
251110_at	putative protein various alpha amyla	---	AT5G01260	1.88	0.91	4.75	0.002317	0.013989	-1.63
246960_at	unknown protein	---	AT5G24710	-1.42	-0.50	-4.75	0.002319	0.013992	-1.63

249266_at	6-phosphogluconate dehydrogenas	---	AT5G41670	1.69	0.76	4.75	0.002325	0.014025	-1.63
256978_at	phosphoribosyamidoimidazole-suc	PUR7	AT3G21110	2.08	1.05	4.75	0.002327	0.014034	-1.63
255117_s_a	putative protein various hypothetica	ATPUP13	AT4G08700	1.35	0.44	4.75	0.002329	0.01404	-1.63
262198_at	pectin methylesterase (PMEU1), p	ATPME2	AT1G53830	-3.35	-1.75	-4.75	0.002331	0.01405	-1.63
247845_at	beta-1,3 glucanase - like protein be	---	AT5G58090	-1.44	-0.53	-4.74	0.002337	0.014076	-1.64
258316_at	unknown protein	---	AT3G22660	1.81	0.85	4.74	0.002337	0.014076	-1.64
251823_at	putative protein	---	AT3G55080	-1.37	-0.46	-4.74	0.002337	0.014076	-1.64
264308_at	hypothetical protein similar to hypot	---	AT1G61990	1.32	0.40	4.74	0.00234	0.014089	-1.64
254640_at	ion transporter - like protein integra	NRAMP5	AT4G18790	-1.46	-0.54	-4.74	0.002341	0.014094	-1.64
247706_at	putative ripening-related protein - lik	---	AT5G59480	-2.45	-1.29	-4.74	0.002343	0.014103	-1.64
253732_at	putative protein several hypothetica	---	AT4G29140	-1.46	-0.54	-4.74	0.002344	0.014106	-1.64
263054_at	putative cation transport protein	---	AT2G04620	1.45	0.53	4.74	0.002349	0.014125	-1.64
249900_at	unknown protein	emb1211	AT5G22640	1.43	0.52	4.74	0.00235	0.014127	-1.64
256696_at	unknown protein	---	AT3G20650	1.42	0.51	4.74	0.002353	0.014141	-1.64
248163_at	auxin-responsive-like protein ; supp	DFL1	AT5G54510	-6.05	-2.60	-4.74	0.002353	0.014141	-1.64
259380_at	putative ABC transporter similar to	PDR1	AT3G16340	-1.59	-0.67	-4.74	0.002355	0.01415	-1.65
260395_at	homeobox gene 13 protein identica	ATHB13	AT1G69780	-2.37	-1.25	-4.74	0.002356	0.01415	-1.65
250988_at	putative protein thyroid receptor int	UPL4	AT5G02880	1.38	0.46	4.74	0.002357	0.01415	-1.65
246423_at	putative protein ribosomal protein L	---	AT5G16950	1.64	0.71	4.73	0.002359	0.01416	-1.65
260780_at	valyl-tRNA synthetase, putative sin	TWN2	AT1G14610	1.38	0.46	4.73	0.002362	0.014172	-1.65
258995_at	putative ribosomal protein L13 simil	---	AT3G01790	1.74	0.80	4.73	0.002365	0.014187	-1.65
250525_at	putative protein contains similarity t	ILP1	AT5G08550	1.43	0.52	4.73	0.002366	0.01419	-1.65
265773_at	unknown protein ; supported by cD	RPH1	AT2G48070	-1.51	-0.59	-4.73	0.002371	0.014216	-1.65
245841_s_a	ribosomal protein S2, putative simi	XW6	AT1G58380	1.84	0.88	4.73	0.002371	0.014216	-1.65
257843_at	hypothetical protein predicted by g	---	---	-1.39	-0.47	-4.73	0.002374	0.014221	-1.65
251537_at	putative protein several hypothetica	---	AT3G58470	1.97	0.98	4.73	0.002376	0.014229	-1.65
261427_at	protein kinase, putative similar to C	IBS1	AT1G18670	-2.04	-1.03	-4.73	0.002376	0.014229	-1.65
252663_at	putative protein several hypothetica	---	AT3G44070	-1.35	-0.44	-4.73	0.00238	0.014248	-1.66
250075_at	putative protein	---	AT5G17670	-2.00	-1.00	-4.73	0.002381	0.014248	-1.66
260350_at	Eukaryotic initiation factor 5A , put	ELF5A-3	AT1G69410	1.72	0.79	4.73	0.002383	0.014255	-1.66
245413_at	asparagine--tRNA ligase	NS1	AT4G17300	-1.45	-0.53	-4.73	0.002384	0.014255	-1.66
251185_at	60S RIBOSOMAL PROTEIN L7A p	---	AT3G62870	1.65	0.72	4.72	0.002386	0.014267	-1.66
248357_at	putative protein similar to unknown	---	AT5G52380	2.12	1.08	4.72	0.002388	0.014272	-1.66
251831_at	pectate lyase -like protein pectate l	---	AT3G55140	-1.35	-0.43	-4.72	0.002388	0.014272	-1.66
250231_at	AP2/EREBP-like transcription facto	LEP	AT5G13910	-1.40	-0.49	-4.72	0.002391	0.014283	-1.66

253850_at	pectinesterase like protein pectines	sks10	AT4G28090	-1.61	-0.69	-4.72	0.002394	0.014301	-1.66
260418_s_a	hypothetical protein predicted by ge	COX19-1	AT1G66590	2.47	1.31	4.72	0.002396	0.014302	-1.66
257687_at	hypothetical protein predicted by ge	SDRB	AT3G12800	1.45	0.54	4.72	0.002396	0.014302	-1.66
259311_at	putative SAR DNA-binding protein-1	---	AT3G05060	2.08	1.06	4.72	0.002401	0.01433	-1.67
267055_at	unknown protein	PRA1.B4	AT2G38360	-2.73	-1.45	-4.72	0.002402	0.014332	-1.67
251526_at	putative protein several serine/threo	---	AT3G58640	-1.55	-0.63	-4.72	0.002405	0.014342	-1.67
264286_at	unknown protein ;supported by full-	PPR336	AT1G61870	1.94	0.96	4.72	0.002409	0.014361	-1.67
264164_at	Expressed protein ; supported by fu	---	AT1G65295	-1.58	-0.66	-4.72	0.00241	0.014361	-1.67
253988_at	putative protein elongation factor P	---	AT4G26310	1.41	0.50	4.72	0.00241	0.014361	-1.67
265390_at	unknown protein ; supported by ful	---	AT2G20820	1.41	0.50	4.72	0.002413	0.014368	-1.67
258534_at	thioredoxin, putative similar to thior	---	AT3G06730	1.42	0.50	4.71	0.002415	0.01438	-1.67
255403_at	putative GH3-like protein similar to	DFL2	AT4G03400	-1.62	-0.70	-4.71	0.00242	0.014403	-1.67
264561_at	uracil phosphoribosyltransferase, p	---	AT1G55810	1.72	0.79	4.71	0.002421	0.014404	-1.68
267484_at	E2, ubiquitin-conjugating enzyme 2	ATUBC2	AT2G02760	1.45	0.54	4.71	0.002421	0.014404	-1.68
253453_at	protein phosphatase 2C - like prote	---	AT4G31860	1.37	0.45	4.71	0.002422	0.014404	-1.68
253213_at	RNA helicase (RH16)	---	AT4G34910	1.46	0.54	4.71	0.002425	0.01442	-1.68
248239_at	ribosomal protein L11 methyltransfe	---	AT5G53920	1.77	0.83	4.71	0.002427	0.014427	-1.68
247408_at	ZAP - like protein nuclear protein Z	---	AT5G62760	-1.36	-0.44	-4.71	0.002428	0.014427	-1.68
266614_at	unknown protein	---	AT2G14910	1.51	0.60	4.71	0.002428	0.014427	-1.68
259514_at	hypothetical protein ; supported by	OZS1	AT1G12480	-1.48	-0.56	-4.71	0.002431	0.014441	-1.68
255735_at	transmembrane protein, putative si	---	AT1G25520	2.01	1.00	4.71	0.002432	0.014441	-1.68
265037_at	putative prohibitin 2 B-cell receptor	ATPHB2	AT1G03860	1.56	0.64	4.71	0.002433	0.014443	-1.68
250817_at	SET-domain protein-like ; supporte	SUVH1	AT5G04940	1.43	0.52	4.70	0.002442	0.01449	-1.68
265230_s_a	hypothetical protein	---	AT2G07707	1.69	0.76	4.70	0.002449	0.01453	-1.69
260273_at	unknown protein	---	AT1G80550	1.56	0.64	4.70	0.002453	0.014547	-1.69
260537_at	hypothetical protein predicted by ge	---	AT2G43450	-1.34	-0.43	-4.70	0.002453	0.014547	-1.69
249238_at	GAMM1 protein-like ; supported by	---	AT5G41970	1.45	0.53	4.70	0.002456	0.014558	-1.69
265318_at	putative D-amino acid dehydrogena	---	AT2G22650	1.70	0.77	4.70	0.002461	0.014582	-1.69
248081_at	putative protein similar to unknown	EDM2	AT5G55390	1.38	0.46	4.70	0.002464	0.014593	-1.69
246630_at	hypothetical protein predicted by ge	---	AT1G50730	-1.93	-0.95	-4.70	0.002466	0.014601	-1.69
246198_at	geranylgeranyl pyrophosphate synt	GGPS1	AT4G36810	-1.66	-0.73	-4.70	0.002467	0.014607	-1.70
254224_at	calcium-dependent protein kinase (	CDPK6	AT4G23650	-1.41	-0.49	-4.69	0.002473	0.014639	-1.70
261817_at	hypothetical protein predicted by ge	---	AT1G08180	2.27	1.19	4.69	0.002474	0.014641	-1.70
255227_at	D123 -like protein protein D123, Ra	EDA35	AT4G05440	1.63	0.70	4.69	0.002477	0.014654	-1.70
252276_at	hypothetical protein ; supported by	---	AT3G49490	1.53	0.61	4.69	0.002478	0.014657	-1.70

258970_at	putative serine carboxypeptidase pr	scpl49	AT3G10410	1.66	0.73	4.69	0.002485	0.014686	-1.70
256367_at	C-x8-C-x5-C-x3-H type Zinc finger	---	AT1G66810	-1.49	-0.57	-4.69	0.002487	0.014686	-1.70
249434_at	putative protein GTP-binding protei	---	AT5G39960	1.68	0.75	4.69	0.002487	0.014686	-1.70
252406_at	putative protein pectinesterase - Ar	---	AT3G47670	-1.35	-0.44	-4.69	0.002487	0.014686	-1.70
263031_at	glucosyltransferase, putative simila	ATCSLA10	AT1G24070	-5.62	-2.49	-4.69	0.002488	0.014686	-1.70
256677_at	unknown protein contains Pfam pro	PHF1	AT3G52190	1.53	0.61	4.69	0.002492	0.014704	-1.71
261745_at	unknown protein identical to unknow	---	AT1G08500	-2.61	-1.38	-4.69	0.002494	0.01471	-1.71
250354_at	putative protein predicted proteins,	---	AT5G11640	-1.45	-0.53	-4.69	0.002496	0.01471	-1.71
266330_at	unknown protein ; supported by cD	MLP329	AT2G01530	1.80	0.85	4.69	0.002496	0.01471	-1.71
262154_at	unknown protein contains similarity	---	AT1G52700	-1.80	-0.85	-4.69	0.002496	0.01471	-1.71
263065_at	MAP kinase (ATMPK7) identical to	ATMPK7	AT2G18170	1.89	0.92	4.69	0.002496	0.01471	-1.71
253761_at	putative protein thyroid receptor int	---	AT4G28820	1.54	0.63	4.68	0.002497	0.01471	-1.71
262641_at	unknown protein ;supported by full-	---	AT1G62730	1.32	0.40	4.68	0.002497	0.01471	-1.71
253922_at	putative protein ; supported by cDN	VTC2	AT4G26850	1.79	0.84	4.68	0.0025	0.014726	-1.71
246733_at	serine protease-like protein serine	---	AT5G27660	1.51	0.59	4.68	0.002503	0.014735	-1.71
258138_at	Expressed protein GC donor splice	MSH7	AT3G24495	1.38	0.46	4.68	0.002504	0.014738	-1.71
258712_s_a	putative 40S ribosomal protein S23	---	AT3G09680	1.60	0.68	4.68	0.002505	0.014743	-1.71
247792_at	Expressed protein ; supported by fu	---	AT5G58787	1.89	0.92	4.68	0.00251	0.014768	-1.71
266158_at	putative ABC transporter	---	AT2G28070	1.69	0.75	4.68	0.002512	0.014769	-1.72
253186_at	putative protein predicted proteins,	---	AT4G35270	1.71	0.78	4.68	0.002515	0.014787	-1.72
248232_at	putative protein contains similarity t	MLO11	AT5G53760	-1.60	-0.68	-4.68	0.002521	0.014819	-1.72
262295_at	putative U2 snRNP auxiliary factor	ATU2AF35	AT1G27650	1.61	0.69	4.68	0.002522	0.014822	-1.72
264122_at	GTP-binding protein, ara-5 ras hon	ARA-5	AT1G02130	1.32	0.40	4.68	0.002524	0.014828	-1.72
245368_at	hypothetical protein ; supported by	---	AT4G15510	-1.86	-0.90	-4.67	0.002525	0.014831	-1.72
250905_at	protein kinase -like protein protein	---	AT5G03640	1.37	0.45	4.67	0.002526	0.014831	-1.72
249510_at	putative protein similar to unknown	---	AT5G38510	1.48	0.57	4.67	0.002529	0.014842	-1.72
266911_at	putative protein kinase contains a p	---	AT2G45910	1.65	0.72	4.67	0.00253	0.014842	-1.72
256771_at	unknown protein	---	AT3G13700	-1.39	-0.47	-4.67	0.002531	0.014843	-1.72
254991_at	putative protein conserved hypothe	---	AT4G10620	1.57	0.65	4.67	0.002534	0.01486	-1.72
253804_at	putative protein	---	AT4G28230	1.52	0.61	4.67	0.002538	0.014876	-1.73
246951_at	unknown protein	---	AT5G04880	2.19	1.13	4.67	0.002538	0.014876	-1.73
254655_s_a	ribosomal protein L32 -like protein	---	AT4G18100	1.87	0.90	4.67	0.002539	0.014876	-1.73
256607_at	unknown protein ; supported by cDN	---	AT3G32930	-1.39	-0.47	-4.67	0.00254	0.014876	-1.73
265442_at	hypothetical protein predicted by ge	---	AT2G20940	2.06	1.04	4.67	0.002543	0.01489	-1.73
261565_at	beta-1,3-glucanase, putative simila	---	AT1G33220	-1.34	-0.42	-4.67	0.002543	0.01489	-1.73

262777_at	hypothetical protein predicted by ge	---	AT1G13030	1.80	0.85	4.67	0.002544	0.014892	-1.73
267299_at	putative glucosyltransferase	---	AT2G30150	1.62	0.69	4.67	0.002548	0.014908	-1.73
255578_at	predicted protein of unknown funct	---	AT4G01450	2.29	1.20	4.67	0.002552	0.014925	-1.73
249144_at	squamosa promoter binding protein	SPL2	AT5G43270	-1.48	-0.56	-4.66	0.002554	0.014934	-1.73
264048_at	unknown protein	---	AT2G22400	1.72	0.78	4.66	0.002555	0.014939	-1.73
246879_at	endopeptidase - like protein O-sialo	---	AT5G26110	1.46	0.54	4.66	0.002558	0.014949	-1.74
255240_at	coded for by A. thaliana cDNA Z180	IBR1	AT4G05530	1.43	0.52	4.66	0.00256	0.014954	-1.74
252286_at	putative protein	---	AT3G49070	-1.32	-0.40	-4.66	0.002561	0.014958	-1.74
245117_at	putative Ca2+-ATPase ; supported	ACA4	AT2G41560	1.71	0.77	4.66	0.002568	0.014991	-1.74
258963_at	putative myotubularin similar to myo	---	AT3G10550	-1.50	-0.59	-4.66	0.00257	0.014994	-1.74
250384_at	putative protein various predicted p	---	AT5G11500	1.42	0.50	4.66	0.002579	0.015044	-1.74
254573_at	putative pectinacetylesterase pecti	---	AT4G19420	2.16	1.11	4.66	0.002581	0.015047	-1.74
261607_at	unknown protein similar to putative	AtCXE5	AT1G49660	2.64	1.40	4.66	0.002582	0.01505	-1.75
259323_at	unknown protein	---	AT3G05280	-1.32	-0.40	-4.65	0.002588	0.015076	-1.75
266456_at	putative bHLH transcription factor	NAI1	AT2G22770	-1.50	-0.58	-4.65	0.002588	0.015077	-1.75
246965_at	methyltransferase-like protein meth	---	AT5G24840	1.81	0.86	4.65	0.002594	0.015105	-1.75
251881_at	DIPHOSPHOMEVALONATE DECA	---	AT3G54250	-2.07	-1.05	-4.65	0.002597	0.015114	-1.75
266057_at	putative methionyl-tRNA synthetase	---	AT2G40660	1.39	0.47	4.65	0.002598	0.015119	-1.75
251134_at	hypothetical protein RNA polymera	AtQSOX2	AT2G01270	1.46	0.55	4.65	0.0026	0.015126	-1.75
257990_at	putative myc-like DNA-binding prote	---	AT3G19860	-1.66	-0.73	-4.65	0.002606	0.015154	-1.76
260627_at	hypothetical protein predicted by ge	---	AT1G62310	-1.78	-0.83	-4.65	0.002607	0.015156	-1.76
251741_at	putative protein predicted protein, A	UGP3	AT3G56040	2.17	1.12	4.65	0.002608	0.015163	-1.76
266746_s_a	putative glutathione S-transferase	ATGSTF3	AT2G02930	5.04	2.33	4.65	0.002612	0.01518	-1.76
257940_at	putative UDP-glucose glucosyltrans	---	AT3G21790	1.58	0.66	4.64	0.002613	0.015182	-1.76
247241_at	unknown protein	---	AT5G64680	1.98	0.99	4.64	0.002622	0.015228	-1.76
248196_at	putative protein similar to unknown	---	AT5G54150	-1.39	-0.48	-4.64	0.002623	0.015229	-1.76
266703_at	unknown protein predicted by gens	---	AT2G19880	-1.77	-0.82	-4.64	0.002626	0.015242	-1.76
253013_at	heat shock protein 70 like protein c	mtHsc70-1	AT4G37910	2.05	1.04	4.64	0.002627	0.015242	-1.76
260843_at	unknown protein similar to hypothet	---	AT1G29060	1.33	0.41	4.64	0.002628	0.015248	-1.76
259789_at	Expressed protein ; supported by fu	COR414-TI	AT1G29395	-1.51	-0.60	-4.64	0.002639	0.015303	-1.77
253815_at	putative beta-expansin/allergen pro	ATEXPB3	AT4G28250	-1.76	-0.82	-4.64	0.002641	0.015307	-1.77
257956_at	unknown protein	---	AT3G25400	1.62	0.69	4.63	0.002647	0.015335	-1.77
247705_at	putative protein ;supported by full-l	---	AT5G59460	1.44	0.52	4.63	0.002649	0.015342	-1.77
245957_at	putative protein predicted proteins -	---	AT5G19590	1.36	0.44	4.63	0.002653	0.015358	-1.77
262220_at	calcium-dependent protein kinase,	CPK30	AT1G74740	-1.39	-0.47	-4.63	0.002653	0.015358	-1.77

251538_at	putative protein PBK1 protein, Hom	---	AT3G58660	1.85	0.89	4.63	0.002656	0.015374	-1.78
258166_at	WD-repeat protein, putative contai	---	AT3G21540	2.12	1.09	4.63	0.002664	0.015413	-1.78
267533_at	unknown protein ;supported by full-	ATNUDX23	AT2G42070	1.48	0.57	4.63	0.002666	0.015425	-1.78
261606_at	peroxidase, putative similar to pero	---	AT1G49570	-1.60	-0.68	-4.63	0.00267	0.015438	-1.78
250453_at	putative protein predicted protein, E	---	AT5G10620	-1.60	-0.68	-4.63	0.002671	0.015438	-1.78
246074_at	hypothetical protein ; supported by	---	AT5G20130	1.40	0.49	4.63	0.002671	0.015438	-1.78
248242_at	aldo/keto reductase-like protein	---	AT5G53580	1.61	0.68	4.62	0.002674	0.015452	-1.78
261238_at	hypothetical protein predicted by ge	---	AT1G32810	1.46	0.55	4.62	0.002676	0.015462	-1.78
255495_at	predicted protein of unknown funct	---	AT4G02720	1.45	0.53	4.62	0.002678	0.015466	-1.78
257857_s_a	unknown protein contains an RNase	RRP45a	AT3G12990	1.76	0.82	4.62	0.002679	0.015467	-1.79
245565_at	hypothetical protein	---	AT4G14605	1.48	0.57	4.62	0.00268	0.015467	-1.79
260879_at	hypothetical protein predicted by ge	---	AT1G21480	-1.47	-0.55	-4.62	0.00268	0.015467	-1.79
259677_at	putative phosphatidylinositol-4-phos	---	AT1G77740	-1.63	-0.70	-4.62	0.00268	0.015467	-1.79
255226_at	UV-damaged DNA binding factor -	DDB1A	AT4G05420	1.39	0.47	4.62	0.002685	0.015486	-1.79
257002_at	oligouridylate binding protein, putat	---	AT3G14100	1.59	0.67	4.62	0.002685	0.015486	-1.79
259605_at	unknown protein	PUB45	AT1G27910	-1.95	-0.96	-4.62	0.002686	0.015486	-1.79
266064_at	expressed protein	---	AT2G18780	1.62	0.69	4.62	0.002686	0.015486	-1.79
261313_at	hypothetical protein predicted by ge	---	AT1G52970	-1.35	-0.44	-4.62	0.002688	0.015487	-1.79
265352_at	cytosolic cyclophilin (ROC3) ;suppo	ROC3	AT2G16600	1.48	0.57	4.62	0.002693	0.01551	-1.79
262342_at	unknown protein similar to hypothet	---	AT1G64150	-1.89	-0.92	-4.62	0.002693	0.01551	-1.79
264855_at	3-hydroxy-3-methylglutaryl-coenzy	HSK	AT2G17265	1.40	0.48	4.62	0.002695	0.015518	-1.79
257876_at	hypothetical protein predicted by ge	---	AT3G17130	-2.00	-1.00	-4.62	0.002696	0.015518	-1.79
255225_at	U3 snoRNP-associated -like protei	---	AT4G05410	2.33	1.22	4.62	0.002698	0.015527	-1.79
247197_at	receptor-like protein kinase	---	AT5G65240	2.32	1.22	4.62	0.002702	0.015541	-1.79
262594_at	putative 60s ribosomal protein L37	---	AT1G15250	2.75	1.46	4.61	0.002705	0.015544	-1.80
259292_at	unknown protein	---	AT3G11560	1.47	0.56	4.61	0.002705	0.015544	-1.80
254054_at	putative protein DNA-binding prote	---	AT4G25320	1.70	0.76	4.61	0.002706	0.015544	-1.80
249838_at	unknown protein ;supported by full-	---	AT5G23460	1.64	0.72	4.61	0.002706	0.015544	-1.80
247791_at	cyclophilin ROC7 ;supported by ful	ROC7	AT5G58710	1.65	0.72	4.61	0.002706	0.015544	-1.80
255018_at	hypothetical protein ; supported by	---	AT4G10090	1.62	0.69	4.61	0.002707	0.015544	-1.80
257652_at	unknown protein contains Pfam pro	APUM24	AT3G16810	2.02	1.02	4.61	0.002707	0.015544	-1.80
253825_at	Expressed protein ; supported by fu	---	AT4G28025	-1.68	-0.75	-4.61	0.00271	0.015557	-1.80
263384_at	hypothetical protein predicted by ge	---	AT2G40130	-1.51	-0.60	-4.61	0.002711	0.015558	-1.80
260645_at	hypothetical protein predicted by ge	TTL1	AT1G53300	-1.93	-0.95	-4.61	0.002712	0.015558	-1.80
255261_s_a	putative protein equilibrative nucle	FUR1	AT4G05110	-2.81	-1.49	-4.61	0.002713	0.015558	-1.80

257761_at	unknown protein	---	AT3G23090	-2.01	-1.01	-4.61	0.002713	0.015558	-1.80
254078_at	putative protein various predicted p	---	AT4G25710	1.50	0.58	4.61	0.002716	0.015565	-1.80
255224_at	putative protein hypothetical protei	---	AT4G05400	1.58	0.66	4.61	0.002717	0.015565	-1.80
245266_at	hypothetical protein ; supported by	---	AT4G17070	1.89	0.92	4.61	0.002718	0.015568	-1.80
245059_at	unknown protein ;supported by full-	RHC2A	AT2G39720	-1.38	-0.46	-4.61	0.002718	0.015568	-1.80
260984_at	Expressed protein ; supported by fu	---	AT1G53645	2.01	1.01	4.61	0.00272	0.015571	-1.80
251357_at	ribosomal protein S27 ; supported	ARS27A	AT3G61110	1.95	0.97	4.61	0.002727	0.015606	-1.80
265726_at	putative inositol polyphosphate 5'-p	---	AT2G32010	1.69	0.76	4.61	0.002727	0.015606	-1.80
266036_s_a	20S proteasome subunit (PAA2) ide	PAA2	AT2G05840	1.43	0.51	4.61	0.002735	0.015641	-1.81
260112_at	unknown protein similar to unknown	---	AT1G63310	-1.61	-0.69	-4.60	0.002736	0.015643	-1.81
255249_at	3(2),5-BISPHOSPHATE NUCLEOT	---	AT4G05090	-1.83	-0.87	-4.60	0.00274	0.015665	-1.81
258996_at	putative ribosome recycling factor s	---	AT3G01800	1.61	0.69	4.60	0.002741	0.015665	-1.81
251784_at	putative protein photosystem II oxy	PPL1	AT3G55330	-1.84	-0.88	-4.60	0.002742	0.015667	-1.81
246775_at	putative protein GTP-binding protei	MIRO1	AT5G27540	1.36	0.44	4.60	0.002743	0.015667	-1.81
260786_s_a	unknown protein similar to Cryptosp	MEE5	AT1G06220	1.56	0.64	4.60	0.002746	0.015682	-1.81
248975_at	unknown protein ; supported by cD	---	AT5G45040	-1.46	-0.54	-4.60	0.002747	0.015682	-1.81
264731_at	hypothetical protein predicted by ge	---	AT1G62150	1.48	0.56	4.60	0.002751	0.015702	-1.81
258046_at	MAP kinase kinase 5 identical to GI	MKK5	AT3G21220	1.45	0.54	4.60	0.002763	0.015758	-1.82
259176_at	putative cell division control protein	CDC48C	AT3G01610	1.47	0.55	4.60	0.002764	0.015762	-1.82
245164_at	mitochondrial chaperonin (HSP60)	HSP60-2	AT2G33210	2.44	1.29	4.59	0.002785	0.015879	-1.83
245373_s_a	Expressed protein ; supported by fu	ATARD2	AT4G14710	1.45	0.54	4.59	0.00279	0.015899	-1.83
245999_at	putative protein predicted protein, A	COPT5	AT5G20650	1.38	0.47	4.59	0.002794	0.015918	-1.83
264064_at	hypothetical protein predicted by ge	---	AT2G27900	-1.42	-0.50	-4.59	0.002795	0.015918	-1.83
267093_at	high affinity Ca2+ antiporter identic	CAX1	AT2G38170	1.83	0.87	4.59	0.002798	0.015931	-1.83
245543_at	glucosyltransferase	---	AT4G15260	1.53	0.61	4.58	0.002799	0.015931	-1.83
266662_at	hypothetical protein predicted by ge	---	AT2G25830	-1.62	-0.70	-4.58	0.002799	0.015931	-1.83
260465_at	membrane-associated salt-inducible	---	AT1G10910	1.32	0.40	4.58	0.002811	0.015995	-1.84
266568_at	unknown protein	---	AT2G24070	-1.37	-0.46	-4.58	0.002817	0.016026	-1.84
266776_at	putative ubiquitin fusion-degradation	---	AT2G29070	1.55	0.63	4.58	0.002822	0.016047	-1.84
254023_at	putative protein hypothetical protei	---	AT4G25770	-1.53	-0.62	-4.58	0.002827	0.016065	-1.84
253507_at	serine threonine protein kinase like	---	AT4G32000	-1.74	-0.80	-4.58	0.002827	0.016065	-1.84
259461_at	unknown protein	---	AT1G18900	1.81	0.86	4.58	0.002829	0.016074	-1.84
264670_s_a	unknown protein Similar to elongat	---	AT1G09640	1.56	0.64	4.58	0.00283	0.016074	-1.84
262395_at	hypothetical protein similar to hypot	---	AT1G49540	2.03	1.02	4.57	0.002833	0.01608	-1.85
262310_at	unknown protein contains similarity	MLP31	AT1G70840	-1.45	-0.54	-4.57	0.002835	0.016088	-1.85

252929_at	putative fructose-bisphosphate ald	---	AT4G38970	-1.43	-0.51	-4.57	0.002835	0.016089	-1.85
255593_at	hypothetical protein	---	AT4G01650	1.44	0.53	4.57	0.002837	0.016092	-1.85
245100_at	ubiquitin-specific protease 5 (UBP5	UBP5	AT2G40930	1.42	0.51	4.57	0.002841	0.016109	-1.85
253547_at	chloroplast omega-6 fatty acid desa	FAD6	AT4G30950	-1.42	-0.51	-4.57	0.002841	0.016109	-1.85
257775_at	short-chain alcohol dehydrogenase	---	AT3G29260	-1.48	-0.57	-4.57	0.002845	0.016117	-1.85
253072_at	putative protein ;supported by full-l	---	AT4G37830	1.36	0.45	4.57	0.002845	0.016117	-1.85
250133_at	thioredoxin f2 (gb|AAD35004.1) ; s	TRXF2	AT5G16400	-1.95	-0.96	-4.57	0.002846	0.016117	-1.85
261753_at	unknown protein	---	AT1G76340	-1.52	-0.61	-4.57	0.002846	0.016117	-1.85
256110_at	Ser Thr protein kinase, putative sin	---	AT1G16900	1.69	0.75	4.57	0.002847	0.016117	-1.85
246258_at	PPR-repeat protein, putative conta	---	AT1G31840	1.40	0.49	4.57	0.002847	0.016117	-1.85
259824_at	copper homeostasis factor, putative	ATX1	AT1G66240	1.40	0.48	4.57	0.002847	0.016117	-1.85
258243_at	carbamoyl phosphate synthetase s	CARA	AT3G27740	1.73	0.79	4.57	0.002861	0.016186	-1.86
253849_at	putative protein 150-kD protein, Di	---	AT4G28080	-1.40	-0.49	-4.57	0.002862	0.016186	-1.86
252943_at	cinnamyl-alcohol dehydrogenase C	CAD9	AT4G39330	-12.83	-3.68	-4.56	0.002868	0.016212	-1.86
255677_at	putative calmodulin-binding heat sh	---	AT4G00500	1.75	0.81	4.56	0.00287	0.016212	-1.86
259682_at	transcription factor DREB1A, putat	---	AT1G63040	-1.67	-0.74	-4.56	0.00287	0.016212	-1.86
257060_at	unknown protein	---	AT3G18230	1.47	0.56	4.56	0.002872	0.016216	-1.86
254761_at	Expressed protein ; supported by fu	CLE44	AT4G13195	1.70	0.76	4.56	0.002872	0.016216	-1.86
257009_at	unknown protein ; supported by cD	---	AT3G14160	1.38	0.46	4.56	0.002874	0.016217	-1.86
259837_at	aquaporin, putative similar to delta	---	AT1G52180	-1.78	-0.83	-4.56	0.002874	0.016217	-1.86
265254_at	unknown protein ; supported by cD	DRB2	AT2G28380	-1.45	-0.54	-4.56	0.002878	0.016238	-1.86
248409_at	putative protein contains similarity t	LPA2	AT5G51540 /	-1.56	-0.64	-4.56	0.00288	0.016246	-1.86
248744_at	putative protein contains similarity t	---	AT5G48250	1.66	0.73	4.56	0.002883	0.016256	-1.87
250833_at	myotubularin - like protein myotubu	---	AT5G04540	1.41	0.50	4.56	0.002883	0.016256	-1.87
247410_at	putative protein similar to unknown	emb1692	AT5G62990	1.99	0.99	4.56	0.002887	0.016264	-1.87
256314_at	hypothetical protein	TOC75-I	AT1G35860	-1.45	-0.54	-4.56	0.002888	0.016264	-1.87
247608_at	replication protein A1 - like probabl	---	AT5G60990	1.56	0.64	4.56	0.002888	0.016264	-1.87
261634_at	ATP-dependent Clp protease prote	CLPR1	AT1G49970	1.61	0.69	4.56	0.002889	0.016264	-1.87
258904_at	putative ATPase (ISW2-like) simila	CHR11	AT3G06400	1.48	0.57	4.56	0.002889	0.016264	-1.87
259067_at	F-box protein family, AtFBL12 cont	---	AT3G07550	-1.38	-0.46	-4.56	0.00289	0.016267	-1.87
267550_at	putative protein kinase contains a p	AP4.3A	AT2G32800	-2.40	-1.26	-4.56	0.002892	0.016272	-1.87
246782_at	putative protein PrMC3 - Pinus rad	GID1C	AT5G27320	2.08	1.05	4.56	0.002893	0.016272	-1.87
258924_at	putative diadenosine 5',5'''-P1,P4-t	ATNUDX26	AT3G10620	-1.39	-0.48	-4.55	0.002903	0.016325	-1.87
246767_at	putative protein CG6949 - Drosoph	---	AT5G27395	2.55	1.35	4.55	0.002905	0.016325	-1.87
256078_at	hypothetical protein predicted by ge	---	AT1G20670	-1.45	-0.54	-4.55	0.002906	0.016325	-1.87

258041_at	Expressed protein ; supported by fu	ZML1	AT3G21175	1.46	0.55	4.55	0.002906	0.016325	-1.87
249748_at	thaumatin-like protein	---	AT5G24620	-1.48	-0.57	-4.55	0.002909	0.016325	-1.87
258226_at	putative phospholipase D similar to	PLDALPHA	AT3G15730	-1.45	-0.53	-4.55	0.002909	0.016325	-1.87
246565_at	biotin carboxyl carrier protein precu	BCCP2	AT5G15530	-1.46	-0.54	-4.55	0.00291	0.016325	-1.88
266671_at	anthranilate synthase, alpha subun	ASA2	AT2G29690	-1.49	-0.57	-4.55	0.00291	0.016325	-1.88
259754_at	hypothetical protein predicted by ge	---	AT1G71090	1.48	0.57	4.55	0.002911	0.016328	-1.88
267646_at	putative phosphate transporter	PHT5	AT2G32830	1.76	0.82	4.55	0.002916	0.016349	-1.88
255756_at	endo-beta-1,4-D-glucanase, putativ	AtGH9B5	AT1G19940	-1.47	-0.55	-4.55	0.002917	0.016349	-1.88
258974_at	unknown protein similar to brahma	---	AT3G01890	1.48	0.57	4.55	0.002925	0.016388	-1.88
246339_at	RP19 gene for chloroplast ribosoma	RPL9	AT3G44890	-1.48	-0.56	-4.54	0.002931	0.016414	-1.88
254262_at	putative protein annexin VII, Dictyo	---	AT4G23470	-1.57	-0.66	-4.54	0.002932	0.016414	-1.88
250234_at	transaldolase - like protein transald	---	AT5G13420	1.69	0.76	4.54	0.002932	0.016414	-1.88
259159_at	unknown protein contains Pfam pro	ACBP4	AT3G05420	-1.64	-0.71	-4.54	0.002934	0.01642	-1.88
259162_at	unknown protein similar to unknown	---	AT3G01640	-1.55	-0.63	-4.54	0.002943	0.016451	-1.89
264690_at	hypothetical protein similar to hypot	SNL4	AT1G70060	-1.58	-0.66	-4.54	0.002943	0.016451	-1.89
254945_at	putative RING finger protein ring fir	---	AT4G10940	-1.55	-0.64	-4.54	0.002943	0.016451	-1.89
267095_at	putative AMP deaminase ; support	FAC1	AT2G38280	1.51	0.60	4.54	0.002945	0.016455	-1.89
255494_at	predicted protein of unknown funct	---	AT4G02710	1.39	0.48	4.54	0.002951	0.016482	-1.89
251576_at	putative protein several hypothetica	---	AT3G58200	1.40	0.48	4.54	0.002951	0.016482	-1.89
266453_at	putative protein kinase contains a p	---	AT2G43230	-1.35	-0.43	-4.54	0.002956	0.016507	-1.89
260731_at	P-type ATPase, putative similar to	---	AT1G17500	-1.52	-0.60	-4.54	0.002958	0.016508	-1.89
255413_at	putative alcohol dehydrogenase sim	---	AT4G03140	-1.49	-0.58	-4.54	0.002959	0.016508	-1.89
254952_at	UDP-galactose 4-epimerase - like p	UGE5	AT4G10955 /	1.52	0.61	4.54	0.002959	0.016508	-1.89
246294_at	ribosomal protein, chloroplast ribos	PSRP5	AT3G56910	-1.40	-0.48	-4.54	0.002959	0.016508	-1.89
258317_at	hypothetical protein predicted by ge	---	AT3G22670	1.40	0.49	4.54	0.002964	0.016528	-1.90
248228_at	unknown protein ;supported by full-	---	AT5G53800	1.63	0.70	4.53	0.002969	0.016552	-1.90
252223_at	MYB -like protein DNA-binding prot	TRB3	AT3G49850	1.72	0.78	4.53	0.002971	0.01656	-1.90
252118_at	putative protein various predicted p	---	AT3G51400	1.32	0.40	4.53	0.002974	0.016568	-1.90
258563_at	putative long-chain-fatty-acid--CoA	LACS6	AT3G05970	1.47	0.56	4.53	0.002978	0.016568	-1.90
248743_at	unknown protein	---	AT5G48240	1.81	0.85	4.53	0.002978	0.016568	-1.90
244985_at	ribosomal protein L22	---	---	-1.76	-0.81	-4.53	0.002978	0.016568	-1.90
258717_at	unknown protein ; supported by cD	SYP71	AT3G09740	-1.33	-0.41	-4.53	0.002978	0.016568	-1.90
257324_at	hypothetical protein	---	---	2.77	1.47	4.53	0.00298	0.016568	-1.90
248014_at	putative protein similar to unknown	---	AT5G56340	-1.60	-0.68	-4.53	0.002981	0.016568	-1.90
260585_at	unknown protein	EMB2777	AT2G43650	2.14	1.10	4.53	0.002982	0.016568	-1.90

264270_at	beta-glucosidase, putative similar t	---	---	-1.37	-0.45	-4.53	0.002984	0.016572	-1.90
253657_at	cadmium-transporting ATPase-like	HMA2	AT4G30110	2.57	1.36	4.53	0.002984	0.016572	-1.90
255886_at	plastocyanin, putative similar to pla	DRT112	AT1G20340	-1.39	-0.48	-4.53	0.002985	0.016573	-1.90
254867_at	hypothetical proteins	---	AT4G12240	1.68	0.75	4.53	0.002992	0.016606	-1.91
267530_at	putative receptor-like protein kinase	---	AT2G41890	2.32	1.21	4.53	0.002994	0.016613	-1.91
253589_at	puative protein membrane-associa	---	AT4G30825	1.67	0.74	4.53	0.002996	0.01662	-1.91
250235_s_a	ubiquinol--cytochrome-c reductase	---	AT5G13430	1.36	0.44	4.53	0.002998	0.016625	-1.91
260641_at	hypothetical protein predicted by ge	---	AT1G53200	1.98	0.99	4.52	0.003	0.016636	-1.91
263171_at	unknown protein	CCX4	AT1G54115	1.46	0.54	4.52	0.003006	0.016658	-1.91
254132_at	putative protein hypothetical protei	ATHB22	AT4G24660	1.77	0.82	4.52	0.003006	0.016658	-1.91
258217_at	unknown protein contains Pfam pro	XPL1	AT3G17998	-1.88	-0.91	-4.52	0.003007	0.016658	-1.91
265899_s_a	SKP1/ASK1 (At3), putative similar	ASK4	AT1G20140	1.48	0.56	4.52	0.003008	0.016659	-1.91
250321_at	zinc finger transcription factor -like	---	AT5G12850	-1.46	-0.55	-4.52	0.003012	0.016681	-1.91
252927_at	cysteine proteinase RD19A identic	RD19	AT4G39090	1.37	0.46	4.52	0.003014	0.016685	-1.91
264367_at	unknown protein ESTs gb|T21276,	---	AT1G03350	-1.40	-0.49	-4.52	0.003015	0.016687	-1.91
251710_at	putative protein several hypothetica	---	AT3G56930	-2.02	-1.01	-4.52	0.003016	0.016687	-1.91
261037_at	lipoxygenase identical to GB:CAB5	LOX3	AT1G17420	-5.47	-2.45	-4.52	0.003017	0.016687	-1.91
248303_at	FtsH protease, putative similar to A	FTSH11	AT5G53170	1.45	0.54	4.52	0.003017	0.016687	-1.91
259536_s_a	putative protein hypothetical protein	ATCOAB	AT1G12350	1.33	0.41	4.52	0.003018	0.016687	-1.91
265586_at	pathogenesis-related protein (PR-1	PR-1-LIKE	AT2G19990	-1.56	-0.64	-4.52	0.00302	0.016689	-1.92
256405_at	calcium-dependent protein kinase s	CPK33	AT1G50700	1.38	0.47	4.52	0.00302	0.016689	-1.92
253362_s_a	putative protein cyclopropane-fatty	---	AT4G33110	-1.66	-0.73	-4.52	0.00302	0.016689	-1.92
260148_at	putative oxidoreductase similar to g	---	AT1G52800	1.74	0.80	4.52	0.003021	0.016691	-1.92
246908_at	dehydration-induced protein RD22	RD22	AT5G25610	-4.93	-2.30	-4.52	0.003022	0.016693	-1.92
260575_at	hypothetical protein predicted by ge	---	AT2G47300	1.45	0.54	4.52	0.003028	0.016718	-1.92
256106_at	unknown protein	---	AT1G16870	1.40	0.48	4.52	0.003028	0.016718	-1.92
259277_at	putative glycogen synthase similar t	AtSS2	AT3G01180	-1.74	-0.80	-4.52	0.003031	0.016725	-1.92
246155_at	putative protein predicted proteins,	---	AT5G20030	-1.34	-0.42	-4.52	0.003032	0.01673	-1.92
253977_at	putative protein various Caenorhab	---	AT4G26630	1.73	0.79	4.52	0.003034	0.016738	-1.92
254819_at	pEARLI 1-like protein Arabidopsis t	---	AT4G12500	11.09	3.47	4.51	0.003036	0.016743	-1.92
260862_at	unknown protein	SEU	AT1G43850	1.42	0.50	4.51	0.00304	0.016756	-1.92
260632_at	homeobox protein, putative similar	STM	AT1G62360	-1.45	-0.54	-4.51	0.00304	0.016756	-1.92
263605_at	hypothetical protein predicted by ge	---	AT2G16485	1.56	0.64	4.51	0.003042	0.016763	-1.92
252504_at	telomere repeat-binding protein ho	TRFL1	AT3G46590	1.41	0.50	4.51	0.003044	0.016773	-1.92
258480_at	unknown protein ;supported by full-	---	AT3G02640	-1.47	-0.56	-4.51	0.003047	0.01678	-1.93

264952_at	Tub family protein, putative contair	AtTLP1	AT1G76900	-1.36	-0.45	-4.51	0.003047	0.01678	-1.93
266239_at	unknown protein ; supported by cD	---	AT2G29530	2.19	1.13	4.51	0.00305	0.016794	-1.93
257964_at	hypothetical protein similar to hypot	---	AT3G19850	-1.79	-0.84	-4.51	0.003053	0.016807	-1.93
249356_at	putative protein predicted proteins,	---	AT5G40520	1.84	0.88	4.51	0.003056	0.016809	-1.93
245925_at	bZIP transcription factor family prot	BZO2H3	AT5G28770	-1.68	-0.75	-4.51	0.003056	0.016809	-1.93
255021_at	hypothetical protein	---	AT4G10330	1.44	0.52	4.51	0.003056	0.016809	-1.93
250564_at	putative protein sigma factor F inhi	---	AT5G08060	1.34	0.42	4.51	0.003057	0.016809	-1.93
266701_at	profilin 1 ; supported by cDNA: gi_	PRF1	AT2G19760	-1.34	-0.42	-4.51	0.003057	0.016809	-1.93
249574_at	putative protein embryonic abunda	PDLP7	AT5G37660	-1.45	-0.54	-4.51	0.00306	0.016818	-1.93
246122_at	putative protein brain specific Na+-	PHT4;5	AT5G20380	1.51	0.59	4.51	0.003061	0.016818	-1.93
249828_at	succinyl-CoA synthetase, alpha su	---	AT5G23250	1.56	0.64	4.51	0.003061	0.016818	-1.93
260916_at	predicted protein ; supported by cD	---	AT1G02475	1.46	0.55	4.51	0.003067	0.016847	-1.93
260266_at	putative B-box zinc finger protein co	---	AT1G68520	-1.65	-0.72	-4.51	0.003068	0.016847	-1.93
264018_at	putative triosephosphate isomerase	TIM	AT2G21170	-1.42	-0.51	-4.51	0.00307	0.016852	-1.93
248886_at	phosphate/triose-phosphate translo	APE2	AT5G46110	-1.46	-0.55	-4.50	0.003071	0.016854	-1.93
263526_at	hypothetical protein predicted by ge	---	AT2G24830	1.39	0.47	4.50	0.003072	0.016854	-1.93
261561_at	unknown protein ;supported by full-	---	AT1G01730	1.69	0.76	4.50	0.003077	0.016878	-1.94
260616_at	hypothetical protein contains Pfam	---	AT1G53280	1.63	0.71	4.50	0.003082	0.016901	-1.94
255520_at	putative ribosomal protein L19 simil	---	AT4G02230	1.86	0.90	4.50	0.003085	0.016916	-1.94
249248_at	unknown protein	---	AT5G42320	-1.51	-0.60	-4.50	0.003087	0.016924	-1.94
257610_at	zinc finger protein, putative similar	AtIDD11	AT3G13810	2.33	1.22	4.50	0.003089	0.016929	-1.94
250156_at	putative protein predicted proteins,	---	AT5G15170	1.36	0.44	4.50	0.00309	0.016931	-1.94
253726_at	ribosomal protein S15a homolog ri	rps15ae	AT4G29430	2.18	1.12	4.50	0.003091	0.016931	-1.94
248833_at	Bax inhibitor-1 like ;supported by fu	ATBI1	AT5G47120	1.55	0.63	4.50	0.003093	0.016939	-1.94
254326_at	putative protein AIR1, Arabidopsis	---	AT4G22610	-1.46	-0.55	-4.49	0.003112	0.017033	-1.95
254765_at	hypothetical protein	---	AT4G13270	1.49	0.57	4.49	0.003114	0.017038	-1.95
263696_at	putative aspartate kinase-homoseri	AK-HSDH I	AT1G31230	1.79	0.84	4.49	0.00312	0.017061	-1.95
252527_at	dTDP-glucose 4-6-dehydratases-lil	UXS5	AT3G46440	1.53	0.62	4.49	0.00312	0.017061	-1.95
246211_at	G-box-binding factor 1 ; supported I	GBF1	AT4G36730	-2.00	-1.00	-4.49	0.003123	0.017069	-1.95
259348_at	hypothetical protein may contain C	---	AT3G03770	-1.82	-0.86	-4.49	0.003129	0.017099	-1.95
261553_at	hypothetical protein	---	AT1G63420	-1.50	-0.59	-4.49	0.003131	0.017108	-1.95
255979_at	hypothetical protein	UPF3	AT1G33980	1.43	0.52	4.49	0.003134	0.017119	-1.96
256510_at	ATP-dependent Clp protease ATP-	---	AT1G33360	1.48	0.57	4.49	0.003137	0.017126	-1.96
263120_at	cytochrome P450, putative similar t	CYP708A3	AT1G78490	-7.43	-2.89	-4.49	0.003138	0.017128	-1.96
251092_at	putative protein ;supported by full-l	---	AT5G01470	-1.44	-0.53	-4.49	0.00314	0.017136	-1.96

264864_at	hypothetical protein predicted by ge	---	AT1G24310	1.80	0.85	4.48	0.003144	0.017149	-1.96
264693_at	hypothetical protein predicted by ge	CLE26	AT1G69970	-1.42	-0.50	-4.48	0.003144	0.017149	-1.96
266093_at	unknown protein ;supported by full-	---	AT2G37990	1.72	0.78	4.48	0.003149	0.017172	-1.96
260145_at	putative G protein-coupled receptor	GPCR	AT1G52920	1.33	0.41	4.48	0.003158	0.017215	-1.96
262005_at	ABC transporter protein, putative s	ATGCN3	AT1G64550	1.65	0.72	4.48	0.003159	0.017219	-1.96
262487_at	unknown protein ESTs gb|T42839 a	---	AT1G21610	1.30	0.38	4.48	0.003163	0.017232	-1.97
251841_at	putative protein CRP1, Zea mays,	---	AT3G54980	1.81	0.85	4.48	0.003163	0.017232	-1.97
259919_at	putative exportin, tRNA (nuclear ex	PSD	AT1G72560	1.63	0.70	4.48	0.003164	0.017232	-1.97
263600_at	hypothetical protein predicted by ge	DRD1	AT2G16390	-1.48	-0.57	-4.48	0.003168	0.017245	-1.97
259718_at	unknown protein	VIP5	AT1G61040	1.34	0.42	4.48	0.003168	0.017245	-1.97
257086_at	hypothetical protein predicted by ge	---	AT3G20490	1.70	0.77	4.48	0.003169	0.017245	-1.97
246817_at	putative protein various predicted p	---	AT5G27240	1.49	0.57	4.48	0.00317	0.017249	-1.97
259849_at	phosphoglycerate dehydrogenase,	---	AT1G72190	1.34	0.42	4.48	0.003173	0.017258	-1.97
264214_s_a	MADS-box transcription factor, put	AGL38	AT1G65300	-1.38	-0.47	-4.47	0.003179	0.017284	-1.97
245626_at	unknown protein ;supported by full-	---	AT1G56700	1.67	0.74	4.47	0.003179	0.017284	-1.97
253383_at	putative protein ;supported by full-l	---	AT4G32900	1.69	0.76	4.47	0.003181	0.017286	-1.97
245593_at	IAA7 like protein	IAA14	AT4G14550	-2.51	-1.33	-4.47	0.003182	0.017286	-1.97
258099_at	unknown protein similar to hypothet	---	AT3G23540	1.41	0.50	4.47	0.003183	0.017286	-1.97
258160_at	glutamine synthetase, putative sim	GLN1.3	AT3G17820	1.89	0.92	4.47	0.003187	0.017296	-1.97
247176_at	acyl-CoA oxidase (gb|AAC13497.1)	ACX2	AT5G65110	1.76	0.81	4.47	0.003187	0.017296	-1.97
253248_at	Putative S-phase-specific ribosoma	---	AT4G34670	2.47	1.30	4.47	0.003187	0.017296	-1.97
260590_at	phosphoenolpyruvate carboxylase	ATPPC1	AT1G53310	1.59	0.66	4.47	0.003195	0.017329	-1.98
253020_at	hypothetical protein	---	AT4G38020	1.61	0.68	4.47	0.003198	0.017335	-1.98
266574_at	hypothetical protein predicted by ge	---	AT2G23890	2.09	1.06	4.47	0.003198	0.017335	-1.98
250390_at	separation anxiety protein - like se	---	AT5G11340	1.32	0.40	4.47	0.003198	0.017335	-1.98
248491_at	unknown protein ;supported by full-	---	AT5G51010	1.67	0.74	4.47	0.003199	0.017335	-1.98
252849_at	Histon H3 ;supported by full-length	---	AT4G40040	1.41	0.50	4.47	0.0032	0.017335	-1.98
250350_at	putative protein predicted proteins,	---	AT5G12010	-2.09	-1.06	-4.47	0.003202	0.017345	-1.98
265284_at	unknown protein ;supported by full-	---	AT2G20230	1.48	0.56	4.47	0.003204	0.017345	-1.98
265384_at	unknown protein ;supported by full-	---	AT2G20760	-1.33	-0.41	-4.47	0.003204	0.017345	-1.98
246263_at	unknown protein	---	AT1G31780	-1.58	-0.66	-4.47	0.003205	0.017345	-1.98
264678_at	putative U2 small nuclear ribonucle	U2A'	AT1G09760	1.44	0.53	4.47	0.003206	0.017345	-1.98
254851_at	putative disease resistance protein	---	AT4G12010	1.92	0.94	4.47	0.003206	0.017345	-1.98
260480_at	Ser/Thr kinase ;supported by full-le	SNRK2.4	AT1G10940	1.32	0.40	4.47	0.003208	0.017349	-1.98
263243_at	putative glyoxalase II	GLX2-5	AT2G31350	1.50	0.59	4.47	0.003209	0.017349	-1.98

261207_at	peroxisomal targeting signal type 2	---	AT1G12830	1.74	0.80	4.47	0.003209	0.017349	-1.98
250571_at	hypothetical protein	---	AT5G08200	-1.66	-0.73	-4.47	0.003216	0.017381	-1.98
252186_at	putative protein	---	AT3G50810	1.52	0.60	4.46	0.003217	0.017386	-1.98
251249_at	putative protein 10-deacetylbaccati	---	AT3G62160	-1.45	-0.54	-4.46	0.00322	0.017393	-1.99
263386_at	unknown protein ; supported by cD	---	AT2G40150	-2.08	-1.06	-4.46	0.003222	0.017401	-1.99
258026_at	abscisic acid responsive elements-I	ABF4	AT3G19290	1.61	0.69	4.46	0.003224	0.017402	-1.99
251310_at	homeobox protein Anthocyaninless	HDG1	AT3G61150	-1.59	-0.67	-4.46	0.003225	0.017402	-1.99
252335_at	putative protein putative protein - A	---	AT3G48860	1.49	0.57	4.46	0.003225	0.017402	-1.99
259533_at	hypothetical protein	---	AT1G12530	1.33	0.41	4.46	0.003227	0.017402	-1.99
250625_at	calnexin homolog	---	AT5G07340	1.67	0.74	4.46	0.003227	0.017402	-1.99
264065_at	hypothetical protein predicted by ge	---	AT2G27900	-1.47	-0.55	-4.46	0.003227	0.017402	-1.99
254559_at	putative protein glycine/proline-rich	---	AT4G19200	-1.70	-0.76	-4.46	0.003232	0.017414	-1.99
251656_at	putative protein N-ACETYLGLUCO	---	AT3G57170	-1.44	-0.53	-4.46	0.003232	0.017414	-1.99
246244_at	receptor kinase-like protein	---	AT4G37250	-1.40	-0.48	-4.46	0.003232	0.017414	-1.99
258502_at	unknown protein	---	AT3G02490	1.86	0.89	4.46	0.003234	0.017416	-1.99
247607_at	putative protein various predicted p	---	AT5G60960	1.95	0.97	4.46	0.003235	0.01742	-1.99
245806_at	light-harvesting complex protein sim	LHCA5	AT1G45474	-1.85	-0.88	-4.46	0.003237	0.017426	-1.99
265119_at	flavin-containing monooxygenase,	FMO GS-O	AT1G62570	-2.16	-1.11	-4.46	0.003238	0.017426	-1.99
256288_at	arginine N-methyltransferase 3, pu	PRMT3	AT3G12270	2.23	1.16	4.46	0.00324	0.017435	-1.99
267442_at	unknown protein ;supported by full-	---	AT2G19080	1.37	0.45	4.46	0.003242	0.017437	-1.99
258297_at	Expressed protein ; supported by fu	---	AT3G23325	1.46	0.55	4.46	0.003243	0.017437	-1.99
258945_at	calmodulin-domain protein kinase C	CPK2	AT3G10660	-1.34	-0.42	-4.46	0.003243	0.017437	-1.99
256858_at	hypothetical protein predicted by ge	---	AT3G15140	1.69	0.76	4.46	0.003244	0.017437	-1.99
261709_at	RNA-binding protein, putative simil	CID11	AT1G32790	1.40	0.49	4.46	0.003247	0.017445	-1.99
265301_s_a	putative MYB family transcription fa	---	AT2G13960 ,	1.45	0.53	4.46	0.003249	0.017451	-2.00
247271_at	nuclear protein-like	---	AT5G64270	1.50	0.59	4.46	0.00325	0.017452	-2.00
253648_at	pathogenesis related homeodomain	PRHA	AT4G29940	1.35	0.43	4.45	0.003257	0.017486	-2.00
257456_at	unknown protein predicted by gens	SRS4	AT2G18120	-1.45	-0.53	-4.45	0.00326	0.017497	-2.00
258174_at	hypothetical protein contains Pfam	---	AT3G21470	1.45	0.54	4.45	0.003262	0.017497	-2.00
250966_at	putative protein various predicted p	---	AT5G02910	1.54	0.62	4.45	0.003262	0.017497	-2.00
259723_at	putative iron-regulated transporter s	IRT3	AT1G60960	2.20	1.14	4.45	0.00327	0.017536	-2.00
266520_at	cyclic nucleotide and calmodulin-reg	ATCNGC6	AT2G23980	1.51	0.59	4.45	0.003273	0.017543	-2.00
247601_at	zinc finger protein OBP4 - like zinc	OBP4	AT5G60850	-1.71	-0.78	-4.45	0.003276	0.017556	-2.00
263975_at	hypothetical protein predicted by ge	---	AT2G42710	1.47	0.56	4.45	0.003279	0.017571	-2.01
260331_at	hypothetical protein contains Pfam	---	AT1G80270	4.57	2.19	4.44	0.003294	0.017644	-2.01

252055_at	putative ribosomal protein S14 ribo	---	AT3G52580	1.56	0.64	4.44	0.003295	0.017644	-2.01
259648_at	hypothetical protein predicted by ge	---	---	-1.34	-0.42	-4.44	0.003298	0.017647	-2.01
261348_at	hypothetical protein contains similar	TED3	AT1G79810	1.37	0.46	4.44	0.003298	0.017647	-2.01
265032_at	ribosomal protein identical to riboso	RPL3B	AT1G61580	2.20	1.14	4.44	0.003298	0.017647	-2.01
250144_at	putative protein various predicted p	---	AT5G14680	1.86	0.90	4.44	0.003301	0.017659	-2.01
252566_at	cytoplasmic ribosomal protein S15a	RPS15AD	AT3G46040	1.59	0.67	4.44	0.003305	0.017672	-2.01
258375_at	hypothetical protein predicted by ge	---	AT3G17470	-1.38	-0.46	-4.44	0.003313	0.017707	-2.02
246601_at	copper amine oxidase, putative sim	---	AT1G31710	-11.77	-3.56	-4.44	0.003313	0.017707	-2.02
245560_at	indole-3-acetate beta-glucosyltrans	UGT84A1	AT4G15480	-1.52	-0.60	-4.44	0.003316	0.017712	-2.02
253901_at	ribosomal protein L14 -like protein	---	AT4G27090	1.80	0.84	4.44	0.003316	0.017712	-2.02
245256_at	Expressed protein ; supported by cI	FAR1	AT4G15090	1.50	0.59	4.44	0.003317	0.017712	-2.02
261898_at	hypothetical protein predicted by ge	---	AT1G80720	2.08	1.06	4.44	0.003317	0.017712	-2.02
256986_at	unknown protein	---	AT3G28700	1.65	0.72	4.44	0.003319	0.017718	-2.02
259711_at	putative DNA-binding protein simila	---	AT1G77570	2.27	1.18	4.44	0.003322	0.017726	-2.02
248265_at	putative protein contains similarity t	SDG29	AT5G53430	1.63	0.71	4.44	0.003322	0.017726	-2.02
246549_at	putative protein predicted proteins,	---	AT5G15050	-2.00	-1.00	-4.44	0.003327	0.017742	-2.02
262986_at	unknown protein similar to hypothet	---	AT1G23390	-1.99	-0.99	-4.44	0.003331	0.017757	-2.02
257047_at	hypothetical protein predicted by ge	---	AT3G19570	-1.66	-0.73	-4.43	0.003333	0.017757	-2.02
254546_at	lectin like protein lectin 2, phloem-s	PP2-A2	AT4G19850	2.72	1.44	4.43	0.003333	0.017757	-2.02
246904_at	DNA methyltransferase PMT1 - like	DNMT2	AT5G25480	1.51	0.59	4.43	0.003342	0.017801	-2.03
246591_at	putative cation transport protein pu	---	AT5G14880	1.53	0.62	4.43	0.003343	0.017801	-2.03
260073_at	putative protein kinase contains Pfa	---	AT1G73660	1.59	0.67	4.43	0.003354	0.017851	-2.03
254615_at	RNase L inhibitor-like protein RNas	RLI2	AT4G19210	1.39	0.48	4.43	0.003357	0.017863	-2.03
251652_at	putative protein hypothetical protei	---	AT3G57380	1.75	0.81	4.43	0.003358	0.017865	-2.03
260868_at	unknown protein ;supported by full-	---	AT1G43860	1.58	0.66	4.43	0.003364	0.017889	-2.03
257755_at	unknown protein ; supported by ful	---	AT3G18760	1.85	0.89	4.43	0.003367	0.0179	-2.03
262654_at	putative mandelonitrile lyase simila	---	AT1G14180	-1.59	-0.67	-4.43	0.003368	0.017901	-2.03
265676_at	putative CCR4-associated factor ; s	---	AT2G32070	-1.38	-0.46	-4.42	0.003373	0.017922	-2.04
262284_at	hypothetical protein predicted by ge	---	AT1G68670	1.71	0.78	4.42	0.00338	0.017955	-2.04
260961_at	unknown protein similar to hypothet	---	AT1G05960	-1.33	-0.42	-4.42	0.003382	0.017963	-2.04
249525_at	putative protein similar to unknown	---	AT5G38650	1.38	0.47	4.42	0.003384	0.017966	-2.04
267186_at	unknown protein ; supported by cD	---	AT2G44090	-1.41	-0.50	-4.42	0.003387	0.017977	-2.04
258029_at	serine/threonine-protein kinase, PK	ATPK7	AT3G27580	1.36	0.45	4.42	0.00339	0.01799	-2.04
261049_at	hypothetical protein similar to hypot	---	AT1G01430	-1.74	-0.80	-4.42	0.003392	0.017991	-2.04
258011_at	putative homeobox protein, HAT3.1	HAT3.1	AT3G19510	1.59	0.67	4.42	0.003392	0.017992	-2.04

266860_at	putative phospholipase C	---	AT2G26870	-1.98	-0.98	-4.42	0.003397	0.018004	-2.04
264799_at	putative violaxanthin de-epoxidase	NPQ1	AT1G08550	-1.53	-0.61	-4.42	0.0034	0.018016	-2.04
250673_at	serine threonine protein kinase-like	CIPK2	AT5G07070	-1.62	-0.70	-4.42	0.003402	0.018016	-2.04
266183_at	unknown protein	ATPT2	AT2G38940	1.57	0.65	4.42	0.003402	0.018016	-2.04
247349_at	putative protein similar to unknown	---	AT5G63820	-1.38	-0.47	-4.41	0.003411	0.018058	-2.05
245770_at	hypothetical protein predicted by ge	---	AT1G30240	1.83	0.87	4.41	0.003413	0.018061	-2.05
252998_at	probable H+-transporting ATPase I	---	AT4G38510	-1.48	-0.56	-4.41	0.003417	0.018081	-2.05
253947_at	putative protein hypothetical protei	MAP65-2	AT4G26760	1.50	0.58	4.41	0.003418	0.018081	-2.05
256534_at	unknown protein	---	AT1G33270	1.41	0.49	4.41	0.003422	0.018099	-2.05
249230_at	putative protein similar to unknown	---	AT5G42070	-1.55	-0.63	-4.41	0.003426	0.018115	-2.05
251437_at	putative protein hypothetical protei	---	AT3G59910	1.36	0.44	4.41	0.00343	0.018134	-2.05
262324_at	hypothetical protein similar to hypot	ATCHX16	AT1G64170	6.04	2.60	4.41	0.003432	0.01814	-2.05
254323_at	putative protein auxin-induced prot	---	AT4G22620	-2.27	-1.19	-4.41	0.003435	0.018149	-2.06
263638_at	unknown protein	---	AT2G25310	1.33	0.41	4.41	0.003438	0.018164	-2.06
266304_at	hypothetical protein	---	AT2G27090	1.39	0.48	4.41	0.003439	0.018164	-2.06
249040_at	eukaryotic translation initiation facto	---	AT5G44320	1.44	0.52	4.41	0.003441	0.018169	-2.06
250049_at	putative protein predicted proteins,	---	AT5G17780	-1.45	-0.54	-4.41	0.003442	0.018169	-2.06
250836_at	putative protein various predicted p	DME	AT5G04560	-1.33	-0.42	-4.41	0.003443	0.018172	-2.06
253190_at	UDP-galactose transporter - like pr	---	AT4G35335	-1.44	-0.53	-4.41	0.00345	0.018202	-2.06
256413_at	hypothetical protein predicted by ge	---	AT3G11100	1.45	0.53	4.40	0.003456	0.01823	-2.06
262969_at	unknown protein	---	AT1G75710	-1.62	-0.70	-4.40	0.003466	0.018269	-2.07
254438_at	translation initiation factor eIF3 - lik	---	AT4G20980	1.55	0.64	4.40	0.003467	0.018269	-2.07
259402_at	developmental protein, putative sin	VPS46.1	AT1G17730	-1.38	-0.47	-4.40	0.003467	0.018269	-2.07
251062_at	putative protein putative proteins - A	OFP1	AT5G01840	-1.44	-0.53	-4.40	0.00347	0.018281	-2.07
250748_at	AtPH1-like protein ;supported by fu	---	AT5G05710	1.50	0.59	4.40	0.003482	0.018337	-2.07
248998_at	unknown protein	---	AT5G45320	-1.37	-0.45	-4.40	0.003487	0.01836	-2.07
247830_at	putative protein predicted proteins,	---	AT5G58530	-1.74	-0.80	-4.40	0.003488	0.018361	-2.07
256486_at	chloroplast nucleoid DNA binding p	---	AT1G31450	-1.43	-0.52	-4.39	0.003493	0.01838	-2.07
267361_at	hypothetical protein predicted by ge	---	AT2G39920	1.81	0.86	4.39	0.003496	0.018389	-2.07
257750_at	unknown protein ;supported by full-	---	AT3G18800	-1.51	-0.59	-4.39	0.003499	0.018402	-2.08
253609_at	H+-transporting ATPase type 2, pla	AHA2	AT4G30190	1.51	0.60	4.39	0.003503	0.018419	-2.08
257352_at	putative RING3 protein	IMB1	AT2G34900	1.52	0.61	4.39	0.003504	0.018419	-2.08
250723_at	lysine decarboxylase-like protein	---	AT5G06300	-1.63	-0.70	-4.39	0.003505	0.018419	-2.08
254415_at	subtilisin-like serine protease conta	ATSBT3.12	AT4G21326	-1.34	-0.42	-4.39	0.003506	0.018422	-2.08
264722_at	unknown protein location of EST en	---	AT1G22970	1.59	0.67	4.39	0.003507	0.018422	-2.08

258529_at	hypothetical protein predicted by g	---	AT3G06740	-1.62	-0.70	-4.39	0.003511	0.018439	-2.08
250741_at	putative protein contains similarity t	---	AT5G05790	2.03	1.02	4.39	0.003515	0.018449	-2.08
248674_at	non-phototropic hypocotyl-like prote	---	AT5G48800	1.96	0.97	4.39	0.003515	0.018449	-2.08
258707_at	putative histone H2B similar to histo	---	AT3G09480	1.57	0.65	4.39	0.003522	0.018479	-2.08
254444_at	putative protein riboflavin biosynthe	---	AT4G20960	-1.89	-0.92	-4.39	0.003524	0.018487	-2.08
249237_at	putative protein similar to unknown	---	AT5G42050	1.72	0.78	4.39	0.003527	0.018492	-2.08
266370_at	hypothetical protein predicted by ge	---	AT2G41350	-1.33	-0.41	-4.39	0.003532	0.01851	-2.09
266087_at	putative alcohol dehydrogenase	---	AT2G37790	-1.53	-0.62	-4.38	0.003535	0.018523	-2.09
249386_at	disease resistance -like protein dov	---	AT5G40060	1.42	0.50	4.38	0.003536	0.018523	-2.09
251762_at	sedoheptulose-bisphosphatase pre	SBPASE	AT3G55800	-1.44	-0.53	-4.38	0.00354	0.018535	-2.09
257743_at	unknown protein	---	AT3G27390	1.64	0.71	4.38	0.003541	0.018535	-2.09
258432_at	unknown protein ;supported by full-	RALFL23	AT3G16570	-1.81	-0.85	-4.38	0.003541	0.018535	-2.09
263046_at	unknown protein ; supported by cD	GRP3S	AT2G05380	2.01	1.01	4.38	0.003541	0.018535	-2.09
252065_at	putative protein ;supported by full-l	---	AT3G51610	1.53	0.61	4.38	0.003546	0.018554	-2.09
257401_at	hypothetical protein identical to hyp	SRO2	AT1G23550	1.57	0.65	4.38	0.003552	0.018577	-2.09
246442_at	hypothetical protein ;supported by	---	AT5G17610	1.36	0.44	4.38	0.003554	0.018582	-2.09
256023_at	hypothetical protein predicted by ge	ZW2	AT1G58330	-1.51	-0.59	-4.38	0.003557	0.018599	-2.09
253183_at	putative protein predicted protein, A	---	AT4G35220	1.35	0.43	4.38	0.00356	0.018606	-2.09
251679_at	putative protein strictosidine syntha	---	AT3G57030	-1.95	-0.96	-4.38	0.003564	0.018624	-2.10
247555_at	putative protein various predicted p	ECT3	AT5G61020	1.81	0.86	4.38	0.003566	0.01863	-2.10
255537_at	protoporphyrinogen oxidase ; supp	PPOX	AT4G01690	-1.55	-0.63	-4.38	0.003567	0.018631	-2.10
266699_at	putative ribosomal protein L28 ; su	---	AT2G19730	1.76	0.82	4.38	0.003568	0.018631	-2.10
245229_at	putative protein contains EST gb:T4	---	AT4G25620	-1.90	-0.92	-4.37	0.003576	0.018667	-2.10
247454_at	unknown protein	---	AT5G62440	1.97	0.98	4.37	0.003579	0.018671	-2.10
254822_at	polyubiquitin-like protein polyubiqu	UPL5	AT4G12570	1.38	0.47	4.37	0.003581	0.018678	-2.10
247698_at	putative protein predicted proteins,	---	AT5G59830	1.47	0.56	4.37	0.003582	0.018679	-2.10
252359_at	putative protein predicted proteins,	---	AT3G48440	-1.59	-0.67	-4.37	0.00359	0.018715	-2.10
254508_at	putative protein gene F4P9.34 chro	---	AT4G20170	-1.52	-0.60	-4.37	0.00359	0.018715	-2.10
267375_at	G protein alpha subunit 1 (GPA1) ic	GP ALPHA	AT2G26300	1.49	0.58	4.37	0.003593	0.018719	-2.10
249398_at	RING finger -like protein RING-H2	---	AT5G40250	1.47	0.56	4.37	0.003593	0.018719	-2.10
262188_at	Avr9 elicitor response protein, puta	---	AT1G77810	1.49	0.58	4.37	0.003594	0.018719	-2.10
245395_at	hypothetical protein ;supported by f	---	AT4G17420	-1.52	-0.61	-4.37	0.003597	0.018733	-2.11
257611_at	unknown protein	---	AT3G26580	1.60	0.68	4.37	0.003598	0.018734	-2.11
252408_at	putative transcription factor MYB94	ATMYB94	AT3G47600	-1.34	-0.42	-4.37	0.003614	0.018812	-2.11
261178_at	synaptobrevin 7B, putative similar t	ATVAMP72	AT1G04760	-1.35	-0.44	-4.36	0.003618	0.018829	-2.11

249331_at	50S ribosomal protein L27 ;support	RPL27	AT5G40950	-1.43	-0.52	-4.36	0.003621	0.018837	-2.11
261148_at	unknown protein ; supported by full-	GAMMA C/	AT1G19580	-1.35	-0.44	-4.36	0.003627	0.018853	-2.11
259121_at	unknown protein	---	AT3G02220	1.57	0.65	4.36	0.003627	0.018853	-2.11
252469_at	protein kinase - like protein Raf pro	---	AT3G46920	1.59	0.67	4.36	0.003627	0.018853	-2.11
262533_at	hypothetical protein Pfam	---	AT1G17090	1.45	0.54	4.36	0.003628	0.018853	-2.11
260533_at	putative U1 small nuclear ribonucle	---	AT2G43370	1.37	0.45	4.36	0.003639	0.018902	-2.12
257280_at	9-cis-epoxycarotenoid dioxygenase	NCED3	AT3G14440	-1.81	-0.85	-4.36	0.003641	0.01891	-2.12
256444_at	hypothetical protein predicted by ge	---	AT3G11060	-1.35	-0.44	-4.36	0.003645	0.018924	-2.12
249718_at	putative protein contains similarity t	---	AT5G35740	-1.52	-0.60	-4.36	0.003655	0.018974	-2.12
250842_at	putative protein predicted proteins,	VTE5	AT5G04490	-1.61	-0.69	-4.36	0.003658	0.018982	-2.12
245673_at	hypothetical protein similar to putati	---	AT1G56690	1.49	0.57	4.36	0.003658	0.018982	-2.12
260593_at	poly(A)-specific ribonuclease, puta	AHG2	AT1G55870	1.57	0.65	4.36	0.00366	0.018985	-2.12
251444_at	putative protein ETHIONINE AMIN	MAP2B	AT3G59990	1.36	0.44	4.35	0.00367	0.019028	-2.13
248631_at	putative protein strong similarity to u	---	AT5G49000	1.52	0.60	4.35	0.003672	0.019037	-2.13
255292_s_a	putative calcium dependent protein	CPK22	AT4G04710	1.56	0.64	4.35	0.003675	0.019047	-2.13
262428_at	hypothetical protein predicted by ge	---	AT1G47570	1.40	0.48	4.35	0.003676	0.019047	-2.13
265569_at	unknown protein ;supported by full-	PGR5	AT2G05620	1.42	0.51	4.35	0.00368	0.019064	-2.13
258776_at	putative T-complex protein 1, ETA	---	AT3G11830	1.49	0.58	4.35	0.003683	0.019076	-2.13
261351_at	hypothetical protein predicted by ge	---	AT1G79790	-1.47	-0.55	-4.35	0.003687	0.019087	-2.13
262455_at	Mlo protein, putative similar to Mlo	MLO2	AT1G11310	1.63	0.71	4.35	0.003694	0.019116	-2.13
247504_at	putative protein various predicted p	---	AT5G61990	1.65	0.72	4.35	0.003694	0.019116	-2.13
253798_at	predicted protein CUC2 (involved i	ANAC073	AT4G28500	-1.45	-0.54	-4.35	0.003695	0.019116	-2.13
248853_at	protein kinase-like protein	BSK2	AT5G46570	-1.55	-0.63	-4.35	0.003698	0.019127	-2.14
257315_at	proline oxidase, mitochondrial pred	ERD5	AT3G30775	-3.60	-1.85	-4.35	0.0037	0.019131	-2.14
260295_at	putative aminopeptidase similar to a	---	AT1G63770	1.51	0.60	4.35	0.0037	0.019131	-2.14
262098_at	transcription factor, putative similar	NF-YC2	AT1G56170	1.58	0.66	4.34	0.003704	0.019147	-2.14
265985_at	hypothetical protein predicted by ge	ATPUP5	AT2G24220	-1.36	-0.44	-4.34	0.003713	0.019184	-2.14
257073_at	hypothetical protein predicted by ge	---	AT3G19650	1.55	0.64	4.34	0.003717	0.019202	-2.14
255953_at	transcription factor identical to trans	TGA3	AT1G22070	1.60	0.67	4.34	0.00372	0.01921	-2.14
262134_at	sulfate transporter, putative similar	AST56	AT1G77990	1.96	0.97	4.34	0.003722	0.019211	-2.14
266898_at	unknown protein ; supported by cDI	---	AT2G45990	-1.51	-0.60	-4.34	0.003724	0.019219	-2.14
264450_s_a	unknown protein similar to hypothet	SNL6	AT1G10250	1.75	0.81	4.34	0.003726	0.019223	-2.14
253391_at	hypothetical protein ; supported by	---	AT4G32590	-1.69	-0.76	-4.34	0.00373	0.019242	-2.14
254878_at	heat shock transcription factor - like	AT-HSFB2	AT4G11660	-1.82	-0.87	-4.34	0.003735	0.019258	-2.15
255770_at	hypothetical protein predicted by ge	---	AT1G18560	-1.41	-0.50	-4.34	0.003738	0.019268	-2.15

251446_at	Expressed protein ; supported by fu	---	AT3G59840	1.35	0.43	4.34	0.00374	0.019268	-2.15
262712_at	mercaptopyruvate sulfurtransferase	ATRDH2	AT1G16460	1.49	0.58	4.34	0.00374	0.019268	-2.15
265580_at	putative protein phosphatase 2C	---	AT2G20050	-1.53	-0.61	-4.33	0.003749	0.019304	-2.15
256309_at	photosystem I subunit X precursor i	PSAK	AT1G30380	-1.37	-0.45	-4.33	0.003762	0.019366	-2.15
257188_at	hypothetical protein contains Pfam	---	AT3G13150	1.47	0.56	4.33	0.003764	0.019374	-2.15
248749_at	eukaryotic release factor 1 homolog	ERF1-1	AT5G47880	1.35	0.43	4.33	0.003779	0.019443	-2.16
247415_at	unknown protein	---	AT5G63060	-1.96	-0.97	-4.33	0.003791	0.019502	-2.16
249528_at	putative protein predicted protein, [	---	AT5G38720	1.75	0.81	4.32	0.003794	0.019515	-2.16
250027_at	putative protein similar to unknown	---	AT5G18250	1.55	0.64	4.32	0.003796	0.01952	-2.16
254675_at	RNA helicase - like protein Cdc28p	---	AT4G18465	1.71	0.78	4.32	0.0038	0.019538	-2.17
262375_at	unknown protein ; supported by cDI	SUVH3	AT1G73100	1.50	0.58	4.32	0.003802	0.019539	-2.17
257235_at	ras-related GTP-binding protein cor	AtRABA1g	AT3G15060	-3.42	-1.77	-4.32	0.003805	0.019547	-2.17
257658_at	unknown protein ; supported by ful	---	AT3G13230	2.33	1.22	4.32	0.003806	0.019551	-2.17
258219_at	thioredoxin, putative similar to THI(	ATTDX	AT3G17880	1.39	0.48	4.32	0.00381	0.019558	-2.17
258433_at	translationally controlled tumor prot	TCTP	AT3G16640	1.50	0.59	4.32	0.00381	0.019558	-2.17
266474_at	hypothetical protein predicted by gr	---	AT2G31110	2.86	1.51	4.32	0.003815	0.019575	-2.17
246756_at	protein phosphatase - like protein p	---	AT5G27930	-1.55	-0.64	-4.32	0.00382	0.019595	-2.17
249827_at	unknown protein ;supported by full-	---	AT5G23330	1.51	0.59	4.32	0.00382	0.019595	-2.17
259937_s_a	putative ABC transporter contains F	ATNAP5	AT1G71330 ,	1.43	0.51	4.32	0.003823	0.019603	-2.17
248080_at	wax synthase-like protein	---	AT5G55380	-1.55	-0.64	-4.32	0.003828	0.019623	-2.17
254162_at	transcription factor IIA small subuni	---	AT4G24440	1.60	0.68	4.32	0.003832	0.019639	-2.17
266181_at	putative glutathione S-transferase	ATGSTZ1	AT2G02390	1.52	0.61	4.32	0.003834	0.019644	-2.17
260010_at	putative trehalose-6-phosphate syn	ATTPS6	AT1G68020	-1.74	-0.80	-4.31	0.003841	0.019675	-2.18
255508_at	putative zinc finger protein identical	---	AT4G02220	1.48	0.57	4.31	0.003843	0.019679	-2.18
245581_at	hypothetical protein	---	AT4G14840	-1.49	-0.58	-4.31	0.003845	0.019689	-2.18
254495_at	hypothetical protein	EMB1895	AT4G20060	1.69	0.76	4.31	0.003859	0.019754	-2.18
253172_at	putative protein ;supported by full-le	---	AT4G35060	-2.60	-1.38	-4.31	0.00386	0.019755	-2.18
245020_at	cytochrome f	---	---	-1.41	-0.50	-4.31	0.003863	0.019767	-2.18
264448_at	putative sensory transduction histid	AHK3	AT1G27320	1.34	0.42	4.31	0.003872	0.0198	-2.19
247429_at	putative protein strong similarity to u	---	AT5G62620	1.60	0.68	4.31	0.003873	0.0198	-2.19
255816_at	unknown protein ;supported by full-	GLTP1	AT2G33470	1.33	0.42	4.31	0.003877	0.019815	-2.19
245993_at	putative protein predicted proteins,	---	AT5G20700	1.75	0.81	4.30	0.00389	0.019879	-2.19
265682_at	similar to avrRpt2-induced protein 2	---	AT2G24390	1.60	0.68	4.30	0.003891	0.019879	-2.19
266700_at	60S ribosomal protein L31 ; suppor	---	AT2G19740	1.62	0.69	4.30	0.003893	0.01988	-2.19
247545_at	putative protein rhoGAP, Homo sa	---	AT5G61530	1.36	0.45	4.30	0.003896	0.019892	-2.19

258617_at	putative calmodulin similar to calmo	---	AT3G03000	-1.33	-0.42	-4.30	0.003902	0.019914	-2.19
258881_at	unknown protein ; supported by ful	---	AT3G06310	1.43	0.52	4.30	0.003903	0.019918	-2.19
248189_at	DNA mismatch repair protein MutS2	---	AT5G54090	1.52	0.60	4.30	0.003905	0.019921	-2.19
259493_at	unknown protein	---	AT1G15840	-1.41	-0.50	-4.30	0.003906	0.019922	-2.19
251151_at	hypothetical protein ; supported by	---	AT3G63170	-1.51	-0.60	-4.30	0.003909	0.019935	-2.20
248958_at	unknown protein ; supported by ful	---	AT5G45590	-1.62	-0.70	-4.30	0.003913	0.019952	-2.20
251821_at	protein phosphatase 2C - like prote	---	AT3G55050	-2.03	-1.02	-4.30	0.003914	0.019952	-2.20
264848_at	unknown protein ;supported by full-	---	AT2G17350	1.32	0.40	4.30	0.003917	0.019961	-2.20
267557_at	unknown protein ; supported by cD	KRP4	AT2G32710	1.54	0.62	4.30	0.003924	0.019991	-2.20
257754_at	GTP binding protein, putative simila	ATRAB7B	AT3G18820	-1.37	-0.45	-4.30	0.003925	0.019991	-2.20
267269_at	unknown protein	CAND1	AT2G02560	1.45	0.53	4.30	0.003929	0.020007	-2.20
256836_at	pyruvate kinase, putative similar to	PKP-ALPH	AT3G22960	1.54	0.62	4.29	0.003933	0.020024	-2.20
254684_at	glycine-rich RNA-binding protein A	GR-RBP2	AT4G13850	1.67	0.74	4.29	0.003934	0.020026	-2.20
250584_at	WD-repeat protein-like	---	AT5G07590	-1.49	-0.58	-4.29	0.003935	0.020026	-2.20
257851_at	unknown protein	---	AT3G12940	-1.42	-0.51	-4.29	0.003942	0.020057	-2.20
254105_at	magnesium-protoporphyrin IX meth	CHLM	AT4G25080	-1.46	-0.55	-4.29	0.003947	0.020078	-2.21
261904_at	unknown protein contains similarity	---	AT1G65040	1.33	0.41	4.29	0.003949	0.020086	-2.21
251725_at	putative protein	---	AT3G56260	-1.73	-0.79	-4.29	0.003951	0.02009	-2.21
267115_s_a	putative cellulose synthase	ATCSLB04	AT2G32530	2.22	1.15	4.29	0.003953	0.020095	-2.21
265114_at	hypothetical protein contains simila	LRX2	AT1G62440	-1.95	-0.96	-4.29	0.003955	0.0201	-2.21
255118_at	nitrilase 1 like protein nitrilase 1 - M	---	AT4G08790	1.50	0.58	4.29	0.003961	0.020128	-2.21
253329_at	hypothetical protein	---	AT4G33480	1.35	0.43	4.29	0.00397	0.020166	-2.21
262680_at	anter-specific proline-rich -like prote	---	AT1G75880	1.76	0.82	4.29	0.003971	0.020168	-2.21
252595_at	putative transporter protein peptide	---	AT3G45690	-1.44	-0.52	-4.29	0.003973	0.020176	-2.21
265990_at	putative prolylcarboxypeptidase	---	AT2G24280	1.73	0.79	4.29	0.003976	0.020185	-2.21
256068_at	hypothetical protein contains simila	ATE1	AT1G13690	1.37	0.45	4.28	0.00398	0.020196	-2.22
266170_at	unknown protein ;supported by full-	---	AT2G39050	-1.89	-0.91	-4.28	0.003986	0.020223	-2.22
252202_at	anthranilate N-hydroxycinnamoyl/b	---	AT3G50300	-1.40	-0.49	-4.28	0.00399	0.02024	-2.22
251657_at	putative protein MULTICOPY SUP	---	AT3G57000	1.78	0.83	4.28	0.003993	0.020251	-2.22
245572_at	hypothetical protein	PPD2	AT4G14720	1.82	0.86	4.28	0.004	0.020278	-2.22
247694_at	GTP cyclohydrolase II / 3,4-dihydro	---	AT5G59750	1.44	0.53	4.28	0.004001	0.020278	-2.22
263921_at	putative fructose bisphosphate aldo	---	AT2G36460	1.55	0.64	4.28	0.004008	0.02031	-2.22
265043_at	Expressed protein ; supported by cl	ATNAP4	AT1G03900	1.44	0.53	4.28	0.004014	0.020338	-2.22
246443_at	hypothetical protein ;supported by	---	AT5G17620	-1.47	-0.55	-4.28	0.004016	0.020344	-2.23
248685_at	putative protein similar to unknown	---	AT5G48500	1.70	0.76	4.28	0.004022	0.02037	-2.23

258780_at	putative ubiquitin carboxyl-terminal	UBP13	AT3G11910	1.31	0.39	4.27	0.004036	0.020435	-2.23
260589_at	hypothetical protein predicted by ge	---	AT1G53400	-1.32	-0.41	-4.27	0.004037	0.020436	-2.23
267088_at	30S ribosomal protein S31 ;suppor	PSRP4	AT2G38140	-1.55	-0.63	-4.27	0.004038	0.020436	-2.23
264726_at	Expressed protein ; supported by fu	---	AT1G22985	1.56	0.64	4.27	0.00404	0.020444	-2.23
263415_at	hypothetical protein similar to GB:A	EMB2762	AT2G17250	1.61	0.69	4.27	0.004049	0.020476	-2.23
251795_at	putative protein MtN24, Medicago t	---	AT3G55390	1.68	0.75	4.27	0.004051	0.020479	-2.23
249147_at	cytosolic malate dehydrogenase ;su	---	AT5G43330	1.56	0.64	4.27	0.004054	0.02049	-2.24
250403_at	argininosuccinate lyase (AtArgH) ;	---	AT5G10920	1.90	0.93	4.27	0.00406	0.020511	-2.24
259528_at	hypothetical protein	---	AT1G12330	-1.66	-0.73	-4.27	0.004062	0.020518	-2.24
258699_at	unknown protein	---	AT3G09470	1.47	0.55	4.27	0.004065	0.020529	-2.24
245852_at	ribosomal protein L10-like ribosom	---	AT5G13510	-1.53	-0.61	-4.27	0.00407	0.020549	-2.24
257726_at	hypothetical protein predicted by ge	---	AT3G18360	-1.37	-0.46	-4.27	0.004071	0.020549	-2.24
265728_at	hypothetical protein predicted by ge	---	AT2G31990	1.60	0.68	4.27	0.004073	0.020555	-2.24
249076_at	putative protein similar to unknown	TOM22-V	AT5G43970	1.61	0.69	4.26	0.004075	0.020555	-2.24
260408_at	putative thioredoxin similar to thiore	ATH8	AT1G69880	1.50	0.59	4.26	0.004075	0.020555	-2.24
261804_at	UDP glucose:flavonoid 3-o-glucosy	UGT78D1	AT1G30530	-1.69	-0.76	-4.26	0.004078	0.020563	-2.24
263137_at	gamma glutamyl hydrolase, putativ	ATGGH1	AT1G78660	-2.04	-1.03	-4.26	0.004078	0.020563	-2.24
250135_at	putative protein	---	AT5G15360	1.41	0.49	4.26	0.004085	0.020591	-2.24
267446_s_a	putative tyrosyl-tRNA synthetase	---	AT1G28350 /	1.35	0.44	4.26	0.004086	0.020591	-2.24
249247_at	maize crp1 protein-like	---	AT5G42310	1.54	0.63	4.26	0.004089	0.020601	-2.24
261378_at	calcium-dependent protein kinase,	ATCDPK1	AT1G18890	-1.71	-0.77	-4.26	0.004089	0.020601	-2.24
251262_at	hypothetical protein ; supported by	---	AT3G62080	1.71	0.77	4.26	0.004092	0.020609	-2.25
247282_at	putative protein strong similarity to u	AtMC3	AT5G64240	-1.45	-0.53	-4.26	0.004098	0.020634	-2.25
250906_at	1,4-alpha-glucan branching enzym	SBE2.2	AT5G03650	-1.45	-0.54	-4.26	0.004099	0.020634	-2.25
253302_at	Expressed protein ; supported by fu	---	AT4G33660	-1.63	-0.71	-4.26	0.0041	0.020636	-2.25
247339_at	unknown protein	---	AT5G63690	1.50	0.58	4.26	0.00411	0.020679	-2.25
263844_at	unknown protein ; supported by cD	---	AT2G36930	2.15	1.11	4.26	0.00411	0.020679	-2.25
260781_at	decoy identical to GB:AAB51588 G	DECOY	AT1G14620	2.73	1.45	4.26	0.004114	0.020692	-2.25
254032_at	predicted protein destination factor	---	AT4G25940	-1.56	-0.64	-4.26	0.004116	0.020697	-2.25
258608_at	unknown protein ;supported by full-	---	AT3G03020	-1.96	-0.97	-4.26	0.00412	0.020714	-2.25
264171_at	hypothetical protein predicted by ge	---	AT1G02100	1.50	0.59	4.26	0.004121	0.020714	-2.25
264875_at	putative glucanase	AKR2B	AT2G17390	1.30	0.38	4.25	0.004132	0.020764	-2.26
262522_at	putative amino acid transporter Ver	CAT8	AT1G17120	-1.44	-0.53	-4.25	0.004134	0.020768	-2.26
249134_at	unknown protein	---	AT5G43150	1.82	0.86	4.25	0.004134	0.020768	-2.26
246864_at	cytochrome P450 GA3 ; supported	GA3	AT5G25900	-1.92	-0.94	-4.25	0.004141	0.020797	-2.26

249742_at	putative protein contains similarity t	---	AT5G24490	-1.50	-0.59	-4.25	0.004143	0.0208	-2.26
258027_at	hypothetical protein predicted by ge	---	AT3G19515	1.40	0.49	4.25	0.004147	0.020818	-2.26
261350_at	hypothetical protein predicted by ge	---	AT1G79770	1.69	0.76	4.25	0.004155	0.020853	-2.26
258643_at	unknown protein ;supported by full-	ATAB2	AT3G08010	1.33	0.41	4.25	0.004163	0.020889	-2.26
252822_at	Expressed protein ; supported by fu	---	AT4G39955	1.48	0.57	4.25	0.004169	0.020914	-2.27
257806_at	hypothetical protein predicted by ge	---	AT3G18670	-1.39	-0.47	-4.24	0.004188	0.020998	-2.27
262245_at	unknown protein	NPSN12	AT1G48240	-1.55	-0.63	-4.24	0.004189	0.020998	-2.27
261601_at	ARP protein, putative similar to AR	NQR	AT1G49670	1.37	0.46	4.24	0.004189	0.020998	-2.27
262107_at	hypothetical protein predicted by ge	---	AT1G02750	1.36	0.44	4.24	0.004189	0.020998	-2.27
263705_at	unknown protein contains similarity	IMPL1	AT1G31190	-1.59	-0.67	-4.24	0.004191	0.021002	-2.27
259462_at	hypothetical protein contains similar	---	AT1G18940	-1.61	-0.68	-4.24	0.004193	0.021004	-2.27
251830_at	phosphoribosylformylglycinamidine	PUR5	AT3G55010	1.85	0.88	4.24	0.004194	0.021005	-2.27
248032_at	myosin heavy chain-like	---	AT5G55860	1.55	0.63	4.24	0.004199	0.021025	-2.27
250193_at	putative protein proline-rich protein	---	AT5G14540	-1.41	-0.49	-4.24	0.004201	0.021027	-2.27
259397_at	hypothetical protein predicted by ge	---	AT1G17680	1.37	0.46	4.24	0.004201	0.021027	-2.27
266556_at	hypothetical protein predicted by ge	---	AT2G46230	1.49	0.58	4.24	0.004203	0.021027	-2.27
247810_at	26S proteasome AAA-ATPase subu	RPT3	AT5G58290	1.36	0.45	4.24	0.004203	0.021027	-2.27
262624_at	CCR4-associated factor, putative s	---	AT1G06450	1.45	0.54	4.24	0.004204	0.021027	-2.27
258300_at	putative casein kinase I similar to ca	ckl10	AT3G23340	1.65	0.73	4.24	0.004205	0.021031	-2.27
267310_at	unknown protein	AIR9	AT2G34680	-2.51	-1.33	-4.24	0.00421	0.021051	-2.28
261694_at	60S ribosomal protein L10A, putati	---	AT1G08360	1.62	0.70	4.24	0.004215	0.021068	-2.28
247642_at	putative protein various predicted p	---	AT5G60590	1.37	0.46	4.24	0.004218	0.021078	-2.28
251246_at	auxin-induced protein homolog aux	IAA30	AT3G62100	-1.32	-0.40	-4.24	0.004218	0.021078	-2.28
267612_at	putative nitrate transporter	---	AT2G26690	-2.64	-1.40	-4.23	0.004225	0.021107	-2.28
263787_at	unknown protein ; supported by cD	---	AT2G46420	1.66	0.73	4.23	0.004228	0.021115	-2.28
247926_at	protein carboxyl methylase-like	---	AT5G57280	2.05	1.04	4.23	0.004229	0.021115	-2.28
247248_at	putative protein contains similarity t	---	AT5G64560	-1.83	-0.87	-4.23	0.00423	0.021115	-2.28
263432_at	putative beta-hydroxyacyl-ACP deh	---	AT2G22230	-1.55	-0.63	-4.23	0.004234	0.021121	-2.28
246038_s_a	putative protein thiamin pyrophosp	atnudt20	AT5G19460 ,	-1.58	-0.66	-4.23	0.004234	0.021121	-2.28
264601_at	hypothetical protein similar to hypot	---	AT1G04540	-1.41	-0.50	-4.23	0.004234	0.021121	-2.28
266336_at	putative Fe(II) transporter identical	ZIP3	AT2G32270	-1.52	-0.61	-4.23	0.004235	0.021121	-2.28
259750_at	hypothetical protein predicted by ge	---	AT1G71130	1.69	0.75	4.23	0.004237	0.021123	-2.28
260420_at	unknown protein	---	AT1G69610	1.41	0.49	4.23	0.004238	0.021123	-2.28
251321_at	RING finger protein ;supported by f	BRH1	AT3G61460	-1.71	-0.78	-4.23	0.004239	0.021125	-2.28
265732_at	predicted by genscan and genefind	---	AT2G01300	-6.90	-2.79	-4.23	0.004241	0.021132	-2.28

247455_at	MYB96 transcription factor-like prot	MYB96	AT5G62470	-1.79	-0.84	-4.23	0.004243	0.021137	-2.28
246842_at	Expressed protein ; supported by fu	---	AT5G26731	1.80	0.85	4.23	0.004246	0.021146	-2.29
AFFX-Athal-	Arabidopsis thaliana /REF=U37281	---	---	-1.51	-0.59	-4.23	0.004247	0.021146	-2.29
259519_at	hypothetical protein	---	AT1G12270	1.53	0.61	4.23	0.004248	0.021146	-2.29
247569_at	Cf-5 disease resistance protein - lik	---	AT5G61240	-1.64	-0.71	-4.23	0.004251	0.02116	-2.29
261304_at	unknown protein ;supported by full-	---	AT1G48440	-1.30	-0.38	-4.23	0.004253	0.021161	-2.29
267035_at	putative beta-alanine-pyruvate amir	AGT3	AT2G38400	-2.77	-1.47	-4.23	0.004254	0.021164	-2.29
259188_at	unknown protein ;supported by full-	---	AT3G01510	-1.62	-0.70	-4.23	0.004256	0.021169	-2.29
246877_at	putative protein various predicted p	---	AT5G26150	-1.31	-0.39	-4.23	0.004265	0.02121	-2.29
248136_at	RNA helicase-like protein	---	AT5G54910	1.84	0.88	4.23	0.00427	0.021229	-2.29
265400_at	unknown protein ;supported by full-	---	AT2G10940	-117.42	-6.88	-4.23	0.004272	0.021229	-2.29
259160_at	unknown protein	---	AT3G05410	1.45	0.54	4.23	0.004272	0.021229	-2.29
263735_s_a	hypothetical protein predicted by ge	AGL50	AT1G59810	-1.33	-0.41	-4.23	0.004273	0.021229	-2.29
259436_at	hypothetical protein predicted by ge	---	AT1G01500	1.39	0.47	4.22	0.004281	0.021254	-2.29
264692_at	DNA binding protein MybSt1, putat	---	AT1G70000	1.52	0.60	4.22	0.004281	0.021254	-2.29
250720_at	putative protein similar to unknown	---	AT5G06180	1.64	0.71	4.22	0.004281	0.021254	-2.29
247081_at	AtRAD17 (dbj|BAA90479.1) ; supp	ATRAD17	AT5G66130	1.48	0.57	4.22	0.004282	0.021254	-2.29
257171_at	unknown protein ; supported by ful	---	AT3G23760	-1.46	-0.55	-4.22	0.00429	0.021291	-2.30
259381_s_a	putative lectin contains Pfam profile	NSP1	AT3G16390	-4.10	-2.04	-4.22	0.004296	0.021313	-2.30
267062_at	putative spliceosome associated pr	---	AT2G32600	1.51	0.59	4.22	0.004298	0.021315	-2.30
265798_at	unknown protein ; supported by cDI	---	AT2G35790	1.67	0.74	4.22	0.004299	0.021316	-2.30
263333_at	unknown protein ; supported by cD	---	AT2G03890	1.59	0.67	4.22	0.0043	0.021316	-2.30
260096_at	hypothetical protein predicted by ge	---	AT1G73240	1.50	0.59	4.22	0.004302	0.021323	-2.30
257059_at	unknown protein ; supported by cD	---	AT3G15280	-1.35	-0.44	-4.22	0.004304	0.021328	-2.30
250009_at	putative protein nuclear FMRP inte	---	AT5G18440	2.05	1.03	4.22	0.004309	0.021348	-2.30
267135_at	cyclin-dependent kinase inhibitor pr	ICK1	AT2G23430	1.93	0.95	4.22	0.004311	0.021353	-2.30
248762_at	Expressed protein ; supported by fu	---	AT5G47455	1.75	0.80	4.22	0.004314	0.02136	-2.30
254259_s_a	putative protein rape mRNA, Brass	---	AT4G23420	-1.67	-0.74	-4.22	0.004314	0.02136	-2.30
254425_at	putative membrane associated pro	---	AT4G21450	1.33	0.41	4.22	0.004315	0.02136	-2.30
252938_at	hypothetical protein	---	AT4G39190	2.79	1.48	4.22	0.004322	0.021388	-2.30
261905_at	hypothetical protein contains similar	---	AT1G65070	1.39	0.47	4.22	0.004324	0.021388	-2.31
256426_at	hypothetical protein predicted by ge	---	AT1G33420	2.40	1.27	4.22	0.004324	0.021388	-2.31
266743_at	ribonuclease, RNS1 identical to rib	RNS1	AT2G02990	-5.41	-2.43	-4.22	0.004327	0.021389	-2.31
253903_at	kinesin-related protein katB ; suppo	ATK2	AT4G27180	1.29	0.37	4.21	0.004335	0.021427	-2.31
258678_at	E2, ubiquitin-conjugating enzyme 1	UBC11	AT3G08690	1.56	0.64	4.21	0.004337	0.021431	-2.31

257779_at	protein kinase, putative similar to P	CDG1	AT3G26940	-1.29	-0.37	-4.21	0.004339	0.021431	-2.31
256944_at	unknown protein similar to unknown	VRN1	AT3G18990	1.61	0.69	4.21	0.00434	0.021431	-2.31
253566_at	DNA topoisomerase like- protein Ba	---	AT4G31210	2.53	1.34	4.21	0.00434	0.021431	-2.31
252313_at	RNA-binding - like protein RNA-bin	CID10	AT3G49390	1.82	0.86	4.21	0.004342	0.021435	-2.31
259113_at	hypothetical protein predicted by ge	---	AT3G05510	1.47	0.55	4.21	0.004356	0.021496	-2.31
262407_at	hypothetical protein predicted by ge	---	AT1G34630	-1.82	-0.86	-4.21	0.004365	0.021536	-2.32
266673_at	putative thiamin biosynthesis protei	THIC	AT2G29630	1.73	0.79	4.21	0.004373	0.02157	-2.32
265941_s_a	recA, putative similar to GB:CAA39	---	AT2G19490	1.74	0.80	4.21	0.004376	0.02158	-2.32
264309_at	coatomer alpha subunit, putative si	---	AT1G62020	-1.33	-0.42	-4.21	0.004377	0.021584	-2.32
258832_at	putative protein kinase similar to AF	---	AT3G07070	-1.44	-0.52	-4.21	0.004379	0.021585	-2.32
246008_at	putative protein predicted proteins,	---	AT5G08320	-1.49	-0.57	-4.20	0.004382	0.021596	-2.32
255801_at	putative protein heat shock protein	---	AT4G10130	1.34	0.42	4.20	0.004384	0.021601	-2.32
249579_at	ADP-ribosylation factor - like protei	ATARLA1A	AT5G37680	1.40	0.48	4.20	0.00439	0.021626	-2.32
250823_at	putative protein similar to unknown	---	AT5G05180	-1.40	-0.49	-4.20	0.004391	0.021627	-2.32
258544_at	disease resistance gene (RPM1) id	RPM1	AT3G07040	-1.83	-0.87	-4.20	0.004394	0.021638	-2.32
267060_at	unknown protein ; supported by ful	---	AT2G32580	-1.68	-0.75	-4.20	0.004396	0.021642	-2.32
252181_at	Expressed protein ; supported by fu	---	AT3G50685	-1.37	-0.46	-4.20	0.004398	0.021648	-2.32
248861_at	senescence-associated protein 5-lik	TRN2	AT5G46700	-2.97	-1.57	-4.20	0.0044	0.021652	-2.32
266157_at	unknown protein	ATFUC1	AT2G28100	-1.29	-0.37	-4.20	0.004403	0.021662	-2.32
250066_at	unknown protein	---	AT5G17930	1.74	0.80	4.20	0.004405	0.021669	-2.33
267205_at	unknown protein	---	AT2G30820	-1.43	-0.52	-4.20	0.004408	0.021678	-2.33
263525_at	putative MADS-box protein	AGL61	AT2G24840	-1.52	-0.60	-4.20	0.00441	0.021683	-2.33
263199_at	hypothetical protein Similar to hexo	HEXO2	AT1G05590	-1.56	-0.64	-4.20	0.004411	0.021683	-2.33
266702_at	hexokinase (ATHXK2) ; supported	HXK2	AT2G19860	1.38	0.46	4.20	0.004413	0.02169	-2.33
258504_at	putative sterol-C5-desaturase ident	---	AT3G02590	-1.31	-0.39	-4.20	0.004429	0.021753	-2.33
258284_at	putative ribosomal protein similar to	---	AT3G16080	2.08	1.06	4.20	0.00443	0.021753	-2.33
265002_at	putative lysine/histidine-specific pe	LHT2	AT1G24400	1.65	0.72	4.20	0.00443	0.021753	-2.33
245296_at	isp4 like protein ; supported by cDN	ATOPT3	AT4G16370	-1.83	-0.87	-4.20	0.00443	0.021753	-2.33
262159_at	hypothetical protein predicted by ge	---	AT1G52720	-2.19	-1.13	-4.20	0.004431	0.021753	-2.33
257621_at	calmodulin-domain protein kinase C	CPK9	AT3G20410	1.87	0.90	4.19	0.004439	0.021788	-2.33
255839_at	putative CONSTANS-like B-box zin	---	AT2G33500	1.34	0.42	4.19	0.00447	0.021938	-2.34
245040_at	hypothetical protein predicted by ge	---	AT2G26520	-1.44	-0.53	-4.19	0.004473	0.021947	-2.34
260878_at	scarecrow-like 1 identical to scarec	SCL1	AT1G21450	1.62	0.69	4.19	0.004477	0.021958	-2.34
255864_at	putative pseudouridine synthase	---	AT2G30320	-1.46	-0.54	-4.19	0.004477	0.021958	-2.34
260858_at	hypothetical protein predicted by ge	---	AT1G43770	1.40	0.49	4.18	0.004487	0.022003	-2.35

245351_at	casein kinase II beta chain CKB2 ;s	CKB2	AT4G17640	-1.41	-0.50	-4.18	0.00449	0.022011	-2.35
254663_at	potassium channel protein KAT2	KAT2	AT4G18290	-1.60	-0.68	-4.18	0.004493	0.022021	-2.35
245616_at	kinase like protein	---	AT4G14480	1.89	0.92	4.18	0.004494	0.022021	-2.35
256724_at	unknown protein ;supported by full-	---	AT2G34040	1.44	0.53	4.18	0.004501	0.022049	-2.35
265599_at	unknown protein	AT2G1425	---	-1.39	-0.47	-4.18	0.004509	0.022087	-2.35
266003_at	hypothetical protein predicted by ge	---	AT2G37320	1.58	0.66	4.18	0.004511	0.022088	-2.35
256343_at	unknown protein ; supported by cD	---	AT1G72090	1.47	0.56	4.18	0.004519	0.022119	-2.35
249479_at	germin - like protein probable germ	---	AT5G38960	-1.53	-0.61	-4.18	0.004524	0.02214	-2.35
264679_s_a	putative 60S ribosomal protein L21	---	AT1G09590	1.77	0.83	4.18	0.004529	0.022159	-2.36
251719_at	putative protein PUTATIVE PROTE	---	AT3G56140	1.49	0.57	4.18	0.004537	0.022191	-2.36
266588_at	putative proline-rich protein ; suppo	AGP9	AT2G14890	-5.86	-2.55	-4.18	0.00454	0.022194	-2.36
259012_at	hypothetical protein similar to hypot	PUB9	AT3G07360	-1.56	-0.64	-4.18	0.00454	0.022194	-2.36
246139_at	putative protein predicted protein, I	---	AT5G19900	1.48	0.56	4.17	0.004543	0.022202	-2.36
251353_at	putative protein hypothetical protei	---	AT3G61080	-1.54	-0.62	-4.17	0.004546	0.022204	-2.36
254796_at	putative protein protein kinase hom	---	AT4G13000	-1.48	-0.56	-4.17	0.004546	0.022204	-2.36
265438_at	hypothetical protein	---	AT2G20970	-1.32	-0.40	-4.17	0.004546	0.022204	-2.36
258313_at	unknown protein contains RanBP1	---	AT3G15970	1.35	0.43	4.17	0.004548	0.022204	-2.36
245635_at	zinc finger protein ID1, putative sin	AtIDD16	AT1G25250	-1.32	-0.40	-4.17	0.004552	0.022213	-2.36
257826_at	RING zinc finger protein, putative c	---	AT3G26730	1.37	0.46	4.17	0.004552	0.022213	-2.36
247670_at	putative protein predicted proteins,	---	AT5G60190	1.55	0.63	4.17	0.004558	0.022237	-2.36
253297_at	beta-xylan endohydrolase -like pro	---	AT4G33810	-1.38	-0.47	-4.17	0.004559	0.022237	-2.36
264157_at	xyloglucan endotransglycosylase, p	XTH17	AT1G65310	-1.87	-0.90	-4.17	0.00456	0.022237	-2.36
257489_at	hypothetical protein predicted by ge	---	AT1G07330	-1.38	-0.47	-4.17	0.004562	0.022238	-2.36
250107_at	ids-4 protein - like ids-4 protein, Ho	SPX4	AT5G15330	-1.63	-0.71	-4.17	0.004563	0.022238	-2.36
252096_at	hypothetical protein ; supported by	---	AT3G51180	1.79	0.84	4.17	0.004563	0.022238	-2.36
259089_at	hypothetical protein similar to GB:C	---	AT3G04960	-1.38	-0.46	-4.17	0.004564	0.022238	-2.36
263516_at	AtRer1B ;supported by full-length c	ATRER1B	AT2G21600	-1.43	-0.52	-4.17	0.004576	0.022292	-2.37
251306_at	putative DNA-binding protein DNA-	---	AT3G61260	-1.54	-0.62	-4.17	0.004578	0.022294	-2.37
247815_at	ribosomal protein S4 - like ribosom	---	AT5G58420	1.65	0.73	4.17	0.004582	0.022308	-2.37
262722_at	sterol glucosyltransferase, putative	---	AT1G43620	-1.36	-0.44	-4.17	0.00459	0.022336	-2.37
247528_at	MIM protein ; supported by cDNA:	MIM	AT5G61460	1.47	0.55	4.17	0.004594	0.022351	-2.37
264748_at	hypothetical protein predicted by ge	EMB25	AT1G70070	1.32	0.40	4.17	0.004595	0.022352	-2.37
259531_at	hypothetical protein	---	AT1G12460	-1.66	-0.73	-4.17	0.004596	0.022354	-2.37
251840_at	protein disulfide-isomerase-like pro	ATPDIL1-3	AT3G54960	1.93	0.95	4.16	0.004603	0.022379	-2.37
246977_at	putative protein CONSTANS-like p	COL4	AT5G24930	-1.33	-0.41	-4.16	0.004607	0.022382	-2.37

259254_at	putative P-protein: chorismate mut	ADT2	AT3G07630	1.45	0.54	4.16	0.004608	0.022382	-2.37
246543_at	putative protein	---	AT5G15060	-1.30	-0.37	-4.16	0.004609	0.022382	-2.37
267181_at	putative alcohol dehydrogenase ; s	---	AT2G37760	1.89	0.92	4.16	0.004609	0.022382	-2.37
251119_at	putative protein nifR3 protein, Azos	---	AT3G63510	-1.60	-0.68	-4.16	0.004614	0.0224	-2.38
265225_at	putative PHD-type zinc finger protei	---	AT2G36720	1.39	0.47	4.16	0.004617	0.022413	-2.38
262916_at	glutathione S-transferase, putative	ATGSTU16	AT1G59700	1.75	0.80	4.16	0.004625	0.022443	-2.38
262572_at	unknown protein Contains a PF|001	---	AT1G15140	-1.52	-0.60	-4.16	0.004625	0.022443	-2.38
263090_at	hypothetical protein predicted by ge	---	AT2G16190	-1.34	-0.43	-4.16	0.004629	0.022454	-2.38
266274_at	putative protein phosphatase 2C	---	AT2G29380	-1.39	-0.47	-4.16	0.004632	0.02246	-2.38
249269_at	cell cycle control crn (crooked neck	---	AT5G41770	1.55	0.64	4.16	0.004649	0.022529	-2.38
248231_at	putative protein contains similarity t	---	AT5G53770	1.44	0.53	4.16	0.004649	0.022529	-2.38
259070_at	digalactosyldiacylglycerol synthase	DGD1	AT3G11670	1.95	0.96	4.16	0.004649	0.022529	-2.38
262575_at	putative ABC transporter Similar to	PDR7	AT1G15210	-1.41	-0.50	-4.16	0.00465	0.022529	-2.38
247731_at	glucuronosyl transferase - like prot	UGT76E2	AT5G59590	-1.39	-0.47	-4.16	0.004652	0.022534	-2.38
257423_at	hypothetical protein predicted by ge	---	AT1G62010	1.46	0.55	4.15	0.004658	0.022552	-2.39
253144_at	putative protein transcription factor	---	AT4G35540	1.37	0.46	4.15	0.004661	0.022563	-2.39
251376_at	scarecrow - like protein scarecrow-	---	AT3G60630	-1.71	-0.77	-4.15	0.004671	0.022605	-2.39
259823_at	beta-1,3-glucanase precursor, puta	---	AT1G66250	1.47	0.56	4.15	0.004673	0.02261	-2.39
261145_at	thioredoxin identical to GB:CAA846	ATTRX4	AT1G19730	1.40	0.48	4.15	0.004674	0.02261	-2.39
257147_at	unknown protein	---	AT3G27270	1.62	0.69	4.15	0.004687	0.022668	-2.39
260712_at	protein phosphatase 2C, putative s	HAB2	AT1G17550	1.57	0.66	4.15	0.00469	0.022678	-2.39
252107_at	sugar transporter-like protein suga	TMT3	AT3G51490	-1.58	-0.66	-4.15	0.004696	0.022703	-2.39
249077_at	alcohol dehydrogenase (EC 1.1.1.1	HOT5	AT5G43940	1.43	0.51	4.15	0.004706	0.022741	-2.40
259994_at	putative C2H2-type zinc finger prot	AtIDD14	AT1G68130	-1.68	-0.75	-4.15	0.004708	0.022744	-2.40
266345_at	hypothetical protein predicted by ge	---	AT2G01410	-1.35	-0.44	-4.15	0.004708	0.022744	-2.40
249108_at	steroid sulfotransferase-like	---	AT5G43690	-1.29	-0.36	-4.14	0.004714	0.022764	-2.40
266514_at	putative zinc-finger protein (B-box z	---	AT2G47890	1.58	0.66	4.14	0.004718	0.022776	-2.40
256276_at	geranylgeranyl transferase type II b	---	AT3G12070	1.41	0.50	4.14	0.004719	0.022776	-2.40
259977_at	unknown protein ;supported by full-	---	AT1G76590	2.35	1.23	4.14	0.004719	0.022776	-2.40
265897_at	hypothetical protein predicted by ge	MOT1	AT2G25680	1.75	0.81	4.14	0.004722	0.022786	-2.40
249092_at	putative protein similar to unknown	---	AT5G43710	1.45	0.54	4.14	0.004723	0.022786	-2.40
253320_at	putative protein ThiJ protein, Esche	---	AT4G34020	1.39	0.47	4.14	0.004726	0.022786	-2.40
261213_at	unknown protein ; supported by cDI	---	AT1G12970	1.38	0.47	4.14	0.004726	0.022786	-2.40
246075_at	monogalactosyldiacylglycerol synth	MGD2	AT5G20410	2.71	1.44	4.14	0.004726	0.022786	-2.40
261832_at	S-ribonuclease binding protein SBI	---	AT1G10650	1.68	0.75	4.14	0.004738	0.022837	-2.40

265027_at	putative ribonuclease III similar to E	NFD2	AT1G24450	1.54	0.62	4.14	0.004739	0.022841	-2.40
253575_at	putative protein TINY, Arabidopsis	---	AT4G31060	-1.38	-0.47	-4.14	0.004744	0.022855	-2.41
250216_at	unknown protein	---	AT5G14090	-1.52	-0.60	-4.14	0.004744	0.022855	-2.41
260598_at	unknown protein	---	AT1G55930	1.62	0.70	4.14	0.004748	0.022866	-2.41
259373_at	hypothetical protein predicted by ge	---	AT1G69160	-2.29	-1.20	-4.14	0.004752	0.022882	-2.41
257112_at	cytochrome P450, putative contain	CYP705A2	AT3G20120	1.41	0.50	4.14	0.004755	0.022892	-2.41
245732_at	unknown protein contains similarity	---	AT1G73440	1.38	0.46	4.14	0.004759	0.022908	-2.41
246231_at	putative protein	---	AT4G37080	-1.57	-0.65	-4.14	0.004766	0.022933	-2.41
259379_at	putative MYB family transcription fa	---	AT3G16350	-2.10	-1.07	-4.13	0.004769	0.022945	-2.41
255444_at	LUMINIDEPENDENS protein ; sup	LD	AT4G02560	1.38	0.46	4.13	0.004772	0.022957	-2.41
249926_at	E1, ubiquitin-like activating enzyme	ECR1	AT5G19180	1.49	0.57	4.13	0.00478	0.022989	-2.41
254048_at	putative protein various predicted p	---	AT4G25680	-1.73	-0.79	-4.13	0.004786	0.02301	-2.41
265818_at	putative peptidyl-prolyl cis-trans iso	PIN1AT	AT2G18040	1.36	0.44	4.13	0.004789	0.023023	-2.42
246094_at	putative protein predicted proteins,	---	AT5G19300	1.72	0.78	4.13	0.004792	0.023026	-2.42
AFFX-r2-At-	Arabidopsis thaliana /REF=M6411	---	---	1.93	0.95	4.13	0.004813	0.023124	-2.42
254803_at	putative protein several hypothetica	---	AT4G13100	1.65	0.73	4.13	0.004822	0.023156	-2.42
264895_at	putative 10kd chaperonin strongly s	---	AT1G23100	2.24	1.16	4.13	0.004823	0.023156	-2.42
265998_at	putative NADP-dependent glyceral	ALDH11A3	AT2G24270	1.42	0.51	4.13	0.004823	0.023156	-2.42
250752_at	cytochrome P450 90A1 (sp|Q4256	CPD	AT5G05690	-1.50	-0.59	-4.13	0.004824	0.023157	-2.42
249944_at	NAM (no apical meristem)-like prot	anac089	AT5G22290	2.24	1.16	4.12	0.004834	0.0232	-2.43
254346_at	symbiosis-related like protein symb	APG8A	AT4G21980	1.78	0.83	4.12	0.004836	0.023202	-2.43
264022_at	Expressed protein ; supported by fu	---	AT2G21185	1.54	0.62	4.12	0.00484	0.023212	-2.43
247407_at	putative protein predicted proteins,	---	AT5G62900	1.54	0.62	4.12	0.004841	0.023212	-2.43
258218_at	methyltransferase, putative similar	XPL1	AT3G17998	-1.64	-0.71	-4.12	0.004842	0.023212	-2.43
261581_at	serine/threonine kinase, putative si	CIPK9	AT1G01140	1.59	0.67	4.12	0.004847	0.023229	-2.43
260618_at	flower development protein cycloid	TCP3	AT1G53230	-1.48	-0.57	-4.12	0.004848	0.023229	-2.43
248027_at	outward rectifying potassium chann	ATKCO1	AT5G55630	-1.48	-0.56	-4.12	0.004849	0.023229	-2.43
260249_s_a	unknown protein similar to unknow	---	AT1G74280	1.50	0.58	4.12	0.00485	0.023229	-2.43
258849_at	putative UDP-glucose pyrophospho	UGP	AT3G03250	1.53	0.62	4.12	0.004851	0.023229	-2.43
253948_at	Avr9 elicitor response like protein A	---	AT4G26940	-1.39	-0.47	-4.12	0.004858	0.023253	-2.43
255258_at	contains similarity to Arabidopsis th	---	AT4G05060	-1.44	-0.53	-4.12	0.004858	0.023253	-2.43
258240_at	oleosin isoform identical to oleosin i	OLEO4	AT3G27660	-1.42	-0.51	-4.12	0.004863	0.023267	-2.43
251186_at	putative protein unknown protein A	---	AT3G62790	1.29	0.37	4.12	0.004865	0.023271	-2.43
256226_at	unknown protein ; supported by cD	ATDI19	AT1G56280	1.57	0.65	4.12	0.004867	0.023271	-2.43
250279_at	ABA-responsive protein - like ABA-	---	AT5G13200	-1.72	-0.78	-4.12	0.004867	0.023271	-2.43

245134_s_a	unknown protein	---	AT2G45250	1.39	0.47	4.12	0.004873	0.023294	-2.43
252110_at	disease resistance-like protein TMV	---	AT3G51560	-1.33	-0.41	-4.11	0.004892	0.023378	-2.44
264285_at	symbiosis-related protein, putative	ATG8C	AT1G62040	1.43	0.51	4.11	0.004894	0.023385	-2.44
250073_at	unknown protein ; supported by cD	ENH1	AT5G17170	1.99	0.99	4.11	0.004898	0.023397	-2.44
264921_at	RNA binding protein, putative simil	---	AT1G60650	1.43	0.51	4.11	0.004904	0.023418	-2.44
266991_at	putative CDP-diacylglycerol--glycer	PGP1	AT2G39290	-1.30	-0.38	-4.11	0.004904	0.023418	-2.44
265481_at	unknown protein ; supported by cD	---	AT2G15960	1.41	0.49	4.11	0.004907	0.023425	-2.44
261206_at	heat shock factor protein hsf8, puta	---	AT1G12800	-1.50	-0.59	-4.11	0.004916	0.023457	-2.44
262981_at	unknown protein ;supported by full-	---	AT1G75590	-1.54	-0.63	-4.11	0.004917	0.023458	-2.44
265336_at	hypothetical protein predicted by ge	APC10	AT2G18290	1.35	0.43	4.11	0.004918	0.023459	-2.44
248955_at	putative protein contains similarity t	GAS41	AT5G45600	1.35	0.43	4.11	0.00492	0.023459	-2.44
247844_at	ARR2 - like protein ARR2 protein, A	ARR18	AT5G58080	-1.33	-0.41	-4.11	0.004921	0.023459	-2.45
251392_at	dihydrodipicolinate synthase precur	DHDPS1	AT3G60880	1.36	0.44	4.11	0.004923	0.023459	-2.45
265388_s_a	unknown protein ;supported by full-	---	AT2G20990	-1.36	-0.45	-4.11	0.004923	0.023459	-2.45
245731_at	MAP kinase, putative similar to MA	MKK9	AT1G73500	1.98	0.98	4.11	0.004923	0.023459	-2.45
260889_at	hypothetical protein predicted by ge	---	AT1G29120	1.34	0.42	4.11	0.004933	0.023497	-2.45
245881_at	putative protein	---	AT5G09460	1.66	0.73	4.11	0.004933	0.023497	-2.45
266540_at	hypothetical protein predicted by ge	---	AT2G35310	-1.35	-0.43	-4.11	0.00494	0.023522	-2.45
259739_at	unknown protein supported by cDN	SEH1H	AT1G64350	1.40	0.48	4.11	0.004942	0.023529	-2.45
266500_at	Expressed protein ; supported by fu	PLA2-ALPH	AT2G06925	-1.79	-0.84	-4.10	0.004945	0.023534	-2.45
249993_at	GTP-binding protein obg -like GTP	---	AT5G18570	1.30	0.38	4.10	0.004948	0.02354	-2.45
250437_at	AtAGP4 ; supported by cDNA: gi_1	AGP4	AT5G10430	-3.77	-1.92	-4.10	0.004949	0.02354	-2.45
247099_at	putative protein contains similarity t	---	AT5G66500	1.45	0.54	4.10	0.00495	0.02354	-2.45
261108_at	1-aminocyclopropane-1-carboxylat	ACS10	AT1G62960	1.52	0.60	4.10	0.004951	0.023542	-2.45
265730_at	60S ribosomal protein L27	---	AT2G32220	2.89	1.53	4.10	0.004954	0.02355	-2.45
263237_at	hypothetical protein contains similar	---	AT1G10610	1.74	0.80	4.10	0.00496	0.023574	-2.45
252915_at	EF-Hand containing protein -like st	---	AT4G38810	-1.51	-0.59	-4.10	0.004964	0.023587	-2.45
246060_at	rev interacting protein mis3 - like re	---	AT5G08420	1.31	0.39	4.10	0.004966	0.023596	-2.46
254671_at	MuDR transposable element - like	---	---	-1.36	-0.44	-4.10	0.004969	0.023601	-2.46
255294_at	putative sugar transporter	---	AT4G04750	1.40	0.49	4.10	0.00497	0.023605	-2.46
254708_at	ubiquitin-specific protease 20 (UBF	UBP20	AT4G17895	-1.37	-0.45	-4.10	0.004971	0.023605	-2.46
259821_at	subtilisin-like serine protease conta	---	AT1G66220	-1.53	-0.62	-4.10	0.004973	0.023606	-2.46
248869_at	putative protein contains similarity t	---	AT5G46840	1.78	0.83	4.10	0.004978	0.023623	-2.46
246192_at	translation initiation factor eIF-2 be	EIF2 BETA	AT5G20920	1.33	0.41	4.10	0.004978	0.023623	-2.46
245991_at	24 kDa vacuolar protein - like 24 kD	---	AT5G20660	1.36	0.44	4.10	0.00498	0.023628	-2.46

257860_at	Expressed protein ; supported by cl	---	AT3G13062	1.65	0.72	4.10	0.004982	0.02363	-2.46
249849_at	putative protein similar to unknown	NIC2	AT5G23230	1.50	0.59	4.10	0.004983	0.023632	-2.46
258152_at	chaperonin subunit, putative simila	---	AT3G18190	1.45	0.54	4.10	0.004986	0.023638	-2.46
262067_at	hypothetical protein predicted by ge	---	AT1G80060	-1.49	-0.57	-4.10	0.004987	0.023638	-2.46
249322_at	protein import receptor TOM20, mi	TOM20-4	AT5G40930	1.32	0.40	4.10	0.004988	0.023641	-2.46
252912_at	ribosomal protein S25 ribosomal pr	---	AT4G39200	1.68	0.75	4.10	0.004992	0.023655	-2.46
245016_at	carboxytransferase beta subunit	---	---	-1.35	-0.44	-4.10	0.004998	0.023675	-2.46
248832_at	putative protein similar to unknown	---	AT5G47170	-1.32	-0.40	-4.10	0.004999	0.023675	-2.46
254830_at	putative protein predicted protein, S	---	AT4G12590	1.29	0.37	4.10	0.005004	0.023695	-2.46
263326_at	unknown protein ; supported by cD	---	AT2G04280	-1.62	-0.70	-4.10	0.005005	0.023696	-2.46
261270_at	initiation factor 5A-4, putative simila	FBR12	AT1G26630	1.34	0.42	4.09	0.005009	0.023709	-2.46
245054_at	unknown protein	---	AT2G26460	1.46	0.55	4.09	0.005012	0.023721	-2.47
264677_at	hypothetical protein predicted by ge	HYL1	AT1G09700	1.29	0.37	4.09	0.005016	0.023731	-2.47
259109_at	putative serine threonine protein ph	---	AT3G05580	-1.67	-0.74	-4.09	0.005021	0.023751	-2.47
251175_at	homocysteine S-methyltransferase	HMT2	AT3G63250	1.39	0.47	4.09	0.005024	0.02376	-2.47
249594_at	putative protein SIAH1 protein, Bra	---	AT5G37910	-1.34	-0.43	-4.09	0.005025	0.02376	-2.47
260126_at	putative hydroxymethyltransferase s	SHM7	AT1G36370	-1.68	-0.75	-4.09	0.005026	0.023762	-2.47
261923_at	UDP-glucose glucosyltransferase,	AtUGT85A:	AT1G22380	-1.44	-0.53	-4.09	0.005034	0.023792	-2.47
259668_at	hypothetical protein predicted by ge	---	AT1G52390	-1.37	-0.45	-4.09	0.005036	0.023797	-2.47
261742_at	hypothetical protein predicted by ge	---	AT1G08390	-1.34	-0.42	-4.09	0.005037	0.023798	-2.47
259544_at	hypothetical protein ; supported by f	CAT3	AT1G20620	1.37	0.45	4.09	0.00504	0.023804	-2.47
257905_at	unknown protein	---	---	-1.44	-0.53	-4.09	0.005049	0.023841	-2.47
251471_at	putative protein GTPASE-ACTIVAT	---	AT3G59570	-1.42	-0.51	-4.09	0.00505	0.023841	-2.47
255968_at	Expressed protein ; supported by fu	---	AT1G22270	1.47	0.55	4.09	0.00505	0.023841	-2.47
266606_at	putative AP2 domain transcription fa	CRF5	AT2G46310	1.80	0.85	4.09	0.005054	0.023851	-2.47
265779_at	unknown protein	---	AT2G07360	-1.67	-0.74	-4.09	0.00506	0.023874	-2.48
255136_s_a	putative protein chromatin structura	---	AT4G08350 ,	1.45	0.54	4.09	0.005062	0.023875	-2.48
257317_at	hypothetical protein	AT2G0774:	---	1.45	0.53	4.08	0.005071	0.023907	-2.48
249385_at	thioredoxin ; supported by full-leng	ATTRX2	AT5G39950	1.50	0.58	4.08	0.005071	0.023907	-2.48
249875_at	photosystem II stability/assembly fa	HCF136	AT5G23120	-1.70	-0.76	-4.08	0.005083	0.023951	-2.48
249286_at	transcription factor-like protein	---	AT5G41580	1.41	0.49	4.08	0.005083	0.023951	-2.48
255417_at	F-box protein GRR1-like protein 1,	GRH1	AT4G03190	-3.56	-1.83	-4.08	0.005089	0.023975	-2.48
258413_at	unknown protein ;supported by full-	---	AT3G17300	1.46	0.54	4.08	0.005093	0.023988	-2.48
262236_at	hypothetical protein similar to hypot	---	AT1G48330	-1.95	-0.96	-4.08	0.005107	0.024048	-2.49
250875_at	putative protein fimbriae-associated	---	AT5G04020	-2.29	-1.20	-4.08	0.005108	0.024049	-2.49

256612_at	hypothetical protein predicted by ge	---	AT3G29280	-1.47	-0.56	-4.08	0.005109	0.024049	-2.49
251502_at	putative protein several hypothetica	---	AT3G59130	-1.33	-0.41	-4.08	0.005112	0.024053	-2.49
248427_at	subtilisin-like serine protease simila	ATSBT1.3	AT5G51750	-1.54	-0.62	-4.08	0.005112	0.024053	-2.49
260692_at	hypothetical protein predicted by ge	---	AT1G32430	-1.36	-0.44	-4.08	0.005115	0.024061	-2.49
253083_at	aldehyde dehydrogenase like prote	ALDH3F1	AT4G36250	-1.96	-0.97	-4.08	0.005119	0.024073	-2.49
253360_at	aminopeptidase- like protein amino	APM1	AT4G33090	1.44	0.53	4.08	0.00512	0.024076	-2.49
250423_s_a	cytochrome P450 - like protein cyto	CYP81K2	AT5G10600	1.33	0.42	4.08	0.005124	0.024091	-2.49
266189_at	unknown protein	---	AT2G39020	-1.30	-0.38	-4.08	0.005127	0.024096	-2.49
246115_at	putative protein chloroplast outer e	---	AT5G20300	1.46	0.55	4.08	0.005129	0.024101	-2.49
260544_at	unknown protein predicted by gens	---	AT2G43540	1.62	0.70	4.08	0.00513	0.024101	-2.49
260654_at	hypothetical protein predicted by ge	---	AT1G19290	1.53	0.61	4.07	0.005132	0.024107	-2.49
253866_at	putative protein various putative pro	---	AT4G27480	1.45	0.53	4.07	0.005135	0.024114	-2.49
255394_at	hypothetical protein similar to A. th	---	---	-1.38	-0.47	-4.07	0.005139	0.024126	-2.49
258830_at	unknown protein	---	AT3G07090	-1.94	-0.96	-4.07	0.005141	0.024128	-2.49
259367_at	hypothetical protein predicted by ge	---	AT1G69070	1.67	0.74	4.07	0.005144	0.024137	-2.49
255627_at	Expressed protein ; supported by fu	---	AT4G00955	-1.33	-0.41	-4.07	0.005145	0.02414	-2.49
251379_at	putative protein various predicted A	---	AT3G60680	1.75	0.80	4.07	0.00515	0.024153	-2.49
252937_at	SEC14 - like protein phosphatidylin	SEC14	AT4G39180	-1.35	-0.44	-4.07	0.00515	0.024153	-2.49
250542_at	hypothetical protein (fragment at BA	---	AT5G08270	1.87	0.91	4.07	0.005153	0.024155	-2.50
246748_at	putative protein predicted protein, I	---	AT5G27730	1.39	0.47	4.07	0.005158	0.024172	-2.50
257844_at	prolyl 4-hydroxylase, putative simila	---	AT3G28480	1.56	0.64	4.07	0.005159	0.024172	-2.50
254327_at	RCc3- like protein RCc3 protein, O	---	AT4G22490	-2.07	-1.05	-4.07	0.00516	0.024172	-2.50
250429_at	putative protein TH65 protein, Arab	---	AT5G10470	1.39	0.48	4.07	0.005162	0.024179	-2.50
264475_s_a	hypothetical protein predicted by ge	---	AT1G77150	1.51	0.60	4.07	0.00517	0.024209	-2.50
252644_at	putative protein KIAA0073, Homo s	CYP71	AT3G44600	1.48	0.56	4.07	0.005191	0.024304	-2.50
262607_at	unknown protein ;supported by full-	---	AT1G13990	1.50	0.59	4.06	0.005195	0.024317	-2.50
256826_at	hypothetical protein predicted by ge	---	AT3G18540	-1.30	-0.38	-4.06	0.005202	0.024347	-2.51
264510_at	putative phytochrome-associated pr	PIF3	AT1G09530	1.82	0.87	4.06	0.005208	0.024366	-2.51
267584_at	putative protein kinase contains a p	---	AT2G41930	-1.38	-0.46	-4.06	0.005209	0.024366	-2.51
257269_at	unknown protein contains Pfam pro	NPSN13	AT3G17440	-1.61	-0.69	-4.06	0.005214	0.024384	-2.51
257863_at	hypothetical protein similar to GRAI	anac057	AT3G17730	-1.30	-0.38	-4.06	0.005217	0.024394	-2.51
259868_at	thioredoxin-like protein similar to th	ATY1	AT1G76760	1.35	0.44	4.06	0.00523	0.024444	-2.51
246501_at	putative protein KIAA1012 protein -	---	AT5G16280	-1.37	-0.45	-4.06	0.005242	0.024494	-2.51
264822_at	putative ribonucleoprotein	---	AT1G03457	-1.83	-0.87	-4.06	0.005246	0.024506	-2.51
252650_at	putative protein	---	AT3G44690	1.34	0.42	4.06	0.005249	0.024513	-2.51

252207_at	putative protein mRNA of unknown	---	AT3G50370	1.40	0.48	4.06	0.00525	0.024516	-2.52
258345_at	unknown protein	---	AT3G22845	1.35	0.44	4.06	0.005252	0.024521	-2.52
265805_s_a	60S ribosomal protein L2	EMB2296	AT2G18020	1.58	0.66	4.06	0.005255	0.024522	-2.52
248035_at	sucrose cleavage protein-like ;supp	---	AT5G55900	1.36	0.44	4.06	0.005255	0.024522	-2.52
255706_at	putative ribosomal protein S13 simi	ATRPS13A	AT4G00100	1.64	0.72	4.06	0.005256	0.024522	-2.52
246001_at	putative protein predicted protein, A	---	AT5G20790	1.99	0.99	4.05	0.005262	0.024545	-2.52
249607_at	putative protein RING-H2 finger pro	---	AT5G37280	-1.34	-0.43	-4.05	0.005276	0.024604	-2.52
259294_at	hypothetical protein predicted by ge	ATN	AT3G05330	1.62	0.70	4.05	0.005282	0.024623	-2.52
264115_at	hypothetical protein	---	AT2G31290	1.48	0.57	4.05	0.005283	0.024623	-2.52
264884_at	unknown protein ;supported by full-	---	AT1G61170	-1.95	-0.97	-4.05	0.005283	0.024623	-2.52
264259_at	hypothetical protein This gene is co	---	AT1G09290	1.64	0.72	4.05	0.005289	0.024644	-2.52
253126_at	putative protein probable membran	---	AT4G36050	1.77	0.82	4.05	0.005295	0.024668	-2.52
263295_at	putative MADS-box protein ANR1	AGL44	AT2G14210	1.31	0.39	4.05	0.005296	0.024668	-2.52
250884_at	signal recognition particle 54CP pro	CPSRP54	AT5G03940	-1.34	-0.43	-4.05	0.005305	0.024702	-2.53
246489_at	14-3-3 protein GF14upsilon (grf5) id	GRF5	AT5G16050	1.41	0.50	4.05	0.005309	0.024707	-2.53
255760_at	vacuolar-type H+-translocating inor	---	AT1G16780	1.39	0.47	4.05	0.005316	0.024735	-2.53
254061_at	putative protein several hypothetica	---	AT4G25360	-1.38	-0.47	-4.05	0.005318	0.024741	-2.53
261821_at	thioredoxin h, putative similar to thi	ATCXXS1	AT1G11530	1.40	0.49	4.05	0.005319	0.024741	-2.53
256807_at	unknown protein contains Pfam pro	NFU4	AT3G20970	1.38	0.47	4.05	0.005321	0.024742	-2.53
258789_at	Mutator-like transposase similar to I	---	---	1.39	0.47	4.04	0.005331	0.024784	-2.53
251023_at	putative protein amino acid transpo	---	AT5G02170	-1.44	-0.53	-4.04	0.005332	0.024785	-2.53
245927_at	sterol glucosyltransferase - like pro	---	AT5G24750	-1.28	-0.35	-4.04	0.005338	0.024804	-2.53
258141_at	hypothetical protein	HON4	AT3G18035	1.51	0.59	4.04	0.005351	0.024852	-2.54
261362_s_a	ribosomal protein identical to GB:CA	---	AT1G41880	1.75	0.81	4.04	0.005351	0.024852	-2.54
264587_at	putative ligand-gated ion channel pr	ATGLR3.4	AT1G05200	2.95	1.56	4.04	0.005352	0.024852	-2.54
252327_at	MTN3-like protein MtN3 gene prod	---	AT3G48740	1.61	0.69	4.04	0.005356	0.024868	-2.54
253881_at	putative protein Ran_GTP binding	---	AT4G27640	1.35	0.43	4.04	0.00536	0.024877	-2.54
257937_at	hypothetical protein predicted by ge	---	AT3G19810	1.31	0.39	4.04	0.00537	0.02491	-2.54
248838_at	carnitine/acylcarnitine translocase-l	BOU	AT5G46800	-1.73	-0.79	-4.04	0.00537	0.02491	-2.54
266382_at	hypothetical protein predicted by ge	---	---	-1.31	-0.39	-4.04	0.005371	0.02491	-2.54
251506_at	putative protein ; supported by cDN	---	AT3G59090	-1.30	-0.38	-4.04	0.005372	0.02491	-2.54
253886_at	cytochrome P450 - like protein cyto	CYP709B3	AT4G27710	-1.34	-0.43	-4.04	0.005374	0.024915	-2.54
251882_at	peptide transport - like protein pept	PTR1	AT3G54140	-1.46	-0.55	-4.04	0.005381	0.024943	-2.54
258495_at	unknown protein ;supported by full-	---	AT3G02690	1.34	0.42	4.03	0.005389	0.024964	-2.54
258907_at	putative sodium proton exchanger s	NHX4	AT3G06370	-1.42	-0.51	-4.03	0.005389	0.024964	-2.54

265857_s_a	serine threonine protein phosphatas	PP2A-4	AT2G42500 ,	1.42	0.51	4.03	0.005391	0.02497	-2.54
252622_at	cysteine proteinase AALP, putative	---	AT3G45310	1.42	0.51	4.03	0.005395	0.024979	-2.54
250414_at	putative protein Glr5, Arabidopsis t	GLR2.6	AT5G11180	-1.33	-0.41	-4.03	0.005396	0.024979	-2.54
245133_at	putative nucleotide sugar epimerase	GAE4	AT2G45310	-1.68	-0.75	-4.03	0.0054	0.024992	-2.55
245270_at	tubulin alpha-6 chain (TUA6) ; supp	TUA6	AT4G14960	-1.87	-0.90	-4.03	0.005401	0.024992	-2.55
249577_at	calmodulin-binding heat-shock prot	---	AT5G37710	-1.65	-0.73	-4.03	0.005402	0.024992	-2.55
252018_at	putative protein various predicted p	AtPP2-A15	AT3G53000	-1.57	-0.65	-4.03	0.005404	0.024996	-2.55
265602_at	putative retroelement pol polyprotei	---	---	-1.44	-0.52	-4.03	0.005405	0.024996	-2.55
257442_at	legumin-like protein contains a 11-S	---	AT2G28680	-1.59	-0.67	-4.03	0.005406	0.024996	-2.55
251194_at	putative protein	---	AT3G62920	1.69	0.75	4.03	0.005408	0.024996	-2.55
262491_at	putative SecA-type chloroplast prot	---	AT1G21650	1.43	0.51	4.03	0.005408	0.024996	-2.55
249320_at	disease resistance protein-like non-	---	AT5G40910	1.62	0.70	4.03	0.005436	0.025106	-2.55
253167_at	calmodulin-domain protein kinase C	CPK5	AT4G35310	-1.48	-0.57	-4.03	0.005437	0.025107	-2.55
253495_at	putative protein crp1 protein, Zea n	PGR3	AT4G31850	1.63	0.70	4.03	0.005449	0.025151	-2.56
251303_at	NAM-like protein no apical meristem	ANAC066	AT3G61910	-1.32	-0.40	-4.03	0.005452	0.025156	-2.56
262536_at	SOUL-like protein Similar to SOUL	---	AT1G17100	1.95	0.97	4.03	0.005454	0.025156	-2.56
265417_at	unknown protein	---	AT2G20920	1.74	0.80	4.02	0.005463	0.025189	-2.56
264805_at	unknown protein similar to putative	ATRX	AT1G08600	1.39	0.48	4.02	0.005463	0.025189	-2.56
246327_at	receptor-like serine/threonine kinas	---	AT1G16670	1.51	0.59	4.02	0.005468	0.025203	-2.56
264474_s_a	ribulose-bisphosphate carboxylase	RBCS1A	AT1G06820 ,	-1.29	-0.36	-4.02	0.005468	0.025203	-2.56
262977_at	transcription factor, putative similar	---	AT1G75490	-1.44	-0.53	-4.02	0.005474	0.025224	-2.56
251552_at	ribosomal protein L11 -like ribosom	---	AT3G58700	1.81	0.86	4.02	0.005475	0.025224	-2.56
265958_at	putative adenylate kinase ;support	ADK	AT2G37250	1.75	0.81	4.02	0.005479	0.025236	-2.56
258265_at	putative beta-1,3-glucanase precur	---	AT3G15800	-1.40	-0.49	-4.02	0.005486	0.025267	-2.56
250794_at	putative protein contains similarity t	---	AT5G05270	-1.36	-0.44	-4.02	0.005489	0.025267	-2.56
256862_at	dihydroxyacid dehydratase, putativ	---	AT3G23940	2.15	1.11	4.02	0.00549	0.025267	-2.56
252392_s_a	putative protein various predicted p	---	AT3G47890 ,	1.53	0.62	4.02	0.00549	0.025267	-2.56
258635_at	unknown protein	---	AT3G08020	1.30	0.38	4.02	0.005491	0.025267	-2.56
255924_at	unknown protein contains similarity	---	AT1G22170	-1.46	-0.55	-4.02	0.005492	0.025267	-2.56
257741_at	hypothetical protein predicted by ge	---	AT3G27340	1.58	0.66	4.02	0.0055	0.025299	-2.57
256957_at	hypothetical protein predicted by ge	---	AT3G13420	-1.38	-0.46	-4.02	0.005504	0.025313	-2.57
261122_at	ornithine carbamoyltransferase pred	OTC	AT1G75330	1.56	0.64	4.02	0.005508	0.025318	-2.57
259971_at	unknown protein similar to SPL1-re	---	AT1G76580	-1.61	-0.69	-4.02	0.00551	0.02532	-2.57
245851_at	putative protein HERC2 - Homo sa	KEG	AT5G13530	1.38	0.46	4.02	0.00551	0.02532	-2.57
260580_at	unknown protein	---	AT2G47390	1.30	0.38	4.02	0.005517	0.025345	-2.57

246524_at	Carboxylesterase-like protein stero	ATPCME	AT5G15860	1.61	0.69	4.02	0.005518	0.025346	-2.57
254722_at	putative protein predicted protein, A	---	AT4G13530	1.80	0.85	4.02	0.005522	0.025359	-2.57
257293_at	unknown protein similar to symbios	APG8H	AT3G15580	1.59	0.67	4.01	0.005532	0.025393	-2.57
261426_at	unknown protein contains similarity	---	AT1G18680	-1.33	-0.42	-4.01	0.005532	0.025393	-2.57
251578_at	putative protein contains alternative	---	AT3G58180	1.36	0.44	4.01	0.005538	0.025417	-2.57
262544_at	hypothetical protein predicted by ge	---	AT1G15420	1.45	0.54	4.01	0.005539	0.025417	-2.57
254858_at	putative protein other predicted pro	---	AT4G12070	1.37	0.46	4.01	0.005543	0.025427	-2.57
247374_at	topoisomerase-like protein ;suppor	---	AT5G63190	1.64	0.71	4.01	0.005544	0.025427	-2.57
252759_at	putative protein hypothetical protein	---	AT3G42630	1.29	0.37	4.01	0.005545	0.025428	-2.57
253402_at	HD-zip transcription factor (athb-8)	ATHB-8	AT4G32880	-1.69	-0.75	-4.01	0.005546	0.025428	-2.57
255601_at	putative protein (fragment) various	---	AT4G01030	1.43	0.52	4.01	0.005551	0.025447	-2.58
252672_at	hypothetical protein	---	AT3G44230	-1.31	-0.39	-4.01	0.005554	0.025454	-2.58
265309_at	putative myosin heavy chain	XIG	AT2G20290	-1.61	-0.69	-4.01	0.005558	0.025466	-2.58
259013_at	unknown protein similar to putative	emb1990	AT3G07430	1.36	0.45	4.01	0.005563	0.025482	-2.58
266601_at	hypothetical protein predicted by ge	---	AT2G46060	1.73	0.79	4.01	0.005564	0.025482	-2.58
245253_at	hydroperoxide lyase (HPOL) like pr	HPL1	AT4G15440	-5.13	-2.36	-4.01	0.005564	0.025482	-2.58
264442_at	hypothetical protein similar to lecith	---	AT1G27480	1.67	0.74	4.01	0.005568	0.025487	-2.58
261761_at	hypothetical protein predicted by ge	CENP-C	AT1G15660	1.35	0.44	4.01	0.005569	0.025488	-2.58
260377_at	unknown protein	---	AT1G73930	-1.29	-0.37	-4.01	0.00557	0.025488	-2.58
255522_at	putative GTP pyrophosphokinase s	RSH1	AT4G02260	1.41	0.49	4.01	0.005571	0.025488	-2.58
248258_at	putative protein contains similarity t	---	AT5G53400	1.89	0.92	4.01	0.005575	0.025494	-2.58
245913_at	predicted protein	---	AT5G19860	1.41	0.50	4.01	0.005575	0.025494	-2.58
253116_at	putative protein CRP1, Zea mays, g	---	AT4G35850	1.81	0.86	4.01	0.005576	0.025494	-2.58
261333_at	hypothetical protein predicted by ge	---	AT1G44910	1.62	0.69	4.01	0.005579	0.025503	-2.58
247221_at	Expressed protein ; supported by cI	LIP1	AT5G64813	-1.29	-0.37	-4.01	0.005584	0.025519	-2.58
251557_at	v-ATPase subunit D (vATPD) ; sup	---	AT3G58730	-1.37	-0.45	-4.00	0.005596	0.025565	-2.58
264685_at	endo-1,4-beta-glucanase, putative	KOR2	AT1G65610	-1.42	-0.51	-4.00	0.005628	0.025694	-2.59
248552_at	RNA-binding protein-like ; supporte	---	AT5G50250	1.38	0.46	4.00	0.00563	0.025694	-2.59
246536_at	putative protein KIAA0594 - Homo	---	AT5G15920	2.18	1.12	4.00	0.00563	0.025694	-2.59
245227_s_a	gunknown protein contains non-con	---	AT1G08410	1.64	0.71	4.00	0.005631	0.025694	-2.59
252899_at	putative protein leaf protein, Ipomo	---	AT4G39530	1.41	0.50	4.00	0.005634	0.025704	-2.59
247258_at	expressed protein similar to unknow	---	AT5G64816	1.45	0.53	4.00	0.005636	0.025706	-2.59
256961_at	transcription initiation factor TFIID-1	TBP1	AT3G13445	1.40	0.49	4.00	0.00564	0.025716	-2.59
257607_at	hypothetical protein predicted by ge	---	AT3G13880	1.29	0.37	4.00	0.00564	0.025716	-2.59
257050_at	putative protein phosphatase type 2	---	AT3G15260	1.51	0.60	4.00	0.005645	0.025732	-2.59

248681_at	pectate lyase non-consensus AG d	---	AT5G48900	-13.42	-3.75	-4.00	0.005649	0.025737	-2.59
260042_at	hypothetical protein C-term similar t	---	AT1G68820	1.71	0.77	4.00	0.005649	0.025737	-2.59
253293_at	Expressed protein ; supported by c	---	AT4G33905	-1.65	-0.73	-4.00	0.005657	0.025768	-2.60
247182_at	putative protein contains similarity t	HB25	AT5G65410	-1.45	-0.53	-3.99	0.005662	0.025784	-2.60
252193_at	R2R3-MYB transcription factor ; su	MYB77	AT3G50060	-2.40	-1.26	-3.99	0.005664	0.025789	-2.60
253474_at	hypothetical protein	---	AT4G32270	1.46	0.55	3.99	0.005667	0.025792	-2.60
260840_at	hypothetical protein similar to hypot	---	AT1G29050	1.49	0.57	3.99	0.005668	0.025792	-2.60
262968_at	hypothetical protein predicted by ge	---	AT1G75720	-1.37	-0.46	-3.99	0.005669	0.025792	-2.60
265220_at	histidine transport protein (PTR2-B)	PTR2	AT2G02040	1.31	0.39	3.99	0.005669	0.025792	-2.60
255718_at	unknown protein ;supported by full-	ATNSI	AT1G32070	1.39	0.47	3.99	0.005676	0.025818	-2.60
251883_at	ribosomal protein L17 -like protein	---	AT3G54210	-1.37	-0.45	-3.99	0.00568	0.025829	-2.60
249924_at	kinase associated protein phosphat	KAPP	AT5G19280	1.49	0.57	3.99	0.005681	0.025829	-2.60
247376_at	nucleotide diphosphate kinase Ia (e	NDPK2	AT5G63310	-1.33	-0.41	-3.99	0.005686	0.025844	-2.60
250400_at	protein phosphatase 2C -like prote	---	AT5G10740	1.68	0.75	3.99	0.005709	0.025943	-2.61
259441_at	cathepsin B-like cysteine protease,	---	AT1G02300	1.49	0.58	3.99	0.005711	0.025947	-2.61
251333_at	putative protein putative protein F2	---	AT3G61690	1.28	0.35	3.99	0.005713	0.025948	-2.61
249619_at	stelar K+ outward rectifying channe	GORK	AT5G37500	-1.61	-0.69	-3.99	0.005717	0.02596	-2.61
248540_at	FRO1 and FRO2-like protein	FRO8	AT5G50160	1.29	0.37	3.99	0.005718	0.02596	-2.61
262108_at	unknown protein similar to unknowr	---	AT1G02740	1.46	0.54	3.99	0.005728	0.025996	-2.61
248010_at	peroxisomal targeting signal type 1	PEX5	AT5G56290	1.32	0.40	3.98	0.005742	0.026057	-2.61
257054_at	Expressed protein ; supported by fu	MT3	AT3G15353	1.28	0.36	3.98	0.00575	0.026081	-2.61
260361_at	unknown protein ; supported by cDI	---	AT1G69360	-1.55	-0.63	-3.98	0.005751	0.026081	-2.61
259102_at	unknown protein similar to hin1 GB	NHL1	AT3G11660	1.47	0.56	3.98	0.005751	0.026081	-2.61
266495_at	cycloartenol synthase identical to S	CAS1	AT2G07050	-1.42	-0.51	-3.98	0.005761	0.026114	-2.62
249628_at	putative protein tropomyosin gene	---	AT5G37590	1.48	0.56	3.98	0.005761	0.026114	-2.62
252332_at	putative protein	---	AT3G48810	-1.55	-0.63	-3.98	0.005762	0.026116	-2.62
263100_at	hypothetical protein predicted by ge	AtPOT1a	AT2G05210	-1.55	-0.63	-3.98	0.005764	0.02612	-2.62
247320_at	photosystem I reaction centre subu	PSAN	AT5G64040	-1.36	-0.45	-3.98	0.005767	0.026123	-2.62
250562_at	putative protein	TOM5	AT5G08040	1.46	0.55	3.98	0.005767	0.026123	-2.62
246480_at	TCP-1 chaperonin-like protein t-co	---	AT5G16070	1.54	0.62	3.98	0.00577	0.026126	-2.62
262616_at	oxidoreductase, putative contains F	---	AT1G06620	-1.88	-0.91	-3.98	0.005771	0.026126	-2.62
261883_at	protein kinase, putative similar to re	---	AT1G80870	-1.40	-0.49	-3.98	0.005772	0.026127	-2.62
250786_at	putative protein strong similarity to u	SDN2	AT5G05540	1.55	0.63	3.98	0.005775	0.026138	-2.62
245348_at	trehalose-6-phosphate synthase like	ATTPS5	AT4G17770	1.57	0.65	3.98	0.005782	0.026162	-2.62
252105_at	protein phosphatase 2C-like protei	---	AT3G51470	-1.49	-0.57	-3.98	0.005792	0.026203	-2.62

261603_at	DNA binding protein ACBF, putativ	ATRBP47A	AT1G49600	1.90	0.93	3.98	0.005796	0.026216	-2.62
253138_at	ribosomal protein L11 homolog sev	MRPL11	AT4G35490	1.32	0.40	3.98	0.005802	0.026237	-2.62
261530_at	unknown protein contains Pfam pro	---	AT1G63460	1.52	0.60	3.97	0.005805	0.026247	-2.62
251255_at	putative protein early nodule-speci	---	AT3G62280	-1.31	-0.38	-3.97	0.00581	0.026263	-2.62
266526_at	putative tetracycline transporter pro	---	AT2G16980	-1.42	-0.50	-3.97	0.005811	0.026265	-2.63
246778_at	mevalonate kinase supported by fu	MK	AT5G27450	-1.43	-0.52	-3.97	0.005814	0.026273	-2.63
257792_at	TOM20, putative similar to TOM20	TOM20-3	AT3G27080	1.46	0.55	3.97	0.005817	0.02628	-2.63
260829_s_a	chloroplast thylakoidal processing	---	AT1G06870	1.58	0.66	3.97	0.005821	0.026285	-2.63
247691_at	heat shock protein 18 ;supported b	HSP18.2	AT5G59720	-1.39	-0.47	-3.97	0.005822	0.026285	-2.63
265627_at	Expressed protein ; supported by fu	---	AT2G27285	1.42	0.50	3.97	0.005824	0.026289	-2.63
257667_at	1,4-alpha-glucan branching enzym	EMB2729	AT3G20440	1.65	0.72	3.97	0.005826	0.026296	-2.63
247154_at	receptor protein kinase-like protein	HSL2	AT5G65710	-1.34	-0.42	-3.97	0.00583	0.026306	-2.63
263730_at	beta-glucosidase, putative similar t	BGLU4	AT1G60090	-1.33	-0.41	-3.97	0.005838	0.026337	-2.63
264783_at	putative calcium-dependent protein	PPCK1	AT1G08650	1.92	0.94	3.97	0.005851	0.02639	-2.63
253632_at	senescence-associated protein hor	TET9	AT4G30430	-1.28	-0.35	-3.97	0.005853	0.026393	-2.63
264141_at	hypothetical protein predicted by ge	---	AT1G79090	1.31	0.39	3.97	0.005854	0.026393	-2.63
260127_at	hypothetical protein similar to hypo	---	AT1G36320	1.57	0.65	3.97	0.005856	0.026393	-2.63
247209_at	putative protein contains similarity t	---	AT5G64830	1.35	0.44	3.97	0.005856	0.026393	-2.63
262985_s_a	60s ribosomal protein l27a. similar t	RPL27AB	AT1G23290	1.71	0.77	3.97	0.005858	0.026396	-2.63
248097_at	protein phosphatase X isoform 2 (g	PPX2	AT5G55260	1.46	0.54	3.97	0.005863	0.026411	-2.63
266344_at	hypothetical protein predicted by ge	---	AT2G01580	-1.34	-0.42	-3.97	0.005864	0.026411	-2.63
244945_at	cytochrome c biogenesis orf206	---	---	1.49	0.57	3.97	0.005865	0.026411	-2.63
254817_at	putative SWH1 protein SWH1 prote	ORP2B	AT4G12460	1.40	0.49	3.97	0.005866	0.026411	-2.64
256765_at	aminotransferase, putative similar t	POP2	AT3G22200	1.52	0.60	3.97	0.005875	0.026445	-2.64
262347_at	unknown protein similar to hypothet	---	AT1G64110	-1.42	-0.51	-3.96	0.005885	0.026487	-2.64
260004_at	unknown protein ;supported by full-	---	AT1G67860	1.75	0.80	3.96	0.005888	0.026493	-2.64
256468_at	ferredoxin, putative similar to GB:D	---	AT1G32550	-1.45	-0.53	-3.96	0.005889	0.026497	-2.64
260638_at	unknown protein similar to putative	---	AT1G62390	1.71	0.77	3.96	0.005897	0.026523	-2.64
262394_at	unknown protein	emb1273	AT1G49510	-1.66	-0.73	-3.96	0.005898	0.026525	-2.64
250651_at	cytochrome P450	CYP93D1	AT5G06900	-1.29	-0.36	-3.96	0.005899	0.026525	-2.64
262962_at	NADP-isocitrate dehydrogenase, p	ICDH	AT1G54340	1.28	0.35	3.96	0.005906	0.026549	-2.64
267435_at	30S ribosomal protein S5 ; support	---	AT2G33800	-1.32	-0.40	-3.96	0.005922	0.026608	-2.65
261113_at	hypothetical protein predicted by ge	---	AT1G75400	1.52	0.60	3.96	0.005922	0.026608	-2.65
251684_at	putative protein	---	AT3G56410	1.72	0.78	3.96	0.005922	0.026608	-2.65
248167_at	putative protein similar to unknown	---	AT5G54530	1.55	0.63	3.96	0.005929	0.026629	-2.65

253385_at	putative protein predicted protein, (	---	AT4G32930	1.33	0.41	3.96	0.005929	0.026629	-2.65
260651_at	hypothetical protein predicted by ge	---	AT1G32460	-1.35	-0.43	-3.96	0.005931	0.026629	-2.65
259834_at	H-protein promoter binding factor-2	---	AT1G69570	-1.81	-0.85	-3.96	0.005932	0.026629	-2.65
267539_at	putative RING zinc finger protein ;	---	AT2G42030	-1.42	-0.50	-3.96	0.00594	0.026659	-2.65
261098_at	unknown protein	---	AT1G62970	1.36	0.45	3.96	0.005947	0.026686	-2.65
258038_at	unknown protein ;supported by full-	---	AT3G21260	1.75	0.80	3.96	0.005948	0.026688	-2.65
246095_at	homeotic gene regulator - like prote	---	AT5G19310	1.66	0.73	3.96	0.00595	0.02669	-2.65
248620_at	putative protein similar to unknown	---	AT5G49320	-1.36	-0.44	-3.95	0.005952	0.026692	-2.65
254178_at	hypothetical protein	---	AT4G23880	3.70	1.89	3.95	0.005954	0.026692	-2.65
262157_at	unknown protein contains similarity	---	AT1G52670	-1.40	-0.49	-3.95	0.005954	0.026692	-2.65
261784_at	hypothetical protein predicted by ge	---	AT1G08220	1.34	0.43	3.95	0.005962	0.026721	-2.65
262380_at	threonine synthase, putative simila	---	AT1G72810	1.43	0.51	3.95	0.005981	0.026804	-2.66
260044_at	Expressed protein ; supported by fu	---	AT1G73655	-1.37	-0.46	-3.95	0.005993	0.026853	-2.66
249957_at	putative protein predicted proteins,	---	AT5G18900	1.47	0.56	3.95	0.006	0.026875	-2.66
262912_at	oligopeptide transporter, putative s	---	AT1G59740	-3.12	-1.64	-3.95	0.006001	0.026875	-2.66
252423_at	putative protein putative hydrolyse	---	AT3G47590	-1.34	-0.42	-3.95	0.006007	0.0269	-2.66
260579_at	putative cytochrome c oxidase Vc s	---	AT2G47380	1.40	0.48	3.95	0.006015	0.026929	-2.66
259657_at	phospholipase D alpha, putative si	PLDEPSIL(	AT1G55180	-1.34	-0.42	-3.95	0.006017	0.026932	-2.66
252080_at	putative protein polyphosphoinositi	---	AT3G51670	-1.71	-0.77	-3.94	0.006025	0.026965	-2.66
264364_at	unknown protein Similar to gb|U852	---	AT1G03330	1.32	0.40	3.94	0.006029	0.026976	-2.66
256227_at	unknown protein ; supported by cDI	---	AT1G56290	1.32	0.40	3.94	0.006035	0.026998	-2.67
252557_at	putative protein cim1 induced allerg	ATEXLA3	AT3G45960	-1.34	-0.42	-3.94	0.006041	0.027017	-2.67
260749_at	hypothetical protein predicted by ge	---	AT1G49110	-1.43	-0.51	-3.94	0.006046	0.027037	-2.67
266713_at	unknown protein	---	AT2G46760	-1.36	-0.44	-3.94	0.006055	0.027069	-2.67
257447_at	hypothetical protein predicted by ge	---	AT2G04230	1.27	0.35	3.94	0.006057	0.027071	-2.67
253950_at	putative dihydrolipoamide succinylt	---	AT4G26910	1.71	0.77	3.94	0.006058	0.027071	-2.67
262062_s_a	hypothetical protein	emb1427	AT1G20570	1.39	0.47	3.94	0.006059	0.027071	-2.67
258283_at	hypothetical protein predicted by ge	---	AT3G26750	1.38	0.47	3.94	0.00606	0.027071	-2.67
259950_at	hypothetical protein predicted by g	---	AT1G71410	-1.41	-0.49	-3.94	0.006061	0.027071	-2.67
262859_at	hypothetical protein predicted by ge	---	AT1G64790	1.38	0.46	3.94	0.006062	0.027072	-2.67
254721_at	hypothetical protein ;supported by f	SMAP1	AT4G13520	1.39	0.47	3.94	0.006064	0.027073	-2.67
250394_at	putative protein yllC, Enterococcus	---	AT5G10910	1.46	0.55	3.94	0.006065	0.027073	-2.67
249527_at	proline oxidase, mitochondrial pred	---	AT5G38710	1.60	0.68	3.94	0.006066	0.027073	-2.67
251926_at	60S RIBOSOMAL PROTEIN L36 h	---	AT3G53740	1.81	0.86	3.94	0.00607	0.027085	-2.67
247578_at	putative protein predicted protein, A	---	AT5G61300	-1.45	-0.53	-3.94	0.006072	0.027085	-2.67

259543_at	hypothetical protein ; supported by f	---	AT1G20580	1.43	0.52	3.94	0.006073	0.027085	-2.67
262071_at	unknown protein ;supported by full-	CF9	AT1G59510	1.43	0.52	3.94	0.006074	0.027086	-2.67
259992_at	putative heat shock transcription fa	HSFA8	AT1G67970	1.70	0.76	3.94	0.006076	0.027088	-2.67
245660_at	unknown protein	---	AT1G28240	-1.68	-0.75	-3.94	0.006077	0.027088	-2.67
262963_at	dihydrolipoamide S-acetyltransfera	---	AT1G54220	-1.32	-0.40	-3.94	0.006079	0.027092	-2.67
251985_at	thioredoxin -like protein cop c2, Co	---	AT3G53220	1.47	0.56	3.94	0.006083	0.027106	-2.67
252180_at	putative protein cyclin-dependent k	KRP2	AT3G50630	-1.63	-0.70	-3.94	0.00609	0.02713	-2.68
246305_at	putative protein protein At2g40060	---	AT3G51890	1.45	0.54	3.94	0.006094	0.027144	-2.68
253314_at	hypothetical protein	---	AT4G33890	1.31	0.39	3.93	0.006103	0.027179	-2.68
257190_at	30S ribosomal protein S10, putativ	---	AT3G13120	-1.63	-0.70	-3.93	0.006119	0.027244	-2.68
260055_at	unknown protein similar to putative	---	AT1G78150	1.32	0.40	3.93	0.006127	0.027268	-2.68
246954_at	unknown protein ; supported by cD	---	AT5G04830	-1.31	-0.39	-3.93	0.006128	0.027268	-2.68
258532_at	ribosomal protein L29, putative sim	---	AT3G06700	2.23	1.15	3.93	0.006132	0.027281	-2.68
259178_at	unknown protein similar to hypothe	RGLG1	AT3G01650	1.50	0.59	3.93	0.006135	0.027291	-2.68
256484_at	PPR-repeat protein contains multipl	---	AT1G31430	1.41	0.50	3.93	0.00614	0.027306	-2.68
258998_at	putative adenylate kinase similar to	---	AT3G01820	1.47	0.55	3.93	0.006141	0.027306	-2.68
247193_at	putative protein strong similarity to u	---	AT5G65380	-1.54	-0.63	-3.93	0.006147	0.027325	-2.69
248786_at	unknown protein	---	AT5G47410	1.74	0.80	3.93	0.00615	0.027326	-2.69
248883_at	putative protein strong similarity to u	---	AT5G46190	-1.34	-0.42	-3.93	0.00615	0.027326	-2.69
265377_at	putative beta-1,3-glucanase	---	AT2G05790	-4.85	-2.28	-3.93	0.006151	0.027326	-2.69
248589_at	von Hippel-Lindau binding protein (V	PDF3	AT5G49510	1.33	0.41	3.93	0.006152	0.027326	-2.69
245790_at	glycerol-3-phosphate acyltransferas	ATS1	AT1G32200	-1.44	-0.53	-3.93	0.006155	0.027334	-2.69
264330_at	multi-drug resistance protein Strong	ATMRP5	AT1G04120	-1.35	-0.44	-3.93	0.006168	0.027384	-2.69
264661_at	hypothetical protein Similar to Nicot	---	AT1G09950	-1.75	-0.81	-3.93	0.006168	0.027384	-2.69
254052_at	UMP/CMP kinase like protein UMP	---	AT4G25280	-1.44	-0.53	-3.93	0.006173	0.0274	-2.69
253861_at	putative protein MSP1 protein, Sac	---	AT4G27680	1.36	0.44	3.92	0.006178	0.027408	-2.69
263466_at	hypothetical protein predicted by ge	---	AT2G31740	1.31	0.39	3.92	0.006179	0.027408	-2.69
257962_at	hypothetical protein predicted by ge	---	AT3G19790	-1.68	-0.75	-3.92	0.006185	0.027429	-2.69
258946_at	hypothetical protein predicted by ge	---	AT3G10650	1.46	0.54	3.92	0.006186	0.027429	-2.69
266880_at	hypothetical protein predicted by ge	---	AT2G44770	-1.44	-0.53	-3.92	0.006191	0.027447	-2.69
260526_at	putative WD-40 repeat protein	---	AT2G47410	-1.34	-0.42	-3.92	0.006197	0.027468	-2.69
245509_at	hypothetical protein	---	AT4G15730	1.45	0.54	3.92	0.006198	0.027468	-2.69
252108_at	putative protein various predicted p	---	AT3G51530	1.36	0.44	3.92	0.006199	0.027468	-2.69
258136_at	hypothetical protein predicted by ge	RSY3	AT3G24560	1.43	0.51	3.92	0.006202	0.027474	-2.70
255005_at	putative protein putative proteins Ar	---	AT4G09990	-1.42	-0.50	-3.92	0.006209	0.027499	-2.70

249231_at	putative protein similar to unknown	ABIL4	AT5G42030	1.41	0.49	3.92	0.006211	0.027503	-2.70
248941_s_a	putative protein similar to unknown	---	AT5G45460	-1.47	-0.55	-3.92	0.006213	0.027507	-2.70
265814_at	hypothetical protein similar to hypo	---	AT2G17930	1.37	0.45	3.92	0.006216	0.027517	-2.70
248547_at	putative protein contains similarity t	EMB1006	AT5G50280	1.68	0.75	3.92	0.00622	0.027527	-2.70
266792_at	putative sucrose/H+ symporter	SUT2	AT2G02860	-2.08	-1.06	-3.92	0.006227	0.027551	-2.70
253264_at	protein kinase - like protein protein	OST1	AT4G33950	-1.31	-0.39	-3.92	0.006232	0.02757	-2.70
248152_at	DNA repair and meiosis protein Mre	MRE11	AT5G54260	-1.55	-0.63	-3.92	0.006243	0.027615	-2.70
261938_at	hypothetical protein predicted by ge	---	AT1G22510	-1.88	-0.91	-3.92	0.006248	0.027632	-2.70
258090_at	putative 60S ribosomal protein L18/	---	AT3G14600	1.54	0.62	3.92	0.00625	0.027634	-2.70
263716_at	26S proteasome regulatory subunit	RPN1A	AT2G20580	1.29	0.37	3.91	0.006255	0.027647	-2.70
265887_at	biotin holocarboxylase synthetase i	HCS1	AT2G25710	1.46	0.54	3.91	0.006272	0.027715	-2.71
254738_at	RNA-binding protein like	AT4G1386	---	-1.39	-0.47	-3.91	0.006283	0.027758	-2.71
260875_at	hypothetical protein predicted by ge	SKP2A	AT1G21410	1.88	0.91	3.91	0.006285	0.027763	-2.71
245155_at	putative protein similarity to predict	---	AT5G12470	-1.61	-0.69	-3.91	0.006287	0.027766	-2.71
252805_at	putative protein	---	AT3G42380	-1.31	-0.39	-3.91	0.006296	0.027793	-2.71
258149_at	hypothetical protein contains Pfam	EMB1270	AT3G18110	1.46	0.55	3.91	0.006296	0.027793	-2.71
260332_at	hypothetical protein predicted by ge	---	AT1G70470	-1.27	-0.35	-3.91	0.006299	0.0278	-2.71
259063_at	putative 5B-anther specific protein s	---	AT3G07450	-1.28	-0.36	-3.91	0.006303	0.027812	-2.71
251319_at	putative protein POSSIBLE APOSI	---	AT3G61610	-1.56	-0.64	-3.91	0.006308	0.02783	-2.71
249181_at	putative protein contains similarity t	---	AT5G42920	1.38	0.46	3.91	0.00631	0.027832	-2.71
249842_at	putative protein contains similarity t	---	AT5G23550	1.32	0.40	3.91	0.006311	0.027832	-2.71
260053_at	hypothetical protein similar to unkno	---	AT1G78120	-1.48	-0.57	-3.91	0.006316	0.027849	-2.71
264046_at	putative non-LTR retroelement reve	---	AT2G22440	-1.37	-0.45	-3.91	0.006318	0.02785	-2.72
261036_at	developmentally regulated GTP-bir	ATDRG1	AT1G17470	1.31	0.39	3.91	0.006318	0.02785	-2.72
254776_at	3-hydroxyisobutyryl-coenzyme A h	---	AT4G13360	1.28	0.35	3.91	0.00632	0.027851	-2.72
253969_at	putative protein hypothetical protei	CSN6B	AT4G26430	1.73	0.79	3.90	0.006338	0.027922	-2.72
246460_at	cellulose synthase catalytic subuni	CSLD2	AT5G16910	-1.68	-0.75	-3.90	0.00634	0.027922	-2.72
258573_at	hypothetical protein contains Pfam	PTAC3	AT3G04260	1.30	0.38	3.90	0.006346	0.027937	-2.72
254617_s_a	ribosomal protein L11, cytosolic ;s	RPL16B	AT4G18730	1.63	0.70	3.90	0.006347	0.027937	-2.72
248388_at	putative protein contains similarity t	---	AT5G51980	1.42	0.50	3.90	0.006347	0.027937	-2.72
256394_at	unknown protein contains non-cons	---	AT3G06290	1.33	0.42	3.90	0.006349	0.02794	-2.72
258827_at	hypothetical protein predicted by ge	---	AT3G07150	1.55	0.63	3.90	0.006353	0.02795	-2.72
251076_at	putative protein putative protein At2	---	AT5G01970	-1.64	-0.72	-3.90	0.006356	0.027957	-2.72
260543_at	putative membrane transporter	INT1	AT2G43330	-1.63	-0.70	-3.90	0.006359	0.027963	-2.72
253285_at	fatty acid elongase - like protein fat	KCS16	AT4G34250	-1.83	-0.87	-3.90	0.006363	0.027976	-2.72

246307_at	putative nuclear DNA-binding prote	ATG2	AT3G51800	1.62	0.69	3.90	0.006366	0.027981	-2.72
251492_at	putative protein CGI-136 protein - I	TXR1	AT3G59280	1.29	0.36	3.90	0.006367	0.027981	-2.72
256575_at	dTDP-glucose 4,6-dehydratase, pu	RHM3	AT3G14790	-1.55	-0.64	-3.90	0.00638	0.028034	-2.73
260416_at	putative cullin similar to cullin 3 GB:	CUL3B	AT1G69670	1.35	0.43	3.90	0.006389	0.028067	-2.73
266707_at	hypothetical protein predicted by ge	---	AT2G03310	1.34	0.42	3.90	0.006394	0.028085	-2.73
262645_at	unknown protein similar to elongatio	SCO1	AT1G62750	1.49	0.57	3.90	0.006397	0.028094	-2.73
255296_at	hypothetical protein	---	AT4G04790	1.84	0.88	3.90	0.006399	0.028096	-2.73
266059_at	predicted protein ;supported by cDN	---	AT2G40765	1.87	0.90	3.90	0.006406	0.02812	-2.73
262775_at	unknown protein	---	AT1G13000	1.33	0.41	3.90	0.006411	0.028134	-2.73
247172_at	anthocyanidin-3-glucoside rhamnos	---	AT5G65550	-1.33	-0.41	-3.89	0.006414	0.028141	-2.73
248946_at	putative protein similar to unknown	---	AT5G45530	-1.31	-0.39	-3.89	0.00642	0.028153	-2.73
256311_at	auxin response factor 6 (ARF6) ide	ARF6	AT1G30330	-1.72	-0.78	-3.89	0.006422	0.028153	-2.73
251861_at	putative protein GATA transcription	BME3	AT3G54810	-2.68	-1.42	-3.89	0.006422	0.028153	-2.73
252294_at	60S ribosomal protein L13, BBC1 p	ATBBC1	AT3G49010	2.13	1.09	3.89	0.006423	0.028153	-2.73
259204_s_a	hypothetical protein predicted by ge	---	---	-1.33	-0.41	-3.89	0.006424	0.028153	-2.73
263647_at	unknown protein	---	AT2G04690	1.51	0.60	3.89	0.006427	0.028161	-2.73
247218_at	asparagine synthetase (gb|AAC728	ASN2	AT5G65010	3.32	1.73	3.89	0.00643	0.028167	-2.73
262600_at	unknown protein ESTs gb|H37032,	MBD10	AT1G15340	1.56	0.64	3.89	0.006435	0.028185	-2.74
253232_at	hypothetical protein ;supported by f	TAF8	AT4G34340	1.40	0.48	3.89	0.006444	0.028217	-2.74
261220_at	ER lumen protein-retaining recepto	---	AT1G19970	-2.05	-1.04	-3.89	0.006446	0.028217	-2.74
249295_at	putative protein contains similarity t	---	AT5G41280	-1.30	-0.37	-3.89	0.006446	0.028217	-2.74
260208_s_a	unknown protein similar to Ca+2-bin	---	AT1G70670	-1.84	-0.88	-3.89	0.006452	0.028232	-2.74
263134_at	dTDP-glucose 4,6-dehydratase, pu	RHM1	AT1G78570	-1.56	-0.65	-3.89	0.006455	0.028232	-2.74
253753_at	glycine-rich protein like glycine-rich	---	AT4G29030	-1.39	-0.47	-3.89	0.006455	0.028232	-2.74
261163_x_a	auxin response factor, putative sim	ARF21	AT1G34410	-1.28	-0.35	-3.89	0.006473	0.028303	-2.74
249906_at	unknown protein	---	AT5G22730	1.30	0.38	3.89	0.006475	0.028303	-2.74
247849_at	putative protein fibrinogen-binding	---	AT5G58130	1.48	0.56	3.89	0.006482	0.02833	-2.74
261012_at	CLE9, putative CLAVATA3/ESR-R	CLE9	AT1G26600	-1.27	-0.34	-3.89	0.00649	0.028357	-2.74
260768_at	Expressed protein ; supported by fu	---	AT1G49245	1.37	0.46	3.88	0.006503	0.028412	-2.75
246527_at	ribosomal protein-like 40S RIBOSO	---	AT5G15750	2.08	1.06	3.88	0.006517	0.028468	-2.75
257413_at	putative RNA-binding protein conta	---	AT1G22910	-1.60	-0.68	-3.88	0.006523	0.028477	-2.75
254049_at	putative ribosomal protein S10 40S	---	AT4G25740	1.80	0.85	3.88	0.006524	0.028477	-2.75
247041_at	floral homeotic protein apetala2-like	TOE3	AT5G67180	-1.42	-0.50	-3.88	0.006526	0.028477	-2.75
247400_at	putative protein ;supported by full-l	---	AT5G62840	-1.54	-0.63	-3.88	0.006526	0.028477	-2.75
257864_at	hypothetical protein predicted by ge	---	AT3G17740	1.55	0.63	3.88	0.006528	0.028477	-2.75

259036_at	putative laccase similar to laccase (	LAC7	AT3G09220	-1.31	-0.38	-3.88	0.006529	0.028477	-2.75
264987_at	unknown protein ; supported by cDI	---	AT1G27030	-1.99	-1.00	-3.88	0.00653	0.028477	-2.75
249138_at	putative protein contains similarity t	WPP1	AT5G43070	-1.35	-0.43	-3.88	0.00653	0.028477	-2.75
267532_at	unknown protein ;supported by full-	---	AT2G42040	-1.70	-0.77	-3.88	0.006531	0.028477	-2.75
247745_at	copper transport protein	COPT1	AT5G59030	1.29	0.37	3.88	0.006535	0.02849	-2.75
245866_s_a	unknown protein	---	AT1G57980	-3.55	-1.83	-3.88	0.006559	0.028589	-2.76
250914_at	myb -like protein telomeric DNA bir	TRFL10	AT5G03780	1.41	0.50	3.88	0.006561	0.028589	-2.76
265898_at	hypothetical protein predicted by ge	---	AT2G25690	1.72	0.79	3.88	0.006562	0.028589	-2.76
246852_at	putative protein various predicted b	---	AT5G26880	1.33	0.41	3.88	0.006562	0.028589	-2.76
250481_at	putative protein	---	AT5G10310	1.36	0.44	3.88	0.006572	0.028626	-2.76
263045_at	hypothetical protein predicted by ge	---	AT2G05370	-1.36	-0.44	-3.88	0.006575	0.028631	-2.76
262484_at	putative transcriptional regulatory p	ATSWI3C	AT1G21700	-1.26	-0.33	-3.87	0.006578	0.02864	-2.76
265858_at	putative ribophorin I	---	AT2G01720	1.45	0.54	3.87	0.006587	0.028658	-2.76
265005_at	Expressed protein ; supported by fu	---	AT1G61667	-1.74	-0.80	-3.87	0.006588	0.028658	-2.76
252295_at	signal recognition particle subunit 9	---	AT3G49100	1.54	0.63	3.87	0.006589	0.028658	-2.76
250874_at	putative protein	---	AT5G04010	-1.41	-0.49	-3.87	0.00659	0.028658	-2.76
245674_at	chitinase, putative similar to chitina	---	AT1G56680	-1.32	-0.40	-3.87	0.006592	0.028658	-2.76
259685_at	unknown protein	AtPP2-A11	AT1G63090	-1.70	-0.77	-3.87	0.006592	0.028658	-2.76
258851_at	glutathione S-transferase identical t	ATGSTF11	AT3G03190	-2.06	-1.04	-3.87	0.006592	0.028658	-2.76
255511_at	Expressed protein ; supported by cl	PIT1	AT4G02075	1.91	0.93	3.87	0.006602	0.028692	-2.76
260799_at	UDP-glucose glucosyltransferase,	AtUGT85A	AT1G78270	1.44	0.53	3.87	0.006603	0.028692	-2.76
246232_at	hypothetical protein	EMB2754	AT4G36630	1.35	0.43	3.87	0.00663	0.028804	-2.77
248790_at	membrane channel protein-like; aqu	TIP2;3	AT5G47450	-1.58	-0.66	-3.87	0.006634	0.028816	-2.77
249430_at	GTP-binding protein-like GTP-bind	---	AT5G39900	1.60	0.68	3.87	0.006635	0.028816	-2.77
255458_at	small nuclear riboprotein Sm-D1-lik	---	AT4G02840	1.31	0.39	3.87	0.006636	0.028816	-2.77
264491_at	hypothetical protein predicted by ge	---	AT1G27410	1.34	0.42	3.87	0.006642	0.028837	-2.77
255582_at	putative DNA-binding protein simila	NGA4	AT4G01500	-1.32	-0.40	-3.87	0.006645	0.028845	-2.77
250729_at	ubiquitin activating enzyme 2 (UBA2	UBA 2	AT5G06460	1.36	0.44	3.87	0.006655	0.028882	-2.77
267206_at	putative dioxygenase	---	AT2G30830	-1.35	-0.44	-3.86	0.006662	0.028908	-2.77
257516_at	unknown protein	ACR4	AT1G69040	-1.64	-0.71	-3.86	0.006664	0.028911	-2.77
266980_at	60S ribosomal protein L35 ;suppor	---	AT2G39390	1.79	0.84	3.86	0.006669	0.028923	-2.77
251393_at	putative protein symbiosis-related p	ATG8G	AT3G60640	1.57	0.65	3.86	0.00667	0.028923	-2.77
252960_at	hypothetical protein	---	AT4G38750	1.72	0.78	3.86	0.006672	0.028926	-2.77
246758_at	60S ribosomal protein - like 60S rib	---	AT5G27850	1.74	0.80	3.86	0.006679	0.028949	-2.78
AFFX-Athal-	Arabidopsis thaliana /REF=M64116	GAPC1	---	1.87	0.90	3.86	0.006679	0.028949	-2.78

249523_at	cytochrome b-561 (gb|AAD45585.1	ACYB-1	AT5G38630	-1.36	-0.44	-3.86	0.006684	0.028962	-2.78
255656_at	putative protein (fragment) DNA-bi	---	AT4G00990	1.42	0.50	3.86	0.006685	0.028962	-2.78
251645_at	putative protein polygalacturonase	---	AT3G57790	-1.40	-0.49	-3.86	0.00669	0.028974	-2.78
252895_at	hypothetical protein	---	AT4G39450	1.33	0.41	3.86	0.006692	0.028974	-2.78
245120_at	hypothetical protein predicted by ge	HOS1	AT2G39810	1.45	0.53	3.86	0.006692	0.028974	-2.78
245967_at	proline-rich protein extensins	---	AT5G19800	-1.34	-0.42	-3.86	0.006693	0.028974	-2.78
249327_at	anion channel protein (gb|AAC0574	ATCLC-A	AT5G40890	-1.91	-0.93	-3.86	0.006699	0.028996	-2.78
247460_at	AtOXA1 ;supported by full-length c	OXA1	AT5G62050	1.26	0.33	3.86	0.006703	0.029006	-2.78
245844_at	hypothetical protein predicted by ge	---	AT1G26170	1.37	0.45	3.86	0.006708	0.029021	-2.78
247098_at	GTP-binding protein-like	---	AT5G66470	1.64	0.72	3.86	0.006709	0.029022	-2.78
247077_at	putative protein similar to unknown	---	AT5G66420	-1.44	-0.52	-3.86	0.006716	0.029048	-2.78
261175_at	unknown protein	---	AT1G04800	-1.38	-0.46	-3.86	0.00672	0.029058	-2.78
263004_at	hypothetical protein contains simila	ATNEK1	AT1G54510	1.40	0.49	3.86	0.006722	0.029061	-2.78
246910_at	exonuclease - like protein ribonucle	---	AT5G25800	1.60	0.68	3.86	0.006724	0.029063	-2.78
246434_at	root cap 1 (RCP1) ;supported by fu	RCP1	AT5G17520	-1.52	-0.60	-3.86	0.006725	0.029063	-2.78
259672_at	DNA-directed RNA polymerase ide	---	AT1G68990	2.17	1.12	3.86	0.006726	0.029063	-2.78
253029_at	hypothetical protein	FRS9	AT4G38170	1.92	0.94	3.86	0.006736	0.029099	-2.78
252484_at	glucuronosyl transferase-like prote	---	AT3G46690	1.45	0.54	3.86	0.006741	0.029118	-2.79
245877_at	unknown protein	---	AT1G26220	-1.53	-0.61	-3.85	0.006754	0.029163	-2.79
252015_at	putative protein growth-regulating f	AtGRF4	AT3G52910	1.27	0.34	3.85	0.006754	0.029163	-2.79
248059_at	putative protein similar to unknown	TRN1	AT5G55540	-1.80	-0.85	-3.85	0.006758	0.029174	-2.79
246114_at	seed imbitition protein-like seed im	DIN10	AT5G20250	-1.69	-0.75	-3.85	0.006762	0.02918	-2.79
251917_at	putative protein	---	AT3G53970	1.32	0.40	3.85	0.006782	0.02926	-2.79
258354_at	putative zinc finger protein similar to	---	AT3G14320	-1.29	-0.37	-3.85	0.006785	0.029269	-2.79
255221_at	putative protein various predicted p	---	AT4G05150	1.41	0.50	3.85	0.00679	0.029282	-2.79
245331_at	hypothetical protein ;supported by f	---	AT4G14410	1.34	0.42	3.85	0.006794	0.029292	-2.79
253463_at	Expressed protein ; supported by fu	---	AT4G32105	-1.35	-0.43	-3.85	0.006795	0.029292	-2.79
245771_at	hypothetical protein predicted by ge	---	AT1G30250	-5.08	-2.35	-3.85	0.006807	0.029329	-2.80
257374_at	hypothetical protein predicted by ge	---	AT2G43280	1.54	0.62	3.85	0.006814	0.029354	-2.80
248981_at	regulatory protein NPR1-like; transc	NPR3	AT5G45110	-1.36	-0.45	-3.85	0.006816	0.029355	-2.80
266894_at	hypothetical protein predicted by ge	---	AT2G26050	-1.28	-0.35	-3.85	0.006818	0.029355	-2.80
252764_at	nucleic acid binding protein-like nu	AL3	AT3G42790	1.35	0.44	3.85	0.006818	0.029355	-2.80
254192_at	acyl-CoA synthetase - like protein a	---	AT4G23850	-1.36	-0.45	-3.85	0.006823	0.029371	-2.80
253569_at	receptor kinase - like protein recep	---	AT4G31250	-1.45	-0.54	-3.84	0.006831	0.029397	-2.80
260178_at	hypothetical protein similar to unkno	---	AT1G70720	-1.37	-0.46	-3.84	0.006832	0.029397	-2.80

250303_at	putative protein similar to unknown	---	AT5G12100	1.77	0.82	3.84	0.006842	0.029433	-2.80
259936_at	putative UDP-glucose:glycoprotein	EBS1	AT1G71220	1.32	0.40	3.84	0.006845	0.029443	-2.80
244940_at	ribosomal protein S12 (trans-splice	---	---	-1.46	-0.54	-3.84	0.006861	0.029507	-2.80
250937_at	putative protein various predicted p	---	AT5G03230	1.71	0.77	3.84	0.006863	0.029509	-2.80
257856_at	hypothetical protein predicted by ge	---	AT3G12930	-1.29	-0.37	-3.84	0.006899	0.029654	-2.81
247367_at	oxygen-independent coproporphyrin	---	AT5G63290	1.66	0.73	3.84	0.006902	0.029656	-2.81
263038_at	conserved hypothetical protein prot	---	AT1G23270	-1.33	-0.41	-3.84	0.006902	0.029656	-2.81
259321_at	unknown protein	GAUT13	AT3G01040	-1.51	-0.60	-3.84	0.006904	0.029658	-2.81
247676_at	putative protein RNaseP protein p3	---	AT5G59980	1.72	0.78	3.84	0.00691	0.029678	-2.81
246390_at	1-aminocyclopropane-1-carboxylat	---	AT1G77330	-1.52	-0.60	-3.83	0.006916	0.029698	-2.81
255672_at	hypothetical protein ; supported by	EDA8	AT4G00310	-1.41	-0.49	-3.83	0.006919	0.029705	-2.81
259762_at	hypothetical protein predicted by ge	---	AT1G77600	1.40	0.49	3.83	0.006931	0.02975	-2.82
247043_at	protein kinase, 41K (EC 2.7.1.-) (pi	SNRK2.3	AT5G66880	1.65	0.72	3.83	0.006932	0.02975	-2.82
263311_at	hypothetical protein predicted by ge	---	AT2G10340	-1.41	-0.49	-3.83	0.006934	0.02975	-2.82
253189_at	putative protein	---	AT4G35335	-1.61	-0.69	-3.83	0.006935	0.02975	-2.82
254368_at	DNA-directed RNA polymerase (EC	NRPB2	AT4G21710	1.34	0.42	3.83	0.006937	0.029752	-2.82
250388_at	putative protein various predicted p	---	AT5G11310	2.01	1.01	3.83	0.006946	0.029781	-2.82
266119_at	protease inhibitor II identical to GB	LCR69	AT2G02100	-3.02	-1.60	-3.83	0.006949	0.029789	-2.82
264336_at	hypothetical protein contains similar	---	AT1G70360	1.42	0.51	3.83	0.00695	0.029789	-2.82
267237_s_a	unknown protein ;supported by full-	---	AT2G44040	-1.96	-0.97	-3.83	0.006954	0.029797	-2.82
251854_at	putative protein various predicted p	---	AT3G54800	-1.33	-0.41	-3.83	0.006955	0.029797	-2.82
266125_at	putative GATA-type zinc finger tran	---	AT2G45050	-1.46	-0.55	-3.83	0.006958	0.029801	-2.82
256912_at	hypothetical protein predicted by ge	---	AT3G23850	-1.36	-0.44	-3.83	0.006958	0.029801	-2.82
267063_at	hypothetical protein predicted by ge	---	AT2G41120	1.51	0.60	3.83	0.006963	0.029817	-2.82
245345_at	proteinase like protein ;supported b	---	AT4G16640	-1.32	-0.40	-3.83	0.006965	0.029817	-2.82
265671_at	40S ribosomal protein S12 ;suppor	---	AT2G32060	1.97	0.98	3.83	0.006979	0.029867	-2.82
266950_at	expressed protein supported by full-	---	AT2G18910	-1.60	-0.68	-3.83	0.006979	0.029867	-2.82
255585_at	putative NAM-like protein	anac069	AT4G01550	1.62	0.69	3.83	0.006998	0.029941	-2.83
254948_at	putative protein various predicted p	---	AT4G11000	-1.40	-0.48	-3.83	0.007003	0.029956	-2.83
259786_at	lipase/hydrolase, putative contains	---	AT1G29660	-13.89	-3.80	-3.82	0.007005	0.029956	-2.83
252272_at	receptor protein kinase - like protei	BAM2	AT3G49670	-1.92	-0.94	-3.82	0.007006	0.029956	-2.83
264192_at	hypothetical protein predicted by ge	ATATG18H	AT1G54710	1.28	0.35	3.82	0.007007	0.029956	-2.83
246769_at	hypothetical protein	---	AT5G27440	1.34	0.42	3.82	0.007008	0.029956	-2.83
253105_at	putative protein zinc finger protein,	---	AT4G35840	1.57	0.65	3.82	0.007009	0.029956	-2.83
255267_at	putative protein LpxD gene, Brucel	---	AT4G05210	-1.27	-0.35	-3.82	0.007012	0.029961	-2.83

244997_at	RNA polymerase beta' subunit-2	---	---	-1.38	-0.46	-3.82	0.007013	0.029962	-2.83
258151_at	beta-glucosidase, putative similar t	BGLU44	AT3G18080	1.82	0.87	3.82	0.007019	0.029983	-2.83
257522_at	Yippee-like protein similar to Yippee	---	AT3G08990	-1.30	-0.38	-3.82	0.007025	0.030003	-2.83
261133_at	DYW7 protein identical to DYW7 p	---	AT1G19715	-1.62	-0.69	-3.82	0.007041	0.030065	-2.83
253469_at	putative protein gene C42D8.3, Ca	ATPANK2	AT4G32180	1.35	0.44	3.82	0.007042	0.030065	-2.83
255488_at	putative serine/threonine protein kin	---	AT4G02630	1.49	0.58	3.82	0.007046	0.030071	-2.83
265205_at	putative poly(A) binding protein	PAB7	AT2G36660	-1.33	-0.41	-3.82	0.007058	0.030116	-2.83
245092_at	putative TGACG-sequence-specific	BZIP17	AT2G40950	1.44	0.52	3.82	0.007066	0.030142	-2.84
264637_at	allyl alcohol dehydrogenase, putati	---	AT1G65560	1.59	0.67	3.82	0.007067	0.030142	-2.84
252256_at	PRE-MRNA SPLICING FACTOR S	SRp34a	AT3G49430	1.47	0.55	3.82	0.007069	0.030143	-2.84
259124_at	floral homeotic protein AGL4 identi	ספט-02	AT3G02310	-1.27	-0.35	-3.82	0.007075	0.030165	-2.84
257381_at	unknown protein	---	AT2G37950	-1.76	-0.82	-3.82	0.007085	0.0302	-2.84
258914_at	hypothetical protein predicted by ge	AGP27	AT3G06360	-1.30	-0.37	-3.82	0.007088	0.030211	-2.84
251028_at	putative protein putative hydrolase	---	AT5G02230	1.42	0.51	3.81	0.007095	0.03023	-2.84
244999_at	RNA polymerase beta subunit	---	---	-1.64	-0.71	-3.81	0.007096	0.03023	-2.84
251669_at	putative protein hypothetical protei	---	AT3G57180	1.87	0.90	3.81	0.007097	0.030231	-2.84
261256_at	disease resistance protein RTM1 id	RTM1	AT1G05760	-1.48	-0.56	-3.81	0.007101	0.030244	-2.84
260525_at	putative pre-mRNA splicing factor R	---	AT2G47250	1.31	0.39	3.81	0.007107	0.030263	-2.84
249224_at	mitochondrial carrier protein-like	---	AT5G42130	1.29	0.37	3.81	0.007111	0.030275	-2.84
257949_at	putative UDP-glucose glucosyltrans	UGT71B1	AT3G21750	-1.57	-0.65	-3.81	0.007113	0.030278	-2.84
249192_at	brain and reproductive organ-expre	---	AT5G42470	1.28	0.35	3.81	0.007117	0.030285	-2.84
256874_at	cytochrome P450, putative contain	CYP71B36	AT3G26320	1.41	0.49	3.81	0.007118	0.030285	-2.84
251851_at	structural maintenance of chromos	TTN8	AT3G54670	1.37	0.45	3.81	0.007119	0.030285	-2.84
253539_at	putative protein RING-finger protei	---	AT4G31450	1.39	0.48	3.81	0.007134	0.030341	-2.85
266310_at	putative PCI domain protein protea	FUS12	AT2G26990	1.39	0.48	3.81	0.007138	0.030351	-2.85
251322_at	cysteine synthase AtcysC1 ;suppor	CYSC1	AT3G61440	2.12	1.09	3.81	0.007139	0.030351	-2.85
257012_at	RNA-binding protein, putative simil	TEL1	AT3G26120	-1.31	-0.39	-3.81	0.007149	0.03039	-2.85
246558_at	putative protein IDN3-B - Homo sa	EMB2773	AT5G15540	1.44	0.52	3.81	0.007151	0.030391	-2.85
255857_at	unknown protein ;supported by full-	ABA4	AT1G67080	1.29	0.37	3.81	0.007155	0.030404	-2.85
248737_at	unknown protein	---	AT5G48120	1.56	0.64	3.81	0.007163	0.030431	-2.85
246807_at	ion channel - like protein ligand gat	GLR2.1	AT5G27100	-1.54	-0.62	-3.81	0.007167	0.030442	-2.85
248646_at	unknown protein ;supported by full-	---	AT5G49100	-1.86	-0.90	-3.81	0.007171	0.030453	-2.85
248111_at	wax synthase-like protein	---	AT5G55330	-1.32	-0.40	-3.81	0.007172	0.030453	-2.85
249054_at	unknown protein	---	AT5G02870	1.43	0.51	3.81	0.007178	0.030473	-2.85
255907_at	homeobox protein, putative similar	HDG12	AT1G17920	-1.31	-0.39	-3.80	0.007189	0.030515	-2.85

250912_at	histone deacetylase -like protein hi	HD2C	AT5G03740	1.83	0.87	3.80	0.007195	0.03053	-2.86
254430_at	reticuline oxidase - like protein retic	---	AT4G20820	2.07	1.05	3.80	0.007195	0.03053	-2.86
262710_at	unknown protein ESTs gb|T04357 a	---	AT1G16210	1.31	0.39	3.80	0.007199	0.030541	-2.86
254421_at	VP1 like protein transcription activa	VAL3	AT4G21550	1.55	0.64	3.80	0.007202	0.030548	-2.86
253447_at	hypothetical protein	---	AT4G32630	-1.28	-0.35	-3.80	0.007208	0.030567	-2.86
262504_at	putative protein disulfide isomerase	ATPDIL1-1	AT1G21750	1.61	0.68	3.80	0.007212	0.030575	-2.86
247847_at	putative protein predicted proteins,	---	AT5G58110	1.62	0.69	3.80	0.007216	0.030588	-2.86
257690_at	unknown protein contains similarity	---	AT3G12830	2.25	1.17	3.80	0.007223	0.030609	-2.86
262794_at	unknown protein EST gb|N37870 c	emb1745	AT1G13120	1.37	0.45	3.80	0.007236	0.030652	-2.86
258051_at	hypothetical protein predicted by ge	TRZ4	AT3G16260	1.49	0.58	3.80	0.007239	0.030659	-2.86
250306_at	putative protein similar to unknown	---	AT5G12170	1.58	0.66	3.80	0.007244	0.03067	-2.86
253932_at	putative protein other Arabidopsis p	---	AT4G26800	1.29	0.37	3.80	0.007245	0.03067	-2.86
258715_at	putative 60S ribosomal protein L1 s	---	AT3G09630	1.56	0.64	3.80	0.007251	0.030684	-2.86
255919_at	putative protein	---	---	-1.36	-0.44	-3.80	0.007252	0.030684	-2.86
250986_at	putative protein	---	AT5G02850	-1.34	-0.42	-3.80	0.007252	0.030684	-2.86
265347_at	similar to mammalian MHC III regio	SUVH6	AT2G22740	-1.27	-0.35	-3.80	0.007253	0.030684	-2.86
260095_at	putative transcription factor BTF3 (	---	AT1G73230	1.58	0.66	3.80	0.007264	0.030716	-2.87
266417_at	putative non-green plastid inner env	---	AT2G38550	1.44	0.53	3.80	0.007265	0.030716	-2.87
262685_at	unknown protein ; supported by cDI	---	AT1G76010	1.57	0.65	3.80	0.007265	0.030716	-2.87
245637_at	hypothetical protein similar to putati	---	AT1G25230	-2.45	-1.29	-3.80	0.007266	0.030716	-2.87
262052_at	hypothetical protein identical to hyp	---	---	1.38	0.47	3.79	0.007276	0.030752	-2.87
254574_at	expressed protein supported by cD	---	AT4G19430	3.42	1.77	3.79	0.007283	0.03076	-2.87
249285_at	unknown protein	---	AT5G41960	1.44	0.52	3.79	0.007283	0.03076	-2.87
263009_at	hypothetical protein contains similar	NHX5	AT1G54370	1.33	0.41	3.79	0.007291	0.030784	-2.87
250751_at	glucuronosyl transferase-like protei	---	AT5G05890	-1.41	-0.50	-3.79	0.007293	0.030784	-2.87
247721_at	elongin - like protein elongin C, Dro	---	AT5G59140	1.28	0.35	3.79	0.007294	0.030784	-2.87
261404_at	alpha galactosyltransferase, putativ	---	AT1G18690	1.69	0.75	3.79	0.007295	0.030784	-2.87
259363_at	methionine aminopeptidase I (MAP	MAP1C	AT1G13270	1.38	0.47	3.79	0.007296	0.030784	-2.87
252166_at	protein kinase SPK-2 ;supported by	SNRK2.2	AT3G50500	1.45	0.54	3.79	0.007299	0.030793	-2.87
258575_at	putative O-linked GlcNAc transferas	SEC	AT3G04240	1.32	0.41	3.79	0.007304	0.030808	-2.87
260976_at	hypothetical protein	CID8	AT1G53650	1.54	0.62	3.79	0.007317	0.030857	-2.87
263958_at	putative salt-inducible protein	---	AT2G36240	1.27	0.34	3.79	0.00732	0.030859	-2.87
250386_at	MYB like protein MYB like protein,	MYB3R-4	AT5G11510	-1.45	-0.54	-3.79	0.00732	0.030859	-2.87
255113_at	putative protein various hypothetica	---	---	-1.32	-0.40	-3.79	0.007324	0.030866	-2.87
257601_at	hypothetical protein predicted by ge	---	AT3G24820	1.32	0.40	3.79	0.007325	0.030866	-2.87

251929_at	sigma factor SigC ; supported by c	SIGC	AT3G53920	1.34	0.42	3.79	0.007334	0.030899	-2.88
256599_at	hypothetical protein predicted by ge	---	AT3G14760	-1.32	-0.40	-3.79	0.007338	0.03091	-2.88
258187_at	HSC70-interacting protein, putative	ATTDX	AT3G17880	1.34	0.43	3.79	0.007346	0.030938	-2.88
264460_at	hypothetical protein similar to transc	ATNFXL1	AT1G10170	1.47	0.55	3.79	0.007348	0.030943	-2.88
252044_at	outer envelope membrane protein-	OEP7	AT3G52420	1.29	0.37	3.79	0.007353	0.030952	-2.88
254088_at	putative protein snurportin1 m3G-c	---	AT4G24880	1.53	0.61	3.79	0.007353	0.030952	-2.88
257625_at	cytochrome P450, putative contain	CYP71B24	AT3G26230	4.42	2.14	3.79	0.007361	0.030977	-2.88
253025_at	hypothetical protein	---	AT4G38120	1.57	0.66	3.79	0.007362	0.030977	-2.88
267535_at	putative C2H2-type zinc finger prote	ZFP8	AT2G41940	-1.77	-0.82	-3.78	0.007371	0.031008	-2.88
250299_at	putative esterase - like protein cinl	---	AT5G11910	1.37	0.45	3.78	0.007374	0.031012	-2.88
259583_at	hypothetical protein	---	AT1G28070	1.41	0.50	3.78	0.007374	0.031012	-2.88
255434_at	predicted protein of unknown functi	---	AT4G03180	1.36	0.44	3.78	0.00738	0.03103	-2.88
247863_at	SKP1 interacting partner 1 (SKIP1)	---	AT5G57890	1.36	0.45	3.78	0.007392	0.031075	-2.88
266519_at	unknown protein ; supported by cD	---	AT2G35155	-1.40	-0.49	-3.78	0.007393	0.031075	-2.88
249161_at	20S proteasome subunit PAF1 (gb|	PAF1	AT5G42790	1.26	0.34	3.78	0.007395	0.031077	-2.88
253032_at	guanine nucleotide-exchange prote	---	AT4G38200	-1.31	-0.39	-3.78	0.007409	0.031121	-2.89
258259_s_a	unknown protein ; supported by cD	---	AT3G26820	1.37	0.45	3.78	0.007409	0.031121	-2.89
249200_at	5-3 exoribonuclease 2 ; supported b	XRN2	AT5G42540	1.47	0.55	3.78	0.00741	0.031121	-2.89
248564_at	putative protein contains similarity t	---	AT5G49700	1.53	0.61	3.78	0.007413	0.031131	-2.89
262070_at	far-red impaired response protein,	FRS8	AT1G80010	1.32	0.40	3.78	0.007418	0.031144	-2.89
258072_at	unknown protein	RGS1	AT3G26090	1.30	0.38	3.78	0.007421	0.03115	-2.89
248862_at	unknown protein	---	AT5G46730	-1.57	-0.65	-3.78	0.007422	0.03115	-2.89
248074_at	putative protein similar to unknown	FLA1	AT5G55730	-2.33	-1.22	-3.78	0.007428	0.03117	-2.89
247275_at	beta-ureidopropionase	BETA-UP	AT5G64370	1.69	0.76	3.78	0.007432	0.031178	-2.89
263145_at	hypothetical protein predicted by ge	ATEXO70D	AT1G54090	-1.50	-0.58	-3.78	0.007442	0.031214	-2.89
245198_at	Expressed protein ; supported by fu	---	AT1G67700	-1.34	-0.42	-3.78	0.007446	0.031229	-2.89
266484_at	putative putative sister-chromatide (	SCC3	AT2G47980	1.37	0.46	3.78	0.00745	0.031236	-2.89
255683_at	putative malate oxidoreductase	---	AT4G00570	-1.41	-0.50	-3.78	0.007452	0.031236	-2.89
266182_at	hypothetical protein predicted by ge	---	AT2G02370	1.50	0.58	3.78	0.007454	0.031236	-2.89
254820_s_a	pEARLI 1-like protein Arabidopsis t	---	AT4G12510	-1.95	-0.96	-3.78	0.007454	0.031236	-2.89
264373_at	putative vesicle transport protein Co	SEC22	AT1G11890	-1.50	-0.58	-3.78	0.007455	0.031237	-2.89
260463_at	DNA helicase isolog	RECQ4A	AT1G10930	-1.28	-0.35	-3.78	0.007459	0.031243	-2.89
258281_at	putative shikimate kinase similar to	---	AT3G26900	1.26	0.34	3.78	0.007459	0.031243	-2.89
258640_at	hypothetical protein predicted by ge	---	AT3G07930	1.46	0.55	3.77	0.007463	0.031251	-2.89
267065_at	hypothetical protein predicted by ge	---	AT2G41080	1.41	0.50	3.77	0.007477	0.031307	-2.90

265162_at	hypothetical protein predicted by ge	---	AT1G30910	1.39	0.48	3.77	0.007486	0.031332	-2.90
264146_at	hypothetical protein contains similar	CER1	AT1G02205	-16.94	-4.08	-3.77	0.007486	0.031332	-2.90
259075_at	putative N2,N2-dimethylguanosine	---	AT3G02320	1.39	0.48	3.77	0.007494	0.031359	-2.90
265460_at	putative caltractin ;supported by ful	---	AT2G46600	1.70	0.76	3.77	0.007497	0.031359	-2.90
266202_at	putative cinnamoyl-CoA reductase	---	AT2G02400	-1.31	-0.39	-3.77	0.007502	0.031375	-2.90
260012_at	Expressed protein ; supported by cI	---	AT1G67865	1.78	0.84	3.77	0.007507	0.031389	-2.90
265440_at	pEARLI 4 protein Same as GB: L43	pEARLI4	AT2G20960	1.74	0.80	3.77	0.007509	0.031393	-2.90
250929_at	putative protein ; supported by cDN	---	AT5G03290	1.58	0.66	3.77	0.00751	0.031393	-2.90
259512_at	hypothetical protein ; supported by	KEU	AT1G12360	-1.28	-0.36	-3.77	0.007512	0.031393	-2.90
248877_at	putative protein similar to unknown	---	AT5G46140	-1.33	-0.41	-3.77	0.007516	0.031405	-2.90
258180_at	unknown protein	---	AT3G21480	1.67	0.74	3.77	0.007526	0.031424	-2.90
253368_at	myosin - like protein unconventiona	XI-I	AT4G33200	1.35	0.43	3.77	0.007526	0.031424	-2.90
248195_at	membrane associated protein ; sup	ATMAMI	AT5G54110	-1.45	-0.53	-3.77	0.007533	0.031435	-2.90
260683_at	ribosomal protein, putative similar t	HLL	AT1G17560	1.37	0.45	3.77	0.007536	0.031435	-2.91
246103_at	putative protein	AN3	AT5G28640	-1.26	-0.34	-3.77	0.007536	0.031435	-2.91
258855_at	unknown protein	---	AT3G02070	-1.67	-0.74	-3.77	0.007537	0.031435	-2.91
247955_at	nucleosome assembly protein	NAP1;3	AT5G56950	1.45	0.53	3.77	0.007546	0.031466	-2.91
252413_at	40S ribosomal protein S20-like pro	---	AT3G47370	1.84	0.88	3.77	0.007552	0.031487	-2.91
256231_at	unknown protein ; supported by full-	---	AT3G12630	1.33	0.41	3.76	0.007564	0.031531	-2.91
250664_at	putative protein 10-deacetylbaccati	---	AT5G07080	-1.97	-0.98	-3.76	0.007575	0.031563	-2.91
248787_at	putative protein similar to unknown	---	AT5G47420	-1.57	-0.65	-3.76	0.007575	0.031563	-2.91
256505_at	unknown protein	---	AT1G75200	1.52	0.60	3.76	0.007576	0.031563	-2.91
267416_at	putative phosphatidylinositol-glycan	SETH1	AT2G34980	-1.32	-0.40	-3.76	0.007578	0.031565	-2.91
259590_at	hypothetical protein contains similar	---	AT1G28160	-1.38	-0.46	-3.76	0.007581	0.031574	-2.91
261560_at	acyl CoA thioesterase, putative sim	---	AT1G01710	-1.28	-0.36	-3.76	0.007584	0.031579	-2.91
262249_at	root hairless 1 (RHL1) similar to roc	RHL1	AT1G48380	-1.38	-0.47	-3.76	0.007585	0.031579	-2.91
257817_at	putative RNA-binding protein conta	---	AT3G25150	-1.34	-0.42	-3.76	0.007595	0.031609	-2.91
259043_at	unknown protein similar to putative	---	AT3G03440	1.61	0.69	3.76	0.007595	0.031609	-2.91
248784_at	putative protein similar to unknown	---	AT5G47380	-1.58	-0.66	-3.76	0.0076	0.031621	-2.91
267604_at	hypothetical protein predicted by ge	ZFN2	AT2G32930	-1.84	-0.88	-3.76	0.007601	0.031621	-2.91
248436_at	putative protein contains similarity t	---	AT5G51220	1.33	0.41	3.76	0.007604	0.031629	-2.91
257046_at	ATPase, putative similar to spastin	---	AT3G19740	1.51	0.59	3.76	0.007613	0.031657	-2.92
245003_at	PSII 43 KDa protein	---	---	-1.37	-0.46	-3.76	0.007618	0.031674	-2.92
267561_at	putative protein kinase contains a p	---	AT2G45590	-1.68	-0.75	-3.76	0.007631	0.031721	-2.92
263435_at	putative ATP-dependent RNA helica	---	AT2G28600	1.73	0.79	3.76	0.007636	0.031737	-2.92

249866_at	2-isopropylmalate synthase-like; ho	MAM1	AT5G23010	-1.85	-0.89	-3.76	0.007651	0.031795	-2.92
247300_at	unknown protein	FCLY	AT5G63910	1.38	0.46	3.75	0.007658	0.031816	-2.92
257798_at	unknown protein	NAI2	AT3G15950	-1.52	-0.60	-3.75	0.007677	0.031883	-2.93
249810_at	unknown protein	---	AT5G23920	1.61	0.68	3.75	0.007681	0.031892	-2.93
248526_at	putative protein contains similarity t	---	AT5G50740	-1.55	-0.63	-3.75	0.007681	0.031892	-2.93
265449_at	putative arginine/serine-rich splicing	---	AT2G46610	1.57	0.65	3.75	0.007692	0.031931	-2.93
251581_at	putative protein CARBON CATABO	---	AT3G58560	1.42	0.51	3.75	0.007695	0.031936	-2.93
245378_at	GLABRA2 like protein	HDG4	AT4G17710	-1.30	-0.38	-3.75	0.007696	0.031936	-2.93
247281_at	immunophilin (gb|AAB57847.1) ;su	FKBP12	AT5G64350	1.30	0.38	3.75	0.007698	0.031937	-2.93
259659_at	hypothetical protein contains similar	---	AT1G55170	1.52	0.60	3.75	0.007706	0.031964	-2.93
246343_at	putative protein	---	AT3G56720	1.25	0.32	3.75	0.007708	0.031965	-2.93
252671_at	putative protein dehydrogenases of	---	AT3G44190	-1.47	-0.56	-3.75	0.007712	0.031972	-2.93
252586_at	dof6 zinc finger protein	---	AT3G45610	-1.38	-0.46	-3.75	0.007737	0.032071	-2.93
255397_at	putative transposon protein	---	---	-1.48	-0.56	-3.75	0.007745	0.032091	-2.93
256611_at	unknown protein similar to putative	---	AT3G29270	1.42	0.51	3.75	0.00775	0.032105	-2.94
248267_at	NADH-dependent glutamate syntha	GLT1	AT5G53460	1.56	0.64	3.74	0.007758	0.032133	-2.94
258822_s_a	unknown protein contains Pfam pro	---	AT3G07220	1.67	0.74	3.74	0.007762	0.032144	-2.94
262542_at	hypothetical protein similar to NAM-	anac016	AT1G34180	1.67	0.74	3.74	0.007764	0.032144	-2.94
257224_at	P-type ATPase, putative similar to	---	AT3G27870	-1.40	-0.49	-3.74	0.007765	0.032144	-2.94
263821_s_a	40S ribosomal protein S16 Same as	---	AT2G09990	1.55	0.63	3.74	0.007776	0.032181	-2.94
246955_at	calcium-dependent protein kinase	CPK1	AT5G04870	-2.06	-1.04	-3.74	0.007777	0.032181	-2.94
261314_at	putative GTP-binding protein simila	---	AT1G52980	1.56	0.64	3.74	0.007778	0.032182	-2.94
255723_at	hypothetical protein	AFP3	AT3G29575	-2.08	-1.06	-3.74	0.00778	0.032184	-2.94
253778_at	heat-shock protein heat-shock prot	---	AT4G28480	-1.43	-0.51	-3.74	0.007787	0.032201	-2.94
263607_at	unknown protein predicted by gens	---	AT2G16270	-1.49	-0.58	-3.74	0.007788	0.032201	-2.94
266583_at	Expressed protein ; supported by cl	---	AT2G46220	1.52	0.61	3.74	0.007788	0.032201	-2.94
258569_at	ribosomal protein L17, putative sim	emb2171	AT3G04400	1.55	0.63	3.74	0.007794	0.032217	-2.94
262224_at	hypothetical protein contains Pfam	OTP43	AT1G74900	1.29	0.37	3.74	0.007796	0.032219	-2.94
256992_at	hypothetical protein predicted by ge	---	AT3G28630	-1.37	-0.45	-3.74	0.007805	0.032253	-2.94
246220_at	putative protein ; supported by cDN	---	AT4G37210	1.31	0.39	3.74	0.00781	0.032263	-2.94
248816_at	prematurely terminated mRNA deca	LBA1	AT5G47010	1.31	0.39	3.74	0.007811	0.032263	-2.94
250428_at	putative protein phosphatase prote	PAS2	AT5G10480	-1.72	-0.78	-3.74	0.007824	0.032313	-2.95
252164_at	hypothetical protein nodH - Rhizob	---	AT3G50620	-1.91	-0.94	-3.74	0.007829	0.032329	-2.95
254846_at	Expressed protein ; supported by fu	PLDGAMM	AT4G11830	-1.45	-0.54	-3.74	0.007833	0.032336	-2.95
252481_at	putative protein DCL PROTEIN, CH	---	AT3G46630	-1.42	-0.50	-3.74	0.007834	0.032336	-2.95

265917_at	putative disease resistance protein	AtRLP19	AT2G15080	1.48	0.56	3.74	0.007835	0.032336	-2.95
260177_at	hypothetical protein predicted by ge	---	AT1G70650	1.60	0.68	3.74	0.007837	0.032336	-2.95
260666_at	unknown protein ;supported by full-	PARVUS	AT1G19300	-2.12	-1.08	-3.74	0.007853	0.032398	-2.95
246575_at	bystin, putative similar to bystin GI:	---	AT1G31660	1.63	0.71	3.73	0.007855	0.032402	-2.95
254194_at	auxin response factor 9 (ARF9) ; su	ARF9	AT4G23980	1.56	0.64	3.73	0.007866	0.032433	-2.95
245319_at	Expressed protein ; supported by fu	---	AT4G16146	1.69	0.76	3.73	0.007879	0.032474	-2.95
247871_at	xyloglucan endotransglycosylase ;	---	AT5G57530	-1.28	-0.36	-3.73	0.00788	0.032475	-2.95
262622_at	hypothetical protein predicted by ge	---	AT1G06510	1.31	0.38	3.73	0.007882	0.032476	-2.95
259165_at	homeobox-leucine zipper protein H/	ATHB-1	AT3G01470	1.84	0.88	3.73	0.007884	0.03248	-2.95
251104_at	F-box protein family, AtFBL3 conta	---	AT5G01720	1.48	0.57	3.73	0.007888	0.032487	-2.95
253486_at	putative protein KIAA0260 gene, hu	---	AT4G31600	-1.34	-0.43	-3.73	0.007904	0.032548	-2.96
266074_at	putative small nuclear ribonucleopro	---	AT2G18740	1.63	0.71	3.73	0.007905	0.032548	-2.96
254360_at	CDP-diacylglycerol synthetase-like	---	AT4G22340	-1.33	-0.41	-3.73	0.00791	0.032556	-2.96
258052_at	putative hydrolase predicted by gen	---	AT3G16190	1.39	0.48	3.73	0.007913	0.032563	-2.96
260753_at	RING-H2 finger protein RHA3a, pu	---	AT1G49230	2.08	1.06	3.73	0.007918	0.032577	-2.96
248583_at	putative protein strong similarity to u	emb1441	AT5G49930	1.42	0.50	3.73	0.007922	0.032586	-2.96
254290_at	hypothetical protein	---	AT4G23000	1.54	0.62	3.73	0.007926	0.032586	-2.96
252132_at	putative LEA protein Picea glauca	---	AT3G50790	-1.83	-0.87	-3.73	0.007926	0.032586	-2.96
256772_at	galactosidase, putative similar to B	BGAL1	AT3G13750	-2.77	-1.47	-3.73	0.007928	0.032586	-2.96
263579_at	hypothetical protein similar to hypot	---	AT2G17030	1.50	0.58	3.73	0.007929	0.032586	-2.96
262951_at	nodulin-like protein similar to MtN2	---	AT1G75500	-1.47	-0.56	-3.73	0.007933	0.032596	-2.96
247899_at	Expressed protein ; supported by fu	---	AT5G57345	-1.44	-0.52	-3.73	0.00794	0.032618	-2.96
254564_at	neoxanthin cleavage enzyme-like p	NCED4	AT4G19170	1.65	0.72	3.73	0.007941	0.032618	-2.96
246450_at	Heat Shock Factor 3	HSF3	AT5G16820	1.31	0.38	3.73	0.007953	0.032662	-2.96
260801_at	hypothetical protein predicted by ge	---	AT1G78430	-1.26	-0.33	-3.72	0.007955	0.032665	-2.96
251632_at	hypothetical protein	---	AT3G57440	-1.44	-0.53	-3.72	0.007965	0.032701	-2.96
264824_at	unknown protein similar to gb|T454	2A6	---	-1.63	-0.70	-3.72	0.007967	0.032702	-2.97
245775_at	serine/threonine kinase, putative si	CIPK23	AT1G30270	1.35	0.43	3.72	0.007972	0.032717	-2.97
267159_at	putative SCARECROW gene regula	---	AT2G37650	1.35	0.44	3.72	0.007974	0.032717	-2.97
247787_at	putative protein various predicted p	---	AT5G58640	1.30	0.37	3.72	0.007975	0.032717	-2.97
259491_at	chlorophyll binding protein, putative	LHCB6	AT1G15820	-1.39	-0.48	-3.72	0.007983	0.032746	-2.97
264821_at	hypothetical protein predicted by ge	---	AT1G03470	1.65	0.72	3.72	0.007985	0.032748	-2.97
264134_at	unknown protein contains similarity	---	AT1G79070	1.27	0.34	3.72	0.008005	0.032818	-2.97
264319_at	subtilisin-like serine protease, puta	SDD1	AT1G04110	-1.43	-0.51	-3.72	0.008005	0.032818	-2.97
252012_at	ubiquinol--cytochrome-c reductase	---	AT3G52730	1.33	0.41	3.72	0.008016	0.032844	-2.97

247388_s_a	transcription factor Hap5a-like prote	NF-YC1	AT3G48590	-1.83	-0.87	-3.72	0.008016	0.032844	-2.97
264984_at	unknown protein ;supported by full-	---	AT1G27000	1.27	0.35	3.72	0.00802	0.032844	-2.97
261558_at	unknown protein	---	AT1G01770	1.63	0.70	3.72	0.00802	0.032844	-2.97
245415_at	G2484-1 protein	ATG2484-1	AT4G17330	1.29	0.37	3.72	0.008022	0.032844	-2.97
257661_at	unknown protein contains Pfam pro	---	AT3G13340	1.26	0.34	3.72	0.008023	0.032844	-2.97
263918_at	putative proline transporter ;suppor	ProT3	AT2G36590	-2.99	-1.58	-3.72	0.008024	0.032844	-2.97
267236_at	GDP dissociation inhibitor identical	ATGDI1	AT2G44100	1.35	0.44	3.72	0.008025	0.032844	-2.97
258797_at	auxin-induced protein identical to G	IAA16	AT3G04730	-1.54	-0.62	-3.72	0.008026	0.032844	-2.97
257530_at	unknown protein	---	AT3G03040	1.32	0.40	3.72	0.008033	0.032867	-2.97
258576_at	putative 40S ribosomal protein S16	---	AT3G04230	1.51	0.60	3.72	0.008035	0.032867	-2.97
260687_at	inner mitochondrial membrane prot	ATTIM23-1	AT1G17530	1.58	0.66	3.72	0.008039	0.032876	-2.97
260336_at	putative protease IV similar to prote	SPPA	AT1G73990	-1.42	-0.51	-3.72	0.008039	0.032876	-2.97
260443_at	Expressed protein ; supported by fu	---	AT1G68185	1.27	0.34	3.72	0.008047	0.0329	-2.98
256866_at	unknown protein similar to alpha ga	---	AT3G26380	1.48	0.56	3.72	0.008051	0.03291	-2.98
247491_at	putative protein supported by cDNA	---	AT5G61880	1.80	0.85	3.71	0.008062	0.032948	-2.98
251324_at	plasma membrane intrinsic protein	PIP1A	AT3G61430	-1.43	-0.52	-3.71	0.008064	0.032951	-2.98
255682_at	putative dTDP-6-deoxy-L-mannose	---	AT4G00560	-1.35	-0.43	-3.71	0.008065	0.032951	-2.98
262721_at	thioredoxin, putative similar to thior	Aty2	AT1G43560	-1.43	-0.52	-3.71	0.008067	0.032951	-2.98
259123_at	unknown protein	---	AT3G02200	1.31	0.39	3.71	0.008068	0.032951	-2.98
264339_at	trehalose-6-phosphate synthase, p	TPS8	AT1G70290	-1.59	-0.67	-3.71	0.00807	0.032953	-2.98
259175_at	unknown protein	---	AT3G01560	-1.43	-0.51	-3.71	0.008071	0.032953	-2.98
261975_at	unknown protein	---	AT1G64640	-2.44	-1.29	-3.71	0.008079	0.032974	-2.98
254981_at	putative alpha NAC stong similarity	---	AT4G10480	1.68	0.75	3.71	0.008079	0.032974	-2.98
259068_at	unknown protein	PEX13	AT3G07560	1.37	0.45	3.71	0.008093	0.033004	-2.98
258725_at	putative RNA helicase similar to R	---	AT3G09620	-1.30	-0.38	-3.71	0.008093	0.033004	-2.98
266790_at	expansin AtEx6 identical to GB U30	ATEXPA6	AT2G28950	-4.02	-2.01	-3.71	0.008097	0.033017	-2.98
247882_at	Expressed protein ; supported by fu	---	AT5G57785	-2.73	-1.45	-3.71	0.008101	0.033024	-2.98
256402_at	hypothetical protein ; supported by	---	AT3G06130	-1.87	-0.90	-3.71	0.008102	0.033024	-2.98
254728_at	hypothetical protein	---	AT4G13690	-1.32	-0.40	-3.71	0.008104	0.033027	-2.98
257814_at	acyl-(acyl carrier protein) thioestera	AtFaTA	AT3G25110	-1.92	-0.94	-3.71	0.008112	0.033055	-2.98
255556_at	putative NifU-like metallocluster as	NFU1	AT4G01940	1.47	0.55	3.71	0.008115	0.033059	-2.98
249217_at	ubiquitin-like protein ; supported by	UBL5	AT5G42300	1.26	0.33	3.71	0.008119	0.033069	-2.99
258322_at	putative selenocysteine methyltrans	HMT3	AT3G22740	-2.54	-1.35	-3.71	0.008126	0.033091	-2.99
267454_at	putative U5 small nuclear ribonucle	---	AT2G33730	1.32	0.40	3.71	0.008131	0.033107	-2.99
261746_at	unknown protein ;supported by full-	PSAO	AT1G08380	-1.29	-0.37	-3.71	0.008136	0.033124	-2.99

245977_at	glucose-6-phosphate 1-dehydrogen	G6PD2	AT5G13110	1.78	0.83	3.71	0.008143	0.033138	-2.99
256628_at	membrane import protein, putative	TOM40	AT3G20000	1.62	0.69	3.71	0.008143	0.033138	-2.99
246576_at	hypothetical protein predicted by ge	ROPGEF1	AT1G31650	1.28	0.35	3.71	0.008151	0.033154	-2.99
267285_at	unknown protein	---	AT2G23690	-1.70	-0.76	-3.71	0.008151	0.033154	-2.99
247283_at	2-nitropropane dioxygenase-like pro	---	AT5G64250	1.38	0.46	3.70	0.008165	0.033203	-2.99
253002_at	phosphoinositide-specific phospholi	ATPLC1	AT4G38530	-1.40	-0.49	-3.70	0.008166	0.033203	-2.99
247188_at	14-3-3 protein GF14kappa (grf8) id	GRF8	AT5G65430	-1.38	-0.47	-3.70	0.008171	0.033212	-2.99
262918_at	hypothetical protein predicted by ge	---	AT1G65000	1.38	0.47	3.70	0.008171	0.033212	-2.99
261300_at	hypothetical protein predicted by ge	---	AT1G48560	1.40	0.48	3.70	0.008176	0.033225	-2.99
254869_at	protein kinase - like protein KI dom	---	AT4G11890	1.99	0.99	3.70	0.008181	0.033242	-2.99
259406_at	hypothetical protein predicted by ge	---	AT1G17690	1.29	0.37	3.70	0.008195	0.033292	-3.00
250739_at	putative protein similar to unknown	EGY2	AT5G05740	-1.55	-0.63	-3.70	0.008197	0.033292	-3.00
254159_at	putative DNA-binding protein DNA-	WRKY7	AT4G24240	1.85	0.89	3.70	0.0082	0.0333	-3.00
247831_at	putative protein serine/threonine-sp	---	AT5G58540	1.69	0.76	3.70	0.008203	0.033304	-3.00
254212_at	putative protein many putative prot	---	AT4G23580	-1.33	-0.41	-3.70	0.008204	0.033305	-3.00
256671_at	unknown protein	IQD3	AT3G52290	-1.56	-0.65	-3.70	0.008209	0.033316	-3.00
267246_at	putative WRKY-type DNA binding p	WRKY25	AT2G30250	-1.76	-0.82	-3.70	0.008213	0.033327	-3.00
254603_at	putative protein hypothetical protei	---	AT4G19045	-1.39	-0.47	-3.70	0.008221	0.033353	-3.00
259799_at	drought induced protein, putative s	---	AT1G72290	-1.27	-0.34	-3.70	0.008225	0.033366	-3.00
264138_at	beta-Amyrin Synthase, putative sin	---	AT1G78950	-1.39	-0.47	-3.70	0.008228	0.033368	-3.00
250039_at	disease resistance protein -like dis	---	AT5G18370	1.42	0.51	3.70	0.00823	0.033368	-3.00
266535_s_a	50S ribosomal protein L27 ;suppor	---	AT2G16930	1.47	0.56	3.70	0.00823	0.033368	-3.00
249059_at	subtilisin-like serine protease conta	---	AT5G44530	-1.61	-0.69	-3.70	0.008238	0.033394	-3.00
245219_at	viral resistance protein, putative sir	---	AT1G58807	1.33	0.41	3.70	0.008242	0.033404	-3.00
257987_at	fertilization-independent endosperm	FIE	AT3G20740	1.32	0.40	3.70	0.008257	0.033446	-3.00
267177_at	Expressed protein ; supported by fu	---	AT2G37580	-1.64	-0.72	-3.70	0.008257	0.033446	-3.00
260195_at	hypothetical protein predicted by ge	---	AT1G67540	-1.35	-0.43	-3.70	0.008258	0.033446	-3.00
250533_at	flavonol synthase (FLS) (sp|Q9633	FLS	AT5G08640	-1.30	-0.38	-3.70	0.00826	0.033447	-3.00
248043_s_a	heat shock protein (emb|CAA72514	---	AT5G56000	-1.69	-0.76	-3.70	0.008262	0.03345	-3.00
264852_at	similar to Mlo proteins from H. vulga	MLO8	AT2G17480	-2.07	-1.05	-3.69	0.008266	0.033459	-3.00
246700_at	cysteine synthase - like cysteine sy	---	AT5G28030	1.82	0.86	3.69	0.008267	0.033459	-3.00
252618_at	lipoxygenase AtLOX2 ; supported b	LOX2	AT3G45140	-1.51	-0.60	-3.69	0.008272	0.033468	-3.01
265092_at	hypothetical protein predicted by ge	---	AT1G03910	1.28	0.36	3.69	0.008272	0.033468	-3.01
245342_at	ribosomal protein ;supported by full-	---	AT4G16720	1.54	0.62	3.69	0.008275	0.033473	-3.01
253842_at	putative protein putative proteins fro	---	AT4G27860	-2.36	-1.24	-3.69	0.008276	0.033473	-3.01

260794_at	unknown protein ; supported by cDI	---	AT1G06210	1.37	0.46	3.69	0.008279	0.033475	-3.01
257788_at	unknown protein	TCP20	AT3G27010	1.46	0.55	3.69	0.00828	0.033475	-3.01
266472_at	unknown protein	---	AT2G31150	-1.27	-0.34	-3.69	0.008285	0.033488	-3.01
260564_at	putative small nuclear ribonucleopr	---	AT2G43810	1.41	0.49	3.69	0.008286	0.033488	-3.01
253470_s_a	putative protein dnaK-type molecul	SDH3-2	AT4G32210	1.30	0.38	3.69	0.008294	0.033512	-3.01
264133_at	hypothetical protein predicted by ge	---	AT1G79080	1.35	0.43	3.69	0.008298	0.033519	-3.01
258404_at	ribosomal protein L3	RPL3P	AT3G17465	1.43	0.51	3.69	0.00832	0.033593	-3.01
262230_at	alpha-xylosidase precursor identica	XYL1	AT1G68560	-1.99	-0.99	-3.69	0.008322	0.033598	-3.01
264429_at	hypothetical protein similar to meml	---	AT1G61670	-1.29	-0.37	-3.69	0.008326	0.033605	-3.01
261757_at	hypothetical protein similar to putati	---	AT1G08210	1.57	0.65	3.69	0.008327	0.033605	-3.01
264531_at	hypothetical protein predicted by ge	---	AT1G30790	-1.32	-0.40	-3.69	0.00833	0.033612	-3.01
258471_at	NPK1-related protein kinase 3 iden	ANP3	AT3G06030	-1.36	-0.44	-3.69	0.008336	0.033621	-3.01
249023_at	putative protein strong similarity to u	---	AT5G44850	-1.90	-0.93	-3.69	0.008339	0.033621	-3.01
263186_at	lectin receptor kinase, putative sim	---	---	-1.51	-0.59	-3.69	0.008339	0.033621	-3.01
263347_at	putative U4/U6 small nuclear ribonu	---	AT2G05720	-1.47	-0.56	-3.69	0.00834	0.033621	-3.01
252034_at	putative protein ;supported by full-l	---	AT3G52040	1.78	0.83	3.69	0.008343	0.033623	-3.01
256221_at	DnaJ protein, putative contains Pfa	---	AT1G56300	1.31	0.39	3.69	0.008343	0.033623	-3.01
245756_at	hyoscyamine 6-dioxygenase hydro	---	AT1G35190	1.54	0.62	3.69	0.008353	0.033657	-3.02
254986_at	putative protein SF16 protein -Helia	IQD16	AT4G10640	-1.26	-0.34	-3.69	0.008355	0.033658	-3.02
260671_at	unknown protein ;supported by full-	---	AT1G19310	-1.42	-0.51	-3.69	0.008364	0.033688	-3.02
262277_at	transmembrane protein, putative si	---	AT1G68650	1.93	0.95	3.69	0.008367	0.033695	-3.02
260913_at	s-adenosylmethionine synthetase id	SAM1	AT1G02500	1.66	0.73	3.69	0.008368	0.033695	-3.02
266927_at	aquaporin (plasma membrane intri	PIP1B	AT2G45960	-1.45	-0.54	-3.69	0.00837	0.033695	-3.02
267409_at	hypothetical protein predicted by ge	---	AT2G34910	-1.26	-0.34	-3.68	0.008381	0.033732	-3.02
250091_at	unknown protein	---	AT5G17340	-1.26	-0.33	-3.68	0.008382	0.033733	-3.02
246674_at	bcnt-like protein ; supported by full-	---	AT5G30490	1.34	0.42	3.68	0.008398	0.033789	-3.02
250585_at	putative protein contains similarity t	---	AT5G07620	-1.91	-0.93	-3.68	0.008399	0.033789	-3.02
267475_at	expressed protein ; supported by fu	---	AT2G02730	1.42	0.50	3.68	0.008414	0.033845	-3.02
255400_at	hypothetical protein	---	---	-1.38	-0.47	-3.68	0.008421	0.033859	-3.02
266578_at	putative cinnamoyl CoA reductase	---	AT2G23910	1.42	0.51	3.68	0.008423	0.033859	-3.02
253033_at	putative protein aminoacylase I - S	---	AT4G38220	-1.33	-0.41	-3.68	0.008423	0.033859	-3.02
259375_at	putative APG protein similar to ante	---	AT3G16370	-15.93	-3.99	-3.68	0.008425	0.033859	-3.03
255483_at	putative glycosyltransferase similar	XT2	AT4G02500	-1.54	-0.62	-3.68	0.008425	0.033859	-3.03
259513_at	hypothetical protein ; supported by	ARK3	AT1G12430	1.37	0.45	3.68	0.008428	0.033861	-3.03
246424_at	Exportin1 (XPO1) protein ; support	XPO1A	AT5G17020	1.40	0.49	3.68	0.008429	0.033861	-3.03

246396_at	carbonic anhydrase, putative simila	BCA6	AT1G58180	-1.57	-0.65	-3.68	0.00844	0.033899	-3.03
244939_at	ribosomal protein S12 (trans-splice	---	---	-1.63	-0.70	-3.68	0.008443	0.0339	-3.03
251595_at	putative protein galactose oxidase	---	AT3G57620	-1.36	-0.44	-3.68	0.008443	0.0339	-3.03
257372_at	hypothetical protein predicted by ge	---	AT2G43220	-1.31	-0.39	-3.68	0.008453	0.03393	-3.03
248241_at	unknown protein	---	AT5G53960	-1.31	-0.39	-3.68	0.008454	0.03393	-3.03
247368_at	putative protein contains similarity t	---	AT5G63320	1.42	0.51	3.68	0.008458	0.033941	-3.03
261903_at	chromomethylase identical to GB:A	CMT1	AT1G80740	-1.28	-0.35	-3.68	0.008459	0.033941	-3.03
253979_at	putative protein other putative prote	---	AT4G26680	1.36	0.44	3.68	0.008466	0.033961	-3.03
263037_at	conserved hypothetical protein	---	AT1G23230	1.42	0.51	3.68	0.008469	0.033968	-3.03
253769_at	putative protein (fragment) protein	RIC7	AT4G28560	-1.44	-0.53	-3.68	0.008471	0.033969	-3.03
249019_at	unknown protein	---	AT5G44780	2.03	1.02	3.67	0.008489	0.034037	-3.03
256620_at	disease resistance protein, putative	---	AT3G24480	-1.69	-0.76	-3.67	0.008491	0.034041	-3.03
259433_at	hypothetical protein predicted by ge	---	AT1G01570	-1.40	-0.49	-3.67	0.008495	0.034048	-3.03
261324_at	unknown protein	---	AT1G44770	1.49	0.58	3.67	0.008499	0.034053	-3.03
258642_at	unknown protein ;supported by full-	---	AT3G07910	1.30	0.37	3.67	0.008504	0.034068	-3.04
265266_at	putative RNA-binding protein ; sup	AML2	AT2G42890	1.43	0.51	3.67	0.008506	0.034068	-3.04
259307_at	unknown protein similar to MICROS	---	AT3G05230	-1.47	-0.55	-3.67	0.008511	0.034082	-3.04
257647_at	Expressed protein ; supported by cl	---	AT3G25805	-1.70	-0.76	-3.67	0.008517	0.034093	-3.04
250999_at	putative protein predicted proyeins	---	AT5G02630	1.48	0.57	3.67	0.008519	0.034093	-3.04
267400_at	unknown protein	---	AT2G26240	1.47	0.55	3.67	0.00852	0.034093	-3.04
260610_at	putative SF16 protein {Helianthus a	IQD14	AT2G43680	1.61	0.69	3.67	0.008521	0.034093	-3.04
264013_at	unknown protein	FIO1	AT2G21070	1.26	0.33	3.67	0.008526	0.034107	-3.04
260927_at	hypothetical protein predicted by ge	CAT9	AT1G05940	1.43	0.51	3.67	0.008529	0.034115	-3.04
257986_at	Expressed protein ; supported by fu	AGP40	AT3G20865	-1.59	-0.67	-3.67	0.008534	0.034123	-3.04
249612_at	putative protein CG14881, Drosoph	---	AT5G37290	1.76	0.81	3.67	0.008534	0.034123	-3.04
251664_at	leucine zipper-containing protein AT	CRD1	AT3G56940	-1.40	-0.49	-3.67	0.008539	0.034137	-3.04
250007_at	beta-amylase-like proten beta-amy	BMY3	AT5G18670	-3.23	-1.69	-3.67	0.008543	0.034144	-3.04
246755_at	F-box protein family contains simila	---	AT5G27920	-1.92	-0.94	-3.67	0.008544	0.034144	-3.04
251295_at	putative protein several O-methyltra	---	AT3G62000	1.48	0.57	3.67	0.008548	0.034151	-3.04
257052_at	putative 3-hydroxybutyryl-CoA dehy	---	AT3G15290	-1.31	-0.39	-3.67	0.008549	0.034151	-3.04
245400_at	ATP-dependent Clp protease prote	---	AT4G17040	1.44	0.52	3.67	0.008553	0.034162	-3.04
251132_at	putative protein Myb-related transc	---	AT5G01200	1.50	0.58	3.67	0.008555	0.034162	-3.04
246437_at	putative protein hypersensitivity-rel	---	AT5G17540	-1.30	-0.38	-3.67	0.008556	0.034163	-3.04
257189_at	hypothetical protein	GIP1	AT3G13222	1.29	0.37	3.67	0.008563	0.034183	-3.04
255485_at	predicted protein of unknown funct	---	AT4G02550	1.33	0.41	3.67	0.008572	0.034214	-3.04

248367_at	actin depolymerizing factor-like ;su	ADF10	AT5G52360	-1.42	-0.50	-3.67	0.008583	0.034253	-3.05
266467_at	Not56-like protein ; supported by c	ALG3	AT2G47760	-1.39	-0.48	-3.67	0.008585	0.034254	-3.05
247439_at	plasma membrane proton ATPase-	AHA11	AT5G62670	-1.83	-0.87	-3.67	0.00859	0.034268	-3.05
260056_at	hypothetical protein predicted by ge	---	AT1G78140	-1.58	-0.66	-3.66	0.008614	0.034351	-3.05
AFFX-BioB-	E. coli /GEN=bioB /DB_XREF=gb:J	---	---	-1.80	-0.85	-3.66	0.008616	0.034353	-3.05
255820_at	putative protein kinase contains a p	---	AT2G40580	-1.39	-0.47	-3.66	0.00863	0.034401	-3.05
261539_at	hypothetical protein predicted by ge	---	AT1G01840	-1.38	-0.47	-3.66	0.008631	0.034401	-3.05
247644_s_a	translation elongation factor eEF-1	---	AT1G07920	1.33	0.41	3.66	0.008641	0.034435	-3.05
250238_at	unknown protein	DCP2	AT5G13570	1.32	0.40	3.66	0.008657	0.03449	-3.05
260948_at	delta 9 desaturase, putative similar	---	AT1G06100	-1.38	-0.46	-3.66	0.008659	0.03449	-3.05
256704_at	deacetylvindoline 4-O-acetyltransfe	---	AT3G30280	-1.30	-0.38	-3.66	0.00866	0.03449	-3.05
259670_at	protein kinase, putative similar to G	---	AT1G52310	1.42	0.51	3.66	0.00867	0.034527	-3.06
255583_at	hypothetical protein similar to C. el	ARV2	AT4G01510	1.39	0.47	3.66	0.008679	0.034555	-3.06
253662_at	transcription factor-like protein Ara	ARF16	AT4G30080	-1.36	-0.44	-3.66	0.008684	0.034569	-3.06
264615_at	unknown protein ; supported by cD	---	AT1G04590	1.60	0.67	3.66	0.008688	0.034579	-3.06
262991_at	hypothetical protein predicted by ge	BRX-LIKE3	AT1G54180	-1.27	-0.34	-3.66	0.008692	0.034588	-3.06
262887_at	hypothetical protein	---	AT1G14780	-2.64	-1.40	-3.66	0.008693	0.034588	-3.06
263950_at	hypothetical protein predicted by ge	HVA22J	AT2G36020	-1.25	-0.32	-3.66	0.008697	0.034597	-3.06
251195_at	glutaredoxin -like protein glutaredo	---	AT3G62930	-1.65	-0.72	-3.65	0.008727	0.034699	-3.06
263666_at	putative pre-mRNA splicing factor F	---	AT1G04510	1.52	0.60	3.65	0.008739	0.034734	-3.06
261085_at	hypothetical protein similar to hypot	IQD7	AT1G17480	-1.30	-0.38	-3.65	0.008746	0.03475	-3.07
254578_at	putative pectinacetylesterase prote	---	AT4G19400	-1.59	-0.67	-3.65	0.008748	0.03475	-3.07
261666_at	hypothetical protein contains simila	---	AT1G18440	-1.45	-0.54	-3.65	0.008749	0.03475	-3.07
261110_at	E2, ubiquitin-conjugating enzyme 1	UBC16	AT1G75440	-1.50	-0.58	-3.65	0.008752	0.03475	-3.07
260051_at	hypothetical protein	---	AT1G78210	-1.90	-0.92	-3.65	0.008752	0.03475	-3.07
253237_at	putative aldehyde dehydrogenase	ALDH3I1	AT4G34240	-1.60	-0.68	-3.65	0.008752	0.03475	-3.07
253205_at	putative cyclase associated protein	ATCAP1	AT4G34490	-1.70	-0.77	-3.65	0.008758	0.034769	-3.07
261823_at	hypothetical protein predicted by ge	PYM	AT1G11400	-1.33	-0.42	-3.65	0.008767	0.034795	-3.07
255667_at	phospholipase D, putative	PLDBETA2	AT4G00240	-1.32	-0.40	-3.65	0.00877	0.034802	-3.07
251525_at	putative protein several hypothetica	---	AT3G59000	1.39	0.48	3.65	0.008777	0.034817	-3.07
266421_at	putative nonspecific lipid-transfer pr	LP1	AT2G38540	-2.71	-1.44	-3.65	0.008778	0.034817	-3.07
253628_at	xyloglucan endo-1,4-beta-D-glucar	XTH18	AT4G30280	-7.92	-2.99	-3.65	0.008784	0.034835	-3.07
252754_at	putative protein hypothetical protei	---	---	1.66	0.73	3.65	0.008788	0.034838	-3.07
265391_at	Expressed protein ; supported by c	GAUT10	AT2G20810	-1.27	-0.35	-3.65	0.008788	0.034838	-3.07
250243_at	cobalamin biosynthesis protein	GUN5	AT5G13630	-1.50	-0.58	-3.65	0.008796	0.034861	-3.07

252417_at	putative calcium-binding protein cal	---	AT3G47480	4.31	2.11	3.65	0.008797	0.034861	-3.07
245211_at	putative protein similarity to sec10,	SEC10	AT5G12370	-1.26	-0.33	-3.65	0.008799	0.034861	-3.07
254442_at	putative protein predicted protein T	---	AT4G21060	1.32	0.40	3.65	0.008802	0.034861	-3.07
245196_at	F12A21.12 similar to pectate lyase	---	AT1G67750	-1.98	-0.98	-3.65	0.008802	0.034861	-3.07
267166_at	unknown protein	---	AT2G37720	-1.24	-0.31	-3.65	0.008803	0.034861	-3.07
250397_at	histon H3 protein HISTONE H3.2,	---	AT5G10980	1.24	0.31	3.65	0.008805	0.034863	-3.07
253216_at	cyclophilin - like protein cyclophilin,	---	AT4G34960	1.42	0.50	3.65	0.008807	0.034864	-3.07
265544_at	putative cyclic nucleotide and calm	ATCNGC1	AT2G28260	-1.49	-0.57	-3.65	0.008812	0.034876	-3.07
263685_at	unknown protein similar to cell diffe	ATCUL3	AT1G26830	1.27	0.34	3.64	0.008818	0.034893	-3.07
258799_at	putative 40S ribosomal protein iden	RPSAb	AT3G04770	1.49	0.58	3.64	0.008821	0.034895	-3.07
263802_at	unknown protein ;supported by full-	---	AT2G40430	1.68	0.75	3.64	0.008823	0.034897	-3.07
256365_at	arginyl-tRNA synthetase nearly ider	---	AT1G66530	1.61	0.69	3.64	0.008827	0.034907	-3.08
251928_at	putative protein hypothetical protei	---	AT3G53980	-2.50	-1.32	-3.64	0.008829	0.034907	-3.08
245386_at	hypothetical protein ;supported by f	RALFL32	AT4G14010	-1.99	-0.99	-3.64	0.008843	0.034959	-3.08
248913_at	unknown protein ;supported by full-	---	AT5G45760	1.32	0.41	3.64	0.008849	0.034971	-3.08
247640_at	putative protein various predicted p	---	AT5G60610	1.34	0.42	3.64	0.008849	0.034971	-3.08
250591_at	alpha galactosyltransferase protein	---	AT5G07720	-1.42	-0.51	-3.64	0.008856	0.034985	-3.08
263428_at	ubiquitin-specific protease 4 (UBP4	ATUBP4	AT2G22310	1.37	0.45	3.64	0.008865	0.035012	-3.08
249198_s_a	putative protein similar to unknown	---	AT5G42350	-1.34	-0.42	-3.64	0.008867	0.035012	-3.08
252565_at	actin depolymerizing factor 2 (ADF2	ADF2	AT3G46000	1.27	0.35	3.64	0.008868	0.035012	-3.08
267092_at	unknown protein ;supported by full-	AUX1	AT2G38120	-1.97	-0.98	-3.64	0.008869	0.035012	-3.08
247134_at	putative protein similar to unknown	---	AT5G66230	-1.32	-0.40	-3.64	0.008881	0.035052	-3.08
267336_at	putative small heat shock protein ;s	---	AT2G19310	1.52	0.60	3.64	0.008892	0.035091	-3.08
253700_at	threonine synthase	MTO2	AT4G29840	1.56	0.64	3.64	0.008895	0.035096	-3.08
260497_at	40S ribosomal protein S2 ;support	---	AT2G41840	1.88	0.91	3.64	0.008912	0.035153	-3.09
266881_at	putative casein kinase II beta subur	CKB4	AT2G44680	1.34	0.42	3.64	0.008915	0.035159	-3.09
266542_at	unknown protein	ARAD1	AT2G35100	-1.29	-0.37	-3.64	0.008927	0.035199	-3.09
247333_at	1-aminocyclopropane-1-carboxylic a	FLS5	AT5G63600	1.90	0.92	3.63	0.008932	0.035213	-3.09
250168_at	putative protein predicted protein, A	---	AT5G15320	1.32	0.40	3.63	0.008935	0.035219	-3.09
252947_at	putative protein ubiquitin fusion deg	KAK	AT4G38600	1.27	0.34	3.63	0.008943	0.035243	-3.09
249482_at	putative protein ;supported by full-l	---	AT5G38980	2.00	1.00	3.63	0.008948	0.035253	-3.09
245303_at	hypothetical protein ; supported by	---	AT4G17010	1.29	0.37	3.63	0.008948	0.035253	-3.09
248717_at	Expressed protein ; supported by fu	---	AT5G48175	-1.26	-0.34	-3.63	0.00895	0.035253	-3.09
248586_at	putative protein similar to unknown	---	AT5G49610	1.36	0.44	3.63	0.008958	0.035279	-3.09
245005_at	ribosomal protein S14	---	---	-1.62	-0.70	-3.63	0.008975	0.035331	-3.09

258030_at	hypothetical protein predicted by ge	---	AT3G27590	-1.40	-0.49	-3.63	0.008979	0.035342	-3.09
247120_at	amino acid transporter protein-like	---	AT5G65990	1.33	0.41	3.63	0.008982	0.03535	-3.09
248523_s_a	ubiquitin activating enzyme ;suppo	SAE1B	AT5G50580	1.42	0.51	3.63	0.008987	0.035356	-3.09
257570_at	dirigent protein, putative similar to	---	AT3G13662	-1.32	-0.40	-3.63	0.008989	0.035358	-3.09
257638_at	terpene synthase-related protein co	ATTPS-CIN	AT3G25820	-1.31	-0.39	-3.63	0.008996	0.035378	-3.10
245411_at	hypothetical protein	---	AT4G17240	-1.67	-0.74	-3.63	0.009007	0.035414	-3.10
259286_at	hypothetical protein similar to hypot	BSMT1	AT3G11480	-11.30	-3.50	-3.63	0.009008	0.035414	-3.10
266948_at	unknown protein	---	AT2G18850	1.65	0.73	3.63	0.009012	0.03542	-3.10
250332_at	putative protein predicted proteins,	---	AT5G11680	-1.41	-0.50	-3.63	0.009013	0.03542	-3.10
246112_at	putative protein predicted proteins -	---	AT5G20200	1.41	0.49	3.63	0.00902	0.035441	-3.10
250284_at	protein kinase precursor - like rece	CRN	AT5G13290	1.55	0.63	3.63	0.009022	0.035446	-3.10
246130_at	putative protein various predicted p	---	---	-1.54	-0.62	-3.63	0.009035	0.035491	-3.10
246427_at	ADP/ATP translocase-like protein a	ER-ANT1	AT5G17400	-1.40	-0.49	-3.63	0.009046	0.035522	-3.10
267031_at	hypothetical protein predicted by ge	---	AT2G38420	1.30	0.38	3.62	0.009047	0.035522	-3.10
256055_at	mitochondrial carrier protein, putati	---	AT1G07030	1.31	0.39	3.62	0.009049	0.035522	-3.10
245425_at	carboxyl-terminal proteinase like pr	UCH3	AT4G17510	1.24	0.31	3.62	0.009051	0.035522	-3.10
246907_at	AtBgamma - like protein B' regulato	---	AT5G25510	-1.42	-0.50	-3.62	0.009051	0.035522	-3.10
262532_at	hypothetical protein predicted by ge	---	AT1G17210	1.34	0.42	3.62	0.009059	0.035545	-3.10
266164_at	putative salt-inducible protein	---	AT2G28050	-1.62	-0.70	-3.62	0.009065	0.035563	-3.10
251520_at	protein kinase like GCN2 - Saccha	---	AT3G59410	1.30	0.37	3.62	0.009067	0.035563	-3.10
250259_at	putative protein similar to unknown	---	AT5G13800	1.49	0.57	3.62	0.009068	0.035563	-3.10
247306_at	ser/thr protein phosphatase catalyti	PP7	AT5G63870	-1.42	-0.51	-3.62	0.009071	0.035569	-3.10
254633_at	putative protein (fragment)	MRH1	AT4G18640	-1.58	-0.66	-3.62	0.009074	0.035571	-3.10
254751_at	hypothetical protein	---	AT4G13150	1.30	0.38	3.62	0.009074	0.035571	-3.10
265403_at	putative Ta11-like non-LTR retroele	---	---	-1.38	-0.46	-3.62	0.009076	0.035571	-3.11
254019_at	putative protein mature-parasite-inf	---	AT4G26190	1.24	0.32	3.62	0.009079	0.035576	-3.11
264562_at	hypothetical protein predicted by ge	---	AT1G55760	1.76	0.82	3.62	0.009081	0.035578	-3.11
255244_at	putative protein various hypothetica	---	AT4G05620	-1.33	-0.41	-3.62	0.009085	0.035588	-3.11
266257_at	putative chorismate mutase/prephe	PD1	AT2G27820	-1.48	-0.57	-3.62	0.0091	0.035641	-3.11
247178_at	Expressed protein ; supported by cl	---	AT5G65205	1.95	0.97	3.62	0.009107	0.03566	-3.11
265274_at	putative RNA methyltransferase	---	AT2G28450	1.41	0.50	3.62	0.009108	0.03566	-3.11
244929_at	NADH dehydrogenase subunit 4	---	---	1.58	0.66	3.62	0.009119	0.03568	-3.11
247299_at	putative protein contains similarity t	---	AT5G63900	-1.29	-0.36	-3.62	0.00912	0.03568	-3.11
262262_at	unknown protein identical to unknow	---	AT1G70780	1.63	0.70	3.62	0.009122	0.03568	-3.11
246433_at	glutamine-rich protein predicted pr	---	AT5G17510	1.52	0.60	3.62	0.009122	0.03568	-3.11

254890_at	phospholipid hydroperoxide glutathi	ATGPX6	AT4G11600	1.34	0.43	3.62	0.009123	0.03568	-3.11
248329_at	putative protein similar to unknown	---	AT5G52780	1.52	0.60	3.62	0.009124	0.03568	-3.11
258587_at	hypothetical protein predicted by ge	---	AT3G04310	1.72	0.78	3.62	0.009126	0.035681	-3.11
252378_at	receptor kinase-like protein protein	---	AT3G47570	-1.58	-0.66	-3.62	0.009128	0.035684	-3.11
251533_at	putative protein several hypothetica	ATRBL15	AT3G58460	1.27	0.34	3.62	0.009141	0.035729	-3.11
254990_at	RNA-binding protein ;supported by	CID12	AT4G10610	1.26	0.33	3.62	0.00915	0.035756	-3.11
251214_at	putative protein hypothetical protein	---	AT3G62500	-1.32	-0.40	-3.62	0.00916	0.035791	-3.12
248531_at	putative protein contains similarity t	---	AT5G49950	1.35	0.43	3.61	0.009167	0.035806	-3.12
251834_at	ribosomal protein L35 -like various	---	AT3G55170	1.32	0.40	3.61	0.009169	0.035806	-3.12
264470_at	cytochrome P450, putative similar	CYP735A2	AT1G67110	-1.45	-0.54	-3.61	0.00917	0.035806	-3.12
250998_at	ankyrin - like protein ankyrin G119,	---	AT5G02620	1.52	0.61	3.61	0.009179	0.035834	-3.12
261999_at	hypothetical protein predicted by ge	---	AT1G33800	-1.42	-0.50	-3.61	0.009189	0.03586	-3.12
258514_at	unknown protein ; supported by ful	---	AT3G06610	1.29	0.37	3.61	0.009189	0.03586	-3.12
253555_at	putative protein predicted proetein,	---	AT4G31080	1.53	0.61	3.61	0.0092	0.035894	-3.12
251244_at	pectinacetylesterase precursor-like	---	AT3G62060	-1.37	-0.45	-3.61	0.009201	0.035894	-3.12
258448_at	unknown protein	---	AT3G22290	-1.27	-0.35	-3.61	0.009209	0.035919	-3.12
264108_at	hypothetical protein predicted by ge	CALS5	AT2G13680	-1.29	-0.37	-3.61	0.009211	0.035922	-3.12
258498_at	unknown protein similar to pollen co	---	AT3G02480	-1.93	-0.95	-3.61	0.00922	0.035949	-3.12
260565_at	unknown protein predicted by gene	---	AT2G43800	-1.67	-0.74	-3.61	0.009224	0.035961	-3.12
260950_s_a	delta 9 desaturase, putative similar	---	AT1G06090	-1.40	-0.49	-3.61	0.009236	0.036001	-3.12
246743_at	putative protein predicted proteins,	---	AT5G27750	1.38	0.46	3.61	0.009253	0.036059	-3.13
250131_at	unknown protein ; supported by cD	---	AT5G16550	-1.57	-0.65	-3.61	0.00926	0.036081	-3.13
252128_at	transcription factor-like protein Ara	MNP	AT3G50870	-1.30	-0.38	-3.61	0.009266	0.0361	-3.13
258430_at	phospholipase D, putative similar t	PLDP1	AT3G16785	-1.28	-0.36	-3.60	0.0093	0.036224	-3.13
264523_at	unknown protein Location of EST g	ERG28	AT1G10030	-1.29	-0.37	-3.60	0.009312	0.036264	-3.13
248865_at	putative protein similar to unknown	---	AT5G46790	1.47	0.55	3.60	0.009319	0.036285	-3.13
264163_at	Expressed protein ; supported by fu	---	AT1G65445	-1.39	-0.48	-3.60	0.009326	0.036294	-3.13
263274_at	putative protein kinase contains a p	CRCK3	AT2G11520	1.38	0.47	3.60	0.009326	0.036294	-3.13
251931_at	putative protein glyoxal oxidase (gl	---	AT3G53950	-1.61	-0.69	-3.60	0.009326	0.036294	-3.13
267207_at	putative dioxygenase	---	AT2G30840	2.02	1.01	3.60	0.009332	0.036314	-3.14
265614_at	Expressed protein ; supported by cl	---	AT2G25355	1.46	0.54	3.60	0.009345	0.036349	-3.14
251918_at	photoassimilate-responsive protein	---	AT3G54040	-1.30	-0.38	-3.60	0.009349	0.036358	-3.14
261620_s_a	ribosomal protein L9, putative simil	PGY2	AT1G33120	1.48	0.56	3.60	0.009354	0.036373	-3.14
257820_at	immunophilin (FKBP15-1) identical	FKBP15-1	AT3G25220	1.39	0.48	3.60	0.00936	0.036383	-3.14
250532_at	putative protein similar to unknown	---	AT5G08540	1.40	0.49	3.60	0.009362	0.036386	-3.14

257855_at	hypothetical protein predicted by ge	---	AT3G13040	1.31	0.39	3.60	0.009377	0.036436	-3.14
248999_at	putative protein contains similarity t	---	AT5G44970	-1.29	-0.37	-3.60	0.009381	0.036448	-3.14
264686_at	shaggy-like protien kinase, kappa I	ATSK41	AT1G09840	-1.26	-0.34	-3.60	0.009385	0.036456	-3.14
256920_at	hypothetical protein predicted by ge	---	AT3G18980	-1.27	-0.35	-3.60	0.009396	0.036492	-3.14
258358_at	unknown protein similar to unknowr	---	AT3G14380	-1.35	-0.44	-3.60	0.009398	0.036492	-3.14
259695_at	hypothetical protein predicted by ge	---	AT1G63170	-1.40	-0.48	-3.60	0.0094	0.036492	-3.14
261139_at	homeodomain protein, putative sim	BEL10	AT1G19700	1.29	0.37	3.60	0.009401	0.036492	-3.14
255468_at	putative WD-repeat protein similar	---	AT4G03020	1.31	0.39	3.60	0.009403	0.036497	-3.14
246503_at	40S ribosomal protein S7-like 40S	---	AT5G16130	1.75	0.81	3.59	0.009413	0.03653	-3.14
260165_at	30S ribosomal protein S17, chlorop	RPS17	AT1G79850	-1.49	-0.57	-3.59	0.009429	0.036577	-3.15
246759_at	putative protein kinesin heavy chai	---	AT5G27950	1.48	0.57	3.59	0.009432	0.036583	-3.15
245387_at	nuclear RNA binding protein A-like	---	AT4G17520	1.46	0.54	3.59	0.009447	0.036634	-3.15
263881_at	unknown protein ; supported by cD	---	AT2G21820	1.33	0.41	3.59	0.00945	0.036638	-3.15
264362_at	unknown protein ESTs gb|H36966,	---	AT1G03290	1.54	0.62	3.59	0.009462	0.036672	-3.15
250600_at	dimethylaniline monooxygenase-like	---	AT5G07800	-1.31	-0.39	-3.59	0.009464	0.036672	-3.15
254089_at	putative protein apoptosis gene MA	---	AT4G24800	-1.35	-0.43	-3.59	0.009465	0.036672	-3.15
250420_at	HY5(bZIP transcription factor family	HY5	AT5G11260	1.40	0.48	3.59	0.009465	0.036672	-3.15
267215_at	hypothetical protein	---	AT2G02630	-1.25	-0.32	-3.59	0.00948	0.036721	-3.15
251041_at	ubiquitin-protein ligase E3-alpha -li	PRT6	AT5G02310	-1.49	-0.57	-3.59	0.009481	0.036721	-3.15
248497_at	putative protein strong similarity to u	ATEXO70F	AT5G50380	1.56	0.64	3.59	0.009487	0.036737	-3.15
255319_at	putative disease resistance protein	AtRLP46	AT4G04220	1.84	0.88	3.59	0.009488	0.036737	-3.15
256479_at	unknown protein	---	AT1G33400	1.47	0.56	3.59	0.009496	0.036764	-3.15
245044_at	unknown protein Alternative splice f	---	AT2G26500	1.25	0.33	3.59	0.009513	0.036821	-3.16
261806_at	ferrodoxin NADP oxidoreductase, p	ATRFNR2	AT1G30510	1.74	0.80	3.59	0.00952	0.036841	-3.16
251136_at	mRNA capping enzyme - like prote	---	AT5G01290	1.27	0.35	3.59	0.009524	0.036848	-3.16
261305_at	glutamine synthetase, putative sim	GLN1;5	AT1G48470	1.27	0.34	3.59	0.009525	0.036848	-3.16
252713_at	calmodulin 7 ; supported by full-len	CAM7	AT3G43810	-1.35	-0.44	-3.59	0.009526	0.036848	-3.16
247058_at	dolichyl-di-phosphooligosaccharide-	DGL1	AT5G66680	1.39	0.47	3.58	0.009529	0.036853	-3.16
252457_at	zinc-finger protein-like protein DNA	---	AT3G47180	-1.29	-0.37	-3.58	0.009533	0.036861	-3.16
250111_at	acetolactate synthase-like protein	---	AT5G16290	-1.54	-0.62	-3.58	0.009556	0.03694	-3.16
256485_at	unknown protein	---	AT1G31440	1.32	0.40	3.58	0.009559	0.03694	-3.16
263709_at	unknown protein ESTs gb|T20589,	---	AT1G09310	-4.60	-2.20	-3.58	0.00956	0.03694	-3.16
263067_at	hypothetical protein predicted by ge	---	AT2G17550	-1.64	-0.71	-3.58	0.00957	0.036971	-3.16
261624_at	nucleotide sugar epimerase, putati	GAE2	AT1G02000	-1.68	-0.75	-3.58	0.009571	0.036971	-3.16
257313_at	gamma tonoplast intrinsic protein no	TIP2	AT3G26520	-1.43	-0.52	-3.58	0.009573	0.036974	-3.16

245895_at	SIR2-family protein sirtuin type 4 (S	SRT2	AT5G09230	-1.35	-0.43	-3.58	0.00958	0.036994	-3.16
267456_at	E2, ubiquitin-conjugating enzyme,	PHO2	AT2G33770	1.89	0.92	3.58	0.009585	0.037007	-3.16
252616_at	serine threonine-protein kinase-like	GRIK1	AT3G45240	1.43	0.51	3.58	0.009595	0.037039	-3.16
259085_at	unknown protein similar to GB:CAB	---	AT3G05000	-1.33	-0.42	-3.58	0.009607	0.037078	-3.17
261846_at	hypothetical protein similar to hypot	---	AT1G11540	-1.36	-0.45	-3.58	0.00961	0.037085	-3.17
263239_at	amidophosphoribosyltransferase ide	ATASE1	AT2G16570	-1.37	-0.45	-3.58	0.009615	0.037097	-3.17
260152_at	putative IAA6 protein similar to IAA6	IAA6	AT1G52830	-1.92	-0.94	-3.58	0.009621	0.037116	-3.17
256332_at	trihelix DNA-binding protein (GT2)	GT2	AT1G76890	-1.93	-0.95	-3.58	0.009639	0.037178	-3.17
249119_at	unknown protein ;supported by full-	---	AT5G43720	1.25	0.32	3.58	0.009651	0.037217	-3.17
258312_at	putative long-chain acyl-CoA synthe	---	AT3G16170	1.51	0.59	3.57	0.009664	0.037261	-3.17
250634_at	putative protein 2-oxoglutarate-dep	---	AT5G07480	-1.29	-0.37	-3.57	0.009666	0.037263	-3.17
247595_at	ring finger protein - like ring finger p	---	AT5G60820	-1.45	-0.54	-3.57	0.009668	0.037263	-3.17
261265_at	hypothetical protein predicted by ge	---	AT1G26800	-1.67	-0.74	-3.57	0.00968	0.037302	-3.17
246837_at	pectin acetylesterase precursor - lil	---	AT5G26670	-1.64	-0.72	-3.57	0.009682	0.037304	-3.17
257151_at	blue copper protein, putative simila	---	AT3G27200	-1.43	-0.52	-3.57	0.009696	0.037346	-3.18
254753_at	putative protein Pto kinase interact	---	AT4G13190	-1.28	-0.35	-3.57	0.009698	0.037348	-3.18
254602_at	hypothetical protein	EDR2	AT4G19040	1.32	0.40	3.57	0.009715	0.037407	-3.18
251992_at	putative protein myosin heavy chai	---	AT3G53350	1.48	0.57	3.57	0.009723	0.03743	-3.18
254481_at	hypothetical protein ; supported by	---	AT4G20480	1.55	0.63	3.57	0.009726	0.037432	-3.18
251461_at	putative protein	---	AT3G59780	-1.43	-0.52	-3.57	0.009726	0.037432	-3.18
259492_at	hypothetical protein predicted by ge	---	AT1G15830	-1.59	-0.67	-3.57	0.009741	0.037482	-3.18
267229_s_a	putative translation initiation factor e	---	AT2G44070	1.79	0.84	3.57	0.009753	0.037513	-3.18
249086_at	putative protein strong similarity to u	---	AT5G44180	1.34	0.42	3.57	0.009754	0.037513	-3.18
253198_at	putative protein various predicted p	---	AT4G35360	-1.34	-0.42	-3.57	0.009763	0.037542	-3.18
260732_at	myb-related DNA-binding protein, p	---	AT1G17520	1.30	0.37	3.57	0.009768	0.037549	-3.18
255019_at	putative protein IB1C3-1 protein, A	---	AT4G10210	-1.30	-0.38	-3.57	0.009768	0.037549	-3.18
262499_at	unknown protein EST gb|T41993 co	---	AT1G21770	1.48	0.57	3.57	0.009772	0.037559	-3.18
258657_at	hypothetical protein predicted by ge	---	AT3G09890	1.52	0.61	3.57	0.009774	0.037559	-3.18
266832_at	putative protein kinase contains a p	MAPKKK14	AT2G30040	1.57	0.65	3.57	0.009777	0.037562	-3.18
266847_at	hypothetical protein predicted by gr	emb1345	AT2G26060	1.42	0.51	3.56	0.009782	0.037578	-3.19
266122_at	putative helicase ; supported by cD	ETL1	AT2G02090	1.31	0.39	3.56	0.009786	0.037585	-3.19
264383_at	putative glutathione peroxidase Co	ATGPX1	AT2G25080	-1.43	-0.51	-3.56	0.009791	0.037598	-3.19
259795_at	hypothetical protein predicted by ge	---	AT1G64290	-1.37	-0.45	-3.56	0.009797	0.037602	-3.19
258201_at	hypothetical protein predicted by ge	---	AT3G13910	1.74	0.80	3.56	0.009797	0.037602	-3.19
247932_at	putative protein similar to unknown	---	AT5G56920	-1.27	-0.34	-3.56	0.009812	0.037651	-3.19

264840_at	unknown protein similar to leucine-r	---	AT1G03440	-1.30	-0.38	-3.56	0.009815	0.037658	-3.19
259924_at	putative DNA-binding protein simila	---	AT1G72740	1.37	0.46	3.56	0.009818	0.037665	-3.19
249265_at	E2, ubiquitin-conjugating enzyme 8	---	AT5G41080	1.25	0.33	3.56	0.009832	0.037713	-3.19
266807_at	hypothetical protein	---	AT2G29920	-1.41	-0.49	-3.56	0.009841	0.03774	-3.19
255384_at	hypothetical protein	---	AT4G03580	-1.26	-0.33	-3.56	0.009843	0.037742	-3.19
253559_at	1,3-beta-glucanase - like protein 1,	---	AT4G31140	-1.31	-0.39	-3.56	0.009884	0.037892	-3.20
249769_at	sigma-like factor (emb CAA77213.1	SIGE	AT5G24120	1.50	0.59	3.56	0.009889	0.037899	-3.20
255780_at	hypothetical protein contains Pfam	GAUT11	AT1G18580	-1.40	-0.49	-3.56	0.009891	0.037899	-3.20
260798_at	unknown protein contains similarity	---	AT1G78280	-1.58	-0.66	-3.56	0.009891	0.037899	-3.20
245149_at	putative SWI/SNF family transcriptio	ATSWI3A	AT2G47620	1.35	0.43	3.56	0.009894	0.037899	-3.20
263686_at	putative 60s ribosomal protein L10	---	AT1G26910	1.57	0.65	3.56	0.009894	0.037899	-3.20
253047_at	Expressed protein ; supported by fu	---	AT4G37295	1.67	0.74	3.56	0.009898	0.037904	-3.20
251804_at	beta-1,3-glucanase - like protein pr	---	AT3G55430	-1.50	-0.59	-3.56	0.009899	0.037904	-3.20
250109_at	GASA4 ; supported by cDNA: gi_9	GASA4	AT5G15230	-5.25	-2.39	-3.56	0.009907	0.037925	-3.20
261608_at	unknown protein similar to putative	---	AT1G49650	-1.36	-0.44	-3.55	0.009909	0.037925	-3.20
260264_at	hypothetical protein predicted by ge	---	AT1G68500	-1.50	-0.58	-3.55	0.009909	0.037925	-3.20
245358_at	PRL1 protein ;supported by full-leng	PRL1	AT4G15900	1.39	0.47	3.55	0.009911	0.037926	-3.20
258482_at	putative chaperonin similar to chape	---	AT3G02530	1.40	0.48	3.55	0.009918	0.037944	-3.20
251258_at	PECTINESTERASE-like protein PE	VGDH2	AT3G62170	-1.39	-0.48	-3.55	0.009935	0.037998	-3.20
247859_at	unknown protein	---	AT5G58410	1.24	0.32	3.55	0.009943	0.038021	-3.20
248199_at	membrane related protein-like ;sup	---	AT5G54170	-1.86	-0.90	-3.55	0.009954	0.038053	-3.20
255043_at	putative protein several hypothetica	---	AT4G09640	-1.65	-0.72	-3.55	0.009955	0.038053	-3.20
261199_at	unknown protein	---	AT1G12950	-1.26	-0.33	-3.55	0.009958	0.038062	-3.20
254422_at	putative protein hypothetical protei	VPS28-1	AT4G21560	-1.72	-0.78	-3.55	0.009961	0.038064	-3.20
266308_at	putative cytochrome P450	CYP705A9	AT2G27010	1.38	0.47	3.55	0.009962	0.038064	-3.21
253270_at	cyclin delta-3	CYCD3;1	AT4G34160	-1.53	-0.62	-3.55	0.009967	0.038077	-3.21
246897_at	putative protein predicted proteins,	---	AT5G25560	-1.40	-0.48	-3.55	0.009969	0.038078	-3.21
263687_at	unknown protein ;supported by full-	---	AT1G26940	1.50	0.58	3.55	0.009972	0.038083	-3.21
258551_at	hypothetical protein predicted by ge	---	AT3G06890	-1.25	-0.32	-3.55	0.009977	0.038087	-3.21
253567_at	protein kinase-like protein serine/th	---	AT4G31230	-1.45	-0.54	-3.55	0.009977	0.038087	-3.21
249472_at	hypothetical protein ;supported by f	CRR7	AT5G39210	1.65	0.73	3.55	0.009993	0.038144	-3.21
245803_at	cysteine proteinase RD21A identica	RD21	AT1G47128	1.43	0.52	3.55	0.009998	0.038146	-3.21
249777_at	putative protein similar to unknown	---	AT5G24210	-1.47	-0.55	-3.55	0.009998	0.038146	-3.21
257362_at	hypothetical protein predicted by ge	---	AT2G45840	-1.26	-0.33	-3.55	0.009999	0.038146	-3.21
258906_at	unknown protein similar to C-termin	ATTLP9	AT3G06380	1.39	0.48	3.55	0.010003	0.038156	-3.21

254957_at	putative protein ; supported by full-	---	AT4G10970	1.37	0.45	3.55	0.010006	0.038161	-3.21
262586_at	hypothetical protein predicted by ge	---	AT1G15480	1.63	0.70	3.55	0.010008	0.038161	-3.21
267132_at	unknown protein	NAPRT2	AT2G23420	1.70	0.76	3.55	0.010018	0.03819	-3.21
260209_at	putative AP2 domain transcription fa	CPuORF53	AT1G68550	1.37	0.46	3.55	0.010019	0.03819	-3.21
258329_at	putative protein disulfide isomerase	ATPDIL1-6	AT3G16110	1.32	0.40	3.54	0.01004	0.038263	-3.21
258860_at	putative potassium transporter simil	KUP3	AT3G02050	1.62	0.70	3.54	0.010047	0.038285	-3.21
264983_at	hypothetical protein predicted by ge	---	AT1G27240	-1.29	-0.37	-3.54	0.010049	0.038286	-3.21
259352_at	unknown protein	---	AT3G05170	-1.65	-0.72	-3.54	0.010051	0.038286	-3.21
246236_at	hypothetical protein	---	AT4G36470	-1.39	-0.47	-3.54	0.010053	0.038286	-3.21
265000_at	hypothetical protein predicted by ge	---	AT1G67410	-1.35	-0.44	-3.54	0.010054	0.038286	-3.21
263042_at	conserved hypothetical protein prot	---	AT1G23340	-1.37	-0.46	-3.54	0.010058	0.038295	-3.22
252878_at	mitochondrial carrier - like protein A	SAMC1	AT4G39460	-1.33	-0.41	-3.54	0.010075	0.038345	-3.22
260966_at	NBS/LRR disease resistance prote	RPS5	AT1G12220	1.33	0.41	3.54	0.01008	0.038354	-3.22
257187_at	hypothetical protein predicted by ge	---	AT3G13140	-1.31	-0.39	-3.54	0.010082	0.038354	-3.22
264283_at	hypothetical protein contains similar	---	AT1G61850	-1.33	-0.42	-3.54	0.010093	0.038388	-3.22
258550_at	putative mudrA protein similar to mu	---	---	1.37	0.45	3.54	0.010128	0.038513	-3.22
261002_at	hypothetical protein contains similar	---	AT1G26520	1.33	0.41	3.54	0.010129	0.038513	-3.22
246483_at	receptor protein kinase-like protein	NIK1	AT5G16000	-1.94	-0.96	-3.54	0.010137	0.038538	-3.22
257075_at	unknown protein contains Pfam pro	---	AT3G19670	1.46	0.54	3.54	0.010141	0.038548	-3.22
263793_at	putative glucosyltransferase	ATCSLC08	AT2G24630	-1.79	-0.84	-3.54	0.010143	0.038548	-3.22
262992_at	hypothetical protein predicted by ge	ATG12A	AT1G54210	1.48	0.56	3.54	0.01015	0.038566	-3.23
267355_at	putative LIM-domain protein	---	AT2G39900	-1.48	-0.57	-3.54	0.010154	0.038578	-3.23
256250_at	integral membrane protein, putative	---	AT3G11320	-1.46	-0.55	-3.54	0.010163	0.038604	-3.23
259368_at	aspartic protease, putative similar t	---	AT1G69100	-1.30	-0.38	-3.54	0.01017	0.038623	-3.23
252038_at	gamma response I protein ; suppor	---	AT3G52120	-1.46	-0.55	-3.53	0.010172	0.038623	-3.23
267009_at	unknown protein	---	AT2G39260	1.27	0.35	3.53	0.010174	0.038623	-3.23
266804_at	cyclophilin identical to GB:AF02043	CYP5	AT2G29960	1.35	0.43	3.53	0.010175	0.038623	-3.23
253734_at	putative serine/threonine-specific re	---	AT4G29180	-1.34	-0.42	-3.53	0.010179	0.038634	-3.23
248901_at	putative protein contains similarity t	---	AT5G46410	1.40	0.49	3.53	0.010186	0.038652	-3.23
246420_at	putative protein various predicted p	---	AT5G16870	-1.41	-0.49	-3.53	0.010187	0.038652	-3.23
255194_at	score=37.6, E=2.9e-07, N=3	---	AT4G07410	1.53	0.62	3.53	0.010193	0.038667	-3.23
245676_at	nodule-specific protein, putative sir	---	AT1G56670	-1.35	-0.43	-3.53	0.010197	0.038676	-3.23
259416_at	Expressed protein ; supported by cl	---	AT1G02305	1.44	0.52	3.53	0.010207	0.0387	-3.23
259202_at	putative mRNA capping enzyme, R	---	AT3G09100	1.35	0.44	3.53	0.010207	0.0387	-3.23
250461_at	hypothetical protein	---	AT5G10010	1.43	0.51	3.53	0.010211	0.038706	-3.23

253953_at	putative protein extensin precursor	---	AT4G26750	-1.57	-0.65	-3.53	0.010213	0.038706	-3.23
251759_at	tetrahydrofolylpolyglutamate syntha	ATDFD	AT3G55630	1.69	0.75	3.53	0.010214	0.038706	-3.23
251289_at	auxin response factor-like protein a	ARF18	AT3G61830	1.44	0.53	3.53	0.010215	0.038706	-3.23
263322_at	putative ribonuclease E	RNEE/G	AT2G04270	1.59	0.67	3.53	0.01022	0.038719	-3.23
246693_at	putative protein	---	AT5G29070	-1.30	-0.38	-3.53	0.010232	0.038758	-3.23
253527_at	pathogenesis-related protein homo	---	AT4G31470	-1.34	-0.42	-3.53	0.010234	0.038759	-3.23
246612_at	putative protein ;supported by full-l	---	AT5G35320	1.40	0.49	3.53	0.010245	0.038792	-3.24
252769_at	arabinose kinase - like protein puta	---	AT3G42850	-1.31	-0.39	-3.53	0.010256	0.038828	-3.24
249925_at	putative protein predicted proteins,	---	AT5G19150	1.50	0.58	3.53	0.01027	0.038875	-3.24
256645_at	hypothetical protein predicted by ge	---	AT3G24250	-1.27	-0.35	-3.53	0.010272	0.038875	-3.24
259119_at	transport protein SEC13, putative s	---	AT3G01340	-1.27	-0.34	-3.53	0.010292	0.038939	-3.24
255591_at	putative expansin	ATEXPA17	AT4G01630	-1.33	-0.41	-3.53	0.010296	0.038946	-3.24
265723_at	putative disease resistance protein	---	AT2G32140	-1.26	-0.34	-3.53	0.010302	0.038965	-3.24
267469_at	unknown protein ; supported by cD	WRKY21	AT2G30590	-1.36	-0.44	-3.52	0.010306	0.038971	-3.24
253017_at	cinnamyl alcohol dehydrogenase -l	CAD6	AT4G37970	-1.27	-0.34	-3.52	0.010309	0.038973	-3.24
258805_at	putative beta-1,3-glucanase similar	---	AT3G04010	1.52	0.60	3.52	0.01031	0.038973	-3.24
252456_at	hypersensitivity-related protein-like	---	AT3G47170	-1.34	-0.42	-3.52	0.010315	0.038988	-3.24
253037_at	putative protein other predicted Ara	GAUT3	AT4G38270	1.32	0.40	3.52	0.01032	0.038995	-3.24
257199_at	Tic22, putative similar to Tic22 GB	---	AT3G23710	1.38	0.47	3.52	0.010321	0.038995	-3.24
259055_at	unknown protein ;supported by full-	UNE6	AT3G03340	1.31	0.39	3.52	0.010322	0.038995	-3.24
259255_at	putative glycerol-3-phosphate dehy	---	AT3G07690	1.69	0.76	3.52	0.010328	0.03901	-3.24
250343_at	putative protein density regulated p	---	AT5G11900	1.32	0.40	3.52	0.010338	0.03904	-3.24
261050_at	transcription factor MYC7E, putativ	---	AT1G01260	-1.58	-0.66	-3.52	0.010339	0.03904	-3.24
259631_at	heat shock protein, putative similar	ERD2	AT1G56410	-1.27	-0.34	-3.52	0.010347	0.039062	-3.25
255928_at	unknown protein	---	AT1G12640	1.55	0.63	3.52	0.01036	0.039103	-3.25
259412_at	DNA binding protein GT-1, putative	---	AT1G13450	-1.32	-0.40	-3.52	0.010365	0.039113	-3.25
246040_at	peptidyl-prolyl cis-trans isomerase -	---	AT5G19370	-1.32	-0.40	-3.52	0.010366	0.039113	-3.25
253058_at	ribosomal - like protein ribosomal p	---	AT4G37660	1.52	0.60	3.52	0.010368	0.039115	-3.25
247637_at	putative protein gcpE protein, Chla	HDS	AT5G60600	1.46	0.55	3.52	0.010376	0.03914	-3.25
253619_at	glycine-rich protein glycine-rich cel	---	AT4G30460	-1.50	-0.59	-3.52	0.010382	0.039155	-3.25
254112_at	putative protein KIAA0136 gene, H	---	AT4G24970	1.32	0.40	3.52	0.010388	0.039165	-3.25
248708_at	putative protein contains similarity t	---	AT5G48560	-1.86	-0.89	-3.52	0.010388	0.039165	-3.25
257648_at	RNA helicase, putative similar to R	---	AT3G16840	1.65	0.72	3.52	0.010397	0.039194	-3.25
248558_at	permease	---	AT5G49990	1.58	0.66	3.52	0.010399	0.039194	-3.25
248792_at	ras-related small GTP-binding prote	ATRAB1A	AT5G47200	-1.34	-0.43	-3.52	0.010403	0.039203	-3.25

259327_at	putative lectin contains Pfam profil	---	AT3G16460	-3.38	-1.76	-3.52	0.010406	0.039207	-3.25
250318_at	dihydropyrimidinase ; supported by	---	AT5G12200	1.52	0.61	3.52	0.010413	0.03922	-3.25
246056_at	katanin p80 subunit - like protein k	---	AT5G08390	-1.40	-0.48	-3.52	0.010416	0.039225	-3.25
266230_at	hypothetical protein predicted by ge	---	AT2G28830	1.74	0.80	3.52	0.010417	0.039225	-3.25
248720_at	protein kinase (EC 2.7.1.37) 5 (pir||	D6PKL2	AT5G47750	1.31	0.39	3.52	0.010427	0.039254	-3.25
252106_at	putative glutamate receptor putativ	ATGLR3.6	AT3G51480	-1.46	-0.55	-3.52	0.010439	0.039291	-3.26
261771_at	hypothetical protein contains simila	---	AT1G76150	1.24	0.31	3.51	0.010441	0.039293	-3.26
267157_at	putative MYB family transcription fa	AS1	AT2G37630	1.56	0.64	3.51	0.010451	0.039325	-3.26
250305_at	putative protein contains similarity t	---	AT5G12150	-1.41	-0.50	-3.51	0.010454	0.039325	-3.26
248298_at	putative protein similar to unknown	---	AT5G53110	1.59	0.67	3.51	0.010455	0.039325	-3.26
257707_at	hypothetical protein predicted by ge	---	AT3G12650	1.29	0.37	3.51	0.01046	0.03934	-3.26
255501_at	hypothetical protein	---	AT4G02400	1.54	0.62	3.51	0.010473	0.03938	-3.26
251885_at	fructose-bisphosphatase precursor	---	AT3G54050	-1.57	-0.65	-3.51	0.010485	0.039421	-3.26
266033_at	putative translation initiation factor e	---	AT2G05830	1.43	0.51	3.51	0.010488	0.039427	-3.26
247266_at	beta-xylosidase	XYL4	AT5G64570	1.63	0.71	3.51	0.010493	0.039439	-3.26
265123_at	hypothetical protein similar to putati	---	AT1G55440	-1.27	-0.35	-3.51	0.010495	0.039439	-3.26
266315_at	unknown protein ; supported by cD	SE	AT2G27100	1.27	0.34	3.51	0.010498	0.03944	-3.26
248019_at	putative protein similar to unknown	---	AT5G56480	-1.32	-0.40	-3.51	0.0105	0.03944	-3.26
267525_at	putative glycine-rich protein	---	AT2G30560	-1.24	-0.31	-3.51	0.010502	0.03944	-3.26
261370_at	cell division-related protein, putativ	---	AT1G53050	-1.30	-0.38	-3.51	0.010502	0.03944	-3.26
252450_s_a	receptor kinase-like protein recepto	---	AT3G47090	1.77	0.82	3.51	0.010504	0.039441	-3.26
257233_at	hypothetical protein similar to SF16	IQD10	AT3G15050	-1.30	-0.38	-3.51	0.010518	0.039485	-3.26
260734_at	disease resistance protein RPP1-W	---	AT1G17600	-1.41	-0.50	-3.51	0.010537	0.039552	-3.27
250483_at	alpha-hydroxynitrile lyase-like prote	MES5	AT5G10300	1.48	0.57	3.51	0.010552	0.039601	-3.27
248871_at	putative protein strong similarity to u	---	AT5G46680	1.36	0.44	3.51	0.010558	0.039616	-3.27
259463_at	hypothetical protein predicted by ge	---	AT1G18950	1.63	0.71	3.51	0.010562	0.039626	-3.27
262900_at	hypothetical protein contains simila	SNL5	AT1G59890	1.39	0.47	3.50	0.010588	0.039708	-3.27
255665_at	hypothetical protein	CRP	AT4G00450	1.29	0.36	3.50	0.010595	0.03973	-3.27
247142_at	putative protein strong similarity to u	---	AT5G65570	1.34	0.42	3.50	0.010604	0.039756	-3.27
248387_at	unknown protein	---	AT5G51960	1.38	0.47	3.50	0.010606	0.039756	-3.27
259566_at	hypothetical protein	---	AT1G20520	-1.27	-0.34	-3.50	0.010612	0.039775	-3.27
261626_at	unknown protein ; supported by cDI	---	AT1G01990	1.40	0.48	3.50	0.010636	0.039858	-3.28
255810_at	hypothetical protein ; supported by	---	AT4G10140	-1.32	-0.40	-3.50	0.010652	0.039909	-3.28
247744_at	putative protein various predicted p	---	AT5G59020	-1.45	-0.54	-3.50	0.010657	0.039922	-3.28
247970_at	cytosolic malate dehydrogenase	---	AT5G56720	-1.30	-0.38	-3.50	0.010663	0.039938	-3.28

246815_at	putative protein integrin analogue,	---	AT5G27220	1.27	0.35	3.50	0.010665	0.039939	-3.28
255982_at	hypothetical protein contains similar	OHP2	AT1G34000	-1.30	-0.38	-3.50	0.010669	0.039946	-3.28
259588_at	unknown protein ;supported by full-	---	AT1G27930	1.24	0.31	3.50	0.010673	0.039953	-3.28
253714_at	putative protein adaptor protein - H	---	AT4G29380	1.32	0.40	3.50	0.010674	0.039953	-3.28
246385_at	mitotic cyclin a2-type, putative simi	CYCA1;2	AT1G77390	-1.98	-0.98	-3.50	0.010686	0.039992	-3.28
257733_at	unknown protein ; supported by cD	---	AT3G18350	-1.36	-0.44	-3.50	0.010697	0.040026	-3.28
259395_at	GTP-binding protein(RAB11D), put	ARA-2	AT1G06400	-1.30	-0.38	-3.50	0.010699	0.040026	-3.28
265699_at	putative esterase (contains an ester	---	AT2G03550	1.34	0.42	3.50	0.010713	0.040068	-3.28
265112_at	hypothetical protein predicted by ge	---	AT1G62530	-1.25	-0.32	-3.50	0.010713	0.040068	-3.28
260680_s_a	GATA transcription factor 3, putativ	---	AT1G08000	-1.26	-0.33	-3.50	0.010717	0.040073	-3.28
262476_at	phosphoprotein phosphatase identi	---	AT1G50370	1.30	0.38	3.49	0.010721	0.040081	-3.28
246240_at	nucleoporin-like protein	---	AT4G37130	1.25	0.32	3.49	0.010725	0.040092	-3.28
256573_at	hypothetical protein predicted by ge	---	AT3G14860	1.32	0.40	3.49	0.010733	0.040114	-3.28
259389_at	hypothetical protein predicted by ge	---	AT1G06320	-1.28	-0.36	-3.49	0.010735	0.040114	-3.29
246617_at	GSH-dependent dehydroascorbate	---	AT5G36270	-1.34	-0.42	-3.49	0.010741	0.040132	-3.29
255558_at	P II nitrogen sensing protein GLB I	GLB1	AT4G01900	-1.36	-0.45	-3.49	0.01075	0.04016	-3.29
258372_at	ubiquitin-specific protease 25 (UBP	UBP25	AT3G14400	1.29	0.37	3.49	0.010757	0.040179	-3.29
261852_at	unknown protein ;supported by full-	---	AT1G50440	-1.26	-0.33	-3.49	0.010761	0.040187	-3.29
264242_at	hypothetical protein predicted by ge	---	AT1G54640	-1.24	-0.31	-3.49	0.010765	0.040187	-3.29
261614_at	poly(A)-binding protein, putative sir	PAB8	AT1G49760	1.48	0.56	3.49	0.010766	0.040187	-3.29
262143_at	unknown protein	---	AT1G52630	-1.34	-0.43	-3.49	0.010767	0.040187	-3.29
247935_at	30S ribosomal protein S16 ; suppo	---	AT5G56940	1.46	0.54	3.49	0.010779	0.040223	-3.29
253366_at	putative protein predicted protein, A	---	AT4G33180	-1.44	-0.53	-3.49	0.01078	0.040223	-3.29
256914_at	hypothetical protein	---	AT3G23880	-1.57	-0.65	-3.49	0.010784	0.040232	-3.29
248429_at	putative protein contains similarity t	---	AT5G51770	1.79	0.84	3.49	0.010796	0.040271	-3.29
267445_at	hypothetical protein predicted by ge	---	AT2G33680	1.36	0.44	3.49	0.010799	0.040271	-3.29
247158_at	branched-chain amino acid aminotr	ATBCAT-5	AT5G65780	1.29	0.37	3.49	0.010801	0.040271	-3.29
265012_at	putative b-keto acyl reductase simi	KCR2	AT1G24470	-1.33	-0.41	-3.49	0.010801	0.040271	-3.29
259706_at	unknown protein similar to GB:AAD	---	AT1G77540	1.38	0.46	3.49	0.01081	0.040293	-3.29
256587_at	histone-H4-like protein similar to H4	---	AT3G28780	-1.34	-0.43	-3.49	0.010811	0.040293	-3.29
248636_at	putative protein similar to unknown	---	---	-1.33	-0.41	-3.49	0.010814	0.040293	-3.29
258959_at	putative amino acid transporter sim	CAT7	AT3G10600	-1.31	-0.39	-3.49	0.010816	0.040293	-3.29
257198_at	DNA-binding protein, putative simil	---	AT3G23690	-1.38	-0.47	-3.49	0.010817	0.040293	-3.29
251480_at	putative protein inducible carbonyl	---	AT3G59710	-1.44	-0.53	-3.49	0.010818	0.040293	-3.29
246776_at	kinesin-like protein kinesin-like hea	---	AT5G27550	-1.49	-0.57	-3.49	0.010824	0.04031	-3.29

255792_at	AT-hook DNA-binding protein (AHP	---	AT2G33620	1.42	0.50	3.49	0.010833	0.040335	-3.29
255599_at	cyclic nucleotide gated channel (CI	ATCNGC13	AT4G01010	1.76	0.82	3.49	0.010834	0.040335	-3.29
252959_at	putative protein NADH dehydrogen	---	AT4G38640	1.32	0.41	3.49	0.010838	0.040342	-3.30
263286_at	40S ribosomal protein S14 ;suppor	---	AT2G36160	1.54	0.62	3.49	0.010856	0.040402	-3.30
251730_at	N2,N2-dimethylguanosine tRNA m	---	AT3G56330	1.42	0.50	3.48	0.01088	0.040478	-3.30
247613_at	xABC transporter - like protein brea	---	AT5G60740	-1.39	-0.48	-3.48	0.01088	0.040478	-3.30
259466_at	response regulator 5, putative simi	ARR7	AT1G19050	-2.83	-1.50	-3.48	0.010899	0.040535	-3.30
266073_at	putative signal recognition particle r	---	AT2G18770	-1.31	-0.39	-3.48	0.010917	0.040597	-3.30
266511_at	putative ATP-dependent RNA helica	---	AT2G47680	1.40	0.48	3.48	0.010932	0.040634	-3.30
259198_at	hypothetical protein similar to F1N2	---	AT3G03610	-1.34	-0.42	-3.48	0.010932	0.040634	-3.30
249647_at	putative protein hypothetical protein	---	AT5G37040	-1.42	-0.51	-3.48	0.010934	0.040634	-3.30
252324_at	protein translocation complex sec6	---	AT3G48570	-1.36	-0.44	-3.48	0.010952	0.040693	-3.31
252135_at	putative cold acclimation protein co	COR413-P	AT3G50830	-1.48	-0.56	-3.48	0.010958	0.040707	-3.31
250955_at	putative protein various predicted p	CPUORF47	AT5G03190	-2.90	-1.54	-3.48	0.010963	0.040711	-3.31
257671_at	hypothetical protein predicted by ge	---	AT3G20450	-1.38	-0.47	-3.48	0.010964	0.040711	-3.31
260436_at	unknown protein	---	AT1G68140	1.41	0.50	3.48	0.01097	0.040726	-3.31
254790_at	probable photosystem I chain XI pr	PSAL	AT4G12800	-1.28	-0.36	-3.48	0.010977	0.040744	-3.31
260262_at	hypothetical protein similar to hypo	---	AT1G68470	-2.01	-1.01	-3.48	0.010979	0.040744	-3.31
266864_at	putative hydroxymethylglutaryl-CoA	---	AT2G26800	-1.43	-0.52	-3.48	0.010989	0.040765	-3.31
263909_at	hypothetical protein predicted by ge	DML1	AT2G36490	-1.59	-0.67	-3.48	0.010989	0.040765	-3.31
249279_at	SCARECROW gene regulator-like p	---	AT5G41920	-1.26	-0.33	-3.48	0.010992	0.04077	-3.31
246352_at	hypothetical protein	---	---	-1.25	-0.32	-3.48	0.010994	0.04077	-3.31
252298_at	putative protein various predicted p	---	AT3G49060	-1.28	-0.36	-3.48	0.010999	0.040781	-3.31
249580_at	putative protein predicted proteins,	---	AT5G37740	1.42	0.51	3.48	0.011001	0.040781	-3.31
260902_at	hypothetical protein similar to carbo	---	AT1G21440	-1.64	-0.71	-3.47	0.011017	0.040836	-3.31
255275_at	putative protein various ubiquitin-lik	---	AT4G05310	-1.23	-0.30	-3.47	0.01103	0.040876	-3.31
253577_at	putative protein	---	AT4G31080	1.60	0.67	3.47	0.011035	0.040888	-3.31
248694_at	unknown protein	---	AT5G48340	1.30	0.37	3.47	0.01104	0.040901	-3.32
247516_at	germin-like protein - like germin-like	---	AT5G61750	-1.34	-0.43	-3.47	0.011044	0.040901	-3.32
252766_at	putative protein hypothetical protein	---	---	-1.45	-0.54	-3.47	0.011044	0.040901	-3.32
258127_at	unknown protein	---	AT3G24570	-1.28	-0.36	-3.47	0.011049	0.040915	-3.32
247533_at	putative protein kinase-like transme	---	AT5G61570	-1.95	-0.97	-3.47	0.011053	0.040916	-3.32
259029_at	putative oxysterol-binding protein si	ORP3B	AT3G09300	-1.39	-0.47	-3.47	0.011053	0.040916	-3.32
265495_at	unknown protein predicted by gens	---	AT2G15695	1.33	0.41	3.47	0.01106	0.040935	-3.32
267192_at	hypothetical protein predicted by gr	---	AT2G30890	-1.41	-0.50	-3.47	0.011082	0.041002	-3.32

246793_at	putative protein seven transmembr	---	AT5G27210	1.30	0.38	3.47	0.011084	0.041002	-3.32
249416_at	putative protein DNA DAMAGE-RE	---	AT5G39570	-1.33	-0.41	-3.47	0.011095	0.041038	-3.32
261925_at	oligopeptide transporter, putative s	---	AT1G22540	-1.32	-0.40	-3.47	0.01112	0.041115	-3.32
254865_s_a	putative protein, fragment other pre	---	AT4G05350	-1.28	-0.35	-3.47	0.011124	0.041125	-3.32
248397_at	unknown protein	---	AT5G52140	-1.32	-0.40	-3.47	0.011133	0.041144	-3.32
265252_at	putative Na+/H+ antiporter	SOS1	AT2G01980	1.26	0.34	3.47	0.011133	0.041144	-3.32
245980_at	putative protein ;supported by full-l	---	AT5G13140	-6.55	-2.71	-3.47	0.011136	0.041148	-3.32
264899_at	unknown protein similar to ripening	---	AT1G23130	1.75	0.81	3.47	0.01114	0.041158	-3.32
258698_at	unknown protein	---	AT3G09690	-1.36	-0.45	-3.47	0.011143	0.041161	-3.33
248976_at	putative protein strong similarity to u	ARAD2	AT5G44930	1.41	0.50	3.47	0.011149	0.041172	-3.33
244931_at	hypothetical protein	---	---	1.50	0.58	3.47	0.01115	0.041172	-3.33
260094_at	GDP-4-keto-6-deoxy-D-mannose-3	GER1	AT1G73250	-1.42	-0.51	-3.46	0.011156	0.041189	-3.33
264228_at	hypothetical protein contains similar	GCS1	AT1G67490	1.32	0.40	3.46	0.011164	0.041211	-3.33
247080_at	20S proteasome subunit PAD2 (gb|	PAD2	AT5G66140	1.34	0.42	3.46	0.011166	0.041214	-3.33
247790_at	putative PRL1 associated protein ;s	---	AT5G58720	-1.35	-0.44	-3.46	0.011171	0.041225	-3.33
262464_at	hypothetical protein contains similar	---	AT1G50280	1.65	0.72	3.46	0.01118	0.041252	-3.33
255062_at	hypothetical protein	---	AT4G08940	1.39	0.48	3.46	0.011189	0.041273	-3.33
257721_at	hypothetical protein predicted by ge	---	AT3G18310	1.25	0.33	3.46	0.011189	0.041273	-3.33
252173_at	scarecrow-like 7 (SCL7)	---	AT3G50650	-1.67	-0.74	-3.46	0.011197	0.041295	-3.33
257178_at	hemolysin-like protein similar to her	---	AT3G13070	1.35	0.44	3.46	0.011222	0.041378	-3.33
266458_at	unknown protein ;supported by full-	---	AT2G47710	1.27	0.35	3.46	0.011224	0.041379	-3.33
258837_at	putative 60S ribosomal protein L13A	---	AT3G07110	1.94	0.95	3.46	0.011228	0.041388	-3.33
262120_at	glutathione-S-transferase, putative	ATGSTF4	AT1G02950	-1.45	-0.54	-3.46	0.011238	0.041416	-3.33
256412_at	hypothetical protein predicted by ge	ELO1	AT3G11220	1.35	0.43	3.46	0.011239	0.041416	-3.33
254996_at	putative protein kinase CLV1 recep	---	AT4G10390	-1.43	-0.51	-3.46	0.01125	0.041443	-3.34
252665_at	valine-rich protein	---	AT3G44140	-1.36	-0.45	-3.46	0.011251	0.041443	-3.34
261060_at	unknown protein	---	AT1G17340	-1.40	-0.48	-3.46	0.011252	0.041443	-3.34
258018_at	hypothetical protein predicted by ge	---	AT3G19500	-1.46	-0.54	-3.46	0.011265	0.041484	-3.34
254577_at	putative protein ;supported by full-l	---	AT4G19450	1.51	0.59	3.46	0.011278	0.041526	-3.34
265122_at	similar to flavin-binding monooxyge	FMO GS-O	AT1G62540	-1.24	-0.31	-3.46	0.011291	0.041565	-3.34
254821_at	hypothetical protein	---	AT4G12540	-1.28	-0.36	-3.46	0.011292	0.041565	-3.34
245879_at	putative subunit of TOC complex c	ATTOC64-V	AT5G09420	2.07	1.05	3.46	0.011296	0.041572	-3.34
257226_at	hypothetical protein predicted by ge	---	AT3G27880	2.08	1.05	3.45	0.011298	0.041573	-3.34
252920_at	putative endo-1,4-beta-glucanase e	AtGH9B17	AT4G39000	-1.36	-0.45	-3.45	0.011311	0.041615	-3.34
253337_at	putative protein acetylpolyamine ar	hda14	AT4G33470	-1.60	-0.68	-3.45	0.011322	0.041646	-3.34

264973_at	nitrate transporter, putative nitrate t	---	AT1G27040	-1.40	-0.49	-3.45	0.011325	0.041652	-3.34
262602_at	unknown protein ESTs gb|AA6508	---	AT1G15270	1.26	0.34	3.45	0.011331	0.041668	-3.34
253916_at	hypothetical protein ;supported by f	---	AT4G27240	-1.67	-0.74	-3.45	0.011366	0.04179	-3.35
249666_at	phytochrome C (sp|P14714)	PHYC	AT5G35840	1.41	0.50	3.45	0.011373	0.041809	-3.35
261273_at	unknown protein ; supported by cD	---	AT1G26650	-1.53	-0.61	-3.45	0.011381	0.04183	-3.35
249782_at	dipeptidyl peptidase IV-like protein	---	AT5G24260	-1.32	-0.40	-3.45	0.011403	0.041904	-3.35
246568_at	E2F transcription factor -like protei	DEL2	AT5G14960	1.39	0.48	3.45	0.011412	0.041931	-3.35
245396_at	hypothetical protein ;supported by f	---	AT4G14870	-1.60	-0.67	-3.45	0.011422	0.041959	-3.35
248360_at	putative protein contains similarity t	---	AT5G52430	-1.49	-0.57	-3.45	0.011432	0.041985	-3.35
256897_at	hypothetical protein predicted by ge	---	AT3G24640	-1.26	-0.33	-3.44	0.011459	0.042068	-3.35
259524_at	hypothetical protein	---	AT1G12550	-1.49	-0.57	-3.44	0.011467	0.042091	-3.36
253381_at	Tic22 -like protein strong homology	---	AT4G33350	1.36	0.45	3.44	0.011469	0.042091	-3.36
249244_at	FtsH protease, putative similar to F	VAR1	AT5G42270	-1.33	-0.41	-3.44	0.01147	0.042091	-3.36
255596_at	putative DNA-binding protein simila	WRKY47	AT4G01720	1.93	0.95	3.44	0.011493	0.042168	-3.36
264825_at	hypothetical protein predicted by ge	---	AT1G03720	-1.35	-0.43	-3.44	0.011495	0.042168	-3.36
258241_at	hypothetical protein similar to hypot	LBD25	AT3G27650	-1.46	-0.54	-3.44	0.011504	0.042195	-3.36
255120_x_a	putative transposon protein similar t	---	---	-1.27	-0.34	-3.44	0.01152	0.042238	-3.36
247511_at	Fdr1 Cen - like protein Fdr1, Oryza	---	AT5G62040	-1.37	-0.45	-3.44	0.011522	0.042238	-3.36
261682_at	unknown protein similar to hypothet	---	AT1G47330	1.38	0.47	3.44	0.011523	0.042238	-3.36
253413_at	putative protein Fe(II) transport pro	ZIP9	AT4G33020	1.22	0.29	3.44	0.011525	0.042238	-3.36
267462_at	Expressed protein ; supported by fu	---	AT2G33735	1.65	0.73	3.44	0.011531	0.042252	-3.36
249393_at	disease resistance - like protein res	AtRLP54	AT5G40170	1.74	0.80	3.44	0.011543	0.04229	-3.36
255966_at	14-3-3 protein GF14epsilon (grf10)	GRF10	AT1G22300	1.38	0.47	3.44	0.011556	0.042323	-3.36
245687_at	unknown protein	DEG15	AT1G28320	1.24	0.31	3.44	0.011557	0.042323	-3.36
258086_at	dihydrolipoamide S-acetyltransferas	LTA2	AT3G25860	-1.39	-0.47	-3.44	0.011558	0.042323	-3.36
265387_at	unknown protein ;supported by full-	---	AT2G20670	-2.10	-1.07	-3.44	0.01156	0.042324	-3.36
260119_at	AIG1-like protein similar to AIG1 pr	---	AT1G33930	-1.30	-0.38	-3.44	0.011562	0.042325	-3.36
245466_at	glucosyltransferase like protein	---	AT4G16600	-1.40	-0.48	-3.44	0.011566	0.042332	-3.36
249546_at	putative protein chromosome segre	PMI15	AT5G38150	1.57	0.65	3.44	0.011569	0.042338	-3.37
252292_at	RNA polymerase III subunit - like p	---	AT3G49000	1.35	0.43	3.44	0.011583	0.042381	-3.37
254832_at	pEARLI 1-like protein Arabidopsis t	---	AT4G12490	5.15	2.36	3.44	0.011585	0.042383	-3.37
264332_at	hypothetical protein predicted by ge	---	AT1G61920	-1.29	-0.37	-3.44	0.011589	0.042389	-3.37
252090_at	5B protein like protein cysteine-rich	---	AT3G52130	-1.29	-0.36	-3.44	0.011591	0.042389	-3.37
246727_at	major latex protein homolog - like r	---	AT5G28010	1.44	0.53	3.43	0.011602	0.04242	-3.37
261195_at	transport inhibitor response 1 (TIR1	AFB3	AT1G12820	1.33	0.41	3.43	0.011603	0.04242	-3.37

266207_at	unknown protein ; supported by cD	---	AT2G27680	-1.43	-0.52	-3.43	0.011606	0.042426	-3.37
248907_at	putative protein contains similarity t	---	AT5G46340	1.30	0.38	3.43	0.011612	0.04244	-3.37
248548_at	transmembrane transport protein-lik	---	AT5G50300	-1.27	-0.34	-3.43	0.011618	0.042456	-3.37
247580_at	putative protein traube, Mus muscu	---	AT5G61330	1.37	0.46	3.43	0.011634	0.042505	-3.37
252368_at	cytochrome P450-like protein cytoc	CYP94B3	AT3G48520	-1.94	-0.96	-3.43	0.011635	0.042505	-3.37
265888_at	putative cleavage and polyadenylat	RIE1	AT2G01735	-1.26	-0.34	-3.43	0.011641	0.042518	-3.37
250237_at	putative protein strong similarity to u	---	AT5G13560	-1.40	-0.49	-3.43	0.011644	0.042518	-3.37
249324_at	cytochrome c1 precursor ; supporte	---	AT5G40810	1.26	0.33	3.43	0.011654	0.042545	-3.37
258911_at	unknown protein similar to hypothe	---	AT3G06470	-1.35	-0.43	-3.43	0.011657	0.042545	-3.37
267070_at	putative DnaJ protein	---	AT2G41000	1.53	0.61	3.43	0.011657	0.042545	-3.37
254933_at	putative RNA-directed RNA polyme	RDR2	AT4G11130	1.39	0.47	3.43	0.011669	0.04258	-3.37
252151_at	putative protein several hypothetica	---	AT3G51320	1.26	0.33	3.43	0.011675	0.042594	-3.37
263541_at	unknown protein ; supported by ful	---	AT2G24860	-1.30	-0.38	-3.43	0.011691	0.042643	-3.38
245332_at	isomerase like protein ;supported b	---	AT4G15940	1.23	0.30	3.43	0.011692	0.042643	-3.38
252472_at	GTP-binding protein Rab11 ; suppo	ATRABA2C	AT3G46830	-1.36	-0.45	-3.43	0.011696	0.042647	-3.38
265960_at	putative histone H2B ;supported by	---	AT2G37470	1.68	0.75	3.43	0.011698	0.042647	-3.38
266131_at	putative SCARECROW gene regula	---	AT2G45160	-1.52	-0.60	-3.43	0.011698	0.042647	-3.38
250674_at	laccase-like protein laccase - Popu	LAC13	AT5G07130	-1.56	-0.64	-3.43	0.011703	0.042655	-3.38
247450_at	ripening-related protein - like ripeni	---	AT5G62350	-1.42	-0.50	-3.43	0.01171	0.042668	-3.38
264644_at	hypothetical protein Similar to Caen	CAX11	AT1G08960	-1.37	-0.46	-3.43	0.011714	0.042677	-3.38
266922_s_a	SKP1/ASK1-related, putative simila	ASK20	AT2G45950	1.61	0.69	3.43	0.01172	0.042693	-3.38
244949_at	hypothetical protein	---	---	1.34	0.42	3.43	0.011742	0.042752	-3.38
251205_at	glutathione peroxidase -like protein	ATGPX5	AT3G63080	-1.41	-0.50	-3.43	0.011742	0.042752	-3.38
245719_at	DNA gyrase subunit B - like protein	---	AT5G04110	1.30	0.37	3.43	0.011743	0.042752	-3.38
260328_at	putative aspartate aminotransferase	---	AT1G80360	1.25	0.32	3.43	0.011744	0.042752	-3.38
246310_at	sulfate transporter ATST1 ; suppor	SULTR3;1	AT3G51895	1.59	0.67	3.43	0.01175	0.042765	-3.38
266501_at	unknown protein ; supported by cD	HEN2	AT2G06990	1.36	0.45	3.43	0.011754	0.042775	-3.38
262988_at	putative alanine aminotrans	GGT1	AT1G23310	1.27	0.35	3.42	0.01176	0.042788	-3.38
263075_at	hypothetical protein predicted by ge	---	AT2G17570	1.50	0.58	3.42	0.011769	0.042816	-3.38
263765_at	putative phosphatidylinositol phosph	SFH3	AT2G21540	-2.42	-1.28	-3.42	0.011772	0.042821	-3.38
256165_at	hypothetical protein predicted by ge	---	AT1G48780	-1.43	-0.51	-3.42	0.011778	0.042834	-3.38
249885_at	putative protein strong similarity to u	F8H	AT5G22940	-1.67	-0.74	-3.42	0.011789	0.042869	-3.39
248165_at	putative protein similar to unknown	PHOS32	AT5G54430	1.39	0.47	3.42	0.011794	0.04288	-3.39
267465_at	subtilisin-like serine protease conta	SLP3	AT2G19170	-1.95	-0.96	-3.42	0.011802	0.042902	-3.39
258070_at	hypothetical protein predicted by ge	---	AT3G26040	-1.41	-0.50	-3.42	0.011806	0.04291	-3.39

251010_at	putative protein ;supported by full-l	---	AT5G02550	-1.42	-0.51	-3.42	0.011811	0.042919	-3.39
261116_at	sec14 cytosolic factor, putative sim	---	AT1G75370	-1.31	-0.39	-3.42	0.011829	0.042965	-3.39
264571_at	hypothetical protein predicted by ge	---	AT1G05330	-1.25	-0.32	-3.42	0.011829	0.042965	-3.39
255171_at	hypothetical protein	---	AT4G07990	1.28	0.36	3.42	0.011833	0.042971	-3.39
262124_at	nucleoporin 98-like protein similar to	---	AT1G59660	1.26	0.33	3.42	0.011837	0.042975	-3.39
263414_at	putative bZIP transcription factor co	---	AT2G21230	1.30	0.38	3.42	0.011851	0.043012	-3.39
246754_at	transcription factor - like protein Ha	NF-YC8	AT5G27910	-1.44	-0.52	-3.42	0.011851	0.043012	-3.39
266394_at	Ras-related GTP-binding protein (A	ARA4	AT2G43130	-1.32	-0.40	-3.42	0.011866	0.043058	-3.39
252375_at	rac GTP binding protein Arac8 ; su	ROP10	AT3G48040	-1.34	-0.43	-3.42	0.011868	0.043058	-3.39
246653_at	putative protein clathrin assembly p	---	AT5G35200	1.32	0.40	3.42	0.01189	0.043131	-3.39
247835_at	putative protein similar to unknown	---	AT5G57910	1.72	0.79	3.42	0.011908	0.043191	-3.40
248507_at	putative protein strong similarity to u	---	AT5G50420	-1.40	-0.48	-3.41	0.011924	0.043239	-3.40
247540_at	ethylene responsive element bindir	---	AT5G61590	-1.88	-0.91	-3.41	0.011927	0.043244	-3.40
261550_at	hypothetical protein	---	AT1G63450	-1.29	-0.37	-3.41	0.011942	0.043285	-3.40
261592_at	double-stranded RNA-specific edita	---	AT1G01760	-1.34	-0.43	-3.41	0.011948	0.043302	-3.40
246572_at	putative protein predicted proteins,	---	AT5G15010	1.33	0.41	3.41	0.011955	0.043318	-3.40
248590_at	receptor protein kinase	---	AT5G49660	1.30	0.37	3.41	0.011957	0.043318	-3.40
265394_at	predicted protein ; supported by cD	---	AT2G20725	1.38	0.46	3.41	0.011964	0.043339	-3.40
266802_at	Expressed protein ; supported by cl	---	AT2G22900	-3.26	-1.70	-3.41	0.011967	0.043341	-3.40
251983_at	putative protein MtN21, Medicago t	---	AT3G53210	1.50	0.58	3.41	0.011976	0.043367	-3.40
265073_at	unknown protein similar to putative	---	AT1G55480	-1.68	-0.75	-3.41	0.011997	0.043417	-3.40
264357_at	hypothetical protein Similar to hypot	ATRRP4	AT1G03360	2.16	1.11	3.41	0.012002	0.043428	-3.40
253579_at	SERINE CARBOXYPEPTIDASE II	BRS1	AT4G30610	-1.94	-0.95	-3.41	0.012005	0.043432	-3.40
255449_at	hypothetical protein	---	AT4G02820	1.45	0.53	3.41	0.012014	0.043458	-3.41
252036_at	putative protein ;supported by full-l	---	AT3G52070	1.42	0.51	3.41	0.012021	0.043476	-3.41
257928_at	hypothetical protein contains Pfam	PPR40	AT3G16890	1.24	0.31	3.41	0.012024	0.043478	-3.41
266869_at	putative glucosyltransferase	---	AT2G44660	-1.53	-0.62	-3.41	0.012027	0.043484	-3.41
246206_at	Expressed protein ; supported by cl	ATPBRP	---	-1.28	-0.36	-3.41	0.012034	0.043502	-3.41
263034_at	pollen allergen-like protein similar to	MLP423	AT1G24020	-1.73	-0.79	-3.41	0.012039	0.043511	-3.41
252874_at	putative protein ipiB1 gene, Phytop	---	---	-1.31	-0.39	-3.41	0.01204	0.043511	-3.41
258560_at	hypothetical protein predicted by ge	---	AT3G06020	-1.47	-0.55	-3.41	0.012043	0.043513	-3.41
260076_at	putative calmodulin similar to calmo	---	AT1G73630	1.54	0.63	3.41	0.01205	0.043532	-3.41
249506_at	putative protein	---	AT5G38880	-1.36	-0.44	-3.41	0.012055	0.043543	-3.41
256922_at	hypothetical protein contains simila	---	AT3G19010	1.88	0.91	3.41	0.012061	0.043552	-3.41
247524_at	thioredoxin-like 3 ; supported by cD	ACHT5	AT5G61440	-2.33	-1.22	-3.41	0.012061	0.043552	-3.41

262343_at	phosphoprotein phosphatase 1 ider	TOPP3	AT1G64040	1.28	0.36	3.40	0.012082	0.04362	-3.41
266968_at	putative protein kinase contains a p	---	AT2G39360	-1.91	-0.93	-3.40	0.012087	0.043628	-3.41
AFFX-r2-At-	/FEATURE=cds-2 /GENE= /LABEL	---	---	-1.32	-0.40	-3.40	0.012088	0.043628	-3.41
261203_at	Expressed protein ; supported by fu	---	AT1G12845	-2.88	-1.53	-3.40	0.012094	0.043643	-3.41
251644_at	putative protein putative DNA bindi	---	AT3G57540	1.70	0.76	3.40	0.012101	0.04366	-3.41
250250_at	putative protein similar to unknown	---	AT5G13610	1.34	0.42	3.40	0.01211	0.04368	-3.41
246042_at	cinnamyl-alcohol dehydrogenase -	---	AT5G19440	1.85	0.89	3.40	0.012114	0.043688	-3.41
260137_at	unknown protein ;supported by full-	---	AT1G66330	1.50	0.58	3.40	0.012119	0.043691	-3.41
266942_at	putative ATP binding protein	TXND9	AT2G18990	-1.55	-0.64	-3.40	0.01214	0.043758	-3.42
253754_at	glycine-rich protein like glycine-rich	---	AT4G29020	-2.64	-1.40	-3.40	0.01215	0.043788	-3.42
246957_at	unknown protein	---	AT5G24670	1.24	0.31	3.40	0.012152	0.04379	-3.42
245268_at	hypothetical protein ; supported by	PRMT7	AT4G16570	-1.63	-0.71	-3.40	0.012157	0.043798	-3.42
258164_at	surfeit 1 identical to GB:AAF19609	SURF1	AT3G17910	1.53	0.62	3.40	0.012158	0.043798	-3.42
251816_at	Expressed protein ; supported by cl	TON1B	AT3G55005	-1.45	-0.54	-3.40	0.012162	0.043805	-3.42
246921_at	putative protein small unique nucle	---	AT5G25080	1.51	0.59	3.40	0.01217	0.043824	-3.42
253218_at	subtilisin-like serine protease simila	SLP2	AT4G34980	1.71	0.78	3.40	0.012172	0.043824	-3.42
266012_s_a	predicted protein	---	AT2G07699	1.71	0.78	3.40	0.012174	0.043824	-3.42
256652_at	hypothetical protein similar to putati	LPAT5	AT3G18850	-1.96	-0.97	-3.40	0.012175	0.043824	-3.42
253729_at	beta-1,3-glucanase-like protein bet	---	AT4G29360	-1.39	-0.47	-3.40	0.012181	0.043837	-3.42
251751_at	putative protein predicted proteins,	---	AT3G55720	-1.24	-0.31	-3.40	0.012182	0.043837	-3.42
266895_at	hypothetical protein predicted by ge	---	AT2G26040	-1.29	-0.36	-3.40	0.012185	0.04384	-3.42
246583_at	protein kinase -like protein Ste-20 r	---	AT5G14720	-1.39	-0.48	-3.40	0.012193	0.043861	-3.42
253669_at	dihydropteroate synthase-like prote	---	AT4G30000	1.29	0.36	3.40	0.012196	0.043864	-3.42
263691_at	60s ribosomal protein L34 identical	---	AT1G26880	1.68	0.74	3.40	0.012198	0.043866	-3.42
249822_at	unknown protein	---	AT5G23710	1.33	0.41	3.40	0.012203	0.043873	-3.42
256089_at	hypothetical protein predicted by ge	---	AT1G20830	1.51	0.60	3.40	0.012204	0.043873	-3.42
247741_at	putative protein predicted proteins,	GIL1	AT5G58960	1.68	0.75	3.40	0.012217	0.043904	-3.42
258549_at	putative arginine methyltransferase	PRMT4B	AT3G06930	1.25	0.32	3.40	0.012219	0.043904	-3.42
265689_at	hypothetical protein predicted by ge	---	AT2G24310	-1.25	-0.32	-3.40	0.012221	0.043904	-3.42
253206_at	putative squalene synthase non-co	SQS1	AT4G34640	-1.34	-0.42	-3.40	0.012222	0.043904	-3.42
253757_at	rac GTP binding protein Arac7 ; su	ROP9	AT4G28950	-1.36	-0.45	-3.40	0.012223	0.043904	-3.42
249531_at	putative protein predicted protein, A	AtGDU7	AT5G38770	-1.29	-0.37	-3.40	0.012224	0.043904	-3.42
255925_at	unknown protein ;supported by full-	---	AT1G22200	1.41	0.50	3.39	0.012234	0.043934	-3.42
245665_at	unknown protein ;supported by full-	---	AT1G28250	-1.34	-0.42	-3.39	0.012246	0.043968	-3.43
248465_at	putative protein contains similarity t	---	AT5G51200	1.48	0.57	3.39	0.012248	0.04397	-3.43

256818_at	hypothetical protein contains Pfam	---	AT3G21420	-1.27	-0.35	-3.39	0.012261	0.044007	-3.43
251717_at	putative protein EXOENZYMES RE	LAF3	AT3G55850	1.43	0.51	3.39	0.012268	0.044022	-3.43
251163_at	putative protein S1R protein - Hom	---	AT3G63310	1.24	0.31	3.39	0.012269	0.044022	-3.43
261869_at	hypothetical protein predicted by ge	---	AT1G11470	-1.35	-0.43	-3.39	0.012272	0.044028	-3.43
255082_at	putative protein polyphosphoinositi	---	AT4G09160	-1.29	-0.37	-3.39	0.012275	0.044032	-3.43
247094_at	GDP-D-mannose 4,6-dehydratase;	GMD1	AT5G66280	-1.32	-0.40	-3.39	0.012282	0.044049	-3.43
246644_at	putative protein	---	AT5G35100	-1.49	-0.57	-3.39	0.012291	0.044068	-3.43
249344_at	prohibitin (gb AAC49691.1) ;suppor	ATPHB3	AT5G40770	1.61	0.69	3.39	0.012291	0.044068	-3.43
264882_at	NAM protein, putative similar to NA	anac025	AT1G61110	-1.28	-0.35	-3.39	0.012309	0.044117	-3.43
266401_s_a	putative cell division control protein	CDKB1;2	AT2G38620	-1.25	-0.32	-3.39	0.012311	0.044117	-3.43
249145_at	enoyl CoA hydratase-like protein	ATDCI1	AT5G43280	-1.27	-0.34	-3.39	0.012312	0.044117	-3.43
262489_at	unknown protein EST gb|T21171 co	---	AT1G21830	-1.45	-0.54	-3.39	0.012313	0.044117	-3.43
251711_at	phosphatidylinositol-4-phosphate 5	PIP5K4	AT3G56960	1.41	0.50	3.39	0.012315	0.04412	-3.43
250122_at	unknown protein	---	AT5G16520	1.33	0.41	3.39	0.01232	0.04413	-3.43
265090_at	putative protein phosphatase 2A H	---	AT1G03960	1.31	0.39	3.39	0.012333	0.044168	-3.43
264368_at	hypothetical protein Contains simila	---	AT1G03280	1.29	0.37	3.39	0.012347	0.044211	-3.43
246611_at	putative protein predicted proteins,	MBD02	AT5G35330	1.61	0.69	3.39	0.012354	0.044232	-3.44
265006_at	unknown protein similar to small zin	TIM13	AT1G61570	1.86	0.90	3.39	0.012357	0.044235	-3.44
264038_at	putative ubiquinone biosynthesis pr	---	AT2G03690	-1.42	-0.51	-3.39	0.01236	0.044238	-3.44
261667_at	unknown protein similar to putative	---	AT1G18460	-1.47	-0.55	-3.39	0.012364	0.044246	-3.44
245036_at	putative SF16 protein {Helianthus a	Iqd4	AT2G26410	-1.26	-0.34	-3.39	0.012395	0.044347	-3.44
263808_at	hypothetical protein predicted by ge	---	AT2G04340	1.30	0.38	3.38	0.012398	0.044347	-3.44
250761_at	ankyrin-like protein	---	AT5G06050	-1.34	-0.42	-3.38	0.012401	0.044347	-3.44
260325_at	putative monodehydroascorbate red	MDAR6	AT1G63940	1.44	0.53	3.38	0.012402	0.044347	-3.44
251059_at	serine/threonine protein kinase ATP	CIPK15	AT5G01810	-2.10	-1.07	-3.38	0.012406	0.044354	-3.44
248346_at	GTP-binding protein-like ;supported	ATGB1	AT5G52210	-1.59	-0.67	-3.38	0.012418	0.044384	-3.44
260855_at	phosphatidylinositol-4-phosphate 5	---	AT1G21920	1.49	0.57	3.38	0.012418	0.044384	-3.44
265506_at	putative non-LTR retroelement reve	---	---	-1.38	-0.47	-3.38	0.012442	0.044463	-3.44
251756_at	putative protein	---	AT3G55820	-1.27	-0.35	-3.38	0.012453	0.044493	-3.44
266416_at	unknown protein ; supported by ful	---	AT2G38710	1.48	0.56	3.38	0.012455	0.044494	-3.44
260787_at	Ring3-like bromodomain protein sir	GTE4	AT1G06230	1.33	0.41	3.38	0.01246	0.044505	-3.44
246548_at	putative protein ;supported by full-l	---	AT5G14910	-1.33	-0.42	-3.38	0.012464	0.044513	-3.44
253721_at	hypothetical protein ;supported by f	---	AT4G29250	-1.43	-0.51	-3.38	0.01248	0.044555	-3.45
260299_at	putative gibberellin 3 beta-hydroxyla	GA3OX4	AT1G80330	-1.24	-0.31	-3.38	0.01248	0.044555	-3.45
265715_s_a	expressed protein non-consensus G	---	AT1G13860	-1.66	-0.73	-3.38	0.012482	0.044555	-3.45

262631_at	hypothetical protein predicted by ge	---	AT1G06500	1.29	0.37	3.38	0.012487	0.044568	-3.45
266731_at	unknown protein	---	AT2G03260	-1.26	-0.33	-3.38	0.012495	0.044588	-3.45
266636_at	glycine decarboxylase complex H-p	GDCH	AT2G35370	-1.36	-0.44	-3.38	0.012503	0.044611	-3.45
248535_at	putative protein contains similarity t	---	AT5G50120	-1.40	-0.48	-3.38	0.012513	0.044639	-3.45
251042_at	eceriferum3 (CER3)	PRT6	AT5G02310	1.28	0.36	3.38	0.012521	0.044661	-3.45
246961_at	unknown protein	---	AT5G24710	-1.62	-0.70	-3.38	0.012526	0.04467	-3.45
261747_at	unknown protein similar to putative	---	AT1G08350	1.43	0.51	3.38	0.012533	0.044688	-3.45
255412_at	auxin-binding protein 1 precursor ;s	ABP1	AT4G02980	1.31	0.39	3.38	0.012537	0.044697	-3.45
247917_at	putative protein similar to unknown	BIO1	AT5G57590	-1.28	-0.35	-3.38	0.012541	0.044703	-3.45
259117_at	unknown protein similar to hypothe	SNL1	AT3G01320	1.28	0.36	3.38	0.012543	0.044703	-3.45
259234_at	unknown protein similar to unknowr	---	AT3G11620	-1.33	-0.41	-3.38	0.012549	0.04472	-3.45
258888_at	putative pectinesterase similar to pe	---	AT3G05620	-1.59	-0.67	-3.38	0.012561	0.044747	-3.45
247108_at	ReMembR-H2 protein JR700 (gb|A	RMR1	AT5G66160	1.36	0.44	3.38	0.012561	0.044747	-3.45
245968_at	proline-rich protein extensins	---	AT5G19800	-1.39	-0.47	-3.37	0.01257	0.044767	-3.45
261661_at	lysophospholipase homolog, putati	---	AT1G18360	-1.55	-0.63	-3.37	0.012571	0.044767	-3.45
252249_at	putative protein heat shock protein	---	AT3G49770	-1.38	-0.46	-3.37	0.012579	0.044789	-3.45
262793_at	putative cytochrome P450 monoox	CYP71B7	AT1G13110	1.89	0.92	3.37	0.012586	0.044804	-3.46
246462_at	putative protein predicted protein, I	---	AT5G16940	-1.54	-0.63	-3.37	0.012587	0.044804	-3.46
247110_at	putative protein contains similarity t	---	AT5G65830	-1.46	-0.54	-3.37	0.012592	0.044813	-3.46
260141_at	gibberellin regulatory protein, putat	RGL1	AT1G66350	-2.71	-1.44	-3.37	0.012594	0.044813	-3.46
257252_at	glutathione reductase, cytosolic ide	ATGR1	AT3G24170	1.69	0.76	3.37	0.012595	0.044813	-3.46
249732_at	6-phosphogluconolactonase-like pr	---	AT5G24420	-7.75	-2.95	-3.37	0.012602	0.044832	-3.46
247716_at	putative protein predicted protein, A	---	AT5G59350	1.53	0.61	3.37	0.012607	0.044839	-3.46
258438_at	hypothetical protein predicted by ge	---	AT3G17230	-1.24	-0.31	-3.37	0.012608	0.044839	-3.46
250621_at	membrane protein membrane prote	ATRBL3	AT5G07250	-1.33	-0.41	-3.37	0.012613	0.044849	-3.46
256210_at	hypothetical protein	---	AT1G50950	2.19	1.13	3.37	0.012627	0.044884	-3.46
258458_at	Expressed protein ; supported by cl	---	AT3G22435	1.50	0.59	3.37	0.012627	0.044884	-3.46
248171_at	putative protein similar to unknown	ILR3	AT5G54680	1.26	0.33	3.37	0.012634	0.0449	-3.46
245962_at	putative protein predicted proteins -	---	AT5G19700	-1.33	-0.41	-3.37	0.012636	0.044902	-3.46
256061_at	unknown protein ;supported by full-	---	AT1G07040	1.56	0.64	3.37	0.012644	0.044925	-3.46
264537_at	receptor kinase, putative similar to	BRL1	AT1G55610	1.50	0.59	3.37	0.012655	0.044957	-3.46
266100_at	similar to axi 1 protein from Nicotiar	---	AT2G37980	1.59	0.67	3.37	0.012657	0.044957	-3.46
253592_at	hypothetical protein ;supported by f	---	AT4G30840	1.28	0.35	3.37	0.012678	0.045017	-3.46
250256_at	GTP-binding protein typA (tyrosine	---	AT5G13650	1.29	0.37	3.37	0.01268	0.045017	-3.46
253758_at	Expressed protein ;supported by cD	emb2726	AT4G29060	1.38	0.47	3.37	0.01268	0.045017	-3.46

265374_at	hypothetical protein ;supported by f	PSBX	AT2G06520	-1.25	-0.33	-3.37	0.012712	0.045122	-3.47
253009_at	glycine hydroxymethyltransferase l	SHM1	AT4G37930	-1.44	-0.53	-3.37	0.012717	0.045132	-3.47
256440_at	unknown protein ;supported by full-	---	AT3G10970	1.28	0.36	3.37	0.012725	0.045156	-3.47
253456_at	putative protein norbin, Rattus norv	---	AT4G32050	1.41	0.49	3.36	0.012742	0.045207	-3.47
254186_at	putative protein cellulose synthase	CSLG1	AT4G24010	-1.32	-0.40	-3.36	0.012757	0.045247	-3.47
265464_at	putative myosin heavy chain	---	AT2G37080	1.25	0.32	3.36	0.01276	0.045251	-3.47
253219_at	MYB-like protein myb-related prote	AtMYB32	AT4G34990	1.63	0.71	3.36	0.012767	0.045269	-3.47
261605_at	CDPK-related protein kinase, putat	---	AT1G49580	-1.40	-0.48	-3.36	0.012772	0.045278	-3.47
247202_at	GTP-binding protein ; supported by	AtRABA4a	AT5G65270	-1.37	-0.45	-3.36	0.012773	0.045278	-3.47
253010_at	ovule development protein aintegur	ANT	AT4G37750	-1.34	-0.42	-3.36	0.012779	0.045284	-3.47
258771_at	unknown protein ;supported by full-	---	AT3G10770	1.32	0.40	3.36	0.012781	0.045284	-3.47
246451_at	Inositol 1,3,4-Trisphosphate 5/6 kir	---	AT5G16760	1.24	0.31	3.36	0.012781	0.045284	-3.47
247184_at	putative protein contains similarity t	UBP17	AT5G65450	-1.41	-0.49	-3.36	0.012786	0.045294	-3.47
264101_at	unknown protein similar to putative	HAC1	AT1G79000	1.37	0.46	3.36	0.012795	0.045319	-3.47
245043_at	putative cyclin ;supported by full-le	RCY1	AT2G26430	1.57	0.65	3.36	0.012808	0.045356	-3.47
265714_at	putative integral membrane protein	ATUPS4	AT2G03520	-1.25	-0.33	-3.36	0.012809	0.045356	-3.47
265222_at	putative endosomal protein ; suppo	---	AT2G01970	-1.24	-0.32	-3.36	0.012813	0.045358	-3.47
251451_s_a	putative protein several hypothetica	---	AT3G60040 ,	1.32	0.40	3.36	0.012814	0.045358	-3.47
265512_at	hypothetical protein predicted by ge	---	AT2G05590	-1.41	-0.49	-3.36	0.012817	0.04536	-3.47
267510_at	unknown protein ; supported by ful	SAP18	AT2G45640	1.43	0.52	3.36	0.012827	0.045389	-3.48
251758_at	transcription factor L2 ;supported b	---	AT3G55770	1.33	0.42	3.36	0.012836	0.045413	-3.48
248839_at	putative protein contains similarity t	bHLH071	AT5G46690	-2.03	-1.02	-3.36	0.012846	0.045445	-3.48
248187_at	zinc-binding protein-like ; supporte	---	AT5G53940	1.33	0.41	3.36	0.012853	0.045462	-3.48
251749_at	putative protein predicted proteins,	---	AT3G55690	-1.28	-0.35	-3.36	0.012858	0.045472	-3.48
257699_at	phosphoglycerate kinase, putative	PGK1	AT3G12780	-1.39	-0.47	-3.36	0.012867	0.045488	-3.48
245401_at	hypothetical protein ;supported by f	---	AT4G17670	2.03	1.02	3.36	0.012873	0.045504	-3.48
256752_at	hypothetical protein predicted by ge	---	AT3G27180	1.57	0.65	3.36	0.012876	0.045506	-3.48
246050_s_a	protein phosphatase 2A regulatory	---	AT5G28850 ,	1.24	0.31	3.36	0.012881	0.045516	-3.48
254806_at	putative trehalose-6-phosphate pho	---	AT4G12430 ,	1.32	0.40	3.36	0.01289	0.045538	-3.48
253955_at	putative DNA binding protein DNA	ATRBP45C	AT4G27000	1.50	0.59	3.36	0.012891	0.045538	-3.48
262170_at	hypothetical protein predicted by ge	---	AT1G74940	1.51	0.59	3.36	0.012895	0.045538	-3.48
264173_at	unknown protein	---	AT1G02160	1.28	0.36	3.36	0.012895	0.045538	-3.48
261895_at	metal ion transporter identical to GE	NRAMP1	AT1G80830	1.92	0.94	3.36	0.012901	0.045552	-3.48
251880_at	hypothetical protein ;supported by f	---	AT3G54290	1.43	0.52	3.35	0.012909	0.045575	-3.48
247636_at	putative protein predicted proteins f	---	AT5G60570	-1.30	-0.38	-3.35	0.012915	0.045586	-3.48

266585_at	putative retroelement pol polyprotei	---	---	-1.37	-0.46	-3.35	0.012916	0.045586	-3.48
252911_at	cytochrome P450 -like protein cyto	CYP96A12	AT4G39510	-2.08	-1.05	-3.35	0.012927	0.045615	-3.48
254298_at	putative protein ;supported by full-l	PGR5-LIKE	AT4G22890	-1.42	-0.51	-3.35	0.012933	0.045627	-3.48
264999_at	hypothetical protein contains similar	---	AT1G67310	1.28	0.36	3.35	0.012934	0.045627	-3.48
261670_at	hypothetical protein contains similar	TET11	AT1G18520	-1.24	-0.31	-3.35	0.012942	0.045645	-3.48
250653_at	putative protein strong similarity to u	---	AT5G06930	-1.87	-0.90	-3.35	0.012945	0.045645	-3.49
247628_at	putative protein	---	AT5G60400	-1.45	-0.53	-3.35	0.012945	0.045645	-3.49
266224_at	chloroplast membrane protein (ALB	ALB3	AT2G28800	-1.26	-0.33	-3.35	0.012961	0.045693	-3.49
247127_at	putative protein similar to unknown	---	AT5G66100	-1.29	-0.36	-3.35	0.012965	0.0457	-3.49
254042_at	xyloglucan endo-1,4-beta-D-glucan	XTR6	AT4G25810	-3.54	-1.82	-3.35	0.012974	0.045726	-3.49
246552_at	putative protein	---	AT5G15420	-1.71	-0.77	-3.35	0.012985	0.045759	-3.49
265959_at	unknown protein ;supported by full-	---	AT2G37240	-1.78	-0.83	-3.35	0.01299	0.045769	-3.49
266591_at	expressed protein supported by cD	ABIL1	AT2G46225	-1.55	-0.63	-3.35	0.012996	0.045784	-3.49
262148_at	chloroplast-localized small heat sh	---	AT1G52560	1.79	0.84	3.35	0.013009	0.045821	-3.49
249317_at	glutathione transferase	ATGSTT2	AT5G41240	-1.29	-0.37	-3.35	0.013018	0.045847	-3.49
263551_at	hypothetical protein contains NLS (	---	AT2G17110	1.75	0.81	3.35	0.013028	0.045873	-3.49
258189_at	unknown protein ;supported by full-	JAZ3	AT3G17860	-1.68	-0.75	-3.35	0.013032	0.045876	-3.49
258238_at	unknown protein	RST1	AT3G27670	1.25	0.32	3.35	0.013033	0.045876	-3.49
245792_at	pinoresinol-lariciresinol reductase,	PRR1	AT1G32100	-2.30	-1.20	-3.35	0.013044	0.04591	-3.49
260626_at	storage protein, putative similar to	---	AT1G08040	-1.35	-0.44	-3.35	0.013049	0.045921	-3.49
245949_at	elongation factor 1B alpha-subunit	---	AT5G19510	1.42	0.50	3.35	0.013056	0.045938	-3.49
252574_at	receptor like protein kinase recepto	---	AT3G45430	-1.33	-0.42	-3.35	0.013068	0.045972	-3.50
264625_at	hypothetical protein Contains simila	SNF4	AT1G09020	-1.30	-0.38	-3.34	0.013093	0.046052	-3.50
252716_at	putative protein CAF protein, Arabi	DCL3	AT3G43920	1.39	0.47	3.34	0.013119	0.046138	-3.50
263556_at	hypothetical protein predicted by ge	---	AT2G16365	1.36	0.44	3.34	0.013137	0.046193	-3.50
246929_at	putative protein ; supported by full-	---	AT5G25210	-1.43	-0.52	-3.34	0.013153	0.046243	-3.50
263158_at	CCAAT-binding factor B subunit, p	NF-YA5	AT1G54160	-1.30	-0.38	-3.34	0.013161	0.046264	-3.50
251251_at	serine/threonine protein kinase-like	---	AT3G62220	-1.34	-0.42	-3.34	0.013168	0.046281	-3.50
264535_at	Phosphatidylinositol Transfer Prote	---	AT1G55690	-1.99	-1.00	-3.34	0.013174	0.046293	-3.50
263570_at	aldehyde oxidase identical to GP:3	AAO3	AT2G27150	1.58	0.66	3.34	0.013185	0.046325	-3.50
250570_at	putative protein hypothetical bacteri	EMB1873	AT5G08170	1.44	0.52	3.34	0.01319	0.046327	-3.51
257251_at	type 1 membrane protein, putative	PMP	AT3G24160	-1.31	-0.39	-3.34	0.013191	0.046327	-3.51
249208_at	allene oxide synthase (emb CAA73	AOS	AT5G42650	-2.12	-1.08	-3.34	0.013191	0.046327	-3.51
249088_at	selenium-binding protein-like	---	AT5G44230	1.45	0.54	3.34	0.0132	0.04635	-3.51
253095_at	putative protein ribonuclease III, Ba	---	AT4G37510	1.32	0.40	3.34	0.013218	0.046402	-3.51

259577_at	unknown protein	---	AT1G35340	-1.41	-0.50	-3.34	0.013219	0.046402	-3.51
265319_at	auxin-regulated protein (IAA8) ;sup	IAA8	AT2G22670	-1.72	-0.79	-3.34	0.013243	0.046472	-3.51
249911_at	glucosyltransferase-like protein ; su	CSLA02	AT5G22740	-2.67	-1.42	-3.33	0.013255	0.046506	-3.51
267362_at	hypothetical protein predicted by ge	---	AT2G39910	1.25	0.32	3.33	0.013276	0.046575	-3.51
245786_at	G-Box binding protein, putative sim	---	AT1G32150	-1.23	-0.30	-3.33	0.01328	0.04658	-3.51
252352_at	Expressed protein ; supported by fu	---	AT3G48185	1.41	0.50	3.33	0.013287	0.0466	-3.51
247654_at	cytoplasmic ribosomal protein S15a	---	AT5G59850	1.94	0.96	3.33	0.013292	0.046609	-3.51
254628_at	Expressed protein ; supported by fu	---	AT4G18593	1.23	0.30	3.33	0.013302	0.046635	-3.51
261041_at	hypothetical protein contains simila	EER4	AT1G17440	1.31	0.39	3.33	0.013304	0.046635	-3.51
250395_at	putative protein cylicin II, bovine, P	---	AT5G10950	1.51	0.60	3.33	0.013305	0.046635	-3.51
265799_at	unknown protein ; supported by cD	---	AT2G35680	1.34	0.43	3.33	0.013316	0.046663	-3.52
260006_at	phosphatidylinositol synthase (PIS1	ATPIS1	AT1G68000	-1.23	-0.30	-3.33	0.013329	0.046702	-3.52
252575_at	receptor like protein kinase recepto	---	AT3G45440	-1.45	-0.54	-3.33	0.013341	0.046737	-3.52
248807_at	pectin methylesterase-like	---	AT5G47500	-1.22	-0.28	-3.33	0.013356	0.046783	-3.52
250950_at	putative protein	ATEXO70A	AT5G03540	-1.39	-0.47	-3.33	0.013359	0.046786	-3.52
249256_at	putative protein strong similarity to u	---	AT5G41620	1.45	0.54	3.33	0.013362	0.046789	-3.52
260853_at	hypothetical protein predicted by ge	---	AT1G21950	-1.48	-0.57	-3.33	0.013366	0.046796	-3.52
248971_at	putative protein contains similarity t	---	AT5G45000	-1.53	-0.61	-3.33	0.01337	0.046803	-3.52
251445_at	putative protein hypothetical protei	---	AT3G59870	-1.25	-0.32	-3.33	0.013389	0.046857	-3.52
263209_at	Expressed protein ; supported by fu	---	AT1G10522	1.70	0.76	3.33	0.01339	0.046857	-3.52
256881_at	hypothetical protein ; supported by	---	AT3G26410	1.30	0.38	3.33	0.013398	0.046881	-3.52
245650_at	F5A9.20 similar to S-adenosyl-L-me	---	AT1G24735	-1.34	-0.42	-3.33	0.013404	0.046895	-3.52
249254_at	unknown protein	---	AT5G42110	-1.32	-0.40	-3.33	0.013407	0.046896	-3.52
260156_at	NAM-like protein similar to NAM (no	NAM	AT1G52880	1.58	0.66	3.33	0.01342	0.046933	-3.52
253425_at	putative protein myosin heavy chai	---	AT4G32190	-1.57	-0.65	-3.32	0.013429	0.046954	-3.52
261498_at	receptor kinase, putative similar to	HSL1	AT1G28440	-1.42	-0.50	-3.32	0.01343	0.046954	-3.52
261584_at	CAF protein identical to RNA helica	DCL1	AT1G01040	1.47	0.55	3.32	0.013445	0.046999	-3.53
249876_at	putative protein similar to unknown	CaS	AT5G23060	-1.38	-0.47	-3.32	0.013457	0.047037	-3.53
247025_at	zeaxanthin epoxidase precursor ; s	ABA1	AT5G67030	-1.40	-0.48	-3.32	0.013467	0.047062	-3.53
249734_at	putative protein strong similarity to u	IRE1-1	AT5G24360	-1.24	-0.31	-3.32	0.013484	0.047113	-3.53
252241_at	putative protein CND41, chloroplas	---	AT3G50050	-1.31	-0.39	-3.32	0.013485	0.047113	-3.53
258196_at	hypothetical protein predicted by ge	---	AT3G13980	-2.24	-1.16	-3.32	0.013498	0.047145	-3.53
265195_at	hypothetical protein	---	AT2G36730	1.26	0.33	3.32	0.013499	0.047145	-3.53
253542_at	putative protein deubiquitinating en	UBP18	AT4G31670	1.29	0.37	3.32	0.013501	0.047145	-3.53
256155_at	MYB-family transcription factor, pu	MYB83	AT3G08500	-1.26	-0.33	-3.32	0.013508	0.047163	-3.53

263956_at	putative homeodomain transcriptior	BLH1	AT2G35940	1.62	0.69	3.32	0.013513	0.047172	-3.53
257809_at	ribonucleotide reductase small sub	TSO2	AT3G27060	-1.47	-0.56	-3.32	0.013518	0.047184	-3.53
253847_at	putative protein CRP1, Zea mays, /	---	AT4G28010	1.29	0.36	3.32	0.01352	0.047184	-3.53
245627_at	water stress-induced protein, putat	AtGolS2	AT1G56600	1.77	0.83	3.32	0.013525	0.047194	-3.53
258570_at	phosphoenolpyruvate carboxylase	PPCK2	AT3G04530	1.39	0.47	3.32	0.013527	0.047194	-3.53
253098_at	cytochrome P450-like protein	CYP81D3	AT4G37340	-1.35	-0.44	-3.32	0.013532	0.047204	-3.53
255913_at	kinase, putative similar to leaf rust	---	AT1G66980	1.24	0.30	3.32	0.013541	0.047228	-3.53
247014_at	RuvB DNA helicase-like protein ; s	---	AT5G67630	1.72	0.78	3.32	0.013544	0.04723	-3.53
260929_at	transcription factor TFIID, putative	TAF13	AT1G02680	1.48	0.57	3.32	0.013549	0.04724	-3.53
264329_at	unknown protein Contains similarity	---	AT1G04140	1.26	0.34	3.32	0.013555	0.047257	-3.53
248364_at	fibrillarin 3 (AtFib3) similar to fibrilla	---	AT5G52490	-1.26	-0.33	-3.32	0.013568	0.047294	-3.54
259733_at	nucellin-like protein similar to nucell	---	AT1G77480	1.56	0.64	3.32	0.013576	0.047313	-3.54
258683_at	putative protein kinase contains Pfa	ATSIK	AT3G08760	1.61	0.69	3.32	0.01358	0.047319	-3.54
261678_at	disease resistance protein, putative	---	AT1G47370	-1.35	-0.44	-3.32	0.013585	0.04733	-3.54
246763_at	NHE1 Na+/H+ exchanger ; support	NHX1	AT5G27150	1.56	0.64	3.32	0.013588	0.047333	-3.54
247287_at	unknown protein ; supported by cD	---	AT5G64230	1.65	0.72	3.32	0.013595	0.047345	-3.54
265717_at	unknown protein ;supported by full-	---	AT2G03510	1.40	0.49	3.32	0.013596	0.047345	-3.54
260552_at	putative glyoxalase II ; supported b	GLX2-1	AT2G43430	-1.25	-0.32	-3.32	0.013598	0.047345	-3.54
261538_at	unknown protein ; supported by cDI	---	AT1G01830	1.74	0.80	3.32	0.0136	0.047345	-3.54
253792_at	ammonium transporter - like protei	AMT1;4	AT4G28700	-1.38	-0.47	-3.32	0.013603	0.047351	-3.54
251785_at	ABC transporter - like protein breas	ATWBC19	AT3G55130	-1.78	-0.83	-3.32	0.013608	0.047359	-3.54
248534_at	putative protein contains similarity t	---	AT5G50030	-1.28	-0.36	-3.31	0.013615	0.047376	-3.54
246513_at	hypothetical protein	---	AT5G15680	1.26	0.33	3.31	0.013623	0.047396	-3.54
264804_at	putative receptor kinase, CLV1 sim	---	AT1G08590	-1.37	-0.46	-3.31	0.013625	0.047396	-3.54
260684_at	transcription factor, putative similar	NF-YA8	AT1G17590	1.45	0.53	3.31	0.013637	0.047432	-3.54
252707_at	transporter-like protein multidrug-e	ZIFL2	AT3G43790	1.39	0.47	3.31	0.013645	0.047454	-3.54
245989_s_a	polyubiquitin (UBQ4) identical to GI	UBQ4	AT5G20620	1.33	0.41	3.31	0.013662	0.047504	-3.54
263853_at	putative mutT domain protein	---	AT2G04440	-1.47	-0.56	-3.31	0.013686	0.047579	-3.54
264560_at	hypothetical protein predicted by ge	---	AT1G55820	1.26	0.34	3.31	0.013691	0.047586	-3.54
255560_at	hypothetical protein similar to C. el	---	AT4G02030	-1.25	-0.32	-3.31	0.013694	0.047586	-3.55
247904_at	putative protein contains similarity t	AIL5	AT5G57390	-1.41	-0.49	-3.31	0.013696	0.047586	-3.55
264023_at	Expressed protein ; supported by fu	---	AT2G21195	1.58	0.66	3.31	0.013696	0.047586	-3.55
260527_at	unknown protein ;supported by full-	---	AT2G47270	2.58	1.37	3.31	0.013703	0.047605	-3.55
251528_at	putative protein hypothetical protei	---	AT3G58600	-1.37	-0.46	-3.31	0.013715	0.047639	-3.55
250740_at	t-SNARE SED5 (gb|AAC06291.1) ;	SYP31	AT5G05760	-1.25	-0.32	-3.31	0.013723	0.04766	-3.55

247977_at	putative protein similar to unknown	---	AT5G56850	-1.44	-0.53	-3.31	0.013743	0.047705	-3.55
250220_at	receptor protein kinase-like protein	---	AT5G14210	-1.80	-0.85	-3.31	0.013758	0.047744	-3.55
266323_at	putative calcium-dependent protein	CRK3	AT2G46700	-1.32	-0.40	-3.31	0.013758	0.047744	-3.55
261969_at	hypothetical protein contains similar	---	AT1G65950	1.26	0.34	3.31	0.01377	0.047777	-3.55
262459_at	hypothetical protein similar to hypot	---	AT1G50400	1.43	0.51	3.31	0.013775	0.047787	-3.55
260167_at	hypothetical protein predicted by ge	---	AT1G71970	1.36	0.44	3.31	0.013786	0.047818	-3.55
265550_at	hypothetical protein predicted by ge	---	---	-1.30	-0.37	-3.31	0.013789	0.047818	-3.55
260553_at	unknown protein similar to T11A07.	---	AT2G41800	-1.27	-0.35	-3.31	0.01379	0.047818	-3.55
260365_at	hypothetical protein predicted by ge	---	AT1G70630	-1.31	-0.39	-3.30	0.013795	0.047827	-3.55
261169_at	sucrose-phosphate synthase, puta	ATSPS3F	AT1G04920	-1.33	-0.42	-3.30	0.013799	0.047828	-3.55
248771_at	unknown protein	---	AT5G47790	1.37	0.45	3.30	0.013801	0.047828	-3.55
264775_at	putative endo-1,4-beta-D-glucanas	CEL5	AT1G22880	-1.38	-0.46	-3.30	0.01381	0.047851	-3.55
247747_at	putative protein predicted proteins,	---	AT5G59000	-1.43	-0.51	-3.30	0.01382	0.047875	-3.55
245064_at	Expressed protein ; supported by fu	---	AT2G39725	1.58	0.66	3.30	0.013821	0.047875	-3.55
255579_at	putative bHLH DNA-binding protein	---	AT4G01460	-1.75	-0.81	-3.30	0.013852	0.047976	-3.56
264168_at	unknown protein Similar to yeast ge	---	AT1G02080	1.33	0.41	3.30	0.013855	0.047977	-3.56
263756_at	putative ubiquitin fusion-degradatio	---	AT2G21270	1.24	0.32	3.30	0.013877	0.048047	-3.56
254492_at	endomembrane-associated protein	PCAP1	AT4G20260	1.33	0.41	3.30	0.013881	0.048054	-3.56
266450_s_a	hypothetical protein predicted by ge	---	AT2G43190	1.44	0.53	3.30	0.013887	0.048062	-3.56
259649_at	Serine/arginine-rich protein, putativ	TAF7	AT1G55300	-1.29	-0.36	-3.30	0.013888	0.048062	-3.56
251103_at	putative protein protein phosphatas	---	AT5G01700	-1.43	-0.52	-3.30	0.013893	0.04807	-3.56
253905_s_a	putative protein retrotransposon O	---	---	-1.23	-0.30	-3.30	0.013894	0.04807	-3.56
267172_at	unknown protein ;supported by full-	---	AT2G37660	-1.40	-0.49	-3.30	0.013905	0.048101	-3.56
258446_at	hypothetical protein predicted by ge	---	AT3G22430	1.28	0.36	3.30	0.013927	0.048163	-3.56
250042_at	putative protein non-consensus GC	---	AT5G18420	1.30	0.37	3.30	0.013938	0.048191	-3.56
259570_at	hypothetical protein	COR47	AT1G20440	-1.66	-0.73	-3.30	0.01395	0.048228	-3.56
245459_at	hypothetical protein	---	---	-1.49	-0.58	-3.30	0.013956	0.048237	-3.57
254362_at	Expressed protein ; supported by fu	---	AT4G22160	-1.44	-0.53	-3.30	0.013957	0.048237	-3.57
265038_at	putative protein kinase highly simila	---	AT1G03920	-1.31	-0.39	-3.30	0.013965	0.048248	-3.57
263000_at	hypothetical protein similar to hypot	---	AT1G54350	-1.34	-0.42	-3.30	0.013966	0.048248	-3.57
246061_at	RAN GTPase activating protein 2 ;	RANGAP2	AT5G19320	1.22	0.29	3.30	0.013968	0.048248	-3.57
250229_at	putative protein contains similarity t	NACA3	AT5G13850	1.56	0.64	3.30	0.013969	0.048248	-3.57
257954_at	putative UDP-glucose glucosyltrans	---	AT3G21760	1.35	0.43	3.30	0.013976	0.048263	-3.57
260134_at	Myb-related transcription factor, pu	MYB113	AT1G66370	-1.40	-0.49	-3.30	0.013977	0.048263	-3.57
263864_at	hypothetical protein predicted by ge	CPZ	AT2G04530	1.32	0.40	3.29	0.013982	0.048267	-3.57

266053_at	unknown protein	---	AT2G40730	-1.33	-0.41	-3.29	0.013987	0.048273	-3.57
255814_at	unknown protein	---	AT1G19900	-1.27	-0.34	-3.29	0.013994	0.048283	-3.57
261752_at	putative AMP-binding protein conta	---	AT1G76290	-1.31	-0.39	-3.29	0.013994	0.048283	-3.57
256939_at	multicatalytic endopeptidase comp	PBD1	AT3G22630	1.46	0.54	3.29	0.014011	0.048336	-3.57
254616_at	shaggy-like protein kinase etha (EC	BIN2	AT4G18710	-1.28	-0.36	-3.29	0.014034	0.048406	-3.57
249303_at	putative protein strong similarity to u	---	AT5G41460	-1.51	-0.60	-3.29	0.014048	0.048447	-3.57
264883_s_a	secretory carrier membrane protein	---	AT1G11180	1.29	0.37	3.29	0.014076	0.048539	-3.57
261676_at	Expressed protein ; supported by fu	---	AT1G18480	1.39	0.47	3.29	0.014083	0.048556	-3.57
246180_at	putative protein predicted proteins,	---	AT5G20840	-1.28	-0.35	-3.29	0.014094	0.048583	-3.58
261174_at	26S proteasome regulatory subuni	---	AT1G04810	1.37	0.45	3.29	0.014096	0.048583	-3.58
255931_at	hypothetical protein similar to putati	AtPP2-A12	AT1G12710	-1.56	-0.65	-3.29	0.014098	0.048583	-3.58
248268_at	importin beta	---	AT5G53480	-1.37	-0.45	-3.29	0.014105	0.0486	-3.58
249551_at	putative protein predicted proteins,	---	AT5G38220	1.39	0.47	3.29	0.014117	0.048618	-3.58
255739_at	unknown protein similar to hypothet	ANAC008	AT1G25580	1.44	0.53	3.29	0.014118	0.048618	-3.58
251414_at	hypothetical protein	---	AT3G60370	-1.39	-0.48	-3.29	0.014118	0.048618	-3.58
248472_at	cyclin-dependent protein kinase-like	---	AT5G50860	1.33	0.42	3.29	0.014119	0.048618	-3.58
262432_at	unknown protein	---	AT1G47530	1.65	0.72	3.29	0.014121	0.048621	-3.58
257804_at	protein kinase, putative similar to s	---	AT3G18810	-1.26	-0.34	-3.29	0.014124	0.048621	-3.58
255696_at	putative nucleotide sugar epimeras	GAE3	AT4G00110	-1.33	-0.41	-3.29	0.014131	0.048639	-3.58
264735_s_a	hypothetical protein similar to hypot	---	AT1G62000	-1.40	-0.49	-3.29	0.014144	0.048676	-3.58
246730_at	40S ribosomal protein S19 - like 40	---	AT5G28060	1.61	0.69	3.29	0.014151	0.048682	-3.58
258884_at	threonine dehydratase/deaminase	OMR1	AT3G10050	1.64	0.72	3.29	0.014152	0.048682	-3.58
245672_at	polygalacturonase, putative similar	---	AT1G56710	1.42	0.51	3.29	0.014154	0.048682	-3.58
252161_at	proline-rich protein	---	AT3G50580	-1.22	-0.29	-3.29	0.014157	0.048682	-3.58
258916_at	putative ABC transporter ATPase s	ATNAP7	AT3G10670	-1.28	-0.36	-3.29	0.014159	0.048682	-3.58
263223_at	coatomer-like protein, epsilon subu	---	AT1G30630	-1.34	-0.42	-3.29	0.01416	0.048682	-3.58
263804_at	putative protein kinase contains a p	---	AT2G40270	-1.39	-0.48	-3.29	0.014164	0.048685	-3.58
248064_at	unknown protein	---	AT5G55570	-1.93	-0.95	-3.28	0.014204	0.048815	-3.58
250688_at	transcription factor-like protein ;sup	NF-YA10	AT5G06510	1.27	0.34	3.28	0.014213	0.048829	-3.58
246015_at	putative protein predicted proteins,	---	AT5G10700	-1.28	-0.35	-3.28	0.014214	0.048829	-3.58
253457_at	putative protein C56A3.6,Caenorha	---	AT4G32060	-1.35	-0.43	-3.28	0.014222	0.04885	-3.59
255752_at	secretory carrier membrane protein	---	AT1G32050	-1.41	-0.50	-3.28	0.014232	0.048856	-3.59
252242_at	F-box protein family, AtFBL16 cont	VFB2	AT3G50080	1.32	0.40	3.28	0.014233	0.048856	-3.59
259215_at	unknown protein largely predicted b	DEX1	AT3G09090	1.27	0.35	3.28	0.014236	0.048856	-3.59
261699_at	hypothetical protein identical to hyp	---	AT1G32660	-1.33	-0.41	-3.28	0.014236	0.048856	-3.59

255003_at	AIG1-like protein AIG1 - Arabidops	---	AT4G09950	-1.26	-0.33	-3.28	0.014237	0.048856	-3.59
247793_at	putative protein ; supported by cDN	PSY1	AT5G58650	1.48	0.56	3.28	0.014242	0.048861	-3.59
245080_at	putative receptor-like protein kinase	---	AT2G23300	-1.44	-0.53	-3.28	0.014268	0.048941	-3.59
253738_at	photosystem I subunit PSI-E - like	PSAE-1	AT4G28750	-1.22	-0.29	-3.28	0.014274	0.048954	-3.59
256766_at	Expressed protein ; supported by cl	PCC1	AT3G22231	-13.63	-3.77	-3.28	0.014276	0.048954	-3.59
245116_at	unknown protein ; supported by cD	---	AT2G41620	1.72	0.78	3.28	0.014286	0.048978	-3.59
253755_at	26S proteasome subunit 4-like prot	RPT2a	AT4G29040	1.31	0.39	3.28	0.014288	0.048979	-3.59
245320_at	Expressed protein ; supported by fu	---	AT4G16444	1.27	0.35	3.28	0.014294	0.048994	-3.59
247965_at	putative protein similar to unknown	AGP14	AT5G56540	-1.53	-0.61	-3.28	0.014302	0.049013	-3.59
245458_at	kinase like protein	---	AT4G16970	-1.30	-0.38	-3.28	0.014308	0.049024	-3.59
265914_at	En/Spm-like transposon protein rela	---	---	-1.24	-0.31	-3.28	0.01431	0.049024	-3.59
246826_at	UTP-glucose glucosyltransferase -	UGT72E3	AT5G26310	-1.29	-0.37	-3.28	0.014319	0.04905	-3.59
251288_at	exonuclease RRP41 ; supported by	RRP41	AT3G61620	1.44	0.52	3.28	0.014322	0.049053	-3.59
245194_at	F12A21.5 similar to protein phospha	---	AT1G67820	1.61	0.69	3.28	0.014332	0.049077	-3.59
261500_at	hypothetical protein predicted by ge	---	AT1G28400	1.99	0.99	3.28	0.014335	0.049081	-3.59
251964_at	hypothetical protein ; supported by	---	AT3G53370	-1.53	-0.61	-3.28	0.014338	0.049083	-3.59
253482_at	Expressed protein ; supported by cl	---	AT4G31985	1.96	0.97	3.28	0.014342	0.049091	-3.59
245517_at	hypothetical protein	---	AT4G15840	1.22	0.28	3.28	0.014348	0.049102	-3.59
247517_at	100 kDa coactivator - like protein 1	---	AT5G61780	-1.38	-0.46	-3.28	0.014352	0.049111	-3.60
253921_at	glutamine amidotransferase/cyclase	AT-HF	AT4G26900	1.34	0.42	3.28	0.014359	0.049127	-3.60
265905_at	putative transcription elongation fac	---	AT2G25640	-1.24	-0.31	-3.27	0.01438	0.049188	-3.60
262730_at	putative transport protein may be a	ATOCT3	AT1G16390	-1.34	-0.42	-3.27	0.014384	0.049188	-3.60
262760_at	hypothetical protein contains similar	---	AT1G10770	2.38	1.25	3.27	0.014385	0.049188	-3.60
251820_at	putative protein In2-1 protein, Zea	GSTL2	AT3G55040	-1.72	-0.78	-3.27	0.014386	0.049188	-3.60
245930_at	transcriptional co-activator-like prot	---	AT5G09240	-1.53	-0.61	-3.27	0.014389	0.049188	-3.60
251723_at	putative protein speckle-type prote	---	AT3G56230	-1.36	-0.45	-3.27	0.01439	0.049188	-3.60
249958_at	plasma membrane associated prot	---	AT5G18970	-1.44	-0.52	-3.27	0.014398	0.049208	-3.60
258772_at	putative ubiquinol-cytochrome C rec	---	AT3G10860	1.37	0.45	3.27	0.014403	0.04922	-3.60
248423_at	putative protein contains similarity t	---	AT5G51670	-1.51	-0.59	-3.27	0.014409	0.04923	-3.60
261933_at	3-deoxy-D-arabino-heptulosonate 7	---	AT1G22410	1.71	0.78	3.27	0.014419	0.049257	-3.60
253303_at	putative protein ; supported by full-	---	AT4G33780	1.31	0.39	3.27	0.014431	0.049291	-3.60
249094_at	dimethylaniline monooxygenase-like	YUC5	AT5G43890	-1.38	-0.46	-3.27	0.01444	0.049314	-3.60
259653_at	unknown protein	---	AT1G55240	-1.24	-0.31	-3.27	0.014444	0.049322	-3.60
262588_at	unknown protein Similar to gb|AJ00	---	AT1G15130	-1.31	-0.39	-3.27	0.014455	0.049342	-3.60
255775_at	hypothetical protein predicted by ge	RBL12	AT1G18600	-1.28	-0.36	-3.27	0.014462	0.049342	-3.60

267461_at	putative auxin-regulated protein ;su	---	AT2G33830	1.37	0.45	3.27	0.014464	0.049342	-3.60
251562_at	putative protein hypothetical protei	---	AT3G57890	-1.28	-0.35	-3.27	0.014465	0.049342	-3.60
247508_at	auxin response factor - like protein	ARF2	AT5G62000	-1.46	-0.55	-3.27	0.014465	0.049342	-3.60
256060_at	hypothetical protein contains similar	---	AT1G07050	1.51	0.59	3.27	0.014465	0.049342	-3.60
254913_at	putative protein beta-transducin rep	---	AT4G11270	1.28	0.36	3.27	0.014471	0.049353	-3.60
253160_at	putative protein predicted protein, S	---	AT4G35760	1.50	0.59	3.27	0.014478	0.049373	-3.60
250812_at	putative protein similar to unknown	---	AT5G04900	1.27	0.35	3.27	0.014485	0.049386	-3.60
262923_at	Dhp1-like protein similar to Dhp1 pr	XRN3	AT1G75660	1.29	0.37	3.27	0.014488	0.04939	-3.61
251170_at	ATMRK1 ;supported by full-length	ATMRK1	AT3G63260	1.23	0.30	3.27	0.014495	0.049404	-3.61
253240_at	putative ketoacyl-CoA synthase fat	KCS17	AT4G34510	-1.25	-0.32	-3.27	0.014496	0.049404	-3.61
248890_at	disease resistance protein-like	---	AT5G46270	1.28	0.35	3.27	0.014516	0.049464	-3.61
246407_at	disease resistance protein RPS4, p	---	AT1G57670	-1.24	-0.31	-3.27	0.014518	0.049464	-3.61
264441_at	Expressed protein ; supported by fu	---	AT1G27435	1.36	0.44	3.27	0.014522	0.049464	-3.61
254096_at	acid phosphatase - like protein acid	---	AT4G25150	-1.33	-0.41	-3.27	0.014525	0.049464	-3.61
264289_at	hypothetical protein similar to hypot	---	AT1G61890	-1.98	-0.99	-3.27	0.014527	0.049464	-3.61
247107_at	senescence-associated protein alm	STR16	AT5G66040	1.53	0.61	3.27	0.014527	0.049464	-3.61
266604_at	E2, ubiquitin-conjugating enzyme 6	UBC6	AT2G46030	-1.63	-0.71	-3.27	0.014541	0.049504	-3.61
257973_at	unknown protein	---	AT3G20720	1.38	0.46	3.27	0.01455	0.049525	-3.61
257376_at	hypothetical protein predicted by ge	---	AT2G32350	-1.31	-0.39	-3.27	0.014552	0.049525	-3.61
251088_at	putative protein several hypothetica	---	AT5G01480	-1.48	-0.57	-3.27	0.014553	0.049525	-3.61
256876_at	hypothetical protein predicted by ge	---	AT3G26360	1.22	0.28	3.26	0.014557	0.049527	-3.61
248637_at	DNAJ-like protein	---	AT5G49060	1.28	0.35	3.26	0.014559	0.049527	-3.61
260313_at	putative disease resistance protein	---	AT1G63870	-1.39	-0.47	-3.26	0.014568	0.049548	-3.61
257679_at	glycine-rich protein, putative identic	GRP5	AT3G20470	-2.30	-1.20	-3.26	0.014569	0.049548	-3.61
264003_at	Expressed protein ; supported by fu	GEM	AT2G22475	-1.40	-0.49	-3.26	0.014593	0.049623	-3.61
245228_at	phytochelatin synthetase-like protei	COBL2	AT3G29810	-1.85	-0.89	-3.26	0.0146	0.049638	-3.61
250212_at	putative protein contains similarity t	SUVH4	AT5G13960	1.31	0.39	3.26	0.014608	0.049659	-3.61
251139_at	cell division - like protein JM23 pro	---	AT5G01230	1.38	0.46	3.26	0.014613	0.049661	-3.61
254483_at	putative protein CRP1, Zea mays,	---	AT4G20740	1.43	0.52	3.26	0.014613	0.049661	-3.61
257108_x_a	hypothetical protein	---	---	-1.25	-0.32	-3.26	0.014617	0.049667	-3.61
264025_at	AUX1-like amino acid permease ; s	---	AT2G21050	-2.44	-1.29	-3.26	0.014621	0.04967	-3.61
250895_at	RIBOSOMAL PROTEIN S28- like r	---	AT5G03850	1.51	0.60	3.26	0.014622	0.04967	-3.61
249290_at	putative protein strong similarity to u	---	AT5G41060	-1.58	-0.66	-3.26	0.014631	0.049692	-3.62
260334_at	homeotic protein (ATK1) identical to	KNAT2	AT1G70510	-1.26	-0.33	-3.26	0.014639	0.049712	-3.62
260999_at	hypothetical protein similar to putati	---	AT1G26580	1.26	0.33	3.26	0.014672	0.049816	-3.62

247289_at	2-oxoglutarate/malate translocator	DIT2.1	AT5G64290	-1.38	-0.46	-3.26	0.014676	0.049816	-3.62
262736_at	lipase, putative contains Pfam prof	---	AT1G28570	1.28	0.35	3.26	0.014676	0.049816	-3.62
248982_at	chloroplast nucleoid DNA-binding p	---	AT5G45120	-1.32	-0.40	-3.26	0.014682	0.049818	-3.62
264553_s_a	putative cinnamyl alcohol dehydrog	---	AT1G09480	1.36	0.45	3.26	0.014683	0.049818	-3.62
253684_at	nucleotide pyrophosphatase - like p	---	AT4G29690	-1.34	-0.42	-3.26	0.014683	0.049818	-3.62
264277_at	polygalacturonase isoenzyme 1 be	---	AT1G60390	-1.31	-0.39	-3.26	0.01469	0.049834	-3.62
253307_at	putative protein auxin-induced prot	---	AT4G33670	1.34	0.43	3.26	0.014699	0.049855	-3.62
259806_at	mysoin-like protein similar to GI:23	---	AT1G47900	-1.48	-0.56	-3.26	0.01471	0.049886	-3.62
259304_at	putative nucleotide repair protein si	ERCC1	AT3G05210	1.29	0.37	3.26	0.014729	0.049937	-3.62
259287_at	putative rac GTPase activating prot	---	AT3G11490	-1.36	-0.45	-3.26	0.014731	0.049937	-3.62
266171_at	putative CCAAT-binding transcriptic	NF-YB1	AT2G38880	1.39	0.47	3.26	0.014731	0.049937	-3.62
256050_at	leucine zipper protein, putative sim	ATEXO70E	AT1G07000	-1.31	-0.39	-3.26	0.014738	0.049953	-3.62
244921_s_a	hypothetical protein	---	AT2G07674	1.48	0.57	3.26	0.014741	0.049955	-3.62
252420_at	putative protein several hypothetica	---	AT3G47530	1.48	0.57	3.26	0.014744	0.049956	-3.62
249291_at	glutathione transferase AtGST 10 (e	ATGSTT1	AT5G41210	1.33	0.41	3.25	0.014755	0.049987	-3.62
257004_s_a	glycolate oxidase, putative similar t	---	AT3G14130	1.25	0.33	3.25	0.014762	0.050003	-3.62
263841_at	putative xyloglucan endo-transglyco	---	AT2G36870	-6.06	-2.60	-3.25	0.014766	0.050004	-3.63
253458_at	putative protein predicted protein, A	---	AT4G32070	1.53	0.61	3.25	0.014767	0.050004	-3.63
265050_at	jasmonate inducible protein, putativ	---	AT1G52070	-1.26	-0.33	-3.25	0.014796	0.050095	-3.63
251292_at	putative protein hypothetical protei	---	AT3G61920	-1.43	-0.52	-3.25	0.0148	0.050102	-3.63
253633_at	putative protein tetratricopeptide re	---	AT4G30480	1.35	0.44	3.25	0.01481	0.050127	-3.63
260279_at	putative DNA repair protein similar t	---	AT1G80420	-1.29	-0.37	-3.25	0.014813	0.05013	-3.63
255045_at	putative protein hypothetical protei	---	AT4G09690	-1.33	-0.41	-3.25	0.014817	0.050131	-3.63
262431_at	trypsin inhibitor 2, putative similar t	---	AT1G47540	-1.28	-0.35	-3.25	0.014817	0.050131	-3.63
246335_at	lethal leaf-spot 1 homolog Lls1	ACD1	AT3G44880	1.42	0.50	3.25	0.01482	0.050132	-3.63
251013_at	putative protein various predicted p	---	AT5G02540	-4.56	-2.19	-3.25	0.014827	0.050148	-3.63
262767_at	putative oxysterol-binding protein si	ORP1D	AT1G13170	-1.74	-0.80	-3.25	0.014836	0.050171	-3.63
252234_at	putative protein ; supported by full-	ATPSK4	AT3G49780	2.77	1.47	3.25	0.014849	0.050208	-3.63
263733_at	polyprotein, putative similar to poly	---	---	-1.22	-0.29	-3.25	0.014854	0.050215	-3.63
262664_at	hypothetical protein predicted by ge	---	AT1G13970	1.29	0.37	3.25	0.014855	0.050215	-3.63
265945_at	putative cytokinin oxidase ; suppor	CKX2	AT2G19500	-1.28	-0.36	-3.25	0.014877	0.050281	-3.63
252786_at	putative protein putative DNA-helic	CHR38	AT3G42670	1.65	0.72	3.25	0.014891	0.050321	-3.63
256045_at	MAP3K gamma protein kinase, put	MAPKKK1:	AT1G07150	-1.33	-0.42	-3.25	0.014896	0.050325	-3.63
258366_at	DNA-binding protein contains AP2	RAP2.2	AT3G14230	1.33	0.41	3.25	0.014897	0.050325	-3.63
257881_at	putative serine carboxypeptidase II	scpl33	AT3G17180	-1.38	-0.46	-3.25	0.014899	0.050325	-3.63

259578_at	unknown protein	---	AT1G27990	-1.51	-0.59	-3.25	0.014904	0.050335	-3.64
254284_at	putative fizzy-related protein fizzy-r	FZR2	AT4G22910	-1.47	-0.55	-3.25	0.014911	0.050352	-3.64
251610_at	putative protein hypothetical protei	---	AT3G57930	-2.23	-1.16	-3.25	0.014921	0.050376	-3.64
262370_at	hypothetical protein predicted by ge	---	AT1G73090	1.42	0.51	3.25	0.014924	0.050376	-3.64
265863_at	putative membrane protein	VIT1	AT2G01770	-1.39	-0.48	-3.25	0.014925	0.050376	-3.64
265357_at	E2, ubiquitin-conjugating enzyme,	UBC29	AT2G16740	1.44	0.53	3.24	0.014972	0.050526	-3.64
249278_at	putative protein strong similarity to u	---	AT5G41900	-5.67	-2.50	-3.24	0.014988	0.050573	-3.64
265943_at	putative cytidine deaminase ;suppo	CDA1	AT2G19570	-1.73	-0.79	-3.24	0.01499	0.050573	-3.64
261574_at	cytochrome P450, putative similar t	CYP78A8	AT1G01190	-1.71	-0.78	-3.24	0.014996	0.050587	-3.64
258122_at	hypothetical protein contains similar	ATGSL04	AT3G14570	-1.57	-0.65	-3.24	0.015004	0.050599	-3.64
251487_at	cysteine synthase	OASC	AT3G59760	-1.68	-0.75	-3.24	0.015004	0.050599	-3.64
266633_at	putative DnaJ protein	---	AT2G35540	1.34	0.42	3.24	0.015009	0.050606	-3.64
246346_at	hypothetical protein	---	AT3G56810	-1.50	-0.58	-3.24	0.015016	0.050623	-3.64
262254_at	unknown protein similar to GDSL-m	GLIP5	AT1G53920	-1.72	-0.78	-3.24	0.015052	0.050738	-3.65
252025_at	putative protein chromosome asse	---	AT3G52900	-1.32	-0.40	-3.24	0.015071	0.05079	-3.65
259721_at	putative phosphatidylinositol-4-phos	---	AT1G60890	1.46	0.55	3.24	0.015072	0.05079	-3.65
261355_at	putative malate oxidoreductase sim	ATNADP-M	AT1G79750	1.35	0.43	3.24	0.015079	0.050801	-3.65
264581_at	hypothetical protein predicted by ge	---	AT1G05210	-1.75	-0.81	-3.24	0.01508	0.050801	-3.65
256754_at	unknown protein	CHUP1	AT3G25690	-1.33	-0.41	-3.24	0.015089	0.050826	-3.65
265246_at	putative pectinesterase	ATPMEPCI	AT2G43050	-1.36	-0.44	-3.24	0.015093	0.050828	-3.65
256653_at	hypothetical protein predicted by ge	---	AT3G18870	1.30	0.38	3.24	0.015095	0.050828	-3.65
260102_at	unknown protein	---	AT1G35470	1.34	0.43	3.24	0.015098	0.050828	-3.65
255943_at	UDP-glucose glucosyltransferase,	AtUGT85A	AT1G22370	2.23	1.16	3.24	0.015099	0.050828	-3.65
260065_at	putative RING zinc finger protein c	---	AT1G73760	1.33	0.41	3.24	0.015104	0.050836	-3.65
248243_at	unknown protein	CPuORF50	AT5G53588	1.24	0.31	3.24	0.015106	0.050837	-3.65
256438_s_a	ribosomal protein L41 identical to ri	---	AT2G40205	1.76	0.82	3.24	0.015109	0.050837	-3.65
246036_at	alpha-galactosidase - like protein a	AtAGAL2	AT5G08370	-1.73	-0.79	-3.24	0.015111	0.050837	-3.65
265850_at	putative DnaJ protein	---	AT2G35720	1.43	0.51	3.24	0.015113	0.050837	-3.65
245640_at	helix-loop-helix protein homolog, pu	---	AT1G25330	-1.29	-0.36	-3.24	0.01512	0.050849	-3.65
267007_at	60S ribosomal protein L18A ; suppo	---	AT2G34480	1.54	0.62	3.24	0.015121	0.050849	-3.65
246944_at	ubiquinol--cytochrome-c reductase	---	AT5G25450	1.31	0.39	3.24	0.015146	0.050927	-3.65
249039_at	late embryogenesis abundant prote	---	AT5G44310	-1.33	-0.41	-3.24	0.015155	0.050949	-3.65
253312_s_a	putative protein (1,4)-beta-xylan en	---	AT4G33850	-1.28	-0.36	-3.23	0.015159	0.050953	-3.65
254238_at	putative protein probable membran	---	AT4G23540	1.39	0.48	3.23	0.015176	0.051003	-3.65
245946_at	putative protein glyoxal oxidase pre	---	AT5G19580	-1.39	-0.48	-3.23	0.015185	0.051018	-3.66

251884_at	embryonic abundant protein -like e	---	AT3G54150	3.51	1.81	3.23	0.015186	0.051018	-3.66
253200_at	vacuolar H+-transporting ATPase 1	AVA-P1	AT4G34720	-1.37	-0.45	-3.23	0.015187	0.051018	-3.66
254119_at	putative pectate lyase pectate lyas	---	AT4G24780	-2.81	-1.49	-3.23	0.015202	0.05106	-3.66
266761_at	putative alcohol dehydrogenase	---	AT2G47130	1.88	0.91	3.23	0.015205	0.051063	-3.66
252626_at	putative protein predicted protein, A	---	AT3G44940	-1.40	-0.48	-3.23	0.015224	0.051119	-3.66
262155_at	glycosyl transferase, putative simila	---	AT1G52420	1.34	0.42	3.23	0.01523	0.051133	-3.66
256885_at	chaperone-like ATPase contains Pf	---	AT3G15120	1.35	0.43	3.23	0.015238	0.051145	-3.66
252147_at	putative protein several hypothetica	---	AT3G51270	1.48	0.56	3.23	0.015238	0.051145	-3.66
253743_at	putative protein vegetative storage	---	AT4G28940	-1.21	-0.28	-3.23	0.015246	0.051164	-3.66
255553_at	predicted protein of unknown functi	---	AT4G01960	1.44	0.52	3.23	0.015257	0.051193	-3.66
252528_at	E2, ubiquitin-conjugating enzyme 1	UBC13	AT3G46460	-1.26	-0.33	-3.23	0.015261	0.051199	-3.66
252780_at	alcohol dehydrogenase (ATA1) ;su	ATA1	AT3G42960	-1.50	-0.58	-3.23	0.015286	0.051277	-3.66
248916_at	receptor protein kinase-like protein	---	AT5G45840	-1.87	-0.90	-3.23	0.015303	0.051321	-3.66
248421_at	unknown protein ;supported by full-	---	AT5G51510	1.31	0.38	3.23	0.015304	0.051321	-3.66
254074_at	transcriptional activator CBF1 CRT	CBF1	AT4G25490	1.78	0.83	3.23	0.015312	0.05134	-3.66
259738_at	predicted protein	---	AT1G64355	-1.31	-0.39	-3.23	0.015314	0.05134	-3.66
249870_at	unknown protein	TGH	AT5G23080	1.84	0.88	3.23	0.015324	0.051357	-3.66
245605_at	ribonucleoprotein like protein	---	AT4G14300	-1.23	-0.30	-3.23	0.015324	0.051357	-3.66
260832_at	unknown protein ; supported by cDI	GAUT6	AT1G06780	1.34	0.42	3.23	0.015358	0.051463	-3.67
265988_at	hypothetical protein predicted by ge	---	AT2G24255	-1.27	-0.34	-3.22	0.015377	0.051521	-3.67
251714_at	putative protein leucine-rich recept	---	AT3G56370	-1.84	-0.88	-3.22	0.015391	0.051558	-3.67
254014_at	NPR1 like protein regulatory protei	---	AT4G26120	1.88	0.91	3.22	0.015393	0.051558	-3.67
250531_at	GTP-binding protein LepA homolog	---	AT5G08650	-1.48	-0.57	-3.22	0.015399	0.051573	-3.67
255791_at	plastid protein identical to GB:Z860	DAL1	AT2G33430	1.42	0.51	3.22	0.015402	0.051575	-3.67
256063_at	Expressed protein ; supported by fu	STN1	AT1G07130	1.32	0.40	3.22	0.015409	0.051589	-3.67
247896_at	26S proteasome regulatory subuni	---	AT5G57950	1.25	0.32	3.22	0.015417	0.051609	-3.67
253139_at	ankyrin repeat-containing protein 2	AKR2	AT4G35450	-1.25	-0.32	-3.22	0.015426	0.051626	-3.67
249233_at	putative protein contains similarity t	---	AT5G42150	2.05	1.04	3.22	0.015427	0.051626	-3.67
263173_at	hypothetical protein predicted by ge	BRX-LIKE3	AT1G54180	-1.25	-0.32	-3.22	0.01543	0.05163	-3.67
247002_at	putative protein strong similarity to u	HOS15	AT5G67320	1.34	0.42	3.22	0.015433	0.051632	-3.67
262870_at	alcohol dehydrogenase, putative si	---	AT1G64710	2.51	1.33	3.22	0.015445	0.051664	-3.67
263202_at	putative inositol 1,4,5-trisphosphate	5PTASE13	AT1G05630	-1.87	-0.91	-3.22	0.01545	0.05167	-3.67
248088_at	2-oxoglutarate dehydrogenase E2 s	---	AT5G55070	1.44	0.53	3.22	0.015453	0.05167	-3.67
267376_at	putative receptor-like protein kinas	ER	AT2G26330	-2.80	-1.48	-3.22	0.015453	0.05167	-3.67
263913_at	putative receptor-like protein kinase	---	AT2G36570	-1.54	-0.62	-3.22	0.015485	0.051763	-3.68

245356_at	adenosylhomocysteinase ;supporte	MEE58	AT4G13940	1.46	0.55	3.22	0.015486	0.051763	-3.68
247071_at	putative protein similar to unknown	DAR3	AT5G66640	-2.31	-1.21	-3.22	0.015491	0.051774	-3.68
252991_at	protein kinase like protein protein k	---	AT4G38470	-2.32	-1.21	-3.22	0.015498	0.051788	-3.68
257782_at	hypothetical protein predicted by ge	---	AT3G27120	-1.31	-0.39	-3.22	0.015503	0.051797	-3.68
264914_at	clathrin adaptor medium chain prot	HAP13	AT1G60780	-1.31	-0.39	-3.22	0.015511	0.051817	-3.68
246223_at	UDP-glucuronyltransferase-like pro	IRX14	AT4G36890	1.26	0.33	3.22	0.015515	0.051823	-3.68
245127_at	putative Na+/Ca2+ antiporter ; sup	ATMHX	AT2G47600	-1.60	-0.68	-3.22	0.015522	0.051837	-3.68
263129_at	unknown protein ;supported by full-	---	AT1G78620	-1.28	-0.36	-3.22	0.01553	0.051856	-3.68
259218_at	hypothetical protein similar to putat	---	AT3G03580	1.33	0.41	3.22	0.015537	0.051874	-3.68
262944_at	phosphoglycerate kinase, putative	PGK	AT1G79550	1.44	0.53	3.22	0.015549	0.051896	-3.68
259584_at	hypothetical protein predicted by ge	---	AT1G28080	-1.25	-0.32	-3.22	0.015552	0.051896	-3.68
266770_at	putative expansin Experimental evid	ATEXPA15	AT2G03090	-1.52	-0.61	-3.22	0.015554	0.051896	-3.68
263618_at	hypothetical protein predicted by ge	APC2	AT2G04660	1.37	0.46	3.22	0.015556	0.051896	-3.68
252440_at	aquaporin-like protein aquaporin - \	TIP5;1	AT3G47440	-1.26	-0.33	-3.22	0.015557	0.051896	-3.68
263289_at	ubiquitin extension protein (UBQ2)	---	AT2G36170	1.61	0.69	3.22	0.015557	0.051896	-3.68
254816_at	putative adenine phosphoribosyltra	APT4	AT4G12440	-1.22	-0.29	-3.22	0.015562	0.051905	-3.68
259372_at	homeotic protein boi1AP1, putative	AP1	AT1G69120	-1.29	-0.37	-3.22	0.015566	0.051906	-3.68
253498_at	putative protein various predicted p	---	AT4G31890	-1.74	-0.80	-3.22	0.015567	0.051906	-3.68
256629_at	serine/threonine protein phosphata	ATFYPP3	AT3G19980	1.28	0.36	3.22	0.015571	0.051911	-3.68
256976_at	unknown protein similar to putative	---	---	1.46	0.55	3.21	0.015579	0.051931	-3.68
255544_at	predicted protein of unknown functi	---	AT4G01880	1.39	0.47	3.21	0.015594	0.051972	-3.68
252194_at	putative tyrosine phosphatase phos	ATPEN3	AT3G50110	-1.34	-0.42	-3.21	0.015603	0.051996	-3.68
259006_at	putative 60S acidic ribosomal prote	---	AT3G09200	1.54	0.63	3.21	0.01561	0.05201	-3.68
252587_at	putative protein Human (H326) trar	---	AT3G45620	1.64	0.71	3.21	0.015613	0.052012	-3.68
256006_at	hypothetical protein	---	AT1G34070	-1.25	-0.33	-3.21	0.015621	0.052031	-3.69
267277_at	putative protein transport protein SE	---	AT2G30050	-1.29	-0.37	-3.21	0.015625	0.052036	-3.69
249833_at	putative protein contains similarity t	---	AT5G23430	-1.37	-0.45	-3.21	0.015628	0.052041	-3.69
254207_at	putative protein Dof zinc finger prot	---	AT4G24060	-1.32	-0.40	-3.21	0.015644	0.052085	-3.69
263961_at	F-box protein family, AtFBL11 cont	---	AT2G36370	1.38	0.46	3.21	0.015648	0.052092	-3.69
255429_at	hypothetical protein	---	AT4G03410	-1.41	-0.50	-3.21	0.015657	0.052113	-3.69
258987_at	putative SCO1 protein similar to SC	---	AT3G08950	1.69	0.75	3.21	0.015667	0.052139	-3.69
245124_at	small nuclear ribonucleoprotein U1A	U1A	AT2G47580	1.31	0.38	3.21	0.015675	0.052157	-3.69
247164_at	NifS-like aminotranfserase ; suppo	NFS1	AT5G65720	1.30	0.38	3.21	0.015677	0.052157	-3.69
266981_at	60S ribosomal protein L23A identica	RPL23AA	AT2G39460	1.69	0.75	3.21	0.01569	0.052193	-3.69
266758_at	unknown protein	POL	AT2G46920	1.32	0.40	3.21	0.015693	0.052196	-3.69

267136_at	putative GTP-binding protein (extra	XLG1	AT2G23460	-1.31	-0.39	-3.21	0.015708	0.052238	-3.69
245621_at	AMP-binding protein	AAE15	AT4G14070	1.46	0.54	3.21	0.015715	0.052247	-3.69
247880_at	putative protein similar to unknown	---	AT5G57780	-2.55	-1.35	-3.21	0.015715	0.052247	-3.69
262886_at	hypothetical protein	KNATM	AT1G14760	-1.39	-0.47	-3.21	0.01572	0.052256	-3.69
247118_at	uridylyl transferases-like ; supporte	ACR1	AT5G65890	-1.53	-0.61	-3.21	0.015723	0.052257	-3.69
259662_at	Expressed protein ; supported by fu	HUB2	AT1G55250	1.41	0.50	3.21	0.015729	0.052269	-3.69
258417_at	Expressed protein ; supported by cl	---	AT3G17365	1.40	0.48	3.21	0.015734	0.052277	-3.69
257104_at	ER lumen retaining receptor (HDEI	---	AT3G25040	-1.28	-0.36	-3.21	0.015737	0.052282	-3.69
261412_at	L-ascorbate peroxidase identical to	APX1	AT1G07890	1.62	0.70	3.21	0.015744	0.052294	-3.69
252978_at	galactosidase like protein ss-1,4-ex	BGAL14	AT4G38590	-1.39	-0.47	-3.21	0.015745	0.052294	-3.69
251781_at	P-glycoprotein - like P-glycoprotein	PGP20	AT3G55320	1.30	0.37	3.21	0.015757	0.052325	-3.69
252199_at	anthranilate N-hydroxycinnamoyl/b	---	AT3G50270	1.35	0.43	3.21	0.01577	0.052361	-3.70
248274_at	isp4 protein	ATOPT9	AT5G53510	-1.26	-0.34	-3.20	0.015795	0.052434	-3.70
264522_at	putative xylan endohydrolase simila	---	AT1G10050	1.36	0.44	3.20	0.015817	0.0525	-3.70
266283_at	putative proline-rich protein	---	AT2G29210	1.31	0.39	3.20	0.015827	0.052527	-3.70
249988_at	putative protein predicted proteins,	---	AT5G18310	1.39	0.48	3.20	0.015842	0.052568	-3.70
255598_at	putative protein similar to nucleolin	---	AT4G00830	1.39	0.48	3.20	0.015866	0.052641	-3.70
257378_s_a	hypothetical protein predicted by ge	---	AT2G02290 ,	-1.32	-0.40	-3.20	0.015869	0.052642	-3.70
256478_at	unknown protein	---	---	-1.32	-0.40	-3.20	0.015876	0.052658	-3.70
248334_s_a	unknown protein ; supported by cD	---	AT5G52370 ,	1.57	0.65	3.20	0.015884	0.052678	-3.70
260276_at	unknown protein	---	AT1G80450	1.84	0.88	3.20	0.015895	0.052702	-3.70
245734_at	lysophospholipase homolog, putati	---	AT1G73480	-1.57	-0.65	-3.20	0.015897	0.052702	-3.70
251190_at	RING-H2 zinc finger protein ATL5 ;	ATL5	AT3G62690	-1.64	-0.71	-3.20	0.015899	0.052702	-3.70
258181_at	nitrate transporter identical to nitrate	---	AT3G21670	-1.48	-0.56	-3.20	0.015901	0.052702	-3.70
263167_at	unknown protein Belongs to PF|01	---	AT1G03030	1.35	0.43	3.20	0.015908	0.052718	-3.70
259023_at	unknown protein ; supported by cD	---	AT3G07510	-1.53	-0.62	-3.20	0.015912	0.052723	-3.70
250886_at	putative protein	---	AT5G04440	1.33	0.41	3.20	0.015923	0.052752	-3.71
254873_at	putative protein a 162 kDa compon	EIF3A	AT4G11420	1.26	0.34	3.20	0.015936	0.052786	-3.71
265120_at	3-ketoacyl-acyl carrier protein synth	KAS III	AT1G62640	-1.37	-0.45	-3.20	0.015938	0.052786	-3.71
258817_at	hypothetical protein similar to hypo	---	AT3G04750	1.24	0.31	3.20	0.015941	0.05279	-3.71
254969_at	hypothetical protein ;supported by f	---	AT4G10360	1.26	0.34	3.20	0.01597	0.052878	-3.71
254798_at	oleoyl-[acyl-carrier-protein] hydrola	---	AT4G13050	1.57	0.65	3.20	0.015984	0.052915	-3.71
246387_at	hypothetical protein predicted by ge	---	AT1G77400	1.41	0.49	3.19	0.016001	0.052966	-3.71
247846_at	putative protein	---	AT5G58100	1.29	0.37	3.19	0.016012	0.052995	-3.71
259546_at	unknown protein	---	AT1G35350	-1.91	-0.93	-3.19	0.016021	0.053015	-3.71

247624_at	aspartyl aminopeptidase - like prot	---	AT5G60160	1.22	0.29	3.19	0.016023	0.053015	-3.71
262116_at	Expressed protein ; supported by fu	---	AT1G02816	-1.71	-0.78	-3.19	0.016026	0.053017	-3.71
253333_at	2-dehydro-3-deoxyphosphoheptona	DHS2	AT4G33510	1.30	0.38	3.19	0.016049	0.053086	-3.71
246334_at	putative protein serine kinase SRP	---	AT3G44850	1.47	0.56	3.19	0.016061	0.053114	-3.71
250918_at	putative protein proline-rich protein	---	AT5G03610	3.07	1.62	3.19	0.016063	0.053114	-3.71
259609_at	myosin-like protein contains Pfam	TSA1	AT1G52410	-3.13	-1.65	-3.19	0.016066	0.053114	-3.71
250961_at	putative protein various predicted p	---	AT5G02980	-1.29	-0.37	-3.19	0.016067	0.053114	-3.72
254818_at	pEARLI 1-like protein Arabidopsis t	---	AT4G12470	2.94	1.56	3.19	0.016069	0.053114	-3.72
246959_at	unknown protein	---	AT5G24690	1.68	0.75	3.19	0.016075	0.053122	-3.72
246871_at	extensin - like protein pAP8 protein	---	AT5G26070	-1.24	-0.32	-3.19	0.016079	0.053122	-3.72
256371_at	hypothetical protein	---	AT1G66770	-1.27	-0.35	-3.19	0.016079	0.053122	-3.72
255121_at	putative mitogen-activated protein k	MAPKKK9	AT4G08480	1.48	0.56	3.19	0.016081	0.053122	-3.72
254233_at	98b like protein 98b - Daucus carot	---	AT4G23800	-1.53	-0.62	-3.19	0.016096	0.053164	-3.72
259736_at	endo-beta-1,4-glucanase, putative	AtGH9C2	AT1G64390	-3.77	-1.91	-3.19	0.01611	0.053203	-3.72
251570_at	putative protein several hypothetica	---	AT3G58300	-1.31	-0.39	-3.19	0.01613	0.053258	-3.72
246868_at	protein phosphatase - like protein p	---	AT5G26010	-1.27	-0.35	-3.19	0.016132	0.053258	-3.72
246317_at	putative protein unnamed ORF - H	---	AT3G56900	1.46	0.54	3.19	0.016137	0.053265	-3.72
266460_at	unknown protein ; supported by cDI	AGP26	AT2G47930	-5.67	-2.50	-3.19	0.016138	0.053265	-3.72
266649_at	putative aquaporin (tonoplast intrins	TIP4;1	AT2G25810	-1.25	-0.33	-3.19	0.016144	0.053266	-3.72
246822_at	putative protein predicted proteins,	---	AT5G26960	-1.33	-0.41	-3.19	0.016145	0.053266	-3.72
250524_at	putative protein contains similarity t	---	AT5G08520	-1.27	-0.34	-3.19	0.016146	0.053266	-3.72
245347_at	ferredoxin ;supported by full-length	---	AT4G14890	1.40	0.49	3.19	0.016153	0.053275	-3.72
265200_s_a	putative glucosyl transferase an ES	UGT73C6	AT2G36790	1.96	0.97	3.19	0.016153	0.053275	-3.72
256301_at	unknown protein ;supported by full-	---	AT1G69510	1.28	0.36	3.19	0.016162	0.05329	-3.72
247959_at	unknown protein	---	AT5G57080	1.43	0.52	3.19	0.016165	0.05329	-3.72
248478_at	ubiquitin-conjugating enzyme-like p	UBC27	AT5G50870	-1.24	-0.31	-3.19	0.016166	0.05329	-3.72
247401_at	1-deoxy-D-xylulose 5-phosphate re	DXR	AT5G62790	-1.48	-0.56	-3.19	0.016168	0.05329	-3.72
257598_at	PRT1 identical to GB:CAA11891 fr	PRT1	AT3G24800	-1.39	-0.47	-3.19	0.016171	0.05329	-3.72
258385_at	putative jasmonic acid regulatory pr	ATNAC2	AT3G15510	1.45	0.54	3.19	0.016171	0.05329	-3.72
249020_at	helicase-like protein	CHR4	AT5G44800	1.33	0.42	3.19	0.016175	0.053293	-3.72
258592_at	hypothetical protein contains Pfam	---	AT3G04130	1.33	0.41	3.19	0.016177	0.053294	-3.72
258956_at	hypothetical protein predicted by ge	---	AT3G01440	2.14	1.10	3.19	0.016183	0.053303	-3.72
256272_at	unknown protein	---	AT3G12100	-1.28	-0.35	-3.19	0.016185	0.053303	-3.72
263248_at	putative endonuclease ;supported	ATNTH1	AT2G31450	1.28	0.35	3.19	0.016196	0.053325	-3.72
262065_at	hypothetical protein predicted by ge	---	AT1G56180	1.55	0.63	3.19	0.016198	0.053325	-3.72

265664_at	unknown protein predicted by gens	---	AT2G24420	1.34	0.42	3.19	0.016199	0.053325	-3.72
266876_at	hypothetical protein predicted by ge	---	AT2G44820	1.31	0.40	3.19	0.016207	0.053344	-3.72
247323_at	putative protein similar to unknown	---	AT5G64170	1.45	0.54	3.19	0.016211	0.05335	-3.72
249087_at	DNA binding protein EREBP-3-like	ERF9	AT5G44210	-1.50	-0.59	-3.19	0.016215	0.053355	-3.72
257894_at	unknown protein ; supported by cDI	---	AT3G17100	-1.64	-0.72	-3.18	0.016223	0.053376	-3.73
252275_at	ubiquitin-specific protease 26 (UBF	UBP26	AT3G49600	1.43	0.52	3.18	0.016242	0.053428	-3.73
251893_at	putative protein	---	AT3G54380	1.27	0.34	3.18	0.016244	0.053428	-3.73
258552_at	putative pectate lyase similar to pec	---	AT3G07010	-4.42	-2.15	-3.18	0.016255	0.05345	-3.73
260606_at	putative splicing factor	---	AT2G43770	1.48	0.56	3.18	0.016255	0.05345	-3.73
266106_at	putative microtubule-associated pro	ATATG8E	AT2G45170	-1.51	-0.59	-3.18	0.016262	0.053464	-3.73
265373_at	putative replication protein A1	RPA1A	AT2G06510	1.61	0.69	3.18	0.016265	0.053466	-3.73
255310_at	Expressed protein ; supported by fu	ATALN	AT4G04955	1.88	0.91	3.18	0.016273	0.053486	-3.73
257725_at	DNA mismatch repair protein MSH	MSH2	AT3G18524	1.43	0.51	3.18	0.016288	0.053526	-3.73
248342_at	unknown protein	MBD13	AT5G52230	1.24	0.31	3.18	0.016302	0.053564	-3.73
261518_at	peroxidase ATP4a identical to GB:(	---	AT1G71695	-1.36	-0.44	-3.18	0.016304	0.053565	-3.73
256899_at	putative kinase-like protein TMKL1	TMKL1	AT3G24660	1.31	0.38	3.18	0.016312	0.053581	-3.73
251026_at	hypothetical protein	FHL	AT5G02200	-2.64	-1.40	-3.18	0.016322	0.053602	-3.73
247989_at	pyruvate kinase ;supported by full-l	---	AT5G56350	1.44	0.52	3.18	0.016325	0.053602	-3.73
249334_at	putative protein strong similarity to u	YSL4	AT5G41000	1.46	0.54	3.18	0.016325	0.053602	-3.73
262786_at	putative lipase similar to gb|X02844	---	AT1G10740	-1.43	-0.51	-3.18	0.016333	0.053622	-3.73
259861_at	unknown protein similar to ribosoma	---	AT1G80620	1.37	0.46	3.18	0.016338	0.053629	-3.73
256143_at	40S ribosomal protein S7 homolog	---	AT1G48830	1.39	0.47	3.18	0.01634	0.053629	-3.73
263396_at	unknown protein ;supported by full-	---	AT2G31710	1.31	0.39	3.18	0.016352	0.053657	-3.73
252188_at	putative clathrin coat assembly pro	---	AT3G50860	1.54	0.63	3.18	0.016354	0.053657	-3.73
248253_at	putative protein contains similarity t	CRF3	AT5G53290	-1.27	-0.34	-3.18	0.016356	0.053657	-3.73
256936_at	prefoldin-like protein similar to pref	PDF2	AT3G22480	1.29	0.36	3.18	0.016366	0.053682	-3.73
255424_at	putative glycosylation enzyme simi	---	AT4G03340	-1.28	-0.36	-3.18	0.016369	0.053684	-3.73
256757_at	membrane transporter, putative sin	---	AT3G25620	-1.30	-0.38	-3.18	0.016395	0.053762	-3.74
263066_at	unknown protein	---	AT2G17540	-1.23	-0.29	-3.18	0.016423	0.053845	-3.74
252424_at	putative protein activating signal co	---	AT3G47610	1.40	0.48	3.18	0.016428	0.053855	-3.74
264224_at	hypothetical protein predicted by ge	emb1688	AT1G67440	1.32	0.40	3.17	0.016447	0.053902	-3.74
258735_at	low temperature and salt responsive	RCI2A	AT3G05880	-1.53	-0.61	-3.17	0.016447	0.053902	-3.74
255080_at	arabinogalactan-protein homolog a	AGP10	AT4G09030	-1.56	-0.65	-3.17	0.016461	0.05394	-3.74
245365_at	hypothetical protein ;supported by f	---	AT4G17720	1.54	0.63	3.17	0.016468	0.053956	-3.74
254608_at	major intrinsic protein (MIP)- like no	NIP1;2	AT4G18910	1.84	0.88	3.17	0.016493	0.054026	-3.74

253545_at	AIG2-like protein AIG2 protein, Ara	---	AT4G31310	1.40	0.48	3.17	0.016494	0.054026	-3.74
255994_at	hypothetical protein predicted by ge	---	AT1G29760	1.26	0.33	3.17	0.016497	0.054027	-3.74
250695_at	lectin-like protein kinase	---	AT5G06740	2.04	1.03	3.17	0.016513	0.054071	-3.74
252219_at	putative protein some putative prote	---	AT3G50160	-1.34	-0.42	-3.17	0.016518	0.05408	-3.74
259582_at	hypothetical protein	---	AT1G28060	1.35	0.43	3.17	0.016523	0.054089	-3.74
249835_s_a	putative protein similar to unknown	---	AT5G23490	1.66	0.73	3.17	0.016526	0.054091	-3.74
260404_at	unknown protein similar to activator	---	---	1.52	0.61	3.17	0.016534	0.05411	-3.75
265051_at	jasmonate inducible protein, putativ	---	AT1G52100	-1.40	-0.49	-3.17	0.016539	0.054119	-3.75
245932_at	3'(2'),5'-bisphosphate nucleotidase	---	AT5G09290	-1.77	-0.82	-3.17	0.016546	0.054131	-3.75
247447_at	nitrate transporter NTL1 - like prote	---	AT5G62730	-1.86	-0.90	-3.17	0.016548	0.054131	-3.75
256624_at	hypothetical protein predicted by ge	---	AT3G19990	1.26	0.34	3.17	0.016552	0.054138	-3.75
266040_at	hypothetical protein	---	AT2G07738	-1.24	-0.30	-3.17	0.016568	0.054182	-3.75
258705_at	hypothetical protein predicted by ge	---	AT3G09470	1.30	0.38	3.17	0.016573	0.05419	-3.75
260002_at	putative ABC transporter similar to	ATNAP3	AT1G67940	1.51	0.60	3.17	0.016579	0.054202	-3.75
264365_s_a	unknown protein Strong similarity t	---	AT1G03220	1.91	0.93	3.17	0.016588	0.054225	-3.75
264931_at	polygalacturonase, putative similar	---	AT1G60590	-1.30	-0.37	-3.17	0.016603	0.054266	-3.75
248474_at	pyruvate dehydrogenase E1 compo	MAB1	AT5G50850	1.36	0.44	3.17	0.016611	0.054278	-3.75
255691_at	hypothetical protein	ANTR2	AT4G00370	1.33	0.41	3.17	0.016612	0.054278	-3.75
246738_at	replication factor C - like replication	EMB2775	AT5G27740	1.28	0.36	3.17	0.016624	0.054303	-3.75
261369_at	protein kinase, putative similar to m	---	AT1G53060	-1.39	-0.48	-3.17	0.016624	0.054303	-3.75
267552_at	putative purple acid phosphatase pr	PAP13	AT2G32770	-1.25	-0.32	-3.17	0.01663	0.054316	-3.75
258175_at	FKBP-type peptidyl-prolyl cis-trans	TWD1	AT3G21640	1.29	0.36	3.17	0.016639	0.054335	-3.75
263856_at	unknown protein	---	AT2G04410	1.42	0.50	3.17	0.016652	0.054371	-3.75
246401_at	DNA-binding protein, putative simil	AtMYB50	AT1G57560	-1.52	-0.60	-3.16	0.016671	0.054424	-3.75
264420_at	hypothetical protein predicted by ge	---	AT1G43320	-1.24	-0.31	-3.16	0.016687	0.054468	-3.76
257460_at	auxin induced protein, putative sim	---	AT1G75580	-1.27	-0.34	-3.16	0.01669	0.05447	-3.76
266028_at	hypothetical protein predicted by ge	---	AT2G06000	1.42	0.51	3.16	0.016692	0.05447	-3.76
262211_at	AP2 domain containing protein, pu	ORA47	AT1G74930	2.58	1.36	3.16	0.016699	0.05448	-3.76
255059_at	putative protein downy mildew resi	---	AT4G09420	-1.81	-0.86	-3.16	0.0167	0.05448	-3.76
264456_at	unknown protein similar to T cell re	---	AT1G10390	1.29	0.37	3.16	0.016706	0.054491	-3.76
266079_at	unknown protein	LCD1	AT2G37860	1.37	0.45	3.16	0.016708	0.054492	-3.76
248647_at	sucrose synthase	SUS2	AT5G49190	-1.25	-0.32	-3.16	0.016713	0.054499	-3.76
245437_at	RNA helicase like protein	---	AT4G16630	1.46	0.55	3.16	0.016725	0.054531	-3.76
247435_at	glutathione S-transferase-like prote	ATGSTU9	AT5G62480	4.27	2.09	3.16	0.016728	0.054532	-3.76
267240_at	hypothetical protein predicted by ge	---	AT2G02680	-1.32	-0.41	-3.16	0.016754	0.054608	-3.76

247378_at	ATP-dependent RNA helicase-like p	---	AT5G63120	-1.41	-0.50	-3.16	0.016761	0.054625	-3.76
259830_at	hypothetical protein predicted by ge	---	AT1G80630	-1.31	-0.38	-3.16	0.01677	0.054639	-3.76
266185_at	unknown protein	---	AT2G38950	1.26	0.33	3.16	0.016772	0.054639	-3.76
255608_at	hypothetical protein	---	AT4G01140	-1.39	-0.47	-3.16	0.016774	0.054639	-3.76
249899_at	putative protein similar to unknown	---	AT5G22620	-1.58	-0.66	-3.16	0.016775	0.054639	-3.76
252592_at	mitogen-activated protein kinase 3	NAXT1	AT3G45640	2.04	1.03	3.16	0.016787	0.054671	-3.76
253749_at	phytochrome-associated protein PA	PAP2	AT4G29080	-3.56	-1.83	-3.16	0.016803	0.054716	-3.76
255551_at	putative potassium channel ;suppo	KCO5	AT4G01840	1.23	0.30	3.16	0.016807	0.054721	-3.76
245481_at	hypothetical protein	---	AT4G16170	1.26	0.34	3.16	0.016822	0.054761	-3.76
267368_at	citrate synthase similar to GB:X175	ATCS	AT2G44350	1.33	0.41	3.16	0.016826	0.054766	-3.76
262278_at	transcription factor PERIANTHIA id	PAN	AT1G68640	-1.36	-0.44	-3.16	0.016871	0.054904	-3.77
265430_at	hypothetical protein predicted by ge	---	AT2G20700	-1.54	-0.62	-3.16	0.01688	0.054925	-3.77
260714_at	chaperonin CPN10 identical to SP:F	CPN10	AT1G14980	1.61	0.69	3.16	0.016891	0.054954	-3.77
254725_at	putative protein various predicted p	---	AT4G13650	1.34	0.43	3.15	0.0169	0.054968	-3.77
250984_at	protein kinase - like protein kinase	---	AT5G02800	1.23	0.30	3.15	0.0169	0.054968	-3.77
246426_at	ovule development protein aintegu	BBM	AT5G17430	-1.24	-0.32	-3.15	0.016905	0.05497	-3.77
258976_at	putative dehydrogenase contains F	---	AT3G01980	-1.24	-0.32	-3.15	0.016907	0.05497	-3.77
262570_at	unknown protein EST gb|H77143 co	---	AT1G15200	1.29	0.37	3.15	0.01691	0.05497	-3.77
258040_at	unknown protein ;supported by full-	---	AT3G21190	-3.84	-1.94	-3.15	0.01691	0.05497	-3.77
264866_at	putative metalloproteinase similar to	---	AT1G24140	4.08	2.03	3.15	0.016913	0.05497	-3.77
264293_at	cell division cycle protein, putative	APC6	AT1G78770	1.24	0.31	3.15	0.016938	0.055046	-3.77
246553_at	putative protein hypothetical protei	EDL1	AT5G15440	-1.31	-0.39	-3.15	0.016949	0.055073	-3.77
265026_at	putative 3-oxoacyl [acyl-carrier pro	---	AT1G24360	1.45	0.54	3.15	0.016981	0.055168	-3.77
264140_at	proteasome alpha subunit, putative	---	AT1G79210	1.26	0.33	3.15	0.016986	0.055169	-3.77
253060_at	putative protein predicted protein, A	---	AT4G37710	1.85	0.89	3.15	0.016986	0.055169	-3.77
250464_at	hypothetical protein	---	AT5G10040	-1.21	-0.28	-3.15	0.01699	0.055173	-3.77
249787_at	putative protein contains similarity t	---	AT5G24320	1.24	0.32	3.15	0.016996	0.05518	-3.77
266272_at	hypothetical protein predicted by ge	---	AT2G29600	-1.29	-0.37	-3.15	0.016997	0.05518	-3.77
265215_at	hypothetical protein predicted by ge	---	AT1G05040	-1.23	-0.30	-3.15	0.017007	0.055207	-3.78
263624_at	putative ferredoxin-thioredoxin redu	---	AT2G04700	-1.37	-0.45	-3.15	0.017015	0.055222	-3.78
248445_at	unknown protein ;supported by full-	---	AT5G51170	1.37	0.46	3.15	0.01702	0.055232	-3.78
260375_at	unknown protein	---	AT1G73950	1.30	0.37	3.15	0.017023	0.055234	-3.78
252351_at	putative protein ;supported by full-l	---	AT3G48210	1.37	0.46	3.15	0.017026	0.055234	-3.78
263379_at	putative CCCH-type zinc finger prot	CZF1	AT2G40140	2.83	1.50	3.15	0.017049	0.055302	-3.78
267595_at	putative glucanse	AtGH9B8	AT2G32990	-5.52	-2.46	-3.15	0.017054	0.055311	-3.78

255710_at	predicted protein of unknown functi	---	AT4G00030	-1.55	-0.64	-3.15	0.017062	0.055331	-3.78
266920_at	hypothetical protein predicted by ge	---	AT2G45750	-1.33	-0.41	-3.15	0.017075	0.055364	-3.78
262567_at	hypothetical protein contains similar	---	AT1G34300	1.50	0.58	3.15	0.01709	0.055405	-3.78
256776_at	multispanning membrane protein, p	---	AT3G13772	1.27	0.34	3.15	0.017093	0.055407	-3.78
259705_at	GRAB1-like protein similar to GRA	anac032	AT1G77450	1.54	0.62	3.15	0.017099	0.055418	-3.78
255466_at	putative protein similar to A. thaliar	---	AT4G03000	1.30	0.38	3.15	0.017105	0.055424	-3.78
267358_at	proline transporter 1 ;supported by	PROT1	AT2G39890	1.36	0.44	3.15	0.017106	0.055424	-3.78
260309_at	putative alanine aminotransferase s	AOAT2	AT1G70580	-1.39	-0.48	-3.15	0.017111	0.055434	-3.78
246246_at	putative protein	---	AT4G37170	1.37	0.46	3.15	0.017115	0.055438	-3.78
246650_at	putative protein p76, Homo sapiens	---	AT5G35160	-1.30	-0.38	-3.15	0.017117	0.055438	-3.78
254335_at	putative protein alternative oxidase	IM	AT4G22260	1.26	0.33	3.15	0.017121	0.05544	-3.78
253291_at	Expressed protein ; supported by fu	---	AT4G33865	1.42	0.50	3.14	0.017126	0.055445	-3.78
267317_at	putative proline-rich glycoprotein	---	AT2G34700	-1.27	-0.34	-3.14	0.017127	0.055445	-3.78
262132_at	ribosomal protein L22, putative sim	---	AT1G02830	-1.26	-0.33	-3.14	0.017147	0.055503	-3.78
261352_at	DNA repair protein RAD23, putativ	RAD23	AT1G79650	-1.30	-0.38	-3.14	0.017157	0.055525	-3.78
263538_at	hypothetical protein predicted by ge	---	AT2G25010	1.43	0.51	3.14	0.017165	0.055544	-3.79
252001_at	plastid division protein FtsZ-like Fts	FTSZ2-2	AT3G52750	-1.62	-0.70	-3.14	0.017197	0.055641	-3.79
264077_at	putative RAD51B-like DNA repair p	RAD51B	AT2G28560	-1.28	-0.35	-3.14	0.01721	0.055676	-3.79
250991_at	metallothionein 2b ; supported by f	MT2B	AT5G02380	1.31	0.39	3.14	0.017225	0.055715	-3.79
249589_at	pyridoxal kinase -like protein pyrido	SOS4	AT5G37850	1.22	0.29	3.14	0.017229	0.055721	-3.79
266355_at	hypothetical protein predicted by ge	---	AT2G01400	1.63	0.70	3.14	0.017263	0.055823	-3.79
247259_at	CPR5 protein, putative Constitutive	CPR5	AT5G64930	1.42	0.51	3.14	0.017266	0.055823	-3.79
250026_at	putative protein similar to unknown	---	AT5G18090	-1.32	-0.41	-3.14	0.01727	0.055823	-3.79
250170_at	putative protein ribulose-1,5-bisphc	---	AT5G14260	-1.33	-0.41	-3.14	0.017271	0.055823	-3.79
262486_at	putative bzip-like transcription facto	---	AT1G21740	-1.44	-0.53	-3.14	0.017295	0.05589	-3.79
261105_at	unknown protein ;supported by full-	NRS/ER	AT1G63000	-1.39	-0.47	-3.14	0.017296	0.05589	-3.79
253361_at	putative protein various predicted p	---	AT4G33100	1.55	0.63	3.14	0.017305	0.055911	-3.79
264807_at	putative presenilin similar to presen	---	AT1G08700	1.40	0.48	3.14	0.017316	0.055937	-3.79
252121_at	GDP-D-mannose-4,6-dehydratase	MUR1	AT3G51160	-1.65	-0.72	-3.14	0.017333	0.055984	-3.80
251318_at	putative protein putative protein At2	GDAP1	AT3G61570	-1.25	-0.33	-3.14	0.01734	0.055993	-3.80
246818_at	putative protein crp1, Zea mays, PI	EMB976	AT5G27270	1.68	0.75	3.14	0.01734	0.055993	-3.80
265074_at	hypothetical protein ; supported by	emb1011	AT1G55540	1.33	0.41	3.14	0.017344	0.055996	-3.80
251332_at	putative protein putative protein At2	---	AT3G61670	1.44	0.52	3.14	0.017347	0.055999	-3.80
251843_x_a	extensin precursor -like protein ext	ATHRGP1	AT3G54590	-1.61	-0.69	-3.14	0.017361	0.056036	-3.80
262066_at	hypothetical protein contains similar	---	AT1G79950	1.60	0.68	3.13	0.017364	0.056037	-3.80

252228_at	porin - like protein porin, Mesembry	VDAC5	AT3G49920	-1.24	-0.31	-3.13	0.017369	0.056047	-3.80
252680_at	putative protein predicted protein, (	---	AT3G44330	1.22	0.29	3.13	0.01738	0.056074	-3.80
267308_at	putative malonyl-CoA:Acyl carrier p	---	AT2G30200	1.34	0.42	3.13	0.017389	0.056093	-3.80
263488_at	hypothetical protein predicted by ge	---	AT2G31840	1.40	0.48	3.13	0.017391	0.056093	-3.80
254966_at	COP1 like protein photomorphoger	SPA2	AT4G11110	1.21	0.28	3.13	0.017397	0.056105	-3.80
257606_at	root hair defective 3 (RHD3) identic	RHD3	AT3G13870	-1.36	-0.44	-3.13	0.017402	0.056114	-3.80
249173_at	unknown protein	---	AT5G43000	-1.37	-0.45	-3.13	0.017406	0.056116	-3.80
258223_at	unknown protein ; supported by cD	PIFI	AT3G15840	1.33	0.41	3.13	0.017422	0.056159	-3.80
265415_at	unknown protein ;supported by full-	PSB29	AT2G20890	-1.31	-0.39	-3.13	0.017424	0.056159	-3.80
246259_at	amino acid permease, putative cor	---	AT1G31830	1.34	0.42	3.13	0.017426	0.05616	-3.80
264316_at	unknown protein ; supported by cDI	ENT1,AT	AT1G70330	-1.36	-0.44	-3.13	0.017429	0.05616	-3.80
265396_at	predicted protein	---	AT2G21040	-1.45	-0.53	-3.13	0.017433	0.056165	-3.80
264405_at	putative mitogen-activated protein	ATMPK1	AT1G10210	1.66	0.73	3.13	0.017436	0.056166	-3.80
260969_at	beta-fructosidase identical to beta-f	ATBETAFR	AT1G12240	-1.78	-0.83	-3.13	0.017454	0.056211	-3.80
247050_at	At14a protein-like	---	AT5G66660	-1.43	-0.51	-3.13	0.017455	0.056211	-3.80
259617_at	hypothetical protein predicted by ge	---	AT1G47970	1.34	0.42	3.13	0.017461	0.056222	-3.80
266192_at	unknown protein	---	AT2G39080	1.23	0.30	3.13	0.017493	0.056319	-3.81
265661_at	putative protein kinase contains a p	---	AT2G24360	1.34	0.42	3.13	0.017506	0.05635	-3.81
262495_at	unknown protein Contains similarity	---	AT1G21780	1.44	0.52	3.13	0.017508	0.05635	-3.81
255477_at	coded for by A. thaliana cDNA R64	---	AT4G02370	1.38	0.46	3.13	0.017512	0.056355	-3.81
251493_at	putative protein hypothetical protei	---	AT3G59300	1.36	0.44	3.13	0.017516	0.056362	-3.81
263965_at	putative retroelement pol polyprotei	---	---	-1.29	-0.37	-3.13	0.017519	0.056362	-3.81
251579_at	putative protein Bet1 homolog - Ra	BS14A	AT3G58170	1.26	0.33	3.13	0.017546	0.056438	-3.81
251639_at	acetylglutamate kinase-like protein	---	AT3G57560	-1.37	-0.45	-3.13	0.017548	0.056438	-3.81
250508_at	selenium-binding protein-like	---	AT5G09950	1.28	0.35	3.13	0.017563	0.056474	-3.81
264452_at	unknown protein similar to salt-indu	GRP23	AT1G10270	2.10	1.07	3.13	0.017564	0.056474	-3.81
254797_at	putative protein	---	AT4G13030	1.53	0.62	3.13	0.017582	0.056526	-3.81
248190_at	unknown protein	---	AT5G54130	1.29	0.36	3.13	0.017599	0.056572	-3.81
245454_at	disease resistance RPP5 like protei	---	AT4G16920	1.23	0.30	3.12	0.017603	0.056576	-3.81
256996_at	unknown protein similar to unknowr	---	AT3G25850	-1.23	-0.29	-3.12	0.01761	0.056591	-3.81
263980_at	hypothetical protein predicted by gr	---	AT2G42770	-1.37	-0.45	-3.12	0.017623	0.056604	-3.81
257452_at	hypothetical protein predicted by ge	---	AT1G05740	-1.24	-0.31	-3.12	0.017624	0.056604	-3.81
262929_at	hypothetical protein ;supported by f	---	AT1G65720	1.42	0.50	3.12	0.017624	0.056604	-3.81
256579_at	hypothetical protein	---	AT3G28720	1.24	0.31	3.12	0.017624	0.056604	-3.81
246691_at	putative protein predicted proteins -	---	---	-1.23	-0.30	-3.12	0.017641	0.056652	-3.81

267000_at	Expressed protein ; supported by fu	---	AT2G34310	-1.59	-0.67	-3.12	0.017655	0.056686	-3.81
261446_at	unknown protein	SEC5B	AT1G21170	-1.40	-0.48	-3.12	0.01766	0.056692	-3.82
250143_at	ADP-ribosylation factor - like protei	ATARFA1E	AT5G14670	-1.29	-0.37	-3.12	0.017664	0.056692	-3.82
262578_at	hypothetical protein Similar to gi|22	---	---	1.29	0.37	3.12	0.017664	0.056692	-3.82
254801_at	endoxyloglucan transferase-like pro	---	AT4G13080	-1.21	-0.27	-3.12	0.017687	0.056756	-3.82
245383_at	SUPERMAN like protein	---	AT4G17810	-1.58	-0.66	-3.12	0.017689	0.056756	-3.82
263842_at	Expressed protein ; supported by fu	---	AT2G36835	1.44	0.53	3.12	0.017691	0.056756	-3.82
245372_at	ribosomal protein ;supported by full-	---	AT4G15000	1.63	0.71	3.12	0.017699	0.056773	-3.82
258981_at	hypothetical protein predicted by ge	---	AT3G08880	1.23	0.30	3.12	0.017701	0.056773	-3.82
262024_at	protein disulfide isomerase, putativ	ATPDIL5-2	AT1G35620	1.36	0.44	3.12	0.017728	0.056849	-3.82
265626_at	unknown protein ;supported by full-	---	AT2G27260	-1.31	-0.39	-3.12	0.017731	0.056849	-3.82
263164_at	putative glutamate/aspartate-bindin	---	AT1G03070	-1.29	-0.36	-3.12	0.017732	0.056849	-3.82
258211_at	hypothetical protein predicted by ge	---	AT3G17890	1.40	0.48	3.12	0.017755	0.056913	-3.82
256929_at	unknown protein	---	---	-1.32	-0.40	-3.12	0.017759	0.056916	-3.82
257435_at	putative RSZp22 splicing factor	---	AT2G24590	1.40	0.48	3.12	0.017763	0.056922	-3.82
258100_at	unknown protein	---	AT3G23550	3.52	1.81	3.12	0.017767	0.056927	-3.82
252026_at	serine protein kinase - like serine p	SRPK4	AT3G53030	-1.29	-0.37	-3.12	0.017771	0.056932	-3.82
260424_at	putative receptor-like protein kinase	---	AT1G72460	-1.27	-0.34	-3.12	0.017791	0.056989	-3.82
248391_at	putative protein similar to unknown	---	AT5G52030	-1.30	-0.38	-3.12	0.0178	0.057009	-3.82
246245_at	putative protein	---	AT4G36440	1.28	0.35	3.12	0.017808	0.057025	-3.82
262903_at	NADPH-dependent codeinone redu	---	AT1G59950	2.83	1.50	3.12	0.017812	0.057031	-3.82
266794_at	hypothetical protein	---	AT2G02980	1.25	0.33	3.12	0.01782	0.057048	-3.82
263028_at	protein kinase, putative similar to P	---	AT1G24030	-1.40	-0.48	-3.12	0.017829	0.05707	-3.83
251925_at	hypothetical protein ;supported by f	---	AT3G54000	1.49	0.58	3.11	0.017854	0.05714	-3.83
252919_at	putative endo-1,4-beta-glucanase e	AtGH9B16	AT4G38990	-1.39	-0.48	-3.11	0.017881	0.057219	-3.83
266921_at	putative cytochrome P450	CYP86A8	AT2G45970	-2.00	-1.00	-3.11	0.017904	0.057284	-3.83
257637_at	terpene synthase-related protein co	---	AT3G25810	-1.23	-0.30	-3.11	0.017907	0.057284	-3.83
245175_at	putative protein disulfide-isomerase	UNE5	AT2G47470	1.57	0.65	3.11	0.017908	0.057284	-3.83
248532_at	Expressed protein ; supported by cl	NFU2	AT5G49940	1.34	0.43	3.11	0.017916	0.057299	-3.83
246640_x_a	putative protein predicted proteins,	---	---	-1.29	-0.36	-3.11	0.017923	0.057311	-3.83
260624_at	high-affinity nitrate transporter ACH	NRT2.2	AT1G08100	-1.43	-0.52	-3.11	0.017925	0.057311	-3.83
259571_at	hypothetical protein	---	AT1G20410	1.40	0.48	3.11	0.01793	0.057319	-3.83
245608_at	protein kinase	---	AT4G14350	-1.43	-0.52	-3.11	0.017932	0.057319	-3.83
248587_at	Expressed protein ; supported by fu	---	AT5G49550	-1.29	-0.37	-3.11	0.01794	0.057335	-3.83
252304_at	hypothetical protein	---	AT3G49230	-1.22	-0.29	-3.11	0.017942	0.057335	-3.83

256196_at	hypothetical protein predicted by ge	---	---	-1.32	-0.40	-3.11	0.017949	0.05735	-3.83
253131_at	homeodomain - like protein several	WOX13	AT4G35550	-1.35	-0.43	-3.11	0.017962	0.057384	-3.83
262185_at	MADS box transcription factor, puta	AGL67	AT1G77950	-1.22	-0.28	-3.11	0.01797	0.057394	-3.83
248222_at	GTPase activator protein of Rab-lik	---	AT5G53570	1.37	0.45	3.11	0.017971	0.057394	-3.83
256242_at	actin-like protein contains Pfam pro	ATARP5	AT3G12380	1.38	0.46	3.11	0.017975	0.057394	-3.83
251653_at	putative protein regulatory protein I	BOP1	AT3G57130	-1.34	-0.42	-3.11	0.017976	0.057394	-3.83
253272_at	putative protein ;supported by full-l	ʋɒɔ-01	AT4G34190	-1.45	-0.54	-3.11	0.017985	0.057413	-3.83
246119_at	ankyrin-repeat protein patsas prote	TIP1	AT5G20350	1.34	0.42	3.11	0.017987	0.057413	-3.83
262060_at	polygalacturonase, putative similar	---	AT1G80170	-1.30	-0.38	-3.11	0.018011	0.057482	-3.84
266780_at	putative ligand-gated ion channel pr	ATGLR2.8	AT2G29110	-1.48	-0.57	-3.11	0.018016	0.05749	-3.84
252198_x_a	putative protein predicted protein, A	---	AT3G50250	-1.46	-0.55	-3.11	0.018028	0.057521	-3.84
260937_at	hypothetical protein contains similar	---	AT1G45160	-1.22	-0.29	-3.11	0.018036	0.05754	-3.84
263160_at	oligouridylate binding protein, putat	UBP1A	AT1G54080	1.30	0.37	3.11	0.018043	0.057551	-3.84
251375_at	putative protein various predicted p	---	AT3G60410	-1.44	-0.53	-3.11	0.018045	0.057551	-3.84
254881_at	hypothetical protein	---	AT4G11700	-1.24	-0.31	-3.11	0.018047	0.057551	-3.84
249473_at	His-Asp Phosphotransfer Signal Tra	AHP3	AT5G39340	1.31	0.39	3.11	0.018062	0.057582	-3.84
253818_at	hypothetical protein	---	AT4G28330	-1.38	-0.47	-3.11	0.018062	0.057582	-3.84
247049_at	putative protein similar to unknown	---	AT5G66440	3.07	1.62	3.11	0.018067	0.057589	-3.84
249298_at	putative protein similar to unknown	---	AT5G41330	-1.27	-0.35	-3.11	0.018071	0.057595	-3.84
260215_at	unknown protein	---	AT1G74530	-1.28	-0.36	-3.11	0.018075	0.0576	-3.84
255859_at	putative protein embryonic abunda	---	AT5G34930	1.48	0.57	3.11	0.018083	0.057608	-3.84
255776_at	60S ribosomal protein L6, putative	---	AT1G18540	1.75	0.81	3.11	0.018083	0.057608	-3.84
258669_at	hypothetical protein predicted by ge	---	AT3G08800	1.25	0.32	3.10	0.018091	0.057614	-3.84
264823_at	putative serine/threonine protein ph	BSU1	AT1G03445	-1.41	-0.50	-3.10	0.018094	0.057614	-3.84
245941_at	putative protein karyopherin beta 3	emb2734	AT5G19820	-1.32	-0.40	-3.10	0.018095	0.057614	-3.84
250563_at	putative protein predicted protein, A	---	AT5G08050	-1.45	-0.54	-3.10	0.018095	0.057614	-3.84
256494_at	hypothetical protein predicted by ge	---	AT1G31520	-1.25	-0.32	-3.10	0.018121	0.057682	-3.84
247839_at	eukaryotic initiation factor 4, eIF4-li	---	AT5G57870	1.46	0.55	3.10	0.018121	0.057682	-3.84
259655_at	unknown protein	---	AT1G55210	2.17	1.12	3.10	0.018127	0.057692	-3.84
263589_at	hypothetical protein predicted by ge	---	AT2G25280	1.23	0.30	3.10	0.018144	0.057739	-3.84
246182_at	beta-1,3-glucanase - like protein pr	---	AT5G20870	-1.36	-0.44	-3.10	0.018154	0.057763	-3.84
256348_at	hypothetical protein predicted by ge	---	AT1G54880	-1.28	-0.35	-3.10	0.018163	0.057771	-3.84
264843_at	putative 1-aminocyclopropane-1-ca	---	AT1G03400	-1.53	-0.61	-3.10	0.018164	0.057771	-3.84
249368_at	putative protein predicted proteins,	---	AT5G40640	1.39	0.48	3.10	0.018165	0.057771	-3.85
251828_at	putative protein unnamed sequenc	---	AT3G55070	-1.30	-0.38	-3.10	0.018169	0.057778	-3.85

255865_at	unknown protein	---	AT2G30330	1.22	0.29	3.10	0.018175	0.057787	-3.85
267356_at	putative isoamylase	ISA1	AT2G39930	1.29	0.37	3.10	0.018182	0.057801	-3.85
265346_at	hypothetical protein	---	---	-1.27	-0.35	-3.10	0.018193	0.05783	-3.85
264957_at	F-box protein family, AtFBL5 conta	SKP2B	AT1G77000	1.55	0.63	3.10	0.018202	0.05785	-3.85
251522_at	hypothetical protein	---	AT3G59430	1.31	0.39	3.10	0.018247	0.057978	-3.85
259625_at	glyceraldehyde-3-phosphate dehyd	GAPB	AT1G42970	-1.37	-0.45	-3.10	0.018247	0.057978	-3.85
257426_at	hypothetical protein contains similar	---	AT1G54850	1.38	0.46	3.10	0.018254	0.057985	-3.85
253305_at	Expressed protein ; supported by fu	---	AT4G33666	-1.90	-0.93	-3.10	0.018257	0.057985	-3.85
246226_at	thiol-disulfide interchange like prote	HCF164	AT4G37200	1.41	0.50	3.10	0.018257	0.057985	-3.85
258447_at	hypothetical protein predicted by ge	---	AT3G22450	1.24	0.31	3.10	0.018269	0.058014	-3.85
264548_at	unknown protein ; supported by cDI	---	AT1G55680	1.28	0.35	3.10	0.018279	0.058036	-3.85
258648_at	putative auxin-independent growth	---	AT3G07900	-1.51	-0.60	-3.10	0.018281	0.058036	-3.85
252916_at	kinesin like protein kinesin-related	---	AT4G38950	-1.28	-0.35	-3.10	0.018286	0.058036	-3.85
262219_at	hypothetical protein predicted by ge	---	AT1G74750	1.49	0.57	3.10	0.018286	0.058036	-3.85
246511_at	UDP-glucose dehydrogenase-like p	---	AT5G15490	-1.79	-0.84	-3.10	0.018291	0.058044	-3.85
255728_at	unknown protein	---	AT1G25500	1.55	0.63	3.10	0.018314	0.058108	-3.85
267250_at	similar to hookless1 (HLS1)	---	AT2G23060	-1.24	-0.31	-3.10	0.018321	0.058123	-3.85
252905_at	putative protein predicted protein, A	---	AT4G39720	-1.37	-0.45	-3.10	0.018325	0.058125	-3.85
253278_at	receptor protein kinase - like protei	---	AT4G34220	-2.23	-1.16	-3.10	0.018327	0.058125	-3.85
264378_at	hypothetical protein predicted by ge	---	AT2G25220	-1.97	-0.98	-3.09	0.018367	0.058245	-3.86
262448_at	En/Spm-like transposon protein, pu	---	AT1G49450	-1.29	-0.36	-3.09	0.01837	0.058246	-3.86
264918_at	interferon-induced GTP-binding pro	---	AT1G60530	-1.27	-0.35	-3.09	0.018377	0.058259	-3.86
253719_at	putative protein, fragment prolidase	---	AT4G29490	1.22	0.28	3.09	0.018382	0.058261	-3.86
246086_at	beta-1,3-glucanase - like protein be	---	AT5G20560	-1.24	-0.31	-3.09	0.018382	0.058261	-3.86
261276_at	v-SNARE protein AtVTI1b, putative	VTI1B	AT1G26670	1.42	0.50	3.09	0.018387	0.058267	-3.86
266223_at	putative thaumatin ;supported by fu	---	AT2G28790	-2.47	-1.31	-3.09	0.018404	0.058308	-3.86
266838_at	similar to jasmonate-inducible prote	---	AT2G25980	-1.28	-0.35	-3.09	0.018405	0.058308	-3.86
247584_at	60S ribosomal protein L12 - like 60	---	AT5G60670	1.84	0.88	3.09	0.018423	0.058358	-3.86
259490_at	hypothetical protein preedicted by g	---	AT1G15800	-1.28	-0.35	-3.09	0.01844	0.058404	-3.86
266114_at	hypothetical protein predicted by ge	---	AT2G02150	-1.22	-0.28	-3.09	0.018446	0.058414	-3.86
247194_at	putative protein similar to unknown	---	AT5G65480	1.45	0.53	3.09	0.01845	0.058414	-3.86
245940_at	putative protein predicted proteins -	---	AT5G19630	-1.34	-0.42	-3.09	0.018451	0.058414	-3.86
260402_at	putative gamma-glutamyltransferas	GGT3	AT1G69820	-1.38	-0.47	-3.09	0.018461	0.058427	-3.86
247999_at	E2, ubiquitin-conjugating enzyme,	UBC30	AT5G56150	1.29	0.37	3.09	0.018462	0.058427	-3.86
246379_s_a	60S ribosomal protein L21, putative	---	AT1G57660	1.47	0.55	3.09	0.018465	0.058427	-3.86

267624_at	putative protein kinase	BIK1	AT2G39660	1.74	0.80	3.09	0.018466	0.058427	-3.86
254710_at	multidrug resistance protein/P-glyc	PGP9	AT4G18050	1.53	0.61	3.09	0.018481	0.058457	-3.86
267591_at	Expressed protein ; supported by fu	RTFL8	AT2G39705	2.02	1.01	3.09	0.018482	0.058457	-3.86
252129_at	putative protein hypothetical protei	AtHB28	AT3G50890	-1.31	-0.39	-3.09	0.018484	0.058457	-3.86
255259_at	coded for by A. thaliana cDNA W43	NDB2	AT4G05020	3.01	1.59	3.09	0.018485	0.058457	-3.86
264751_at	putative superoxide-generating NA	FRO3	AT1G23020	-2.24	-1.17	-3.09	0.01849	0.058465	-3.86
251615_at	putative protein hypothetical protei	---	AT3G57980	-1.46	-0.55	-3.09	0.018514	0.058531	-3.87
266438_at	putative carboxyphosphonoenolpyr	---	AT2G43180	1.36	0.45	3.09	0.018516	0.058531	-3.87
266913_at	hypothetical protein predicted by ge	ROPGEF4	AT2G45890	-1.22	-0.29	-3.09	0.018519	0.058532	-3.87
245246_at	hypothetical protein predicted by ge	---	AT1G44224	-1.25	-0.32	-3.09	0.018524	0.05854	-3.87
266796_at	predicted by genscan	---	AT2G02880	1.28	0.35	3.09	0.01854	0.058575	-3.87
258374_at	hypothetical protein predicted by ge	---	AT3G14360	-1.62	-0.70	-3.09	0.018541	0.058575	-3.87
246666_at	hypothetical protein	---	AT5G34830	-1.22	-0.28	-3.09	0.018568	0.058652	-3.87
250734_at	putative protein similar to unknown	---	AT5G06270	-1.88	-0.91	-3.09	0.018591	0.058716	-3.87
263979_at	En/Spm-like transposon protein rela	PDF1	AT2G42840	-1.42	-0.51	-3.08	0.018593	0.058717	-3.87
259423_at	unknown protein similar to putative	---	AT1G13880	-1.40	-0.49	-3.08	0.018599	0.058719	-3.87
249396_at	DegP protease contains similarity to	DegP9	AT5G40200	1.24	0.31	3.08	0.018599	0.058719	-3.87
245500_at	reverse transcriptase like protein	---	---	-1.25	-0.32	-3.08	0.018604	0.058727	-3.87
256263_at	5,10-methylenetetrahydrofolate del	---	AT3G12290	-1.30	-0.38	-3.08	0.018618	0.058755	-3.87
251431_at	protein synthesis initiation factor - I	EIF4G	AT3G60240	1.23	0.30	3.08	0.018618	0.058755	-3.87
256545_at	hypothetical protein predicted by ge	---	---	-1.29	-0.37	-3.08	0.018632	0.058789	-3.87
248305_at	putative protein similar to unknown	ATUSP	AT5G52560	-1.37	-0.45	-3.08	0.018634	0.058789	-3.87
261381_at	hypothetical protein predicted by ge	SDE3	AT1G05460	1.23	0.30	3.08	0.018638	0.058792	-3.87
264674_at	Expressed protein ; supported by fu	POLD4	AT1G09815	1.31	0.39	3.08	0.018646	0.058809	-3.87
247112_at	unknown protein	---	AT5G65950	1.29	0.37	3.08	0.018661	0.058849	-3.87
250698_at	putative protein similar to unknown	---	AT5G06810	1.25	0.32	3.08	0.018672	0.058875	-3.87
263150_at	heat-shock protein, putative similar	---	AT1G54050	-1.64	-0.71	-3.08	0.018674	0.058875	-3.87
250565_at	putative protein various predicted p	E13L3	AT5G08000	-1.37	-0.45	-3.08	0.018678	0.058878	-3.87
245081_at	unknown protein overlap with T20D	---	---	-1.47	-0.56	-3.08	0.018695	0.058923	-3.88
258213_at	hypothetical protein predicted by ge	---	AT3G17950	1.46	0.55	3.08	0.018697	0.058923	-3.88
246351_at	unknown protein contains similarity	---	AT1G16570	1.26	0.33	3.08	0.018706	0.058942	-3.88
260748_at	RING-H2 finger protein RHA3a, pu	---	AT1G49210	-2.52	-1.34	-3.08	0.018715	0.058962	-3.88
258388_at	putative expansin S2 precursor sim	ATEXPA12	AT3G15370	-1.23	-0.30	-3.08	0.018736	0.059019	-3.88
257420_at	hypothetical protein predicted by ge	---	AT1G77250	-1.34	-0.42	-3.08	0.018741	0.059029	-3.88
251754_at	beta-1,3-glucanase - like protein be	---	AT3G55780	-1.32	-0.40	-3.08	0.018761	0.059084	-3.88

255052_at	rab7 -like protein rab7 protein -Vigr	ATRABG3A	AT4G09720	-1.32	-0.40	-3.08	0.018776	0.05912	-3.88
266112_x_a	hypothetical protein predicted by ge	---	---	-1.33	-0.41	-3.08	0.018778	0.05912	-3.88
258697_at	putative minichromosome maintena	MCM8	AT3G09660	1.34	0.42	3.08	0.018794	0.059162	-3.88
263633_at	putative SCARECROW gene regula	SCL21	AT2G04890	1.35	0.44	3.08	0.018805	0.059189	-3.88
245688_at	prolin-rich protein, putative similar t	AGP31	AT1G28290	-3.73	-1.90	-3.08	0.018813	0.059207	-3.88
254688_at	DnaJ-like protein DnaJ-like protein	J20	AT4G13830	-1.27	-0.34	-3.08	0.018817	0.05921	-3.88
265498_at	putative C2H2-type zinc finger prote	---	AT2G15740	-1.23	-0.30	-3.08	0.018827	0.059233	-3.88
256969_at	unknown protein	---	AT3G21080	2.35	1.23	3.08	0.018831	0.059239	-3.88
259488_at	hypothetical protein preedicted by g	---	AT1G15780	1.37	0.46	3.08	0.018842	0.059263	-3.88
260951_at	hypothetical protein similar to hypot	---	AT1G06145	1.36	0.44	3.07	0.018851	0.059284	-3.88
263306_at	putative serine carboxypeptidase II	SCPL43	AT2G12480	-1.26	-0.33	-3.07	0.018854	0.059286	-3.88
262913_at	NADPH-dependent codeinone redu	---	AT1G59960	1.97	0.98	3.07	0.018875	0.059345	-3.89
257408_at	unknown protein similar to A. thalia	SON1	AT2G17310	-1.32	-0.40	-3.07	0.018891	0.059385	-3.89
262517_at	putative glutathione transferase Se	ATGSTU25	AT1G17180	4.22	2.08	3.07	0.018896	0.059388	-3.89
265375_at	unknown protein ;supported by full-	VPS2.1	AT2G06530	-1.37	-0.45	-3.07	0.018897	0.059388	-3.89
250932_s_a	putative protein MED7, Homo sapi	---	AT5G03220	1.26	0.33	3.07	0.018904	0.059402	-3.89
266598_at	3-methyl-2-oxobutanoate hydroxy-m	KPHMT1	AT2G46110	1.37	0.45	3.07	0.01891	0.059414	-3.89
249424_s_a	hypothetical protein ; supported by	---	AT5G39800	1.32	0.40	3.07	0.018919	0.059429	-3.89
256273_at	senescence-assocated protein, put	TET6	AT3G12090	1.33	0.42	3.07	0.01892	0.059429	-3.89
253531_at	putative protein tomato leucine zipp	ATEXO70G	AT4G31540	1.26	0.33	3.07	0.018928	0.059437	-3.89
259054_at	putative hypersensitivity-related ge	CHAT	AT3G03480	3.83	1.94	3.07	0.018928	0.059437	-3.89
263406_at	subtilisin-like serine protease AIR3	AIR3	AT2G04160	1.97	0.98	3.07	0.01894	0.059459	-3.89
247630_at	putative protein	SIZ1	AT5G60410	1.32	0.40	3.07	0.018941	0.059459	-3.89
250990_at	serine/threonine-specific protein kin	NAK	AT5G02290	1.60	0.68	3.07	0.018943	0.059459	-3.89
261084_at	tropinone reductase-I, putative sim	---	AT1G07440	1.51	0.60	3.07	0.018957	0.059494	-3.89
253864_at	hypothetical protein	---	AT4G27460	-1.35	-0.43	-3.07	0.018963	0.059507	-3.89
245374_at	hypothetical protein	---	AT4G17620	1.30	0.38	3.07	0.018973	0.059529	-3.89
251862_at	putative protein arm repeat contain	PUB14	AT3G54850	1.25	0.33	3.07	0.018981	0.059546	-3.89
245728_at	steroid 22-alpha-hydroxylase, puta	---	AT1G73340	-1.35	-0.43	-3.07	0.019016	0.059647	-3.89
262990_at	E2, ubiquitin-conjugating enzyme,	MMZ1	AT1G23260	-1.25	-0.32	-3.07	0.019038	0.059709	-3.89
247145_at	receptor protein kinase-like protein	---	AT5G65600	3.74	1.90	3.07	0.019056	0.059755	-3.90
252215_at	kinesin -like protein KINESIN-LIKE	KICP-02	AT3G50240	-2.02	-1.02	-3.07	0.019059	0.059756	-3.90
250385_at	aspartate aminotransferase (Asp3)	ASP3	AT5G11520	1.62	0.69	3.07	0.019067	0.059772	-3.90
245260_at	kinase like protein ; supported by cD	---	AT4G16360	1.23	0.30	3.07	0.019069	0.059772	-3.90
253079_s_a	putative protein F56F10.1, Caenor	---	AT4G36190	1.39	0.48	3.06	0.019109	0.059891	-3.90

266736_at	putative cytochrome P450 ; suppor	CYP709B1	AT2G46960	-1.24	-0.31	-3.06	0.019115	0.0599	-3.90
266016_at	putative RING zinc finger protein ;	---	AT2G18670	1.30	0.37	3.06	0.019119	0.059903	-3.90
264654_s_a	putative sugar transport protein, EF	---	AT1G08890	-1.51	-0.59	-3.06	0.019121	0.059903	-3.90
246621_at	putative protein strong similarity to	---	AT5G36230	1.20	0.27	3.06	0.019124	0.059903	-3.90
266449_at	hypothetical protein predicted by ge	AT-P4H-1	AT2G43080	1.32	0.40	3.06	0.019128	0.059907	-3.90
262285_at	hypothetical protein predicted by ge	---	AT1G68680	1.26	0.34	3.06	0.019131	0.05991	-3.90
265948_at	1-aminocyclopropane-1-carboxylate	ACO1	AT2G19590	-1.30	-0.38	-3.06	0.019155	0.059968	-3.90
262574_at	unknown protein ESTs gb|R30529,	---	AT1G15230	1.53	0.61	3.06	0.019155	0.059968	-3.90
250298_at	putative protein NLI-interacting fact	---	AT5G11860	1.30	0.38	3.06	0.019173	0.060016	-3.90
256717_at	hypothetical protein predicted by ge	---	AT2G34110	-1.32	-0.40	-3.06	0.019177	0.060019	-3.90
250070_at	phosphoribosylanthranilate transfer	---	AT5G17980	-1.53	-0.61	-3.06	0.01918	0.060021	-3.90
261311_at	disease resistance protein RTM1,	---	AT1G05770	-1.43	-0.51	-3.06	0.019192	0.060051	-3.90
246300_at	putative transmembrane protein G5	SAC8	AT3G51830	1.25	0.32	3.06	0.019209	0.060095	-3.90
259811_at	hypothetical protein predicted by ge	---	AT1G49830	-1.27	-0.35	-3.06	0.019235	0.060168	-3.91
246832_at	putative protein isopenicillin N epim	---	AT5G26600	1.32	0.40	3.06	0.019252	0.060212	-3.91
258525_at	hypothetical protein predicted by ge	IBR3	AT3G06810	1.41	0.49	3.06	0.019256	0.060212	-3.91
245505_at	glutaredoxin	---	AT4G15690	-1.93	-0.95	-3.06	0.019257	0.060212	-3.91
249568_at	glucosyltransferase-like protein UD	---	AT5G38040	1.33	0.41	3.06	0.019271	0.06025	-3.91
262854_at	unknown protein	---	AT1G20870	-1.28	-0.35	-3.06	0.019282	0.060273	-3.91
265041_at	unknown protein Contains similarity	TOM22-I	AT1G04070	1.48	0.57	3.06	0.019313	0.060362	-3.91
246849_at	putative protein various predicted p	---	AT5G26850	-1.75	-0.80	-3.06	0.019322	0.060383	-3.91
258273_at	unknown protein ;supported by full-	GRX4	AT3G15660	1.21	0.28	3.06	0.019334	0.060411	-3.91
263197_at	polyubiquitin, putative similar to po	---	AT1G53930	-1.32	-0.40	-3.06	0.01935	0.060447	-3.91
263826_at	hypothetical protein predicted by ge	---	AT2G40410	-1.40	-0.48	-3.06	0.01935	0.060447	-3.91
254984_s_a	putative protein Homo sapiens deu	UBP10	AT4G10570	1.20	0.26	3.06	0.019358	0.060464	-3.91
261511_at	polyadenylate-binding protein 5 ide	PAB5	AT1G71770	-1.31	-0.39	-3.06	0.019368	0.060485	-3.91
261414_at	Expressed protein ; supported by fu	---	AT1G07795	-1.44	-0.52	-3.05	0.019376	0.060498	-3.91
254922_at	RING-H2 finger protein RHA1a -like	RHA1A	AT4G11370	4.20	2.07	3.05	0.019377	0.060498	-3.91
262716_at	multicatalytic endopeptidase identi	PAB1	AT1G16470	1.38	0.46	3.05	0.019428	0.060648	-3.92
261460_at	MAP kinase, putative similar to GB	ATMPK13	AT1G07880	-1.21	-0.27	-3.05	0.019436	0.06066	-3.92
247843_at	aluminium tolerance associated - lik	SVL4	AT5G58050	-1.32	-0.40	-3.05	0.019437	0.06066	-3.92
258043_at	unknown protein ; supported by cD	---	AT3G21290	1.22	0.29	3.05	0.019441	0.060664	-3.92
258015_at	unknown protein	---	AT3G19340	-1.31	-0.39	-3.05	0.019444	0.060665	-3.92
264325_at	putative phosphoribosylanthranilate	---	AT1G04150	-1.37	-0.45	-3.05	0.019453	0.060683	-3.92
251113_at	hypothetical protein	---	AT5G01370	-1.35	-0.43	-3.05	0.019466	0.060718	-3.92

249464_at	putative protein crp1, Zea mays, Pl	EMB2745	AT5G39710	1.82	0.87	3.05	0.01948	0.060744	-3.92
247346_at	diacylglycerol kinase	ATDGK2	AT5G63770	1.44	0.53	3.05	0.01948	0.060744	-3.92
260601_at	Expressed protein ; supported by cl	ZIP11	AT1G55910	1.54	0.63	3.05	0.01949	0.060761	-3.92
259083_at	putative kinase similar to NIMA-rela	ATNEK2	AT3G04810	-1.26	-0.33	-3.05	0.019491	0.060761	-3.92
262175_at	unknown protein ; supported by cDI	NDH-O	AT1G74880	1.39	0.48	3.05	0.019494	0.060761	-3.92
245306_at	Expressed protein ; supported by fu	ELIP2	AT4G14690	-1.34	-0.42	-3.05	0.019512	0.06081	-3.92
266749_at	putative protein kinase contains a p	---	AT2G47060	-1.67	-0.74	-3.05	0.019519	0.060822	-3.92
259626_at	bZIP transcription factor, putative c	ATBZIP60	AT1G42990	2.44	1.29	3.05	0.01953	0.060849	-3.92
261712_at	alcohol dehydrogenase, putative si	---	AT1G32780	-1.30	-0.38	-3.05	0.019543	0.060881	-3.92
264341_at	hypothetical protein predicted by ge	---	AT1G70270	-1.22	-0.29	-3.05	0.01955	0.060896	-3.92
251631_at	putative protein several hypothetica	---	AT3G57430	1.31	0.39	3.05	0.019559	0.060915	-3.92
253506_at	putative protein EREBP-4 homolog	---	AT4G31980	-1.50	-0.59	-3.05	0.019597	0.061018	-3.93
263584_at	NAM (no apical meristem)-like prote	anac036	AT2G17040	2.16	1.11	3.05	0.019599	0.061018	-3.93
260297_at	unknown protein contains Pfam pro	---	AT1G80280	-1.30	-0.38	-3.05	0.019603	0.061018	-3.93
259118_at	unknown protein similar to unknow	---	AT3G01310	1.36	0.44	3.05	0.019604	0.061018	-3.93
254179_at	putative protein various predicted p	---	AT4G23910	1.42	0.50	3.05	0.019608	0.061018	-3.93
246989_at	unknown protein	---	AT5G67350	1.90	0.92	3.05	0.019608	0.061018	-3.93
257010_at	Expressed protein ; supported by cl	ATEXO70D	AT3G14090	-1.46	-0.55	-3.05	0.019617	0.061036	-3.93
253043_at	putative protein predicted protein, A	LBD39	AT4G37540	1.60	0.68	3.05	0.019619	0.061036	-3.93
250648_at	late embryogenesis abundant prote	---	AT5G06760	-1.30	-0.38	-3.05	0.01964	0.061092	-3.93
250764_at	putative protein similar to unknown	---	AT5G05960	-6.32	-2.66	-3.04	0.019649	0.061112	-3.93
266126_at	putative metalloproteinase ; suppor	---	AT2G45040	1.51	0.59	3.04	0.019652	0.061114	-3.93
254359_at	putative protein hypothetical protei	---	AT4G22360	-1.30	-0.38	-3.04	0.019688	0.061215	-3.93
251266_at	putative protein hypothetical protei	---	AT3G62320	-1.33	-0.41	-3.04	0.01969	0.061215	-3.93
247476_at	putative protein	---	AT5G62330	-1.26	-0.33	-3.04	0.019693	0.061216	-3.93
250944_at	putative protein	---	AT5G03380	2.40	1.26	3.04	0.019707	0.06125	-3.93
259552_at	hypothetical protein	---	AT1G21320	-1.32	-0.40	-3.04	0.019709	0.06125	-3.93
245700_at	carbonate dehydratase - like protein	ACA3	AT5G04180	-1.24	-0.31	-3.04	0.019721	0.061276	-3.93
245934_at	putative protein NAC1 - Medicago t	anac082	AT5G09330	1.37	0.45	3.04	0.019724	0.061278	-3.93
252773_at	putative protein predicted proteins,	---	---	-1.23	-0.30	-3.04	0.019734	0.0613	-3.93
247131_at	ferredoxin-NADP+ reductase	FNR1	AT5G66190	-1.24	-0.31	-3.04	0.019739	0.061309	-3.93
265788_at	putative auxin-induced protein	IAA32	AT2G01200	-1.23	-0.29	-3.04	0.019749	0.06133	-3.93
260925_at	DNA-binding protein, putative conta	---	AT1G21340	-1.31	-0.39	-3.04	0.019771	0.061387	-3.93
261212_at	hypothetical protein predicted by ge	atnudt12	AT1G12880	1.46	0.54	3.04	0.019773	0.061387	-3.93
248852_at	unknown protein	---	AT5G46560	1.21	0.28	3.04	0.019776	0.061391	-3.93

262875_at	gamma-tocopherol methyltransfera	G-TMT	AT1G64970	1.32	0.40	3.04	0.019783	0.061404	-3.94
254265_s_a	serine threonine kinase - like prote	CRK6	AT4G23140	2.48	1.31	3.04	0.019806	0.061465	-3.94
264862_at	hypothetical protein predicted by ge	---	AT1G24330	-1.45	-0.53	-3.04	0.019813	0.061478	-3.94
249501_s_a	expansin-like protein expansin - Pr	ATEXP26	AT5G39270	-1.21	-0.28	-3.04	0.019834	0.061536	-3.94
259139_at	hypothetical protein similar to F16N	---	AT3G10240	-1.26	-0.34	-3.04	0.01984	0.061541	-3.94
252828_at	DnaJ - like protein DnaJ-like protei	---	AT4G39960	1.31	0.38	3.04	0.019841	0.061541	-3.94
247814_at	polyneuridine aldehyde esterase-lik	MES18	AT5G58310	-2.83	-1.50	-3.04	0.019854	0.061573	-3.94
245206_at	putative protein similarity to NCA2	DGS1	AT5G12290	1.31	0.39	3.04	0.019869	0.061607	-3.94
252470_at	protein kinase 6-like protein protein	---	AT3G46930	1.96	0.97	3.04	0.01987	0.061607	-3.94
266273_at	putative zinc transporter	MTPB1	AT2G29410	2.48	1.31	3.04	0.019877	0.061621	-3.94
265296_at	hypothetical protein predicted by ge	---	AT2G14060	-1.25	-0.32	-3.04	0.019882	0.061627	-3.94
251594_at	putative protein DOC4, Mus muscu	---	AT3G57630	2.12	1.08	3.04	0.019886	0.061631	-3.94
258296_at	putative ribosomal protein similar to	---	AT3G23390	1.72	0.78	3.04	0.019899	0.061663	-3.94
258214_at	amidase, putative similar to amidas	atToc64-III	AT3G17970	1.55	0.64	3.03	0.019939	0.061779	-3.94
252235_at	60S RIBOSOMAL PROTEIN - like	---	AT3G49910	1.60	0.67	3.03	0.019942	0.061779	-3.94
249221_at	serine/threonine protein kinase-like	---	AT5G42440	1.36	0.44	3.03	0.019962	0.061821	-3.94
250730_at	C3HC4-type RING zinc finger prote	---	AT5G06490	-1.28	-0.35	-3.03	0.019964	0.061821	-3.94
249974_at	putative protein predicted proteins,	---	AT5G18780	1.63	0.71	3.03	0.019964	0.061821	-3.94
265116_at	unknown protein ; supported by full-	---	AT1G62480	3.16	1.66	3.03	0.019967	0.061821	-3.94
251513_at	pirin-like protein pirin - Lycopersico	PRN	AT3G59220	-2.41	-1.27	-3.03	0.019972	0.061829	-3.95
255538_at	putative transcription factor ; suppo	MYB55	AT4G01680	-1.92	-0.94	-3.03	0.019984	0.061859	-3.95
248283_at	pyruvate kinase ;supported by full-l	PKP-BETA	AT5G52920	1.54	0.62	3.03	0.020009	0.061921	-3.95
247874_at	101 kDa heat shock protein; HSP10	---	AT5G57710	-1.45	-0.53	-3.03	0.02001	0.061921	-3.95
245248_at	Expressed protein ; supported by cl	ATB' GAMM	AT4G15415	1.19	0.26	3.03	0.020016	0.061933	-3.95
247643_at	auxin response factor 4 ; supporte	ARF4	AT5G60450	1.47	0.56	3.03	0.02003	0.061967	-3.95
250072_at	putative protein similar to unknown	---	AT5G17210	-1.42	-0.50	-3.03	0.020049	0.062019	-3.95
245524_at	cytochrome c oxidoreductase like p	---	AT4G15920	-1.99	-1.00	-3.03	0.020062	0.062049	-3.95
250568_at	putative protein several putative AT	---	AT5G08110	1.28	0.35	3.03	0.020065	0.062049	-3.95
259985_at	unknown protein ; supported by cD	---	AT1G76620	-1.26	-0.34	-3.03	0.020073	0.062054	-3.95
248025_at	NOI protein, nitrate-induced	NOI	AT5G55850	-1.33	-0.42	-3.03	0.020074	0.062054	-3.95
255883_at	unknown protein similar to putative	---	AT1G20270	1.34	0.43	3.03	0.020074	0.062054	-3.95
256333_at	Sm-like protein contains Pfam profil	---	AT1G76860	1.47	0.56	3.03	0.020081	0.062068	-3.95
267350_at	putative DNA-directed RNA polyme	NRPD1B	AT2G40030	-1.34	-0.42	-3.03	0.020095	0.062084	-3.95
259313_at	hypothetical protein predicted by ge	---	AT3G05090	1.32	0.40	3.03	0.020097	0.062084	-3.95
266309_at	unknown protein ;supported by full-	---	AT2G27140	-1.41	-0.49	-3.03	0.020098	0.062084	-3.95

267017_at	unknown protein	SVR1	AT2G39140	1.71	0.77	3.03	0.020098	0.062084	-3.95
252037_at	putative calmodulin calmodulin - Te	CAM9	AT3G51920	1.47	0.56	3.03	0.0201	0.062084	-3.95
256261_at	ras-related GTP-binding protein cor	RABA4D	AT3G12160	-1.26	-0.34	-3.03	0.020124	0.062149	-3.95
259801_at	blue copper protein, putative simila	---	AT1G72230	-1.82	-0.87	-3.03	0.020145	0.062205	-3.95
260479_at	hypothetical protein predicted by ge	---	AT1G11020	1.23	0.30	3.03	0.020153	0.062222	-3.95
245359_at	carnitine racemase like protein ;sup	IBR10	AT4G14430	1.56	0.65	3.03	0.020167	0.062255	-3.96
267605_at	putative protein disulfide isomerase	ATPDIL2-3	AT2G32920	1.38	0.46	3.02	0.020192	0.062325	-3.96
258289_at	hypothetical protein predicted by ge	---	AT3G23450	-1.24	-0.31	-3.02	0.020195	0.062325	-3.96
257540_at	hypothetical protein	---	AT3G21520	-1.30	-0.38	-3.02	0.020202	0.062337	-3.96
248639_at	anthranilate N-benzoyltransferase	HCT	AT5G48930	1.48	0.57	3.02	0.020204	0.062337	-3.96
245264_at	Expressed protein ; supported by cl	---	AT4G17245	-2.09	-1.06	-3.02	0.020208	0.062341	-3.96
267579_at	metallothionein-like protein	---	AT2G42000	-1.22	-0.29	-3.02	0.020217	0.06236	-3.96
256614_at	hypothetical protein predicted by ge	---	AT3G29300	-1.26	-0.34	-3.02	0.020222	0.062367	-3.96
261402_at	wall-associated kinase 4, putative s	RFO1	AT1G79670	1.53	0.62	3.02	0.02024	0.062409	-3.96
253838_at	putative zinc finger protein zinc fing	---	AT4G27880	-1.45	-0.53	-3.02	0.020243	0.062409	-3.96
245532_at	cytochrome P450 like protein	CYP97B3	AT4G15110	-1.20	-0.27	-3.02	0.020244	0.062409	-3.96
261536_at	K Efflux antiporter KEA1 identical to	KEA1	AT1G01790	-1.39	-0.47	-3.02	0.020249	0.062415	-3.96
248799_at	ethylene responsive element bindin	ERF5	AT5G47230	3.63	1.86	3.02	0.020258	0.062437	-3.96
247177_at	unknown protein ; supported by cDI	---	AT5G65300	2.13	1.09	3.02	0.020265	0.062448	-3.96
261879_at	cytochrome P450, putative similar t	CYP705A2	AT1G50520	-1.24	-0.31	-3.02	0.020285	0.062502	-3.96
253552_at	ubiquitin-specific protease 24 (UBF	UBP24	AT4G30890	1.25	0.33	3.02	0.02032	0.062595	-3.96
259784_at	auxin-induced protein, putative sim	---	AT1G29450	-1.67	-0.74	-3.02	0.020322	0.062595	-3.96
254752_at	putative protein IFA-binding proteir	---	AT4G13160	1.28	0.36	3.02	0.020323	0.062595	-3.96
249790_at	putative protein similar to unknown	---	AT5G24290	-2.68	-1.42	-3.02	0.020332	0.062611	-3.96
251550_at	putative protein proline-rich protein	---	AT3G58800	-1.29	-0.37	-3.02	0.020381	0.062756	-3.97
249613_at	putative protein mDj10, Mus muscu	---	AT5G37380	1.35	0.43	3.02	0.020387	0.062766	-3.97
264314_at	unknown protein ; supported by cDI	---	AT1G70420	1.50	0.58	3.02	0.02039	0.062767	-3.97
247428_at	unknown protein	---	AT5G62600	1.22	0.29	3.02	0.020402	0.062792	-3.97
246283_at	putative protein	---	AT4G36860	-1.33	-0.42	-3.02	0.020404	0.062792	-3.97
248597_at	DNA (cytosine-5)-methyltransferase	MET1	AT5G49160	1.73	0.79	3.02	0.020445	0.062909	-3.97
262746_at	Expressed protein ; supported by fu	ATNUDX15	AT1G28960	1.21	0.28	3.02	0.020455	0.062933	-3.97
250862_s_a	40S ribosomal protein S17 -like 40	---	AT2G04390	1.51	0.60	3.02	0.020462	0.062936	-3.97
262050_at	unknown protein ;supported by full-	---	AT1G80130	-2.81	-1.49	-3.02	0.020463	0.062936	-3.97
265646_at	putative lipase	---	AT2G27360	1.30	0.38	3.02	0.020464	0.062936	-3.97
262768_at	putative beta-1,4-N-acetylglucosan	---	AT1G12990	-1.74	-0.80	-3.02	0.020469	0.06294	-3.97

246563_at	putative protein	---	AT5G15581	-1.33	-0.41	-3.01	0.020474	0.062949	-3.97
246655_at	hypothetical protein	---	AT5G35230	-1.26	-0.33	-3.01	0.020479	0.062952	-3.97
254225_at	putative major latex protein major l	---	AT4G23670	-2.90	-1.54	-3.01	0.020481	0.062952	-3.97
262260_at	major latex protein (MLP149), puta	MLP34	AT1G70850	-1.42	-0.51	-3.01	0.02049	0.062972	-3.97
246779_at	putative protein peroxisomal integra	PNC2	AT5G27520	-1.57	-0.65	-3.01	0.020512	0.063028	-3.97
261794_at	hypothetical protein contains similar	---	AT1G16060	-1.34	-0.43	-3.01	0.020514	0.063028	-3.97
254053_s_a	SRG1-like protein strong homology	---	AT4G25300	1.56	0.65	3.01	0.020555	0.063147	-3.98
260785_at	hypothetical protein predicted by ge	---	AT1G06200	1.26	0.34	3.01	0.020576	0.063203	-3.98
253476_at	S-receptor kinase -like protein serin	SD2-5	AT4G32300	-1.64	-0.71	-3.01	0.020607	0.063289	-3.98
253695_at	arginine methyltransferase (pam1)	PRMT11	AT4G29510	1.41	0.50	3.01	0.020615	0.063306	-3.98
263504_s_a	hypothetical protein	---	AT2G07677	1.24	0.31	3.01	0.020619	0.063308	-3.98
258437_at	unknown protein contains protein pl	---	AT3G16560	1.46	0.55	3.01	0.020627	0.063318	-3.98
260499_at	unknown protein ; supported by cD	---	AT2G41760	-1.24	-0.31	-3.01	0.020628	0.063318	-3.98
260944_at	beta-galactosidase, putative simila	BGAL5	AT1G45130	-4.32	-2.11	-3.01	0.020644	0.063358	-3.98
248591_at	xylulose kinase ; supported by cDN	---	AT5G49650	-1.31	-0.39	-3.01	0.020648	0.063358	-3.98
246323_at	hypothetical protein predicted by ge	---	AT1G16690	-1.33	-0.41	-3.01	0.020649	0.063358	-3.98
257279_at	unknown protein ;supported by full-	---	AT3G14430	1.27	0.34	3.01	0.020656	0.06337	-3.98
248889_at	unknown protein ;supported by full-	---	AT5G46230	1.50	0.59	3.01	0.020673	0.063415	-3.98
265770_at	Expressed protein ; supported by fu	---	AT2G48100	1.34	0.43	3.01	0.020687	0.063449	-3.98
245888_at	putative protein DNA-binding prote	---	AT5G09450	1.28	0.35	3.01	0.020691	0.063453	-3.98
261387_at	hypothetical protein predicted by ge	---	AT1G05410	1.52	0.61	3.01	0.02071	0.063504	-3.98
262441_at	hypothetical protein similar to hypot	OSB1	AT1G47720	1.32	0.40	3.01	0.020728	0.063548	-3.98
259120_at	unknown protein	---	AT3G02240	4.43	2.15	3.00	0.020761	0.063641	-3.99
256613_at	hypothetical protein predicted by ge	emb2076	AT3G29290	1.36	0.45	3.00	0.020802	0.06376	-3.99
258249_s_a	unknown protein ; supported by ful	---	AT3G15820	-1.43	-0.51	-3.00	0.020823	0.063815	-3.99
263585_at	60S ribosomal protein L39	---	AT2G25210	1.69	0.76	3.00	0.020837	0.063845	-3.99
266231_at	putative protein kinase	PSKR1	AT2G02220	2.21	1.14	3.00	0.020838	0.063845	-3.99
250163_at	putative protein various predicted p	---	AT5G15270	-1.30	-0.38	-3.00	0.020845	0.063857	-3.99
261698_at	unknown protein identical to hypoth	---	AT1G32630	1.34	0.42	3.00	0.020855	0.063878	-3.99
262046_at	hypothetical protein predicted by ge	OFP14	AT1G79960	-1.35	-0.44	-3.00	0.020882	0.063946	-3.99
263663_at	putative malate dehydrogenase sim	---	AT1G04410	1.26	0.34	3.00	0.020883	0.063946	-3.99
248818_at	Lon protease homolog 1 precursor	LON2	AT5G47040	-1.37	-0.46	-3.00	0.020887	0.063951	-3.99
247139_at	unknown protein ;supported by full-	---	AT5G66090	1.28	0.36	3.00	0.020892	0.063953	-3.99
246987_at	myb-related protein, 33.3K (pir |S7	MYBR1	AT5G67300	-1.58	-0.66	-3.00	0.020893	0.063953	-3.99
255104_at	Expressed protein ; supported by fu	SAH7	AT4G08685	-2.37	-1.24	-3.00	0.020907	0.063981	-3.99

260874_at	hypothetical protein predicted by ge	emb2170	AT1G21390	1.33	0.41	3.00	0.020908	0.063981	-3.99
248108_at	molybdopterin synthase sulphurylas	CNX5	AT5G55130	1.31	0.38	3.00	0.020914	0.063992	-3.99
247873_at	diacylglycerol kinase-like protein	---	AT5G57690	-1.24	-0.31	-3.00	0.020927	0.064021	-3.99
259753_at	putative isoprenylated protein simila	---	AT1G71050	-1.44	-0.52	-3.00	0.020939	0.064051	-4.00
250449_at	embryonic abundant protein -like e	---	AT5G10830	-1.69	-0.76	-3.00	0.020942	0.064051	-4.00
258350_at	unknown protein similar to serine/th	CIPK1	AT3G17510	-1.67	-0.74	-3.00	0.020951	0.064071	-4.00
262275_at	ATPase, putative similar to chroma	---	AT1G68710	1.30	0.38	3.00	0.020959	0.064084	-4.00
255385_at	putative hydrolase	---	AT4G03610	-1.36	-0.44	-3.00	0.020977	0.064131	-4.00
251207_at	putative protein ;supported by full-l	---	AT3G63050	1.57	0.65	3.00	0.020988	0.064157	-4.00
249601_at	quinone oxidoreductase -like prote	---	AT5G37980	-1.36	-0.45	-3.00	0.020997	0.064162	-4.00
266768_s_a	ubiquitin extension protein (UBQ6)	UBQ6	AT2G47110	1.44	0.53	3.00	0.020997	0.064162	-4.00
253317_at	putative protein	---	AT4G33960	1.74	0.80	3.00	0.020998	0.064162	-4.00
252502_at	copper transport protein - like prote	COPT2	AT3G46900	1.99	0.99	3.00	0.021008	0.064185	-4.00
253149_at	NAD+ dependent isocitrate dehydr	---	AT4G35650	-1.28	-0.36	-3.00	0.021016	0.064198	-4.00
266949_s_a	unknown protein ;supported by full-	---	AT2G18840	-1.34	-0.42	-3.00	0.021018	0.064198	-4.00
265792_at	predicted by genscan and genefind	---	AT2G01390	-1.31	-0.39	-3.00	0.021024	0.064202	-4.00
245514_at	OEP8 like protein	---	AT4G15810	-1.44	-0.53	-3.00	0.021025	0.064202	-4.00
255808_at	putative protein predicted protein, A	---	AT4G10280	1.29	0.36	3.00	0.021034	0.06422	-4.00
254164_at	putative protein ;supported by full-l	TIFY1	AT4G24470	1.28	0.35	2.99	0.021045	0.064245	-4.00
257102_at	endoxyloglucan transferase, putati	XTH3	AT3G25050	-1.21	-0.28	-2.99	0.021066	0.064296	-4.00
256452_at	hypothetical protein predicted by ge	AtHB33	AT1G75240	-2.32	-1.21	-2.99	0.021068	0.064296	-4.00
262593_at	putative ubiquinol--cytochrome-c re	---	AT1G15120	1.47	0.55	2.99	0.02107	0.064296	-4.00
253767_at	12S cruciferin seed storage protein	CRU3	AT4G28520	-1.27	-0.34	-2.99	0.021086	0.064334	-4.00
267030_at	unknown protein	SCAR2	AT2G38440	-1.24	-0.32	-2.99	0.021095	0.064353	-4.00
249720_at	fimbrin ; supported by cDNA: gi_15	FIM2	AT5G35700	1.25	0.33	2.99	0.021099	0.064359	-4.00
252833_at	Expressed protein ; supported by c	AGP3	AT4G40090	-1.24	-0.31	-2.99	0.021111	0.064385	-4.00
257689_at	myb-related protein, putative myb-r	AtMYB10	AT3G12820	-1.28	-0.35	-2.99	0.02112	0.0644	-4.00
255704_at	putative proline-rich protein ;suppo	---	AT4G00170	1.22	0.28	2.99	0.021121	0.0644	-4.00
250676_at	harpin-induced protein-like ; suppo	NHL3	AT5G06320	-1.51	-0.59	-2.99	0.021129	0.0644	-4.00
255487_at	tryptophan synthase alpha 1-like p	---	AT4G02610	1.30	0.38	2.99	0.02113	0.0644	-4.00
253260_at	hypothetical protein	---	AT4G34420	-1.28	-0.36	-2.99	0.02113	0.0644	-4.00
260271_at	unknown protein similar to unknown	---	AT1G63690	1.25	0.32	2.99	0.021134	0.064404	-4.00
252282_at	6-phosphogluconolactonase - like p	---	AT3G49360	1.23	0.30	2.99	0.021144	0.064428	-4.01
246084_at	putative protein predicted proteins,	ATBRXL4	AT5G20540	-1.29	-0.37	-2.99	0.021169	0.064492	-4.01
259027_at	hypothetical protein predicted by ge	---	AT3G09280	-1.21	-0.27	-2.99	0.021185	0.064534	-4.01

246760_at	putative protein predicted proteins,	---	AT5G27970	-1.24	-0.31	-2.99	0.021195	0.064548	-4.01
258936_s_a	hypothetical protein similar to hypo	---	---	-1.25	-0.32	-2.99	0.021196	0.064548	-4.01
245308_at	putative protein ; supported by cDN	---	AT4G17486	-1.23	-0.30	-2.99	0.021198	0.064548	-4.01
265184_at	unknown protein ;supported by full-	---	AT1G23710	2.55	1.35	2.99	0.021201	0.064548	-4.01
251986_at	putative protein various predicted p	---	AT3G53310	1.68	0.75	2.99	0.021222	0.064605	-4.01
245713_at	S-adenosyl-L-methionine:salicylic a	NAMT1	AT5G04370	-1.40	-0.49	-2.99	0.021228	0.064612	-4.01
266425_at	putative strictosidine synthase	---	AT2G41300	-1.29	-0.37	-2.99	0.021255	0.064685	-4.01
248521_s_a	nodulin-like protein ; supported by c	---	AT5G50520 ,	1.27	0.34	2.99	0.021284	0.064766	-4.01
258983_at	putative aminotransferase similar to	---	AT3G08860	2.14	1.10	2.99	0.02132	0.064866	-4.01
264392_at	hypothetical protein Similar to salt-i	---	AT1G11900	1.34	0.42	2.98	0.021356	0.064966	-4.02
253984_at	isp4 like protein isp4 protein -Schiz	OPT5	AT4G26590	-1.29	-0.37	-2.98	0.021358	0.064966	-4.02
265441_at	unknown protein ;supported by full-	---	AT2G20870	-1.37	-0.46	-2.98	0.021378	0.065018	-4.02
256767_at	hypothetical protein predicted by ge	---	AT3G13680	-1.26	-0.33	-2.98	0.021389	0.065038	-4.02
265096_at	unknown protein Location of ESTs	---	AT1G04030	1.31	0.39	2.98	0.021391	0.065038	-4.02
257080_at	unknown protein	---	AT3G15240	-1.20	-0.27	-2.98	0.021413	0.065099	-4.02
249342_at	putative protein ATP12 protein pre	---	AT5G40660	1.30	0.38	2.98	0.021417	0.065102	-4.02
258737_at	unknown protein similar to unknown	---	AT3G05940	-1.24	-0.32	-2.98	0.02143	0.065133	-4.02
246796_at	putative protein myosin heavy chai	---	AT5G26770	-1.38	-0.47	-2.98	0.021433	0.065133	-4.02
253756_at	Cdk-activating kinase CAK1At ; sup	CAK1AT	AT4G28980	-1.21	-0.28	-2.98	0.021444	0.065146	-4.02
251115_at	putative protein symplekin - Homo	ESP4	AT5G01400	1.27	0.35	2.98	0.021448	0.065146	-4.02
259604_at	20S proteasome beta subunit (PBG	PBG1	AT1G56450	1.25	0.33	2.98	0.021455	0.065146	-4.02
262952_at	hypothetical protein predicted by ge	---	AT1G75770	-1.34	-0.42	-2.98	0.021455	0.065146	-4.02
245504_at	glutaredoxin	---	AT4G15660	-1.49	-0.57	-2.98	0.021455	0.065146	-4.02
245346_at	putative beta-amylase ;supported b	CT-BMY	AT4G17090	-1.34	-0.42	-2.98	0.021456	0.065146	-4.02
265157_at	putative reverse transcriptase simila	---	---	-1.28	-0.35	-2.98	0.021462	0.065146	-4.02
262346_at	unknown protein similar to hypothet	---	AT1G63980	1.38	0.46	2.98	0.021462	0.065146	-4.02
264938_at	unknown protein contains similarity	---	AT1G60610	1.27	0.35	2.98	0.021463	0.065146	-4.02
264186_at	hypothetical protein predicted by ge	---	AT1G54570	1.86	0.90	2.98	0.021505	0.065257	-4.02
256543_at	unknown protein supported by full-l	---	AT1G42480	1.31	0.39	2.98	0.021506	0.065257	-4.02
261610_at	hypothetical protein predicted by ge	---	AT1G49560	1.49	0.57	2.98	0.021512	0.06527	-4.02
248884_at	putative protein similar to unknown	---	AT5G46030	1.22	0.29	2.98	0.021521	0.065286	-4.02
259909_at	unknown protein ; supported by full-	MEE9	AT1G60870	1.24	0.31	2.98	0.021527	0.065292	-4.02
260700_at	hypothetical protein identical to hyp	---	AT1G32260	1.31	0.39	2.98	0.021528	0.065292	-4.02
252583_at	putative protein several hypothetica	---	AT3G45540	-1.28	-0.35	-2.98	0.021546	0.065336	-4.03
244984_at	ribosomal protein S3	---	---	-1.43	-0.52	-2.98	0.02157	0.065402	-4.03

258110_at	putative cytochrome P450 similar to	CYP72A7	AT3G14610	1.35	0.43	2.98	0.021581	0.065425	-4.03
264440_at	unknown protein similar to fim prot	---	AT1G27340	1.24	0.31	2.98	0.021592	0.065451	-4.03
261365_at	proton pump interactor, putative sir	---	AT1G53110	1.88	0.91	2.98	0.021601	0.065469	-4.03
252590_at	putative protein kinase NPK1-relate	---	AT3G45670	-1.27	-0.35	-2.98	0.021611	0.06549	-4.03
256209_at	electron transport flavoprotein, puta	ETFALPHA	AT1G50940	1.27	0.34	2.98	0.021615	0.065491	-4.03
257705_at	unknown protein ; supported by cD	---	AT3G12760	1.19	0.26	2.98	0.021617	0.065491	-4.03
267599_at	putative serine/threonine protein kir	---	AT2G32850	-1.23	-0.30	-2.98	0.021622	0.065498	-4.03
249774_at	squalene monooxygenase	SQP1	AT5G24150	-1.53	-0.61	-2.97	0.021635	0.065529	-4.03
250366_at	putative protein predicted proteins	---	AT5G11420	-4.46	-2.16	-2.97	0.021638	0.065529	-4.03
255847_at	putative thioredoxin	ACHT3	AT2G33270	-1.21	-0.27	-2.97	0.021698	0.065701	-4.03
262933_at	polyamine oxidase, putative similar	ATPAO4	AT1G65840	-1.48	-0.57	-2.97	0.021707	0.065719	-4.03
246924_at	putative protein KIAA0332 - Homo	---	AT5G25060	1.21	0.28	2.97	0.021723	0.065759	-4.03
254231_at	putative protein AR411 - Arabidops	WRKY53	AT4G23810	3.75	1.91	2.97	0.021728	0.065759	-4.03
262711_at	hypothetical protein predicted by ge	---	AT1G16500	1.43	0.52	2.97	0.021733	0.065759	-4.03
250873_at	lipase -like protein lipase Arab-1, A	---	AT5G03980	-1.28	-0.36	-2.97	0.021734	0.065759	-4.03
257854_at	putative CREB-binding protein simil	HAC5	AT3G12980	-1.27	-0.34	-2.97	0.021739	0.065759	-4.03
246261_at	unknown protein	---	AT1G31810	1.29	0.36	2.97	0.02174	0.065759	-4.03
263254_x_a	hypothetical protein similar to hypot	---	---	-1.39	-0.48	-2.97	0.02174	0.065759	-4.03
248693_at	regulator of chromosome condensa	---	AT5G48330	1.42	0.51	2.97	0.021761	0.065815	-4.04
253076_at	NAM like protein P.hybrida NAM ge	ANAC076	AT4G36160	-1.26	-0.33	-2.97	0.021766	0.06582	-4.04
264446_at	putative nuclear transport factor sir	NTF2A	AT1G27310	1.28	0.36	2.97	0.02183	0.066004	-4.04
264221_s_a	unknown protein contains similarity	emb1220	AT1G60170 ,	1.28	0.36	2.97	0.021843	0.066035	-4.04
256905_at	mitochondrial chaperonin hsp60 ide	HSP60	AT3G23990	1.75	0.81	2.97	0.021874	0.066119	-4.04
264421_at	ribosomal protein identical to riboso	ARP1	AT1G43170	1.41	0.50	2.97	0.021876	0.066119	-4.04
253763_at	xyloglucan endotransglycosylase -	---	AT4G28850	-1.22	-0.29	-2.97	0.021899	0.066171	-4.04
250726_at	putative protein similar to unknown	---	AT5G06410	1.34	0.42	2.97	0.0219	0.066171	-4.04
257177_at	cyanate lyase (CYN) identical to cy	CYN	AT3G23490	1.42	0.50	2.97	0.021902	0.066171	-4.04
261595_at	hypothetical protein predicted by ge	---	AT1G33070	-1.21	-0.27	-2.97	0.021914	0.066196	-4.04
259342_at	unknown protein ;supported by full-	---	AT3G03890	1.39	0.48	2.97	0.021918	0.066196	-4.04
267569_at	putative photosystem II oxygen-evo	PSBP-2	AT2G30790	-1.52	-0.60	-2.97	0.021919	0.066196	-4.04
250105_at	putative protein contains similarity t	RAD4	AT5G16630	1.56	0.64	2.97	0.021936	0.066228	-4.04
258085_at	unknown protein ; supported by cD	---	AT3G26100	1.31	0.39	2.97	0.021936	0.066228	-4.04
261482_at	unknown protein ;supported by full-	THH1	AT1G14530	-1.28	-0.36	-2.96	0.021939	0.066228	-4.04
245874_at	unknown protein	---	AT1G26250	-1.29	-0.36	-2.96	0.021945	0.066235	-4.04
265922_at	putative sugar transporter	---	AT2G18480	-1.25	-0.32	-2.96	0.021947	0.066235	-4.04

260085_at	putative D-ribulose-5-phosphate sir	---	AT1G63290	1.24	0.31	2.96	0.021951	0.066239	-4.04
251788_at	putative protein	---	AT3G55420	-1.75	-0.80	-2.96	0.021957	0.066249	-4.05
266642_at	putative chloroplast RNA binding pr	---	AT2G35410	-1.28	-0.35	-2.96	0.021991	0.066343	-4.05
262715_at	putative myb-related transcription fa	MYB58	AT1G16490	-1.20	-0.26	-2.96	0.022018	0.066414	-4.05
265571_s_a	unknown protein	---	AT2G28020	1.24	0.31	2.96	0.022052	0.066508	-4.05
265707_at	unknown protein	---	AT2G03390	1.32	0.40	2.96	0.02206	0.066518	-4.05
259087_at	hypothetical protein, contains DnaJ	---	AT3G04980	1.21	0.27	2.96	0.022061	0.066518	-4.05
246846_at	putative protein	---	AT5G26760	1.23	0.30	2.96	0.022075	0.066542	-4.05
251199_at	transport inhibitor response 1 (TIR1	TIR1	AT3G62980	-1.50	-0.59	-2.96	0.022075	0.066542	-4.05
259140_at	lycopene beta cyclase identical to ly	LYC	AT3G10230	1.23	0.30	2.96	0.022081	0.066551	-4.05
258291_at	putative protein kinase similar to pro	---	AT3G23310	-1.30	-0.38	-2.96	0.022109	0.066628	-4.05
258133_at	ethylene-responsive transcriptional	MBF1C	AT3G24500	1.40	0.48	2.96	0.022112	0.066629	-4.05
266915_at	hypothetical protein predicted by ge	---	AT2G45870	-1.24	-0.31	-2.96	0.022124	0.066654	-4.05
264343_at	unknown protein ; supported by ful	---	AT1G11850	-1.46	-0.55	-2.96	0.022144	0.066707	-4.05
260107_at	fructokinase, putative similar to fruc	---	AT1G66430	-2.46	-1.30	-2.96	0.022155	0.066721	-4.05
254399_at	G10 - like protein Maternal G10 like	---	AT4G21110	1.22	0.28	2.96	0.022156	0.066721	-4.05
246072_at	PISTILLATA ; supported by full-len	PI	AT5G20240	1.23	0.30	2.96	0.022157	0.066721	-4.05
246159_at	Expressed protein ; supported by fu	---	AT5G20935	1.56	0.64	2.96	0.022169	0.066746	-4.06
250877_at	putative protein predicted protein, A	SDP1	AT5G04040	-1.40	-0.48	-2.96	0.022176	0.066758	-4.06
256777_at	hypothetical protein predicted by ge	---	AT3G13780	1.25	0.33	2.96	0.022183	0.066771	-4.06
259419_at	acyl-CoA oxidase ACX3, putative s	ACX6	AT1G06310	-1.25	-0.32	-2.96	0.022192	0.06679	-4.06
246010_at	putative protein predicted proteins,	---	AT5G08440	1.21	0.28	2.96	0.022209	0.066831	-4.06
257297_at	unknown protein contains Pfam pro	---	AT3G28040	-1.79	-0.84	-2.96	0.022212	0.066831	-4.06
258063_at	putative cytochrome P450 similar to	CYP72A8	AT3G14620	2.07	1.05	2.96	0.022218	0.066837	-4.06
260459_at	hypothetical protein predicted by ge	---	AT1G68240	-1.28	-0.35	-2.96	0.022219	0.066837	-4.06
253296_at	putative protein inositol 1,3,4-trisph	---	AT4G33770	-1.25	-0.32	-2.96	0.022237	0.06688	-4.06
263760_at	unknown protein ; supported by cD	SULA	AT2G21280	-1.48	-0.57	-2.96	0.022239	0.06688	-4.06
254182_at	putative protein various npredicted	---	AT4G23950	1.32	0.40	2.95	0.022262	0.066928	-4.06
257729_at	histone deacetylase, putative conta	HDA15	AT3G18520	1.41	0.49	2.95	0.022263	0.066928	-4.06
252141_at	putative protein	---	AT3G51100	1.22	0.28	2.95	0.022266	0.066928	-4.06
254482_at	TWIN SISTER OF FT (TSF) termin	TSF	AT4G20370	-1.32	-0.40	-2.95	0.022267	0.066928	-4.06
256928_at	unknown protein	---	AT3G22590	-1.28	-0.35	-2.95	0.022275	0.066939	-4.06
264240_at	protein kinase, putative Simisimilar	---	AT1G54820	-2.60	-1.38	-2.95	0.022277	0.066939	-4.06
255833_at	unknown protein	---	AT2G33390	1.30	0.38	2.95	0.022282	0.066946	-4.06
251083_at	hypothetical protein ; supported by	---	AT5G01590	-1.27	-0.35	-2.95	0.022309	0.067018	-4.06

251707_at	protein kinase C inhibitor-like prote	---	AT3G56490	1.29	0.36	2.95	0.022317	0.067031	-4.06
262761_at	unknown protein ESTs gb|AA3957	---	AT1G10720	-1.40	-0.48	-2.95	0.022319	0.067031	-4.06
264071_at	putative carboxypeptidase ; suppor	SCPL51	AT2G27920	-1.53	-0.61	-2.95	0.022355	0.067128	-4.06
263665_at	putative putative 60S ribosomal pro	---	AT1G04480	1.54	0.62	2.95	0.022357	0.067128	-4.06
254810_at	putative protein pectinesterase - C	PME1	AT4G12390	1.73	0.79	2.95	0.022375	0.067168	-4.06
257461_at	hypothetical protein predicted by ge	ATPUP15	AT1G75470	-1.21	-0.27	-2.95	0.022377	0.067168	-4.07
255366_x_a	putative transposon protein	---	---	-1.27	-0.34	-2.95	0.022407	0.067252	-4.07
257841_at	nitrate transporter, putative similar	---	AT3G25260	-1.24	-0.31	-2.95	0.022429	0.067308	-4.07
267210_at	dihydroxypolyprenylbenzoate methy	ATCOQ3	AT2G30920	-1.20	-0.26	-2.95	0.022452	0.067368	-4.07
261731_s_a	hypothetical protein similar to hypot	---	AT1G47780	-1.21	-0.28	-2.95	0.022458	0.067378	-4.07
260813_at	VirE2-interacting protein VIP1 ident	VIP1	AT1G43700	1.40	0.48	2.95	0.022468	0.06739	-4.07
262467_at	hypothetical protein similar to hypot	FU	AT1G50240	-1.32	-0.40	-2.95	0.022468	0.06739	-4.07
262512_at	Expressed protein ; supported by fu	---	AT1G17145	1.69	0.76	2.95	0.022478	0.067412	-4.07
267603_at	unknown protein ; supported by cD	ATZW10	AT2G32900	-1.33	-0.41	-2.95	0.022485	0.067415	-4.07
247409_at	dihydroneopterin aldolase-like prote	---	AT5G62980	1.45	0.53	2.95	0.022492	0.067415	-4.07
262158_at	somatic embryogenesis receptor-li	---	AT1G52540	1.44	0.53	2.95	0.022493	0.067415	-4.07
251412_at	RING-H2 zinc finger protein ATL4	ATL4	AT3G60220	-1.72	-0.78	-2.95	0.022494	0.067415	-4.07
265369_s_a	putative retroelement pol polyprotei	---	---	-1.24	-0.31	-2.95	0.022494	0.067415	-4.07
249914_at	protease-like protein	---	AT5G22850	1.36	0.44	2.95	0.0225	0.067417	-4.07
258896_at	putative syntaxin protein, AtSNAP3	SYP43	AT3G05710	-1.29	-0.37	-2.95	0.022501	0.067417	-4.07
250123_at	putative protein contains similarity t	PIN5	AT5G16530	1.32	0.40	2.95	0.022519	0.067462	-4.07
263941_at	unknown protein	---	---	-1.33	-0.41	-2.95	0.022526	0.067474	-4.07
258049_at	hypothetical protein similar to CGI-1	---	AT3G16220	1.22	0.29	2.94	0.022558	0.067546	-4.07
263840_at	Expressed protein ; supported by fu	---	AT2G36885	-1.41	-0.50	-2.94	0.022558	0.067546	-4.07
247542_at	autophagocytosis protein - like aut	ATG3	AT5G61500	1.32	0.40	2.94	0.022559	0.067546	-4.07
263385_at	ABA-regulated gene (ATEM6) ; su	GEA6	AT2G40170	-1.33	-0.41	-2.94	0.022571	0.067575	-4.07
247383_at	putative protein contains similarity t	---	AT5G63410	1.56	0.64	2.94	0.022576	0.067576	-4.07
253431_at	putative protein predicted protein,	---	AT4G32440	1.28	0.36	2.94	0.022578	0.067576	-4.07
264613_at	lipoyltransferase identical to GB:BA	LIP2	AT1G04640	-1.28	-0.36	-2.94	0.022596	0.067613	-4.08
260351_at	hypothetical protein predicated by g	---	AT1G69380	1.64	0.71	2.94	0.022596	0.067613	-4.08
247423_at	putative protein predicted proteins,	---	AT5G62830	-1.22	-0.29	-2.94	0.022606	0.067634	-4.08
249606_at	putative protein DNA-binding prote	RVE2	AT5G37260	1.28	0.35	2.94	0.022611	0.06764	-4.08
248684_at	Expressed protein ; supported by fu	DIR1	AT5G48485	1.99	0.99	2.94	0.022635	0.067702	-4.08
247073_at	33 kDa polypeptide of oxygen-evolv	PSBO1	AT5G66570	-1.22	-0.29	-2.94	0.022654	0.067751	-4.08
255093_s_a	putative microfibril-associated prote	---	AT4G08580	1.22	0.29	2.94	0.022663	0.067765	-4.08

246047_at	putative protein predicted proteins,	---	---	-1.27	-0.34	-2.94	0.022664	0.067765	-4.08
258796_at	unknown protein ;supported by full-	WDL1	AT3G04630	-1.69	-0.76	-2.94	0.022675	0.067786	-4.08
248514_s_a	unknown protein	---	AT5G50510	-1.23	-0.30	-2.94	0.022684	0.067804	-4.08
247864_s_a	anthranilate synthase beta chain ;s	---	AT1G24807	2.38	1.25	2.94	0.022689	0.06781	-4.08
250172_at	putative protein	---	AT5G14330	-1.38	-0.46	-2.94	0.022692	0.06781	-4.08
249453_at	v-SNARE AtVTI1a	SGR4	AT5G39510	1.21	0.28	2.94	0.022713	0.067865	-4.08
261249_at	hypothetical protein predicted by ge	---	AT1G05880	-1.27	-0.35	-2.94	0.022731	0.06791	-4.08
257824_at	unknown protein ; supported by cD	---	AT3G25290	1.58	0.66	2.94	0.022734	0.06791	-4.08
258389_at	hypothetical protein predicted by ge	SDE5	AT3G15390	1.34	0.42	2.94	0.02274	0.067919	-4.08
249150_at	inorganic phosphate transporter (db	PHT6	AT5G43340	-1.23	-0.30	-2.94	0.022749	0.067938	-4.08
256043_at	hypothetical protein contains Pfam	---	AT1G07210	1.42	0.50	2.94	0.022773	0.068	-4.08
255185_at	hypothetical protein	---	AT4G07740	-1.27	-0.34	-2.94	0.022796	0.068061	-4.08
260596_at	unknown protein	TIM50	AT1G55900	1.49	0.58	2.94	0.022801	0.068066	-4.08
264069_at	putative rubisco subunit binding-pro	CPN60A	AT2G28000	1.45	0.53	2.94	0.022804	0.068066	-4.08
253704_at	X-Pro dipeptidase - like protein (fra	---	AT4G29490	1.28	0.35	2.94	0.022807	0.068066	-4.09
258691_at	unknown protein	---	AT3G08630	-1.88	-0.91	-2.94	0.022829	0.068123	-4.09
261068_at	tropinone reductase-I, putative sim	---	AT1G07450	-1.27	-0.34	-2.94	0.022869	0.068232	-4.09
264011_at	unknown protein	---	AT2G21090	1.29	0.36	2.93	0.022885	0.068269	-4.09
265694_at	hypothetical protein predicted by ge	---	AT2G24440	1.30	0.37	2.93	0.022887	0.068269	-4.09
254646_at	putative protein	---	AT4G18530	1.36	0.45	2.93	0.022902	0.068306	-4.09
260081_at	unknown protein	---	AT1G78170	-2.94	-1.56	-2.93	0.022907	0.06831	-4.09
255252_at	contains similarity to Gossypium hir	---	AT4G04990	-1.25	-0.32	-2.93	0.022912	0.068317	-4.09
267128_at	putative acetone-cyanohydrin lyase	MES1	AT2G23620	-1.33	-0.41	-2.93	0.022925	0.068348	-4.09
246889_at	putative protein various predicted p	---	AT5G25470	-1.32	-0.41	-2.93	0.02296	0.068439	-4.09
265861_at	hypothetical protein predicted by ge	---	AT2G01790	-1.27	-0.34	-2.93	0.022962	0.068439	-4.09
264700_at	hypothetical protein predicted by ge	---	AT1G70100	-1.33	-0.41	-2.93	0.022973	0.068464	-4.09
249898_at	alkaline/neutral invertase ; support	---	AT5G22510	1.40	0.48	2.93	0.022995	0.068516	-4.09
245679_at	clathrin-associated protein, putative	---	AT1G56590	-1.28	-0.36	-2.93	0.022997	0.068516	-4.09
262782_at	Expressed protein ; supported by fu	---	AT1G13195	1.24	0.31	2.93	0.023007	0.068538	-4.09
246971_at	Protein phosphatase 2C-like protei	---	AT5G24940	-1.24	-0.31	-2.93	0.023019	0.068563	-4.09
257965_at	unknown protein ;supported by full-	---	AT3G19900	1.44	0.52	2.93	0.023032	0.068595	-4.10
253572_at	hypothetical protein	---	AT4G31010	1.28	0.36	2.93	0.023058	0.068663	-4.10
265871_at	unknown protein ;supported by full-	---	AT2G01680	1.37	0.46	2.93	0.023064	0.068672	-4.10
250616_at	receptor-like protein kinase-like pro	EMS1	AT5G07280	-1.31	-0.39	-2.93	0.023078	0.068705	-4.10
247432_at	microtubule-associated protein EB1	ATEB1B	AT5G62500	-1.29	-0.36	-2.93	0.023088	0.068724	-4.10

246484_at	UVB-resistance protein-like UVB-re	---	AT5G16040	1.29	0.37	2.93	0.023111	0.068776	-4.10
247372_at	unknown protein ;supported by full-	---	AT5G63280	1.43	0.52	2.93	0.023111	0.068776	-4.10
258034_at	hypothetical protein predicted by ge	---	AT3G21300	1.76	0.81	2.93	0.023141	0.068845	-4.10
261262_at	hypothetical protein predicted by ge	SDG35	AT1G26760	-1.22	-0.29	-2.93	0.023141	0.068845	-4.10
246459_at	receptor protein kinase -like protein	---	AT5G16900	-1.25	-0.32	-2.93	0.023144	0.068845	-4.10
256856_at	Expressed protein ; supported by fu	---	AT3G15110	-1.41	-0.50	-2.93	0.023162	0.068892	-4.10
261438_at	metallothionein-like protein identica	---	AT1G07590	1.23	0.29	2.92	0.023211	0.069019	-4.10
256350_at	hypothetical protein contains similar	PGSIP4	AT1G54940	-1.22	-0.29	-2.92	0.023211	0.069019	-4.10
264967_at	unknown protein ;supported by full-	---	AT1G67250	1.22	0.29	2.92	0.023228	0.06906	-4.10
250806_at	putative protein similar to unknown	---	AT5G05070	-1.24	-0.31	-2.92	0.023243	0.069095	-4.10
261971_at	type 2 peroxiredoxin, putative simil	---	AT1G65990	-1.32	-0.40	-2.92	0.02326	0.069135	-4.11
252239_at	putative protein putative protein - D	---	AT3G49990	1.48	0.57	2.92	0.023262	0.069135	-4.11
266744_at	putative nucleoside triphosphatase	---	AT2G02970	-1.21	-0.27	-2.92	0.023284	0.069186	-4.11
252879_at	putative protein predicted proteins,	NST-K1	AT4G39390	-1.82	-0.87	-2.92	0.023286	0.069186	-4.11
256179_at	ubiquitin-specific protease 6 (UBP6	UBP6	AT1G51710	1.22	0.29	2.92	0.023294	0.069204	-4.11
266917_at	unknown protein	DTA2	AT2G45830	1.24	0.31	2.92	0.023307	0.069232	-4.11
251773_at	putative protein HSPC058, Homo s	---	AT3G55960	1.47	0.55	2.92	0.023318	0.069257	-4.11
250187_at	putative protein predicted proteins,	---	AT5G14370	-1.52	-0.60	-2.92	0.023326	0.06927	-4.11
265677_at	putative purine-rich single-stranded	PUR ALPH	AT2G32080	-1.23	-0.30	-2.92	0.023334	0.069286	-4.11
246162_at	actin interacting protein ; supported	---	AT4G36400	1.35	0.43	2.92	0.023339	0.069291	-4.11
264202_at	TINY-like transcription factor Conta	---	AT1G22810	-2.08	-1.06	-2.92	0.023357	0.069337	-4.11
255644_at	coded for by A. thaliana cDNA T438	---	AT4G00870	1.24	0.31	2.92	0.023361	0.069337	-4.11
266026_at	putative non-LTR retroelement reve	---	---	-1.29	-0.36	-2.92	0.023389	0.069388	-4.11
261052_at	hypothetical protein similar to hypot	---	AT1G01440	1.34	0.42	2.92	0.023391	0.069388	-4.11
266883_at	putative triacylglycerol lipase	DAD1	AT2G44810	-1.25	-0.32	-2.92	0.023393	0.069388	-4.11
255564_s_a	hypothetical protein T15B16.8	RGXT2	AT4G01750	1.29	0.36	2.92	0.023393	0.069388	-4.11
261920_at	isocitrate dehydrogenase, putative	---	AT1G65930	1.28	0.36	2.92	0.023395	0.069388	-4.11
259398_at	hypothetical protein predicted by ge	PRA1.F1	AT1G17700	-2.00	-1.00	-2.92	0.023396	0.069388	-4.11
258403_at	hypothetical protein predicted by ge	---	AT3G15380	1.25	0.33	2.92	0.023402	0.069397	-4.11
265276_at	hypothetical protein predicted by ge	---	AT2G28400	-1.45	-0.54	-2.92	0.023407	0.069398	-4.11
266853_at	putative salt-inducible protein	---	AT2G26790	1.23	0.30	2.92	0.023408	0.069398	-4.11
261433_s_a	endoplasmic reticulum-type calcium	---	AT1G07670	1.21	0.27	2.92	0.023424	0.069434	-4.11
260892_at	cysteine proteinase contains similar	---	AT1G29110	-1.29	-0.37	-2.92	0.023442	0.06948	-4.11
252210_at	DNA binding protein	OBP1	AT3G50410	-1.44	-0.53	-2.92	0.023456	0.069506	-4.11
247997_at	putative protein contains similarity t	ATARP8	AT5G56180	1.31	0.39	2.92	0.023457	0.069506	-4.11

261932_at	hypothetical protein predicted by ge	---	AT1G22420	-1.21	-0.28	-2.92	0.023462	0.069511	-4.11
262766_at	hypothetical protein predicted by ge	---	AT1G13160	1.52	0.60	2.92	0.023476	0.069544	-4.12
257345_s_a	pseudogene	---	---	-1.21	-0.28	-2.92	0.023489	0.069574	-4.12
266211_at	putative retroelement pol polyprotei	---	---	-1.26	-0.33	-2.92	0.023493	0.069577	-4.12
252163_at	hypothetical protein	---	AT3G50610	-1.27	-0.35	-2.92	0.023516	0.069635	-4.12
247703_at	putative protein predicted proteins,	SRR1	AT5G59560	1.29	0.37	2.91	0.023524	0.069639	-4.12
247307_at	UVB-resistance protein UVR8 (gb|A	---	AT5G21326	1.24	0.31	2.91	0.023528	0.069639	-4.12
254645_at	putative protein	---	AT4G18520	1.29	0.37	2.91	0.023529	0.069639	-4.12
267401_at	unknown protein	---	AT2G26210	1.26	0.33	2.91	0.023531	0.069639	-4.12
251627_at	glucan endo-1,3-beta-D-glucosidas	BG1	AT3G57270	-1.28	-0.35	-2.91	0.023532	0.069639	-4.12
266823_at	hypothetical protein predicted by ge	---	AT2G44930	-1.26	-0.34	-2.91	0.023543	0.06966	-4.12
245611_at	hypothetical protein	---	AT4G14390	-1.41	-0.50	-2.91	0.023546	0.069662	-4.12
254557_at	hypothetical protein ;supported by	---	AT4G19140	-1.58	-0.66	-2.91	0.023549	0.069662	-4.12
266538_at	hypothetical protein predicted by ge	---	AT2G35230	-1.26	-0.33	-2.91	0.023553	0.069663	-4.12
251229_at	beta-glucosidase-like protein severa	BGLU7	AT3G62740	-1.22	-0.29	-2.91	0.02356	0.069674	-4.12
246676_at	putative protein	---	---	-1.21	-0.27	-2.91	0.023575	0.069711	-4.12
266655_at	putative protein kinase contains a p	AtAUR2	AT2G25880	-1.35	-0.44	-2.91	0.023587	0.069737	-4.12
265109_s_a	NBS/LRR disease resistance prote	---	AT1G62630	1.47	0.56	2.91	0.023594	0.069749	-4.12
263369_at	unknown protein	---	AT2G20480	1.22	0.28	2.91	0.023607	0.069779	-4.12
257494_at	hypothetical protein predicted by ge	---	AT1G32375	-1.21	-0.27	-2.91	0.023629	0.069824	-4.12
267489_s_a	putative DNA2-NAM7 helicase fami	---	AT2G19120	1.43	0.51	2.91	0.023629	0.069824	-4.12
265963_s_a	40S ribosomal protein S5 identical t	ATRPS5B	AT2G37270	1.59	0.67	2.91	0.023636	0.069837	-4.12
264509_at	unknown protein Location of EST g	---	AT1G09520	1.28	0.36	2.91	0.023639	0.069837	-4.12
255699_at	putative pectinesterase	PME38	AT4G00190	-1.22	-0.29	-2.91	0.023646	0.069847	-4.12
245181_at	putative protein similarity to various	---	AT5G12420	1.89	0.92	2.91	0.023655	0.069865	-4.12
252954_at	putative thaumatin-like protein thau	---	AT4G38660	-2.28	-1.19	-2.91	0.023707	0.070011	-4.13
249111_at	unknown protein	---	AT5G43770	-1.21	-0.27	-2.91	0.023712	0.070015	-4.13
264298_at	hypothetical protein contains similar	---	AT1G78690	-1.27	-0.34	-2.91	0.023715	0.070015	-4.13
263296_at	unknown protein	---	AT2G38800	1.63	0.70	2.91	0.023735	0.070058	-4.13
266086_at	putative Na+-dependent inorganic p	PHT4;2	AT2G38060	-1.28	-0.35	-2.91	0.023736	0.070058	-4.13
254940_at	putative protein predicted proteins,	---	AT4G10820	-1.24	-0.32	-2.91	0.02376	0.070119	-4.13
248101_at	chaperone GrpE-like protein ;supp	---	AT5G55200	1.74	0.80	2.91	0.023762	0.070119	-4.13
245551_at	cytochrome P450 like protein	CYP705A2	AT4G15350	-1.22	-0.28	-2.91	0.023782	0.070166	-4.13
262203_at	transfactor-like protein similar to tra	---	AT2G01060	1.28	0.36	2.91	0.023784	0.070166	-4.13
265130_at	unknown protein similar to ESTs gb	---	AT1G30890	-1.19	-0.26	-2.91	0.023796	0.070192	-4.13

257406_at	hypothetical protein predicted by g	---	AT1G27060	1.33	0.41	2.91	0.023804	0.070205	-4.13
245873_at	bHLH transcription factor GBOF-1,	CIB5	AT1G26260	-1.34	-0.43	-2.91	0.023817	0.070235	-4.13
255837_at	unknown protein	RIC1	AT2G33460	-1.26	-0.33	-2.91	0.023829	0.07026	-4.13
265807_at	unknown protein	---	AT2G17990	1.27	0.35	2.90	0.023857	0.070335	-4.13
263422_s_a	putative ubiquitin-like protein ; supp	---	AT2G17190	1.22	0.29	2.90	0.023864	0.070347	-4.13
258989_at	rhodanese-like family protein contai	---	AT3G08920	-1.30	-0.38	-2.90	0.023881	0.070388	-4.13
256706_at	auxin-independent growth promote	---	AT3G30300	1.28	0.36	2.90	0.023898	0.070427	-4.13
263048_s_a	unknown protein ;supported by full-	---	AT2G05310	-1.31	-0.39	-2.90	0.023908	0.07045	-4.13
249859_at	serine protein kinase-like protein ;s	---	AT5G22840	1.54	0.62	2.90	0.023922	0.070479	-4.14
257950_at	putative UDP-glucose glucosyltrans	UGT71B6	AT3G21780	2.14	1.10	2.90	0.02395	0.070542	-4.14
261007_at	hypothetical protein similar to reticu	---	AT1G26400	1.19	0.25	2.90	0.023952	0.070542	-4.14
247216_at	4-alpha-glucanotransferase ; supp	DPE1	AT5G64860	1.37	0.46	2.90	0.023955	0.070542	-4.14
254129_at	putative tRNA isopentenyltransfera	IPT4	AT4G24650	-1.23	-0.30	-2.90	0.023955	0.070542	-4.14
259053_at	unknown protein similar to hypothet	---	AT3G03320	1.28	0.36	2.90	0.02399	0.070633	-4.14
267520_at	putative integral membrane protein	---	AT2G30460	-1.48	-0.56	-2.90	0.023992	0.070633	-4.14
261825_at	endo-xyloglucan transferase, putat	---	AT1G11545	-3.83	-1.94	-2.90	0.02401	0.070669	-4.14
250650_at	anthranilate phosphoribosyltransfer	---	AT5G06850	1.45	0.53	2.90	0.024011	0.070669	-4.14
258622_at	unknown protein contains Pfam pro	---	AT3G02720	-1.25	-0.32	-2.90	0.024024	0.070693	-4.14
255766_at	unknown protein ;supported by full-	---	AT1G16750	-1.35	-0.43	-2.90	0.024025	0.070693	-4.14
258466_at	hypothetical protein contains simila	---	AT3G06040	1.31	0.39	2.90	0.024044	0.070741	-4.14
261450_s_a	O-methyltransferase, putative simil	---	AT1G21110	5.53	2.47	2.90	0.02405	0.070747	-4.14
245602_at	Expressed protein ;supported by cD	---	AT4G14270	1.42	0.51	2.90	0.024057	0.070756	-4.14
262089_s_a	hypothetical protein predicted by ge	---	AT1G55980	-1.38	-0.46	-2.90	0.024059	0.070756	-4.14
261232_at	unknown protein ; supported by cDI	---	AT1G20220	-1.25	-0.32	-2.90	0.024069	0.070778	-4.14
249633_at	sucrose synthase-like protein sucro	SUS5	AT5G37180	-1.23	-0.30	-2.90	0.02409	0.070829	-4.14
260078_at	hypothetical protein predicted by ge	---	AT1G73790	1.29	0.36	2.90	0.024094	0.070833	-4.14
258798_at	hypothetical protein predicted by ge	---	AT3G04540	-1.28	-0.36	-2.90	0.024108	0.070864	-4.14
257886_at	pectinesterase, putative similar to p	---	AT3G17060	-1.26	-0.34	-2.90	0.024111	0.070865	-4.14
250497_at	putative protein predicted proteins,	---	AT5G09630	1.34	0.43	2.90	0.024121	0.070885	-4.14
267204_at	putative blue copper-binding protein	---	AT2G31050	-1.27	-0.35	-2.90	0.024141	0.070934	-4.14
249404_at	ankyrin repeat protein EMB506 ; su	EMB506	AT5G40160	1.36	0.45	2.90	0.024153	0.070959	-4.15
255880_at	hypothetical protein predicted by ge	---	AT1G67060	-1.24	-0.31	-2.90	0.024162	0.070976	-4.15
252322_at	putative protein putative zinc-finger	---	AT3G48550	-1.65	-0.72	-2.89	0.024191	0.071053	-4.15
257879_at	unknown protein	---	AT3G17160	1.47	0.56	2.89	0.024241	0.071192	-4.15
266238_at	phosphoprotein phosphatase, type	TOPP1	AT2G29400	1.45	0.53	2.89	0.024248	0.071201	-4.15

254854_at	putative protein hypothetical protei	---	AT4G12130	1.38	0.46	2.89	0.024265	0.071242	-4.15
AFFX-BioC-	E. coli /GEN=bioC /DB_XREF=gb:J	---	---	-1.54	-0.62	-2.89	0.024287	0.07128	-4.15
265761_at	putative stress protein	---	AT2G01330	1.25	0.32	2.89	0.024288	0.07128	-4.15
251623_at	MADS transcription factor-like prot	AGL18	AT3G57390	1.21	0.28	2.89	0.02429	0.07128	-4.15
250952_at	putative protein	ATIREG2	AT5G03570	-1.35	-0.43	-2.89	0.024293	0.07128	-4.15
246167_at	putative protein	---	---	-1.32	-0.40	-2.89	0.024293	0.07128	-4.15
245499_at	membrane transporter like protein	INT4	AT4G16480	-1.23	-0.30	-2.89	0.024311	0.071315	-4.15
259996_at	hypothetical protein predicted by ge	---	AT1G67910	-1.36	-0.44	-2.89	0.024311	0.071315	-4.15
261873_at	serine/threonine kinase, putative si	SD1-13	AT1G11350	-1.48	-0.57	-2.89	0.024315	0.071317	-4.15
251721_s_a	alpha-soluble NSF attachment prote	ALPHA-SN	AT3G56190	1.30	0.37	2.89	0.024327	0.071342	-4.15
255637_at	hypothetical protein	---	AT4G00750	1.41	0.49	2.89	0.024332	0.071348	-4.15
255346_at	hypothetical protein	---	---	-1.19	-0.25	-2.89	0.024338	0.071356	-4.15
248186_at	unknown protein ;supported by full-	---	AT5G53880	-2.51	-1.32	-2.89	0.024353	0.07139	-4.15
259696_at	unknown protein ; supported by cDI	---	AT1G63150	1.26	0.34	2.89	0.024404	0.07151	-4.16
263906_at	plastid division protein (FtsZ) identi	FTSZ2-1	AT2G36250	1.30	0.38	2.89	0.024405	0.07151	-4.16
262033_at	terminal ear1, putative similar to te	MCT1	AT1G37140	-1.27	-0.35	-2.89	0.024406	0.07151	-4.16
266332_at	hypothetical protein predicted by ge	---	AT2G32415	-1.27	-0.34	-2.89	0.024406	0.07151	-4.16
247875_at	putative protein similar to unknown	---	AT5G57720	-1.28	-0.35	-2.89	0.024409	0.07151	-4.16
257818_at	hypothetical protein predicted by ge	---	AT3G25120	1.30	0.37	2.89	0.024418	0.071526	-4.16
256744_at	ATHP2, putative similar to ATHP2	AHP2	AT3G29350	1.32	0.40	2.89	0.024427	0.071542	-4.16
250805_at	unknown protein	---	AT5G05050	-1.23	-0.30	-2.89	0.02443	0.071542	-4.16
266901_at	hypothetical protein predicted by ge	JAZ7	AT2G34600	-2.01	-1.01	-2.89	0.024463	0.071629	-4.16
266620_at	unknown protein	---	AT2G35450	-1.32	-0.40	-2.89	0.024509	0.071756	-4.16
251797_at	putative protein AT-hook protein 1	AGF2	AT3G55560	1.36	0.45	2.88	0.024522	0.071776	-4.16
250455_at	unknown protein ; supported by ful	PROPEP4	AT5G09980	-1.39	-0.48	-2.88	0.024522	0.071776	-4.16
255916_at	putative protein	---	AT5G28550	-1.31	-0.39	-2.88	0.024528	0.071784	-4.16
262939_s_a	glyceraldehyde-3-phosphate dehyd	GAPCP-2	AT1G16300	2.57	1.36	2.88	0.024533	0.071785	-4.16
259396_at	shaggy-like kinase, putative similar	GSK1	AT1G06390	-1.22	-0.29	-2.88	0.024538	0.071785	-4.16
259366_at	allene oxide cyclase, putative simil	AOC4	AT1G13280	-1.41	-0.49	-2.88	0.024538	0.071785	-4.16
262072_at	hypothetical protein predicted by ge	ZCF37	AT1G59590	1.80	0.85	2.88	0.024551	0.071813	-4.16
245531_at	hydroxynitrile lyase like protein	scpl30	AT4G15100	-1.39	-0.48	-2.88	0.024572	0.071867	-4.16
245909_at	putative protein DNA-directed RNA	---	AT5G09380	1.33	0.41	2.88	0.024577	0.071873	-4.16
263112_at	unknown protein Strong similarity to	---	AT1G03080	1.29	0.37	2.88	0.024596	0.071918	-4.16
250191_at	kinase - like protein RING3 protein	ATBET9	AT5G14270	1.28	0.36	2.88	0.024599	0.071918	-4.16
254371_at	beta-glucosidase -like protein beta-	BGLU47	AT4G21760	-1.23	-0.30	-2.88	0.024606	0.071929	-4.16

265338_at	putative ribosomal protein L6 ; sup	---	AT2G18400	1.26	0.33	2.88	0.02461	0.071932	-4.16
257677_at	unknown protein contains Pfam pro	ATEHD1	AT3G20290	1.29	0.36	2.88	0.024638	0.072003	-4.17
247620_at	receptor like protein kinase recepto	---	AT5G60310	-1.28	-0.35	-2.88	0.024658	0.072053	-4.17
258690_at	putative phosphatidylinositol-4-phos	---	AT3G07960	-1.24	-0.31	-2.88	0.024675	0.072093	-4.17
265845_at	unknown protein	---	AT2G35610	1.19	0.24	2.88	0.024679	0.072097	-4.17
250136_at	putative protein de novo DNA meth	DRM1	AT5G15380	-1.52	-0.61	-2.88	0.024713	0.072187	-4.17
253696_at	cytokinin oxidase - like protein cyto	CKX4	AT4G29740	-1.78	-0.83	-2.88	0.024725	0.072213	-4.17
246891_at	putative protein various predicted p	---	AT5G25490	-1.21	-0.27	-2.88	0.024735	0.072234	-4.17
254167_at	serine/threonine kinase-like protein	CIPK8	AT4G24400	1.29	0.36	2.88	0.024753	0.072276	-4.17
265275_at	En/Spm-like transposon protein rela	---	AT2G28440	-1.50	-0.59	-2.88	0.024777	0.072336	-4.17
247310_at	putative protein contains similarity t	CHR24	AT5G63950	1.38	0.46	2.88	0.024789	0.072363	-4.17
261039_at	Expressed protein ; supported by fu	ELF4-L4	AT1G17455	-1.42	-0.51	-2.88	0.024798	0.072379	-4.17
253403_at	putative serine/threonine protein ki	AtAUR1	AT4G32830	-1.31	-0.39	-2.88	0.024803	0.072384	-4.17
247944_at	putative protein similar to unknown	---	AT5G57100	-1.31	-0.39	-2.88	0.024808	0.072389	-4.17
253607_at	small nuclear ribonucleoprotein ho	---	AT4G30330	1.37	0.45	2.88	0.024811	0.072389	-4.17
257532_at	hypothetical protein similar to hypot	---	AT3G04700	2.35	1.23	2.88	0.024819	0.072404	-4.17
257887_at	protein phosphatase-2c, putative s	---	AT3G17090	-1.32	-0.40	-2.88	0.024831	0.072429	-4.17
252967_at	amidophosphoribosyltransferase -	ATASE3	AT4G38880	-1.26	-0.34	-2.88	0.024854	0.072487	-4.18
253868_at	proton pump interactor non-consen	PPI1	AT4G27500	-1.29	-0.37	-2.88	0.024861	0.072497	-4.18
253475_at	putative protein predicted protein, A	---	AT4G32290	-1.56	-0.64	-2.87	0.024878	0.072532	-4.18
254431_at	reticuline oxidase - like protein retic	---	AT4G20840	1.47	0.55	2.87	0.024879	0.072532	-4.18
252975_s_a	putative protein other predicted prot	ROPGEF1	AT4G38430	-1.24	-0.31	-2.87	0.024888	0.07254	-4.18
255888_at	hypothetical protein contains similar	---	AT1G20300	1.87	0.91	2.87	0.024889	0.07254	-4.18
260971_at	hypothetical protein predicted by ge	---	AT1G53620	-1.24	-0.31	-2.87	0.024891	0.07254	-4.18
251149_at	putative protein CGI-77 protein, Ho	---	AT3G62940	1.19	0.26	2.87	0.024923	0.072612	-4.18
245897_at	potassium transport protein-like sev	KUP7	AT5G09400	-1.37	-0.46	-2.87	0.024924	0.072612	-4.18
267365_at	putative phosphoribosyl pyrophosph	---	AT2G44530	1.20	0.26	2.87	0.024926	0.072612	-4.18
252162_at	hypothetical protein	---	AT3G50590	-1.47	-0.55	-2.87	0.024937	0.072627	-4.18
246499_at	putative protein Tat-SF1 - Homo sa	---	AT5G16260	1.34	0.42	2.87	0.02494	0.072627	-4.18
250973_at	60S ribosomal protein - like 60S ro	---	AT5G02870	1.53	0.61	2.87	0.02494	0.072627	-4.18
248352_at	low-temperature-induced 65 kD pro	LTI65	AT5G52300	-2.21	-1.15	-2.87	0.024946	0.072633	-4.18
252887_at	putative protein various predicted p	---	AT4G39240	1.20	0.26	2.87	0.024949	0.072633	-4.18
256075_at	mitogen-activated protein kinase, p	ATMPK8	AT1G18150	1.32	0.40	2.87	0.024981	0.072719	-4.18
258200_at	putative ATPase similar to ATPase	---	AT3G13900	-1.32	-0.40	-2.87	0.025018	0.072804	-4.18
258606_at	unknown protein ;supported by full-	---	AT3G02840	4.84	2.27	2.87	0.025019	0.072804	-4.18

250441_at	oligopeptidase A - like protein oligo	---	AT5G10540	1.21	0.27	2.87	0.02502	0.072804	-4.18
249682_at	putative protein similar to unknown	---	AT5G36080	-1.22	-0.28	-2.87	0.025039	0.072849	-4.18
251470_at	putative protein hypothetical protei	---	AT3G59570	-1.33	-0.41	-2.87	0.02506	0.0729	-4.18
256577_at	unknown protein	---	AT3G28220	-2.39	-1.26	-2.87	0.025063	0.0729	-4.18
261132_at	hypothetical protein predicted by ge	TGD1	AT1G19800	-1.24	-0.31	-2.87	0.025085	0.072955	-4.19
256581_at	hypothetical protein	---	AT3G28830	-1.20	-0.26	-2.87	0.025094	0.072972	-4.19
246319_at	putative protein HYPOTHETICAL F	---	AT3G56680	-1.24	-0.31	-2.87	0.025105	0.072996	-4.19
260070_at	putative helix-loop-helix DNA-bindir	BEE3	AT1G73830	-2.81	-1.49	-2.87	0.025123	0.073026	-4.19
255208_at	see GB:AL022580	---	---	-1.25	-0.33	-2.87	0.025125	0.073026	-4.19
258391_at	hypothetical protein predicted by ge	---	AT3G15420	1.43	0.51	2.87	0.025126	0.073026	-4.19
262779_at	putative cytochrome P450 monooxy	---	AT1G13070	-1.20	-0.26	-2.87	0.025129	0.073026	-4.19
260198_at	F12A21.23 hypothetical protein	---	AT1G67635	-1.22	-0.28	-2.87	0.025147	0.073069	-4.19
255574_at	putative calcium-regulated protein	CBL5	AT4G01420	1.26	0.34	2.87	0.025172	0.073133	-4.19
262197_at	AP2 domain containing protein, pu	RAP2.12	AT1G53910	1.49	0.57	2.87	0.025175	0.073133	-4.19
253827_at	Expressed protein ; supported by cl	---	AT4G28085	-1.99	-0.99	-2.87	0.025181	0.073142	-4.19
251866_at	putative protein MSP58 - nucleolar	emb1967	AT3G54350	1.21	0.28	2.86	0.025218	0.073235	-4.19
250958_at	laccase precursor - like laccase pre	LAC11	AT5G03260	-2.06	-1.04	-2.86	0.025225	0.073235	-4.19
255130_at	hypothetical protein ; supported by	---	AT4G08240	-1.29	-0.37	-2.86	0.025226	0.073235	-4.19
253404_at	putative pyrophosphate--fructose-6	PFK6	AT4G32840	-1.32	-0.40	-2.86	0.025226	0.073235	-4.19
248045_at	HEAT SHOCK PROTEIN 81-2 (HSI	HSP81-2	AT5G56030	-1.80	-0.85	-2.86	0.025237	0.073257	-4.19
248057_at	putative protein strong similarity to u	---	AT5G55520	-1.22	-0.28	-2.86	0.025248	0.073281	-4.19
247566_at	40S ribsomal protein S19 - like 40S	---	AT5G61170	1.68	0.74	2.86	0.025268	0.073326	-4.19
263290_at	unknown protein	---	AT2G10930	-1.20	-0.26	-2.86	0.02527	0.073326	-4.19
260541_at	putative trypsin inhibitor	---	AT2G43530	-2.10	-1.07	-2.86	0.025275	0.073331	-4.19
266312_at	20S proteasome subunit C8 (PAG1	PAG1	AT2G27020	1.29	0.37	2.86	0.025285	0.07335	-4.19
252269_at	putative protein ;supported by full-l	LSU1	AT3G49580	1.91	0.93	2.86	0.025305	0.073397	-4.19
262687_at	PPR-repeat protein contains multipl	---	AT1G62670	1.21	0.28	2.86	0.025308	0.073397	-4.19
258347_at	unknown protein identical to LEA-lik	---	AT3G17520	-1.28	-0.35	-2.86	0.025313	0.073404	-4.19
257752_at	hypothetical protein predicted by ge	---	AT3G18720	-1.27	-0.35	-2.86	0.025338	0.073449	-4.20
251958_at	putative protein predicted proteins,	---	AT3G53560	1.54	0.62	2.86	0.025338	0.073449	-4.20
253535_at	putaive DNA-binding protein DNA-I	WRKY11	AT4G31550	2.37	1.25	2.86	0.02534	0.073449	-4.20
262717_s_a	putative cytochrome P450 similar to	CYP79F2	AT1G16400 ,	-1.42	-0.50	-2.86	0.025342	0.073449	-4.20
265412_at	putative chloroplast outer membran	TOC132	AT2G16640	1.20	0.27	2.86	0.025352	0.07347	-4.20
253580_at	putative protein RING-H2 finger pro	---	AT4G30400	-1.67	-0.74	-2.86	0.025355	0.07347	-4.20
248205_at	cotton fiber expressed protein 1-like	---	AT5G54300	-2.05	-1.04	-2.86	0.025365	0.073489	-4.20

258025_at	putative D-3-phosphoglycerate deh	---	AT3G19480	-1.48	-0.56	-2.86	0.025369	0.073492	-4.20
267094_at	putative diphenol oxidase ; support	IRX12	AT2G38080	-2.20	-1.14	-2.86	0.025376	0.073502	-4.20
247556_at	putative protein predicted protein, /	---	AT5G61040	1.33	0.42	2.86	0.025383	0.073513	-4.20
263643_at	unknown protein predicted by gene	---	AT2G04740	1.42	0.50	2.86	0.025397	0.073543	-4.20
251218_at	CP12 protein precursor-like protein	CP12-2	AT3G62410	1.35	0.43	2.86	0.025414	0.073585	-4.20
256025_at	xylan endohydrolase, putative simi	RXF12	AT1G58370	-1.45	-0.54	-2.86	0.025418	0.073585	-4.20
266918_at	putative LIM-domain protein	---	AT2G45800	-1.22	-0.28	-2.86	0.025436	0.073627	-4.20
262589_s_a	hypothetical protein Strong similarit	---	AT1G15150	-1.24	-0.31	-2.86	0.025443	0.073627	-4.20
256815_at	unknown protein contains Pfam pro	---	AT3G21380	-1.24	-0.31	-2.86	0.025444	0.073627	-4.20
259484_at	calcium-dependent protein kinase,	PEPKR1	AT1G12580	-1.33	-0.41	-2.86	0.025447	0.073627	-4.20
264267_at	hypothetical protein contains simila	---	AT1G60200	-1.28	-0.36	-2.86	0.025448	0.073627	-4.20
263629_at	hypothetical protein predicted by ge	---	AT2G04850	-1.46	-0.54	-2.86	0.025463	0.073659	-4.20
255882_x_a	hypothetical protein contains simila	---	AT1G20280	1.28	0.36	2.86	0.025485	0.073715	-4.20
254692_at	putative protein predicted protein, /	---	AT4G17860	-1.25	-0.33	-2.86	0.025494	0.073731	-4.20
251317_at	putative protein polygalacturonase	---	AT3G61490	-1.43	-0.51	-2.86	0.025512	0.07377	-4.20
261397_at	hexose transporter, putative simila	SGB1	AT1G79820	-1.50	-0.58	-2.86	0.025514	0.07377	-4.20
245037_at	putative phosphatidylinositol-4-phos	PIP5K3	AT2G26420	-1.21	-0.27	-2.86	0.025523	0.073786	-4.20
255009_at	putative DNA-directed RNA polyme	---	AT4G10070	1.39	0.48	2.86	0.025528	0.073786	-4.20
256065_at	ribosomal protein, putative similar t	---	AT1G07070	1.58	0.66	2.86	0.025529	0.073786	-4.20
266049_at	putative translation initiation factor	---	AT2G40780	1.25	0.32	2.85	0.025573	0.073902	-4.21
257848_at	hypothetical protein predicted by ge	---	AT3G13030	1.33	0.41	2.85	0.025578	0.073902	-4.21
258653_at	hypothetical protein similar to auxir	---	AT3G09870	-1.32	-0.40	-2.85	0.025579	0.073902	-4.21
246725_at	putative protein various predicted p	---	AT5G29040	-1.41	-0.49	-2.85	0.025602	0.07396	-4.21
249273_at	unknown protein	---	AT5G41850	1.31	0.39	2.85	0.025608	0.073966	-4.21
250577_at	putative protein densin-180, Rattus	---	AT5G07910	-1.22	-0.28	-2.85	0.025615	0.073968	-4.21
245785_at	cellulose synthase catalytic subuni	ATCSLD6	AT1G32180	-1.26	-0.33	-2.85	0.025615	0.073968	-4.21
254315_at	putative glycine-rich protein glycine	---	---	-1.38	-0.47	-2.85	0.02562	0.073968	-4.21
246887_at	hexose transporter - like protein he	---	AT5G26250	-1.26	-0.33	-2.85	0.025621	0.073968	-4.21
259887_at	putative protein kinase similar to pro	---	AT1G76360	1.27	0.34	2.85	0.025627	0.073972	-4.21
263087_s_a	putative sugar transporter	---	AT2G16120	-1.26	-0.33	-2.85	0.025629	0.073972	-4.21
260770_at	RING-H2 finger protein RHA3a, pu	---	AT1G49200	-2.73	-1.45	-2.85	0.025646	0.074012	-4.21
250462_at	receptor protein kinase -like(fragme	---	AT5G10020	-1.19	-0.25	-2.85	0.025653	0.074022	-4.21
259409_at	hypothetical protein contains simila	---	AT1G13330	1.24	0.31	2.85	0.02569	0.074116	-4.21
251870_at	putative protein Various predicted p	---	AT3G54510	-1.24	-0.31	-2.85	0.025692	0.074116	-4.21
245716_at	Expressed protein ; supported by cI	NDC1	AT5G08740	-1.48	-0.57	-2.85	0.025697	0.074122	-4.21

259615_at	dessication-related protein, putativ	---	AT1G47980	-1.31	-0.38	-2.85	0.025731	0.07421	-4.21
262576_at	unknown protein ESTs gb|R90323,	---	AT1G15280	1.21	0.28	2.85	0.025735	0.074212	-4.21
257536_at	unknown protein	---	AT3G02800	3.09	1.63	2.85	0.025749	0.074243	-4.21
251073_at	putative protein hypothetical protein	---	AT5G01750	-1.20	-0.26	-2.85	0.025768	0.074279	-4.21
256122_at	hypothetical protein predicted by ge	---	AT1G18180	-1.27	-0.35	-2.85	0.02577	0.074279	-4.21
258365_s_a	putative diaminopimelate decarboxy	---	AT3G14390	1.28	0.36	2.85	0.025772	0.074279	-4.21
255521_at	putative sucrose synthetase similar	SUS3	AT4G02280	1.45	0.53	2.85	0.025775	0.074279	-4.21
246207_at	RNA-binding like protein non-conse	---	AT4G36960	-1.24	-0.31	-2.85	0.025782	0.074287	-4.21
256994_s_a	limonene cyclase, putative similar t	ATTPS-CIN	AT3G25820	-1.24	-0.31	-2.85	0.025784	0.074287	-4.21
257396_at	predicted protein	EPF1	AT2G20875	-1.34	-0.42	-2.85	0.025847	0.07446	-4.22
246800_at	glycine hydroxymethyltransferase -	SHM2	AT5G26780	1.33	0.41	2.85	0.025869	0.074513	-4.22
251499_at	putative protein putative callose sy	ATGSL11	AT3G59100	-1.25	-0.32	-2.85	0.025873	0.074516	-4.22
249948_at	eIF-4AII - like protein EUKARYOTI	---	AT5G19210	1.35	0.43	2.85	0.02588	0.074528	-4.22
249143_at	putative protein strong similarity to u	---	AT5G43230	-1.20	-0.27	-2.85	0.025886	0.074534	-4.22
257890_s_a	putative protein peroxidase - Lyco	---	AT3G17070	-1.36	-0.45	-2.85	0.025893	0.074545	-4.22
263133_at	hypothetical protein predicted by ge	---	AT1G78450	-2.45	-1.30	-2.85	0.025897	0.074546	-4.22
259485_at	ATP synthase gamma-subunit, put	ATPC2	AT1G15700	1.65	0.72	2.85	0.025902	0.074547	-4.22
246580_at	ABC transporter, putative contains	---	AT1G31770	1.33	0.41	2.85	0.025903	0.074547	-4.22
266675_s_a	hypothetical protein predicted by ge	---	AT2G29610	-1.19	-0.25	-2.85	0.025921	0.074588	-4.22
257312_at	Expressed protein ; supported by fu	AT3G2659	---	1.22	0.29	2.85	0.025928	0.074594	-4.22
251650_at	hypothetical protein	---	AT3G57360	1.38	0.47	2.85	0.02593	0.074594	-4.22
258586_s_a	putative trypsin inhibitor similar to tr	---	AT3G04320	1.77	0.83	2.84	0.025934	0.074597	-4.22
254814_at	hypothetical protein ; supported by	---	AT4G12340	1.19	0.25	2.84	0.025939	0.074601	-4.22
255154_at	putative protein several hypothetica	---	---	1.20	0.27	2.84	0.025962	0.074659	-4.22
249722_at	putative protein similar to unknown	---	AT5G35430	-1.20	-0.26	-2.84	0.025973	0.074675	-4.22
251161_at	putative protein hypothetical protei	---	AT3G63290	1.27	0.35	2.84	0.025974	0.074675	-4.22
254377_at	subtilisin proteinase - like subtilisin	---	AT4G21650	-3.17	-1.66	-2.84	0.026001	0.074743	-4.22
263727_at	putative cap-binding protein ; supp	ABH1	AT2G13540	1.33	0.41	2.84	0.026013	0.074764	-4.22
259497_at	unknown protein	---	AT1G15860	1.20	0.26	2.84	0.026015	0.074764	-4.22
259608_at	unknown protein	ECT9	AT1G27960	-1.28	-0.36	-2.84	0.026024	0.07478	-4.22
265007_s_a	Expressed protein ; supported by fu	RALFL8	AT1G61563	-1.19	-0.25	-2.84	0.026035	0.074802	-4.22
265126_s_a	hypothetical protein similar to hypot	---	AT1G55380	-1.27	-0.35	-2.84	0.026071	0.074898	-4.23
250795_at	RING zinc finger protein-like	CPuORF64	AT5G05280	1.29	0.37	2.84	0.026092	0.074943	-4.23
245558_at	hypothetical protein	---	AT4G15430	-1.21	-0.27	-2.84	0.026094	0.074943	-4.23
245079_at	putative retroelement pol polyprotei	---	---	-1.21	-0.28	-2.84	0.026102	0.074957	-4.23

253426_at	putative protein polygalacturonase	---	AT4G32375	-1.23	-0.30	-2.84	0.026119	0.074997	-4.23
264652_at	putative sugar transport protein, EF	---	AT1G08920	-1.78	-0.83	-2.84	0.026127	0.075009	-4.23
249678_at	protein kinase-like	---	AT5G35980	-1.20	-0.26	-2.84	0.026161	0.0751	-4.23
260248_at	heat shock protein 101 (HSP101) id	ATHSP101	AT1G74310	-1.59	-0.66	-2.84	0.026187	0.075163	-4.23
249635_at	putative glucan synthase glucan syı	ATGSL09	AT5G36870	-1.27	-0.34	-2.84	0.026199	0.075185	-4.23
248469_at	putative protein similar to unknown	anac097	AT5G50820	-1.34	-0.42	-2.84	0.026201	0.075185	-4.23
249524_at	putative protein similar to unknown	---	AT5G38520	-1.44	-0.53	-2.84	0.026211	0.075196	-4.23
267423_at	putative potassium transporter	KUP11	AT2G35060	1.50	0.59	2.84	0.026211	0.075196	-4.23
263888_at	unknown protein	---	AT2G37000	-1.35	-0.43	-2.84	0.026231	0.075242	-4.23
267029_at	unknown protein	ATIREG1	AT2G38460	-1.30	-0.38	-2.84	0.02625	0.075287	-4.23
250746_at	glucuronosyl transferase-like protei	---	AT5G05880	-1.22	-0.29	-2.84	0.026256	0.075296	-4.23
262131_at	hypothetical protein predicted by ge	RALF1	AT1G02900	-2.21	-1.14	-2.84	0.026279	0.075349	-4.23
255026_at	hypothetical protein	MES12	AT4G09900	1.55	0.63	2.84	0.026281	0.075349	-4.23
252550_at	putative protein MtN21, Medicago t	---	AT3G45870	-1.31	-0.39	-2.84	0.026293	0.075374	-4.23
253295_at	putative aspartate--tRNA ligase as	---	AT4G33760	1.41	0.49	2.83	0.02631	0.075405	-4.23
250502_at	heat shock protein 70 (Hsc70-5) ; s	MTHSC70-	AT5G09590	1.65	0.73	2.83	0.026311	0.075405	-4.23
248229_at	caffeic acid 3-O-methyltransferase-l	---	AT5G53810	-1.32	-0.40	-2.83	0.026319	0.07542	-4.24
265564_s_a	hypothetical protein predicted by ge	---	---	-1.25	-0.33	-2.83	0.026334	0.07544	-4.24
260427_at	hypothetical protein predicted by ge	---	AT1G72430	-1.74	-0.80	-2.83	0.026336	0.07544	-4.24
256415_at	unknown protein ; supported by full-	---	AT3G11210	-1.95	-0.96	-2.83	0.026336	0.07544	-4.24
255107_at	hypothetical protein	---	AT4G08730	-1.33	-0.41	-2.83	0.026351	0.075473	-4.24
253065_at	putative protein predicted proteins,	AtGRF2	AT4G37740	-1.36	-0.45	-2.83	0.026362	0.075489	-4.24
258940_at	putative lipase acylhydrolase contai	---	AT3G09930	-1.20	-0.26	-2.83	0.026363	0.075489	-4.24
266262_at	hypothetical protein predicted by ge	---	AT2G27590	1.49	0.58	2.83	0.026374	0.075509	-4.24
263240_s_a	unknown protein ; supported by cD	---	AT1G72590	1.40	0.49	2.83	0.026379	0.075509	-4.24
250770_at	laccase (diphenol oxidase)	LAC12	AT5G05390	-1.37	-0.46	-2.83	0.02638	0.075509	-4.24
263931_at	unknown protein ;supported by full-	---	AT2G36220	1.79	0.84	2.83	0.026398	0.075552	-4.24
249009_at	unknown protein ; supported by ful	MAP18	AT5G44610	1.46	0.55	2.83	0.026439	0.075657	-4.24
264379_at	hypothetical protein predicted by gr	---	AT2G25200	-1.55	-0.63	-2.83	0.026444	0.075663	-4.24
253162_at	phosphoserine aminotransferase ;s	PSAT	AT4G35630	2.35	1.23	2.83	0.026479	0.075754	-4.24
263994_at	putative VSF-1-like b-ZIP transcript	---	AT2G12900	-1.20	-0.27	-2.83	0.026484	0.075758	-4.24
264599_at	putative myosin heavy chain similar	XIA	AT1G04600	-1.24	-0.31	-2.83	0.026503	0.075804	-4.24
260210_at	putative xyloglucan fucosyltransfer	FUT3	AT1G74420	-1.33	-0.41	-2.83	0.026528	0.075867	-4.24
260211_at	hypothetical protein similar to YGL0	---	AT1G74440	-2.05	-1.04	-2.83	0.026541	0.075892	-4.24
263216_s_a	putative reticuline oxidase-like prot	---	AT1G30720	3.33	1.73	2.83	0.026561	0.075941	-4.24

256152_at	ethylene-responsive RNA helicase	---	AT1G55150	1.20	0.27	2.83	0.026571	0.07596	-4.25
262764_at	putative cytochrome P450 monooxy	CYP86C3	AT1G13140	-1.24	-0.31	-2.83	0.026586	0.075993	-4.25
248911_at	tumor-related protein-like	DOG1	AT5G45830	-1.37	-0.45	-2.83	0.026597	0.076015	-4.25
263776_s_a	putative cyclic nucleotide-regulated	ATCNGC3	AT2G46430	2.74	1.46	2.83	0.026618	0.076066	-4.25
254100_at	hypothetical protein ;supported by	---	AT4G25020	1.53	0.61	2.83	0.026635	0.076105	-4.25
262386_at	hypothetical protein predicted by ge	---	AT1G49370	-1.34	-0.42	-2.83	0.026641	0.076112	-4.25
256720_at	putative DOF zinc finger protein	---	AT2G34140	-1.59	-0.67	-2.83	0.026645	0.076115	-4.25
246956_at	unknown protein ; supported by cD	RTM2	AT5G04890	-1.25	-0.33	-2.83	0.02665	0.076119	-4.25
251744_at	putative protein CHLOROPLAST 3	---	AT3G56010	1.32	0.40	2.82	0.026678	0.07619	-4.25
258557_at	hypothetical protein similar to recep	---	AT3G05990	-1.24	-0.31	-2.82	0.026685	0.076193	-4.25
260034_at	putative DNA-binding protein Pfam	---	AT1G68810	-1.91	-0.93	-2.82	0.026687	0.076193	-4.25
246888_at	putative protein	---	AT5G26270	-1.21	-0.27	-2.82	0.026689	0.076193	-4.25
258857_at	putative serine carboxypeptidase II	scpl25	AT3G02110	-2.17	-1.12	-2.82	0.026706	0.076232	-4.25
259137_at	unknown protein Pfam HMM hit: EF	---	AT3G10300	-1.45	-0.53	-2.82	0.026719	0.076258	-4.25
245067_at	S-like ribonuclease RNS2 identical	RNS2	AT2G39780	1.27	0.35	2.82	0.026726	0.07627	-4.25
256007_at	allinase, putative similar to allinase	SAMC2	AT1G34065	-1.36	-0.44	-2.82	0.026745	0.076314	-4.25
262392_at	hypothetical protein predicted by ge	---	AT1G49520	-1.39	-0.47	-2.82	0.026748	0.076314	-4.25
266289_at	putative C-4 sterol methyl oxidase	SMO2-2	AT2G29390	-1.24	-0.31	-2.82	0.026762	0.076342	-4.25
263569_at	putative chromosome associated pr	TTN7	AT2G27170	1.42	0.50	2.82	0.026775	0.07637	-4.25
246523_at	CONSTANS-like 1	COL1	AT5G15850	-1.44	-0.53	-2.82	0.026784	0.076388	-4.25
255037_at	DNA-binding protein ;supported by	ATMYB6	AT4G09460	-1.64	-0.71	-2.82	0.026802	0.07643	-4.25
267488_at	putative cadmium-transporting ATP	HMA4	AT2G19110	1.39	0.48	2.82	0.02682	0.076471	-4.26
251648_at	protein kinase - like protein S-recep	---	AT3G57720	-1.34	-0.42	-2.82	0.026841	0.076521	-4.26
245187_s_a	F12A21.17 similar to signal recogni	---	AT1G67650	1.24	0.31	2.82	0.026864	0.076573	-4.26
260940_at	26S proteasome regulatory particle	---	AT1G45000	1.30	0.38	2.82	0.026866	0.076573	-4.26
256321_at	lipoxygenase, putative similar to lip	LOX1	AT1G55020	2.72	1.44	2.82	0.026871	0.076577	-4.26
255300_at	putative phosphatidylglycerotransfe	CLS	AT4G04870	-1.37	-0.46	-2.82	0.026885	0.076602	-4.26
263871_at	hypothetical protein predicted by ge	RKP	AT2G22010	1.31	0.38	2.82	0.026886	0.076602	-4.26
251407_at	Arm repeat containing protein - like	---	AT3G60350	1.27	0.34	2.82	0.026907	0.076651	-4.26
257926_at	unknown protein contains Pfam pro	---	AT3G23280	1.19	0.26	2.82	0.026923	0.076688	-4.26
267182_at	unknown protein	---	AT2G23360	-1.32	-0.40	-2.82	0.026984	0.076852	-4.26
249642_at	putative protein	---	AT5G36960	-1.30	-0.38	-2.82	0.027026	0.076961	-4.26
249104_at	sucrose transporter protein	ATSUC6	AT5G43610	1.24	0.31	2.82	0.027032	0.076969	-4.26
255851_at	hypothetical protein predicted by ge	---	AT1G67040	-1.31	-0.39	-2.82	0.027036	0.07697	-4.26
258637_at	putative RHO GDP-dissociation inh	---	AT3G07880	-1.32	-0.40	-2.81	0.027095	0.077129	-4.27

262453_at	hypothetical protein predicted by ge	---	AT1G11240	1.20	0.27	2.81	0.027102	0.07714	-4.27
245376_at	peroxidase like protein	---	AT4G17690	-1.22	-0.28	-2.81	0.027111	0.077147	-4.27
255288_at	coded for by A. thaliana cDNA T416	---	AT4G04670	1.42	0.51	2.81	0.027111	0.077147	-4.27
247457_at	putative protein various predicted p	---	AT5G62170	-1.22	-0.29	-2.81	0.027128	0.077184	-4.27
260848_at	pectinesterase, putative similar to p	sks8	AT1G21850	-1.36	-0.44	-2.81	0.027144	0.07722	-4.27
255689_at	hypothetical protein	---	AT4G00670	-1.20	-0.26	-2.81	0.027154	0.077239	-4.27
247046_at	unknown protein ; supported by cD	---	AT5G66540	1.27	0.34	2.81	0.027186	0.077307	-4.27
257128_at	cytochrome P450, putative contain	CYP705A1	AT3G20080	1.33	0.41	2.81	0.027186	0.077307	-4.27
256681_at	unknown protein ; supported by cD	SPP2	AT3G52340	1.97	0.98	2.81	0.027188	0.077307	-4.27
264236_at	hypothetical protein predicted by ge	---	AT1G54680	1.27	0.34	2.81	0.027204	0.077343	-4.27
245562_at	OBP33pep like protein contains nor	---	AT4G15520	1.33	0.41	2.81	0.02721	0.077349	-4.27
247958_at	putative protein similar to unknown	---	AT5G57070	-1.27	-0.35	-2.81	0.027215	0.077356	-4.27
253701_at	choline monooxygenase - like prote	---	AT4G29890	1.34	0.42	2.81	0.027227	0.077371	-4.27
255276_at	putative fatty acid desaturase simil	DES-1-LIKE	AT4G04930	-1.22	-0.29	-2.81	0.027227	0.077371	-4.27
260217_at	hypothetical protein contains Pfam	---	AT1G74600	1.21	0.27	2.81	0.027239	0.077395	-4.27
252844_at	putative protein several hypothetica	---	AT3G42140	-1.29	-0.37	-2.81	0.027265	0.077459	-4.27
259364_at	DNA-binding protein RAV1 identica	RAV1	AT1G13260	-1.30	-0.38	-2.81	0.027307	0.077568	-4.27
265397_at	hypothetical protein predicted by ge	---	---	-1.22	-0.28	-2.81	0.027321	0.077599	-4.27
260617_at	Expressed protein ; supported by fu	---	AT1G53345	1.30	0.38	2.81	0.027326	0.077599	-4.27
251173_at	putative protein hypothetical protei	---	AT3G63270	1.20	0.27	2.81	0.027332	0.077599	-4.27
262039_at	adenine phosphoribosyltransferase	APT2	AT1G80050	-1.35	-0.43	-2.81	0.027332	0.077599	-4.27
256252_at	glucosyl transferase, putative simila	---	AT3G11340	3.84	1.94	2.81	0.027336	0.077599	-4.27
259467_at	hypothetical protein predicted by ge	---	AT1G19060	-1.18	-0.24	-2.81	0.027338	0.077599	-4.28
257753_at	60S ribosomal protein, putative sim	---	AT3G18740	1.41	0.49	2.81	0.027359	0.077647	-4.28
258376_at	hypothetical protein similar to GB:A	---	AT3G17680	-1.21	-0.28	-2.81	0.027363	0.077649	-4.28
256506_at	unknown protein	---	AT1G75160	-1.29	-0.37	-2.81	0.027371	0.077664	-4.28
259456_s_a	unknown protein similar to putative	---	---	-1.26	-0.34	-2.81	0.027375	0.077665	-4.28
250166_at	putative protein many predicted pro	---	AT5G15300	1.23	0.30	2.81	0.027383	0.077676	-4.28
252596_at	receptor-like protein kinase recepto	---	AT3G45330	-1.21	-0.28	-2.81	0.027394	0.077686	-4.28
252709_at	putative protein predicted proteins,	---	AT3G43840	-1.19	-0.25	-2.81	0.027396	0.077686	-4.28
246272_at	hydroxynitrile lyase like protein	MES9	AT4G37150	1.88	0.91	2.81	0.027399	0.077686	-4.28
253945_at	putative protein gene F23F1.15 Ara	---	AT4G27050	1.20	0.26	2.81	0.027399	0.077686	-4.28
252127_at	putative protein ATP binding protei	PLP3a	AT3G50960	-1.35	-0.44	-2.81	0.027413	0.077706	-4.28
260442_at	unknown protein ; supported by ful	---	AT1G68220	1.34	0.42	2.81	0.027415	0.077706	-4.28
250019_at	putative protein similar to unknown	---	AT5G18160	-1.30	-0.38	-2.81	0.027417	0.077706	-4.28

267171_at	putative DOF zinc finger protein	DOF2.4	AT2G37590	-1.32	-0.41	-2.81	0.027421	0.077708	-4.28
260308_at	putative ABC transporter contains F	ATTAP1	AT1G70610	-1.58	-0.66	-2.81	0.027429	0.077723	-4.28
262832_s_a	unknown protein	---	AT1G14870	7.19	2.85	2.80	0.027445	0.077757	-4.28
254524_at	hypothetical protein	---	AT4G20000	2.20	1.14	2.80	0.027461	0.077777	-4.28
252331_s_a	serine palmitoyltransferase-like prot	SPT1	AT3G48780	1.28	0.35	2.80	0.027462	0.077777	-4.28
261257_at	tat-binding protein, putative similar	---	AT1G05910	1.33	0.41	2.80	0.027462	0.077777	-4.28
254375_at	putative protein (fragment) ATP(GT	QQT2	AT4G21800	1.25	0.32	2.80	0.027482	0.077822	-4.28
266631_at	putative serpin	---	AT2G35570	-1.19	-0.25	-2.80	0.027486	0.077825	-4.28
267126_s_a	putative acetone-cyanohydrin lyase	ACL	AT2G23590	-1.44	-0.52	-2.80	0.02753	0.077939	-4.28
247645_at	late embryonic abundant protein - l	---	AT5G60530	-1.24	-0.31	-2.80	0.027539	0.077955	-4.28
265852_at	putative RING zinc finger protein	---	AT2G42350	1.50	0.58	2.80	0.02755	0.077978	-4.28
261101_at	transcription factor DREB1A, putat	ddf2	AT1G63030	-1.33	-0.41	-2.80	0.027557	0.077986	-4.28
250155_at	putative protein predicted proteins,	---	AT5G15160	-1.77	-0.82	-2.80	0.027578	0.078035	-4.28
246250_at	cysteine proteinase similar to cystei	CP1	AT4G36880	3.22	1.69	2.80	0.027581	0.078035	-4.28
262785_at	putative carboxyl-terminal peptidase	---	AT1G10750	-1.42	-0.50	-2.80	0.027616	0.078124	-4.29
249417_at	calcium-binding protein - like cbp1	---	AT5G39670	3.67	1.87	2.80	0.027628	0.078148	-4.29
255186_at	putative transposon protein similar t	---	---	-1.41	-0.50	-2.80	0.027632	0.078148	-4.29
246374_at	light repressible receptor protein ki	---	AT1G51840	-1.19	-0.26	-2.80	0.027634	0.078148	-4.29
262092_at	unknown protein	---	AT1G56150	1.99	0.99	2.80	0.027647	0.078175	-4.29
250846_at	sulphite reductase ; supported by c	SIR	AT5G04590	1.53	0.61	2.80	0.027652	0.078177	-4.29
260630_at	subtilisin-like serine protease conta	ALE1	AT1G62340	-1.34	-0.43	-2.80	0.027661	0.078194	-4.29
251070_at	translation initiation factor eIF-2 be	---	AT5G01940	1.47	0.56	2.80	0.027666	0.078197	-4.29
252995_at	hypothetical protein ;supported by f	---	AT4G38370	-1.32	-0.40	-2.80	0.027679	0.078226	-4.29
256530_at	unknown protein	---	AT1G33290	1.25	0.32	2.80	0.027699	0.078274	-4.29
248185_at	flavonol 3-O-glucosyltransferase-lik	UF3GT	AT5G54060	-1.24	-0.31	-2.80	0.027731	0.078352	-4.29
261144_s_a	unknown protein ;supported by full-	---	AT1G19660	1.26	0.33	2.80	0.027735	0.078353	-4.29
261715_at	hypothetical protein similar to hypot	---	AT1G18485	-1.26	-0.33	-2.80	0.027752	0.078393	-4.29
246055_at	alpha-galactosidase - like protein a	AtAGAL1	AT5G08380	1.66	0.73	2.80	0.027772	0.078439	-4.29
259965_at	transcriptional regulator, putative fo	MSRB1	AT1G53670	-1.32	-0.40	-2.80	0.027789	0.078478	-4.29
255473_at	putative protein similar to several pr	---	AT4G02450	1.21	0.28	2.80	0.0278	0.078498	-4.29
256649_at	serine/arginine-rich protein, putativ	SCL30A	AT3G13570	1.33	0.42	2.80	0.027803	0.078498	-4.29
249127_at	actin-like protein ; supported by cD	ATARP9	AT5G43500	1.29	0.37	2.80	0.027807	0.078498	-4.29
256904_at	unknown protein	---	AT3G23980	-1.20	-0.26	-2.79	0.027846	0.078599	-4.29
247821_at	WD-40 repeat protein MSI1 (sp|O22	MSI1	AT5G58230	1.28	0.35	2.79	0.027858	0.078625	-4.29
258899_at	Expressed protein ; supported by fu	---	AT3G05675	1.32	0.40	2.79	0.027866	0.078638	-4.30

258978_at	unknown protein ; supported by cD	---	AT3G09320	1.22	0.29	2.79	0.027881	0.078671	-4.30
262503_at	hypothetical protein predicted by ge	---	AT1G21670	1.58	0.66	2.79	0.027889	0.078683	-4.30
248458_at	putative protein contains similarity t	EYE	AT5G51430	-1.23	-0.30	-2.79	0.027904	0.078706	-4.30
262149_at	hypothetical protein predicted by ge	---	AT1G52530	-1.34	-0.42	-2.79	0.027904	0.078706	-4.30
266862_at	putative MYB family transcription fa	AtMYB104	AT2G26950	-1.20	-0.26	-2.79	0.027925	0.078755	-4.30
252643_at	acidic ribosomal protein P2 -like ac	---	AT3G44590	1.72	0.78	2.79	0.027939	0.078785	-4.30
248988_at	putative protein contains similarity t	---	AT5G45190	1.27	0.34	2.79	0.027958	0.078829	-4.30
263174_at	jasmonate inducible protein, putativ	ESP	AT1G54040	-1.82	-0.87	-2.79	0.027973	0.078857	-4.30
257302_at	hypothetical protein predicted by ge	---	AT3G30220	-1.21	-0.27	-2.79	0.027975	0.078857	-4.30
247237_at	auxin-independent growth promoter	---	AT5G64600	-1.29	-0.36	-2.79	0.027982	0.078867	-4.30
263275_at	putative methylmalonate semi-aldel	ALDH6B2	AT2G14170	1.38	0.46	2.79	0.027986	0.078868	-4.30
257553_at	unknown protein contains Pfam pro	TPR2	AT3G16830	1.43	0.51	2.79	0.02799	0.07887	-4.30
263130_at	hypothetical protein predicted by ge	POLD3	AT1G78650	1.52	0.60	2.79	0.028012	0.078921	-4.30
262258_at	20S proteasome subunit PAE1 ider	PAE1	AT1G53850	1.30	0.38	2.79	0.028018	0.078929	-4.30
259458_at	glycine-rich protein, putative simila	---	AT1G44085	-1.28	-0.35	-2.79	0.028034	0.078949	-4.30
260764_at	unknown protein	---	AT1G48950	1.31	0.39	2.79	0.028038	0.078949	-4.30
256669_s_a	hypothetical protein similar to hypot	---	AT3G32200 ,	-1.29	-0.37	-2.79	0.028039	0.078949	-4.30
265965_at	putative glutamate/ornithine acetyltr	---	AT2G37500	1.26	0.34	2.79	0.028041	0.078949	-4.30
256304_at	Expressed protein ; supported by cl	---	AT1G69523	-1.48	-0.57	-2.79	0.028042	0.078949	-4.30
265778_at	unknown protein	---	AT2G07360	-1.21	-0.28	-2.79	0.028046	0.07895	-4.30
251649_at	Ca2+-transporting ATPase-like pro	ACA11	AT3G57330	1.46	0.55	2.79	0.028054	0.078962	-4.30
251486_at	60S RIBOSOMAL PROTEIN L38-li	---	AT3G59540	1.43	0.52	2.79	0.028065	0.078983	-4.30
250325_s_a	putative protein contains similarity t	---	AT5G12060 ,	-1.20	-0.27	-2.79	0.028071	0.078991	-4.30
245240_at	polyprotein, putative similar to poly	---	---	-1.30	-0.37	-2.79	0.02816	0.079233	-4.31
267585_s_a	putative actin	ACT9	AT2G42090	-1.23	-0.30	-2.79	0.028165	0.079235	-4.31
262790_at	putative translation initiation factor s	TIF3H1	AT1G10840	1.18	0.24	2.79	0.028177	0.07926	-4.31
261499_at	cytochrome P450 (CYP93A1), puta	CYP705A2	AT1G28430	-1.27	-0.35	-2.79	0.028206	0.079327	-4.31
252510_at	putative protein serine/threonine-sp	---	AT3G46270	-1.24	-0.32	-2.79	0.028208	0.079327	-4.31
248248_at	spermidine synthase ;supported by	SPDS3	AT5G53120	-1.48	-0.57	-2.78	0.02822	0.079347	-4.31
248542_at	putative protein similar to unknown	AT5G5019(	---	-1.22	-0.29	-2.78	0.028222	0.079347	-4.31
245892_at	putative lipid transfer protein lipid tr	---	AT5G09370	-1.37	-0.46	-2.78	0.028237	0.079379	-4.31
261551_at	ATP dependent copper transporter	HMA5	AT1G63440	1.24	0.31	2.78	0.028285	0.079503	-4.31
267079_at	hypothetical protein predicted by ge	---	AT2G41200	1.21	0.28	2.78	0.028288	0.079503	-4.31
258905_at	unknown protein similar to hypothet	---	AT3G06390	-1.45	-0.54	-2.78	0.028291	0.079503	-4.31
264709_at	putative DNA-binding protein, Myb	ATCDC5	AT1G09770	1.44	0.52	2.78	0.028295	0.079504	-4.31

256102_at	unknown protein	---	AT1G13680	-1.21	-0.28	-2.78	0.028312	0.07954	-4.31
250480_at	protein serine/threonine kinase-like	---	AT5G10290	1.79	0.84	2.78	0.028322	0.079558	-4.31
259338_at	s-syntaxin-like protein similar to s-s	SYP131	AT3G03800	-1.21	-0.28	-2.78	0.028325	0.079558	-4.31
246354_at	hypothetical protein	---	AT1G38950	-1.27	-0.34	-2.78	0.028339	0.079582	-4.31
264797_at	unknown protein similar to ESTs gb	---	AT1G08710	1.27	0.34	2.78	0.02834	0.079582	-4.31
256219_at	hypothetical protein predicted by ge	---	AT1G56260	1.25	0.33	2.78	0.028346	0.079587	-4.31
254886_at	putative protein bp4A protein, Bras	LCR17	AT4G11760	-1.27	-0.34	-2.78	0.028352	0.079595	-4.31
264545_at	photosystem I subunit V precursor,	PSAG	AT1G55670	-1.19	-0.25	-2.78	0.028393	0.0797	-4.31
262879_at	plastid RNA polymerase sigma-sub	SIGA	AT1G64860	1.28	0.35	2.78	0.028427	0.079786	-4.32
248867_at	bHLH transcription factor	---	AT5G46830	-1.23	-0.30	-2.78	0.028457	0.07986	-4.32
261478_at	unknown protein	MIOX1	AT1G14520	-1.39	-0.47	-2.78	0.028472	0.079892	-4.32
258646_at	MATE efflux family protein, putative	FRD3	AT3G08040	-1.22	-0.29	-2.78	0.028503	0.079969	-4.32
250551_at	SNAP25 - like protein SNAP25A pr	SNAP29	AT5G07880	-1.26	-0.33	-2.78	0.028539	0.080062	-4.32
264156_at	unknown protein	---	AT1G65280	1.19	0.25	2.78	0.028547	0.080073	-4.32
255980_at	hypothetical protein ;supported by f	---	AT1G33970	1.27	0.35	2.78	0.028574	0.080141	-4.32
250725_at	putative protein similar to unknown	---	AT5G06400	1.30	0.38	2.77	0.028636	0.080303	-4.32
259229_at	unknown protein similar to transcrip	ADA2A	AT3G07740	1.33	0.41	2.77	0.028661	0.080357	-4.32
257952_at	putative peroxidase almost identica	---	AT3G21770	-1.39	-0.48	-2.77	0.028663	0.080357	-4.32
247740_at	receptor-like protein kinase precurs	CRCK1	AT5G58940	1.93	0.95	2.77	0.028666	0.080357	-4.32
247936_at	lycopene epsilon cyclase ; support	LUT2	AT5G57030	-1.38	-0.46	-2.77	0.028671	0.08036	-4.32
246144_at	dynein light chain - like protein dyn	---	AT5G20110	-1.45	-0.53	-2.77	0.028677	0.08036	-4.33
247686_at	receptor-like protein kinase precurs	---	AT5G59700	1.93	0.95	2.77	0.028681	0.08036	-4.33
247008_at	ADP-ribosylation factor-like protein	ATARLA1D	AT5G67560	-1.23	-0.30	-2.77	0.028681	0.08036	-4.33
264672_at	hypothetical protein predicted by ge	---	AT1G09750	-6.48	-2.70	-2.77	0.028712	0.080436	-4.33
251228_at	beta-D-glucan exohydrolase-like pr	---	AT3G62710	-1.23	-0.30	-2.77	0.028728	0.080474	-4.33
253533_at	putative protein cellulose synthase	ATCSLC5	AT4G31590	-2.02	-1.02	-2.77	0.028736	0.080485	-4.33
258658_at	putative adenosine kinase similar to	ADK1	AT3G09820	-1.64	-0.71	-2.77	0.028756	0.080531	-4.33
251755_at	putative protein predicted protein, A	---	AT3G55790	1.43	0.52	2.77	0.028783	0.080597	-4.33
255266_at	receptor protein kinase - like protei	---	AT4G05200	-1.25	-0.32	-2.77	0.028801	0.080628	-4.33
252673_at	CCR4-associated factor 1-like prot	---	AT3G44240	-1.24	-0.31	-2.77	0.028801	0.080628	-4.33
256861_at	beta-amylase, putative similar to be	BAM1	AT3G23920	1.51	0.60	2.77	0.028812	0.080649	-4.33
254904_at	putative translation initiation factor	---	AT4G11160	1.37	0.45	2.77	0.028834	0.0807	-4.33
247634_at	late embryonic abundant protein - l	---	AT5G60520	-1.19	-0.25	-2.77	0.028841	0.08071	-4.33
254249_at	serine /threonine kinase - like prote	---	AT4G23280	1.78	0.84	2.77	0.028856	0.08074	-4.33
260808_at	polyprotein, putative similar to poly	---	---	-1.21	-0.27	-2.77	0.028862	0.08074	-4.33

251955_at	putative protein predicted proteins,	---	AT3G53680	-1.25	-0.33	-2.77	0.028862	0.08074	-4.33
262892_at	succinic semialdehyde dehydroger	ALDH5F1	AT1G79440	1.23	0.29	2.77	0.028869	0.08074	-4.33
266801_at	putative nucleotide-binding protein	EMB2001	AT2G22870	1.57	0.65	2.77	0.02887	0.08074	-4.33
252263_at	putative protein several hypothetica	---	AT3G49520	-1.29	-0.37	-2.77	0.028885	0.080774	-4.33
245480_at	transcription factor like protein	---	AT4G16150	1.20	0.26	2.77	0.028892	0.080782	-4.33
248768_at	60S acidic ribosomal protein P1-like	---	AT5G47700	1.45	0.54	2.77	0.028898	0.080784	-4.33
260022_at	unknown protein	---	AT1G30020	-1.25	-0.32	-2.77	0.0289	0.080784	-4.33
255989_at	putative protein	---	AT5G35069	-1.30	-0.38	-2.77	0.028904	0.080786	-4.33
252742_at	putative protein glyceraldehyde-3-p	---	AT3G43290	-1.32	-0.40	-2.77	0.028944	0.080883	-4.33
254992_at	putative transcriptional regulator	SPT16	AT4G10710	1.36	0.44	2.77	0.028946	0.080883	-4.33
264021_at	putative auxin-regulated protein ;su	---	AT2G21200	-1.67	-0.74	-2.77	0.028956	0.080902	-4.34
259007_at	putative MYB family transcription fa	MYB3R-3	AT3G09370	-1.33	-0.41	-2.77	0.028961	0.080905	-4.34
262223_at	hypothetical protein predicted by ge	---	AT1G74680	1.44	0.53	2.77	0.028972	0.08092	-4.34
264340_at	unknown protein similar to hypothet	---	AT1G70280	-1.30	-0.37	-2.77	0.028974	0.08092	-4.34
250968_at	putative protein ;supported by full-l	---	AT5G02890	-1.67	-0.74	-2.77	0.028977	0.08092	-4.34
261572_at	unknown protein	---	AT1G01170	1.30	0.37	2.77	0.028997	0.080966	-4.34
251783_at	ribosomal L23a - like protein variou	RPL23AB	AT3G55280	1.61	0.69	2.77	0.029003	0.080974	-4.34
264929_at	auxin-induced protein, putative sim	---	AT1G60730	2.63	1.39	2.77	0.029007	0.080977	-4.34
255777_at	glycine-rich RNA-binding protein, p	GR-RBP6	AT1G18630	1.34	0.42	2.77	0.029013	0.080982	-4.34
262810_at	putative salt-inducible protein simila	---	AT1G11710	1.40	0.49	2.76	0.029019	0.080989	-4.34
265595_at	26S proteasome subunit 4 ;suppor	---	AT2G20140	-1.30	-0.38	-2.76	0.029029	0.081004	-4.34
262232_at	unknown protein ; supported by cDI	---	AT1G68600	-1.42	-0.50	-2.76	0.029032	0.081004	-4.34
245746_at	bHLH transcription factor, putative	---	AT1G51070	-1.32	-0.40	-2.76	0.029055	0.081054	-4.34
265129_at	putative zinc finger protein similar t	SUF4	AT1G30970	-1.26	-0.33	-2.76	0.029057	0.081054	-4.34
255727_at	hypothetical protein similar to unkno	---	AT1G25510	-1.27	-0.34	-2.76	0.029069	0.081079	-4.34
252225_at	ADP-RIBOSYLATION FACTOR -li	ATARLA1C	AT3G49870	-1.22	-0.29	-2.76	0.029086	0.081114	-4.34
254405_at	putative protein arm repeat contain	PUB8	AT4G21350	1.40	0.48	2.76	0.02909	0.081114	-4.34
265590_at	SKP1/ASK1 (At17), putative simila	MEO	AT2G20160	-1.23	-0.30	-2.76	0.029092	0.081114	-4.34
247591_at	high-affinity nitrate transporter ACH	NRT2.4	AT5G60770	-1.23	-0.29	-2.76	0.029107	0.081145	-4.34
252101_at	rac-like GTP binding protein Arac11	ROP1	AT3G51300	-1.25	-0.32	-2.76	0.029123	0.08118	-4.34
248517_at	St12p protein	---	AT5G50550	-1.24	-0.31	-2.76	0.029132	0.081195	-4.34
264538_at	hypothetical protein predicted by ge	WRKY10	AT1G55600	-1.30	-0.38	-2.76	0.029148	0.081221	-4.34
259982_at	putative RING zinc finger protein c	ATL8	AT1G76410	-1.86	-0.90	-2.76	0.029151	0.081221	-4.34
247436_at	dehydrogenase ; supported by cDN	ALDH12A1	AT5G62530	1.35	0.43	2.76	0.029152	0.081221	-4.34
264279_s_a	glycoprotein(EP1), putative similar	---	AT1G78820	-1.36	-0.44	-2.76	0.029157	0.081224	-4.34

256435_at	putative leucoanthocyanidin dioxyge	---	AT3G11180	-1.26	-0.33	-2.76	0.029167	0.081244	-4.34
265889_at	hypothetical protein predicted by ge	---	AT2G15130	-1.29	-0.36	-2.76	0.029179	0.081267	-4.34
248230_at	putative protein similar to unknown	---	AT5G53830	1.56	0.64	2.76	0.02919	0.081289	-4.34
253135_at	cytoplasmatic aconitate hydratase (	---	AT4G35830	1.38	0.46	2.76	0.029199	0.081301	-4.34
261775_at	hypothetical protein predicted by ge	---	AT1G76280	1.27	0.35	2.76	0.029205	0.081308	-4.34
251969_at	Cytochrom P450 -like protein CYT(	LUT1	AT3G53130	-1.33	-0.41	-2.76	0.029215	0.081328	-4.34
248359_at	unknown protein	---	AT5G52410	-1.29	-0.36	-2.76	0.029243	0.081394	-4.35
247746_at	uncoupling protein AtUCP2 ;suppo	ATUCP2	AT5G58970	-1.35	-0.43	-2.76	0.029251	0.081407	-4.35
252552_at	putative protein	---	AT3G45900	1.36	0.45	2.76	0.029261	0.081425	-4.35
265216_at	putative NPK1-related MAP kinase	MAPKKK18	AT1G05100	-1.46	-0.55	-2.76	0.029273	0.081448	-4.35
252350_at	ataxia-telangiectasia mutated prote	ATM	AT3G48190	1.21	0.27	2.76	0.02928	0.081458	-4.35
261008_at	hypothetical protein contains similar	---	AT1G26370	1.32	0.40	2.76	0.029316	0.081548	-4.35
255877_at	putative PTR2 family peptide transp	---	AT2G40460	-1.82	-0.87	-2.76	0.029347	0.081624	-4.35
255988_at	putative protein	---	---	-1.24	-0.31	-2.76	0.02935	0.081624	-4.35
254157_at	putative protein protein induced up	VEP1	AT4G24220	-1.26	-0.33	-2.76	0.029361	0.081644	-4.35
252848_at	putative protein several hypothetica	---	---	-1.28	-0.36	-2.76	0.029381	0.081676	-4.35
258157_at	putative MYB family transcription fa	MYB4R1	AT3G18100	1.40	0.49	2.76	0.029382	0.081676	-4.35
254744_at	Expressed protein ; supported by cl	MEE55	AT4G13345	1.38	0.46	2.76	0.029384	0.081676	-4.35
267244_at	putative reverse transcriptase	---	AT2G02650	-1.22	-0.29	-2.76	0.029387	0.081676	-4.35
251527_at	putative protein hypothetical protei	---	AT3G58650	-1.23	-0.30	-2.76	0.029394	0.081687	-4.35
252891_at	putative WRKY DNA-binding prote	WRKY13	AT4G39410	-1.36	-0.44	-2.76	0.029402	0.081697	-4.35
257427_at	hypothetical protein predicted by ge	---	AT1G79060	-1.44	-0.53	-2.76	0.029412	0.081715	-4.35
245800_at	heat shock transcription factor, put	HSFB4	AT1G46264	-1.24	-0.31	-2.75	0.029442	0.081789	-4.35
267061_at	unknown protein ;supported by full-	---	AT2G32480	1.24	0.31	2.75	0.029461	0.081832	-4.35
260722_at	protein phosphatase-2C, putative s	---	AT1G48040	1.45	0.53	2.75	0.029465	0.081835	-4.35
257787_at	actin related protein, putative simila	ARP2	AT3G27000	1.19	0.25	2.75	0.029488	0.081886	-4.35
262468_at	alanine--tRNA ligase, putative simi	ALATS	AT1G50200	1.35	0.43	2.75	0.029492	0.081888	-4.35
265389_at	putative riboflavin synthase alpha c	---	AT2G20690	1.29	0.36	2.75	0.029507	0.081917	-4.35
250165_at	putative protein many predicted pro	---	AT5G15290	-1.24	-0.31	-2.75	0.02951	0.081917	-4.35
248528_at	putative protein similar to unknown	---	AT5G50760	3.72	1.90	2.75	0.029513	0.081917	-4.35
252830_at	putative protein 70K peroxisomal m	PXA1	AT4G39850	1.20	0.26	2.75	0.029534	0.081965	-4.36
255843_at	unknown protein	CPL3	AT2G33540	-1.20	-0.27	-2.75	0.029561	0.08203	-4.36
247783_at	light harvesting pigment - like prote	---	AT5G58800	1.54	0.62	2.75	0.029566	0.082035	-4.36
249301_at	putative protein similar to unknown	---	AT5G41440	-1.30	-0.38	-2.75	0.029608	0.082133	-4.36
264371_at	pEARLI 1-like protein may be induc	ELP	AT1G12090	-1.55	-0.63	-2.75	0.029609	0.082133	-4.36

251685_at	putative protein unknown protein A	---	AT3G56430	1.64	0.71	2.75	0.029631	0.082185	-4.36
256877_at	unknown protein ;supported by full-	---	AT3G26470	1.83	0.87	2.75	0.029676	0.082299	-4.36
264569_at	unknown protein EST gb|ATTS113	---	---	-1.21	-0.28	-2.75	0.029697	0.082349	-4.36
252212_at	protein kinase -like protein NTPK1	MAPKKK2	AT3G50310	1.72	0.78	2.75	0.029718	0.08239	-4.36
248651_at	unknown protein	---	AT5G49260	-1.21	-0.27	-2.75	0.02972	0.08239	-4.36
255525_at	coded for by A. thaliana cDNA T042	---	AT4G02340	-1.28	-0.36	-2.75	0.029731	0.08241	-4.36
252343_at	putative protein hypothetical protei	---	AT3G48610	-2.03	-1.02	-2.75	0.029743	0.082434	-4.36
250208_at	unknown protein ;supported by full-	anac084	AT5G14000	1.93	0.95	2.75	0.029746	0.082434	-4.36
247713_at	putative protein nonspecific lipid-tra	---	AT5G59330	-1.24	-0.31	-2.75	0.029758	0.082456	-4.36
260955_at	unknown protein contains similarity	---	AT1G06000	-1.64	-0.71	-2.75	0.029829	0.082638	-4.37
259371_at	unknown protein ;supported by full-	---	AT1G69080	-1.96	-0.97	-2.75	0.029831	0.082638	-4.37
248777_at	putative protein similar to unknown	---	AT5G47920	-1.27	-0.34	-2.74	0.029865	0.082723	-4.37
261557_at	kinesin-like protein C-terminal regio	---	AT1G63640	1.35	0.44	2.74	0.029889	0.08278	-4.37
249130_at	putative protein contains similarity t	---	AT5G43100	1.21	0.27	2.74	0.029911	0.082826	-4.37
248576_at	methionine S-methyltransferase (gb	MMT	AT5G49810	1.23	0.30	2.74	0.029913	0.082826	-4.37
251876_at	TATA box binding protein (TBP) as	RGD3	AT3G54280	1.26	0.33	2.74	0.029924	0.082836	-4.37
264879_at	cotton fiber expressed protein, puta	---	AT1G61260	-1.67	-0.74	-2.74	0.029924	0.082836	-4.37
265022_at	putative agp1 similar to GB:CAA48	BCP1	AT1G24520	-1.34	-0.42	-2.74	0.029933	0.082847	-4.37
257172_at	hypothetical protein predicted by g	---	AT3G23700	-1.32	-0.40	-2.74	0.029935	0.082847	-4.37
259206_at	hypothetical protein similar to putati	---	AT3G09040	1.29	0.37	2.74	0.029948	0.082872	-4.37
249406_at	putative protein MtN21, Medicago t	---	AT5G40210	-1.30	-0.38	-2.74	0.029957	0.082879	-4.37
247822_at	putative protein sorting nexin 2, Ho	SNX2a	AT5G58440	-1.29	-0.37	-2.74	0.029959	0.082879	-4.37
248682_at	cytochrome b5 (dbj|BAA74840.1) ;	CB5-D	AT5G48810	1.23	0.30	2.74	0.029961	0.082879	-4.37
262014_at	unknown protein	---	AT1G35660	1.51	0.59	2.74	0.030002	0.082982	-4.37
254566_at	hypothetical protein	---	AT4G19240	1.32	0.40	2.74	0.030021	0.083018	-4.37
247291_at	unknown protein	---	AT5G64480	-1.25	-0.32	-2.74	0.030023	0.083018	-4.37
245678_at	unknown protein	---	AT1G56610	1.35	0.43	2.74	0.030031	0.083023	-4.37
251138_at	putative protein various predicted p	---	AT5G01160	1.27	0.34	2.74	0.030032	0.083023	-4.37
256824_at	hypothetical protein predicted by ge	AT3G2212	---	-1.46	-0.55	-2.74	0.030049	0.083062	-4.37
263181_at	unknown protein ;supported by full-	---	AT1G05720	1.22	0.28	2.74	0.030053	0.083062	-4.37
245606_at	hypothetical protein	---	AT4G14310	-1.26	-0.34	-2.74	0.030092	0.083154	-4.38
264901_at	putative sulphate transporter protei	AST91	AT1G23090	2.00	1.00	2.74	0.030094	0.083154	-4.38
251388_at	putative protein protein regulating c	MAP65-4	AT3G60840	-1.27	-0.34	-2.74	0.030107	0.083181	-4.38
254201_at	putative protein predicted proteins	---	AT4G24130	-1.34	-0.43	-2.74	0.030121	0.08321	-4.38
250349_at	putative receptor - like kinase rece	---	AT5G12000	-1.27	-0.34	-2.74	0.030149	0.083275	-4.38

257972_at	putative protein kinase, ATN1 almo	ATN1	AT3G27560	1.27	0.34	2.74	0.030155	0.083282	-4.38
261043_at	hypothetical protein similar to unkno	---	AT1G01220	1.31	0.38	2.74	0.030177	0.083334	-4.38
251660_at	Expressed protein ; supported by fu	---	AT3G57160	-1.36	-0.45	-2.74	0.03026	0.083552	-4.38
266206_at	unknown protein ; supported by ful	---	AT2G27730	1.33	0.41	2.73	0.030313	0.083678	-4.38
260175_at	neutral invertase, putative similar to	---	AT1G72000	-1.21	-0.27	-2.73	0.030313	0.083678	-4.38
248396_at	unknown protein	---	AT5G52130	-1.28	-0.36	-2.73	0.030324	0.083679	-4.38
264226_at	hypothetical protein similar to putati	---	AT1G67510	-1.25	-0.32	-2.73	0.030326	0.083679	-4.38
255632_at	putative actin-depolymerizing factor	ADF8	AT4G00680	-1.19	-0.25	-2.73	0.030326	0.083679	-4.38
264112_at	putative 1,3-beta-D-glucan synthas	CALS5	AT2G13680	-1.28	-0.36	-2.73	0.030328	0.083679	-4.38
265263_at	putative DNA binding protein	---	AT2G42940	-1.23	-0.30	-2.73	0.030363	0.083766	-4.38
262492_at	unknown protein Contains similarity	---	AT1G21630	-1.25	-0.32	-2.73	0.030396	0.083849	-4.39
264255_at	putative SF2/ASF splicing modulato	---	AT1G09140	1.35	0.43	2.73	0.030443	0.083967	-4.39
255402_at	Expressed protein	hemf2	AT4G03205	-1.54	-0.63	-2.73	0.030466	0.08402	-4.39
259469_at	hypothetical protein predicted by ge	---	AT1G19100	1.22	0.29	2.73	0.030478	0.084039	-4.39
244951_s_a	cytochrome c biogenesis orf452	---	AT2G07723	1.56	0.64	2.73	0.03048	0.084039	-4.39
253790_at	photosystem II protein W - like pho	PSB28	AT4G28660	-1.45	-0.54	-2.73	0.030506	0.084094	-4.39
248759_at	putative protein similar to unknown	---	AT5G47610	1.66	0.73	2.73	0.03051	0.084094	-4.39
259384_at	putative lectin contains Pfam profile	---	AT3G16450	-1.31	-0.39	-2.73	0.030511	0.084094	-4.39
265563_s_a	hypothetical protein predicted by ge	---	---	-1.18	-0.24	-2.73	0.030548	0.084173	-4.39
258824_at	unknown protein	---	AT3G07190	1.34	0.42	2.73	0.030551	0.084173	-4.39
267447_at	putative GTP-binding protein	ArRABA1h	AT2G33870	-1.19	-0.26	-2.73	0.030551	0.084173	-4.39
266440_at	unknown protein	---	AT2G43235	1.23	0.30	2.73	0.030631	0.084383	-4.39
257963_at	hypothetical protein predicted by ge	---	AT3G19840	1.49	0.57	2.73	0.030636	0.084388	-4.39
254469_at	hypothetical protein	---	AT4G20470	-1.23	-0.30	-2.73	0.030648	0.084402	-4.39
247456_at	iron-regulated transporter - like pro	AtZIP12	AT5G62160	-1.23	-0.30	-2.73	0.030649	0.084402	-4.39
255426_at	putative protein	---	AT4G03370	-1.23	-0.30	-2.73	0.030657	0.084413	-4.39
258517_at	galactose kinase identical to GB:A/	GALK	AT3G06580	-1.25	-0.32	-2.72	0.030707	0.084542	-4.40
252974_at	putative protein ; supported by cDN	MTN1	AT4G38800	1.22	0.29	2.72	0.030721	0.08457	-4.40
254588_at	putative protein hypothetical protein	---	AT4G18840	1.23	0.30	2.72	0.030729	0.084574	-4.40
255058_s_a	putative protein various hypothetica	---	---	-1.19	-0.25	-2.72	0.030731	0.084574	-4.40
259453_at	gibberelin 20-oxidase, putative, 5' p	ATGA20OX	AT1G44090	-1.35	-0.43	-2.72	0.030734	0.084574	-4.40
262521_at	unknown protein Location of est 27	---	AT1G17130	1.25	0.32	2.72	0.030756	0.084614	-4.40
246002_at	ripening-related protein - like grip28	---	AT5G20740	-1.60	-0.68	-2.72	0.030759	0.084614	-4.40
261685_at	3-beta-hydroxysteroid dehydrogena	AT3BETAH	AT1G47290	-1.61	-0.69	-2.72	0.030763	0.084614	-4.40
260397_at	hypothetical protein predicted by ge	---	AT1G69680	1.25	0.32	2.72	0.030766	0.084614	-4.40

263086_at	putative non-LTR retrolelement reve	---	AT2G16110	-1.24	-0.31	-2.72	0.030769	0.084614	-4.40
254847_at	putative phospholipase D-gamma	PLDGAMM	AT4G11850	1.72	0.79	2.72	0.030771	0.084614	-4.40
254961_at	putative acyl-CoA synthetase acyl-	---	AT4G11030	-1.25	-0.32	-2.72	0.030774	0.084614	-4.40
250301_at	putative protein predicted proteins,	---	AT5G11970	-1.36	-0.45	-2.72	0.030788	0.084641	-4.40
262787_at	putative clathrin-coat assembly prot	---	AT1G10730	1.42	0.50	2.72	0.030794	0.084649	-4.40
254789_at	blue copper-binding protein, 15K (l	---	AT4G12880	-3.59	-1.85	-2.72	0.030805	0.08467	-4.40
247254_at	CLE21, putative CLAVATA3/ESR-l	CLE21	AT5G64800	1.51	0.59	2.72	0.030846	0.084756	-4.40
266240_at	putative RNA-binding protein ; sup	---	AT2G29580	1.18	0.24	2.72	0.030847	0.084756	-4.40
253467_at	putative protein hypothetical protei	---	AT4G32140	-1.28	-0.35	-2.72	0.030848	0.084756	-4.40
250178_at	putative protein various unknown o	---	AT5G14430	1.21	0.27	2.72	0.030899	0.084883	-4.40
247898_at	F-box protein ZTL/LFK1/ADO1, At	ZTL	AT5G57360	1.25	0.32	2.72	0.030902	0.084883	-4.40
264123_at	unknown protein ; supported by cD	---	AT1G02270	1.28	0.35	2.72	0.030928	0.084936	-4.40
250274_at	putative protein predicted protein,	---	AT5G13020	1.27	0.35	2.72	0.030929	0.084936	-4.40
248635_at	unknown protein	---	AT5G49050	-1.22	-0.29	-2.72	0.030933	0.084936	-4.40
266275_at	putative tropinone reductase	---	AT2G29370	-1.31	-0.39	-2.72	0.030936	0.084936	-4.40
254393_at	putative NADPH quinone oxidored	---	AT4G21580	1.24	0.31	2.72	0.03094	0.084936	-4.40
257946_at	hypothetical protein predicted by ge	---	AT3G21710	1.23	0.30	2.72	0.030984	0.085035	-4.41
262938_at	hypothetical protein predicted by ge	---	AT1G79540	1.27	0.34	2.72	0.030985	0.085035	-4.41
252064_at	putative protein various predicted p	---	AT3G51520	1.23	0.30	2.72	0.03099	0.085035	-4.41
248612_at	putative protein contains similarity t	---	AT5G49530	1.40	0.49	2.72	0.030991	0.085035	-4.41
251489_at	putative protein putative proteins - A	---	AT3G59460	-1.28	-0.35	-2.72	0.031001	0.085054	-4.41
257829_at	unknown protein	SNM1	AT3G26680	1.47	0.56	2.72	0.031017	0.085087	-4.41
262246_at	leaf development protein Argonaute	AGO1	AT1G48410	-1.27	-0.35	-2.72	0.031042	0.085145	-4.41
252179_at	putative protein UDP-glucose:(gluc	GATL2	AT3G50760	-1.39	-0.48	-2.72	0.031054	0.085169	-4.41
252863_at	myo-inositol-1-phosphate synthase	MIPS1	AT4G39800	-2.95	-1.56	-2.72	0.031077	0.085219	-4.41
258918_at	putative cytochrome P450 similar to	UNE9	AT3G10560	-1.38	-0.47	-2.72	0.031083	0.085219	-4.41
255199_at	coded for by A. thaliana cDNA N64	---	AT4G07390	1.22	0.28	2.72	0.031084	0.085219	-4.41
259998_at	hypothetical protein similar to unkn	BPC3	AT1G68120	-1.36	-0.44	-2.72	0.031115	0.085295	-4.41
254429_at	Expressed protein ; supported by fu	---	AT4G21105	1.24	0.31	2.72	0.031132	0.085325	-4.41
261654_at	hypothetical protein contains simila	---	AT1G01920	1.34	0.42	2.72	0.031134	0.085325	-4.41
262415_at	40S ribosomal protein S17, putativ	emb1129	AT1G49400	1.32	0.40	2.71	0.03115	0.085361	-4.41
262353_at	hypothetical protein similar to hypot	---	AT1G64210	-1.19	-0.25	-2.71	0.03116	0.085377	-4.41
250816_at	coatomer delta subunit (delta-coat p	---	AT5G05010	-1.33	-0.41	-2.71	0.031178	0.085415	-4.41
246597_at	L-aspartate oxidase -like protein L-	AO	AT5G14760	2.03	1.02	2.71	0.031227	0.085531	-4.41
249808_at	unknown protein	---	AT5G23890	1.52	0.61	2.71	0.031228	0.085531	-4.41

265500_at	putative non-LTR retroelement reve	---	---	-1.42	-0.50	-2.71	0.031244	0.085565	-4.41
253497_at	putative protein microtubule-associ	---	AT4G31880	1.28	0.36	2.71	0.031255	0.085586	-4.41
253045_at	Expressed protein ; supported by fu	---	AT4G37445	-1.42	-0.51	-2.71	0.031262	0.085595	-4.41
260634_at	initiation factor isozyme 4F p82 sub	---	AT1G62410	-1.25	-0.32	-2.71	0.031272	0.085611	-4.42
262127_at	hypothetical protein predicted by ge	---	AT1G52550	1.34	0.42	2.71	0.031277	0.085615	-4.42
252297_at	cytosolic ribosomal protein S11 ;su	EMB1080	AT3G48930	1.52	0.61	2.71	0.031284	0.085624	-4.42
248530_at	putative protein strong similarity to u	---	AT5G49945	-1.30	-0.38	-2.71	0.031296	0.085647	-4.42
252087_at	putative protein putative Na+/H+-ex	chx28	AT3G52080	-1.22	-0.29	-2.71	0.031306	0.085663	-4.42
248213_at	putative protein similar to unknown	AtGRF7	AT5G53660	-1.26	-0.33	-2.71	0.031312	0.085669	-4.42
246627_s_a	5-enolpyruvylshikimate-3-phosphat	---	AT1G48860	1.74	0.80	2.71	0.03132	0.08568	-4.42
256998_at	hypothetical protein predicted by ge	---	AT3G14180	-1.32	-0.40	-2.71	0.031338	0.08572	-4.42
246564_at	putative protein hypothetical protei	---	AT5G15610	1.19	0.26	2.71	0.031346	0.085731	-4.42
255219_at	hypothetical protein	---	AT4G07720	-1.21	-0.28	-2.71	0.0314	0.085868	-4.42
248961_at	subtilisin-like protease	---	AT5G45650	1.89	0.92	2.71	0.031425	0.085927	-4.42
256509_at	NADPH oxidoreductase, putative s	---	AT1G75300	-1.49	-0.58	-2.71	0.031442	0.085937	-4.42
265019_at	putative myosin heavy chain similar	---	AT1G24560	1.20	0.26	2.71	0.031443	0.085937	-4.42
245103_at	putative Ta11-like non-LTR retroele	---	AT2G41590	-1.21	-0.27	-2.71	0.031446	0.085937	-4.42
266265_at	putative tropinone reductase ; supp	---	AT2G29340	-1.42	-0.50	-2.71	0.031446	0.085937	-4.42
251389_at	putative protein	---	AT3G60850	1.22	0.28	2.71	0.031447	0.085937	-4.42
246413_at	unknown protein	---	AT1G77310	1.26	0.33	2.71	0.031475	0.085991	-4.42
246099_at	blue copper binding protein ;suppo	ATBCB	AT5G20230	1.49	0.58	2.71	0.031475	0.085991	-4.42
262397_at	hypothetical protein predicted by ge	---	AT1G49380	-1.45	-0.54	-2.71	0.031482	0.085991	-4.42
252014_at	putative protein predicted proteins,	---	AT3G52870	-1.91	-0.93	-2.71	0.031482	0.085991	-4.42
249572_at	chromosome condensation protein	EMB2656	AT5G37630	-1.30	-0.38	-2.71	0.031496	0.086019	-4.42
264399_at	postsynaptic protein CRIPT, putativ	---	AT1G61780	1.18	0.24	2.71	0.031553	0.086165	-4.42
248112_at	wax synthase-like protein	---	AT5G55350	-1.31	-0.39	-2.71	0.03157	0.086194	-4.43
262514_at	NAM(no apical meristem) protein, p	anac017	AT1G34190	1.20	0.26	2.71	0.031572	0.086194	-4.43
261111_at	homeodomain protein BELL1, puta	BLH11	AT1G75430	-1.20	-0.26	-2.70	0.031594	0.086244	-4.43
260689_at	hypothetical protein predicted by ge	---	AT1G32290	-1.31	-0.39	-2.70	0.031605	0.086255	-4.43
266525_at	putative tetracycline transporter pro	MEE15	AT2G16970	-1.28	-0.36	-2.70	0.031609	0.086255	-4.43
253187_at	putative zinc-finger protein PEThy;	---	AT4G35280	-1.20	-0.26	-2.70	0.031609	0.086255	-4.43
266571_at	unknown protein	---	AT2G23830	1.56	0.64	2.70	0.031624	0.086284	-4.43
260461_at	membrane protein PTM1 precursor	---	AT1G10980	-1.26	-0.33	-2.70	0.03163	0.086289	-4.43
266815_at	F-box protein family, AtFBX5 conta	---	AT2G44900	-1.28	-0.36	-2.70	0.031633	0.086289	-4.43
262025_at	growth regulator, putative similar to	---	AT1G35510	-1.28	-0.36	-2.70	0.031642	0.086303	-4.43

253444_at	putative protein ring finger protein,	---	AT4G32600	1.21	0.28	2.70	0.031647	0.086306	-4.43
256193_at	hypothetical protein similar to hypot	---	AT1G30200	-1.22	-0.29	-2.70	0.031674	0.086369	-4.43
252451_at	hypothetical protein	---	AT3G47100	-1.21	-0.27	-2.70	0.031678	0.086371	-4.43
250924_at	putative protein ;supported by full-l	---	AT5G03440	-1.25	-0.32	-2.70	0.031693	0.086401	-4.43
264798_at	putative myosin MYA1, class V (Z2	XIC	AT1G08730	-1.23	-0.30	-2.70	0.031702	0.086413	-4.43
264588_at	unknown protein contains similarity	---	AT2G17730	1.59	0.67	2.70	0.031705	0.086413	-4.43
266297_at	putative proliferating cell nuclear ar	PCNA2	AT2G29570	2.12	1.09	2.70	0.03171	0.086417	-4.43
258973_at	putative cytochrome P450 similar to	CYP94B2	AT3G01900	-1.26	-0.33	-2.70	0.031718	0.086427	-4.43
262109_at	hypothetical protein similar to cellulo	ATCSLD5	AT1G02730	-1.51	-0.60	-2.70	0.031739	0.086475	-4.43
257884_at	hypothetical protein predicted by ge	---	AT3G16960	-1.24	-0.32	-2.70	0.031755	0.086508	-4.43
248308_at	putative protein similar to unknown	---	AT5G52530	1.22	0.29	2.70	0.031764	0.086522	-4.43
252914_at	putative protein dehydrin, Glycine r	---	AT4G39130	-1.32	-0.40	-2.70	0.031781	0.086559	-4.43
264253_at	putative kinesin similar to GB:AAB6	---	AT1G09170	-1.39	-0.48	-2.70	0.03179	0.086572	-4.43
262163_at	ribosomal protein L30, putative sim	---	AT1G77940	1.63	0.71	2.70	0.031803	0.086588	-4.43
263408_at	predicted protein	---	AT2G04060	-1.21	-0.28	-2.70	0.031803	0.086588	-4.43
255153_at	putative protein several hypothetica	---	AT4G08210	-1.31	-0.39	-2.70	0.031809	0.086592	-4.43
256256_at	unknown protein ; supported by full-	---	AT3G11230	1.25	0.32	2.70	0.031849	0.086686	-4.43
261408_s_a	histone H4 identical to GB:M36659	---	AT1G07660	1.35	0.43	2.70	0.031851	0.086686	-4.43
260143_at	sucrose transport protein SUC1 ide	SUC1	AT1G71880	1.68	0.75	2.70	0.031861	0.086689	-4.43
256127_at	small GTP-binding protein (RAB11	AtRABA6b	AT1G18200	-1.28	-0.36	-2.70	0.031862	0.086689	-4.43
262325_at	dirigent protein, putative similar to	---	AT1G64160	1.60	0.67	2.70	0.031863	0.086689	-4.43
258601_at	putative histidyl tRNA synthetase si	---	AT3G02760	1.28	0.36	2.70	0.03188	0.086724	-4.44
259694_at	uridine diphosphate glucose epime	UGE3	AT1G63180	1.86	0.90	2.70	0.031902	0.086773	-4.44
262611_at	Expressed protein ; supported by fu	---	AT1G14060	1.44	0.52	2.70	0.031907	0.086776	-4.44
256761_at	hypothetical protein predicted by ge	---	AT3G25670	-1.32	-0.40	-2.70	0.031918	0.086795	-4.44
264490_at	putative protein import receptor sim	TOM20-2	AT1G27390	1.43	0.52	2.70	0.031931	0.086822	-4.44
247732_at	putative protein various predicted p	---	AT5G59600	1.19	0.26	2.70	0.031964	0.086902	-4.44
261003_at	hypothetical protein predicted by ge	---	AT1G26500	1.29	0.37	2.70	0.031986	0.086942	-4.44
259910_at	putative P-type transporting ATPase	---	AT1G72700	-1.53	-0.61	-2.70	0.031987	0.086942	-4.44
256191_at	unknown protein similar to hypothet	---	AT1G30130	-1.27	-0.34	-2.70	0.031991	0.086943	-4.44
252796_at	putative protein predicted proteins,	---	---	-1.37	-0.45	-2.70	0.031996	0.086947	-4.44
254376_at	putative protein ; supported by cDN	TOM1	AT4G21790	-1.18	-0.24	-2.70	0.032018	0.086995	-4.44
249659_s_a	putative protein similar to unknown	---	AT5G36710	-1.33	-0.42	-2.70	0.032034	0.087029	-4.44
251895_at	class IV chitinase (CHIV)	ATEP3	AT3G54420	1.78	0.84	2.69	0.032038	0.08703	-4.44
248207_at	tyrosine aminotransferase	---	AT5G53970	1.89	0.91	2.69	0.032042	0.08703	-4.44

266924_at	unknown protein ;supported by full-	---	AT2G45730	1.30	0.38	2.69	0.032047	0.087033	-4.44
250953_at	putative protein ;supported by full-l	DPB	AT5G03415	1.38	0.47	2.69	0.032056	0.087048	-4.44
260074_at	putative ras-related GTP-binding pr	AtRABA6a	AT1G73640	-1.22	-0.29	-2.69	0.032082	0.0871	-4.44
248127_at	transport protein particle componen	---	AT5G54750	-1.21	-0.28	-2.69	0.032083	0.0871	-4.44
251641_at	protease-like protein INSULIN-DEC	---	AT3G57470	-1.36	-0.45	-2.69	0.032113	0.087172	-4.44
260138_at	calmodulin-4 identical to GB:Q035	CAM4	AT1G66410	1.29	0.37	2.69	0.032155	0.087268	-4.44
257534_at	hypothetical protein predicted by ge	---	AT3G09670	1.22	0.28	2.69	0.032157	0.087268	-4.44
254170_at	LG27/30-like gene LG27/30 gene -	---	AT4G24430	-1.19	-0.26	-2.69	0.03216	0.087268	-4.44
247316_at	ankyrin-like protein	---	AT5G64030	-1.22	-0.29	-2.69	0.032169	0.087282	-4.44
258269_at	putative acetyl-CoA carboxylase bio	---	AT3G15690	-1.23	-0.29	-2.69	0.032182	0.087295	-4.45
261225_at	unknown protein ;supported by full-	---	AT1G20100	1.22	0.28	2.69	0.032187	0.087295	-4.45
246772_at	putative protein unknown protein T	---	AT5G27490	-1.51	-0.59	-2.69	0.032187	0.087295	-4.45
263677_at	unknown protein	PDLP2	AT1G04520	-1.25	-0.33	-2.69	0.03219	0.087295	-4.45
265423_at	unknown protein	---	AT2G20790	-1.44	-0.53	-2.69	0.032193	0.087295	-4.45
254121_at	putative protein putative protein F6	---	AT4G24760	1.48	0.56	2.69	0.032203	0.087311	-4.45
258596_at	unknown protein	LSH2	AT3G04510	-1.28	-0.35	-2.69	0.032218	0.087341	-4.45
245288_at	transcriptional adaptor like protein ;	ADA2B	AT4G16420	1.23	0.30	2.69	0.032235	0.087372	-4.45
260987_at	hypothetical protein predicted by ge	NTMC2T6.	AT1G53590	1.31	0.39	2.69	0.032237	0.087372	-4.45
259628_at	hypothetical protein contains Pfam	---	AT1G56440	1.36	0.45	2.69	0.032241	0.087373	-4.45
255325_at	putative membrane trafficking facto	PUX4	AT4G04210	1.23	0.30	2.69	0.032247	0.087378	-4.45
255454_at	hypothetical protein	---	AT4G02910	-1.20	-0.27	-2.69	0.032273	0.087438	-4.45
246586_at	putative protein predicted protein, A	---	AT5G14770	1.34	0.42	2.69	0.032313	0.087536	-4.45
254619_at	myb - like protein transforming prot	MYB98	AT4G18770	-1.23	-0.30	-2.69	0.032345	0.087613	-4.45
264907_at	unknown protein similar to S.pombe	---	AT2G17280	-1.81	-0.86	-2.69	0.032351	0.087619	-4.45
253854_at	putative protein zinc-finger protein	---	AT4G27900	1.80	0.85	2.69	0.03236	0.087634	-4.45
254754_at	pectate lyase like protein pectate ly	---	AT4G13210	-1.20	-0.26	-2.69	0.032368	0.087644	-4.45
263900_at	unknown protein ;supported by full-	---	AT2G36290	1.31	0.39	2.69	0.032388	0.087687	-4.45
265056_at	mitochondrial processing peptidase	---	AT1G51980	1.33	0.41	2.69	0.032391	0.087687	-4.45
245024_at	ATPase alpha subunit	---	---	-1.20	-0.26	-2.69	0.032407	0.087718	-4.45
258727_at	ethylene-responsive protein, putati	---	AT3G11930	1.29	0.36	2.69	0.032413	0.087725	-4.45
263510_s_a	hypothetical protein	---	AT2G07681 ,	1.20	0.26	2.69	0.032422	0.087735	-4.45
253788_at	aromatic amino-acid decarboxylase	---	AT4G28680	-1.27	-0.35	-2.69	0.032425	0.087735	-4.45
265257_at	succinyl-CoA ligase beta subunit ;s	---	AT2G20420	1.51	0.60	2.69	0.032436	0.087756	-4.45
262802_at	putative cell division control protein	CDKB2;2	AT1G20930	-1.19	-0.25	-2.69	0.032473	0.087846	-4.45
251774_at	nematode resistance protein-like p	---	AT3G55840	-2.24	-1.16	-2.68	0.032507	0.087926	-4.46

265381_at	putative non-LTR retroelement reve	---	---	-1.34	-0.42	-2.68	0.032517	0.087945	-4.46
250479_at	GTP-binding protein GTP-binding p	AtRABH1e	AT5G10260	-1.25	-0.32	-2.68	0.032551	0.088024	-4.46
253236_at	putative protein ARI protein, Droso	ARI1	AT4G34370	1.20	0.26	2.68	0.03258	0.088093	-4.46
251563_at	anthranilate phosphoribosyltransfe	---	AT3G57880	-1.48	-0.56	-2.68	0.032598	0.088131	-4.46
247988_at	putative protein similar to unknown	---	AT5G56910	1.40	0.48	2.68	0.032603	0.088135	-4.46
262540_at	hypothetical protein predicted by ge	---	AT1G34260	-1.41	-0.50	-2.68	0.032622	0.088174	-4.46
266428_at	putative protein kinase contains a p	---	AT2G07180	1.30	0.38	2.68	0.032653	0.088247	-4.46
256825_at	unknown protein similar to cell wall-	CWLP	AT3G22120	-5.47	-2.45	-2.68	0.032658	0.088252	-4.46
263904_at	putative ABC transporter related to	PDR6	AT2G36380	1.88	0.91	2.68	0.032674	0.088285	-4.46
250880_at	putative protein Ube-1a, Mus musc	---	AT5G04070	1.20	0.26	2.68	0.032705	0.088334	-4.46
260642_at	plastid protein, putative similar to p	---	AT1G53260	-1.37	-0.45	-2.68	0.032709	0.088334	-4.46
249961_at	putative protein predicted proteins,	---	AT5G18770	1.27	0.35	2.68	0.03271	0.088334	-4.46
266411_at	putative peptidyl-prolyl cis-trans iso	---	AT2G38730	-1.32	-0.40	-2.68	0.032713	0.088334	-4.46
263539_at	putative tyrosine aminotransferase	TAT3	AT2G24850	-3.95	-1.98	-2.68	0.032715	0.088334	-4.46
251254_at	putative protein anion exchanger 3	---	AT3G62270	1.29	0.37	2.68	0.032716	0.088334	-4.46
260880_at	hypothetical protein similar to unkno	---	AT1G21380	-1.25	-0.32	-2.68	0.032722	0.088339	-4.46
260455_at	hypothetical protein predicted by ge	---	AT1G72500	-1.72	-0.78	-2.68	0.032741	0.088382	-4.46
256072_at	WD-40 repeat protein identical to W	ATARCA	AT1G18080	1.56	0.64	2.68	0.032748	0.088389	-4.46
249000_at	putative protein contains similarity t	---	AT5G44980	-1.25	-0.32	-2.68	0.032756	0.088397	-4.46
250900_at	putative protein ; supported by cDN	ATB' ALPH	AT5G03470	1.34	0.42	2.68	0.032759	0.088397	-4.46
255785_at	sulfate adenylyltransferase identica	APS2	AT1G19920	1.25	0.32	2.68	0.032774	0.08843	-4.46
258101_at	unknown protein	RFR1	AT3G23590	-1.52	-0.61	-2.68	0.032827	0.088551	-4.47
248948_at	putative protein strong similarity to u	---	AT5G45560	-1.36	-0.44	-2.68	0.032827	0.088551	-4.47
258056_at	hypothetical protein similar to GB:A	---	AT3G29010	-1.33	-0.42	-2.68	0.032832	0.088554	-4.47
246304_at	acyl-coA dehydrogenase Mus musc	ACX4	AT3G51840	1.35	0.44	2.68	0.032846	0.08858	-4.47
255435_at	putative component of cytochrome	PETC	AT4G03280	-1.25	-0.33	-2.68	0.032854	0.088591	-4.47
247147_at	putative protein contains similarity t	GTE7	AT5G65630	-1.34	-0.43	-2.68	0.032863	0.088605	-4.47
262135_at	AP2 domain containing protein, put	RAP2.4	AT1G78080	1.53	0.61	2.68	0.032925	0.088762	-4.47
257248_at	hypothetical protein predicted by ge	---	AT3G24150	-1.34	-0.42	-2.68	0.032933	0.088773	-4.47
249229_at	putative protein similar to unknown	---	AT5G42090	1.24	0.31	2.68	0.032948	0.088798	-4.47
260695_at	RNA helicase, putative similar to G	ESP3	AT1G32490	1.18	0.24	2.68	0.03295	0.088798	-4.47
246301_at	calcium-dependent protein kinase	CPK13	AT3G51850	1.22	0.29	2.68	0.03296	0.088809	-4.47
258718_at	unknown protein ; supported by cD	---	AT3G09760	1.25	0.32	2.67	0.032962	0.088809	-4.47
265238_s_a	hypothetical protein	---	AT2G07696	1.38	0.46	2.67	0.032993	0.088883	-4.47
253918_at	putative protein ; supported by full-	PHOS34	AT4G27320	1.24	0.32	2.67	0.032997	0.088883	-4.47

262795_at	hypothetical protein predicted by ge	---	AT1G13130	-1.36	-0.44	-2.67	0.033031	0.088965	-4.47
261226_at	expansin S2 precursor, putative sir	ATEXPA11	AT1G20190	-4.05	-2.02	-2.67	0.033078	0.089079	-4.47
256711_at	unknown protein contains Pfam pro	---	AT3G30390	1.42	0.50	2.67	0.033085	0.089088	-4.47
254893_at	putative phospholipase D-gamma p	PLDGAMM	AT4G11830	-1.44	-0.53	-2.67	0.033092	0.089097	-4.47
252571_at	putative protein syntaxin 8 - Homo	SYP72	AT3G45280	-1.27	-0.34	-2.67	0.033109	0.089133	-4.47
261669_at	unknown protein similar to hypothet	---	AT1G18490	-1.18	-0.23	-2.67	0.033135	0.089193	-4.48
262872_at	hypothetical protein predicted by ge	---	AT1G64690	-1.36	-0.45	-2.67	0.033155	0.089235	-4.48
245057_at	En/Spm-like transposon protein rela	---	AT2G26490	-1.18	-0.23	-2.67	0.033164	0.08924	-4.48
263788_at	putative sarcosine oxidase	---	AT2G24580	1.42	0.50	2.67	0.033165	0.08924	-4.48
253097_at	cytochrome P450-like protein	CYP81D5	AT4G37320	1.79	0.84	2.67	0.033176	0.08926	-4.48
247151_at	unknown protein ;supported by full-	bHLH093	AT5G65640	-1.43	-0.52	-2.67	0.033185	0.089275	-4.48
247538_at	ABC family transporter - like protei	ATH16	AT5G61700	-1.18	-0.24	-2.67	0.033197	0.089295	-4.48
262338_at	Expressed protein ; supported by fu	---	AT1G64185	1.35	0.43	2.67	0.033233	0.08938	-4.48
253923_at	hypothetical protein Arabidopsis th	TOR1	AT4G27060	-1.24	-0.31	-2.67	0.033241	0.089384	-4.48
245453_at	disease resistance RPP5 like protei	---	AT4G16900	-1.24	-0.31	-2.67	0.033242	0.089384	-4.48
267472_at	putative basic blue protein (plantacy	ARPN	AT2G02850	-1.26	-0.33	-2.67	0.033251	0.089399	-4.48
251055_at	putative protein putative protein F4	---	AT5G01710	-1.22	-0.28	-2.67	0.033272	0.089445	-4.48
266096_at	putative vacuolar protein sorting-as	VCL1	AT2G38020	1.22	0.28	2.67	0.033276	0.089446	-4.48
260166_at	homeobox protein (GLABRA2) iden	GL2	AT1G79840	-1.20	-0.27	-2.67	0.033306	0.089508	-4.48
267578_at	Expressed protein ; supported by cl	---	AT2G30695	-1.51	-0.59	-2.67	0.033308	0.089508	-4.48
246997_at	putative protein similar to unknown	---	AT5G67390	1.37	0.46	2.67	0.033324	0.089542	-4.48
AFFX-Athal-	Arabidopsis thaliana /REF=U84969	---	---	1.18	0.24	2.67	0.033331	0.089549	-4.48
250903_at	putative protein coil protein PO22,	---	AT5G03600	-1.32	-0.41	-2.67	0.033385	0.089682	-4.48
248126_at	protein translation factor Sui1 homo	ROC5	AT4G34870	1.18	0.24	2.67	0.03339	0.089682	-4.48
248502_at	putative protein strong similarity to u	---	AT5G50450	-1.33	-0.41	-2.67	0.033392	0.089682	-4.48
267455_at	hypothetical protein predicted by ge	---	AT2G33760	1.31	0.39	2.67	0.033396	0.089682	-4.48
247470_at	putative protein various predicted p	---	AT5G62220	-1.95	-0.97	-2.67	0.033424	0.089748	-4.48
254339_at	glucosidase like protein furostanol	BGLU3	AT4G22100	-1.22	-0.29	-2.66	0.033442	0.089785	-4.48
247042_at	putative protein similar to unknown	---	AT5G67220	-1.22	-0.28	-2.66	0.033451	0.089798	-4.49
251160_at	inositol-1,4,5-trisphosphate 5-Phos	---	AT3G63240	-1.26	-0.33	-2.66	0.033462	0.089817	-4.49
261160_at	hypothetical protein	---	AT1G34480	-1.23	-0.30	-2.66	0.033469	0.089825	-4.49
266234_at	SKP1 interacting partner 3 (SKIP3)	SKIP3	AT2G02350	1.26	0.34	2.66	0.033473	0.089827	-4.49
267373_at	unknown protein	CID7	AT2G26280	1.24	0.31	2.66	0.033493	0.089869	-4.49
264012_at	hypothetical protein predicted by ge	---	AT2G21080	-1.30	-0.38	-2.66	0.033505	0.08989	-4.49
251054_at	receptor like protein kinase recepto	LECRKA4.	AT5G01540	2.71	1.44	2.66	0.033514	0.089905	-4.49

250893_at	putative protein predicted proteins -	EMB175	AT5G03795	-1.20	-0.27	-2.66	0.033519	0.089908	-4.49
264390_at	putative DNA-binding protein Conta	---	AT1G11950	1.29	0.37	2.66	0.033538	0.089938	-4.49
248330_at	putative protein contains similarity t	---	AT5G52810	1.52	0.60	2.66	0.033542	0.089938	-4.49
252369_at	putative protein deoxycytidylate de	---	AT3G48540	-1.22	-0.29	-2.66	0.033542	0.089938	-4.49
259614_at	dioxygenase, putative similar to dic	ATGA2OX4	AT1G47990	-1.25	-0.32	-2.66	0.033555	0.08996	-4.49
258330_at	unknown protein	ROPGEF13	AT3G16130	-1.25	-0.32	-2.66	0.033565	0.089978	-4.49
257105_at	unknown protein	---	AT3G15300	1.38	0.46	2.66	0.033573	0.089989	-4.49
266260_at	putative ATPase skd1 family; suppo	SKD1	AT2G27600	-1.20	-0.26	-2.66	0.033591	0.090025	-4.49
247141_at	putative protein strong similarity to u	---	AT5G65560	1.38	0.46	2.66	0.033605	0.090053	-4.49
260057_at	putative protein phosphatase 2C sir	---	AT1G78200	1.21	0.27	2.66	0.033612	0.090062	-4.49
258752_at	hypothetical protein predicted by ge	ATEXO70H	AT3G09520	1.55	0.63	2.66	0.033632	0.090105	-4.49
253764_s_a	protein kinase - like protein ADK1 p	ckl4	AT4G28860	1.29	0.37	2.66	0.033639	0.090113	-4.49
245121_at	60S ribosomal protein L7A ;suppor	---	AT2G47610	1.51	0.60	2.66	0.033689	0.090234	-4.49
262753_at	putative 3-deoxy-D-manno-2-octulo	ATKDSA2	AT1G16340	1.23	0.29	2.66	0.033695	0.09024	-4.49
245506_at	glutaredoxin homolog	---	AT4G15700	-1.61	-0.69	-2.66	0.033713	0.09027	-4.49
264408_at	hypothetical protein predicted by ge	FRS11	AT1G10240	1.22	0.29	2.66	0.033714	0.09027	-4.49
263806_at	hypothetical protein predicted by ge	---	AT2G04305	-1.61	-0.69	-2.66	0.033749	0.090345	-4.49
254681_at	putative protein HYA22 protein, Ho	---	AT4G18140	-1.40	-0.49	-2.66	0.03375	0.090345	-4.49
251350_at	cytochrome P450 monooxygenase	CYP76C7	AT3G61040	-1.40	-0.49	-2.66	0.03376	0.090362	-4.49
267110_at	hypothetical protein predicted by ge	---	AT2G14800	1.30	0.38	2.66	0.033791	0.090432	-4.50
254931_at	serine/threonine kinase-like protein	---	AT4G11460	-1.33	-0.41	-2.66	0.033796	0.090432	-4.50
257135_at	hypothetical protein similar to oxido	---	AT3G12900	-1.30	-0.38	-2.66	0.033798	0.090432	-4.50
267108_at	hypothetical protein predicted by ge	---	AT2G14830	-1.20	-0.26	-2.66	0.033818	0.090476	-4.50
266653_at	hypothetical protein predicted by ge	---	AT2G25740	1.26	0.33	2.66	0.033835	0.09051	-4.50
258722_at	putative pathogenesis-related prote	---	AT3G09590	-1.23	-0.30	-2.66	0.03384	0.090514	-4.50
263279_x_a	hypothetical protein predicted by ge	---	---	-1.25	-0.32	-2.66	0.033872	0.090588	-4.50
255268_at	putative protein ubiquitin, Tetrahym	---	AT4G05230	-1.26	-0.34	-2.66	0.033906	0.090668	-4.50
263115_at	Expressed protein ; supported by cl	---	AT1G03055	-1.48	-0.57	-2.65	0.033925	0.090709	-4.50
261219_at	hypothetical protein predicted by ge	---	AT1G19980	1.28	0.35	2.65	0.033932	0.090716	-4.50
252416_at	chromosome assembly protein hor	ATSMC2	AT3G47460	-1.17	-0.23	-2.65	0.033974	0.090816	-4.50
260261_at	unknown protein	---	AT1G68450	2.24	1.17	2.65	0.033979	0.09082	-4.50
265844_at	putative receptor-like protein kinase	FEI2	AT2G35620	-1.36	-0.44	-2.65	0.034014	0.090885	-4.50
246108_at	putative protein retinal glutamic aci	---	AT5G28630	-1.63	-0.71	-2.65	0.034014	0.090885	-4.50
256070_at	hypothetical protein predicted by ge	---	AT1G13730	1.22	0.28	2.65	0.034015	0.090885	-4.50
251220_at	cytochrome c oxidase subunit 5c-li	---	AT3G62400	1.35	0.43	2.65	0.034026	0.090904	-4.50

256432_at	hypothetical protein similar to hypot	---	AT3G10990	-1.20	-0.26	-2.65	0.034036	0.090904	-4.50
248310_at	putative protein similar to unknown	CPuORF14	AT5G52550	1.22	0.28	2.65	0.034037	0.090904	-4.50
247317_at	putative protein contains similarity t	anac103	AT5G64060	-1.26	-0.34	-2.65	0.034038	0.090904	-4.50
267393_at	similar to axi 1 protein from Nicotiar	---	AT2G44500	-1.48	-0.56	-2.65	0.034043	0.090905	-4.50
247723_at	protein phosphatase 2C - like ABA	---	AT5G59220	1.50	0.59	2.65	0.034072	0.09097	-4.50
252922_at	putative protein	---	AT4G39040	-1.23	-0.30	-2.65	0.034076	0.09097	-4.50
250520_at	putative protein contains similarity t	PEX1	AT5G08470	1.18	0.24	2.65	0.034079	0.09097	-4.50
253171_at	putative protein ;supported by full-l	---	AT4G35040	1.27	0.35	2.65	0.034107	0.09103	-4.51
252174_at	putative protein APRIL protein, Hor	---	AT3G50690	1.19	0.26	2.65	0.034109	0.09103	-4.51
250158_at	putative protein	---	AT5G15190	-1.45	-0.54	-2.65	0.034147	0.091101	-4.51
252458_at	putative protein various predicted p	---	AT3G47210	-1.22	-0.29	-2.65	0.034147	0.091101	-4.51
249830_at	dihydroorotate dehydrogenase prec	PYRD	AT5G23300	1.50	0.59	2.65	0.034148	0.091101	-4.51
260426_at	putative 40S ribosomal protein SA (	P40	AT1G72370	1.48	0.57	2.65	0.034152	0.091101	-4.51
248158_at	unknown protein	---	AT5G54440	-1.19	-0.25	-2.65	0.034156	0.091101	-4.51
265045_s_a	putative protein kinase, ADK1 dual	ADK1	AT1G03930	-1.50	-0.58	-2.65	0.034164	0.091113	-4.51
259482_at	receptor-like serine/threonine kinas	RKF2	AT1G19090	-1.25	-0.32	-2.65	0.03417	0.091113	-4.51
257644_at	unknown protein ;supported by full-	AOC3	AT3G25780	-2.34	-1.23	-2.65	0.034172	0.091113	-4.51
261310_at	hypothetical protein predicted by ge	PDE247	AT1G05750	1.31	0.39	2.65	0.034238	0.091273	-4.51
263430_at	unknown protein ;supported by full-	---	AT2G22270	-1.28	-0.36	-2.65	0.03424	0.091273	-4.51
259399_at	hypothetical protein similar to putati	---	AT1G17710	1.32	0.40	2.65	0.034296	0.091411	-4.51
257827_at	unknown protein contains Pfam pro	---	AT3G26630	1.43	0.52	2.65	0.0343	0.091411	-4.51
258012_at	unknown protein similar to GB:CAB	---	AT3G19310	-1.18	-0.24	-2.65	0.034329	0.091462	-4.51
249212_at	putative protein strong similarity to u	---	AT5G42690	-1.25	-0.32	-2.65	0.03433	0.091462	-4.51
261271_at	Expressed protein ; supported by fu	---	AT1G26795	-1.26	-0.33	-2.65	0.034331	0.091462	-4.51
259377_at	putative poly(A) binding protein sim	PAB6	AT3G16380	-1.32	-0.40	-2.65	0.034343	0.091483	-4.51
256308_s_a	ABC transporter, putative contains	ATMRP13	AT1G30410	1.43	0.51	2.65	0.034349	0.091487	-4.51
264230_at	hypothetical protein predicted by ge	---	AT1G67460	-1.22	-0.29	-2.65	0.034363	0.091507	-4.51
264539_at	hypothetical protein predicted by ge	---	AT1G55590	-1.26	-0.33	-2.65	0.034364	0.091507	-4.51
257719_at	unknown protein	AtALMT9	AT3G18440	1.34	0.42	2.65	0.034385	0.091551	-4.51
266990_at	putative ABC transporter	ATATH8	AT2G39190	-1.51	-0.59	-2.65	0.034389	0.091551	-4.51
254963_at	putative protein single strand DNA-	MTSSB	AT4G11060	1.27	0.34	2.64	0.034466	0.091736	-4.52
251004_at	putative protein predicted protein, A	---	AT5G02720	-1.24	-0.31	-2.64	0.034466	0.091736	-4.52
254460_at	putative protein sigma70 operon, S	ATRP1	AT4G21210	-1.32	-0.40	-2.64	0.034474	0.091745	-4.52
266978_at	putative disease resistance respons	---	AT2G39430	-1.23	-0.30	-2.64	0.034478	0.091745	-4.52
249554_at	CRS2 -like protein CRS2, Zea may	---	AT5G38290	-1.68	-0.75	-2.64	0.034511	0.091823	-4.52

245540_at	ABC transporter like protein	PDR2	AT4G15230	-1.95	-0.96	-2.64	0.034525	0.091849	-4.52
258405_at	unknown protein almost identical to	BSH	AT3G17590	1.36	0.45	2.64	0.034553	0.091914	-4.52
254840_at	hypothetical protein	---	AT4G11930	-1.34	-0.42	-2.64	0.034561	0.091924	-4.52
267577_at	unknown protein ; supported by cD	---	AT2G30710	-1.18	-0.24	-2.64	0.034591	0.091994	-4.52
257537_at	hypothetical protein predicted by ge	---	AT3G22350	-1.32	-0.40	-2.64	0.034608	0.09201	-4.52
261042_at	GTP-binding protein, putative simil	ATRABA3	AT1G01200	-1.28	-0.35	-2.64	0.03461	0.09201	-4.52
258493_at	Expressed protein ; supported by fu	---	AT3G02555	1.25	0.32	2.64	0.03461	0.09201	-4.52
245357_at	putative protein ;supported by full-le	---	AT4G17560	-1.33	-0.41	-2.64	0.034615	0.09201	-4.52
258198_at	putative transcription factor similar t	NF-YA6	AT3G14020	1.27	0.34	2.64	0.034618	0.09201	-4.52
251886_at	putative protein various predicted p	---	AT3G54260	-1.42	-0.51	-2.64	0.034643	0.092066	-4.52
263218_at	putative serine/threonine protein ki	---	AT1G30570	-1.32	-0.40	-2.64	0.034668	0.092122	-4.52
251453_at	putative protein hypothetical protei	---	AT3G60070	-1.24	-0.32	-2.64	0.034681	0.092141	-4.52
265637_at	unknown protein	ATCOAE	AT2G27490	1.23	0.30	2.64	0.034683	0.092141	-4.52
250756_at	putative protein strong similarity to u	ROPGEF5	AT5G05940	-1.24	-0.32	-2.64	0.034689	0.092146	-4.52
245516_at	hypothetical protein	---	AT4G15820	1.29	0.37	2.64	0.034726	0.092234	-4.52
257458_at	hypothetical protein predicted by ge	---	AT2G05400	-1.18	-0.24	-2.64	0.034756	0.092302	-4.52
258078_at	unknown protein ;supported by full-	---	AT3G25870	1.56	0.64	2.64	0.034774	0.092332	-4.53
250617_at	Mei2-like protein Mei2-like protein -	AML4	AT5G07290	1.28	0.35	2.64	0.034775	0.092332	-4.53
264640_at	pollen allergen, putative similar to p	ATEXPB2	AT1G65680	-1.39	-0.47	-2.64	0.034788	0.092335	-4.53
267213_at	60S ribosomal protein L7 ;supporte	---	AT2G44120	1.51	0.59	2.64	0.034792	0.092335	-4.53
250828_at	unknown protein ; supported by cDI	---	AT5G05250	-3.13	-1.65	-2.64	0.034794	0.092335	-4.53
257150_at	unknown protein ;supported by full-	---	AT3G27230	-1.30	-0.38	-2.64	0.034795	0.092335	-4.53
249220_at	putative protein strong similarity to u	---	AT5G42420	-1.27	-0.35	-2.64	0.034797	0.092335	-4.53
258902_at	putative pyruvate dehydrogenase k	PDK	AT3G06483	-1.20	-0.26	-2.64	0.034827	0.092404	-4.53
250887_at	putative protein	---	---	1.26	0.33	2.64	0.034842	0.092434	-4.53
255832_at	hypothetical protein predicted by ge	---	AT2G33360	1.34	0.42	2.64	0.034848	0.09244	-4.53
246745_at	60S ribosomal protein L22 - like rib	---	AT5G27770	1.46	0.54	2.64	0.034869	0.092479	-4.53
256520_at	hypothetical protein	CYP96A14	AT1G66030	-1.22	-0.29	-2.64	0.034871	0.092479	-4.53
247288_at	non-phototropic hypocotyl 3 (gb|AA	NPH3	AT5G64330	-1.96	-0.97	-2.64	0.034881	0.092495	-4.53
245397_at	auxin-responsive protein IAA1 ;sup	IAA1	AT4G14560	-1.81	-0.86	-2.63	0.034891	0.092508	-4.53
257067_at	unknown protein contains Pfam pro	CYP77A5P	AT3G18270	1.27	0.34	2.63	0.034896	0.092508	-4.53
264664_at	putative elongation factor similar to	---	AT1G09660	1.37	0.46	2.63	0.034899	0.092508	-4.53
246574_at	copper amine oxidase, putative sim	---	AT1G31670	-1.49	-0.58	-2.63	0.034914	0.092539	-4.53
247334_at	cdc2-like protein kinase-like protein	CDKE;1	AT5G63610	-1.18	-0.23	-2.63	0.034949	0.092621	-4.53
258383_at	hypothetical protein predicted by ge	---	AT3G15440	1.60	0.68	2.63	0.034961	0.092635	-4.53

260549_at	Expressed protein ; supported by fu	---	AT2G43535	-1.90	-0.92	-2.63	0.034962	0.092635	-4.53
264638_at	flowering signals mediating protein	FT	AT1G65480	1.79	0.84	2.63	0.034969	0.092642	-4.53
265708_at	unknown protein member of a large	---	AT2G03380	1.22	0.28	2.63	0.034989	0.092685	-4.53
248644_at	disease resistance protein-like	---	AT5G49140	-1.18	-0.24	-2.63	0.034997	0.092693	-4.53
253635_at	putative protein hypothetical protei	---	AT4G30620	1.20	0.27	2.63	0.035031	0.092772	-4.53
247553_at	MAD box containing protein NAP1-	AGL8	AT5G60910	1.50	0.59	2.63	0.035038	0.092774	-4.53
253102_at	Expressed protein ; supported by fu	---	AT4G36105	-1.35	-0.43	-2.63	0.035039	0.092774	-4.53
247507_at	putative protein predicted protein,	APO3	AT5G61930	1.52	0.60	2.63	0.03505	0.092791	-4.53
262273_at	hypothetical protein predicted by ge	---	AT1G68730	-1.20	-0.27	-2.63	0.03506	0.092805	-4.53
245880_at	putative hydrolase EtbD1 - Rhodoc	---	AT5G09430	-1.31	-0.39	-2.63	0.035063	0.092805	-4.53
258933_at	unknown protein similar to unknow	---	AT3G09980	-1.24	-0.31	-2.63	0.03509	0.09286	-4.53
254537_at	putative protein chitinase / lysozym	---	AT4G19730	-1.25	-0.32	-2.63	0.035092	0.09286	-4.53
265577_at	unknown protein	---	AT2G20100	1.21	0.28	2.63	0.035097	0.092861	-4.54
260247_at	putative disease resistance protein	AtRLP16	AT1G74200	-1.19	-0.26	-2.63	0.035106	0.092874	-4.54
255303_at	predicted protein of unknown functio	DER2.2	AT4G04860	1.24	0.31	2.63	0.03512	0.092897	-4.54
247762_at	cell wall protein	---	AT5G59170	-1.64	-0.71	-2.63	0.035122	0.092897	-4.54
259003_at	hypothetical protein similar to hypo	---	AT3G02010	1.37	0.46	2.63	0.035131	0.092909	-4.54
267434_at	3-beta-hydroxysteroid dehydrogena	AT3BETAH	AT2G26260	-1.28	-0.35	-2.63	0.035141	0.092923	-4.54
261541_at	unknown protein contains Pfam pro	---	AT1G63600	-1.25	-0.32	-2.63	0.035169	0.092989	-4.54
251712_at	putative protein non-consensus TT	---	AT3G56120	1.19	0.25	2.63	0.035176	0.092996	-4.54
245216_at	Expressed protein non-consensus (	IMPA-2	AT4G16143	-1.17	-0.23	-2.63	0.03521	0.093055	-4.54
250309_at	putative protein similar to unknown	---	AT5G12220	1.33	0.41	2.63	0.035211	0.093055	-4.54
247253_at	beta-1,3-glucanase	---	AT5G64790	1.86	0.90	2.63	0.035211	0.093055	-4.54
245778_at	RNA-binding glycine-rich protein, p	---	AT1G73530	-1.36	-0.44	-2.63	0.035257	0.093165	-4.54
263400_s_a	40S ribosomal protein; contains C-t	---	AT2G31610	1.28	0.36	2.63	0.035271	0.093191	-4.54
252383_at	ABC-type transport protein-like pro	ATH6	AT3G47780	1.81	0.85	2.63	0.035275	0.093191	-4.54
264160_at	hypothetical protein predicted by ge	---	AT1G65450	-1.28	-0.36	-2.63	0.03529	0.093209	-4.54
263797_at	putative WRKY-type DNA binding p	WRKY17	AT2G24570	2.09	1.06	2.63	0.03529	0.093209	-4.54
262778_at	hypothetical protein predicted by ge	---	AT1G13050	1.19	0.25	2.63	0.035294	0.093209	-4.54
253852_at	putative protein diacylglycerol kina	---	AT4G28130	-1.21	-0.27	-2.63	0.035315	0.093255	-4.54
257083_s_a	non-race specific disease resistanc	---	AT3G20590	1.47	0.56	2.63	0.035322	0.093264	-4.54
253995_at	Col-0 casein kinase I-like protein ;	CK1	AT4G26100	-1.32	-0.40	-2.63	0.035343	0.093281	-4.54
263882_at	putative ribonucleoside-diphosphate	RNR1	AT2G21790	1.65	0.72	2.63	0.035344	0.093281	-4.54
261484_at	E2, ubiquitin-conjugating enzyme 1	UBC1	AT1G14400	1.21	0.28	2.63	0.035344	0.093281	-4.54
266546_at	putative AT-hook DNA-binding prote	---	AT2G35270	-1.20	-0.26	-2.63	0.035346	0.093281	-4.54

259486_at	embryo abundance protein (EMB2(	---	AT1G15710	-1.27	-0.34	-2.63	0.035351	0.093285	-4.54
261372_at	zinc finger protein, putative similar	---	AT1G53010	-1.27	-0.35	-2.62	0.035393	0.093386	-4.54
262840_at	linker histone protein, putative cont	HMGA	AT1G14900	1.26	0.34	2.62	0.035405	0.093406	-4.54
267568_at	hypothetical protein predicted by ge	---	AT2G30780	1.25	0.32	2.62	0.035425	0.093449	-4.54
252449_at	FtsH protease, putative contains si	ftsh7	AT3G47060	1.18	0.24	2.62	0.035439	0.093474	-4.55
261162_at	hypothetical protein predicted by ge	---	AT1G34440	-1.28	-0.35	-2.62	0.035443	0.093474	-4.55
255042_at	putative protein hypothetical protei	---	AT4G09630	-1.23	-0.30	-2.62	0.03547	0.093533	-4.55
267598_at	hypothetical protein predicted by ge	---	AT2G33010	-1.29	-0.36	-2.62	0.035492	0.093573	-4.55
249505_at	putative protein	---	---	-1.53	-0.61	-2.62	0.035493	0.093573	-4.55
262306_s_a	asparaginyl-tRNA synthetase(SYN	SYNC3	AT1G70980	1.31	0.38	2.62	0.035523	0.093632	-4.55
245324_at	lactate dehydrogenase (LDH1) ;sup	---	AT4G17260	-1.66	-0.73	-2.62	0.035524	0.093632	-4.55
261589_at	hypothetical protein predicted by ge	---	AT1G01690	-1.29	-0.37	-2.62	0.035535	0.093646	-4.55
265479_at	hypothetical protein ;supported by	---	AT2G15760	2.60	1.38	2.62	0.035537	0.093646	-4.55
253564_at	protein kinase - like protein protein	---	AT4G31170	-1.25	-0.32	-2.62	0.035553	0.093675	-4.55
245350_at	nuclear antigen homolog ;supportec	---	AT4G16830	1.27	0.34	2.62	0.035556	0.093675	-4.55
249951_at	S-adenosyl-L-methionine decarbox	BUD2	AT5G18930	-1.39	-0.48	-2.62	0.035619	0.093829	-4.55
261816_at	unknown protein	CHX6B	AT1G08135	-1.22	-0.29	-2.62	0.035625	0.093831	-4.55
266638_at	putative fibrillin ;supported by full-le	---	AT2G35490	-1.25	-0.32	-2.62	0.035628	0.093831	-4.55
263092_at	hypothetical protein predicted by ge	---	AT2G16210	-1.20	-0.26	-2.62	0.035649	0.093858	-4.55
253673_at	putative protein putative phosphata	---	AT4G29530	-1.26	-0.33	-2.62	0.035649	0.093858	-4.55
253560_at	putative protein KIAA0800 protein,	DCAF1	AT4G31160	1.25	0.32	2.62	0.03565	0.093858	-4.55
265952_at	unknown protein supported by cDN	---	AT2G37478	1.23	0.30	2.62	0.035659	0.093869	-4.55
262121_at	endo-1,4-beta-glucanase precurso	ATCEL2	AT1G02800	-1.29	-0.36	-2.62	0.035676	0.093903	-4.55
248691_at	putative protein strong similarity to u	---	AT5G48310	-1.22	-0.29	-2.62	0.035698	0.093951	-4.55
252086_at	hypothetical protein	---	AT3G52030	1.24	0.31	2.62	0.035717	0.09399	-4.55
250845_at	rna binding protein - like rna bindin	---	AT5G04600	1.36	0.45	2.62	0.035757	0.094085	-4.55
245138_at	unknown protein predicted by gens	AFO	AT2G45190	-1.42	-0.50	-2.62	0.03582	0.094238	-4.56
266081_at	putative protein kinase contains a p	---	AT2G37840	-1.22	-0.28	-2.62	0.035827	0.094248	-4.56
250701_at	bZIP transcription factor-like protein	---	AT5G06839	-1.26	-0.33	-2.62	0.035837	0.094263	-4.56
256169_at	receptor protein kinase, putative co	---	AT1G51800	2.52	1.33	2.62	0.035843	0.094266	-4.56
265304_at	putative AP2 domain transcription fa	---	AT2G20350	-1.19	-0.25	-2.62	0.035896	0.094386	-4.56
267508_at	hypothetical protein predicted by ge	---	AT2G45700	1.22	0.29	2.62	0.035896	0.094386	-4.56
262914_at	auxin response factor 1 identical to	ARF1	AT1G59750	-1.25	-0.32	-2.61	0.03593	0.094453	-4.56
247437_at	AtHVA22b-like protein ; supported	HVA22B	AT5G62490	-1.29	-0.36	-2.61	0.035933	0.094453	-4.56
250885_at	ABC transporter -like protein ABC-	ATH12	AT5G03910	-1.21	-0.28	-2.61	0.035934	0.094453	-4.56

260033_at	putative mutT protein contains Pfar	ATNUDX1	AT1G68760	-1.27	-0.35	-2.61	0.035947	0.094466	-4.56
262883_at	ammonium transporter, puitative si	ATAMT1;2	AT1G64780	-1.44	-0.52	-2.61	0.035947	0.094466	-4.56
253759_at	AIM1 protein ; supported by cDNA:	AIM1	AT4G29010	1.19	0.25	2.61	0.035986	0.094557	-4.56
263742_at	predicted protein	---	AT2G20625	-1.20	-0.27	-2.61	0.035993	0.094564	-4.56
266600_at	putative mitogen-activated protein k	MPK12	AT2G46070	1.41	0.50	2.61	0.036018	0.094618	-4.56
260389_at	Expressed protein ; supported by fu	---	AT1G74055	-1.37	-0.45	-2.61	0.036031	0.094641	-4.56
261051_at	cytochrome P450, putative similar t	CYP703A2	AT1G01280	-1.22	-0.28	-2.61	0.036036	0.094645	-4.56
256518_at	hypothetical protein	---	AT1G66080	-1.28	-0.36	-2.61	0.036044	0.094647	-4.56
261958_at	peptide transporter, putative predic	---	AT1G64500	1.53	0.61	2.61	0.036046	0.094647	-4.56
246425_at	cellulose synthase catalytic subunit	IRX3	AT5G17420	-1.60	-0.68	-2.61	0.036057	0.094666	-4.56
262516_at	putative glutathione transferase On	ATGSTU26	AT1G17190	-1.24	-0.32	-2.61	0.036064	0.094668	-4.56
247890_at	cyclic nucleotide and calmodulin-reg	ATCNGC5	AT5G57940	-1.28	-0.35	-2.61	0.036067	0.094668	-4.56
255051_at	RNA-directed DNA polymerase -lik	---	---	-1.39	-0.47	-2.61	0.03607	0.094668	-4.56
262227_s_a	26S proteasome ATPase subunit, p	---	AT1G53750	1.20	0.26	2.61	0.036079	0.094679	-4.56
250101_at	putative protein contains similarity t	BGLU2	AT5G16580	-1.47	-0.56	-2.61	0.036089	0.094695	-4.56
258972_at	hypothetical protein predicted by ge	---	AT3G01920	-1.40	-0.48	-2.61	0.036094	0.094697	-4.56
264354_s_a	unknown protein similar to Lambda-	---	AT1G03200	1.24	0.31	2.61	0.03616	0.09486	-4.57
256533_at	unknown protein	---	AT1G33250	-1.34	-0.42	-2.61	0.03619	0.094927	-4.57
247191_at	homeobox-leucine zipper protein AT	ATHB5	AT5G65310	-1.44	-0.52	-2.61	0.036212	0.094976	-4.57
260106_at	unknown protein ;supported by full-	---	AT1G35420	-1.24	-0.31	-2.61	0.036229	0.095008	-4.57
260988_at	MAP3K alpha protein kinase, putat	MAP3KA	AT1G53570	-1.38	-0.46	-2.61	0.036254	0.095055	-4.57
246005_at	lipoic acid synthase - like protein lip	---	AT5G08415	1.35	0.43	2.61	0.036255	0.095055	-4.57
259039_at	unknown protein ;supported by full-	---	AT3G09250	1.22	0.29	2.61	0.03626	0.095056	-4.57
255291_at	putative calcium dependent protein	CPK27	AT4G04700	-1.26	-0.33	-2.61	0.036281	0.095101	-4.57
261987_at	hypothetical protein predicted by ge	---	AT1G33710	-1.17	-0.23	-2.61	0.036298	0.095135	-4.57
257600_at	hypothetical protein predicted by ge	CLE41	AT3G24770	-1.29	-0.37	-2.61	0.036319	0.095178	-4.57
256909_at	hypothetical protein predicted by ge	---	AT3G24060	-1.28	-0.36	-2.61	0.036324	0.095181	-4.57
266340_at	similar to axi 1 protein from Nicotiar	---	AT2G01480	1.18	0.24	2.61	0.036334	0.095195	-4.57
246018_at	Expressed protein ; supported by fu	---	AT5G10695	-1.95	-0.96	-2.61	0.03634	0.095201	-4.57
258125_s_a	hypothetical protein contains Pfam	---	AT3G23510	-1.71	-0.78	-2.61	0.036373	0.095276	-4.57
250683_x_a	putative protein similar to unknown	---	AT5G06640	-1.37	-0.45	-2.61	0.036381	0.095286	-4.57
253845_at	putative protein myosin heavy chai	---	AT4G27980	-1.17	-0.23	-2.60	0.03642	0.095379	-4.57
260970_at	unknown protein	---	AT1G53640	-1.27	-0.34	-2.60	0.036426	0.095381	-4.57
267504_at	unknown protein ;supported by full-	---	AT2G45530	1.29	0.37	2.60	0.036457	0.095453	-4.57
265989_at	unknown protein	---	AT2G24260	1.21	0.28	2.60	0.036467	0.095469	-4.57

257484_at	unknown protein	---	AT1G01650	-1.20	-0.27	-2.60	0.036491	0.095521	-4.58
261224_at	subtilisin-like serine protease simila	ATSBT5.2	AT1G20160	-1.74	-0.80	-2.60	0.036505	0.095544	-4.58
264737_at	unknown protein	---	AT1G62210	-1.33	-0.42	-2.60	0.036538	0.095622	-4.58
257221_at	MYB family transcription factor (GL	ATMYB0	AT3G27920	-1.31	-0.39	-2.60	0.036547	0.095633	-4.58
254693_at	bHLH protein - like bHLH protein, A	---	AT4G17880	1.91	0.94	2.60	0.036585	0.095713	-4.58
266799_at	unknown protein	ATPSK2	AT2G22860	-1.50	-0.59	-2.60	0.036586	0.095713	-4.58
248501_at	golgi SNARE protein	MEMB12	AT5G50440	-1.28	-0.36	-2.60	0.036596	0.095729	-4.58
249176_at	thioredoxin (clone GIF1) (pir||S5811	ATTRX3	AT5G42980	1.19	0.25	2.60	0.036608	0.095749	-4.58
250359_at	putative protein predicted proteins,	---	AT5G11350	1.21	0.28	2.60	0.036619	0.095757	-4.58
266047_at	unknown protein	---	AT2G40800	-1.29	-0.37	-2.60	0.036619	0.095757	-4.58
260730_at	lipoamide dehydrogenase precurso	mtLPD1	AT1G48030	-1.33	-0.41	-2.60	0.036645	0.095807	-4.58
250159_at	40S ribosomal protein - like 40S rib	---	AT5G15200	1.37	0.46	2.60	0.036647	0.095807	-4.58
248110_at	wax synthase-like protein	---	AT5G55320	-1.18	-0.24	-2.60	0.036654	0.095816	-4.58
267193_at	hypothetical protein predicted by gr	---	AT2G30900	-1.23	-0.29	-2.60	0.036673	0.095853	-4.58
248499_at	putative protein contains similarity t	PAP27	AT5G50400	1.19	0.25	2.60	0.036711	0.095935	-4.58
258786_at	putative syntaxin contains Pfam pro	SYP121	AT3G11820	-1.60	-0.67	-2.60	0.036713	0.095935	-4.58
248613_at	phytoene dehydrogenase-like	---	AT5G49555	-1.34	-0.42	-2.60	0.036721	0.095946	-4.58
258521_at	ribosomal protein L29, putative sim	---	AT3G06680	1.68	0.75	2.60	0.03674	0.095983	-4.58
259725_at	Expressed protein ; supported by fu	---	AT1G61065	1.39	0.48	2.60	0.036754	0.096002	-4.58
255518_at	putative pectinesterase similar to se	---	AT4G02300	-1.24	-0.32	-2.60	0.036755	0.096002	-4.58
253326_at	putative protein polygalacturonase	---	AT4G33440	-1.24	-0.31	-2.60	0.036784	0.096056	-4.58
257602_at	hydrolase, putative similar to Metal	---	AT3G13800	-1.23	-0.29	-2.60	0.036785	0.096056	-4.58
248361_at	putative protein similar to unknown	EDA41	AT5G52460	-1.25	-0.33	-2.60	0.036789	0.096056	-4.58
254486_at	putative protein crp1 protein, Zea m	---	AT4G20770	1.26	0.33	2.60	0.036819	0.096125	-4.58
263769_at	5S ribosomal RNA	---	---	-1.27	-0.34	-2.60	0.036824	0.096127	-4.58
264550_at	hypothetical protein predicted by ge	---	AT1G09450	-1.40	-0.49	-2.60	0.036841	0.09616	-4.59
245907_at	ubiquitin-like protein ubiquitins - diff	---	AT5G09340	1.18	0.24	2.60	0.036848	0.096167	-4.59
258318_at	unknown protein	---	AT3G22680	1.44	0.52	2.60	0.036867	0.096206	-4.59
253239_at	putative serine/threonine protein ki	---	AT4G34500	-1.39	-0.47	-2.60	0.036872	0.096207	-4.59
246901_at	putative protein Leaf protein, Pharb	---	AT5G25630	1.22	0.28	2.60	0.036881	0.096221	-4.59
266854_at	putative C2H2-type zinc finger prote	---	AT2G26940	-1.31	-0.39	-2.60	0.036898	0.096254	-4.59
259090_at	putative ribosomal protein s19 or s2	---	AT3G04920	1.43	0.52	2.60	0.036914	0.096283	-4.59
260252_at	putative mitochondrial carrier protei	---	AT1G74240	1.34	0.42	2.60	0.036921	0.096283	-4.59
259331_at	putative auxin-induced protein simil	---	AT3G03840	-1.32	-0.40	-2.60	0.036922	0.096283	-4.59
258894_at	putative disease resistance protein	AtRLP32	AT3G05650	-1.62	-0.69	-2.59	0.036959	0.096369	-4.59

247232_at	ABC transporter-like	ATH13	AT5G64940	-1.31	-0.39	-2.59	0.036985	0.096426	-4.59
252491_at	disease resistance protein RPP8-li	---	AT3G46730	1.18	0.24	2.59	0.037005	0.096466	-4.59
248939_at	tyrosine-specific protein phosphatas	---	AT5G45790	1.24	0.31	2.59	0.03702	0.096495	-4.59
261390_at	hypothetical protein similar to hypot	---	AT1G35040	-1.20	-0.26	-2.59	0.03709	0.096665	-4.59
261612_at	fucosyltransferase c3 protein, puta	FUT12	AT1G49710	1.21	0.28	2.59	0.03716	0.096839	-4.59
250850_at	putative protein ; supported by cDN	---	AT5G04550	-1.29	-0.37	-2.59	0.037172	0.096859	-4.59
263440_at	unknown protein predicted by gens	ATEXO70H	AT2G28640	-1.17	-0.23	-2.59	0.037181	0.096865	-4.59
247652_at	histone H2B - like protein histone H	HTB4	AT5G59910	1.29	0.37	2.59	0.037185	0.096865	-4.59
259774_at	plasma membrane associated prot	---	AT1G29520	-1.36	-0.44	-2.59	0.037187	0.096865	-4.59
247068_at	putative protein similar to unknown	---	AT5G66800	-1.27	-0.34	-2.59	0.037217	0.096919	-4.60
250263_at	putative protein	---	AT5G13470	1.28	0.36	2.59	0.037219	0.096919	-4.60
267470_at	cinnamate-4-hydroxylase molecular	C4H	AT2G30490	1.79	0.84	2.59	0.037221	0.096919	-4.60
261681_at	hypothetical protein similar to hypot	---	AT1G47340	1.40	0.48	2.59	0.037238	0.096951	-4.60
251798_at	putative RNA binding protein Serin	SCL30	AT3G55460	1.22	0.29	2.59	0.037242	0.096952	-4.60
265531_at	hypothetical protein predicted by ge	AtGRF6	AT2G06200	-1.23	-0.30	-2.59	0.037267	0.097005	-4.60
251638_at	40S ribosomal protein S2 homolog	---	AT3G57490	1.52	0.60	2.59	0.037276	0.097011	-4.60
257081_at	RING zinc finger protein contains F	---	AT3G30460	-1.21	-0.28	-2.59	0.037278	0.097011	-4.60
262095_at	hypothetical protein predicted by ge	---	AT1G56090	1.32	0.40	2.59	0.037285	0.097016	-4.60
259283_at	putative transcription factor similar t	MYB65	AT3G11440	-1.24	-0.31	-2.59	0.037288	0.097016	-4.60
248223_at	unknown protein ; supported by cD	---	AT5G53620	1.17	0.23	2.59	0.037304	0.097045	-4.60
263519_at	40S ribosomal protein S25 ; suppo	---	AT2G21580	1.49	0.58	2.59	0.03731	0.097045	-4.60
253647_at	putative protein KIAA0210 gene, H	---	AT4G29950	-1.28	-0.35	-2.59	0.037312	0.097045	-4.60
251056_at	putative protein KIAA1303 protein -	RAPTOR2	AT5G01770	1.30	0.38	2.59	0.037353	0.09714	-4.60
266748_at	putative squamosa-promoter bindin	SPL1	AT2G47070	-1.40	-0.48	-2.59	0.037391	0.097228	-4.60
266488_at	putative pectinesterase ; supported	---	AT2G47670	1.38	0.47	2.59	0.037397	0.097234	-4.60
253321_at	putative protein prolyl 4-hydroxylas	---	AT4G33910	1.21	0.28	2.59	0.037412	0.097261	-4.60
246003_at	chloroplast Cpn21 protein ; suppor	CPN20	AT5G20720	1.26	0.33	2.59	0.037445	0.097336	-4.60
262866_at	cytochrome p450, putative similar t	CYP89A6	AT1G64940	-1.19	-0.25	-2.59	0.037461	0.097365	-4.60
255419_at	putative receptor kinase	---	AT4G03230	-1.25	-0.32	-2.58	0.037468	0.097373	-4.60
251586_at	zinc finger-like protein several zinc f	GIS	AT3G58070	-1.31	-0.39	-2.58	0.037513	0.097479	-4.60
249596_s_a	glucuronosyl transferase-like prote	---	AT5G37950	-1.39	-0.48	-2.58	0.037518	0.097482	-4.60
266607_at	hypothetical protein predicted by ge	---	AT2G46300	-1.26	-0.33	-2.58	0.037527	0.097494	-4.60
255387_at	putative nodulin similar to M. trunca	---	AT4G03630	-1.30	-0.38	-2.58	0.037536	0.097496	-4.60
252206_at	centrin	ATCEN2	AT3G50360	-1.24	-0.31	-2.58	0.037537	0.097496	-4.60
251396_at	transketolase - like protein transket	---	AT3G60750	1.21	0.28	2.58	0.03759	0.097613	-4.61

255861_at	putative vacuolar sorting receptor	---	AT2G30290	-1.19	-0.25	-2.58	0.03759	0.097613	-4.61
260189_at	putative urease similar to urease GI	URE	AT1G67550	1.27	0.35	2.58	0.037601	0.097631	-4.61
262695_at	vacuolor sorting protein 35, putative	VPS35B	AT1G75850	1.21	0.27	2.58	0.037613	0.097645	-4.61
257994_at	unknown protein	---	AT3G19920	1.34	0.43	2.58	0.037615	0.097645	-4.61
257651_at	polygalacturonase, putative similar	---	AT3G16850	-1.51	-0.60	-2.58	0.037679	0.097786	-4.61
263659_at	unknown protein EST gb|ATTS5672	---	AT1G04470	-1.23	-0.30	-2.58	0.037683	0.097786	-4.61
260410_at	putative peptide transporter similar	---	AT1G69870	-1.51	-0.59	-2.58	0.037684	0.097786	-4.61
264540_at	hypothetical protein predicted by ge	---	AT1G55630	1.24	0.31	2.58	0.037687	0.097786	-4.61
264728_at	unknown protein location of EST gb	---	AT1G22850	-1.28	-0.35	-2.58	0.037709	0.097832	-4.61
248730_at	putative protein similar to unknown	---	AT5G48050	-1.30	-0.38	-2.58	0.037722	0.097854	-4.61
252097_at	putative protein hypothetical protein	---	AT3G51090	1.20	0.27	2.58	0.037747	0.097909	-4.61
256590_at	recombination signal sequence rec	ATHMG	AT3G28730	1.31	0.39	2.58	0.037768	0.097951	-4.61
262742_at	GTP-binding protein, putative simil	AtRABA1i	AT1G28550	-1.21	-0.28	-2.58	0.037824	0.098086	-4.61
267054_at	hypothetical protein	---	AT2G38370	1.40	0.49	2.58	0.037858	0.098164	-4.61
266448_s_a	unknown protein ;supported by full-	---	AT2G43210	-1.22	-0.28	-2.58	0.037876	0.098199	-4.61
266679_at	putative helicase ; supported by cD	---	AT2G03270	1.25	0.32	2.58	0.037883	0.098205	-4.61
251944_at	ABC transporter -like protein breas	---	AT3G53510	-1.32	-0.40	-2.58	0.037898	0.098234	-4.61
257655_at	unknown protein contains Pfam pro	---	AT3G13350	1.53	0.62	2.58	0.037902	0.098234	-4.61
265705_at	unknown protein related to MO25 (r	---	AT2G03410	1.75	0.80	2.58	0.037915	0.098255	-4.61
248312_at	unknown protein	---	AT5G52580	-1.25	-0.33	-2.58	0.037938	0.098304	-4.62
258829_at	putative Sec24-like COPII protein s	---	AT3G07100	1.17	0.23	2.58	0.037982	0.098391	-4.62
245824_at	hypothetical protein predicted by ge	ATPUP17	AT1G57943	-1.37	-0.45	-2.58	0.037988	0.098391	-4.62
257524_at	hypothetical protein similar to putati	DEL3	AT3G01330	-1.25	-0.33	-2.58	0.037989	0.098391	-4.62
249969_at	putative protein glycine-rich protein	---	AT5G19090	-1.47	-0.55	-2.58	0.037989	0.098391	-4.62
261760_at	hypothetical protein similar to putati	---	AT1G15600	-1.33	-0.41	-2.58	0.037993	0.098391	-4.62
259760_at	hypothetical protein similar to GB:A	---	AT1G77580	-1.18	-0.24	-2.57	0.038022	0.098447	-4.62
267594_at	hypothetical protein predicted by ge	---	AT2G33000	-1.21	-0.28	-2.57	0.038024	0.098447	-4.62
266772_s_a	predicted by genefinder	---	AT2G03020	-1.38	-0.47	-2.57	0.038028	0.098447	-4.62
252934_at	Inositol monophosphatase - like pro	IMPL2	AT4G39120	1.31	0.39	2.57	0.038039	0.098453	-4.62
245578_at	RNA helicase like protein	ATSUV3	AT4G14790	1.29	0.36	2.57	0.038039	0.098453	-4.62
257270_at	unknown protein ; supported by ful	---	AT3G28140	1.19	0.25	2.57	0.038044	0.098454	-4.62
248148_at	putative protein similar to unknown	---	AT5G54930	1.39	0.47	2.57	0.038052	0.098464	-4.62
250774_at	ATP-dependent RNA helicase-like p	---	AT5G05450	-1.33	-0.41	-2.57	0.038069	0.098498	-4.62
246151_at	putative protein predicted proteins f	---	AT5G19950	1.26	0.33	2.57	0.038074	0.0985	-4.62
262820_at	putative obtusifoliol 14-alpha deme	CYP51G1	AT1G11680	1.22	0.29	2.57	0.038078	0.0985	-4.62

246522_at	bZIP DNA-binding protein-like puta	AtbZIP3	AT5G15830	1.71	0.78	2.57	0.038102	0.098545	-4.62
247730_at	glucuronosyl transferase - like prot	UGT76E1	AT5G59580	-1.41	-0.50	-2.57	0.038105	0.098545	-4.62
262673_at	unknown protein	EMB1793	AT1G76060	1.23	0.30	2.57	0.038111	0.098551	-4.62
266982_at	unknown protein ; supported by cD	---	AT2G39500	1.30	0.38	2.57	0.038136	0.098603	-4.62
250412_at	putative protein synaptobrevin-like	ATVAMP71	AT5G11150	1.30	0.38	2.57	0.038158	0.098641	-4.62
246026_at	putative protein predicted protein - (	---	AT5G21070	-1.22	-0.29	-2.57	0.038159	0.098641	-4.62
262880_at	hypothetical protein contains similar	---	AT1G64880	1.68	0.75	2.57	0.038169	0.098654	-4.62
262867_at	hypothetical protein predicted by ge	---	AT1G64960	1.24	0.31	2.57	0.038196	0.09871	-4.62
258145_at	integral membrane protein, putative	---	AT3G18200	-1.28	-0.35	-2.57	0.038199	0.09871	-4.62
253494_at	putative protein	---	AT4G31830	-1.23	-0.30	-2.57	0.03822	0.098751	-4.62
254894_at	putative phospholipase D-gamma p	PLDGAMM	AT4G11840	1.36	0.45	2.57	0.038223	0.098751	-4.62
248957_at	26S proteasome subunit-like protein	---	AT5G45620	-1.22	-0.29	-2.57	0.038228	0.098751	-4.62
259496_at	hypothetical protein predicted by ge	---	AT1G15900	-1.22	-0.29	-2.57	0.038234	0.098755	-4.62
261443_at	glutaredoxin, putative similar to glu	GRX480	AT1G28480	1.87	0.90	2.57	0.038263	0.098819	-4.62
247990_at	unknown protein ;supported by full-	---	AT5G56360	1.20	0.26	2.57	0.038277	0.098845	-4.62
265480_at	similar to cold acclimation protein W	COR413-P	AT2G15970	-1.42	-0.51	-2.57	0.038322	0.098949	-4.63
247534_at	pyrophosphate-dependent phospho	PFK4	AT5G61580	1.19	0.25	2.57	0.038336	0.098975	-4.63
256052_at	spliceosomal protein (U2B), putativ	---	AT1G06960	-1.24	-0.31	-2.57	0.03834	0.098975	-4.63
256910_at	hypothetical protein	---	AT3G24080	1.29	0.36	2.57	0.038345	0.098977	-4.63
253107_at	putative protein predicted proteins,	---	AT4G35880	-1.30	-0.38	-2.57	0.038363	0.09901	-4.63
248584_at	putative protein similar to unknown	---	AT5G49960	-1.38	-0.46	-2.57	0.038374	0.09902	-4.63
265467_at	putative receptor-like protein kinase	---	AT2G37050	-1.36	-0.44	-2.57	0.038378	0.09902	-4.63
249063_at	NBD-like protein (gb|AAD20643.1)	POP1	AT5G44110	-1.33	-0.41	-2.57	0.038379	0.09902	-4.63
260017_at	hypothetical protein predicted by ge	---	---	-1.40	-0.48	-2.57	0.038396	0.099048	-4.63
263583_at	putative NAD+ dependent isocitrate	IDH2	AT2G17130	1.36	0.45	2.57	0.038401	0.099048	-4.63
258866_at	unknown protein	---	AT3G03180	1.20	0.26	2.57	0.038403	0.099048	-4.63
249888_s_a	zinc finger protein-like ; supported	---	AT5G22480	1.23	0.30	2.57	0.038442	0.099135	-4.63
257236_at	hypothetical protein	---	AT3G15095	-1.30	-0.38	-2.57	0.038446	0.099135	-4.63
264505_at	putative nodulin protein, N21 Simila	---	AT1G09380	-1.36	-0.45	-2.57	0.038452	0.099141	-4.63
259450_at	hypothetical protein predicted by ge	DRL1	AT1G13870	-1.26	-0.33	-2.57	0.038465	0.099162	-4.63
246633_at	hypothetical protein ; supported by	---	AT1G29720	-1.84	-0.88	-2.57	0.038492	0.099219	-4.63
255570_at	putative carrier protein similar to mi	ADNT1	AT4G01100	1.24	0.31	2.57	0.038496	0.099219	-4.63
262922_at	hypothetical protein predicted by ge	---	AT1G79420	-1.31	-0.39	-2.57	0.038501	0.099221	-4.63
253819_at	receptor protein kinase like protein	---	AT4G28350	2.86	1.51	2.57	0.038534	0.099295	-4.63
252421_at	endochitinase-like protein BASIC E	---	AT3G47540	3.13	1.65	2.57	0.038548	0.099313	-4.63

254850_at	putative protein predicted proteins A	---	AT4G12000	-1.34	-0.42	-2.57	0.03855	0.099313	-4.63
246835_at	putative protein putative APC11 an	---	AT5G26640	-1.27	-0.35	-2.56	0.038557	0.099314	-4.63
260707_s_a	Expressed protein ; supported by fu	---	AT1G32410	-1.30	-0.38	-2.56	0.038559	0.099314	-4.63
253118_at	ras-related small GTP-binding prot	ARAC6	AT4G35950	-1.30	-0.38	-2.56	0.038563	0.099315	-4.63
250266_at	histone H3 -like protein histone H3	---	AT5G12910	-1.25	-0.32	-2.56	0.038577	0.099338	-4.63
248193_at	homogentisate 1,2-dioxygenase ;s	HGO	AT5G54080	1.48	0.56	2.56	0.03859	0.099356	-4.63
265916_at	hypothetical protein similar to En/Sp	---	---	-1.21	-0.28	-2.56	0.038592	0.099356	-4.63
264942_at	unknown protein ; supported by cDI	---	AT1G67340	1.32	0.40	2.56	0.038612	0.099396	-4.63
253584_at	putative protein several hypothetica	---	AT4G30700	1.22	0.29	2.56	0.038645	0.099468	-4.63
252909_at	putative protein	---	AT4G39680	1.20	0.26	2.56	0.038655	0.099483	-4.63
252785_at	putative protein AND-1 protein - Ho	---	AT3G42660	-1.46	-0.55	-2.56	0.038662	0.099491	-4.64
261672_at	unknown protein contains similarity	ATARP4	AT1G18450	1.32	0.40	2.56	0.038676	0.099498	-4.64
264845_at	putative thioredoxin-m similar to thi	ATHM1	AT1G03680	-1.25	-0.32	-2.56	0.038678	0.099498	-4.64
254475_at	putative snRNP protein small nucle	smB	AT4G20440	1.45	0.53	2.56	0.038682	0.099498	-4.64
249001_at	putative protein strong similarity to u	---	AT5G44990	1.95	0.96	2.56	0.038682	0.099498	-4.64
248831_at	putative protein contains similarity t	---	AT5G47160	1.25	0.32	2.56	0.038723	0.09959	-4.64
250247_at	unknown protein	---	AT5G13720	1.20	0.26	2.56	0.03876	0.099675	-4.64
255471_at	putative oxidoreductase similar to A	AOP3	AT4G03050	-1.23	-0.30	-2.56	0.038767	0.099682	-4.64
251341_at	ribosomal protein S13 -like AtRPS1	---	AT3G60770	1.50	0.58	2.56	0.038802	0.099762	-4.64
261094_at	hypothetical protein predicted by ge	---	AT1G62920	-1.35	-0.43	-2.56	0.038816	0.099786	-4.64
245825_at	protein kinase, putative similar to p	ATSK42	AT1G57870	-1.23	-0.30	-2.56	0.038838	0.099818	-4.64
262234_at	putative G protein coupled receptor	GCR1	AT1G48270	-1.34	-0.43	-2.56	0.038841	0.099818	-4.64
257276_at	disease resistance protein, putative	---	AT3G14460	-1.19	-0.25	-2.56	0.038842	0.099818	-4.64
258578_at	germin-like protein contains Pfam p	---	AT3G04200	-1.38	-0.47	-2.56	0.038853	0.099836	-4.64
248099_at	DNA topoisomerase I (sp P30181)	TOP1ALPH	AT5G55300	1.20	0.26	2.56	0.038876	0.099883	-4.64
264859_at	glucose-6-phosphate 1-dehydroger	G6PD3	AT1G24280	1.90	0.93	2.56	0.038888	0.099891	-4.64
253610_at	putative protein ; supported by cDN	VEL1	AT4G30200	1.27	0.34	2.56	0.038888	0.099891	-4.64
255478_at	putative protein ;supported by full-l	EID1	AT4G02440	-1.25	-0.32	-2.56	0.038894	0.099896	-4.64
248779_at	acetoacyl-CoA-thiolase ;supported	---	AT5G47720	1.43	0.51	2.56	0.038904	0.099901	-4.64
265103_at	UDP-N-acetylglucosamine pyropho	---	AT1G31070	-1.29	-0.37	-2.56	0.038905	0.099901	-4.64
247474_at	putative protein predicted proteins,	---	AT5G62280	-3.49	-1.80	-2.56	0.038928	0.099948	-4.64
245156_at	calcium-dependent protein kinase-	CPK7	AT5G12480	1.23	0.30	2.56	0.038946	0.099976	-4.64
250684_at	zinc finger-like protein	GIS2	AT5G06650	-1.23	-0.30	-2.56	0.038947	0.099976	-4.64
258744_at	hypothetical protein similar to plant	---	AT3G05830	1.27	0.34	2.56	0.038972	0.100029	-4.64
254428_at	putative protein Human mRNA for	ZAC	AT4G21160	1.22	0.29	2.56	0.038991	0.100065	-4.64

266388_at	hypothetical protein predicted by ge	---	AT2G32340	1.27	0.35	2.56	0.039008	0.100097	-4.64
256199_at	unknown protein	SAB	AT1G58250	1.30	0.38	2.56	0.039025	0.10011	-4.64
245075_at	putative cytochrome P450	CYP96A1	AT2G23180	1.24	0.31	2.56	0.039028	0.10011	-4.64
257255_at	hypothetical protein contains Pfam	---	AT3G21960	-1.23	-0.30	-2.56	0.039029	0.10011	-4.64
260697_at	unknown protein	---	AT1G32530	1.37	0.45	2.56	0.03903	0.10011	-4.64
254547_at	putative protein	---	AT4G19860	1.37	0.46	2.56	0.039039	0.10012	-4.65
264557_at	putative pectinacetylesterase precu	---	AT1G09550	-1.34	-0.42	-2.56	0.039045	0.10012	-4.65
255781_at	unknown protein	---	AT1G19880	-1.20	-0.27	-2.56	0.039047	0.10012	-4.65
267273_at	putative non-LTR retroelement reve	---	AT2G02520	-1.18	-0.23	-2.56	0.039065	0.100155	-4.65
267590_at	putative expansin ;supported by ful	ATEXPA4	AT2G39700	-1.51	-0.59	-2.56	0.039074	0.100165	-4.65
258456_at	putative protein kinase similar to mi	WNK2	AT3G22420	1.74	0.80	2.55	0.03911	0.100247	-4.65
267564_at	putative protein kinase contains a p	---	AT2G30740	-1.26	-0.33	-2.55	0.039118	0.100257	-4.65
264238_at	hypothetical protein predicted by ge	---	AT1G54740	-1.60	-0.67	-2.55	0.039148	0.100322	-4.65
260064_at	ethylene-insensitive3-like3 (EIL3) i	EIL3	AT1G73730	1.31	0.39	2.55	0.039153	0.100323	-4.65
258670_at	unknown protein	---	AT3G08810	-1.28	-0.36	-2.55	0.039191	0.100393	-4.65
251910_at	serine/threonine-specific kinase lik	---	AT3G53810	2.16	1.11	2.55	0.039191	0.100393	-4.65
263061_at	putative AAA-type ATPase	---	AT2G18190	-1.25	-0.33	-2.55	0.039193	0.100393	-4.65
257789_at	unknown protein ;supported by full-	YSL6	AT3G27020	1.39	0.48	2.55	0.039208	0.100419	-4.65
256697_at	hypothetical protein contains simila	AtOCT4	AT3G20660	1.55	0.64	2.55	0.039212	0.100419	-4.65
263404_s_a	hypothetical protein similar to hypot	---	AT2G04090	1.95	0.97	2.55	0.039239	0.100476	-4.65
252751_at	putative protein RING-H2 zinc finge	---	AT3G43430	-1.54	-0.62	-2.55	0.039287	0.100588	-4.65
248802_at	Expressed protein ; supported by c	---	AT5G47435	1.24	0.31	2.55	0.039317	0.100654	-4.65
261793_at	unknown protein ; supported by cD	---	AT1G16080	-1.59	-0.67	-2.55	0.03933	0.100674	-4.65
254184_at	hypothetical protein	---	AT4G23970	-1.22	-0.29	-2.55	0.039343	0.100697	-4.65
260088_at	tonoplast intrinsic protein, alpha (a	TIP3;1	AT1G73190	-1.27	-0.34	-2.55	0.03936	0.100726	-4.65
247660_at	ankyrin-like protein ankyrin 3, Mus	---	AT5G60070	-1.20	-0.27	-2.55	0.039363	0.100726	-4.65
259351_at	putative sugar transporter similar t	---	AT3G05150	-1.22	-0.29	-2.55	0.039371	0.100734	-4.65
254126_at	RNA-binding protein RNP-T precurs	RBP31	AT4G24770	-1.24	-0.31	-2.55	0.03938	0.100746	-4.65
248529_at	protein kinase ;supported by full-le	---	AT5G50000	-1.35	-0.43	-2.55	0.039407	0.100804	-4.65
251805_at	putative protein RING-H2 finger pr	SDIR1	AT3G55530	1.21	0.27	2.55	0.039415	0.100814	-4.65
249036_at	nuclear cap-binding protein; CBP2	CBP20	AT5G44200	1.26	0.34	2.55	0.039444	0.100877	-4.66
262209_at	hypothetical protein contains simila	---	AT1G74720	1.28	0.35	2.55	0.039454	0.100891	-4.66
266478_at	putative cysteinyl-tRNA synthetase	SYCO ARA	AT2G31170	-1.39	-0.47	-2.55	0.039461	0.100898	-4.66
258991_at	unknown protein contains Pfam pro	---	AT3G08820	1.23	0.30	2.55	0.039474	0.100919	-4.66
250291_at	aspartate kinase ;supported by full-	AK-LYS1	AT5G13280	1.37	0.45	2.55	0.039503	0.100983	-4.66

261835_at	hypothetical protein contains similar	---	AT1G16040	1.24	0.31	2.55	0.039514	0.100999	-4.66
249674_at	unknown protein	---	AT5G35930	1.19	0.25	2.55	0.039537	0.101047	-4.66
257263_at	unknown protein ;supported by full-	---	AT3G22070	-1.44	-0.52	-2.55	0.039547	0.101053	-4.66
255952_at	MADS-box protein (ZAP1), putative	AGL104	AT1G22130	1.18	0.24	2.55	0.039555	0.101053	-4.66
246051_at	epimerase/dehydratase - like prote	GME	AT5G28840	1.29	0.37	2.55	0.039557	0.101053	-4.66
262609_at	unknown protein ;supported by full-	---	AT1G13930	1.26	0.34	2.55	0.039558	0.101053	-4.66
266518_at	hypothetical protein predicted by ge	---	AT2G35170	-1.25	-0.32	-2.55	0.039562	0.101053	-4.66
265179_at	unknown protein predicted by gene	---	AT1G23650	-1.30	-0.38	-2.55	0.039577	0.101079	-4.66
255301_at	putative protein similar to transcripti	ATMSRB3	AT4G04800	1.46	0.54	2.55	0.039588	0.101097	-4.66
246197_at	caltractin-like protein ;supported by	CEN2	AT4G37010	1.94	0.96	2.55	0.039603	0.101125	-4.66
248716_at	unknown protein ;supported by full-	---	AT5G48210	-1.29	-0.37	-2.55	0.039608	0.101125	-4.66
253928_at	phosphoprotein phosphatase (PPX-	PPX1	AT4G26720	1.23	0.29	2.55	0.039633	0.101177	-4.66
259405_at	Expressed protein ; supported by cl	---	AT1G17744	1.35	0.44	2.55	0.039645	0.101197	-4.66
257373_at	hypothetical protein predicted by ge	---	AT2G43140	1.30	0.38	2.55	0.039668	0.101246	-4.66
255013_at	putative protein ;supported by full-l	---	AT4G10000	1.26	0.34	2.54	0.039692	0.101296	-4.66
265010_at	T-complex chaperonin protein , eps	---	AT1G24510	1.36	0.44	2.54	0.039701	0.101307	-4.66
245439_at	hypothetical protein	---	AT4G16670	1.53	0.61	2.54	0.039726	0.10136	-4.66
257914_at	Expressed protein ; supported by cl	---	AT3G25545	1.28	0.35	2.54	0.039734	0.101367	-4.66
247285_at	putative protein similar to unknown	---	AT5G64400	1.20	0.27	2.54	0.039743	0.101379	-4.66
258607_at	thioredoxin f1 identical to thioredoxi	TRXF1	AT3G02730	-1.30	-0.38	-2.54	0.039794	0.101497	-4.66
259093_at	unknown protein similar to putative	---	AT3G04860	-1.49	-0.58	-2.54	0.03982	0.101543	-4.67
265312_at	unknown protein	---	AT2G20240	-1.52	-0.60	-2.54	0.039824	0.101543	-4.67
254222_at	hypothetical protein	---	AT4G23840	-1.25	-0.32	-2.54	0.039825	0.101543	-4.67
259415_at	hypothetical protein contains similar	---	AT1G02330	-1.30	-0.38	-2.54	0.039831	0.101547	-4.67
254683_at	hypothetical protein ;supported by f	---	AT4G13800	-1.24	-0.31	-2.54	0.039887	0.101678	-4.67
247292_at	unknown protein	---	AT5G64490	1.25	0.32	2.54	0.039891	0.101679	-4.67
253125_at	DnaJ-like protein DnaJ-like protein	---	AT4G36040	1.30	0.38	2.54	0.039939	0.101789	-4.67
264593_at	putative GTP-binding protein ; supp	ARAC1	AT2G17800	-1.23	-0.30	-2.54	0.039974	0.101844	-4.67
258503_at	unknown protein	---	AT3G02500	-1.28	-0.36	-2.54	0.039974	0.101844	-4.67
245338_at	Expressed protein ; supported by fu	---	AT4G16442	-1.32	-0.40	-2.54	0.039981	0.101844	-4.67
263225_at	putative DNA-binding protein simila	WRKY14	AT1G30650	-1.25	-0.32	-2.54	0.039984	0.101844	-4.67
257274_at	geranylgeranyl pyrophosphate syn	---	AT3G14510	-1.25	-0.32	-2.54	0.039987	0.101844	-4.67
245272_at	hypothetical protein ; supported by	---	AT4G17250	1.51	0.59	2.54	0.039988	0.101844	-4.67
245736_at	Dr4(protease inhibitor) identical to D	ATDR4	AT1G73330	-1.60	-0.68	-2.54	0.04	0.101864	-4.67
248449_at	putative protein similar to unknown	---	AT5G51110	-1.19	-0.25	-2.54	0.040007	0.10187	-4.67

246345_at	calcium-dependent protein kinase-	---	AT3G56760	1.29	0.37	2.54	0.040027	0.101909	-4.67
246519_at	proline-rich protein other proline-ricl	---	AT3G23750	-3.01	-1.59	-2.54	0.040031	0.101909	-4.67
260597_at	hypothetical protein predicted by ge	---	AT1G55915	1.36	0.44	2.54	0.040036	0.10191	-4.67
252059_at	E2, ubiquitin-conjugating enzyme,	UEV1D-4	AT3G52560	1.21	0.28	2.54	0.040072	0.10199	-4.67
258667_at	hypothetical protein	---	AT3G08750	-1.21	-0.28	-2.54	0.040099	0.102043	-4.67
248990_at	disease resistance protein-like	---	AT5G45210	-1.22	-0.28	-2.54	0.040101	0.102043	-4.67
249816_at	cleavage and polyadenylation spec	CPSF100	AT5G23880	1.32	0.40	2.54	0.040114	0.102063	-4.67
245150_at	photolyase/blue-light receptor (PHR	PHR2	AT2G47590	-1.27	-0.34	-2.54	0.040122	0.102071	-4.67
260193_at	putative amino acid permease simil	---	AT1G67640	-1.31	-0.39	-2.54	0.040126	0.102071	-4.67
265128_at	hypothetical protein predicted by ge	---	AT1G30860	-1.30	-0.38	-2.54	0.040135	0.102071	-4.67
263895_at	hypothetical protein predicted by ge	---	AT2G21920	-1.18	-0.23	-2.54	0.040139	0.102071	-4.67
261586_at	hypothetical protein predicted by g	---	AT1G01640	1.56	0.64	2.54	0.040139	0.102071	-4.67
251856_at	Peptidase - like protein ileal peptid	AMP1	AT3G54720	-1.41	-0.49	-2.54	0.040145	0.102075	-4.67
245656_at	hypothetical protein predicted by ge	---	AT1G56620	-1.22	-0.29	-2.54	0.040154	0.102086	-4.67
247198_at	unknown protein	---	AT5G65290	-1.20	-0.27	-2.54	0.04018	0.102131	-4.67
260979_at	MAP kinase ATMPK9, putative sim	ATMPK18	AT1G53510	-1.21	-0.27	-2.54	0.040181	0.102131	-4.67
259621_at	unknown protein	---	AT1G42710	-1.19	-0.25	-2.54	0.040221	0.102222	-4.68
256296_at	hypothetical protein	---	AT1G69480	1.19	0.26	2.54	0.040231	0.102235	-4.68
267173_at	origin recognition complex protein i	ORC2	AT2G37560	-1.41	-0.50	-2.53	0.040297	0.102373	-4.68
249326_at	unknown protein ; supported by cD	SYN2	AT5G40840	1.23	0.30	2.53	0.0403	0.102373	-4.68
250181_at	tRNA synthase - like protein tRNA	---	AT5G14460	1.23	0.30	2.53	0.040303	0.102373	-4.68
262084_at	hypothetical protein predicted by ge	---	AT1G56080	-1.25	-0.33	-2.53	0.040303	0.102373	-4.68
260556_at	putative endochitinase	---	AT2G43620	3.34	1.74	2.53	0.040311	0.102381	-4.68
265843_at	putative acyl-CoA oxidase possibly	ACX5	AT2G35690	-1.23	-0.30	-2.53	0.040315	0.102381	-4.68
254242_at	serine /threonine kinase - like prote	---	AT4G23200	-1.94	-0.96	-2.53	0.040319	0.102381	-4.68
249994_at	putative protein cell polarity protein	---	AT5G18590	1.24	0.31	2.53	0.040326	0.102381	-4.68
264380_at	unknown protein predicted by gens	---	AT2G25190	-1.17	-0.22	-2.53	0.040328	0.102381	-4.68
253048_at	formamidase - like protein formami	---	AT4G37560	1.74	0.80	2.53	0.040345	0.102402	-4.68
252864_at	putative protein regulatory protein	---	AT4G39740	1.46	0.54	2.53	0.040351	0.102402	-4.68
260449_at	unknown protein	---	AT1G72390	1.18	0.23	2.53	0.040352	0.102402	-4.68
247220_at	cdc2-like protein kinase ; supporte	CDKC2	AT5G64960	1.23	0.29	2.53	0.040355	0.102402	-4.68
266473_at	unknown protein	---	AT2G31110	1.81	0.85	2.53	0.040362	0.102409	-4.68
259236_at	unknown protein ; supported by ful	---	AT3G11530	1.20	0.27	2.53	0.0404	0.102494	-4.68
265160_at	unknown protein similar to EST gb	---	AT1G31050	-1.28	-0.36	-2.53	0.040437	0.102576	-4.68
246746_at	putative protein predicted proteins,	---	AT5G27820	1.26	0.33	2.53	0.040497	0.102716	-4.68

256784_at	hypothetical protein predicted by ge	---	AT3G13674	-1.20	-0.26	-2.53	0.040508	0.102734	-4.68
245864_at	hypothetical protein predicted by ge	---	AT1G58070	-1.42	-0.50	-2.53	0.040526	0.102755	-4.68
262133_at	high affinity sulphate transporter, p	SULTR1;2	AT1G78000	1.95	0.97	2.53	0.040529	0.102755	-4.68
257791_at	unknown protein contains Pfam pro	---	AT3G27110	1.32	0.40	2.53	0.04053	0.102755	-4.68
244932_at	PSI 9KDa protein	---	---	-1.26	-0.34	-2.53	0.040572	0.102851	-4.68
267587_at	unknown protein	---	AT2G42080	-1.33	-0.41	-2.53	0.040577	0.102851	-4.68
246660_at	putative protein predicted protein, A	---	AT5G35180	-1.22	-0.29	-2.53	0.040585	0.10286	-4.69
251845_at	Expressed protein ; supported by fu	ATGCN4	AT3G54540	1.32	0.40	2.53	0.040619	0.102935	-4.69
262166_at	myb-related transcription activator,	---	AT1G74840	-1.30	-0.38	-2.53	0.040654	0.103012	-4.69
259421_at	disease resistance protein, putative	---	AT1G13910	1.28	0.36	2.53	0.040718	0.103162	-4.69
267448_at	putative protein phosphatase 2C	---	AT2G33700	1.18	0.23	2.53	0.040723	0.103164	-4.69
259715_at	hypothetical protein predicted by ge	---	AT1G60990	1.28	0.36	2.53	0.040728	0.103165	-4.69
261134_at	cytochrome P450, putative similar t	CYP722A1	AT1G19630	-1.21	-0.28	-2.53	0.040784	0.103295	-4.69
262632_at	23 kDa polypeptide of oxygen-evolv	PSBP-1	AT1G06680	-1.22	-0.29	-2.53	0.040805	0.103324	-4.69
255549_at	predicted protein of unknown functi	GPAT3	AT4G01950	-2.65	-1.41	-2.53	0.040807	0.103324	-4.69
263690_at	putative DNA-binding protein Simila	AtHB23	AT1G26960	-1.44	-0.52	-2.53	0.040809	0.103324	-4.69
251776_at	eukaryotic translation initiation fact	emb1624	AT3G55620	1.32	0.40	2.52	0.04084	0.103382	-4.69
257545_at	hypothetical protein predicted by ge	---	AT3G23200	1.35	0.43	2.52	0.040841	0.103382	-4.69
263601_s_a	dihydrofolate reductase-thymidylate	THY-1	AT2G16370	1.30	0.38	2.52	0.040854	0.103395	-4.69
259309_at	putative cyclin-dependent protein ki	---	AT3G05050	1.31	0.39	2.52	0.040856	0.103395	-4.69
262208_at	hypothetical protein predicted by ge	---	AT1G74800	-1.26	-0.34	-2.52	0.040864	0.103395	-4.69
266835_at	putative NADH dehydrogenase (ubi	NDA2	AT2G29990	1.77	0.82	2.52	0.040864	0.103395	-4.69
248559_at	unknown protein	---	AT5G50010	-1.23	-0.30	-2.52	0.040893	0.103456	-4.69
254545_at	putative protein FKBP-type peptidy	---	AT4G19830	-1.42	-0.51	-2.52	0.040922	0.103512	-4.69
251293_at	hypothetical protein	---	AT3G61930	3.58	1.84	2.52	0.040924	0.103512	-4.69
256708_at	hypothetical protein predicted by ge	---	AT3G30320	-1.19	-0.26	-2.52	0.040933	0.103523	-4.69
265535_at	hypothetical protein predicted by ge	---	AT2G15900	1.20	0.27	2.52	0.040945	0.103542	-4.69
262215_at	hypothetical protein contains simila	---	AT1G74790	-1.59	-0.66	-2.52	0.040954	0.103553	-4.69
264904_s_a	putative thioredoxin reductase	NTRA	AT2G17420	1.31	0.39	2.52	0.040965	0.103569	-4.69
261948_at	unknown protein ;supported by full-	---	AT1G64680	-1.43	-0.52	-2.52	0.04099	0.103622	-4.70
262640_at	hypothetical protein	---	AT1G62760	4.83	2.27	2.52	0.041023	0.103694	-4.70
254660_at	receptor serine/threonine kinase-lik	---	AT4G18250	1.41	0.50	2.52	0.041049	0.103745	-4.70
258321_at	early light-induced protein identical	ELIP1	AT3G22840	-1.25	-0.32	-2.52	0.041052	0.103745	-4.70
249624_at	putative protein predicted proteins,	---	AT5G37570	1.30	0.38	2.52	0.041079	0.1038	-4.70
261326_s_a	aminoacylase, putative similar to a	---	AT1G44180	1.37	0.45	2.52	0.041102	0.103848	-4.70

263911_at	hypothetical protein predicted by ge	---	AT2G36560	-1.19	-0.26	-2.52	0.041126	0.103898	-4.70
258156_at	unknown protein ; supported by cD	---	AT3G18050	-1.49	-0.58	-2.52	0.041163	0.103979	-4.70
246383_at	hypothetical protein predicted by ge	---	AT1G77360	1.42	0.50	2.52	0.041202	0.104067	-4.70
263073_at	unknown protein ;supported by full-	---	AT2G17500	4.78	2.26	2.52	0.041214	0.104083	-4.70
260451_at	putative AP2 domain transcription fa	---	AT1G72360	-2.06	-1.04	-2.52	0.041222	0.104092	-4.70
249702_at	axi 1 (auxin-independent growth pro	---	AT5G35570	-1.66	-0.73	-2.52	0.041226	0.104092	-4.70
262361_at	unknown protein contains similarity	GTE3	AT1G73150	1.22	0.28	2.52	0.041241	0.104119	-4.70
255289_at	score=11/3, E=0.23, N=1 contains	---	AT4G04690	-1.23	-0.30	-2.52	0.041262	0.10416	-4.70
250252_at	transporter-like protein ;supported b	ZIFL1	AT5G13750	2.03	1.02	2.52	0.041277	0.104186	-4.70
250173_at	MYB40 - putative transcription facto	AtMYB40	AT5G14340	-1.25	-0.33	-2.52	0.041293	0.104214	-4.70
255442_at	predicted NADH dehydrogenase 24	---	AT4G02580	-1.20	-0.26	-2.52	0.0413	0.104221	-4.70
267156_at	hypothetical protein predicted by ge	---	AT2G37610	-1.23	-0.30	-2.52	0.04131	0.104233	-4.70
245787_at	unknown protein	---	AT1G32130	1.24	0.31	2.52	0.041314	0.104233	-4.70
259596_at	auxin-regulated GH3 protein, putat	GH3.17	AT1G28130	-1.57	-0.66	-2.52	0.04132	0.104237	-4.70
253982_at	calcineurin B-like protein 3 ;suppor	ATCBL3	AT4G26570	-1.36	-0.44	-2.52	0.041349	0.104295	-4.70
262017_at	elongation factor, putative similar to	---	AT1G35550	1.34	0.42	2.52	0.041352	0.104295	-4.70
245039_at	putative beta-1,3-glucanase ;supp	---	AT2G26600	1.23	0.29	2.52	0.041367	0.104321	-4.70
247486_at	putative protein	---	AT5G62140	-1.36	-0.45	-2.51	0.041431	0.104471	-4.71
260849_at	pectinesterase, putative similar to p	sks7	AT1G21860	-1.18	-0.24	-2.51	0.041436	0.104471	-4.71
262658_at	ribonuclease contains similarity to S	---	AT1G14220	-1.19	-0.25	-2.51	0.041476	0.104562	-4.71
264986_at	glutathione transferase, putative si	ATGSTU13	AT1G27130	2.22	1.15	2.51	0.041494	0.104595	-4.71
264526_at	putative calcium ATPase very simil	ECA3	AT1G10130	1.26	0.33	2.51	0.041519	0.104642	-4.71
253890_s_a	putative protein contains similarity t	---	AT4G27585 ,	1.61	0.69	2.51	0.041522	0.104642	-4.71
263794_at	similar to S-locus glycoproteins	---	AT2G24620	-1.18	-0.24	-2.51	0.041532	0.104656	-4.71
260414_at	nitrate transporter (NTL1) identical t	ATNRT1:2	AT1G69850	-1.26	-0.33	-2.51	0.041547	0.104656	-4.71
251908_at	putative protein late embryogenis a	---	AT3G53770	-1.24	-0.31	-2.51	0.04155	0.104656	-4.71
258494_at	cell division protein FtsH-like protei	---	AT3G02450	1.28	0.36	2.51	0.04155	0.104656	-4.71
263590_at	putative receptor-like protein kinase	---	AT2G01820	-1.20	-0.27	-2.51	0.04155	0.104656	-4.71
256678_at	RNA-binding protein (cp33) identic	CP33	AT3G52380	1.24	0.31	2.51	0.041558	0.104664	-4.71
257977_at	hypothetical protein predicted by ge	---	AT3G20850	-1.22	-0.28	-2.51	0.041587	0.104726	-4.71
260825_at	hypothetical protein predicted by ge	---	AT1G06810	-1.21	-0.27	-2.51	0.041597	0.104738	-4.71
245333_at	Expressed protein ; supported by fu	---	AT4G14615	1.17	0.22	2.51	0.04162	0.104786	-4.71
267075_at	putative bZIP transcription factor co	EEL	AT2G41070	1.19	0.25	2.51	0.041625	0.104786	-4.71
254623_at	protein ch-42 precursor, chloroplas	CHLI1	AT4G18480	-1.32	-0.40	-2.51	0.041643	0.104817	-4.71
266943_at	putative reverse transcriptase	---	---	-1.34	-0.43	-2.51	0.041646	0.104817	-4.71

266868_at	hypothetical protein predicted by ge	---	AT2G44630	1.21	0.28	2.51	0.041672	0.10487	-4.71
264972_at	meiotic asynaptic mutant asy1, put	ASY1	AT1G67370	1.36	0.44	2.51	0.041682	0.104874	-4.71
248012_at	putative protein strong similarity to u	---	AT5G56310	1.29	0.37	2.51	0.041683	0.104874	-4.71
245468_at	pectinesterase like protein	---	AT4G15980	-1.20	-0.26	-2.51	0.041694	0.104891	-4.71
251933_at	putative protein mucin MUC3, Hom	---	AT3G54060	-1.28	-0.35	-2.51	0.041712	0.104911	-4.71
265796_at	Expressed protein ; supported by fu	---	AT2G35730	2.97	1.57	2.51	0.041713	0.104911	-4.71
256083_at	hypothetical protein predicted by ge	---	AT1G20730	-1.17	-0.23	-2.51	0.041716	0.104911	-4.71
264148_at	NAM (no apical meristem)-like prot	ANAC003	AT1G02220	5.32	2.41	2.51	0.041722	0.104915	-4.71
265039_at	unknown protein Location of ESTs	---	AT1G04000	-1.21	-0.28	-2.51	0.041728	0.104917	-4.71
258928_at	unknown protein similar to hypothet	TAF12	AT3G10070	1.30	0.38	2.51	0.041793	0.10507	-4.72
249631_at	sen1-like protein SEN1 - Saccharo	---	AT5G37160	-1.22	-0.29	-2.51	0.041824	0.105135	-4.72
250835_at	putative protein various predicted p	DME	AT5G04560	-1.26	-0.34	-2.51	0.041834	0.10515	-4.72
249836_at	unknown protein ;supported by full-	HMGB6	AT5G23420	1.53	0.62	2.51	0.041847	0.10517	-4.72
245985_at	peptidylprolyl isomerase-like protei	---	AT5G13120	-1.29	-0.36	-2.51	0.04186	0.105192	-4.72
250323_at	putative protein hydroxyproline-rich	---	AT5G12880	2.10	1.07	2.51	0.041877	0.105222	-4.72
249333_at	GDSL-motif lipase/hydrolase-like pr	GLIP1	AT5G40990	2.19	1.13	2.51	0.041881	0.105222	-4.72
263341_at	hypothetical protein predicted by ge	---	---	-1.24	-0.31	-2.51	0.041917	0.1053	-4.72
266447_at	putative calcium binding protein ;su	MSS3	AT2G43290	-1.70	-0.77	-2.51	0.041926	0.105302	-4.72
263020_at	unknown protein	---	AT1G23880	-1.21	-0.27	-2.51	0.041931	0.105302	-4.72
250859_at	cytochrom P450 - like protein cytoc	CYP77A4	AT5G04660	-1.16	-0.22	-2.51	0.041932	0.105302	-4.72
264044_at	putative pyrophosphate--fructose-6-	PFK5	AT2G22480	1.22	0.28	2.51	0.041956	0.105352	-4.72
262978_at	tubulin beta-1 chain identical to GB	TUB1	AT1G75780	-3.54	-1.82	-2.50	0.042042	0.105557	-4.72
254464_at	putative protein retinoblastoma bin	---	AT4G20400	1.33	0.41	2.50	0.042076	0.105631	-4.72
251032_at	putative homeodomain protein seve	RPL	AT5G02030	1.63	0.71	2.50	0.042115	0.105715	-4.72
264361_at	unknown protein Strong similarity to	---	AT1G03300	-1.23	-0.30	-2.50	0.04216	0.105819	-4.72
247895_at	putative protein bHLH transcription	---	AT5G58010	-1.21	-0.28	-2.50	0.042169	0.105827	-4.72
257922_at	ethylene receptor, putative (ETR2)	ETR2	AT3G23150	1.49	0.57	2.50	0.042189	0.105868	-4.72
266994_at	MADS-box protein (AGL29)	AGL29	AT2G34440	-1.25	-0.33	-2.50	0.042198	0.105878	-4.73
263355_at	putative RNA-binding protein ;supp	---	AT2G22100	1.28	0.36	2.50	0.042205	0.105884	-4.73
265680_at	putative hydrolase ; supported by c	---	AT2G32150	-1.43	-0.51	-2.50	0.042219	0.105908	-4.73
245392_at	glutaredoxin ;supported by full-leng	---	AT4G15680	-2.15	-1.11	-2.50	0.042224	0.105908	-4.73
262644_at	beta-VPE nearly identical to beta-V	BETA-VPE	AT1G62710	-1.23	-0.30	-2.50	0.042269	0.106006	-4.73
248668_at	putative protein similar to unknown	XRI1	AT5G48720	1.40	0.49	2.50	0.042272	0.106006	-4.73
252884_at	SEC14 - like protein phosphatidylin	---	AT4G39170	-1.26	-0.33	-2.50	0.042305	0.106076	-4.73
254579_at	hypothetical protein ;supported by f	---	AT4G19400	-1.27	-0.35	-2.50	0.042312	0.106083	-4.73

256215_at	unknown protein ;supported by full-	---	AT1G50900	-1.24	-0.31	-2.50	0.042318	0.106085	-4.73
256819_at	unknown protein contains Pfam pro	---	AT3G21390	-1.21	-0.27	-2.50	0.042338	0.106124	-4.73
251825_at	ABC transporter - like protein ATP-	---	AT3G55100	-1.23	-0.30	-2.50	0.042372	0.106197	-4.73
248075_at	selenium-binding protein-like	CRR21	AT5G55740	1.37	0.45	2.50	0.042381	0.106208	-4.73
247149_at	unknown protein ;supported by full-	---	AT5G65660	-1.53	-0.62	-2.50	0.042411	0.106271	-4.73
253836_at	putative protein hypothetical protei	---	AT4G27840	1.22	0.28	2.50	0.042455	0.10637	-4.73
260509_at	natural resistance-associated macr	NRAMP2	AT1G47240	-1.17	-0.23	-2.50	0.042469	0.106393	-4.73
267464_at	putative pectinesterase	---	AT2G19150	-1.23	-0.30	-2.50	0.042481	0.106413	-4.73
251539_at	serine/threonine-specific protein ki	---	AT3G58690	-1.30	-0.38	-2.50	0.042504	0.106457	-4.73
259832_at	transfactor, putative similar to GI:4	---	AT1G69580	-1.67	-0.74	-2.50	0.04251	0.106461	-4.73
264819_at	hypothetical protein similar to meml	---	AT1G03510	1.37	0.46	2.50	0.042517	0.106467	-4.73
259612_at	60S ribosomal protein L37, putative	---	AT1G52300	1.51	0.60	2.50	0.042541	0.106516	-4.73
246994_at	putative protein similar to unknown	---	AT5G67460	-1.64	-0.71	-2.50	0.04255	0.106527	-4.73
262804_at	putative RNA-binding protein simila	---	AT1G20880	1.28	0.36	2.50	0.042575	0.106577	-4.73
262580_at	hypothetical protein Similar to gb|U	---	AT1G15330	-1.32	-0.40	-2.50	0.042592	0.106604	-4.73
260518_at	cinnamyl alcohol dehydrogenase, p	---	AT1G51410	-1.18	-0.24	-2.50	0.042598	0.106604	-4.73
253419_at	putative protein predicted proteins,	---	AT4G32780	-1.26	-0.33	-2.50	0.0426	0.106604	-4.73
245191_at	F12A21.10 similar to terminal ear1	TEL2	AT1G67770	-1.37	-0.46	-2.50	0.042612	0.106624	-4.74
263839_at	putative cis-Golgi SNARE protein ;	MEMB11	AT2G36900	1.35	0.44	2.49	0.042629	0.106654	-4.74
250431_at	cyclin protein - like cyclin D2.1 prot	CYCD4;2	AT5G10440	-1.21	-0.28	-2.49	0.042634	0.106654	-4.74
266084_at	hypothetical protein predicted by ge	---	AT2G37810	-1.29	-0.37	-2.49	0.042644	0.106669	-4.74
261004_at	hypothetical protein similar to putat	---	AT1G26450	-1.38	-0.46	-2.49	0.042709	0.10682	-4.74
264716_at	matrix metalloproteinase, putative	MMP	AT1G70170	1.73	0.79	2.49	0.042752	0.106915	-4.74
264349_at	putative Proline synthetase associa	---	AT1G11930	1.16	0.22	2.49	0.042757	0.106915	-4.74
249711_at	putative protein contains similarity t	---	AT5G35680	1.28	0.35	2.49	0.042775	0.106947	-4.74
264098_at	hypothetical protein predicted by ge	---	AT1G79260	-1.41	-0.49	-2.49	0.042779	0.106947	-4.74
256392_at	putative glycosyl transferase contai	GATL4	AT3G06260	-1.24	-0.31	-2.49	0.042785	0.10695	-4.74
263893_at	putative WRKY-type DNA binding p	WRKY59	AT2G21900	-1.31	-0.39	-2.49	0.04286	0.107127	-4.74
250361_at	putative protein various predicted p	---	AT5G11370	-1.18	-0.24	-2.49	0.042912	0.107245	-4.74
253858_at	carbohydrate kinase - like protein p	---	AT4G27600	1.19	0.25	2.49	0.042923	0.10726	-4.74
259689_x_a	unknown protein	---	AT1G63130	-1.42	-0.51	-2.49	0.042939	0.10729	-4.74
247397_at	Dof zinc finger protein - like Dof zir	---	AT5G62940	-1.40	-0.48	-2.49	0.043024	0.107488	-4.75
264519_at	putative reverse transcriptase simila	---	AT1G10000	-1.27	-0.35	-2.49	0.043032	0.107497	-4.75
251313_at	putative protein several hypothetica	---	AT3G61360	1.23	0.30	2.49	0.043042	0.10751	-4.75
246774_at	polygalacturonase-like protein poly	---	AT5G27530	-1.22	-0.28	-2.49	0.043055	0.10753	-4.75

248575_at	ATP-dependent Clp protease ATP-	---	AT5G49840	1.19	0.25	2.49	0.043064	0.107543	-4.75
247902_at	plasma membrane ATPase 3 (proto	AHA3	AT5G57350	1.26	0.34	2.49	0.043086	0.107586	-4.75
253793_at	myosin heavy chain - like protein (f	XIH	AT4G28710	-1.18	-0.24	-2.49	0.043118	0.107652	-4.75
255135_at	putative protein several hypothetica	---	---	-1.22	-0.29	-2.49	0.043122	0.107652	-4.75
263626_at	transcription factor ZAP1 identical t	ZAP1	AT2G04880	1.18	0.24	2.49	0.043129	0.107658	-4.75
245098_at	ethylene response sensor (ERS) ide	ERS1	AT2G40940	-1.56	-0.64	-2.49	0.043148	0.107692	-4.75
262327_at	hypothetical protein similar to hypot	---	AT1G64130	-1.21	-0.28	-2.49	0.043163	0.107705	-4.75
250661_at	nucleoid DNA-binding-like protein ;	---	AT5G07030	-3.32	-1.73	-2.49	0.043173	0.107705	-4.75
253690_at	putative protein various predicted p	---	AT4G29550	-1.33	-0.41	-2.49	0.043173	0.107705	-4.75
258926_s_a	unknown protein N-terminus similar	anac052	AT3G10480	1.30	0.38	2.49	0.043178	0.107705	-4.75
263177_at	hypothetical protein predicted by ge	---	AT1G05540	-1.21	-0.27	-2.49	0.04318	0.107705	-4.75
265966_at	putative RNA-binding protein ; sup	---	AT2G37220	1.29	0.37	2.49	0.043185	0.107705	-4.75
248007_at	S-adenosylmethionine:2-demethylm	---	AT5G56260	1.18	0.24	2.49	0.043186	0.107705	-4.75
254993_at	putative protein kinase	---	AT4G10730	1.20	0.26	2.49	0.043193	0.10771	-4.75
254859_at	putative protein Arabidopsis thaliar	---	AT4G12060	-1.29	-0.37	-2.49	0.043228	0.107787	-4.75
256879_at	unknown protein similar to auxin-inc	---	AT3G26370	1.17	0.23	2.48	0.043238	0.107801	-4.75
261343_s_a	putative protein hypothetical protein	---	---	-1.19	-0.25	-2.48	0.043278	0.107886	-4.75
255420_at	putative frataxin-like protein similar	FH	AT4G03240	1.18	0.24	2.48	0.043289	0.107903	-4.75
251328_at	putative protein putative protein At2	ATPOB1	AT3G61600	-1.18	-0.24	-2.48	0.043306	0.107934	-4.75
245704_at	glycine-rich protein	KTF1	AT5G04290	1.18	0.24	2.48	0.043356	0.108047	-4.75
251662_at	putative protein DNA-DIRECTED R	NRPE5	AT3G57080	1.28	0.36	2.48	0.043389	0.108118	-4.75
250438_at	putative protein predicted protein, A	---	AT5G10580	-1.19	-0.25	-2.48	0.043413	0.108164	-4.75
260168_at	unknown protein contains zinc fing	---	AT1G71980	1.29	0.37	2.48	0.04344	0.10822	-4.75
248170_at	ankyrin-repeat-containing protein-lik	---	AT5G54620	-1.30	-0.38	-2.48	0.043456	0.108249	-4.76
258479_at	hypothetical protein predicted by ge	---	AT3G02440	-1.23	-0.29	-2.48	0.043503	0.108353	-4.76
252701_at	hypothetical protein SPOP protein,	ATBPM6	AT3G43700	1.28	0.36	2.48	0.043516	0.108375	-4.76
257770_at	unknown protein ;supported by full-	LFR	AT3G22990	1.23	0.30	2.48	0.043537	0.108404	-4.76
264688_at	hypothetical protein similar to LG27	---	AT1G09890	-1.32	-0.41	-2.48	0.043542	0.108404	-4.76
247592_at	high affinity nitrate transporter prot	NRT2.3	AT5G60780	-1.19	-0.25	-2.48	0.043542	0.108404	-4.76
253221_at	peptidylprolyl isomerase (cyclophilin	ROC5	AT4G34870	1.29	0.37	2.48	0.043593	0.108519	-4.76
262434_at	lysine and histidine specific transpo	---	AT1G47670	-1.46	-0.55	-2.48	0.043604	0.108534	-4.76
246348_at	putative protein putative glycerol-3-	---	AT3G56840	1.16	0.21	2.48	0.043616	0.108551	-4.76
248585_at	unknown protein	---	AT5G49640	1.22	0.28	2.48	0.043641	0.108602	-4.76
253824_at	putative protein protein kinase LeP	MTM1	AT4G27940	1.93	0.95	2.48	0.043663	0.108636	-4.76
265488_at	hypothetical protein predicted by ge	---	AT2G15610	-1.23	-0.29	-2.48	0.043664	0.108636	-4.76

248616_at	putative protein contains similarity t	---	AT5G49580	1.30	0.38	2.48	0.043669	0.108636	-4.76
258523_at	pectin methylesterase, putative sim	---	AT3G06830	-1.16	-0.22	-2.48	0.043683	0.10866	-4.76
247256_at	putative protein contains similarity t	---	AT5G64730	1.18	0.24	2.48	0.043688	0.108661	-4.76
248835_at	NBS/LRR disease resistance protei	---	AT5G47250	1.42	0.50	2.48	0.043709	0.108696	-4.76
246985_at	putative protein contains similarity t	---	AT5G67290	1.24	0.31	2.48	0.043712	0.108696	-4.76
250215_at	putative protein similar to unknown	---	AT5G14080	1.21	0.28	2.48	0.043755	0.108791	-4.76
260941_at	peroxidase, putative similar to pero	---	AT1G44970	-1.35	-0.43	-2.48	0.04377	0.108807	-4.76
263668_at	putative 1-aminocyclopropane-1-ca	---	AT1G04350	1.25	0.33	2.48	0.043771	0.108807	-4.76
262557_at	photosystem I subunit III precursor	---	AT1G31330	-1.22	-0.29	-2.48	0.043781	0.108821	-4.76
251948_at	diaminopimelate epimerase - like p	---	AT3G53580	1.25	0.32	2.48	0.043801	0.108852	-4.76
260400_at	putative peptide transporter similar	---	AT1G69860	-1.18	-0.24	-2.48	0.043803	0.108852	-4.76
266572_at	unknown protein	---	AT2G23840	1.34	0.42	2.48	0.04382	0.108883	-4.76
266909_at	hypothetical protein predicted by ge	BRM	AT2G46020	-1.27	-0.35	-2.48	0.043855	0.108957	-4.76
248353_at	cytochrome P450	CYP96A4	AT5G52320	-1.20	-0.26	-2.47	0.043866	0.108971	-4.76
249931_at	putative protein similar to unknown	QQT1	AT5G22370	1.32	0.40	2.47	0.043875	0.108983	-4.77
257833_at	transcriptional regulator protein, pu	LWD2	AT3G26640	-1.18	-0.24	-2.47	0.043896	0.109022	-4.77
245341_at	Expressed protein ; supported by fu	---	AT4G16447	1.71	0.78	2.47	0.0439	0.109022	-4.77
249251_at	putative protein similar to unknown	---	AT5G42000	-1.29	-0.36	-2.47	0.043927	0.109077	-4.77
251505_at	putative protein polyamine oxidase	CPuORF18	AT3G59050	-1.38	-0.47	-2.47	0.04394	0.109098	-4.77
257468_at	hypothetical protein contains similar	---	AT1G47470	-1.18	-0.24	-2.47	0.04397	0.10915	-4.77
259349_at	unknown protein ; supported by cD	ATAPRL5	AT3G03860	1.19	0.25	2.47	0.043971	0.10915	-4.77
250160_at	putative protein various predicted p	ATHB30	AT5G15210	1.60	0.68	2.47	0.043979	0.109158	-4.77
267083_at	calmodulin-like protein identical to C	TCH3	AT2G41100	-1.52	-0.60	-2.47	0.044	0.109199	-4.77
262129_at	mutM homologue-1 identical to mut	ATMMH-1	AT1G52500	1.28	0.35	2.47	0.044009	0.109208	-4.77
AFFX-r2-At-	Arabidopsis thaliana /REF=U37281	---	---	-1.18	-0.24	-2.47	0.044023	0.109232	-4.77
260025_at	unknown protein ;supported by full-	---	AT1G30070	1.38	0.47	2.47	0.044032	0.109242	-4.77
254695_at	putative protein predicted protein, S	ATL4H	AT4G17905	-1.25	-0.32	-2.47	0.044057	0.109293	-4.77
259074_at	putative protein kinase contains Pfa	RPK2	AT3G02130	-1.35	-0.44	-2.47	0.04408	0.109337	-4.77
249990_at	hypothetical protein	---	AT5G18540	1.20	0.26	2.47	0.044113	0.109397	-4.77
264057_at	putative AP2 domain transcription fa	RAP2.7	AT2G28550	-1.37	-0.45	-2.47	0.044116	0.109397	-4.77
245183_at	putative protein similarity to predict	---	AT5G12440	1.30	0.38	2.47	0.044118	0.109397	-4.77
255763_at	hypothetical protein predicted by ge	---	AT1G16730	1.27	0.34	2.47	0.044211	0.109614	-4.77
259606_at	hypothetical protein predicted by ge	MAP65-8	AT1G27920	-1.18	-0.24	-2.47	0.044233	0.109657	-4.77
262059_at	DnaJ protein, putative similar to Dr	---	AT1G80030	-1.32	-0.40	-2.47	0.044252	0.109693	-4.77
246785_at	glutathione synthetase gsh2 non-co	GSH2	AT5G27380	1.48	0.56	2.47	0.044286	0.10976	-4.77

267296_at	hypothetical protein predicted by ge	LBD10	AT2G23660	-1.22	-0.28	-2.47	0.044289	0.10976	-4.77
245975_at	putative protein CGI-107 protein - I	---	AT5G13070	1.27	0.34	2.47	0.044312	0.109806	-4.78
260669_at	hypothetical protein predicted by ge	---	AT1G19340	1.22	0.29	2.47	0.044348	0.109882	-4.78
244978_at	RNA polymerase alpha subunit	---	---	-1.20	-0.26	-2.47	0.044355	0.109887	-4.78
251449_at	Rab GDP dissociation inhibitor ; su	ATGDI2	AT3G59920	1.28	0.35	2.47	0.044363	0.109895	-4.78
251690_at	putative protein TATA-binding prote	---	AT3G56510	1.22	0.28	2.47	0.044371	0.109904	-4.78
254131_at	protein kinase (AFC2) ; supported	AFC2	AT4G24740	1.25	0.32	2.47	0.04438	0.109904	-4.78
266738_at	unknown protein predicted by gene	---	AT2G47010	-1.33	-0.41	-2.47	0.044381	0.109904	-4.78
250347_at	putative protein	---	AT5G11960	-1.20	-0.27	-2.47	0.044387	0.109907	-4.78
266465_at	putative auxin-responsive protein ;	GH3.9	AT2G47750	-1.24	-0.31	-2.47	0.044406	0.109942	-4.78
265973_at	putative non-LTR retroelement reve	---	---	-1.18	-0.23	-2.47	0.044419	0.109944	-4.78
249207_at	C2H2-type zinc finger protein-like	---	AT5G42640	-1.21	-0.28	-2.47	0.044431	0.109944	-4.78
252415_at	glutamine-dependent asparagine sy	ASN1	AT3G47340	-3.76	-1.91	-2.47	0.044433	0.109944	-4.78
264968_at	stress related protein, putative simi	---	AT1G67360	1.60	0.68	2.47	0.044435	0.109944	-4.78
262909_at	serine/threonine protein phosphata	PP2A-1	AT1G59830	-1.23	-0.30	-2.47	0.044436	0.109944	-4.78
264856_at	3-hydroxy-3-methylglutaryl-coenzy	HMG2	AT2G17370	-1.31	-0.39	-2.47	0.044442	0.109944	-4.78
256921_at	unknown protein ;supported by full-	---	AT3G18940	1.23	0.30	2.47	0.044443	0.109944	-4.78
253212_s_a	xanthine dehydrogenase - like prot	XDH1	AT4G34890	1.30	0.38	2.47	0.044445	0.109944	-4.78
252784_at	putative protein predicted proteins,	---	---	-1.17	-0.22	-2.47	0.044453	0.109953	-4.78
261233_at	hypothetical protein similar to hypot	---	AT1G32810	1.24	0.32	2.47	0.044479	0.110003	-4.78
266988_at	putative myrosinase-binding protein	JAL22	AT2G39310	-1.36	-0.45	-2.46	0.044502	0.110048	-4.78
257153_at	unknown protein ; supported by cD	---	AT3G27220	1.34	0.42	2.46	0.044512	0.110061	-4.78
247009_at	unknown protein ;supported by full-	---	AT5G67600	1.49	0.58	2.46	0.044536	0.110109	-4.78
250459_at	unknown protein ; supported by cD	---	AT5G09860	1.22	0.29	2.46	0.044573	0.110188	-4.78
266548_at	unknown protein ;supported by full-	RPA	AT2G35210	-1.34	-0.43	-2.46	0.044578	0.110188	-4.78
261357_at	hypothetical protein predicted by ge	---	---	-1.18	-0.24	-2.46	0.044594	0.110216	-4.78
264802_at	putative syntaxin-related protein (U	SYP111	AT1G08560	-1.50	-0.59	-2.46	0.044601	0.110223	-4.78
261512_at	actin capping protein beta-2, putati	---	AT1G71790	-1.36	-0.45	-2.46	0.044612	0.110238	-4.78
262745_at	lipase, putative contains Pfam prof	---	AT1G28600	-1.29	-0.36	-2.46	0.044619	0.110243	-4.78
262483_at	putative translation initiation factor	FUG1	AT1G17220	1.20	0.26	2.46	0.044641	0.110284	-4.78
255263_at	4-coumarate--CoA ligase - like prot	---	AT4G05160	1.18	0.24	2.46	0.044651	0.110297	-4.78
263268_at	unknown protein	---	---	-1.22	-0.29	-2.46	0.044657	0.110301	-4.78
247987_at	putative protein similar to unknown	---	AT5G56900	1.23	0.30	2.46	0.04467	0.11031	-4.78
250466_at	putative protein TETRATRICOPEP	---	AT5G10090	-1.19	-0.25	-2.46	0.044671	0.11031	-4.78
255373_s_a	putative protein	---	---	-1.24	-0.31	-2.46	0.044678	0.110316	-4.78

249030_at	putative protein similar to unknown	---	---	-1.19	-0.25	-2.46	0.044689	0.110316	-4.78
264656_at	unknown protein Contains similarity	---	AT1G09010	1.20	0.26	2.46	0.044691	0.110316	-4.78
261269_at	unknown protein ; supported by full-	---	AT1G26690	1.26	0.33	2.46	0.044692	0.110316	-4.78
261159_s_a	cyclin, putative similar to GB:AAB0	CYCB1;5	AT1G34460	1.27	0.34	2.46	0.044741	0.110424	-4.79
257734_at	unknown protein contains Pfam pro	ATSYTF	AT3G18370	1.17	0.23	2.46	0.044787	0.110514	-4.79
264555_at	hypothetical protein predicted by ge	---	AT1G09360	-1.21	-0.28	-2.46	0.044787	0.110514	-4.79
255516_at	hypothetical protein similar to exter	---	AT4G02270	-1.33	-0.42	-2.46	0.044817	0.110577	-4.79
264233_at	ribosomal protein, putative similar t	---	AT1G67430	1.50	0.59	2.46	0.044822	0.110577	-4.79
255012_at	putative protein ;supported by full-l	---	AT4G10030	-1.41	-0.49	-2.46	0.04486	0.110659	-4.79
259474_at	unknown protein ;supported by full-	---	AT1G19130	1.60	0.68	2.46	0.044867	0.110664	-4.79
257023_at	RNA helicase, putative similar to R	---	AT3G19760	1.22	0.28	2.46	0.044872	0.110664	-4.79
260560_at	putative endochitinase	---	AT2G43590	1.38	0.46	2.46	0.044914	0.110751	-4.79
257505_at	hypothetical protein	---	AT1G47940	-1.16	-0.22	-2.46	0.044918	0.110751	-4.79
248811_at	putative protein contains similarity t	---	AT5G47310	-1.24	-0.31	-2.46	0.044922	0.110751	-4.79
251371_at	putative protein CGI-94 protein, Ho	EDA14	AT3G60360	1.41	0.49	2.46	0.04493	0.110757	-4.79
249704_at	Myb-related transcription factor LBM	TT2	AT5G35550	-1.29	-0.37	-2.46	0.044937	0.110757	-4.79
257597_at	protein kinase, putative similar to p	---	AT3G24790	-1.19	-0.25	-2.46	0.04494	0.110757	-4.79
264832_at	unknown unknown	---	AT1G03660	1.30	0.38	2.46	0.044944	0.110757	-4.79
263986_at	putative citrate synthase ;supporte	CSY3	AT2G42790	1.30	0.38	2.46	0.04497	0.110809	-4.79
257260_at	hypothetical protein predicted by ge	---	AT3G22104	1.32	0.40	2.46	0.044978	0.110817	-4.79
262115_at	Expressed protein ; supported by fu	---	AT1G02813	-1.22	-0.28	-2.46	0.044988	0.11083	-4.79
254247_at	putative protein receptor protein kir	---	AT4G23260	1.51	0.59	2.46	0.045021	0.110899	-4.79
258232_at	unknown protein	---	AT3G27750	1.34	0.42	2.46	0.045031	0.110902	-4.79
254985_x_a	putative reverse-transcriptase -like	---	---	-1.28	-0.36	-2.46	0.045032	0.110902	-4.79
264546_at	Expressed protein ; supported by cl	---	AT1G55805	1.45	0.54	2.46	0.04507	0.110981	-4.79
251283_at	seven in absentia-like protein SINA	---	AT3G61790	1.21	0.27	2.46	0.045073	0.110981	-4.79
252092_at	mucin-like protein hemomucin, Dro	SSL4	AT3G51420	1.41	0.49	2.46	0.045099	0.111024	-4.79
264370_at	hybrid proline-rich protein, putative	---	AT1G12100	-1.22	-0.29	-2.46	0.045101	0.111024	-4.79
256157_at	longevity assurance protein, putativ	LAG13	AT1G13580	-1.30	-0.38	-2.45	0.045141	0.111108	-4.79
267111_s_a	hypothetical protein predicted by ge	---	---	-1.27	-0.35	-2.45	0.045145	0.111108	-4.79
258013_at	unknown protein	---	AT3G19320	-1.29	-0.36	-2.45	0.04517	0.111136	-4.80
265983_at	putative homeodomain transcription	HB-2	AT2G18550	-1.19	-0.26	-2.45	0.045171	0.111136	-4.80
258334_at	hypothetical protein predicted by ge	---	AT3G16010	-1.22	-0.29	-2.45	0.045173	0.111136	-4.80
267341_at	hypothetical protein predicted by ge	---	AT2G44200	1.31	0.38	2.45	0.045176	0.111136	-4.80
258146_at	putative WD-repeat protein (WDR1	---	AT3G18060	-1.25	-0.32	-2.45	0.045195	0.111171	-4.80

264454_at	unknown protein similar to U2 small	---	AT1G10320	1.25	0.33	2.45	0.045253	0.111302	-4.80
254177_at	putative protein gene T22C1.1, Ca	---	AT4G23860	1.32	0.40	2.45	0.045258	0.111302	-4.80
266836_at	hypothetical protein predicted by gr	---	AT2G26000	1.30	0.38	2.45	0.045295	0.111382	-4.80
259241_at	unknown protein similar to unknowr	---	AT3G33530	-1.18	-0.24	-2.45	0.045301	0.111385	-4.80
253331_at	nucellin -like protein nucellin - Horc	---	AT4G33490	1.34	0.42	2.45	0.045327	0.111437	-4.80
252401_at	putative protein predicted proteins,	---	AT3G48030	-1.26	-0.33	-2.45	0.045339	0.111446	-4.80
251118_at	putative chloroplast inner envelope	APG1	AT3G63410	-1.29	-0.37	-2.45	0.045341	0.111446	-4.80
245951_at	aspartate aminotransferase Asp2 ;	ASP2	AT5G19550	1.41	0.50	2.45	0.045407	0.111597	-4.80
254455_at	hypothetical protein	---	AT4G21140	1.36	0.44	2.45	0.04543	0.111628	-4.80
257038_at	longevity factor-like protein similar	LOH2	AT3G19260	1.44	0.52	2.45	0.045431	0.111628	-4.80
259812_at	unknown protein	---	AT1G49840	-1.34	-0.43	-2.45	0.045434	0.111628	-4.80
245681_at	alkaline/neutral invertase, putative	---	AT1G56560	1.18	0.24	2.45	0.045446	0.111645	-4.80
265182_at	putative auxin-induced protein ;sup	---	AT1G23740	-1.33	-0.41	-2.45	0.045466	0.111683	-4.80
261786_at	cyclic nucleotide and calmodulin-re	ATCNGC7	AT1G15990	-1.21	-0.27	-2.45	0.045483	0.111706	-4.80
255675_at	MYC1 transcription factor	ATMYC1	AT4G00480	-1.23	-0.30	-2.45	0.045486	0.111706	-4.80
265264_at	hypothetical protein predicted by ge	---	AT2G42930	-1.19	-0.25	-2.45	0.045499	0.111711	-4.80
263260_at	putative protein-tyrosine phosphata	OTS2	AT1G10570	-1.19	-0.25	-2.45	0.045502	0.111711	-4.80
253306_at	dynamin-like protein ADL2	DRP3A	AT4G33650	1.19	0.26	2.45	0.045505	0.111711	-4.80
263673_at	unknown protein	---	AT2G04800	-1.36	-0.44	-2.45	0.045507	0.111711	-4.80
257319_at	hypothetical protein	---	---	1.32	0.40	2.45	0.045513	0.111714	-4.80
250183_at	putative protein various predicted p	---	AT5G14510	-1.34	-0.43	-2.45	0.045537	0.111749	-4.80
250765_at	unknown protein ;supported by full-	---	AT5G05930	-1.34	-0.42	-2.45	0.045537	0.111749	-4.80
253745_at	putative protein the C-terminus of t	---	AT4G29090	-1.20	-0.27	-2.45	0.045562	0.111797	-4.80
258820_at	putative tryptophanyl-tRNA syntheta	---	AT3G04600	1.18	0.24	2.45	0.04558	0.111817	-4.80
265541_at	hypothetical protein predicted by ge	SQN	---	-1.19	-0.25	-2.45	0.045584	0.111817	-4.80
254283_s_a	anthocyanidin synthase - like prote	---	AT4G22870	-1.29	-0.36	-2.45	0.045585	0.111817	-4.80
263899_at	hypothetical protein predicted by ge	EMB2219	AT2G21710	1.27	0.35	2.45	0.045594	0.111828	-4.80
250682_x_a	putative protein similar to unknown	---	AT5G06630	-1.59	-0.66	-2.45	0.045633	0.111911	-4.81
255624_at	MAP kinase 4 (MPK4) ;supported b	ATMPK4	AT4G01370	1.29	0.37	2.45	0.045644	0.111926	-4.81
265447_at	putative laccase (diphenol oxidase)	LAC6	AT2G46570	-1.28	-0.35	-2.45	0.045672	0.111983	-4.81
247315_at	unknown protein	---	AT5G64010	1.22	0.28	2.45	0.045691	0.112018	-4.81
263684_at	hypothetical protein predicted by ge	---	AT1G26900	1.23	0.30	2.45	0.04572	0.112076	-4.81
265736_at	putative ribosomal protein L7 ;supp	---	AT2G01250	1.48	0.56	2.45	0.045737	0.112106	-4.81
266825_at	unknown protein	---	AT2G22890	-1.49	-0.57	-2.45	0.045765	0.112163	-4.81
245921_at	putative protein predicted protein, A	---	AT5G28800	-1.18	-0.23	-2.45	0.045783	0.112191	-4.81

245484_at	enoyl-CoA hydratase like protein	ECHIA	AT4G16210	-1.21	-0.28	-2.45	0.045786	0.112191	-4.81
262176_at	3-ketoacyl-ACP synthase, putative	FAB1	AT1G74960	-1.26	-0.33	-2.44	0.0458	0.112213	-4.81
248245_at	MtN3 protein-like	---	AT5G53190	-1.26	-0.33	-2.44	0.045808	0.11222	-4.81
257065_at	diacylglycerol pyrophosphate phos	---	AT3G18220	-1.35	-0.43	-2.44	0.04584	0.112275	-4.81
253615_at	cyclic nucleotide and calmodulin-re	CNGC17	AT4G30360	1.33	0.41	2.44	0.04584	0.112275	-4.81
254358_at	putative protein light induced prote	---	AT4G22310	1.16	0.21	2.44	0.045922	0.112463	-4.81
247564_at	RNA helicase - like protein RNA he	---	AT5G61140	1.23	0.30	2.44	0.045995	0.112627	-4.81
267270_at	hypothetical protein predicted by ge	---	AT2G02550	-1.29	-0.37	-2.44	0.045999	0.112627	-4.81
257463_at	phosphatidylinositol/phosphatidylch	---	AT1G14820	-1.28	-0.36	-2.44	0.046016	0.112647	-4.81
245224_at	gene_id:K17E7.8~unknown protein	---	AT3G29796	-1.18	-0.24	-2.44	0.04602	0.112647	-4.81
248069_at	unknown protein	---	AT5G55650	-1.23	-0.30	-2.44	0.046022	0.112647	-4.81
248725_at	acetyl-CoA:benzylalcohol acetyltran	---	AT5G47980	-1.22	-0.29	-2.44	0.046049	0.112702	-4.81
259822_at	myb-related transcription factor, pu	MYB20	AT1G66230	-1.46	-0.55	-2.44	0.046056	0.112706	-4.81
258190_at	hypothetical protein predicted by ge	---	AT3G29080	-1.19	-0.25	-2.44	0.046065	0.112708	-4.82
259897_at	putative beta-glucanase similar to b	ATCEL3	AT1G71380	-1.21	-0.27	-2.44	0.046066	0.112708	-4.82
258980_at	putative reversibly glycosylatable po	RGP3	AT3G08900	-1.20	-0.27	-2.44	0.046099	0.112767	-4.82
263138_at	hypothetical protein predicted by ge	---	AT1G65090	-1.18	-0.24	-2.44	0.0461	0.112767	-4.82
263731_at	metalloproteinase, putative similar	---	AT1G59970	-1.83	-0.88	-2.44	0.046115	0.11279	-4.82
251546_at	putative protein predicted proteins,	---	AT3G58830	1.36	0.44	2.44	0.046148	0.112859	-4.82
266137_at	unknown protein ;supported by full-	---	AT2G45010	-1.21	-0.27	-2.44	0.046168	0.112897	-4.82
258582_at	germin-like protein contains Pfam p	---	AT3G04150	-1.16	-0.22	-2.44	0.046191	0.112941	-4.82
264978_at	unknown protein contains Pfam pro	---	AT1G27120	-1.46	-0.55	-2.44	0.046244	0.113057	-4.82
252747_at	putative protein retrotransposon fin	---	---	-1.24	-0.31	-2.44	0.046252	0.113057	-4.82
249530_at	putative protein asparaginyl-tRNA s	---	AT5G38750	-1.18	-0.24	-2.44	0.046254	0.113057	-4.82
258756_at	hypothetical protein similar to hypot	---	AT3G11960	1.23	0.30	2.44	0.046295	0.113146	-4.82
253822_at	hypothetical protein	---	AT4G28410	-1.20	-0.26	-2.44	0.046308	0.113157	-4.82
261983_at	protein kinase, putative similar to c	---	AT1G33770	-1.38	-0.46	-2.44	0.046309	0.113157	-4.82
245584_at	amine oxidase like protein	ATAO1	AT4G14940	-1.17	-0.23	-2.44	0.046314	0.113157	-4.82
250561_at	glycerophosphodiester phosphodie	---	AT5G08030	-1.27	-0.35	-2.44	0.046325	0.113171	-4.82
263661_at	unknown protein EST gb|T45093 co	---	AT1G04290	-1.40	-0.48	-2.44	0.046338	0.113185	-4.82
264058_at	F-box protein family, AtFBX7 conta	---	AT2G03560	-1.19	-0.25	-2.44	0.046341	0.113185	-4.82
260374_at	unknown protein	TAF2	AT1G73960	1.21	0.27	2.44	0.04636	0.11322	-4.82
261575_at	hypothetical protein contains similar	---	AT1G01130	-1.36	-0.44	-2.44	0.046369	0.11323	-4.82
264384_at	putative chromodomain-helicase-D	PKL	AT2G25170	1.17	0.23	2.44	0.046394	0.11328	-4.82
248106_at	putative protein similar to unknown	---	AT5G55100	-1.18	-0.24	-2.44	0.046429	0.113352	-4.82

246205_at	hypothetical protein ; supported by	---	AT4G36970	1.45	0.53	2.43	0.046466	0.11343	-4.82
264870_at	hypothetical protein ;supported by	---	AT1G24210	1.24	0.31	2.43	0.046475	0.113439	-4.82
247007_at	porin-like protein ;supported by full	VDAC2	AT5G67500	-1.27	-0.35	-2.43	0.046492	0.113468	-4.82
257350_x_a	hypothetical protein predicted by ge	RALFL12	AT2G19040	-1.22	-0.28	-2.43	0.046503	0.113484	-4.82
254905_at	RPP1-WsA-like disease resistance	---	AT4G11170	1.91	0.94	2.43	0.046535	0.113539	-4.83
263027_at	hypothetical protein predicted by ge	---	AT1G24010	-1.17	-0.23	-2.43	0.04654	0.113539	-4.83
264059_at	Expressed protein ; supported by fu	INH3	AT2G31305	1.22	0.29	2.43	0.046546	0.113539	-4.83
246686_at	putative protein predicted proteins -	---	---	-1.29	-0.37	-2.43	0.046551	0.113539	-4.83
263770_at	Mutator-like transposase similar to	---	---	-1.32	-0.40	-2.43	0.046555	0.113539	-4.83
245111_at	putative Ta11-like non-LTR retroele	---	---	-1.22	-0.29	-2.43	0.046557	0.113539	-4.83
254270_at	gamma-glutamylcysteine synthetas	GSH1	AT4G23100	1.65	0.72	2.43	0.046565	0.113539	-4.83
247366_at	putative protein contains similarity t	---	AT5G63260	-1.26	-0.34	-2.43	0.046566	0.113539	-4.83
250583_at	zinc finger transcription factor ; sup	PEI1	AT5G07500	-1.20	-0.26	-2.43	0.046597	0.113592	-4.83
247892_at	putative protein contains similarity t	---	AT5G57970	-1.38	-0.47	-2.43	0.046597	0.113592	-4.83
253796_at	hypothetical protein	---	AT4G28460	2.78	1.47	2.43	0.046617	0.113627	-4.83
251031_at	one helix protein (OHP) ;supported	OHP	AT5G02120	-1.25	-0.32	-2.43	0.046639	0.113671	-4.83
254868_at	copper amine oxidase like protein (	---	AT4G12270	1.27	0.35	2.43	0.046678	0.113752	-4.83
264050_at	putative non-LTR retroelement reve	---	---	-1.30	-0.38	-2.43	0.046703	0.113796	-4.83
263424_at	putative RING zinc finger protein	---	AT2G31510	1.21	0.28	2.43	0.046706	0.113796	-4.83
247229_at	putative protein contains similarity t	---	AT5G65160	-1.34	-0.43	-2.43	0.046729	0.11384	-4.83
258106_at	ribonucleoside-diphosphate reducta	RNR2A	AT3G23580	1.27	0.34	2.43	0.046737	0.113843	-4.83
259902_at	disease resistance protein, putative	AtRLP13	AT1G74170	1.54	0.62	2.43	0.046741	0.113843	-4.83
266695_at	putative CCCH-type zinc finger prot	---	AT2G19810	-1.59	-0.67	-2.43	0.046745	0.113843	-4.83
252883_at	GTP-binding - like protein GTP-bin	---	AT4G39520	1.29	0.37	2.43	0.046804	0.113965	-4.83
249954_at	putative protein ;supported by full-l	---	AT5G18920	1.16	0.21	2.43	0.046805	0.113965	-4.83
256641_at	hypothetical protein similar to putati	---	---	-1.26	-0.34	-2.43	0.046854	0.114068	-4.83
260727_at	polygalacturonase PG1, putative si	---	AT1G48100	-1.29	-0.37	-2.43	0.046857	0.114068	-4.83
256239_at	hypothetical protein predicted by ge	---	AT3G12470	-1.24	-0.31	-2.43	0.046907	0.114167	-4.83
246127_s_a	putative protein	---	---	-1.16	-0.22	-2.43	0.046908	0.114167	-4.83
253882_at	pelota (PEL1)	PEL1	AT4G27650	-1.26	-0.34	-2.43	0.046933	0.114204	-4.83
267548_at	putative disease resistance protein	AtRLP22	AT2G32660	1.77	0.82	2.43	0.046933	0.114204	-4.83
251548_at	putative protein predicted protein, A	---	---	-1.27	-0.34	-2.43	0.046987	0.114322	-4.84
253797_at	putative protein 55.11 protein homo	RPN1B	AT4G28470	1.16	0.21	2.43	0.047006	0.114357	-4.84
251675_at	helicase-like protein DNA helicase	INO80	AT3G57300	1.18	0.24	2.43	0.047049	0.114448	-4.84
263868_at	hypothetical protein predicted by ge	---	AT2G36840	1.24	0.31	2.43	0.047081	0.114516	-4.84

252220_at	putative protein hypothetical protei	LBD38	AT3G49940	-1.38	-0.47	-2.43	0.047089	0.114522	-4.84
252389_at	Expressed protein ; supported by fu	---	AT3G47833	1.44	0.53	2.43	0.047097	0.114528	-4.84
262698_at	AMP-binding protein, putative cont	---	AT1G75960	-1.60	-0.68	-2.42	0.047136	0.114611	-4.84
265363_at	hypothetical protein predicted by ge	---	---	-1.18	-0.23	-2.42	0.047147	0.114619	-4.84
258613_at	putative myo-inositol monophospha	VTC4	AT3G02870	-1.43	-0.51	-2.42	0.047149	0.114619	-4.84
253828_at	putative protein predicted protein, A	SLAH2	AT4G27970	-1.28	-0.36	-2.42	0.047159	0.11462	-4.84
255286_at	see T14P8.10 (GB:AF069298)	---	AT4G04650	-1.32	-0.40	-2.42	0.04716	0.11462	-4.84
245467_at	hypothetical protein	---	AT4G16610	-1.35	-0.44	-2.42	0.047191	0.114684	-4.84
251826_at	ABC transporter - like protein ATP-	---	AT3G55110	-1.24	-0.31	-2.42	0.047239	0.114788	-4.84
265256_at	hypothetical protein predicted by ge	---	AT2G28390	-1.16	-0.22	-2.42	0.0473	0.114924	-4.84
267262_at	putative serine carboxypeptidase I	SNG1	AT2G22990	-1.33	-0.41	-2.42	0.047314	0.114946	-4.84
257654_at	DnaJ protein, putative contains Pfa	---	AT3G13310	1.85	0.89	2.42	0.047359	0.115043	-4.84
253353_at	pathogenesis-related protein - like	---	AT4G33730	-1.18	-0.24	-2.42	0.047396	0.115121	-4.84
252252_at	putative protein GTP-binding prote	RID3	AT3G49180	1.45	0.54	2.42	0.047413	0.115139	-4.84
250175_at	putative protein CGI-67, Homo sap	---	AT5G14390	1.42	0.51	2.42	0.047413	0.115139	-4.84
259125_at	unknown protein Pfam HMM hit: Re	---	AT3G02300	-1.18	-0.24	-2.42	0.047429	0.115156	-4.84
257632_at	cytochrome P450, putative similar t	CYP71B21	AT3G26190	1.44	0.53	2.42	0.047431	0.115156	-4.84
246642_s_a	putative protein predicted proteins,	---	---	-1.16	-0.22	-2.42	0.047486	0.115277	-4.85
266664_at	hypothetical protein predicted by gr	---	AT2G29800	-1.21	-0.28	-2.42	0.047501	0.115304	-4.85
264464_at	putative heat-shock protein similar t	---	AT1G10350	-1.24	-0.31	-2.42	0.047527	0.115354	-4.85
253706_at	putative protein D-threonine dehyd	---	AT4G29120	1.20	0.27	2.42	0.047539	0.115366	-4.85
266193_at	unknown protein	---	AT2G39080	-1.25	-0.32	-2.42	0.047542	0.115366	-4.85
245316_at	hypothetical protein ;supported by f	---	AT4G14000	1.23	0.30	2.42	0.047552	0.115376	-4.85
249559_at	putative protein	---	AT5G38320	1.31	0.39	2.42	0.047574	0.115407	-4.85
255731_at	phosphoprotein phosphatase 2A, r	RCN1	AT1G25490	-1.17	-0.22	-2.42	0.047574	0.115407	-4.85
255004_at	putative protein	---	AT4G09970	1.28	0.36	2.42	0.047632	0.115535	-4.85
261860_at	scarecrow-like protein similar to SC	SCL5	AT1G50600	1.26	0.34	2.42	0.047714	0.115722	-4.85
256773_at	hypothetical protein predicted by ge	---	AT3G13630	-1.23	-0.30	-2.42	0.047724	0.115732	-4.85
254463_at	putative protein transcription initiati	TAF11	AT4G20280	1.26	0.33	2.42	0.047731	0.115738	-4.85
264246_at	trehalose-6-phosphate synthase, p	ATTPS10	AT1G60140	-1.77	-0.82	-2.42	0.047749	0.115767	-4.85
264026_at	glycine-rich protein (AtGRP2) Same	ATGRP2B	AT2G21060	1.44	0.52	2.42	0.047753	0.115767	-4.85
248760_at	unknown protein ;supported by full-	---	AT5G47570	1.30	0.38	2.42	0.047773	0.1158	-4.85
248302_at	putative protein similar to unknown	---	AT5G53160	1.31	0.39	2.42	0.047777	0.1158	-4.85
265455_at	hypothetical protein predicted by ge	---	AT2G46560	1.20	0.26	2.41	0.047839	0.115939	-4.85
261289_at	hypothetical protein predicted by ge	---	AT1G37000	1.18	0.24	2.41	0.047845	0.115939	-4.85

258536_at	Rab escort protein, putative similar	---	AT3G06540	1.24	0.31	2.41	0.047871	0.115991	-4.85
260952_at	hypothetical protein predicted by ge	---	AT1G06140	1.31	0.39	2.41	0.047876	0.115991	-4.85
266300_at	putative auxin transport protein ; su	PIN4	AT2G01420	-1.95	-0.97	-2.41	0.047909	0.116048	-4.86
248773_at	kinesin-like protein	FRA1	AT5G47820	-1.28	-0.35	-2.41	0.04791	0.116048	-4.86
266218_s_a	putative cytochrome P450	CYP710A4	AT2G28850 ,	-1.20	-0.26	-2.41	0.047937	0.116102	-4.86
263416_at	hypothetical protein contains protein	---	AT2G17170	-1.21	-0.28	-2.41	0.047949	0.116117	-4.86
255053_at	putative protein RNA helicase -Mus	---	AT4G09730	1.62	0.70	2.41	0.04797	0.116156	-4.86
251330_at	putative protein putative protein At2	---	AT3G61550	-1.32	-0.40	-2.41	0.04805	0.116338	-4.86
264606_at	unknown protein ;supported by full-	---	AT1G04660	-1.31	-0.39	-2.41	0.048065	0.116354	-4.86
265084_at	hypothetical protein contains Cys3H	SOM	AT1G03790	-1.21	-0.27	-2.41	0.048067	0.116354	-4.86
247960_at	N-myristoyl transferase ;supported	NMT1	AT5G57020	-1.18	-0.23	-2.41	0.048075	0.116361	-4.86
264624_at	zinc finger protein ATZF1, putative	ERD6	AT1G08930	-1.31	-0.39	-2.41	0.048095	0.116387	-4.86
251504_at	putative protein putative multidrug e	TT12	AT3G59030	-1.24	-0.31	-2.41	0.048096	0.116387	-4.86
247023_at	putative protein similar to unknown	HEC1	AT5G67060	-1.30	-0.38	-2.41	0.048111	0.116411	-4.86
264212_at	putative tRNA adenylyltransferase S	---	AT1G22660	1.35	0.43	2.41	0.048127	0.116439	-4.86
267507_at	putative ribosomal protein S27 ;sup	---	AT2G45710	1.34	0.43	2.41	0.048171	0.116532	-4.86
249640_at	cationic amino acid transporter -like	CAT3	AT5G36940	-1.36	-0.44	-2.41	0.048199	0.116587	-4.86
260583_x_a	putative polyribonucleotide phospho	---	AT2G47220	1.31	0.38	2.41	0.048233	0.116658	-4.86
267527_at	unknown protein	---	AT2G45610	-1.25	-0.32	-2.41	0.04825	0.116685	-4.86
248309_at	putative protein contains similarity t	---	AT5G52540	-1.72	-0.79	-2.41	0.048255	0.116685	-4.86
256935_at	unknown protein ;supported by full-	---	AT3G22570	-1.18	-0.24	-2.41	0.048314	0.116815	-4.86
248463_at	putative protein contains similarity t	---	AT5G51130	1.57	0.66	2.41	0.048356	0.116905	-4.86
247364_at	putative protein similar to unknown	---	AT5G63200	1.18	0.24	2.41	0.048361	0.116905	-4.86
252436_x_a	putative protein several hypothetica	---	---	-1.42	-0.51	-2.41	0.048412	0.117017	-4.87
260563_at	putative glucosyltransferase	UGT74F1	AT2G43840	1.25	0.32	2.41	0.048428	0.117042	-4.87
258874_at	hypothetical protein similar to unkno	---	AT3G03230	-1.27	-0.35	-2.41	0.048453	0.117089	-4.87
252480_at	putative protein methyl-CpG-bindin	MBD5	AT3G46580	-1.17	-0.22	-2.41	0.048484	0.11714	-4.87
266693_at	unknown protein predicted by gens	MIOX2	AT2G19800	-2.80	-1.49	-2.41	0.048484	0.11714	-4.87
245971_at	putative protein ; supported by cDN	NPH4	AT5G20730	-1.20	-0.27	-2.41	0.048505	0.117178	-4.87
260039_at	CLE12 CLAVATA3/ESR-Related 12	CLE12	AT1G68795	-1.26	-0.33	-2.40	0.048535	0.117238	-4.87
264729_at	putative metal-binding protein highly	---	AT1G22990	-1.41	-0.49	-2.40	0.048569	0.117302	-4.87
254529_at	ATP binding protein - like nucleotid	INDL	AT4G19540	-1.19	-0.25	-2.40	0.048575	0.117302	-4.87
255711_at	putative WD repeat membrane prot	---	AT4G00090	1.25	0.33	2.40	0.048577	0.117302	-4.87
246405_at	disease resistance protein RPP1-W	---	AT1G57630	2.71	1.44	2.40	0.048614	0.117379	-4.87
254669_at	HhoA protease precursor, putative	DEG5	AT4G18370	-1.30	-0.38	-2.40	0.048621	0.117384	-4.87

257497_at	hypothetical protein predicted by ge	---	AT1G51430	-1.36	-0.44	-2.40	0.048645	0.117429	-4.87
248029_at	beta-amylase ; supported by cDNA	BAM4	AT5G55700	1.23	0.30	2.40	0.048657	0.117445	-4.87
267352_at	hypothetical protein predicted by ge	---	AT2G40050	-1.18	-0.24	-2.40	0.048683	0.117489	-4.87
246313_at	PPR-repeat protein, putative conta	---	AT1G31920	1.54	0.62	2.40	0.048685	0.117489	-4.87
264082_at	unknown protein ;supported by full-	---	AT2G28570	-1.21	-0.27	-2.40	0.048706	0.117513	-4.87
265104_at	unknown protein 3'-end of protein c	ATO2	AT1G31020	1.21	0.28	2.40	0.048707	0.117513	-4.87
261674_at	unknown protein	---	AT1G18270	1.41	0.49	2.40	0.04871	0.117513	-4.87
246525_at	CONSTANS ;supported by full-leng	CO	AT5G15840	1.23	0.30	2.40	0.048766	0.117633	-4.87
245544_at	UTP-glucose glucosyltransferase lik	---	AT4G15270	-1.24	-0.31	-2.40	0.04879	0.117681	-4.87
258952_at	putative RNase H contains Pfam pr	---	AT3G01410	-1.25	-0.32	-2.40	0.048799	0.117681	-4.87
253140_at	RING-H2 finger protein RHA3b ;su	RHA3B	AT4G35480	-1.27	-0.35	-2.40	0.048804	0.117681	-4.87
261083_at	unknown protein ; supported by cDI	---	AT1G07310	-1.25	-0.32	-2.40	0.048806	0.117681	-4.87
266088_at	hypothetical protein predicted by ge	---	AT2G37780	-1.28	-0.36	-2.40	0.04882	0.117704	-4.87
253986_at	putative protein predicted protein, A	---	AT4G26210	1.39	0.48	2.40	0.048872	0.117807	-4.88
263470_at	putative unconventional myosin	XIF	AT2G31900	-1.19	-0.25	-2.40	0.048874	0.117807	-4.88
264103_at	unknown protein	---	AT2G13660	1.23	0.30	2.40	0.048931	0.117932	-4.88
263089_at	putative retroelement pol polyprotei	---	---	-1.27	-0.35	-2.40	0.048949	0.117963	-4.88
262726_at	hypothetical protein similar to putati	AtTLP5	AT1G43640	-1.26	-0.33	-2.40	0.04899	0.11805	-4.88
250478_at	non-phototropic hypocotyl 3-like pr	DOT3	AT5G10250	-1.27	-0.34	-2.40	0.049053	0.118191	-4.88
254191_at	hypothetical protein ;supported by f	---	AT4G23900	-1.27	-0.34	-2.40	0.049104	0.118293	-4.88
249780_at	ubiquitin	---	AT5G24240	1.26	0.34	2.40	0.049107	0.118293	-4.88
259060_at	hypothetical protein predicted by ge	---	AT3G07400	-1.29	-0.36	-2.40	0.049119	0.118311	-4.88
254891_at	putative protein Fly Fas-associated	SAY1	AT4G11740	1.18	0.24	2.40	0.049172	0.118416	-4.88
258508_at	protein kinase, putative similar to S	---	AT3G06640	-1.16	-0.22	-2.40	0.049173	0.118416	-4.88
265062_at	receptor kinase, putative similar to	---	AT1G61550	1.39	0.48	2.40	0.049185	0.118432	-4.88
254631_at	putative protein predicted proteins,	LSH9	AT4G18610	-1.53	-0.62	-2.40	0.049208	0.118474	-4.88
251507_at	putative protein CND41, chloroplas	---	AT3G59080	-2.33	-1.22	-2.39	0.049221	0.118484	-4.88
252542_at	nuclear receptor binding factor-like	---	AT3G45770	1.18	0.23	2.39	0.049222	0.118484	-4.88
250189_at	putative protein ;supported by full-l	---	AT5G14410	-1.37	-0.46	-2.39	0.049241	0.118518	-4.88
246561_at	putative protein hypothetical protei	---	AT5G15570	1.21	0.28	2.39	0.049253	0.118534	-4.88
246288_at	unknown protein	---	AT1G31850	-1.29	-0.37	-2.39	0.049265	0.118549	-4.88
254656_at	putative protein ;supported by full-l	---	AT4G18070	-1.32	-0.40	-2.39	0.049274	0.118559	-4.88
253998_at	putative peroxidase peroxidase AT	---	AT4G26010	-1.17	-0.23	-2.39	0.049304	0.11862	-4.88
259289_at	hypothetical protein predicted by ge	---	AT3G11350	-1.24	-0.31	-2.39	0.049343	0.118689	-4.89
250031_at	transfactor-like protein ; supported	MYR1	AT5G18240	-1.32	-0.40	-2.39	0.049346	0.118689	-4.89

248845_at	disease resistance protein-like	---	AT5G46470	-1.27	-0.34	-2.39	0.049357	0.118689	-4.89
245389_at	putative protein ;supported by full-le	---	AT4G17480	-1.27	-0.34	-2.39	0.049359	0.118689	-4.89
267566_at	unknown protein predicted by gens	---	AT2G30760	-1.16	-0.21	-2.39	0.049359	0.118689	-4.89
246410_at	hypothetical protein predicted by ge	---	AT1G57760	-1.18	-0.24	-2.39	0.049411	0.118801	-4.89
265774_at	hypothetical protein predicted by ge	---	AT2G07240	-1.29	-0.37	-2.39	0.049433	0.118842	-4.89
258816_at	putative T-complex protein 1, theta	---	AT3G03960	1.32	0.41	2.39	0.049448	0.118865	-4.89
250717_at	putative protein similar to unknown	---	AT5G06200	-1.28	-0.36	-2.39	0.049479	0.118926	-4.89
258853_at	unknown protein similar to GB:AAD	---	AT3G06440	-1.20	-0.27	-2.39	0.049521	0.119007	-4.89
246135_at	putative protein non-consensus do	---	AT5G20885	-1.45	-0.54	-2.39	0.049523	0.119007	-4.89
256433_at	unknown protein	SAG20	AT3G10985	1.32	0.41	2.39	0.049531	0.119014	-4.89
252165_at	hypothetical protein ;supported by	---	AT3G50550	1.25	0.32	2.39	0.049539	0.119021	-4.89
252972_at	auxin-induced protein - like auxin-i	---	AT4G38840	-1.59	-0.67	-2.39	0.049553	0.119041	-4.89
258863_at	unknown protein similar to RNA hel	DCL2	AT3G03300	1.38	0.46	2.39	0.04957	0.119069	-4.89
258429_at	putative GTP-binding protein simila	TOC120	AT3G16620	1.21	0.28	2.39	0.049633	0.119209	-4.89
261364_at	dynamin-like protein similar to GB:U	---	AT1G53140	-1.23	-0.30	-2.39	0.049648	0.119232	-4.89
252182_at	U1 snRNP 70K protein ; supported	U1-70K	AT3G50670	1.20	0.27	2.39	0.049659	0.119247	-4.89
256014_at	hypothetical protein predicted by ge	---	AT1G19200	1.77	0.83	2.39	0.049696	0.119322	-4.89
261383_at	hypothetical protein predicted by ge	---	AT1G05380	1.23	0.30	2.39	0.049718	0.119362	-4.89
258457_at	Expressed protein ; supported by fu	IGPD	AT3G22425	1.29	0.36	2.39	0.049727	0.119373	-4.89
245751_s_a	hypothetical protein contains similar	---	AT1G25682 ,	-1.41	-0.50	-2.39	0.04974	0.119391	-4.89
253544_at	hypothetical protein	---	AT4G31280	-1.18	-0.24	-2.39	0.049835	0.119604	-4.90
247962_at	protein kinase MEK1 homolog	MKK6	AT5G56580	-1.20	-0.26	-2.39	0.049839	0.119604	-4.90
256544_at	Mlo-like protein similar to Mlo [Hord	MLO9	AT1G42560	-1.24	-0.31	-2.39	0.049857	0.119627	-4.90
249608_at	multispanning membrane protein -	---	AT5G37310	1.24	0.31	2.39	0.04986	0.119627	-4.90
265663_at	unknown protein predicted by gens	---	AT2G24290	1.24	0.31	2.39	0.049869	0.119639	-4.90
261058_at	histone, putative similar to histone	HTR12	AT1G01370	-1.18	-0.23	-2.39	0.049885	0.119662	-4.90
264687_at	cysteine protease XBCP3 identical	XBCP3	AT1G09850	-1.17	-0.23	-2.39	0.049894	0.119672	-4.90
253653_at	hypothetical protein	---	AT4G30050	-1.22	-0.28	-2.39	0.049907	0.11968	-4.90
253313_at	putative peroxidase peroxidase AT	---	AT4G33870	-1.22	-0.29	-2.39	0.049908	0.11968	-4.90
262569_at	hypothetical protein Strong similarit	---	AT1G15180	-1.36	-0.44	-2.39	0.049929	0.119715	-4.90
266148_x_a	En/Spm-like transposon protein rela	---	---	-1.24	-0.31	-2.38	0.049938	0.119715	-4.90
251847_at	tryptophan synthase alpha chain ; s	TSA1	AT3G54640	2.31	1.21	2.38	0.049938	0.119715	-4.90
257169_at	Expressed protein ; supported by fu	AtSec20	AT3G24315	-1.24	-0.31	-2.38	0.049954	0.119741	-4.90
264826_at	putative 1-aminocyclopropane-1-ca	2A6	AT1G03410	1.28	0.36	2.38	0.049969	0.119763	-4.90
257168_at	mrp protein, putative similar to mrp	HCF101	AT3G24430	-1.52	-0.61	-2.38	0.049988	0.119798	-4.90

253028_at	putative protein	pde191	AT4G38160	1.21	0.27	2.38	0.05004	0.11991	-4.90
267509_at	MADS-box protein (AGL20) ; suppo	AGL20	AT2G45660	1.32	0.40	2.38	0.050057	0.119938	-4.90
265545_at	putative protein kinase contains a p	NCRK	AT2G28250	-1.24	-0.31	-2.38	0.050105	0.12004	-4.90
245709_at	putative protein	---	AT5G04320	1.28	0.35	2.38	0.050128	0.120081	-4.90
262819_at	putative cytochrome P450 strong si	CYP77B1	AT1G11600	-1.18	-0.24	-2.38	0.050135	0.120086	-4.90
256597_at	acidic ribosomal protein P2b (rpp2b	---	AT3G28500	1.60	0.68	2.38	0.050199	0.120215	-4.90
248271_at	putative protein similar to unknown	---	AT5G53420	1.57	0.65	2.38	0.050199	0.120215	-4.90
265170_at	putative carbonic anhydrase	BCA3	AT1G23730	2.95	1.56	2.38	0.050219	0.120238	-4.90
256222_at	hypothetical protein predicted by ge	---	AT1G56210	-1.34	-0.43	-2.38	0.050219	0.120238	-4.90
256974_at	unknown protein similar to putative	---	---	-1.17	-0.23	-2.38	0.050233	0.120257	-4.90
260401_at	unknown protein similar to hypothet	---	AT1G69840	-1.26	-0.33	-2.38	0.05025	0.120264	-4.90
258436_at	putative RING zinc finger protein sir	ATL2	AT3G16720	1.71	0.77	2.38	0.050251	0.120264	-4.90
248638_at	fatty acid elongase; beta-ketoacyl-C	KCS21	AT5G49070	-1.16	-0.21	-2.38	0.050252	0.120264	-4.90
262474_at	hypothetical protein identical to hyp	FU	AT1G50240	-1.23	-0.30	-2.38	0.050272	0.120302	-4.90
247780_at	dehydrodolichyl diphosphate - like	---	AT5G58770	1.71	0.77	2.38	0.050301	0.12035	-4.90
252930_at	putative endo-1,4-beta-glucanase e	AtGH9B18	AT4G39010	-1.26	-0.34	-2.38	0.050303	0.12035	-4.91
251535_at	hypothetical protein ;supported by f	---	AT3G58540	1.32	0.40	2.38	0.050328	0.120397	-4.91
262949_at	pectinesterase, putative similar to p	sks18	AT1G75790	-1.21	-0.28	-2.38	0.05035	0.120438	-4.91
266060_at	unknown protein unusual splice site	---	AT2G18630	-1.30	-0.38	-2.38	0.050362	0.120453	-4.91
262959_at	protein translation factor Sui1 homo	---	AT1G54290	1.24	0.31	2.38	0.050384	0.120492	-4.91
256104_at	trehalose-6-phosphate synthase, p	ATTPS2	AT1G16980	-1.18	-0.24	-2.38	0.050393	0.120502	-4.91
246662_at	putative protein	---	AT5G35290	-1.21	-0.27	-2.38	0.05042	0.120541	-4.91
265307_at	hypothetical protein predicted by ge	RIN13	AT2G20310	1.23	0.30	2.38	0.05042	0.120541	-4.91
259404_at	type 2A protein serine/threonine ph	ATB BETA	AT1G17720	1.16	0.21	2.38	0.050485	0.120674	-4.91
265297_at	putative disease resistance protein	---	AT2G14080	1.18	0.24	2.38	0.050486	0.120674	-4.91
255290_at	coded for by A. thaliana cDNA AA0	ATPC1	AT4G04640	-1.16	-0.21	-2.38	0.050528	0.120762	-4.91
246279_at	homeodomain protein	ATHB40	AT4G36740	-1.30	-0.38	-2.38	0.050536	0.120768	-4.91
254531_at	putative protein predicted protein, A	---	AT4G19650	1.30	0.38	2.38	0.050576	0.120851	-4.91
246068_at	putative protein ribosomal protein S	---	AT5G20290	1.44	0.53	2.38	0.050627	0.12096	-4.91
246088_at	putative protein	---	AT5G20600	1.31	0.39	2.38	0.050638	0.120971	-4.91
264480_at	hypothetical protein predicted by ge	EFS	AT1G77300	-1.22	-0.29	-2.38	0.050647	0.120971	-4.91
252736_at	kinesin -like protein kinesin-like pro	TES	AT3G43210	-1.20	-0.26	-2.38	0.050648	0.120971	-4.91
253768_at	putative protein	---	AT4G28550	-1.35	-0.43	-2.37	0.050663	0.120995	-4.91
257344_s_a	pseudogene, putative mutator-like	---	---	-1.31	-0.39	-2.37	0.050691	0.121049	-4.91
248536_at	ankyrin-like protein	---	AT5G50140	-1.20	-0.27	-2.37	0.050796	0.121287	-4.91

265359_at	putative MYB family transcription fa	MYB7	AT2G16720	1.68	0.75	2.37	0.050812	0.121307	-4.92
251742_at	putative protein kinase serine threo	---	AT3G56050	1.20	0.26	2.37	0.050815	0.121307	-4.92
249462_at	putative protein predicted proteins,	EMB2744	AT5G39680	1.23	0.30	2.37	0.050864	0.121413	-4.92
249968_at	putative protein predicted proteins,	---	AT5G19070	-1.51	-0.60	-2.37	0.050889	0.121459	-4.92
248067_at	unknown protein	---	AT5G55600	1.26	0.33	2.37	0.050903	0.121479	-4.92
260905_at	hypothetical protein predicted by ge	---	AT1G02710	-1.16	-0.21	-2.37	0.050921	0.121509	-4.92
267425_at	putative berberine bridge enzyme	---	AT2G34810	-1.80	-0.85	-2.37	0.050976	0.121627	-4.92
AFFX-Athal-	Arabidopsis thaliana /REF=X52320	---	---	-1.30	-0.37	-2.37	0.051015	0.121709	-4.92
264977_at	unknown protein	---	AT1G27090	-1.37	-0.46	-2.37	0.051066	0.121816	-4.92
265197_at	putative glucosyl transferase	UGT73C1	AT2G36750	-1.16	-0.22	-2.37	0.051088	0.121857	-4.92
267311_at	hypothetical protein	---	AT2G34670	-1.16	-0.22	-2.37	0.051125	0.121933	-4.92
248005_at	unknown protein	---	AT5G56240	1.27	0.34	2.37	0.051133	0.121938	-4.92
260030_at	putative bZIP transcription factor sir	AtbZIP	AT1G68880	-1.20	-0.26	-2.37	0.051144	0.121952	-4.92
252862_at	putative L-ascorbate oxidase L-asc	---	AT4G39830	-1.66	-0.74	-2.37	0.051164	0.121987	-4.92
255783_at	hypothetical protein contains Pfam	iqd32	AT1G19870	1.33	0.41	2.37	0.05117	0.121989	-4.92
255890_at	rhodanese-like family protein predic	---	AT1G17850	1.39	0.47	2.37	0.051193	0.122023	-4.92
265203_at	unknown protein	---	AT2G36630	-1.25	-0.33	-2.37	0.051195	0.122023	-4.92
259517_at	hypothetical protein ; supported by	CAT1	AT1G20630	-1.35	-0.43	-2.37	0.051221	0.122064	-4.92
245494_at	salt-inducible protein homolog	---	AT4G16390	1.23	0.30	2.37	0.051223	0.122064	-4.92
257055_at	Expressed protein ; supported by fu	---	AT3G15351	1.20	0.27	2.37	0.051277	0.122179	-4.92
261165_at	dihydrolipoamide S-acetyltransfera	EMB3003	AT1G34430	-1.21	-0.28	-2.37	0.051326	0.122284	-4.93
261193_at	unknown protein ; supported by cDI	---	AT1G32920	1.75	0.80	2.37	0.051336	0.122291	-4.93
256259_at	hypothetical protein predicted by ge	---	AT3G12460	-1.29	-0.36	-2.37	0.05134	0.122291	-4.93
261457_at	Expressed protein ; supported by fu	---	AT1G21065	-1.23	-0.29	-2.37	0.051361	0.122328	-4.93
254872_at	putative protein several hypothetica	---	AT4G11560	1.19	0.25	2.37	0.051366	0.122329	-4.93
257234_at	hypothetical protein predicted by ge	---	AT3G14880	-1.28	-0.36	-2.36	0.05141	0.12242	-4.93
261411_at	histone H2B identical to GB:CAA73	HTB1	AT1G07790	1.33	0.42	2.36	0.051426	0.122441	-4.93
265942_at	putative casein kinase I ;supported	ckl5	AT2G19470	1.20	0.26	2.36	0.051429	0.122441	-4.93
248175_at	histone H2A (gb|AAF64418.1) ; su	RAT5	AT5G54640	1.22	0.29	2.36	0.05144	0.122454	-4.93
257571_at	hypothetical protein predicted by ge	---	AT3G16870	1.43	0.51	2.36	0.051452	0.12247	-4.93
249710_at	glutamate-ammonia ligase (EC 6.3	GS2	AT5G35630	-1.22	-0.28	-2.36	0.051484	0.122523	-4.93
266068_at	putative geranylgeranyl pyrophosph	GGPS4	AT2G18640	-1.25	-0.32	-2.36	0.051485	0.122523	-4.93
254013_at	putative leucine-rich-repeat protein	---	AT4G26050	-1.33	-0.41	-2.36	0.051494	0.12253	-4.93
260182_at	unknown protein similar to IFA-bind	---	AT1G70750	1.23	0.29	2.36	0.051505	0.122544	-4.93
264438_at	putative 60S ribosomal protein L17	---	AT1G27400	1.39	0.47	2.36	0.05152	0.122568	-4.93

247975_at	putative protein similar to unknown	---	AT5G56800	-1.17	-0.23	-2.36	0.051532	0.122583	-4.93
257214_at	hypothetical protein ;supported by	---	AT3G15040	-1.25	-0.33	-2.36	0.051538	0.122584	-4.93
247759_at	copper transport protein - like ;supp	COPT3	AT5G59040	-1.18	-0.23	-2.36	0.051606	0.122734	-4.93
250519_at	GDSL-motif lipase/acylhydrolase-lik	---	AT5G08460	-1.24	-0.30	-2.36	0.051623	0.12276	-4.93
255138_at	extensin-like protein extensin class	---	AT4G08380	-1.18	-0.24	-2.36	0.051635	0.122776	-4.93
251508_at	putative protein mitochondrial RNA	---	AT3G58970	1.26	0.33	2.36	0.051643	0.122782	-4.93
261956_at	oxidoreductase, putative similar to	---	AT1G64590	-1.40	-0.48	-2.36	0.051673	0.122841	-4.93
250659_at	MtN21 nodulin protein-like	---	AT5G07050	-1.25	-0.32	-2.36	0.051682	0.122847	-4.93
250164_at	putative protein various predicted p	---	AT5G15280	-1.28	-0.36	-2.36	0.051689	0.122847	-4.93
253834_at	protein phosphatase homolog (PPH	---	AT4G27800	1.24	0.31	2.36	0.051692	0.122847	-4.93
251530_at	putative protein several hypothetica	---	AT3G58520	1.30	0.38	2.36	0.051723	0.122909	-4.93
249908_at	putative protein similar to unknown	---	AT5G22760	1.19	0.26	2.36	0.051741	0.122939	-4.93
246814_at	acyl carrier protein precursor - like	ACP5	AT5G27200	-1.18	-0.24	-2.36	0.051746	0.122939	-4.93
254107_at	putative protein putative glycerol-3-	---	AT4G25220	-1.27	-0.34	-2.36	0.051758	0.122954	-4.93
252787_at	putative protein several En/Spm-like	---	---	-1.15	-0.20	-2.36	0.05177	0.122969	-4.93
259974_at	putative phosphate transporter simi	---	AT1G76430	-1.26	-0.34	-2.36	0.051781	0.122983	-4.93
265681_at	putative protein kinase contains a p	---	AT2G24370	-1.18	-0.24	-2.36	0.051792	0.122995	-4.93
250226_at	putative protein contains similarity t	---	AT5G13780	1.24	0.31	2.36	0.051797	0.122996	-4.93
264060_at	hypothetical protein predicted by ge	---	AT2G27980	1.18	0.24	2.36	0.051812	0.123008	-4.94
261711_at	unknown protein similar to hypothet	---	AT1G32700	1.22	0.29	2.36	0.051813	0.123008	-4.94
262247_at	unknown protein contains similarity	D-CDES	AT1G48420	1.20	0.26	2.36	0.051819	0.123008	-4.94
258915_at	unknown protein similar to hypothet	VPS60.1	AT3G10640	-1.26	-0.34	-2.36	0.051824	0.123008	-4.94
263350_at	alanine-glyoxylate aminotransferase	AGT	AT2G13360	1.34	0.42	2.36	0.051864	0.123089	-4.94
259674_at	thaumatin-like protein similar to thau	---	AT1G77700	-1.23	-0.30	-2.36	0.051876	0.123106	-4.94
259966_at	unknown protein	SOB3	AT1G76500	-1.22	-0.29	-2.36	0.051888	0.123122	-4.94
250530_at	unknown protein	---	AT5G08630	1.22	0.29	2.36	0.05191	0.123148	-4.94
261179_at	unknown protein	---	AT1G04985	-1.24	-0.31	-2.36	0.05191	0.123148	-4.94
258556_at	UDP-glucose:sterol glucosyltransfe	---	AT3G07020	1.24	0.31	2.36	0.051959	0.123242	-4.94
253063_at	plasma membrane-type calcium AT	ACA2	AT4G37640	1.41	0.49	2.36	0.051962	0.123242	-4.94
266306_at	hypothetical protein predicted by gr	---	AT2G26970	1.23	0.30	2.36	0.05197	0.123242	-4.94
261959_s_a	polyprotein, putative similar to poly	---	---	-1.24	-0.31	-2.36	0.051972	0.123242	-4.94
244948_at	hypothetical protein	---	---	1.18	0.24	2.36	0.051976	0.123242	-4.94
258931_at	hypothetical protein similar to hypo	DML2	AT3G10010	1.38	0.46	2.36	0.052029	0.123352	-4.94
254454_at	hypothetical protein	EMB2271	AT4G21130	-1.21	-0.28	-2.36	0.052034	0.123353	-4.94
251082_at	chlorophyll a/b-binding protein CP2	---	AT5G01530	-1.15	-0.20	-2.36	0.052046	0.123368	-4.94

246874_at	arabinosidase - like protein arabino	ASD2	AT5G26120	-1.22	-0.29	-2.36	0.052063	0.123394	-4.94
260844_at	peroxisomal targeting signal type 2	PEX7	AT1G29260	1.19	0.25	2.36	0.05209	0.123447	-4.94
245302_at	Expressed protein ; supported by cl	KAN3	AT4G17695	1.38	0.46	2.36	0.052106	0.123473	-4.94
256823_at	hypothetical protein predicted by ge	---	AT3G22220	1.24	0.31	2.36	0.052139	0.123529	-4.94
265335_at	Expressed protein ; supported by fu	---	AT2G18245	-1.16	-0.21	-2.35	0.052141	0.123529	-4.94
253989_at	uncharacterized protein ;supported	---	AT4G26130	1.35	0.43	2.35	0.052189	0.12363	-4.94
256525_at	unknown protein ;supported by full-	---	AT1G66180	-1.47	-0.55	-2.35	0.052199	0.123636	-4.94
245033_at	putative disease resistance protein	---	AT2G26380	-1.19	-0.26	-2.35	0.052202	0.123636	-4.94
245429_at	Bax inhibitor-1-like protein	---	AT4G17580	-1.16	-0.22	-2.35	0.052208	0.123636	-4.94
255074_at	putative protein RING-H2 zinc finge	---	AT4G09100	-1.22	-0.28	-2.35	0.052229	0.123668	-4.94
257084_at	unknown protein	---	AT3G20620	1.34	0.43	2.35	0.052232	0.123668	-4.94
255461_at	ent-kaurene synthetase A - like prot	GA1	AT4G02780	-1.22	-0.29	-2.35	0.052244	0.123671	-4.94
245694_at	EF - hand Calcium binding protein -	---	AT5G04170	-1.19	-0.25	-2.35	0.052244	0.123671	-4.94
247677_at	oxysterol-binding protein - like oxys	ORP3C	AT5G59420	1.18	0.24	2.35	0.052254	0.12368	-4.94
258900_at	putative 60S ribosomal protein L18	RPL18	AT3G05590	1.40	0.49	2.35	0.052288	0.12375	-4.94
256184_at	epsilon subunit of mitochondrial F1-	---	AT1G51650	1.31	0.39	2.35	0.052303	0.12377	-4.94
263834_at	hypothetical protein	---	AT2G40316	-1.23	-0.30	-2.35	0.052317	0.123791	-4.94
262373_at	hypothetical protein predicted by ge	---	AT1G73120	-1.53	-0.61	-2.35	0.052333	0.123813	-4.95
251778_at	putative protein various predicted p	ROPGEF6	AT3G55660	-1.26	-0.33	-2.35	0.052342	0.123813	-4.95
255918_at	putative protein	---	---	-1.17	-0.23	-2.35	0.052342	0.123813	-4.95
253338_at	somatic embryogenesis receptor-lil	BAK1	AT4G33430	-1.21	-0.28	-2.35	0.052353	0.123826	-4.95
261543_at	unknown protein contains Pfam pro	---	AT1G63550	-1.26	-0.33	-2.35	0.052376	0.123866	-4.95
250094_at	2-hydroxyphytanoyl-CoA lyase-like	---	AT5G17380	1.61	0.68	2.35	0.052395	0.1239	-4.95
247701_at	putative protein many predicted pro	---	AT5G59900	1.19	0.25	2.35	0.052402	0.123902	-4.95
246749_at	putative protein ; supported by cDN	---	AT5G27830	1.31	0.39	2.35	0.05242	0.123917	-4.95
252042_at	putative serine/threonine protein ki	---	AT3G51990	-1.25	-0.33	-2.35	0.052425	0.123917	-4.95
249721_at	Expressed protein ; supported by fu	LSU4	AT5G24655	1.16	0.22	2.35	0.052429	0.123917	-4.95
262400_at	unknown protein ; supported by cDI	RTV1	AT1G49480	1.16	0.22	2.35	0.05243	0.123917	-4.95
265422_at	putative NADH-ubiquinone oxireduc	NDB4	AT2G20800	-1.23	-0.30	-2.35	0.052439	0.123926	-4.95
264542_at	hypothetical protein predicted by ge	---	AT1G55830	1.17	0.23	2.35	0.05252	0.124106	-4.95
254724_at	putative protein IFA-binding protein	---	AT4G13630	1.25	0.32	2.35	0.052529	0.124113	-4.95
253204_at	GTP binding protein beta subunit ;	AGB1	AT4G34460	1.16	0.22	2.35	0.052535	0.124115	-4.95
249133_at	putative protein strong similarity to u	TAF4	AT5G43130	1.17	0.22	2.35	0.052544	0.124123	-4.95
245515_at	hypothetical protein	---	AT4G15810	1.41	0.49	2.35	0.052553	0.124131	-4.95
256390_at	putative MAP kinase similar to MAP	ATMKK8	AT3G06230	-1.18	-0.24	-2.35	0.052559	0.124132	-4.95

254340_at	putative protein hypothetical protei	---	AT4G22120	1.54	0.62	2.35	0.052581	0.124171	-4.95
253427_at	putative protein phosphate/phosph	---	AT4G32390	-1.20	-0.26	-2.35	0.052586	0.124171	-4.95
249048_at	auxin-repressed protein-like ;suppo	---	AT5G44300	-1.21	-0.27	-2.35	0.052659	0.124331	-4.95
264886_at	S-linalool synthase, putative simila	TPS04	AT1G61120	-4.07	-2.03	-2.35	0.052673	0.124349	-4.95
259652_at	unknown protein	HUB2	AT1G55250	1.30	0.38	2.35	0.052684	0.124362	-4.95
264567_s_a	putative peroxidase ATP12a Strong	---	AT1G05240	-1.23	-0.30	-2.35	0.052699	0.124385	-4.95
266329_at	unknown protein ; supported by cD	CRR3	AT2G01590	-1.47	-0.55	-2.35	0.052709	0.124396	-4.95
260701_at	heat shock transcription factor HSF	ATHSFA1D	AT1G32330	1.25	0.32	2.35	0.052715	0.124399	-4.95
249793_at	putative protein contains similarity t	---	AT5G23680	1.31	0.39	2.35	0.052767	0.124508	-4.95
260223_at	putative exonuclease contains Pfan	---	AT1G74390	1.20	0.27	2.35	0.052781	0.124527	-4.95
264320_at	unknown protein	---	AT1G04090	-1.16	-0.22	-2.35	0.052798	0.124554	-4.95
249310_at	putative protein contains similarity t	---	AT5G41520	1.23	0.30	2.35	0.052803	0.124554	-4.95
259559_at	hypothetical protein	WAK3	AT1G21240	1.34	0.42	2.35	0.052848	0.124646	-4.96
260894_at	hypothetical protein contains similar	---	AT1G29220	1.19	0.25	2.34	0.052957	0.124891	-4.96
264113_at	unknown protein	APG9	AT2G31260	1.25	0.32	2.34	0.052993	0.124964	-4.96
256657_at	unknown protein contains similarity	---	AT3G18860	1.20	0.26	2.34	0.05301	0.124991	-4.96
260793_at	unknown protein ;supported by full-	---	AT1G06190	1.21	0.27	2.34	0.053125	0.12525	-4.96
250023_at	beta-1,3-glucanase-like protein	---	AT5G18220	-1.24	-0.31	-2.34	0.053136	0.125261	-4.96
247255_at	putative protein similar to unknown	---	AT5G64780	1.43	0.52	2.34	0.053151	0.125285	-4.96
266960_at	putative retroelement pol polyprotei	---	---	-1.24	-0.31	-2.34	0.053177	0.125333	-4.96
246990_at	subtilisin-like serine protease ARA1	ARA12	AT5G67360	-1.37	-0.45	-2.34	0.053203	0.125381	-4.96
247905_at	unknown protein	---	AT5G57400	-1.16	-0.22	-2.34	0.053218	0.125403	-4.96
256266_at	unknown protein ; supported by cDI	---	AT3G12320	1.45	0.53	2.34	0.053276	0.125511	-4.96
246392_at	hypothetical protein predicted by ge	---	AT1G58120	-1.17	-0.23	-2.34	0.053279	0.125511	-4.96
261092_at	unknown protein	---	AT1G62870	-1.16	-0.21	-2.34	0.053283	0.125511	-4.96
260154_at	putative histone H2A similar to histo	HTA9	AT1G52740	1.30	0.38	2.34	0.053286	0.125511	-4.96
262204_at	unknown protein ; supported by ful	---	AT2G01100	1.20	0.26	2.34	0.053305	0.125542	-4.96
257143_at	cytochrome P450, putative contain	CYP705A2	AT3G20110	1.24	0.31	2.34	0.053331	0.125591	-4.96
256779_at	beta-fructofuranosidase, putative s	AtcwINV5	AT3G13784	-1.16	-0.21	-2.34	0.053351	0.125625	-4.96
264085_at	putative glutamyl tRNA reductase	HEMA3	AT2G31250	-1.18	-0.24	-2.34	0.053357	0.125626	-4.96
258230_s_a	hypothetical protein similar to hypo	ARI13	AT3G27710	-1.17	-0.23	-2.34	0.05343	0.125785	-4.97
260677_at	translation elongation factor EF-1 a	RNL	AT1G07910	1.38	0.47	2.34	0.053458	0.12582	-4.97
248480_at	ATP-dependent Clp protease ATP-l	CLPC1	AT5G50920	1.19	0.25	2.34	0.053461	0.12582	-4.97
260505_at	Cyclin, putative similar to GB:CAB7	CYCA3;4	AT1G47230	-1.19	-0.24	-2.34	0.053461	0.12582	-4.97
265502_at	putative poly(A) binding protein	---	AT2G15500	-1.25	-0.32	-2.34	0.053567	0.126045	-4.97

263085_at	hypothetical protein predicted by ge	ARI12	AT2G16090	1.28	0.36	2.34	0.053568	0.126045	-4.97
253282_at	putative protein hypothetical protei	LEJ1	AT4G34120	1.16	0.22	2.34	0.053618	0.126139	-4.97
267370_at	unknown protein ;supported by full-	---	AT2G44525	1.16	0.21	2.34	0.053619	0.126139	-4.97
246600_at	putative protein disease resistance	SAG101	AT5G14930	-1.43	-0.51	-2.34	0.053624	0.126139	-4.97
267614_at	putative cytochrome P450	BAS1	AT2G26710	-1.79	-0.84	-2.34	0.053635	0.126143	-4.97
262409_at	hypothetical protein predicted by ge	---	---	-1.25	-0.33	-2.34	0.053637	0.126143	-4.97
267141_at	putative MYB family transcription fa	---	AT2G38090	1.28	0.36	2.34	0.053649	0.126158	-4.97
249956_at	putative protein CGI-66 protein, Ho	---	AT5G18940	1.20	0.26	2.33	0.053671	0.126196	-4.97
266590_at	hypothetical protein predicted by ge	BAG6	AT2G46240	-1.38	-0.47	-2.33	0.05369	0.126229	-4.97
263828_at	putative GDSL-motif lipase/hydrolas	---	AT2G40250	-1.27	-0.34	-2.33	0.053723	0.126293	-4.97
249401_at	putative protein LIM7, Lily, EMBL:L	---	AT5G40260	-1.17	-0.23	-2.33	0.053749	0.126341	-4.97
263359_at	putative lysosomal acid lipase	ATLIP1	AT2G15230	1.25	0.32	2.33	0.053762	0.126358	-4.97
263567_at	hypothetical protein predicted by ge	---	AT2G15440	-1.24	-0.31	-2.33	0.05383	0.126499	-4.97
262128_at	late embryogenesis-abundant prote	---	AT1G52690	-3.17	-1.66	-2.33	0.053833	0.126499	-4.97
249255_at	Na+/H+ antiporter-like protein	CHX18	AT5G41610	1.24	0.31	2.33	0.053846	0.126507	-4.97
257518_at	hypothetical protein	---	AT3G03880	1.16	0.22	2.33	0.053847	0.126507	-4.97
245305_at	Expressed protein ; supported by fu	---	AT4G17215	1.96	0.97	2.33	0.053871	0.126543	-4.97
263971_at	hypothetical protein predicted by ge	AtRLP29	AT2G42800	-1.26	-0.33	-2.33	0.053874	0.126543	-4.97
262200_at	unknown protein similar to membra	---	AT2G01070	-1.26	-0.34	-2.33	0.053884	0.126555	-4.97
249788_at	putative protein contains similarity t	ATXR6	AT5G24330	-1.32	-0.40	-2.33	0.053893	0.126562	-4.98
267143_at	putative peptide/amino acid transpo	---	AT2G38100	-1.21	-0.28	-2.33	0.053901	0.126568	-4.98
249372_at	glucose-6-phosphate dehydrogenas	G6PD6	AT5G40760	1.43	0.52	2.33	0.053922	0.126604	-4.98
260888_at	hypothetical protein similar to allerg	---	AT1G29140	-1.19	-0.26	-2.33	0.053931	0.126612	-4.98
263938_at	hypothetical protein predicted by ge	---	AT2G35900	1.20	0.26	2.33	0.053941	0.126622	-4.98
256077_at	hypothetical protein contains similar	---	AT1G18090	-1.26	-0.33	-2.33	0.053951	0.12663	-4.98
259408_at	protein phosphatase 2A 65 kDa reg	PP2AA2	AT3G25800	1.17	0.23	2.33	0.053955	0.12663	-4.98
245034_at	putative serpin	---	AT2G26390	-1.27	-0.35	-2.33	0.053991	0.126702	-4.98
246364_at	hypothetical protein	---	---	-1.17	-0.23	-2.33	0.054005	0.126711	-4.98
260577_at	hypothetical protein predicted by ge	---	AT2G47340	-1.20	-0.26	-2.33	0.054007	0.126711	-4.98
264302_at	ATPase 70 kDa subunit, putative s	VHA-A	AT1G78900	-1.23	-0.30	-2.33	0.054032	0.126745	-4.98
246138_at	putative protein predicted proteins f	---	AT5G19870	-1.16	-0.21	-2.33	0.054032	0.126745	-4.98
261057_at	hypothetical protein predicted by ge	---	AT1G01230	-1.21	-0.27	-2.33	0.054104	0.126894	-4.98
262898_at	hypothetical protein predicted by ge	---	AT1G59850	-1.23	-0.30	-2.33	0.054107	0.126894	-4.98
260660_at	hypothetical protein contains Pfam	---	AT1G19485	1.26	0.33	2.33	0.054117	0.126905	-4.98
267165_at	putative receptor-like protein kinase	RLK	AT2G37710	1.59	0.67	2.33	0.054123	0.126906	-4.98

262671_at	calcium-dependent protein kinase,	CPK29	AT1G76040	1.28	0.36	2.33	0.054136	0.126925	-4.98
263148_at	ubiquitin, putative similar to ubiquit	---	AT1G53950	-1.22	-0.28	-2.33	0.054166	0.126982	-4.98
261942_at	unknown protein similar to putative	AGL87	AT1G22590	-1.44	-0.52	-2.33	0.054201	0.127049	-4.98
267437_at	hypothetical protein predicted by ge	---	AT2G19200	-1.20	-0.26	-2.33	0.054212	0.127057	-4.98
258794_at	ankyrin-like protein similar to ankyr	---	AT3G04710	1.51	0.60	2.33	0.054215	0.127057	-4.98
245649_at	F5A9.21 unknown protein; similar t	---	AT1G24706	1.25	0.32	2.33	0.054296	0.127233	-4.98
263352_at	En/Spm-like transposon protein rela	---	AT2G22080	1.32	0.41	2.33	0.054308	0.127233	-4.98
261403_at	unknown protein ;supported by full-	SYP52	AT1G79590	-1.17	-0.23	-2.33	0.054312	0.127233	-4.98
262646_at	aspartate aminotransferase nearly	ASP4	AT1G62800	-1.46	-0.54	-2.33	0.054325	0.127233	-4.98
264643_at	unknown protein	PGSIP5	AT1G08990	-1.18	-0.23	-2.33	0.054326	0.127233	-4.98
254341_at	serine/threonine protein kinase like	SRF8	AT4G22130	-1.65	-0.72	-2.33	0.054328	0.127233	-4.98
253783_at	putative protein predicted protein, A	NPGR2	AT4G28600	-1.22	-0.29	-2.33	0.054333	0.127233	-4.98
262253_s_a	unknown protein contains similarity	---	AT1G53880 .	1.23	0.30	2.33	0.054339	0.127233	-4.98
257795_at	hypothetical protein predicted by ge	---	AT3G15910	-1.27	-0.34	-2.33	0.05434	0.127233	-4.98
250292_at	putative protein predicted proteins,	JAZ10	AT5G13220	-2.34	-1.23	-2.33	0.054398	0.127355	-4.98
250869_at	Terminal flower1 (TFL1) ; supportec	TFL1	AT5G03840	-1.20	-0.26	-2.33	0.054413	0.127377	-4.98
254877_at	putative protein threonine dehydrat	ATSR	AT4G11640	-1.23	-0.29	-2.33	0.054422	0.127384	-4.99
265044_at	hypothetical protein Similar to hum	VPS2.3	AT1G03950	-1.26	-0.34	-2.33	0.054435	0.127402	-4.99
266970_at	hypothetical protein predicted by ge	---	AT2G39560	-1.21	-0.28	-2.33	0.054448	0.127419	-4.99
248020_at	putative protein strong similarity to u	---	AT5G56490	-1.17	-0.22	-2.32	0.054499	0.127513	-4.99
261802_at	hypothetical protein similar to hypot	---	AT1G30550	1.21	0.27	2.32	0.054502	0.127513	-4.99
252765_at	putative protein hypothetical protein	---	AT3G42800	-1.42	-0.50	-2.32	0.054505	0.127513	-4.99
260274_at	putative glycerol kinase similar to G	NHO1	AT1G80460	1.27	0.34	2.32	0.054534	0.127556	-4.99
260385_at	putative flavonol sulfotransferase si	SOT18	AT1G74090	1.26	0.33	2.32	0.054534	0.127556	-4.99
254092_at	respiratory burst oxidase - like prot	---	AT4G25090	-1.15	-0.21	-2.32	0.054541	0.127558	-4.99
258540_at	hypothetical protein predicted by ge	---	AT3G06990	-1.20	-0.26	-2.32	0.054552	0.127565	-4.99
251146_at	neoxanthin cleavage enzyme nc1	CCD1	AT3G63520	1.29	0.37	2.32	0.054555	0.127565	-4.99
252540_at	putative protein CDP-alcohol phosp	---	AT3G45740	-1.19	-0.25	-2.32	0.054599	0.127655	-4.99
253720_at	acid phosphatase-like protein acid	---	AT4G29270	-1.18	-0.24	-2.32	0.054634	0.127708	-4.99
259914_at	hypothetical protein predicted by ge	---	AT1G72640	-1.26	-0.34	-2.32	0.054643	0.127708	-4.99
258008_at	putative late embryogenesis abunda	---	AT3G19430	-1.20	-0.26	-2.32	0.054646	0.127708	-4.99
247752_at	putative protein	---	AT5G59060	-1.16	-0.21	-2.32	0.054649	0.127708	-4.99
248843_at	homeobox protein	HB-7	AT5G46880	-1.23	-0.30	-2.32	0.05465	0.127708	-4.99
245312_at	Expressed protein ; supported by fu	---	AT4G14600	1.27	0.34	2.32	0.054661	0.127721	-4.99
255060_at	putative protein TMV resistance pr	---	AT4G09430	-1.23	-0.30	-2.32	0.054674	0.127739	-4.99

267526_at	photosystem II reaction center 6.1K	PSBW	AT2G30570	-1.15	-0.21	-2.32	0.054687	0.127756	-4.99
252454_at	putative protein various predicted p	---	AT3G47130	-1.16	-0.22	-2.32	0.054707	0.12779	-4.99
266391_at	putative strictosidine synthase ident	---	AT2G41290	-1.27	-0.35	-2.32	0.054725	0.127815	-4.99
264585_at	auxin-resistance protein AXR1 E1 u	AXR1	AT1G05180	1.16	0.21	2.32	0.054731	0.127815	-4.99
253840_at	putative acyl-CoA binding protein s	ACBP2	AT4G27780	1.27	0.34	2.32	0.054735	0.127815	-4.99
254056_at	putative protein Group I Pectineste	---	AT4G25250	-1.21	-0.28	-2.32	0.054766	0.127875	-4.99
250539_at	WD-repeat protein-like ; supported	---	AT5G08560	1.26	0.33	2.32	0.054776	0.127885	-4.99
264185_at	unknown protein ;supported by full-	---	AT1G54780	-1.19	-0.25	-2.32	0.054795	0.127916	-4.99
254273_at	glycoprotein endopeptidase - like p	---	AT4G22720	1.32	0.40	2.32	0.054808	0.127935	-4.99
266252_at	hypothetical protein predicted by ge	---	AT2G27520	-1.21	-0.27	-2.32	0.054866	0.128056	-4.99
246550_at	putative protein predicted protein, A	---	AT5G14920	-2.08	-1.06	-2.32	0.05489	0.128099	-4.99
255036_at	putative protein similarity RING zin	---	AT4G09560	-1.38	-0.47	-2.32	0.054913	0.12814	-4.99
256970_at	ABC transporter, putative similar to	---	AT3G21090	-1.22	-0.28	-2.32	0.054983	0.128289	-5.00
266439_s_a	hypothetical protein predicted by gr	---	AT2G43200	1.21	0.27	2.32	0.054992	0.128298	-5.00
262206_at	putative ubiquinol-cytochrome c red	---	AT2G01090	-1.25	-0.33	-2.32	0.055011	0.128329	-5.00
265189_at	unknown protein ; supported by cD	---	AT1G23840	1.49	0.57	2.32	0.055028	0.128355	-5.00
262668_at	flavin-containing amine oxidase cor	LDL1	AT1G62830	1.21	0.27	2.32	0.055044	0.12838	-5.00
260489_at	hypothetical protein ; supported by c	---	AT1G51610	-1.19	-0.25	-2.32	0.05506	0.128405	-5.00
245908_at	laccase-like protein laccase - Acer	LAC14	AT5G09360	-1.34	-0.42	-2.32	0.05507	0.128413	-5.00
255701_at	predicted protein of unknown functio	JLO	AT4G00220	-1.18	-0.23	-2.32	0.055075	0.128413	-5.00
246546_at	voltage-dependent anion-selective c	VDAC3	AT5G15090	1.31	0.39	2.32	0.055121	0.128507	-5.00
259755_at	unknown protein similar to glucosar	---	AT1G71070	-1.24	-0.32	-2.32	0.055144	0.128548	-5.00
250575_at	putative protein	---	AT5G08240	1.39	0.48	2.32	0.055166	0.128586	-5.00
264486_at	hypothetical protein contains similar	---	AT1G77180	1.24	0.31	2.32	0.055184	0.12861	-5.00
253516_at	hypothetical protein	---	AT4G31360	-1.34	-0.42	-2.32	0.055188	0.12861	-5.00
265950_s_a	putative protein kinase contains a p	---	AT2G18530 /	-1.21	-0.27	-2.31	0.055307	0.128874	-5.00
247092_at	putative protein contains similarity t	ATFOLT1	AT5G66380	-1.25	-0.32	-2.31	0.055329	0.128908	-5.00
246651_at	adenylate kinase -like protein aden	---	AT5G35170	-1.29	-0.37	-2.31	0.055333	0.128908	-5.00
263635_at	hypothetical protein predicted by ge	---	---	-1.16	-0.21	-2.31	0.055376	0.128995	-5.00
255142_at	stromal ascorbate peroxidase ; sup	SAPX	AT4G08390	2.03	1.03	2.31	0.055381	0.128995	-5.00
266771_s_a	unknown protein	---	AT2G03010 /	1.19	0.25	2.31	0.055401	0.129028	-5.00
266010_at	putative C2H2-type zinc finger prote	---	AT2G37430	3.64	1.87	2.31	0.055413	0.129043	-5.00
257528_at	hypothetical protein predicted by ge	---	AT3G02125	-1.18	-0.24	-2.31	0.05542	0.129046	-5.00
247548_at	putative protein various predicted p	---	AT5G61400	1.28	0.36	2.31	0.055436	0.129059	-5.00
259752_at	spore coat protein-like protein simila	LPR2	AT1G71040	1.30	0.38	2.31	0.055437	0.129059	-5.00

257290_at	hypothetical protein predicted by ge	---	AT3G15550	-1.18	-0.24	-2.31	0.055449	0.129074	-5.00
251726_at	protein kinase NPK1-related protei	---	AT3G56280	-1.15	-0.20	-2.31	0.055464	0.129095	-5.00
257691_at	hypothetical protein predicted by ge	FLA14	AT3G12660	-1.30	-0.38	-2.31	0.055509	0.129187	-5.01
247103_at	putative protein similar to unknown	DAR7	AT5G66610	-1.19	-0.25	-2.31	0.05554	0.129246	-5.01
266121_at	unknown protein ; supported by cD	---	AT2G02160	1.28	0.35	2.31	0.05555	0.129257	-5.01
266930_at	hypothetical protein predicted by ge	---	AT2G45930	-1.25	-0.33	-2.31	0.055557	0.129258	-5.01
266054_at	hypothetical protein predicted by ge	---	AT2G40640	1.26	0.34	2.31	0.055584	0.129302	-5.01
251048_at	putative protein potassium channel	---	AT5G02410	1.23	0.30	2.31	0.055587	0.129302	-5.01
260205_at	hypothetical protein predicted by ge	TIFY7	AT1G70700	-1.56	-0.65	-2.31	0.055619	0.129365	-5.01
245935_at	hypothetical protein	---	AT5G19840	1.18	0.24	2.31	0.055626	0.129367	-5.01
253485_at	Expressed protein ; supported by fu	WRKY18	AT4G31800	-1.50	-0.59	-2.31	0.055642	0.129391	-5.01
252808_at	putative protein various predicted p	---	---	-1.23	-0.30	-2.31	0.055657	0.129391	-5.01
248897_at	KCO-like protein 3 (emb|CAB40380	KCO3	AT5G46360	-1.22	-0.29	-2.31	0.055658	0.129391	-5.01
255846_at	putative SWI/SNF complex subunit	ATSWI3B	AT2G33610	1.27	0.34	2.31	0.055659	0.129391	-5.01
258039_at	unknown protein ;supported by full-	---	AT3G21200	-1.20	-0.26	-2.31	0.055668	0.129398	-5.01
261718_at	wall-associated kinase, putative sir	---	AT1G18390	1.76	0.81	2.31	0.055675	0.129403	-5.01
261710_at	hypothetical protein identical to hyp	---	AT1G32730	1.24	0.30	2.31	0.055705	0.129418	-5.01
251107_at	putative protein several hypothetica	---	AT5G01610	1.25	0.32	2.31	0.055706	0.129418	-5.01
256130_at	unknown protein contains Pfam pro	---	AT1G18170	1.24	0.31	2.31	0.055706	0.129418	-5.01
247825_at	RNA/ssDNA-binding protein - like F	TAF15b	AT5G58470	-1.22	-0.29	-2.31	0.055706	0.129418	-5.01
264418_at	nuclear ribonucleoprotein, putative	---	AT1G43190	-1.30	-0.38	-2.31	0.055714	0.129418	-5.01
255628_at	coded for by A. thaliana cDNA T454	GIF3	AT4G00850	1.20	0.27	2.31	0.055716	0.129418	-5.01
245911_at	oligosaccharyl transferase STT3-lik	STT3A	AT5G19690	1.17	0.23	2.31	0.055725	0.129425	-5.01
264860_at	hypothetical protein contains ATP/G	---	AT1G24290	-1.17	-0.23	-2.31	0.05578	0.12954	-5.01
257713_at	succinate dehydrogenase iron-prot	SDH2-1	AT3G27380	1.37	0.45	2.31	0.055801	0.129575	-5.01
260237_at	putative MYB family transcription fa	MYB95	AT1G74430	-1.58	-0.66	-2.31	0.055846	0.129667	-5.01
253392_at	potassium channel protein AtKC pc	ATKC1	AT4G32650	1.81	0.86	2.31	0.055858	0.129681	-5.01
255436_at	Expressed protein ; supported by cl	---	AT4G03150	1.15	0.21	2.31	0.055869	0.129692	-5.01
266199_at	putative calcium-dependent protein	CPK20	AT2G38910	-1.18	-0.24	-2.31	0.055874	0.129692	-5.01
262364_at	disease resistance protein, putative	---	AT1G72860	-1.18	-0.23	-2.31	0.055889	0.129714	-5.01
245310_at	hypothetical protein ;supported by f	RALFL31	AT4G13950	-1.56	-0.64	-2.31	0.055924	0.129783	-5.01
259158_at	hypothetical protein predicted by g	---	AT3G05430	-1.16	-0.22	-2.31	0.055941	0.129809	-5.01
256828_at	unknown protein	---	AT3G22968	1.46	0.55	2.31	0.055961	0.12984	-5.01
259873_s_a	putative translation initiation factor I	---	AT1G76720	-1.28	-0.36	-2.31	0.056013	0.129949	-5.01
262623_at	integral membrane protein, putative	---	AT1G06470	-1.24	-0.31	-2.31	0.05604	0.129997	-5.01

259378_at	unknown protein supported by full-	---	AT3G16310	1.47	0.56	2.30	0.056066	0.130045	-5.02
258665_at	thioredoxin-like protein similar to thi	ATH9	AT3G08710	1.41	0.49	2.30	0.056108	0.13013	-5.02
257889_at	S1 self-incompatibility protein, puta	---	AT3G17080	-1.25	-0.32	-2.30	0.056142	0.130196	-5.02
252200_at	anthranilate N-hydroxycinnamoyl/b	---	AT3G50280	1.74	0.80	2.30	0.056174	0.130257	-5.02
253357_at	Dem -like protein Dem (defective e	---	AT4G33400	-1.19	-0.26	-2.30	0.056189	0.130277	-5.02
244917_at	hypothetical protein	---	---	-1.19	-0.25	-2.30	0.056209	0.13031	-5.02
245091_at	unknown protein	---	AT2G40910	-1.21	-0.28	-2.30	0.056242	0.130374	-5.02
256664_at	DNA-3-methlyadenine glycosylase	---	AT3G12040	1.27	0.35	2.30	0.056254	0.130388	-5.02
249907_at	putative protein DNA repair protein	RAD5	AT5G22750	1.18	0.24	2.30	0.056284	0.130431	-5.02
262643_at	unknown protein ;supported by full-	---	AT1G62770	-1.39	-0.48	-2.30	0.056287	0.130431	-5.02
257078_at	putative MAP kinase very similar to	---	AT3G15220	1.19	0.26	2.30	0.05629	0.130431	-5.02
252268_at	kinesin-like protein several kinesin-l	---	AT3G49650	-1.29	-0.36	-2.30	0.056314	0.130473	-5.02
250607_at	putative protein mammalian inosito	IPK2a	AT5G07370	1.15	0.21	2.30	0.056351	0.130547	-5.02
246508_at	sugar transporter-like protein putat	PGLCT	AT5G16150	-1.25	-0.33	-2.30	0.05636	0.130547	-5.02
248234_at	putative protein contains similarity t	---	AT5G53850	-1.17	-0.23	-2.30	0.056362	0.130547	-5.02
247056_at	SWI2/SNF2-like protein (gb|AAD28	CHR1	AT5G66750	1.20	0.26	2.30	0.056373	0.130559	-5.02
249168_at	putative protein strong similarity to u	---	AT5G42870	1.23	0.30	2.30	0.056478	0.130789	-5.02
264653_at	unknown protein Contains similarity	AMI1	AT1G08980	-1.30	-0.38	-2.30	0.05649	0.130802	-5.02
258859_at	Expressed protein ; supported by fu	---	AT3G02120	-1.16	-0.21	-2.30	0.056507	0.130817	-5.02
264397_at	hypothetical protein	---	AT1G11820	-1.30	-0.38	-2.30	0.056508	0.130817	-5.02
250685_at	kinesin heavy chain-like protein	---	AT5G06670	1.15	0.20	2.30	0.05655	0.130901	-5.02
260513_at	cyanogenic beta-glucosidase, puta	BGLU36	AT1G51490	-1.19	-0.25	-2.30	0.056567	0.130928	-5.02
263963_at	putative DNA-binding protein RAV2	---	AT2G36080	-1.32	-0.40	-2.30	0.056594	0.130978	-5.02
259717_at	putative cleavage and polyadenylat	CPSF73-I	AT1G61010	1.24	0.31	2.30	0.056627	0.131029	-5.03
248997_at	beta-amylase-like	BMY2	AT5G45300	1.31	0.39	2.30	0.056628	0.131029	-5.03
262659_at	putative nucleoside triphosphatase	---	AT1G14240	-1.20	-0.26	-2.30	0.056638	0.131039	-5.03
262605_at	hypothetical protein Strong similarit	---	AT1G15170	1.82	0.86	2.30	0.056651	0.131055	-5.03
267486_at	putative protein kinase contains a p	APK2B	AT2G02800	1.33	0.41	2.30	0.056701	0.131158	-5.03
250116_at	putative protein similar to unknown	---	AT5G16430	-1.27	-0.34	-2.30	0.056718	0.131178	-5.03
260388_at	hypothetical protein predicted by g	---	AT1G74070	-1.46	-0.54	-2.30	0.056721	0.131178	-5.03
247321_s_a	phosphatidylinositol 4-kinase (emb|	PI-4KBETA	AT5G09350	1.28	0.35	2.30	0.056743	0.131216	-5.03
248406_at	pectinesterase	---	AT5G51490	-1.30	-0.38	-2.30	0.056761	0.131239	-5.03
253682_at	cytidine deaminase 5 (CDA5)	---	AT4G29640	-1.25	-0.33	-2.30	0.056765	0.131239	-5.03
256270_at	unknown protein ; supported by cDI	---	AT3G12300	1.20	0.27	2.30	0.056842	0.131393	-5.03
260027_at	unknown protein	CPuORF34	AT1G29950	1.29	0.37	2.30	0.056843	0.131393	-5.03

260965_s_a	hypothetical protein predicted by ge	---	---	-1.17	-0.22	-2.29	0.056868	0.131439	-5.03
260162_at	unknown protein	GC5	AT1G79830	1.15	0.21	2.29	0.056901	0.13149	-5.03
263291_at	unknown protein	---	AT2G10920	1.20	0.26	2.29	0.056904	0.13149	-5.03
249583_at	CALMODULIN-RELATED PROTE	TCH2	AT5G37770	-1.93	-0.95	-2.29	0.056909	0.13149	-5.03
263597_at	hypothetical protein predicted by ge	---	AT2G01870	-1.59	-0.66	-2.29	0.056914	0.13149	-5.03
248249_at	protein phosphatase 2C-like ; supp	---	AT5G53140	1.19	0.25	2.29	0.056935	0.13152	-5.03
244915_s_a	hypothetical protein	---	---	-1.19	-0.25	-2.29	0.056938	0.13152	-5.03
256302_at	Expressed protein ; supported by fu	---	AT1G69526	-1.25	-0.32	-2.29	0.056949	0.131532	-5.03
266470_at	putative non-LTR retroelement reve	---	---	-1.38	-0.47	-2.29	0.056956	0.131536	-5.03
264893_at	unknown protein similar to unknown	---	AT1G23140	1.26	0.33	2.29	0.056973	0.131561	-5.03
260535_at	hypothetical protein predicted by ge	---	AT2G43390	-1.16	-0.22	-2.29	0.057009	0.131631	-5.03
263033_s_a	unknown protein ; supported by full-	---	AT1G23950	-1.34	-0.42	-2.29	0.057021	0.131634	-5.03
265386_at	unknown protein ;supported by full-	---	AT2G20930	-1.18	-0.24	-2.29	0.057022	0.131634	-5.03
249648_at	putative protein hypothetical protei	---	AT5G37050	-1.30	-0.38	-2.29	0.057053	0.131689	-5.03
263885_at	unknown protein	---	AT2G36940	-1.19	-0.25	-2.29	0.057058	0.131689	-5.03
267164_at	CER1-like protein may be involved	---	AT2G37700	-1.20	-0.26	-2.29	0.057071	0.131708	-5.03
258990_at	unknown protein ;supported by full-	---	AT3G08840	1.35	0.44	2.29	0.057086	0.131728	-5.03
249025_at	putative protein contains similarity t	---	AT5G44720	-1.40	-0.49	-2.29	0.0571	0.131746	-5.03
256020_at	glutamyl-tRNA reductase, putative	HEMA1	AT1G58290	-1.32	-0.40	-2.29	0.057113	0.131765	-5.03
256799_at	unknown protein ; supported by cD	---	AT3G18560	1.57	0.65	2.29	0.057127	0.131778	-5.03
252567_at	zinc finger -like protein zinc finger p	---	AT3G46070	-1.18	-0.24	-2.29	0.057136	0.131778	-5.03
250706_at	deoxyhypusine synthase ;supporte	DHS	AT5G05920	1.24	0.31	2.29	0.057137	0.131778	-5.03
261170_at	growth regulator protein similar to G	---	AT1G04910	-1.20	-0.27	-2.29	0.057173	0.131849	-5.04
249945_at	60S ribosomal protein L10A ; supp	---	AT5G22440	1.50	0.58	2.29	0.057184	0.13186	-5.04
264668_at	putative 2,3-bisphosphoglycerate-i	---	AT1G09780	1.27	0.35	2.29	0.057196	0.131875	-5.04
248096_at	embryo-specific protein 1; Ca2+-bin	---	AT5G55240	-1.19	-0.25	-2.29	0.057221	0.131918	-5.04
250221_at	protoporphyrinogen oxidase - like p	HEMG2	AT5G14220	1.33	0.41	2.29	0.057241	0.131945	-5.04
258410_at	putative ribosomal protein similar to	---	AT3G16780	1.57	0.65	2.29	0.057244	0.131945	-5.04
250422_at	putative protein KIAA0255 gene, H	---	AT5G10840	-1.35	-0.43	-2.29	0.057255	0.131958	-5.04
246063_at	putative protein predicted proteins,	---	AT5G19340	-1.18	-0.24	-2.29	0.057274	0.131982	-5.04
259495_at	NBS/LRR disease resistance prote	---	AT1G15890	-1.23	-0.30	-2.29	0.057281	0.131982	-5.04
255178_at	may be a pseudogene	---	---	-1.17	-0.23	-2.29	0.057283	0.131982	-5.04
258753_at	hypothetical protein predicted by ge	ATEXO70H	AT3G09530	-1.15	-0.20	-2.29	0.05729	0.131983	-5.04
264297_at	hypothetical protein similar to hypot	---	AT1G78710	-1.15	-0.20	-2.29	0.057295	0.131983	-5.04
260756_at	hypothetical protein contains simila	---	AT1G48970	1.17	0.23	2.29	0.057327	0.132038	-5.04

267463_at	Expressed protein ; supported by fu	---	AT2G33845	1.41	0.49	2.29	0.057334	0.132038	-5.04
265071_at	transcription initiation factor TFIID-2	TBP2	AT1G55520	1.20	0.27	2.29	0.057336	0.132038	-5.04
259079_at	unknown protein	---	AT3G04950	1.28	0.36	2.29	0.057349	0.132045	-5.04
247981_at	putative protein similar to unknown	MIOX5	AT5G56640	-1.17	-0.23	-2.29	0.057353	0.132045	-5.04
245715_s_a	Expressed protein ; supported by cl	---	AT5G08670 ,	1.22	0.29	2.29	0.057357	0.132045	-5.04
263490_at	F-box protein ORE9, AtFBL7 identi	MAX2	AT2G42620	-1.43	-0.51	-2.29	0.057367	0.132055	-5.04
245919_at	putative protein various predicted p	---	AT5G28780	-1.16	-0.21	-2.29	0.05739	0.132094	-5.04
251155_at	putative protein outer envelope me	---	AT3G63160	1.17	0.23	2.29	0.05742	0.13215	-5.04
264256_at	putative importin alpha similar to GI	IMPA-4	AT1G09270	-1.17	-0.23	-2.29	0.057451	0.132209	-5.04
246154_at	putative protein predicted protein, A	---	AT5G19940	-1.20	-0.26	-2.29	0.057458	0.132213	-5.04
246916_at	disease resistance protein - like dis	AtRLP52	AT5G25910	-1.16	-0.22	-2.29	0.057496	0.132276	-5.04
257562_at	hypothetical protein	---	---	-1.24	-0.31	-2.29	0.057497	0.132276	-5.04
256742_at	transcription initiation factor IIB, pu	---	AT3G29380	-1.19	-0.25	-2.29	0.057549	0.132381	-5.04
264126_at	hypothetical protein predicted by ge	NUA	AT1G79280	1.16	0.22	2.29	0.057556	0.132384	-5.04
253913_at	myosin heavy chain - like protein m	VIIIB	AT4G27370	-1.18	-0.24	-2.29	0.057572	0.132408	-5.04
245417_at	phosphoribosylglycinamide formyltr	---	AT4G17360	1.34	0.42	2.29	0.057583	0.132411	-5.04
247298_at	glucosidase II alpha subunit	RSW3	AT5G63840	1.19	0.25	2.29	0.057585	0.132411	-5.04
254291_at	putative protein UDP-galactose tra	UTR2	AT4G23010	1.33	0.41	2.28	0.057705	0.132666	-5.04
261065_at	hypothetical protein predicted by ge	---	AT1G07500	-1.27	-0.35	-2.28	0.057712	0.132666	-5.04
255680_at	hypothetical protein	---	AT4G00530	1.26	0.34	2.28	0.057713	0.132666	-5.04
252131_at	BCS1 protein-like protein Homo sa	BCS1	AT3G50930	2.38	1.25	2.28	0.057758	0.132755	-5.05
257701_at	unknown protein contains similarity	---	AT3G12710	-2.12	-1.09	-2.28	0.057769	0.132767	-5.05
254047_at	hypothetical protein	---	AT4G26040	-1.26	-0.34	-2.28	0.057781	0.13278	-5.05
265145_at	unknown protein similar to GB:CAA	PLT2	AT1G51190	-1.21	-0.28	-2.28	0.057794	0.132797	-5.05
256690_at	hypothetical protein predicted by ge	---	AT3G32160	-1.24	-0.31	-2.28	0.057848	0.132907	-5.05
261902_at	unknown protein ; supported by full-	PLMT	AT1G80860	1.18	0.24	2.28	0.057874	0.132955	-5.05
252485_at	disease resistance protein RPP13-	RPP13	AT3G46530	1.20	0.26	2.28	0.057894	0.132987	-5.05
264292_at	hypothetical protein predicted by ge	---	AT1G78790	1.24	0.31	2.28	0.05792	0.133034	-5.05
249055_at	calmodulin-like protein	---	AT5G44460	-1.16	-0.21	-2.28	0.057929	0.13304	-5.05
258194_at	unknown protein ; supported by ful	---	AT3G29170	-1.28	-0.36	-2.28	0.057936	0.133044	-5.05
260231_at	putative RING zinc finger protein co	---	AT1G74410	1.27	0.34	2.28	0.057959	0.133079	-5.05
263324_at	hypothetical protein predicted by ge	---	AT2G04220	-1.18	-0.24	-2.28	0.057964	0.133079	-5.05
267477_at	putative receptor-like protein kinase	PLPB	AT2G02710	-1.42	-0.51	-2.28	0.057969	0.133079	-5.05
262908_at	pyruvate dehydrogenase e1 alpha	AT-E1 ALP	AT1G59900	1.19	0.25	2.28	0.057978	0.133087	-5.05
265934_at	unknown protein	EER5	AT2G19560	1.16	0.21	2.28	0.058001	0.133113	-5.05

249626_at	putative protein nucleoid DNA-bind	---	AT5G37540	-1.43	-0.52	-2.28	0.058001	0.133113	-5.05
266923_at	unknown protein ;supported by full-	---	AT2G45980	1.20	0.27	2.28	0.058007	0.133113	-5.05
245031_at	putative mitochondrial carrier protei	---	AT2G26360	-1.17	-0.22	-2.28	0.05802	0.13313	-5.05
254636_at	putative protein kinase ; supported	CIPK12	AT4G18700	1.17	0.23	2.28	0.058044	0.13317	-5.05
253967_at	putative protein probable membrar	---	AT4G26550	-1.31	-0.39	-2.28	0.058052	0.133175	-5.05
251814_at	chlorophyll a/b-binding protein ; su	LHCA1	AT3G54890	-1.15	-0.20	-2.28	0.05812	0.133319	-5.05
252843_at	glycine-rich protein other glycine-ric	---	AT3G42130	-1.26	-0.34	-2.28	0.058226	0.133549	-5.05
260008_at	putative RING zinc finger protein c	---	AT1G68070	1.18	0.24	2.28	0.058254	0.133599	-5.05
262214_at	germin subunit, putative similar to g	---	AT1G74820	-1.18	-0.24	-2.28	0.058266	0.13361	-5.05
260174_at	unknown protein ;supported by full-	---	AT1G71940	-1.18	-0.24	-2.28	0.058271	0.13361	-5.05
257368_at	hypothetical protein predicted by ge	---	AT2G29860	-1.18	-0.24	-2.28	0.058289	0.133638	-5.05
249566_at	cytochrome P450-like protein	CYP735A1	AT5G38450	-1.19	-0.25	-2.28	0.058301	0.133652	-5.05
248072_at	unknown protein	---	AT5G55680	-1.21	-0.28	-2.28	0.058311	0.133663	-5.05
262738_at	hypothetical protein	---	AT1G28530	1.25	0.32	2.28	0.058343	0.133712	-5.06
258128_at	chloroplast thylakoidal processing	PLSP1	AT3G24590	-1.25	-0.33	-2.28	0.058349	0.133712	-5.06
252789_s_a	hypothetical protein ; supported by	---	AT1G21930	1.17	0.23	2.28	0.05835	0.133712	-5.06
250108_at	homeobox-leucine zipper protein H/	ATHB-3	AT5G15150	-1.26	-0.33	-2.28	0.058397	0.133806	-5.06
249662_s_a	putative protein hypothetical protein	---	AT5G36680	-1.15	-0.20	-2.28	0.058529	0.134095	-5.06
261440_at	hypothetical protein predicted by ge	---	AT1G28510	-1.19	-0.25	-2.27	0.058575	0.134187	-5.06
255038_at	neutral invertase like protein Dauc	CINV2	AT4G09510	-1.51	-0.60	-2.27	0.058608	0.13425	-5.06
248874_at	putative protein strong similarity to	---	AT5G46460	1.32	0.40	2.27	0.058621	0.134266	-5.06
246080_at	putative protein	---	AT5G20460	1.19	0.25	2.27	0.058631	0.134274	-5.06
256420_at	unknown protein	---	AT1G33475	-1.26	-0.34	-2.27	0.058637	0.134275	-5.06
260572_at	putative molybdopterin synthase lar	---	AT2G43760	-1.18	-0.24	-2.27	0.058653	0.134297	-5.06
267327_at	putative protein kinase contains a p	---	AT2G19410	-1.18	-0.23	-2.27	0.058695	0.134373	-5.06
258882_at	unknown protein ; supported by ful	---	AT3G06330	1.22	0.28	2.27	0.058697	0.134373	-5.06
259256_at	putative coated vesicle membrane	---	AT3G07680	1.19	0.25	2.27	0.058716	0.134391	-5.06
254065_at	gibberellin 20-oxidase - Arabidopsis	GA20OX1	AT4G25420	-1.59	-0.67	-2.27	0.058717	0.134391	-5.06
251201_at	putative protein predicted protein, /	---	AT3G63020	-1.16	-0.21	-2.27	0.058755	0.134463	-5.06
252318_at	glutamate-1-semialdehyde aminotra	GSA2	AT3G48730	-1.33	-0.42	-2.27	0.058786	0.134519	-5.06
256067_at	unknown protein ;supported by full-	---	AT1G07170	1.28	0.35	2.27	0.058791	0.134519	-5.06
246885_at	putative protein predicted protein, /	---	AT5G26230	-1.62	-0.70	-2.27	0.058808	0.134544	-5.06
254456_at	putative protein ribophorin II precur	HAP6	AT4G21150	1.24	0.31	2.27	0.058835	0.134592	-5.06
251475_at	putative protein other hypothetical p	---	AT3G59660	1.36	0.44	2.27	0.058889	0.134703	-5.06
264641_at	ATP-dependent Clp protease prote	---	AT1G09130	1.23	0.29	2.27	0.0589	0.134714	-5.07

267239_at	unknown protein	---	AT2G02510	1.21	0.27	2.27	0.058927	0.134751	-5.07
260723_at	unknown protein	---	AT1G48070	-1.17	-0.23	-2.27	0.058928	0.134751	-5.07
257550_at	hypothetical protein similar to GB:A	---	AT3G18460	-1.24	-0.31	-2.27	0.058958	0.134807	-5.07
255056_at	putative protein helix-loop-helix pro	TT8	AT4G09820	-1.16	-0.21	-2.27	0.059014	0.134921	-5.07
246043_at	putative protein predicted protein, A	---	AT5G19380	-1.41	-0.49	-2.27	0.059024	0.13493	-5.07
266709_at	unknown protein ;supported by full-	ATSPP	AT2G03120	1.29	0.37	2.27	0.059037	0.13493	-5.07
261462_at	hypothetical protein predicted by ge	---	AT1G07850	-1.23	-0.30	-2.27	0.059041	0.13493	-5.07
250946_at	putative protein	---	AT5G03420	1.18	0.24	2.27	0.059042	0.13493	-5.07
254410_at	serine/threonine kinase - like protei	---	AT4G21410	1.63	0.71	2.27	0.059069	0.134979	-5.07
246867_at	putative protein various predicted p	---	AT5G25990	1.16	0.22	2.27	0.059086	0.134993	-5.07
253119_at	Expressed protein ; supported by fu	---	AT4G35980	1.18	0.24	2.27	0.059087	0.134993	-5.07
266848_at	unknown protein ;supported by full-	---	AT2G25950	1.29	0.36	2.27	0.059117	0.135047	-5.07
245869_at	hypothetical protein predicted by ge	---	AT1G26330	-1.18	-0.24	-2.27	0.059162	0.135137	-5.07
245074_at	putative protein kinase contains a p	---	AT2G23200	1.87	0.91	2.27	0.0592	0.1352	-5.07
256144_at	guanine nucleotide-binding protein	RACK1B_A	AT1G48630	1.68	0.75	2.27	0.059207	0.1352	-5.07
249676_at	serine/threonine protein kinase-like	---	AT5G35960	-1.23	-0.30	-2.27	0.059207	0.1352	-5.07
250789_at	putative protein contains similarity t	---	AT5G05630	1.21	0.27	2.27	0.059231	0.135242	-5.07
254009_at	pyruvate kinase like protein pyruva	---	AT4G26390	-1.33	-0.42	-2.27	0.05925	0.135271	-5.07
262798_at	putative SPL1-related protein simila	SPL14	AT1G20980	1.21	0.27	2.27	0.059288	0.135323	-5.07
262126_at	disease resistance protein, putative	CW9	AT1G59620	1.29	0.37	2.27	0.05929	0.135323	-5.07
247806_at	ABC transporter-like protein ; suppo	STA1	AT5G58270	-1.18	-0.24	-2.27	0.059291	0.135323	-5.07
259112_at	putative 60S ribosomal protein L22	---	AT3G05560	1.44	0.53	2.27	0.059357	0.13546	-5.07
257182_s_a	crn-like protein similar to crn GB:CA	---	AT3G13210	1.28	0.36	2.27	0.059372	0.135482	-5.07
264766_at	receptor kinase, putative similar to	---	AT1G61420	1.50	0.58	2.26	0.059394	0.135508	-5.07
251817_at	small GTP-binding protein - like (fra	ARA6	AT3G54840	1.17	0.23	2.26	0.059395	0.135508	-5.07
265986_at	putative receptor-like protein kinase	---	AT2G24230	-1.31	-0.38	-2.26	0.059406	0.135519	-5.07
260521_at	putative villin 2	VLN2	AT2G41740	-1.33	-0.41	-2.26	0.059426	0.13555	-5.07
255796_at	similar to mammalian MHC III regio	SUVH2	AT2G33290	1.20	0.27	2.26	0.059456	0.135606	-5.07
265099_at	unknown protein Location of EST 2	---	AT1G03990	-1.60	-0.68	-2.26	0.059495	0.135676	-5.08
260257_at	putative dolichyl-phosphate mannos	---	AT1G74340	-1.18	-0.24	-2.26	0.059499	0.135676	-5.08
247664_at	APETALA2 protein - like APETALA	TOE2	AT5G60120	-1.20	-0.26	-2.26	0.059512	0.135693	-5.08
253176_at	putative protein Human (H326) mR	---	AT4G35140	1.32	0.40	2.26	0.059542	0.135747	-5.08
252524_at	putative protein mitochondrial ATP	---	AT3G46430	1.23	0.29	2.26	0.059556	0.135766	-5.08
265445_at	60S ribosomal protein L12 ;suppor	---	AT2G37190	1.62	0.70	2.26	0.059565	0.135772	-5.08
245485_at	proline-rich, APG like protein	---	AT4G16230	-1.22	-0.29	-2.26	0.059606	0.135844	-5.08

250655_at	transcription factor HBP-1b (gb|AAD	OBF5	AT5G06960	1.17	0.23	2.26	0.05961	0.135844	-5.08
263573_at	unknown protein similar to A. thaliar	---	AT2G17150	1.28	0.36	2.26	0.059614	0.135844	-5.08
245006_at	PSI P700 apoprotein A2	---	---	-1.15	-0.20	-2.26	0.059631	0.135868	-5.08
262523_at	ubiquitin-specific protease 15 (UBF	UBP15	AT1G17110	-1.16	-0.21	-2.26	0.059652	0.135899	-5.08
265454_at	putative ARF1 family auxin respons	ARF11	AT2G46530	1.76	0.82	2.26	0.059656	0.135899	-5.08
265670_s_a	unknown protein ;supported by full-	---	AT2G32190	1.76	0.81	2.26	0.059683	0.135946	-5.08
266694_at	putative clathrin assembly protein ;	---	AT2G19790	-1.16	-0.21	-2.26	0.059708	0.135989	-5.08
256048_at	hypothetical protein contains similar	---	AT1G07025	-1.18	-0.24	-2.26	0.059744	0.136057	-5.08
253057_at	putative protein amino-acid N-acety	---	AT4G37670	1.66	0.73	2.26	0.059759	0.13607	-5.08
265885_at	hypothetical protein predicted by ge	---	AT2G42330	-1.28	-0.36	-2.26	0.059761	0.13607	-5.08
262781_s_a	proteasome epsilon chain precurso	PBE1	AT1G13060	1.24	0.32	2.26	0.059867	0.136297	-5.08
247797_at	predicted protein	---	AT5G58780	-1.23	-0.30	-2.26	0.059876	0.136304	-5.08
254512_at	terpene cyclase like protein vetispi	---	AT4G20230	-1.21	-0.28	-2.26	0.059901	0.136347	-5.08
260448_at	hypothetical protein predicted by ge	---	AT1G72410	-1.22	-0.29	-2.26	0.059914	0.136364	-5.08
246166_at	putative protein	---	---	-1.20	-0.26	-2.26	0.059991	0.136525	-5.08
246415_at	putative protein (fragment) predicte	---	AT5G17160	-1.33	-0.41	-2.26	0.060087	0.136729	-5.09
245476_at	hypothetical protein	---	AT4G16090	-1.21	-0.27	-2.26	0.060098	0.136742	-5.09
245421_at	hypothetical protein	---	AT4G17430	1.16	0.21	2.26	0.060179	0.136901	-5.09
251878_at	putative protein predicted protein, A	---	AT3G54310	-1.16	-0.22	-2.26	0.06018	0.136901	-5.09
256049_at	unknown protein	---	AT1G07010	1.27	0.34	2.26	0.060195	0.136921	-5.09
265697_at	hypothetical protein predicted by ge	---	AT2G32170	1.18	0.24	2.26	0.060232	0.136991	-5.09
260341_at	squamosa-promoter binding protei	---	AT1G69170	1.28	0.36	2.26	0.060239	0.136993	-5.09
261766_at	auxin-induced protein IAA5, putativ	IAA5	AT1G15580	-1.69	-0.76	-2.25	0.060292	0.1371	-5.09
250572_at	putative protein	MIR834A	---	1.28	0.35	2.25	0.060302	0.137111	-5.09
255129_at	nodulin-like protein nodulin gene M	---	AT4G08290	1.56	0.64	2.25	0.060341	0.137186	-5.09
264607_at	putative K+ channel, beta subunit s	KAB1	AT1G04690	1.15	0.21	2.25	0.060369	0.137224	-5.09
262390_at	hypothetical protein predicted by ge	---	AT1G49260	-1.22	-0.29	-2.25	0.06037	0.137224	-5.09
252422_at	putative protein PIT1 - Arabidopsis	---	AT3G47550	-1.31	-0.39	-2.25	0.060422	0.137328	-5.09
257659_at	Expressed protein ; supported by fu	---	AT3G13226	1.16	0.22	2.25	0.060442	0.137361	-5.09
257432_at	unknown protein	---	AT2G21850	-1.25	-0.32	-2.25	0.060489	0.137452	-5.09
261072_at	hexose transporter, putative similar	ATSTP2	AT1G07340	-1.24	-0.31	-2.25	0.060502	0.137456	-5.09
255729_at	hypothetical protein similar to putati	---	AT1G25470	1.20	0.26	2.25	0.060502	0.137456	-5.09
245407_at	GTP-binding RAB2A like protein	RABB1C	AT4G17170	-1.19	-0.25	-2.25	0.060523	0.13749	-5.09
260584_at	putative beta-1,3-glucanase, C terr	---	AT2G43660	-1.18	-0.23	-2.25	0.060534	0.137501	-5.09
261531_at	RB-binding protein -like similar to re	---	AT1G63490	1.30	0.38	2.25	0.060549	0.137508	-5.09

265785_at	hypothetical protein predicted by ge	---	AT2G07290	-1.16	-0.21	-2.25	0.060549	0.137508	-5.09
258459_at	hypothetical protein predicted by g	---	---	-1.21	-0.27	-2.25	0.060579	0.137556	-5.09
267474_at	hypothetical protein predicted by ge	WHY3	AT2G02740	1.22	0.29	2.25	0.060585	0.137556	-5.09
250416_at	putative protein predicted proteins,	---	AT5G11220	-1.19	-0.26	-2.25	0.060588	0.137556	-5.09
263097_at	hypothetical protein predicted by ge	PDV2	AT2G16070	1.25	0.33	2.25	0.060648	0.137677	-5.09
248994_at	disease resistance protein-like	RPS4	AT5G45250	1.26	0.33	2.25	0.060658	0.137686	-5.09
252606_at	carboxypeptidase precursor-like pr	scpl48	AT3G45010	-1.60	-0.68	-2.25	0.060706	0.137782	-5.10
261164_at	hypothetical protein ;supported by f	---	AT1G34470	-1.21	-0.28	-2.25	0.060743	0.137838	-5.10
248527_at	putative protein contains similarity t	---	AT5G50740	-1.21	-0.27	-2.25	0.060743	0.137838	-5.10
256988_s_a	adenosine triphosphatase, putative	---	AT3G28710	-1.16	-0.21	-2.25	0.060766	0.137875	-5.10
255260_at	score=63.7, E=3.9e-15, N=8 ; sup	---	AT4G05040	1.25	0.33	2.25	0.060774	0.137875	-5.10
251463_at	hypothetical protein	---	AT3G59800	-1.21	-0.27	-2.25	0.060782	0.137875	-5.10
248972_at	putative protein similar to unknown	ATDSS1(V	AT5G45010	1.35	0.43	2.25	0.060784	0.137875	-5.10
254960_at	predicted protein	---	AT4G10845	-1.22	-0.28	-2.25	0.060814	0.13793	-5.10
253851_at	putative transcription factor MYB41	AtMYB41	AT4G28110	-1.23	-0.30	-2.25	0.06082	0.13793	-5.10
265337_at	putative ADP-ribosylation factor ;su	TTN5	AT2G18390	-1.32	-0.40	-2.25	0.060886	0.13806	-5.10
245876_at	chaperonin precursor, putative sim	---	AT1G26230	1.38	0.47	2.25	0.060889	0.13806	-5.10
254294_at	putative membrane protein membr	ATRBL7	AT4G23070	-1.40	-0.48	-2.25	0.060903	0.138077	-5.10
256108_at	hypothetical protein predicted by ge	---	AT1G16940	-1.28	-0.36	-2.25	0.060911	0.138081	-5.10
245965_at	pectin methylesterase-like protein	---	AT5G19730	-1.39	-0.47	-2.25	0.060916	0.138081	-5.10
262290_at	Expressed protein ; supported by fu	---	AT1G70985	-1.31	-0.38	-2.25	0.060926	0.138089	-5.10
245900_at	putative protein predicted proteins,	---	AT5G11040	-1.23	-0.30	-2.25	0.060948	0.138124	-5.10
265486_at	unknown protein predicted by gens	---	AT2G15560	1.25	0.32	2.25	0.060964	0.138147	-5.10
252463_at	putative protein ;supported by full-l	---	AT3G47070	-1.43	-0.52	-2.25	0.060971	0.138148	-5.10
259096_at	putative 40S ribosomal protein S3A	---	AT3G04840	1.50	0.59	2.25	0.061017	0.138237	-5.10
262612_at	unknown protein location of ESTs	---	AT1G14150	-1.58	-0.66	-2.25	0.061022	0.138237	-5.10
264994_at	hypothetical protein contains similar	---	AT1G67270	-1.18	-0.23	-2.25	0.061045	0.138275	-5.10
264945_at	MADS-box protein (MADS2), putat	MAF1	AT1G77080	-1.44	-0.53	-2.25	0.061082	0.138333	-5.10
253888_at	hypothetical protein	ISI1	AT4G27750	-1.17	-0.23	-2.25	0.061087	0.138333	-5.10
249239_at	MAP kinase ; supported by cDNA:	CPuORF58	AT5G41990	1.17	0.23	2.25	0.061089	0.138333	-5.10
263917_at	putative AP2 domain transcription fa	HRD	AT2G36450	-1.30	-0.38	-2.25	0.061101	0.138347	-5.10
262348_at	unknown protein similar to transcrip	---	AT2G48160	1.17	0.23	2.25	0.061131	0.138403	-5.10
266569_at	unknown protein	---	AT2G24080	-1.17	-0.23	-2.24	0.061167	0.138465	-5.10
260288_at	nodulin-like protein similar to nodule	---	AT1G80530	1.32	0.40	2.24	0.061171	0.138465	-5.10
253598_at	ribosomal protein S11 - like ribosor	---	AT4G30800	1.66	0.73	2.24	0.061178	0.138468	-5.10

248337_at	low-temperature-induced protein 78	LTI78	AT5G52310	-2.50	-1.32	-2.24	0.061226	0.138562	-5.10
256497_at	ORF1, putative similar to ORF1 GI	ECS1	AT1G31580	-1.44	-0.52	-2.24	0.061261	0.138626	-5.10
251602_at	Expressed protein ; supported by fu	---	AT3G57785	1.19	0.25	2.24	0.061268	0.13863	-5.10
265660_at	putative disease resistance protein	AtRLP21	AT2G25470	1.30	0.38	2.24	0.061295	0.138674	-5.11
253865_at	putative RING zinc finger protein A	---	AT4G27470	-1.32	-0.40	-2.24	0.0613	0.138674	-5.11
262876_at	unknown protein ;supported by full-	ATDSS1(I)	AT1G64750	1.26	0.33	2.24	0.06132	0.138705	-5.11
265951_at	hypothetical protein predicted by ge	OFP7	AT2G18500	-1.35	-0.43	-2.24	0.061328	0.13871	-5.11
256277_at	omega-6 fatty acid desaturase, enc	FAD2	AT3G12120	-1.37	-0.45	-2.24	0.061356	0.13876	-5.11
252699_at	hypothetical protein predicted prote	---	---	-1.36	-0.44	-2.24	0.061499	0.139068	-5.11
250964_at	putative protein various predicted p	---	AT5G03010	-1.24	-0.31	-2.24	0.061515	0.139091	-5.11
259169_at	unknown protein similar to unknown	---	AT3G03520	1.75	0.80	2.24	0.061533	0.139118	-5.11
265588_at	putative pathogenesis-related prote	---	AT2G19970	-1.96	-0.97	-2.24	0.061543	0.13912	-5.11
260305_at	putative ADP-ribosylation factor 1 n	ARFA1D	AT1G70490	-1.23	-0.30	-2.24	0.061546	0.13912	-5.11
266851_at	similar to avrRpt2-induced protein 1	ATPP2-A3	AT2G26820	-1.15	-0.20	-2.24	0.061584	0.139193	-5.11
247761_at	phosphoprotein phosphatase 1 cata	TOPP2	AT5G59160	-1.20	-0.27	-2.24	0.06162	0.139261	-5.11
257908_at	unknown protein contains Pfam pro	---	AT3G25410	1.21	0.28	2.24	0.061649	0.139306	-5.11
253087_at	purple acid phosphatase like protein	PAP25	AT4G36350	-1.22	-0.28	-2.24	0.061653	0.139306	-5.11
257336_at	hypothetical protein	---	---	1.16	0.22	2.24	0.061668	0.139317	-5.11
250460_at	putative protein similar to unknown	---	AT5G09850	-1.17	-0.23	-2.24	0.06167	0.139317	-5.11
260555_at	hypothetical protein predicted by ge	---	AT2G41780	1.63	0.71	2.24	0.061685	0.139338	-5.11
260128_at	hypothetical protein similar to Yml0	---	AT1G36310	1.21	0.28	2.24	0.061703	0.139364	-5.11
251580_at	putative protein ethylene-responsiv	---	AT3G58450	-1.30	-0.38	-2.24	0.061746	0.139449	-5.11
255086_at	putative protein chromosome I BAC	---	AT4G09300	-1.22	-0.29	-2.24	0.061811	0.13958	-5.11
254607_at	hypothetical protein ;supported by f	---	AT4G18920	-1.18	-0.23	-2.24	0.061841	0.139634	-5.11
253103_at	putative auxin-induced protein high	---	AT4G36110	-2.39	-1.25	-2.24	0.061857	0.139657	-5.11
266398_at	hypothetical protein predicted by ge	---	AT2G38680	-1.49	-0.57	-2.24	0.061877	0.139689	-5.11
250444_at	beta-xylosidase - like protein beta-x	---	AT5G10560	-1.21	-0.28	-2.24	0.061888	0.139699	-5.12
250588_at	SMC-like protein	---	AT5G07660	-1.18	-0.24	-2.24	0.061944	0.139812	-5.12
245045_at	unknown protein similar to glycopro	---	AT2G26590	1.16	0.22	2.24	0.061958	0.139819	-5.12
260603_at	membrane related protein CP5, pu	---	AT1G55960	1.32	0.40	2.24	0.061959	0.139819	-5.12
250927_at	lysine decarboxylase - like protein	---	AT5G03270	-1.26	-0.33	-2.24	0.061978	0.139844	-5.12
252337_at	CELL DIVISION CONTROL PROTE	CDC2	AT3G48750	1.34	0.42	2.24	0.061983	0.139844	-5.12
248340_at	putative protein similar to unknown	---	AT5G52180	-1.19	-0.25	-2.24	0.061998	0.139865	-5.12
246512_at	putative phytochelatin synthetase p	IRX6	AT5G15630	-1.25	-0.33	-2.24	0.062009	0.139874	-5.12
252216_at	putative protein several hypothetica	---	AT3G50420	1.24	0.32	2.24	0.062014	0.139874	-5.12

246909_at	putative protein predicted protein, (	---	AT5G25770	1.54	0.63	2.23	0.062069	0.139973	-5.12
252640_at	acyl CoA reductase -like protein ac	FAR8	AT3G44560	-1.17	-0.23	-2.23	0.062071	0.139973	-5.12
265291_at	unknown protein predicted by gens	---	AT2G22720	-1.20	-0.27	-2.23	0.062145	0.140105	-5.12
265102_at	putative peroxidase similar to cation	---	AT1G30870	-1.21	-0.28	-2.23	0.062147	0.140105	-5.12
253258_at	putative protein NF-180, Petromyz	---	AT4G34400	-1.23	-0.30	-2.23	0.062151	0.140105	-5.12
263372_at	60S ribosomal protein L14 ;suppor	---	AT2G20450	1.48	0.57	2.23	0.062165	0.140105	-5.12
254146_at	endo-1,4-beta-glucanase like prote	ATGH9A3	AT4G24260	-1.24	-0.31	-2.23	0.062172	0.140105	-5.12
254391_at	putative bifunctional nuclease bifur	ENDO3	AT4G21590	-1.17	-0.23	-2.23	0.062179	0.140105	-5.12
252260_at	putative protein several hypothetica	---	AT3G49480	-1.28	-0.36	-2.23	0.062188	0.140105	-5.12
257421_at	hypothetical protein predicted by ge	---	AT1G12030	-1.37	-0.46	-2.23	0.062194	0.140105	-5.12
257869_at	ER lumen protein retaining recepto	---	AT3G25160	-1.16	-0.21	-2.23	0.062195	0.140105	-5.12
254977_s_a	subtilisin-like serine protease conta	---	AT4G10520	-1.15	-0.20	-2.23	0.062198	0.140105	-5.12
253968_at	putative calcineurin B-like protein c	CBL7	AT4G26560	-1.18	-0.24	-2.23	0.062202	0.140105	-5.12
255242_at	putative protein putative retrotransp	---	---	-1.18	-0.23	-2.23	0.062205	0.140105	-5.12
264718_at	receptor-like kinase, putative simila	---	AT1G70130	-1.16	-0.22	-2.23	0.062209	0.140105	-5.12
263522_at	hypothetical protein	---	AT2G24950	-1.16	-0.21	-2.23	0.06222	0.140116	-5.12
249245_at	putative protein contains similarity t	---	AT5G42280	-1.29	-0.36	-2.23	0.06229	0.140247	-5.12
258730_at	putative GTP-binding protein (ATFF	ATFP8	AT3G11730	1.15	0.20	2.23	0.062292	0.140247	-5.12
264485_at	unknown protein ; supported by cDI	---	AT1G77220	-1.19	-0.25	-2.23	0.0623	0.140247	-5.12
260702_at	calmodulin, putative similar to calm	---	AT1G32250	-1.18	-0.24	-2.23	0.062303	0.140247	-5.12
254202_at	putative protein hydroxymuconic se	---	AT4G24140	-1.34	-0.42	-2.23	0.062328	0.140289	-5.12
264410_s_a	hypothetical protein predicted by ge	---	---	1.15	0.21	2.23	0.062339	0.140302	-5.12
261505_at	carboxypeptidase, putative similar	SOL1	AT1G71696	-1.26	-0.33	-2.23	0.062351	0.140306	-5.12
259407_at	protein phosphatase 2A regulatory	PP2AA3	AT1G13320	-1.16	-0.21	-2.23	0.062357	0.140306	-5.12
263328_at	hypothetical protein similar to hypot	---	AT2G15280	-1.25	-0.33	-2.23	0.06236	0.140306	-5.12
246834_at	putative protein homeotic protein b	---	AT5G26630	-1.21	-0.27	-2.23	0.0624	0.140382	-5.12
245184_at	F12A21.8 hypothetical protein	---	AT1G67790	-1.22	-0.28	-2.23	0.062483	0.140556	-5.12
250473_at	annexin -like protein annexin (Ann/	ANN6	AT5G10220	-1.15	-0.21	-2.23	0.062498	0.140565	-5.12
257684_s_a	unknown protein contains Pfam pro	VCS	AT3G13290	1.23	0.30	2.23	0.062499	0.140565	-5.12
263667_at	putative 40S ribosomal protein S15	RPS15	AT1G04270	1.27	0.34	2.23	0.062535	0.140612	-5.13
265685_at	NAM (no apical meristem)-like prote	ANAC038	AT2G24430	-1.24	-0.31	-2.23	0.062536	0.140612	-5.13
263196_at	hypothetical protein predicted by ge	---	AT1G36070	1.34	0.42	2.23	0.062541	0.140612	-5.13
258444_at	expressed protein supported by cDI	TIC	AT3G22380	1.19	0.26	2.23	0.062548	0.140612	-5.13
259170_at	hypothetical protein similar to putati	---	AT3G03510	-1.17	-0.23	-2.23	0.062551	0.140612	-5.13
250637_at	glycine-rich protein atGRP-7	GRP17	AT5G07530	-1.20	-0.27	-2.23	0.062573	0.140648	-5.13

263702_at	hypothetical protein identical to hyp	---	AT1G31240	-1.19	-0.25	-2.23	0.062585	0.14066	-5.13
253709_at	pyrophosphate-dependent phosph	PFK1	AT4G29220	1.27	0.35	2.23	0.062591	0.140661	-5.13
263887_at	hypothetical protein predicted by ge	---	AT2G36980	1.26	0.33	2.23	0.062608	0.140684	-5.13
248551_at	putative protein similar to unknown	WR3	AT5G50200	1.66	0.73	2.23	0.062717	0.140916	-5.13
264179_at	hypothetical protein predicted by ge	---	AT1G02180	-1.32	-0.40	-2.23	0.06274	0.140954	-5.13
263157_at	aldehyde dehydrogenase homolog	ALDH7B4	AT1G54100	1.38	0.46	2.23	0.062787	0.141038	-5.13
247638_at	arabinogalactan protein - like arabi	FLA12	AT5G60490	-1.48	-0.56	-2.23	0.06279	0.141038	-5.13
254240_at	Expressed protein ; supported by fu	SP1L5	AT4G23496	-1.19	-0.25	-2.23	0.062849	0.141157	-5.13
253660_at	putative protein proline-rich protein	---	AT4G30140	-1.18	-0.24	-2.23	0.062856	0.141158	-5.13
255961_at	UDP-glucose glucosyltransferase,	AtUGT85A	AT1G22340	-1.37	-0.45	-2.23	0.062869	0.141174	-5.13
262312_at	hypothetical protein similar to putati	MLP28	AT1G70830	-4.52	-2.18	-2.23	0.062929	0.141282	-5.13
259702_at	putative endo-1,3-beta-glucanase s	---	AT1G77780	-1.18	-0.24	-2.23	0.06293	0.141282	-5.13
262759_at	unknown protein	---	AT1G10800	-1.18	-0.23	-2.22	0.062962	0.14134	-5.13
248174_at	50S ribosomal protein L24, chlorop	---	AT5G54600	-1.20	-0.26	-2.22	0.062972	0.14135	-5.13
256237_at	leucine rich repeat protein, putative	DRT100	AT3G12610	-6.11	-2.61	-2.22	0.062997	0.141391	-5.13
260047_at	putative UDP-N-acetylglucosamine-	---	AT1G73740	-1.19	-0.25	-2.22	0.063023	0.141436	-5.13
256280_at	unknown protein	---	AT3G12590	-1.22	-0.29	-2.22	0.063046	0.141471	-5.13
263195_at	non-specific lipid transfer protein, p	---	AT1G36150	-1.28	-0.35	-2.22	0.063051	0.141471	-5.13
261945_at	unknown protein	---	AT1G64530	-1.38	-0.47	-2.22	0.063065	0.141483	-5.13
249913_at	GDSL-motif lipase/hydrolase-like pr	---	AT5G22810	-1.22	-0.28	-2.22	0.063069	0.141483	-5.13
263253_at	bZIP transcription factor (POSF21)	---	AT2G31370	-1.25	-0.32	-2.22	0.06308	0.141493	-5.13
266372_at	putative two-component response	ATRR3	AT2G41310	1.22	0.29	2.22	0.063096	0.141508	-5.13
251047_at	putative protein mature-parasite-inf	---	AT5G02390	-1.23	-0.29	-2.22	0.063098	0.141508	-5.13
249214_at	beta-1,3-glucanase-like protein	---	AT5G42720	-1.42	-0.51	-2.22	0.063114	0.141516	-5.13
256682_at	putative protein dihydrolipoamide S	LTA3	AT3G52200	1.16	0.22	2.22	0.063115	0.141516	-5.13
259765_at	unknown protein ;supported by full-	---	AT1G64370	1.30	0.37	2.22	0.063122	0.141518	-5.13
259024_at	hypothetical protein predicted by ge	---	---	-1.26	-0.33	-2.22	0.063186	0.141648	-5.14
257238_at	unknown protein ; supported by cD	---	AT3G17430	-1.28	-0.35	-2.22	0.063215	0.14169	-5.14
261974_at	protein kinase, putative similar to m	CPuORF57	AT1G64628	1.19	0.25	2.22	0.063217	0.14169	-5.14
267265_at	putative serine carboxypeptidase I	SCPL13	AT2G22980	-1.30	-0.38	-2.22	0.063237	0.14172	-5.14
246517_at	ribosomal protein 3 precursor-like p	---	AT5G15760	-1.23	-0.30	-2.22	0.063257	0.141751	-5.14
245132_at	hypothetical protein predicted by ge	---	AT2G45320	1.30	0.38	2.22	0.063289	0.14181	-5.14
267289_at	putative protein kinase contains a p	---	AT2G23770	-1.17	-0.22	-2.22	0.063324	0.141875	-5.14
261892_at	transcription factor, putative similar	WRKY40	AT1G80840	3.02	1.60	2.22	0.063334	0.141883	-5.14
261211_at	uridine diphosphate glucose epime	UGE1	AT1G12780	1.37	0.45	2.22	0.063392	0.141999	-5.14

262221_at	hypothetical protein predicted by ge	ARC3	AT1G75010	1.26	0.33	2.22	0.06342	0.142048	-5.14
AFFX-r2-At-	Arabidopsis thaliana /REF=U8496	---	---	1.17	0.23	2.22	0.06344	0.142068	-5.14
266180_at	putative PHD-type zinc finger protei	AL6	AT2G02470	1.26	0.33	2.22	0.063442	0.142068	-5.14
250820_at	receptor-like protein kinase	---	AT5G05160	-1.23	-0.30	-2.22	0.063453	0.142079	-5.14
248142_at	putative protein similar to unknown	---	AT5G55040	-1.17	-0.23	-2.22	0.063515	0.142205	-5.14
252197_at	receptor protein kinase -like protei	---	AT3G50230	-1.16	-0.21	-2.22	0.063551	0.142271	-5.14
251340_at	actin - like protein actin 3, Drosoph	ATARP7	AT3G60830	1.19	0.25	2.22	0.063581	0.142324	-5.14
253089_at	putative protein hypothetical protei	---	AT4G36290	1.17	0.22	2.22	0.06364	0.142441	-5.14
257028_at	hypothetical protein predicted by g	---	AT3G19230	-1.36	-0.45	-2.22	0.063646	0.142441	-5.14
260896_at	flower pigmentation protein ATAN1	---	AT1G29310	-1.42	-0.50	-2.22	0.06366	0.142456	-5.14
246704_at	putative protein	---	AT5G28090	-1.19	-0.25	-2.22	0.063665	0.142456	-5.14
258176_at	B regulatory subunit of PP2A, puta	---	AT3G21650	1.40	0.48	2.22	0.063703	0.142526	-5.14
259629_at	disease resistance protein contains	WRR4	AT1G56510	-1.27	-0.35	-2.22	0.063722	0.142549	-5.14
246562_at	putative protein unknown protein F	LNG1	AT5G15580	-2.72	-1.44	-2.22	0.063725	0.142549	-5.14
251802_at	E2, ubiquitin-conjugating enzyme 1	UBC14	AT3G55380	1.17	0.23	2.22	0.063777	0.14265	-5.15
256900_at	pectate lyase, putative similar to pe	---	AT3G24670	-1.23	-0.30	-2.22	0.063791	0.142668	-5.15
244933_at	NADH dehydrogenase ND4L	---	---	-1.21	-0.27	-2.22	0.063822	0.142724	-5.15
251916_at	transporter-like protein oligopeptide	---	AT3G53960	-1.31	-0.39	-2.22	0.063835	0.142726	-5.15
253862_at	NOZZLE/SPOROCYTELESS ; sup	SPL	AT4G27330	-1.26	-0.34	-2.22	0.063835	0.142726	-5.15
267420_at	hypothetical protein predicted by gr	---	AT2G35030	1.44	0.52	2.21	0.063929	0.142921	-5.15
251703_at	putative protein hypothetical protein	---	AT3G56670	-1.26	-0.34	-2.21	0.063941	0.142934	-5.15
248509_at	Expressed protein ; supported by fu	---	AT5G50335	-5.51	-2.46	-2.21	0.063953	0.142946	-5.15
256602_at	unknown protein	---	AT3G28310	-1.27	-0.34	-2.21	0.063959	0.142946	-5.15
255150_at	putative xylan endohydrolase	---	AT4G08160	-1.35	-0.44	-2.21	0.063975	0.142968	-5.15
245979_at	putative protein leucine zipper-cont	ATEXO70C	AT5G13150	-1.15	-0.20	-2.21	0.063985	0.142976	-5.15
263294_at	hypothetical protein predicted by ge	---	---	-1.26	-0.33	-2.21	0.064033	0.143067	-5.15
259871_at	nodulin-like protein similar to noduli	---	AT1G76800	1.53	0.61	2.21	0.064044	0.143067	-5.15
264649_at	hypothetical protein Similar to Vicia	---	AT1G09060	-1.24	-0.30	-2.21	0.064045	0.143067	-5.15
267016_at	hypothetical protein predicted by ge	---	AT2G39160	-1.19	-0.25	-2.21	0.064063	0.143086	-5.15
245449_at	retrotransposon like protein	---	---	-1.35	-0.43	-2.21	0.064069	0.143086	-5.15
256538_x_a	hypothetical protein predicted by ge	---	---	-1.17	-0.23	-2.21	0.064074	0.143086	-5.15
258454_at	putative 40S ribosomal protein S10	RPS10	AT3G22300	1.28	0.36	2.21	0.064078	0.143086	-5.15
262457_at	hypothetical protein similar to hypot	---	AT1G11200	1.36	0.45	2.21	0.064121	0.143167	-5.15
262182_at	hypothetical protein predicted by ge	---	AT1G77890	-1.18	-0.24	-2.21	0.064129	0.14317	-5.15
246673_at	ribosomal protein S1 ;supported by	RPS1	AT5G30510	-1.17	-0.23	-2.21	0.064138	0.14317	-5.15

265083_at	hypothetical protein predicted by ge	---	AT1G03820	-1.44	-0.53	-2.21	0.064141	0.14317	-5.15
260834_at	sugar kinase, putative similar to rib	---	AT1G06730	1.24	0.32	2.21	0.06416	0.143198	-5.15
249963_at	putative protein SPECKLE-TYPE F	ATBPM1	AT5G19000	-1.24	-0.31	-2.21	0.064167	0.1432	-5.15
253621_at	putative protein component of anili	---	AT4G30540	-1.20	-0.26	-2.21	0.06419	0.143229	-5.15
263848_at	putative reverse transcriptase	---	---	-1.22	-0.28	-2.21	0.064193	0.143229	-5.15
258848_at	Expressed protein ; supported by fu	DCL2	AT3G03300	1.18	0.24	2.21	0.064202	0.143237	-5.15
252177_at	protein kinase ATN1 - like protein p	---	AT3G50730	-1.23	-0.30	-2.21	0.064211	0.143242	-5.15
254084_at	ubiquitin activating enzyme - like pr	SAE1A	AT4G24940	1.37	0.45	2.21	0.06426	0.143338	-5.15
261991_at	hypothetical protein predicted by ge	---	AT1G33700	-1.26	-0.33	-2.21	0.064269	0.143344	-5.15
245952_at	putative protein predicted proteins,	---	AT5G28500	1.25	0.32	2.21	0.064401	0.143624	-5.16
248553_at	putative protein strong similarity to u	---	AT5G50170	1.44	0.52	2.21	0.064421	0.143655	-5.16
249892_at	unknown protein	---	AT5G22600	-1.16	-0.21	-2.21	0.06445	0.143704	-5.16
254235_at	putative Ap2 domain protein DNA-b	CRF2	AT4G23750	-1.18	-0.24	-2.21	0.064458	0.143708	-5.16
257646_at	methionine aminopeptidase, putativ	MAP1B	AT3G25740	-1.41	-0.50	-2.21	0.064469	0.14372	-5.16
266509_at	DegP2 protease identical to DegP2	DEGP2	AT2G47940	-1.22	-0.29	-2.21	0.064482	0.143728	-5.16
253191_at	cysteine protease XCP1 identical to	XCP1	AT4G35350	-1.84	-0.88	-2.21	0.06449	0.143728	-5.16
260191_at	putative protein kinase Pfam HMM	---	AT1G67580	1.17	0.22	2.21	0.064497	0.143728	-5.16
257744_at	hypothetical protein contains Pfam	---	AT3G29230	1.24	0.31	2.21	0.064498	0.143728	-5.16
256154_at	hypothetical protein predicted by ge	---	AT3G08490	-1.23	-0.29	-2.21	0.064548	0.143824	-5.16
260837_at	fructose 1,6-bisphosphatase, putat	---	AT1G43670	-1.39	-0.48	-2.21	0.064559	0.143836	-5.16
267380_at	putative thromboxane-A synthase ;	CYP711A1	AT2G26170	1.47	0.56	2.21	0.06458	0.143856	-5.16
266493_at	Mutator-like transposase similar to	---	---	-1.18	-0.24	-2.21	0.064581	0.143856	-5.16
265996_at	putative leucine aminopeptidase ide	---	AT2G24200	1.47	0.56	2.21	0.064594	0.143858	-5.16
246035_at	succinyl-CoA-ligase alpha subunit	---	AT5G08300	1.54	0.62	2.21	0.064594	0.143858	-5.16
258566_at	putative glutamate receptor (GLR1)	GLR1.1	AT3G04110	1.36	0.45	2.21	0.064618	0.143884	-5.16
245755_at	hypothetical protein predicted by ge	---	AT1G35210	2.87	1.52	2.21	0.064619	0.143884	-5.16
246762_at	cyclin H-like protein cycH (CAK ass	CYCH;1	AT5G27620	1.32	0.40	2.21	0.064661	0.143964	-5.16
248735_at	laccase (diphenol oxidase)	TT10	AT5G48100	-1.18	-0.24	-2.21	0.064702	0.144042	-5.16
250232_at	unknown protein	---	AT5G13950	-1.18	-0.23	-2.21	0.06471	0.144045	-5.16
251383_at	beta-tubulin cofactor - like protein b	TTN1	AT3G60740	1.32	0.40	2.21	0.064723	0.144056	-5.16
262690_at	PPR-repeat protein contains multipl	---	AT1G62720	1.18	0.24	2.21	0.064727	0.144056	-5.16
258775_at	hydroxyacylglutathione hydrolase c	GLY2	AT3G10850	1.19	0.25	2.21	0.06475	0.144065	-5.16
252492_at	chloroplast import-associated chan	TOC75-III	AT3G46740	1.22	0.29	2.21	0.064755	0.144065	-5.16
261568_at	DNA-binding protein, putative simil	NGA3	AT1G01030	-1.20	-0.26	-2.21	0.064756	0.144065	-5.16
250265_at	putative protein predicted proteins,	---	AT5G12900	-1.30	-0.37	-2.21	0.064761	0.144065	-5.16

263403_at	hypothetical protein similar to hypot	TX1	AT2G04040	2.51	1.33	2.21	0.064763	0.144065	-5.16
250975_at	putative protein	---	AT5G03050	-1.24	-0.31	-2.20	0.064839	0.144219	-5.16
265348_at	putative RNA-binding protein	---	AT2G22600	-1.21	-0.27	-2.20	0.064858	0.144247	-5.16
252869_at	putative protein	---	AT4G39930	-1.18	-0.24	-2.20	0.064883	0.144249	-5.16
261171_at	unknown protein	---	AT1G04880	-1.19	-0.24	-2.20	0.064886	0.144249	-5.16
248706_at	unknown protein	---	AT5G48530	-1.22	-0.29	-2.20	0.064886	0.144249	-5.16
246741_at	glycine rich protein - like glycine ric	emb1644	AT5G27720	1.23	0.30	2.20	0.064889	0.144249	-5.16
265385_at	putative diacylglycerol kinase ;supp	---	AT2G20900	1.36	0.45	2.20	0.064896	0.144249	-5.16
259875_s_a	12-oxophytodienoate reductase (OI	OPR1	AT1G76680	1.68	0.75	2.20	0.064896	0.144249	-5.16
267041_at	unknown protein	---	AT2G34315	-1.15	-0.20	-2.20	0.064909	0.144263	-5.16
255645_at	coded for by A. thaliana cDNA T43	---	AT4G00880	1.34	0.42	2.20	0.064919	0.144266	-5.16
253460_at	hypothetical protein	---	AT4G32130	1.24	0.31	2.20	0.064926	0.144266	-5.16
258088_at	hypothetical protein predicted by ge	---	AT3G14580	1.18	0.24	2.20	0.064934	0.144266	-5.16
263503_s_a	hypothetical protein	---	AT2G07676	1.17	0.23	2.20	0.064935	0.144266	-5.16
245295_at	hypothetical protein ; supported by	---	AT4G16100	1.23	0.30	2.20	0.064955	0.144295	-5.16
267026_at	DREB-like AP2 domain transcriptio	---	AT2G38340	1.75	0.80	2.20	0.064978	0.144322	-5.16
255894_at	nuclear poly(A) polymerase, putati	---	AT1G17980	-1.20	-0.26	-2.20	0.06498	0.144322	-5.16
267292_at	hypothetical protein predicted by ge	---	AT2G23740	-1.20	-0.26	-2.20	0.064988	0.144327	-5.16
246266_at	30S ribosomal protein S11, putativ	NFD3	AT1G31817	1.28	0.36	2.20	0.065019	0.144373	-5.16
262547_at	hypothetical protein predicted by ge	---	AT1G31270	-1.15	-0.20	-2.20	0.065022	0.144373	-5.16
248673_at	putative protein contains similarity t	---	AT5G48780	-1.17	-0.22	-2.20	0.065031	0.144376	-5.16
253957_at	putative protein ; supported by cDN	AGP13	AT4G26320	-1.30	-0.38	-2.20	0.065036	0.144376	-5.16
246913_at	GATA transcription factor - like GA	---	AT5G25830	-1.26	-0.34	-2.20	0.065047	0.144386	-5.17
248151_at	Lhcb3 chlorophyll a/b binding protei	LHCB3	AT5G54270	-1.39	-0.48	-2.20	0.06506	0.144397	-5.17
252030_at	hypothetical protein predicted prote	---	AT3G52690	-1.16	-0.21	-2.20	0.065064	0.144397	-5.17
260635_at	unknown protein	---	AT1G62420	-1.25	-0.33	-2.20	0.065098	0.144459	-5.17
263572_at	putative disease resistance protein	---	AT2G17060	-1.17	-0.22	-2.20	0.065121	0.144496	-5.17
249518_at	putative protein similar to unknown	---	AT5G38610	-1.19	-0.25	-2.20	0.065128	0.144497	-5.17
247509_at	heat shock factor 6	HSFB2A	AT5G62020	1.23	0.30	2.20	0.065156	0.144532	-5.17
265164_at	putative OBP32pep protein	---	AT1G23600	-1.17	-0.23	-2.20	0.065157	0.144532	-5.17
263021_at	hypothetical protein predicted by ge	---	AT1G23910	-1.15	-0.20	-2.20	0.065167	0.144541	-5.17
246702_at	putative cytidine deaminase - like p	---	AT5G28050	-1.63	-0.71	-2.20	0.065239	0.144687	-5.17
262193_at	eukaryotic initiation factor 5 (eIF-5)	---	AT1G77840	1.29	0.36	2.20	0.065251	0.144699	-5.17
267185_at	unknown protein ; supported by cD	OEP37	AT2G43950	1.21	0.28	2.20	0.065264	0.144714	-5.17
258869_at	hypothetical protein similar to xylos	AtVGT1	AT3G03090	-1.23	-0.30	-2.20	0.065403	0.144982	-5.17

258650_at	putative protein kinase similar to pro	---	AT3G09830	1.37	0.46	2.20	0.065404	0.144982	-5.17
248950_at	ATP-dependent Clp protease prote	CLPP4	AT5G45390	-1.22	-0.29	-2.20	0.065404	0.144982	-5.17
267518_at	unknown protein	---	AT2G30500	1.64	0.71	2.20	0.065459	0.145089	-5.17
251399_at	putative protein	---	AT3G60590	-1.23	-0.30	-2.20	0.065478	0.145119	-5.17
247971_at	zinc protease PQQL-like protein	---	AT5G56730	1.18	0.24	2.20	0.065494	0.14514	-5.17
262748_at	lipase, putative contains Pfam prof	---	AT1G28610	1.37	0.45	2.20	0.065539	0.145225	-5.17
249287_at	putative protein strong similarity to u	---	AT5G41250	-1.22	-0.28	-2.20	0.065551	0.145237	-5.17
253340_s_a	WD-repeat protein -like protein WD	CDC20.2	AT4G33260	1.19	0.25	2.20	0.065584	0.145296	-5.17
258222_at	putative zinc finger protein contains	---	AT3G15680	-1.39	-0.48	-2.20	0.065613	0.145337	-5.17
261419_at	hypothetical protein predicted by ge	---	AT1G07840	1.15	0.20	2.20	0.065615	0.145337	-5.17
244914_at	hypothetical protein	---	---	-1.19	-0.25	-2.20	0.065677	0.14546	-5.17
265570_at	unknown protein	---	AT2G28310	1.24	0.32	2.20	0.065715	0.145529	-5.18
264779_at	putative thioredoxin similar to ESTs	ACHT4	AT1G08570	-1.49	-0.57	-2.20	0.065728	0.145544	-5.18
258842_at	unknown protein similar to putative	CLPS3	AT3G04680	1.26	0.33	2.19	0.065748	0.145575	-5.18
262279_at	hypothetical protein predicted by ge	---	AT1G68630	1.45	0.54	2.19	0.065805	0.145686	-5.18
262178_at	membrane protein, putative similar	KOM	AT1G77860	-1.28	-0.35	-2.19	0.06587	0.145818	-5.18
262809_at	putative glycogen synthase strong s	ATSS3	AT1G11720	-1.56	-0.64	-2.19	0.065913	0.145898	-5.18
249420_at	NAC domain/ NAM like protein NA	anac094	AT5G39820	-1.18	-0.24	-2.19	0.065922	0.145904	-5.18
265468_at	hypothetical protein predicted by ge	---	AT2G37210	-1.19	-0.26	-2.19	0.065962	0.145977	-5.18
265927_at	hypothetical protein	---	AT2G18590	-1.23	-0.30	-2.19	0.065974	0.14599	-5.18
253779_at	receptor-like protein kinase 5 precu	HAE	AT4G28490	1.46	0.55	2.19	0.066015	0.146066	-5.18
258496_at	putative plant adhesion molecule si	---	AT3G02460	-1.29	-0.37	-2.19	0.066071	0.146177	-5.18
258660_at	hypothetical protein predicted by ge	---	AT3G09850	1.21	0.28	2.19	0.066078	0.146177	-5.18
253129_at	glycine-rich protein glycine-rich pro	CSDP1	AT4G36020	1.29	0.37	2.19	0.066134	0.146287	-5.18
250982_at	putative protein zinc finger protein,	JKD	AT5G03150	-1.36	-0.44	-2.19	0.066155	0.146306	-5.18
253220_s_a	putative protein phospholipase C, L	---	AT4G34920	-1.28	-0.35	-2.19	0.066155	0.146306	-5.18
263113_at	unknown protein Belongs to PF|005	---	AT1G03150	-1.16	-0.21	-2.19	0.06617	0.146325	-5.18
245444_at	limonene cyclase like protein	ATTPS03	AT4G16740	-1.25	-0.32	-2.19	0.066212	0.146403	-5.18
267412_at	putative vacuolar sorting receptor	---	AT2G34940	-1.71	-0.77	-2.19	0.066224	0.146415	-5.18
255813_at	hypothetical protein contains Pfam	---	AT1G19930	-1.20	-0.26	-2.19	0.06627	0.146502	-5.18
257398_at	hypothetical protein predicted by ge	---	AT2G01990	1.15	0.21	2.19	0.066284	0.14652	-5.18
253196_at	NAD+ dependent isocitrate dehydro	IDH1	AT4G35260	1.27	0.34	2.19	0.066359	0.146667	-5.19
252434_s_a	putative protein mudrA protein - ma	---	---	-1.20	-0.26	-2.19	0.066367	0.146667	-5.19
267286_at	putative seed maturation protein	---	AT2G23640	-1.16	-0.21	-2.19	0.06637	0.146667	-5.19
263024_at	hypothetical protein predicted by ge	---	AT1G23980	-1.25	-0.32	-2.19	0.066386	0.146689	-5.19

249856_at	serine carboxypeptidase	scpl47	AT5G22980	-1.31	-0.39	-2.19	0.066397	0.146699	-5.19
257176_s_a	cyclopropane-fatty-acyl-phospholip	---	AT3G23510	-1.80	-0.85	-2.19	0.06642	0.146735	-5.19
246014_at	putative protein	---	AT5G10680	-1.19	-0.25	-2.19	0.066438	0.14676	-5.19
251998_at	putative protein KIAA0010 protein,	UPL7	AT3G53090	1.19	0.25	2.19	0.066478	0.146825	-5.19
253387_at	P-Protein - like protein P-Protein pr	AtGLDP1	AT4G33010	-1.46	-0.54	-2.19	0.066484	0.146825	-5.19
250745_at	putative protein strong similarity to u	---	AT5G05850	1.31	0.39	2.19	0.066486	0.146825	-5.19
260851_at	nodulin-like protein similar to MtN2	---	AT1G21890	-1.20	-0.26	-2.19	0.066513	0.14687	-5.19
266077_at	putative non-LTR retroelement reve	---	---	-1.21	-0.27	-2.19	0.066526	0.146884	-5.19
253112_at	putative ascorbate peroxidase asc	APX5	AT4G35970	-1.31	-0.39	-2.19	0.066541	0.146902	-5.19
246883_at	putative protein many predicted pro	---	AT5G26190	-1.16	-0.21	-2.19	0.066615	0.147053	-5.19
263155_at	transcriptional activation factor TAF	TAFII21	AT1G54140	-1.22	-0.28	-2.19	0.066658	0.147133	-5.19
249544_at	anti-silencing factor 1-like protein S	ASF1B	AT5G38110	-1.28	-0.36	-2.19	0.066672	0.14715	-5.19
262915_at	response regulator 3 identical to res	ARR3	AT1G59940	-1.23	-0.29	-2.19	0.066697	0.14719	-5.19
259541_at	protein kinase, putative similar to p	---	AT1G20650	-1.33	-0.41	-2.18	0.066719	0.147224	-5.19
249690_at	acyl-peptide hydrolase-like	---	AT5G36210	1.18	0.24	2.18	0.066741	0.147259	-5.19
267554_at	E2, ubiquitin-conjugating enzyme,	---	AT2G32790	-1.23	-0.30	-2.18	0.06678	0.147331	-5.19
252657_at	hypothetical protein	---	AT3G44780	-1.15	-0.20	-2.18	0.066816	0.147395	-5.19
263321_at	ADP-ribosylation factor 1 ;supporte	ARF1A1C	AT2G47170	-1.18	-0.24	-2.18	0.066835	0.147423	-5.19
263283_at	hypothetical protein predicted by ge	---	AT2G36090	1.18	0.24	2.18	0.066844	0.14743	-5.19
253591_at	putative protein hypothetical protei	MUS81	AT4G30870	1.29	0.36	2.18	0.066883	0.147501	-5.19
266235_at	putative phloem-specific lectin	AtPP2-B10	AT2G02360	1.26	0.34	2.18	0.066926	0.147582	-5.19
251767_at	putative protein U4/U6 small nucle	---	AT3G55930	1.15	0.20	2.18	0.066982	0.14769	-5.19
254696_at	hypothetical protein	---	AT4G17930	-1.16	-0.22	-2.18	0.066993	0.1477	-5.19
250506_at	ABC transporter, ATP-binding prot	GCN2	AT5G09930	-1.21	-0.28	-2.18	0.067005	0.147713	-5.19
259141_at	putative cytoskeleton-associated pr	---	AT3G10220	1.19	0.25	2.18	0.067024	0.147741	-5.20
266933_at	putative Ta11-like non-LTR retroele	---	AT2G07760	-1.24	-0.31	-2.18	0.067041	0.147764	-5.20
247471_at	putative protein receptor-like protei	ERL1	AT5G62230	-1.21	-0.27	-2.18	0.067057	0.147779	-5.20
263429_at	putative aspartate aminotransferase	AAT	AT2G22250	1.81	0.86	2.18	0.067061	0.147779	-5.20
259580_at	oxidoreductase, putative contains F	---	AT1G28030	-1.25	-0.32	-2.18	0.067102	0.147855	-5.20
261114_at	bZIP transcription factor ATB2, put	AtbZIP44	AT1G75388	2.71	1.44	2.18	0.06712	0.14787	-5.20
253408_at	putative protein phosphoprotein ph	---	AT4G32950	-1.16	-0.21	-2.18	0.067122	0.14787	-5.20
264172_at	unknown protein	VAD1	AT1G02120	1.30	0.37	2.18	0.067188	0.147994	-5.20
247708_at	putative protein COP1-interacting p	---	AT5G59550	1.94	0.96	2.18	0.067191	0.147994	-5.20
253938_at	putative homeodomain protein hom	---	AT4G26920	-1.21	-0.27	-2.18	0.067206	0.148013	-5.20
261677_at	unknown protein ;supported by full-	---	AT1G18470	1.31	0.39	2.18	0.067221	0.148028	-5.20

254837_at	putative protein	---	AT4G12360	-1.22	-0.29	-2.18	0.067226	0.148028	-5.20
261327_at	transcription factor, putative contai	---	AT1G44830	-2.69	-1.43	-2.18	0.067278	0.148128	-5.20
261950_at	zinc finger protein, putative similar	---	AT1G64620	-1.31	-0.39	-2.18	0.067301	0.148162	-5.20
253935_at	putative aspartate-tRNA ligase asp	---	AT4G26870	1.51	0.60	2.18	0.06731	0.148162	-5.20
245903_at	CLB1 - like protein CLB1 protein, L	SYTD	AT5G11100	-1.19	-0.25	-2.18	0.067313	0.148162	-5.20
262398_at	hypothetical protein predicted by ge	---	AT1G49350	1.15	0.21	2.18	0.067321	0.148165	-5.20
267529_at	putative protein kinase contains a p	AtAUR3	AT2G45490	-1.24	-0.31	-2.18	0.067378	0.148274	-5.20
265131_at	putative polygalacuronase isoenzyn	JP630	AT1G23760	-1.51	-0.59	-2.18	0.067383	0.148274	-5.20
259989_at	hypothetical protein predicted by ge	---	---	-1.21	-0.28	-2.18	0.067392	0.148279	-5.20
249910_at	chorismate mutase/prephenate deh	ADT5	AT5G22630	-1.68	-0.75	-2.18	0.067414	0.148309	-5.20
254188_at	UDPglucose 4-epimerase - like pro	UGE2	AT4G23920	1.46	0.55	2.18	0.06742	0.148309	-5.20
262202_at	TATC-like protein similar to GB:AAI	APG2	AT2G01110	-1.30	-0.38	-2.18	0.067425	0.148309	-5.20
254689_at	putative pectate lyase A11 ; suppo	---	AT4G13710	-1.28	-0.35	-2.18	0.067436	0.14832	-5.20
259209_at	putative RNA-binding protein identi	---	AT3G09160	-1.14	-0.19	-2.18	0.06749	0.148424	-5.20
256449_at	mutator transposase MUDRA, puta	---	---	-1.18	-0.24	-2.18	0.067511	0.148455	-5.20
246201_at	minor allergen ;supported by full-ler	---	AT4G36750	-1.15	-0.21	-2.18	0.067527	0.148471	-5.20
249766_at	peroxidase-like protein	---	AT5G24070	-1.20	-0.26	-2.18	0.067531	0.148471	-5.20
258257_at	putative short chain alcohol dehydro	---	AT3G26770	-1.16	-0.21	-2.18	0.067644	0.148705	-5.20
253209_at	putative protein membrane-associa	---	AT4G34830	1.25	0.32	2.17	0.067698	0.14881	-5.21
247820_at	serine/threonine protein kinase ; su	SIP1	AT5G58380	1.26	0.33	2.17	0.067713	0.148827	-5.21
258206_at	hypothetical protein predicted by ge	CID4	AT3G14010	1.19	0.25	2.17	0.067741	0.148875	-5.21
252537_at	putative transporter protein peptide	---	AT3G45710	-1.19	-0.26	-2.17	0.067755	0.148891	-5.21
262672_at	RNA pseudouridylate synthase, pu	---	AT1G76050	-1.19	-0.25	-2.17	0.067793	0.148959	-5.21
249512_at	DNA repair protein RAD23 homolog	---	AT5G38470	1.14	0.20	2.17	0.067829	0.149022	-5.21
251316_s_a	putative protein putative protein Bc	---	AT3G61480	1.19	0.26	2.17	0.067835	0.149022	-5.21
249643_at	putative transposase putative trans	---	---	-1.23	-0.30	-2.17	0.067841	0.149022	-5.21
251301_at	cytochrome p450 (CYP78A9) ;supp	CYP78A9	AT3G61880	1.22	0.29	2.17	0.067852	0.149033	-5.21
248919_at	putative protein contains similarity t	---	AT5G45920	-1.21	-0.27	-2.17	0.067863	0.149041	-5.21
264458_at	unknown protein similar to ESTs gb	---	AT1G10410	1.19	0.25	2.17	0.06789	0.149088	-5.21
254134_at	argininosuccinate synthase -like pr	---	AT4G24830	-1.38	-0.46	-2.17	0.068011	0.14934	-5.21
265008_at	Mlo protein, putative similar to Mlo	MLO6	AT1G61560	1.90	0.93	2.17	0.068021	0.149347	-5.21
254441_at	prolamin box binding protein - like	---	AT4G21050	-1.16	-0.21	-2.17	0.068042	0.149368	-5.21
259938_at	hypothetical protein predicted by ge	---	AT1G71310	1.20	0.26	2.17	0.068044	0.149368	-5.21
263420_at	unknown protein ; supported by ful	---	AT2G17240	1.17	0.23	2.17	0.068123	0.149528	-5.21
249713_at	putative protein contains similarity t	---	AT5G35690	-1.23	-0.30	-2.17	0.068132	0.149532	-5.21

252315_at	putative protein PrMC3 - Pinus rad	CXE12	AT3G48690	1.24	0.31	2.17	0.068195	0.149656	-5.21
247097_at	mannan endo-1,4-beta-mannosida	---	AT5G66460	1.28	0.36	2.17	0.06823	0.149718	-5.21
264623_at	putative NPK1-related protein kinas	ANP1	AT1G09000	-1.16	-0.21	-2.17	0.06825	0.149748	-5.21
249691_at	translation releasing factor RF-2 ;s	HCF109	AT5G36170	1.15	0.20	2.17	0.068291	0.149824	-5.21
266094_at	Expressed protein ; supported by fu	---	AT2G37975	-1.21	-0.27	-2.17	0.0683	0.149826	-5.21
253940_at	putative protein other Arabidopsis h	---	AT4G26950	1.22	0.29	2.17	0.068305	0.149826	-5.21
258320_at	hypothetical protein predicted by ge	---	AT3G22710	-1.17	-0.22	-2.17	0.068325	0.149838	-5.21
265854_at	hypothetical protein predicted by ge	---	AT2G42370	1.16	0.22	2.17	0.068328	0.149838	-5.21
247560_at	putative protein predicted proteins,	---	AT5G61090	-1.24	-0.31	-2.17	0.06833	0.149838	-5.21
245032_at	En/Spm-like transposon protein rela	---	---	-1.29	-0.37	-2.17	0.068351	0.149852	-5.21
258806_at	unknown protein	---	AT3G04020	1.35	0.43	2.17	0.068355	0.149852	-5.21
256710_at	hypothetical protein predicted by ge	---	AT3G30350	-1.34	-0.43	-2.17	0.068357	0.149852	-5.21
262463_at	hexose transporter, putative similar	STP9	AT1G50310	-1.37	-0.46	-2.17	0.068369	0.149864	-5.22
258779_at	putative protein kinase contains Pfa	---	AT3G11870	-1.15	-0.20	-2.17	0.068377	0.149869	-5.22
253599_at	putative protein ash1 protein, Dros	SDG4	AT4G30860	-1.30	-0.37	-2.17	0.068422	0.149948	-5.22
255639_at	putative protein	APRR8	AT4G00760	-1.24	-0.32	-2.17	0.068427	0.149948	-5.22
259880_at	hypothetical protein similar to hypo	---	AT1G76730	-1.30	-0.38	-2.17	0.068436	0.149955	-5.22
264162_at	acyl carrier protein, putative similar	mtACP2	AT1G65290	1.23	0.29	2.17	0.068448	0.149967	-5.22
264590_at	unknown protein ;supported by full-	---	AT2G17710	2.20	1.13	2.17	0.068461	0.149981	-5.22
260574_at	hypothetical protein predicted by ge	---	AT2G47300	-1.21	-0.28	-2.17	0.068471	0.149988	-5.22
250239_at	ABC transporter-like protein	---	AT5G13580	-1.27	-0.35	-2.17	0.068616	0.150281	-5.22
247682_at	serine/threonine-specific protein kir	---	AT5G59650	-1.21	-0.27	-2.17	0.068618	0.150281	-5.22
262500_at	unknown protein Contains PF|0064	FBP7	AT1G21760	1.18	0.23	2.17	0.06863	0.150293	-5.22
262283_at	unknown protein ;supported by full-	---	AT1G68590	-1.26	-0.33	-2.16	0.068719	0.150474	-5.22
259424_at	hypothetical protein contains simila	---	AT1G13830	1.29	0.37	2.16	0.06875	0.150526	-5.22
263093_at	putative replication protein A1	---	---	-1.17	-0.22	-2.16	0.068781	0.15058	-5.22
263377_at	AP2 domain transcription factor (AE	ABI4	AT2G40220	-1.16	-0.21	-2.16	0.068804	0.150617	-5.22
267540_at	hypothetical protein predicted by ge	---	AT2G32760	-1.16	-0.22	-2.16	0.068813	0.150622	-5.22
260908_at	maternal embryogenesis control pr	MEA	AT1G02580	1.38	0.47	2.16	0.068827	0.150637	-5.22
248208_at	unknown protein	ATHB52	AT5G53980	-1.28	-0.35	-2.16	0.068837	0.150645	-5.22
245701_at	ferredoxin-dependent glutamate syr	GLU1	AT5G04140	-1.29	-0.36	-2.16	0.068844	0.150646	-5.22
247939_at	putative protein contains similarity t	ATLIG4	AT5G57160	1.18	0.24	2.16	0.068917	0.150792	-5.22
254115_at	putative protein binding protein HP	---	AT4G24710	-1.17	-0.23	-2.16	0.068957	0.150864	-5.22
251995_at	nuclear envelope membrane protei	FK	AT3G52940	-1.16	-0.22	-2.16	0.069001	0.150917	-5.22
260263_at	hypothetical protein predicted by ge	JAG	AT1G68480	-1.15	-0.20	-2.16	0.069003	0.150917	-5.22

249604_x_a	putative protein RING-H2 finger pro	---	AT5G37230	-1.20	-0.26	-2.16	0.069007	0.150917	-5.22
255055_at	hypothetical protein	---	AT4G09810	1.25	0.33	2.16	0.069008	0.150917	-5.22
257639_at	hypothetical protein	---	AT3G25720	-1.20	-0.26	-2.16	0.069041	0.150976	-5.22
252089_at	putative protein	---	AT3G52110	-1.28	-0.35	-2.16	0.06914	0.151177	-5.23
264763_at	hypothetical protein contains similar	---	AT1G61450	-1.15	-0.21	-2.16	0.069156	0.151188	-5.23
260573_at	putative pectinesterase	---	AT2G47280	-1.20	-0.27	-2.16	0.069159	0.151188	-5.23
247610_at	putative protein predicted protein, A	---	AT5G60630	2.60	1.38	2.16	0.069165	0.151188	-5.23
266462_at	unknown protein ; supported by cD	---	AT2G47770	-1.67	-0.74	-2.16	0.069212	0.151277	-5.23
265343_at	unknown protein ; supported by full-	NIC1	AT2G22570	1.37	0.45	2.16	0.069227	0.151277	-5.23
249064_at	putative protein contains similarity t	---	AT5G44250	-1.16	-0.21	-2.16	0.06923	0.151277	-5.23
248041_at	unknown protein ;supported by full-	emb2731	AT5G55940	-1.15	-0.21	-2.16	0.069232	0.151277	-5.23
252514_at	GTP-binding protein ara-3 ; suppor	ATRAB8A	AT3G46060	1.16	0.22	2.16	0.069317	0.151448	-5.23
251403_at	putative protein RING finger protein	---	AT3G60300	1.27	0.35	2.16	0.069361	0.151531	-5.23
265289_at	unknown protein predicted by gens	---	AT2G22690	1.15	0.20	2.16	0.069417	0.151638	-5.23
250715_at	splicing factor 3a ; supported by cD	ATO	AT5G06160	1.27	0.34	2.16	0.069446	0.151686	-5.23
259300_at	hypothetical protein predicted by ge	---	AT3G05100	-1.18	-0.24	-2.16	0.069467	0.151719	-5.23
264218_at	unknown protein	---	AT1G60200	1.15	0.20	2.16	0.069568	0.151924	-5.23
253155_at	putative protein predicted protein, A	---	AT4G35720	-1.20	-0.27	-2.16	0.069602	0.151971	-5.23
245361_at	hypothetical protein ;supported by f	---	AT4G17790	1.40	0.48	2.16	0.069603	0.151971	-5.23
263376_at	putative surface protein ; supported	FLA6	AT2G20520	-1.15	-0.20	-2.16	0.069618	0.151989	-5.23
263074_at	putative HMG protein ;supported b	HMGB4	AT2G17560	1.23	0.30	2.16	0.069661	0.152064	-5.23
258050_at	unknown protein	---	AT3G16200	-1.23	-0.29	-2.16	0.069666	0.152064	-5.23
263224_at	putative GTP-binding protein Simila	---	AT1G30580	1.26	0.33	2.15	0.069782	0.152304	-5.24
261537_at	carbonyl reductase, putative simila	---	AT1G01800	1.15	0.20	2.15	0.069796	0.15232	-5.24
258620_at	putative MYB family transcription fa	MYB107	AT3G02940	-1.14	-0.18	-2.15	0.069814	0.152344	-5.24
261651_at	unknown protein similar to unknown	SAT32	AT1G27760	1.41	0.49	2.15	0.069847	0.152403	-5.24
261013_at	fatty acid elongase 3-ketoacyl-CoA	ATUPS5	AT1G26440	1.28	0.35	2.15	0.069871	0.15244	-5.24
254141_at	glucose-6-phosphate isomerase gl	PGI1	AT4G24620	-1.18	-0.24	-2.15	0.069888	0.152462	-5.24
261749_at	dehydrin, putative similar to dehydr	ERD14	AT1G76180	-1.18	-0.23	-2.15	0.069904	0.152483	-5.24
263558_at	putative phosphatidylinositol/phospl	---	AT2G16380	-1.27	-0.34	-2.15	0.069912	0.152486	-5.24
251938_at	60S RIBOSOMAL PROTEIN L12 -	---	AT3G53430	1.26	0.33	2.15	0.069939	0.152529	-5.24
247725_at	putative Rab5-interacting protein -	---	AT5G59410	1.31	0.39	2.15	0.069946	0.152531	-5.24
254243_at	serine/threonine kinase - like prote	CRK13	AT4G23210	2.21	1.15	2.15	0.069975	0.152559	-5.24
250687_at	putative protein similar to unknown	---	AT5G06660	1.15	0.20	2.15	0.06999	0.152559	-5.24
246402_at	jasmonate inducible protein, putativ	---	AT1G57570	-1.16	-0.21	-2.15	0.069991	0.152559	-5.24

265169_x_a	unknown protein	---	AT1G23720	-1.19	-0.25	-2.15	0.070001	0.152559	-5.24
256459_at	acetyl-CoA carboxylase, putative s	ACC2	AT1G36180	1.24	0.31	2.15	0.070002	0.152559	-5.24
264520_at	putative amino acid permease GC	AAP8	AT1G10010	-1.32	-0.40	-2.15	0.070003	0.152559	-5.24
246541_at	ubiquitin-fusion degradation protei	---	AT5G15400	1.18	0.24	2.15	0.070006	0.152559	-5.24
253351_at	putative protein hemolysin, 38K, Sy	---	AT4G33700	1.19	0.26	2.15	0.070018	0.15257	-5.24
246740_at	putative protein	---	AT5G27810	-1.15	-0.21	-2.15	0.070037	0.152585	-5.24
244910_s_a	hypothetical protein	---	---	-1.19	-0.25	-2.15	0.070038	0.152585	-5.24
250469_at	pollen allergen -like protein SAH7 p	---	AT5G10130	-1.25	-0.32	-2.15	0.070062	0.152622	-5.24
245725_at	sucrose synthase, putative similar t	SUS6	AT1G73370	-1.45	-0.53	-2.15	0.070089	0.152666	-5.24
263743_at	coatomer alpha subunit	---	AT2G21390	-1.16	-0.21	-2.15	0.070101	0.152678	-5.24
259934_at	unknown protein	---	AT1G71340	1.29	0.37	2.15	0.070111	0.152687	-5.24
251420_at	transcription factor - like protein tra	---	AT3G60490	-1.27	-0.34	-2.15	0.070139	0.152731	-5.24
257668_at	sugar transporter, putative similar t	---	AT3G20460	-1.28	-0.36	-2.15	0.07016	0.152763	-5.24
267106_s_a	putative vacuolar sorting receptor id	ATVSR3	AT2G14720 ,	1.24	0.31	2.15	0.070185	0.152802	-5.24
246217_at	APETALA2 protein ; supported by c	AP2	AT4G36920	-1.37	-0.45	-2.15	0.07022	0.152866	-5.24
259259_at	spindly (gibberellin signal transduct	SPY	AT3G11540	1.19	0.25	2.15	0.070234	0.15288	-5.24
258017_at	unknown protein	---	AT3G19370	-1.40	-0.48	-2.15	0.070252	0.152906	-5.24
261323_at	hypothetical protein predicted by ge	---	AT1G44760	1.33	0.41	2.15	0.07027	0.152919	-5.24
254862_at	putative transport protein Na(+) de	---	AT4G12030	-1.36	-0.45	-2.15	0.070272	0.152919	-5.24
265567_at	unknown protein predicted by gene	---	AT2G05580	-1.22	-0.29	-2.15	0.070283	0.15293	-5.24
267025_at	unknown protein predicted by gens	---	AT2G38350	-1.14	-0.19	-2.15	0.070292	0.152933	-5.24
249497_at	putative protein predicted proteins -	---	AT5G39220	-1.22	-0.29	-2.15	0.070337	0.153012	-5.24
245525_at	dynein light chain like protein	---	AT4G15930	1.14	0.19	2.15	0.070341	0.153012	-5.24
255641_at	contains region of similarity to beta	---	AT4G00800	-1.19	-0.25	-2.15	0.070363	0.153046	-5.24
252958_at	putative transcription factor (MYB4)	MYB4	AT4G38620	-1.58	-0.66	-2.15	0.070404	0.153117	-5.24
247144_at	DOF zinc finger protein-like	---	AT5G65590	-1.30	-0.38	-2.15	0.070418	0.153117	-5.24
256360_at	hypothetical protein	---	AT1G66440	-1.27	-0.34	-2.15	0.070422	0.153117	-5.24
254811_at	putative protein 2-hydroxy-6-oxohe	---	AT4G12230	1.21	0.27	2.15	0.070423	0.153117	-5.24
252694_at	nodulin -like protein nodulin-21, Gly	---	AT3G43630	-1.19	-0.25	-2.15	0.070453	0.153167	-5.25
266357_at	putative beta-amylase	BAM6	AT2G32290	1.64	0.72	2.15	0.070473	0.153196	-5.25
252669_at	putative protein hypothetical protei	---	AT3G44100	1.18	0.24	2.15	0.070506	0.153253	-5.25
249537_at	cysteine-tRNA ligase -like protein c	---	AT5G38830	1.25	0.32	2.15	0.070557	0.153345	-5.25
264274_at	disease resistance protein RPS4, p	---	AT1G60320	-1.18	-0.24	-2.15	0.070561	0.153345	-5.25
247684_at	serine/threonine-specific protein kir	---	AT5G59670	-2.84	-1.50	-2.15	0.070604	0.153423	-5.25
264395_at	putative GDP-dissociation inhibitor	---	AT1G12070	-1.25	-0.32	-2.15	0.070612	0.153426	-5.25

257885_at	S1 self-incompatibility protein, puta	---	AT3G16970	-1.17	-0.23	-2.15	0.070628	0.153434	-5.25
257042_s_a	hypothetical protein predicted by ge	---	AT3G28640	1.17	0.22	2.15	0.07063	0.153434	-5.25
260636_at	hypothetical protein contains similar	ATCDS1	AT1G62430	-1.37	-0.45	-2.15	0.070636	0.153434	-5.25
265245_at	unknown protein	---	AT2G43060	1.38	0.47	2.14	0.070694	0.153544	-5.25
251718_at	putative protein hypothetical protein	MRLK	AT3G56100	-1.25	-0.32	-2.14	0.070721	0.153589	-5.25
257626_s_a	hypothetical protein	---	AT3G26240	-1.20	-0.26	-2.14	0.070752	0.153642	-5.25
258986_at	putative heat shock protein similar	---	AT3G08910	-1.19	-0.26	-2.14	0.070765	0.153655	-5.25
267015_at	unknown protein	---	AT2G39340	-1.23	-0.30	-2.14	0.070791	0.153697	-5.25
262647_at	putative growth regulator protein si	---	AT1G14020	-1.28	-0.36	-2.14	0.070833	0.153761	-5.25
257865_at	protein kinase, putative contains Pf	---	AT3G17750	-1.15	-0.20	-2.14	0.070834	0.153761	-5.25
262251_at	hypothetical protein predicted by ge	---	AT1G53760	1.36	0.44	2.14	0.070844	0.153767	-5.25
262657_at	ribonuclease contains similarity to R	---	AT1G14210	-1.51	-0.59	-2.14	0.070876	0.153824	-5.25
256965_at	branched chain alpha-keto acid de	DIN4	AT3G13450	1.46	0.54	2.14	0.070914	0.153892	-5.25
265969_at	En/Spm-like transposon protein rela	---	---	-1.18	-0.24	-2.14	0.070922	0.153893	-5.25
263940_at	putative calcium-dependent protein	CPK25	AT2G35890	-1.16	-0.21	-2.14	0.070941	0.153919	-5.25
245699_at	putative protein ;supported by full-le	---	AT5G04250	1.37	0.45	2.14	0.070947	0.153919	-5.25
254976_at	subtilisin-like serine protease conta	---	AT4G10510	-1.18	-0.23	-2.14	0.070973	0.153961	-5.25
261141_at	unknown protein contains Pfam pro	---	AT1G19740	1.21	0.28	2.14	0.071004	0.154014	-5.25
260110_at	hypothetical protein similar to NBS/	---	AT1G63350	-1.16	-0.22	-2.14	0.071019	0.154031	-5.25
264964_at	hypothetical protein predicted by ge	---	AT1G60460	-1.23	-0.30	-2.14	0.071037	0.154056	-5.25
248629_at	mitochondrial carrier protein-like	---	AT5G48970	-1.26	-0.34	-2.14	0.071097	0.154171	-5.25
258729_at	putative amino acid transporter prot	ANT1	AT3G11900	1.16	0.22	2.14	0.071132	0.154232	-5.25
247726_at	telomere repeat-binding protein	TRP1	AT5G59430	1.24	0.31	2.14	0.07115	0.154242	-5.25
258652_at	putative Ras-like GTP-binding prote	ATRABC2E	AT3G09910	-1.37	-0.45	-2.14	0.071153	0.154242	-5.25
258694_at	putative non-phototropic hypocotyl s	---	AT3G08660	-1.39	-0.47	-2.14	0.071157	0.154242	-5.26
248484_at	carbonyl reductase-like protein	---	AT5G51030	1.15	0.20	2.14	0.071164	0.154243	-5.26
250134_at	Expressed protein ; supported by cl	---	---	1.22	0.29	2.14	0.071183	0.154269	-5.26
251386_at	putative protein rec, Homo sapiens	---	AT3G60800	-1.15	-0.20	-2.14	0.071196	0.154282	-5.26
252951_at	disease resistance response like p	---	AT4G38700	-1.32	-0.40	-2.14	0.07121	0.154299	-5.26
258462_at	unknown protein	---	AT3G17350	-1.48	-0.57	-2.14	0.071218	0.154302	-5.26
249532_at	AtPP - like protein AtPP protein, Br	---	AT5G38780	1.48	0.57	2.14	0.071232	0.154317	-5.26
251614_at	hypothetical protein	---	AT3G57970	-1.23	-0.30	-2.14	0.071246	0.154332	-5.26
252046_at	putative protein extensin -Nicotiana	---	AT3G52460	-1.21	-0.28	-2.14	0.071281	0.154395	-5.26
251400_at	putative protein prib5, Ribes nigrun	---	AT3G60420	-2.03	-1.02	-2.14	0.071296	0.154411	-5.26
262037_at	hypothetical protein predicted by ge	---	---	-1.19	-0.25	-2.14	0.071302	0.154411	-5.26

250341_at	MAP3K delta-1 protein kinase	---	AT5G11850	-1.15	-0.21	-2.14	0.071325	0.154445	-5.26
251659_at	hypothetical protein ;supported by f	BIGYIN	AT3G57090	1.19	0.26	2.14	0.071376	0.15454	-5.26
254176_at	hypothetical protein hypothetical pr	---	AT4G24200	1.18	0.24	2.14	0.071399	0.154575	-5.26
247059_at	UTP-glucose glucosyltransferase ;s	UGT72E2	AT5G66690	1.18	0.24	2.14	0.071422	0.154597	-5.26
246428_at	spindle pole body protein-like spind	---	AT5G17410	1.24	0.30	2.14	0.071425	0.154597	-5.26
246059_at	putative protein KED, Nicotiana tab	---	AT5G08450	1.17	0.23	2.14	0.071429	0.154597	-5.26
245624_at	glucosyltransferase like protein	---	AT4G14090	-1.25	-0.32	-2.14	0.07148	0.154692	-5.26
255359_at	putative glucose-6-phosphate/phos	---	AT4G03950	-1.19	-0.25	-2.14	0.071525	0.154776	-5.26
248297_at	protochlorophyllide reductase; oxido	---	AT5G53100	-1.15	-0.20	-2.14	0.071532	0.154776	-5.26
250942_at	putative protein	---	AT5G03350	-4.36	-2.12	-2.14	0.071557	0.154811	-5.26
248037_at	sexual differentiation process protei	OPT1	AT5G55930	-1.51	-0.59	-2.14	0.071562	0.154811	-5.26
265999_at	unknown protein ; supported by cD	---	AT2G24100	-1.26	-0.33	-2.14	0.071598	0.154875	-5.26
245547_at	cytochrome P450 like protein	CYP702A2	AT4G15300	-1.16	-0.21	-2.14	0.071605	0.154875	-5.26
248282_at	unknown protein ;supported by full-	---	AT5G52900	-1.78	-0.84	-2.14	0.071646	0.154938	-5.26
261689_at	hypothetical protein contains similar	---	AT1G50140	1.26	0.33	2.14	0.071648	0.154938	-5.26
260715_at	hypothetical protein predicted by ge	---	AT1G48200	1.38	0.46	2.14	0.07167	0.154968	-5.26
260356_at	unknown protein ; supported by ful	ATMINE1	AT1G69390	-1.18	-0.24	-2.14	0.071675	0.154968	-5.26
260816_at	hypothetical protein predicted by ge	---	AT1G06930	-1.25	-0.32	-2.14	0.07169	0.154986	-5.26
246535_at	putative protein several hypothetica	---	AT5G15900	-1.27	-0.34	-2.14	0.071717	0.155028	-5.26
265630_at	unknown protein similar to ovarian t	---	AT2G27350	1.16	0.21	2.13	0.071739	0.155062	-5.26
260735_at	disease resistance protein, putative	---	AT1G17610	-1.39	-0.48	-2.13	0.071759	0.155089	-5.26
260863_at	hypothetical protein predicted by ge	---	---	-1.29	-0.37	-2.13	0.071769	0.155097	-5.26
247157_at	nuclear matrix constituent protein 1	LINC4	AT5G65770	1.19	0.25	2.13	0.071841	0.155239	-5.26
261130_at	unknown protein ; supported by cDI	PRMT10	AT1G04870	1.39	0.47	2.13	0.071894	0.155298	-5.27
264792_at	hypothetical protein similar to hypot	---	AT1G08620	1.18	0.24	2.13	0.071898	0.155298	-5.27
244942_at	hypothetical protein	---	---	-1.17	-0.22	-2.13	0.071899	0.155298	-5.27
250198_at	expressed protein contains similari	NAP14	AT5G14100	1.30	0.38	2.13	0.0719	0.155298	-5.27
265754_x_a	En/Spm transposon protein (mosaic	---	---	-1.16	-0.21	-2.13	0.071903	0.155298	-5.27
258423_at	hypothetical protein predicted by ge	---	AT3G16730	1.16	0.21	2.13	0.07193	0.155343	-5.27
266531_at	putative plasma membrane intrinsic	---	AT2G16835	-1.15	-0.20	-2.13	0.071968	0.155404	-5.27
254230_at	polyprenyltransferase like protein p	AtPPT1	AT4G23660	1.19	0.25	2.13	0.071972	0.155404	-5.27
261652_at	dimethyladenosine transferase, pu	PFC1	AT1G01860	1.21	0.28	2.13	0.071991	0.15543	-5.27
259252_at	hypothetical protein similar to hypo	IBM1	AT3G07610	1.17	0.22	2.13	0.072022	0.155471	-5.27
255327_at	putative malonyl-CoA decarboxylas	---	AT4G04320	1.15	0.20	2.13	0.072024	0.155471	-5.27
265492_at	hypothetical protein	AGL95	AT2G15660	-1.18	-0.24	-2.13	0.072039	0.155488	-5.27

255507_at	AtKAP alpha similar to AtKAP, Ger	MOS6	AT4G02150	1.15	0.20	2.13	0.072046	0.155489	-5.27
265136_at	hypothetical protein similar to disea	---	AT1G51270	-1.14	-0.19	-2.13	0.072056	0.155496	-5.27
265847_at	unknown protein	---	AT2G35750	1.16	0.22	2.13	0.072106	0.155582	-5.27
252160_at	proline-rich protein	---	AT3G50570	-1.25	-0.32	-2.13	0.072109	0.155582	-5.27
263465_at	hypothetical protein predicted by ge	---	AT2G31940	-1.23	-0.30	-2.13	0.072129	0.15561	-5.27
250202_at	putative protein similar to unknown	---	AT5G13970	1.21	0.28	2.13	0.072138	0.155615	-5.27
259428_at	MAP kinase, putative similar to MA	ATMPK11	AT1G01560	2.42	1.28	2.13	0.072158	0.15564	-5.27
248390_at	importin alpha subunit	IMPA-8	AT5G52000	-1.14	-0.19	-2.13	0.072166	0.15564	-5.27
266565_at	putative serine carboxypeptidase II	scpl23	AT2G24010	-1.20	-0.27	-2.13	0.07217	0.15564	-5.27
266595_at	hypothetical protein predicted by ge	GC4	AT2G46180	-1.20	-0.27	-2.13	0.0722	0.15569	-5.27
252158_at	CDPK-related protein kinase	CRK	AT3G50530	1.22	0.29	2.13	0.072214	0.155705	-5.27
254123_at	Bnm1 like protein pollen-specific pr	APPB1	AT4G24640	-1.18	-0.24	-2.13	0.072249	0.155765	-5.27
246764_s_a	putative protein At2g27780 and At2	---	AT2G13640	-1.15	-0.21	-2.13	0.072268	0.155782	-5.27
252506_at	putative protein UDP-galactose tra	UTR5	AT3G46180	1.21	0.27	2.13	0.07227	0.155782	-5.27
254643_at	putative protein DNA polymerase I	---	AT4G18820	-1.19	-0.26	-2.13	0.072284	0.155796	-5.27
263172_at	hypothetical protein predicted by ge	CID3	AT1G54170	-1.14	-0.19	-2.13	0.072292	0.155798	-5.27
252690_at	putative protein	---	---	-1.25	-0.32	-2.13	0.072346	0.155893	-5.27
259883_at	putative ribophorin I (dolichyl-dipho	---	AT1G76400	1.23	0.30	2.13	0.072349	0.155893	-5.27
254106_at	geranylgeranylated protein ATGP4	ATGP4	AT4G24990	1.23	0.29	2.13	0.072429	0.15605	-5.27
252253_at	Expressed protein ; supported by fu	---	AT3G49300	-1.14	-0.19	-2.13	0.072496	0.156179	-5.27
257903_at	unknown protein	---	AT3G28460	-1.18	-0.23	-2.13	0.072545	0.156272	-5.27
252965_at	putative auxin-induced protein auxi	---	AT4G38860	-1.54	-0.62	-2.13	0.072591	0.156349	-5.27
262662_at	hypothetical protein similar to hypot	---	AT1G13920	-1.22	-0.28	-2.13	0.072595	0.156349	-5.28
259852_at	disulfide bond formation protein, pu	AERO1	AT1G72280	1.46	0.55	2.13	0.072649	0.156438	-5.28
258367_at	putative protein kinase similar to pro	WAG2	AT3G14370	-1.20	-0.26	-2.13	0.072651	0.156438	-5.28
265021_at	unknown protein ;supported by full-	---	AT1G24610	-1.15	-0.20	-2.13	0.072657	0.156438	-5.28
261906_at	hypothetical protein predicted by ge	---	AT1G65080	1.34	0.42	2.13	0.072712	0.156528	-5.28
261837_s_a	transcriptional regulator, putative s	---	AT1G15910	1.25	0.33	2.13	0.072712	0.156528	-5.28
251651_at	putative protein transcription initiati	---	AT3G57370	1.14	0.19	2.13	0.072746	0.156586	-5.28
252685_at	disease resistence - like protein RF	---	AT3G44410	-1.36	-0.44	-2.13	0.07276	0.156602	-5.28
249966_at	putative protein COLD-INDUCIBLE	---	AT5G19030	1.38	0.47	2.12	0.072863	0.1568	-5.28
266092_at	hypothetical protein predicted by ge	---	AT2G37880	-1.19	-0.25	-2.12	0.072866	0.1568	-5.28
261829_at	P-glycoprotein, putative similar to F	PGP10	AT1G10680	-1.23	-0.30	-2.12	0.072874	0.156801	-5.28
250147_at	putative protein predicted proteins,	---	AT5G14600	1.30	0.37	2.12	0.072891	0.156824	-5.28
246537_at	putative protein protochlorophyllide	---	AT5G15940	-1.20	-0.26	-2.12	0.072899	0.156825	-5.28

266210_at	60S ribosomal protein L10A ; supp	PGY1	AT2G27530	1.36	0.44	2.12	0.072906	0.156827	-5.28
250574_at	putative protein putative transcripti	---	AT5G08230	1.16	0.22	2.12	0.072918	0.156838	-5.28
256864_at	topoisomerase II identical to topois	TOPII	AT3G23890	-1.25	-0.32	-2.12	0.072947	0.156884	-5.28
251681_at	putative protein hypothetical protei	---	AT3G57100	-1.21	-0.28	-2.12	0.072975	0.156931	-5.28
252553_at	putative protein	---	AT3G45910	-1.18	-0.23	-2.12	0.073035	0.157045	-5.28
252396_at	hypothetical protein	---	AT3G47980	1.17	0.22	2.12	0.073046	0.157053	-5.28
254223_at	putative protein Pennisetum ciliare	---	AT4G23730	1.15	0.20	2.12	0.073092	0.157138	-5.28
265439_at	senescence-associated protein con	---	AT2G21045	1.97	0.98	2.12	0.073168	0.157285	-5.28
267426_at	hypothetical protein predicted by ge	---	AT2G34820	-1.17	-0.23	-2.12	0.073224	0.157377	-5.28
252126_at	putative disease resistance protein	---	AT3G50950	-1.27	-0.35	-2.12	0.073224	0.157377	-5.28
246886_at	CLC-d chloride channel protein	CLC-D	AT5G26240	1.19	0.26	2.12	0.073242	0.157387	-5.28
264549_at	putative ser/thr protein kinase simila	---	AT1G09440	-1.17	-0.23	-2.12	0.073245	0.157387	-5.28
258124_at	Expressed protein ; supported by cl	---	AT3G18215	1.31	0.39	2.12	0.07325	0.157387	-5.28
251793_at	regulator of chromosome condensa	---	AT3G55580	-1.14	-0.19	-2.12	0.073284	0.157446	-5.28
260983_at	hypothetical protein predicted by ge	---	AT1G53560	-1.37	-0.45	-2.12	0.073364	0.157604	-5.29
246030_at	Expressed protein genomic sequer	---	AT5G21105	-1.18	-0.24	-2.12	0.073391	0.157648	-5.29
259128_at	unknown protein similar to pushove	BIG	AT3G02260	1.30	0.38	2.12	0.073434	0.157723	-5.29
245321_at	Expressed protein ; supported by fu	---	AT4G15545	1.15	0.20	2.12	0.073462	0.157768	-5.29
265842_at	putative AP2 domain transcription fa	ERF38	AT2G35700	-1.45	-0.54	-2.12	0.073479	0.157789	-5.29
261734_at	hypothetical protein similar to hypot	---	AT1G47790	-1.16	-0.22	-2.12	0.073487	0.157789	-5.29
257596_at	unknown protein	---	AT3G24760	1.21	0.27	2.12	0.073492	0.157789	-5.29
259226_at	unknown protein similar to hypothe	---	AT3G07700	-1.24	-0.31	-2.12	0.073543	0.157883	-5.29
261046_at	flavonol 3-o-glucosyltransferase, pu	---	AT1G01390	1.30	0.38	2.12	0.073612	0.157997	-5.29
262620_at	hypothetical protein predicted by ge	---	AT1G06540	-1.16	-0.21	-2.12	0.073615	0.157997	-5.29
248928_at	Rac-like gtp binding protein ARAC2	ARAC2	AT5G45970	-1.13	-0.18	-2.12	0.073616	0.157997	-5.29
261638_at	Expressed protein ; supported by fu	---	AT1G49975	-1.41	-0.49	-2.12	0.073638	0.158028	-5.29
251722_at	putative protein neuronal glutamine	---	AT3G56200	1.37	0.45	2.12	0.073652	0.158044	-5.29
267640_at	photomorphogenesis repressor (CO	COP1	AT2G32950	1.24	0.31	2.12	0.073661	0.158048	-5.29
248264_at	putative protein similar to unknown	---	AT5G53310	-1.21	-0.27	-2.12	0.073706	0.15812	-5.29
261405_at	unknown protein ;supported by full-	---	AT1G18740	-1.41	-0.50	-2.12	0.073709	0.15812	-5.29
249989_at	putative protein FACTOR ASSOCI	---	AT5G18525	1.21	0.27	2.12	0.073739	0.158172	-5.29
246757_at	Expressed protein ; supported by fu	WPP3	AT5G27940	-1.15	-0.20	-2.12	0.073786	0.158257	-5.29
265538_at	unknown protein predicted by gens	---	AT2G15860	1.26	0.33	2.11	0.073838	0.158354	-5.29
264412_at	hypothetical protein similar to putati	---	AT1G43260	-1.16	-0.22	-2.11	0.073901	0.158474	-5.29
252340_at	myb-like protein	AtMYB45	AT3G48920	1.30	0.38	2.11	0.073945	0.158552	-5.29

245769_at	hypothetical protein similar to putati	INT2	AT1G30220	1.16	0.21	2.11	0.073955	0.158559	-5.29
251071_at	putative protein receptor protein kin	---	AT5G01950	1.33	0.41	2.11	0.073969	0.158559	-5.29
258023_at	cinnamyl alcohol dehydrogenase id	ATCAD4	AT3G19450	1.53	0.61	2.11	0.073973	0.158559	-5.29
259177_at	hypothetical protein similar to putati	---	AT3G01630	-1.16	-0.22	-2.11	0.073976	0.158559	-5.29
265308_at	putative protein kinase contains a p	ALE2	AT2G20300	-1.14	-0.19	-2.11	0.07399	0.158576	-5.29
266850_at	unknown protein	---	AT2G26850	-1.16	-0.21	-2.11	0.074009	0.1586	-5.29
250065_at	putative protein predicted proteins,	---	AT5G17910	-1.27	-0.34	-2.11	0.074027	0.158623	-5.29
252254_at	hypothetical protein ; supported by	DMS3	AT3G49250	-1.16	-0.21	-2.11	0.074079	0.15872	-5.30
255033_at	putative protein phosphonopyruvat	---	AT4G09520	-1.23	-0.30	-2.11	0.074162	0.158884	-5.30
248945_at	putative protein strong similarity to u	---	AT5G45510	-1.22	-0.29	-2.11	0.074183	0.158901	-5.30
263889_at	putative ABC transporter	ATNAP12	AT2G37010	-1.21	-0.27	-2.11	0.074184	0.158901	-5.30
256081_at	hypothetical protein contains Pfam	WOX14	AT1G20700	-1.20	-0.27	-2.11	0.074263	0.159056	-5.30
267284_at	unknown protein predicted by gens	---	AT2G23700	-1.24	-0.31	-2.11	0.074297	0.159092	-5.30
267058_at	putative protein kinase contains a p	MAPKKK17	AT2G32510	-1.31	-0.39	-2.11	0.074302	0.159092	-5.30
264854_at	putative RING zinc finger protein ; s	RHA3A	AT2G17450	-1.21	-0.28	-2.11	0.074307	0.159092	-5.30
257160_at	hypothetical protein predicted by ge	---	---	-1.29	-0.37	-2.11	0.074308	0.159092	-5.30
251172_at	putative protein chloroplast ribosom	RRF	AT3G63190	-1.17	-0.23	-2.11	0.074333	0.159132	-5.30
246544_at	auxin transport protein - like auxin t	PIN8	AT5G15100	-1.16	-0.21	-2.11	0.074406	0.159272	-5.30
262830_at	purple acid phosphatase, putative (	PAP3	AT1G14700	1.31	0.39	2.11	0.074423	0.159293	-5.30
262460_s_a	fructokinase, putative similar to fruc	---	AT1G06030	1.16	0.22	2.11	0.074448	0.159331	-5.30
261287_at	hypothetical protein predicted by ge	---	AT1G37020	-1.14	-0.18	-2.11	0.074501	0.159419	-5.30
263047_at	putative phosphoserine aminotrans	---	AT2G17630	1.42	0.51	2.11	0.074503	0.159419	-5.30
253807_at	putative GPI-anchored protein GPI	---	AT4G28280	-1.25	-0.32	-2.11	0.074538	0.15948	-5.30
264684_at	unknown protein	HEXO3	AT1G65590	-1.40	-0.48	-2.11	0.074576	0.159542	-5.30
250387_at	putative protein predicted proteins,	---	AT5G11290	-1.15	-0.20	-2.11	0.074581	0.159542	-5.30
247477_at	putative protein 21K protein precur	---	AT5G62340	-1.26	-0.34	-2.11	0.074618	0.159606	-5.30
248181_at	cytochrome c biogenesis protein pr	---	AT5G54290	-1.27	-0.34	-2.11	0.074685	0.159735	-5.30
261156_s_a	hypothetical protein predicted by ge	---	AT1G34490	-1.17	-0.22	-2.11	0.074733	0.159821	-5.30
266278_at	putative tropinone reductase	---	AT2G29300	-1.56	-0.64	-2.11	0.07474	0.159821	-5.30
247808_at	contains similarity to unknown prote	ECT10	AT5G58190	-1.15	-0.21	-2.11	0.074796	0.159928	-5.30
265880_at	unknown protein	---	AT2G42300	1.28	0.36	2.11	0.074824	0.159971	-5.31
250077_at	putative protein	---	AT5G16680	1.16	0.21	2.11	0.074865	0.160045	-5.31
251752_at	proline transporter 2	PROT2	AT3G55740	-1.31	-0.39	-2.10	0.07492	0.160147	-5.31
255203_at	hypothetical protein	---	---	-1.25	-0.32	-2.10	0.074932	0.160154	-5.31
248084_at	sodium proton exchanger	NHX3	AT5G55470	-1.26	-0.33	-2.10	0.07494	0.160154	-5.31

250225_at	Expressed protein ; supported by fu	---	AT5G14105	1.25	0.32	2.10	0.074944	0.160154	-5.31
250296_at	heat shock protein 17.6-II ;support	HSP17.6II	AT5G12020	1.47	0.56	2.10	0.075119	0.160492	-5.31
251250_at	putative protein pectin methylester	---	AT3G62180	-1.25	-0.32	-2.10	0.07512	0.160492	-5.31
263221_at	UDP-galactose 4-epimerase-like pr	MUR4	AT1G30620	1.67	0.74	2.10	0.075124	0.160492	-5.31
265143_at	unknown protein	NFU5	AT1G51390	1.22	0.28	2.10	0.075201	0.160641	-5.31
253512_at	putative protein protein phosphatas	WIN2	AT4G31750	-1.18	-0.24	-2.10	0.075257	0.160732	-5.31
251466_at	putative protein hypothetical protei	---	AT3G59340	-1.22	-0.29	-2.10	0.075257	0.160732	-5.31
257609_at	Expressed protein ; supported by fu	---	AT3G13845	1.21	0.28	2.10	0.075266	0.160736	-5.31
264584_at	unknown protein Similar to Synecho	---	AT1G05140	1.21	0.27	2.10	0.075381	0.160958	-5.31
266384_at	hypothetical protein predicted by ge	---	AT2G14660	-1.21	-0.27	-2.10	0.075384	0.160958	-5.31
259435_at	hypothetical protein similar to putati	---	AT1G01450	-1.16	-0.21	-2.10	0.075454	0.161092	-5.31
259864_at	hypothetical protein predicted by ge	---	AT1G72800	1.36	0.44	2.10	0.075495	0.16114	-5.31
255985_at	putative protein contains similarity t	---	AT1G34150	1.15	0.21	2.10	0.075495	0.16114	-5.31
254282_at	hypothetical protein	---	AT4G22860	-1.17	-0.22	-2.10	0.075498	0.16114	-5.31
263678_at	hypothetical protein Similar to Babe	---	AT1G04420	-1.32	-0.40	-2.10	0.075505	0.16114	-5.31
264056_at	putative DOF zinc finger protein ;su	---	AT2G28510	-1.27	-0.34	-2.10	0.075513	0.16114	-5.31
264208_at	putative polyA-binding protein, PAB	PAB3	AT1G22760	-1.31	-0.39	-2.10	0.075519	0.16114	-5.31
263325_at	putative RING zinc finger protein ;	XERICO	AT2G04240	1.41	0.49	2.10	0.075527	0.161142	-5.31
246109_at	hypothetical protein (fragment?)	---	AT5G20120	-1.22	-0.29	-2.10	0.075592	0.16126	-5.32
264579_at	Expressed protein ; supported by fu	---	AT1G05205	1.26	0.34	2.10	0.075596	0.16126	-5.32
259440_at	hypothetical protein predicted by ge	BPS1	AT1G01550	1.17	0.22	2.10	0.075627	0.161311	-5.32
254972_at	putative protein more than 10 putati	---	AT4G10440	-1.20	-0.26	-2.10	0.075642	0.161327	-5.32
245435_at	hypothetical protein	AT4G1714	---	-1.15	-0.20	-2.10	0.075665	0.161362	-5.32
248813_at	Expressed protein ; supported by fu	VAM3	AT5G46860	1.27	0.34	2.10	0.075697	0.161414	-5.32
254118_at	DNA polymerase III like protein DN	---	AT4G24790	1.16	0.22	2.10	0.075712	0.16143	-5.32
259587_at	unknown protein ;supported by full-	---	AT1G28120	1.19	0.25	2.10	0.075764	0.161527	-5.32
261027_at	cyclic nucleotide and calmodulin-re	ATCNGC1	AT1G01340	1.64	0.71	2.10	0.075814	0.161619	-5.32
264061_at	putative cyclin-dependent kinase re	CKS2	AT2G27970	1.43	0.52	2.10	0.075843	0.161664	-5.32
255895_at	12-oxophytodienoate reductase, pu	---	AT1G17990	-1.82	-0.86	-2.10	0.075905	0.161767	-5.32
264016_at	putative auxin-regulated protein	---	AT2G21220	-1.19	-0.26	-2.10	0.075905	0.161767	-5.32
252526_at	putative protein ethylene-forming e	---	AT3G46480	-1.23	-0.30	-2.10	0.075926	0.161797	-5.32
259325_at	unknown protein	---	AT3G05320	-2.78	-1.47	-2.10	0.075936	0.161804	-5.32
249791_at	amino acid transporter ; supported	AAP7	AT5G23810	-1.32	-0.40	-2.09	0.076016	0.161958	-5.32
258497_at	putative flowering-time gene CONS	COL2	AT3G02380	-1.19	-0.25	-2.09	0.076028	0.161968	-5.32
257572_at	hypothetical protein predicted by ge	---	AT3G16880	-1.22	-0.28	-2.09	0.076043	0.161985	-5.32

247012_at	putative protein contains similarity t	TRB2	AT5G67580	1.19	0.25	2.09	0.076068	0.162024	-5.32
263219_at	putative serine proteinase Strong si	---	AT1G30600	-1.27	-0.34	-2.09	0.076095	0.162059	-5.32
261764_at	receptor lectin kinase, putative sim	---	AT1G15530	-1.20	-0.27	-2.09	0.076104	0.162059	-5.32
266426_x_a	hypothetical protein predicted by ge	---	AT2G07140	1.35	0.43	2.09	0.076106	0.162059	-5.32
261461_at	hypothetical protein predicted by ge	---	AT1G07860	-1.21	-0.27	-2.09	0.076139	0.162115	-5.32
250410_at	putative protein putative ATP/GTP-	---	AT5G11010	1.29	0.37	2.09	0.076156	0.162136	-5.32
249869_at	acetyl-CoA synthetase-like protein	AAE17	AT5G23050	1.28	0.36	2.09	0.076164	0.162138	-5.32
265086_at	hypothetical protein contains Zinc f	RING1B	AT1G03770	1.27	0.34	2.09	0.076232	0.162267	-5.32
258611_at	unknown protein ;supported by full-	---	AT3G02860	1.18	0.24	2.09	0.076259	0.162284	-5.32
257759_at	hypothetical protein	---	AT3G23070	1.29	0.37	2.09	0.076262	0.162284	-5.32
259792_at	unknown protein ; supported by cDI	CAD1	AT1G29690	-1.62	-0.70	-2.09	0.076266	0.162284	-5.32
258930_at	unknown protein predicted by gens	---	AT3G10040	-1.17	-0.23	-2.09	0.076269	0.162284	-5.32
252061_at	hypothetical protein phosphate acty	---	AT3G52620	-1.16	-0.22	-2.09	0.076278	0.162284	-5.32
254706_at	putative protein DNA-binding prote	---	AT4G17950	-1.26	-0.33	-2.09	0.076283	0.162284	-5.32
252152_at	putative protein several hypothetica	---	AT3G51350	-1.17	-0.22	-2.09	0.076372	0.162458	-5.33
251590_at	pollen-specific protein BAN102-like	AGP23	AT3G57690	-1.27	-0.34	-2.09	0.07638	0.162461	-5.33
249223_at	receptor lectin kinase-like protein	---	AT5G42120	1.20	0.27	2.09	0.076554	0.162815	-5.33
248713_at	putative protein contains similarity t	NSP5	AT5G48180	1.45	0.54	2.09	0.07657	0.162834	-5.33
263079_at	putative retroelement pol polyprotei	---	---	-1.16	-0.22	-2.09	0.076612	0.162895	-5.33
257404_at	hypothetical protein predicted by ge	SUVR1	AT1G04050	-1.18	-0.24	-2.09	0.076615	0.162895	-5.33
246675_at	putative protein	---	AT5G30420	-1.19	-0.26	-2.09	0.07662	0.162895	-5.33
265457_at	unknown protein ; supported by ful	---	AT2G46550	1.24	0.30	2.09	0.076637	0.162907	-5.33
246125_at	Expressed protein ; supported by fu	---	AT5G19875	1.71	0.77	2.09	0.07664	0.162907	-5.33
267176_at	putative zinc finger protein	---	AT2G37730	-1.19	-0.26	-2.09	0.076672	0.16296	-5.33
251126_at	putative protein various predicted p	---	AT5G01070	-1.16	-0.22	-2.09	0.076685	0.162963	-5.33
251965_at	putative protein variety of predictec	---	AT3G53360	1.19	0.25	2.09	0.076688	0.162963	-5.33
251729_at	alpha-galactosidase-like protein alp	---	AT3G56310	1.36	0.44	2.09	0.076705	0.162984	-5.33
254989_at	retrotransposon like protein copia-l	---	---	-1.21	-0.27	-2.09	0.076745	0.163055	-5.33
261447_at	transcription factor, putative similar	---	AT1G21160	1.18	0.23	2.09	0.076755	0.163061	-5.33
262382_at	virus resistance protein, putative si	---	AT1G72920	2.31	1.21	2.09	0.076786	0.163112	-5.33
255271_at	putative protein (ubiquitin like) to va	---	AT4G05260	-1.20	-0.26	-2.09	0.07684	0.1632	-5.33
257941_at	putative UDP-glucose glucosyltrans	UGT71B8	AT3G21800	-1.17	-0.22	-2.09	0.076843	0.1632	-5.33
261125_at	zinc finger protein 2, putative simila	---	AT1G04990	1.37	0.45	2.09	0.076849	0.1632	-5.33
263058_at	putative non-LTR retrolelement reve	---	---	-1.19	-0.25	-2.09	0.07687	0.163228	-5.33
266781_at	putative protein kinase contains a p	---	AT2G28940	1.25	0.32	2.09	0.076918	0.163315	-5.33

246033_at	hydroxymethylbilane synthase ;sup	HEMC	AT5G08280	-1.26	-0.34	-2.09	0.076938	0.163344	-5.33
246507_at	lipase-like protein monoglyceride li	---	AT5G16120	-1.29	-0.37	-2.09	0.076999	0.163443	-5.33
245089_at	putative transketolase precursor ; s	---	AT2G45290	2.19	1.13	2.09	0.077	0.163443	-5.33
252267_at	putative protein NITROGEN REGU	---	AT3G49640	-1.27	-0.35	-2.09	0.077018	0.163466	-5.33
266285_at	unknown protein	---	AT2G29180	-1.39	-0.47	-2.09	0.077082	0.163587	-5.33
248295_at	putative protein contains similarity t	---	AT5G53070	1.20	0.26	2.09	0.077096	0.163588	-5.33
264001_at	putative peroxidase ;supported by f	---	AT2G22420	-1.39	-0.47	-2.09	0.077096	0.163588	-5.33
266859_at	putative MADS-box protein	AGL41	AT2G26880	-1.21	-0.28	-2.09	0.077119	0.16362	-5.34
251224_at	hypothetical protein	---	AT3G62620	1.20	0.27	2.08	0.077128	0.163625	-5.34
252948_at	putative protein NLS receptor - Ory	KAK	AT4G38600	-1.20	-0.26	-2.08	0.07714	0.163634	-5.34
248380_at	phosphoglucomutase (emb|CAB64	PGM	AT5G51820	1.40	0.48	2.08	0.077179	0.163702	-5.34
260349_at	mitotic checkpoint protein, putative	---	AT1G69400	-1.20	-0.26	-2.08	0.077211	0.163751	-5.34
251624_at	hypothetical protein ;supported by f	---	AT3G57280	1.23	0.30	2.08	0.077216	0.163751	-5.34
257999_at	hypothetical protein similar to hypot	---	AT3G27540	-1.37	-0.45	-2.08	0.077251	0.163805	-5.34
262086_at	GTP-binding protein, putative simil	---	AT1G56050	1.22	0.29	2.08	0.07726	0.163805	-5.34
248066_at	putative protein contains similarity t	QRT1	AT5G55590	-1.19	-0.25	-2.08	0.077263	0.163805	-5.34
252217_at	putative protein some putative prote	---	AT3G50140	-1.28	-0.35	-2.08	0.077275	0.163814	-5.34
265236_s_a	hypothetical protein	---	AT2G07714	-1.18	-0.24	-2.08	0.077286	0.163823	-5.34
262287_at	unknown protein ;supported by full-	---	AT1G68660	1.20	0.26	2.08	0.077297	0.16383	-5.34
250830_at	putative protein similar to unknown	---	AT5G04910	1.25	0.32	2.08	0.077316	0.163857	-5.34
247442_s_a	tubulin beta-2/beta-3 chain (sp|P29	TUB2	AT5G62690	-1.33	-0.42	-2.08	0.077333	0.163877	-5.34
248967_at	unknown protein ;supported by full-	---	AT5G45350	-1.24	-0.30	-2.08	0.077351	0.163877	-5.34
258022_at	hypothetical protein predicted by ge	---	AT3G19460	-1.14	-0.18	-2.08	0.077356	0.163877	-5.34
253929_s_a	putative protein H+-transporting AT	---	AT2G16030	-1.20	-0.26	-2.08	0.077363	0.163877	-5.34
250348_at	putative protein	---	AT5G11990	-1.22	-0.29	-2.08	0.077364	0.163877	-5.34
265542_at	putative SNF2 subfamily transcripti	SYD	AT2G28290	1.15	0.21	2.08	0.077369	0.163877	-5.34
259585_at	hypothetical protein contains similar	---	AT1G28090	1.17	0.22	2.08	0.077413	0.163954	-5.34
266609_at	unknown protein ; supported by cD	SRO1	AT2G35510	1.26	0.33	2.08	0.077483	0.164081	-5.34
251415_at	putative protein	---	AT3G60380	-1.17	-0.23	-2.08	0.077499	0.164081	-5.34
253594_at	Expressed protein ; supported by fu	---	AT4G30820	1.21	0.28	2.08	0.0775	0.164081	-5.34
246409_at	CRK1 protein, putative similar to C	---	AT1G57700	-1.26	-0.34	-2.08	0.077505	0.164081	-5.34
259647_at	acetyl-CoA synthetase, putative sir	---	AT1G55325	1.21	0.27	2.08	0.077509	0.164081	-5.34
245966_at	AP2 domain containing protein RAF	RAP2.11	AT5G19790	-1.19	-0.25	-2.08	0.077532	0.164116	-5.34
250154_at	putative aldose 1-epimerase - like	---	AT5G15140	-1.14	-0.19	-2.08	0.077553	0.164137	-5.34
249684_s_a	cytochrome P450-like	---	AT5G36110	-1.16	-0.21	-2.08	0.077557	0.164137	-5.34

265957_at	hypothetical protein predicted by ge	---	AT2G37300	-1.13	-0.18	-2.08	0.077583	0.164178	-5.34
264682_at	polygalacturonase, putative similar	---	AT1G65570	-1.15	-0.20	-2.08	0.077615	0.16423	-5.34
263619_at	putative GDP-mannose pyrophosp	---	AT2G04650	-1.18	-0.24	-2.08	0.077628	0.164241	-5.34
265777_at	hypothetical protein predicted by ge	---	AT2G07340	1.16	0.22	2.08	0.077637	0.164247	-5.34
258862_at	putative mitochondrial processing p	MPPBETA	AT3G02090	1.18	0.24	2.08	0.07765	0.164258	-5.34
255255_at	coded for by A. thaliana cDNA T447	---	AT4G05070	1.32	0.40	2.08	0.077666	0.164276	-5.34
257749_at	actin 2 identical to GB:AAB37098 f	ACT2	AT3G18780	-1.16	-0.21	-2.08	0.077733	0.164388	-5.34
266657_at	unknown protein ;supported by full-	---	AT2G25910	-1.14	-0.18	-2.08	0.077733	0.164388	-5.34
245812_at	hypothetical protein predicted by ge	---	---	-1.16	-0.22	-2.08	0.077775	0.164462	-5.34
253934_at	putative beta-1,3-glucanase beta-1	---	AT4G26830	-1.17	-0.22	-2.08	0.077808	0.164501	-5.34
250336_at	alpha-glucosidase 1 ; supported by	---	AT5G11720	-1.22	-0.29	-2.08	0.077808	0.164501	-5.34
246325_at	hypothetical protein contains similar	ABA3	AT1G16540	1.50	0.58	2.08	0.077818	0.164507	-5.34
260663_at	hypothetical protein predicted by ge	NFD5	AT1G19520	1.32	0.40	2.08	0.077845	0.164548	-5.34
249959_at	NADH:ubiquinone oxidoreductase	---	AT5G18800	1.24	0.30	2.08	0.077853	0.16455	-5.34
260120_at	hypothetical protein	ATPP2-A4	AT1G33920	-1.18	-0.24	-2.08	0.077884	0.1646	-5.34
266301_at	putative MYB family transcription fa	AtMYB81	AT2G26960	-1.27	-0.34	-2.08	0.077892	0.164602	-5.35
259554_at	hypothetical protein	---	---	1.25	0.32	2.08	0.077929	0.164666	-5.35
248898_at	outward rectifying potassium chann	KCO2	AT5G46370	-1.21	-0.28	-2.08	0.077941	0.164676	-5.35
249162_at	Expressed protein ; supported by fu	---	AT5G42765	-1.26	-0.33	-2.08	0.077978	0.164739	-5.35
245204_at	1-AMINOCYCLOPROPANE-1-CAR	---	AT5G12270	-1.28	-0.36	-2.08	0.078047	0.164869	-5.35
245764_s_a	hypothetical protein contains similar	ATCSA-1	AT1G19750 ,	-1.16	-0.21	-2.08	0.078107	0.16498	-5.35
251280_at	putative protein beta-1,3-glucanase	---	AT3G61810	-1.17	-0.23	-2.08	0.078171	0.1651	-5.35
245928_s_a	vegetative storage protein Vsp1 ;su	VSP1	AT5G24770 ,	-3.02	-1.59	-2.08	0.078189	0.165124	-5.35
250658_at	C3HC4-type RING zinc finger prote	---	AT5G07040	-1.21	-0.27	-2.08	0.078206	0.165143	-5.35
267633_at	hypothetical protein predicted by ge	---	AT2G42180	-1.15	-0.20	-2.08	0.07823	0.16518	-5.35
254634_at	putative protein transcription factor	---	AT4G18650	-1.20	-0.26	-2.07	0.078284	0.165277	-5.35
246270_at	putative protein	---	AT4G36500	1.81	0.86	2.07	0.078309	0.165316	-5.35
254269_at	putative serine/threonine kinase M/	---	AT4G23050	-1.22	-0.29	-2.07	0.078324	0.165327	-5.35
262490_at	hypothetical protein predicted by ge	UREF	AT1G21840	-1.23	-0.30	-2.07	0.078329	0.165327	-5.35
253233_at	putative protein various predicted p	---	AT4G34290	1.32	0.40	2.07	0.078353	0.165363	-5.35
262152_s_a	hypothetical protein predicted by ge	---	AT1G52430 ,	1.17	0.22	2.07	0.078416	0.165479	-5.35
254108_at	putative protein	RIN2	AT4G25230	-1.21	-0.27	-2.07	0.078473	0.165584	-5.35
260163_at	putative carnitine/acylcarnitine trans	BAC2	AT1G79900	1.68	0.75	2.07	0.078512	0.165652	-5.35
263878_s_a	unknown protein	---	AT2G22040 ,	1.22	0.28	2.07	0.078536	0.16568	-5.35
265016_at	unknown protein similar to deacety	---	AT1G24420	-1.14	-0.19	-2.07	0.07854	0.16568	-5.35

264158_at	hypothetical protein contains similar	PTAC4	AT1G65260	-1.22	-0.29	-2.07	0.078552	0.16569	-5.35
249069_at	unknown protein	---	AT5G44010	-1.44	-0.52	-2.07	0.078628	0.165836	-5.35
253766_at	putative protein ;supported by full-l	---	AT4G28770	-1.17	-0.23	-2.07	0.078652	0.16587	-5.35
249171_at	putative protein contains similarity t	---	AT5G42940	1.23	0.30	2.07	0.078707	0.16597	-5.36
249452_at	Sec7/gnom -like protein GNOM, Ar	---	AT5G39500	-1.17	-0.23	-2.07	0.078728	0.166	-5.36
261517_at	unknown protein ; supported by full-	---	AT1G71780	1.20	0.26	2.07	0.078754	0.166039	-5.36
256668_at	hypothetical protein similar to hypot	---	AT3G32190	-1.20	-0.27	-2.07	0.078806	0.166133	-5.36
265794_at	unknown protein	---	AT2G01310	1.15	0.20	2.07	0.078821	0.166149	-5.36
250317_at	tubulin beta-6 chain (sp|P29514) ; s	TUB6	AT5G12250	-1.63	-0.70	-2.07	0.078864	0.166226	-5.36
250527_at	serine/threonine-protein kinase	SNRK2.1	AT5G08590	1.16	0.21	2.07	0.079074	0.166652	-5.36
259602_at	disease resistance protein, putative	---	AT1G56520	-1.15	-0.20	-2.07	0.079096	0.166683	-5.36
245170_at	60S ribosomal protein L18, 5'partia	---	AT2G47570	-1.18	-0.24	-2.07	0.079123	0.166724	-5.36
263757_at	cysteine proteinase similar to cyste	---	AT2G21430	1.30	0.38	2.07	0.079131	0.166725	-5.36
266317_at	calmodulin identical to GB:L18912;	CAM5	AT2G27030	-1.17	-0.23	-2.07	0.07916	0.166772	-5.36
251819_at	phosphatidylglycerophosphate syn	PGPS2	AT3G55030	-1.19	-0.25	-2.07	0.079169	0.166776	-5.36
253202_at	Expressed protein ; supported by fu	---	AT4G34555	1.40	0.49	2.07	0.07921	0.166846	-5.36
267354_at	putative MYB family transcription fa	MYB25	AT2G39880	-1.30	-0.38	-2.07	0.079236	0.166873	-5.36
250146_at	putative protein polypeptide deform	PDF1B	AT5G14660	-1.32	-0.40	-2.07	0.079237	0.166873	-5.36
247267_at	40S ribosomal protein S28 (sp|P341	RPS28	AT5G64140	1.42	0.50	2.07	0.079245	0.166873	-5.36
262451_at	receptor-associated kinase isolog	SUB	AT1G11130	-1.33	-0.41	-2.07	0.079263	0.166897	-5.36
266549_at	Expressed protein ; supported by fu	EXL1	AT2G35150	-1.19	-0.25	-2.07	0.079293	0.166944	-5.36
262356_at	hypothetical protein predicted by ge	---	AT1G73000	-1.16	-0.21	-2.07	0.079326	0.166997	-5.36
253898_s_a	tryptophan synthase beta-subunit (T	TSB2	AT4G27070	1.65	0.72	2.07	0.079339	0.167008	-5.36
254404_at	hypothetical protein	B70	AT4G21340	-1.13	-0.18	-2.07	0.079345	0.167008	-5.36
255333_at	putative polyprotein	---	---	-1.16	-0.21	-2.07	0.079362	0.167028	-5.36
257823_at	integral membrane protein, putative	---	AT3G25190	-1.20	-0.26	-2.06	0.079413	0.16712	-5.36
264154_at	unknown protein	---	AT1G65430	-1.17	-0.23	-2.06	0.079443	0.167168	-5.36
252692_at	cysteine proteinase contains simila	---	AT3G43960	-1.48	-0.57	-2.06	0.079453	0.167173	-5.36
249775_at	squalene monooxygenase 1,2 (squ	SQE6	AT5G24160	1.32	0.40	2.06	0.079478	0.16721	-5.37
259076_at	hypothetical protein predicted by ge	TMAC2	AT3G02140	-2.00	-1.00	-2.06	0.079496	0.167211	-5.37
260011_at	hypothetical protein similar to hypo	---	AT1G68110	-1.22	-0.29	-2.06	0.079497	0.167211	-5.37
262305_at	hypothetical protein predicted by ge	---	AT1G70950	-1.17	-0.22	-2.06	0.0795	0.167211	-5.37
248741_at	putative protein similar to unknown	SLY2	AT5G48170	1.29	0.37	2.06	0.079526	0.167249	-5.37
259987_at	thaumatin-like protein (PR-5) simila	ATLP-3	AT1G75030	-1.24	-0.31	-2.06	0.079538	0.167256	-5.37
254927_at	putative protein hypothetical protei	---	AT4G11400	-1.21	-0.28	-2.06	0.079544	0.167256	-5.37

254281_at	predicted protein ileal sodium-depe	---	AT4G22840	1.68	0.74	2.06	0.079587	0.167331	-5.37
255101_at	putative lipid transfer protein simila	---	AT4G08670	-1.23	-0.30	-2.06	0.079716	0.167587	-5.37
246608_at	putative protein various predicted p	---	AT5G35380	-1.21	-0.27	-2.06	0.079809	0.167767	-5.37
250401_at	putative protein HSPC184, Homo s	---	AT5G10780	1.15	0.21	2.06	0.079816	0.167767	-5.37
263846_at	putative RNA polymerase sigma-70	SIGF	AT2G36990	1.27	0.35	2.06	0.079834	0.167789	-5.37
263381_at	putative MADS-box protein	AGL48	AT2G40210	-1.16	-0.21	-2.06	0.079948	0.168013	-5.37
247663_at	putative protein predicted proteins,	APUM18	AT5G60110	1.15	0.20	2.06	0.080007	0.168119	-5.37
259018_at	unknown protein similar to unknown	AIR12	AT3G07390	1.72	0.79	2.06	0.080013	0.168119	-5.37
258977_s_a	putative aspartate kinase similar to	AK3	AT3G02020	-1.30	-0.37	-2.06	0.080035	0.168149	-5.37
247614_at	putative protein	---	AT5G60240	-1.16	-0.21	-2.06	0.080057	0.168177	-5.37
245631_at	tRNA-glutamine synthetase, putativ	OVA9	AT1G25350	1.16	0.22	2.06	0.080063	0.168177	-5.37
254932_at	putative translation elongation facto	---	AT4G11120	1.29	0.37	2.06	0.080075	0.168187	-5.37
245017_at	PSI I protein	---	---	-1.20	-0.27	-2.06	0.080171	0.168374	-5.37
252660_at	putative protein	---	AT3G44440	-1.15	-0.21	-2.06	0.080213	0.168446	-5.37
252984_at	cinnamyl-alcohol dehydrogenase E	ELI3-2	AT4G37990	1.68	0.75	2.06	0.08023	0.168467	-5.37
245904_at	sucrose-phosphate synthase -like p	ATSPS2F	AT5G11110	1.21	0.28	2.06	0.080287	0.16857	-5.38
247554_at	putative protein leucine zipper-cont	ATEXO70E	AT5G61010	1.55	0.63	2.06	0.080303	0.168579	-5.38
262861_at	hypothetical protein similar to hypot	---	AT1G64820	-1.18	-0.24	-2.06	0.080306	0.168579	-5.38
252918_at	putative protein	---	AT4G38980	1.14	0.19	2.06	0.080329	0.168613	-5.38
253665_at	hypothetical protein	---	AT4G30230	1.34	0.42	2.06	0.080361	0.168657	-5.38
252348_at	B12D-like protein B12D protein - H	---	AT3G48140	1.17	0.22	2.06	0.080365	0.168657	-5.38
253451_at	putative myb-protein transforming p	PC-MYB1	AT4G32730	-1.17	-0.22	-2.06	0.080382	0.168676	-5.38
261470_at	ethylene-responsive element bindir	ERF11	AT1G28370	2.17	1.12	2.06	0.08047	0.168839	-5.38
AFFX-r2-At-	Arabidopsis thaliana /REF=U84969	---	---	1.13	0.18	2.06	0.080479	0.168839	-5.38
248457_at	putative protein contains similarity t	---	AT5G51420	-1.25	-0.32	-2.06	0.080482	0.168839	-5.38
262293_at	heat-shock protein 90, putative ide	---	AT1G27640	-1.28	-0.35	-2.06	0.080509	0.16888	-5.38
267398_at	putative glucanase	AtGH9B11	AT2G44560	-1.20	-0.27	-2.06	0.08053	0.168896	-5.38
265803_at	hypothetical protein predicted by ge	---	AT2G18115	-1.23	-0.30	-2.06	0.080537	0.168896	-5.38
261627_at	arsA homolog (hASNA-I), putative	---	AT1G01910	-1.15	-0.21	-2.06	0.080539	0.168896	-5.38
254921_at	putative protein hypothetical protei	---	AT4G11300	1.29	0.37	2.05	0.080562	0.16893	-5.38
260344_at	polyneuridine aldehyde esterase, p	MES15	AT1G69240	-1.19	-0.25	-2.05	0.080571	0.168933	-5.38
264740_at	unknown protein	---	AT1G62070	-1.17	-0.23	-2.05	0.080581	0.168938	-5.38
248966_at	unknown protein ;supported by full-	---	AT5G45360	1.19	0.25	2.05	0.080607	0.168977	-5.38
255370_at	putative protein	---	---	-1.15	-0.20	-2.05	0.080624	0.168996	-5.38
248379_at	RAR1 (gb|AAF18433.1) non-conse	PBS2	AT5G51700	1.19	0.25	2.05	0.080654	0.168996	-5.38

263398_at	putative RAS superfamily GTP-bind	AtRABA5d	AT2G31680	-1.19	-0.25	-2.05	0.080657	0.168996	-5.38
249972_at	tRNA isopentenyltransferase -like p	IPT5	AT5G19040	-1.18	-0.23	-2.05	0.080661	0.168996	-5.38
262954_at	rubredoxin, putative similar to rubre	---	AT1G54500	-1.23	-0.30	-2.05	0.080663	0.168996	-5.38
265321_at	putative Tub family protein	AtTLP2	AT2G18280	-1.15	-0.20	-2.05	0.080666	0.168996	-5.38
265648_at	putative beta-1,3-glucanase ;supp	---	AT2G27500	1.45	0.54	2.05	0.080673	0.168996	-5.38
264177_at	hypothetical protein contains Pfam	---	AT1G02150	1.17	0.23	2.05	0.080675	0.168996	-5.38
262502_at	unknown protein similar to hypothet	PTAC6	AT1G21600	1.18	0.23	2.05	0.08075	0.169126	-5.38
259417_at	unknown protein contains similarity	HFR1	AT1G02340	-1.32	-0.40	-2.05	0.080752	0.169126	-5.38
264262_at	Expressed protein ; supported by fu	---	AT1G09200	-2.06	-1.04	-2.05	0.080788	0.169187	-5.38
261656_at	unknown protein similar to kinesin-l	ARK2	AT1G01950	-1.54	-0.62	-2.05	0.080822	0.169243	-5.38
253349_at	putative protein	---	---	1.15	0.20	2.05	0.080853	0.169283	-5.38
258144_at	hypothetical protein	---	AT3G18180	-1.15	-0.20	-2.05	0.080857	0.169283	-5.38
264151_at	hypothetical protein predicted by g	---	AT1G02070	-1.19	-0.25	-2.05	0.080867	0.169289	-5.38
267617_at	heme oxygenase 1 (HO1) identical	TED4	AT2G26670	1.30	0.38	2.05	0.080891	0.169315	-5.38
252894_at	hypothetical protein	---	AT4G39440	-1.21	-0.27	-2.05	0.080894	0.169315	-5.38
263179_at	unknown protein similar to unknown	---	AT1G05710	1.36	0.45	2.05	0.080902	0.169316	-5.38
261074_at	hypothetical protein predicted by ge	---	AT1G07290	-1.17	-0.23	-2.05	0.080926	0.16933	-5.38
248758_at	RNA-binding protein-like ;supporte	---	AT5G47620	1.21	0.28	2.05	0.080927	0.16933	-5.38
258111_at	putative cytochrome P450 similar to	CYP72A9	AT3G14630	-1.23	-0.30	-2.05	0.080931	0.16933	-5.38
261519_at	unknown protein ; supported by cDI	---	AT1G71810	-1.27	-0.34	-2.05	0.081003	0.169466	-5.38
260931_at	hypothetical protein predicted by ge	---	AT1G02550	-1.22	-0.29	-2.05	0.081028	0.169503	-5.38
248822_at	peroxidase ;supported by full-lengt	---	AT5G47000	-1.16	-0.21	-2.05	0.08111	0.169659	-5.39
250205_at	unknown protein	---	AT5G14020	-1.17	-0.22	-2.05	0.081236	0.169906	-5.39
246312_at	G-protein, putative contains Pfam p	XLG3	AT1G31930	1.46	0.55	2.05	0.081244	0.169906	-5.39
249801_at	calcium-dependent protein kinase (	CDPK9	AT5G23580	1.18	0.24	2.05	0.081277	0.16996	-5.39
254908_at	putative protein other hypothetical p	---	---	-1.20	-0.26	-2.05	0.081304	0.169976	-5.39
264642_at	putative CCAAT-binding transcriptio	NF-YB4	AT1G09030	-1.18	-0.24	-2.05	0.081305	0.169976	-5.39
245541_at	hypothetical protein	---	AT4G15240	1.28	0.35	2.05	0.081313	0.169976	-5.39
261476_at	hypothetical protein contains similar	---	AT1G14480	1.44	0.52	2.05	0.081314	0.169976	-5.39
249930_at	synaptobrevin-like protein	ATVAMP71	AT5G22360	-1.17	-0.22	-2.05	0.081325	0.169982	-5.39
255949_at	hypothetical protein predicted by ge	---	AT1G22080	-1.13	-0.18	-2.05	0.081353	0.170027	-5.39
248121_at	putative protein contains similarity t	GAUT12	AT5G54690	-1.28	-0.35	-2.05	0.08138	0.170065	-5.39
256100_at	unknown protein	---	AT1G13750	1.66	0.73	2.05	0.081386	0.170065	-5.39
253284_at	putative protein hydroxyproline-rich	---	AT4G34150	-1.42	-0.50	-2.05	0.081459	0.170201	-5.39
266522_at	putative ubiquitin-conjugating enzyn	UBC23	AT2G16920	1.20	0.26	2.05	0.081529	0.170323	-5.39

252229_at	putative protein lea32, Arabidopsis	---	AT3G49890	1.23	0.29	2.05	0.081532	0.170323	-5.39
258886_at	putative importin alpha similar to GI	IMPA-7	AT3G05720	-1.18	-0.24	-2.05	0.081597	0.170443	-5.39
250214_at	endoxyloglucan transferase (dbj|BA	EXGT-A4	AT5G13870	1.21	0.28	2.05	0.081653	0.170534	-5.39
261127_at	unknown protein ; supported by cDI	---	AT1G04790	1.24	0.31	2.05	0.08166	0.170534	-5.39
246917_at	serine-rich protein ;supported by fu	---	AT5G25280	1.37	0.46	2.05	0.081663	0.170534	-5.39
252538_at	putative transporter protein peptide	---	AT3G45720	-1.15	-0.20	-2.05	0.081689	0.170572	-5.39
252287_at	30S ribosomal protein S9 -like simil	---	AT3G49080	1.29	0.37	2.04	0.081788	0.170763	-5.39
259460_at	unknown protein ; supported by cDI	---	AT1G44000	-1.36	-0.45	-2.04	0.081814	0.170801	-5.39
266964_at	putative ABC transporter related to	PGP6	AT2G39480	-1.13	-0.18	-2.04	0.081827	0.170812	-5.39
257718_at	organ separation protein, putative s	anac058	AT3G18400	-1.19	-0.25	-2.04	0.081845	0.170834	-5.39
251093_at	putative protein several hypothetica	---	AT5G01360	-1.31	-0.39	-2.04	0.081908	0.170951	-5.39
257968_at	hypothetical protein predicted by ge	---	AT3G27550	1.28	0.36	2.04	0.081942	0.171006	-5.40
249943_at	putative protein similar to unknown	---	AT5G22280	1.39	0.48	2.04	0.081979	0.171057	-5.40
246118_at	beta-1,3-glucanase bg5	BG5	AT5G20340	-1.20	-0.26	-2.04	0.081981	0.171057	-5.40
258615_at	putative aspartyl protease contains	---	AT3G02740	1.23	0.30	2.04	0.082052	0.17119	-5.40
247823_at	putative protein predicted proteins	---	AT5G58450	1.14	0.19	2.04	0.082124	0.171302	-5.40
252273_at	putative WD-40 repeat - protein be	---	AT3G49660	1.19	0.25	2.04	0.08213	0.171302	-5.40
266506_at	unknown protein	---	AT2G47850	1.16	0.22	2.04	0.082145	0.171302	-5.40
266944_at	unknown protein predicted by gene	---	AT2G18870	-1.19	-0.25	-2.04	0.082147	0.171302	-5.40
257681_at	hypothetical protein predicted by ge	---	AT3G13370	-1.14	-0.19	-2.04	0.082151	0.171302	-5.40
248655_at	60S ribosomal protein L13a ;suppo	---	AT5G48760	1.26	0.33	2.04	0.082151	0.171302	-5.40
257474_at	DNA-3-methyladenine glycosidase	---	AT1G80850	-1.20	-0.27	-2.04	0.082246	0.171483	-5.40
249258_at	putative protein similar to unknown	---	AT5G41650	1.15	0.20	2.04	0.082276	0.17153	-5.40
253409_at	putative protein	---	AT4G32960	1.15	0.20	2.04	0.082299	0.171562	-5.40
258424_at	hypothetical protein predicted by ge	---	AT3G16750	1.20	0.26	2.04	0.082319	0.171589	-5.40
256013_at	unknown protein	DA1	AT1G19270	1.23	0.30	2.04	0.082363	0.171666	-5.40
260582_at	hypothetical protein predicted by ge	---	AT2G47200	1.20	0.26	2.04	0.082381	0.171679	-5.40
254605_at	protein kinase - like protein protein	---	AT4G18950	-1.48	-0.57	-2.04	0.082385	0.171679	-5.40
262828_at	major latex protein type1 identical to	---	AT1G14950	-1.31	-0.39	-2.04	0.082396	0.171687	-5.40
255990_at	glycyl tRNA synthetase, putative si	---	AT1G29870	-1.18	-0.24	-2.04	0.082507	0.171902	-5.40
246599_at	putative protein ATP-dependent RN	---	AT5G14900	-1.14	-0.19	-2.04	0.082522	0.171918	-5.40
264498_at	Expressed protein ; supported by fu	DIS2	AT1G30825	-1.20	-0.27	-2.04	0.082536	0.171927	-5.40
246744_at	putative protein predicted protein, A	---	AT5G27760	1.70	0.76	2.04	0.082542	0.171927	-5.40
264070_at	putative cyclin-dependent kinase re	CKS1	AT2G27960	1.27	0.35	2.04	0.082591	0.172015	-5.40
247527_at	leucine-rich receptor-like protein ki	---	AT5G61480	-1.28	-0.36	-2.04	0.082634	0.172089	-5.40

251812_at	hypothetical protein ;supported by	---	AT3G54970	-1.25	-0.32	-2.04	0.082645	0.172094	-5.40
251456_at	beta-glucosidase-like protein severa	BGLU27	AT3G60120	4.02	2.01	2.04	0.082727	0.17225	-5.40
255152_at	Rab-like protein Rab11 protein - Ar	---	AT4G08190	-1.13	-0.18	-2.04	0.082772	0.172329	-5.41
254365_at	putative protein hypothetical protei	---	AT4G22030	-1.17	-0.23	-2.04	0.082795	0.17236	-5.41
263361_at	putative senescence-associated pro	TET13	AT2G03840	-1.18	-0.24	-2.04	0.082813	0.172381	-5.41
246609_at	putative receptor-like protein kinas	---	AT5G35390	-1.15	-0.20	-2.04	0.082844	0.17243	-5.41
265287_at	putative photosystem I reaction cen	PSAE-2	AT2G20260	-1.14	-0.18	-2.04	0.08287	0.172469	-5.41
261353_at	hypothetical protein contains simila	---	AT1G79600	-1.28	-0.35	-2.04	0.08289	0.172495	-5.41
256161_at	unknown protein ; supported by cD	---	AT1G30090	1.18	0.24	2.03	0.083012	0.172733	-5.41
245101_at	putative cytochrome P450 ; suppor	CYP98A3	AT2G40890	1.27	0.35	2.03	0.083024	0.172744	-5.41
256705_at	cytochrome P450, putative similar	CYP702A8	AT3G30290	-1.21	-0.28	-2.03	0.083045	0.172771	-5.41
246066_at	putative protein predicted proteins,	SMG7	AT5G19400	1.19	0.25	2.03	0.083054	0.172773	-5.41
253132_at	NAM / CUC2 -like protein nap gene	NTL9	AT4G35580	1.22	0.28	2.03	0.083157	0.172971	-5.41
265725_at	putative alanine acetyl transferase	---	AT2G32030	3.17	1.67	2.03	0.083171	0.172986	-5.41
245087_at	hypothetical protein predicted by ge	DAR2	AT2G39830	1.28	0.35	2.03	0.083187	0.173002	-5.41
254892_at	putative protein other hypothetical p	---	AT4G11800	1.21	0.27	2.03	0.083227	0.173055	-5.41
245471_at	probable ribosomal protein	---	AT4G16030	-1.15	-0.20	-2.03	0.083227	0.173055	-5.41
263002_at	hypothetical protein predicted by ge	---	AT1G54200	-1.46	-0.55	-2.03	0.083258	0.173104	-5.41
251337_at	proteasome component C5 ;suppo	PBF1	AT3G60820	1.14	0.19	2.03	0.083345	0.173269	-5.41
263314_at	putative membrane transporter	---	AT2G05760	-1.14	-0.19	-2.03	0.083359	0.17328	-5.41
267280_at	diacylglycerol O-acyltransferase ; su	TAG1	AT2G19450	-1.25	-0.33	-2.03	0.083384	0.17328	-5.41
264851_at	putative calmodulin-domain protein	CPK6	AT2G17290	-1.35	-0.44	-2.03	0.083384	0.17328	-5.41
255871_at	putative small nuclear ribonucleopro	U2B"	AT2G30260	1.19	0.25	2.03	0.083385	0.17328	-5.41
261126_at	TATA binding protein-associated fa	TAF6	AT1G04950	1.16	0.21	2.03	0.083389	0.17328	-5.41
261167_at	disulfide isomerase-related protein	ATPDIL2-2	AT1G04980	1.70	0.76	2.03	0.083441	0.173373	-5.41
246213_at	serine C-palmitoyltransferase like p	ATLCB1	AT4G36480	-1.22	-0.29	-2.03	0.083492	0.173463	-5.41
249942_at	Nitrilase 4 (sp|P46011) ; supported	GAPB	AT1G42970	2.96	1.56	2.03	0.083517	0.173489	-5.41
267072_at	unknown protein	---	AT2G41170	-1.22	-0.29	-2.03	0.083523	0.173489	-5.41
264106_at	unknown protein	SERK4	AT2G13790	-1.16	-0.21	-2.03	0.083527	0.173489	-5.41
246209_at	BEL1-like homeobox 2 protein (BLH	BLH2	AT4G36870	-1.31	-0.39	-2.03	0.083568	0.173557	-5.41
256447_at	nitrate transporter NTL1, putative s	---	AT1G33440	-1.22	-0.29	-2.03	0.083613	0.173635	-5.42
263728_at	gamma-adaptin, putative similar to	---	AT1G60070	-1.18	-0.24	-2.03	0.083639	0.173674	-5.42
267253_at	putative serine carboxypeptidase I	---	AT2G22960	-1.15	-0.20	-2.03	0.083703	0.173786	-5.42
262179_at	hypothetical protein predicted by ge	AGL66	AT1G77980	-1.21	-0.28	-2.03	0.083708	0.173786	-5.42
266203_at	putative phloem-specific lectin	AtPP2-B1	AT2G02230	1.50	0.59	2.03	0.083731	0.173812	-5.42

251940_at	putative protein lysine decarboxyla	---	AT3G53450	-1.17	-0.23	-2.03	0.083736	0.173812	-5.42
250089_at	homeobox protein	HDG9	AT5G17320	-1.21	-0.28	-2.03	0.083807	0.173944	-5.42
267301_at	ubiquitin activating enzyme 1 (UBA	ATUBA1	AT2G30110	1.18	0.24	2.03	0.083844	0.174004	-5.42
249622_at	putative protein	---	AT5G37550	-1.26	-0.33	-2.03	0.083999	0.174308	-5.42
259781_at	reverse transcriptase, putative simi	---	---	-1.20	-0.26	-2.03	0.084006	0.174308	-5.42
260348_at	PPR-repeat protein, putative conta	---	AT1G69350	1.26	0.33	2.03	0.084045	0.174375	-5.42
250127_at	putative protein similar to unknown	---	AT5G16380	-1.22	-0.29	-2.03	0.084066	0.174402	-5.42
252176_at	protein kinase ATN1 -like protein p	---	AT3G50720	-1.21	-0.27	-2.03	0.084105	0.174466	-5.42
246149_at	peroxidase ATP N ;supported by fu	---	AT5G19890	2.37	1.25	2.03	0.084119	0.17448	-5.42
257698_at	regulatory protein of P-starvation a	---	AT3G12730	1.18	0.24	2.03	0.084128	0.174482	-5.42
252611_at	oxidosqualene cyclase - like protei	LAS1	AT3G45130	-1.50	-0.58	-2.02	0.084166	0.174534	-5.42
266566_at	unknown protein	---	AT2G24040	1.20	0.26	2.02	0.084172	0.174534	-5.42
245766_at	En/Spm-like transposon protein, pu	---	---	-1.17	-0.23	-2.02	0.084176	0.174534	-5.42
256683_at	unknown protein ; supported by cDI	---	AT3G52220	-1.14	-0.19	-2.02	0.084195	0.174559	-5.42
253365_at	putative protein various predicted p	---	AT4G33170	1.17	0.23	2.02	0.084249	0.174653	-5.42
246654_s_a	putative protein predicted protein fr	EGY1	AT5G35210 /	1.13	0.18	2.02	0.084279	0.174701	-5.42
248417_at	unknown protein	---	AT5G51650	-1.14	-0.18	-2.02	0.084288	0.174704	-5.42
249806_at	putative protein contains similarity t	---	AT5G23850	-1.41	-0.49	-2.02	0.084321	0.174756	-5.42
258511_at	unknown protein ;supported by full-	---	AT3G06590	1.21	0.28	2.02	0.084329	0.174756	-5.42
258671_at	putative vacuolar ATP synthase sub	VHA-E2	AT3G08560	-1.23	-0.30	-2.02	0.084369	0.174823	-5.42
245659_at	hypothetical protein predicted by ge	---	AT1G28260	1.36	0.44	2.02	0.084376	0.174823	-5.42
250744_at	putative protein strong similarity to u	---	AT5G05840	-1.22	-0.28	-2.02	0.084427	0.174913	-5.42
250080_at	translocon Tic40-like protein ; supp	TIC40	AT5G16620	1.14	0.19	2.02	0.084456	0.174956	-5.43
250854_at	aspartyl aminopeptidase -like prote	---	AT5G04710	1.22	0.29	2.02	0.084585	0.175187	-5.43
245607_at	kinesin like protein	---	AT4G14330	-1.18	-0.24	-2.02	0.084595	0.175187	-5.43
256011_at	respiratory burst oxidase protein, p	---	AT1G19230	-1.18	-0.24	-2.02	0.084596	0.175187	-5.43
263337_at	hypothetical protein predicted by ge	---	---	-1.13	-0.18	-2.02	0.084598	0.175187	-5.43
262410_at	MAGE protein, putative identical to	---	AT1G34770	1.31	0.39	2.02	0.084622	0.175212	-5.43
263610_at	putative beta-1,3-glucanase	---	AT2G16230	-1.21	-0.27	-2.02	0.084627	0.175212	-5.43
264251_at	hypothetical protein identical to GB:	---	AT1G09190	1.25	0.32	2.02	0.084633	0.175212	-5.43
263413_at	unknown protein ;supported by full-	BPC4	AT2G21240	1.20	0.27	2.02	0.084641	0.175212	-5.43
245853_at	putative protein predicted protein A	---	AT5G13500	-1.24	-0.31	-2.02	0.084674	0.175261	-5.43
257210_at	hypothetical protein	TTL2	AT3G14950	-1.14	-0.19	-2.02	0.084682	0.175261	-5.43
245765_at	hypothetical protein similar to putati	---	AT1G33600	1.68	0.75	2.02	0.084688	0.175261	-5.43
244954_s_a	hypothetical protein	---	AT2G07732	1.40	0.49	2.02	0.08477	0.175415	-5.43

264963_at	hypothetical protein predicted by ge	ABC4	AT1G60600	-1.27	-0.35	-2.02	0.0848	0.175452	-5.43
251672_at	MADS-box transcription factor-like	AGL16	AT3G57230	-1.16	-0.21	-2.02	0.084803	0.175452	-5.43
247463_at	embryo-specific protein - like embr	---	AT5G62210	-1.19	-0.25	-2.02	0.084823	0.175477	-5.43
251957_at	putative protein predicted proteins,	---	AT3G53690	1.19	0.25	2.02	0.084835	0.175486	-5.43
245643_at	myb-related transcription factor (cp	MYB116	AT1G25340	-1.17	-0.23	-2.02	0.084849	0.175488	-5.43
263982_at	hypothetical protein predicted by ge	---	AT2G42860	-1.23	-0.29	-2.02	0.084859	0.175488	-5.43
265962_at	nodulin-like protein ;supported by f	---	AT2G37460	1.55	0.63	2.02	0.084865	0.175488	-5.43
257901_at	hypothetical protein predicted by ge	---	---	-1.17	-0.23	-2.02	0.084867	0.175488	-5.43
266212_at	putative retroelement pol polyprotei	---	---	-1.23	-0.29	-2.02	0.084893	0.175526	-5.43
251367_at	SKP1 interacting partner 4 (SKIP4)	SKIP4	AT3G61350	-1.21	-0.28	-2.02	0.084927	0.175576	-5.43
245964_at	putative protein C-terminus of gluta	---	AT5G19720	-1.21	-0.27	-2.02	0.084932	0.175576	-5.43
254007_s_a	putative protein RNA-directed DNA	---	AT1G60720	-1.16	-0.21	-2.02	0.085038	0.175779	-5.43
258431_at	hypothetical protein predicted by ge	---	AT3G16580	-1.15	-0.21	-2.02	0.085057	0.175801	-5.43
250972_at	putative protein various Myb relate	LCL1	AT5G02840	1.30	0.38	2.02	0.085072	0.175817	-5.43
257735_at	pectate lyase, putative similar to pe	---	AT3G27400	-1.33	-0.41	-2.02	0.085084	0.175826	-5.43
258159_at	receptor kinase, putative similar to	RLK902	AT3G17840	-1.38	-0.46	-2.02	0.085115	0.175866	-5.43
265529_at	hypothetical protein predicted by ge	---	---	-1.15	-0.20	-2.02	0.085119	0.175866	-5.43
254095_at	oleosin, 18.5K	OLEO1	AT4G25140	-1.18	-0.23	-2.02	0.085132	0.175877	-5.43
261097_at	protein kinase, putative contains pr	---	AT1G62950	-1.27	-0.35	-2.02	0.085168	0.175935	-5.43
267225_at	hypothetical protein predicted by ge	---	AT2G44000	-1.18	-0.24	-2.02	0.085188	0.17596	-5.43
260317_at	E2, ubiquitin-conjugating enzyme 5	UBC5	AT1G63800	1.31	0.39	2.02	0.085245	0.176056	-5.43
245095_at	putative protein kinase contains a p	---	AT2G40860	-1.27	-0.34	-2.02	0.085249	0.176056	-5.43
264457_at	puative glucosyl transferase similar	---	AT1G10400	-1.18	-0.24	-2.02	0.085291	0.176126	-5.43
252016_at	putative protein predicted protein, A	---	AT3G52950	1.17	0.23	2.02	0.085323	0.176177	-5.44
260287_at	unknown protein contains two Kelch	---	AT1G80440	1.31	0.39	2.02	0.085349	0.176213	-5.44
254037_at	hypothetical protein hypothetical pr	ATGDU2	AT4G25760	-1.16	-0.22	-2.01	0.085433	0.176371	-5.44
266098_at	hypothetical protein predicted by ge	---	AT2G37870	-1.26	-0.33	-2.01	0.085446	0.176383	-5.44
257676_at	unknown protein ;supported by full-	TGD2	AT3G20320	1.18	0.24	2.01	0.085497	0.176472	-5.44
265619_at	hypothetical protein	---	AT2G27320	-1.23	-0.30	-2.01	0.085522	0.176507	-5.44
245364_at	hypothetical protein ;supported by f	---	AT4G15790	-1.22	-0.29	-2.01	0.085567	0.176584	-5.44
266075_s_a	unknown protein	---	AT2G40710	1.22	0.29	2.01	0.085618	0.176673	-5.44
251394_at	endosperm specific protein - like en	FLA10	AT3G60900	-1.22	-0.29	-2.01	0.08565	0.176723	-5.44
267119_at	putative cellulose synthase	ATCSLB01	AT2G32610	-1.14	-0.19	-2.01	0.085671	0.176744	-5.44
253300_at	carbonate dehydratase - like protei	BCA5	AT4G33580	-1.29	-0.37	-2.01	0.085682	0.176744	-5.44
266130_at	putative transcription activator	---	AT2G44980	1.29	0.37	2.01	0.085683	0.176744	-5.44

263738_at	hypothetical protein predicted by ge	---	AT1G60060	-1.57	-0.65	-2.01	0.085719	0.176803	-5.44
258644_at	putative RNA-binding protein conta	---	AT3G07810	-1.14	-0.19	-2.01	0.085751	0.176839	-5.44
253289_at	putative protein F10M10_100, Arab	---	AT4G34320	-1.17	-0.23	-2.01	0.085753	0.176839	-5.44
260151_at	unknown protein ;supported by full-	---	AT1G52910	-1.74	-0.80	-2.01	0.08578	0.176869	-5.44
254395_at	subtilisin-like protease subtilisin-like	---	AT4G21640	-1.14	-0.19	-2.01	0.085782	0.176869	-5.44
266129_at	hypothetical protein predicted by ge	CCD7	AT2G44990	-1.67	-0.74	-2.01	0.085812	0.176902	-5.44
248947_at	putative protein similar to unknown	---	AT5G45540	-1.24	-0.30	-2.01	0.085814	0.176902	-5.44
249615_x_a	contains similarity to unknown prote	AGL105	AT5G37420	-1.18	-0.24	-2.01	0.085828	0.176914	-5.44
248633_at	arginine methyltransferase-like prot	PRMT4A	AT5G49020	1.19	0.25	2.01	0.085851	0.176947	-5.44
259532_at	hypothetical protein	---	AT1G12470	-1.14	-0.19	-2.01	0.085874	0.176977	-5.44
267153_at	glutathione S-transferase identical t	ATGSTF9	AT2G30860	1.25	0.33	2.01	0.085945	0.177108	-5.44
252977_at	Phospholipase like protein Arabido	---	AT4G38560	1.55	0.64	2.01	0.085955	0.177108	-5.44
254227_at	putative protein chS-Rex-b - Gallus	BTI1	AT4G23630	-1.26	-0.34	-2.01	0.085969	0.177108	-5.44
253113_at	putative protein physical impedanc	---	AT4G35985	-1.32	-0.40	-2.01	0.085973	0.177108	-5.44
260777_at	mitochondrial carrier protein, putati	---	AT1G14560	-1.18	-0.24	-2.01	0.085976	0.177108	-5.44
261881_at	nodulin-like protein similar to noduli	NIP6;1	AT1G80760	-1.31	-0.40	-2.01	0.086005	0.177141	-5.44
260246_at	hypothetical protein similar to GB:A	---	AT1G74220	-1.16	-0.22	-2.01	0.086008	0.177141	-5.44
246156_at	putative protein predicted proteins f	---	AT5G20090	1.20	0.26	2.01	0.086016	0.177141	-5.44
248400_at	putative protein contains similarity t	---	AT5G52020	-1.23	-0.30	-2.01	0.086025	0.177141	-5.44
267623_at	unknown protein	---	AT2G39650	1.95	0.96	2.01	0.086031	0.177141	-5.44
254915_s_a	cysteine proteinase contains simila	---	AT4G11310	-2.13	-1.09	-2.01	0.086054	0.177171	-5.44
244920_s_a	NADH dehydrogenase subunit 3 Pr	---	AT2G07751	1.20	0.26	2.01	0.086084	0.177218	-5.44
262526_at	putative prenyl transferase (prephy	SPS2	AT1G17050	1.54	0.63	2.01	0.086141	0.177319	-5.44
258083_at	Expressed protein ; supported by fu	---	AT3G25855	-1.20	-0.26	-2.01	0.086162	0.177341	-5.44
248703_at	dermal glycoprotein precursor, extr	---	AT5G48430	3.32	1.73	2.01	0.086167	0.177341	-5.44
256794_at	ribosomal protein L27, putative sim	---	AT3G22230	1.44	0.53	2.01	0.086185	0.177361	-5.45
246895_at	putative protein predicted protein, A	CID6	AT5G25540	-1.16	-0.22	-2.01	0.086196	0.177368	-5.45
265659_at	putative disease resistance protein	AtRLP20	AT2G25440	1.45	0.54	2.01	0.086216	0.177394	-5.45
251204_at	putative protein HMW prekininogen	AT3G6309	---	-1.19	-0.25	-2.01	0.086233	0.177413	-5.45
247086_at	GATA-binding transcription factor-li	---	AT5G66320	1.92	0.94	2.01	0.086253	0.177437	-5.45
252853_at	hypothetical protein ; supported by	---	AT4G39710	-1.45	-0.53	-2.01	0.086261	0.177437	-5.45
256626_at	hypothetical protein, 5' partial	---	AT3G20015	-1.49	-0.57	-2.01	0.086275	0.177451	-5.45
251408_at	palmitoyl-protein thioesterase prec	---	AT3G60340	1.22	0.29	2.01	0.086287	0.177456	-5.45
255602_at	Expressed protein ; supported by fu	---	AT4G01026	-1.20	-0.27	-2.01	0.086293	0.177456	-5.45
258353_s_a	unknown protein similar to GB:AAD	YSL5	AT3G17650	1.24	0.31	2.01	0.086332	0.177514	-5.45

260123_at	AIG1-like protein similar to AIG1 pr	---	AT1G33890	-1.17	-0.22	-2.01	0.086337	0.177514	-5.45
253853_at	putative diacylglycerol kinase (frag	---	AT4G28130	-1.16	-0.22	-2.01	0.086371	0.177568	-5.45
264863_at	putative peroxidase similar to perox	---	AT1G24110	-1.20	-0.26	-2.01	0.086389	0.177588	-5.45
249135_at	putative protein similar to unknown	---	AT5G43160	-1.37	-0.45	-2.01	0.086473	0.177745	-5.45
255262_at	putative protein equilibrative nucle	---	AT4G05140	-1.16	-0.22	-2.01	0.086509	0.177787	-5.45
264927_at	phosphatidylinositol 3-kinase, puta	VPS34	AT1G60490	1.13	0.18	2.01	0.086525	0.177787	-5.45
267226_at	unknown protein	---	AT2G44010	1.46	0.54	2.01	0.086527	0.177787	-5.45
258073_at	putative auxin-resistance protein, A	---	AT3G25880	-1.16	-0.21	-2.01	0.086537	0.177787	-5.45
259454_at	unknown protein similar to hypothet	---	AT1G44050	-1.19	-0.25	-2.01	0.086538	0.177787	-5.45
260037_at	putative DNA-binding protein (RAV2	RAV2	AT1G68840	-1.87	-0.90	-2.01	0.08655	0.177787	-5.45
265802_at	predicted protein	---	AT2G35733	1.21	0.27	2.01	0.086555	0.177787	-5.45
245817_at	hypothetical protein similar to unkno	---	AT1G26160	1.14	0.19	2.01	0.086555	0.177787	-5.45
246136_at	peptide transporter - like protein pe	---	AT5G28470	-1.19	-0.25	-2.01	0.08657	0.177801	-5.45
267331_at	putative salt-inducible protein	---	AT2G19280	-1.21	-0.28	-2.00	0.086633	0.177915	-5.45
245441_at	decarboxylase like protein	PSD1	AT4G16700	1.23	0.30	2.00	0.086653	0.177939	-5.45
251902_at	uncoupling protein (ucp/PUMP) ; s	PUMP1	AT3G54110	1.46	0.54	2.00	0.086672	0.177962	-5.45
263477_at	putative glucosyltransferase ; supp	---	AT2G31790	-1.27	-0.35	-2.00	0.086704	0.178012	-5.45
260728_at	Pto kinase interactor 1, putative sir	---	AT1G48210	1.38	0.46	2.00	0.086722	0.178034	-5.45
255490_at	putative protein lysosomal traffickir	---	AT4G02660	-1.17	-0.23	-2.00	0.08675	0.178075	-5.45
254043_at	putative protein ; supported by cDN	CIL	AT4G25990	1.64	0.71	2.00	0.086782	0.17811	-5.45
248042_at	putative protein similar to unknown	---	AT5G55960	-1.22	-0.29	-2.00	0.086783	0.17811	-5.45
251286_at	putative protein Bcl-2-interacting pr	---	AT3G61710	1.16	0.21	2.00	0.086806	0.178141	-5.45
252171_at	hypothetical protein ; supported by	---	AT3G50590	-1.17	-0.22	-2.00	0.086814	0.178142	-5.45
246269_at	putative protein	---	AT4G37110	-1.40	-0.49	-2.00	0.086847	0.178193	-5.45
256254_at	hypothetical protein predicted by ge	---	AT3G11290	-1.23	-0.29	-2.00	0.086854	0.178193	-5.45
255175_at	putative glucosyltransferase ;suppo	ATCSLC12	AT4G07960	-1.18	-0.24	-2.00	0.08699	0.178456	-5.45
259959_at	hypothetical protein predicted by ge	CYP59	AT1G53720	1.20	0.27	2.00	0.087135	0.178728	-5.46
246590_at	cyclic nucleotide and calmodulin-re	CNGC18	AT5G14870	-1.14	-0.19	-2.00	0.087139	0.178728	-5.46
259930_at	hypothetical protein predicted by ge	---	AT1G34355	-1.18	-0.24	-2.00	0.087147	0.178728	-5.46
255184_at	putative athila transposon protein	---	---	-1.14	-0.19	-2.00	0.087175	0.17877	-5.46
246528_at	putative mitochondrial carrier prote	---	AT5G15640	-1.32	-0.40	-2.00	0.087215	0.178837	-5.46
263344_at	unknown protein ;supported by full-	---	AT2G04940	1.23	0.30	2.00	0.087266	0.178925	-5.46
255621_at	hypothetical protein	---	AT4G01390	1.99	0.99	2.00	0.087299	0.178965	-5.46
249319_s_a	putative protein similar to unknown	---	AT5G40880	1.20	0.27	2.00	0.087301	0.178965	-5.46
250736_s_a	putative protein contains similarity t	---	AT1G01350	1.25	0.32	2.00	0.087354	0.179038	-5.46

264527_at	putative reticuline oxidase-like prot	---	AT1G30760	-1.43	-0.52	-2.00	0.08736	0.179038	-5.46
251148_at	hypothetical protein	TKL	AT3G63180	1.17	0.22	2.00	0.087361	0.179038	-5.46
249483_at	Expressed protein ; supported by cl	---	AT5G38895	1.21	0.28	2.00	0.087392	0.179087	-5.46
260923_at	glycine-rich RNA-binding protein gr	---	AT1G21570	-1.18	-0.24	-2.00	0.087423	0.179134	-5.46
248520_at	11-beta-hydroxysteroid dehydrogen	AtHSD1	AT5G50600	-1.27	-0.35	-2.00	0.087529	0.179335	-5.46
255318_at	hypothetical protein	---	AT4G04190	1.20	0.26	2.00	0.087573	0.179409	-5.46
247784_at	subtilisin-like serine protease conta	---	AT5G58810	-1.16	-0.22	-2.00	0.087587	0.179417	-5.46
265543_at	unknown protein	---	AT2G28270	-1.13	-0.18	-2.00	0.087593	0.179417	-5.46
245630_at	hypothetical protein similar to putati	---	AT1G25360	1.17	0.23	2.00	0.087642	0.179502	-5.46
255996_s_a	receptor-like serine threonine kinas	---	AT1G29730	-1.21	-0.27	-2.00	0.087668	0.179539	-5.46
261828_at	unknown protein ; supported by cDI	---	AT1G11360	1.16	0.22	2.00	0.08772	0.17963	-5.46
256707_at	hypothetical protein predicted by ge	---	---	-1.15	-0.20	-2.00	0.087795	0.179768	-5.46
253348_at	putative protein SMT4 protein - Sa	---	AT4G33620	1.14	0.18	2.00	0.087816	0.179794	-5.46
250537_at	Expressed protein ; supported by fu	---	AT5G08565	1.30	0.37	2.00	0.087829	0.179804	-5.46
258583_at	putative SRF-type transcription fact	AGL57	AT3G04100	-1.15	-0.20	-2.00	0.087841	0.179814	-5.46
265020_at	putative cytochrome P450 similar t	CYP86C1	AT1G24540	-1.15	-0.20	-1.99	0.0879	0.179896	-5.46
267090_at	unknown protein ; supported by ful	CXIP2	AT2G38270	1.14	0.19	1.99	0.087901	0.179896	-5.46
259249_at	hypothetical protein predicted by ge	---	AT3G07790	-1.26	-0.34	-1.99	0.087914	0.179896	-5.46
264707_at	unknown protein	---	AT1G09730	1.16	0.22	1.99	0.087919	0.179896	-5.46
249277_at	GDSL-motif lipase/hydrolase-like pr	---	AT5G41890	-1.54	-0.63	-1.99	0.087944	0.179896	-5.47
256731_at	hypothetical protein contains Pfam	---	AT3G30340	-1.16	-0.22	-1.99	0.087944	0.179896	-5.47
258821_at	putative wound-induced basic prote	---	AT3G07230	1.22	0.29	1.99	0.087944	0.179896	-5.47
251654_at	putative protein several hypothetica	SDP1-LIKE	AT3G57140	-1.15	-0.20	-1.99	0.087945	0.179896	-5.47
262684_s_a	vacuolar ATP synthase subunit B id	---	AT1G20260	-1.21	-0.27	-1.99	0.08797	0.179931	-5.47
257627_at	cytochrome P450, putative contain	CYP71B25	AT3G26270	1.19	0.25	1.99	0.087989	0.179954	-5.47
250595_at	unknown protein	---	AT5G07770	-1.28	-0.36	-1.99	0.088002	0.179964	-5.47
256087_at	hypothetical protein predicted by ge	---	AT1G20800	-1.16	-0.22	-1.99	0.08801	0.179964	-5.47
254310_at	putative protein predicted protein, A	---	AT4G22430	-1.13	-0.18	-1.99	0.088029	0.179988	-5.47
265098_at	unknown protein	---	AT1G04010	-1.39	-0.48	-1.99	0.088039	0.179993	-5.47
266717_at	Expressed protein ; supported by fu	---	AT2G46735	-1.17	-0.22	-1.99	0.088115	0.180131	-5.47
247536_at	PREG-like - like protein PREG-like	CYCP4;2	AT5G61650	-1.21	-0.28	-1.99	0.088139	0.180163	-5.47
255802_s_a	putative protein RING-H2 finger pro	---	AT4G10150	-1.15	-0.20	-1.99	0.088189	0.180251	-5.47
266666_at	hypothetical protein predicted by ge	---	AT2G29780	-1.17	-0.22	-1.99	0.088198	0.180253	-5.47
246178_s_a	putative protein predicted proteins -	---	---	1.22	0.28	1.99	0.088208	0.180257	-5.47
254967_at	putative protein various predicted p	---	AT4G10410	-1.16	-0.22	-1.99	0.088281	0.18039	-5.47

254345_at	hypothetical protein ;supported by f	---	AT4G22000	1.29	0.37	1.99	0.088298	0.180408	-5.47
259810_at	Na+/H+ antiporter, putative similar	ATNHD2	AT1G49810	-1.15	-0.20	-1.99	0.088314	0.180425	-5.47
264592_at	putative prolyl 4-hydroxylase, alpha	---	AT2G17720	1.51	0.59	1.99	0.088369	0.18052	-5.47
245568_at	hypothetical protein	---	AT4G14650	-1.24	-0.31	-1.99	0.088468	0.180707	-5.47
256171_s_a	hypothetical protein similar to revers	---	---	-1.18	-0.24	-1.99	0.088559	0.180877	-5.47
251782_at	beta-N-acetylhexosaminidase -like	HEXO1	AT3G55260	-1.26	-0.33	-1.99	0.088576	0.180896	-5.47
258402_at	unknown protein very similar to unk	---	AT3G15450	-1.46	-0.55	-1.99	0.08859	0.180908	-5.47
264008_at	unknown protein	---	AT2G21120	-1.24	-0.30	-1.99	0.088643	0.181	-5.47
262323_at	6-phosphogluconate dehydrogenas	---	AT1G64190	1.14	0.19	1.99	0.088673	0.181044	-5.47
262989_at	F28C11.5 similar to INNER NO OU	INO	AT1G23420	-1.20	-0.27	-1.99	0.088698	0.18108	-5.47
261735_at	hypothetical protein similar to hypot	---	AT1G47765	-1.18	-0.24	-1.99	0.088766	0.181203	-5.47
246340_s_a	AtPP -like protein AtPP protein, Bra	---	AT3G44860	-1.41	-0.50	-1.99	0.08879	0.181236	-5.47
246306_at	putative protein hypothetical protei	ATBAG4	AT3G51780	1.13	0.18	1.99	0.088799	0.181238	-5.47
262924_s_a	vacuolar ATP synthase, putative si	AVA-P4	AT1G19910	1.20	0.27	1.99	0.088831	0.181271	-5.47
249381_at	acidic ribosomal protein P2 -like ac	---	AT5G40040	-1.21	-0.28	-1.99	0.088831	0.181271	-5.47
257935_at	unknown protein	---	AT3G25440	1.17	0.22	1.99	0.088844	0.181274	-5.48
263327_at	putative receptor-like protein kinase	---	AT2G15300	-1.14	-0.20	-1.99	0.088849	0.181274	-5.48
263518_at	Expressed protein ; supported by fu	---	AT2G21655	-1.17	-0.22	-1.99	0.088865	0.181292	-5.48
249687_at	putative protein oxidosqualene cyc	ATPEN3	AT5G36150	-1.16	-0.22	-1.99	0.0889	0.181346	-5.48
266256_at	60S acidic ribosomal protein P2 ;su	---	AT2G27710	1.34	0.42	1.99	0.088954	0.181439	-5.48
266893_at	hypothetical protein predicted by ge	RTE1	AT2G26070	-1.19	-0.25	-1.99	0.088969	0.181455	-5.48
260860_at	hypothetical protein predicted by ge	---	AT1G43810	-1.17	-0.23	-1.99	0.089002	0.181505	-5.48
264993_at	glyoxal oxidase (glx1), putative sim	---	AT1G67290	-1.32	-0.40	-1.99	0.089019	0.181523	-5.48
254895_at	hypothetical protein	---	AT4G11870	1.22	0.29	1.99	0.089128	0.18173	-5.48
248294_at	RNA-binding protein-like	---	AT5G53060	1.19	0.25	1.99	0.089136	0.18173	-5.48
256625_at	uteroglobin promoter-binding prote	---	AT3G20010	1.30	0.38	1.99	0.089158	0.181759	-5.48
251167_at	putative protein proteinase inhibitor	---	AT3G63360	-1.15	-0.20	-1.99	0.08918	0.181786	-5.48
252907_at	putative gamma-glutamyltransferas	GGT2	AT4G39650	-1.15	-0.20	-1.98	0.089203	0.181818	-5.48
252513_at	putative protein KIAA0776 protein,	---	AT3G46220	1.17	0.23	1.98	0.089228	0.181838	-5.48
259745_at	unknown protein	SAG18	AT1G71190	1.19	0.24	1.98	0.089229	0.181838	-5.48
247231_at	transcription factor-like protein	AtMYB53	AT5G65230	-1.17	-0.22	-1.98	0.089244	0.181853	-5.48
261448_at	tonoplast intrinsic protein, alpha (al	---	AT1G21140	-1.29	-0.37	-1.98	0.089262	0.181873	-5.48
262073_at	unknown protein ;supported by full-	ZCW32	AT1G59640	1.36	0.45	1.98	0.089286	0.181905	-5.48
261644_s_a	hypothetical protein contains simila	ATCSA-1	AT1G19750	1.19	0.25	1.98	0.089314	0.181947	-5.48
265244_at	putative amine oxidase	ATPAO2	AT2G43018	-1.36	-0.45	-1.98	0.08933	0.181963	-5.48

255860_at	putative protein heparanase, Homo	AtGUS3	AT5G34940	3.12	1.64	1.98	0.089355	0.181992	-5.48
249757_at	Expressed protein ; supported by fu	---	AT5G24316	-1.20	-0.26	-1.98	0.08936	0.181992	-5.48
248563_at	anthocyanidin-3-glucoside rhamnos	---	AT5G49690	1.30	0.37	1.98	0.089385	0.182018	-5.48
247093_at	zinc finger protein SHI-like ; suppo	SHI	AT5G66350	1.25	0.32	1.98	0.089389	0.182018	-5.48
245225_at	mitochondrial protein-like; contains	---	AT3G29800	-1.23	-0.30	-1.98	0.089402	0.182028	-5.48
247653_at	transcriptional coactivator - like pro	---	AT5G59950	1.15	0.20	1.98	0.089423	0.182054	-5.48
252811_at	putative protein several hypothetica	---	---	-1.18	-0.24	-1.98	0.089445	0.182082	-5.48
262667_at	amine oxidase, putative similar to a	---	AT1G62810	1.28	0.36	1.98	0.089482	0.182142	-5.48
250709_at	putative protein similar to unknown	LBD33	AT5G06080	-1.17	-0.23	-1.98	0.089509	0.182181	-5.48
246604_at	putative protein	---	---	-1.17	-0.22	-1.98	0.089572	0.182285	-5.48
253581_at	stress responsive protein homolog	---	AT4G30660	-1.42	-0.50	-1.98	0.089576	0.182285	-5.48
260329_at	putative cyclin similar to cyclin GB:S	CYCA2;4	AT1G80370	-1.20	-0.26	-1.98	0.089601	0.18232	-5.48
249917_at	putative protein strong similarity to u	---	AT5G22460	-2.19	-1.13	-1.98	0.089696	0.182497	-5.48
254180_at	putative protein hin1 protein homol	---	AT4G23930	-1.30	-0.38	-1.98	0.089737	0.18255	-5.48
257878_at	hypothetical protein predicted by ge	---	AT3G17150	-1.17	-0.23	-1.98	0.089738	0.18255	-5.48
267158_at	putative expansin	EXP3	AT2G37640	-2.27	-1.18	-1.98	0.089785	0.182629	-5.49
249456_at	unknown protein ;supported by full-	---	AT5G39410	1.18	0.24	1.98	0.089823	0.18269	-5.49
262442_at	unknown protein identical to hypoth	---	AT1G47420	1.15	0.20	1.98	0.089868	0.182765	-5.49
250894_s_a	putative protein proline-rich protein	---	AT5G03810	-1.19	-0.25	-1.98	0.089884	0.182779	-5.49
259813_at	glutathione S-transferase, putative	ATGSTF14	AT1G49860	-1.18	-0.24	-1.98	0.089899	0.182779	-5.49
258878_at	hypothetical protein predicted by ge	---	AT3G03170	1.15	0.20	1.98	0.089903	0.182779	-5.49
247842_at	SPP30 - like protein SPP30, Solan	---	AT5G58030	-1.14	-0.19	-1.98	0.089907	0.182779	-5.49
255955_at	hypothetical protein predicted by ge	---	AT1G22030	-1.33	-0.41	-1.98	0.089945	0.182833	-5.49
260628_at	hypothetical protein predicted by ge	---	AT1G62320	-1.15	-0.20	-1.98	0.08995	0.182833	-5.49
258117_at	hypothetical protein predicted by ge	---	AT3G14700	1.16	0.21	1.98	0.089966	0.182851	-5.49
249480_s_a	protein kinase - like protein recepto	---	AT5G38990	1.47	0.56	1.98	0.09003	0.182963	-5.49
254155_at	putative protein kinase putative pro	---	AT4G24480	-1.20	-0.26	-1.98	0.090093	0.183076	-5.49
251565_at	putative protein several hypothetica	LBD29	AT3G58190	-1.15	-0.20	-1.98	0.090111	0.183097	-5.49
253772_at	Expressed protein ; supported by fu	A7	AT4G28395	-1.13	-0.18	-1.98	0.090131	0.183119	-5.49
257919_at	myb-related transcription factor, pu	MYB15	AT3G23250	2.35	1.24	1.98	0.090203	0.18325	-5.49
250881_at	putative protein	---	AT5G04080	1.26	0.34	1.98	0.09031	0.18345	-5.49
259293_at	putative DNA binding protein simila	---	AT3G11580	1.32	0.40	1.98	0.090321	0.183456	-5.49
246164_at	putative protein predicted proteins,	---	---	-1.16	-0.22	-1.98	0.090353	0.183487	-5.49
263380_at	hypothetical protein	---	AT2G40200	-1.16	-0.22	-1.98	0.090359	0.183487	-5.49
257165_at	beta-1,3-glucanase, putative simila	---	AT3G24330	-1.26	-0.33	-1.98	0.09036	0.183487	-5.49

249004_at	unknown protein	---	AT5G44570	-1.22	-0.29	-1.98	0.090403	0.183535	-5.49
265703_at	putative ankyrin	---	AT2G03430	1.21	0.28	1.98	0.090407	0.183535	-5.49
259560_at	hypothetical protein	WAK2	AT1G21270	-1.64	-0.72	-1.98	0.090411	0.183535	-5.49
259712_at	putative 20S proteasome beta subu	PBC2	AT1G77440	1.15	0.20	1.98	0.090416	0.183535	-5.49
257622_at	hypothetical protein contains Pfam	---	AT3G20280	1.26	0.33	1.98	0.090428	0.183544	-5.49
246551_at	putative protein predicted proteins,	---	AT5G15070	-1.18	-0.24	-1.98	0.090438	0.183548	-5.49
258165_at	unknown protein ; supported by cD	---	AT3G17900	-1.18	-0.23	-1.98	0.090475	0.183606	-5.49
252218_at	putative protein some putative prote	---	AT3G50150	-1.16	-0.21	-1.97	0.090493	0.183615	-5.49
245056_at	putative glucosyltransferase	UGT76D1	AT2G26480	-1.26	-0.33	-1.97	0.090497	0.183615	-5.49
257029_at	dem-like protein similar to dem GE	---	AT3G19240	1.17	0.23	1.97	0.090504	0.183615	-5.49
262797_at	putative sugar transporter protein r	TMT1	AT1G20840	1.32	0.40	1.97	0.090515	0.183618	-5.49
259133_at	sugar transporter, putative similar t	---	AT3G05400	1.40	0.48	1.97	0.090521	0.183618	-5.49
251152_at	RAN GTPase activating protein 1 p	RANGAP1	AT3G63130	1.28	0.35	1.97	0.09053	0.183619	-5.49
250710_at	MYB family transcription factor-like	MYB33	AT5G06100	1.16	0.22	1.97	0.090544	0.183633	-5.49
245294_at	casein kinase I ; supported by cDN/	CKI1	AT4G14340	-1.17	-0.22	-1.97	0.090674	0.18388	-5.50
245311_at	ribosomal protein ; supported by ful	---	AT4G14320	1.28	0.35	1.97	0.090708	0.183932	-5.50
253702_at	Ca2+-transporting ATPase - like pr	ACA10	AT4G29900	1.24	0.31	1.97	0.090729	0.183952	-5.50
249533_at	putative protein predicted protein, /	---	AT5G38790	-1.18	-0.24	-1.97	0.090734	0.183952	-5.50
245093_at	hypothetical protein predicted by ge	---	AT2G40815	-1.17	-0.23	-1.97	0.090746	0.183959	-5.50
261120_at	homeodomain protein BELL1, puta	BLH3	AT1G75410	1.27	0.35	1.97	0.090782	0.184006	-5.50
244989_s_a	hypothetical protein	---	---	-1.24	-0.31	-1.97	0.090785	0.184006	-5.50
245286_at	cytosolic O-acetylserine(thiol)lyase	OASA1	AT4G14880	-1.17	-0.23	-1.97	0.090844	0.18411	-5.50
257939_at	monosaccharide transport protein,	STP4	AT3G19930	-1.32	-0.40	-1.97	0.090889	0.184176	-5.50
257670_at	unknown protein	---	AT3G20340	-1.43	-0.52	-1.97	0.090893	0.184176	-5.50
259220_at	expressed protein predicted by ger	---	AT3G03550	-1.21	-0.27	-1.97	0.090934	0.184242	-5.50
257513_s_a	hypothetical protein	---	AT1G12340	1.18	0.24	1.97	0.09102	0.1844	-5.50
252455_at	putative protein various predicted p	---	AT3G47140	-1.13	-0.18	-1.97	0.091066	0.18447	-5.50
266795_at	unknown protein	---	AT2G03070	1.20	0.26	1.97	0.09107	0.18447	-5.50
252390_at	Expressed protein ; supported by cl	---	AT3G47836	1.19	0.26	1.97	0.091104	0.184522	-5.50
247812_at	peroxidase ;supported by full-length	---	AT5G58390	-1.61	-0.69	-1.97	0.091217	0.184733	-5.50
250815_s_a	putative protein similar to unknown	---	AT5G05040	-1.23	-0.29	-1.97	0.091249	0.184782	-5.50
265418_at	AP2 domain transcription factor	---	AT2G20880	-1.78	-0.83	-1.97	0.091289	0.184847	-5.50
266630_at	putative serpin	---	AT2G35580	-1.15	-0.20	-1.97	0.091303	0.184858	-5.50
246992_at	N-acetyltransferase hookless1-like	---	AT5G67430	-1.17	-0.22	-1.97	0.091384	0.185003	-5.50
265105_s_a	unknown protein contains similarity	---	AT1G62580	-1.13	-0.18	-1.97	0.091394	0.185003	-5.50

263623_at	hypothetical protein predicted by ge	---	AT2G04860	-1.14	-0.18	-1.97	0.091399	0.185003	-5.50
267572_at	3-hydroxyisobutyryl-coenzyme A hy	---	AT2G30660	-1.17	-0.23	-1.97	0.091485	0.185162	-5.50
256352_at	proline-rich protein, putative similar	ATPRP1	AT1G54970	-1.22	-0.29	-1.97	0.091507	0.18519	-5.50
261221_at	hypothetical protein predicted by ge	---	AT1G19960	2.98	1.58	1.97	0.091562	0.185284	-5.50
256647_at	unknown protein contains similarity	---	AT3G13610	3.67	1.87	1.97	0.091576	0.185297	-5.50
255687_at	hypothetical protein	KEA2	AT4G00630	1.37	0.45	1.97	0.091694	0.185504	-5.51
253637_at	putative protein ; supported by full-	---	AT4G30390	1.29	0.36	1.97	0.091696	0.185504	-5.51
248849_at	multidrug resistance p-glycoprotein	PGP7	AT5G46540	-1.15	-0.20	-1.97	0.09171	0.185504	-5.51
245533_at	putative phosphocholine cytidylyltra	CCT2	AT4G15130	-1.49	-0.57	-1.97	0.091711	0.185504	-5.51
258950_at	unknown protein contains Pfam pro	MBD9	AT3G01460	-1.13	-0.17	-1.97	0.091724	0.185513	-5.51
256951_at	hypothetical protein predicted by ge	---	AT3G19085	-1.13	-0.18	-1.97	0.091741	0.185513	-5.51
267024_s_a	putative aquaporin (plasma membra	NIP2;1	AT2G29870 ,	-1.21	-0.27	-1.97	0.091743	0.185513	-5.51
245186_at	F12A21.15 hypothetical protein	ARR11	AT1G67710	-1.44	-0.53	-1.97	0.091754	0.185513	-5.51
254844_at	hypothetical protein supported by fu	---	AT4G11790	1.29	0.36	1.97	0.091756	0.185513	-5.51
259336_at	hypothetical protein C-term portion	SDG20	AT3G03750	-1.28	-0.36	-1.97	0.091788	0.185561	-5.51
253021_at	putative protein permease 1 - Mese	---	AT4G38050	-1.19	-0.25	-1.97	0.091804	0.185576	-5.51
252797_at	putative protein	---	---	-1.24	-0.31	-1.96	0.091822	0.185597	-5.51
252655_at	hypothetical protein with predicted h	---	AT3G44760	-1.15	-0.20	-1.96	0.091839	0.185614	-5.51
262752_at	putative mitotic cyclin similar to gb|[	CYCB3;1	AT1G16330	-1.21	-0.27	-1.96	0.091906	0.185734	-5.51
263364_at	unknown protein predicted by gens	---	AT2G20540	1.14	0.19	1.96	0.091927	0.185759	-5.51
258714_at	putative RING zinc finger protein c	---	AT3G09770	1.26	0.33	1.96	0.092003	0.18589	-5.51
266319_s_a	putative beta-ketoacyl-CoA synthas	KCS13	AT2G46720 ,	1.28	0.36	1.96	0.092008	0.18589	-5.51
256955_at	L-ascorbate oxidase precursor, put	sks11	AT3G13390	-1.26	-0.33	-1.96	0.092021	0.1859	-5.51
267544_at	putative cytochrome b5	CB5-B	AT2G32720	1.50	0.58	1.96	0.092051	0.185935	-5.51
263405_s_a	hypothetical protein similar to hypot	---	AT2G04066 ,	-1.14	-0.19	-1.96	0.09206	0.185935	-5.51
259168_at	unknown protein similar to hypothet	---	AT3G03570	-1.19	-0.25	-1.96	0.092063	0.185935	-5.51
261831_at	ADP-ribosylation factor, putative si	ATARFA1F	AT1G10630	-1.25	-0.33	-1.96	0.092074	0.185941	-5.51
256679_at	putative protein similar to ATP synth	ATPQ	AT3G52300	1.18	0.24	1.96	0.092105	0.185988	-5.51
261724_at	Expressed protein ; supported by fu	---	AT1G76185	1.16	0.22	1.96	0.092125	0.186012	-5.51
249365_at	putative protein predicted protein, A	---	AT5G40600	1.16	0.22	1.96	0.092248	0.186243	-5.51
257590_s_a	hypothetical protein predicted by ge	---	AT3G24870 ,	1.22	0.28	1.96	0.092276	0.186283	-5.51
262538_at	Expressed protein ; supported by fu	---	AT1G17140	-1.35	-0.44	-1.96	0.092287	0.186289	-5.51
256694_at	hypothetical protein predicted gens	---	---	-1.18	-0.23	-1.96	0.092323	0.186345	-5.51
261961_at	hypothetical protein predicted by ge	---	AT1G36510	-1.19	-0.25	-1.96	0.092372	0.186412	-5.51
247082_at	putative protein contains similarity t	ATGRIP	AT5G66030	1.19	0.25	1.96	0.092372	0.186412	-5.51

255140_x_a	extensin-like protein hydroxyproline	---	AT4G08410	-1.28	-0.36	-1.96	0.092394	0.186439	-5.51
257769_at	indoleacetic acid (IAA)-inducible ge	IAA7	AT3G23050	-2.71	-1.44	-1.96	0.092427	0.186489	-5.51
266451_at	3-isopropylmalate dehydratase, sm	---	AT2G43090	1.16	0.22	1.96	0.092456	0.186532	-5.51
255762_at	unknown protein contains Pfam pro	HAC12	AT1G16710	-1.17	-0.23	-1.96	0.092467	0.186536	-5.51
245326_at	Expressed protein ; supported by fu	---	AT4G14100	1.23	0.30	1.96	0.092496	0.186578	-5.51
253390_at	hypothetical protein ;supported by	---	AT4G32750	1.23	0.30	1.96	0.092514	0.186598	-5.51
249154_at	Nicotiana EREBP-3 like	---	AT5G43410	1.95	0.97	1.96	0.092527	0.186609	-5.51
258709_at	putative 60S ribosomal protein L35	---	AT3G09500	1.37	0.45	1.96	0.092606	0.18675	-5.52
249584_s_a	Membrane integral protein (MIP) -li	NIP4;1	AT5G37810	-1.20	-0.26	-1.96	0.092666	0.186857	-5.52
244922_s_a	hypothetical protein	---	AT2G07674	1.21	0.28	1.96	0.092724	0.186957	-5.52
259136_at	unknown protein	---	AT3G05470	-1.25	-0.32	-1.96	0.092849	0.187191	-5.52
261780_at	cyclin, putative similar to B-like cyc	CYCB2;4	AT1G76310	-1.18	-0.24	-1.96	0.092876	0.187227	-5.52
267458_at	similar to Mlo proteins from H. vulga	MLO5	AT2G33670	-1.22	-0.28	-1.96	0.092883	0.187227	-5.52
245842_at	proline-rich protein, putative similar	RXF26	AT1G58430	-1.14	-0.19	-1.96	0.092897	0.187234	-5.52
256926_at	hypothetical protein predicted by ge	---	AT3G22540	-1.24	-0.31	-1.96	0.092903	0.187234	-5.52
252818_at	putative protein several hypothetica	---	---	-1.19	-0.25	-1.96	0.092912	0.187236	-5.52
257240_at	monooxygenase, putatve contains	---	AT3G24200	1.16	0.21	1.96	0.092967	0.187328	-5.52
263854_at	putative mutT domain protein	atnudt5	AT2G04430	-1.16	-0.21	-1.96	0.092976	0.187328	-5.52
261977_at	hypothetical protein contains similar	---	---	-1.15	-0.20	-1.96	0.092983	0.187328	-5.52
265644_at	hypothetical protein predicted by ge	ATEPR1	AT2G27380	-1.15	-0.21	-1.96	0.093017	0.187382	-5.52
266361_at	putative O-GlcNAc transferase	---	AT2G32450	-1.36	-0.44	-1.96	0.093039	0.187408	-5.52
251569_at	putative protein several hypothetica	---	AT3G58290	-1.20	-0.26	-1.96	0.093047	0.187409	-5.52
262355_at	unknown protein	---	AT1G72820	-1.24	-0.30	-1.96	0.093056	0.187409	-5.52
259891_at	putative Eukaryotic initiation factor	---	AT1G72730	-1.20	-0.26	-1.96	0.093077	0.187437	-5.52
262270_x_a	polyprotein, putative similar to poly	---	---	-1.13	-0.18	-1.95	0.093158	0.187581	-5.52
260538_at	60S ribosomal protein L38	---	AT2G43460	1.27	0.34	1.95	0.09319	0.18763	-5.52
245573_at	hypothetical protein	---	AT4G14730	-1.14	-0.18	-1.95	0.093203	0.187639	-5.52
265713_at	putative integral membrane protein	UPS2	AT2G03530	1.58	0.66	1.95	0.093223	0.187663	-5.52
265764_at	hypothetical protein predicted by ge	---	AT2G48060	-1.20	-0.27	-1.95	0.093258	0.187718	-5.52
257953_at	predicted protein supported by full-l	PEX22	AT3G21865	1.29	0.37	1.95	0.093366	0.187919	-5.52
249035_at	putative protein contains similarity t	GLK2	AT5G44190	-1.20	-0.27	-1.95	0.093411	0.187992	-5.52
256180_at	light repressible receptor protein ki	---	AT1G51810	-1.12	-0.17	-1.95	0.093441	0.188026	-5.52
245647_at	F5A9.22 hypothetical protein	---	AT1G24706	-1.13	-0.18	-1.95	0.093444	0.188026	-5.52
264143_at	latex-abundant protein, putative sir	ATMC5	AT1G79330	-1.16	-0.22	-1.95	0.09347	0.188062	-5.52
255207_at	Expressed protein ; supported by fu	---	AT4G07515	-1.13	-0.17	-1.95	0.093487	0.188078	-5.53

264033_at	hypothetical protein predicted by ge	---	AT2G03580	-1.20	-0.26	-1.95	0.093549	0.188187	-5.53
266637_at	hypothetical protein ;supported by	BRXL1	AT2G35600	-1.28	-0.36	-1.95	0.093576	0.188225	-5.53
256638_at	RNA-binding protein, putative simil	---	AT3G19090	-1.25	-0.32	-1.95	0.093599	0.188254	-5.53
264310_at	hypothetical protein similar to hypot	---	AT1G62030	1.19	0.25	1.95	0.093632	0.188304	-5.53
259650_at	leucoanthocyanidin dioxygenase 2	---	AT1G55290	-1.17	-0.23	-1.95	0.09364	0.188304	-5.53
247238_at	histone acetyltransferase	HAM1	AT5G64610	1.14	0.19	1.95	0.093652	0.188311	-5.53
256839_at	calmodulin, putative similar to calm	---	AT3G22930	1.19	0.26	1.95	0.093667	0.188325	-5.53
246939_at	AP2 domain containing protein AP	SHN2	AT5G25390	-1.17	-0.23	-1.95	0.093692	0.188352	-5.53
245619_at	hypothetical protein	---	AT4G13990	-1.26	-0.33	-1.95	0.093697	0.188352	-5.53
245884_at	branched-chain alpha keto-acid de	---	AT5G09300	1.25	0.33	1.95	0.093728	0.188398	-5.53
262439_at	hypothetical protein identical to hyp	---	AT1G47730	-1.13	-0.18	-1.95	0.093741	0.188408	-5.53
266303_at	putative receptor-like protein kinase	---	AT2G27060	-1.33	-0.41	-1.95	0.093754	0.188417	-5.53
247503_at	GCN4-complementing protein - like	AGD1	AT5G61980	-1.20	-0.26	-1.95	0.093778	0.188448	-5.53
265635_at	hypothetical protein predicted by ge	---	AT2G25360	-1.14	-0.18	-1.95	0.093812	0.188489	-5.53
247616_at	putative protein various predicted p	---	AT5G60260	-1.16	-0.22	-1.95	0.093822	0.188489	-5.53
254275_at	HSP associated protein like p48, H	AtHip1	AT4G22670	1.21	0.28	1.95	0.093823	0.188489	-5.53
250596_at	putative protein contains similarity t	---	AT5G07780	-1.26	-0.33	-1.95	0.093845	0.188516	-5.53
255356_x_a	putative transposon protein	---	---	-1.18	-0.24	-1.95	0.093868	0.188545	-5.53
265860_at	hypothetical protein predicted by ge	---	AT2G01810	-1.15	-0.20	-1.95	0.093936	0.188666	-5.53
259991_at	putative S-adenosyl-L-methionine:s	---	AT1G68040	-1.17	-0.22	-1.95	0.093948	0.188674	-5.53
253367_at	hypothetical protein	---	AT4G33180	-1.30	-0.37	-1.95	0.093969	0.188692	-5.53
249792_at	putative protein contains similarity t	PHS1	AT5G23720	1.15	0.20	1.95	0.093974	0.188692	-5.53
265585_at	CDC27/NUC2-like protein TPR-rep	HBT	AT2G20000	1.21	0.27	1.95	0.094001	0.188728	-5.53
251932_at	pasticcino 1 ; supported by cDNA:	PAS1	AT3G54010	1.16	0.21	1.95	0.094014	0.188728	-5.53
259630_at	peptide chain release factor 2, puta	---	AT1G56350	-1.27	-0.35	-1.95	0.094017	0.188728	-5.53
264002_at	putative DnaJ protein ;supported b	---	AT2G22360	1.23	0.30	1.95	0.094032	0.188728	-5.53
258922_at	putative 40S ribosomal protein S17	---	AT3G10610	1.49	0.57	1.95	0.094033	0.188728	-5.53
250085_at	unknown protein	---	AT5G17250	-1.22	-0.29	-1.95	0.094089	0.188825	-5.53
259224_at	putative thymidine kinase similar to	---	AT3G07800	-1.24	-0.31	-1.95	0.094116	0.188862	-5.53
266897_at	remorin identical to GB:M25268, a	---	AT2G45820	-1.30	-0.38	-1.95	0.094178	0.188958	-5.53
247623_at	receptor-like protein kinase recepto	---	AT5G60320	-1.16	-0.21	-1.95	0.094181	0.188958	-5.53
258255_at	hypothetical protein predicted by ge	---	AT3G26800	1.15	0.20	1.95	0.094217	0.189015	-5.53
244935_at	NADH dehydrogenase subunit	---	---	-1.16	-0.21	-1.95	0.094261	0.189071	-5.53
256938_at	LEA protein, putative similar to LEA	ATECP31	AT3G22500	-1.18	-0.24	-1.95	0.094262	0.189071	-5.53
267125_at	putative acetone-cyanohydrin lyase	MES4	AT2G23580	-1.14	-0.19	-1.95	0.094313	0.189156	-5.53

255779_at	unknown protein similar to beta-1,3	PDCB3	AT1G18650	-1.40	-0.49	-1.95	0.094372	0.189243	-5.53
258927_at	putative folylpolyglutamate syntheta	DFC	AT3G10160	1.16	0.22	1.95	0.094372	0.189243	-5.53
258628_at	unknown protein	---	AT3G02890	1.36	0.44	1.95	0.094389	0.189259	-5.53
254812_at	nucleotide sugar epimerase -like pr	GAE5	AT4G12250	1.45	0.54	1.95	0.094425	0.189315	-5.53
244924_at	hypothetical protein	---	---	1.18	0.24	1.95	0.094437	0.189321	-5.53
246878_at	putative protein self-incompatibility	---	AT5G26060	-1.22	-0.28	-1.95	0.094507	0.189445	-5.54
245472_at	hypothetical protein	---	AT4G16040	-1.21	-0.27	-1.94	0.09461	0.189622	-5.54
248860_at	putative protein contains similarity t	---	AT5G46670	-1.16	-0.21	-1.94	0.094613	0.189622	-5.54
256782_at	dirigent protein, putative similar to (	---	AT3G13660	-1.13	-0.18	-1.94	0.09462	0.189622	-5.54
258406_at	hypothetical protein similar to hypot	ATRBL14	AT3G17611	1.13	0.17	1.94	0.094643	0.189651	-5.54
248411_at	unknown protein	---	AT5G51580	-1.14	-0.19	-1.94	0.094658	0.189651	-5.54
263645_at	putative GTP-binding protein	---	AT2G04720	-1.13	-0.17	-1.94	0.094659	0.189651	-5.54
251035_at	Expressed protein ; supported by fu	---	AT5G02220	-1.26	-0.34	-1.94	0.094671	0.189658	-5.54
248649_at	putative protein contains similarity t	APRR4	AT5G49240	-1.19	-0.25	-1.94	0.094688	0.189674	-5.54
266204_at	hypothetical protein predicted by ge	---	AT2G02410	-1.24	-0.31	-1.94	0.094741	0.189765	-5.54
260435_at	putative transcription factor similar t	MYB62	AT1G68320	1.45	0.54	1.94	0.09475	0.189766	-5.54
252068_at	mucin -like protein hemomucin, Dro	---	AT3G51440	1.53	0.61	1.94	0.094805	0.189859	-5.54
246515_at	putative protein unusual floral orga	---	AT5G15710	-1.13	-0.17	-1.94	0.094826	0.189865	-5.54
261583_at	pyruvate dehydrogenase E1 alpha s	PDH-E1 AL	AT1G01090	-1.16	-0.22	-1.94	0.094833	0.189865	-5.54
245053_at	putative pectinesterase	---	AT2G26450	-1.24	-0.31	-1.94	0.094835	0.189865	-5.54
256499_at	unknown protein	---	AT1G36640	1.34	0.42	1.94	0.094843	0.189865	-5.54
249563_at	similar to unknown protein (pir |T03	---	AT5G38360	-1.16	-0.21	-1.94	0.09485	0.189865	-5.54
256869_at	nodulin, putative similar to nodulin	---	AT3G26420 ,	1.26	0.33	1.94	0.094865	0.189878	-5.54
251012_at	putative protein ;supported by full-l	---	AT5G02580	2.28	1.19	1.94	0.094872	0.189878	-5.54
246140_at	SOH1 - like protein SOH1, Saccha	---	AT5G19910	-1.13	-0.18	-1.94	0.094886	0.189888	-5.54
246918_at	putative protein hypothetical protei	---	AT5G25340	-1.22	-0.28	-1.94	0.094957	0.190014	-5.54
249006_at	putative protein similar to unknown	---	AT5G44650 ,	1.26	0.33	1.94	0.094986	0.190056	-5.54
263739_at	putative CONSTANS-like B-box zin	---	AT2G21320	1.35	0.43	1.94	0.095031	0.190129	-5.54
245406_at	GTP-binding RAB2A like protein	ATRABB1A	AT4G17160	-1.14	-0.19	-1.94	0.095089	0.190217	-5.54
266871_at	unknown protein	---	AT2G44710	1.12	0.17	1.94	0.095092	0.190217	-5.54
250515_at	putative protein	---	AT5G09570	1.12	0.16	1.94	0.09513	0.19026	-5.54
267625_at	putative RNA-binding protein	---	AT2G42240	-1.17	-0.22	-1.94	0.095146	0.19026	-5.54
263459_at	ankyrin-like protein	---	AT2G31820	1.27	0.34	1.94	0.095148	0.19026	-5.54
254219_at	putative protein	---	AT4G23760	1.17	0.22	1.94	0.095156	0.19026	-5.54
267450_at	hypothetical protein predicted by ge	---	AT2G33720	-1.26	-0.33	-1.94	0.09516	0.19026	-5.54

267498_at	unknown protein	---	AT2G45720	1.24	0.31	1.94	0.095163	0.19026	-5.54
251966_at	receptor lectin kinase -like protein r	---	AT3G53380	-1.23	-0.30	-1.94	0.095191	0.190298	-5.54
258397_at	Expressed protein ; supported by fu	---	AT3G15357	1.15	0.21	1.94	0.09524	0.190381	-5.54
262862_at	hypothetical protein predicted by ge	---	AT1G64830	-1.26	-0.34	-1.94	0.095353	0.190575	-5.54
250201_at	ankyrin - like protein 270 kDa anky	---	AT5G14230	-1.47	-0.56	-1.94	0.095354	0.190575	-5.54
250558_at	flavonoid 3-hydroxylase - like prote	TT7	AT5G07990	-1.20	-0.26	-1.94	0.095366	0.190581	-5.54
257101_at	disease resistance protein, putative	AtRLP42	AT3G25020	1.17	0.23	1.94	0.095419	0.19067	-5.55
264042_at	putative steroid sulfotransferase ; s	ST	AT2G03760	-2.03	-1.02	-1.94	0.095484	0.190784	-5.55
258584_at	hypothetical protein predicted by ge	SIP1A	AT3G04090	-1.13	-0.17	-1.94	0.095567	0.190933	-5.55
247416_at	40S ribosomal protein S15-like prot	---	AT5G63070	-1.19	-0.25	-1.94	0.095672	0.191127	-5.55
265579_at	hypothetical protein predicted by ge	---	AT2G20070	-1.16	-0.21	-1.94	0.095684	0.191133	-5.55
256322_at	unknown protein ; supported by cDI	AXR4	AT1G54990	1.37	0.45	1.94	0.095701	0.191149	-5.55
246179_at	putative protein hypothetical protei	---	AT5G28440	-1.23	-0.30	-1.94	0.095716	0.191149	-5.55
248880_at	putative protein contains similarity t	---	AT5G46200	-1.15	-0.20	-1.94	0.095717	0.191149	-5.55
260040_at	Expressed protein ; supported by fu	IDA	AT1G68765	2.06	1.04	1.94	0.095748	0.191174	-5.55
246473_at	putative protein cathepsin L-like cy	---	AT5G17140	-1.18	-0.24	-1.94	0.095754	0.191174	-5.55
263954_at	unknown protein ; supported by cD	---	AT2G35840	-1.21	-0.28	-1.94	0.095755	0.191174	-5.55
266708_at	putative chloroplast nucleoid DNA b	---	AT2G03200	-1.24	-0.31	-1.94	0.095787	0.191208	-5.55
258004_at	unknown protein similar to putative	MYB110	AT3G29020	-1.17	-0.23	-1.94	0.095789	0.191208	-5.55
251919_at	putative protein Hsp70 binding prot	---	AT3G53800	-1.27	-0.35	-1.94	0.09581	0.191234	-5.55
265766_at	unknown protein	---	AT2G48080	-1.23	-0.30	-1.93	0.095932	0.191462	-5.55
249955_at	sugar transporter - like protein prob	---	AT5G18840	-1.47	-0.56	-1.93	0.095954	0.191489	-5.55
247226_at	putative protein contains similarity t	---	AT5G65100	-1.14	-0.18	-1.93	0.095988	0.19154	-5.55
249151_at	inorganic phosphate transporter (db	PHT3	AT5G43360	-1.17	-0.22	-1.93	0.09604	0.191627	-5.55
245938_at	putative protein protein phosphatas	---	AT5G19680	1.23	0.30	1.93	0.096107	0.191742	-5.55
251545_at	zinc transporter -like protein zinc tr	MTPA2	AT3G58810	-1.24	-0.32	-1.93	0.096145	0.191802	-5.55
254898_at	serine/threonine kinase-like protein	---	AT4G11480	1.85	0.89	1.93	0.096309	0.192113	-5.55
253085_s_a	putative protein hypothetical protei	---	AT4G36270 ,	-1.29	-0.37	-1.93	0.096321	0.192119	-5.55
251276_at	putative protein hypothetical protei	SDG14	AT3G61740	1.15	0.20	1.93	0.096347	0.192154	-5.55
251448_at	allyl alcohol dehydrogenase-like pr	---	AT3G59845	-1.15	-0.21	-1.93	0.096383	0.192211	-5.55
261726_at	putative auxin-independent growth	---	AT1G76270	-1.31	-0.39	-1.93	0.096397	0.192221	-5.55
256842_at	hypothetical protein predicted by ge	---	---	-1.16	-0.21	-1.93	0.096413	0.192236	-5.56
247102_at	putative protein similar to unknown	---	AT5G66550	-1.19	-0.26	-1.93	0.09643	0.192253	-5.56
246505_at	putative protein hypothetical protei	---	AT5G16250	-1.32	-0.40	-1.93	0.096449	0.192274	-5.56
248485_at	calcium-binding transporter-like pro	---	AT5G51050	1.25	0.32	1.93	0.096473	0.192305	-5.56

246264_at	hypothetical protein	FRL2	AT1G31814	1.17	0.23	1.93	0.096503	0.192348	-5.56
260847_s_a	alanine aminotransferase, putative	AlaAT1	AT1G17290	1.16	0.22	1.93	0.096549	0.192422	-5.56
249708_at	unknown protein	---	AT5G35660	-1.35	-0.43	-1.93	0.096574	0.192456	-5.56
251732_at	putative protein prenylated Rab ac	PRA1.B1	AT3G56110	1.15	0.20	1.93	0.096617	0.192526	-5.56
262494_at	myosin-like protein similar to myosi	---	AT1G21810	-1.21	-0.28	-1.93	0.096706	0.192685	-5.56
264992_at	integral membrane protein, putative	---	AT1G67300	-1.21	-0.28	-1.93	0.096742	0.192741	-5.56
267216_at	hypothetical protein	---	AT2G02620	-1.16	-0.22	-1.93	0.096798	0.192835	-5.56
247414_at	unknown protein	EMB2759	AT5G63050	1.21	0.27	1.93	0.096812	0.192846	-5.56
249038_at	putative protein contains similarity t	RING1A	AT5G44280	1.18	0.24	1.93	0.096884	0.192972	-5.56
263905_at	putative beta-fructofuranosidase (in	AtcwINV4	AT2G36190	-1.25	-0.32	-1.93	0.096942	0.193071	-5.56
258721_at	unknown protein similar to hypothet	---	AT3G09560	1.26	0.33	1.93	0.096982	0.193135	-5.56
264455_at	hypothetical protein predicted by ge	---	AT1G10330	1.20	0.26	1.93	0.097042	0.193236	-5.56
258416_at	hypothetical protein predicted by g	---	AT3G17310	1.17	0.23	1.93	0.097066	0.193267	-5.56
255902_at	transcription factor, putative similar	---	AT1G17880	1.16	0.22	1.93	0.097105	0.193326	-5.56
246771_at	putative protein putative replication	---	---	-1.14	-0.19	-1.93	0.097119	0.193326	-5.56
260079_s_a	helix-loop-helix protein 1A, putative	---	AT1G35460	-1.17	-0.22	-1.93	0.097121	0.193326	-5.56
266958_at	putative trans-prenyltransferase	GPS1	AT2G34630	1.25	0.32	1.93	0.097156	0.193379	-5.56
265611_at	unknown protein ;supported by full-	---	AT2G25510	-1.32	-0.41	-1.93	0.097209	0.193469	-5.56
260367_at	unknown protein ;supported by full-	---	AT1G69760	1.54	0.63	1.93	0.09723	0.193494	-5.56
256132_at	hypothetical protein similar to hypot	---	AT1G13610	-1.16	-0.21	-1.93	0.097261	0.193534	-5.56
265001_at	hypothetical protein contains simila	---	AT1G67350	1.16	0.21	1.93	0.097268	0.193534	-5.56
250380_at	putative protein	---	AT5G11600	1.16	0.22	1.92	0.097356	0.193694	-5.56
247998_at	unknown protein	---	AT5G56200	1.30	0.38	1.92	0.097373	0.19371	-5.56
262371_at	putative glycosyl transferase contai	---	AT1G73160	-1.15	-0.20	-1.92	0.09739	0.193727	-5.56
255609_s_a	hypothetical protein	---	AT4G01180	1.13	0.18	1.92	0.097562	0.194052	-5.57
246181_at	pectinesterase - like protein probab	---	AT5G20860	-1.14	-0.19	-1.92	0.097642	0.194178	-5.57
256317_at	hypothetical protein predicted by ge	---	AT1G35900	-1.27	-0.34	-1.92	0.097643	0.194178	-5.57
246790_at	putative protein unknown protein F	---	AT5G27610	-1.23	-0.30	-1.92	0.097685	0.194243	-5.57
254015_at	putative beta-galactosidase beta-g	BGAL12	AT4G26140	-1.27	-0.34	-1.92	0.097701	0.194243	-5.57
265886_at	putative protein phosphatase 2C ; s	---	AT2G25610	1.36	0.45	1.92	0.097701	0.194243	-5.57
264888_at	hypothetical protein	---	AT1G23070	-1.17	-0.23	-1.92	0.097718	0.19426	-5.57
261985_at	terpene synthase, putative similar t	---	AT1G33750	-1.14	-0.18	-1.92	0.097798	0.194402	-5.57
257438_at	hypothetical protein similarity to pla	---	AT2G15325	-1.14	-0.19	-1.92	0.097836	0.194449	-5.57
263514_at	putative poly(ADP-ribose) glycohyd	---	AT2G31860	-1.17	-0.22	-1.92	0.097845	0.194449	-5.57
248505_at	putative protein similar to unknown	---	AT5G50360	-1.20	-0.27	-1.92	0.097847	0.194449	-5.57

249858_at	myb-related transcription factor-like	MYB37	AT5G23000	1.23	0.30	1.92	0.097869	0.194477	-5.57
257930_at	hypothetical protein	---	AT3G17010	-1.23	-0.30	-1.92	0.097934	0.194589	-5.57
263852_at	putative mutT domain protein	ATNUDT6	AT2G04450	-2.18	-1.13	-1.92	0.09801	0.194723	-5.57
251079_at	hypothetical protein	---	AT5G02000	-1.15	-0.20	-1.92	0.098035	0.194755	-5.57
248257_at	putative protein similar to unknown	---	AT5G53410	-1.23	-0.29	-1.92	0.098094	0.194855	-5.57
250328_at	putative protein	---	AT5G11780	-1.19	-0.25	-1.92	0.098118	0.194878	-5.57
266367_s_a	unknown protein	---	AT2G41390	-1.15	-0.20	-1.92	0.098122	0.194878	-5.57
257430_at	hypothetical protein similar to hypot	---	AT2G28580	-1.21	-0.28	-1.92	0.098175	0.194965	-5.57
260061_at	cell division protein kinase similar to	CDKD1;1	AT1G73690	-1.26	-0.33	-1.92	0.09819	0.194965	-5.57
262704_at	hypothetical protein predicted by ge	ASL9	AT1G16530	-1.42	-0.51	-1.92	0.098198	0.194965	-5.57
257107_at	transmembrane trafficking protein,	---	AT3G29070	-1.18	-0.24	-1.92	0.0982	0.194965	-5.57
246941_at	putative protein several hypothetica	---	AT5G25415	-1.16	-0.21	-1.92	0.098282	0.19511	-5.57
264576_at	unknown protein Similar to Arabidop	---	AT1G05360	1.16	0.22	1.92	0.098293	0.195115	-5.57
252070_at	short-chain alcohol dehydrogenase	---	AT3G51680	-1.21	-0.27	-1.92	0.098302	0.195116	-5.57
250742_at	putative protein similar to unknown	---	AT5G05800	1.18	0.24	1.92	0.098338	0.19517	-5.57
255515_at	putative pistil-specific protein simila	---	AT4G02250	-1.14	-0.19	-1.92	0.09837	0.195207	-5.57
257031_at	fucosyltransferase, putative similar	FUT11	AT3G19280	1.17	0.23	1.92	0.098374	0.195207	-5.57
247223_at	MADS box transcription factor-like	MAF4	AT5G65070	-1.13	-0.17	-1.92	0.09839	0.195222	-5.57
254440_at	putative protein desiccation-related	---	AT4G21020	-1.15	-0.20	-1.92	0.098421	0.195267	-5.58
248128_at	thiazole biosynthetic enzyme precu	THI1	AT5G54770	1.22	0.28	1.92	0.098441	0.195289	-5.58
253522_at	predicted protein cation transport p	---	AT4G31290	-1.54	-0.63	-1.92	0.098467	0.195324	-5.58
263803_at	hypothetical protein predicted by ge	---	AT2G40440	-1.15	-0.20	-1.92	0.098476	0.195325	-5.58
252724_at	putative protein hypothetical protein	---	AT3G43540	1.20	0.27	1.92	0.098489	0.195334	-5.58
267517_at	unknown protein	RPT2	AT2G30520	1.19	0.25	1.92	0.098519	0.195377	-5.58
252019_at	late embryogenesis abundant prote	---	AT3G53040	-1.13	-0.18	-1.92	0.098558	0.195423	-5.58
257683_at	hypothetical protein predicted by ge	---	AT3G13280	-1.16	-0.21	-1.92	0.09856	0.195423	-5.58
245335_at	pore protein homolog ;supported by	ATOEP16-1	AT4G16160	-1.21	-0.27	-1.92	0.098585	0.195455	-5.58
251051_at	putative zinc finger protein zinc fing	---	AT5G02460	-1.18	-0.24	-1.92	0.098664	0.195586	-5.58
261996_at	AIG1-like protein similar to AIG1 pr	---	AT1G33830	1.13	0.18	1.92	0.098669	0.195586	-5.58
249844_at	putative protein similar to unknown	---	AT5G23590	1.19	0.25	1.92	0.098676	0.195586	-5.58
254987_s_a	hypothetical protein	---	AT4G10660	1.13	0.17	1.91	0.098756	0.195727	-5.58
250791_at	nucleic acid binding protein-like ;su	AL1	AT5G05610	1.15	0.21	1.91	0.098773	0.195732	-5.58
258153_at	Expressed protein ; supported by fu	MOS4	AT3G18165	1.20	0.27	1.91	0.098775	0.195732	-5.58
251677_at	putative protein hypothetical protein	BHLH039	AT3G56980	-1.64	-0.71	-1.91	0.098805	0.195761	-5.58
256644_at	unknown protein similar to hypothet	---	---	-1.15	-0.21	-1.91	0.098807	0.195761	-5.58

247278_at	fructose-bisphosphatase-like protei	---	AT5G64380	-1.20	-0.27	-1.91	0.098848	0.195814	-5.58
253817_at	hypothetical protein ;supported by	---	AT4G28310	-1.52	-0.60	-1.91	0.098851	0.195814	-5.58
264461_s_a	hypothetical protein	---	AT1G10190	-1.12	-0.17	-1.91	0.09892	0.195927	-5.58
265928_at	Ac-like transposase related to Ac/D	---	---	-1.20	-0.26	-1.91	0.098925	0.195927	-5.58
256699_at	hypothetical protein predicted by ge	---	AT3G20690	-1.20	-0.26	-1.91	0.098952	0.195958	-5.58
265030_at	receptor-like kinase, putative simila	---	AT1G61610	1.38	0.46	1.91	0.098958	0.195958	-5.58
246773_at	protein kinase-like protein MAP3Ka	---	AT5G27510	-1.24	-0.31	-1.91	0.098968	0.195961	-5.58
254853_at	putative DNA-binding protein AT-h	---	AT4G12080	1.22	0.29	1.91	0.099005	0.196017	-5.58
266529_at	putative salt-inducible protein	---	AT2G16880	1.19	0.25	1.91	0.099021	0.19603	-5.58
246356_x_a	hypothetical protein, 5' partial	---	---	-1.14	-0.19	-1.91	0.099039	0.19603	-5.58
257289_at	hypothetical protein	---	AT3G29690	-1.14	-0.19	-1.91	0.099043	0.19603	-5.58
246697_at	putative protein	---	AT5G29210	-1.18	-0.24	-1.91	0.099046	0.19603	-5.58
263755_at	unknown protein ;supported by full-	---	AT2G21340	-1.23	-0.29	-1.91	0.099097	0.196111	-5.58
264858_at	hypothetical protein	SNL3	AT1G24190	-1.18	-0.24	-1.91	0.099104	0.196111	-5.58
260822_at	RNA polymerase, putative similar t	---	AT1G06790	1.16	0.21	1.91	0.099152	0.196175	-5.58
255664_at	putative protein	---	AT4G00440	-1.14	-0.19	-1.91	0.099154	0.196175	-5.58
248263_at	pectinesterase ; supported by cDN	PMEPCRF	AT5G53370	-1.37	-0.45	-1.91	0.099198	0.196244	-5.58
260284_at	unknown protein ; supported by ful	---	AT1G80380	-1.15	-0.20	-1.91	0.099262	0.196356	-5.58
248657_at	peptidylprolyl isomerase	ROF2	AT5G48570	-2.68	-1.42	-1.91	0.099297	0.196396	-5.58
257838_at	hypothetical protein contains Pfam	---	AT3G25210	1.15	0.20	1.91	0.099306	0.196396	-5.58
247087_at	putative protein contains similarity t	---	AT5G66330	-1.32	-0.40	-1.91	0.099312	0.196396	-5.58
255941_at	mitochondrial inner membrane tran	ATTIM17-1	AT1G20350	-1.28	-0.36	-1.91	0.099321	0.196396	-5.58
246638_at	putative protein predicted proteins,	---	---	-1.17	-0.22	-1.91	0.099326	0.196396	-5.58
259818_at	unknown protein ; supported by cDI	---	AT1G49890	-1.24	-0.32	-1.91	0.099419	0.196562	-5.59
250552_at	putative protein microtubule binding	---	AT5G07890	1.15	0.20	1.91	0.099468	0.196616	-5.59
249679_at	unknown protein	---	AT5G35980	1.12	0.16	1.91	0.09947	0.196616	-5.59
262048_at	cytochrome c oxidase subunit, puta	---	AT1G80230	1.18	0.24	1.91	0.099472	0.196616	-5.59
247052_at	homeodomain transcription factor-li	HB53	AT5G66700	-1.18	-0.24	-1.91	0.099508	0.19667	-5.59
267006_at	putative membrane transporter ; su	---	AT2G34190	-1.18	-0.24	-1.91	0.099545	0.196716	-5.59
267307_at	putative laccase	LAC3	AT2G30210	-1.22	-0.29	-1.91	0.099548	0.196716	-5.59
255047_at	AX110P -like protein AX110P -Dau	---	AT4G09670	1.16	0.22	1.91	0.099608	0.196816	-5.59
250719_at	putative protein contains similarity t	---	AT5G06250	-1.20	-0.26	-1.91	0.09966	0.196904	-5.59
264075_at	envelope-like protein identical to GE	---	---	-1.23	-0.30	-1.91	0.099674	0.196914	-5.59
254477_at	zinc-finger protein Lsd1 ;supported	---	AT4G20380	-1.22	-0.29	-1.91	0.099786	0.197118	-5.59
261463_at	hypothetical protein predicted by ge	---	AT1G07730	1.14	0.19	1.91	0.099809	0.197147	-5.59

267241_at	hypothetical protein predicted by ge	---	AT2G02490	-1.14	-0.19	-1.91	0.099838	0.197187	-5.59
264137_at	lupeol synthase, putative similar to	ATLUP2	AT1G78960	1.27	0.34	1.91	0.099937	0.197365	-5.59
262784_at	unknown protein strong similarity to	SEX1	AT1G10760	1.27	0.35	1.91	0.100023	0.197518	-5.59
252370_at	putative protein	---	AT3G48600	-1.31	-0.38	-1.91	0.100119	0.19769	-5.59
258743_s_a	shaggy related protein kinase, ASK	ATSK12	AT3G05840	1.19	0.25	1.90	0.100169	0.197771	-5.59
246750_at	putative protein predicted proteins,	AT5G2786(	---	1.29	0.36	1.90	0.100327	0.198067	-5.59
252661_at	putative protein	---	AT3G44450	1.60	0.68	1.90	0.100367	0.198128	-5.59
254835_s_a	flavonoid 3,5-hydroxylase -like prot	CYP706A5	AT4G12310	1.35	0.43	1.90	0.100377	0.198132	-5.59
256340_at	DnaJ protein, putative contains Pfa	---	AT1G72070	-1.19	-0.25	-1.90	0.100391	0.198141	-5.59
252358_at	putative protein cfxY protein, Alcali	---	AT3G48425	1.15	0.20	1.90	0.100421	0.198178	-5.59
266696_at	copia-like retroelement pol polyprot	---	AT2G19680	1.20	0.26	1.90	0.100433	0.198178	-5.59
246268_at	cytochrome P450, putative contain	CYP97A3	AT1G31800	-1.21	-0.28	-1.90	0.100436	0.198178	-5.59
259269_at	putative pectate lyase similar to pec	---	AT3G01270	-1.17	-0.23	-1.90	0.100447	0.198178	-5.60
266882_at	unknown protein ;supported by full-	---	AT2G44670	1.48	0.56	1.90	0.100453	0.198178	-5.60
257371_at	putative CCAAT-box binding trancri	NF-YB5	AT2G47810	-1.20	-0.26	-1.90	0.100476	0.198206	-5.60
249855_at	unknown protein	---	AT5G22970	-1.17	-0.22	-1.90	0.100566	0.198367	-5.60
259815_at	hypothetical protein predicted by ge	---	AT1G49870	-1.16	-0.21	-1.90	0.100647	0.198509	-5.60
258612_at	putative replication factor A similar t	---	AT3G02920	-1.17	-0.23	-1.90	0.100656	0.19851	-5.60
262180_at	phosphoglycerate mutase, putative	PGM	AT1G78050	-1.15	-0.20	-1.90	0.100751	0.198681	-5.60
265829_at	unknown protein	---	AT2G14490	-1.14	-0.18	-1.90	0.100769	0.198697	-5.60
254809_at	putative protein auxin-induced prot	---	AT4G12410	-1.25	-0.32	-1.90	0.100802	0.198745	-5.60
255165_at	putative protein putative centromer	---	---	-1.30	-0.38	-1.90	0.100825	0.198775	-5.60
253770_at	protein kinase ADK1-like protein pro	CKL6	AT4G28540	1.14	0.19	1.90	0.100874	0.198853	-5.60
249299_at	E2, ubiquitin-conjugating enzyme 4	UBC4	AT5G41340	-1.20	-0.27	-1.90	0.100924	0.198934	-5.60
260164_at	putative D-isomer specific 2-hydrox	---	AT1G79870	1.16	0.22	1.90	0.100959	0.198986	-5.60
264355_at	unknown protein similar to PHZF, c	---	AT1G03210	-1.26	-0.34	-1.90	0.100976	0.199003	-5.60
259986_at	thaumatin-like protein similar to thau	---	AT1G75050	-1.18	-0.24	-1.90	0.101003	0.19904	-5.60
259191_at	unknown protein	---	AT3G01720	-1.21	-0.27	-1.90	0.101049	0.199098	-5.60
263099_at	putative non-LTR retroelement reve	---	---	-1.22	-0.29	-1.90	0.101051	0.199098	-5.60
246360_at	hypothetical protein	---	---	-1.16	-0.21	-1.90	0.10106	0.1991	-5.60
247941_at	putative protein strong similarity to u	---	AT5G57200	-1.14	-0.19	-1.90	0.101098	0.199157	-5.60
259452_at	En/Spm-like transposon protein, pu	---	---	-1.17	-0.22	-1.90	0.101137	0.199217	-5.60
245215_at	F12A21.4 similar to iEP4 gb|AAD11	ATFXG1	AT1G67830	1.40	0.48	1.90	0.101156	0.199238	-5.60
251875_at	putative protein various predicted p	---	AT3G54270	-1.21	-0.27	-1.90	0.101168	0.199243	-5.60
254522_at	hypothetical protein	---	AT4G19980	-1.13	-0.17	-1.90	0.101181	0.199252	-5.60

251409_at	Expressed protein ; supported by fu	---	AT3G60245	1.46	0.54	1.90	0.101194	0.19926	-5.60
250800_at	ubiquinol-cytochrome C reductase c	HPR	AT1G68010	1.41	0.49	1.90	0.101254	0.199361	-5.60
245097_at	Expressed protein ; supported by fu	---	AT2G40935	1.13	0.18	1.90	0.101287	0.199409	-5.60
262110_at	SF2/ASF-like splicing modulator Sr	SR1	AT1G02840	1.21	0.27	1.90	0.101437	0.199687	-5.60
262040_at	receptor protein kinase, putative si	TMM	AT1G80080	-1.17	-0.23	-1.90	0.101476	0.199748	-5.61
248434_at	mitochondrial heat shock 22 kd prot	---	AT5G51440	2.25	1.17	1.90	0.101495	0.199766	-5.61
262082_s_a	wall-associated kinase 2, putative s	---	AT1G56120	1.52	0.60	1.90	0.101552	0.199847	-5.61
265808_at	hypothetical protein predicted by ge	---	AT2G17960	-1.15	-0.21	-1.90	0.101553	0.199847	-5.61
263426_at	putative glutathione peroxidase ;su	ATGPX2	AT2G31570	1.26	0.33	1.90	0.101585	0.199887	-5.61
263603_at	hypothetical protein	---	AT2G16340	-1.21	-0.27	-1.90	0.101591	0.199887	-5.61
251089_at	heat shock protein 40-like heat sho	---	AT5G01390	1.15	0.20	1.89	0.101636	0.199959	-5.61
264941_at	auxin-induced protein, putative sim	---	AT1G60680	-1.31	-0.39	-1.89	0.101658	0.199984	-5.61
256380_at	pheromone receptor, putative (AR4	AR401	AT1G66680	1.19	0.26	1.89	0.10168	0.20001	-5.61
AFFX-BioB-	E. coli /GEN=bioB /DB_XREF=gb:J	---	---	-1.57	-0.65	-1.89	0.101711	0.200055	-5.61
248800_at	40S ribosomal protein S19 ; suppo	RPS19	AT5G47320	1.17	0.22	1.89	0.101739	0.200093	-5.61
247635_at	serine/threonine-protein kinase - lik	GRIK2	AT5G60548	-1.14	-0.18	-1.89	0.101782	0.200158	-5.61
256997_at	subtilisin-like serine protease, puta	---	AT3G14067	1.27	0.35	1.89	0.101807	0.200192	-5.61
261374_at	hypothetical protein contains thiorec	---	AT1G52990	-1.14	-0.19	-1.89	0.10188	0.200317	-5.61
252902_at	putative protein various predicted p	---	AT4G39560	1.16	0.22	1.89	0.101905	0.200349	-5.61
251696_at	putative protein	---	---	-1.20	-0.26	-1.89	0.101961	0.200443	-5.61
250779_at	eukaryotic translation initiation facto	EIF2 ALPH	AT5G05470	1.27	0.34	1.89	0.101982	0.200466	-5.61
248259_at	proline-rich cell wall protein-like ;su	---	AT5G53330	1.16	0.22	1.89	0.102008	0.200499	-5.61
254163_s_a	putative protein storage protein - P	---	AT4G24340	1.88	0.91	1.89	0.102049	0.200558	-5.61
256121_at	MAP kinase, putative similar to MA	---	AT1G18160	1.17	0.22	1.89	0.102055	0.200558	-5.61
246341_x_a	putative protein predicted proteins,	---	AT3G43150	-1.21	-0.28	-1.89	0.102114	0.200643	-5.61
253651_at	putative protein nucleoid DNA-bind	---	AT4G30030	-1.19	-0.26	-1.89	0.102116	0.200643	-5.61
246318_at	putative protein predicted protein B	---	AT3G56740	1.20	0.26	1.89	0.102128	0.200644	-5.61
263043_at	conserved hypothetical protein prec	---	AT1G23350	-1.19	-0.26	-1.89	0.102134	0.200644	-5.61
261294_at	dihydroxyacetone kinase, putative	---	AT1G48430	1.24	0.31	1.89	0.102149	0.200655	-5.61
259383_at	putative lectin contains Pfam profile	JR1	AT3G16470	-2.02	-1.01	-1.89	0.102166	0.200673	-5.61
266269_at	putative glutathione S-transferase	ATGSTU2	AT2G29480	1.54	0.62	1.89	0.102231	0.200783	-5.61
254020_at	beta-carotene hydroxylase ; suppo	BETA-OHA	AT4G25700	1.67	0.74	1.89	0.102254	0.20081	-5.61
254490_at	CTP synthase like protein CTP syn	---	AT4G20320	1.27	0.35	1.89	0.102284	0.200851	-5.61
262003_at	unknown protein	---	AT1G64460	1.17	0.23	1.89	0.102329	0.200922	-5.61
248568_at	receptor protein kinase-like	---	AT5G49760	1.20	0.26	1.89	0.10243	0.201104	-5.61

248489_at	unknown protein	---	AT5G51090	-1.14	-0.19	-1.89	0.102446	0.201119	-5.61
250002_at	proline-richh protein predicted prot	AGP25	AT5G18690	-1.58	-0.66	-1.89	0.102466	0.201139	-5.61
259876_at	putative DnaJ protein similar to dna	---	AT1G76700	-1.32	-0.40	-1.89	0.102538	0.201264	-5.62
254970_at	chlorophyll a/b-binding protein - like	LHCB5	AT4G10340	-1.15	-0.21	-1.89	0.102653	0.20146	-5.62
246157_at	cytochrome-b5 reductase - like pro	---	AT5G20080	-1.21	-0.28	-1.89	0.102656	0.20146	-5.62
253170_at	putative protein various predicted p	---	AT4G35130	1.13	0.18	1.89	0.102708	0.201546	-5.62
258274_at	putative cytochrome c oxidase subu	---	AT3G15640	1.19	0.26	1.89	0.102728	0.201568	-5.62
263639_at	unknown protein	---	AT2G25300	1.18	0.24	1.89	0.102749	0.201592	-5.62
245082_at	hypothetical protein predicted by ge	---	AT2G23270	2.64	1.40	1.89	0.102808	0.201689	-5.62
249085_at	putative protein similar to unknown	---	AT5G44170	-1.18	-0.23	-1.89	0.102899	0.201834	-5.62
246219_at	aminopeptidase-like protein ; suppo	APP1	AT4G36760	1.24	0.31	1.89	0.102899	0.201834	-5.62
263779_at	putative photomorphogenesis repre	SPA1	AT2G46340	1.19	0.25	1.89	0.102914	0.201846	-5.62
250819_at	putative protein strong similarity to u	---	AT5G05100	1.18	0.24	1.89	0.102932	0.201864	-5.62
263390_at	hypothetical protein similar to Ta1-1	---	---	-1.14	-0.19	-1.89	0.10295	0.201883	-5.62
248924_at	GDSL-motif lipase/hydrolase-like pr	---	AT5G45960	-1.14	-0.20	-1.89	0.103006	0.20196	-5.62
262365_at	disease resistance protein, putative	---	AT1G72870	-1.20	-0.26	-1.89	0.103008	0.20196	-5.62
265787_at	Mutator-like transposase similar to	---	---	-1.22	-0.29	-1.89	0.103035	0.201996	-5.62
261808_at	DNA damage repair protein, putativ	DRT111	AT1G30480	1.15	0.20	1.89	0.103049	0.202006	-5.62
265748_at	putative Athila retroelement ORF1 p	---	---	-1.16	-0.22	-1.89	0.103086	0.202062	-5.62
246467_at	UDP glucose:flavonoid 3-o-glucosy	---	AT5G17040	-1.25	-0.33	-1.89	0.103103	0.202077	-5.62
252372_at	aldehyde dehydrogenase (NAD+)-l	ALDH2B4	AT3G48000	1.24	0.31	1.88	0.103118	0.202089	-5.62
255161_at	putative reverse transcriptase	---	---	1.15	0.20	1.88	0.103146	0.202128	-5.62
253288_at	hypothetical protein	---	AT4G34310	-1.17	-0.23	-1.88	0.103236	0.202287	-5.62
250511_at	putative protein similar to unknown	---	AT5G09960	-1.17	-0.22	-1.88	0.103339	0.202462	-5.62
266405_at	hypothetical protein predicted by ge	---	AT2G38580	-1.31	-0.39	-1.88	0.103344	0.202462	-5.62
248300_at	PP2A regulatory subunit ;supporte	TAP46	AT5G53000	-1.22	-0.28	-1.88	0.103361	0.202478	-5.62
261841_at	BTG1 binding factor 1, putative sim	---	AT1G15920	1.16	0.21	1.88	0.103444	0.202624	-5.62
254117_at	putative protein	---	AT4G24750	-1.23	-0.29	-1.88	0.103515	0.202735	-5.62
247992_at	unknown protein	---	AT5G56520	1.16	0.21	1.88	0.103518	0.202735	-5.62
245620_at	hypothetical protein	---	AT4G14050	1.18	0.24	1.88	0.103544	0.202765	-5.62
261914_at	dirigent protein, putative similar to d	---	AT1G65870	-1.24	-0.31	-1.88	0.103552	0.202765	-5.62
266545_at	hypothetical protein predicted by ge	---	AT2G35290	-1.33	-0.42	-1.88	0.103593	0.202829	-5.63
255491_at	putative zinc finger protein similar t	AtIDD12	AT4G02670	-1.13	-0.17	-1.88	0.103608	0.20283	-5.63
259468_at	unknown protein	TTN10	AT1G19080	1.19	0.25	1.88	0.103611	0.20283	-5.63
264406_at	putative phragmoplastin similar to d	ADL6	AT1G10290	1.18	0.24	1.88	0.103682	0.202951	-5.63

254239_at	water channel - like protein plasma	PIP1;5	AT4G23400	-1.30	-0.38	-1.88	0.103706	0.202981	-5.63
247588_at	protein disulfide isomerase precurs	ATPDIL1-4	AT5G60640	1.17	0.22	1.88	0.103733	0.203015	-5.63
253966_at	fructose-bisphosphate aldolase	---	AT4G26520	-1.22	-0.29	-1.88	0.103781	0.203092	-5.63
262628_at	glucan synthase, putative similar to	ATGSL07	AT1G06490	-1.21	-0.28	-1.88	0.103872	0.203253	-5.63
264004_at	Expressed protein ; supported by fu	---	AT2G22425	-1.19	-0.25	-1.88	0.10394	0.203357	-5.63
249645_at	thionin Thi2.2 ;supported by full-len	THI2.2	AT5G36910	-1.23	-0.30	-1.88	0.103952	0.203357	-5.63
267160_at	putative WD-40 repeat protein	---	AT2G37670	-1.14	-0.18	-1.88	0.103952	0.203357	-5.63
252609_at	putative protein	---	AT3G45110	-1.16	-0.22	-1.88	0.103975	0.203385	-5.63
266333_at	putative ubiquitin activating enzyme	AXL	AT2G32410	1.19	0.25	1.88	0.104023	0.203461	-5.63
253697_at	nucleotide pyrophosphatase -like p	---	AT4G29700	2.24	1.16	1.88	0.104038	0.203472	-5.63
248980_at	putative protein similar to unknown	AtPP2-A7	AT5G45090	-1.21	-0.27	-1.88	0.10411	0.203597	-5.63
251727_at	putative protein	---	AT3G56290	-1.40	-0.49	-1.88	0.104128	0.203615	-5.63
266434_s_a	unknown protein	---	AT2G07120	-1.14	-0.19	-1.88	0.104148	0.203636	-5.63
247133_at	chalcone isomerase-like protein	---	AT5G66220	-1.32	-0.40	-1.88	0.104157	0.203637	-5.63
255500_at	NAD+ ADP-ribosyltransferase	APP	AT4G02390	1.21	0.28	1.88	0.104169	0.203642	-5.63
260791_at	lipase-like protein similar to lipase C	---	AT1G06250	-1.16	-0.21	-1.88	0.104212	0.203708	-5.63
263720_at	putative Na/H antiporter	ATCHX15	AT2G13620	-1.14	-0.19	-1.88	0.104227	0.203708	-5.63
259455_x_a	unknown protein	---	AT1G44030	-1.21	-0.28	-1.88	0.10423	0.203708	-5.63
266999_at	putative HMG protein ; supported by	---	AT2G34450	1.23	0.29	1.88	0.104242	0.203715	-5.63
257033_at	metalloprotease, putative similar to	ATPREP1	AT3G19170	-1.36	-0.44	-1.88	0.104259	0.203731	-5.63
257020_at	mitotic checkpoint protein, putative	---	AT3G19590	-1.18	-0.24	-1.88	0.104303	0.2038	-5.63
260899_at	hypothetical protein predicted by ge	---	AT1G21370	1.20	0.27	1.88	0.104325	0.203825	-5.63
261078_at	unknown protein identical to SP:O5	RPL4	AT1G07320	-1.16	-0.21	-1.88	0.104369	0.203894	-5.63
AFFX-BioDr	E. coli /GEN=bioD /DB_XREF=gb:J	---	---	-1.42	-0.50	-1.88	0.10438	0.203898	-5.63
250754_at	arginine-tRNA-protein transferase 1	ATE1	AT5G05700	-1.15	-0.20	-1.88	0.104447	0.20401	-5.63
246087_at	putative protein predicted protein, A	---	AT5G20580	1.26	0.33	1.88	0.104492	0.204082	-5.63
262257_at	hypothetical protein predicted by ge	---	AT1G53860	-1.17	-0.23	-1.88	0.104519	0.204116	-5.63
246538_at	40S RIBOSOMAL PROTEIN S19 -	---	AT5G15520	1.28	0.36	1.88	0.104532	0.204123	-5.63
247331_at	putative protein contains similarity t	FP3	AT5G63530	1.35	0.43	1.88	0.10454	0.204123	-5.63
246161_at	putative protein predicted proteins,	JAZ12	AT5G20900	-1.24	-0.31	-1.88	0.104574	0.204171	-5.63
256335_at	hypothetical protein predicted by ge	---	AT1G72110	-1.19	-0.25	-1.87	0.104657	0.204316	-5.63
248926_at	Ole e I (main olive allergen)-like pro	---	AT5G45880	-1.32	-0.40	-1.87	0.104777	0.204533	-5.64
267482_s_a	unknown protein	---	AT2G02770	-1.46	-0.55	-1.87	0.104799	0.20455	-5.64
249509_at	strong similarity to unknown protein	---	AT5G38390	-1.13	-0.17	-1.87	0.104804	0.20455	-5.64
267114_at	Expressed protein ; supported by fu	---	AT2G14835	-1.16	-0.21	-1.87	0.104816	0.204553	-5.64

265207_at	unknown protein	---	AT2G36680	-1.14	-0.19	-1.87	0.104823	0.204553	-5.64
262367_at	hypothetical protein contains similar	VPS46.2	AT1G73030	-1.20	-0.26	-1.87	0.104851	0.204589	-5.64
262399_at	unknown protein ;supported by full-	---	AT1G49500	1.25	0.33	1.87	0.10486	0.20459	-5.64
262349_at	unknown protein ;supported by full-	---	AT2G48130	-1.24	-0.31	-1.87	0.104981	0.204808	-5.64
252973_s_a	peptidylprolyl isomerase ROC1 ;su	ROC1	AT4G38740	1.25	0.32	1.87	0.105053	0.204932	-5.64
263984_at	hypothetical protein predicted by ge	---	AT2G42670	-1.16	-0.22	-1.87	0.10509	0.204984	-5.64
255297_x_a	hypothetical protein	---	---	-1.19	-0.25	-1.87	0.105098	0.204984	-5.64
261341_s_a	putative purple acid phosphatase si	PAP5	AT1G52940	-1.12	-0.17	-1.87	0.105136	0.205041	-5.64
250396_at	zinc finger -like protein CCHH finge	---	AT5G10970	-1.41	-0.50	-1.87	0.105169	0.205088	-5.64
253473_at	putative protein mixed-lineage prot	---	AT4G32250	1.33	0.41	1.87	0.105194	0.205118	-5.64
263631_at	unknown protein ; supported by ful	---	AT2G04900	1.14	0.19	1.87	0.105224	0.205159	-5.64
255736_at	unknown protein contains similarity	---	AT1G25380	1.17	0.22	1.87	0.10525	0.205193	-5.64
247350_at	unknown protein	---	AT5G63830	1.17	0.23	1.87	0.105388	0.205444	-5.64
252204_at	putative protein predicted protein, A	---	AT3G50340	-1.53	-0.62	-1.87	0.105419	0.205485	-5.64
263229_s_a	putative polygalacturonase similar t	---	AT1G05650	-1.14	-0.18	-1.87	0.105427	0.205485	-5.64
257544_at	putative DNA-binding protein simila	---	AT3G20880	-1.20	-0.27	-1.87	0.105454	0.20552	-5.64
245795_at	unknown protein contains similarity	---	AT1G32160	1.15	0.20	1.87	0.105486	0.205566	-5.64
258082_at	Expressed protein ; supported by fu	CLE27	AT3G25905	-1.34	-0.42	-1.87	0.105533	0.20562	-5.64
255228_at	F-box protein family, AtFBL21 cont	---	AT4G05470	-1.14	-0.19	-1.87	0.105539	0.20562	-5.64
266878_at	putative RING zinc finger protein	---	AT2G44580	-1.16	-0.22	-1.87	0.105541	0.20562	-5.64
264296_at	protein transport protein sec61 alpl	---	AT1G78720	-1.26	-0.33	-1.87	0.105589	0.205695	-5.64
248931_at	peptide transporter	---	AT5G46040	-1.13	-0.17	-1.87	0.105636	0.20574	-5.64
252612_at	hypothetical protein	---	AT3G45160	-2.15	-1.11	-1.87	0.105639	0.20574	-5.64
261809_at	rac GTPase activating protein, puta	---	AT1G08340	-1.24	-0.31	-1.87	0.105639	0.20574	-5.64
259802_at	thionin identical to GI:1181531 fron	THI2.1	AT1G72260	2.49	1.32	1.87	0.105659	0.205761	-5.64
254086_at	putative proteasome regulatory sub	---	AT4G24820	1.13	0.17	1.87	0.105675	0.205774	-5.64
262758_at	hypothetical protein predicted by ge	---	AT1G10780	1.17	0.23	1.87	0.105725	0.205851	-5.64
245394_at	hypothetical protein ;supported by f	---	AT4G16450	1.22	0.28	1.87	0.105732	0.205851	-5.64
258823_at	putative RING zinc finger protein c	---	AT3G07200	1.22	0.28	1.87	0.105753	0.205873	-5.65
254703_at	putative protein ; supported by full-	---	AT4G17960	1.23	0.30	1.87	0.105787	0.205913	-5.65
253515_at	auxin induced like-protein auxin-ind	---	AT4G31320	-1.21	-0.28	-1.87	0.105791	0.205913	-5.65
254786_at	putative protein gamma-interferon-	---	AT4G12890	-1.17	-0.23	-1.87	0.105822	0.205956	-5.65
264167_at	unknown protein Location of EST g	---	AT1G02060	1.23	0.30	1.87	0.10584	0.205973	-5.65
247781_at	dehydrodolichyl diphosphate synth	---	AT5G58784	-1.22	-0.29	-1.87	0.105853	0.205981	-5.65
253751_at	hypothetical protein ;supported by f	---	AT4G29070	1.33	0.42	1.87	0.105869	0.205994	-5.65

249650_at	Expressed protein ; supported by cl	SEF	AT5G37055	-1.19	-0.25	-1.87	0.105976	0.206186	-5.65
265075_at	hypothetical protein similar to embry	---	AT1G55450	1.74	0.80	1.87	0.106011	0.206235	-5.65
248827_at	calcium sensor protein, calcineurin	CBL9	AT5G47100	1.26	0.33	1.87	0.106088	0.206367	-5.65
263246_at	hypothetical protein predicted by ge	---	AT2G31460	-1.20	-0.26	-1.87	0.10612	0.206413	-5.65
250010_at	AP2-domain DNA-binding protein -	---	AT5G18450	-1.22	-0.29	-1.87	0.106131	0.206415	-5.65
267118_at	hypothetical protein	---	AT2G32590	-1.18	-0.24	-1.86	0.10627	0.206669	-5.65
262976_at	unknown protein	SRS5	AT1G75520	-1.20	-0.27	-1.86	0.106323	0.206755	-5.65
247876_at	unknown protein	---	AT5G57730	1.14	0.19	1.86	0.106348	0.206776	-5.65
260346_at	hypothetical protein predicted by ge	CLE10	AT1G69320	-1.19	-0.26	-1.86	0.106357	0.206776	-5.65
263121_at	receptor protein kinase, putative si	---	AT1G78530	-1.19	-0.25	-1.86	0.106362	0.206776	-5.65
256356_s_a	hypothetical protein	---	AT1G66500	-1.18	-0.24	-1.86	0.106468	0.206967	-5.65
252008_at	hypothetical protein ; supported by	---	AT3G52610	-1.17	-0.22	-1.86	0.106494	0.206998	-5.65
246711_at	putative protein	---	AT5G28190	-1.14	-0.19	-1.86	0.106529	0.207049	-5.65
261235_x_a	hypothetical protein predicted by ge	---	---	-1.14	-0.18	-1.86	0.106572	0.207114	-5.65
246860_at	putative protein various predicted p	---	AT5G25840	1.50	0.58	1.86	0.106673	0.207293	-5.65
258047_at	putative 4-coumarate:CoA ligase 2	4CL2	AT3G21240	1.77	0.82	1.86	0.10674	0.207406	-5.65
267639_at	putative squamosa-promoter bindin	SPL9	AT2G42200	1.14	0.19	1.86	0.10675	0.207408	-5.65
264189_s_a	hypothetical protein similar to acyl-c	ACP3	AT1G54580	-1.22	-0.29	-1.86	0.10685	0.207585	-5.66
265504_at	putative Ta11-like non-LTR retroele	---	---	-1.17	-0.22	-1.86	0.106964	0.207788	-5.66
249428_at	putative protein predicted protein K	---	AT5G39870	-1.12	-0.17	-1.86	0.106979	0.2078	-5.66
260458_at	unknown protein	---	AT1G68250	-1.16	-0.21	-1.86	0.106991	0.207805	-5.66
256189_at	hypothetical protein predicted by ge	---	AT1G30140	-1.14	-0.19	-1.86	0.107041	0.207878	-5.66
250868_at	malate synthase -like protein malat	MLS	AT5G03860	-1.25	-0.32	-1.86	0.107047	0.207878	-5.66
263345_s_a	putative chlorophyll a/b binding prot	LHCB2.1	AT2G05070	-1.47	-0.56	-1.86	0.107066	0.207897	-5.66
247715_at	putative protein predicted protein, A	---	AT5G59360	-1.26	-0.33	-1.86	0.107163	0.208069	-5.66
263718_at	putative CCAAT-box binding trancri	NF-YB7	AT2G13570	-1.19	-0.25	-1.86	0.107225	0.208172	-5.66
260675_at	unknown protein ; supported by cDI	---	AT1G19430	-1.20	-0.27	-1.86	0.107242	0.208187	-5.66
266201_at	similar to MtN3 protein ;supported	---	AT2G39060	-1.17	-0.23	-1.86	0.107255	0.208194	-5.66
252936_at	putative protein transcription initiati	---	AT4G39160	1.26	0.33	1.86	0.107269	0.208204	-5.66
249824_at	putative protein similar to unknown	---	AT5G23380	-1.15	-0.21	-1.86	0.10729	0.208227	-5.66
247841_at	putative protein predicted proteins,	ATFIP1[V]	AT5G58040	1.17	0.23	1.86	0.107304	0.208237	-5.66
266925_at	unknown protein ;supported by full-	PEX11D	AT2G45740	1.14	0.19	1.86	0.107397	0.208398	-5.66
257633_at	hypothetical protein	CYP86C2	AT3G26125	-1.14	-0.19	-1.86	0.107497	0.20857	-5.66
260809_at	hypothetical protein predicted by ge	---	AT1G43730	-1.13	-0.18	-1.86	0.107504	0.20857	-5.66
250782_at	SYN1 splice variant 1 (gb AAF0898	SYN1	AT5G05490	-1.16	-0.22	-1.86	0.107514	0.208573	-5.66

257132_at	hypothetical protein predicted by ge	---	AT3G20230	-1.15	-0.21	-1.86	0.107535	0.208595	-5.66
253965_at	hypothetical protein	---	AT4G26490	1.21	0.28	1.85	0.107697	0.208891	-5.66
247923_at	porin-like protein ;supported by full-	VDAC4	AT5G57490	1.18	0.24	1.85	0.107904	0.209277	-5.66
263125_at	hypothetical protein predicted by ge	---	AT1G78470	-1.15	-0.21	-1.85	0.107938	0.209323	-5.66
247795_at	putative protein zinc finger transcri	---	AT5G58620	1.18	0.24	1.85	0.107965	0.209359	-5.67
254120_at	putative mitochondrial uncoupling p	DIC2	AT4G24570	2.80	1.49	1.85	0.108059	0.209523	-5.67
267387_at	hypothetical protein predicted by ge	---	AT2G44440	-1.12	-0.17	-1.85	0.108091	0.209567	-5.67
248987_at	dimethylaniline monooxygenase (N-	---	AT5G45180	-1.14	-0.19	-1.85	0.108175	0.209706	-5.67
258858_at	putative 40S ribosomal protein S19	---	AT3G02080	1.31	0.39	1.85	0.108181	0.209706	-5.67
255836_at	putative splicing factor U2AF large c	---	AT2G33440	1.23	0.30	1.85	0.10827	0.20986	-5.67
259350_at	putative serine/threonine protein kin	RBK2	AT3G05140	-1.18	-0.24	-1.85	0.108309	0.209917	-5.67
248061_at	putative protein contains similarity t	---	AT5G55340	-1.13	-0.17	-1.85	0.108385	0.210049	-5.67
263358_x_a	hypothetical protein similar to hypot	---	---	-1.17	-0.22	-1.85	0.108405	0.210069	-5.67
266135_at	putative transcription factor IIIB 70 k	---	AT2G45100	-1.28	-0.36	-1.85	0.108556	0.210344	-5.67
250242_at	putative protein similar to unknown	---	AT5G13620	-1.15	-0.20	-1.85	0.108613	0.210435	-5.67
257757_at	hypothetical protein predicted by ge	PGSIP1	AT3G18660	-1.15	-0.20	-1.85	0.108744	0.210672	-5.67
251524_at	3-isopropylmalate dehydratase-like	---	AT3G58980	1.39	0.47	1.85	0.108783	0.21073	-5.67
254502_at	hypothetical protein	PTAC14	AT4G20130	1.19	0.26	1.85	0.108854	0.21085	-5.67
259208_at	hypothetical protein similar to GB:A	---	AT3G09060	1.16	0.21	1.85	0.108875	0.210873	-5.67
248752_at	unknown protein	---	AT5G47600	-1.14	-0.19	-1.85	0.108902	0.210907	-5.67
257896_at	basic chitinase, putative similar to b	---	AT3G16920	-1.70	-0.76	-1.85	0.108949	0.210979	-5.67
257247_at	helix-loop-helix DNA-binding protein	FMA	AT3G24140	1.23	0.30	1.85	0.108961	0.210984	-5.67
248666_at	cyclin C-like protein ;supported by f	---	AT5G48630	-1.21	-0.27	-1.85	0.109011	0.211064	-5.67
263050_at	unknown protein ; supported by cD	ATVPS11	AT2G05170	-1.16	-0.21	-1.85	0.10904	0.211073	-5.67
251226_at	proline-rich protein proline-rich prot	PRP3	AT3G62680	-1.17	-0.22	-1.85	0.10904	0.211073	-5.67
265313_at	unknown protein	---	AT2G20210	1.18	0.24	1.85	0.109044	0.211073	-5.67
248490_at	putative protein similar to unknown	---	AT5G50940	-1.46	-0.54	-1.85	0.10908	0.211125	-5.68
246421_at	TOM (target of myb1) -like protein	---	AT5G16880	-1.19	-0.25	-1.85	0.109111	0.211168	-5.68
261106_at	homeodomain-containing protein H	KNAT7	AT1G62990	-1.14	-0.19	-1.85	0.109123	0.211173	-5.68
263907_at	abscisic acid insensitive 5 (ABI5) co	ABI5	AT2G36270	-1.21	-0.27	-1.85	0.109185	0.211276	-5.68
260681_at	hypothetical protein predicted by ge	AT1G0803	---	1.14	0.19	1.84	0.109267	0.211415	-5.68
245027_at	heme oxygenase 2 (HO2)	HO2	AT2G26550	-1.15	-0.21	-1.84	0.109379	0.211614	-5.68
265589_at	hypothetical protein predicted by ge	---	AT2G20170	-1.17	-0.22	-1.84	0.109423	0.211683	-5.68
263946_at	hypothetical protein ;supported by f	---	AT2G36000	-1.18	-0.24	-1.84	0.10957	0.211948	-5.68
255669_at	predicted protein	MBD3	AT4G00416	-1.12	-0.17	-1.84	0.109652	0.212088	-5.68

256151_at	multispanning membrane protein, p	---	AT1G55130	1.23	0.30	1.84	0.109703	0.212169	-5.68
255950_at	hypothetical protein predicted by ge	---	AT1G22110	-1.22	-0.28	-1.84	0.109719	0.212171	-5.68
245661_at	purine permease, putative similar t	ATPUP3	AT1G28220	-1.18	-0.23	-1.84	0.109723	0.212171	-5.68
257702_at	CTP-synthetase, putative similar to	emb2742	AT3G12670	1.19	0.25	1.84	0.109776	0.212257	-5.68
245804_at	hypothetical protein predicted by ge	---	AT1G46696	-1.13	-0.18	-1.84	0.109806	0.212296	-5.68
253137_at	protein kinase - like protein protein	---	AT4G35500	1.21	0.27	1.84	0.109861	0.212378	-5.68
247344_at	ARI-like RING zinc finger protein-lik	---	AT5G63750	-1.18	-0.24	-1.84	0.109867	0.212378	-5.68
249423_at	Expressed protein ; supported by fu	---	AT5G39785	1.21	0.28	1.84	0.109881	0.212388	-5.68
253528_s_a	putative protein bcop gene product	---	AT4G31480	-1.13	-0.18	-1.84	0.109919	0.212409	-5.68
256428_at	putative disease resistance protein	AtRLP35	AT3G11080	1.30	0.38	1.84	0.10992	0.212409	-5.68
258765_at	putative pectinesterase contains sir	---	AT3G10710	-1.16	-0.21	-1.84	0.10992	0.212409	-5.68
259839_at	peptide transporter, putative simila	---	AT1G52190	-2.25	-1.17	-1.84	0.109951	0.212452	-5.68
256566_at	hypothetical protein	---	AT3G19530	-1.14	-0.19	-1.84	0.109964	0.212458	-5.68
259946_at	putative TINY similar to TINY GB:C	---	AT1G71450	-1.12	-0.16	-1.84	0.110039	0.212585	-5.68
255214_at	hypothetical protein	---	---	-1.13	-0.18	-1.84	0.110184	0.212846	-5.68
246359_x_a	hypothetical protein	---	---	-1.21	-0.27	-1.84	0.110228	0.212914	-5.69
248702_at	unknown protein	---	AT5G48420	-1.14	-0.19	-1.84	0.110256	0.212942	-5.69
263753_at	putative dehydrin ;supported by ful	LEA	AT2G21490	-1.12	-0.16	-1.84	0.110261	0.212942	-5.69
253071_at	putative protein various predicted p	---	AT4G37880	-1.15	-0.20	-1.84	0.110306	0.213011	-5.69
260964_at	E2, ubiquitin-conjugating enzyme 1	ATUBC2-1	AT1G45050	1.13	0.17	1.84	0.110356	0.213074	-5.69
244976_at	cytochrome B6	---	---	1.14	0.19	1.84	0.110358	0.213074	-5.69
262205_at	hypothetical protein predicted by ge	---	AT2G01080	1.17	0.22	1.84	0.110406	0.213149	-5.69
255173_at	putative transposon protein similar	---	---	-1.18	-0.24	-1.84	0.110429	0.213176	-5.69
244988_s_a	ribosomal protein L23	---	---	-1.20	-0.26	-1.84	0.110444	0.213179	-5.69
251404_at	putative protein	---	AT3G60310	-1.17	-0.23	-1.84	0.110449	0.213179	-5.69
247651_at	histone H2A - like protein histone H	HTA6	AT5G59870	-1.81	-0.86	-1.84	0.110474	0.213209	-5.69
250573_at	putative protein	---	AT5G08220	1.19	0.25	1.84	0.11051	0.213261	-5.69
247313_at	3(2),5-bisphosphate nucleotidase	SAL1	AT5G63980	1.24	0.31	1.84	0.110554	0.213318	-5.69
250137_at	rRNA methylase - like protein rRNA	---	AT5G15390	-1.16	-0.22	-1.84	0.110559	0.213318	-5.69
258675_at	putative nonspecific lipid-transfer pr	LTP6	AT3G08770	-1.16	-0.22	-1.84	0.110584	0.213348	-5.69
262856_at	unknown protein contains Pfam pro	---	AT1G14910	-1.19	-0.25	-1.84	0.110633	0.213425	-5.69
249442_at	putative protein ; supported by cDN	---	AT5G39590	1.41	0.50	1.84	0.110717	0.213569	-5.69
248776_at	unknown protein	---	AT5G47900	-1.15	-0.20	-1.84	0.110733	0.213583	-5.69
263478_at	putative receptor-like protein kinase	---	AT2G31880	1.55	0.63	1.84	0.110748	0.213592	-5.69
245254_at	ATP-sulfurylase ; supported by cDN	APS3	AT4G14680	-1.80	-0.85	-1.84	0.110791	0.213658	-5.69

254012_at	putative ribosomal protein ribosom	---	AT4G26230	1.33	0.41	1.84	0.110808	0.213672	-5.69
265933_at	putative esterase contains esterase	---	AT2G19550	-1.18	-0.24	-1.84	0.110818	0.213674	-5.69
260092_at	putative serine carboxypeptidase s	scpl3	AT1G73280	-1.14	-0.18	-1.83	0.110925	0.213861	-5.69
250947_at	putative protein	---	AT5G03450	1.21	0.27	1.83	0.110934	0.213861	-5.69
267389_at	putative beta-glucosidase	BGLU28	AT2G44460	3.73	1.90	1.83	0.110966	0.213898	-5.69
253896_at	putative protein NBS/LRR disease	---	AT4G27190	-1.16	-0.22	-1.83	0.110972	0.213898	-5.69
245115_at	putative esterase D ;supported by f	SFGH	AT2G41530	1.27	0.34	1.83	0.11099	0.213915	-5.69
248686_at	33 kDa secretory protein-like ; supp	---	AT5G48540	1.67	0.74	1.83	0.111012	0.213939	-5.69
247425_at	unknown protein	---	AT5G62550	-1.13	-0.18	-1.83	0.111064	0.214021	-5.69
260381_at	putative kinesin similar to kinesin-C	---	AT1G73860	-1.18	-0.24	-1.83	0.111113	0.214093	-5.69
245473_at	hypothetical protein	---	AT4G16050	1.13	0.18	1.83	0.11112	0.214093	-5.69
263053_at	similar to glucose inhibited division	---	AT2G13440	-1.16	-0.22	-1.83	0.111145	0.214107	-5.69
247269_at	alternative oxidase 2 (sp|O22049)	AOX2	AT5G64210	-1.14	-0.19	-1.83	0.111146	0.214107	-5.69
246291_at	hypothetical protein ; supported by	---	AT3G56820	1.17	0.23	1.83	0.111158	0.214113	-5.69
264445_at	unknown protein similar to ESTs gb	---	AT1G27290	-1.29	-0.37	-1.83	0.111208	0.21419	-5.69
262199_at	unknown protein ; supported by cDN	---	AT1G53800	-1.22	-0.29	-1.83	0.111245	0.214244	-5.69
245818_at	cytochrome b561, putative similar t	---	AT1G26100	-1.32	-0.40	-1.83	0.111263	0.214259	-5.69
248630_at	putative protein strong similarity to u	---	AT5G48990	1.16	0.21	1.83	0.111295	0.214303	-5.69
248462_at	nucleotide-binding protein ; suppor	NBP35	AT5G50960	1.18	0.24	1.83	0.11131	0.214309	-5.69
264943_at	hypothetical protein predicted by ge	---	AT1G76910	-1.20	-0.26	-1.83	0.111316	0.214309	-5.69
248083_at	unknown protein	---	AT5G55420	1.63	0.70	1.83	0.111346	0.214348	-5.69
247914_at	xyloglucan endotransglycosylase	---	AT5G57540	-1.17	-0.23	-1.83	0.111395	0.214423	-5.70
248882_at	cullin ;supported by full-length cDN/	CUL4	AT5G46210	1.13	0.18	1.83	0.111422	0.214458	-5.70
266710_at	putative Ser/Thr protein kinase	---	AT2G46850	-1.16	-0.21	-1.83	0.11146	0.214508	-5.70
245963_at	putative protein HPt phosphotransm	---	AT5G19710	-1.13	-0.17	-1.83	0.111467	0.214508	-5.70
267180_at	unknown protein ; supported by cD	SLT1	AT2G37570	1.16	0.21	1.83	0.111484	0.214522	-5.70
246073_at	ribosomal protein L36-like ribosom	---	AT5G20180	-1.17	-0.23	-1.83	0.111504	0.214543	-5.70
262145_at	unknown protein contains similarity	---	AT1G52730	1.19	0.25	1.83	0.111537	0.214589	-5.70
254997_s_a	putative protein various predicted p	---	AT4G09920 ,	1.14	0.19	1.83	0.11162	0.214708	-5.70
266783_at	putative laccase (diphenol oxidase)	LAC2	AT2G29130	-1.24	-0.31	-1.83	0.111624	0.214708	-5.70
257574_at	hypothetical protein predicted by ge	---	AT3G20710	-1.17	-0.23	-1.83	0.111628	0.214708	-5.70
265061_at	hypothetical protein contains similar	---	AT1G61640	1.41	0.50	1.83	0.111683	0.214796	-5.70
252525_at	putative protein hypothetical protein	---	AT3G46450	-1.16	-0.22	-1.83	0.111748	0.214904	-5.70
258548_at	hypothetical protein	ULP1A	AT3G06910	1.21	0.27	1.83	0.111781	0.214949	-5.70
254962_at	putative glucanase endo-beta-1,4-I	AtGH9C3	AT4G11050	-1.17	-0.22	-1.83	0.111845	0.215054	-5.70

265053_at	myrosinase binding protein, putativ	---	AT1G52000	-1.34	-0.42	-1.83	0.111861	0.215066	-5.70
258562_at	unknown protein ;supported by full-	---	AT3G05980	-1.13	-0.18	-1.83	0.111879	0.215083	-5.70
263577_at	putative protein kinase identical to h	SSP	AT2G17090	-1.19	-0.25	-1.83	0.111933	0.215169	-5.70
246376_at	auxin regulated protein IAA18, puta	IAA18	AT1G51950	-1.22	-0.29	-1.83	0.111966	0.215214	-5.70
245119_at	unknown protein ; supported by cD	---	AT2G41640	1.95	0.97	1.83	0.112019	0.215298	-5.70
253880_at	Expressed protein ;supported by cD	---	AT4G27590	-1.19	-0.25	-1.83	0.112066	0.215366	-5.70
258908_at	putative dehydroquinase shikimate	MEE32	AT3G06350	1.23	0.30	1.83	0.112074	0.215366	-5.70
256211_at	gibberellin 20-oxidase, putative sim	GA2OX7	AT1G50960	-1.13	-0.17	-1.83	0.112123	0.215443	-5.70
264796_at	unknown protein similar to ESTs gb	ZIGA4	AT1G08680	1.16	0.21	1.83	0.112164	0.215495	-5.70
245096_at	putative cysteine proteinase inhibito	ATCYSA	AT2G40880	1.18	0.23	1.83	0.112169	0.215495	-5.70
252762_at	putative protein hypothetical protei	---	AT3G42780	-1.22	-0.29	-1.83	0.112198	0.215532	-5.70
267230_at	unknown protein	ARL	AT2G44080	2.14	1.10	1.83	0.112282	0.215677	-5.70
266952_at	putative gibberellin 2-oxidase	ATGA2OX3	AT2G34555	-1.17	-0.23	-1.82	0.112493	0.216063	-5.70
245416_at	putative protein	---	AT4G17350	-1.19	-0.25	-1.82	0.112525	0.216101	-5.71
248437_at	putative protein similar to unknown	EMF2	AT5G51230	-1.22	-0.29	-1.82	0.112532	0.216101	-5.71
250697_at	putative protein contains similarity t	---	AT5G06800	1.18	0.24	1.82	0.112561	0.216139	-5.71
261100_at	RNA polymerase IIA large subunit,	NRPD1A	AT1G63020	-1.16	-0.22	-1.82	0.112709	0.216405	-5.71
254344_at	alcohol dehydrogenase like protein	---	AT4G22110	-1.26	-0.33	-1.82	0.112823	0.216607	-5.71
264124_at	hypothetical protein predicted by ge	ATOCT2	AT1G79360	-1.17	-0.23	-1.82	0.112844	0.216623	-5.71
250693_at	ubiquitin-specific protease 12 (UBP	UBP12	AT5G06600	1.14	0.19	1.82	0.112851	0.216623	-5.71
250050_at	putative protein predicted protein, A	VAR3	AT5G17790	1.17	0.22	1.82	0.112865	0.216629	-5.71
266697_at	profilin 4 ; supported by full-length c	PRF5	AT2G19770	-1.20	-0.27	-1.82	0.112873	0.216629	-5.71
255750_at	hypothetical protein predicted by ge	---	AT1G31960	-1.15	-0.20	-1.82	0.112886	0.216636	-5.71
256021_at	unknown protein contains Pfam pro	ZW9	AT1G58270	-1.25	-0.32	-1.82	0.11298	0.216798	-5.71
264306_at	unknown protein predicted by gene	---	AT1G78890	1.18	0.24	1.82	0.113023	0.216861	-5.71
246100_at	putative protein predicted protein, A	---	---	-1.15	-0.21	-1.82	0.113064	0.216923	-5.71
248468_at	UDP-glucose:protein transglucosyla	RGP4	AT5G50750	-1.19	-0.25	-1.82	0.113094	0.216962	-5.71
266083_at	hypothetical protein predicted by ge	---	AT2G37820	-1.12	-0.16	-1.82	0.113121	0.216996	-5.71
260841_at	Expressed protein ; supported by fu	---	AT1G29195	1.19	0.25	1.82	0.113144	0.21702	-5.71
263820_at	hypothetical protein predicted by ge	---	---	-1.24	-0.32	-1.82	0.113163	0.217039	-5.71
249091_at	AtCLH2 (gb|AAF27046.1) ; suppor	CLH2	AT5G43860	1.16	0.22	1.82	0.113236	0.217156	-5.71
247243_at	nodulin-like protein	---	AT5G64700	-1.27	-0.34	-1.82	0.113251	0.217156	-5.71
249353_at	oleosin ; supported by cDNA: gi_72	OLEO2	AT5G40420	-1.20	-0.26	-1.82	0.113253	0.217156	-5.71
252465_x_a	putative protein several hypothetica	---	---	-1.15	-0.20	-1.82	0.113262	0.217156	-5.71
256126_at	hypothetical protein similar to MuDR	---	AT1G18050	-1.23	-0.30	-1.82	0.113308	0.217212	-5.71

255646_at	hypothetical protein	---	AT4G00890	-1.16	-0.22	-1.82	0.11331	0.217212	-5.71
250088_at	APG5 (autophagy 5)-like protein	APG5	AT5G17290	1.16	0.21	1.82	0.113331	0.217233	-5.71
255380_at	putative calcium channel ; supporte	ATTPC1	AT4G03560	-1.23	-0.30	-1.82	0.113384	0.217306	-5.71
251720_at	putative protein	---	AT3G56160	-1.34	-0.42	-1.82	0.113388	0.217306	-5.71
266248_at	hypothetical protein predicted by ge	---	AT2G27650	-1.12	-0.16	-1.82	0.113466	0.217438	-5.71
254068_at	putative protein similarity to multidr	NAP8	AT4G25450	1.17	0.22	1.82	0.113492	0.21747	-5.71
245456_at	disease resistance RPP5 like protei	RPP5	AT4G16950	1.19	0.25	1.82	0.11353	0.217523	-5.71
266941_at	peroxidase (ATP22a) identical to G	---	AT2G18980	-1.20	-0.26	-1.82	0.113568	0.217578	-5.71
246237_at	putative protein	---	AT4G36390	1.18	0.24	1.82	0.113594	0.21761	-5.71
250899_at	putative protein ; supported by cDN	---	AT5G03340	1.23	0.30	1.82	0.113677	0.217721	-5.71
262718_at	hypothetical protein similar to putati	---	---	-1.16	-0.21	-1.82	0.113685	0.217721	-5.71
263879_at	hypothetical protein predicted by ge	---	AT2G22030	-1.13	-0.18	-1.82	0.113688	0.217721	-5.71
253730_at	putative protein hypothetical protei	---	AT4G29480	1.29	0.36	1.82	0.113691	0.217721	-5.71
259239_at	putative 40S ribosomal protein s14	---	AT3G11510	1.46	0.55	1.82	0.113714	0.217749	-5.72
255044_at	putative protein	---	AT4G09680	1.17	0.22	1.82	0.113826	0.217944	-5.72
248150_at	kinesin-like protein ; supported by c	ATK3	AT5G54670	-1.41	-0.50	-1.82	0.113837	0.217947	-5.72
259527_at	hypothetical protein	---	AT1G12600	-1.12	-0.17	-1.82	0.113896	0.218041	-5.72
257527_at	hypothetical protein similar to hypo	---	AT3G01880	-1.15	-0.21	-1.82	0.113921	0.218072	-5.72
246382_at	hypothetical protein	---	---	-1.19	-0.25	-1.82	0.113959	0.218125	-5.72
251628_at	translation initiation factor 3 -like pr	EIF3E	AT3G57290	1.18	0.24	1.82	0.114053	0.218287	-5.72
267329_at	hypothetical protein	---	AT2G19250	1.15	0.21	1.81	0.114098	0.218355	-5.72
252652_at	putative chloroplast prephenate deh	ADT4	AT3G44720	1.70	0.77	1.81	0.114162	0.218458	-5.72
251459_at	putative protein hypothetical protei	---	AT3G60200	-1.50	-0.59	-1.81	0.114188	0.218491	-5.72
250033_at	eukaryotic cap-binding protein (gb|A	NCBP	AT5G18110	1.14	0.19	1.81	0.114206	0.218507	-5.72
259031_at	putative transcription factor similar t	---	AT3G09360	1.22	0.28	1.81	0.114223	0.218508	-5.72
246027_at	homoserine dehydrogenase-like pr	---	AT5G21060	-1.26	-0.33	-1.81	0.114226	0.218508	-5.72
265750_x_a	putative Athila retroelement ORF1 p	---	---	-1.25	-0.32	-1.81	0.114239	0.218514	-5.72
252022_at	fructose bisphosphate aldolase - lik	---	AT3G52930	1.24	0.31	1.81	0.114269	0.218553	-5.72
255190_x_a	may be a pseudogene similar to a t	---	---	-1.13	-0.17	-1.81	0.114327	0.218647	-5.72
251188_at	putative protein DESSICATION-RE	---	AT3G62730	-1.16	-0.22	-1.81	0.114353	0.218664	-5.72
254613_at	ankyrin-like protein ankyrin 3, Mus	---	AT4G19150	-1.16	-0.22	-1.81	0.114355	0.218664	-5.72
249129_at	cyclin A-type	CYCA3;1	AT5G43080	-1.17	-0.22	-1.81	0.11438	0.21867	-5.72
264533_s_a	H+/Ca2+ antiporter, putative simila	CAX5	AT1G55720	-1.16	-0.21	-1.81	0.114381	0.21867	-5.72
248663_at	putative protein strong similarity to u	---	AT5G48590	1.12	0.17	1.81	0.114387	0.21867	-5.72
262021_at	unknown protein	AT1G3554	---	1.17	0.23	1.81	0.114439	0.218751	-5.72

253603_at	putative protein SP8 binding protei	WRKY32	AT4G30935	1.13	0.18	1.81	0.114465	0.218783	-5.72
251153_at	putative protein predicted protein, /	NPL41	AT3G63000	1.18	0.24	1.81	0.114504	0.218839	-5.72
256005_at	hypothetical protein similar to putati	---	---	-1.15	-0.20	-1.81	0.114556	0.21891	-5.72
247031_at	UDP-glucuronyltransferase-like pro	---	AT5G67230	-1.23	-0.30	-1.81	0.11456	0.21891	-5.72
251766_at	putative protein PA26, p53 regulate	---	AT3G55890	1.15	0.21	1.81	0.114616	0.218965	-5.72
264462_at	putative transcription factor similar	WLIM1	AT1G10200	1.26	0.33	1.81	0.114624	0.218965	-5.72
257217_at	phosphoenolpyruvate carboxylase (	ATPPC3	AT3G14940	-1.23	-0.30	-1.81	0.114624	0.218965	-5.72
263807_at	putative indole-3-glycerol phosphate	---	AT2G04400	1.92	0.94	1.81	0.114636	0.218965	-5.72
256760_at	SKP1/ASK1 (At15), putative simila	ASK15	AT3G25650	-1.12	-0.16	-1.81	0.114637	0.218965	-5.72
261554_at	PPR-repeat protein contains multipl	---	AT1G63400	1.12	0.16	1.81	0.114683	0.219033	-5.72
261432_at	hypothetical protein predicted by ge	---	AT1G07680	-1.14	-0.19	-1.81	0.114696	0.219041	-5.72
262815_at	putative cytochrome P450 strong si	CYP71A18	AT1G11610	1.32	0.41	1.81	0.114762	0.219129	-5.72
248524_s_a	putative protein contains similarity t	---	AT5G50570	-1.63	-0.71	-1.81	0.114762	0.219129	-5.72
260773_at	gibberellin 2- oxidase identical to gi	ATGA2OX1	AT1G78440	-1.18	-0.24	-1.81	0.114825	0.219231	-5.72
258308_at	putative reverse transcriptase simila	---	---	-1.32	-0.40	-1.81	0.114834	0.219231	-5.72
267495_at	myb-like protein isolog	ETC2	AT2G30420	-1.34	-0.42	-1.81	0.114914	0.219364	-5.73
257448_s_a	putative protein various predicted p	---	AT2G11620	1.15	0.21	1.81	0.115043	0.219592	-5.73
245199_at	Expressed protein ; supported by fu	KCR1	AT1G67730	-1.19	-0.25	-1.81	0.115069	0.219617	-5.73
258398_at	thioredoxin m4 identical to GB:AAF	TRX-M4	AT3G15360	-1.14	-0.18	-1.81	0.115079	0.219617	-5.73
255798_at	unknown protein predicted by gene	---	AT2G33255	1.17	0.23	1.81	0.115085	0.219617	-5.73
257997_at	hypothetical protein similar to hypot	---	AT3G27500	-1.13	-0.18	-1.81	0.115188	0.219796	-5.73
257356_s_a	hypothetical protein predicted by ge	---	AT2G32490	-1.17	-0.22	-1.81	0.115242	0.21988	-5.73
263542_at	putative protein transport protein SE	---	AT2G21630	-1.23	-0.30	-1.81	0.115255	0.219887	-5.73
255934_at	cytochrome P450, putative similar	CYP87A2	AT1G12740	-1.14	-0.19	-1.81	0.115299	0.219953	-5.73
260959_at	unknown protein ;supported by full-	---	AT1G06050	1.15	0.21	1.81	0.115435	0.220193	-5.73
263378_at	protein phosphatase 2C (AthPP2C5	ATHPP2C5	AT2G40180	-1.25	-0.32	-1.81	0.115452	0.220207	-5.73
248493_at	putative protein contains similarity t	FSD2	AT5G51100	1.17	0.23	1.81	0.115493	0.220267	-5.73
251585_at	putative protein hypothetical protei	---	AT3G58630	-1.29	-0.37	-1.81	0.115532	0.220313	-5.73
262315_at	unknown protein ;supported by full-	---	AT1G70990	-1.36	-0.44	-1.81	0.115536	0.220313	-5.73
262677_at	unknown protein	---	AT1G75860	-1.30	-0.38	-1.81	0.115557	0.220334	-5.73
245994_at	putative protein various predicted p	---	---	-1.17	-0.23	-1.81	0.115697	0.220582	-5.73
246587_at	putative protein predicted proteins,	---	---	-1.16	-0.22	-1.81	0.115737	0.220626	-5.73
263013_at	knotted-like homeobox protein, put	KNAT6	AT1G23380	-1.18	-0.24	-1.81	0.115739	0.220626	-5.73
255693_s_a	putative BRCA2 homolog	BRCA2(IV)	AT4G00020	-1.16	-0.22	-1.80	0.115784	0.220693	-5.73
254953_at	Expressed protein ; supported by fu	---	AT4G10925	1.16	0.21	1.80	0.115822	0.220747	-5.73

253713_at	putative protein several hypothetica	---	AT4G29370	-1.14	-0.19	-1.80	0.115919	0.2209	-5.73
247379_at	unknown protein	---	---	-1.21	-0.28	-1.80	0.115929	0.2209	-5.73
249519_at	putative protein similar to unknown	APE1	AT5G38660	-1.20	-0.27	-1.80	0.115931	0.2209	-5.73
245233_at	putative protein similarity to low-ter	---	AT4G25580	-1.17	-0.23	-1.80	0.115995	0.221003	-5.73
257888_at	seed maturation protein, putative s	---	AT3G16990	1.29	0.37	1.80	0.116031	0.221036	-5.73
245832_at	chorismate synthase, putative simi	EMB1144	AT1G48850	1.54	0.63	1.80	0.116032	0.221036	-5.73
261800_at	HD-Zip protein identical to HD-Zip p	PHV	AT1G30490	-1.18	-0.24	-1.80	0.116048	0.221048	-5.73
266989_at	putative myrosinase-binding protein	JAL23	AT2G39330	-2.28	-1.19	-1.80	0.116095	0.22112	-5.74
252264_at	putative protein hypothetical protei	---	AT3G49590	-1.15	-0.20	-1.80	0.116136	0.221179	-5.74
246358_x_a	hypothetical protein	---	AT2G06570	-1.16	-0.21	-1.80	0.11616	0.221205	-5.74
255983_at	polyneuridine aldehyde esterase, p	MES14	AT1G33990	1.16	0.21	1.80	0.116172	0.221211	-5.74
261429_at	hypothetical protein contains simila	WRKY61	AT1G18860	1.15	0.20	1.80	0.116186	0.221219	-5.74
248204_at	myosin heavy chain	ATM2	AT5G54280	-1.20	-0.26	-1.80	0.1162	0.221227	-5.74
266475_at	putative lipase	---	AT2G31100	-1.15	-0.20	-1.80	0.116276	0.221354	-5.74
248000_at	WD-repeat protein-like ;supported	---	AT5G56190	-1.18	-0.23	-1.80	0.116353	0.221481	-5.74
246719_at	homology to many predicted putati	---	---	-1.12	-0.17	-1.80	0.116386	0.221514	-5.74
252746_at	sucrose synthase -like protein SUC	SUS4	AT3G43190	-1.15	-0.21	-1.80	0.11639	0.221514	-5.74
249463_s_a	putative protein predicted proteins,	anac064	AT3G56530	-1.16	-0.21	-1.80	0.116399	0.221514	-5.74
252775_s_a	putative protein various predicted p	---	---	1.15	0.20	1.80	0.116436	0.221556	-5.74
264965_at	hypothetical protein predicted by ge	---	AT1G60560	1.19	0.26	1.80	0.116444	0.221556	-5.74
254075_at	DRE CRT-binding protein DREB1C	CBF2	AT4G25470	1.28	0.36	1.80	0.116451	0.221556	-5.74
256774_at	hypothetical protein predicted by ge	---	AT3G13760	-1.12	-0.17	-1.80	0.116588	0.221799	-5.74
246571_at	putative protein	---	AT5G15000	-1.12	-0.16	-1.80	0.116598	0.221801	-5.74
264547_at	unknown protein ; supported by cDI	CLC-F	AT1G55620	-1.24	-0.31	-1.80	0.116611	0.221802	-5.74
252718_at	putative protein predicted proteins,	---	AT3G43940	-1.17	-0.23	-1.80	0.116618	0.221802	-5.74
251345_at	putative protein hypothetical protein	---	AT3G60940	-1.15	-0.21	-1.80	0.116729	0.221994	-5.74
256588_at	unknown protein	---	AT3G28790	-1.18	-0.24	-1.80	0.116756	0.222027	-5.74
251144_at	anthranilate N-benzoyltransferase	---	AT5G01210	1.33	0.41	1.80	0.116777	0.222049	-5.74
257100_at	disease resistance protein, putative	AtRLP41	AT3G25010	-1.19	-0.25	-1.80	0.116803	0.222074	-5.74
264861_at	putative glucosidase similar to a-gl	---	AT1G24320	-1.13	-0.18	-1.80	0.116811	0.222074	-5.74
258309_at	putative transposase related protein	---	---	-1.14	-0.19	-1.80	0.116823	0.222079	-5.74
258949_at	unknown protein	ATCFM2	AT3G01370	1.21	0.28	1.80	0.116864	0.222139	-5.74
264891_at	putative pectinesterase similar to G	---	AT1G23200	-1.26	-0.33	-1.80	0.116907	0.222202	-5.74
252610_x_a	putative protein putative MuDR pro	---	---	-1.12	-0.16	-1.80	0.11692	0.222209	-5.74
250840_at	putative protein predicted protein, A	---	---	-1.21	-0.28	-1.80	0.116938	0.222221	-5.74

253863_at	putative protein D.melanogaster P	---	AT4G27420	-1.18	-0.24	-1.80	0.116946	0.222221	-5.74
263560_s_a	unknown protein	FUT5	AT2G15350	-1.17	-0.23	-1.80	0.117041	0.222383	-5.74
253166_at	putative protein putative ligand-gat	GLUR2	AT4G35290	1.25	0.33	1.80	0.117078	0.222429	-5.74
248122_at	unknown protein	---	AT5G54700	-1.15	-0.20	-1.80	0.117085	0.222429	-5.74
266755_at	putative alcohol dehydrogenase	---	AT2G47150	-1.17	-0.23	-1.80	0.1171	0.222432	-5.74
246920_at	phytocyanin-related protein-like ph	---	AT5G25090	-1.13	-0.18	-1.80	0.117106	0.222432	-5.74
254787_at	putative protein hypothetical protein	---	AT4G12690	-1.27	-0.35	-1.80	0.117146	0.222489	-5.74
262169_at	hypothetical protein predicted by ge	---	AT1G74860	1.18	0.24	1.80	0.117239	0.222648	-5.74
248036_at	nucleolar protein-like	OLI2	AT5G55920	1.23	0.30	1.80	0.117295	0.222733	-5.74
246801_at	threonyl-tRNA synthetase ; suppor	---	AT5G26830	1.14	0.19	1.80	0.117305	0.222733	-5.74
267367_at	unknown protein ;supported by full-	---	AT2G44210	-1.23	-0.30	-1.80	0.117313	0.222733	-5.75
254517_at	putative protein conserved hypothe	---	AT4G20310	-1.15	-0.20	-1.80	0.117325	0.222738	-5.75
255805_at	zinc-finger - like protein zinc-finger	---	AT4G10240	-1.15	-0.21	-1.80	0.117371	0.222798	-5.75
253376_at	hypothetical protein Arabidopsis th	---	AT4G33290	-1.12	-0.16	-1.80	0.117377	0.222798	-5.75
261556_s_a	unknown protein	---	AT1G62600	-1.21	-0.27	-1.80	0.117391	0.222798	-5.75
249716_at	sterile apetala (emb|CAA11128.1)	SAP	AT5G35770	-1.16	-0.21	-1.80	0.117396	0.222798	-5.75
248034_at	serine threonine-specific protein kin	D6PK	AT5G55910	-1.17	-0.23	-1.80	0.11742	0.222825	-5.75
251069_at	(1-4)-beta-mannan endohydrolase-	---	AT5G01930	-1.38	-0.46	-1.79	0.11745	0.222862	-5.75
258477_at	hypothetical protein predicted by ge	NBS1	AT3G02680	1.13	0.18	1.79	0.117488	0.222902	-5.75
266175_at	NAM (no apical meristem)-like prote	ANAC035	AT2G02450	-1.15	-0.20	-1.79	0.117494	0.222902	-5.75
257155_at	hypothetical protein predicted by ge	---	AT3G27290	-1.19	-0.25	-1.79	0.1175	0.222902	-5.75
264468_at	unknown protein similar to daunoru	---	AT1G10310	1.20	0.26	1.79	0.117509	0.222902	-5.75
257584_at	hypothetical protein similar to putati	---	---	-1.24	-0.32	-1.79	0.117538	0.222937	-5.75
251994_at	protein kinase - like protein kinase,	KIPK	AT3G52890	1.32	0.40	1.79	0.117605	0.223046	-5.75
251102_at	putative transporter protein Na+/H+	CHX27	AT5G01690	-1.24	-0.31	-1.79	0.117696	0.2232	-5.75
246718_at	putative protein various predicted p	---	---	-1.17	-0.22	-1.79	0.117712	0.223211	-5.75
253563_at	putative protein predicted protein, S	---	AT4G31150	1.20	0.27	1.79	0.117737	0.22324	-5.75
255584_at	putative reverse transcriptase-like p	---	---	-1.14	-0.18	-1.79	0.117764	0.223274	-5.75
245750_at	histone H2A, putative similar to his	HTA10	AT1G51060	1.35	0.43	1.79	0.11783	0.223379	-5.75
262652_at	hypothetical protein One of a cluste	FUT9	AT1G14110	-1.14	-0.19	-1.79	0.117867	0.223431	-5.75
258400_at	unknown protein ; supported by cD	---	AT3G15470	-1.20	-0.26	-1.79	0.117906	0.223486	-5.75
262863_at	rhamnosyltransferase, putative sim	---	AT1G64910	-1.16	-0.21	-1.79	0.117976	0.2236	-5.75
265265_at	unknown protein	---	AT2G42900	-1.24	-0.31	-1.79	0.117993	0.223614	-5.75
260614_at	hypothetical protein contains Pfam	---	AT1G53390	1.13	0.17	1.79	0.118011	0.22363	-5.75
260259_at	unknown protein similar to unknown	---	AT1G74300	-1.15	-0.21	-1.79	0.118034	0.223647	-5.75

264769_at	hypothetical protein predicted by ge	---	AT1G61350	1.36	0.45	1.79	0.11804	0.223647	-5.75
263529_at	ADP-ribosylation factor 3	ARF3	AT2G24765	-1.14	-0.19	-1.79	0.118051	0.22365	-5.75
256384_at	hypothetical protein	---	AT1G66660	1.17	0.23	1.79	0.118108	0.223739	-5.75
251467_at	putative protein putative proteins - A	---	AT3G59470	1.19	0.25	1.79	0.118154	0.223807	-5.75
251358_at	shaggy-like kinase beta	---	AT3G61160	1.39	0.48	1.79	0.118213	0.22389	-5.75
246855_at	putative protein various predicted p	---	AT5G26280	1.48	0.56	1.79	0.118217	0.22389	-5.75
249936_at	putative protein similar to unknown	---	AT5G22450	1.18	0.24	1.79	0.118313	0.224052	-5.75
262328_at	hypothetical protein similar to hypot	---	AT1G64100	-1.11	-0.16	-1.79	0.118397	0.224194	-5.75
252314_at	hypothetical protein	---	AT3G49400	1.16	0.22	1.79	0.118424	0.224226	-5.75
262728_at	receptor kinase (CLV1) identical to	CLV1	AT1G75820	-1.30	-0.38	-1.79	0.118435	0.224228	-5.75
260472_at	hypothetical protein predicted by ge	---	AT1G10990	2.29	1.20	1.79	0.118464	0.224256	-5.75
252187_at	putative protein UV inducible prote	---	AT3G50850	-1.18	-0.24	-1.79	0.118469	0.224256	-5.75
249476_at	germin - like protein probable germ	---	AT5G38910	-1.19	-0.25	-1.79	0.118507	0.224308	-5.75
255421_at	putative protein phosphatase regula	---	AT4G03260	1.17	0.23	1.79	0.118527	0.224328	-5.75
251275_at	putative protein anthranilate phosp	---	AT3G61720	-1.25	-0.32	-1.79	0.118578	0.224381	-5.76
252203_at	hypothetical protein	---	AT3G50320	-1.13	-0.18	-1.79	0.118583	0.224381	-5.76
255657_at	acidic ribosomal protein p1 similar t	---	AT4G00810	1.18	0.24	1.79	0.118585	0.224381	-5.76
266113_x_a	hypothetical protein predicted by ge	---	---	-1.18	-0.24	-1.79	0.118621	0.224432	-5.76
261597_at	hypothetical protein predicted by ge	PUB26	AT1G49780	-1.69	-0.76	-1.79	0.118653	0.224472	-5.76
251187_at	putative protein putative proteins - c	AtATG18a	AT3G62770	1.12	0.16	1.79	0.118662	0.224472	-5.76
257098_at	hypothetical protein	---	---	-1.14	-0.19	-1.79	0.118714	0.22455	-5.76
249095_at	myosin heavy chain MYA2 (pir||S51	MYA2	AT5G43900	-1.26	-0.34	-1.79	0.118723	0.22455	-5.76
256299_at	expansin (At-EXP1) identical to exp	ATEXPA1	AT1G69530	-1.87	-0.90	-1.79	0.118769	0.224604	-5.76
251354_at	putative protein hypothetical protei	---	AT3G61090	-1.13	-0.18	-1.79	0.118771	0.224604	-5.76
254196_at	putative protein	---	AT4G24030	-1.20	-0.27	-1.79	0.118783	0.224608	-5.76
249205_at	cycloartenol synthase	MRN1	AT5G42600	-1.28	-0.36	-1.79	0.118803	0.224627	-5.76
258948_at	putative sulfite oxidase similar to su	SOX	AT3G01910	1.13	0.18	1.79	0.118835	0.224669	-5.76
255610_at	putative phosphatidylinositol kinase	ATPIPK10	AT4G01190	-1.15	-0.20	-1.79	0.118856	0.22469	-5.76
263256_at	unknown protein similar to ESTs gb	ATCPISCA	AT1G10500	1.21	0.27	1.79	0.118938	0.224819	-5.76
250262_at	putative protein peptidyl-prolyl cis-t	---	AT5G13410	-1.29	-0.36	-1.79	0.118944	0.224819	-5.76
257449_at	hypothetical protein predicted by ge	---	AT2G31420	-1.16	-0.21	-1.79	0.118978	0.224864	-5.76
254604_at	cadmium-induced protein	---	AT4G19070	-1.17	-0.23	-1.79	0.119051	0.224983	-5.76
248408_at	ripening-related protein-like	---	AT5G51520	-1.13	-0.18	-1.79	0.119064	0.22499	-5.76
248645_at	unknown protein	GEX2	AT5G49150	-1.17	-0.23	-1.79	0.119094	0.225028	-5.76
253943_at	putative protein gene F19K23.12 o	---	AT4G27030	1.50	0.58	1.79	0.11913	0.225032	-5.76

253981_at	putative protein ;supported by full-le	---	AT4G26670	1.14	0.19	1.79	0.11914	0.225032	-5.76
267581_at	putative RING zinc finger protein	---	AT2G41980	-1.28	-0.35	-1.79	0.11914	0.225032	-5.76
248912_at	GDSL-motif lipase/hydrolase-like pr	---	AT5G45670	-1.20	-0.26	-1.79	0.119142	0.225032	-5.76
255978_at	hypothetical protein	---	AT1G34010	-1.14	-0.18	-1.78	0.119146	0.225032	-5.76
245015_at	large subunit of riblose-1,5-bisphos	---	---	1.27	0.34	1.78	0.119188	0.225093	-5.76
245047_at	PSII 32 KDa protein	---	---	1.14	0.19	1.78	0.11923	0.225153	-5.76
257955_at	unknown protein ; supported by cD	---	AT3G21810	1.34	0.42	1.78	0.119266	0.225204	-5.76
256141_at	lysine and histidine specific transpo	---	AT1G48640	-1.12	-0.16	-1.78	0.119318	0.225283	-5.76
257573_at	putative SF16 protein {Helianthus a	iqd9	AT2G33990	-1.23	-0.30	-1.78	0.119357	0.225337	-5.76
267513_at	unknown protein ; supported by cD	---	AT2G45620	1.16	0.21	1.78	0.119402	0.225398	-5.76
267634_at	putative actin	---	AT2G42100	-1.13	-0.17	-1.78	0.119409	0.225398	-5.76
267493_at	unknown protein	OFP2	AT2G30400	-1.25	-0.32	-1.78	0.119433	0.225425	-5.76
250399_at	putative protein predicted proteins,	---	AT5G10750	-1.21	-0.27	-1.78	0.119469	0.225467	-5.76
250834_at	putative protein various predicted p	DME	AT5G04560	-1.21	-0.27	-1.78	0.119475	0.225467	-5.76
265219_at	unknown protein ; supported by ful	---	AT2G02050	1.19	0.25	1.78	0.119508	0.22551	-5.76
256123_at	hypothetical protein predicted by ge	GC2	AT1G18190	-1.12	-0.16	-1.78	0.119568	0.225606	-5.76
262614_at	putative pattern formation protein E	GN	AT1G13980	1.27	0.34	1.78	0.119579	0.225608	-5.76
262031_x_a	hypothetical protein similar to putati	---	---	-1.15	-0.20	-1.78	0.119603	0.225635	-5.76
265875_at	unknown protein ; supported by cD	---	AT2G01690	-1.19	-0.26	-1.78	0.119635	0.225646	-5.76
251401_at	stellacyanin (uclacyanin 3) - like pr	---	AT3G60270	-1.20	-0.26	-1.78	0.119643	0.225646	-5.76
263124_at	beta-Amyrin Synthase, putative sin	---	AT1G78480	1.21	0.28	1.78	0.119643	0.225646	-5.76
246234_at	putative protein	---	AT4G37280	-1.18	-0.23	-1.78	0.119649	0.225646	-5.76
253600_at	SERINE CARBOXYPEPTIDASE II	scpl29	AT4G30810	-1.15	-0.20	-1.78	0.119707	0.225736	-5.76
254521_at	putative protein similar to unknown	---	AT5G44820	1.16	0.21	1.78	0.119729	0.225759	-5.76
248541_at	protein kinase ATN1-like protein	---	AT5G50180	-1.22	-0.28	-1.78	0.11983	0.225931	-5.77
251325_s_a	Lhca2 protein ;supported by full-ler	LHCA2	AT3G61470	-1.11	-0.16	-1.78	0.119861	0.225941	-5.77
254148_at	hsp 70-like protein heat shock 70 p	cpHsc70-1	AT4G24280	1.15	0.21	1.78	0.119864	0.225941	-5.77
254501_at	hypothetical protein	PTAC14	AT4G20130	1.28	0.35	1.78	0.119865	0.225941	-5.77
266611_at	putative auxin-regulated protein	GH3.1	AT2G14960	-1.15	-0.20	-1.78	0.119894	0.225978	-5.77
245996_at	putative protein predicted non-LTR	---	---	-1.14	-0.19	-1.78	0.119922	0.226011	-5.77
266463_at	hypothetical protein predicted by ge	---	AT2G47840	-1.17	-0.23	-1.78	0.119969	0.226064	-5.77
250057_at	putative protein ; supported by full-	---	AT5G17840	-1.13	-0.18	-1.78	0.11997	0.226064	-5.77
254248_at	serine/threonine kinase serine/thre	---	AT4G23270	1.30	0.38	1.78	0.120031	0.22616	-5.77
262917_at	hypothetical protein contains similar	---	AT1G64800	-1.19	-0.25	-1.78	0.120072	0.226203	-5.77
252439_at	pectinesterase-like protein pectines	---	AT3G47400	-1.13	-0.17	-1.78	0.120073	0.226203	-5.77

251738_at	PROBABLE EUKARYOTIC TRANS	EIF3C	AT3G56150	1.14	0.19	1.78	0.1201	0.226235	-5.77
252676_at	putative protein	---	AT3G44280	1.12	0.16	1.78	0.120146	0.226304	-5.77
251989_at	RNA helicase -like protein RNA he	LOS4	AT3G53110	1.17	0.22	1.78	0.120164	0.226319	-5.77
249056_at	putative protein similar to unknown	---	AT5G44470	-1.14	-0.19	-1.78	0.120203	0.226372	-5.77
254561_at	putative protein ; supported by cDN	---	AT4G19160	-1.26	-0.33	-1.78	0.120255	0.226439	-5.77
266687_at	putative arginine N-methyltransfera	PRMT1A	AT2G19670	1.30	0.38	1.78	0.120267	0.226439	-5.77
255524_at	hypothetical protein similar to pectir	ATPMEPC	AT4G02330	-1.95	-0.96	-1.78	0.120277	0.226439	-5.77
262302_at	hypothetical protein predicted by ge	---	AT1G70910	-1.17	-0.22	-1.78	0.120278	0.226439	-5.77
255967_at	protein phosphatase type 2C, puta	---	AT1G22280	-1.19	-0.25	-1.78	0.120309	0.22648	-5.77
247594_at	putative protein farnesylated protei	---	AT5G60800	-1.20	-0.27	-1.78	0.120334	0.226508	-5.77
263990_at	putative phosphoprotein phosphata	PP5.2	AT2G42810	1.15	0.20	1.78	0.120384	0.226572	-5.77
247947_at	auxin transport protein EIR1 (gb|AA	EIR1	AT5G57090	-1.19	-0.25	-1.78	0.120388	0.226572	-5.77
257025_at	hypothetical protein predicted by ge	ATG2	AT3G19190	-1.22	-0.29	-1.78	0.12051	0.226783	-5.77
253799_at	putative DNA-binding protein AP2 d	---	AT4G28140	-2.32	-1.21	-1.78	0.120526	0.226786	-5.77
248180_at	unknown protein ;supported by full-	AGD5	AT5G54310	-1.12	-0.17	-1.78	0.120532	0.226786	-5.77
252041_at	DNA-directed RNA polymerase II 1	NRPB11	AT3G52090	1.15	0.21	1.78	0.120558	0.226816	-5.77
252558_at	actin depolymerising like protein Ac	---	AT3G45990	-1.14	-0.19	-1.78	0.120571	0.226823	-5.77
251827_at	chalcone isomerase ;supported by	TT5	AT3G55120	1.22	0.29	1.78	0.120606	0.22687	-5.77
260450_at	hypothetical protein predicted by ge	---	AT1G72390	-1.23	-0.30	-1.78	0.120628	0.226892	-5.77
262577_at	unknown protein ESTs gb|F20110 a	---	AT1G15290	1.21	0.28	1.78	0.120653	0.22691	-5.77
256363_at	unknown protein ; supported by cDI	---	AT1G66510	-1.31	-0.39	-1.78	0.120666	0.22691	-5.77
263771_at	putative Athila retroelement ORF1 p	---	---	-1.12	-0.16	-1.78	0.120667	0.22691	-5.77
267586_at	hypothetical protein predicted by ge	---	AT2G41950	1.21	0.28	1.78	0.120691	0.226937	-5.77
263188_at	hypothetical protein predicted by ge	---	AT1G36095	-1.16	-0.21	-1.78	0.120744	0.227018	-5.77
262334_at	hypothetical protein similar to putati	---	AT1G64010	-1.16	-0.21	-1.78	0.120758	0.227024	-5.77
254944_at	putative protein proliferation potent	---	AT4G10930	1.11	0.15	1.78	0.120774	0.227037	-5.77
264647_at	putative respiratory burst oxidase p	ATRBOHB	AT1G09090	-1.17	-0.23	-1.78	0.120804	0.227074	-5.77
253265_at	putative protein RING-finger protei	---	AT4G34040	1.13	0.17	1.78	0.120849	0.227139	-5.77
258645_s_a	exopolygalacturonase identical to e	---	AT3G07850	-1.16	-0.21	-1.77	0.120905	0.227227	-5.77
264294_at	hypothetical protein similar to heat s	---	AT1G78750	1.15	0.20	1.77	0.120987	0.227361	-5.77
254320_at	putative protein various predicted p	---	AT4G22580	-1.13	-0.18	-1.77	0.121002	0.227371	-5.77
264962_at	auxin transport protein EIR1, putati	PIN6	AT1G77110	-1.21	-0.28	-1.77	0.121092	0.227515	-5.78
251728_at	cysteine-tRNA ligase-like protein se	---	AT3G56300	-1.15	-0.20	-1.77	0.121099	0.227515	-5.78
246243_at	trichohyalin like protein	---	AT4G36520	-1.13	-0.17	-1.77	0.12112	0.227537	-5.78
266149_x_a	En/Spm-like transposon protein rela	---	---	-1.15	-0.20	-1.77	0.121145	0.227565	-5.78

253003_s_a	putative protein tapetum specific pr	---	AT4G38300 /	1.17	0.23	1.77	0.121222	0.227691	-5.78
256192_at	diadenosine 5,5-P1,P4-tetraphospl	ATNUDX25	AT1G30110	1.17	0.22	1.77	0.121285	0.227775	-5.78
265906_at	hypothetical protein predicted by ge	---	AT2G25565	-1.16	-0.21	-1.77	0.121287	0.227775	-5.78
255299_at	putative adenosine deaminase simi	---	AT4G04880	1.16	0.22	1.77	0.121325	0.227828	-5.78
262304_at	major latex protein (MLP149), puta	MLP43	AT1G70890	-2.06	-1.04	-1.77	0.121379	0.227911	-5.78
246847_at	putative protein ferroportin1, Mus n	ATIREG3	AT5G26820	-1.28	-0.36	-1.77	0.121427	0.227978	-5.78
265926_at	RUB1-conjugating enzyme, putativ	---	AT2G18600	1.24	0.31	1.77	0.121435	0.227978	-5.78
261059_at	transcription factor TINY, putative s	---	AT1G01250	-1.22	-0.28	-1.77	0.121446	0.227979	-5.78
262697_at	beta-glucosidase, putative similar t	ATA27	AT1G75940	-1.13	-0.18	-1.77	0.121476	0.228017	-5.78
264781_at	putative plastid RNA polymerase sig	SIG2	AT1G08540	-1.21	-0.28	-1.77	0.121502	0.228048	-5.78
250766_at	putative protein similar to unknown	---	AT5G05550	-1.18	-0.23	-1.77	0.121518	0.228059	-5.78
258377_at	hypothetical protein contains similar	CNGC19	AT3G17690	-1.17	-0.22	-1.77	0.121538	0.228078	-5.78
261152_at	hypothetical protein predicted by ge	---	AT1G04930	-1.24	-0.31	-1.77	0.121563	0.228105	-5.78
260648_at	unknown protein	---	AT1G08050	1.28	0.36	1.77	0.121616	0.228187	-5.78
263003_at	protein phosphatase 2A 72 kDa reg	---	AT1G54450	-1.12	-0.17	-1.77	0.121674	0.228277	-5.78
257024_at	CDPK-related kinase identical to GI	---	AT3G19100	1.23	0.30	1.77	0.121694	0.228294	-5.78
255463_at	putative polyprotein of LTR transpo	---	---	-1.16	-0.21	-1.77	0.121795	0.228466	-5.78
267113_at	hypothetical protein predicted by ge	---	AT2G14850	1.13	0.17	1.77	0.121834	0.228521	-5.78
262501_at	putative replication factor Similar to	emb1968	AT1G21690	1.17	0.23	1.77	0.121937	0.228695	-5.78
250115_at	salt-inducible protein-like	---	AT5G16420	1.24	0.30	1.77	0.121978	0.228741	-5.78
262479_at	leucine-rich repeat transmembrane	SUB	AT1G11130	-1.13	-0.17	-1.77	0.121982	0.228741	-5.78
251960_at	GTPase AtRAB8 ;supported by full	HEMB1	AT1G69740 /	-1.15	-0.20	-1.77	0.122077	0.2289	-5.78
246472_at	putative protein predicted protein, A	---	AT5G17130	-1.12	-0.16	-1.77	0.122091	0.228908	-5.78
251380_at	putative protein	---	AT3G60700	-1.14	-0.19	-1.77	0.122112	0.228929	-5.78
247785_at	subtilisin-like serine protease conta	---	AT5G58820	-1.12	-0.16	-1.77	0.122129	0.228941	-5.78
257361_at	putative Ta11-like non-LTR retroele	---	---	-1.18	-0.24	-1.77	0.122209	0.229067	-5.78
AFFX-r2-At-	Arabidopsis thaliana /REF=U37281	---	---	1.15	0.20	1.77	0.122216	0.229067	-5.78
260790_at	hypothetical protein predicted by ge	---	AT1G06240	1.25	0.33	1.77	0.122228	0.229071	-5.78
263681_at	hypothetical protein predicted by ge	ORC6	AT1G26840	1.22	0.29	1.77	0.122276	0.229141	-5.78
257969_at	hypothetical protein predicted by ge	---	AT3G27520	1.28	0.35	1.77	0.122345	0.229251	-5.79
266352_at	unknown protein	---	AT2G01610	-1.52	-0.61	-1.77	0.122433	0.229391	-5.79
260046_at	Expressed protein ; supported by cl	---	AT1G73805	-1.80	-0.85	-1.77	0.122439	0.229391	-5.79
249032_at	putative protein contains similarity t	---	AT5G44910	1.81	0.85	1.77	0.122449	0.229391	-5.79
248731_at	phosphoribosylanthranilate transfer	---	AT5G48060	-1.17	-0.23	-1.77	0.122513	0.229491	-5.79
253830_at	Expressed protein ; supported by fu	---	AT4G27652	1.85	0.89	1.77	0.122537	0.229518	-5.79

264203_at	putative 40S ribosomal protein S18	PFL	AT1G22780	1.36	0.44	1.77	0.122554	0.229531	-5.79
249673_at	cytochrome P450-like	CYP79A4P	AT5G35920	-1.11	-0.15	-1.77	0.122605	0.229607	-5.79
256241_at	nascent polypeptide associated co	---	AT3G12390	1.30	0.37	1.76	0.122648	0.229652	-5.79
247724_at	putative protein predicted protein, A	MBD6	AT5G59380	-1.14	-0.19	-1.76	0.122649	0.229652	-5.79
251666_at	CYSTATHIONINE BETA-LYASE PI	CBL	AT3G57050	1.20	0.27	1.76	0.122691	0.229712	-5.79
252205_at	hypothetical protein various predict	---	AT3G50350	-1.20	-0.26	-1.76	0.122741	0.22976	-5.79
256663_at	unknown protein ;supported by full-	---	AT3G12050	-1.25	-0.33	-1.76	0.122745	0.22976	-5.79
262789_at	putative auxin-induced protein stror	---	AT1G10810	-1.13	-0.18	-1.76	0.122747	0.22976	-5.79
259335_s_a	unknown protein similar to unknowr	---	AT3G03930	1.20	0.26	1.76	0.122789	0.229821	-5.79
264213_at	hypothetical protein contains similar	ATPP2-A5	AT1G65390	-2.06	-1.04	-1.76	0.12283	0.229877	-5.79
262130_at	hypothetical protein predicted by ge	---	AT1G02890	1.17	0.22	1.76	0.122894	0.229961	-5.79
257115_at	kinesin-like protein contains Pfam p	---	AT3G20150	-1.15	-0.21	-1.76	0.122895	0.229961	-5.79
262196_at	ubiquitin-fusion protein, putative sir	MUB5	AT1G77870	-1.33	-0.42	-1.76	0.122924	0.229998	-5.79
263926_at	hypothetical protein predicted by ge	ATEME1A	AT2G21800	-1.17	-0.23	-1.76	0.122963	0.230039	-5.79
259418_at	unknown protein ; supported by cDI	GPAT2	AT1G02390	-1.20	-0.27	-1.76	0.122966	0.230039	-5.79
258567_at	apyrase (Atapy1) identical to apyras	ATAPY1	AT3G04080	-1.20	-0.26	-1.76	0.122984	0.230044	-5.79
262587_at	unknown protein Contains PF|0056	---	AT1G15490	-1.29	-0.36	-1.76	0.12299	0.230044	-5.79
246292_at	putative protein hnRNP A/B related	UBA2A	AT3G56860	-1.18	-0.24	-1.76	0.123139	0.230304	-5.79
257296_at	hypothetical protein predicted by ge	---	AT3G28020	-1.15	-0.21	-1.76	0.123185	0.230371	-5.79
263675_x_a	En/Spm-like transposon protein rela	---	---	-1.22	-0.29	-1.76	0.123216	0.230401	-5.79
248440_at	acid phosphatase	---	AT5G51260	-1.18	-0.24	-1.76	0.12322	0.230401	-5.79
249915_at	putative protein similar to unknown	---	AT5G22870	-1.14	-0.18	-1.76	0.123252	0.230441	-5.79
253924_at	putative protein other hypothetical	COBL11	AT4G27110	-1.17	-0.23	-1.76	0.123269	0.230454	-5.79
266931_at	putative non-LTR retroelement reve	---	---	-1.15	-0.20	-1.76	0.123288	0.23047	-5.79
249511_at	putative protein contains similarity t	---	AT5G38500	-1.14	-0.18	-1.76	0.123335	0.230539	-5.79
253877_at	Expressed protein ; supported by cl	---	AT4G27435	-1.34	-0.42	-1.76	0.123365	0.230561	-5.79
264575_at	putative chloroplast 50S ribosomal	emb2394	AT1G05190	1.15	0.20	1.76	0.123372	0.230561	-5.79
245555_at	HSR201 like protein	---	AT4G15390	-1.14	-0.20	-1.76	0.123377	0.230561	-5.79
266050_at	putative SNF2/SWI2 family transcri	---	AT2G40770	-1.13	-0.18	-1.76	0.123442	0.230663	-5.79
252099_at	putative protein senescence-assoc	---	AT3G51250	1.18	0.24	1.76	0.123454	0.230667	-5.79
251857_at	RNA binding protein - like SEB4 pr	---	AT3G54770	-1.15	-0.20	-1.76	0.12347	0.230678	-5.79
261929_at	hypothetical protein similar to axi 1-	---	AT1G22460	-1.17	-0.23	-1.76	0.123491	0.230698	-5.79
264007_at	putative proline-rich protein	ATPRP2	AT2G21140	-2.30	-1.20	-1.76	0.123508	0.230712	-5.79
259308_at	putative nodulin similar to nodulin G	---	AT3G05180	1.28	0.36	1.76	0.123589	0.230818	-5.79
258893_at	putative disease resistance protein	AtRLP33	AT3G05660	-1.65	-0.72	-1.76	0.123593	0.230818	-5.79

250255_at	sigma-like factor (gb|AAC97954.1)	SIG4	AT5G13730	-1.33	-0.41	-1.76	0.123595	0.230818	-5.79
265879_at	hypothetical protein predicted by ge	---	AT2G42450	1.13	0.18	1.76	0.123606	0.230819	-5.80
254264_at	putative protein various predicted p	---	AT4G23510	-1.14	-0.19	-1.76	0.123627	0.230839	-5.80
257678_at	RNA helicase, putative contains Pf	RTL2	AT3G20420	1.18	0.24	1.76	0.123648	0.230852	-5.80
257261_s_a	hypothetical protein contains Pfam	---	AT3G21920	-1.12	-0.16	-1.76	0.123662	0.230852	-5.80
256467_at	copia-type polyprotein, putative sim	---	---	-1.14	-0.19	-1.76	0.123664	0.230852	-5.80
249340_at	putative protein contains similarity t	---	AT5G41140	-1.18	-0.24	-1.76	0.123754	0.231	-5.80
266293_at	putative tropinone reductase ;supp	---	AT2G29360	-1.14	-0.19	-1.76	0.123783	0.23102	-5.80
263546_at	putative dihydrofolate reductase-thy	---	AT2G21550	1.19	0.25	1.76	0.123784	0.23102	-5.80
256448_s_a	unknown protein	MED21	AT4G04780	-1.14	-0.19	-1.76	0.1238	0.23103	-5.80
258331_at	putative coatomer complex subunit	---	AT3G15980	-1.18	-0.23	-1.76	0.123842	0.23109	-5.80
248048_at	nicotianamine synthase	NAS2	AT5G56080	-1.15	-0.21	-1.76	0.123941	0.231255	-5.80
247078_at	unknown protein ; supported by cD	---	AT5G66000	-1.17	-0.22	-1.76	0.123973	0.231296	-5.80
257103_at	hypothetical protein contains Pfam	---	AT3G25060	1.17	0.22	1.76	0.123991	0.23131	-5.80
245315_at	proteasome chain protein ; supporte	PBD2	AT4G14800	1.13	0.18	1.76	0.124086	0.231469	-5.80
254274_at	putative DNA binding protein DNA-	---	AT4G22770	1.22	0.29	1.76	0.124223	0.231705	-5.80
259501_at	unknown protein ; supported by cDI	TPL	AT1G15750	1.18	0.24	1.76	0.124305	0.23184	-5.80
255937_at	transcriptional activator CBF1, puta	DDF1	AT1G12610	-1.34	-0.43	-1.76	0.124338	0.231882	-5.80
258205_at	unknown protein	---	AT3G13965	-1.14	-0.19	-1.76	0.124398	0.23197	-5.80
257082_at	hypothetical protein	COBL10	AT3G20580	-1.14	-0.19	-1.75	0.124414	0.23197	-5.80
262997_at	disease resistance protein, putative	RPP27	AT1G54470	-1.13	-0.17	-1.75	0.124416	0.23197	-5.80
267271_at	hypothetical protein predicted by ge	ATHB21	AT2G02540	-1.14	-0.19	-1.75	0.124492	0.232094	-5.80
257003_at	unknown protein ;supported by full-	FLU	AT3G14110	1.22	0.29	1.75	0.124526	0.232138	-5.80
265107_s_a	similar to glucose 1-dehydrogenase	---	AT1G62610	1.14	0.19	1.75	0.124568	0.232197	-5.80
264534_at	hypothetical protein predicted by ge	---	AT1G55700	-1.21	-0.28	-1.75	0.124595	0.232228	-5.80
265227_s_a	hypothetical protein	---	AT2G07695	-1.18	-0.24	-1.75	0.124632	0.232278	-5.80
254008_at	hypothetical protein various predict	---	AT4G26380	-1.15	-0.20	-1.75	0.124669	0.232303	-5.80
251362_at	DEAD box RNA helicase RH12	---	AT3G61240	1.15	0.21	1.75	0.124674	0.232303	-5.80
265469_at	unknown protein predicted by gens	---	AT2G37160	-1.12	-0.16	-1.75	0.124676	0.232303	-5.80
254907_at	putative disease resistance respon	---	AT4G11190	-1.15	-0.20	-1.75	0.124687	0.232306	-5.80
263423_at	hypothetical protein predicted by ge	---	AT2G31700	-1.14	-0.19	-1.75	0.124726	0.232359	-5.80
264933_at	hypothetical protein predicted by ge	---	AT1G61160	-1.17	-0.22	-1.75	0.124777	0.232435	-5.80
245992_at	receptor protein kinase - like recep	---	AT5G20690	-1.12	-0.16	-1.75	0.124794	0.232444	-5.80
254334_at	predicted protein	---	AT4G22760	1.17	0.23	1.75	0.124802	0.232444	-5.80
258659_at	B' regulatory subunit of PP2A (AtB'	ATB' BETA	AT3G09880	1.15	0.21	1.75	0.124854	0.232521	-5.80

255330_s_a	putative polyprotein	---	---	-1.12	-0.16	-1.75	0.124869	0.23253	-5.80
245293_at	HSP like protein ; supported by cDN	---	AT4G16660	1.54	0.62	1.75	0.124892	0.232555	-5.80
246589_at	glucosyltransferase -like protein glu	---	AT5G14860	-1.15	-0.20	-1.75	0.12491	0.232568	-5.81
259265_at	hypothetical protein predicted by ge	---	AT3G01250	-1.13	-0.17	-1.75	0.125042	0.232796	-5.81
255457_at	putative photosystem I reaction cer	PSAD-1	AT4G02770	-1.12	-0.16	-1.75	0.125063	0.2328	-5.81
251311_at	FUSCA PROTEIN FUS6 ; supporte	FUS6	AT3G61140	1.14	0.19	1.75	0.125065	0.2328	-5.81
248304_at	polypyrimidine tract-binding RNA tra	PTB2	AT5G53180	1.12	0.17	1.75	0.125086	0.232819	-5.81
257106_at	unknown protein	---	AT3G29060	-1.13	-0.18	-1.75	0.125097	0.232822	-5.81
255927_at	transcriptional activator CBF1, puta	---	AT1G12630	-1.28	-0.36	-1.75	0.12511	0.232827	-5.81
251897_at	RING finger - like protein DNA-bind	---	AT3G54360	1.19	0.25	1.75	0.125134	0.232852	-5.81
254154_at	hypothetical protein	---	AT4G24460	-1.20	-0.26	-1.75	0.125157	0.232876	-5.81
261509_at	hypothetical protein predicted by ge	---	AT1G71740	-1.22	-0.29	-1.75	0.125171	0.232883	-5.81
248272_at	Expressed protein ; supported by fu	---	AT5G53480	-1.20	-0.27	-1.75	0.125304	0.233112	-5.81
248728_at	cytochrome P450-like protein	CYP708A2	AT5G48000	-1.16	-0.21	-1.75	0.125381	0.233223	-5.81
244904_at	hypothetical protein	---	---	-1.26	-0.33	-1.75	0.125384	0.233223	-5.81
261798_at	hypothetical protein similar to hypot	CPSF30	AT1G30460	1.17	0.22	1.75	0.125399	0.233231	-5.81
264096_at	Expressed protein ; supported by fu	---	AT1G78995	1.33	0.42	1.75	0.125413	0.233238	-5.81
266232_at	putative phloem-specific lectin	AtPP2-B6	AT2G02310	-1.12	-0.16	-1.75	0.125473	0.23333	-5.81
252040_at	putative protein hypothetical protei	---	AT3G52060	1.37	0.45	1.75	0.125493	0.23333	-5.81
261436_at	protein kinase, putative (fragment)	---	AT1G07870	-1.23	-0.29	-1.75	0.125495	0.23333	-5.81
260494_at	putative receptor-like protein kinase	---	AT2G41820	-1.19	-0.25	-1.75	0.125503	0.23333	-5.81
257640_at	hypothetical protein	---	AT3G25750	-1.19	-0.25	-1.75	0.125553	0.233403	-5.81
249953_at	FAR1 - like protein far-red impaired	FRS12	AT5G18960	1.17	0.23	1.75	0.125573	0.233414	-5.81
255079_s_a	14-3-3 protein GF14chi (grf1) ident	GF14 PHI	AT1G35160	1.13	0.17	1.75	0.125579	0.233414	-5.81
246567_at	alpha-mannosidase -like protein ma	GMII	AT5G14950	1.12	0.17	1.75	0.125698	0.233617	-5.81
249637_at	hypothetical protein	---	AT5G36900	-1.14	-0.19	-1.75	0.125748	0.23369	-5.81
254256_at	serine/threonine kinase -like protei	CRK10	AT4G23180	1.25	0.32	1.75	0.125907	0.233966	-5.81
247777_at	putative protein MNUDC protein, H	---	AT5G58740	1.13	0.17	1.75	0.125991	0.234103	-5.81
249391_at	putative protein predicted proteins,	---	AT5G40140	1.22	0.28	1.74	0.126301	0.234661	-5.82
248494_at	putative protein contains similarity t	AtHSD6	AT5G50770	-1.16	-0.21	-1.74	0.126411	0.234845	-5.82
248672_at	disease resistance protein	---	AT5G48770	-1.17	-0.22	-1.74	0.126485	0.234965	-5.82
248105_at	cell division protein FtsZ chloroplas	FTSZ1-1	AT5G55280	-1.21	-0.27	-1.74	0.126501	0.234974	-5.82
253339_at	metal-transporting P-type ATPase	PAA1	AT4G33520	1.24	0.31	1.74	0.12652	0.234991	-5.82
260716_at	peroxiredoxin identical to SP:O0400	ATPER1	AT1G48130	-1.19	-0.25	-1.74	0.126544	0.235015	-5.82
249508_at	unknown protein	---	AT5G38380	1.14	0.19	1.74	0.126617	0.235133	-5.82

259483_at	hypothetical protein predicted by ge	---	AT1G43980	1.14	0.19	1.74	0.126705	0.235276	-5.82
246039_at	putative protein serine-arginine-rich	---	AT5G19480	1.14	0.18	1.74	0.126723	0.235291	-5.82
246669_at	galactinol synthase, putative	---	AT5G30500	-1.20	-0.26	-1.74	0.126734	0.235292	-5.82
254523_at	putative protein hypothetical protei	FRS1	AT4G19990	-1.23	-0.30	-1.74	0.126759	0.23532	-5.82
247373_at	unknown protein ;supported by full-	---	AT5G63150	1.18	0.24	1.74	0.126782	0.23533	-5.82
256265_at	serine carboxypeptidase, putative c	scpl16	---	-1.19	-0.25	-1.74	0.126785	0.23533	-5.82
249952_at	putative protein predicted proteins,	---	AT5G18950	1.17	0.22	1.74	0.126845	0.235422	-5.82
265465_at	hypothetical protein predicted by ge	---	AT2G37070	-1.40	-0.48	-1.74	0.126904	0.235512	-5.82
259016_at	unknown protein HMM hit: 2Fe-2S i	---	AT3G07480	-1.20	-0.26	-1.74	0.126917	0.235516	-5.82
255761_at	hypothetical protein predicted by ge	---	AT1G16770	-1.15	-0.20	-1.74	0.126968	0.235592	-5.82
246177_at	putative protein predicted proteins -	---	AT5G28420	-1.12	-0.17	-1.74	0.126981	0.235598	-5.82
248592_at	unknown protein ;supported by full-	---	AT5G49280	-1.42	-0.51	-1.74	0.126991	0.235598	-5.82
267541_at	hypothetical protein predicted by ge	---	AT2G32750	-1.18	-0.24	-1.74	0.127082	0.235747	-5.82
255638_at	predicted protein of unknown functi	---	AT4G00740	-1.20	-0.27	-1.74	0.127118	0.235795	-5.82
247559_at	histone deacetylase - like GR AF-1	HDA18	AT5G61070	-1.13	-0.17	-1.74	0.127198	0.235923	-5.82
244911_at	hypothetical protein	---	---	1.16	0.21	1.74	0.12728	0.236039	-5.82
250632_at	putative protein cyclin 2 - Trypanos	CYCP4;3	AT5G07450	-1.18	-0.23	-1.74	0.127281	0.236039	-5.82
252360_at	putative protein predicted protein, A	---	AT3G48480	1.18	0.24	1.74	0.127308	0.23607	-5.82
253271_s_a	SKP1/ASK1 (At11), putative simila	ASK12	AT4G34210	1.19	0.25	1.74	0.127331	0.236093	-5.82
249011_at	putative protein strong similarity to u	---	AT5G44670	-1.38	-0.47	-1.74	0.127347	0.236104	-5.82
258604_at	putative N-acetlytransferase contair	---	AT3G02980	1.18	0.24	1.74	0.12739	0.236165	-5.82
252356_at	putative protein various predicted p	---	---	-1.20	-0.27	-1.74	0.127421	0.236203	-5.82
247055_at	putative protein similar to unknown	---	AT5G66740	-1.18	-0.24	-1.74	0.127528	0.236382	-5.82
251608_at	putative protein hypothetical protei	UVI4-LIKE	AT3G57860	-1.18	-0.24	-1.74	0.12758	0.236458	-5.83
264352_at	unknown protein Strong similarity to	---	AT1G03270	-1.25	-0.32	-1.74	0.127601	0.236473	-5.83
253604_at	hypothetical protein	---	AT4G30970	-1.13	-0.17	-1.74	0.127608	0.236473	-5.83
246438_at	putative protein non-phototropic hy	---	AT5G17580	1.12	0.16	1.74	0.127622	0.236478	-5.83
250755_at	DnaJ-like protein ; supported by cD	---	AT5G05750	-1.18	-0.24	-1.74	0.127674	0.236556	-5.83
256691_at	hypothetical protein predicted by ge	---	---	-1.12	-0.16	-1.74	0.127741	0.236661	-5.83
264478_at	hypothetical protein predicted by ge	---	AT1G77270	-1.13	-0.18	-1.74	0.127762	0.236664	-5.83
267636_at	hypothetical protein predicted by ge	---	AT2G42110	-1.13	-0.18	-1.74	0.127773	0.236664	-5.83
249758_at	putative protein similar to unknown	---	AT5G24350	1.20	0.26	1.74	0.127779	0.236664	-5.83
260802_at	exopolygalacturonase, putative sim	---	AT1G78400	-1.11	-0.16	-1.74	0.127784	0.236664	-5.83
257784_at	geranylgeranylated protein, putativ	MUB4	AT3G26980	-1.53	-0.62	-1.74	0.1278	0.236675	-5.83
258439_at	lipoamide dehydrogenase precurso	mtLPD2	AT3G17240	1.24	0.31	1.74	0.127861	0.236741	-5.83

251219_at	putative protein several hypothetica	---	AT3G62390	-1.31	-0.39	-1.74	0.127874	0.236741	-5.83
255915_at	putative protein	---	AT5G28620	-1.14	-0.19	-1.74	0.127876	0.236741	-5.83
248269_at	putative protein contains similarity t	ACBP1	AT5G53470	1.19	0.25	1.74	0.127877	0.236741	-5.83
251689_at	putative protein motif in hypothetica	---	AT3G56500	1.63	0.70	1.74	0.127894	0.236752	-5.83
260275_at	hypothetical protein predicted by gr	---	AT1G80610	-1.15	-0.20	-1.74	0.127924	0.236788	-5.83
247862_at	similar to unknown protein (sp|P727	---	AT5G58250	1.15	0.20	1.74	0.128032	0.236944	-5.83
252854_at	ubiquitin-specific protease 27 (UBF	UBP27	AT4G39370	1.12	0.17	1.74	0.128034	0.236944	-5.83
254674_at	EREBP - like protein EREBP-1 (Et	---	AT4G18450	-1.12	-0.16	-1.74	0.128039	0.236944	-5.83
255956_at	Expressed protein ; supported by fu	DD46	AT1G22015	-1.16	-0.22	-1.73	0.128054	0.236953	-5.83
250277_at	putative protein DRT100 protein pr	---	AT5G12940	-2.07	-1.05	-1.73	0.128076	0.236972	-5.83
246092_at	glutaredoxin ;supported by full-leng	---	AT5G20500	-1.16	-0.22	-1.73	0.128085	0.236972	-5.83
257877_at	hypothetical protein	---	AT3G17140	-1.15	-0.20	-1.73	0.128108	0.236994	-5.83
267384_at	unknown protein highly similar to G	---	AT2G44370	1.71	0.77	1.73	0.128132	0.237019	-5.83
247228_at	trehalose-6-phosphate phosphatase	---	AT5G65140	-1.56	-0.65	-1.73	0.128213	0.23715	-5.83
260379_at	putative glucosyltransferase similar	UGT89B1	AT1G73880	1.21	0.27	1.73	0.128358	0.2374	-5.83
257039_at	tRNA isopentenyl transferase, puta	ATIPT8	AT3G19160	-1.16	-0.22	-1.73	0.128409	0.237474	-5.83
263080_at	hypothetical protein predicted by ge	---	---	-1.17	-0.23	-1.73	0.128438	0.237508	-5.83
253809_at	putative (1-4)-beta-mannan endohy	---	AT4G28320	-1.21	-0.27	-1.73	0.128466	0.237542	-5.83
250434_at	histone H3 - like protein histone H3	---	AT5G10390	-1.83	-0.87	-1.73	0.128481	0.23755	-5.83
267391_at	putative beta-glucosidase	BGLU17	AT2G44480	1.23	0.30	1.73	0.12857	0.237696	-5.83
256606_at	hypothetical protein	---	AT3G32960	-1.14	-0.19	-1.73	0.128664	0.237841	-5.83
261227_at	proteasome regulatory subunit S3,	EMB2719	AT1G20200	1.13	0.18	1.73	0.128678	0.237841	-5.83
262472_at	hypothetical protein similar to putati	---	AT1G50160	-1.14	-0.19	-1.73	0.12868	0.237841	-5.83
247722_at	GTP-binding protein rab11 - like G	ATRABA2D	AT5G59150	-1.15	-0.20	-1.73	0.128727	0.237897	-5.83
265551_at	putative replication protein A1	---	---	-1.13	-0.18	-1.73	0.128737	0.237897	-5.83
261471_at	hypothetical protein contains similar	---	AT1G14460	-1.26	-0.33	-1.73	0.128742	0.237897	-5.83
252678_s_a	nitrilase 2 ;supported by full-length	NIT2	AT3G44300	1.13	0.18	1.73	0.128772	0.237921	-5.83
257445_x_a	hypothetical protein predicted by ge	---	---	-1.13	-0.18	-1.73	0.128775	0.237921	-5.83
259097_at	unknown protein similar to GB:AAD	---	AT3G04830	1.13	0.18	1.73	0.128887	0.238108	-5.83
245847_at	MYB DNA-binding - like protein my	MYB64	AT5G11050	-1.18	-0.24	-1.73	0.128912	0.238134	-5.84
253760_at	putative protein (fragment) predicte	AT4G2881	---	-1.19	-0.25	-1.73	0.128929	0.238146	-5.84
251735_at	putative protein ferritin 2 precursor	ATFER3	AT3G56090	1.39	0.47	1.73	0.128949	0.238165	-5.84
250639_at	oleosin-like protein oleosin - Brassi	GRP20	AT5G07560	-1.17	-0.23	-1.73	0.129027	0.238289	-5.84
261063_at	transcription factor scarecrow-like	---	AT1G07520	1.65	0.72	1.73	0.129037	0.238289	-5.84
265797_at	Expressed protein ; supported by fu	---	AT2G35710	-1.77	-0.82	-1.73	0.129129	0.238429	-5.84

265587_at	putative pathogenesis-related prote	---	AT2G19980	-1.15	-0.20	-1.73	0.129134	0.238429	-5.84
252519_at	hypothetical protein	---	AT3G46360	-1.15	-0.20	-1.73	0.129178	0.238483	-5.84
252405_at	putative protein RNA helicase HEL	---	AT3G48120	1.18	0.23	1.73	0.129184	0.238483	-5.84
266034_at	Expressed protein ; supported by fu	FIP1	AT2G06005	-1.13	-0.17	-1.73	0.129239	0.238564	-5.84
250785_at	unknown protein	---	AT5G05510	-1.21	-0.27	-1.73	0.129255	0.238576	-5.84
248561_at	unknown protein	---	AT5G49680	-1.21	-0.27	-1.73	0.129266	0.238576	-5.84
267037_at	hypothetical protein ; supported by	---	AT2G38320	-1.16	-0.21	-1.73	0.129459	0.238913	-5.84
248284_at	Expressed protein ; supported by fu	---	AT5G52975	-1.18	-0.24	-1.73	0.129564	0.239072	-5.84
261791_at	unknown protein ; supported by full-	---	AT1G16170	-1.38	-0.46	-1.73	0.129568	0.239072	-5.84
261308_at	protein kinase, putative contains si	RKL1	AT1G48480	-1.75	-0.81	-1.73	0.129577	0.239072	-5.84
255027_at	hypothetical protein F21E10.5 Arab	---	AT4G09880	-1.15	-0.21	-1.73	0.129611	0.239116	-5.84
263564_s_a	DNA-directed RNA polymerase II, t	NRPB3	AT2G15400 ,	1.15	0.20	1.73	0.129626	0.239124	-5.84
248050_at	unknown protein	---	AT5G56100	1.31	0.39	1.73	0.129658	0.239143	-5.84
267197_at	hypothetical protein predicted by ge	---	AT2G30960	-1.14	-0.19	-1.73	0.129662	0.239143	-5.84
246091_at	putative protein various predicted p	---	AT5G20590	1.18	0.24	1.73	0.129668	0.239143	-5.84
250342_at	putative protein predicted protein, A	---	AT5G11870	-1.21	-0.28	-1.73	0.129742	0.239261	-5.84
250240_at	unknown protein	---	AT5G13590	1.23	0.30	1.73	0.129753	0.239261	-5.84
251982_at	pectate lyase -like protein pectate l	---	AT3G53190	-1.22	-0.29	-1.73	0.129773	0.239278	-5.84
260717_at	serine/threonine phosphatase PP7	---	AT1G48120	1.20	0.26	1.73	0.129783	0.239278	-5.84
266680_s_a	hypothetical protein predicted by ge	---	AT2G19850 ,	-1.12	-0.16	-1.73	0.129884	0.239446	-5.84
261396_at	hypothetical protein contains similar	---	AT1G79800	-1.15	-0.21	-1.72	0.129922	0.239496	-5.84
258045_at	ubiquitin-specific protease 7 (UBP7	UBP7	AT3G21280	-1.16	-0.21	-1.72	0.129945	0.239514	-5.84
252221_at	RuvB DNA helicase - like protein D	---	AT3G49830	-1.16	-0.21	-1.72	0.129953	0.239514	-5.84
247852_at	ATGP1 ; supported by cDNA: gi_1	YKT61	AT5G58060	1.14	0.19	1.72	0.129974	0.239515	-5.84
250489_s_a	putative protein predicted proteins,	---	AT5G09710 ,	1.17	0.23	1.72	0.129974	0.239515	-5.84
247492_at	putative protein AP2 domain conta	---	AT5G61890	-1.47	-0.55	-1.72	0.130006	0.239554	-5.84
259847_at	unknown protein	---	AT1G72170	-1.18	-0.23	-1.72	0.130029	0.239577	-5.84
247279_at	arabinogalactan-protein (gb|AAC77	AGP1	AT5G64310	-1.74	-0.80	-1.72	0.130073	0.239638	-5.84
257599_at	60S ribosomal protein, putative sim	---	AT3G24830	1.35	0.43	1.72	0.130154	0.239768	-5.84
261533_at	hypothetical protein predicted by ge	---	AT1G71690	-1.20	-0.26	-1.72	0.13018	0.239797	-5.84
248809_at	GTP-binding protein-like	AtRABA5a	AT5G47520	-1.17	-0.23	-1.72	0.130229	0.239867	-5.84
254590_at	hypothetical protein	---	AT4G18860	-1.14	-0.18	-1.72	0.13027	0.239925	-5.84
247305_at	Expressed protein ; supported by fu	---	AT5G63905	-1.46	-0.54	-1.72	0.130284	0.23993	-5.85
257043_at	receptor-like protein kinase, putativ	IKU2	AT3G19700	-1.15	-0.20	-1.72	0.13032	0.239963	-5.85
246474_at	putative protein predicted protein, A	---	AT5G17150	-1.15	-0.20	-1.72	0.130323	0.239963	-5.85

253993_at	mitogen activated protein kinase kir	MEK1	AT4G26070	-1.29	-0.37	-1.72	0.130341	0.23997	-5.85
251443_at	putative protein unknown protein A	---	AT3G59940	-1.30	-0.38	-1.72	0.130347	0.23997	-5.85
250933_at	arabinogalactan protein - like arabi	FLA11	AT5G03170	-1.37	-0.46	-1.72	0.130426	0.240095	-5.85
254626_at	hypothetical protein ; supported by	---	AT4G18400	1.17	0.22	1.72	0.130459	0.240118	-5.85
266778_at	putative cytochrome P450	CYP707A2	AT2G29090	1.18	0.24	1.72	0.130459	0.240118	-5.85
258021_at	hypothetical protein predicted gene	PUB25	AT3G19380	1.52	0.61	1.72	0.1305	0.240173	-5.85
259386_at	hypothetical protein contains similar	NUB	AT1G13400	-1.16	-0.22	-1.72	0.130566	0.240275	-5.85
245863_s_a	hypothetical protein contains similar	---	AT1G58050	1.17	0.23	1.72	0.130577	0.240276	-5.85
258704_at	putative protein kinase similar to Pt	CCR1	AT3G09780	-1.22	-0.29	-1.72	0.130626	0.240346	-5.85
252407_at	chloroplast NAD-dependent malate	MDH	AT3G47520	1.19	0.26	1.72	0.130679	0.24041	-5.85
252466_x_a	putative protein several hypothetica	---	---	-1.14	-0.19	-1.72	0.130681	0.24041	-5.85
259841_at	unknown protein ; supported by full-	---	AT1G52200	1.20	0.26	1.72	0.13074	0.240492	-5.85
256056_at	hypothetical protein contains similar	---	AT1G07190	-1.12	-0.16	-1.72	0.130752	0.240492	-5.85
257840_at	protein kinase, putative contains Pf	AGC2-1	AT3G25250	1.99	0.99	1.72	0.130764	0.240492	-5.85
260244_at	putative choline kinase similar to GI	---	AT1G74320	-1.15	-0.20	-1.72	0.130783	0.240492	-5.85
265370_s_a	unknown protein related to GB:AAD	---	AT3G30415	-1.13	-0.17	-1.72	0.130785	0.240492	-5.85
266560_at	putative LRR receptor protein kinas	---	AT2G23950	1.18	0.24	1.72	0.130789	0.240492	-5.85
257399_at	putative OBP32pep protein	---	AT1G23690	-1.11	-0.15	-1.72	0.130801	0.240495	-5.85
249776_at	unknown protein	---	AT5G24170	-1.20	-0.27	-1.72	0.130841	0.240548	-5.85
262421_at	hypothetical protein predicted by ge	---	AT1G50290	1.22	0.29	1.72	0.130859	0.240563	-5.85
263773_at	putative xylulose kinase	---	AT2G21370	-1.20	-0.27	-1.72	0.130909	0.240635	-5.85
251465_at	transporter-like protein UDP-galact	UTR6	AT3G59360	-1.12	-0.16	-1.72	0.130982	0.240738	-5.85
258327_at	unknown protein contains similarity	PAP85	AT3G22640	-1.12	-0.17	-1.72	0.130986	0.240738	-5.85
245850_at	ankyrin-repeat containing protein ar	KEG	AT5G13530	-1.21	-0.28	-1.72	0.131112	0.24095	-5.85
256292_at	unknown protein	---	AT1G69430	-1.17	-0.23	-1.72	0.131126	0.240956	-5.85
254195_at	putative protein various predicted p	NLP7	AT4G24020	-1.14	-0.19	-1.72	0.13114	0.240961	-5.85
261026_at	hypothetical protein predicted by ge	---	AT1G01240	-1.19	-0.25	-1.72	0.131184	0.241004	-5.85
264926_at	cytochrome b5, putative similar to c	CB5LP	AT1G60660	1.15	0.20	1.72	0.131184	0.241004	-5.85
258325_at	putative heat shock protein similar t	HSFA6B	AT3G22830	-1.14	-0.19	-1.72	0.131301	0.241199	-5.85
264572_at	unknown protein ESTs gb|AA0424	---	AT1G05320	1.16	0.21	1.72	0.131322	0.241199	-5.85
249142_at	putative protein similar to unknown	---	AT5G43230	-1.16	-0.22	-1.72	0.131322	0.241199	-5.85
257991_at	unknown protein	---	AT3G19870	-1.19	-0.25	-1.72	0.13142	0.241361	-5.85
255273_at	putative protein various predicted p	---	---	-1.12	-0.17	-1.72	0.131558	0.241576	-5.85
253746_at	putative protein hypothetical protei	---	AT4G29100	1.33	0.41	1.72	0.131559	0.241576	-5.85
254700_at	hypothetical protein	---	AT4G18000	-1.16	-0.21	-1.72	0.131623	0.241674	-5.85

258216_at	unknown protein contains Pfam pro	---	AT3G17980	1.19	0.25	1.72	0.131633	0.241674	-5.85
261719_at	hypothetical protein similar to hypot	---	AT1G18390	1.52	0.61	1.72	0.131659	0.241702	-5.86
256216_at	calreticulin (crt1) identical to calretic	CRT1	AT1G56340	1.32	0.40	1.72	0.131674	0.24171	-5.86
245430_at	hypothetical protein	FIP2	AT4G17060	-1.11	-0.15	-1.72	0.131727	0.241788	-5.86
251129_at	putative protein predicted protein, A	---	AT5G01150	-1.15	-0.20	-1.72	0.131759	0.241828	-5.86
245518_at	ATP-dependent RNA helicase like p	ATRH1	AT4G15850	1.26	0.33	1.72	0.131772	0.241832	-5.86
253623_at	GDP-mannose pyrophosphorylase	---	AT4G30570	-1.15	-0.20	-1.71	0.131823	0.241896	-5.86
260957_at	delta 9 desaturase identical to delta	ADS1	AT1G06080	-1.26	-0.34	-1.71	0.131828	0.241896	-5.86
252258_at	putative protein several hypothetica	---	AT3G49450	-1.18	-0.24	-1.71	0.131861	0.241936	-5.86
256759_at	hypothetical protein predicted by ge	---	AT3G25640	-1.20	-0.26	-1.71	0.13195	0.242073	-5.86
258452_at	alternative oxidase 1a precursor ide	AOX1A	AT3G22370	1.56	0.64	1.71	0.131957	0.242073	-5.86
264681_s_a	permease 1-like protein permease	---	AT1G65550	-1.18	-0.24	-1.71	0.132021	0.242171	-5.86
254006_at	putative protein various predicted p	---	AT4G26340	-1.14	-0.19	-1.71	0.132044	0.242195	-5.86
254423_at	Lsd1 like protein zinc-finger protein	LOL2	AT4G21610	1.20	0.26	1.71	0.132147	0.242363	-5.86
249538_at	kanadaptin - like protein unnamed	---	AT5G38840	1.14	0.19	1.71	0.132206	0.242452	-5.86
253821_at	extensin-like protein extensin-like p	---	AT4G28380	-1.15	-0.21	-1.71	0.132305	0.242613	-5.86
252675_at	putative protein hypothetical protein	---	---	-1.14	-0.19	-1.71	0.132323	0.242628	-5.86
256943_at	hypothetical protein similar to hypot	---	AT3G18910	1.13	0.17	1.71	0.132357	0.242671	-5.86
247135_at	auxin-induced protein-like	---	AT5G66260	-1.11	-0.16	-1.71	0.132389	0.24271	-5.86
248290_at	Ca2+-transporting ATPase-like prot	---	AT5G53010	1.17	0.22	1.71	0.132409	0.242727	-5.86
258854_at	putative UDP-glucosyl transferase	---	AT3G02100	-1.16	-0.21	-1.71	0.132443	0.242769	-5.86
250758_at	eukaryotic translation initiation facto	EIF3G2	AT5G06000	1.21	0.28	1.71	0.132464	0.242789	-5.86
246418_at	quinone oxidoreductase -like prote	---	AT5G16960	1.19	0.25	1.71	0.132484	0.242807	-5.86
260698_at	unknown protein contains similarity	---	AT1G32300	-1.12	-0.16	-1.71	0.132561	0.242927	-5.86
258547_at	unknown protein	---	AT3G06895	1.12	0.16	1.71	0.132627	0.243016	-5.86
248215_at	putative protein contains similarity t	---	AT5G53680	-1.22	-0.29	-1.71	0.132637	0.243016	-5.86
254114_at	putative protein (fragment) MtN21 g	---	AT4G24980	-1.19	-0.25	-1.71	0.132648	0.243016	-5.86
261384_at	hypothetical protein predicted by ge	---	AT1G05440	-1.15	-0.20	-1.71	0.132652	0.243016	-5.86
250608_at	pectin methyl-esterase-like protein	---	AT5G07420	-1.13	-0.17	-1.71	0.132691	0.243069	-5.86
262552_at	hypothetical protein similar to hypot	---	AT1G31350	-1.25	-0.33	-1.71	0.132765	0.243185	-5.86
265255_at	unknown protein ; supported by cD	---	AT2G28420	1.27	0.34	1.71	0.132881	0.243377	-5.86
253570_at	hypothetical protein	---	AT4G31260	-1.18	-0.24	-1.71	0.13292	0.24343	-5.86
257357_at	hypothetical protein predicted by ge	---	AT2G41050	-1.14	-0.19	-1.71	0.132954	0.243472	-5.86
261494_at	homeobox transcription factor Hox	HB-1	AT1G28420	1.17	0.22	1.71	0.133015	0.243563	-5.86
265848_at	putative sugar transporter	INT3	AT2G35740	-1.23	-0.30	-1.71	0.133069	0.243643	-5.87

255663_at	hypothetical protein	---	AT4G00420	-1.15	-0.20	-1.71	0.133103	0.243686	-5.87
265915_at	En/Spm-like transposon protein rela	---	---	1.12	0.17	1.71	0.133119	0.243696	-5.87
265691_at	unknown protein predicted by gens	---	AT2G24330	-1.24	-0.31	-1.71	0.13316	0.243751	-5.87
248522_at	predicted protein	---	AT5G50565	1.13	0.18	1.71	0.133201	0.243793	-5.87
254063_at	receptor kinase-like protein recepto	---	AT4G25390	-1.34	-0.43	-1.71	0.133207	0.243793	-5.87
252636_at	putative protein triacylglycerol lipas	---	AT3G44510	1.15	0.21	1.71	0.133215	0.243793	-5.87
248915_at	putative protein strong similarity to u	---	AT5G45690	-1.17	-0.22	-1.71	0.133233	0.243795	-5.87
249655_at	putative helicase PIF1 protein - Ca	---	---	-1.14	-0.18	-1.71	0.133238	0.243795	-5.87
259316_at	unknown protein	---	AT3G01175	1.19	0.25	1.71	0.133249	0.243796	-5.87
260765_at	actin 8 almost identical to actin 8 G	ACT8	AT1G49240	1.35	0.44	1.71	0.133262	0.2438	-5.87
246579_at	hypothetical protein predicted by ge	---	AT1G31620	-1.18	-0.23	-1.71	0.13328	0.243815	-5.87
265331_at	similar to gibberellin-regulated prote	---	AT2G18420	-1.14	-0.19	-1.71	0.133298	0.243819	-5.87
249051_at	berberine bridge enzyme-like protei	---	AT5G44390	1.18	0.24	1.71	0.133304	0.243819	-5.87
256737_at	farnesyltranstransferase, putative (	---	AT3G29420	-1.13	-0.17	-1.71	0.133372	0.243924	-5.87
253646_at	MAP kinase kinase 2 ; supported b	ATMKK2	AT4G29810	-1.26	-0.33	-1.71	0.133457	0.24406	-5.87
245140_at	unknown protein predicted by gene	LBD18	AT2G45420	-1.12	-0.17	-1.71	0.133501	0.244121	-5.87
256812_at	hypothetical protein contains similar	---	AT3G21350	-1.13	-0.17	-1.71	0.133531	0.244142	-5.87
247045_at	putative protein similar to unknown	---	AT5G66930	1.18	0.24	1.71	0.133534	0.244142	-5.87
248900_at	putative protein strong similarity to u	PRP39-2	AT5G46400	1.22	0.28	1.70	0.133758	0.244504	-5.87
259212_at	hypothetical protein predicted by ge	---	AT3G09180	1.16	0.21	1.70	0.133767	0.244504	-5.87
248834_at	unknown protein ;supported by full-	---	AT5G47090	1.14	0.19	1.70	0.13377	0.244504	-5.87
266436_at	putative clathrin binding protein (ep	---	AT2G43160	-1.28	-0.36	-1.70	0.133775	0.244504	-5.87
263898_at	SKP1 interacting partner 6 (SKIP6)	SKIP6	AT2G21950	1.15	0.21	1.70	0.133834	0.244592	-5.87
267333_at	putative WD-40 repeat protein conta	---	AT2G19430	1.13	0.17	1.70	0.133851	0.244592	-5.87
259025_at	hypothetical protein predicted by ge	---	---	-1.19	-0.26	-1.70	0.133855	0.244592	-5.87
253613_at	PR-1-like protein gene PR-1 protei	---	AT4G30320	-1.17	-0.22	-1.70	0.133936	0.24471	-5.87
260776_at	zinc finger protein, putative similar	---	AT1G14580	-1.39	-0.47	-1.70	0.133942	0.24471	-5.87
252211_at	putative protein predicted proteins,	---	AT3G50220	-1.14	-0.19	-1.70	0.133981	0.244762	-5.87
245238_at	putative protein similarity to cytoch	ACYB-2	AT4G25570	-1.18	-0.24	-1.70	0.134037	0.244846	-5.87
250161_at	putative protein amino acid transpo	---	AT5G15240	1.27	0.35	1.70	0.134087	0.244917	-5.87
260406_at	putative glutathione transferase sim	ATGSTU12	AT1G69920	2.45	1.29	1.70	0.134108	0.244936	-5.87
258834_at	GTP cyclohydrolase I similar to GTI	---	AT3G07270	1.18	0.24	1.70	0.13415	0.244993	-5.87
250962_at	putative protein various predicted p	---	AT5G02990	-1.15	-0.20	-1.70	0.134192	0.245051	-5.87
250603_at	putative protein mature parasite-inf	---	AT5G07820	1.16	0.21	1.70	0.134215	0.245072	-5.87
251691_at	putative protein hypothetical protei	---	AT3G56520	-1.17	-0.23	-1.70	0.13427	0.245154	-5.87

259898_at	hypothetical protein predicted by ge	---	AT1G71200	-1.14	-0.19	-1.70	0.134288	0.245166	-5.87
250813_at	GTP-binding protein (gb|AAD09203	TOC34	AT5G05000	1.18	0.24	1.70	0.134324	0.245202	-5.87
252063_at	lipid transfer protein-like protein lipi	LTP12	AT3G51590	-1.15	-0.20	-1.70	0.134339	0.245202	-5.87
245537_at	hypothetical protein	---	AT4G15200	-1.12	-0.17	-1.70	0.13434	0.245202	-5.87
249439_at	thaumatin-like protein thaumatin-lik	---	AT5G40020	-1.31	-0.39	-1.70	0.134392	0.245261	-5.87
253576_at	putative protein various predicted p	---	AT4G31070	1.12	0.17	1.70	0.134398	0.245261	-5.87
262556_at	hypothetical protein	---	AT1G31370	-1.13	-0.17	-1.70	0.134404	0.245261	-5.87
249249_at	unknown protein	---	AT5G42330	1.16	0.21	1.70	0.134428	0.245285	-5.87
260158_at	unknown protein	---	AT1G79910	-1.16	-0.21	-1.70	0.134446	0.245298	-5.87
245767_at	hypothetical protein similar to putati	---	AT1G33610	-1.11	-0.15	-1.70	0.134466	0.245314	-5.87
257485_at	hypothetical protein contains Pfam	---	AT1G63580	-1.32	-0.40	-1.70	0.134519	0.24539	-5.88
262443_at	Expressed protein ; supported by fu	---	AT1G47655	-1.21	-0.27	-1.70	0.134532	0.24539	-5.88
264727_at	putative cytochrome C strong simila	CYTC-1	AT1G22840	1.34	0.43	1.70	0.13454	0.24539	-5.88
247568_at	putative protein predicted proteins,	---	AT5G61260	1.32	0.40	1.70	0.134572	0.245429	-5.88
254670_at	teosinte branched1 - like protein te	---	AT4G18390	1.26	0.34	1.70	0.134615	0.245471	-5.88
254568_x_a	hypothtetical protein	---	---	-1.17	-0.23	-1.70	0.134617	0.245471	-5.88
253075_at	putative disease resistance protein	---	AT4G36150	1.13	0.17	1.70	0.134642	0.245491	-5.88
245670_at	mitochondrial protein (AtJ1), putati	ATJ1	AT1G28210	1.30	0.38	1.70	0.134649	0.245491	-5.88
251417_at	putative protein prib5, Ribes nigrun	AT3G6042(	---	-1.12	-0.16	-1.70	0.134725	0.24561	-5.88
259969_at	putative pyrophosphate-dependent	---	AT1G76550	1.35	0.43	1.70	0.134748	0.245632	-5.88
250475_at	sulfate transporter ; supported by c	SULTR2;1	AT5G10180	-1.41	-0.50	-1.70	0.134762	0.245638	-5.88
266955_at	mitochondrial ribosomal protein S14	RPS14	AT2G34520	1.17	0.23	1.70	0.134909	0.245887	-5.88
266714_at	NAM (no apical meristem)-like prote	EMB2301	AT2G46770	-1.15	-0.20	-1.70	0.134951	0.245943	-5.88
257347_at	pseudogene, gene_id:K17E7.6	---	---	-1.12	-0.17	-1.70	0.134965	0.245946	-5.88
260339_at	putative serine/threonine kinase sin	SIK1	AT1G69220	-1.21	-0.28	-1.70	0.134974	0.245946	-5.88
253667_at	peroxidase ATP8a ;supported by fu	---	AT4G30170	-1.14	-0.18	-1.70	0.135006	0.245985	-5.88
264785_at	putative non-LTR retroelement reve	---	---	-1.15	-0.20	-1.70	0.135195	0.246309	-5.88
265612_at	hypothetical protein predicted by ge	---	AT2G25390 ,	1.13	0.18	1.70	0.13525	0.24639	-5.88
259282_at	unknown protein similar to unknowr	GPAT5	AT3G11430	-1.20	-0.26	-1.70	0.135354	0.24656	-5.88
248791_at	palmitoyl-protein thioesterase precu	---	AT5G47350	-1.11	-0.16	-1.70	0.135447	0.246697	-5.88
246019_at	putative protein predicted proteins,	---	AT5G10690	-1.19	-0.25	-1.70	0.135451	0.246697	-5.88
255496_at	putative protein similar to plant nucl	BZO2H1	AT4G02640	1.21	0.27	1.69	0.135717	0.247162	-5.88
248114_at	wax synthase-like protein	---	AT5G55370	-1.15	-0.20	-1.69	0.135731	0.247168	-5.88
262212_at	response regulator 7, putative simi	ARR15	AT1G74890	-1.20	-0.26	-1.69	0.135776	0.247231	-5.88
250130_at	amylogenin; reversibly glycosylatab	---	AT5G16510	-1.20	-0.27	-1.69	0.135915	0.247464	-5.89

249707_at	putative protein similar to unknown	---	AT5G35640	-1.22	-0.28	-1.69	0.135934	0.247477	-5.89
261210_at	vacuolar ATP synthase subunit C,	DET3	AT1G12840	-1.12	-0.16	-1.69	0.136002	0.24757	-5.89
261599_at	hypothetical protein similar to hypot	---	AT1G49700	-1.15	-0.21	-1.69	0.136006	0.24757	-5.89
248055_at	serine/threonine-specific kinase like	---	AT5G55830	-1.15	-0.20	-1.69	0.136024	0.247583	-5.89
254618_at	cellulose synthase - like protein cel	IRX1	AT4G18780	-1.27	-0.34	-1.69	0.136056	0.247616	-5.89
246393_at	phosphoglycerate kinase, putative	---	AT1G58150	-1.15	-0.20	-1.69	0.136064	0.247616	-5.89
257821_at	Expressed protein ; supported by fu	RALFL26	AT3G25170	-1.12	-0.16	-1.69	0.136079	0.247624	-5.89
266236_at	putative glutathione S-transferase	ATGSTZ2	AT2G02380	-1.13	-0.18	-1.69	0.136118	0.247674	-5.89
254318_at	putative protein embryo-abundant	---	AT4G22530	1.65	0.73	1.69	0.13615	0.247699	-5.89
257643_at	AP2 domain transcription factor co	---	AT3G25730	1.43	0.51	1.69	0.136173	0.247699	-5.89
257614_at	putative non-LTR retroelement reve	---	---	-1.17	-0.23	-1.69	0.136176	0.247699	-5.89
252395_at	H+-transporting ATPase - like prote	AHA4	AT3G47950	1.23	0.30	1.69	0.136182	0.247699	-5.89
248331_at	putative protein contains similarity t	---	AT5G52650	1.27	0.34	1.69	0.136193	0.247699	-5.89
260281_at	unknown protein	---	AT1G80500	-1.17	-0.22	-1.69	0.136197	0.247699	-5.89
246293_at	SigA binding protein ; supported by	SIB1	AT3G56710	1.82	0.87	1.69	0.136223	0.247727	-5.89
249630_s_a	sen1-like protein SEN1 - Saccharo	---	AT5G37150	-1.11	-0.15	-1.69	0.136272	0.247797	-5.89
265967_at	nodulin-like protein ; supported by	---	AT2G37450	-1.21	-0.27	-1.69	0.136421	0.248029	-5.89
258069_at	hypothetical protein predicted by ge	---	AT3G26010	-1.20	-0.26	-1.69	0.136421	0.248029	-5.89
254666_at	translation initiation factor eIF-2 ga	---	AT4G18330	-1.11	-0.15	-1.69	0.136455	0.248071	-5.89
254436_at	putative protein receptor kinase-lik	---	AT4G20940	-1.12	-0.17	-1.69	0.136473	0.248083	-5.89
262226_at	Expressed protein ; supported by fu	---	AT1G53885	-1.44	-0.53	-1.69	0.136545	0.248194	-5.89
247615_at	putative protein various predicted p	---	AT5G60250	-1.12	-0.16	-1.69	0.136663	0.24839	-5.89
267357_at	putative nematode-resistance prote	HSPRO2	AT2G40000	-1.41	-0.50	-1.69	0.136708	0.248451	-5.89
254715_at	putative protein hypothetical protei	---	AT4G13550	1.22	0.29	1.69	0.136905	0.248774	-5.89
263220_at	hypothetical protein predicted by ge	emb2279	AT1G30610	1.19	0.25	1.69	0.13691	0.248774	-5.89
264714_at	receptor-like protein kinase, putativ	---	AT1G69990	-1.16	-0.21	-1.69	0.136918	0.248774	-5.89
253133_at	DNA-directed RNA polymerase (EC	NRPB1	AT4G35800	1.15	0.20	1.69	0.137	0.248902	-5.89
266805_at	hypothetical protein ;supported by	---	AT2G30010	-2.26	-1.18	-1.69	0.137073	0.249015	-5.89
260121_at	AIG1-like protein similar to AIG1 pr	---	AT1G33910	-1.11	-0.15	-1.69	0.137117	0.249076	-5.89
262473_at	chloroplast FtsH protease almost id	FTSH1	AT1G50250	-1.19	-0.25	-1.69	0.137199	0.249199	-5.89
252924_at	putative zinc finger protein salt-tole	---	AT4G39070	-1.42	-0.50	-1.69	0.137207	0.249199	-5.89
250957_at	photoreceptor-interacting protein -	---	AT5G03250	1.12	0.16	1.69	0.137253	0.249252	-5.89
254103_at	putative protein ;supported by full-l	---	AT4G25030	1.41	0.50	1.69	0.137258	0.249252	-5.89
246761_at	embryonic abundant protein - like e	---	AT5G27980	-1.13	-0.18	-1.69	0.137317	0.249339	-5.89
246937_at	phospholipase D1-like protein phos	PLDALPHA	AT5G25370	-1.13	-0.18	-1.69	0.137331	0.249345	-5.89

248619_at	amino acid permease 6 (emb CAA6	AAP6	AT5G49630	-1.20	-0.27	-1.69	0.137379	0.249412	-5.90
254629_at	Expressed protein ; supported by fu	---	AT4G18425	-1.19	-0.26	-1.69	0.137406	0.249442	-5.90
246695_at	putative protein	---	---	-1.19	-0.25	-1.69	0.137502	0.249596	-5.90
248275_at	isp4 protein	ATOPT8	AT5G53520	-1.12	-0.16	-1.69	0.137518	0.249605	-5.90
262387_s_a	hypothetical protein contains similar	---	AT1G49340 ,	1.14	0.19	1.69	0.137562	0.249665	-5.90
254620_at	pollen-specific protein - like 18.3K p	---	AT4G18590	1.15	0.20	1.68	0.137655	0.249814	-5.90
250922_at	Expressed protein ; supported by fu	---	AT5G03345	-1.14	-0.19	-1.68	0.137701	0.249877	-5.90
258686_at	putative polygalacturonase similar	---	AT3G07840	-1.12	-0.17	-1.68	0.137754	0.249954	-5.90
AFFX-BioB-	E. coli /GEN=bioB /DB_XREF=gb:J	---	---	-1.26	-0.33	-1.68	0.137806	0.249992	-5.90
263845_at	phenylalanine ammonia lyase (PAL	pal1	AT2G37040	1.60	0.68	1.68	0.137807	0.249992	-5.90
253217_at	actin depolymerizing factor - like pr	ADF9	AT4G34970	-1.37	-0.45	-1.68	0.137818	0.249992	-5.90
260750_at	light repressible receptor protein ki	---	AT1G49100	-1.14	-0.19	-1.68	0.137819	0.249992	-5.90
265932_at	hypothetical protein predicted by gr	LBD8	AT2G19510	-1.11	-0.15	-1.68	0.13783	0.249993	-5.90
262301_at	ripening-induced protein, putative s	---	AT1G70880	-1.15	-0.21	-1.68	0.137867	0.25004	-5.90
258955_s_a	unknown protein ;supported by full-	---	AT3G01450 ,	-1.19	-0.26	-1.68	0.137915	0.250107	-5.90
260392_at	putative enolase similar to ENOLAS	---	AT1G74030	2.04	1.03	1.68	0.137927	0.250109	-5.90
256822_at	unknown protein contains PF00612	IQD5	AT3G22190	-1.16	-0.22	-1.68	0.137982	0.250188	-5.90
253786_at	receptor protein kinase-like protein	---	AT4G28650	-1.15	-0.20	-1.68	0.138014	0.250227	-5.90
255498_at	putative vacuolar ATPase similar to	---	AT4G02620	-1.10	-0.14	-1.68	0.138094	0.250351	-5.90
248118_at	GDSL-motif lipase/hydrolase-like pr	---	AT5G55050	-1.94	-0.96	-1.68	0.138199	0.250515	-5.90
258518_at	unknown protein ; supported by cD	---	AT3G06570	1.12	0.17	1.68	0.138206	0.250515	-5.90
247219_at	COP1-interacting protein CIP8 ; su	CIP8	AT5G64920	-1.15	-0.20	-1.68	0.138261	0.250594	-5.90
251298_at	hypothetical protein contains non-co	---	AT3G62040	1.33	0.41	1.68	0.138313	0.250669	-5.90
246492_at	CRS2-like protein CRS2 - Zea may	---	AT5G16140	-1.22	-0.28	-1.68	0.138347	0.250711	-5.90
259845_at	auxin transporter splice variant b, p	PIN1	AT1G73590	-1.22	-0.29	-1.68	0.138385	0.250746	-5.90
261687_at	hypothetical protein predicted by ge	---	AT1G47280	-1.13	-0.18	-1.68	0.138389	0.250746	-5.90
257041_at	unknown protein	---	AT3G28550	-1.20	-0.26	-1.68	0.1384	0.250746	-5.90
256416_at	ferritin subunit, putative similar to fe	ATFER2	AT3G11050	-1.17	-0.23	-1.68	0.138418	0.25076	-5.90
250223_at	glutaredoxin-like protein ;supported	ROXY2	AT5G14070	-1.13	-0.18	-1.68	0.13848	0.250853	-5.90
262675_at	anter-specific proline-rich -like prote	EXL6	AT1G75930	-1.16	-0.21	-1.68	0.138519	0.250887	-5.90
251223_at	putative transcription factor MYB11	ATMYB11	AT3G62610	-1.12	-0.16	-1.68	0.138521	0.250887	-5.90
259147_at	putative RNA binding protein simila	APUM4	AT3G10360	-1.25	-0.33	-1.68	0.138555	0.250918	-5.90
251549_at	putative protein predicted proteins,	---	AT3G58890	-1.13	-0.18	-1.68	0.138561	0.250918	-5.90
267056_at	hypothetical protein predicted by ge	---	AT2G32470	1.19	0.25	1.68	0.138699	0.251149	-5.90
262091_at	Myb-family transcription factor, puta	MYB72	AT1G56160	-1.13	-0.18	-1.68	0.138796	0.25128	-5.90

259935_at	putative GDSL-motif lipase/acylhyd	---	AT1G71250	-1.17	-0.22	-1.68	0.138802	0.25128	-5.90
266494_at	putative receptor-like protein kinase	PRK2A	AT2G07040	-1.13	-0.18	-1.68	0.138805	0.25128	-5.90
257409_at	unknown protein predicted by gens	---	AT2G17470	-1.16	-0.22	-1.68	0.138829	0.251295	-5.91
253565_at	predicted protein DAN26, Homo sa	---	AT4G31200	1.18	0.24	1.68	0.138835	0.251295	-5.91
AFFX-r2-Ec	E. coli /GEN=bioC /DB_XREF=gb:J	---	---	-1.34	-0.42	-1.68	0.138854	0.251302	-5.91
267399_at	hypothetical protein predicted by ge	---	AT2G44195	-1.11	-0.16	-1.68	0.13886	0.251302	-5.91
263318_at	Expressed protein ; supported by fu	AtGDU4	AT2G24762	-1.81	-0.85	-1.68	0.138889	0.251333	-5.91
257183_at	ABC transporter contains Pfam prot	---	AT3G13220	-1.14	-0.19	-1.68	0.138903	0.251339	-5.91
263654_at	unknown protein	---	AT1G04300	1.14	0.19	1.68	0.138922	0.251342	-5.91
256496_at	hypothetical protein similar to hypot	---	AT1G31510	-1.15	-0.20	-1.68	0.138927	0.251342	-5.91
261805_at	hypothetical protein almost identica	---	AT1G30540	-1.15	-0.20	-1.68	0.138979	0.251398	-5.91
256763_at	unknown protein	COBL8	AT3G16860	-1.54	-0.62	-1.68	0.13898	0.251398	-5.91
259904_at	hypothetical protein predicted by ge	---	AT1G74150	-1.17	-0.22	-1.68	0.138998	0.25141	-5.91
262471_at	hypothetical protein similar to hypot	---	AT1G50140	-1.17	-0.23	-1.68	0.139036	0.25146	-5.91
260647_at	hypothetical protein predicted by ge	---	AT1G08030	1.17	0.23	1.68	0.139078	0.251516	-5.91
259740_at	phytochrome, putative similar to GI	---	AT1G64300	1.22	0.28	1.68	0.13922	0.251737	-5.91
263903_at	putative touch-induced calmodulin	---	AT2G36180	-1.12	-0.17	-1.68	0.139222	0.251737	-5.91
246007_at	Expressed protein ; supported by fu	FTRA2	AT5G08410	1.17	0.23	1.68	0.139328	0.251895	-5.91
255869_at	unknown protein	---	AT2G30270	-1.16	-0.21	-1.68	0.139332	0.251895	-5.91
259641_at	putative protease similar to proteas	---	AT1G69020	-1.18	-0.24	-1.68	0.139414	0.252023	-5.91
260818_at	unknown protein	---	AT1G06890	1.11	0.15	1.68	0.139453	0.252075	-5.91
261713_at	protein kinase, putative identical to	MYC2	AT1G32640	1.25	0.32	1.68	0.139482	0.252106	-5.91
253752_at	putative protein ;supported by full-l	---	AT4G28910	-1.18	-0.24	-1.68	0.139519	0.252154	-5.91
255754_at	auxin-induced protein, putative sim	---	AT1G43040	-1.11	-0.15	-1.67	0.139612	0.252301	-5.91
253433_s_a	nodulin - like protein nodulin (clone	---	AT4G28365 ,	-1.17	-0.23	-1.67	0.139645	0.252339	-5.91
265231_s_a	hypothetical protein	---	AT2G07713	-1.14	-0.18	-1.67	0.139655	0.252339	-5.91
248125_at	2S storage protein-like	---	AT5G54740	-1.15	-0.20	-1.67	0.139697	0.252395	-5.91
251619_at	putative protein hypothetical protei	---	AT3G58050	-1.11	-0.15	-1.67	0.13978	0.252525	-5.91
260170_at	putative sucrose transport protein s	SUC5	AT1G71890	-1.22	-0.28	-1.67	0.139797	0.252532	-5.91
247168_at	putative protein contains similarity t	---	AT5G65860	1.18	0.24	1.67	0.139806	0.252532	-5.91
262813_at	unknown protein strong similarity to	---	AT1G11670	-1.48	-0.56	-1.67	0.139906	0.252692	-5.91
250322_at	putative transcription factor (MYB46	MYB46	AT5G12870	-1.11	-0.16	-1.67	0.140024	0.252886	-5.91
262945_at	unknown protein ;supported by full-	---	AT1G79510	1.29	0.36	1.67	0.140103	0.253009	-5.91
259720_at	hypothetical protein predicted by ge	---	AT1G61080	-1.16	-0.22	-1.67	0.140167	0.253104	-5.91
254313_at	extensin like protein extensin like p	---	AT4G22460	-1.17	-0.22	-1.67	0.140272	0.253273	-5.91

245867_at	ATP phosphoribosyl transferase ide	ATATP-PR	AT1G58080	1.16	0.21	1.67	0.140287	0.25328	-5.91
250289_at	putative protein ;supported by full-l	---	AT5G13190	-1.37	-0.46	-1.67	0.140393	0.253451	-5.92
251230_at	beta-glucosidase-like protein severa	BGLU8	AT3G62750	1.12	0.16	1.67	0.140473	0.253577	-5.92
262806_at	putative pyrophosphate-dependent	---	AT1G20950	1.23	0.30	1.67	0.140524	0.253648	-5.92
251064_at	putative zinc finger protein several z	---	AT5G01860	-1.15	-0.20	-1.67	0.140551	0.253662	-5.92
252505_at	dehydrogenase -like protein alcoho	---	AT3G46170	-1.16	-0.21	-1.67	0.14056	0.253662	-5.92
260318_at	hypothetical protein predicted by ge	---	AT1G63960	-1.17	-0.23	-1.67	0.140565	0.253662	-5.92
252349_at	cell division cycle protein 23 homol	APC8	AT3G48150	1.24	0.31	1.67	0.14059	0.253687	-5.92
260922_at	hypothetical protein predicted by ge	---	AT1G21560	1.14	0.19	1.67	0.140669	0.253803	-5.92
267588_at	unknown protein	---	AT2G42060	-1.12	-0.17	-1.67	0.140677	0.253803	-5.92
265485_at	putative Ta11-like non-LTR retroele	---	---	-1.22	-0.29	-1.67	0.140689	0.253805	-5.92
245707_at	NAC2-like protein	anac077	AT5G04400	-1.16	-0.21	-1.67	0.140742	0.253881	-5.92
252094_at	multicatalytic endopeptidase comple	PAD1	AT3G51260	1.22	0.29	1.67	0.140813	0.253988	-5.92
250114_s_a	AMP-binding protein	AAE5	AT5G16340	-1.24	-0.31	-1.67	0.140826	0.253993	-5.92
247090_at	putative protein similar to unknown	---	AT5G66370	-1.14	-0.19	-1.67	0.14095	0.254196	-5.92
267494_at	TCP1-chaperonin cofactor A isolog	KIS	AT2G30410	-1.15	-0.20	-1.67	0.140974	0.254219	-5.92
266730_at	putative RNA-binding protein	---	AT2G03110	-1.15	-0.20	-1.67	0.141011	0.254259	-5.92
246367_at	light repressible receptor protein ki	---	AT1G51880	-1.13	-0.18	-1.67	0.141019	0.254259	-5.92
256326_at	RING zinc-finger protein, putative c	---	AT3G02340	-1.18	-0.24	-1.67	0.141111	0.254406	-5.92
265557_at	unknown protein	---	---	-1.16	-0.21	-1.67	0.141172	0.254495	-5.92
244919_at	cytochrome c biogenesis orf203 Pro	---	AT2G07768	1.15	0.20	1.67	0.141191	0.254497	-5.92
251235_at	putative protein lysophospholipase	---	AT3G62860	-1.26	-0.34	-1.67	0.141195	0.254497	-5.92
264970_at	glyoxalase I, putative similar to glyc	---	AT1G67280	1.13	0.18	1.67	0.141277	0.254624	-5.92
249719_at	Expressed protein ; supported by fu	---	AT5G35735	1.48	0.57	1.67	0.141304	0.254643	-5.92
256722_at	hypothetical protein predicted by ge	---	AT2G34050	1.29	0.37	1.67	0.14131	0.254643	-5.92
257906_at	ribosomal protein, putative similar t	ATL5	AT3G25520	1.39	0.48	1.67	0.141364	0.25472	-5.92
260218_at	putative RING zinc finger protein co	---	AT1G74620	-1.11	-0.15	-1.67	0.14139	0.254734	-5.92
265540_at	Mutator-like transposase similar to	---	---	-1.12	-0.16	-1.67	0.141394	0.254734	-5.92
267112_at	putative adenosine phosphosulfate	APK	AT2G14750	-1.34	-0.42	-1.67	0.141465	0.254842	-5.92
258339_at	putative dynein light chain protein s	---	AT3G16120	-1.11	-0.16	-1.67	0.141504	0.25489	-5.92
245813_at	hypothetical protein contains similar	---	AT1G49920	-1.20	-0.27	-1.67	0.141514	0.25489	-5.92
265578_at	unknown protein	---	AT2G20100	-1.17	-0.23	-1.67	0.141534	0.254906	-5.92
253054_at	probable N-acetyltransferase hookle	HLS1	AT4G37580	1.13	0.18	1.67	0.141562	0.254936	-5.92
260945_at	hypothetical protein predicted by ge	---	AT1G05950	-1.16	-0.22	-1.66	0.141607	0.254988	-5.92
265780_at	Mutator-like transposase similar to	---	---	-1.14	-0.19	-1.66	0.141613	0.254988	-5.92

255526_at	hypothetical protein	---	AT4G02350	-1.17	-0.22	-1.66	0.141717	0.255156	-5.92
263389_at	putative retroelement pol polyprotei	---	---	-1.13	-0.18	-1.66	0.141771	0.255232	-5.92
259002_at	putative glutaredoxin similar to gluta	ROXY1	AT3G02000	-1.21	-0.28	-1.66	0.141876	0.255402	-5.93
250593_at	putative protein similar to unknown	---	AT5G07740	-1.12	-0.17	-1.66	0.14189	0.255407	-5.93
249050_at	cyclin-dependent protein kinase-like	---	AT5G44290	1.15	0.21	1.66	0.141935	0.255466	-5.93
253374_at	hypothetical protein ; supported by	---	AT4G33140	1.12	0.17	1.66	0.141945	0.255466	-5.93
263486_at	AP2 domain transcription factor	---	AT2G22200	-1.13	-0.18	-1.66	0.141998	0.255541	-5.93
247066_at	DNA binding protein-like	---	AT5G66940	-1.12	-0.16	-1.66	0.14203	0.255578	-5.93
266626_at	hypothetical protein predicted by ge	---	AT2G35360	1.20	0.27	1.66	0.142121	0.255711	-5.93
259129_at	unknown protein predicted by gens	PTF1	AT3G02150	-1.36	-0.44	-1.66	0.14213	0.255711	-5.93
265097_at	unknown protein	BARD1	AT1G04020	-1.23	-0.30	-1.66	0.142138	0.255711	-5.93
251193_at	translation releasing factor RF-1 -li	APG3	AT3G62910	-1.22	-0.28	-1.66	0.142175	0.255758	-5.93
251423_at	regulatory protein - like regulatory p	CYCP3;2	AT3G60550	-1.18	-0.24	-1.66	0.142235	0.255846	-5.93
263611_at	hypothetical protein predicted by ge	---	AT2G16450	-1.12	-0.16	-1.66	0.142301	0.255938	-5.93
263914_at	unknown protein	AtGRF3	AT2G36400	-1.27	-0.35	-1.66	0.142309	0.255938	-5.93
259059_at	hypothetical protein	---	AT3G07300	1.14	0.19	1.66	0.142328	0.255952	-5.93
249489_at	acyltransferase -like protein antho	---	AT5G39090	1.18	0.24	1.66	0.14238	0.256026	-5.93
253503_at	cytochrome P450-like protein cytoc	CYP82C3	AT4G31950	1.27	0.35	1.66	0.142453	0.256138	-5.93
265108_s_a	similar to flavin-binding monooxyge	---	AT1G62620 ,	-1.18	-0.24	-1.66	0.142476	0.256158	-5.93
248031_at	unknown protein ; supported by cD	---	AT5G55640	1.11	0.15	1.66	0.142534	0.256242	-5.93
263339_at	Mutator-like transposase similar to	---	---	1.10	0.14	1.66	0.142556	0.256261	-5.93
254149_at	putative protein hypothetical protei	---	AT4G24300	-1.20	-0.27	-1.66	0.142575	0.256267	-5.93
261118_at	protease, putative similar to SP:P3	---	AT1G75460	-1.15	-0.20	-1.66	0.142581	0.256267	-5.93
259709_at	Expressed protein ; supported by fu	---	AT1G77655	-1.16	-0.22	-1.66	0.142603	0.256285	-5.93
266146_at	En/Spm-like transposon protein rela	---	---	-1.12	-0.16	-1.66	0.142642	0.256336	-5.93
252306_at	hypothetical protein	---	AT3G49270	-1.11	-0.15	-1.66	0.14277	0.256538	-5.93
264602_at	putative Ser/Thr protein kinase simi	---	AT1G04700	-1.14	-0.19	-1.66	0.142777	0.256538	-5.93
249539_at	putative protein AtPP - Brassica na	---	AT5G38100	-1.17	-0.23	-1.66	0.142827	0.256607	-5.93
250465_at	putative protein various predicted p	---	AT5G10070	1.11	0.15	1.66	0.142865	0.256656	-5.93
260511_at	anthranilate phosphoribosyltransfe	---	AT1G51570	-1.21	-0.27	-1.66	0.143016	0.256906	-5.93
249665_at	unknown protein	---	AT5G35830	-1.16	-0.21	-1.66	0.143221	0.257254	-5.93
245023_at	PSII I protein	---	---	-1.26	-0.33	-1.66	0.143241	0.257269	-5.93
264738_at	unknown protein contains similarity	---	AT1G62250	-1.18	-0.23	-1.66	0.14344	0.257596	-5.94
260639_at	hypothetical protein predicted by ge	---	AT1G53180	1.39	0.48	1.66	0.143447	0.257596	-5.94
266753_at	auxin-induced protein (IAA20) ident	IAA20	AT2G46990	-1.13	-0.18	-1.66	0.143458	0.257596	-5.94

248936_at	heat shock transcription factor	RHA1	AT5G45710	-1.21	-0.27	-1.66	0.143468	0.257596	-5.94
247563_at	putative protein various predicted p	PDCB1	AT5G61130	1.19	0.25	1.66	0.14349	0.257615	-5.94
265115_at	rho GDP-dissociation inhibitor, puta	---	AT1G62450	-1.14	-0.19	-1.66	0.143531	0.257669	-5.94
255287_at	see T10M13.1 (GB:AF001308) simi	---	AT4G04660	-1.15	-0.20	-1.66	0.143553	0.257688	-5.94
258636_at	putative GTPase activator protein c	---	AT3G07890	-1.15	-0.20	-1.66	0.143608	0.257766	-5.94
249595_at	putative protein SIAH1 protein, Bra	---	AT5G37930	-1.20	-0.26	-1.65	0.143676	0.257868	-5.94
246732_at	putative protein hypothetical protei	---	AT5G27650	1.18	0.24	1.65	0.14372	0.257927	-5.94
245257_at	calmodulin ; supported by cDNA: gi	CAM8	AT4G14640	1.14	0.19	1.65	0.14375	0.25796	-5.94
245623_s_a	hypothetical protein	---	AT4G14096	1.13	0.18	1.65	0.143832	0.258087	-5.94
261267_at	unknown protein ;supported by full-	---	AT1G26750	-1.15	-0.21	-1.65	0.143856	0.258111	-5.94
258344_at	hypothetical protein similar to hypot	CEG	AT3G22650	-1.14	-0.18	-1.65	0.143884	0.258141	-5.94
249252_at	putative protein contains similarity t	---	AT5G42010	1.58	0.66	1.65	0.143946	0.25823	-5.94
265409_at	unknown protein	PIP2;8	AT2G16830	-1.15	-0.20	-1.65	0.143981	0.258274	-5.94
249949_at	SAE1-S9-protein - like SAE1-S9-pr	---	AT5G18880	-1.14	-0.19	-1.65	0.144034	0.258349	-5.94
261522_at	RIBOSOMAL PROTEIN, putative s	---	AT1G71710	-1.31	-0.39	-1.65	0.144101	0.258448	-5.94
247861_at	strong similarity to unknown protein	---	AT5G58160	-1.17	-0.23	-1.65	0.144161	0.258536	-5.94
258597_x_a	hypothetical protein predicted by ge	---	AT3G04390	1.12	0.16	1.65	0.14427	0.258704	-5.94
258490_at	hypothetical protein predicted by ge	---	AT3G02670	-1.13	-0.18	-1.65	0.144278	0.258704	-5.94
262838_at	major latex protein, putative similar	---	AT1G14960	-1.15	-0.20	-1.65	0.144291	0.258707	-5.94
257924_at	unknown protein	---	AT3G23190	-1.20	-0.26	-1.65	0.144367	0.258823	-5.94
263717_at	malate oxidoreductase (malic enzyr	---	AT2G13560	-1.11	-0.15	-1.65	0.14441	0.25888	-5.94
260691_s_a	hypothetical protein predicted by ge	---	---	-1.14	-0.19	-1.65	0.144458	0.258947	-5.94
250055_at	NADH-cytochrome b5 reductase ;s	ATCBR	AT5G17770	1.37	0.46	1.65	0.144476	0.258959	-5.94
247329_at	putative protein similar to unknown	---	AT5G64150	1.27	0.34	1.65	0.144489	0.25896	-5.94
262271_x_a	hypothetical protein predicted by ge	---	---	-1.25	-0.32	-1.65	0.144518	0.258993	-5.94
255447_at	hypothetical protein similar to A. th	---	AT4G02790	1.28	0.35	1.65	0.144665	0.259229	-5.94
250790_at	putative protein similar to unknown	---	AT5G05640	-1.22	-0.28	-1.65	0.144678	0.259229	-5.94
263063_s_a	putative peroxidase	---	AT2G18140	-1.23	-0.30	-1.65	0.144684	0.259229	-5.94
260014_at	hydroxypyruvate reductase (HPR) i	HPR	AT1G68010	-1.17	-0.22	-1.65	0.144713	0.25925	-5.94
262027_at	DNA-binding protein, putative simil	HOS10	AT1G35515	-1.28	-0.36	-1.65	0.144718	0.25925	-5.94
256131_at	bZIP transcription factor, putative s	AtbZIP58	AT1G13600	-1.18	-0.24	-1.65	0.144802	0.259375	-5.94
255730_at	dihydroflavonol 4-reductase, putati	---	AT1G25460	-1.13	-0.18	-1.65	0.144817	0.259375	-5.94
249378_at	putative protein microtubule-associ	---	AT5G40450	-1.22	-0.28	-1.65	0.144822	0.259375	-5.94
252712_at	glutathione transferase-like protein	ATGSTU27	AT3G43800	-1.27	-0.34	-1.65	0.144881	0.259459	-5.95
264114_at	hypothetical protein predicted by ge	CDT1A	AT2G31270	-1.35	-0.44	-1.65	0.144975	0.259608	-5.95

256325_at	hypothetical protein	---	AT3G02330	1.17	0.22	1.65	0.145064	0.259727	-5.95
246890_at	Expressed protein ; supported by cl	---	AT5G25410	-1.15	-0.21	-1.65	0.145065	0.259727	-5.95
246736_at	putative protein hypothetical protei	---	AT5G27560	-1.15	-0.20	-1.65	0.145116	0.259798	-5.95
249894_at	unknown protein ; supported by cD	---	AT5G22580	-5.77	-2.53	-1.65	0.14513	0.259803	-5.95
254505_at	Expressed protein ; supported by fu	---	AT4G19985	-1.21	-0.28	-1.65	0.145164	0.259844	-5.95
251850_at	F-box protein family, AtFBL17 cont	FBL17	AT3G54650	-1.21	-0.28	-1.65	0.145179	0.25985	-5.95
246369_at	light repressible receptor protein ki	---	AT1G51910	-1.18	-0.24	-1.65	0.145192	0.259854	-5.95
257645_at	unknown protein ;supported by full-	---	AT3G25790	-1.18	-0.24	-1.65	0.145226	0.259894	-5.95
247786_at	putative protein various predicted p	PMR5	AT5G58600	1.12	0.17	1.65	0.145248	0.259902	-5.95
245218_s_a	viral resistance protein, putative, 5'	---	AT1G58848	-1.11	-0.15	-1.65	0.145257	0.259902	-5.95
246111_at	putative protein	---	AT5G20170	-1.15	-0.20	-1.65	0.145265	0.259902	-5.95
265013_at	unknown protein similar to EST gb[	---	AT1G24460	-1.13	-0.18	-1.65	0.145289	0.259926	-5.95
260080_at	putative RNA-binding protein conta	APUM7	AT1G78160	-1.11	-0.16	-1.65	0.145309	0.25994	-5.95
255595_at	putative chitinase similar to peanut	---	AT4G01700	1.59	0.67	1.65	0.145367	0.260023	-5.95
253068_at	putative protein Kaposi's sarcoma-	---	AT4G37820	1.14	0.19	1.65	0.145396	0.260054	-5.95
256748_x_a	En/Spm transposon protein, putativ	---	---	-1.15	-0.20	-1.65	0.145422	0.260082	-5.95
257720_at	hypothetical protein similar to GB:A	---	AT3G18450	-1.16	-0.21	-1.65	0.145455	0.260119	-5.95
263304_at	hypothetical protein predicted by ge	---	AT2G01920	-1.14	-0.18	-1.65	0.145488	0.260159	-5.95
250209_at	putative protein similar to unknown	---	AT5G14030	1.19	0.25	1.65	0.145534	0.26022	-5.95
252756_s_a	putative protein hypothetical protein	---	AT1G58520	-1.13	-0.18	-1.65	0.145556	0.260239	-5.95
261467_at	unknown protein	VOZ1	AT1G28520	1.22	0.29	1.65	0.145644	0.260376	-5.95
254044_at	putative xyloglucan endo-1,4-beta-	XTR9	AT4G25820	-1.13	-0.18	-1.64	0.145692	0.260442	-5.95
251937_at	putative protein predicted protein, A	CPuORF46	AT3G53400	-1.52	-0.60	-1.64	0.145766	0.260553	-5.95
246110_at	putative protein predicted proteins -	---	AT5G20140	-1.23	-0.30	-1.64	0.145783	0.260563	-5.95
255202_at	putative polyprotein	---	---	-1.13	-0.18	-1.64	0.145811	0.260593	-5.95
267502_at	putative cytochrome P450	CYP76C4	AT2G45550	-1.13	-0.17	-1.64	0.145837	0.260619	-5.95
259026_at	putative protein kinase similar to hy	---	AT3G09240	-1.15	-0.21	-1.64	0.145982	0.260858	-5.95
266111_at	unknown protein	---	AT2G02060	-1.14	-0.19	-1.64	0.146054	0.260966	-5.95
267573_at	putative tropinone reductase	---	AT2G30670	-1.11	-0.15	-1.64	0.146074	0.260982	-5.95
256523_at	unknown protein ;supported by full-	---	AT1G66070	1.15	0.21	1.64	0.146094	0.260996	-5.95
261479_at	unknown protein ;supported by full-	IQD28	AT1G14380	1.22	0.29	1.64	0.146204	0.261173	-5.95
249018_at	CHP-rich zinc finger protein-like	---	AT5G44770	-1.22	-0.29	-1.64	0.146233	0.261204	-5.95
250889_at	putative protein exostose-related p	---	AT5G04500	-1.14	-0.19	-1.64	0.14626	0.261232	-5.95
264136_at	leucine-rich repeat transmembrane	SRF5	AT1G78980	-1.20	-0.27	-1.64	0.146273	0.261235	-5.95
245370_at	Expressed protein ; supported by fu	---	AT4G16840	1.29	0.37	1.64	0.146308	0.261276	-5.95

246969_at	glutamic acid-rich protein other glut	---	AT5G24880	-1.15	-0.21	-1.64	0.14638	0.261385	-5.95
266877_at	putative glucanase	AtGH9B12	AT2G44570	-1.14	-0.19	-1.64	0.146416	0.261428	-5.96
256095_at	hypothetical protein contains Pfam	---	AT1G13630	1.28	0.36	1.64	0.146435	0.261442	-5.96
260761_at	hypothetical protein predicted by ge	---	AT1G49150	1.24	0.31	1.64	0.146502	0.261523	-5.96
252835_at	putative protein several hypothetica	---	AT3G42060	-1.16	-0.21	-1.64	0.146503	0.261523	-5.96
252581_at	hypothetical protein	---	AT3G45500	-1.15	-0.21	-1.64	0.146685	0.261826	-5.96
247900_at	60S acidic ribosomal protein P3 ; s	---	AT5G57290	1.31	0.39	1.64	0.146766	0.261951	-5.96
261697_at	hypothetical protein identical to hyp	---	AT1G32610	-1.25	-0.32	-1.64	0.146894	0.262159	-5.96
259326_at	putative mitochondrial processing p	MPPalpha	AT3G16480	1.12	0.17	1.64	0.146954	0.262245	-5.96
267048_at	hypothetical protein predicted by ge	---	AT2G34200	1.20	0.26	1.64	0.147016	0.262318	-5.96
248087_at	unknown protein	---	AT5G55060	-1.11	-0.15	-1.64	0.147018	0.262318	-5.96
254082_at	glutamine cyclotransferase precurs	QC	AT4G25720	1.31	0.39	1.64	0.147095	0.262436	-5.96
244909_at	hypothetical protein	---	---	-1.14	-0.19	-1.64	0.147115	0.262448	-5.96
261329_at	hypothetical protein predicted by ge	---	AT1G44890	-1.15	-0.20	-1.64	0.147125	0.262448	-5.96
245427_at	putative protein	---	AT4G17550	1.20	0.26	1.64	0.147181	0.262517	-5.96
253541_at	putative protein various predicted p	---	AT4G31630	-1.12	-0.16	-1.64	0.147187	0.262517	-5.96
267267_at	putative cytochrome P450	CYP71B9	AT2G02580	1.14	0.19	1.64	0.147244	0.262599	-5.96
264733_at	phloem serpin-1, putative similar to	---	AT1G62170	-1.12	-0.16	-1.64	0.147318	0.26271	-5.96
245088_at	subtilisin-like serine protease conta	---	AT2G39850	-1.35	-0.43	-1.64	0.147356	0.262757	-5.96
257629_at	cellulase, putative contains Pfam p	---	AT3G26140	-1.15	-0.21	-1.64	0.147398	0.262811	-5.96
245970_at	beta-galactosidase ; supported by	BGAL7	AT5G20710	-1.17	-0.23	-1.64	0.147504	0.262968	-5.96
254611_at	Expressed protein ; supported by fu	---	AT4G19095	-1.13	-0.17	-1.64	0.147509	0.262968	-5.96
256616_at	hypothetical protein similar to unkno	---	AT3G22260	1.23	0.30	1.64	0.147531	0.262988	-5.96
252503_at	putative protein hypothetical protei	---	AT3G46910	-1.15	-0.20	-1.64	0.147556	0.263012	-5.96
247574_at	putative protein predicted protein, A	---	AT5G61228	1.14	0.19	1.64	0.14758	0.263017	-5.96
260502_at	putative Tub family protein contains	AtTLP6	AT1G47270	1.16	0.21	1.64	0.147582	0.263017	-5.96
264699_at	unknown protein ;supported by full-	---	AT1G69980	1.11	0.15	1.64	0.147598	0.263025	-5.96
246900_at	putative protein flavin-containing m	YUC6	AT5G25620	-1.19	-0.25	-1.64	0.147644	0.263085	-5.96
245014_at	ATPase beta subunit	---	---	-1.11	-0.15	-1.64	0.147656	0.263086	-5.96
252116_at	putative protein ;supported by full-l	---	AT3G51510	-1.17	-0.22	-1.64	0.147706	0.263156	-5.96
249918_at	putative protein predicted protein, A	---	AT5G19240	-1.51	-0.60	-1.64	0.147777	0.263246	-5.96
247190_at	D-type cyclin (emb|CAB41347.1) ;s	CYCD4;1	AT5G65420	-1.15	-0.20	-1.64	0.14778	0.263246	-5.96
261528_at	pectate lyase, putative similar to pe	AT59	AT1G14420	-1.15	-0.20	-1.63	0.147825	0.263305	-5.96
264272_at	hypothetical protein contains simila	ANAC023	AT1G60280	-1.11	-0.16	-1.63	0.147863	0.263352	-5.96
253229_at	putative protein ;supported by full-l	---	AT4G34660	-1.10	-0.14	-1.63	0.147898	0.263388	-5.96

250692_at	putative protein strong similarity to u	---	AT5G06560	1.15	0.20	1.63	0.147906	0.263388	-5.96
258227_at	6-4 photolyase (UVR3) identical to	UVR3	AT3G15620	1.63	0.70	1.63	0.147943	0.263434	-5.96
258324_at	putative DNA binding protein identic	TSO1	AT3G22780	-1.12	-0.16	-1.63	0.14804	0.263586	-5.97
261633_at	hypothetical protein predicted by ge	---	AT1G49930	1.13	0.17	1.63	0.14806	0.2636	-5.97
260921_at	amp-binding protein, putative simila	---	AT1G21540	-1.23	-0.30	-1.63	0.1481	0.263651	-5.97
265881_at	hypothetical protein predicted by ge	---	AT2G42480	-1.15	-0.21	-1.63	0.148129	0.263683	-5.97
263159_at	RSH3 (RelA/SpoT homolog) identic	RSH3	AT1G54130	-1.19	-0.25	-1.63	0.148144	0.263688	-5.97
263227_at	Expressed protein ; supported by cl	---	AT1G30750	-1.16	-0.22	-1.63	0.148165	0.263698	-5.97
255581_at	putative transposon protein similar	---	---	-1.17	-0.23	-1.63	0.148173	0.263698	-5.97
257666_at	bactericidal permeability-increasing	---	AT3G20270	-1.25	-0.32	-1.63	0.148239	0.263796	-5.97
261458_at	unknown protein contains DNA-J do	---	AT1G21080	-1.17	-0.23	-1.63	0.148252	0.263799	-5.97
257980_at	hypothetical protein predicted by ge	---	AT3G20760	-1.14	-0.19	-1.63	0.148274	0.263817	-5.97
247146_at	unknown protein	---	AT5G65610	-1.23	-0.30	-1.63	0.148291	0.263827	-5.97
AFFX-r2-Bs	B. subtilis /GEN=lys /DB_XREF=gb	---	---	1.49	0.58	1.63	0.148315	0.263848	-5.97
252149_at	putative protein hypothetical protei	---	AT3G51290	-1.11	-0.16	-1.63	0.148388	0.263923	-5.97
249625_at	hypothetical protein ;supported by f	---	AT5G37480	-1.13	-0.18	-1.63	0.148392	0.263923	-5.97
258294_at	hypothetical protein predicted by ge	---	AT3G23350	-1.14	-0.18	-1.63	0.148392	0.263923	-5.97
258332_at	putative transport protein similar to	---	AT3G16180	-1.43	-0.52	-1.63	0.148465	0.264033	-5.97
259281_at	putative Myb-related transcription fa	MYB106	AT3G01140	-1.15	-0.21	-1.63	0.148656	0.264351	-5.97
261721_at	unknown protein ; supported by cDN	---	AT1G08480	1.12	0.17	1.63	0.148709	0.264425	-5.97
248191_at	putative protein similar to unknown	---	AT5G54130	1.16	0.21	1.63	0.148728	0.264439	-5.97
263580_at	hypothetical protein similar to hypot	---	AT2G17140	1.15	0.20	1.63	0.148948	0.264751	-5.97
254925_at	beta-adaptin - like protein beta-ada	---	AT4G11380	-1.16	-0.21	-1.63	0.148949	0.264751	-5.97
261424_at	unknown protein	---	AT1G18700	1.11	0.15	1.63	0.14895	0.264751	-5.97
247377_at	pectate lyase ; supported by cDNA	---	AT5G63180	-2.70	-1.43	-1.63	0.14895	0.264751	-5.97
264651_at	putative histone H2A Strong simila	H2AXA	AT1G08880	1.25	0.33	1.63	0.149017	0.26485	-5.97
258154_at	unknown protein ; supported by cD	---	AT3G18210	1.17	0.23	1.63	0.149107	0.264988	-5.97
246839_at	putative protein	---	AT5G26720	1.15	0.20	1.63	0.149194	0.26511	-5.97
245424_at	putative protein	---	AT4G17505	-1.13	-0.18	-1.63	0.149206	0.26511	-5.97
259956_at	unknown protein ;supported by full-	RRA2	AT1G75110	1.13	0.18	1.63	0.149221	0.26511	-5.97
255531_at	hypothetical protein similar to orf of	---	AT4G02160	-1.14	-0.19	-1.63	0.149221	0.26511	-5.97
264936_at	hypothetical protein predicted by ge	EDA16	AT1G61140	1.12	0.17	1.63	0.149275	0.265184	-5.97
254929_at	putative protein mitochondrial carri	---	AT4G11440	-1.14	-0.19	-1.63	0.149291	0.265191	-5.97
248432_at	putative protein similar to unknown	---	AT5G51390	-1.35	-0.43	-1.63	0.149355	0.265248	-5.97
261045_at	pathogenesis related protein, putat	---	AT1G01310	-1.11	-0.15	-1.63	0.149357	0.265248	-5.97

262561_at	late embryogenesis abundant prote	---	AT1G34340	-1.14	-0.19	-1.63	0.149357	0.265248	-5.97
256297_at	cytochrome P450, putative contain	---	AT1G69500	1.17	0.22	1.63	0.149387	0.26528	-5.97
260484_at	putative zinc finger protein similar to	---	AT1G68360	-1.15	-0.20	-1.63	0.149404	0.26529	-5.97
255834_at	putative RNA-binding protein	---	AT2G33410	1.11	0.14	1.63	0.149484	0.26541	-5.97
252071_at	putative protein DNA helicase hom	---	AT3G51690	-1.10	-0.14	-1.63	0.149621	0.265615	-5.98
251582_at	putative protein CARBON CATABO	---	AT3G58580	-1.13	-0.18	-1.63	0.149622	0.265615	-5.98
254654_at	translation initiation factor eIF4E ;s	EIF4E	AT4G18040	1.15	0.20	1.63	0.149719	0.265765	-5.98
246169_at	putative protein predicted protein, I	---	AT5G32470	-1.15	-0.20	-1.63	0.149732	0.265768	-5.98
253641_at	hypothetical protein ;supported by f	---	AT4G29980	-1.16	-0.22	-1.63	0.149896	0.266038	-5.98
266496_at	putative zinc finger protein	---	---	-1.15	-0.20	-1.62	0.14991	0.266044	-5.98
262639_at	calmodulin, putative similar to calm	---	AT1G62820	-1.22	-0.29	-1.62	0.149938	0.266072	-5.98
247617_at	receptor like protein kinase recepto	---	AT5G60270	1.35	0.44	1.62	0.150053	0.266255	-5.98
253664_at	NADPH-ferrihemoprotein reductase	ATR2	AT4G30210	-1.48	-0.57	-1.62	0.150121	0.266352	-5.98
246504_at	hypothetical protein ;supported by	---	AT5G16160	-1.11	-0.14	-1.62	0.150131	0.266352	-5.98
248923_at	putative protein similar to unknown	atnudt11	AT5G45940	-1.20	-0.27	-1.62	0.150164	0.26639	-5.98
259538_at	putative serine/threonine kinase ; su	---	AT1G12310	-1.17	-0.23	-1.62	0.150176	0.26639	-5.98
253848_at	putative protein regulator protein, F	---	AT4G28020	1.19	0.25	1.62	0.150196	0.266406	-5.98
257692_at	hypothetical protein contains similar	---	AT3G12840	-1.12	-0.17	-1.62	0.150268	0.266512	-5.98
247204_at	GTP binding protein-like	AtRABH1a	AT5G64990	1.15	0.20	1.62	0.150308	0.266545	-5.98
246999_at	photoreceptor-interacting protein-lik	NPY3	AT5G67440	-1.28	-0.35	-1.62	0.15032	0.266545	-5.98
267575_at	unknown protein predicted by gens	---	AT2G30690	-1.14	-0.19	-1.62	0.150322	0.266545	-5.98
262165_at	putative acyl-CoA:1-acylglycerol-3-p	LPAT4	AT1G75020	1.24	0.32	1.62	0.150333	0.266545	-5.98
249921_at	reverse-transcriptase - like protein p	---	AT5G19270	-1.13	-0.18	-1.62	0.150375	0.266599	-5.98
258539_at	putative RNA-binding protein conta	---	AT3G06970	-1.12	-0.16	-1.62	0.150389	0.266602	-5.98
249172_at	putative protein similar to unknown	---	AT5G42950	1.15	0.20	1.62	0.150491	0.266762	-5.98
258491_at	unknown protein ;supported by full-	---	AT3G02700	-1.22	-0.28	-1.62	0.150539	0.266827	-5.98
247250_at	FAS2 (dbj|BAA77766.1) ; supporte	FAS2	AT5G64630	-1.23	-0.30	-1.62	0.150566	0.266844	-5.98
266415_at	putative nonspecific lipid-transfer pr	LTP2	AT2G38530	-1.85	-0.89	-1.62	0.150574	0.266844	-5.98
266379_at	1,4-beta-xylan endohydrolase	---	AT2G14690	-1.15	-0.20	-1.62	0.150584	0.266844	-5.98
248626_at	receptor protein kinase-like protein	---	AT5G48940	-1.13	-0.18	-1.62	0.150656	0.266951	-5.98
248425_at	ACC synthase; 1-aminocyclopropar	ACS12	AT5G51690	1.18	0.24	1.62	0.150724	0.267051	-5.98
254916_at	putative protein hypothetical protei	---	AT4G11340	-1.14	-0.19	-1.62	0.150755	0.267084	-5.98
256521_at	AMP-binding enzyme, putative con	---	AT1G66120	-1.13	-0.18	-1.62	0.150778	0.267093	-5.98
250919_at	putative protein myosin heavy chai	---	AT5G03660	1.17	0.22	1.62	0.150783	0.267093	-5.98
264609_at	unknown protein ;supported by full-	---	AT1G04530	1.28	0.35	1.62	0.1508	0.267102	-5.98

265640_at	putative cysteine proteinase	---	AT2G27395	-1.11	-0.15	-1.62	0.150859	0.267153	-5.98
251296_at	putative protein paladin - Mus mus	---	AT3G62010	-1.15	-0.20	-1.62	0.150862	0.267153	-5.98
259240_at	unknown protein ; supported by cD	---	AT3G11590	-1.19	-0.26	-1.62	0.150863	0.267153	-5.98
250288_at	auxin-responsive - like protein Nt-g	---	AT5G13350	-1.15	-0.20	-1.62	0.150906	0.267207	-5.98
259747_at	putative dehydrogenase similar to D	---	AT1G71170	1.16	0.21	1.62	0.150919	0.26721	-5.98
256422_at	unknown protein	MOS2	AT1G33520	1.14	0.19	1.62	0.151021	0.267351	-5.98
251468_at	epsin-like protein Af10-protein - Av	---	AT3G59290	1.14	0.19	1.62	0.151022	0.267351	-5.98
245299_at	ras-related small GTP-binding prote	ATRAB1C	AT4G17530	-1.19	-0.25	-1.62	0.151106	0.267466	-5.98
256502_at	Eukaryotic translation initiation fact	---	AT1G36730	1.20	0.26	1.62	0.15112	0.267466	-5.98
267144_at	unknown protein	GPAT6	AT2G38110	1.36	0.45	1.62	0.151122	0.267466	-5.98
256898_at	abscisic acid-insensitive protein 3 i	ABI3	AT3G24650	-1.12	-0.17	-1.62	0.151149	0.267493	-5.98
266342_at	hypothetical protein predicted by gr	---	AT2G01540	1.20	0.26	1.62	0.151222	0.2676	-5.99
257902_at	receptor kinase, putative similar to	---	AT3G28450	1.24	0.31	1.62	0.151314	0.267743	-5.99
255270_at	putative protein (ubiquitin like) to va	---	AT4G05250	-1.11	-0.15	-1.62	0.151349	0.267772	-5.99
245590_at	hypothetical protein	---	AT4G15060	-1.13	-0.18	-1.62	0.151354	0.267772	-5.99
249904_at	unknown protein	---	AT5G22700	-1.13	-0.18	-1.62	0.151437	0.267888	-5.99
263996_at	unknown protein	---	AT2G22530	-1.16	-0.21	-1.62	0.151443	0.267888	-5.99
264459_at	putative reverse transcriptase simila	---	---	-1.23	-0.29	-1.62	0.151529	0.268019	-5.99
245774_at	hypothetical protein contains similar	TCP24	AT1G30210	-1.12	-0.17	-1.62	0.151577	0.268083	-5.99
259905_s_a	hypothetical protein predicted by ge	---	AT1G74130	-1.10	-0.14	-1.62	0.151667	0.268221	-5.99
258961_at	hypothetical protein similar to myb-	---	AT3G10580	1.11	0.15	1.62	0.151698	0.268252	-5.99
256388_at	unknown protein	---	AT3G06180	-1.14	-0.19	-1.62	0.151717	0.268252	-5.99
264105_x_a	hypothetical protein	---	AT2G13760	-1.16	-0.21	-1.62	0.151725	0.268252	-5.99
257910_at	hypothetical protein similar to hypot	---	AT3G25580	-1.16	-0.22	-1.62	0.151731	0.268252	-5.99
249712_at	eIF4Eiso protein (emb|CAA71579.1	LSP1	AT5G35620	1.20	0.26	1.62	0.151754	0.268272	-5.99
258006_at	cysteine proteinase non-consensus	---	AT3G19400	1.20	0.26	1.62	0.151822	0.268359	-5.99
251030_at	putative protein kinesin light chain	NDP1	AT5G02130	1.21	0.28	1.62	0.151832	0.268359	-5.99
256047_at	hypothetical protein predicted by ge	---	AT1G07060	-1.16	-0.22	-1.62	0.151838	0.268359	-5.99
262734_at	lipase, putative similar to lipase GE	---	AT1G28640	-1.12	-0.17	-1.62	0.151871	0.268364	-5.99
254090_at	MtN3 - like protein MtN3 (induced	---	AT4G25010	-1.17	-0.22	-1.62	0.151876	0.268364	-5.99
253073_at	cytochrome P450 monooxygenase	CYP81F4	AT4G37410	-1.16	-0.22	-1.62	0.151877	0.268364	-5.99
259874_at	unknown protein	---	AT1G76660	-1.20	-0.26	-1.62	0.151897	0.268378	-5.99
255284_at	5-adenylylsulfate reductase ;suppo	אפר-01	AT4G04610	1.54	0.62	1.62	0.151937	0.268428	-5.99
254648_at	lipase-like protein Pn47p, Ipomoea	---	AT4G18550	-1.13	-0.17	-1.62	0.151956	0.268435	-5.99
258610_at	IAA-amino acid hydrolase (ILR1) ide	ILR1	AT3G02875	1.26	0.34	1.62	0.151964	0.268435	-5.99

266009_at	putative kinesin heavy chain	---	AT2G37420	1.14	0.18	1.61	0.152131	0.268709	-5.99
249376_at	Expressed protein ; supported by fu	---	AT5G40645	-1.11	-0.14	-1.61	0.152329	0.269038	-5.99
246025_at	zwille/pinhead-like protein GERp95	---	AT5G21150	-1.18	-0.23	-1.61	0.152349	0.269053	-5.99
265519_at	hypothetical protein	---	AT2G06040	1.13	0.18	1.61	0.15237	0.269069	-5.99
251990_at	putative protein MICRONUCLEAR	---	AT3G53320	-1.21	-0.28	-1.61	0.152385	0.269075	-5.99
255697_at	hypothetical protein	---	AT4G00130	-1.19	-0.25	-1.61	0.152435	0.269141	-5.99
261321_at	hypothetical protein predicted by ge	---	AT1G44740	-1.14	-0.19	-1.61	0.152446	0.269141	-5.99
255103_at	putative phosphoglyceride transfer	---	AT4G08690	-1.13	-0.18	-1.61	0.152507	0.269227	-5.99
249513_at	glucosyltransferase-like protein ; su	---	AT5G38460	-1.13	-0.18	-1.61	0.152547	0.269277	-5.99
252146_at	putative protein steroid sulfotransfe	---	AT3G51210	-1.11	-0.15	-1.61	0.152567	0.269277	-5.99
253342_at	Expressed protein ; supported by cl	PAA1	AT4G33520	-1.35	-0.43	-1.61	0.152571	0.269277	-5.99
259279_at	putative cystathionine gamma-synth	MTO1	AT3G01120	-1.13	-0.18	-1.61	0.152618	0.26934	-5.99
257526_s_a	hypothetical protein similar to putati	---	AT3G09330	-1.12	-0.16	-1.61	0.152722	0.269502	-5.99
262615_at	initiation factor 5A-4 identical to initi	ELF5A-1	AT1G13950	1.17	0.23	1.61	0.152747	0.269526	-5.99
262391_at	geranyl geranyl pyrophosphate syn	GGPS6	AT1G49530	-1.15	-0.20	-1.61	0.152791	0.269573	-5.99
261732_at	hypothetical protein predicted by ge	---	AT1G47770	-1.14	-0.19	-1.61	0.152797	0.269573	-5.99
AFFX-BioDr	E. coli /GEN=bioD /DB_XREF=gb:J	---	---	-1.36	-0.44	-1.61	0.152913	0.269732	-6.00
265949_at	putative vicilin storage protein (glob	---	AT2G18540	1.11	0.15	1.61	0.152921	0.269732	-6.00
245586_at	hypothetical protein	---	AT4G14980	-1.13	-0.18	-1.61	0.152923	0.269732	-6.00
257874_at	hypothetical protein	---	AT3G17110	-1.15	-0.20	-1.61	0.152964	0.269772	-6.00
261955_at	proteasome regulatory subunit, put	RPN12a	AT1G64520	1.12	0.16	1.61	0.152969	0.269772	-6.00
253080_at	hypothatical protein	---	---	-1.13	-0.17	-1.61	0.153003	0.269797	-6.00
245301_at	farnesyl-pyrophosphate synthetase	FPS2	AT4G17190	-1.30	-0.37	-1.61	0.153007	0.269797	-6.00
263144_at	unknown protein similar to putative	---	AT1G54070	-1.16	-0.22	-1.61	0.153047	0.269846	-6.00
257915_at	superman protein identical to super	SUP	AT3G23130	-1.16	-0.21	-1.61	0.153125	0.269962	-6.00
259001_at	hypothetical protein predicted by ge	---	AT3G01960	1.42	0.51	1.61	0.153178	0.270036	-6.00
263554_at	putative Ta11-like non-LTR retroele	---	---	-1.25	-0.33	-1.61	0.153214	0.270074	-6.00
267043_at	unknown protein	---	AT2G34330	-1.15	-0.20	-1.61	0.153223	0.270074	-6.00
253418_at	putative protein tom-1B protein, Ga	---	AT4G32760	-1.12	-0.16	-1.61	0.153379	0.270326	-6.00
252402_s_a	putative protein predicted proteins,	---	AT3G48050	1.12	0.16	1.61	0.153422	0.270382	-6.00
261181_at	monosaccharide transporter, putati	---	AT1G34580	-1.17	-0.23	-1.61	0.153607	0.270687	-6.00
263698_at	hypothetical protein similar to a mu	---	---	-1.17	-0.23	-1.61	0.153636	0.27071	-6.00
261865_at	sterol delta7 reductase identical to (	DWF5	AT1G50430	-1.23	-0.30	-1.61	0.153644	0.27071	-6.00
250667_at	40S ribosomal protein S4 ;supporte	---	AT5G07090	1.31	0.39	1.61	0.153655	0.27071	-6.00
265068_at	hypothetical protein Similar to Kines	---	AT1G55550	1.12	0.16	1.61	0.153697	0.270762	-6.00

258235_at	alternative oxidase 1c precursor ide	AOX1C	AT3G27620	-1.15	-0.20	-1.61	0.15373	0.2708	-6.00
251573_at	phenylalanine-tRNA synthetase-lik	---	AT3G58140	-1.15	-0.20	-1.61	0.153746	0.270806	-6.00
258057_at	unknown protein	AAR3	AT3G28970	1.12	0.16	1.61	0.153844	0.270959	-6.00
260586_at	unknown protein predicted by gene	---	AT2G43630	1.15	0.20	1.61	0.154027	0.271261	-6.00
262418_at	thioredoxin, putative similar to thior	ATHX	AT1G50320	-1.22	-0.28	-1.61	0.154077	0.271326	-6.00
248316_at	putative protein similar to unknown	---	AT5G52670	-1.19	-0.25	-1.61	0.154109	0.271363	-6.00
267402_at	putative SF16 protein {Helianthus a	IQD6	AT2G26180	-1.19	-0.26	-1.61	0.15413	0.271379	-6.00
246023_at	ethylene-insensitive3-like2 (EIL2)	EIL2	AT5G21120	-1.13	-0.18	-1.61	0.154149	0.271391	-6.00
255039_at	calmodulin-domain protein kinase C	CPK4	AT4G09570	1.16	0.21	1.60	0.154266	0.271576	-6.00
256315_at	hypothetical protein predicted by ge	---	AT1G35880	-1.13	-0.17	-1.60	0.154377	0.271743	-6.00
251168_at	putative protein several hypothetica	---	AT3G63370	-1.16	-0.21	-1.60	0.154388	0.271743	-6.00
246625_at	hypothetical protein predicted by ge	---	AT1G48880	-1.14	-0.19	-1.60	0.154398	0.271743	-6.00
250965_at	putative protein various predicted p	---	AT5G03020	-1.12	-0.16	-1.60	0.154409	0.271743	-6.00
245995_at	putative protein various predicted p	---	---	-1.20	-0.26	-1.60	0.154481	0.271844	-6.01
248782_at	palmitoyl-protein thioesterase precu	---	AT5G47340	-1.14	-0.19	-1.60	0.154501	0.271844	-6.01
249698_at	unknown protein	MIS12	AT5G35520	-1.20	-0.26	-1.60	0.154513	0.271844	-6.01
245418_at	putative protein	---	AT4G17370	1.19	0.25	1.60	0.154514	0.271844	-6.01
246456_at	telomerase reverse transcriptase ;	ATTERT	AT5G16850	-1.12	-0.16	-1.60	0.154537	0.271863	-6.01
255246_at	putative protein hypothetical protei	---	---	-1.16	-0.22	-1.60	0.154631	0.272008	-6.01
254768_at	hypothetical protein	---	AT4G13320	-1.15	-0.21	-1.60	0.154716	0.272137	-6.01
245340_at	elicitor like protein ; supported by fu	---	AT4G14420	1.21	0.28	1.60	0.154775	0.27222	-6.01
263613_at	unknown protein	---	AT2G25250	-1.30	-0.38	-1.60	0.154791	0.272226	-6.01
256689_at	hypothetical protein predicted by ge	---	AT3G32150	-1.12	-0.16	-1.60	0.154907	0.272397	-6.01
260998_at	CAF protein similar to C2H2 zinc fir	---	AT1G26590	-1.14	-0.18	-1.60	0.154912	0.272397	-6.01
249232_at	dynamin-like protein (pir||S59558) ;	ADL1	AT5G42080	1.20	0.26	1.60	0.154938	0.272423	-6.01
263367_at	putative retroelement pol polyprotei	---	---	-1.15	-0.21	-1.60	0.154972	0.272447	-6.01
265473_at	Expressed protein ; supported by fu	LCR10	AT2G15535	-1.14	-0.19	-1.60	0.154976	0.272447	-6.01
247789_at	putative protein various predicted p	---	AT5G58680	-1.16	-0.21	-1.60	0.155009	0.272484	-6.01
259654_at	hypothetical protein predicted by ge	---	AT1G55220	-1.15	-0.20	-1.60	0.155038	0.272513	-6.01
251629_at	villin 3 fragment ; supported by cDN	VLN3	AT3G57410	1.18	0.24	1.60	0.155095	0.272594	-6.01
265651_at	hypothetical protein predicted by ge	---	AT2G13900	1.12	0.17	1.60	0.155168	0.2727	-6.01
260286_at	putative acetylornithine transaminas	WIN1	AT1G80600	1.17	0.23	1.60	0.155199	0.272735	-6.01
247011_at	putative protein contains similarity t	FRO1	AT5G67590	1.24	0.31	1.60	0.155219	0.272748	-6.01
261693_at	hypothetical protein similar to hypot	DCP1	AT1G08370	1.11	0.15	1.60	0.155261	0.272801	-6.01
250514_at	GDP dissociation inhibitor GDP dis	---	AT5G09550	-1.12	-0.17	-1.60	0.155311	0.272867	-6.01

257057_at	hypothetical protein predicted by ge	---	---	-1.10	-0.13	-1.60	0.155384	0.272975	-6.01
248804_at	nodulin-like protein	---	AT5G47470	-1.14	-0.19	-1.60	0.155413	0.273005	-6.01
264543_at	hypothetical protein predicted by ge	---	AT1G55780	1.17	0.23	1.60	0.155473	0.273051	-6.01
259465_at	hypothetical protein similar to putati	---	AT1G19025	-1.15	-0.20	-1.60	0.155474	0.273051	-6.01
253283_at	hypothetical protein ; supported by	---	AT4G34090	-1.20	-0.27	-1.60	0.155475	0.273051	-6.01
265556_at	putative Athila retroelement ORF1-l	---	---	-1.14	-0.19	-1.60	0.155636	0.273313	-6.01
245142_at	putative O-sialoglycoprotein endope	---	AT2G45270	-1.14	-0.19	-1.60	0.155649	0.273315	-6.01
250051_at	MYB56 R2R3-MYB factor family me	AtMYB56	AT5G17800	-1.18	-0.24	-1.60	0.155716	0.273411	-6.01
245029_at	hypothetical protein predicted by ge	YAB5	AT2G26580	-1.36	-0.44	-1.60	0.155853	0.273618	-6.01
254021_at	putative protein cell death suppres	ACD1-LIKE	AT4G25650	-1.16	-0.22	-1.60	0.155858	0.273618	-6.01
258010_at	unknown protein similar to receptor	---	AT3G19300	-1.12	-0.17	-1.60	0.155884	0.273634	-6.01
250488_at	beta-glucosidase - like protein beta	---	AT5G09700	-1.13	-0.18	-1.60	0.155891	0.273634	-6.01
248688_at	indole-3-glycerol phosphate syntha	---	AT5G48220	-1.19	-0.25	-1.60	0.155944	0.273706	-6.01
247039_at	putative protein contains similarity t	ATEB1C	AT5G67270	-1.19	-0.25	-1.60	0.155974	0.273737	-6.01
247485_at	putative protein ARR1, Arabidopsis	ARR23	AT5G62120	-1.11	-0.15	-1.60	0.156078	0.273899	-6.01
253420_at	H+-transporting ATP synthase cha	---	AT4G32260	-1.16	-0.21	-1.60	0.156168	0.274031	-6.02
248347_at	putative protein contains similarity t	---	AT5G52250	-1.11	-0.16	-1.60	0.156177	0.274031	-6.02
245587_at	putative protein	---	AT4G15020	1.25	0.32	1.60	0.156237	0.274096	-6.02
254396_at	peptide transporter - like protein pe	---	AT4G21680	1.94	0.95	1.60	0.156239	0.274096	-6.02
266102_at	unknown protein	---	AT2G40720	1.14	0.19	1.60	0.156263	0.274119	-6.02
266376_at	putative endoxyloglucan glycosyltra	---	AT2G14620	-1.18	-0.23	-1.60	0.156302	0.274165	-6.02
246554_at	HSP100/ClpB, putative similar to H	CLPB3	AT5G15450	1.15	0.20	1.60	0.156314	0.274165	-6.02
262894_at	disease resistance protein, putative	---	AT1G59780	-1.15	-0.20	-1.59	0.156562	0.274579	-6.02
262739_at	lipase, putative contains Pfam prof	---	AT1G28650	1.25	0.33	1.59	0.15671	0.274818	-6.02
266099_at	putative alpha-carboxyltransferase	CAC3	AT2G38040	-1.20	-0.26	-1.59	0.156808	0.274969	-6.02
262142_at	hypothetical protein predicted by ge	---	AT1G52640	1.15	0.20	1.59	0.156842	0.275007	-6.02
259290_at	putative cyclin similar to cyclin cyc1	CYCB1;3	AT3G11520	-1.20	-0.26	-1.59	0.156902	0.27509	-6.02
266468_at	unknown protein ; supported by cDI	---	AT2G47960	1.16	0.21	1.59	0.15701	0.275259	-6.02
259250_at	unknown protein	---	AT3G07580	-1.22	-0.29	-1.59	0.157052	0.275312	-6.02
256608_at	hypothetical protein predicted by ge	---	AT3G30840	-1.10	-0.14	-1.59	0.157079	0.275321	-6.02
256619_at	hypothetical protein predicted by ge	---	AT3G24460	-1.13	-0.17	-1.59	0.157082	0.275321	-6.02
256258_at	unknown protein	NF-YC11	AT3G12480	1.11	0.15	1.59	0.157119	0.275346	-6.02
258314_at	putative RAS-related GTP-binding p	ATRABG3C	AT3G16100	1.15	0.20	1.59	0.15712	0.275346	-6.02
247764_at	subtilisin-like serine protease conta	---	AT5G59190	-1.14	-0.19	-1.59	0.157149	0.275376	-6.02
264359_at	hypothetical protein predicted by ge	---	AT1G03320	-1.15	-0.21	-1.59	0.157256	0.275531	-6.02

249304_at	folylpolyglutamate synthase-like pro	GLA1	AT5G41480	1.19	0.25	1.59	0.157266	0.275531	-6.02
249465_at	AIG2 - like protein AIG2, Arabidops	AIG2L	AT5G39720	-1.15	-0.20	-1.59	0.157274	0.275531	-6.02
251238_at	putative protein several hypothetica	---	AT3G62430	1.15	0.21	1.59	0.157303	0.275561	-6.02
259555_at	hypothetical protein	---	---	-1.12	-0.17	-1.59	0.157334	0.275594	-6.02
261338_at	hypothetical protein predicted by ge	---	AT1G44920	-1.16	-0.22	-1.59	0.157371	0.275639	-6.02
265242_at	hypothetical protein	---	AT2G07705	1.25	0.33	1.59	0.157473	0.275795	-6.02
255939_at	hypothetical protein predicted by ge	---	AT1G12730	1.20	0.27	1.59	0.157502	0.275824	-6.02
267415_at	putative translation initiation factor e	---	AT2G34970	1.11	0.15	1.59	0.157513	0.275824	-6.02
263293_x_a	hypothetical protein similar to hypot	---	---	-1.12	-0.16	-1.59	0.157549	0.275866	-6.02
258131_at	protein kinase, putative similar to G	---	AT3G24540	-1.19	-0.25	-1.59	0.157571	0.275882	-6.02
251977_at	putative protein auxin-induced prot	---	AT3G53250	-1.11	-0.15	-1.59	0.157607	0.275925	-6.02
249010_at	unknown protein ; supported by cD	---	AT5G44580	1.25	0.32	1.59	0.157632	0.275947	-6.02
255737_at	unknown protein ;supported by full-	---	AT1G25420	-1.14	-0.20	-1.59	0.157705	0.276043	-6.02
264366_at	unknown protein EST gb|N96383 co	---	AT1G03250	1.18	0.23	1.59	0.157719	0.276043	-6.02
264430_at	cineole synthase, putative similar to	TPS14	AT1G61680	1.13	0.17	1.59	0.157723	0.276043	-6.02
265641_at	putative RNA-binding glycine rich p	---	AT2G27330	1.16	0.21	1.59	0.157891	0.276315	-6.03
245628_at	anthocyanin2, putative similar to ar	PAP1	AT1G56650	1.22	0.29	1.59	0.15807	0.276608	-6.03
248506_at	adenylate kinase ;supported by full	---	AT5G50370	1.39	0.48	1.59	0.158153	0.276732	-6.03
265224_at	putative pectinesterase	---	AT2G36710	-1.13	-0.18	-1.59	0.158215	0.276819	-6.03
248009_at	transcription factor-like; similar to C	CSN6A	AT5G56280	-1.13	-0.18	-1.59	0.158325	0.27694	-6.03
267275_at	putative Na H antiporter	ATCHX13	AT2G30240	-1.18	-0.23	-1.59	0.15836	0.27694	-6.03
250276_at	putative protein mRNA, Moritella m	---	AT5G13030	1.14	0.18	1.59	0.158369	0.27694	-6.03
248476_at	putative protein similar to unknown	---	AT5G50890	-1.32	-0.40	-1.59	0.158384	0.27694	-6.03
265855_at	unknown protein ;supported by full-	---	AT2G42390	1.12	0.16	1.59	0.158391	0.27694	-6.03
248577_at	myrosinase binding protein-like ; su	---	AT5G49870	-1.11	-0.15	-1.59	0.158395	0.27694	-6.03
258941_at	putative monodehydroascorbate red	MDHAR	AT3G09940	-2.62	-1.39	-1.59	0.158396	0.27694	-6.03
258075_at	homocysteine S-methyltransferase	HMT-1	AT3G25900	1.82	0.86	1.59	0.158401	0.27694	-6.03
249751_at	putative protein similar to unknown	---	AT5G24650	1.17	0.23	1.59	0.158402	0.27694	-6.03
247242_at	unknown protein	---	AT5G64690	-1.11	-0.16	-1.59	0.158414	0.27694	-6.03
261031_at	hypothetical protein predicted by ge	---	AT1G17360	-1.28	-0.36	-1.59	0.158429	0.27694	-6.03
259978_at	putative cell division control protein	CDKB2;1	AT1G76540	-1.22	-0.28	-1.59	0.15843	0.27694	-6.03
258233_at	hypothetical protein contains similar	MYB118	AT3G27785	-1.14	-0.19	-1.59	0.158446	0.276946	-6.03
249165_at	putative protein similar to unknown	ATIPK1	AT5G42810	-1.20	-0.27	-1.59	0.158489	0.277	-6.03
247515_at	ABC family transporter - like protei	ATH14	AT5G61740	-1.11	-0.14	-1.59	0.158539	0.277067	-6.03
249113_at	putative protein similar to unknown	---	AT5G43790	1.19	0.25	1.58	0.158745	0.277406	-6.03

255589_s_a	hypothetical protein	---	AT4G01590	1.20	0.27	1.58	0.158795	0.277471	-6.03
256517_at	PHD-finger protein, putative contai	MMD1	AT1G66170	-1.11	-0.15	-1.58	0.15886	0.277563	-6.03
251090_at	putative protein ACR1-protein - Sa	---	AT5G01340	1.45	0.54	1.58	0.158913	0.277635	-6.03
263726_at	putative ABC transporter ;supporte	---	AT2G13610	-1.75	-0.81	-1.58	0.158926	0.277637	-6.03
247260_at	putative protein contains similarity t	---	AT5G64500	-1.20	-0.26	-1.58	0.159097	0.277914	-6.03
265176_at	OBP32pep protein, putative identic	---	AT1G23520	-1.12	-0.17	-1.58	0.159111	0.277918	-6.03
265262_at	putative chloroplast nucleoid DNA b	---	AT2G42980	1.17	0.23	1.58	0.159159	0.277979	-6.03
249804_at	galactinol synthase	AtGolS5	AT5G23790	-1.12	-0.16	-1.58	0.159198	0.278026	-6.03
254354_at	hypothetical protein	---	AT4G22370	-1.10	-0.14	-1.58	0.159261	0.278105	-6.03
247973_at	putative protein contains similarity t	---	AT5G56770	-1.12	-0.17	-1.58	0.159267	0.278105	-6.03
254882_s_a	putative protein various predicted r	---	---	-1.12	-0.17	-1.58	0.15933	0.278194	-6.03
263151_at	hypothetical protein predicted by ge	---	AT1G54120	1.38	0.47	1.58	0.159436	0.278357	-6.03
249288_at	unknown protein ;supported by full-	---	AT5G41050	1.47	0.55	1.58	0.15952	0.278457	-6.03
245114_at	transcription factor IIB (TFIIB) ident	TFIIB	AT2G41630	-1.24	-0.31	-1.58	0.15953	0.278457	-6.03
257212_at	zinc-binding dehydrogenase, putat	---	AT3G15090	1.19	0.25	1.58	0.15953	0.278457	-6.03
259274_at	putative homeobox-leucine zipper	ATHB20	AT3G01220	-1.12	-0.17	-1.58	0.159632	0.278614	-6.04
257642_at	putative HLH DNA-binding protein c	BHLH32	AT3G25710	-1.17	-0.23	-1.58	0.159654	0.278631	-6.04
256915_at	unknown protein	---	AT3G23900	1.16	0.21	1.58	0.159696	0.278682	-6.04
247711_at	receptor-like protein kinase recepto	---	AT5G59270	-1.14	-0.19	-1.58	0.159716	0.278687	-6.04
261609_at	unknown protein similar to MAP3K-	---	AT1G49740	-1.23	-0.30	-1.58	0.159723	0.278687	-6.04
261863_at	unknown protein ; supported by cDI	---	AT1G50630	-1.18	-0.24	-1.58	0.159735	0.278687	-6.04
251271_at	hypothetical protein ; supported by	---	AT3G62050	-1.19	-0.25	-1.58	0.159756	0.278702	-6.04
250579_at	putative protein terminal ear1, Zea	MCT2	AT5G07930	-1.12	-0.17	-1.58	0.159782	0.278727	-6.04
267439_at	putative GDSL-motif lipase/hydrolas	---	AT2G19060	-1.15	-0.20	-1.58	0.159858	0.278827	-6.04
261217_at	hypothetical protein contains simila	UBP11	AT1G32850	-1.11	-0.15	-1.58	0.159864	0.278827	-6.04
262320_at	hypothetical protein predicted by ge	---	AT1G27550	-1.13	-0.18	-1.58	0.15988	0.278833	-6.04
262123_at	hypothetical protein predicted by ge	---	AT1G59630	-1.16	-0.21	-1.58	0.159935	0.278909	-6.04
265566_at	hypothetical protein predicted by ge	---	AT2G05600	-1.15	-0.20	-1.58	0.159959	0.278929	-6.04
257079_at	unknown protein	---	AT3G15240	-1.12	-0.16	-1.58	0.16	0.278978	-6.04
258242_at	hypothetical protein predicted by ge	---	AT3G27640	-1.13	-0.17	-1.58	0.16005	0.279045	-6.04
246560_at	putative protein hypothetical protein	---	AT5G15560	-1.12	-0.17	-1.58	0.160067	0.279046	-6.04
255881_at	phosphomannose isomerase, puta	DIN9	AT1G67070	1.48	0.56	1.58	0.160082	0.279046	-6.04
255001_at	AIG1-like protein AIG1 - Arabidops	---	AT4G09930	-1.17	-0.23	-1.58	0.160087	0.279046	-6.04
262829_at	auxin-independent growth promote	---	AT1G14970	-1.26	-0.33	-1.58	0.16015	0.279127	-6.04
266133_at	putative C2H2-type zinc finger prote	---	AT2G45120	1.13	0.17	1.58	0.160158	0.279127	-6.04

263830_at	hypothetical protein predicted by ge	---	AT2G40260	-1.13	-0.18	-1.58	0.160191	0.279162	-6.04
255031_at	putative proteins various hypothetic	---	AT4G09490	1.18	0.23	1.58	0.160356	0.279428	-6.04
256735_at	hypothetical protein predicted by ge	ATEXO70E	AT3G29400	1.16	0.22	1.58	0.16039	0.279467	-6.04
253429_at	putative protein CDC28/cdc2-like k	---	AT4G32420	1.13	0.18	1.58	0.160403	0.279468	-6.04
253895_at	NWMU3 - 2S albumin 3 precursor	AT2S3	AT4G27160	-1.11	-0.16	-1.58	0.160479	0.279579	-6.04
248160_at	putative protein similar to unknown	---	AT5G54470	-1.20	-0.26	-1.58	0.160527	0.279642	-6.04
259579_at	P-glycoprotein, putative similar to F	PGP14	AT1G28010	-1.56	-0.65	-1.58	0.16058	0.279713	-6.04
260953_at	transcriptional activator RF2a, puta	---	AT1G06070	-1.14	-0.19	-1.58	0.16062	0.27976	-6.04
264659_at	unknown protein Similar to S. pomb	ATOPT2	AT1G09930	-1.12	-0.17	-1.58	0.160648	0.279789	-6.04
254062_at	putative protein zinc finger protein	---	AT4G25380	1.55	0.64	1.58	0.160778	0.279993	-6.04
266316_at	unknown protein ; supported by cD	---	AT2G27080	-1.85	-0.89	-1.58	0.160809	0.280005	-6.04
262548_at	unknown protein contains similarity	AGO2	AT1G31280	-1.16	-0.21	-1.58	0.16081	0.280005	-6.04
247878_at	unknown protein	---	AT5G57760	-2.91	-1.54	-1.58	0.160829	0.280018	-6.04
264717_at	hypothetical protein predicted by ge	ATFH8	AT1G70140	1.29	0.37	1.58	0.160854	0.280039	-6.04
265185_at	OBP32pep protein, putative identic	---	AT1G23670	-1.15	-0.20	-1.58	0.160874	0.280054	-6.04
249163_at	glucose-6-phosphate isomerase, c	---	AT5G42740	1.18	0.24	1.57	0.160984	0.280223	-6.04
263069_at	unknown protein	---	AT2G17590	-1.27	-0.35	-1.57	0.16101	0.280247	-6.04
249992_at	AP2 domain -like protein AP2/ERE	PUCHI	AT5G18560	-1.15	-0.20	-1.57	0.16111	0.2804	-6.04
256105_at	hypothetical protein predicted by ge	LSH8	AT1G16910	-1.15	-0.20	-1.57	0.161145	0.280418	-6.04
252944_at	hypothetical protein ;supported by	---	AT4G39320	-1.12	-0.17	-1.57	0.161145	0.280418	-6.04
257032_at	zinc finger protein, putative similar	---	AT3G19140	-1.10	-0.14	-1.57	0.161161	0.280425	-6.04
260387_at	putative flavonol sulfotransferase si	SOT16	AT1G74100	-1.36	-0.45	-1.57	0.161262	0.280578	-6.04
265509_at	putative retroelement pol polyprotei	---	---	-1.18	-0.24	-1.57	0.161324	0.280665	-6.05
259114_at	26S proteasome AAA-ATPase subu	RPT5A	AT3G05530	1.12	0.17	1.57	0.161336	0.280665	-6.05
256843_at	hypothetical protein predicted by ge	---	---	-1.13	-0.18	-1.57	0.161451	0.280844	-6.05
245520_at	delta-cadinene synthase like protein	ATTS1	AT4G15870	-1.13	-0.18	-1.57	0.161506	0.280913	-6.05
253692_at	putative protein Cs protein, Drosop	ATPAO5	AT4G29720	1.11	0.15	1.57	0.161516	0.280913	-6.05
248906_at	putative protein similar to unknown	---	AT5G46420	1.13	0.17	1.57	0.161549	0.280929	-6.05
258625_at	hypothetical protein predicted by ge	PDLP4	AT3G04370	-1.13	-0.18	-1.57	0.161549	0.280929	-6.05
264944_at	hypothetical protein similar to hypot	---	AT1G76940	-1.19	-0.25	-1.57	0.161612	0.281009	-6.05
258500_at	S-adenosylmethionine decarboxyla	CPuORF9	AT3G02468	1.14	0.19	1.57	0.161627	0.281009	-6.05
259004_at	putative protein kinase C-terminal r	---	AT3G01840	-1.13	-0.18	-1.57	0.161632	0.281009	-6.05
255748_at	hypothetical protein predicted by ge	---	AT1G32000	-1.10	-0.14	-1.57	0.161704	0.281104	-6.05
256686_at	non-LTR reverse transcriptase, put	---	---	-1.10	-0.14	-1.57	0.161712	0.281104	-6.05
259951_at	unknown protein	---	AT1G71490	-1.12	-0.16	-1.57	0.161756	0.28116	-6.05

246221_at	step II splicing factor - like protein ;	SMP2	AT4G37120	1.14	0.18	1.57	0.161784	0.281166	-6.05
266397_at	unknown protein	---	AT2G38690	-1.12	-0.16	-1.57	0.161785	0.281166	-6.05
256164_at	terpene cyclase, putative similar to	---	AT1G48800	1.11	0.15	1.57	0.161799	0.281169	-6.05
249542_at	putative protein transcription factor	NF-YC12	AT5G38140	-1.19	-0.25	-1.57	0.161953	0.281416	-6.05
255852_at	unknown protein	SVL2	AT1G66970	-1.42	-0.50	-1.57	0.161985	0.281451	-6.05
265146_at	putative glucosyl transferase	---	AT1G51210	-1.27	-0.35	-1.57	0.162037	0.281518	-6.05
248627_at	putative protein similar to unknown	---	AT5G48950	-1.18	-0.24	-1.57	0.162158	0.281692	-6.05
261466_at	hypothetical protein predicted by ge	---	AT1G07690	-1.18	-0.24	-1.57	0.162161	0.281692	-6.05
252394_at	heat shock protein-like protein hea	---	AT3G47940	-1.19	-0.25	-1.57	0.162185	0.281711	-6.05
266161_at	hypothetical protein	ATCHX7	AT2G28170	-1.18	-0.24	-1.57	0.162208	0.281731	-6.05
246210_at	ribosomal protein ; supported by cD	---	AT4G36420	1.21	0.27	1.57	0.162226	0.281741	-6.05
258208_at	putative acetyltransferase similar to	---	AT3G13930	1.21	0.28	1.57	0.162276	0.281776	-6.05
264981_at	valyl- tRNA synthetase, putative si	---	AT1G27160	-1.15	-0.20	-1.57	0.162279	0.281776	-6.05
262560_at	hypothetical protein predicted by ge	---	AT1G34280	-1.15	-0.20	-1.57	0.162284	0.281776	-6.05
263620_at	hypothetical protein predicted by ge	---	---	-1.12	-0.17	-1.57	0.16243	0.281985	-6.05
256835_at	ATP sulfurylase, putative similar to	APS1	AT3G22890	-1.29	-0.36	-1.57	0.162441	0.281985	-6.05
248129_at	putative protein contains similarity t	---	AT5G54780	1.15	0.20	1.57	0.162446	0.281985	-6.05
255029_x_a	hypothetical protein	---	AT4G09470	-1.10	-0.14	-1.57	0.162454	0.281985	-6.05
245234_at	myb-like protein similarity to ATMY	AtMYB18	AT4G25560	-1.15	-0.20	-1.57	0.162473	0.281992	-6.05
256659_at	unknown protein similar to putative	---	AT3G12020	-1.14	-0.19	-1.57	0.162488	0.281992	-6.05
266483_at	unknown protein ;supported by full-	CRR6	AT2G47910	1.16	0.21	1.57	0.162495	0.281992	-6.05
264504_at	unknown protein similar to ATP-citr	ACLA-3	AT1G09430	1.13	0.17	1.57	0.162549	0.282047	-6.05
264719_at	receptor-like kinase, putative simila	---	AT1G70110	-1.20	-0.26	-1.57	0.162551	0.282047	-6.05
254070_at	hypothetical protein	---	AT4G25430	-1.20	-0.26	-1.57	0.162625	0.28209	-6.05
262799_at	hypothetical protein predicted by ge	---	AT1G20970	-1.22	-0.29	-1.57	0.162625	0.28209	-6.05
252544_at	respiratory burst oxidase - like prot	---	AT3G45810	-1.16	-0.21	-1.57	0.162628	0.28209	-6.05
247805_at	ATGP1-like protein	ATYKT62	AT5G58180	-1.21	-0.28	-1.57	0.162632	0.28209	-6.05
256501_at	hypothetical protein predicted by ge	---	AT1G36756	-1.13	-0.17	-1.57	0.162638	0.28209	-6.05
259216_at	hypothetical protein ; supported by	---	AT3G09000	1.30	0.38	1.57	0.162664	0.282114	-6.05
263858_at	hypothetical protein predicted by ge	---	AT2G04370	-1.12	-0.16	-1.57	0.162686	0.28213	-6.05
267621_at	unknown protein	---	---	1.16	0.22	1.57	0.162742	0.282206	-6.05
259511_at	unknown protein contains similarity	ATCCS	AT1G12520	1.47	0.56	1.57	0.162794	0.282275	-6.05
250974_at	meiosis specific - like protein Spo1	RHL2	AT5G02820	1.31	0.39	1.57	0.162812	0.282285	-6.05
258598_at	hypothetical protein predicted by ge	---	AT3G04410	-1.18	-0.24	-1.57	0.162854	0.282337	-6.05
247994_at	RNA-binding protein-like	---	AT5G56140	-1.16	-0.21	-1.57	0.162875	0.282351	-6.05

256990_at	hypothetical protein predicted by ge	---	AT3G28590	-1.19	-0.26	-1.57	0.162902	0.282377	-6.05
252725_at	putative protein mudrA transposase	---	---	-1.12	-0.17	-1.57	0.16295	0.282438	-6.05
264808_at	unknown protein similar to EST gb|I	PRA1.E	AT1G08770	-1.13	-0.17	-1.57	0.163003	0.282509	-6.05
247115_at	kinesin-like calmodulin-binding prot	ZWI	AT5G65930	-1.17	-0.22	-1.57	0.16307	0.282599	-6.06
261445_at	unknown protein ; supported by cDI	NSL1	AT1G28380	-1.35	-0.43	-1.57	0.16308	0.282599	-6.06
257063_s_a	unknown protein ;supported by full-	---	AT3G18240	1.11	0.15	1.57	0.1631	0.282609	-6.06
260394_at	putative transcription factor similar t	MYB122	AT1G74080	1.87	0.90	1.57	0.16311	0.282609	-6.06
245498_at	hypothetical protein	---	AT4G16470	1.11	0.16	1.57	0.163217	0.282771	-6.06
265628_at	hypothetical protein predicted by ge	---	AT2G27290	-1.24	-0.31	-1.56	0.163305	0.282903	-6.06
260710_at	protein phosphatase 2C, putative s	---	AT1G17545	-1.11	-0.15	-1.56	0.163329	0.282923	-6.06
257455_s_a	hypothetical protein	---	AT1G27870	-1.11	-0.15	-1.56	0.163354	0.282945	-6.06
263701_at	putative protein kinase C inhibitor (Z	---	AT1G31160	-1.11	-0.15	-1.56	0.163495	0.283168	-6.06
265395_at	putative LRR receptor protein kinas	SRF1	AT2G20850	1.19	0.25	1.56	0.163518	0.283186	-6.06
259893_at	putative disease resistance protein	AtRLP11	AT1G71390	-1.15	-0.20	-1.56	0.163611	0.283326	-6.06
245814_at	mitotic checkpoint protein, putative	---	AT1G49910	-1.16	-0.22	-1.56	0.163657	0.283383	-6.06
246067_at	membrane transporter - like protein	---	AT5G19410	-1.11	-0.16	-1.56	0.163686	0.283413	-6.06
251695_at	putative protein hypothetical protein	---	AT3G56590	-1.23	-0.30	-1.56	0.163777	0.283549	-6.06
AFFX-r2-Bs	B. subtilis /GEN=lys /DB_XREF=gb	---	---	1.25	0.32	1.56	0.163803	0.283563	-6.06
252594_at	putative transporter protein peptide	---	AT3G45680	-1.24	-0.31	-1.56	0.163815	0.283563	-6.06
262583_at	putative phosphatidylserine synthas	---	AT1G15110	1.18	0.24	1.56	0.163823	0.283563	-6.06
260594_at	hypothetical protein predicted by ge	---	AT1G55880	1.10	0.14	1.56	0.163947	0.283753	-6.06
266737_at	putative alcohol dehydrogenase ; s	---	AT2G47140	-1.15	-0.20	-1.56	0.163957	0.283753	-6.06
266759_at	unknown protein predicted by gene	---	AT2G46915	-1.13	-0.18	-1.56	0.163986	0.283782	-6.06
253481_at	aspartate aminotransferase ;suppo	ASP5	AT4G31990	1.14	0.18	1.56	0.164023	0.283812	-6.06
251462_at	mitogen-activated protein kinase-lik	ATMPK10	AT3G59790	-1.16	-0.22	-1.56	0.164029	0.283812	-6.06
255163_at	putative polyprotein	---	---	1.12	0.17	1.56	0.164067	0.283856	-6.06
266787_at	putative receptor-like protein kinase	---	AT2G28990	-1.11	-0.15	-1.56	0.164146	0.283972	-6.06
263960_at	putative kinesin-related cytokinesis	---	AT2G36200	-1.14	-0.18	-1.56	0.164198	0.28404	-6.06
264090_at	cytochrome P-450, putative similar	CYP79C1	AT1G79370	-1.13	-0.18	-1.56	0.164247	0.284103	-6.06
267189_at	putative methionine aminopeptidase	MAP2A	AT2G44180	1.32	0.40	1.56	0.164436	0.284409	-6.06
265523_at	putative Ty3-gypsy-like retroelemen	---	---	-1.11	-0.15	-1.56	0.164476	0.284453	-6.06
248030_at	transcription regulator Sir2-like prot	SRT1	AT5G55760	1.19	0.25	1.56	0.164486	0.284453	-6.06
250020_at	putative protein contains similarity t	---	AT5G18180	-1.16	-0.21	-1.56	0.164591	0.284574	-6.06
260637_at	ACC oxidase, putative similar to AC	ACO2	AT1G62380	1.46	0.55	1.56	0.164592	0.284574	-6.06
255285_at	putative protein A. thaliana hypothe	---	AT4G04630	-1.48	-0.57	-1.56	0.164594	0.284574	-6.06

253400_at	putative protein unknown protein T	---	AT4G32860	-1.12	-0.16	-1.56	0.164647	0.284645	-6.06
258745_at	unknown protein ; supported by full-	---	AT3G05920	-1.14	-0.19	-1.56	0.164829	0.284937	-6.07
245689_at	phosphoglycerate mutase - like pro	---	AT5G04120	-1.14	-0.19	-1.56	0.164897	0.285034	-6.07
248675_at	putative protein similar to unknown	ICK6	AT5G48820	-1.23	-0.30	-1.56	0.164928	0.285047	-6.07
252658_at	hypothetical protein	---	AT3G44800	-1.17	-0.23	-1.56	0.16493	0.285047	-6.07
262217_at	hypothetical protein predicted by ge	---	AT1G74770	-1.10	-0.14	-1.56	0.164964	0.285079	-6.07
249754_at	flavanone 3-hydroxylase-like protei	DMR6	AT5G24530	-1.82	-0.86	-1.56	0.164974	0.285079	-6.07
256586_at	hypothetical protein	---	AT3G28770	-1.11	-0.15	-1.56	0.164996	0.285097	-6.07
250796_at	putative protein similar to unknown	---	AT5G05300	1.78	0.83	1.56	0.165024	0.285122	-6.07
262028_at	DNA binding protein, putative simil	---	AT1G35560	1.12	0.17	1.56	0.165097	0.285228	-6.07
261146_at	hypothetical protein predicted by ge	---	AT1G19620	-1.18	-0.24	-1.56	0.165146	0.285291	-6.07
263270_at	putative replication protein A1	---	---	-1.12	-0.16	-1.56	0.165182	0.285331	-6.07
263974_at	hypothetical protein predicted by gr	---	AT2G42720	-1.13	-0.18	-1.56	0.16521	0.285358	-6.07
262007_at	hypothetical protein predicted by ge	---	AT1G64580	-1.13	-0.18	-1.56	0.16534	0.285561	-6.07
262833_at	cyclin, putative contains Pfam profi	SDS	AT1G14750	-1.11	-0.15	-1.56	0.165428	0.285691	-6.07
246224_at	putative MADS-box protein ;suppor	---	AT4G36590	-1.18	-0.24	-1.56	0.165542	0.285867	-6.07
247128_at	atfp6-like protein	---	AT5G66110	-1.19	-0.25	-1.55	0.165621	0.285962	-6.07
255233_at	putative protein ADRENODOXIN F	MFDX1	AT4G05450	1.14	0.19	1.55	0.165622	0.285962	-6.07
262337_at	hypothetical protein similar to hypot	---	AT1G64260	-1.16	-0.21	-1.55	0.165699	0.286073	-6.07
255997_s_a	photosystem II type I chlorophyll a	CAB3	AT1G29910 ,	-1.20	-0.27	-1.55	0.165741	0.286124	-6.07
263672_at	putative retroelement pol polyprotei	---	---	-1.11	-0.15	-1.55	0.165785	0.286178	-6.07
267536_at	phospholipase D ; supported by cD	PLDBETA1	AT2G42010	-1.15	-0.20	-1.55	0.16586	0.286287	-6.07
258473_s_a	putative stearoyl-acyl carrier protein	---	AT3G02610 ,	1.22	0.28	1.55	0.165933	0.286364	-6.07
267563_at	putative protein kinase contains a p	---	AT2G30730	-1.13	-0.17	-1.55	0.165936	0.286364	-6.07
265893_at	putative disease resistance protein	---	AT2G15040 ,	1.39	0.47	1.55	0.165943	0.286364	-6.07
260980_at	hypothetical protein predicted by ge	---	AT1G53600	-1.19	-0.25	-1.55	0.165977	0.286402	-6.07
253718_at	serine/threonine-specific receptor p	---	AT4G29450	-1.15	-0.20	-1.55	0.166003	0.286423	-6.07
263307_at	hypothetical protein similar to myos	---	---	1.14	0.19	1.55	0.166039	0.286464	-6.07
262477_at	hypothetical protein contains simila	---	AT1G11220	-1.20	-0.27	-1.55	0.166076	0.286492	-6.07
250120_at	putative protein similar to unknown	RIC4	AT5G16490	-1.22	-0.28	-1.55	0.166082	0.286492	-6.07
245848_at	putative protein predicted proteins -	FY	AT5G13480	1.13	0.17	1.55	0.166093	0.286492	-6.07
264437_at	unknown protein EST gb|N65787 c	---	AT1G27510	1.14	0.19	1.55	0.166175	0.286613	-6.07
247656_at	actin depolymerizing factor 4 - like	ADF4	AT5G59890	-1.17	-0.22	-1.55	0.166234	0.286692	-6.07
259231_at	protein phosphatase 2C (PP2C) ide	PP2CA	AT3G11410	1.26	0.33	1.55	0.166377	0.286909	-6.07
260111_at	hypothetical protein similar to unkno	---	AT1G63320	1.10	0.14	1.55	0.166385	0.286909	-6.07

247980_at	putative protein similar to unknown	GNC	AT5G56860	-1.41	-0.50	-1.55	0.166482	0.287056	-6.07
249031_at	putative protein contains similarity t	---	AT5G44900	-1.15	-0.20	-1.55	0.166535	0.287123	-6.07
253536_at	RSZp22 splicing factor ;supported b	SRZ-22	AT4G31580	1.10	0.14	1.55	0.166546	0.287123	-6.07
254874_at	putative protein beta-phosphogluco	---	AT4G11570	-1.18	-0.23	-1.55	0.16656	0.287124	-6.07
251511_at	putative protein several hypothetica	---	AT3G59180	-1.12	-0.16	-1.55	0.166588	0.287151	-6.07
254237_at	cysteine proteinase contains simila	---	AT4G23520	-1.11	-0.15	-1.55	0.166686	0.287279	-6.08
257727_at	unknown protein	---	AT3G18380	-1.13	-0.17	-1.55	0.166687	0.287279	-6.08
251835_at	lipase -like protein monoglyceride l	---	AT3G55180	-1.12	-0.16	-1.55	0.166774	0.287406	-6.08
249409_at	putative protein KED, Nicotiana tab	---	AT5G40340	-1.46	-0.54	-1.55	0.166789	0.287411	-6.08
267228_at	putative polygalacturonase	---	AT2G43890	-1.11	-0.15	-1.55	0.166865	0.287514	-6.08
257895_at	dihydrolipoamide dehydrogenase,	LPD1	AT3G16950	1.28	0.36	1.55	0.166877	0.287514	-6.08
267297_at	putative RING zinc finger protein ;s	---	AT2G23780	-1.14	-0.19	-1.55	0.166887	0.287514	-6.08
251344_at	putative protein beige-like protein ((	---	AT3G60920	-1.13	-0.18	-1.55	0.166924	0.287556	-6.08
254509_at	terpene cyclase like protein 5-epi-a	---	AT4G20200	-1.12	-0.16	-1.55	0.166977	0.287627	-6.08
265367_at	hypothetical protein predicted by ge	---	---	1.10	0.14	1.55	0.167017	0.287661	-6.08
252471_at	phosphoprotein phosphatase ;supp	TOPP5	AT3G46820	-1.14	-0.19	-1.55	0.167022	0.287661	-6.08
257414_at	hypothetical protein similar to hypot	---	AT1G62110	-1.13	-0.17	-1.55	0.167165	0.287885	-6.08
257478_at	hypothetical protein similar to putati	WAKL2	AT1G16130	-1.33	-0.41	-1.55	0.167319	0.28809	-6.08
262218_at	hypothetical protein contains similar	---	AT1G74770	-1.20	-0.27	-1.55	0.167328	0.28809	-6.08
257673_at	unknown protein similar to GB:BAA	---	AT3G20370	-1.17	-0.22	-1.55	0.167356	0.28809	-6.08
253504_at	hypothetical protein	---	AT4G31960	-1.09	-0.13	-1.55	0.16736	0.28809	-6.08
252419_at	putative protein	---	AT3G47510	-1.32	-0.40	-1.55	0.16736	0.28809	-6.08
264234_at	hypothetical protein contains similar	---	AT1G54720	-1.11	-0.15	-1.55	0.16736	0.28809	-6.08
266487_at	hypothetical protein predicted by ge	---	AT2G47660	-1.10	-0.14	-1.55	0.167439	0.288193	-6.08
246298_at	putative protein sequence in GenBa	ETO1	AT3G51770	-1.12	-0.16	-1.55	0.167456	0.288193	-6.08
264030_at	putative steroid sulfotransferase	---	AT2G03770	1.16	0.22	1.55	0.167457	0.288193	-6.08
250581_at	copine-like protein copine VII prote	BON2	AT5G07300	1.12	0.17	1.55	0.167519	0.288277	-6.08
246671_at	putative protein	---	---	-1.11	-0.15	-1.55	0.167559	0.288307	-6.08
261282_at	hypothetical protein predicted by ge	APUM10	AT1G35750	1.18	0.23	1.55	0.167562	0.288307	-6.08
245489_at	FCA gamma protein	FCA	AT4G16280	1.13	0.17	1.55	0.167627	0.288397	-6.08
266153_at	hypothetical protein	---	---	-1.11	-0.15	-1.55	0.167778	0.288635	-6.08
AFFX-r2-Ec	E. coli /GEN=bioC /DB_XREF=gb:J	---	---	-1.32	-0.40	-1.55	0.167909	0.288838	-6.08
266469_at	myb-related protein	MYB14	AT2G31180	1.13	0.18	1.55	0.167936	0.288864	-6.08
253041_at	phosphoenolpyruvate carboxykinas	PCK1	AT4G37870	1.27	0.35	1.55	0.167959	0.28888	-6.08
263370_at	hypothetical protein predicted by ge	---	AT2G20500	-1.28	-0.35	-1.54	0.167971	0.28888	-6.08

260762_at	I-box binding factor, putative simila	---	AT1G49015	-1.13	-0.17	-1.54	0.167987	0.288886	-6.08
254307_at	putative protein various predicted p	---	AT4G22400	-1.22	-0.29	-1.54	0.168081	0.289026	-6.08
260558_at	putative endochitinase	---	AT2G43600	-1.17	-0.23	-1.54	0.168142	0.289108	-6.08
250419_at	RPP1 disease resistance protein -	---	AT5G11250	1.19	0.25	1.54	0.168229	0.289215	-6.08
267467_at	putative C3HC4 zinc finger protein	DRIP2	AT2G30580	-1.14	-0.19	-1.54	0.168229	0.289215	-6.08
257437_s_a	hypothetical protein predicted by ge	---	AT2G16290	1.12	0.17	1.54	0.16829	0.289298	-6.08
264753_at	receptor kinase, putative similar to	---	AT1G61490	1.10	0.14	1.54	0.168307	0.289306	-6.08
246422_at	ADP-ribosylation factor -like protei	ATARFB1E	AT5G17060	-1.30	-0.38	-1.54	0.168387	0.289409	-6.08
251501_at	putative protein several hypothetica	---	AT3G59120	-1.13	-0.17	-1.54	0.168392	0.289409	-6.08
267453_at	putative homeodomain transcriptior	HB-3	AT2G33880	-1.19	-0.25	-1.54	0.168415	0.289426	-6.08
262528_at	H+-transporting ATPase AHA10 Ide	AHA10	AT1G17260	-1.17	-0.22	-1.54	0.168428	0.289426	-6.08
246712_at	putative protein various predicted p	---	---	-1.10	-0.14	-1.54	0.168458	0.289455	-6.09
261048_at	flavonol 3-o-glucosyltransferase, pu	UGT72B3	AT1G01420	-1.15	-0.20	-1.54	0.168708	0.289863	-6.09
262847_at	unknown protein similar to myosin h	ATMAP70-	AT1G14840	-1.29	-0.37	-1.54	0.168724	0.28987	-6.09
258469_at	unknown protein contains Pfam pro	PRXIIF	AT3G06050	-1.12	-0.16	-1.54	0.168834	0.290037	-6.09
260184_s_a	putative protein predicted proteins,	---	---	-1.10	-0.14	-1.54	0.168874	0.290083	-6.09
266356_at	putative uclacyanin I identical to GF	UCC1	AT2G32300	-1.11	-0.14	-1.54	0.168924	0.290146	-6.09
247824_at	putative protein Na+/H+-exchangin	CHX25	AT5G58460	-1.15	-0.20	-1.54	0.168959	0.290173	-6.09
245902_at	putative protein Scythe protein, Xe	---	AT5G11080	-1.20	-0.26	-1.54	0.16897	0.290173	-6.09
259094_at	putative cysteine synthase similar to	CYSD1	AT3G04940	1.20	0.27	1.54	0.168977	0.290173	-6.09
267385_at	unknown protein highly similar to G	---	AT2G44380	1.24	0.31	1.54	0.169031	0.290244	-6.09
260434_at	hypothetical protein predicted by ge	---	AT1G68330	-1.22	-0.29	-1.54	0.169077	0.290301	-6.09
261423_at	homeodomain transcription factor,	AGL65	AT1G18750	1.17	0.22	1.54	0.169145	0.290396	-6.09
255740_at	wall-associated kinase, putative sir	---	AT1G25390	1.38	0.46	1.54	0.169183	0.29044	-6.09
249740_at	unknown protein	---	AT5G24500	-1.20	-0.26	-1.54	0.169204	0.290453	-6.09
255768_at	Expressed protein ; supported by fu	---	AT1G16705	-1.12	-0.17	-1.54	0.169232	0.290479	-6.09
259429_at	cytochrome P450, putative similar t	CYP86A4	AT1G01600	-1.61	-0.69	-1.54	0.169259	0.290503	-6.09
251981_at	putative protein BETA-GALACTOS	---	AT3G53080	-1.13	-0.17	-1.54	0.169344	0.290595	-6.09
263247_at	unknown protein	---	AT2G31440	-1.11	-0.15	-1.54	0.169352	0.290595	-6.09
245284_at	phytoene dehydrogenase precursor	PDS3	AT4G14210	-1.20	-0.26	-1.54	0.169353	0.290595	-6.09
264782_at	putative transcription factor similar t	MYB60	AT1G08810	1.17	0.23	1.54	0.169363	0.290595	-6.09
264333_at	hypothetical protein similar to putati	AtTLP4	AT1G61940	-1.12	-0.16	-1.54	0.169379	0.2906	-6.09
263445_at	unknown protein	---	AT2G31590	-1.11	-0.16	-1.54	0.169432	0.290669	-6.09
256040_at	hypothetical protein similar to putati	---	AT1G07270	-1.12	-0.16	-1.54	0.169463	0.290701	-6.09
265153_at	unusual floral organ (UFO), AtFBX	UFO	AT1G30950	-1.14	-0.19	-1.54	0.169567	0.290857	-6.09

266872_at	unknown protein	---	AT2G44730	-1.15	-0.20	-1.54	0.169636	0.290953	-6.09
262727_at	thaumatin, putative contains Pfam	---	AT1G75800	-1.12	-0.16	-1.54	0.169672	0.290995	-6.09
254912_at	respiratory burst oxidase homolog	---	AT4G11230	-1.13	-0.17	-1.54	0.169697	0.291015	-6.09
248139_at	putative protein similar to unknown	---	AT5G54970	2.29	1.20	1.54	0.169777	0.29113	-6.09
247513_at	quinone oxidoreductase - like prote	---	AT5G61510	1.19	0.25	1.54	0.169843	0.291222	-6.09
246331_at	non-phototropic hypocotyl 3-like pr	---	AT3G44820	-1.16	-0.21	-1.54	0.169864	0.291236	-6.09
259391_s_a	delta 9 desaturase, putative similar	---	AT1G06350	-1.13	-0.18	-1.54	0.170102	0.291621	-6.09
262591_at	hypothetical protein Similar to gb|X8	CCMH	AT1G15220	-1.12	-0.16	-1.54	0.170148	0.291678	-6.09
254600_at	4-coumarate-CoA ligase - like 4-co	---	AT4G19010	1.24	0.31	1.54	0.170317	0.29194	-6.10
262233_at	hypothetical protein contains similar	CHR18	AT1G48310	1.12	0.16	1.54	0.170338	0.29194	-6.10
253114_at	GTP-binding protein GB2 ;support	ATGB2	AT4G35860	-1.15	-0.20	-1.54	0.170339	0.29194	-6.10
249272_at	geranylgeranyl transferase alpha su	---	AT5G41820	-1.20	-0.26	-1.53	0.170421	0.292059	-6.10
249860_at	prolylcarboxypeptidase-like protein	---	AT5G22860	2.02	1.01	1.53	0.170521	0.292209	-6.10
249982_at	putative protein predicted proteins,	---	AT5G18520	-1.13	-0.17	-1.53	0.170589	0.292303	-6.10
256886_at	hypothetical protein contains Pfam	---	AT3G15130	1.10	0.13	1.53	0.170619	0.292323	-6.10
262878_at	hypothetical protein predicted by ge	NDF2	AT1G64770	1.15	0.21	1.53	0.170633	0.292323	-6.10
248796_at	VAMP (vesicle-associated membra	---	AT5G47180	-1.22	-0.29	-1.53	0.170639	0.292323	-6.10
247755_at	subtilisin-like serine protease conta	ATSBT4.12	AT5G59090	-1.11	-0.15	-1.53	0.170695	0.292397	-6.10
254979_at	subtilisin-like serine protease conta	---	AT4G10550	1.13	0.18	1.53	0.170729	0.292433	-6.10
253188_at	putative sugar transporter protein s	TMT2	AT4G35300	1.14	0.19	1.53	0.17087	0.292652	-6.10
263056_at	Mutator-like transposase similar to	---	---	-1.10	-0.14	-1.53	0.170903	0.292656	-6.10
266477_at	CLE4 CLAVATA3/ESR-Related 4 (C	CLE6	AT2G31085	-1.16	-0.21	-1.53	0.170919	0.292656	-6.10
258148_s_a	DNA-directed RNA polymerase II s	NRPD2B	AT3G18090	1.23	0.30	1.53	0.170927	0.292656	-6.10
248810_at	disease resistance protein-like	ADR1-L3	AT5G47280	-1.11	-0.15	-1.53	0.170933	0.292656	-6.10
253169_at	putative protein various predicted p	---	AT4G35120	-1.14	-0.19	-1.53	0.170936	0.292656	-6.10
256168_at	receptor protein kinase, putative co	---	AT1G51805	-1.33	-0.41	-1.53	0.170973	0.292697	-6.10
246964_at	putative protein crp1 protein - Zea	---	AT5G24830	-1.15	-0.21	-1.53	0.170996	0.292714	-6.10
255703_at	putative chalcone synthase similar t	---	AT4G00040	-1.22	-0.28	-1.53	0.171024	0.29274	-6.10
254466_at	subtilisin-like serine protease conta	---	AT4G20430	-1.18	-0.24	-1.53	0.171111	0.292855	-6.10
258960_at	hypothetical protein similar to myb-	---	AT3G10590	-1.12	-0.17	-1.53	0.171117	0.292855	-6.10
247443_at	putative protein predicted proteins,	---	AT5G62720	1.21	0.27	1.53	0.171133	0.292861	-6.10
248451_at	putative protein similar to unknown	---	AT5G51180	-1.10	-0.14	-1.53	0.171176	0.292895	-6.10
252792_at	hypothetical protein	---	---	-1.16	-0.21	-1.53	0.171179	0.292895	-6.10
253434_at	potassium channel - protein	AKT5	AT4G32500	-1.13	-0.17	-1.53	0.171276	0.293039	-6.10
247832_at	putative protein predicted proteins,	EOL2	AT5G58550	-1.15	-0.20	-1.53	0.171325	0.293101	-6.10

257561_at	hypothetical protein predicted by ge	APUM25	AT3G24270	-1.14	-0.18	-1.53	0.171347	0.293118	-6.10
267441_at	putative receptor-like protein kinase	---	AT2G19210	-1.11	-0.15	-1.53	0.171373	0.29314	-6.10
256158_at	hypothetical protein contains similar	ATPSK1	AT1G13590	-1.12	-0.17	-1.53	0.171522	0.293372	-6.10
263451_at	putative triacylglycerol lipase	---	AT2G31690	-1.10	-0.14	-1.53	0.171593	0.293448	-6.10
257762_at	hypothetical protein	XRCC4	AT3G23100	1.13	0.17	1.53	0.171597	0.293448	-6.10
261970_at	glutamate decarboxylase (gad), pu	GAD2	AT1G65960	1.18	0.24	1.53	0.171605	0.293448	-6.10
252608_at	putative protein 2-phosphoglycerat	---	AT3G45090	-1.15	-0.20	-1.53	0.171647	0.293499	-6.10
253124_at	putative protein unknown protein A	ARO3	AT4G36030	-1.43	-0.51	-1.53	0.171693	0.293555	-6.10
248141_at	unknown protein	---	AT5G55010	1.12	0.17	1.53	0.171748	0.293628	-6.10
248793_at	mutT domain protein-like ;supporte	atnudt8	AT5G47240	-1.33	-0.41	-1.53	0.171816	0.293721	-6.10
253262_at	putative serine threonine protein kir	---	AT4G34440	-1.12	-0.17	-1.53	0.171829	0.293721	-6.10
267100_at	unknown protein	---	AT2G41470	-1.12	-0.16	-1.53	0.171847	0.29373	-6.10
254704_at	putative protein predicted protein, A	APRR2	AT4G18020	1.14	0.19	1.53	0.171878	0.293762	-6.10
249644_at	putative serine-rich protein hypothe	---	AT5G37010	-1.16	-0.21	-1.53	0.171929	0.293827	-6.10
264388_at	hypothetical protein contains similar	---	AT1G11970	-1.16	-0.21	-1.53	0.171968	0.293871	-6.10
251364_at	anthranilate phosphoribosyltransfe	---	AT3G61300	-1.14	-0.18	-1.53	0.171984	0.293875	-6.10
259961_at	auxin-induced protein kinase, putat	WAG1	AT1G53700	-1.14	-0.19	-1.53	0.172024	0.293923	-6.10
250980_at	putative protein	---	AT5G03130	-1.32	-0.40	-1.53	0.172115	0.294056	-6.10
247122_at	putative protein non-consensus AT	ATSAC1B	AT5G66020	-1.18	-0.24	-1.53	0.172128	0.294056	-6.10
253051_at	cyclin cyc1 ; supported by cDNA: g	CYCB1;1	AT4G37490	-1.18	-0.24	-1.53	0.17222	0.294184	-6.11
245595_at	hypothetical protein	---	AT4G14170	1.11	0.16	1.53	0.172229	0.294184	-6.11
255812_at	potassium uptake transporter - like	HKT1	AT4G10310	1.24	0.31	1.53	0.172258	0.294193	-6.11
253748_at	putative protein RNA-binding prote	---	AT4G28990	-1.17	-0.22	-1.53	0.172267	0.294193	-6.11
255172_x_a	putative transposon protein similar	---	---	-1.17	-0.23	-1.53	0.172272	0.294193	-6.11
252247_at	putative protein various predicted p	---	AT3G49740	1.12	0.16	1.53	0.172301	0.294203	-6.11
246104_at	DNA-binding protein - like DNA-bin	WRKY74	AT5G28650	1.11	0.15	1.53	0.172304	0.294203	-6.11
262619_at	3-hydroxyisobutyryl-coenzyme A h	---	AT1G06550	1.46	0.55	1.53	0.172342	0.294232	-6.11
248052_at	putative protein contains similarity t	---	AT5G55800	-1.13	-0.18	-1.53	0.172347	0.294232	-6.11
265494_at	putative calmodulin-like protein	---	AT2G15680	-1.18	-0.23	-1.53	0.172432	0.294355	-6.11
254686_at	putative protein putative oncogene	---	AT4G13720	-1.15	-0.20	-1.53	0.17247	0.294398	-6.11
248279_at	putative protein similar to unknown	ATIM	AT5G52910	-1.31	-0.39	-1.53	0.172491	0.294404	-6.11
257431_at	unknown protein	---	AT2G36360	-1.13	-0.18	-1.53	0.172499	0.294404	-6.11
250106_at	putative protein strong similarity to u	---	AT5G16640	1.12	0.16	1.53	0.172559	0.294469	-6.11
257244_at	receptor kinase, putative similar to	---	AT3G24240	-1.26	-0.33	-1.53	0.172563	0.294469	-6.11
264089_at	actin depolymerizing factor 6 ;supp	ADF6	AT2G31200	1.19	0.25	1.53	0.172615	0.294535	-6.11

257380_at	hypothetical protein predicted by ge	---	AT2G28090	-1.15	-0.20	-1.53	0.172639	0.294545	-6.11
247483_at	aldose reductase-like protein	---	AT5G62420	-1.17	-0.22	-1.53	0.172647	0.294545	-6.11
266006_at	putative ABC transporter	---	AT2G37360	-1.17	-0.23	-1.53	0.172665	0.294547	-6.11
248664_at	peptidyl-prolyl cis-trans isomerase-l	FKBP15-2	AT5G48580	1.20	0.26	1.53	0.172674	0.294547	-6.11
263265_at	hypothetical protein predicted by ge	---	AT2G38820	1.22	0.29	1.53	0.172742	0.294624	-6.11
265292_s_a	hypothetical protein predicted by ge	---	---	-1.10	-0.14	-1.53	0.172744	0.294624	-6.11
255867_at	hypothetical protein predicted by ge	---	AT2G30370	-1.12	-0.16	-1.53	0.172774	0.294652	-6.11
246430_at	calmodulin-like protein calmodulin-	---	AT5G17470	-1.12	-0.16	-1.52	0.172836	0.294736	-6.11
257472_at	hypothetical protein predicted by ge	---	AT1G56040	-1.11	-0.15	-1.52	0.17286	0.294756	-6.11
261998_at	putative gag-pol polyprotein similar	---	---	-1.13	-0.18	-1.52	0.172921	0.294838	-6.11
254947_at	putative retrotransposon polyprotei	---	---	-1.21	-0.27	-1.52	0.172951	0.294867	-6.11
248202_at	unknown protein	---	AT5G54220	-1.19	-0.25	-1.52	0.173022	0.294965	-6.11
250694_at	putative protein contains similarity t	HAT14	AT5G06710	1.17	0.23	1.52	0.173065	0.294999	-6.11
254171_at	PsRT17-1 like protein PsRT17-1, F	---	AT4G24530	-1.11	-0.15	-1.52	0.173068	0.294999	-6.11
251040_at	ribonuclease II-like protein ribonucl	EMB2730	AT5G02250	1.22	0.29	1.52	0.173161	0.295135	-6.11
245174_at	putative kinesin heavy chain	---	AT2G47500	-1.25	-0.33	-1.52	0.173231	0.2952	-6.11
260462_at	ZIP4, a putative zinc transporter pe	ZIP4	AT1G10970	1.25	0.32	1.52	0.173233	0.2952	-6.11
263791_at	putative plasma membrane proton /	AHA5	AT2G24520	1.22	0.28	1.52	0.173238	0.2952	-6.11
256443_at	hypothetical protein similar to hypot	---	AT3G10960	-1.26	-0.33	-1.52	0.17329	0.295267	-6.11
253491_at	RNA lariat debranching enzyme - li	---	AT4G31770	-1.24	-0.31	-1.52	0.17341	0.295449	-6.11
252779_at	putative protein	---	AT3G42990	-1.12	-0.17	-1.52	0.17346	0.295513	-6.11
246509_at	valine--tRNA ligase-like protein sev	EMB2247	AT5G16715	1.22	0.29	1.52	0.17349	0.295528	-6.11
259713_at	unknown protein similar to phospha	---	AT1G77610	1.19	0.25	1.52	0.173495	0.295528	-6.11
255612_at	hypothetical protein	---	AT4G01240	-1.17	-0.23	-1.52	0.173512	0.295536	-6.11
249570_at	hypothetical protein	---	AT5G38080	-1.15	-0.20	-1.52	0.173603	0.295668	-6.11
245363_at	hypothetical protein ;supported by f	---	AT4G15120	1.50	0.58	1.52	0.173616	0.295668	-6.11
267255_at	putative Ca2+-ATPase	---	AT2G22950	-1.17	-0.23	-1.52	0.173635	0.295679	-6.11
255588_at	hypothetical protein	---	AT4G01570	1.20	0.26	1.52	0.173664	0.295704	-6.11
263902_at	phosphoribosylformimino-5-aminoir	APG10	AT2G36230	-1.12	-0.17	-1.52	0.173676	0.295704	-6.11
258631_at	putative polygalacturonase similar t	QRT2	AT3G07970	1.47	0.55	1.52	0.173739	0.295789	-6.11
245167_s_a	putative synaptobrevin ;supported	ATVAMP72	AT2G33110	1.11	0.15	1.52	0.173806	0.295873	-6.11
253084_at	putative protein lateral root primorc	STY2	AT4G36260	-1.20	-0.27	-1.52	0.173814	0.295873	-6.11
264482_at	sugar carrier protein, putative simil	---	AT1G77210	1.35	0.43	1.52	0.173853	0.295917	-6.11
256596_at	hypothetical protein predicted by ge	---	AT3G28540	1.26	0.33	1.52	0.173919	0.296008	-6.11
267627_at	putative ATP-dependent RNA helic	---	AT2G42270	1.26	0.33	1.52	0.173975	0.29608	-6.11

260457_at	unknown protein	---	AT1G72480	-1.13	-0.18	-1.52	0.173989	0.296082	-6.11
257533_at	putative alpha/beta hydrolase conta	---	AT3G10840	-1.24	-0.31	-1.52	0.174097	0.296244	-6.12
262772_at	puative calcium-transporting ATPas	ACA.I	AT1G13210	1.28	0.36	1.52	0.174111	0.296246	-6.12
247205_at	unknown protein ;supported by full-	PROPEP2	AT5G64890	1.81	0.86	1.52	0.174128	0.296253	-6.12
252871_at	proliferating-cell nucleolar antigen -	---	AT4G40000	-1.12	-0.17	-1.52	0.174291	0.296507	-6.12
254982_at	putative protein ; supported by cDN	---	AT4G10470	1.16	0.21	1.52	0.174375	0.296629	-6.12
263777_at	putative cyclic nucleotide-regulated	ATCNGC12	AT2G46450	1.62	0.69	1.52	0.174408	0.296648	-6.12
256746_at	alpha-glucan phosphorylase, putat	---	AT3G29320	-1.20	-0.26	-1.52	0.174412	0.296648	-6.12
247021_at	putative protein similar to unknown	---	AT5G67040	-1.14	-0.18	-1.52	0.174518	0.296805	-6.12
250848_at	3-phosphoinositide-dependent prote	PDK1	AT5G04510	-1.14	-0.19	-1.52	0.174546	0.296832	-6.12
258212_at	unknown protein contains Pfam pro	---	AT3G17920	-1.10	-0.13	-1.52	0.174604	0.296907	-6.12
252802_at	putative protein	---	---	1.09	0.13	1.52	0.174641	0.29694	-6.12
263508_s_a	hypothetical protein	---	---	-1.17	-0.23	-1.52	0.174649	0.29694	-6.12
251091_at	pyridoxine biosynthesis protein - like	RSR4	AT5G01410	1.33	0.41	1.52	0.174677	0.296966	-6.12
257708_at	hypothetical protein predicted by ge	---	AT3G13330	-1.13	-0.17	-1.52	0.174758	0.297081	-6.12
254826_at	putative protein predicted protein, A	---	AT4G12640	1.16	0.21	1.52	0.174785	0.297105	-6.12
256172_at	hypothetical protein	---	AT1G51745	-1.11	-0.16	-1.52	0.174891	0.297262	-6.12
263206_at	unknown protein similar to ESTs gb	---	AT1G10590	1.10	0.14	1.52	0.174925	0.297287	-6.12
265791_at	putative transcription factor	MEE65	AT2G01280	-1.15	-0.20	-1.52	0.174931	0.297287	-6.12
256547_at	receptor-like serine/threonine kinas	---	AT3G14840	1.29	0.36	1.52	0.174976	0.297335	-6.12
261481_at	nucleoside triphosphatase, putative	---	AT1G14260	-1.17	-0.23	-1.52	0.174986	0.297335	-6.12
254658_at	putative protein predicted gene M0	---	AT4G18230	-1.13	-0.17	-1.52	0.175014	0.297361	-6.12
252546_at	DNA-binding protein - like DNA-bin	---	AT3G45830	1.12	0.17	1.52	0.175136	0.297546	-6.12
260814_at	histidine decarboxylase, putative si	emb1075	AT1G43710	1.12	0.16	1.52	0.175211	0.297652	-6.12
247020_at	putative protein strong similarity to u	---	AT5G67020	-1.18	-0.24	-1.51	0.175289	0.297762	-6.12
261812_at	hypothetical protein contains similar	---	AT1G08270	-1.10	-0.14	-1.51	0.175381	0.297896	-6.12
259952_at	putative disease resistance protein	AtRLP12	AT1G71400	1.25	0.33	1.51	0.175398	0.297902	-6.12
250855_at	putative protein predicted proteins,	---	AT5G04730	-1.13	-0.17	-1.51	0.175459	0.297968	-6.12
265782_at	putative two-component response r	---	AT2G07440	-1.14	-0.18	-1.51	0.175463	0.297968	-6.12
258155_at	protein kinase C-receptor/G-protein	RACK1C_A	AT3G18130	1.45	0.54	1.51	0.175565	0.29812	-6.12
267618_at	putative cyclin ; supported by cDNA	CYCB1;4	AT2G26760	-1.15	-0.20	-1.51	0.175683	0.298298	-6.12
253093_at	putative protein SPINDLY protein,	SRFR1	AT4G37460	1.13	0.17	1.51	0.175714	0.298328	-6.12
245478_at	galactokinase like protein	ARA1	AT4G16130	1.32	0.40	1.51	0.175987	0.298768	-6.13
254004_at	arginyl-tRNA synthetase	emb1027	AT4G26300	1.10	0.13	1.51	0.176	0.298768	-6.13
259312_at	putative RING-H2 zinc finger protein	ATL6	AT3G05200	1.41	0.49	1.51	0.176068	0.298849	-6.13

257424_at	hypothetical protein predicted by ge	---	AT1G78840	-1.18	-0.24	-1.51	0.176073	0.298849	-6.13
249923_at	conglutin gamma - like protein cong	---	AT5G19120	-1.26	-0.34	-1.51	0.176115	0.298889	-6.13
248395_at	putative protein contains similarity t	AtPP2-A14	AT5G52120	1.19	0.25	1.51	0.176123	0.298889	-6.13
246786_at	putative amino acid aminotransfera	---	AT5G27410	1.15	0.20	1.51	0.176174	0.298954	-6.13
246085_at	putative protein predicted proteins,	ATBRXL4	AT5G20540	-1.15	-0.21	-1.51	0.176193	0.298963	-6.13
254161_at	hypothetical protein ;supported by	---	AT4G24370	-1.13	-0.17	-1.51	0.176207	0.298965	-6.13
251763_at	MYB transcription factor - like prote	MYB109	AT3G55730	-1.15	-0.20	-1.51	0.176228	0.298979	-6.13
266222_at	hypothetical protein predicted by ge	---	AT2G28780	-1.12	-0.16	-1.51	0.176242	0.298979	-6.13
245104_at	hypothetical protein predicted by ge	---	AT2G41600	1.12	0.16	1.51	0.17627	0.299005	-6.13
248722_at	pyrophosphate-fructose-6-phospha	PFK2	AT5G47810	1.10	0.13	1.51	0.176326	0.299077	-6.13
255091_at	putative protein various predicted p	---	---	-1.10	-0.14	-1.51	0.176379	0.299145	-6.13
257123_at	hypothetical protein	---	AT3G20030	-1.10	-0.14	-1.51	0.176493	0.299316	-6.13
266048_at	putative thioredoxin H contains a th	ATCXXS2	AT2G40790	-1.14	-0.19	-1.51	0.17655	0.299392	-6.13
254325_at	putative protein various predicted p	---	AT4G22650	-1.19	-0.25	-1.51	0.176697	0.29961	-6.13
250967_at	putative protein In2, Zea mays, EM	---	AT5G02790	1.18	0.24	1.51	0.176706	0.29961	-6.13
264417_at	hypothetical protein contains similar	---	---	-1.20	-0.27	-1.51	0.176739	0.299629	-6.13
266244_at	unknown protein	---	AT2G27740	-1.38	-0.47	-1.51	0.176743	0.299629	-6.13
255806_at	fructokinase - like protein fructokina	---	AT4G10260	-1.09	-0.13	-1.51	0.176893	0.299861	-6.13
253395_at	protein kinase AME3 ; supported b	AME3	AT4G32660	-1.11	-0.15	-1.51	0.176913	0.299872	-6.13
266442_at	hypothetical protein	---	AT2G43260	1.10	0.14	1.51	0.176967	0.299942	-6.13
260320_at	hypothetical protein similar to putati	---	AT1G63930	-1.19	-0.25	-1.51	0.177004	0.299983	-6.13
264900_at	putative auxin transport protein stro	PIN7	AT1G23080	1.22	0.28	1.51	0.177112	0.300125	-6.13
253077_at	hypothetical protein	---	AT4G36170	1.10	0.14	1.51	0.177114	0.300125	-6.13
253487_at	ribosomal protein S6 - like ribosom	RPS6	AT4G31700	1.37	0.45	1.51	0.177242	0.300319	-6.13
259906_at	unknown protein	---	AT1G74120	1.11	0.15	1.51	0.177265	0.300335	-6.13
253723_at	extensin-like protein ;supported by	---	AT4G29240	-1.14	-0.19	-1.51	0.177311	0.30039	-6.13
247264_at	putative protein contains similarity t	XND1	AT5G64530	1.33	0.42	1.51	0.177414	0.300536	-6.13
254842_at	putative protein other hypothetical p	---	AT4G11950	-1.16	-0.21	-1.51	0.177432	0.300536	-6.13
267543_at	26S proteasome regulatory subunit	---	AT2G32730	1.10	0.14	1.51	0.177438	0.300536	-6.13
259863_at	hypothetical protein ;supported by	ELF4-L2	AT1G72630	-1.12	-0.16	-1.51	0.177449	0.300536	-6.13
265249_at	putative C2H2-type zinc finger prote	SGR5	AT2G01940	1.36	0.44	1.51	0.177466	0.300542	-6.13
250597_at	NAM (no apical meristem)-like prote	ANAC080	AT5G07680	1.28	0.36	1.51	0.177561	0.30068	-6.13
266766_at	hypothetical protein predicted by ge	PAP14	AT2G46880	-1.26	-0.33	-1.51	0.177697	0.300888	-6.13
264604_at	hypothetical protein predicted by ge	---	AT1G04650	1.18	0.23	1.51	0.17771	0.300888	-6.13
263397_s_a	putative GDSL-motif lipase/hydrolas	---	AT2G31540	-1.09	-0.13	-1.51	0.177724	0.300889	-6.13

247263_at	putative protein similar to unknown	---	AT5G64470	1.18	0.24	1.50	0.177821	0.301031	-6.13
254883_at	putative protein histidine-rich glyco	HAP2	AT4G11720	-1.16	-0.21	-1.50	0.177834	0.301031	-6.13
247054_at	zinc finger protein	---	AT5G66730	1.14	0.19	1.50	0.177864	0.30106	-6.13
262757_at	putative Na/H antiporter similar to g	ATCHX1	AT1G16380	-1.13	-0.18	-1.50	0.177903	0.301104	-6.13
262252_at	RING-H2 zinc finger protein ATL3,	---	AT1G53820	-1.12	-0.16	-1.50	0.177926	0.301119	-6.14
265429_at	hypothetical protein predicted by ge	---	AT2G20710	1.16	0.21	1.50	0.178054	0.301314	-6.14
259913_at	putative GTP-binding protein simila	---	AT1G72660	1.10	0.14	1.50	0.178173	0.301493	-6.14
253150_at	putative protein predicted protein, A	---	AT4G35660	-1.10	-0.13	-1.50	0.178239	0.301573	-6.14
252100_at	crooked neck-like protein crooked r	---	AT3G51110	1.24	0.31	1.50	0.178247	0.301573	-6.14
260122_at	AIG1-like protein similar to AIG1 pr	---	AT1G33900	-1.15	-0.20	-1.50	0.178303	0.301646	-6.14
249705_at	serine/threonine protein kinase-like	---	AT5G35580	1.20	0.27	1.50	0.178352	0.301696	-6.14
248562_at	putative protein strong similarity to u	---	AT5G49680	-1.32	-0.40	-1.50	0.178359	0.301696	-6.14
258778_s_a	hypothetical protein predicted by ge	---	AT3G11860	1.15	0.20	1.50	0.178435	0.301802	-6.14
246394_at	myrosinase binding protein, putativ	---	AT1G58160	-1.11	-0.15	-1.50	0.178449	0.301804	-6.14
245890_at	ribosomal protein S15-like ribosom	---	AT5G09490	-1.09	-0.13	-1.50	0.178485	0.301813	-6.14
261495_at	hypothetical protein predicted by ge	---	AT1G28410	1.13	0.18	1.50	0.17849	0.301813	-6.14
253675_at	hypothetical protein	---	AT4G29560	1.12	0.16	1.50	0.178494	0.301813	-6.14
262834_at	hypothetical protein predicted by ge	MAP65-7	AT1G14690	1.13	0.17	1.50	0.178545	0.301847	-6.14
261960_at	hypothetical protein similar to putati	---	---	1.10	0.13	1.50	0.17855	0.301847	-6.14
249153_s_a	putative protein strong similarity to u	---	AT5G43390	-1.14	-0.19	-1.50	0.178554	0.301847	-6.14
257558_s_a	hypothetical protein contains Pfam	---	AT3G22000	-1.12	-0.16	-1.50	0.178624	0.301943	-6.14
267576_at	putative Mutator-like transposase	---	---	1.16	0.22	1.50	0.178645	0.301956	-6.14
246677_at	putative protein predicted proteins -	---	---	-1.14	-0.19	-1.50	0.178664	0.301966	-6.14
245139_at	putative AT-hook DNA-binding prote	---	AT2G45430	-1.13	-0.18	-1.50	0.178755	0.302096	-6.14
261552_at	receptor protein kinase, putative co	---	AT1G63430	1.14	0.19	1.50	0.178767	0.302096	-6.14
251038_at	putative protein unknown protein A	---	AT5G02240	1.11	0.16	1.50	0.178823	0.302159	-6.14
250152_at	putative protein predicted proteins,	---	AT5G15120	-1.11	-0.15	-1.50	0.178831	0.302159	-6.14
251263_at	putative protein DNAJ PROTEIN -	---	AT3G62190	1.13	0.17	1.50	0.178845	0.30216	-6.14
265023_at	unknown protein weak similarity to (	---	AT1G24440	1.18	0.24	1.50	0.178861	0.302164	-6.14
251956_at	RNA-binding protein cp29 protein ;	CP29	AT3G53460	1.13	0.17	1.50	0.178904	0.302207	-6.14
262466_at	hypothetical protein similar to hypot	NTMC2T5.	AT1G50260	1.17	0.22	1.50	0.178913	0.302207	-6.14
264282_at	hypothetical protein similar to hypot	---	AT1G61840	-1.14	-0.18	-1.50	0.179094	0.30249	-6.14
261253_at	unknown protein ;supported by full-	---	AT1G05840	1.19	0.25	1.50	0.179124	0.302519	-6.14
246454_at	Expressed protein ; supported by fu	DHAR3	AT5G16710	-1.13	-0.17	-1.50	0.179204	0.302632	-6.14
257882_at	hypothetical protein predicted by ge	---	AT3G16930	-1.14	-0.18	-1.50	0.179264	0.302711	-6.14

261989_at	disease resistance protein, putative	---	AT1G33670	-1.14	-0.19	-1.50	0.179292	0.302711	-6.14
256601_s_a	At14a-1 protein identical to At14a p	AT14A	AT3G28290	-1.12	-0.16	-1.50	0.179302	0.302711	-6.14
254255_at	serine/threonine kinase - like prote	---	AT4G23220	-1.39	-0.47	-1.50	0.179304	0.302711	-6.14
264589_at	hypothetical protein	---	AT2G17650	1.16	0.22	1.50	0.179337	0.302744	-6.14
256028_at	poly(A)-binding protein, putative sir	PAB1	AT1G34140	-1.17	-0.23	-1.50	0.179355	0.302753	-6.14
256888_at	hypothetical protein predicted by ge	---	AT3G15160	-1.12	-0.16	-1.50	0.179383	0.302776	-6.14
256667_at	hypothetical protein predicted by ge	---	AT3G32180	-1.10	-0.13	-1.50	0.179435	0.302827	-6.14
249148_at	unknown protein ; supported by cD	---	AT5G43260	1.23	0.29	1.50	0.179439	0.302827	-6.14
256488_at	hypothetical protein	NFD4	AT1G31470	-1.12	-0.17	-1.50	0.179461	0.302841	-6.14
256684_at	geranylgeranyl pyrophosphate syn	---	AT3G32040	-1.17	-0.23	-1.50	0.179494	0.302862	-6.14
257223_at	50S ribosomal protein L12-B identi	RPL12-B	AT3G27840	-1.15	-0.20	-1.50	0.1795	0.302862	-6.14
258275_at	unknown protein ;supported by full-	---	AT3G15760	1.23	0.30	1.50	0.179537	0.302902	-6.14
260550_at	putative sterol dehydrogenase ; su	---	AT2G43420	1.19	0.25	1.50	0.179594	0.302972	-6.14
250100_at	Expressed protein ; supported by cl	GLN1;4	AT5G16570	1.39	0.47	1.50	0.179618	0.302972	-6.14
247319_at	glutamate-tRNA ligase ; supported	ERS	AT5G64050	1.13	0.18	1.50	0.179628	0.302972	-6.14
258692_at	unknown protein	---	AT3G08640	-1.24	-0.31	-1.50	0.179631	0.302972	-6.14
261623_at	hypothetical protein similar to reticu	ATSEC1A	AT1G01980	-1.13	-0.17	-1.50	0.179746	0.303143	-6.14
256159_at	Expressed protein ; supported by fu	JAZ8	AT1G30135	1.72	0.78	1.50	0.179829	0.303261	-6.14
263916_at	hypothetical protein predicted by ge	---	AT2G36440	-1.13	-0.18	-1.50	0.179917	0.303361	-6.15
257091_at	DNA polymerase, putative C-term s	POLGAMM	AT3G20540	-1.18	-0.24	-1.50	0.179934	0.303361	-6.15
250230_at	putative protein similar to unknown	---	AT5G13900	-1.22	-0.29	-1.50	0.179939	0.303361	-6.15
250486_at	putative protein predicted proteins,	---	AT5G09670	-1.13	-0.18	-1.50	0.179942	0.303361	-6.15
265838_at	putative spliceosome associated pr	---	AT2G14550	-1.11	-0.15	-1.50	0.179955	0.303361	-6.15
251416_at	putative protein various predicted p	---	AT3G60400	1.12	0.16	1.50	0.179972	0.303367	-6.15
251485_at	putative protein rad21 mitotic cohe	SYN3	AT3G59550	-1.11	-0.15	-1.50	0.18002	0.303426	-6.15
255944_at	putative protein	---	---	-1.14	-0.19	-1.50	0.180161	0.30364	-6.15
266444_at	putative extensin	---	AT2G43150	-1.16	-0.21	-1.50	0.180227	0.303729	-6.15
256027_at	putative protein	---	AT1G34160	1.10	0.14	1.49	0.180285	0.303805	-6.15
253593_at	putative protein hypothetical protei	---	AT4G30760	1.14	0.19	1.49	0.180339	0.303866	-6.15
257162_s_a	ammonium transporter, putative sir	AMT1;3	AT3G24290	-1.11	-0.15	-1.49	0.180348	0.303866	-6.15
255849_at	hypothetical protein predicted by ge	---	AT2G33320	-1.11	-0.16	-1.49	0.180369	0.303879	-6.15
258278_at	putative self-incompatibility protein	---	AT3G26860	-1.12	-0.16	-1.49	0.180453	0.303998	-6.15
256918_s_a	hypothetical protein similar to hypot	---	AT3G18960	-1.17	-0.23	-1.49	0.180516	0.304082	-6.15
262238_at	hypothetical protein predicted by ge	---	AT1G48300	1.22	0.28	1.49	0.180541	0.304101	-6.15
262282_at	hypothetical protein predicted by ge	---	AT1G68610	-1.15	-0.20	-1.49	0.180581	0.304146	-6.15

251458_at	putative protein several retrotranspo	---	---	-1.12	-0.16	-1.49	0.180629	0.304204	-6.15
266896_at	putative ATP-dependent RNA helica	---	AT2G45810	1.10	0.14	1.49	0.180685	0.304277	-6.15
254285_at	putative cdc2 kinase homolog cdc2	---	AT4G22940	1.16	0.22	1.49	0.18072	0.304312	-6.15
245237_at	putative protein similarity to ALR - H	SLK1	AT4G25520	1.12	0.16	1.49	0.18074	0.304324	-6.15
252760_x_a	putative protein several hypothetica	---	---	-1.11	-0.15	-1.49	0.180773	0.304356	-6.15
246024_at	putative protein predicted protein -	---	AT5G21130	-1.15	-0.20	-1.49	0.180802	0.304384	-6.15
257305_at	hypothetical protein predicted by ge	---	---	-1.15	-0.20	-1.49	0.18089	0.304509	-6.15
261604_at	unknown protein contains similarity	---	AT1G49590	1.14	0.19	1.49	0.180989	0.304649	-6.15
246116_at	putative protein predicted proteins -	---	AT5G20310	-1.14	-0.19	-1.49	0.181	0.304649	-6.15
256567_at	amino acid permease, putative con	---	AT3G19553	-1.13	-0.18	-1.49	0.181038	0.304672	-6.15
256190_at	9-cis-epoxycarotenoid dioxygenase	NCED5	AT1G30100	-1.12	-0.17	-1.49	0.18104	0.304672	-6.15
251312_at	MADS-box protein AGL13 ; suppor	AGL13	AT3G61120	-1.12	-0.16	-1.49	0.181059	0.30468	-6.15
249729_at	6-phosphogluconolactonase-like pr	---	AT5G24410	-1.13	-0.18	-1.49	0.18108	0.30468	-6.15
260024_at	beta-1,3-glucanase precursor, puta	---	AT1G30080	-1.10	-0.14	-1.49	0.181085	0.30468	-6.15
250713_at	putative protein similar to unknown	---	AT5G06130	1.14	0.19	1.49	0.181176	0.30481	-6.15
250282_at	putative protein predicted protein, A	---	AT5G13260	-1.18	-0.24	-1.49	0.181232	0.304883	-6.15
251534_at	ATP-dependent RNA helicase-like	---	AT3G58510	1.23	0.29	1.49	0.181252	0.304894	-6.15
251787_at	2-oxoglutarate dehydrogenase, E1	---	AT3G55410	-1.15	-0.20	-1.49	0.18127	0.304902	-6.15
253261_at	putative protein mRNA, partial cds,	CHB3	AT4G34430	-1.14	-0.19	-1.49	0.181342	0.304997	-6.15
255250_at	MYB - like protein cM4 myb transcr	AtMYB74	AT4G05100	-1.58	-0.66	-1.49	0.181356	0.304997	-6.15
259314_at	putative glucose and ribitol dehydro	---	AT3G05260	-1.21	-0.27	-1.49	0.181367	0.304997	-6.15
252251_at	hypothetical protein	---	AT3G49820	-1.11	-0.15	-1.49	0.181383	0.305	-6.15
255198_at	contains similarity to an Arabidopsi	---	---	-1.11	-0.15	-1.49	0.181395	0.305	-6.15
253823_at	putative protein ;supported by full-l	---	AT4G28030	1.10	0.14	1.49	0.181438	0.305044	-6.15
255406_at	hypothetical protein similar to hypo	---	AT4G03450	1.39	0.48	1.49	0.181448	0.305044	-6.15
257958_at	unknown protein similar to disease	---	AT3G25510	-1.15	-0.20	-1.49	0.181586	0.305253	-6.15
247671_at	putative protein slow myosin heavy	---	AT5G60210	-1.10	-0.14	-1.49	0.181766	0.305516	-6.15
250338_at	putative protein ; supported by cDN	---	AT5G11760	1.11	0.15	1.49	0.181769	0.305516	-6.15
259414_at	hypothetical protein contains Pfam	---	AT1G02420	1.15	0.21	1.49	0.181788	0.305524	-6.15
265072_at	hypothetical protein predicted by ge	LAS	AT1G55580	-1.17	-0.23	-1.49	0.18185	0.305606	-6.15
245984_at	putative protein predicted proteins -	---	AT5G13090	1.13	0.18	1.49	0.181989	0.305817	-6.16
259877_at	unknown protein C-terminal portion	SDG26	AT1G76710	-1.16	-0.21	-1.49	0.182012	0.305834	-6.16
257295_at	serine/threonine protein kinase, pu	GPK1	AT3G17420	-1.23	-0.29	-1.49	0.182125	0.306002	-6.16
264902_at	unknown protein Location of EST g	---	AT1G23060	1.28	0.36	1.49	0.182157	0.306017	-6.16
253846_at	putative protein MSP1, Saccharom	---	AT4G28000	-1.10	-0.14	-1.49	0.182161	0.306017	-6.16

260031_at	putative nuclear matrix constituent p	LINC3	AT1G68790	1.13	0.18	1.49	0.182261	0.306162	-6.16
257097_at	hypothetical protein predicted by ge	---	---	-1.11	-0.15	-1.49	0.18231	0.306221	-6.16
264865_at	DNAJ-like heatshock protein similar	ARL1	AT1G24120	-1.11	-0.15	-1.49	0.182354	0.306273	-6.16
259337_at	putative cationic amino acid transpo	CAT4	AT3G03720	-1.12	-0.16	-1.49	0.182394	0.306317	-6.16
255717_at	coded for by A. thaliana cDNA H76	---	AT4G00350	-1.12	-0.16	-1.49	0.182473	0.306428	-6.16
263563_at	putative retroelement pol polyprotei	---	---	-1.12	-0.17	-1.49	0.182689	0.306767	-6.16
259155_at	transcription initiation factor IIB (TF	---	AT3G10330	1.16	0.21	1.49	0.182714	0.306777	-6.16
248430_at	putative protein similar to unknown	---	AT5G51800	-1.13	-0.18	-1.49	0.182729	0.306777	-6.16
264917_at	unknown protein contains Pfam pro	---	AT1G60500	-1.10	-0.14	-1.49	0.182734	0.306777	-6.16
254104_at	putative protein predicted protein, A	---	AT4G25040	-1.15	-0.20	-1.49	0.182807	0.306861	-6.16
245958_at	GNOM-like protein pattern-formatio	---	AT5G19610	-1.12	-0.16	-1.48	0.182812	0.306861	-6.16
263767_at	putative retroelement pol polyprotei	---	---	-1.10	-0.14	-1.48	0.182861	0.306922	-6.16
255126_at	putative protein hypothetical protei	---	AT4G08270	-1.10	-0.14	-1.48	0.182893	0.306952	-6.16
260355_at	transcription factor CRC identical to	CRC	AT1G69180	1.17	0.23	1.48	0.182926	0.306986	-6.16
257834_at	alpha-mannosidase, putative simila	---	AT3G26720	1.10	0.14	1.48	0.182954	0.30701	-6.16
265765_at	hypothetical protein predicted by ge	---	AT2G48060	1.14	0.18	1.48	0.183039	0.307129	-6.16
255773_at	flavonol 4'-sulfotransferase, putativ	SOT17	AT1G18590	1.42	0.51	1.48	0.183072	0.307163	-6.16
253246_at	putative protein	---	AT4G34600	1.15	0.20	1.48	0.18311	0.307203	-6.16
258626_at	transfactor, putative similar to trans	---	AT3G04450	1.14	0.19	1.48	0.183159	0.307263	-6.16
265522_at	putative TPR repeat nuclear phosph	ELF8	AT2G06210	1.14	0.19	1.48	0.183215	0.307334	-6.16
266875_at	putative flavonol synthase	---	AT2G44800	-1.15	-0.20	-1.48	0.183271	0.307405	-6.16
253791_at	early auxin-inducible protein 11 (IA	IAA11	AT4G28640	1.16	0.22	1.48	0.183343	0.307504	-6.16
264880_at	hypothetical protein contains similar	---	AT1G61210	-1.15	-0.20	-1.48	0.183358	0.307506	-6.16
250001_at	tub family-like protein tubby-like pro	AtTLP11	AT5G18680	-1.21	-0.27	-1.48	0.183397	0.307549	-6.16
251397_at	putative protein cim1 protein, Glyci	ATEXPB5	AT3G60570	-1.17	-0.23	-1.48	0.183453	0.30762	-6.16
251560_at	squamosa promoter-binding protei	SPL15	AT3G57920	-1.17	-0.23	-1.48	0.183592	0.307809	-6.16
263315_at	Expressed protein ; supported by fu	---	AT2G05755	-1.11	-0.15	-1.48	0.183593	0.307809	-6.16
254193_at	putative protein predicted proteins,	---	AT4G23870	1.42	0.51	1.48	0.183625	0.30782	-6.16
258077_at	unknown protein ;supported by full-	---	AT3G26110	-1.15	-0.20	-1.48	0.183626	0.30782	-6.16
247650_at	putative protein ;supported by full-l	---	AT5G59960	1.22	0.29	1.48	0.183771	0.308041	-6.16
253649_at	putative protein predicted protein, A	---	AT4G29790	1.25	0.32	1.48	0.18389	0.308217	-6.17
257593_at	phosphatidylinositol transfer protei	---	AT3G24840	-1.11	-0.15	-1.48	0.183911	0.308229	-6.17
259319_at	Snf1-related protein kinase KIN10	AKIN10	AT3G01090	1.10	0.14	1.48	0.184005	0.308364	-6.17
252865_at	Expressed protein ; supported by fu	---	AT4G39753	-1.18	-0.24	-1.48	0.184031	0.308386	-6.17
246972_s_a	cytochrome P-450-like protein cyto	CYP71A14	AT5G24950	1.29	0.37	1.48	0.18405	0.308395	-6.17

251609_at	putative protein hypothetical protei	---	AT3G57900	-1.10	-0.13	-1.48	0.184118	0.308461	-6.17
245768_at	hypothetical protein similar to putati	---	AT1G33590	1.31	0.39	1.48	0.184127	0.308461	-6.17
256477_at	unknown protein	---	---	-1.13	-0.17	-1.48	0.18413	0.308461	-6.17
257001_at	Sm protein, putative similar to GB:/	---	AT3G14080	1.13	0.18	1.48	0.184146	0.308464	-6.17
248194_at	Expressed protein ; supported by fu	---	AT5G54095	1.10	0.13	1.48	0.18416	0.308465	-6.17
250844_at	putative protein ;supported by full-l	SIM	AT5G04470	-1.29	-0.36	-1.48	0.184233	0.308566	-6.17
264121_at	putative GTP-binding protein Carbo	TOC33	AT1G02280	1.15	0.20	1.48	0.184345	0.308731	-6.17
257266_at	RNA-binding protein contains Pfam	---	AT3G15010	1.11	0.15	1.48	0.184424	0.30883	-6.17
261815_at	leucine zipper protein, putative sim	---	AT1G08320	-1.16	-0.22	-1.48	0.184433	0.30883	-6.17
265135_at	unknown protein similar to unknowr	---	AT1G51270	-1.13	-0.17	-1.48	0.184445	0.30883	-6.17
247633_at	putative zinc finger protein finger p	---	AT5G60470	-1.21	-0.28	-1.48	0.184482	0.30887	-6.17
259046_at	calmodulin-like protein similar to ca	---	AT3G03400	-1.13	-0.17	-1.48	0.184538	0.30894	-6.17
245300_at	hypothetical protein ; supported by	CBL6	AT4G16350	1.11	0.14	1.48	0.184567	0.308965	-6.17
255963_at	unknown protein	MBD8	AT1G22310	-1.13	-0.17	-1.48	0.184651	0.309085	-6.17
262911_s_a	heat shock protein, putative similar	---	AT1G07400	-1.49	-0.57	-1.48	0.184682	0.309114	-6.17
AFFX-LysX-	B. subtilis /GEN=lys /DB_XREF=gb	---	---	1.25	0.32	1.48	0.184765	0.309229	-6.17
254839_at	stress-induced protein sti1 -like pro	---	AT4G12400	-1.34	-0.43	-1.48	0.184812	0.309285	-6.17
262242_at	hypothetical protein predicted by ge	---	AT1G48360	-1.16	-0.21	-1.48	0.184838	0.309306	-6.17
250838_at	cytochrome P450 - like protein cyto	CYP77A9	AT5G04630	-1.10	-0.14	-1.48	0.184863	0.309326	-6.17
249405_at	beta subunit of protein farnesyl tran	ERA1	AT5G40280	1.12	0.16	1.48	0.184936	0.309425	-6.17
263993_at	hypothetical protein predicted by ge	---	---	-1.14	-0.19	-1.48	0.184996	0.309502	-6.17
260643_at	hypothetical protein contains similar	---	AT1G53270	-1.12	-0.16	-1.48	0.185019	0.309519	-6.17
250446_at	nucleoid DNA-binding protein cnd4	---	AT5G10770	1.25	0.32	1.48	0.185043	0.309536	-6.17
257539_at	hypothetical protein predicted by ge	---	AT3G29150	-1.11	-0.15	-1.48	0.185116	0.309632	-6.17
249591_at	putative protein predicted protein, A	---	---	-1.10	-0.14	-1.48	0.185129	0.309632	-6.17
246189_at	ABI3-interacting protein 2 ;support	---	AT5G20910	1.18	0.24	1.48	0.185148	0.309632	-6.17
266910_at	unknown protein	---	AT2G45920	1.32	0.41	1.48	0.185157	0.309632	-6.17
250311_at	unknown protein	---	AT5G12240	-1.10	-0.14	-1.48	0.185169	0.309632	-6.17
256099_at	cytochrome P450, putative similar	CYP78A5	AT1G13710	-1.20	-0.26	-1.48	0.185258	0.30976	-6.17
251824_at	ABC transporter - like protein ATP-	---	AT3G55090	-1.13	-0.18	-1.47	0.185411	0.309992	-6.17
249284_at	unknown protein ;supported by full-	---	AT5G41810	1.14	0.18	1.47	0.185483	0.310091	-6.17
AFFX-r2-Bs	B. subtilis /GEN=pheB /DB_XREF=	---	---	1.22	0.29	1.47	0.185702	0.310434	-6.17
265795_at	putative serine carboxypeptidase II	scpl26	AT2G35780	1.13	0.18	1.47	0.185789	0.310556	-6.17
247181_at	putative protein similar to unknown	---	AT5G65370	-1.12	-0.16	-1.47	0.186031	0.310938	-6.18
249080_at	putative protein strong similarity to u	SUVR2	AT5G43990	-1.17	-0.23	-1.47	0.186087	0.311008	-6.18

245129_at	hypothetical protein predicted by ge	CRR4	AT2G45350	-1.10	-0.13	-1.47	0.186165	0.311116	-6.18
256791_at	hypothetical protein predicted by ge	---	---	-1.17	-0.23	-1.47	0.186218	0.311182	-6.18
262529_at	putative receptor protein kinase Ap	AtRLP3	AT1G17250	1.16	0.21	1.47	0.18625	0.311212	-6.18
266151_x_a	En/Spm transposon protein (mosaic	---	---	-1.13	-0.17	-1.47	0.186331	0.311326	-6.18
263644_at	hypothetical protein predicted by ge	---	AT2G04730	-1.13	-0.17	-1.47	0.186355	0.311343	-6.18
262857_at	major latex homologue type2 identi	---	AT1G14930	-1.11	-0.15	-1.47	0.18642	0.311425	-6.18
262156_at	hypothetical protein contains similar	---	AT1G52680	-1.14	-0.19	-1.47	0.186432	0.311425	-6.18
246973_at	putative protein UBIQUINONE BIO	---	AT5G24970	1.20	0.26	1.47	0.186622	0.31172	-6.18
263462_at	similar to Ariadne protein from Dros	ARI11	AT2G31780	-1.12	-0.16	-1.47	0.186636	0.311721	-6.18
AFFX-DapX	B. subtilis /GEN=jojG /DB_XREF=g	---	---	1.15	0.20	1.47	0.186655	0.311729	-6.18
246170_at	putative protein various predicted p	---	AT5G32450	-1.14	-0.19	-1.47	0.18668	0.311748	-6.18
255369_at	putative NifU-like metallocluster ass	ISU3	AT4G04080	-1.13	-0.18	-1.47	0.186709	0.311774	-6.18
245827_at	disease resistance protein RPS4, p	---	AT1G57830	-1.15	-0.20	-1.47	0.18673	0.31178	-6.18
260150_at	putative oxidoreductase similar to g	---	AT1G52820	-1.14	-0.18	-1.47	0.18674	0.31178	-6.18
261843_at	unknown protein	---	AT1G16180	1.11	0.15	1.47	0.186775	0.311816	-6.18
261946_at	unknown protein	---	AT1G64560	-1.16	-0.21	-1.47	0.1869	0.311996	-6.18
250700_at	CDK5 activator-binding protein-like	---	AT5G06830	1.13	0.18	1.47	0.18691	0.311996	-6.18
245105_at	hypothetical protein predicted by ge	---	AT2G41610	-1.22	-0.29	-1.47	0.186953	0.312023	-6.18
245675_at	hypothetical protein similar to putati	---	---	-1.18	-0.24	-1.47	0.186954	0.312023	-6.18
263783_at	putative WRKY-type DNA binding p	WRKY46	AT2G46400	1.64	0.72	1.47	0.186986	0.312038	-6.18
258228_at	unknown protein similar to unknown	---	AT3G27610	1.11	0.15	1.47	0.187001	0.312038	-6.18
250456_at	26S proteasome p55 protein-like ;	EMB2107	AT5G09900	1.17	0.23	1.47	0.187004	0.312038	-6.18
247467_at	putative protein predicted proteins,	---	AT5G62130	-1.27	-0.34	-1.47	0.187022	0.312046	-6.18
256896_at	hypothetical protein predicted by ge	---	AT3G24630	-1.26	-0.34	-1.47	0.18708	0.312119	-6.18
262125_at	heat shock protein, putative similar	EDA1	AT1G59680	-1.13	-0.17	-1.47	0.187097	0.312125	-6.18
256187_at	55 kDa B regulatory subunit of phos	ATB ALPH/	AT1G51690	1.15	0.20	1.47	0.18713	0.312145	-6.18
260447_at	hypothetical protein similar to N-terr	---	AT1G72410	-1.11	-0.14	-1.47	0.187138	0.312145	-6.18
256636_at	S-locus related protein SLR1 homo	---	AT3G12000	-1.11	-0.16	-1.47	0.18715	0.312145	-6.18
260640_at	viral resistance protein, putative sir	---	AT1G53350	1.30	0.38	1.47	0.187206	0.312215	-6.18
264896_at	Hypothetical protein similar to endo	AtGH9B6	AT1G23210	-1.12	-0.17	-1.47	0.187265	0.31229	-6.18
246481_s_a	cold and ABA inducible protein kin1	KIN1	AT5G15960 /	-1.88	-0.91	-1.47	0.18728	0.312292	-6.18
251123_at	putative protein predicted proteins,	---	AT5G01030	1.11	0.15	1.47	0.187293	0.312292	-6.18
264948_at	RNA helicase, putative similar to R	---	AT1G77030	1.11	0.16	1.47	0.18743	0.312497	-6.18
265925_at	hypothetical protein predicted by ge	---	AT2G18610	-1.12	-0.16	-1.47	0.187496	0.312584	-6.18
247337_at	putative protein similar to unknown	PDF2.5	AT5G63660	-1.10	-0.14	-1.47	0.18751	0.312585	-6.18

263593_at	hypothetical protein predicted by ge	EMB975	AT2G01860	1.16	0.21	1.47	0.187547	0.312593	-6.18
246330_at	aldehyde oxidase ; supported by cl	AAO2	AT3G43600	1.33	0.41	1.47	0.187577	0.312593	-6.18
247016_at	putative protein contains similarity t	---	AT5G66970	1.13	0.18	1.47	0.187584	0.312593	-6.18
266209_at	similar to terminal flower ; supporte	ATC	AT2G27550	-1.21	-0.28	-1.47	0.187587	0.312593	-6.18
257322_at	hypothetical protein	AT2G0766	---	-1.14	-0.19	-1.47	0.187596	0.312593	-6.18
260172_s_a	putative histidinol-phosphate amino	---	AT1G71920	1.17	0.23	1.47	0.18761	0.312593	-6.18
251971_at	glucosyltransferase - like protein gl	UGT73C7	AT3G53160	1.83	0.87	1.47	0.18761	0.312593	-6.18
257064_at	hypothetical protein predicted by ge	---	AT3G18260	1.16	0.21	1.47	0.187695	0.312711	-6.18
257273_at	hypothetical protein contains Pfam	UVH3	AT3G28030	1.15	0.20	1.47	0.187712	0.312716	-6.18
248053_at	putative protein similar to unknown	AtNMNAT	AT5G55810	1.14	0.19	1.47	0.187734	0.312731	-6.18
260510_at	hypothetical protein predicted by ge	---	AT1G51580	-1.22	-0.29	-1.47	0.187791	0.312784	-6.18
263407_at	bZIP protein (AtbZIP48) to be publis	AtbZIP48	AT2G04038	-1.12	-0.16	-1.47	0.187794	0.312784	-6.18
252601_s_a	40S ribsomomal protein 40S ribsor	---	AT3G45030	1.25	0.32	1.47	0.187821	0.312807	-6.18
245961_at	putative protein predicted proteins -	---	AT5G19670	1.15	0.20	1.47	0.187846	0.312807	-6.18
266644_at	unknown protein ;supported by full-	---	AT2G29660	1.16	0.22	1.47	0.187849	0.312807	-6.18
246678_at	putative protein predicted replicatio	---	---	1.14	0.18	1.47	0.187922	0.312906	-6.18
249191_at	putative protein similar to unknown	---	AT5G42760	-1.17	-0.22	-1.47	0.187979	0.312965	-6.18
245126_at	putative MYB family transcription fa	MYB12	AT2G47460	-1.11	-0.16	-1.47	0.187985	0.312965	-6.19
267264_at	putative serine carboxypeptidase II	SCPL11	AT2G22970	1.38	0.47	1.46	0.18807	0.313084	-6.19
250379_at	transcription factor like protein tran	TINY2	AT5G11590	-1.11	-0.15	-1.46	0.188086	0.313087	-6.19
257742_at	hypothetical protein predicted by ge	SDH2-1	AT3G27380	-1.12	-0.17	-1.46	0.188116	0.313115	-6.19
254111_at	putative protein acid phosphatase (	PAP24	AT4G24890	-1.20	-0.26	-1.46	0.188154	0.313155	-6.19
256462_at	hypothetical protein predicted by ge	---	AT1G36230	-1.14	-0.18	-1.46	0.188203	0.313213	-6.19
266020_at	hypothetical protein	SDG11	AT2G05900	-1.16	-0.21	-1.46	0.18827	0.313302	-6.19
249183_at	E2, ubiquitin-conjugating enzyme 1	UBC18	AT5G42990	1.11	0.15	1.46	0.188346	0.313407	-6.19
252682_at	putative protein AtOXA1, Arabidop	---	AT3G44370	-1.15	-0.20	-1.46	0.188376	0.313432	-6.19
256024_at	unknown protein contains Pfam pro	ZF14	AT1G58340	1.35	0.44	1.46	0.188389	0.313432	-6.19
262826_at	putative cytochrome P450 monoox	CYP71B2	AT1G13080	1.29	0.37	1.46	0.188505	0.313602	-6.19
255362_at	putative reverse transcriptase	---	---	-1.10	-0.14	-1.46	0.188645	0.313812	-6.19
248092_at	ubiquitin-like protein	SUMO3	AT5G55170	-1.44	-0.52	-1.46	0.188735	0.313939	-6.19
261598_at	unknown protein contains similarity	---	AT1G49750	-1.69	-0.75	-1.46	0.188815	0.31405	-6.19
251950_at	zinc finger - like protein Zat11 zinc	---	AT3G53600	1.92	0.94	1.46	0.188949	0.314249	-6.19
263588_s_a	unknown protein	---	AT2G25320	-1.12	-0.16	-1.46	0.189037	0.314373	-6.19
251080_at	putative protein hypothetical protein	ROPGEF7	AT5G02010	1.32	0.40	1.46	0.189092	0.314441	-6.19
255467_at	putative leucine-rich repeat protein	---	AT4G03010	-1.12	-0.17	-1.46	0.189135	0.314489	-6.19

265657_at	unknown protein ; supported by cD	---	AT2G13840	1.13	0.18	1.46	0.189156	0.314501	-6.19
253731_at	acid phosphatase-like protein acid	---	AT4G29260	1.10	0.14	1.46	0.189176	0.314512	-6.19
259478_at	germin, putative similar to germin G	---	AT1G18980	2.02	1.02	1.46	0.189198	0.314526	-6.19
260967_at	unknown protein contains similarity	---	AT1G12230	-1.16	-0.21	-1.46	0.189232	0.314537	-6.19
260757_at	phytocyanin, putative similar to phy	---	AT1G48940	-1.11	-0.15	-1.46	0.189233	0.314537	-6.19
249560_at	putative protein	LCR80	AT5G38330	-1.20	-0.26	-1.46	0.18928	0.314593	-6.19
258061_at	unknown protein ; supported by cD	---	AT3G25910	1.13	0.17	1.46	0.1893	0.314603	-6.19
263933_s_a	putative harpin-induced protein	---	AT2G35970	-1.13	-0.17	-1.46	0.189313	0.314603	-6.19
260068_at	putative calmodulin-binding protein	---	AT1G73805	-1.44	-0.53	-1.46	0.189398	0.314721	-6.19
260437_at	hypothetical protein predicted by ge	---	AT1G68380	-1.12	-0.17	-1.46	0.189469	0.314816	-6.19
267342_at	putative heme A:farnesyltransferase	COX10	AT2G44520	1.20	0.26	1.46	0.189503	0.31485	-6.19
262621_at	hypothetical protein	---	AT1G06530	1.12	0.17	1.46	0.189675	0.315109	-6.19
267583_at	unknown protein	---	AT2G41960	-1.10	-0.13	-1.46	0.189715	0.315109	-6.19
245432_at	putative serine protease-like protein	---	AT4G17100	-1.10	-0.14	-1.46	0.189719	0.315109	-6.19
257790_at	gda-1, putative similar to gda-1 GB	---	AT3G27090	1.16	0.22	1.46	0.189724	0.315109	-6.19
265737_at	putative phosphatidic acid phospha	ATPAP1	AT2G01180	-1.59	-0.67	-1.46	0.189735	0.315109	-6.19
258537_at	putative disease resistance protein	---	AT3G04210	-1.57	-0.65	-1.46	0.189742	0.315109	-6.19
245638_s_a	F5A9.10 unknown protein; similar t	---	AT1G24822	-1.12	-0.17	-1.46	0.189819	0.315214	-6.19
254533_at	putative protein ARI protein - Dros	---	AT4G19670	1.19	0.25	1.46	0.189849	0.315241	-6.19
261095_at	unknown protein	---	AT1G62930	1.14	0.19	1.46	0.189971	0.31542	-6.19
249436_at	putative protein crp1 protein - Zea	---	AT5G39980	1.20	0.26	1.46	0.19007	0.315561	-6.19
248285_at	putative protein similar to unknown	---	AT5G52960	-1.09	-0.13	-1.46	0.190085	0.315563	-6.20
247484_at	putative protein predicted proteins,	---	AT5G62110	-1.12	-0.16	-1.46	0.190281	0.315866	-6.20
259918_at	putative AP2 domain transcription fa	---	AT1G72570	-1.11	-0.15	-1.46	0.1903	0.315874	-6.20
255497_at	hypothetical protein similar to A. th	UNE12	AT4G02590	1.12	0.16	1.46	0.190333	0.315905	-6.20
265762_at	hypothetical protein	---	AT2G01240	-1.18	-0.23	-1.46	0.190428	0.31604	-6.20
255343_at	hypothetical protein	---	---	-1.09	-0.13	-1.46	0.190468	0.316085	-6.20
246101_at	putative protein	---	---	-1.13	-0.18	-1.46	0.190486	0.316091	-6.20
254414_at	hypothetical protein	HSA32	AT4G21320	-1.16	-0.21	-1.46	0.190561	0.316193	-6.20
248319_at	unknown protein	---	AT5G52710	-1.11	-0.15	-1.46	0.190614	0.316257	-6.20
258844_at	hypothetical protein predicted by ge	SWP	AT3G04740	1.11	0.15	1.46	0.190629	0.316259	-6.20
248599_at	putative protein contains similarity t	---	AT5G49380	-1.20	-0.27	-1.45	0.19072	0.316367	-6.20
265801_at	fertilization-independent seed 2 pro	FIS2	AT2G35670	-1.12	-0.17	-1.45	0.190722	0.316367	-6.20
246862_at	E2, ubiquitin-conjugating enzyme,	PEX4	AT5G25760	1.12	0.16	1.45	0.190872	0.316589	-6.20
252354_at	betaine aldehyde dehydrogenase-l	ALDH10A9	AT3G48170	1.11	0.15	1.45	0.190884	0.316589	-6.20

260492_at	putative polygalacturonase	PGAZAT	AT2G41850	-1.18	-0.24	-1.45	0.190903	0.316589	-6.20
265324_at	hypothetical protein predicted by ge	ATCOAD	AT2G18250	1.15	0.20	1.45	0.190911	0.316589	-6.20
246145_at	peroxidase peroxidase, Lycopersic	---	AT5G19880	1.39	0.47	1.45	0.190931	0.316598	-6.20
252776_at	putative protein predicted proteins,	---	AT3G42940	-1.14	-0.19	-1.45	0.191065	0.316798	-6.20
258348_at	unknown protein ;supported by full-	---	AT3G17710	1.13	0.17	1.45	0.191083	0.316805	-6.20
260508_at	Unknown protein ; supported by cD	GAMMA C/	AT1G47260	-1.11	-0.15	-1.45	0.191175	0.316935	-6.20
255475_at	contains a short region of similarity	---	AT4G02480	-1.15	-0.20	-1.45	0.191248	0.317032	-6.20
259704_at	putative 3'-5' exoribonuclease simila	---	AT1G77680	-1.15	-0.21	-1.45	0.191268	0.317036	-6.20
259022_at	putative asparaginyl-tRNA syntheta	NS2	AT3G07420	-1.20	-0.26	-1.45	0.191278	0.317036	-6.20
246143_at	lipophosphoglycan biosynthetic pro	---	AT5G19980	-1.18	-0.24	-1.45	0.191317	0.317044	-6.20
263162_at	hypothetical protein predicted by ge	---	AT1G54150	-1.10	-0.14	-1.45	0.191321	0.317044	-6.20
247521_at	putative protein ATP/GTP nucleotid	---	AT5G61450	1.14	0.18	1.45	0.191325	0.317044	-6.20
260303_at	putative protein kinase similar to C	---	AT1G70520	1.28	0.35	1.45	0.191357	0.317069	-6.20
256834_at	cyclophilin, putative similar to cyclo	---	AT3G22920	-1.11	-0.15	-1.45	0.191367	0.317069	-6.20
261928_at	uclacyanin II, putative similar to ucl	---	AT1G22480	-1.19	-0.25	-1.45	0.191431	0.317144	-6.20
265210_at	60S ribosomal protein L24 ;suppor	RPL24A	AT2G36620	1.44	0.53	1.45	0.19144	0.317144	-6.20
259135_at	hypothetical protein predicted by ge	RAD9	AT3G05480	-1.19	-0.25	-1.45	0.19147	0.317171	-6.20
267487_at	putative non-LTR retroelement reve	---	---	-1.13	-0.18	-1.45	0.191685	0.317503	-6.20
245898_at	ser/thr specific protein kinase-like p	---	AT5G11020	-1.16	-0.21	-1.45	0.191708	0.317509	-6.20
246355_at	hypothetical protein	---	---	-1.26	-0.33	-1.45	0.191716	0.317509	-6.20
267032_at	putative protein kinase contains a p	CIPK22	AT2G38490	-1.20	-0.26	-1.45	0.191745	0.317531	-6.20
263955_at	putative E2F5 family transcription fa	E2F3	AT2G36010	-1.19	-0.25	-1.45	0.191758	0.317531	-6.20
256347_at	unknown protein similar to putative	---	---	1.13	0.17	1.45	0.191837	0.31764	-6.20
254584_at	putative protein probable membran	VPS54	AT4G19490	-1.11	-0.15	-1.45	0.19189	0.317682	-6.20
245051_at	putative WRKY-type DNA-binding p	WRKY15	AT2G23320	1.31	0.39	1.45	0.19189	0.317682	-6.20
266504_at	hypothetical protein predicted by ge	---	AT2G47820	1.15	0.20	1.45	0.191974	0.317797	-6.20
250068_at	CHP-rich zinc finger protein-like	---	AT5G17960	-1.15	-0.20	-1.45	0.19201	0.317834	-6.20
262250_at	Expressed protein predicted by gen	---	AT1G48280	-1.47	-0.56	-1.45	0.192057	0.317888	-6.20
252134_at	putative protein ; supported by cDN	---	AT3G50910	1.33	0.41	1.45	0.192252	0.318188	-6.21
257262_at	zinc finger protein, putative contain	---	AT3G21890	-1.24	-0.32	-1.45	0.1923	0.318244	-6.21
261261_at	putative aldehyde oxidase	---	AT1G26730	-1.54	-0.62	-1.45	0.192473	0.318508	-6.21
249114_at	putative protein similar to unknown	---	---	-1.16	-0.21	-1.45	0.192494	0.318519	-6.21
265554_at	hypothetical protein predicted by ge	---	AT2G07505	-1.10	-0.14	-1.45	0.192543	0.318578	-6.21
257501_at	hypothetical protein predicted by ge	---	---	-1.09	-0.13	-1.45	0.192583	0.318597	-6.21
256029_at	integral membrane protein, putative	STT3B	AT1G34130	1.26	0.33	1.45	0.192587	0.318597	-6.21

267293_at	hypothetical protein	TET8	AT2G23810	1.23	0.30	1.45	0.192608	0.318597	-6.21
248640_at	selenium-binding protein-like ; supp	---	AT5G48910	1.14	0.19	1.45	0.192611	0.318597	-6.21
259019_at	hypothetical protein similar to serolo	CHIP	AT3G07370	1.13	0.17	1.45	0.192645	0.318631	-6.21
260520_at	mannosyl-oligosaccharide alpha-1,	---	AT1G51590	1.13	0.18	1.45	0.192771	0.318806	-6.21
249083_at	prohibitin non-consensus TT accep	ATPHB7	AT5G44140	1.12	0.16	1.45	0.192779	0.318806	-6.21
263703_at	unknown protein ;supported by full-	SRX	AT1G31170	1.14	0.19	1.45	0.192844	0.31889	-6.21
247017_at	putative protein similar to unknown	---	AT5G66980	-1.10	-0.13	-1.45	0.192987	0.319103	-6.21
260741_at	hypothetical protein contains Pfam	---	AT1G15040	2.56	1.36	1.45	0.193031	0.319153	-6.21
254128_at	ubiquitin-specific protease 16 (UBF	UBP16	AT4G24560	1.09	0.13	1.45	0.193051	0.319163	-6.21
249389_at	disease resistence like - protein do	---	AT5G40100	-1.15	-0.21	-1.45	0.193164	0.319307	-6.21
262144_at	hypothetical protein predicted by ge	---	AT1G52620	1.11	0.15	1.45	0.193166	0.319307	-6.21
257213_at	mitochondrial NAD-dependent mal	---	AT3G15020	1.23	0.29	1.44	0.193453	0.319756	-6.21
245833_at	transposon protein, putative similar	---	---	-1.13	-0.18	-1.44	0.193466	0.319756	-6.21
249771_at	receptor-like protein kinase ; suppo	---	AT5G24080	-1.20	-0.27	-1.44	0.193495	0.319782	-6.21
256206_at	hypothetical protein predicted by ge	---	AT1G50910	-1.17	-0.23	-1.44	0.193533	0.319821	-6.21
261867_at	unknown protein ; supported by cDI	---	AT1G50575	-1.14	-0.19	-1.44	0.193601	0.31991	-6.21
264303_s_a	E2, ubiquitin-conjugating enzyme,	UBC35	AT1G16890	1.12	0.16	1.44	0.193634	0.319941	-6.21
256854_at	hypothetical protein predicted by ge	---	AT3G15180	1.11	0.16	1.44	0.193693	0.320003	-6.21
246685_at	putative protein nucleoid DNA-bind	---	AT5G33350	-1.23	-0.30	-1.44	0.1937	0.320003	-6.21
253971_at	fructose-bisphosphate aldolase - lik	---	AT4G26530	1.28	0.36	1.44	0.19372	0.320015	-6.21
266969_at	similar to gibberellin-regulated prote	---	AT2G39540	-1.15	-0.20	-1.44	0.193782	0.320094	-6.21
255321_at	putative ES43-like protein	---	AT4G04260	1.13	0.17	1.44	0.193898	0.320261	-6.21
251591_at	carboxyl terminal protease - like pr	---	AT3G57680	1.23	0.30	1.44	0.193917	0.320271	-6.21
251034_at	putative protein ;supported by full-l	PRA1.A1	AT5G02040	1.13	0.17	1.44	0.193986	0.32036	-6.21
253483_at	putative protein anthranilate N-hyd	---	AT4G31910	-1.23	-0.30	-1.44	0.194103	0.320531	-6.21
263886_at	hypothetical protein predicted by ge	TKI1	AT2G36960	-1.10	-0.14	-1.44	0.1942	0.320659	-6.21
255092_s_a	putative protein various predicted p	---	---	-1.12	-0.16	-1.44	0.194209	0.320659	-6.21
254556_at	putative protein various predicted p	---	AT4G19220	1.18	0.24	1.44	0.194245	0.320696	-6.21
252498_at	putative protein several hypothetica	---	AT3G46810	-1.10	-0.14	-1.44	0.194404	0.320934	-6.22
258507_at	neutral invertase, putative similar to	---	AT3G06500	1.31	0.39	1.44	0.194512	0.32109	-6.22
262461_at	myosin, putative similar to myosin	VIIIA	AT1G50360	1.23	0.30	1.44	0.19453	0.321097	-6.22
254098_at	superoxide dismutase (EC 1.15.1.1	FSD1	AT4G25100	1.16	0.21	1.44	0.194658	0.321284	-6.22
256032_at	polyprotein, putative similar to poly	---	---	-1.09	-0.12	-1.44	0.194686	0.321306	-6.22
250162_at	FtsH protease, putative similar to F	FTSH6	AT5G15250	-1.10	-0.14	-1.44	0.194768	0.321404	-6.22
256973_at	unknown protein similar to putative	---	AT3G20980	-1.12	-0.16	-1.44	0.194786	0.321404	-6.22

255942_at	UDP-glucose glucosyltransferase id	AtUGT85A	AT1G22360	-1.48	-0.56	-1.44	0.194787	0.321404	-6.22
267266_at	putative metal ion transporter (NRA	NRAMP3	AT2G23150	1.12	0.16	1.44	0.194827	0.321438	-6.22
254644_at	CLE2, putative CLAVATA3/ESR-R	CLE2	AT4G18510	-1.14	-0.18	-1.44	0.194835	0.321438	-6.22
253543_at	hypothetical protein	---	AT4G31270	-1.12	-0.16	-1.44	0.194878	0.321484	-6.22
259900_at	c-Jun coactivator-like protein (AJH2	CSN5B	AT1G71230	1.10	0.14	1.44	0.194918	0.321527	-6.22
266722_at	unknown protein	FUT2	AT2G03210	-1.13	-0.18	-1.44	0.194971	0.321591	-6.22
256680_at	unknown protein ; supported by full-	---	AT3G52230	-1.18	-0.24	-1.44	0.195008	0.321629	-6.22
254917_at	putative protein various hypothetica	---	AT4G11350	1.20	0.27	1.44	0.195039	0.321657	-6.22
263952_s_a	unknown protein ;supported by full-	---	AT2G35810	1.12	0.16	1.44	0.195203	0.321904	-6.22
245235_at	putative protein similarity to predict	---	AT4G25550	1.13	0.17	1.44	0.195244	0.321949	-6.22
264407_at	unknown protein similar to EST gb|	---	AT1G10180	1.15	0.20	1.44	0.195293	0.322007	-6.22
253620_at	receptor-like kinase homolog soma	---	AT4G30520	1.13	0.18	1.44	0.19536	0.322094	-6.22
248618_at	myb-related transcription factor	AtMYB78	AT5G49620	1.14	0.19	1.44	0.19541	0.322153	-6.22
251049_at	putative protein rab11 binding prote	---	AT5G02430	-1.13	-0.18	-1.44	0.195447	0.32219	-6.22
250186_at	putative protein apospory-associate	---	AT5G14500	1.15	0.20	1.44	0.19547	0.322204	-6.22
264767_at	hypothetical protein similar to putati	SD1-29	AT1G61380	1.35	0.44	1.44	0.195526	0.322274	-6.22
249234_at	putative protein similar to unknown	---	AT5G42200	-1.24	-0.31	-1.44	0.195598	0.32237	-6.22
256349_at	late embryogenesis protein, putativ	---	AT1G54890	2.72	1.44	1.44	0.195709	0.322529	-6.22
267131_at	hypothetical protein predicted by ge	---	AT2G23400	1.10	0.14	1.44	0.195791	0.32264	-6.22
258574_at	hypothetical protein	---	AT3G04250	-1.12	-0.16	-1.44	0.19588	0.322764	-6.22
249297_at	unknown protein	---	AT5G41320	-1.11	-0.15	-1.44	0.195987	0.322918	-6.22
258558_at	hypothetical protein predicted by ge	---	AT3G06000	-1.09	-0.12	-1.44	0.196004	0.322922	-6.22
248544_at	putative protein contains similarity t	PIMT2	AT5G50240	1.24	0.31	1.44	0.196026	0.322935	-6.22
248256_at	developmental protein SINA (seven	---	AT5G53360	-1.13	-0.18	-1.43	0.196136	0.323093	-6.22
264552_at	hypothetical protein predicted by ge	---	AT1G09470	-1.19	-0.26	-1.43	0.196198	0.323171	-6.22
246344_at	putative protein predicted proteins -	---	AT3G56730	1.24	0.32	1.43	0.196247	0.323229	-6.22
249609_at	putative protein predicted proteins,	---	---	-1.11	-0.15	-1.43	0.196281	0.323262	-6.22
245329_at	Expressed protein ; supported by fu	---	AT4G14365	-1.35	-0.43	-1.43	0.19631	0.323285	-6.22
256816_at	unknown protein	---	AT3G21400	1.13	0.18	1.43	0.196374	0.323367	-6.22
247532_at	putative protein disease resistance	---	AT5G61560	1.40	0.48	1.43	0.196453	0.323441	-6.22
247431_at	putative protein similar to unknown	SRO5	AT5G62520	-1.29	-0.37	-1.43	0.19647	0.323441	-6.22
256967_at	hypothetical protein predicted by g	---	AT3G21060	-1.13	-0.18	-1.43	0.196471	0.323441	-6.22
251716_at	anthranilate synthase alpha-1 chai	---	AT3G55870	-1.11	-0.15	-1.43	0.196494	0.323441	-6.22
246930_at	KNAT3 homeodomain protein ;sup	KNAT3	AT5G25220	1.15	0.20	1.43	0.196496	0.323441	-6.22
260962_at	pectin methylesterase, putative sim	PME7	AT1G44980	1.13	0.17	1.43	0.196508	0.323441	-6.22

263103_at	putative Ta11-like non-LTR retroele	---	---	-1.12	-0.17	-1.43	0.196528	0.323441	-6.23
252191_at	putative protein various predicted g	---	AT3G50180	-1.14	-0.18	-1.43	0.196532	0.323441	-6.23
263935_at	unknown protein	PUB23	AT2G35930	2.02	1.02	1.43	0.196612	0.323549	-6.23
254413_at	myb-related protein M4 ;supported	ATMYB102	AT4G21440	-1.21	-0.27	-1.43	0.196628	0.323552	-6.23
266007_at	unknown protein	---	AT2G37380	-1.15	-0.20	-1.43	0.196719	0.323679	-6.23
253523_at	putative protein myosin heavy chai	---	AT4G31340	-1.12	-0.17	-1.43	0.196771	0.323741	-6.23
265046_s_a	mutator-like transposase, putative s	---	---	-1.12	-0.16	-1.43	0.196837	0.323826	-6.23
257384_at	unknown protein	---	AT2G15110	-1.12	-0.16	-1.43	0.196963	0.32401	-6.23
252953_at	putative phosphatidylinositol syntha	PIS2	AT4G38570	-1.12	-0.16	-1.43	0.197036	0.324107	-6.23
245048_at	orf within trnK intron orf within trnK	---	---	-1.18	-0.23	-1.43	0.1971	0.324189	-6.23
251352_at	putative protein hypothetical protei	PEX11E	AT3G61070	1.16	0.22	1.43	0.197265	0.324437	-6.23
246255_at	transcription initiation factor like pro	ATPBRP	AT4G36650	1.13	0.18	1.43	0.197427	0.32468	-6.23
252761_at	putative protein several hypothetica	---	AT3G42770	-1.20	-0.26	-1.43	0.197519	0.324808	-6.23
260795_at	CLE3, putative CLAVATA3/ESR-R	CLE3	AT1G06225	-1.12	-0.16	-1.43	0.197563	0.324838	-6.23
252310_at	GTPase activating -like protein GT	---	AT3G49350	-1.25	-0.32	-1.43	0.19759	0.324838	-6.23
247576_at	putative protein predicted protein, A	---	AT5G61280	-1.09	-0.13	-1.43	0.19759	0.324838	-6.23
246416_at	putative protein predicted proteins,	---	AT5G16920	-1.12	-0.16	-1.43	0.197603	0.324838	-6.23
264080_at	putative vacuolar proton-ATPase su	VHA-A1	AT2G28520	-1.10	-0.14	-1.43	0.197608	0.324838	-6.23
264268_at	hypothetical protein predicted by ge	ULP1D	AT1G60220	1.11	0.15	1.43	0.197667	0.324911	-6.23
261790_at	unknown protein ; supported by full-	---	AT1G16000	-1.20	-0.26	-1.43	0.197705	0.324949	-6.23
263928_at	hypothetical protein predicted by ge	---	AT2G36330	1.18	0.24	1.43	0.197731	0.324968	-6.23
264305_at	Expressed protein ; supported by fu	LSH7	AT1G78815	-1.11	-0.15	-1.43	0.197772	0.325014	-6.23
248910_at	serine threonine protein kinase ; su	CIPK20	AT5G45820	-1.23	-0.30	-1.43	0.197805	0.325044	-6.23
266061_at	putative translation initiation factor e	---	AT2G18720	-1.13	-0.17	-1.43	0.19784	0.325058	-6.23
247940_at	phosphatidylserine decarboxylase	PSD2	AT5G57190	1.40	0.48	1.43	0.197852	0.325058	-6.23
265052_at	caffeic acid O-methyltransferase, p	---	AT1G51990	-1.10	-0.14	-1.43	0.197857	0.325058	-6.23
250860_at	amino acid transport - like protein a	CAT6	AT5G04770	-1.28	-0.36	-1.43	0.197955	0.325197	-6.23
257363_at	hypothetical protein predicted by ge	BAP2	AT2G45760	-1.17	-0.22	-1.43	0.197987	0.325226	-6.23
266406_at	hypothetical protein predicted by ge	---	AT2G38570	-1.14	-0.19	-1.43	0.198103	0.325363	-6.23
254572_at	putative protein predicted protein M	---	AT4G19380	-1.12	-0.16	-1.43	0.198105	0.325363	-6.23
264496_at	putative polyprotein similar to GB:A	---	---	-1.14	-0.19	-1.43	0.198114	0.325363	-6.23
253959_at	putative protein	---	AT4G26410	1.11	0.15	1.43	0.198269	0.32552	-6.23
249611_at	putative protein unnamed ORF, Ho	ATSRL1	AT5G37370	1.15	0.20	1.43	0.198273	0.32552	-6.23
257873_at	P-glycoprotein, putative similar to P	PGP17	AT3G28380	-1.15	-0.20	-1.43	0.198275	0.32552	-6.23
262630_at	unknown protein ;supported by full-	GPAT1	AT1G06520	1.51	0.60	1.43	0.198278	0.32552	-6.23

256214_x_a	hypothetical protein predicted by ge	---	AT1G51000	-1.12	-0.16	-1.43	0.19829	0.32552	-6.23
254680_at	phytochrome E	PHYE	AT4G18130	1.16	0.21	1.43	0.198295	0.32552	-6.23
260769_at	Expressed protein ; supported by fu	---	AT1G49010	1.23	0.29	1.43	0.198393	0.325643	-6.23
253961_at	putative protein SPF1 protein, Ipon	WRKY34	AT4G26440	-1.10	-0.14	-1.43	0.198398	0.325643	-6.23
267606_at	putative beta-ketoacyl-CoA synthas	KCS11	AT2G26640	-1.10	-0.14	-1.43	0.198497	0.325763	-6.23
264580_at	unknown protein EST gb|ATTS0295	---	AT1G05340	-1.28	-0.36	-1.43	0.1985	0.325763	-6.23
266929_at	putative AT-hook DNA-binding prote	---	AT2G45850	-1.12	-0.17	-1.43	0.198535	0.325798	-6.23
264711_at	hypothetical protein predicted by ge	---	AT1G09800	1.12	0.16	1.43	0.198572	0.325834	-6.23
AFFX-r2-Bs	B. subtilis /GEN=jojG /DB_XREF=g	---	---	1.21	0.27	1.43	0.198655	0.325947	-6.23
248452_at	putative protein contains similarity t	---	AT5G51300	1.09	0.13	1.42	0.198905	0.326334	-6.24
253859_at	Expressed protein ; supported by fu	---	AT4G27657	2.13	1.09	1.42	0.198991	0.326447	-6.24
247739_at	40S ribosomal protein S8 - like 40S	---	AT5G59240	1.16	0.22	1.42	0.199003	0.326447	-6.24
257703_at	unknown protein ; supported by full-	---	AT3G12640	1.10	0.14	1.42	0.199029	0.326466	-6.24
249852_at	monosaccharide transporter	STP11	AT5G23270	-1.13	-0.17	-1.42	0.199045	0.326469	-6.24
261936_at	seed maturation protein PM27, put	---	AT1G22600	-1.17	-0.23	-1.42	0.199074	0.326494	-6.24
266739_at	unknown protein predicted by gens	---	AT2G46730	1.12	0.16	1.42	0.199288	0.326821	-6.24
248965_at	nodulin-like protein	---	AT5G45370	-1.16	-0.21	-1.42	0.199327	0.32686	-6.24
262445_at	Expressed protein ; supported by fu	---	AT1G47485	-1.12	-0.16	-1.42	0.19934	0.32686	-6.24
264155_at	unknown protein	GTB1	AT1G65440	1.18	0.24	1.42	0.199358	0.326866	-6.24
249112_at	ATP sulfurylase precursor (gb|AAD	APS4	AT5G43780	1.24	0.32	1.42	0.19942	0.326944	-6.24
263208_at	zinc finger protein 5, ZFP5 possible	ZFP5	AT1G10480	-1.15	-0.21	-1.42	0.199548	0.32713	-6.24
254135_at	putative protein MSP1 protein, Sac	---	AT4G24860	-1.13	-0.17	-1.42	0.199579	0.327157	-6.24
249796_at	26S proteasome, non-ATPase regu	---	AT5G23540	1.12	0.16	1.42	0.199723	0.327348	-6.24
254138_at	hypothetical protein	---	AT4G24950	-1.11	-0.16	-1.42	0.199724	0.327348	-6.24
253100_at	cytochrome P450 monooxygenase	CYP81F3	AT4G37400	1.43	0.52	1.42	0.199802	0.327445	-6.24
266965_at	nodulin-like protein	---	AT2G39510	-1.12	-0.16	-1.42	0.199812	0.327445	-6.24
265515_at	hypothetical protein predicted by ge	---	---	-1.12	-0.16	-1.42	0.199853	0.327467	-6.24
253654_at	putative protein several hypothetica	---	AT4G30060	-1.28	-0.36	-1.42	0.199854	0.327467	-6.24
250904_at	subtilisin-like serine protease conta	---	AT5G03620	-1.11	-0.15	-1.42	0.199929	0.327567	-6.24
254748_at	unknown protein	---	---	-1.11	-0.16	-1.42	0.200022	0.327695	-6.24
249967_at	putative protein	---	AT5G19050	1.12	0.17	1.42	0.200037	0.327697	-6.24
258341_at	unknown protein similar to centrome	---	AT3G22790	-1.44	-0.52	-1.42	0.200186	0.327917	-6.24
251905_at	putative protein zinc finger protein	GAPB	AT1G42970	-1.13	-0.18	-1.42	0.200227	0.32796	-6.24
263951_at	putative harpin-induced protein ;su	NHL12	AT2G35960	-1.47	-0.56	-1.42	0.200425	0.328262	-6.24
262596_at	putative phosphatidic acid phospha	LPP2	AT1G15080	-1.14	-0.18	-1.42	0.200494	0.328348	-6.24

264876_at	hypothetical protein	---	AT2G17410	-1.13	-0.17	-1.42	0.200506	0.328348	-6.24
264422_at	unknown protein similar to unknowr	LCV2	AT1G43130	1.13	0.17	1.42	0.200577	0.328415	-6.24
264694_at	receptor serine/threonine kinase P	---	AT1G70250	1.10	0.14	1.42	0.2006	0.328415	-6.24
246770_at	putative protein DNA-binding prote	---	AT5G27460	1.16	0.21	1.42	0.200607	0.328415	-6.24
245513_at	SYBL1 like protein	ATVAMP72	AT4G15780	1.24	0.31	1.42	0.200632	0.328415	-6.24
246720_at	putative protein predicted proteins,	---	AT5G28950	-1.09	-0.12	-1.42	0.200638	0.328415	-6.24
249831_at	putative protein contains similarity t	---	AT5G23340	1.24	0.31	1.42	0.200669	0.328415	-6.24
253241_at	fatty acid elongase 1	KCS18	AT4G34520	-1.17	-0.23	-1.42	0.200669	0.328415	-6.24
245192_at	F12A21.9 hypothetical protein	---	AT1G67780	1.10	0.14	1.42	0.200669	0.328415	-6.24
260212_at	hypothetical protein predicted by ge	---	AT1G74480	-1.10	-0.13	-1.42	0.200677	0.328415	-6.24
260204_at	hypothetical protein predicted by ge	---	AT1G52900	-1.12	-0.16	-1.42	0.200757	0.328522	-6.24
247169_at	unknown protein	---	AT5G65520	-1.17	-0.23	-1.42	0.20091	0.328749	-6.24
261264_at	hypothetical protein predicted by ge	---	AT1G26710	-1.15	-0.20	-1.42	0.200975	0.328813	-6.25
261910_at	zinc finger protein identical to GB:A	ZFP1	AT1G80730	-1.10	-0.14	-1.42	0.200978	0.328813	-6.25
266634_at	putative G-box binding bZIP transcr	---	AT2G35530	-1.11	-0.15	-1.42	0.201043	0.328896	-6.25
249478_at	germin - like protein GLP6 germin -	---	AT5G38950	-1.12	-0.16	-1.42	0.2011	0.328961	-6.25
254958_at	nucleoside diphosphate kinase 3 (n	NDPK3	AT4G11010	1.17	0.23	1.42	0.201112	0.328961	-6.25
264566_at	hypothetical protein predicted by ge	---	AT1G05270	-1.13	-0.18	-1.42	0.201149	0.328998	-6.25
245285_s_a	selenium-binding protein like ; supp	SBP1	AT4G14030 ,	-1.11	-0.15	-1.42	0.201396	0.329369	-6.25
264265_at	unknown protein ; supported by cD	---	AT1G09280	1.15	0.21	1.42	0.201425	0.329369	-6.25
261847_at	hypothetical protein predicted by ge	---	AT1G11560	-1.15	-0.20	-1.42	0.201457	0.329369	-6.25
263646_at	Ac-like transposase related to Ac/D	---	---	1.10	0.13	1.42	0.201458	0.329369	-6.25
263214_at	hypothetical protein predicted by ge	---	AT1G30660	1.14	0.19	1.42	0.20146	0.329369	-6.25
248885_at	putative protein similar to unknown	---	AT5G46150	-1.11	-0.15	-1.42	0.201463	0.329369	-6.25
251567_at	putative protein several hypothetica	---	AT3G58230	1.13	0.17	1.42	0.201477	0.329369	-6.25
265837_at	unknown protein	LURP1	AT2G14560	2.32	1.21	1.42	0.201521	0.329418	-6.25
247978_at	60S ribosomal protein L31 ;suppor	---	AT5G56710	1.31	0.39	1.42	0.201582	0.329493	-6.25
256373_at	hypothetical protein	SGA2	AT1G66740	1.16	0.21	1.42	0.201607	0.329497	-6.25
266221_at	putative nucleotide-sugar dehydrata	UXS6	AT2G28760	1.68	0.75	1.42	0.201613	0.329497	-6.25
245488_at	peroxidase like protein	---	AT4G16270	-1.12	-0.16	-1.42	0.201634	0.329507	-6.25
245344_at	F-box protein family, AtFBL4 conta	---	AT4G15475	-1.15	-0.20	-1.41	0.201744	0.329611	-6.25
260467_at	unknown protein	---	AT1G10890	-1.10	-0.13	-1.41	0.201746	0.329611	-6.25
245776_at	hypothetical protein predicted by ge	---	AT1G30260	1.23	0.30	1.41	0.201748	0.329611	-6.25
248781_at	putative protein similar to unknown	---	AT5G47870	-1.28	-0.36	-1.41	0.201755	0.329611	-6.25
266622_at	hypothetical protein predicted by ge	---	AT2G35430	1.14	0.19	1.41	0.201771	0.329613	-6.25

255905_at	tonoplast intrinsic protein, putative	BETA-TIP	AT1G17810	-1.22	-0.29	-1.41	0.201918	0.329831	-6.25
252860_at	putative protein bZIP protein, Arabi	---	AT4G39790	-1.10	-0.13	-1.41	0.201983	0.329913	-6.25
257014_at	hypothetical protein predicted by ge	---	AT3G26930	1.16	0.21	1.41	0.202016	0.329942	-6.25
265202_at	unknown protein	AT2G3681(	---	-1.13	-0.17	-1.41	0.202254	0.330307	-6.25
265329_at	putative succinate dehydrogenase f	SDH1-2	AT2G18450	-1.13	-0.17	-1.41	0.202336	0.330366	-6.25
245721_s_a	hypothetical protein similar to Ser-T	---	AT1G73450	1.17	0.23	1.41	0.202341	0.330366	-6.25
252727_s_a	putative protein predicted proteins,	---	---	1.10	0.13	1.41	0.202351	0.330366	-6.25
255460_at	hypothetical protein similar to A. tha	---	AT4G02800	-1.11	-0.15	-1.41	0.202359	0.330366	-6.25
262974_at	hypothetical protein predicted by ge	---	AT1G75550	1.12	0.16	1.41	0.202362	0.330366	-6.25
249819_at	unknown protein	---	AT5G23640	1.10	0.14	1.41	0.202452	0.330472	-6.25
262248_at	hypothetical protein predicted by ge	YSL8	AT1G48370	1.24	0.31	1.41	0.202463	0.330472	-6.25
259291_at	unknown protein similar to unknowr	---	AT3G11550	-1.15	-0.20	-1.41	0.202476	0.330472	-6.25
266241_s_a	hypothetical protein predicted by ge	---	AT2G27800	1.13	0.18	1.41	0.202485	0.330472	-6.25
249411_at	raffinose synthase -like protein raff	SIP1	AT5G40390	-1.34	-0.43	-1.41	0.20251	0.330489	-6.25
267287_at	putative pectinesterase	sks16	AT2G23630	-1.10	-0.14	-1.41	0.202539	0.3305	-6.25
245529_at	SEN1 like protein	MAA3	AT4G15570	1.22	0.29	1.41	0.202545	0.3305	-6.25
250061_at	chloroplast GrpE protein ; supporte	EMB1241	AT5G17710	1.15	0.20	1.41	0.202693	0.3307	-6.25
252788_at	putative protein several hypothetica	---	---	-1.11	-0.15	-1.41	0.202699	0.3307	-6.25
246502_at	stearoyl-acyl carrier protein desatu	---	AT5G16240	-1.17	-0.23	-1.41	0.202729	0.3307	-6.25
259644_at	unknown protein	---	AT1G68910	1.15	0.20	1.41	0.202733	0.3307	-6.25
257148_at	cytochrome c, putative similar to cy	---	AT3G27240	1.20	0.27	1.41	0.20274	0.3307	-6.25
260026_at	60S ribosomal protein L18A, putati	RPL18AA	AT1G29970	1.12	0.16	1.41	0.202839	0.330837	-6.25
267322_at	putative leucine-rich-repeat protein	---	AT2G19330	-1.10	-0.14	-1.41	0.202859	0.330842	-6.25
244972_at	PSII 47KDa protein	---	---	-1.34	-0.42	-1.41	0.202871	0.330842	-6.25
267133_at	unknown protein	---	AT2G23440	-1.13	-0.17	-1.41	0.202993	0.331018	-6.25
266967_at	unknown protein	---	AT2G39530	-1.10	-0.14	-1.41	0.203088	0.331148	-6.25
255200_at	hypothetical protein	---	---	-1.13	-0.17	-1.41	0.203138	0.331206	-6.25
255114_at	putative protein various putative rev	---	---	-1.18	-0.23	-1.41	0.203165	0.331226	-6.25
265199_s_a	putative glucosyl transferase	---	AT2G36770	1.20	0.27	1.41	0.203281	0.331393	-6.26
256054_at	unknown protein	---	AT1G07120	-1.09	-0.12	-1.41	0.203345	0.331472	-6.26
262004_at	calcineurin B-like protein similar to (	CBL8	AT1G64480	-1.17	-0.23	-1.41	0.203417	0.331566	-6.26
257146_at	unknown protein contains Pfam pro	GTE8	AT3G27260	1.09	0.13	1.41	0.203481	0.331648	-6.26
257751_at	hypothetical protein predicted by ge	MKS1	AT3G18690	1.47	0.56	1.41	0.203536	0.331687	-6.26
264897_at	dynein light subunit lc6, flagellar ou	---	AT1G23220	1.14	0.18	1.41	0.203551	0.331687	-6.26
248837_at	putative protein similar to unknown	---	AT5G47260	1.09	0.12	1.41	0.203566	0.331687	-6.26

250340_at	putative protein predicted proteins	---	AT5G11840	-1.17	-0.22	-1.41	0.20357	0.331687	-6.26
246029_at	leucine-rich repeat protein ; suppor	---	AT5G21090	1.11	0.16	1.41	0.203578	0.331687	-6.26
252010_at	hypothetical protein ; supported by	---	AT3G52740	-1.16	-0.21	-1.41	0.203627	0.331742	-6.26
257163_at	DNA-binding protein, putative simil	MYB305	AT3G24310	-1.12	-0.16	-1.41	0.203655	0.331742	-6.26
247887_at	unknown protein	---	AT5G57880	1.11	0.15	1.41	0.203655	0.331742	-6.26
250728_at	putative protein similar to unknown	---	AT5G06440	-1.14	-0.19	-1.41	0.203711	0.33179	-6.26
261198_at	nitrate transporter, putative similar	ATNRT2.5	AT1G12940	-1.10	-0.14	-1.41	0.203714	0.33179	-6.26
250890_at	3-oxoacyl-[acyl-carrier-protein] syn	---	AT5G04520	1.11	0.15	1.41	0.203741	0.331806	-6.26
252634_x_a	putative protein predicted proteins,	---	---	-1.13	-0.18	-1.41	0.203753	0.331806	-6.26
248504_at	transcription factor Hap5a-like	NF-YC6	AT5G50480	-1.18	-0.24	-1.41	0.203859	0.331955	-6.26
260445_at	ARG1 protein (Altered Response to	ARG1	AT1G68370	-1.14	-0.19	-1.41	0.203958	0.332093	-6.26
266658_at	Expressed protein ; supported by fu	---	AT2G25735	1.78	0.83	1.41	0.204064	0.33224	-6.26
250313_at	Rab geranylgeranyltransferase, be	---	AT5G12210	1.15	0.20	1.41	0.204136	0.332336	-6.26
249421_at	DegP protease contains similarity to	DEG8	AT5G39830	-1.13	-0.18	-1.41	0.204167	0.332347	-6.26
264432_at	hypothetical protein similar to hypot	---	AT1G61710	-1.11	-0.15	-1.41	0.204173	0.332347	-6.26
263559_at	putative non-LTR retroelement reve	---	---	-1.11	-0.15	-1.41	0.20427	0.332481	-6.26
249873_at	ATP-dependent Clp protease prote	NCLPP7	AT5G23140	-1.13	-0.17	-1.41	0.20437	0.332621	-6.26
251347_at	putative protein ferritin 1 precursor	---	AT3G61010	-1.21	-0.28	-1.41	0.204409	0.332661	-6.26
262606_at	hypothetical protein predicted by ge	---	AT1G15190	-1.15	-0.20	-1.41	0.204457	0.332715	-6.26
245845_at	Pto kinase interactor, putative simil	PERK10	AT1G26150	-1.12	-0.16	-1.40	0.204479	0.332726	-6.26
260082_at	putative mitochondrial carrier protei	---	AT1G78180	-1.16	-0.21	-1.40	0.204577	0.332863	-6.26
259475_at	unknown protein ;supported by full-	---	AT1G19140	1.21	0.27	1.40	0.204697	0.333034	-6.26
255951_at	unknown protein contains similarity	MUB6	AT1G22050	1.15	0.20	1.40	0.204769	0.333117	-6.26
252495_at	putative protein hypothetical protein	---	AT3G46770	-1.13	-0.17	-1.40	0.204795	0.333117	-6.26
262140_at	hypothetical protein predicted by ge	---	AT1G52470	-1.10	-0.14	-1.40	0.204803	0.333117	-6.26
255720_at	phosphoribulokinase precursor iden	PRK	AT1G32060	1.12	0.17	1.40	0.204806	0.333117	-6.26
264989_at	unknown protein ; supported by cDI	---	AT1G27200	-1.17	-0.23	-1.40	0.2049	0.333245	-6.26
248079_at	unknown protein	---	AT5G55790	-1.17	-0.23	-1.40	0.205051	0.333467	-6.26
255439_at	putative phospho-ser/thr phosphata	BSL1	AT4G03080	1.11	0.15	1.40	0.20515	0.333605	-6.26
260409_at	Expressed protein ; supported by fu	SHW1	AT1G69935	-1.21	-0.28	-1.40	0.205325	0.333866	-6.26
247690_at	putative protein	MBD7	AT5G59800	-1.23	-0.30	-1.40	0.205381	0.333931	-6.26
246898_at	putative protein	---	AT5G25580	1.09	0.13	1.40	0.205395	0.333931	-6.26
249825_at	putative protein similar to unknown	---	AT5G23390	1.11	0.16	1.40	0.205436	0.333975	-6.26
264376_at	putative protein phosphatase 2C ;s	---	AT2G25070	1.17	0.22	1.40	0.205481	0.334024	-6.26
249302_at	putative protein similar to unknown	---	AT5G41450	-1.14	-0.19	-1.40	0.205512	0.33405	-6.26

246652_at	extensin -like protein extensin, soy	---	AT5G35190	-1.11	-0.15	-1.40	0.205568	0.334118	-6.27
265077_at	hypothetical protein similar to RING	---	AT1G55530	-1.18	-0.24	-1.40	0.205721	0.334343	-6.27
255209_at	see GB:AF077408	---	---	-1.13	-0.18	-1.40	0.20576	0.334382	-6.27
250624_at	putative protein	---	AT5G07330	-1.13	-0.17	-1.40	0.205774	0.334382	-6.27
253304_at	putative protein ;supported by full-l	---	AT4G33640	-1.13	-0.17	-1.40	0.205792	0.334387	-6.27
246981_at	unknown protein	---	AT5G04840	-1.16	-0.22	-1.40	0.205874	0.334495	-6.27
256364_at	wrky-type DNA binding protein, put	WRKY67	AT1G66550	-1.13	-0.18	-1.40	0.205923	0.334551	-6.27
255725_at	hypothetical protein similar to putati	PFT1	AT1G25540	1.09	0.13	1.40	0.206009	0.334657	-6.27
244959_s_a	hypothetical protein	---	AT2G07708	1.16	0.21	1.40	0.206017	0.334657	-6.27
245273_at	hypothetical protein ; supported by	---	AT4G14950	1.14	0.19	1.40	0.206085	0.334743	-6.27
258509_at	protein kinase, putative similar to S	---	AT3G06620	1.20	0.27	1.40	0.20611	0.334759	-6.27
266251_s_a	unknown protein	---	AT2G18320	-1.10	-0.13	-1.40	0.206203	0.334886	-6.27
263309_at	putative replication protein A1	---	---	-1.18	-0.23	-1.40	0.206409	0.335198	-6.27
261400_at	protein phosphatase-2C, putative s	---	AT1G79630	1.18	0.24	1.40	0.206457	0.335252	-6.27
260866_at	polyprotein, putative similar to poly	---	---	-1.13	-0.17	-1.40	0.206477	0.33526	-6.27
249701_at	putative protein similar to unknown	---	AT5G35460	1.11	0.15	1.40	0.20658	0.335405	-6.27
260469_at	BRCA1-associated RING domain p	AGD4	AT1G10870	-1.15	-0.20	-1.40	0.206701	0.335577	-6.27
249296_at	kinesin-like protein	---	AT5G41310	-1.13	-0.18	-1.40	0.206745	0.335624	-6.27
266141_at	protease inhibitor II contains a gam	PDF2.1	AT2G02120	-1.10	-0.14	-1.40	0.206942	0.335905	-6.27
264937_at	hypothetical protein contains simila	SCPL32	AT1G61130	-1.12	-0.16	-1.40	0.206948	0.335905	-6.27
247501_at	putative protein predicted proteins,	---	AT5G61920	1.09	0.13	1.40	0.207097	0.336124	-6.27
248934_at	serine/threonine protein kinase-like	---	AT5G46080	1.56	0.64	1.40	0.207203	0.336272	-6.27
252201_at	putative protein anthranilate N-hyd	---	AT3G50295	-1.10	-0.14	-1.40	0.207287	0.336384	-6.27
245107_at	putative heat shock transcription fa	AT-HSFB3	AT2G41690	1.35	0.43	1.40	0.207315	0.336406	-6.27
266725_at	SKP1/ASK1 (At14), putative simila	ASK14	AT2G03170	-1.11	-0.15	-1.39	0.207361	0.336457	-6.27
259498_at	unknown protein similar to putative	GOS11	AT1G15880	-1.11	-0.15	-1.39	0.207484	0.336632	-6.27
265069_at	awaiting functional assignment simi	---	AT1G55545	-1.12	-0.16	-1.39	0.207561	0.336732	-6.27
245591_at	hypothetical protein	---	AT4G14530	-1.09	-0.13	-1.39	0.207593	0.33676	-6.27
254995_at	putative protein	---	AT4G10370	-1.14	-0.19	-1.39	0.207673	0.336867	-6.27
265668_at	putative alanine acetyl transferase	---	AT2G32020	1.49	0.58	1.39	0.207691	0.336872	-6.27
248073_at	pectate lyase	---	AT5G55720	-1.12	-0.16	-1.39	0.207726	0.336905	-6.27
267198_at	putative gibberellin-regulated protei	---	AT2G30810	1.10	0.13	1.39	0.207785	0.336976	-6.27
263813_s_a	putative TNP1-like transposon prote	---	---	-1.11	-0.16	-1.39	0.207903	0.337143	-6.28
265772_at	putative protein kinase contains a p	RKF3	AT2G48010	1.16	0.21	1.39	0.207917	0.337143	-6.28
245278_at	syntaxin ; supported by cDNA: gi_2	SYP23	AT4G17730	1.18	0.23	1.39	0.20797	0.337204	-6.28

251872_at	putative protein predicted protein A	---	AT3G54570	-1.10	-0.14	-1.39	0.208039	0.337293	-6.28
257675_at	ethylene responsive element bindir	ERF7	AT3G20310	-1.15	-0.20	-1.39	0.208088	0.337349	-6.28
245691_at	zinc finger protein - like	ELF6	AT5G04240	1.18	0.24	1.39	0.20811	0.33736	-6.28
252154_at	putative DNA-3-methyladenine gly	---	AT3G50880	1.17	0.22	1.39	0.208245	0.337555	-6.28
264749_at	hypothetical protein Contains simila	---	AT1G22830	1.21	0.27	1.39	0.208352	0.337704	-6.28
259594_at	unknown protein contains similarity	---	AT1G28140	1.11	0.15	1.39	0.208434	0.337812	-6.28
255828_at	hypothetical protein predicted by ge	---	AT2G40630	1.10	0.14	1.39	0.208466	0.33784	-6.28
247502_at	putative protein predicted proteins,	---	AT5G61940	1.11	0.15	1.39	0.208626	0.33804	-6.28
254209_at	Expressed protein ; supported by c	---	AT4G23490	-1.20	-0.26	-1.39	0.208628	0.33804	-6.28
256372_at	cell division protein kinase, putative	CAK4	AT1G66750	1.16	0.22	1.39	0.208634	0.33804	-6.28
251210_at	hypothetical protein ;supported by	---	AT3G62810	1.14	0.19	1.39	0.208667	0.33807	-6.28
261040_at	oligouridylate binding protein, puta	UBP1B	AT1G17370	-1.11	-0.16	-1.39	0.208804	0.338226	-6.28
247951_at	oxysterol-binding protein-like	ORP4C	AT5G57240	-1.11	-0.15	-1.39	0.208807	0.338226	-6.28
249535_at	transporter -like protein N system a	---	AT5G38820	-1.15	-0.20	-1.39	0.208808	0.338226	-6.28
260433_at	MtN21-like protein similar to MtN21	---	AT1G68170	-1.13	-0.17	-1.39	0.209025	0.338554	-6.28
261367_at	protein kinase, putative similar to m	---	AT1G53080	-1.81	-0.86	-1.39	0.209088	0.338633	-6.28
259144_at	putative kinesin-like centromere pro	---	AT3G10180	-1.11	-0.15	-1.39	0.20912	0.338655	-6.28
250273_at	pre-mRNA splicing factor ATP-dep	EMB3011	AT5G13010	1.10	0.14	1.39	0.209137	0.338655	-6.28
253711_at	hypothetical protein	DER1	---	-1.11	-0.15	-1.39	0.209152	0.338655	-6.28
255824_at	hypothetical protein predicted by ge	---	AT2G40530	-1.39	-0.47	-1.39	0.209172	0.338655	-6.28
256213_at	protein kinase, putative contains P	---	AT1G50990	-1.13	-0.17	-1.39	0.209179	0.338655	-6.28
252832_at	ubiquitin-specific protease 3 (UBP3	ATUBP3	AT4G39910	1.09	0.12	1.39	0.209196	0.338655	-6.28
255464_s_a	putative protein similar to T4I9.17	AT7SL-1	AT4G02970	-1.10	-0.14	-1.39	0.209206	0.338655	-6.28
250934_at	DNAJ protein - like DNAJ domain-c	---	AT5G03030	-1.20	-0.26	-1.39	0.209366	0.33889	-6.28
251559_at	putative protein hypothetical protei	---	AT3G58010	-1.16	-0.21	-1.39	0.209469	0.339032	-6.28
262336_at	hypothetical protein predicted be ge	TOM7-2	AT1G64220	1.10	0.13	1.39	0.209535	0.339106	-6.28
261660_at	kinesin heavy chain isolog, putative	HIK	AT1G18370	-1.10	-0.14	-1.39	0.209546	0.339106	-6.28
259238_at	putative eukaryotic translation initia	EIF3G1	AT3G11400	1.12	0.16	1.39	0.209561	0.339106	-6.28
255532_at	hypothetical protein similar to orf a	---	AT4G02170	-1.16	-0.22	-1.39	0.209574	0.339106	-6.28
246441_at	putative protein ;supported by full-l	---	AT5G17560	-1.11	-0.15	-1.39	0.209602	0.339124	-6.28
265404_at	putative beta-galactosidase	BGAL13	AT2G16730	-1.16	-0.22	-1.39	0.209615	0.339124	-6.28
254030_at	putative acidic ribosomal protein a	---	AT4G25890	1.32	0.40	1.39	0.209681	0.339208	-6.28
252771_at	receptor protein kinase -like protei	---	AT3G42880	-1.12	-0.16	-1.39	0.209708	0.339227	-6.28
249136_at	putative protein similar to unknown	---	AT5G43180	1.22	0.29	1.39	0.209795	0.339344	-6.28
246993_at	Cys2/His2-type zinc finger protein 1	AZF1	AT5G67450	1.45	0.54	1.39	0.209863	0.339429	-6.28

245663_at	steroid sulfotransferase, putative si	---	AT1G28170	-1.11	-0.15	-1.39	0.210044	0.339699	-6.28
246542_at	transcriptional regulatory - like prot	SNL2	AT5G15020	1.15	0.20	1.39	0.210082	0.339736	-6.28
265286_at	putative NADH-ubiquinone oxireduc	---	AT2G20360	-1.12	-0.16	-1.39	0.210118	0.339747	-6.28
265902_at	hypothetical protein predicted by ge	---	AT2G25590	-1.12	-0.16	-1.39	0.210119	0.339747	-6.28
262568_at	hypothetical protein contains similar	AtRLP5	AT1G34290	-1.13	-0.18	-1.39	0.210149	0.339756	-6.28
247857_at	peroxidase	---	AT5G58400	-1.13	-0.17	-1.39	0.21016	0.339756	-6.28
258349_at	Expressed protein ; supported by fu	HYH	AT3G17609	1.31	0.38	1.39	0.210169	0.339756	-6.28
255448_at	hypothetical protein	---	AT4G02810	-1.15	-0.21	-1.39	0.210189	0.339765	-6.28
258105_at	Expressed protein ; supported by fu	---	AT3G23605	1.24	0.30	1.38	0.210243	0.339814	-6.29
257546_s_a	hypothetical protein predicted by ge	---	AT3G28410	1.09	0.13	1.38	0.21025	0.339814	-6.29
256639_at	hypothetical protein predicted by ge	---	---	-1.14	-0.19	-1.38	0.210399	0.340031	-6.29
259032_at	hypothetical protein similar to hypo	---	AT3G09380	-1.10	-0.13	-1.38	0.210448	0.340085	-6.29
259045_at	calmodulin-like protein similar to ca	---	AT3G03410	1.11	0.15	1.38	0.210462	0.340085	-6.29
251923_at	reductase-like protein abscisic acid	---	AT3G53880	-1.27	-0.35	-1.38	0.210493	0.34011	-6.29
246634_at	epsilon-adaptin, putative similar to	---	AT1G31730	-1.11	-0.15	-1.38	0.210582	0.340231	-6.29
257960_at	S-adenosylmethionine decarboxyla	---	AT3G25570	-1.10	-0.13	-1.38	0.210672	0.340352	-6.29
256320_at	DnaJ protein, putative contains Pfa	---	AT3G12170	-1.14	-0.18	-1.38	0.210812	0.340553	-6.29
266452_at	hypothetical protein predicted by ge	---	AT2G43320	-1.19	-0.25	-1.38	0.210886	0.340649	-6.29
257612_at	unknown protein	ARO4	AT3G26600	1.31	0.39	1.38	0.210917	0.340676	-6.29
254800_at	putative protein several hypothetica	---	AT4G13070	1.19	0.25	1.38	0.211046	0.340859	-6.29
256868_at	eukaryotic translation initiation fact	EIF4B1	AT3G26400	1.21	0.28	1.38	0.211092	0.34091	-6.29
257392_at	hypothetical protein	FLA3	AT2G24450	-1.12	-0.16	-1.38	0.211169	0.341011	-6.29
256732_at	hypothetical protein predicted by ge	---	---	-1.10	-0.14	-1.38	0.211267	0.34114	-6.29
267222_at	putative polygalacturonase	---	AT2G43880	-1.10	-0.14	-1.38	0.21128	0.34114	-6.29
254266_at	protein kinase - like protein serine t	CRK5	AT4G23130	1.42	0.50	1.38	0.211315	0.341173	-6.29
259856_at	unknown protein ;supported by full-	---	AT1G68440	-1.31	-0.39	-1.38	0.211336	0.341183	-6.29
251264_at	multifunctional aminoacyl-tRNA liga	---	AT3G62120	-1.10	-0.14	-1.38	0.211352	0.341185	-6.29
245355_at	60S ribosomal protein L15 homolog	---	AT4G17390	1.28	0.35	1.38	0.211387	0.341218	-6.29
251335_at	1-aminocyclopropane-1-carboxylat	---	AT3G61400	-1.18	-0.24	-1.38	0.211535	0.341432	-6.29
265194_at	1-aminocyclopropane-1-carboxylat	EFE	AT1G05010	1.30	0.38	1.38	0.211577	0.341475	-6.29
266350_at	putative protein transport protein S	STL2P	AT2G01470	1.17	0.22	1.38	0.211688	0.34163	-6.29
246668_at	putative protein	---	---	-1.11	-0.15	-1.38	0.211713	0.341647	-6.29
264742_at	hypothetical protein similar to hypot	---	AT1G62130	-1.14	-0.20	-1.38	0.211858	0.341844	-6.29
248859_at	putative protein contains similarity t	---	AT5G46660	-1.13	-0.18	-1.38	0.211865	0.341844	-6.29
246141_at	TipD - like WD repeat protein TipD	---	AT5G19920	-1.14	-0.19	-1.38	0.211936	0.341934	-6.29

259537_at	hypothetical protein ; supported by	PHR1	AT1G12370	-1.14	-0.19	-1.38	0.21208	0.342142	-6.29
258428_at	unknown protein	---	AT3G16610	1.12	0.16	1.38	0.212194	0.342302	-6.29
251234_s_a	putative dTDP-glucose 4-6-dehydra	UXS4	AT2G47650	1.18	0.23	1.38	0.212226	0.342328	-6.29
252119_at	thioredoxin h ;supported by full-len	ATTRX1	AT3G51030	-1.22	-0.29	-1.38	0.212429	0.342632	-6.29
249747_at	putative protein similar to unknown	---	AT5G24600	-1.14	-0.18	-1.38	0.212467	0.342669	-6.29
245788_at	hypothetical protein similar to hypot	---	AT1G32120	1.30	0.37	1.38	0.212568	0.342808	-6.29
263857_at	unknown protein	---	AT2G04380	-1.09	-0.12	-1.38	0.212597	0.34283	-6.30
250228_at	cell cycle switch protein	FZR3	AT5G13840	-1.14	-0.18	-1.38	0.212616	0.342837	-6.30
259503_at	unknown protein ; supported by cDI	---	AT1G15870	1.17	0.23	1.38	0.212643	0.342856	-6.30
249388_at	putative protein downy mildew resi	---	AT5G40090	-1.13	-0.17	-1.38	0.212716	0.34295	-6.30
260255_at	putative protein kinase similar to p5	---	AT1G74330	-1.22	-0.29	-1.38	0.212733	0.342953	-6.30
246577_at	MADS box transcription factor, puta	AGL92	AT1G31640	-1.11	-0.15	-1.38	0.212763	0.342958	-6.30
256328_at	6-phosphogluconate dehydrogenas	---	AT3G02360	1.23	0.30	1.38	0.212766	0.342958	-6.30
255917_at	putative protein	---	AT5G28560	-1.09	-0.13	-1.38	0.212922	0.343185	-6.30
250660_at	RNA-binding protein-like	---	AT5G07060	1.09	0.13	1.38	0.212964	0.343212	-6.30
262111_at	hypothetical protein predicted by ge	---	AT1G02960	-1.11	-0.15	-1.38	0.212969	0.343212	-6.30
259850_at	hypothetical protein predicted by ge	---	AT1G72240	-1.17	-0.22	-1.38	0.213058	0.343313	-6.30
249565_at	contains similarity to S1 self-incomp	---	AT5G38440	-1.12	-0.16	-1.38	0.213062	0.343313	-6.30
253101_at	cytochrome P450 monooxygenase	CYP91A2	AT4G37430	-1.24	-0.31	-1.38	0.213138	0.343388	-6.30
257476_at	unknown protein	---	AT1G80960	1.09	0.13	1.38	0.213139	0.343388	-6.30
257250_at	transfactor, putative similar to GB:E	---	AT3G24120	-1.12	-0.17	-1.37	0.213216	0.343474	-6.30
266907_at	Expressed protein ; supported by fu	ATGA2OX3	---	-1.14	-0.19	-1.37	0.213222	0.343474	-6.30
254838_at	hypothetical protein	---	AT4G12380	-1.16	-0.21	-1.37	0.213248	0.343491	-6.30
264787_at	putative senescence-associated pro	ERD7	AT2G17840	-1.22	-0.28	-1.37	0.213307	0.34354	-6.30
259382_s_a	jasmonate inducible protein isolog	JAL31	AT3G16420	-1.72	-0.78	-1.37	0.213323	0.34354	-6.30
245522_at	hypothetical protein	---	AT4G15890	-1.11	-0.15	-1.37	0.213323	0.34354	-6.30
257353_at	hypothetical protein predicted by ge	NPY4	AT2G23050	-1.12	-0.16	-1.37	0.213372	0.343595	-6.30
258177_at	ubiquitin-regulatory protein, putativ	---	AT3G21660	-1.11	-0.14	-1.37	0.2134	0.343613	-6.30
256952_at	mannosidase, putative similar to m	---	AT3G21160	1.14	0.19	1.37	0.213426	0.343613	-6.30
255548_at	putative CHP-rich zinc finger protei	---	AT4G01930	-1.09	-0.13	-1.37	0.213429	0.343613	-6.30
259011_s_a	unknown protein similar to putative	---	AT3G07380	-1.12	-0.16	-1.37	0.213445	0.343616	-6.30
266623_at	unknown protein	---	AT2G35390	-1.16	-0.21	-1.37	0.213491	0.34364	-6.30
254451_at	adrenodoxin - like protein adrenodo	---	AT4G21090	1.09	0.13	1.37	0.213501	0.34364	-6.30
249962_at	pectin methyl esterase -like protein	---	AT5G18990	-1.19	-0.25	-1.37	0.213506	0.34364	-6.30
251616_at	putative protein	---	AT3G57990	1.10	0.14	1.37	0.213542	0.343675	-6.30

252679_at	CCR4-associated factor 1-like prot	---	AT3G44260	1.76	0.82	1.37	0.21357	0.343695	-6.30
254938_at	putative oligopeptide transporter si	OPT7	AT4G10770	-1.11	-0.15	-1.37	0.213685	0.343856	-6.30
249363_at	DegP protease contains similarity to	DegP13	AT5G40560	-1.09	-0.13	-1.37	0.213778	0.343941	-6.30
264697_at	cyclin, putative similar to cyclin D G	CYCD1;1	AT1G70210	-1.63	-0.70	-1.37	0.21378	0.343941	-6.30
249516_s_a	myrosinase binding protein-like; sim	---	AT5G38540	-1.12	-0.17	-1.37	0.213818	0.343941	-6.30
253860_at	hypothetical protein ;supported by	---	AT4G27700	-1.11	-0.14	-1.37	0.213818	0.343941	-6.30
257641_s_a	hypothetical protein	AOC1	AT3G25760	-1.24	-0.31	-1.37	0.213824	0.343941	-6.30
262934_s_a	hypothetical protein contains simila	---	AT1G16270	1.16	0.21	1.37	0.213828	0.343941	-6.30
261015_at	14-3-3 protein GF14iota (grf12) ide	GRF12	AT1G26480	1.11	0.15	1.37	0.213909	0.344046	-6.30
255848_at	hypothetical protein predicted by ge	---	AT2G33640	-1.13	-0.17	-1.37	0.213965	0.344113	-6.30
245094_at	4-alpha-glucanotransferase	DPE2	AT2G40840	-1.15	-0.20	-1.37	0.214025	0.344185	-6.30
256329_at	unknown protein	SEC5A	AT1G76850	-1.09	-0.12	-1.37	0.214047	0.344195	-6.30
253446_at	putative protein predicted protein T	---	AT4G32620	1.12	0.16	1.37	0.21409	0.344241	-6.30
248654_at	importin alpha	IMPA-5	AT5G49310	-1.11	-0.15	-1.37	0.214174	0.344351	-6.30
255771_at	hypothetical protein contains simila	---	AT1G18550	-1.12	-0.16	-1.37	0.214202	0.344372	-6.30
266817_at	hypothetical protein similar to hypot	HUB1	AT2G44950	1.16	0.21	1.37	0.214252	0.344429	-6.30
252499_s_a	subtilisin-like serine protease conta	---	AT3G46840	1.12	0.16	1.37	0.214483	0.344747	-6.30
249240_at	putative protein similar to unknown	---	AT5G42220	1.14	0.19	1.37	0.214486	0.344747	-6.30
250308_at	calcium-dependent protein kinase	CPK17	AT5G12180	-1.15	-0.20	-1.37	0.214496	0.344747	-6.30
258777_at	hypothetical protein predicted by ge	---	AT3G11850	1.15	0.21	1.37	0.21453	0.34477	-6.30
252073_at	hypothetical protein	---	AT3G51750	1.53	0.62	1.37	0.214541	0.34477	-6.30
260876_at	hypothetical protein contains simila	---	AT1G21460	-1.18	-0.24	-1.37	0.214818	0.345191	-6.30
265237_s_a	hypothetical protein	---	AT2G07706	-1.12	-0.17	-1.37	0.214856	0.345228	-6.30
252175_at	zinc finger protein zinc finger prote	AtIDD2	AT3G50700	1.09	0.13	1.37	0.214984	0.34541	-6.30
266505_at	unknown protein	---	AT2G47830	-1.11	-0.15	-1.37	0.215053	0.345497	-6.31
262877_at	unknown protein ;supported by full-	---	AT1G64850	1.15	0.20	1.37	0.215079	0.345514	-6.31
267629_at	putative DNA polymerase delta sma	POLD2	AT2G42120	1.16	0.22	1.37	0.21538	0.345974	-6.31
257360_at	unknown protein	---	AT2G39240	-1.10	-0.14	-1.37	0.215431	0.346031	-6.31
253053_at	putative protein beta-ketoadipate e	---	AT4G37470	-1.15	-0.20	-1.37	0.215447	0.346033	-6.31
251127_at	putative protein predicted protein, A	---	AT5G01080	1.13	0.18	1.37	0.215474	0.346051	-6.31
267328_at	putative receptor-like protein kinase	---	AT2G19230	-1.14	-0.18	-1.37	0.215507	0.346079	-6.31
252623_at	hypothetical protein	---	AT3G45320	-1.12	-0.16	-1.37	0.215583	0.346177	-6.31
265501_at	putative glucosyltransferase	UGT73B4	AT2G15490	1.37	0.45	1.37	0.215797	0.346496	-6.31
265458_at	unknown protein ;supported by full-	---	AT2G46580	-1.13	-0.18	-1.37	0.21592	0.346662	-6.31
266639_at	defender against cell death protein	DAD2	AT2G35520	1.11	0.15	1.37	0.215931	0.346662	-6.31

262171_at	unknown protein ;supported by full-	TIFY10B	AT1G74950	-1.19	-0.25	-1.37	0.215971	0.346703	-6.31
251237_at	bZIP transcription factor-like protei	CPuORF3	AT3G62420	-1.12	-0.16	-1.37	0.216028	0.34677	-6.31
256841_x_a	hypothetical protein predicted by ge	---	---	-1.18	-0.24	-1.36	0.216219	0.347053	-6.31
246742_at	TOPP8 serine/threonine protein pho	TOPP8	AT5G27840	1.09	0.12	1.36	0.216278	0.347122	-6.31
262814_at	putative heat-shock protein strong s	---	AT1G11660	1.10	0.14	1.36	0.216441	0.347358	-6.31
249218_at	pitrilysin	---	AT5G42390	1.16	0.22	1.36	0.216465	0.347358	-6.31
250636_at	glycine-rich protein atGRP	GRP18	AT5G07520	-1.12	-0.16	-1.36	0.21647	0.347358	-6.31
251911_at	putative C2H2-type zinc finger prote	---	AT3G53820	-1.12	-0.16	-1.36	0.216513	0.347381	-6.31
264413_s_a	hypothetical protein similar to hypot	---	---	-1.15	-0.20	-1.36	0.216515	0.347381	-6.31
265832_at	putative replication protein A1	---	---	-1.10	-0.14	-1.36	0.216646	0.347566	-6.31
260991_at	hypothetical protein predicted by ge	---	AT1G12160	1.10	0.13	1.36	0.216954	0.348019	-6.31
247192_at	histone H3 (sp|P05203) ; supporte	---	AT5G65360	-1.84	-0.88	-1.36	0.216959	0.348019	-6.31
266408_at	putative retroelement pol polyprotei	---	---	-1.14	-0.19	-1.36	0.21701	0.348049	-6.31
265784_at	hypothetical protein predicted by ge	---	AT2G07280	1.11	0.15	1.36	0.217022	0.348049	-6.31
260428_at	putative translation initiation factor E	---	AT1G72340	-1.16	-0.22	-1.36	0.217023	0.348049	-6.31
263581_at	hypothetical protein identical to hyp	---	AT2G17160	-1.14	-0.18	-1.36	0.217182	0.348279	-6.31
247018_at	AP2 domain transcription factor-like	---	AT5G67000	-1.09	-0.12	-1.36	0.217239	0.348347	-6.31
257672_at	unknown protein ;supported by full-	---	AT3G20300	-1.22	-0.28	-1.36	0.217294	0.34841	-6.31
250151_at	high affinity nitrate transporter - like	ATNRT2.7	AT5G14570	-1.23	-0.29	-1.36	0.21735	0.348475	-6.31
250708_at	Expressed protein ; supported by fu	RBE	AT5G06070	-1.11	-0.15	-1.36	0.217445	0.348555	-6.31
247270_at	ER66 protein-like	---	AT5G64220	-1.10	-0.14	-1.36	0.217452	0.348555	-6.31
254050_s_a	hypothetical protein ; supported by	---	AT4G25670	1.11	0.15	1.36	0.217459	0.348555	-6.31
250374_at	putative protein	EMF1	AT5G11530	1.22	0.29	1.36	0.217474	0.348555	-6.32
252478_at	putative protein mitotic phosphopro	---	AT3G46540	1.20	0.26	1.36	0.217476	0.348555	-6.32
263612_at	putative CDC21 protein	MCM4	AT2G16440	-1.43	-0.52	-1.36	0.217507	0.348576	-6.32
265901_at	putative selenium-binding protein	---	AT2G25580	1.14	0.18	1.36	0.21752	0.348576	-6.32
248935_at	salt-inducible protein-like	---	AT5G46100	1.13	0.18	1.36	0.217653	0.348765	-6.32
265524_at	hypothetical protein	---	---	-1.09	-0.13	-1.36	0.217678	0.34878	-6.32
251137_at	putative protein several hypothetica	---	AT5G01300	1.17	0.22	1.36	0.217749	0.348869	-6.32
257245_at	hypothetical protein contains Pfam	---	AT3G24110	-1.14	-0.19	-1.36	0.217852	0.349011	-6.32
265738_at	NADC homolog identical to GP|121	QPT	AT2G01350	1.12	0.17	1.36	0.217878	0.349026	-6.32
267107_at	hypothetical protein predicted by ge	---	---	-1.12	-0.16	-1.36	0.217893	0.349026	-6.32
264275_at	hypothetical protein predicted by ge	anac024	AT1G60350	-1.09	-0.12	-1.36	0.217953	0.349075	-6.32
254791_at	SERINE CARBOXYPEPTIDASE I	scpl20	AT4G12910	1.14	0.18	1.36	0.217954	0.349075	-6.32
248692_s_a	hypothetical protein	---	AT4G15070	1.12	0.16	1.36	0.218084	0.349239	-6.32

257525_at	hypothetical protein predicted by ge	---	AT3G04900	-1.13	-0.18	-1.36	0.218093	0.349239	-6.32
248634_at	isoleucyl-tRNA synthetase	OVA2	AT5G49030	-1.15	-0.20	-1.36	0.218102	0.349239	-6.32
254720_at	DNA (cytosine-5-)-methyltransferas	MEE57	AT4G13610	-1.12	-0.17	-1.36	0.218131	0.349261	-6.32
254127_at	NADPH-ferrihemoprotein reductase	ATR1	AT4G24520	1.14	0.19	1.36	0.218162	0.349285	-6.32
248578_at	putative protein contains similarity t	emb1879	AT5G49820	-1.16	-0.21	-1.36	0.218211	0.349336	-6.32
264401_at	dihydroflavonol 4-reductase, putati	BAN	AT1G61720	-1.09	-0.12	-1.36	0.218224	0.349336	-6.32
265741_at	putative membrane transporter ; su	---	AT2G01320	1.23	0.30	1.36	0.218377	0.349539	-6.32
260230_at	putative DNA-binding protein contai	---	AT1G74500	-1.11	-0.15	-1.36	0.218381	0.349539	-6.32
262294_at	hypothetical protein identical to hyp	---	AT1G27610	-1.11	-0.15	-1.36	0.21841	0.34954	-6.32
256743_at	Expressed protein ; supported by fu	---	AT3G29370	-1.36	-0.44	-1.36	0.218413	0.34954	-6.32
249543_at	Expressed protein ; supported by fu	---	---	-1.13	-0.17	-1.36	0.218526	0.349698	-6.32
264975_at	hypothetical protein predicted by ge	---	AT1G27070	1.12	0.16	1.36	0.218594	0.349776	-6.32
249929_at	unknown protein	---	AT5G22340	-1.14	-0.19	-1.36	0.218606	0.349776	-6.32
267571_at	3-hydroxyisobutyryl-coenzyme A hy	---	AT2G30650	-1.10	-0.14	-1.36	0.218652	0.349825	-6.32
254029_at	putative protein other hypothetical p	---	AT4G25870	1.13	0.18	1.36	0.218707	0.349888	-6.32
246296_at	putative protein hypothetical protei	---	AT3G56750	1.10	0.14	1.36	0.218723	0.349889	-6.32
262300_at	ripening-induced protein, putative s	---	AT1G70870	1.09	0.12	1.36	0.218754	0.349914	-6.32
251871_at	hypothetical protein	---	AT3G54520	-1.09	-0.12	-1.36	0.218778	0.349928	-6.32
256882_at	RNA-binding protein, putative simil	---	AT3G26420	1.15	0.20	1.36	0.218873	0.350055	-6.32
247438_at	unknown protein ; supported by cD	---	AT5G62460	-1.21	-0.28	-1.36	0.219019	0.350264	-6.32
267349_at	60S acidic ribosomal protein P0	---	AT2G40010	-1.10	-0.13	-1.36	0.219067	0.350316	-6.32
265615_at	putative dioxygenase ; supported b	---	AT2G25450	-1.13	-0.18	-1.35	0.219186	0.350482	-6.32
250909_at	S-receptor kinase - like protein S-r	---	AT5G03700	1.47	0.56	1.35	0.219299	0.350639	-6.32
248117_at	FH protein interacting protein FIP2	FIP2	AT5G55000	1.14	0.19	1.35	0.219353	0.350696	-6.32
259521_at	ATP-dependent Clp protease prote	CLP2	AT1G12410	-1.14	-0.19	-1.35	0.219377	0.350696	-6.32
254478_at	putative protein TRANSCRIPTION	---	AT4G20330	1.15	0.20	1.35	0.219381	0.350696	-6.32
251491_at	fructokinase-like protein fructokinas	---	AT3G59480	-1.17	-0.23	-1.35	0.219453	0.350787	-6.32
259927_at	unknown protein ; supported by cD	JAC1	AT1G75100	-1.19	-0.26	-1.35	0.219479	0.350804	-6.32
255991_at	unknown protein	---	AT1G29820	-1.20	-0.27	-1.35	0.219521	0.350846	-6.32
244947_at	hypothetical protein	---	---	1.14	0.19	1.35	0.219636	0.351005	-6.32
259686_at	transcription factor SCARECROW,	---	AT1G63100	-1.18	-0.24	-1.35	0.219654	0.35101	-6.32
261994_at	hypothetical protein contains similar	---	AT1G33640	-1.10	-0.14	-1.35	0.219714	0.35108	-6.32
261299_at	unknown protein	---	AT1G48550	1.15	0.20	1.35	0.2198	0.351194	-6.32
264391_at	pectate lyase-like protein Similar to	---	AT1G11920	-1.11	-0.16	-1.35	0.219888	0.35131	-6.32
251566_at	putative protein several hypothetica	---	AT3G58210	-1.09	-0.12	-1.35	0.22002	0.351496	-6.33

251216_at	putative protein hypothetical protei	---	---	-1.10	-0.14	-1.35	0.22012	0.35163	-6.33
262511_at	syntaxin-related protein At-SYR1, p	SYP125	AT1G11250	-1.15	-0.20	-1.35	0.220206	0.351731	-6.33
247753_at	hexosyltransferase - like protein	---	AT5G59070	1.32	0.41	1.35	0.220226	0.351731	-6.33
246046_at	putative protein predicted proteins,	---	---	-1.12	-0.17	-1.35	0.220229	0.351731	-6.33
254390_at	calcium-dependent protein kinase -	CPK15	AT4G21940	1.12	0.16	1.35	0.220248	0.351738	-6.33
256971_at	unknown protein contains Pfam pro	---	AT3G21100	1.16	0.22	1.35	0.220266	0.351742	-6.33
245945_at	putative protein various predicted p	ROPGEF1(	AT5G19560	-1.09	-0.13	-1.35	0.220413	0.351951	-6.33
265058_s_a	myrosinase-binding protein homolo	MBP2	AT1G52030 ,	-1.82	-0.86	-1.35	0.22045	0.351986	-6.33
260098_at	tumor-related protein, putative simi	---	AT1G73320	1.21	0.28	1.35	0.220492	0.352006	-6.33
265323_at	putative syntaxin	SYP112	AT2G18260	1.15	0.20	1.35	0.220494	0.352006	-6.33
257867_at	unknown protein	---	AT3G17780	-1.13	-0.18	-1.35	0.220527	0.352035	-6.33
267499_at	hypothetical protein predicted by ge	---	AT2G45500	1.12	0.16	1.35	0.220617	0.352135	-6.33
246303_at	putative carrier protein peroxisoma	---	AT3G51870	1.20	0.26	1.35	0.220621	0.352135	-6.33
250195_at	putative protein	---	AT5G14560	1.09	0.12	1.35	0.220657	0.352169	-6.33
262344_at	cytochrome b245 beta chain homo	ATRBOH F	AT1G64060	-1.36	-0.44	-1.35	0.220682	0.352183	-6.33
266689_at	hypothetical protein predicted by ge	---	AT2G19930	1.21	0.27	1.35	0.220705	0.352195	-6.33
264853_at	putative ligand-gated ion channel p	GLR2	AT2G17260 ,	-1.14	-0.18	-1.35	0.22074	0.352227	-6.33
260358_at	unknown protein ; supported by cDI	---	AT1G69340	1.10	0.14	1.35	0.220799	0.352296	-6.33
248078_at	putative protein contains similarity t	---	AT5G55780	-1.10	-0.14	-1.35	0.220832	0.352324	-6.33
262038_at	invertase, putative similar to neutra	CINV1	AT1G35580	-1.20	-0.26	-1.35	0.220905	0.352415	-6.33
247062_at	putative protein similar to unknown	---	AT5G66810	1.25	0.32	1.35	0.220925	0.352418	-6.33
246893_at	PHD finger protein - like PHD finge	---	AT5G25520	-1.09	-0.12	-1.35	0.220937	0.352418	-6.33
266362_at	unknown protein	---	AT2G32430	1.14	0.19	1.35	0.22103	0.352542	-6.33
263671_at	hypothetical protein predicted by ge	---	AT2G04830	-1.11	-0.15	-1.35	0.221213	0.352785	-6.33
266403_at	putative acid phosphatase contains	---	AT2G38600	-1.13	-0.17	-1.35	0.221214	0.352785	-6.33
246000_at	putative protein predicted proteins,	---	AT5G20820	-1.22	-0.28	-1.35	0.221351	0.35298	-6.33
247769_at	laccase precursor - like protein lac	LAC16	AT5G58910	-1.10	-0.14	-1.35	0.221385	0.353009	-6.33
264439_at	adenine phosphoribosyltransferase	APT1	AT1G27450	1.12	0.16	1.35	0.221486	0.353145	-6.33
251351_s_a	CaLB protein	NTMC2T4	AT3G60950 ,	-1.12	-0.16	-1.35	0.221861	0.353718	-6.33
256980_at	hypothetical protein contains Pfam	DRB3	AT3G26932	1.16	0.21	1.35	0.221923	0.353792	-6.33
245724_at	hypothetical protein predicted by ge	---	AT1G73390	1.20	0.26	1.35	0.222048	0.353967	-6.33
250176_at	putative protein cytochrome P450 (	CYP724A1	AT5G14400	-1.16	-0.21	-1.35	0.222094	0.35399	-6.33
251542_at	putative protein protein-tyrosine kir	---	AT3G58760	-1.14	-0.19	-1.35	0.222105	0.35399	-6.33
256609_at	hypothetical protein predicted by ge	---	---	-1.10	-0.13	-1.35	0.222112	0.35399	-6.33
261789_at	40S ribosomal protein S12, putativ	---	AT1G15930	1.26	0.34	1.35	0.22213	0.35399	-6.33

261347_at	unknown protein	ELF7	AT1G79730	1.13	0.17	1.35	0.22214	0.35399	-6.33
248399_at	putative protein strong similarity to u	---	AT5G52010	1.11	0.14	1.34	0.222254	0.354146	-6.33
254739_at	putative disease resistance protein	AtRLP48	AT4G13880	-1.11	-0.15	-1.34	0.222285	0.354172	-6.33
263582_at	unknown protein similar to hypothet	LYM2	AT2G17120	-1.19	-0.25	-1.34	0.222418	0.354356	-6.33
254337_at	putative protein hypothetical protei	---	AT4G22060	-1.12	-0.16	-1.34	0.222432	0.354356	-6.33
245159_at	putative cellulose synthase	ATCSLD1	AT2G33100	-1.13	-0.18	-1.34	0.222487	0.354419	-6.34
246996_at	putative protein similar to unknown	LBD37	AT5G67420	-1.20	-0.26	-1.34	0.222526	0.354457	-6.34
266032_x_a	putative replication protein A1	---	---	-1.12	-0.16	-1.34	0.222589	0.354532	-6.34
246766_at	hypothetical protein	---	AT5G27340	-1.18	-0.24	-1.34	0.222657	0.354598	-6.34
257763_s_a	disease resistance protein, putative	AtRLP37	AT3G23110	1.38	0.47	1.34	0.222661	0.354598	-6.34
262150_at	hypothetical protein contains similar	FRS6	AT1G52520	-1.13	-0.18	-1.34	0.222732	0.354686	-6.34
261963_at	hypothetical protein predicted by ge	---	---	1.13	0.18	1.34	0.222782	0.354715	-6.34
250780_at	expansin At-EXP2 (gb|AAB38073.1	ATEXPA2	AT5G05290	-1.16	-0.22	-1.34	0.222794	0.354715	-6.34
247675_at	putative protein large number of pr	---	AT5G59940	-1.15	-0.20	-1.34	0.222797	0.354715	-6.34
250447_at	putative protein KIAA0332 gene, H	---	AT5G10800	-1.10	-0.13	-1.34	0.222893	0.354844	-6.34
249166_at	CHP-rich zinc finger protein-like	---	AT5G42840	1.12	0.16	1.34	0.222949	0.354908	-6.34
255673_at	Similar to receptor-like protein kina	RLK4	AT4G00340	-1.11	-0.15	-1.34	0.223011	0.354962	-6.34
253509_at	putative protein kainate receptor, R	GLR2.4	AT4G31710	-1.12	-0.16	-1.34	0.223015	0.354962	-6.34
251016_at	pathogenesis related protein - like	---	AT5G02730	-1.20	-0.26	-1.34	0.223049	0.354962	-6.34
254304_at	hypothetical protein ; supported by	---	AT4G22270	-1.14	-0.19	-1.34	0.223064	0.354962	-6.34
252645_at	bZIP transcription factor -like prote	DPBF2	AT3G44460	-1.12	-0.16	-1.34	0.223071	0.354962	-6.34
246853_at	putative protein	---	AT5G26890	-1.10	-0.14	-1.34	0.223077	0.354962	-6.34
260228_at	putative cytochrome P450 similar to	CYP98A8	AT1G74540	-1.12	-0.16	-1.34	0.223115	0.354998	-6.34
259595_at	CONSTANS family zinc finger prote	---	AT1G28050	1.18	0.24	1.34	0.223264	0.355183	-6.34
263658_at	unknown protein EST gb|T21788 co	---	AT1G04490	-1.10	-0.14	-1.34	0.223282	0.355183	-6.34
252637_at	WD repeat domain protein nuclear	HIRA	AT3G44530	1.13	0.18	1.34	0.223287	0.355183	-6.34
252949_at	putative thaumatin-like protein thau	---	AT4G38670	-1.23	-0.29	-1.34	0.223294	0.355183	-6.34
251789_at	serine/threonine-specific protein kir	---	AT3G55450	1.15	0.20	1.34	0.22331	0.355185	-6.34
257535_at	hypothetical protein predicted by ge	---	AT3G09490	-1.25	-0.33	-1.34	0.223506	0.355472	-6.34
257760_s_a	putative AC-like transposase simila	---	---	-1.11	-0.15	-1.34	0.223632	0.355647	-6.34
256235_at	cysteine proteinase inhibitor, putati	ATCYSB	AT3G12490	-1.09	-0.12	-1.34	0.2237	0.35573	-6.34
246238_at	sugar transporter like protein	---	AT4G36670	-1.31	-0.39	-1.34	0.22373	0.355753	-6.34
244957_at	hypothetical protein	AT2G0783	---	-1.12	-0.16	-1.34	0.223975	0.356117	-6.34
255197_x_a	contains similarity to Oryza sativa re	---	---	-1.13	-0.17	-1.34	0.224036	0.356181	-6.34
253310_at	hypothetical protein ; supported by	EX1	AT4G33630	1.10	0.14	1.34	0.224046	0.356181	-6.34

256176_at	hypothetical protein predicted by ge	ATEXO70C	AT1G51640	-1.10	-0.14	-1.34	0.224095	0.356223	-6.34
264443_at	Expressed protein ; supported by fu	---	AT1G27385	-1.16	-0.21	-1.34	0.224104	0.356223	-6.34
259091_at	hypothetical protein predicted by ge	---	AT3G04890	-1.12	-0.17	-1.34	0.224122	0.356228	-6.34
253111_at	putative protein glutamic acid-rich p	---	AT4G35940	1.16	0.22	1.34	0.224154	0.356253	-6.34
262269_at	hypothetical protein predicted by ge	---	---	-1.10	-0.14	-1.34	0.22417	0.356254	-6.34
259779_s_a	hypothetical protein predicted by ge	---	AT1G29620	1.11	0.16	1.34	0.224198	0.356274	-6.34
249159_at	Nicotiana lesion-inducing like ; sup	---	AT5G43460	1.16	0.22	1.34	0.224287	0.356391	-6.34
252054_at	hypothetical protein ;supported by f	OFP18	AT3G52540	-1.13	-0.17	-1.34	0.224408	0.356558	-6.34
259853_at	leucine-rich receptor-like protein ki	---	AT1G72300	1.16	0.22	1.34	0.224442	0.356587	-6.34
262907_at	hypothetical protein predicted by ge	CRR28	AT1G59720	1.12	0.16	1.34	0.224574	0.356771	-6.34
250633_at	peptide methionine sulfoxide reduc	PMSR2	AT5G07460	-1.41	-0.49	-1.34	0.224739	0.356964	-6.34
258875_at	unknown protein ;supported by full-	---	AT3G03160	-1.16	-0.22	-1.34	0.224748	0.356964	-6.34
248356_at	putative protein strong similarity to u	ATEXO70A	AT5G52350	1.09	0.13	1.34	0.224761	0.356964	-6.34
249219_at	putative protein contains similarity t	SDG25	AT5G42400	-1.14	-0.19	-1.34	0.224769	0.356964	-6.34
249940_at	NAC-domain protein-like ;supporte	anac090	AT5G22380	-1.12	-0.16	-1.34	0.224773	0.356964	-6.34
251131_at	laccase - like protein laccase precu	LAC10	AT5G01190	-1.09	-0.12	-1.34	0.224838	0.357042	-6.34
248537_at	putative protein similar to unknown	---	AT5G50100	-1.15	-0.20	-1.34	0.224866	0.357062	-6.34
265186_at	Expressed protein ; supported by fu	---	AT1G23560	1.15	0.20	1.34	0.224987	0.357191	-6.34
245475_at	hypothetical protein	---	AT4G16080	-1.10	-0.14	-1.34	0.224988	0.357191	-6.34
263968_s_a	PttA-like transposon protein no AT(	---	---	-1.12	-0.16	-1.34	0.224994	0.357191	-6.34
250521_at	selenium-binding protein-like	---	AT5G08490	-1.11	-0.15	-1.34	0.22502	0.357197	-6.35
261897_at	hypothetical protein predicted by ge	---	AT1G80950	1.12	0.17	1.34	0.225046	0.357197	-6.35
267490_at	putative receptor-like protein kinase	---	AT2G19130	1.23	0.30	1.34	0.225058	0.357197	-6.35
244965_at	hypothetical protein	---	---	1.17	0.22	1.34	0.225061	0.357197	-6.35
257551_at	hypothetical protein contains similar	---	---	-1.12	-0.17	-1.34	0.225104	0.35724	-6.35
257785_at	geranylgeranylated protein, putativ	MUB4	AT3G26980	-1.45	-0.53	-1.34	0.225146	0.357266	-6.35
247174_at	unknown protein ; supported by cD	---	AT5G65250	1.10	0.14	1.34	0.225151	0.357266	-6.35
262383_at	disease resistance protein, putative	---	AT1G72940	1.68	0.75	1.34	0.22521	0.357334	-6.35
252488_at	glucuronosyl transferase-like prote	---	AT3G46700	-1.10	-0.14	-1.34	0.225252	0.357376	-6.35
256535_at	unknown protein	ANAC015	AT1G33280	-1.14	-0.19	-1.33	0.225324	0.357464	-6.35
249909_s_a	alpha-adaptin	---	AT5G22770	-1.14	-0.18	-1.33	0.225339	0.357464	-6.35
261030_at	unknown protein contains similarity	---	AT1G17410	1.13	0.18	1.33	0.225527	0.357717	-6.35
264846_at	senescence-associated protein con	---	AT2G17850	-1.11	-0.15	-1.33	0.22553	0.357717	-6.35
265482_at	hypothetical protein contains a dom	---	AT2G15780	2.28	1.19	1.33	0.225604	0.357776	-6.35
262181_at	xylosidase, putative similar to xylos	---	AT1G78060	-1.32	-0.40	-1.33	0.225611	0.357776	-6.35

246875_at	pathogenesis-related protein - like	---	AT5G26130	-1.11	-0.15	-1.33	0.225614	0.357776	-6.35
253831_at	Expressed protein ; supported by fu	---	AT4G27580	-1.12	-0.16	-1.33	0.225648	0.357805	-6.35
249236_at	putative protein similar to unknown	---	AT5G41950	-1.10	-0.13	-1.33	0.225802	0.358025	-6.35
248270_at	putative protein similar to unknown	ORG1	AT5G53450	-1.27	-0.35	-1.33	0.225837	0.358043	-6.35
265878_at	putative SUPERMAN-like C2H2 zin	ZFP11	AT2G42410	-1.09	-0.13	-1.33	0.225845	0.358043	-6.35
250867_at	putative protein predicted protein, A	---	AT5G03880	-1.17	-0.23	-1.33	0.225943	0.358173	-6.35
253872_at	putative protein Arabidopsis thaliar	RD26	AT4G27410	1.19	0.25	1.33	0.225961	0.358176	-6.35
251022_at	putative protein Hsp70 binding prot	---	AT5G02150	1.13	0.17	1.33	0.225991	0.3582	-6.35
247667_at	putative protein predicted protein, A	---	AT5G60150	-1.13	-0.17	-1.33	0.226037	0.358247	-6.35
264658_at	hypothetical protein similar to LG27	---	AT1G09910	1.12	0.16	1.33	0.226109	0.358338	-6.35
260623_at	high-affinity nitrate transporter NRT	ATNRT2:1	AT1G08090	-1.13	-0.18	-1.33	0.226224	0.358489	-6.35
248401_at	putative protein similar to unknown	HCF208	AT5G52110	-1.23	-0.30	-1.33	0.226237	0.358489	-6.35
262009_at	hypothetical protein predicted by ge	---	AT1G35610	-1.13	-0.18	-1.33	0.226337	0.358623	-6.35
252804_at	putative protein	---	---	-1.09	-0.12	-1.33	0.226369	0.358637	-6.35
249597_at	putative protein probable quinone c	---	AT5G37960	-1.09	-0.12	-1.33	0.226377	0.358637	-6.35
264263_at	Expressed protein ; supported by fu	AtPP2-B15	AT1G09155	1.11	0.15	1.33	0.226527	0.35885	-6.35
246598_at	dolichyl-phosphate-mannose--glyco	---	AT5G14850	1.14	0.19	1.33	0.226556	0.358871	-6.35
259758_s_a	putative caffeic acid 3-O-methyltran	---	AT1G77520 ,	-1.11	-0.15	-1.33	0.226578	0.358881	-6.35
245474_at	hypothetical protein	---	AT4G16060	-1.14	-0.18	-1.33	0.226603	0.358892	-6.35
250258_at	floral homeotic protein AGL15 (sp|Q	AGL15	AT5G13790	-1.12	-0.16	-1.33	0.226616	0.358892	-6.35
267020_at	hypothetical protein predicted by ge	---	AT2G39320	-1.12	-0.16	-1.33	0.226736	0.359056	-6.35
266266_at	putative enolase (2-phospho-D-glyc	---	AT2G29560	-1.13	-0.18	-1.33	0.22682	0.359131	-6.35
255930_at	hypothetical protein predicted by ge	---	AT1G12660	-1.10	-0.14	-1.33	0.226826	0.359131	-6.35
256660_at	hypothetical protein similar to hypot	---	AT3G12060	-1.12	-0.16	-1.33	0.226857	0.359131	-6.35
252435_at	putative protein several hypothetica	---	---	-1.12	-0.17	-1.33	0.226861	0.359131	-6.35
250017_at	unknown protein	---	AT5G18140	-1.19	-0.25	-1.33	0.226876	0.359131	-6.35
264820_at	putative coproporphyrinogen III oxid	LIN2	AT1G03475	-1.09	-0.12	-1.33	0.226878	0.359131	-6.35
246175_s_a	putative protein hypothetical protein	---	AT5G28320 ,	-1.11	-0.15	-1.33	0.226899	0.359139	-6.35
267086_at	putative salt-inducible protein	---	AT2G32630	1.14	0.19	1.33	0.226914	0.359139	-6.35
265532_at	hypothetical protein predicted by ge	---	---	-1.12	-0.16	-1.33	0.22695	0.359171	-6.35
263872_at	putative cellulose synthase catalytic	CESA9	AT2G21770	-1.13	-0.18	-1.33	0.227006	0.359235	-6.35
261540_at	unknown protein	---	AT1G63610	-1.13	-0.17	-1.33	0.227044	0.359269	-6.35
249728_at	GTPase activator-like protein of Ra	---	AT5G24390	1.13	0.17	1.33	0.227173	0.35945	-6.35
257657_at	Expressed protein ; supported by fu	---	AT3G13235	1.10	0.13	1.33	0.227202	0.359471	-6.35
246203_at	putative protein ; supported by full-l	---	AT4G36610	-1.22	-0.29	-1.33	0.227225	0.359482	-6.35

248726_at	RAS superfamily GTP-binding prote	ATRABA4C	AT5G47960	1.61	0.69	1.33	0.22727	0.359515	-6.35
258109_at	hypothetical protein contains Pfam	HGL1	AT3G23640	1.12	0.16	1.33	0.227278	0.359515	-6.35
263487_at	hypothetical protein predicted by ge	---	AT2G31850	-1.10	-0.14	-1.33	0.22747	0.359794	-6.35
258985_at	hypothetical protein similar to hypo	---	AT3G08960	-1.10	-0.14	-1.33	0.22751	0.359832	-6.35
260048_at	unknown protein contains Pfam pro	---	AT1G73750	-1.46	-0.54	-1.33	0.227541	0.359857	-6.35
245231_at	hypothetical protein	---	AT4G25600	1.13	0.18	1.33	0.227593	0.359891	-6.35
260600_at	hypothetical protein predicted by ge	---	AT1G55950	-1.12	-0.16	-1.33	0.227594	0.359891	-6.35
250270_at	putative protein FL10, EGF respon	---	AT5G12980	1.13	0.18	1.33	0.227719	0.360063	-6.36
257538_at	hypothetical protein predicted by ge	---	AT3G16160	-1.10	-0.13	-1.33	0.227736	0.360065	-6.36
256526_at	disease resistance protein, putative	---	AT1G66090	-1.12	-0.16	-1.33	0.227849	0.360219	-6.36
246664_at	putative protein predicted proteins -	---	---	-1.11	-0.15	-1.33	0.227918	0.360304	-6.36
258408_at	unknown protein similar to putative	ATCHX19	AT3G17630	1.10	0.14	1.33	0.228001	0.36041	-6.36
256377_at	hypothetical protein	---	AT1G66650	-1.09	-0.12	-1.33	0.228035	0.360438	-6.36
264055_at	putative protein kinase contains a p	---	AT2G28590	1.13	0.18	1.33	0.228071	0.360469	-6.36
256703_at	MADS-box transcription factor simi	AGL79	AT3G30260	1.08	0.11	1.33	0.228117	0.360517	-6.36
252248_at	putative protein resistance gene Hc	AtRLP44	AT3G49750	-1.21	-0.27	-1.33	0.228218	0.360651	-6.36
261442_at	Expressed protein ; supported by fu	---	AT1G28375	1.11	0.15	1.33	0.228273	0.360693	-6.36
258199_at	unknown protein	---	AT3G14030	-1.11	-0.15	-1.33	0.228277	0.360693	-6.36
248888_at	potassium channel protein KAT1 (p	KAT1	AT5G46240	-1.31	-0.39	-1.33	0.228291	0.360693	-6.36
259681_at	nitrate reductase 1 (NR1) identical t	NIA1	AT1G77760	-2.15	-1.10	-1.32	0.228469	0.360949	-6.36
260378_at	putative actin similar to actin GB:CA	ATARP4A	AT1G73910	-1.09	-0.12	-1.32	0.228558	0.361065	-6.36
265864_at	putative myosin heavy chain-like pr	ATMAP70-	AT2G01750	1.16	0.21	1.32	0.228789	0.361405	-6.36
254461_at	receptor kinase - like protein SRK3	---	AT4G21230	1.16	0.22	1.32	0.228871	0.361486	-6.36
262919_at	unknown protein ; supported by cDI	---	AT1G79380	-1.16	-0.21	-1.32	0.228875	0.361486	-6.36
254897_at	serine/threonine kinase-like protein	---	AT4G11470	1.37	0.45	1.32	0.228888	0.361486	-6.36
264017_s_a	putative ER lumen protein retaining	---	AT2G21190 /	1.10	0.14	1.32	0.22898	0.361591	-6.36
264959_at	hypothetical protein predicted by ge	---	AT1G77090	-1.13	-0.17	-1.32	0.228987	0.361591	-6.36
245960_at	subtilisin-like protein subtilisin/kexi	S1P	AT5G19660	1.09	0.12	1.32	0.229015	0.361611	-6.36
248376_at	unknown protein ; supported by full-	---	AT5G51880	-1.11	-0.15	-1.32	0.229087	0.361699	-6.36
265437_at	hypothetical protein predicted by ge	MCM10	AT2G20980	-1.12	-0.16	-1.32	0.229106	0.361705	-6.36
259833_at	MYB-family transcription factor, pu	MYB105	AT1G69560	-1.10	-0.13	-1.32	0.229174	0.361771	-6.36
251410_at	uclacyanin 3 ; supported by cDNA:	UCC3	AT3G60280	-1.10	-0.13	-1.32	0.22918	0.361771	-6.36
263278_at	dynamin-like protein	DRP3B	AT2G14120	1.13	0.17	1.32	0.229348	0.361995	-6.36
259719_at	unknown protein contains gamma-t	LCR66	AT1G61070	-1.10	-0.13	-1.32	0.229353	0.361995	-6.36
253031_at	putative protein cellulose synthase	CSLD4	AT4G38190	-1.12	-0.16	-1.32	0.229428	0.362069	-6.36

253693_at	Thioredoxin - like protein thioredoxi	ACHT2	AT4G29670	-1.19	-0.26	-1.32	0.229432	0.362069	-6.36
254668_at	neoxanthin cleavage enzyme - like	NCED2	AT4G18350	-1.14	-0.19	-1.32	0.229477	0.362116	-6.36
253644_at	putative protein predicted protein, A	---	AT4G29870	1.16	0.21	1.32	0.229508	0.362138	-6.36
265178_at	Pto kinase interactor, putative simil	---	AT1G23540	-1.12	-0.16	-1.32	0.229604	0.362265	-6.36
258311_at	transport inhibitor response 1 (TIR1	AFB2	AT3G26810	-1.12	-0.16	-1.32	0.229702	0.362394	-6.36
254718_at	putative protein disease resistance	---	AT4G13580	-1.11	-0.16	-1.32	0.2298	0.362524	-6.36
262403_s_a	mutator-like transposase, putative s	---	---	1.09	0.13	1.32	0.22986	0.362595	-6.36
259116_at	putative peptide transporter similar	---	AT3G01350	1.21	0.27	1.32	0.229913	0.362652	-6.36
255517_at	putative endo-1,4-beta glucanase s	AtGH9B13	AT4G02290	-1.14	-0.19	-1.32	0.229947	0.362682	-6.36
257913_at	unknown protein ; supported by cD	LAG1	AT3G25540	1.12	0.16	1.32	0.230026	0.362781	-6.36
250095_at	phytoene synthase (gb|AAB65697.1	---	AT5G17230	-1.22	-0.29	-1.32	0.230073	0.36283	-6.36
255390_at	hypothetical protein similar to A. th	---	---	-1.11	-0.15	-1.32	0.230137	0.362892	-6.36
264898_at	Unknown protein ; supported by full	---	AT1G23205	-1.18	-0.24	-1.32	0.230156	0.362892	-6.36
256989_at	hypothetical protein predicted by ge	---	AT3G28580	1.85	0.89	1.32	0.230161	0.362892	-6.36
255892_at	hypothetical protein contains similar	---	AT1G17910	-1.10	-0.14	-1.32	0.230221	0.362963	-6.36
259443_at	chitinase, putative similar to chitina	---	AT1G02360	1.27	0.35	1.32	0.230249	0.362982	-6.37
258384_at	hypothetical protein predicted by ge	---	AT3G15490	-1.10	-0.14	-1.32	0.230294	0.363027	-6.37
255143_at	putative protein several hypothetica	---	AT4G08430	-1.08	-0.11	-1.32	0.230324	0.36305	-6.37
AFFX-r2-Ec	E. coli /GEN=bioB /DB_XREF=gb:J	---	---	-1.33	-0.41	-1.32	0.230344	0.363056	-6.37
252053_at	syntaxin-like protein synt4 ;support	SYP122	AT3G52400	1.40	0.49	1.32	0.230514	0.363277	-6.37
248496_at	MtN3-like protein ;supported by full	---	AT5G50790	-1.14	-0.18	-1.32	0.230516	0.363277	-6.37
244930_at	hypothetical protein	---	AT2G07809	1.10	0.13	1.32	0.230585	0.363361	-6.37
256942_at	hypothetical protein similar to unkno	LSH4	AT3G23290	-1.36	-0.44	-1.32	0.23067	0.363469	-6.37
263625_at	F-box protein family, AtFBX9 conta	---	AT2G04920	-1.10	-0.14	-1.32	0.230719	0.363519	-6.37
250949_at	putative protein	---	AT5G03510	-1.10	-0.13	-1.32	0.230733	0.363519	-6.37
250310_at	unknown protein	---	AT5G12230	1.15	0.20	1.32	0.230771	0.363553	-6.37
265565_at	putative retroelement pol polyprotei	---	---	-1.11	-0.15	-1.32	0.230943	0.363787	-6.37
265198_at	putative glucosyl transferase	UGT73C2	AT2G36760	-1.10	-0.13	-1.32	0.230952	0.363787	-6.37
259742_at	putative proline-rich APG protein sir	GLIP6	AT1G71120	-1.17	-0.23	-1.32	0.230984	0.363814	-6.37
261469_at	unknown protein	AtRLP4	AT1G28340	-1.11	-0.15	-1.32	0.231096	0.363965	-6.37
247526_at	putative protein C2H2 zinc-finger p	---	AT5G61470	-1.10	-0.14	-1.32	0.231222	0.364139	-6.37
251668_at	putative protein strictosidine syntha	---	AT3G57010	-1.26	-0.34	-1.32	0.231271	0.36419	-6.37
262693_at	unknown protein	---	AT1G62780	-1.25	-0.32	-1.32	0.231311	0.364228	-6.37
251245_at	putative protein phytochrome-asso	PIL2	AT3G62090	1.37	0.46	1.32	0.231345	0.364257	-6.37
267290_at	putative SET-domain transcriptiona	---	AT2G23740	-1.13	-0.18	-1.32	0.231384	0.364271	-6.37

263128_at	zinc finger protein, putative similar	LZF1	AT1G78600	1.21	0.27	1.32	0.2314	0.364271	-6.37
257465_at	hypothetical protein predicted by ge	---	AT1G13040	1.09	0.12	1.32	0.231406	0.364271	-6.37
253624_at	putative protein 2-acylglycerophos	ATS2	AT4G30580	1.14	0.19	1.32	0.231419	0.364271	-6.37
251761_at	glucuronosyl transferase - like prot	---	AT3G55700	1.15	0.21	1.32	0.2315	0.364374	-6.37
261777_at	hypothetical protein predicted by ge	---	AT1G76210	-1.11	-0.15	-1.32	0.231532	0.364383	-6.37
266101_at	unknown protein ; supported by cD	---	AT2G37940	1.35	0.43	1.32	0.231538	0.364383	-6.37
266334_at	hypothetical protein predicted by ge	---	AT2G32380	-1.19	-0.25	-1.31	0.231577	0.36442	-6.37
259370_at	hypothetical protein predicted by ge	---	AT1G69050	1.14	0.18	1.31	0.231727	0.364631	-6.37
267528_at	MADS-box protein (AGL6) ; suppo	AGL6	AT2G45650	-1.11	-0.15	-1.31	0.231743	0.364631	-6.37
261877_at	putative indole-3-acetate beta-gluc	---	AT1G50580	-1.11	-0.15	-1.31	0.23177	0.364649	-6.37
265360_s_a	putative Athila retroelement ORF1 p	---	---	-1.11	-0.15	-1.31	0.23186	0.364766	-6.37
249227_at	peroxidase (emb|CAA66960.1)	---	AT5G42180	-1.11	-0.14	-1.31	0.231902	0.364798	-6.37
247390_at	putative protein similar to unknown	---	AT5G63520	1.10	0.13	1.31	0.231913	0.364798	-6.37
246946_at	putative protein	---	AT5G25070	-1.09	-0.13	-1.31	0.231948	0.364827	-6.37
257229_at	putative calmodulin-binding protein	IQD26	AT3G16490	-1.14	-0.20	-1.31	0.231982	0.364842	-6.37
250278_at	2-oxoglutarate/malate translocator	DiT1	AT5G12860	1.20	0.27	1.31	0.231989	0.364842	-6.37
253147_at	protein kinase - like protein serine/	CONNEXIN	AT4G35600	1.21	0.27	1.31	0.232039	0.364895	-6.37
255134_at	putative protein	---	AT4G08330	1.12	0.16	1.31	0.232092	0.364954	-6.37
248978_at	putative protein contains similarity t	AtPP2-A8	AT5G45070	-1.15	-0.20	-1.31	0.23215	0.36502	-6.37
264671_at	unknown protein ; supported by ful	---	AT1G09920	1.13	0.17	1.31	0.23219	0.365045	-6.37
245834_at	transposon protein, putative similar	---	---	-1.09	-0.13	-1.31	0.232198	0.365045	-6.37
248280_at	putative protein similar to unknown	---	AT5G52950	-1.11	-0.15	-1.31	0.232221	0.365055	-6.37
245226_at	gene_id:K17E7.15~unknown protei	---	AT3G29970	1.63	0.71	1.31	0.23227	0.365104	-6.37
244923_s_a	orf153b orf153b	---	AT2G07674	-1.19	-0.26	-1.31	0.232283	0.365104	-6.37
251842_at	extensin precursor -like protein ext	---	AT3G54580	-1.30	-0.37	-1.31	0.232311	0.365122	-6.37
261542_at	unknown protein contains Pfam pro	---	AT1G63560	-1.13	-0.17	-1.31	0.232365	0.365182	-6.37
255353_at	putative transposon protein similar t	---	---	-1.09	-0.13	-1.31	0.232391	0.365197	-6.37
253011_at	putative protein retrotransposon -li	EDA40	AT4G37890	-1.23	-0.30	-1.31	0.232462	0.365255	-6.37
262187_at	hypothetical protein	---	AT1G77960	1.10	0.14	1.31	0.232465	0.365255	-6.37
244903_at	hypothetical protein	---	---	1.32	0.40	1.31	0.232476	0.365255	-6.37
247995_at	putative protein contains similarity t	---	AT5G56160	-1.15	-0.20	-1.31	0.232503	0.365272	-6.37
245455_at	disease resistance RPP5 like protei	---	AT4G16930	-1.10	-0.14	-1.31	0.232534	0.365272	-6.37
255707_at	predicted protein	MEE50	AT4G00231	1.09	0.13	1.31	0.232563	0.365272	-6.37
261915_at	amp-binding protein, putative simil	BZO1	AT1G65880	1.08	0.12	1.31	0.232573	0.365272	-6.37
252236_at	zinc-finger-like protein Cys2/His2-t	---	AT3G49930	1.11	0.15	1.31	0.232582	0.365272	-6.37

246506_at	putative protein hypothetical protei	---	AT5G16110	1.14	0.20	1.31	0.232593	0.365272	-6.37
263299_s_a	hypothetical protein predicted by ge	---	AT2G11570	-1.11	-0.14	-1.31	0.232599	0.365272	-6.37
247433_at	E2, ubiquitin-conjugating enzyme 3	UBC3	AT5G62540	-1.18	-0.23	-1.31	0.232644	0.365318	-6.37
253978_at	putative protein probable kinesin -	---	AT4G26660	-1.14	-0.18	-1.31	0.232675	0.365341	-6.37
257714_at	histone H3, putative similar to histo	---	AT3G27360	-1.13	-0.18	-1.31	0.232735	0.36541	-6.37
250015_at	N-acetylglucosamine-phosphate mu	DRT101	AT5G18070	-1.22	-0.29	-1.31	0.232886	0.365623	-6.37
259944_at	hypothetical protein predicted by ge	---	AT1G71470	-1.10	-0.14	-1.31	0.232998	0.365756	-6.38
266186_at	unknown protein	AERO2	AT2G38960	1.09	0.13	1.31	0.233003	0.365756	-6.38
252577_at	putative protein putative proteins -	---	AT3G45460	-1.09	-0.12	-1.31	0.233076	0.365846	-6.38
267194_s_a	putative ARP2/3 protein complex su	ARPC1A	AT2G30910	-1.11	-0.15	-1.31	0.233203	0.366019	-6.38
258464_at	kinesin-like protein similar to KLP2	POK1	AT3G17360	-1.08	-0.12	-1.31	0.233271	0.366099	-6.38
248724_at	putative protein strong similarity to	---	AT5G47970	1.20	0.26	1.31	0.233291	0.366099	-6.38
264528_at	hypothetical protein similar to Huma	---	AT1G30810	1.09	0.12	1.31	0.233329	0.366099	-6.38
258554_at	putative DEAD/DEAH box helicase	---	AT3G06980	1.10	0.14	1.31	0.233332	0.366099	-6.38
266870_at	putative heterogeneous nuclear ribo	---	AT2G44710	1.17	0.23	1.31	0.233344	0.366099	-6.38
260963_at	hypothetical protein predicted by ge	---	AT1G44990	-1.11	-0.15	-1.31	0.233372	0.366099	-6.38
265166_at	putative OBP32pep protein	---	AT1G23640	1.09	0.13	1.31	0.233378	0.366099	-6.38
247489_at	carbonyl reductase - like protein in	---	AT5G61830	-1.12	-0.17	-1.31	0.233387	0.366099	-6.38
257565_at	hypothetical protein predicted by ge	---	AT3G28620	1.09	0.13	1.31	0.233398	0.366099	-6.38
250626_at	putative protein p100 co-activator -	---	AT5G07350	-1.11	-0.15	-1.31	0.233432	0.366128	-6.38
254001_at	putative protein PRE87 mRNA, Pin	MIOX4	AT4G26260	1.38	0.46	1.31	0.233531	0.366257	-6.38
255597_at	hypothetical protein	---	AT4G01730	-1.12	-0.16	-1.31	0.233554	0.366266	-6.38
249795_at	40S ribosomal protein S11 ;support	RPS11-BE	AT5G23740	1.22	0.29	1.31	0.233572	0.366266	-6.38
254406_at	putative transposable element reve	---	---	-1.09	-0.13	-1.31	0.233585	0.366266	-6.38
267641_at	Argonaute (AGO1)-like protein	AGO6	AT2G32940	-1.09	-0.13	-1.31	0.233736	0.366477	-6.38
247626_at	receptor like protein kinase recepto	---	AT5G60300	-1.38	-0.46	-1.31	0.23382	0.366584	-6.38
259803_at	cytosolic factor, putative similar to	PATL1	AT1G72150	-1.11	-0.14	-1.31	0.233858	0.366619	-6.38
266056_at	hypothetical protein predicted by ge	ATATG18C	AT2G40810	-1.09	-0.13	-1.31	0.233947	0.366733	-6.38
261465_at	unknown protein	---	AT1G07705	-1.10	-0.14	-1.31	0.233981	0.366758	-6.38
251901_at	putative protein axi1 protein, Nicoti	---	AT3G54100	-1.14	-0.19	-1.31	0.233995	0.366758	-6.38
245974_at	putative protein	---	---	-1.18	-0.24	-1.31	0.234087	0.366876	-6.38
252462_at	putative protein various predicted g	---	AT3G47250	-1.10	-0.14	-1.31	0.234103	0.366876	-6.38
256615_at	hypothetical protein predicted by ge	---	AT3G22250	-1.14	-0.19	-1.31	0.2342	0.367003	-6.38
260470_at	hypothetical protein predicted by ge	---	AT1G11120	-1.14	-0.18	-1.31	0.23428	0.367092	-6.38
262096_at	NAC1 identical to NAC1 GB:AAF21	NAC1	AT1G56010	1.26	0.34	1.31	0.234289	0.367092	-6.38

248621_at	putative protein similar to unknown	---	AT5G49350	-1.11	-0.15	-1.31	0.234329	0.367131	-6.38
247699_at	GTP-binding protein ara-3 GTP-bir	---	AT5G59840	1.20	0.26	1.31	0.23436	0.367154	-6.38
258602_at	putative protein phosphatase-2C (P	---	AT3G02750	-1.09	-0.12	-1.31	0.234639	0.367565	-6.38
257849_at	unknown protein	ECT5	AT3G13060	1.15	0.20	1.30	0.234755	0.367713	-6.38
246637_at	putative protein polymorphic memb	---	AT5G34870	-1.12	-0.16	-1.30	0.234772	0.367713	-6.38
256820_at	far-red impaired response protein,	FHY3	AT3G22170	1.09	0.13	1.30	0.234782	0.367713	-6.38
245412_at	hypothetical protein	---	AT4G17280	-1.18	-0.24	-1.30	0.234809	0.367731	-6.38
251904_at	Machado-Joseph disease MJD1a -	---	AT3G54130	1.15	0.20	1.30	0.234912	0.367867	-6.38
263792_at	putative retroelement pol polyprotei	---	---	-1.14	-0.19	-1.30	0.234952	0.367903	-6.38
266686_at	hypothetical protein	---	AT2G19700	-1.11	-0.15	-1.30	0.235002	0.367956	-6.38
252666_at	putative protein hypothetical protein	---	AT3G44180	-1.12	-0.16	-1.30	0.235055	0.367994	-6.38
262655_s_a	putative mandelonitrile lyase simila	---	AT1G14185	1.09	0.13	1.30	0.235058	0.367994	-6.38
256381_at	lipid transfer protein, putative conta	---	AT1G66850	-1.12	-0.17	-1.30	0.235172	0.368148	-6.38
252035_at	beta-ketoacyl-CoA synthase like pr	KCS15	AT3G52160	-1.13	-0.17	-1.30	0.235237	0.368224	-6.38
263439_at	hypothetical protein predicted by ge	ATEXO70H	AT2G28650	-1.12	-0.16	-1.30	0.235307	0.368285	-6.38
254513_at	cytochrome p450 like protein cytoc	CYP71A27	AT4G20240	-1.09	-0.13	-1.30	0.235308	0.368285	-6.38
264801_at	hypothetical protein gene overlaps	emb2411	AT1G08840	-1.11	-0.15	-1.30	0.235475	0.368516	-6.38
244991_s_a	NADH dehydrogenase ND2	---	---	-1.11	-0.14	-1.30	0.235499	0.368516	-6.38
256806_at	CCAAT-binding factor B chain, puta	NF-YA9	AT3G20910	1.12	0.17	1.30	0.235516	0.368516	-6.38
258053_at	unknown protein similar to CGI-18 p	---	AT3G16230	1.09	0.12	1.30	0.23553	0.368516	-6.38
258835_at	putative RNA-binding protein conta	---	AT3G07250	-1.12	-0.17	-1.30	0.235537	0.368516	-6.38
264216_at	hypothetical protein similar to hypot	---	AT1G60180	-1.09	-0.13	-1.30	0.235619	0.368603	-6.39
247083_at	unknown protein	---	AT5G66290	1.10	0.14	1.30	0.235633	0.368603	-6.39
246377_at	low-temperature-specific protein, p	---	AT1G57550	-1.18	-0.24	-1.30	0.235656	0.368603	-6.39
254394_at	subtilisin-like serine protease conta	---	AT4G21630	-1.09	-0.12	-1.30	0.235657	0.368603	-6.39
245564_at	hypothetical protein	emb2739	AT4G14590	1.12	0.17	1.30	0.235694	0.368627	-6.39
252903_at	putative protein various predicted p	---	AT4G39570	1.19	0.25	1.30	0.235704	0.368627	-6.39
261799_at	unknown protein contains similarity	---	AT1G30473	-1.16	-0.21	-1.30	0.235747	0.368667	-6.39
258080_at	unknown protein ;supported by full-	---	AT3G25930	-1.09	-0.12	-1.30	0.235962	0.368972	-6.39
256361_at	formyl transferase, putative contain	pde194	AT1G66520	1.14	0.19	1.30	0.235991	0.368972	-6.39
265413_s_a	hypothetical protein predicted by ge	---	AT2G16650	-1.12	-0.16	-1.30	0.235992	0.368972	-6.39
252172_at	putative protein predicted proteins,	---	AT3G50640	-1.11	-0.15	-1.30	0.236006	0.368972	-6.39
258268_at	putative WD-repeat protein contain	WSIP2	AT3G15880	1.18	0.23	1.30	0.236132	0.369143	-6.39
265835_at	putative retroelement pol polyprotei	---	---	-1.13	-0.18	-1.30	0.236153	0.369151	-6.39
250498_at	microbody NAD-dependent malate	PMDH2	AT5G09660	-1.18	-0.24	-1.30	0.236252	0.369281	-6.39

253602_s_a	leucyl aminopeptidase - like protei	---	AT4G30910	1.10	0.14	1.30	0.236326	0.369348	-6.39
262550_at	hypothetical protein similar to hypot	---	AT1G31310	-1.10	-0.14	-1.30	0.236328	0.369348	-6.39
257077_at	PR-1 protein, putative similar to PR	---	AT3G19690	-1.16	-0.21	-1.30	0.236384	0.36941	-6.39
247551_at	ras-GTPase-activating protein SH3	---	AT5G60980	1.09	0.12	1.30	0.236472	0.369523	-6.39
AFFX-ThrX-	B. subtilis /GEN=thrC /DB_XREF=g	---	---	-1.23	-0.29	-1.30	0.236567	0.369647	-6.39
245366_at	Expressed protein ; supported by fu	AtMAPR4	AT4G14965	1.09	0.13	1.30	0.236733	0.369881	-6.39
254156_at	Rab geranylgeranyl transferase like	---	AT4G24490	-1.15	-0.21	-1.30	0.236751	0.369883	-6.39
244956_s_a	hypothetical protein	---	---	1.12	0.16	1.30	0.236806	0.369943	-6.39
260891_at	cysteine protease contains similarity	---	AT1G29080	-1.11	-0.16	-1.30	0.237085	0.370354	-6.39
254308_at	putative protein snRNP assembly f	---	AT4G22410	1.11	0.14	1.30	0.237144	0.37042	-6.39
247649_at	KED - like protein KED, Nicotiana t	---	AT5G60030	1.12	0.16	1.30	0.237254	0.370567	-6.39
247481_at	putative protein predicted protein, A	---	AT5G62400	-1.09	-0.13	-1.30	0.23729	0.370598	-6.39
266786_at	putative non-LTR retroelement reve	---	---	1.10	0.13	1.30	0.23744	0.370806	-6.39
248435_at	oleosin-like	OLEO3	AT5G51210	-1.13	-0.18	-1.30	0.237517	0.370901	-6.39
256712_at	unknown protein similar to T14G11.	---	AT2G34020	-1.09	-0.12	-1.30	0.237651	0.371085	-6.39
256810_at	adenyl cyclase, putative similar to a	---	AT3G21465	1.12	0.17	1.30	0.237848	0.371368	-6.39
256387_at	hypothetical protein	---	AT3G06170	1.14	0.19	1.30	0.237895	0.371415	-6.39
254829_at	AIR1A-like protein putative cell wal	---	AT4G12530	-1.10	-0.13	-1.29	0.238017	0.371581	-6.39
251634_at	putative protein putative zinc finger	---	AT3G57480	-1.11	-0.15	-1.29	0.238225	0.371881	-6.39
251315_at	putative protein protein kinase hom	---	AT3G61410	-1.13	-0.17	-1.29	0.238349	0.372048	-6.40
257121_at	hypothetical protein similar to auxin	---	AT3G20220	-1.11	-0.15	-1.29	0.238432	0.372153	-6.40
248071_at	unknown protein	---	AT5G55670	1.11	0.15	1.29	0.238506	0.372237	-6.40
262670_s_a	transposase [Escherichia coli] must	---	---	-1.10	-0.14	-1.29	0.238519	0.372237	-6.40
261890_at	hypothetical protein predicted by ge	---	AT1G80970	-1.20	-0.26	-1.29	0.238613	0.372358	-6.40
245705_at	zinc finger transcription factor-like p	---	AT5G04390	1.10	0.13	1.29	0.238638	0.372372	-6.40
264947_at	heat shock protein DnaJ, putative s	---	AT1G77020	-1.14	-0.19	-1.29	0.238714	0.372465	-6.40
253130_at	putative protein ;supported by full-l	---	AT4G35510	-1.19	-0.25	-1.29	0.238818	0.372602	-6.40
264660_at	putative glutamyl-tRNA reductase 2	HEMA2	AT1G09940	1.33	0.41	1.29	0.238924	0.372742	-6.40
263401_at	hypothetical protein similar to hypot	---	AT2G04070	1.18	0.24	1.29	0.238944	0.372747	-6.40
252069_s_a	putative protein putative protein T1	---	AT3G51640	-1.10	-0.14	-1.29	0.239002	0.372812	-6.40
265448_at	putative DOF zinc finger protein	DAG2	AT2G46590	-1.14	-0.19	-1.29	0.239024	0.372822	-6.40
260075_at	putative integral membrane protein	---	AT1G73700	1.14	0.19	1.29	0.239177	0.373034	-6.40
265980_at	hypothetical protein predicted by ge	---	---	-1.12	-0.16	-1.29	0.239282	0.373149	-6.40
256224_at	GTP-binding protein (SAR1B) ident	ATSAR1B	AT1G56330	-1.16	-0.22	-1.29	0.239283	0.373149	-6.40
253739_at	putative protein predicted protein. A	---	AT4G28760	-1.14	-0.19	-1.29	0.239355	0.373236	-6.40

245309_at	putative protein ; supported by cDN	---	AT4G15140	1.19	0.25	1.29	0.239397	0.373275	-6.40
251874_at	putative protein (fragment) esteras	---	AT3G54240	-1.14	-0.19	-1.29	0.239428	0.373299	-6.40
256541_at	ligand-gated ion channel, putative (	GLR3.3	AT1G42540	1.15	0.20	1.29	0.239457	0.373318	-6.40
246534_at	putative protein several hypothetica	---	AT5G15890	-1.14	-0.19	-1.29	0.239597	0.373511	-6.40
248333_at	photoassimilate-responsive protein	---	AT5G52390	1.60	0.68	1.29	0.239746	0.373697	-6.40
267485_at	putative MYB family transcription fa	MYB88	AT2G02820	1.14	0.19	1.29	0.239749	0.373697	-6.40
254295_at	putative protein predicted protein, A	---	AT4G23080	-1.09	-0.12	-1.29	0.239811	0.373768	-6.40
265597_at	Expressed protein ; supported by cI	---	AT2G20142	1.39	0.47	1.29	0.239862	0.373822	-6.40
244973_at	PSII T protein	---	---	-1.27	-0.35	-1.29	0.240069	0.374118	-6.40
249061_at	putative protein similar to unknown	---	AT5G44550	-1.10	-0.14	-1.29	0.240089	0.374124	-6.40
256085_at	hypothetical protein predicted by ge	---	AT1G20760	-1.09	-0.12	-1.29	0.240288	0.374383	-6.40
264703_at	serine threonine protein phosphatas	PP2A	AT1G69960	1.09	0.13	1.29	0.240288	0.374383	-6.40
263213_at	unknown protein Strong similarity to	---	AT1G30560	-1.12	-0.17	-1.29	0.240317	0.374402	-6.40
247391_at	unknown protein	---	AT5G63540	-1.09	-0.12	-1.29	0.240343	0.374417	-6.40
256605_at	hypothetical protein predicted by ge	---	AT3G32940	1.12	0.16	1.29	0.240474	0.374596	-6.40
254361_at	Expressed protein ; supported by fu	---	AT4G22212	-1.14	-0.19	-1.29	0.240532	0.374661	-6.40
247930_at	putative protein similar to unknown	---	AT5G57060	1.17	0.22	1.29	0.240616	0.37476	-6.40
265355_at	hypothetical protein predicted by ge	---	AT2G16760	-1.18	-0.24	-1.29	0.240641	0.37476	-6.40
263118_at	putative 3-methylcrotonyl-CoA carb	MCCA	AT1G03090	-1.30	-0.37	-1.29	0.240645	0.37476	-6.40
250599_at	unknown protein ; supported by cD	---	AT5G07730	-1.15	-0.20	-1.29	0.240677	0.374783	-6.40
260403_at	hypothetical protein predicted by ge	WRKY36	AT1G69810	-1.09	-0.13	-1.29	0.240692	0.374783	-6.40
249099_at	putative protein similar to unknown	---	AT5G43550	-1.08	-0.11	-1.29	0.240747	0.374843	-6.40
256423_at	serine carboxypeptidase, putative s	scpl18	AT1G33540	-1.09	-0.12	-1.29	0.240844	0.374945	-6.40
246168_at	meristem protein - like reproductive	---	AT5G32460	1.17	0.23	1.29	0.240846	0.374945	-6.40
267369_at	unknown protein ;supported by full-	---	AT2G44420	1.12	0.17	1.29	0.240914	0.375026	-6.40
257653_at	hypothetical protein	---	AT3G13225	-1.13	-0.18	-1.29	0.241191	0.375394	-6.41
248115_at	putative protein strong similarity to u	---	AT5G54870	1.20	0.27	1.29	0.241197	0.375394	-6.41
260113_at	hypothetical protein predicted by ge	---	AT1G63300	-1.09	-0.12	-1.29	0.2412	0.375394	-6.41
255914_at	hypothetical protein predicted by ge	---	AT1G20290	-1.13	-0.17	-1.28	0.241265	0.375469	-6.41
254069_at	putative protein similarity to antiser	ATNUDT10	AT4G25434	1.16	0.21	1.28	0.241405	0.375661	-6.41
256857_at	NAM-like protein similar to NAM (no	CUC1	AT3G15170	-1.08	-0.12	-1.28	0.241442	0.375689	-6.41
257417_at	hypothetical protein predicted by ge	---	AT1G10110	-1.11	-0.16	-1.28	0.241456	0.375689	-6.41
252125_at	putative protein hypothetical protei	RTH	AT3G51040	-1.11	-0.15	-1.28	0.241494	0.375715	-6.41
261368_at	protein kinase, putative similar to m	---	AT1G53070	-1.15	-0.20	-1.28	0.241514	0.375715	-6.41
251222_at	putative membrane protein clone:2	---	AT3G62580	-1.19	-0.25	-1.28	0.24153	0.375715	-6.41

257904_at	myb transcription factor, putative si	TDF1	AT3G28470	-1.09	-0.12	-1.28	0.241538	0.375715	-6.41
245891_at	amino acid transport protein AAP2	AAP2	AT5G09220	-1.32	-0.40	-1.28	0.241562	0.375726	-6.41
247603_at	microtubule-associated motor - like	---	AT5G60930	-1.15	-0.20	-1.28	0.241677	0.37588	-6.41
260277_at	hypothetical protein predicted by ge	---	AT1G80520	1.24	0.31	1.28	0.241792	0.376033	-6.41
260238_at	AtHVA22a identical to AtHVA22a C	ATHVA22A	AT1G74520	-1.14	-0.19	-1.28	0.241836	0.376076	-6.41
247273_at	unknown protein	CPuORF40	AT5G64340 /	-1.18	-0.24	-1.28	0.241867	0.376099	-6.41
257967_at	hypothetical protein predicted by ge	---	AT3G19910	1.10	0.14	1.28	0.241887	0.376104	-6.41
251868_at	RNA polymerase 24kDa subunit -li	RPB5E	AT3G54490	-1.11	-0.16	-1.28	0.242258	0.37663	-6.41
249122_at	putative protein similar to unknown	ARD4	AT5G43850	1.16	0.22	1.28	0.242266	0.37663	-6.41
264348_at	putative NPK1-related protein kinas	NRT1.1	AT1G12110	-1.80	-0.85	-1.28	0.242275	0.37663	-6.41
255866_at	hypothetical protein predicted by ge	---	AT2G30350	-1.12	-0.16	-1.28	0.242403	0.376804	-6.41
251009_at	putative protein predicted proteins,	---	AT5G02640	-1.24	-0.32	-1.28	0.242443	0.376839	-6.41
263252_at	putative CONSTANS-like B-box zin	STH	AT2G31380	-1.19	-0.25	-1.28	0.24247	0.376856	-6.41
263141_at	hypothetical protein similar to unkno	---	AT1G65210	-1.09	-0.13	-1.28	0.2425	0.376877	-6.41
259332_at	putative auxin-induced protein simil	---	AT3G03830	-1.11	-0.15	-1.28	0.242656	0.377094	-6.41
249773_at	squalene monooxygenase 2 (squal	SQP2	AT5G24140	-1.11	-0.15	-1.28	0.242775	0.377253	-6.41
265819_at	Expressed protein ; supported by fu	---	AT2G17972	-1.13	-0.17	-1.28	0.24285	0.377344	-6.41
247290_at	putative protein similar to unknown	---	AT5G64450	1.22	0.29	1.28	0.242894	0.377387	-6.41
266785_at	putative receptor-like protein kinase	---	AT2G28970	-1.11	-0.15	-1.28	0.243045	0.377596	-6.41
261202_at	flower pigmentation protein ATAN1	ATAN11	AT1G12910	-1.11	-0.16	-1.28	0.24307	0.377608	-6.41
251060_at	putative protein serine/threonine pr	ATSR1	AT5G01820	1.50	0.58	1.28	0.243121	0.377662	-6.41
256344_at	C2H2-type zinc finger protein, puta	TFIIIA	AT1G72050	1.13	0.17	1.28	0.243172	0.377713	-6.41
253930_at	embryo-specific protein 1 (ATS1)	ATS1	AT4G26740	-1.11	-0.14	-1.28	0.243188	0.377713	-6.41
253878_at	trehalose-6-phosphate synthase - l	TPS4	AT4G27550	-1.09	-0.13	-1.28	0.243204	0.377713	-6.41
254317_at	hypothetical protein	---	AT4G22510	-1.09	-0.12	-1.28	0.243243	0.377746	-6.41
267392_at	putative beta-glucosidase	PEN2	AT2G44490	1.17	0.23	1.28	0.243267	0.377746	-6.41
245146_at	putative cis-Golgi SNARE protein ;	GOS12	AT2G45200	-1.12	-0.16	-1.28	0.243274	0.377746	-6.41
263052_at	unknown protein	---	AT2G13430	-1.10	-0.13	-1.28	0.243494	0.378061	-6.41
247044_at	MAP protein kinase ; supported by	MAPKKK5	AT5G66850	1.15	0.20	1.28	0.243521	0.378078	-6.41
261876_at	pirin-like protein similar to pirin GB:/	---	AT1G50590	1.30	0.38	1.28	0.243546	0.378091	-6.41
253894_at	NWMU2 - 2S albumin 2 precursor	---	AT4G27150	1.09	0.13	1.28	0.2436	0.378148	-6.41
250171_at	prohibitin - like protein prohibitin, A	ATPHB5	AT5G14300	-1.10	-0.14	-1.28	0.243646	0.378195	-6.41
248341_at	unknown protein	---	AT5G52220	-1.14	-0.19	-1.28	0.243755	0.378337	-6.41
265328_at	unknown protein	---	AT2G18200	-1.08	-0.11	-1.28	0.243816	0.378406	-6.41
248076_at	unknown protein	---	AT5G55750	-1.09	-0.13	-1.28	0.243933	0.378562	-6.42

254937_at	putative aldolase similar to 2,4-dihy	---	AT4G10750	1.09	0.13	1.28	0.244002	0.378644	-6.42
250831_at	unknown protein	---	AT5G04920	-1.10	-0.14	-1.28	0.244059	0.378706	-6.42
249160_at	putative protein similar to unknown	---	AT5G42660	1.10	0.13	1.28	0.244116	0.378769	-6.42
246694_at	putative protein	---	---	-1.10	-0.14	-1.28	0.244171	0.378811	-6.42
254994_at	putative methyltransferase	MTA	AT4G10760	1.20	0.27	1.28	0.244176	0.378811	-6.42
262141_s_a	hypothetical protein predicted by ge	---	AT1G52440	1.08	0.11	1.28	0.244245	0.378892	-6.42
265581_at	putative cAMP-dependent protein k	---	AT2G20050	-1.10	-0.14	-1.28	0.244308	0.378964	-6.42
254439_s_a	carbonic anhydrase -like protein ca	ACA6	AT4G20990	-1.08	-0.11	-1.28	0.244327	0.378967	-6.42
256073_at	terminal Flower 1 (TFL1), putative	E12A11	AT1G18100	-1.12	-0.17	-1.28	0.244345	0.37897	-6.42
255269_at	putative protein various predicted p	---	AT4G05240	-1.11	-0.15	-1.28	0.244443	0.379082	-6.42
250118_at	putative protein similar to unknown	---	AT5G16460	-1.11	-0.16	-1.28	0.244451	0.379082	-6.42
252195_at	putative protein various predicted p	---	AT3G50190	-1.12	-0.17	-1.28	0.244504	0.379139	-6.42
249768_at	receptor-like protein kinase	---	AT5G24100	-1.13	-0.18	-1.28	0.244558	0.379197	-6.42
246859_at	putative protein various predicted p	---	AT5G25950	-1.10	-0.14	-1.27	0.244586	0.379215	-6.42
245589_at	subtilisin-like serine protease conta	---	AT4G15040	-1.16	-0.21	-1.27	0.244637	0.379269	-6.42
262681_at	anter-specific proline-rich -like prote	---	AT1G75890	-1.09	-0.12	-1.27	0.244709	0.379354	-6.42
252459_s_a	1-phosphatidylinositol-4,5-bisphos	PLC9	AT3G47220	-1.09	-0.13	-1.27	0.24476	0.379407	-6.42
264991_s_a	putative protein hypothetical protein	---	AT1G67400	-1.16	-0.22	-1.27	0.245004	0.379758	-6.42
252999_at	putative protein other Arabidopsis h	---	AT4G38500	-1.11	-0.15	-1.27	0.24502	0.379758	-6.42
250367_s_a	DEAD BOX RNA helicase RH15 ; s	---	AT5G11170	1.11	0.15	1.27	0.245037	0.37976	-6.42
266537_at	Expressed protein ; supported by fu	---	AT2G16860	1.11	0.15	1.27	0.24516	0.379919	-6.42
266596_at	hypothetical protein predicted by ge	---	AT2G46150	1.29	0.37	1.27	0.245174	0.379919	-6.42
262412_at	14-3-3 protein GF14omicron (grf11)	GRF11	AT1G34760	-1.13	-0.17	-1.27	0.245226	0.379975	-6.42
259975_at	putative cinnamoyl-CoA reductase	---	AT1G76470	1.37	0.45	1.27	0.245307	0.380074	-6.42
249671_at	putative protein similar to unknown	LBD35	AT5G35900	-1.11	-0.15	-1.27	0.245389	0.380175	-6.42
253091_at	cytochrome p450 - like protein cyto	CYP81D2	AT4G37360	-1.10	-0.14	-1.27	0.24549	0.380289	-6.42
251488_at	calmodulin-like protein calcium-bin	---	AT3G59440	-1.08	-0.11	-1.27	0.245496	0.380289	-6.42
264813_at	putative Ca2+-dependent ser/thr p	CPK16	AT2G17890	-1.15	-0.20	-1.27	0.24559	0.38041	-6.42
245807_at	AP2 domain containing protein RAF	RAP2.1	AT1G46768	1.24	0.31	1.27	0.245739	0.380614	-6.42
244966_at	cytochrome b6-f complex, subunit	---	---	-1.17	-0.22	-1.27	0.245845	0.380746	-6.42
252744_at	putative protein hypothetical protei	---	AT3G43310	-1.10	-0.14	-1.27	0.245857	0.380746	-6.42
250334_at	NADH dehydrogenase (ubiquinone)	---	AT5G11770	1.11	0.15	1.27	0.245886	0.380764	-6.42
253616_at	blight-associated protein homolog	EXLB2	AT4G30380	-1.09	-0.13	-1.27	0.245921	0.380792	-6.42
246970_at	cytochrome P450-like protein fatty	CYP714A2	AT5G24900	-1.18	-0.24	-1.27	0.246013	0.38091	-6.42
267059_at	putative carboxymethylenebutenoli	---	AT2G32520	1.11	0.14	1.27	0.246093	0.381007	-6.42

261024_at	hypothetical protein predicted by ge	---	AT1G12190	-1.09	-0.13	-1.27	0.246166	0.381094	-6.42
262844_at	unknown protein similar to pectines	---	AT1G14890	-1.32	-0.40	-1.27	0.246294	0.381247	-6.42
265547_at	hypothetical protein similar to hypot	---	AT2G28305	-1.24	-0.31	-1.27	0.246313	0.381247	-6.42
259330_at	hypothetical protein gene model pre	---	AT3G16270	-1.13	-0.18	-1.27	0.246315	0.381247	-6.42
258326_at	unknown protein similar to putative	SOL1	AT3G22760	-1.15	-0.20	-1.27	0.246418	0.381371	-6.42
253671_at	serine threonine-specific receptor p	---	AT4G29990	1.14	0.19	1.27	0.246428	0.381371	-6.42
247968_at	40S ribosomal protein S30 homolog	---	AT5G56670	1.26	0.33	1.27	0.246475	0.381411	-6.42
266618_at	unknown protein	---	AT2G35480	-1.27	-0.35	-1.27	0.246487	0.381411	-6.42
249124_at	disease resistance protein ; suppor	---	AT5G43740	-1.10	-0.14	-1.27	0.246508	0.381417	-6.42
262358_at	mandelonitrile lyase, putative simila	---	AT1G73050	-1.13	-0.17	-1.27	0.246627	0.381575	-6.42
256563_at	unknown protein	RALFL27	AT3G29780	-1.11	-0.15	-1.27	0.246689	0.381645	-6.42
245830_at	hypothetical protein predicted by ge	---	AT1G57790	-1.10	-0.13	-1.27	0.246928	0.381989	-6.43
257500_s_a	putative serine carboxypeptidase s	scpl2	AT1G73300	-1.08	-0.11	-1.27	0.247135	0.382283	-6.43
265894_at	putative lipid transfer protein	LTP	AT2G15050	-1.32	-0.40	-1.27	0.247175	0.382319	-6.43
261657_at	pathogenesis-related protein 1b pr	---	AT1G50050	-1.11	-0.15	-1.27	0.247358	0.382577	-6.43
262839_at	PhD-finger protein, putative contai	---	AT1G14770	1.19	0.25	1.27	0.247408	0.382628	-6.43
247967_at	putative protein similar to unknown	anac099	AT5G56620	-1.08	-0.12	-1.27	0.247453	0.382664	-6.43
264518_at	unknown protein	---	AT1G09980	-1.15	-0.20	-1.27	0.247465	0.382664	-6.43
256012_at	unknown protein similar to dimethyl	FMO1	AT1G19250	2.29	1.20	1.27	0.247555	0.382777	-6.43
251232_at	shock protein SRC2-like shock pro	---	AT3G62780	-1.38	-0.47	-1.27	0.247719	0.383005	-6.43
257364_at	hypothetical protein predicted by ge	---	AT2G45940	-1.08	-0.12	-1.27	0.247854	0.383188	-6.43
255441_at	putative cullin-like 1 protein similar t	ATCUL1	AT4G02570	1.10	0.14	1.26	0.247954	0.383316	-6.43
248738_at	unknown protein ;supported by full-	---	AT5G48020	-1.11	-0.15	-1.26	0.248001	0.383363	-6.43
254462_at	putative protein ;supported by full-l	---	AT4G20150	1.09	0.12	1.26	0.248102	0.383492	-6.43
259167_at	putative oleosin similar to oleosin G	---	AT3G01570	1.09	0.13	1.26	0.248124	0.383501	-6.43
245625_at	transport protein	---	AT4G14160	1.10	0.14	1.26	0.248176	0.383556	-6.43
266532_at	putative glucosyltransferase ;suppo	---	AT2G16890	-1.67	-0.74	-1.26	0.248203	0.38356	-6.43
263865_at	putative ABC transporter related to	ABCB1	AT2G36910	-1.16	-0.21	-1.26	0.248212	0.38356	-6.43
252226_at	putative protein transcribed gene o	---	AT3G49880	-1.09	-0.13	-1.26	0.248387	0.383789	-6.43
263550_at	Expressed protein ; supported by cl	---	AT2G17033	-1.12	-0.17	-1.26	0.248394	0.383789	-6.43
266105_at	putative transport protein SEC61 be	SEC61 BET	AT2G45070	-1.15	-0.20	-1.26	0.248564	0.384002	-6.43
252067_at	protein phosphatase 2C -like prote	---	AT3G51370	1.12	0.16	1.26	0.248566	0.384002	-6.43
265024_at	Expressed protein ; supported by fu	---	AT1G24600	1.24	0.31	1.26	0.248598	0.384007	-6.43
254662_at	putative protein phospho-N-acetyln	TRANS11	AT4G18270	1.17	0.23	1.26	0.248603	0.384007	-6.43
266046_at	hypothetical protein	---	AT2G07728	-1.14	-0.19	-1.26	0.24879	0.384268	-6.43

257837_at	hypothetical protein predicted by ge	---	AT3G25200	-1.11	-0.16	-1.26	0.248805	0.384268	-6.43
248593_at	pectin methylesterase ;supported b	---	AT5G49180	-1.15	-0.20	-1.26	0.248868	0.384338	-6.43
257945_at	SKP1/ASK1 (At10), putative simila	ASK10	AT3G21860	-1.10	-0.14	-1.26	0.248939	0.384422	-6.43
249620_at	putative protein predicted proteins,	---	AT5G37520	-1.09	-0.13	-1.26	0.24901	0.384462	-6.43
253070_at	putative protein symbiotic ammoniu	---	AT4G37850	-1.11	-0.15	-1.26	0.249011	0.384462	-6.43
266263_at	putative flavonol sulfotransferase	---	AT2G27570	-1.10	-0.14	-1.26	0.249023	0.384462	-6.43
263778_at	putative cytochrome oxidase bioger	OXA1L	AT2G46470	1.12	0.16	1.26	0.249032	0.384462	-6.43
249832_at	disease resistance protein-like	---	AT5G23400	-1.13	-0.18	-1.26	0.249065	0.384487	-6.43
246458_at	putative protein various predicted p	---	AT5G16860	1.12	0.16	1.26	0.249104	0.384521	-6.43
257542_at	hypothetical protein predicted by ge	---	AT3G26050	-1.09	-0.12	-1.26	0.249158	0.384577	-6.43
245666_at	hypothetical protein predicted by ge	---	AT1G28280	1.23	0.29	1.26	0.249174	0.384577	-6.43
250707_at	putative protein similar to unknown	MEE60	AT5G05950	1.15	0.21	1.26	0.249303	0.38473	-6.43
263019_at	trehalose 6-phosphate synthase, p	ATTPS9	AT1G23870	1.34	0.43	1.26	0.249307	0.38473	-6.43
255920_at	putative protein	---	AT5G28590	-1.13	-0.18	-1.26	0.249388	0.384829	-6.43
255159_at	putative polyprotein	---	---	-1.14	-0.18	-1.26	0.249534	0.385028	-6.43
263634_at	putative retroelement gag/pol polyp	---	---	-1.10	-0.14	-1.26	0.249621	0.385137	-6.43
253832_at	Expressed protein ; supported by fu	---	AT4G27654	-1.34	-0.42	-1.26	0.249777	0.385351	-6.44
260300_at	gibberellin 3 beta-hydroxylase ident	GA3OX2	AT1G80340	-1.19	-0.25	-1.26	0.249805	0.385368	-6.44
258672_at	hypothetical protein	---	AT3G08570	-1.12	-0.16	-1.26	0.249851	0.385413	-6.44
251636_at	calcium-dependent protein kinase (	CPK32	AT3G57530	1.39	0.48	1.26	0.249873	0.385421	-6.44
255733_at	unknown protein similar to unknown	---	AT1G25400	-1.19	-0.25	-1.26	0.249915	0.385459	-6.44
264556_at	putative transcription factor contair	MYB61	AT1G09540	-1.08	-0.12	-1.26	0.250052	0.385645	-6.44
252629_at	cytochrome P450 - like protein cyto	---	AT3G44970	-1.09	-0.12	-1.26	0.250119	0.385697	-6.44
257327_at	hypothetical protein	---	---	-1.19	-0.25	-1.26	0.250134	0.385697	-6.44
250016_at	Cu/Zn superoxide dismutase-like pr	CSD3	AT5G18100	1.11	0.15	1.26	0.250143	0.385697	-6.44
257200_at	hypothetical protein predicted by ge	---	---	1.08	0.12	1.26	0.250154	0.385697	-6.44
251734_at	putative protein Centrosome- and (	---	AT3G56270	1.25	0.32	1.26	0.250171	0.385698	-6.44
265159_at	unknown protein similar to EST gb|	---	AT1G31060	-1.12	-0.17	-1.26	0.250211	0.385716	-6.44
261380_at	hypothetical protein predicted by ge	---	AT1G05400	-1.14	-0.19	-1.26	0.250216	0.385716	-6.44
264529_at	CTP synthase-like protein similar to	---	AT1G30820	1.26	0.33	1.26	0.250251	0.385742	-6.44
261093_at	non-LTR reverse transcriptase, put	AT1G6288(	---	-1.13	-0.18	-1.26	0.250284	0.385763	-6.44
261783_at	vacuolar assembly protein vps41, p	---	AT1G08190	-1.12	-0.17	-1.26	0.250298	0.385763	-6.44
257739_at	unknown protein	---	AT3G27470	1.15	0.20	1.26	0.250425	0.385932	-6.44
251745_at	putative protein zinc finger transcri	SZF1	AT3G55980	1.74	0.80	1.26	0.25046	0.385961	-6.44
250606_s_a	pectin methyl-esterase-like protein	PPME1	AT1G69940	1.09	0.13	1.26	0.250625	0.386189	-6.44

256827_at	hypothetical protein contains similar	---	AT3G18570	-1.09	-0.12	-1.26	0.250821	0.386464	-6.44
257620_at	hypothetical protein predicted by ge	---	AT3G24850	-1.12	-0.16	-1.26	0.250858	0.386496	-6.44
263354_at	hypothetical protein predicted by gr	---	AT2G22060	-1.08	-0.11	-1.26	0.250894	0.386525	-6.44
247964_at	profilin-like protein ; supported by fu	PRF3	AT5G56600	-1.16	-0.22	-1.26	0.250935	0.386562	-6.44
258168_at	hypothetical protein predicted by ge	---	AT3G21570	-1.09	-0.12	-1.26	0.25123	0.386973	-6.44
265137_at	F-box protein family, AtFBX10 cont	---	AT1G51290	-1.11	-0.14	-1.26	0.251244	0.386973	-6.44
255715_s_a	hypothetical protein	---	AT4G00320	1.09	0.13	1.26	0.251259	0.386973	-6.44
254569_s_a	hypothetical protein	---	---	-1.09	-0.12	-1.26	0.25127	0.386973	-6.44
260779_at	splicing factor, putative similar to h	---	AT1G14650	1.11	0.15	1.25	0.251315	0.387017	-6.44
251239_at	putative protein several hypothetica	---	AT3G62440	-1.14	-0.19	-1.25	0.25149	0.38726	-6.44
250995_at	dnaK-type molecular chaperone hs	HSC70-1	AT5G02500	-1.15	-0.21	-1.25	0.251607	0.387414	-6.44
257507_at	zinc finger protein, putative similar	---	AT1G29600	1.08	0.11	1.25	0.251836	0.387705	-6.44
264525_at	unknown protein Highly Similar to b	ATBCAT-1	AT1G10060	-1.46	-0.55	-1.25	0.251842	0.387705	-6.44
253008_at	expansin -like protein expansin (G	ATEXPA20	AT4G38210	-1.16	-0.21	-1.25	0.251847	0.387705	-6.44
259306_at	unknown protein ;supported by full-	PNC1	AT3G05290	-1.10	-0.14	-1.25	0.251871	0.387715	-6.44
245969_at	putative protein nuclear gene for ch	---	AT5G19830	-1.20	-0.26	-1.25	0.251897	0.387725	-6.44
251655_at	hypothetical protein	AT3G5715	---	-1.14	-0.19	-1.25	0.251911	0.387725	-6.44
266354_at	putative homeodomain transcription	PFS2	AT2G01500	-1.10	-0.14	-1.25	0.25215	0.388067	-6.44
263178_at	hypothetical protein predicted by ge	---	AT1G05550	-1.12	-0.16	-1.25	0.25224	0.388179	-6.44
263552_x_a	unknown protein proline, tyrosine,	---	AT2G24980	-1.15	-0.21	-1.25	0.25227	0.3882	-6.44
263190_at	mutator-like transposase, putative	---	---	-1.08	-0.11	-1.25	0.252373	0.388331	-6.44
261981_at	unknown protein	---	AT1G33811	-1.38	-0.46	-1.25	0.25249	0.388485	-6.44
245920_at	putative protein	---	---	-1.08	-0.11	-1.25	0.252547	0.388546	-6.44
255965_at	14-3-3 protein (grf14), putative sim	---	AT1G22290	-1.10	-0.13	-1.25	0.252638	0.38866	-6.45
265841_at	putative glycogenin	---	AT2G35710	-1.60	-0.68	-1.25	0.252668	0.388681	-6.45
262823_at	ATP-dependent Clp protease prote	CLPP6	AT1G11750	-1.10	-0.14	-1.25	0.252741	0.388768	-6.45
258352_at	putative auxin-induced protein simil	IAA31	AT3G17600	-1.13	-0.17	-1.25	0.252816	0.388841	-6.45
249262_at	putative protein similar to unknown	---	AT5G41720	-1.10	-0.14	-1.25	0.252831	0.388841	-6.45
267148_at	hypothetical protein predicted by ge	---	AT2G23470	1.13	0.18	1.25	0.252842	0.388841	-6.45
248439_at	putative protein similar to unknown	---	AT5G51250	-1.10	-0.13	-1.25	0.252858	0.388841	-6.45
255909_at	cdc2+/CDC28-related protein kinas	CDKD1;3	AT1G18040	-1.18	-0.23	-1.25	0.253071	0.389135	-6.45
253707_at	hypothetical protein	---	AT4G29200	-1.09	-0.13	-1.25	0.253082	0.389135	-6.45
263983_at	unknown protein ;supported by full-	---	AT2G42780	1.09	0.12	1.25	0.253178	0.38923	-6.45
256295_at	hypothetical protein	---	AT1G69470	-1.10	-0.14	-1.25	0.253179	0.38923	-6.45
264847_at	clathrin assembly protein AP19, sm	AP19	AT2G17380	-1.08	-0.11	-1.25	0.253285	0.389367	-6.45

257030_at	unknown protein	---	---	-1.10	-0.14	-1.25	0.253407	0.389529	-6.45
256552_at	hypothetical protein predicted by ge	---	AT3G31350	-1.12	-0.16	-1.25	0.253479	0.389613	-6.45
259390_at	hypothetical protein predicted by ge	---	AT1G06340	-1.11	-0.15	-1.25	0.253609	0.389786	-6.45
263238_at	putative auxin-induced protein	---	AT2G16580	-1.09	-0.12	-1.25	0.253663	0.389829	-6.45
254548_at	putative protein various predicted p	---	AT4G19865	-1.20	-0.26	-1.25	0.253671	0.389829	-6.45
266220_at	putative dTDP-glucose 4-6-dehydra	---	AT2G28755	1.13	0.18	1.25	0.253737	0.389904	-6.45
258186_s_a	protein kinase, putative similar to s	---	AT1G48490	1.10	0.13	1.25	0.253802	0.389978	-6.45
262427_s_a	thioglucosidase, putative similar to	BGLU34	AT1G47600	-1.09	-0.13	-1.25	0.253844	0.390017	-6.45
248953_at	serine/threonine-protein kinase Mak	---	AT5G45428	-1.10	-0.14	-1.25	0.2539	0.390076	-6.45
258838_at	unknown protein ; supported by cD	---	AT3G07140	1.09	0.13	1.25	0.253994	0.390192	-6.45
255986_x_a	putative protein predicted proteins,	---	---	-1.08	-0.11	-1.25	0.254025	0.390192	-6.45
267418_at	putative RING zinc finger protein	---	AT2G35000	-1.13	-0.17	-1.25	0.254027	0.390192	-6.45
260826_at	hypothetical protein predicted by ge	---	AT1G06710	1.10	0.14	1.25	0.254161	0.390359	-6.45
254532_at	putative protein regulatory protein I	NPR4	AT4G19660	1.18	0.24	1.25	0.254175	0.390359	-6.45
260759_at	hypothetical protein contains similar	---	AT1G49180	-1.19	-0.26	-1.25	0.254188	0.390359	-6.45
252715_x_a	putative protein predicted protein, A	---	AT3G43900	-1.12	-0.16	-1.25	0.254204	0.390359	-6.45
260529_at	putative chloroplast protein CP12 ;	CP12-1	AT2G47400	1.09	0.12	1.25	0.254274	0.390441	-6.45
265537_at	putative retroelement pol polyprotei	---	---	-1.12	-0.16	-1.25	0.254363	0.390549	-6.45
255654_at	Similar to receptor kinase	---	AT4G00970	-1.37	-0.45	-1.25	0.254379	0.390549	-6.45
250623_at	peroxisomal Ca-dependent solute c	---	AT5G07320	-1.10	-0.14	-1.25	0.25441	0.390565	-6.45
253939_at	putative myb-related protein myb-r	MYB97	AT4G26930	-1.15	-0.20	-1.25	0.254424	0.390565	-6.45
246429_at	farnesylated protein ATFP6-like pro	---	AT5G17450	1.29	0.37	1.25	0.254457	0.390586	-6.45
251217_at	Sar1-like GTP binding protein sma	---	AT3G62560	-1.11	-0.16	-1.25	0.254475	0.390586	-6.45
264227_at	hypothetical protein contains similar	ATREV3	AT1G67500	1.10	0.14	1.25	0.254489	0.390586	-6.45
264066_at	Argonaute (AGO1)-like protein	AGO5	AT2G27880	-1.11	-0.15	-1.25	0.254591	0.390717	-6.45
258301_at	hypothetical protein predicted by ge	---	---	1.08	0.11	1.25	0.254618	0.390732	-6.45
245563_at	SNF1 like protein kinase ; supporte	CIPK4	AT4G14580	1.32	0.40	1.25	0.254664	0.390776	-6.45
251006_at	putative protein predicted proteins,	---	AT5G02600	1.12	0.16	1.25	0.25471	0.39082	-6.45
251085_at	putative protein insulinase - Homo	---	AT5G01440	-1.11	-0.15	-1.24	0.25476	0.39083	-6.45
266887_at	unknown protein ; supported by cD	CHL-CPN1	AT2G44650	-1.13	-0.17	-1.24	0.25476	0.39083	-6.45
248940_at	replication protein A1-like	---	AT5G45400	1.12	0.16	1.24	0.254768	0.39083	-6.45
262765_at	putative cytochrome P450 monooxy	CYP86C4	AT1G13150	-1.11	-0.15	-1.24	0.254827	0.390895	-6.45
265747_s_a	hypothetical protein predicted by ge	---	AT2G06570	-1.11	-0.15	-1.24	0.254863	0.390923	-6.45
258401_at	unknown protein ; supported by cD	---	AT3G15430	-1.13	-0.17	-1.24	0.254886	0.390933	-6.45
267291_at	putative C2H2-type zinc finger prote	---	AT2G23740	-1.19	-0.26	-1.24	0.254982	0.391053	-6.45

250037_at	disease resistance protein -like dis	---	AT5G18350	-1.14	-0.19	-1.24	0.25509	0.391193	-6.45
262376_at	unknown protein contains similarity	HTH	AT1G72970	-1.10	-0.13	-1.24	0.255138	0.391215	-6.45
250839_at	MADS-box protein - like SLM3 MA	AGL99	AT5G04640	-1.09	-0.12	-1.24	0.255139	0.391215	-6.45
248133_at	unknown protein	---	AT5G54850	-1.15	-0.21	-1.24	0.255255	0.391368	-6.45
250992_at	expansin precursor - like protein ex	ATEXPA9	AT5G02260	1.28	0.36	1.24	0.255283	0.391372	-6.45
257694_at	nucleolar protein, putative nucleola	---	AT3G12860	1.10	0.13	1.24	0.255301	0.391372	-6.45
254782_at	putative calmodulin calmodulin - C	UNE14	AT4G12860	-1.12	-0.16	-1.24	0.255323	0.391372	-6.45
249367_at	putative protein predicted protein, A	---	AT5G40630	-1.11	-0.15	-1.24	0.255327	0.391372	-6.45
256059_at	hypothetical protein contains similar	---	AT1G06990	-1.13	-0.17	-1.24	0.255396	0.391452	-6.45
259993_at	unknown protein	---	AT1G67960	1.12	0.16	1.24	0.255509	0.391598	-6.45
265921_at	putative C2H2-type zinc finger prote	---	AT2G18490	-1.09	-0.13	-1.24	0.2556	0.391711	-6.46
248157_at	putative protein similar to unknown	---	AT5G54420	-1.10	-0.14	-1.24	0.25581	0.392006	-6.46
267408_at	putative CTP synthase	---	AT2G34890	-1.09	-0.13	-1.24	0.255889	0.392101	-6.46
255896_at	basic blue protein, putative similar	---	AT1G17800	-1.09	-0.12	-1.24	0.255917	0.392118	-6.46
260077_at	thaumatin-like protein contains Pfar	---	AT1G73620	-1.13	-0.18	-1.24	0.255963	0.392156	-6.46
261493_at	DNA-binding protein p24, putative	WHY1	AT1G14410	1.16	0.21	1.24	0.255976	0.392156	-6.46
245197_at	F12A21.7 hypothetical protein;supp	---	AT1G67800	1.10	0.14	1.24	0.256002	0.392161	-6.46
263819_x_a	putative TNP2-like transposon prote	---	---	-1.08	-0.11	-1.24	0.256018	0.392161	-6.46
254289_at	putative protein predicted protein, A	---	AT4G22980	1.53	0.61	1.24	0.256031	0.392161	-6.46
266198_at	unknown protein predicted by gens	---	AT2G38890	-1.10	-0.14	-1.24	0.256048	0.392161	-6.46
249082_at	legumin-like protein	CRA1	AT5G44120	-1.10	-0.14	-1.24	0.256177	0.392332	-6.46
257580_at	hypothetical protein	---	AT3G06210	-1.11	-0.15	-1.24	0.256201	0.392342	-6.46
260419_at	putative protein kinase contains Pfa	---	AT1G69730	1.29	0.37	1.24	0.256284	0.392443	-6.46
249152_s_a	inorganic phosphate transporter (dt	APT1	AT5G43350	1.16	0.22	1.24	0.256454	0.392677	-6.46
255315_at	hypothetical protein	---	---	-1.09	-0.13	-1.24	0.256496	0.392716	-6.46
261899_at	cinnamoyl CoA reductase, putative	CCR2	AT1G80820	1.77	0.82	1.24	0.256518	0.392722	-6.46
257127_at	hypothetical protein predicted by ge	TTN9	AT3G20070	1.13	0.17	1.24	0.256577	0.392786	-6.46
262478_at	hypothetical protein similar to hypot	---	AT1G11170	1.11	0.15	1.24	0.256684	0.392924	-6.46
257184_at	ABC transporter, putative similar to	ATMRP8	AT3G13090	-1.09	-0.13	-1.24	0.256827	0.393117	-6.46
247963_at	beta-1,3-glucanase-like protein	---	AT5G56590	1.10	0.13	1.24	0.256867	0.393141	-6.46
267626_at	putative cytochrome P450	CYP712A1	AT2G42250	-1.17	-0.23	-1.24	0.256878	0.393141	-6.46
264768_at	hypothetical protein similar to putati	---	AT1G61410	-1.10	-0.14	-1.24	0.256929	0.393194	-6.46
246440_at	glycine/proline-rich protein glycine/	---	AT5G17650	-1.23	-0.30	-1.24	0.257001	0.393271	-6.46
256369_at	hypothetical protein predicted by ge	---	AT1G66790	-1.08	-0.11	-1.24	0.257014	0.393271	-6.46
254336_at	aspartic proteinase like protein ory	---	AT4G22050	-1.13	-0.18	-1.24	0.257103	0.393381	-6.46

251248_at	P-glycoprotein-like proetin P-glycop	PGP21	AT3G62150	1.54	0.62	1.24	0.257137	0.393406	-6.46
264508_at	putative phytochrome A similar to G	PHYA	AT1G09570	-1.19	-0.25	-1.24	0.257195	0.393468	-6.46
266476_at	unknown protein ;supported by full-	---	AT2G31090	-1.14	-0.19	-1.24	0.257217	0.393476	-6.46
AFFX-r2-Ec	E. coli /GEN=bioD /DB_XREF=gb:J	---	---	-1.26	-0.33	-1.24	0.257319	0.393606	-6.46
250643_at	putative protein similar to unknown	---	AT5G06780	-1.09	-0.12	-1.24	0.257431	0.393748	-6.46
259701_at	unknown protein similar to GB:AAC	---	AT1G77770	1.13	0.18	1.24	0.257447	0.393748	-6.46
255357_at	putative pectinesterase	---	AT4G03930	-1.09	-0.13	-1.24	0.257505	0.393786	-6.46
260342_at	hypothetical protein predicted by ge	---	AT1G69280	-1.12	-0.16	-1.24	0.257506	0.393786	-6.46
267476_at	putative pectate lyase	---	AT2G02720	-1.10	-0.13	-1.24	0.257551	0.393829	-6.46
254185_at	cellulose synthase catalytic subuni	CSLG3	AT4G23990	-1.17	-0.23	-1.24	0.257611	0.393893	-6.46
251331_s_a	tubulin gamma-1 chain	TUBG1	AT3G61650 ,	1.28	0.36	1.24	0.257627	0.393893	-6.46
256162_at	hypothetical protein similar to hypot	---	AT1G55390	-1.09	-0.12	-1.24	0.257678	0.393944	-6.46
251924_at	histone H4-like protein several histo	---	AT3G53730	1.15	0.21	1.24	0.257786	0.394082	-6.46
250801_at	pectinesterase	---	AT5G04960	1.09	0.13	1.24	0.257821	0.394089	-6.46
251339_at	putative protein various predicted p	---	AT3G60780	-1.14	-0.18	-1.24	0.257825	0.394089	-6.46
266001_at	hypothetical protein predicted by ge	HHP3	AT2G24150	1.09	0.13	1.24	0.258003	0.394319	-6.46
267632_at	hypothetical protein predicted by ge	---	AT2G42160	1.16	0.21	1.24	0.25801	0.394319	-6.46
266358_at	hypothetical protein predicted by ge	---	AT2G32280	1.25	0.32	1.24	0.258042	0.394342	-6.46
245281_at	DEF (CLA1) protein ; supported by	CLA1	AT4G15560	-1.12	-0.16	-1.24	0.25806	0.394342	-6.46
266281_at	putative protein kinase contains a p	---	AT2G29250	-1.12	-0.16	-1.24	0.258101	0.39436	-6.46
257011_at	predicted protein	CAX9	AT3G14070	1.23	0.30	1.24	0.258106	0.39436	-6.46
244913_at	hypothetical protein	---	---	1.11	0.15	1.24	0.258137	0.394381	-6.46
251366_at	putative protein several hypothetica	---	AT3G61340	1.11	0.15	1.24	0.25817	0.394405	-6.46
265376_at	hypothetical protein predicted by ge	---	AT2G05810	-1.11	-0.15	-1.23	0.258448	0.394802	-6.46
264956_at	unknown protein similar to putative	ACR3	AT1G76990	1.31	0.39	1.23	0.258469	0.394809	-6.46
266961_at	Athila retroelement ORF1-like prote	---	---	-1.12	-0.16	-1.23	0.25852	0.394861	-6.46
266830_at	1-aminocyclopropane-1-carboxylate	ACS4	AT2G22810	-1.47	-0.55	-1.23	0.2587	0.395109	-6.47
249323_at	unknown protein	FLA20	AT5G40940	-1.10	-0.14	-1.23	0.258729	0.395126	-6.47
267242_at	hypothetical protein predicted by gr	---	AT2G02670	1.10	0.14	1.23	0.258786	0.395187	-6.47
259268_at	putative lamin similar to blue coppe	---	AT3G01070	-1.08	-0.11	-1.23	0.258812	0.3952	-6.47
266136_at	unknown protein ;supported by full-	---	AT2G45060	1.08	0.11	1.23	0.258893	0.395296	-6.47
251540_at	citrate synthase -like protein citrate	CSY1	AT3G58740	-1.08	-0.11	-1.23	0.258924	0.395296	-6.47
266067_s_a	hypothetical protein predicted by ge	---	AT2G18810 ,	-1.08	-0.11	-1.23	0.258927	0.395296	-6.47
267551_at	ubiquitin-specific protease 1 (UBP1	UBP1	AT2G32780	-1.13	-0.17	-1.23	0.259006	0.395391	-6.47
246009_at	Expressed protein ; supported by cl	ATSTE14B	AT5G08335	1.14	0.19	1.23	0.259112	0.395525	-6.47

257480_at	hypothetical protein similar to putati	---	AT1G15640	-1.20	-0.27	-1.23	0.259172	0.39559	-6.47
252139_at	putative protein hypothetical protei	---	AT3G51050	1.10	0.13	1.23	0.259192	0.395596	-6.47
247974_at	putative protein contains similarity t	---	AT5G56780	-1.09	-0.13	-1.23	0.259252	0.395661	-6.47
254330_at	predicted protein predicted gene F	LBD32	AT4G22700	-1.10	-0.13	-1.23	0.259408	0.395836	-6.47
260562_at	putative protein kinase contains a p	---	AT2G43850	-1.15	-0.20	-1.23	0.259436	0.395836	-6.47
259233_at	hypothetical protein	---	AT3G11380	-1.08	-0.11	-1.23	0.259436	0.395836	-6.47
256313_s_a	RNA-binding protein, putative conta	APUM17	AT1G35850	1.08	0.12	1.23	0.259436	0.395836	-6.47
247152_at	oligopeptidase A ; supported by cD	---	AT5G65620	1.09	0.12	1.23	0.259491	0.395893	-6.47
267150_at	putative anthranilate N-hydroxycinn	SDT	AT2G23510	-1.08	-0.11	-1.23	0.259705	0.396192	-6.47
260240_at	putative tRNA isopentenyl transfera	ATIPT1	AT1G68460	-1.09	-0.13	-1.23	0.259732	0.396208	-6.47
254702_at	putative protein predicted protein, A	---	AT4G17940	-1.11	-0.15	-1.23	0.259779	0.396244	-6.47
255102_s_a	putative MuDR-A-like transposon p	---	---	-1.12	-0.16	-1.23	0.259791	0.396244	-6.47
256916_at	GATA transcription factor 1 (AtGAT	---	AT3G24050	-1.13	-0.18	-1.23	0.259882	0.396357	-6.47
260827_at	mudrA-like protein similar to Mu tra	AT1G0675	---	1.15	0.21	1.23	0.260018	0.396529	-6.47
261632_at	hypothetical protein predicted by ge	---	AT1G50120	1.12	0.16	1.23	0.260045	0.396529	-6.47
258252_at	putative polygalacturonase similar t	---	AT3G15720	1.22	0.29	1.23	0.260047	0.396529	-6.47
248519_at	11-beta-hydroxysteroid dehydrogen	AtHSD7	AT5G50590	-1.11	-0.15	-1.23	0.260089	0.396567	-6.47
257390_x_a	hypothetical protein predicted by ge	---	---	-1.14	-0.19	-1.23	0.260131	0.396604	-6.47
263740_at	hypothetical protein predicted by ge	RALFL14	AT2G20660	-1.13	-0.17	-1.23	0.260233	0.396733	-6.47
247233_at	mitochondrial carrier protein-like	---	AT5G64970	1.12	0.16	1.23	0.260389	0.396912	-6.47
261962_s_a	hypothetical protein predicted by ge	---	---	-1.11	-0.15	-1.23	0.260396	0.396912	-6.47
267105_at	hypothetical protein predicted by ge	---	AT2G14760	-1.13	-0.18	-1.23	0.260403	0.396912	-6.47
246914_at	putative protein various predicted p	---	AT5G25860	-1.14	-0.18	-1.23	0.260511	0.397051	-6.47
250149_at	cinnamoyl CoA reductase - like pro	---	AT5G14700	1.65	0.72	1.23	0.260561	0.397099	-6.47
253573_at	putative protein BEM46 PROTEIN,	---	AT4G31020	-1.12	-0.16	-1.23	0.260599	0.397132	-6.47
260960_at	calcium-binding protein, putative si	---	AT1G05990	-1.11	-0.16	-1.23	0.260646	0.397176	-6.47
266652_at	unknown protein	---	AT2G25760	1.09	0.13	1.23	0.260731	0.397266	-6.47
253553_at	putative protein LipB gene, Prochlo	---	AT4G31050	-1.11	-0.16	-1.23	0.260739	0.397266	-6.47
256473_at	unknown protein	---	---	-1.10	-0.14	-1.23	0.260785	0.397308	-6.47
258169_at	hypothetical protein	---	AT3G21590	-1.11	-0.15	-1.23	0.260833	0.397355	-6.47
263485_at	putative villin ; supported by cDNA	VLN1	AT2G29890	-1.11	-0.14	-1.23	0.260914	0.397452	-6.47
265040_at	G-box binding factor, GBF4 Identic	GBF4	AT1G03970	-1.14	-0.19	-1.23	0.260951	0.397482	-6.47
257979_at	unknown protein	---	AT3G20730	-1.10	-0.14	-1.23	0.260993	0.3975	-6.47
265144_at	putative serine/threonine protein kir	---	AT1G51170	-1.14	-0.19	-1.23	0.260997	0.3975	-6.47
258732_at	putative alkaline/neutral invertase s	---	AT3G05820	-1.10	-0.14	-1.23	0.261166	0.397723	-6.47

255652_at	putative protein	MEE47	AT4G00950	-1.36	-0.44	-1.23	0.261179	0.397723	-6.47
250864_at	putative protein predicted proteins,	---	AT5G03870	-1.11	-0.15	-1.23	0.261204	0.397734	-6.47
262805_at	putative DNA-binding protein simila	ESC	AT1G20900	-1.10	-0.13	-1.23	0.261232	0.397751	-6.47
264734_at	hypothetical protein predicted by ge	SLAH1	AT1G62280	-1.10	-0.13	-1.23	0.261281	0.397766	-6.47
245419_at	putative protein	MSH4	AT4G17380	-1.09	-0.12	-1.23	0.261302	0.397766	-6.47
249492_at	germin-like protein (GLP2a) copy1	GER2	AT5G39130	-1.09	-0.13	-1.23	0.261311	0.397766	-6.47
255539_at	putative actin polymerization factor	CRK	AT4G01710	1.11	0.15	1.23	0.261312	0.397766	-6.47
246827_at	copper binding - like protein mavicy	---	AT5G26330	-1.12	-0.16	-1.23	0.261347	0.397792	-6.47
261635_at	tubulin alpha-6 chain, putative iden	---	AT1G50020	1.14	0.18	1.23	0.261381	0.397819	-6.47
265688_at	putative calmodulin-binding protein	---	AT2G24300	1.08	0.11	1.23	0.261418	0.397823	-6.47
255329_at	hypothetical protein	---	AT4G04370	1.08	0.12	1.23	0.261419	0.397823	-6.47
251433_at	putative protein SERINE/THREON	---	AT3G59830	-1.08	-0.11	-1.23	0.261654	0.398154	-6.47
250286_at	auxin-responsive - like protein Nt-g	PBS3	AT5G13320	1.77	0.83	1.23	0.261711	0.398192	-6.48
265820_at	hypothetical protein predicted by ge	---	AT2G17940	-1.13	-0.18	-1.23	0.261714	0.398192	-6.48
249446_at	receptor protein kinase -like protein	---	AT5G39390	-1.10	-0.13	-1.22	0.261786	0.398255	-6.48
262994_at	hypothetical protein predicted by ge	---	AT1G54420	-1.08	-0.11	-1.22	0.26179	0.398255	-6.48
252155_at	putative protein	---	AT3G50920	1.14	0.18	1.22	0.261817	0.398269	-6.48
261885_at	unknown protein	---	AT1G80930	1.12	0.16	1.22	0.261838	0.398274	-6.48
261836_at	hypothetical protein contains similar	WAKL7	AT1G16090	1.45	0.54	1.22	0.262007	0.398476	-6.48
250642_at	receptor-like protein kinase several	ERL2	AT5G07180	1.08	0.12	1.22	0.262028	0.398476	-6.48
263015_at	hypothetical protein similar to hypot	---	AT1G23440	1.11	0.15	1.22	0.262045	0.398476	-6.48
255659_at	Expressed protein ; supported by fu	---	AT4G00895	1.22	0.29	1.22	0.262045	0.398476	-6.48
265173_at	unknown protein	---	AT1G23530	-1.10	-0.13	-1.22	0.262058	0.398476	-6.48
246635_at	hypothetical protein predicted by ge	---	AT1G31720	-1.16	-0.21	-1.22	0.262116	0.398538	-6.48
267537_at	putative guanylate kinase ; support	GK-1	AT2G41880	1.34	0.42	1.22	0.262183	0.398613	-6.48
262321_at	hypothetical protein predicted by ge	---	AT1G27570	-1.10	-0.14	-1.22	0.262251	0.398691	-6.48
247330_at	putative protein contains similarity t	GAMMA C/	AT5G63510	1.08	0.11	1.22	0.262506	0.39905	-6.48
248120_at	putative protein similar to unknown	---	AT5G54540	1.11	0.15	1.22	0.262546	0.399085	-6.48
246399_at	bZIP transcription factor similar to b	---	AT1G58110	-1.11	-0.15	-1.22	0.262667	0.399237	-6.48
246610_at	putative protein	---	AT5G35400	1.10	0.14	1.22	0.262681	0.399237	-6.48
256429_at	unknown protein	---	AT3G11040	-1.13	-0.18	-1.22	0.262721	0.399271	-6.48
256353_at	unknown protein ; supported by full-	---	AT1G55000	1.10	0.14	1.22	0.262771	0.399293	-6.48
248488_at	putative protein contains similarity t	---	AT5G51080	1.11	0.15	1.22	0.262778	0.399293	-6.48
250699_at	receptor-like protein kinase	SRF2	AT5G06820	-1.11	-0.15	-1.22	0.262802	0.399293	-6.48
252851_at	putative protein CLATHRIN COAT	---	AT4G40080	1.28	0.36	1.22	0.262805	0.399293	-6.48

265706_at	unknown protein	---	AT2G03390	1.14	0.19	1.22	0.262968	0.399514	-6.48
258819_at	unknown protein similar to putative	---	AT3G04590	1.13	0.17	1.22	0.262992	0.399524	-6.48
258781_at	hypothetical protein predicted by ge	---	AT3G11740	-1.08	-0.11	-1.22	0.263107	0.399671	-6.48
252250_at	putative protein predicted protein, A	---	AT3G49790	1.17	0.22	1.22	0.26317	0.399741	-6.48
260910_at	importin alpha, putative similar to ir	IMPA-6	AT1G02690	1.16	0.22	1.22	0.263229	0.399804	-6.48
254205_at	putative protein kinesin heavy chai	---	AT4G24170	-1.12	-0.16	-1.22	0.26325	0.399809	-6.48
258533_at	hypothetical protein	---	AT3G06670	1.11	0.15	1.22	0.263276	0.399822	-6.48
264489_at	putative squamosa-promoter bindin	---	AT1G27370	1.22	0.28	1.22	0.263294	0.399823	-6.48
254552_at	putative protein several hypothetica	---	AT4G19900	1.10	0.14	1.22	0.263416	0.39998	-6.48
261153_at	unknown protein ;supported by full-	---	AT1G04850	1.10	0.14	1.22	0.263433	0.39998	-6.48
256003_at	Ta11 non-LTR retroelement protein	---	AT3G31430	-1.11	-0.15	-1.22	0.263475	0.400018	-6.48
255863_s_a	putative GDSL-motif lipase/hydrolas	---	AT2G30220	-1.09	-0.12	-1.22	0.263543	0.400094	-6.48
258912_at	unknown protein similar to hypothe	---	AT3G06460	-1.15	-0.21	-1.22	0.26362	0.400105	-6.48
257027_at	DNA repair protein, putative similar	ATRAD54	AT3G19210	1.10	0.13	1.22	0.26363	0.400105	-6.48
245546_at	cellulose synthase like protein	ATCSLB05	AT4G15290	-1.16	-0.22	-1.22	0.263635	0.400105	-6.48
261291_at	hypothetical protein predicted by ge	---	AT1G36970	-1.16	-0.22	-1.22	0.263639	0.400105	-6.48
263796_at	unknown protein ;supported by full-	AFR	AT2G24540	-1.18	-0.24	-1.22	0.263654	0.400105	-6.48
255480_at	Expressed protein ; supported by fu	---	AT4G02485	1.09	0.13	1.22	0.263655	0.400105	-6.48
262405_at	hypothetical protein contains similar	HDG10	AT1G34650	-1.11	-0.16	-1.22	0.263903	0.400437	-6.48
248047_at	putative protein similar to unknown	---	AT5G56070	-1.10	-0.13	-1.22	0.263909	0.400437	-6.48
264667_s_a	hypothetical protein identical to hyp	ZW18	AT1G09980	-1.09	-0.13	-1.22	0.264086	0.400678	-6.48
247984_s_a	IAA-amino acid hydrolase homolog	ILL1	AT5G56650	1.15	0.20	1.22	0.264177	0.400791	-6.48
265036_at	unknown protein Contains similarity	PRP39	AT1G04080	1.09	0.13	1.22	0.264203	0.400796	-6.48
250587_at	putative protein contains similarity t	---	AT5G07640	-1.10	-0.13	-1.22	0.264216	0.400796	-6.48
265733_at	unknown protein ;supported by full-	---	AT2G01190	-1.12	-0.16	-1.22	0.264451	0.401126	-6.48
248260_at	putative protein similar to unknown	---	AT5G53240	-1.10	-0.13	-1.22	0.264858	0.401716	-6.49
252043_at	putative protein mttC protein, Esch	---	AT3G52390	1.12	0.16	1.22	0.264961	0.401846	-6.49
260304_at	unknown protein identical to most o	---	AT1G70480	-1.17	-0.23	-1.22	0.265156	0.402115	-6.49
251496_at	putative protein crp1 protein, involv	---	AT3G59040	1.09	0.13	1.22	0.265209	0.402168	-6.49
AFFX-BioC-	E. coli /GEN=bioC /DB_XREF=gb:J	---	---	-1.25	-0.33	-1.22	0.265261	0.40221	-6.49
263336_x_a	hypothetical protein predicted by ge	---	---	-1.09	-0.13	-1.21	0.265271	0.40221	-6.49
263688_at	unknown protein Location of EST 2	---	AT1G26920	-1.38	-0.46	-1.21	0.265334	0.402268	-6.49
266891_at	putative GTP-binding protein ; supp	ARAC9	AT2G44690	-1.12	-0.16	-1.21	0.265345	0.402268	-6.49
248022_at	putative protein contains similarity t	APUM12	AT5G56510	-1.08	-0.11	-1.21	0.265403	0.402316	-6.49
258801_at	unknown protein predicted by gens	---	AT3G04560	1.08	0.11	1.21	0.265412	0.402316	-6.49

258681_at	unknown protein ; supported by ful	---	AT3G08610	1.11	0.15	1.21	0.265513	0.402442	-6.49
253335_at	putative protein YHR077c (NMD2,I	---	AT4G33500	1.11	0.15	1.21	0.265578	0.402514	-6.49
245669_at	hypothetical protein contains similar	LEC2	AT1G28300	-1.13	-0.17	-1.21	0.265652	0.402599	-6.49
249903_at	disease resistance protein-like	---	AT5G22690	-1.36	-0.44	-1.21	0.265714	0.402636	-6.49
247024_at	Expressed protein ; supported by fu	---	AT5G66985	-1.12	-0.17	-1.21	0.265728	0.402636	-6.49
260471_at	hypothetical protein predicted by ge	---	AT1G11070	-1.09	-0.12	-1.21	0.265729	0.402636	-6.49
261737_at	hypothetical protein similar to putati	---	AT1G47885	1.11	0.16	1.21	0.26575	0.402641	-6.49
246128_at	putative protein predicted proteins,	---	---	-1.11	-0.15	-1.21	0.265773	0.402648	-6.49
264451_s_a	putative lectin receptor kinase very	---	---	1.10	0.14	1.21	0.265802	0.402657	-6.49
258266_at	hypothetical protein predicted by ge	---	AT3G15860	-1.13	-0.18	-1.21	0.265813	0.402657	-6.49
265609_at	hypothetical protein predicted by ge	---	AT2G25420	-1.17	-0.22	-1.21	0.265928	0.402803	-6.49
251484_at	putative protein putative protein At2	---	AT3G59640	1.09	0.12	1.21	0.266002	0.402888	-6.49
252279_at	1-aminocyclopropane-1-carboxylat	ACS9	AT3G49700	-1.11	-0.15	-1.21	0.266052	0.402907	-6.49
251140_at	lectin - like protein lectin precursor	---	AT5G01090	-1.11	-0.15	-1.21	0.266061	0.402907	-6.49
252877_at	hypothetical protein supported by fu	---	AT4G39630	-1.11	-0.15	-1.21	0.266084	0.402907	-6.49
267138_s_a	similar to SOR1 from the fungus Ce	PDX1.1	AT2G38210	-1.25	-0.33	-1.21	0.266084	0.402907	-6.49
247749_at	myb-related protein - like myb-relat	MYB119	AT5G58850	-1.13	-0.17	-1.21	0.266232	0.403104	-6.49
248246_at	putative protein contains similarity t	TRY	AT5G53200	-1.11	-0.15	-1.21	0.266307	0.40319	-6.49
262884_at	membrane related protein CP5, pu	CP5	AT1G64720	-1.10	-0.14	-1.21	0.266341	0.403209	-6.49
251521_at	putative protein receptor kinase ho	ACR4	AT3G59420	-1.13	-0.17	-1.21	0.266355	0.403209	-6.49
265507_s_a	Ac-like transposase related to Ac/D	---	---	1.10	0.13	1.21	0.266422	0.403271	-6.49
255804_at	putative protein IB1C3-1 protein, A	---	AT4G10220	-1.12	-0.16	-1.21	0.266439	0.403271	-6.49
249267_at	putative protein contains similarity t	BTI3	AT5G41600	-1.17	-0.23	-1.21	0.266449	0.403271	-6.49
263329_at	hypothetical protein contains C3HC	---	AT2G15260	1.09	0.13	1.21	0.266512	0.40334	-6.49
255550_at	putative raffinose synthase or seed	AtSTS	AT4G01970	-1.09	-0.12	-1.21	0.266555	0.403378	-6.49
261331_at	hypothetical protein predicted by ge	---	---	-1.11	-0.16	-1.21	0.266587	0.403384	-6.49
264165_at	hypothetical protein contains similar	ATNAP11	AT1G65410	1.09	0.12	1.21	0.266594	0.403384	-6.49
247384_at	protein kinase ; supported by cDNA	---	AT5G63370	-1.18	-0.24	-1.21	0.266655	0.403425	-6.49
267438_at	putative GDSL-motif lipase/hydrolas	---	AT2G19050	-1.16	-0.21	-1.21	0.266656	0.403425	-6.49
263014_at	unknown protein similar to hypothet	CAF2	AT1G23400	-1.10	-0.14	-1.21	0.266679	0.403429	-6.49
247599_at	putative protein	---	AT5G60880	-1.20	-0.27	-1.21	0.266695	0.403429	-6.49
257205_at	putative glucosyltransferase similar	UGT88A1	AT3G16520	1.10	0.13	1.21	0.26674	0.403471	-6.49
252002_at	hypothetical protein YIP1 protein, S	---	AT3G52760	-1.10	-0.13	-1.21	0.266904	0.403677	-6.49
267320_at	hypothetical protein predicted by ge	---	AT2G19300	-1.10	-0.14	-1.21	0.266912	0.403677	-6.49
266885_at	putative small GTP-binding protein	RAB6A	AT2G44610	1.12	0.16	1.21	0.266944	0.4037	-6.49

263161_at	myrosinase-associated protein, put	---	AT1G54020	-2.46	-1.30	-1.21	0.267232	0.404108	-6.49
247658_at	actin depolymerizing factor 3 - like	ADF3	AT5G59880	1.08	0.12	1.21	0.267311	0.404201	-6.49
263394_at	hypothetical protein predicted by ge	---	---	-1.10	-0.14	-1.21	0.267353	0.404238	-6.49
266997_at	hypothetical protein predicted by ge	---	AT2G34410	-1.17	-0.22	-1.21	0.267408	0.404277	-6.49
259968_at	unknown protein	---	AT1G76530	-1.11	-0.14	-1.21	0.267419	0.404277	-6.49
262651_at	hypothetical protein One of a cluste	FUT9	AT1G14100	-1.11	-0.16	-1.21	0.267433	0.404277	-6.49
252418_at	hypothetical protein	---	AT3G47490	1.13	0.18	1.21	0.267582	0.404477	-6.49
253802_at	putative protein	---	AT4G28180	-1.10	-0.14	-1.21	0.267673	0.404572	-6.49
251208_at	putative protein amino acid selectiv	ATOEP16-	AT3G62880	1.10	0.14	1.21	0.267695	0.404572	-6.49
251075_at	putative protein leucine-rich recept	---	AT5G01890	-1.23	-0.30	-1.21	0.267699	0.404572	-6.49
266837_x_a	unknown protein	---	AT2G25990	-1.09	-0.12	-1.21	0.267738	0.404587	-6.49
253892_at	hypothetical protein ;supported by	---	AT4G27620	-1.10	-0.13	-1.21	0.267744	0.404587	-6.49
256282_at	unknown protein	---	AT3G12550	-1.12	-0.16	-1.21	0.267822	0.404661	-6.49
245410_at	putative protein	ATMAP70-	AT4G17220	-1.09	-0.13	-1.21	0.267828	0.404661	-6.49
260846_at	unknown protein	---	AT1G17300	-1.10	-0.14	-1.21	0.268023	0.404928	-6.50
248351_at	unknown protein ; supported by cD	---	AT5G52200	-1.17	-0.22	-1.21	0.268101	0.405019	-6.50
262438_at	hypothetical protein identical to hyp	---	AT1G47410	-1.20	-0.26	-1.21	0.268157	0.405076	-6.50
266720_s_a	hypothetical protein predicted by ge	APRR9	AT2G46670	-1.21	-0.27	-1.21	0.268504	0.405573	-6.50
248733_at	unknown protein	ELP1	---	-1.12	-0.16	-1.21	0.268536	0.405577	-6.50
263425_at	putative preprotein translocase SE	EMB2289	AT2G31530	1.12	0.16	1.21	0.268543	0.405577	-6.50
247343_at	unknown protein	---	AT5G63740	-1.09	-0.13	-1.21	0.268567	0.405577	-6.50
254253_at	serine/threonine kinase - like prote	---	AT4G23320	1.34	0.43	1.21	0.268577	0.405577	-6.50
254369_at	putative protein	---	AT4G21720	-1.14	-0.19	-1.21	0.268646	0.405654	-6.50
253347_at	putative protein	---	AT4G33610	-1.09	-0.13	-1.21	0.268677	0.405675	-6.50
247394_at	putative protein predicted proteins,	---	AT5G62860	-1.22	-0.29	-1.21	0.268784	0.405799	-6.50
261328_at	unknown protein	---	AT1G44835	-1.11	-0.15	-1.21	0.268795	0.405799	-6.50
265576_at	unknown protein	CLASP	AT2G20190	-1.09	-0.12	-1.20	0.268951	0.406007	-6.50
264507_at	Expressed protein ; supported by c	NIMIN-3	AT1G09415	-1.14	-0.18	-1.20	0.269041	0.406085	-6.50
263736_at	nucleic acid-binding protein, putativ	---	AT1G60000	1.15	0.20	1.20	0.269067	0.406085	-6.50
257454_at	hypothetical protein predicted by ge	---	AT1G65170	-1.13	-0.18	-1.20	0.269069	0.406085	-6.50
251450_at	squamosa promoter binding protein	SPL12	AT3G60030	-1.12	-0.16	-1.20	0.269075	0.406085	-6.50
256267_at	unknown protein ; supported by cD	---	AT3G12260	1.10	0.13	1.20	0.269105	0.406085	-6.50
263764_at	putative vacuolar proton-ATPase su	VHA-A2	AT2G21410	1.12	0.17	1.20	0.26911	0.406085	-6.50
265976_at	putative Athila retroelement ORF1	---	---	1.10	0.14	1.20	0.269321	0.406362	-6.50
254142_at	putative protein other predicted or	---	AT4G24630	-1.10	-0.14	-1.20	0.269329	0.406362	-6.50

258654_at	unknown protein	---	AT3G09860	1.16	0.22	1.20	0.269372	0.4064	-6.50
259572_at	hypothetical protein	---	AT1G20400	-1.12	-0.16	-1.20	0.26949	0.406552	-6.50
257434_at	hypothetical protein predicted by ge	---	AT2G21740	-1.08	-0.12	-1.20	0.269652	0.406769	-6.50
253944_at	SNF8 like protein SNF8 protein - y	VPS22	AT4G27040	1.10	0.14	1.20	0.269695	0.406808	-6.50
261231_at	unknown protein ; supported by cDI	---	AT1G20110	-1.08	-0.11	-1.20	0.269767	0.406888	-6.50
256410_at	hypothetical protein	---	AT1G66630	-1.07	-0.10	-1.20	0.269841	0.406973	-6.50
258412_at	unknown protein similar to pop3 pe	HS1	AT3G17210	-1.11	-0.15	-1.20	0.269977	0.407152	-6.50
247419_at	unknown protein	---	AT5G63080	1.13	0.18	1.20	0.270033	0.407184	-6.50
258162_at	dehydrogenase, putative similar to	---	AT3G17810	-1.14	-0.19	-1.20	0.270034	0.407184	-6.50
257852_at	unknown protein	---	AT3G12950	1.13	0.17	1.20	0.270073	0.407216	-6.50
259073_at	unknown protein contains zinc fing	---	AT3G02290	-1.20	-0.26	-1.20	0.270199	0.407374	-6.50
250145_at	putative protein predicted protein, A	---	AT5G14690	-1.14	-0.18	-1.20	0.270214	0.407374	-6.50
250373_at	putative protein	---	AT5G11470	-1.08	-0.11	-1.20	0.270268	0.407429	-6.50
246962_s_a	transcription factor-like protein light	BZIP9	AT5G24800	-1.19	-0.25	-1.20	0.270296	0.407445	-6.50
253377_at	putative protein NBS/LRR disease	ADR1-L1	AT4G33300	1.11	0.15	1.20	0.270323	0.407459	-6.50
266107_at	hypothetical protein predicted by ge	---	AT2G37910	-1.16	-0.21	-1.20	0.270362	0.40749	-6.50
256504_at	cytosolic IMP-GMP specific 5'-nucl	---	AT1G75210	-1.10	-0.14	-1.20	0.270479	0.407639	-6.50
251547_at	putative protein predicted proteins,	---	AT3G58860	-1.10	-0.14	-1.20	0.270504	0.40765	-6.50
249449_at	unknown protein	---	AT5G39430	1.08	0.11	1.20	0.270561	0.40771	-6.50
255470_at	hypothetical protein	---	AT4G03040	-1.11	-0.15	-1.20	0.270612	0.407753	-6.50
259691_at	hypothetical protein predicted by ge	---	AT1G63200	-1.15	-0.20	-1.20	0.270626	0.407753	-6.50
255032_at	putative protein anthocyanin rhamn	---	AT4G09500	1.13	0.18	1.20	0.270765	0.407936	-6.50
266430_at	unknown protein	---	AT2G07200	-1.07	-0.10	-1.20	0.270821	0.407994	-6.50
257393_at	unknown protein	---	AT2G20080	-1.08	-0.11	-1.20	0.270994	0.408227	-6.50
255503_at	contains similarity to a protein kina	---	AT4G02420	-1.41	-0.49	-1.20	0.27106	0.408299	-6.50
255432_at	SYR1-like syntaxin ;supported by f	SYP123	AT4G03330	-1.09	-0.13	-1.20	0.2711	0.408333	-6.50
262874_at	hypothetical protein predicted by ge	---	AT1G65020	-1.13	-0.17	-1.20	0.27114	0.408365	-6.51
264385_at	unknown protein	---	AT1G12020	-1.16	-0.21	-1.20	0.271275	0.408542	-6.51
248829_at	putative protein contains similarity t	---	AT5G47130	-1.16	-0.22	-1.20	0.27135	0.408629	-6.51
251622_at	dnaJ-like protein DNAJ protein - Sc	---	AT3G57340	-1.09	-0.12	-1.20	0.271444	0.408742	-6.51
264630_at	hypothetical protein predicted by ge	FAS1	AT1G65470	-1.13	-0.17	-1.20	0.27153	0.408846	-6.51
266760_at	putative RAV-like B3 domain DNA b	NGA1	AT2G46870	-1.13	-0.18	-1.20	0.271576	0.408887	-6.51
264032_at	unknown protein	GEK1	AT2G03800	1.11	0.15	1.20	0.271655	0.408979	-6.51
262013_s_a	mudrA-like protein similar to Mu tra	---	---	-1.13	-0.17	-1.20	0.271776	0.409134	-6.51
260528_at	putative WRKY-type DNA binding p	WRKY23	AT2G47260	-1.26	-0.33	-1.20	0.271921	0.409326	-6.51

254794_at	putative protein	---	AT4G12970	-1.39	-0.48	-1.20	0.272041	0.40948	-6.51
263282_at	Expressed protein ; supported by fu	---	AT2G14095	-1.10	-0.14	-1.20	0.272251	0.409768	-6.51
248343_at	putative protein contains similarity t	AtMYB19	AT5G52260	-1.08	-0.11	-1.20	0.272274	0.409777	-6.51
254928_at	putative protein hypothetical protei	---	AT4G11410	1.10	0.14	1.20	0.272335	0.409842	-6.51
247473_at	putative protein AT-hook protein 2,	---	AT5G62260	-1.14	-0.18	-1.20	0.272494	0.410042	-6.51
267312_at	unknown protein	---	AT2G34750	1.13	0.18	1.19	0.272506	0.410042	-6.51
251482_s_a	receptor lectin kinase 3	---	AT3G59740	1.11	0.15	1.19	0.272522	0.410042	-6.51
257301_at	hypothetical protein predicted by ge	---	---	-1.09	-0.12	-1.19	0.272609	0.410129	-6.51
258065_at	putative pyruvate kinase similar to	---	AT3G25960	-1.11	-0.16	-1.19	0.272616	0.410129	-6.51
266492_at	putative protein kinase contains a p	---	AT2G07020	-1.11	-0.15	-1.19	0.272671	0.410184	-6.51
255456_at	hypothetical protein ;supported by f	---	AT4G02920	1.10	0.14	1.19	0.272786	0.410331	-6.51
264784_s_a	unknown protein similar to Arabido	---	AT2G17830	-1.11	-0.15	-1.19	0.272857	0.41039	-6.51
251531_at	putative protein hypothetical protei	---	AT3G58550	-1.11	-0.15	-1.19	0.272862	0.41039	-6.51
247669_at	putative protein transcriptional repr	---	AT5G60170	1.13	0.18	1.19	0.272927	0.410461	-6.51
263829_at	unknown protein	---	AT2G40435	-1.31	-0.39	-1.19	0.273124	0.41073	-6.51
265059_at	unknown protein ; supported by cDI	AR791	AT1G52080	1.14	0.19	1.19	0.27328	0.410938	-6.51
256750_at	unknown protein contains Pfam pro	---	AT3G27150	-1.13	-0.18	-1.19	0.273315	0.410963	-6.51
255835_at	unknown protein	---	AT2G33420	-1.10	-0.14	-1.19	0.273378	0.411031	-6.51
AFFX-r2-Ec	E. coli /GEN=bioB /DB_XREF=gb:J	---	---	-1.20	-0.27	-1.19	0.273494	0.411178	-6.51
251261_at	putative protein polygalacturonase	---	AT3G62110	-1.23	-0.30	-1.19	0.273634	0.411362	-6.51
264541_at	hypothetical protein predicted by ge	---	AT1G55660	-1.12	-0.16	-1.19	0.27366	0.411374	-6.51
262705_at	putative wall-associated kinase sim	---	AT1G16260	-1.42	-0.50	-1.19	0.273749	0.41148	-6.51
259146_at	putative glycerol-3-phosphate dehy	SDP6	AT3G10370	1.13	0.18	1.19	0.273828	0.411572	-6.51
264111_at	hypothetical protein predicted by ge	---	AT2G13690	-1.08	-0.12	-1.19	0.274081	0.411921	-6.51
261349_at	Expressed protein ; supported by fu	AT1G7961	---	-1.08	-0.12	-1.19	0.274117	0.411921	-6.51
249242_at	alcohol dehydrogenase	---	AT5G42250	1.12	0.17	1.19	0.274121	0.411921	-6.51
262440_at	serpin, putative similar to serpin GE	---	AT1G47710	1.15	0.20	1.19	0.274132	0.411921	-6.51
264194_at	putative wall-associated kinase con	---	AT1G22720	-1.10	-0.13	-1.19	0.274153	0.411925	-6.51
255314_at	putative transposon protein	---	---	1.11	0.15	1.19	0.274215	0.411991	-6.51
260506_at	Cyclin, putative similar to GB:CAB7	CYCA3;2	AT1G47210	-1.21	-0.27	-1.19	0.27428	0.412062	-6.51
251189_at	putative protein putative mitochond	---	AT3G62650	1.14	0.19	1.19	0.274305	0.412071	-6.51
250264_at	glucosyltransferase -like protein glu	---	AT5G12890	1.08	0.10	1.19	0.274323	0.412071	-6.51
262061_at	hypothetical protein contains similar	ATPP2-B1	AT1G80110	1.10	0.14	1.19	0.274436	0.412213	-6.52
265085_at	hypothetical protein predicted by ge	TPX2	AT1G03780	-1.15	-0.20	-1.19	0.274478	0.412244	-6.52
252815_at	hypothetical protein	---	---	-1.12	-0.16	-1.19	0.274492	0.412244	-6.52

256987_at	hypothetical protein predicted by ge	---	AT3G28560	-1.09	-0.13	-1.19	0.27457	0.412333	-6.52
257541_at	hypothetical protein predicted by ge	---	AT3G25950	-1.25	-0.32	-1.19	0.274673	0.412434	-6.52
253127_at	putative Myc-type transcription fact	---	AT4G36060	-1.09	-0.12	-1.19	0.27468	0.412434	-6.52
257617_at	hypothetical protein	---	AT3G26550	-1.09	-0.13	-1.19	0.274691	0.412434	-6.52
254610_at	putative protein predicted protein, A	---	AT4G18890	1.10	0.14	1.19	0.274861	0.412662	-6.52
267422_at	putative protein kinase contains a p	---	AT2G35050	-1.08	-0.12	-1.19	0.274926	0.412733	-6.52
249803_at	putative protein similar to unknown	---	AT5G23780	-1.10	-0.14	-1.19	0.274969	0.41277	-6.52
253156_at	putative protein mRNA for KIAA01	---	AT4G35730	-1.11	-0.15	-1.19	0.275021	0.412789	-6.52
252731_at	putative protein predicted proteins,	---	---	-1.14	-0.18	-1.19	0.275035	0.412789	-6.52
257015_at	hypothetical protein predicted by ge	---	AT3G28680	-1.11	-0.15	-1.19	0.275036	0.412789	-6.52
263988_at	floral homeodomain transcription fa	SHP2	AT2G42830	-1.09	-0.13	-1.19	0.275082	0.41282	-6.52
265493_at	unknown protein predicted by gens	---	AT2G15670	-1.10	-0.13	-1.19	0.275093	0.41282	-6.52
251877_at	synaptobrevin -like protein vesicle-	ATVAMP72	AT3G54300	-1.12	-0.16	-1.19	0.275152	0.412882	-6.52
260232_at	unknown protein contains Pfam pro	---	AT1G74640	-1.19	-0.25	-1.19	0.275181	0.412898	-6.52
249854_at	putative protein contains similarity t	---	AT5G22960	-1.12	-0.16	-1.19	0.275237	0.412945	-6.52
255981_at	hypothetical protein ;supported by f	---	AT1G34020	-1.12	-0.16	-1.19	0.275264	0.412945	-6.52
251512_at	putative protein several hypothetica	---	AT3G59190	-1.10	-0.13	-1.19	0.275267	0.412945	-6.52
264536_at	hypothetical protein predicted by ge	PRA1.G1	AT1G55640	-1.16	-0.21	-1.19	0.275331	0.412982	-6.52
251473_at	putative protein myrosinase-binding	---	AT3G59610	-1.14	-0.19	-1.19	0.275343	0.412982	-6.52
254097_at	putative Ser/Thr protein kinase ser	---	AT4G25160	1.08	0.11	1.19	0.275346	0.412982	-6.52
249840_at	unknown protein ; supported by cD	ATLCBK1	AT5G23450	-1.08	-0.11	-1.19	0.275379	0.413005	-6.52
264350_at	putative seryl-tRNA synthetase sim	SRS	AT1G11870	-1.16	-0.21	-1.19	0.275421	0.413042	-6.52
262982_at	unknown protein ;supported by full-	ING2	AT1G54390	-1.08	-0.12	-1.19	0.275494	0.413124	-6.52
253243_at	putative protein	---	AT4G34560	1.45	0.53	1.19	0.275561	0.413197	-6.52
265883_at	unknown protein	---	AT2G42310	1.09	0.13	1.19	0.275634	0.413279	-6.52
260718_at	unknown protein	ECT7	AT1G48110	1.08	0.10	1.19	0.275655	0.413283	-6.52
253159_at	HMG delta protein ;supported by fu	HMGB5	AT4G35570	1.09	0.12	1.19	0.275703	0.413328	-6.52
255307_at	hypothetical protein similar to A. th	RIC10	AT4G04900	-1.11	-0.15	-1.19	0.275733	0.413346	-6.52
255416_at	hypothetical protein	---	AT4G03170	1.08	0.11	1.19	0.2758	0.413357	-6.52
255755_s_a	polygalacturonase, putative similar	---	AT1G43050	-1.10	-0.14	-1.19	0.275801	0.413357	-6.52
247707_at	scarecrow-like 11 - like scarecrow-	---	AT5G59450	1.49	0.58	1.19	0.275816	0.413357	-6.52
253448_at	putative protein mRNA for KIAA00	---	AT4G32640	1.08	0.11	1.19	0.275816	0.413357	-6.52
259048_at	DegP protease contains similarity to	DegP7	AT3G03380	-1.10	-0.14	-1.19	0.275831	0.413357	-6.52
252597_at	putative protein several hypothetica	---	---	-1.12	-0.16	-1.19	0.27585	0.413359	-6.52
245582_at	hypothetical protein	LOI1	AT4G14850	1.14	0.19	1.19	0.27592	0.413436	-6.52

250417_at	putative protein predicted proteins,	---	AT5G11230	-1.12	-0.17	-1.19	0.275961	0.413471	-6.52
254774_at	hypothetical protein	---	AT4G13440	-1.18	-0.24	-1.19	0.276146	0.41372	-6.52
264473_at	hypothetical protein predicted by ge	---	AT1G67180	-1.20	-0.26	-1.18	0.276285	0.413902	-6.52
263300_at	Mutator-like transposase similar to	---	---	-1.08	-0.11	-1.18	0.276313	0.413904	-6.52
259242_at	putative actin similar to actin 1 GB:I	ATARP6	AT3G33520	1.09	0.13	1.18	0.276323	0.413904	-6.52
256135_at	hypothetical protein predicted by ge	---	AT1G48730	-1.11	-0.15	-1.18	0.27635	0.413917	-6.52
262860_at	unknown protein	APO1	AT1G64810	1.13	0.18	1.18	0.276421	0.413996	-6.52
252386_at	putative protein various predicted p	---	AT3G47840	1.13	0.17	1.18	0.276464	0.41402	-6.52
257277_at	disease resistance protein, putative	---	AT3G14470	-1.11	-0.16	-1.18	0.276491	0.41402	-6.52
258920_at	class 2 non-symbiotic hemoglobin i	---	AT1G04380	-1.22	-0.28	-1.18	0.276491	0.41402	-6.52
251227_at	ABC transporter-like protein glutath	ATMRP10	AT3G62700	-1.18	-0.24	-1.18	0.27659	0.41414	-6.52
254749_at	putative protein various predicted p	---	AT4G13130	-1.07	-0.10	-1.18	0.276646	0.414198	-6.52
253677_at	cytidine deaminase - like protein cy	---	AT4G29580	-1.12	-0.16	-1.18	0.276699	0.41424	-6.52
267560_at	putative cytochrome P450	CYP76C3	AT2G45580	-1.11	-0.15	-1.18	0.276714	0.41424	-6.52
253663_at	putative villin	VLN4	AT4G30160	-1.10	-0.14	-1.18	0.276729	0.41424	-6.52
254856_at	putative ribosomal protein (fragmer	---	AT4G12160	1.10	0.14	1.18	0.276752	0.414245	-6.52
263650_at	hypothetical protein predicted by ge	---	AT1G04360	-1.10	-0.14	-1.18	0.276769	0.414245	-6.52
263923_at	Expressed protein ; supported by cI	---	AT2G36485	1.14	0.19	1.18	0.276805	0.414249	-6.52
264598_at	putative dimethylaniline monooxyge	---	AT1G04610	-1.09	-0.12	-1.18	0.276807	0.414249	-6.52
245160_at	putative leucine-rich repeat disease	AtRLP28	AT2G33080	-1.08	-0.11	-1.18	0.276942	0.414405	-6.52
256330_at	hypothetical protein predicted by ge	---	AT1G76870	1.13	0.18	1.18	0.27696	0.414405	-6.52
245613_at	hypothetical protein	---	AT4G14450	1.65	0.73	1.18	0.276967	0.414405	-6.52
260200_at	unknown protein ; supported by ful	---	AT1G67620	1.10	0.14	1.18	0.276988	0.414411	-6.52
255034_at	putative protein Phaseolus vulgaris	---	---	-1.09	-0.13	-1.18	0.277059	0.414482	-6.52
248384_at	mandelonitrile lyase-like protein	---	AT5G51930	-1.09	-0.12	-1.18	0.277073	0.414482	-6.52
250150_at	putative protein ;supported by full-le	---	AT5G14710	1.15	0.20	1.18	0.277233	0.414695	-6.52
261671_at	hypothetical protein similar to hypot	---	AT1G18340	1.17	0.23	1.18	0.27735	0.414802	-6.52
267294_at	hypothetical protein predicted by ge	YCF37	AT2G23670	-1.14	-0.19	-1.18	0.277358	0.414802	-6.52
264291_at	hypothetical protein contains similar	---	AT1G78800	1.11	0.15	1.18	0.277359	0.414802	-6.52
257732_at	unknown protein contains Pfam pro	AtCASP	AT3G18480	1.10	0.14	1.18	0.277383	0.414806	-6.52
261997_at	hypothetical protein similar C-term c	---	---	-1.09	-0.12	-1.18	0.277398	0.414806	-6.52
258943_at	hypothetical protein predicted by ge	---	AT3G10400	1.11	0.15	1.18	0.277456	0.414865	-6.52
252795_at	putative protein predicted proteins,	---	---	-1.09	-0.13	-1.18	0.27752	0.414934	-6.52
257142_at	cytochrome P450, putative contain	CYP705A1	AT3G20090	1.13	0.18	1.18	0.277639	0.415084	-6.52
258487_at	unknown protein ;supported by full-	LBD41	AT3G02550	1.48	0.56	1.18	0.277727	0.415166	-6.53

249987_at	putative protein predicted proteins,	---	AT5G18490	1.17	0.23	1.18	0.277735	0.415166	-6.53
249484_at	cytochrome P450 - like protein cyto	BR6OX1	AT5G38970	-1.09	-0.12	-1.18	0.277748	0.415166	-6.53
246715_at	putative protein glucose-6-phospha	---	AT5G28230	-1.13	-0.18	-1.18	0.277789	0.4152	-6.53
255940_at	prolyl endopeptidase, putative simi	---	AT1G20380	-1.08	-0.12	-1.18	0.277889	0.415322	-6.53
264903_at	putative phosphoglucomutase simi	---	AT1G23190	1.11	0.15	1.18	0.277982	0.415434	-6.53
266613_at	similar to gibberellin-regulated prote	---	AT2G14900	-2.41	-1.27	-1.18	0.278113	0.415602	-6.53
264198_at	unknown protein ESTs gb|Z34075,	---	AT1G22800	-1.14	-0.19	-1.18	0.278154	0.415637	-6.53
264049_at	putative GTP-binding protein	---	AT2G22390	-1.15	-0.20	-1.18	0.278212	0.415697	-6.53
265994_at	putative multispanning membrane p	---	AT2G24170	-1.23	-0.29	-1.18	0.278467	0.416041	-6.53
258516_at	myb-related protein, putative simila	MYB108	AT3G06490	1.35	0.43	1.18	0.278485	0.416041	-6.53
249555_at	putative protein predicted protein, r	---	AT5G38300	-1.10	-0.14	-1.18	0.278498	0.416041	-6.53
248868_at	putative protein similar to unknown	---	AT5G46780	1.24	0.31	1.18	0.278698	0.416314	-6.53
265285_at	hypothetical protein predicted by ge	---	AT2G20410	1.12	0.16	1.18	0.278795	0.416431	-6.53
253614_at	putative protein heat shock protein	---	AT4G30350	1.18	0.24	1.18	0.278816	0.416435	-6.53
245188_at	F12A21.19 unknown protein; simil	---	AT1G67660	1.10	0.14	1.18	0.278901	0.416535	-6.53
252757_at	putative protein several hypothetica	---	---	-1.11	-0.15	-1.18	0.279104	0.416811	-6.53
258843_at	putative protein kinase similar to hy	---	AT3G04690	-1.09	-0.13	-1.18	0.279173	0.416886	-6.53
260335_at	putative strictosidine synthase simil	SS3	AT1G74000	-1.26	-0.34	-1.18	0.279237	0.416955	-6.53
262743_at	unknown protein similar to unknown	---	AT1G29020	-1.11	-0.14	-1.18	0.279266	0.416971	-6.53
255642_at	hypothetical protein	iqd17	AT4G00820	1.17	0.23	1.18	0.279346	0.417063	-6.53
264281_at	hypothetical protein similar to putati	---	AT1G61830	-1.12	-0.16	-1.18	0.279689	0.417528	-6.53
255822_at	putative expansin ;supported by ful	ATEXPA8	AT2G40610	-2.11	-1.08	-1.18	0.279694	0.417528	-6.53
246526_at	putative protein RXF26 - Arabidops	GLIP7	AT5G15720	-1.09	-0.12	-1.18	0.279865	0.417755	-6.53
259425_at	4,5 PIP kinase, putative similar to 4	PIPK11	AT1G01460	1.09	0.12	1.17	0.280062	0.418022	-6.53
258723_at	putative MYB-related protein similar	---	AT3G09600	1.10	0.13	1.17	0.280098	0.418049	-6.53
255910_at	unknown protein contains similarity	---	AT1G18030	-1.10	-0.14	-1.17	0.280218	0.4182	-6.53
246082_at	receptor protein kinase - like protei	EFR	AT5G20480	1.31	0.39	1.17	0.280273	0.418255	-6.53
250612_s_a	putative protein ARR2 protein - Ara	ARR13	AT2G27070	-1.13	-0.18	-1.17	0.280444	0.418483	-6.53
245648_at	F5A9.12 hypothetical protein	---	---	-1.10	-0.13	-1.17	0.280539	0.418598	-6.53
263393_at	hypothetical protein similar to hypot	---	---	-1.09	-0.12	-1.17	0.280582	0.418608	-6.53
266719_at	MYB-related transcription factor (CC	CCA1	AT2G46830	-1.13	-0.17	-1.17	0.280583	0.418608	-6.53
258328_at	putative zinc finger protein similar to	---	AT3G16090	-1.08	-0.11	-1.17	0.280676	0.418719	-6.53
259983_at	3-hydroxy-3-methylglutaryl CoA red	HMG1	AT1G76490	-1.11	-0.15	-1.17	0.280863	0.418971	-6.53
262555_at	hypothetical protein	---	AT1G31400	-1.08	-0.11	-1.17	0.280974	0.419109	-6.53
263146_at	lipase, putative similar to lipase GI:	GLIP2	AT1G53940	-1.12	-0.16	-1.17	0.281014	0.419141	-6.53

248652_at	putative protein contains similarity t	SHV2	AT5G49270	-1.16	-0.21	-1.17	0.281059	0.419181	-6.54
263305_at	unknown protein ;supported by full-	BPC1	AT2G01930	-1.14	-0.19	-1.17	0.281106	0.419224	-6.54
265613_at	putative DnaJ protein ; supported b	---	AT2G25560	1.12	0.16	1.17	0.281247	0.419407	-6.54
259388_at	steroid sulfotransferase, putative si	ST4B	AT1G13420	1.10	0.13	1.17	0.281271	0.419415	-6.54
266573_at	unknown protein	---	AT2G23860	-1.14	-0.18	-1.17	0.281304	0.419437	-6.54
250476_at	MADS box protein FLOWERING LC	FLC	AT5G10140	-1.10	-0.13	-1.17	0.281331	0.41945	-6.54
261820_at	hypothetical protein predicted by ge	---	AT1G11420	1.14	0.19	1.17	0.281386	0.419504	-6.54
247985_at	putative protein contains similarity t	---	AT5G56790	1.11	0.16	1.17	0.281419	0.419526	-6.54
263353_at	hypothetical protein predicted by ge	---	AT2G22070	1.10	0.14	1.17	0.281478	0.419564	-6.54
248288_at	unknown protein ; supported by cD	---	AT5G52840	-1.12	-0.17	-1.17	0.281492	0.419564	-6.54
254183_at	putative protein predicted protein, A	---	AT4G23960	1.11	0.15	1.17	0.2815	0.419564	-6.54
252208_at	putative protein hypothetical protei	---	AT3G50380	1.15	0.20	1.17	0.281625	0.419718	-6.54
248365_at	putative protein strong similarity to u	---	AT5G52500	-1.10	-0.14	-1.17	0.28164	0.419718	-6.54
264182_at	MADS-box protein, putative similar	AGL23	AT1G65360	-1.09	-0.12	-1.17	0.28166	0.419721	-6.54
254885_at	putative protein various predicted p	---	AT4G11745	-1.13	-0.17	-1.17	0.281683	0.419728	-6.54
247185_at	putative protein contains similarity t	---	AT5G65460	-1.12	-0.16	-1.17	0.281725	0.419763	-6.54
261452_s_a	hypothetical protein predicted by ge	---	---	1.10	0.13	1.17	0.281871	0.419952	-6.54
251597_at	protein kinase - like protein wall-as	---	AT3G57750	1.23	0.30	1.17	0.281928	0.419995	-6.54
259504_at	inorganic pyrophosphatase, putativ	AVP1	AT1G15690	-1.12	-0.16	-1.17	0.281936	0.419995	-6.54
251556_at	putative protein predicted proteins,	---	AT3G58840	1.14	0.19	1.17	0.281959	0.420002	-6.54
251739_at	putative protein protein of unknown	CAN	AT3G56170	-1.14	-0.18	-1.17	0.282043	0.420099	-6.54
247946_at	putative protein similar to unknown	CIA2	AT5G57180	1.28	0.36	1.17	0.282116	0.42018	-6.54
252662_at	putative protein Sec24A protein, H	CEF	AT3G44340	-1.10	-0.14	-1.17	0.282196	0.420273	-6.54
267558_at	unknown protein ; supported by cD	LUH	AT2G32700	-1.09	-0.12	-1.17	0.282242	0.420314	-6.54
244986_at	ribosomal protein S19	---	---	-1.16	-0.22	-1.17	0.28229	0.420341	-6.54
261917_at	hypothetical protein similar to TMV	---	AT1G65920	-1.22	-0.29	-1.17	0.282297	0.420341	-6.54
248609_at	unknown protein ; supported by cD	---	AT5G49440	1.11	0.15	1.17	0.28233	0.420348	-6.54
265229_s_a	hypothetical protein	---	AT2G07701	1.09	0.12	1.17	0.282339	0.420348	-6.54
251162_at	putative protein hypothetical protein	---	AT3G63300	-1.09	-0.13	-1.17	0.282502	0.42054	-6.54
247689_at	putative protein protein tyrosine ph	---	AT5G59770	-1.14	-0.19	-1.17	0.282505	0.42054	-6.54
252717_at	putative protein predicted proteins,	---	AT3G43930	1.19	0.25	1.17	0.282623	0.420688	-6.54
247853_at	non phototropic hypocotyl 1-like ; s	PHOT2	AT5G58140	1.11	0.15	1.17	0.282673	0.420735	-6.54
251839_at	putative protein patatin, Solanum tu	PLA IIIA	AT3G54950	1.17	0.22	1.17	0.282712	0.420766	-6.54
253936_at	STIG1 like protein STIG1 protein -	---	AT4G26880	-1.09	-0.13	-1.17	0.282944	0.421077	-6.54
265218_at	putative transcription factor	GTF2H2	AT1G05055	1.08	0.11	1.17	0.282958	0.421077	-6.54

246751_at	pectin methyl-esterase - like protei	---	AT5G27870	-1.08	-0.11	-1.17	0.283071	0.421196	-6.54
252635_at	putative protein predicted proteins,	---	AT3G44510	1.11	0.16	1.17	0.283075	0.421196	-6.54
248623_at	putative protein similar to unknown	---	AT5G49170	-1.36	-0.45	-1.17	0.283123	0.421239	-6.54
261733_at	clathrin coat assembly protein AP1	---	AT1G47830	1.08	0.12	1.17	0.283143	0.421239	-6.54
257503_at	DAG-like protein similar to DAG pr	---	AT1G72530	1.13	0.18	1.17	0.283167	0.421239	-6.54
258135_at	Expressed protein ; supported by fu	---	AT3G24506	-1.10	-0.14	-1.17	0.283195	0.421239	-6.54
261580_at	unknown protein ;supported by full-	IQD18	AT1G01110	-1.16	-0.21	-1.17	0.283208	0.421239	-6.54
253327_at	putative transcription factor	MYB69	AT4G33450	-1.12	-0.17	-1.17	0.283218	0.421239	-6.54
260740_at	thioredoxin, putative contains Pfam	ATQSOX1	AT1G15020	-1.08	-0.11	-1.17	0.283233	0.421239	-6.54
264009_at	putative disease resistance respons	---	AT2G21110	-1.09	-0.13	-1.17	0.28332	0.421318	-6.54
264503_at	hypothetical protein Similar to Arab	---	AT1G09410	-1.09	-0.12	-1.17	0.283323	0.421318	-6.54
265247_at	50S ribosomal protein L3 ;supporte	---	AT2G43030	-1.09	-0.12	-1.17	0.283428	0.421439	-6.54
266258_at	60S acidic ribosomal protein P2 ;su	---	AT2G27720	1.18	0.24	1.17	0.283454	0.421439	-6.54
249592_at	putative protein SIAH1 protein, Bra	---	AT5G37890	-1.12	-0.16	-1.17	0.28346	0.421439	-6.54
249534_at	bZIP transcription factor - like prote	AtbZIP43	AT5G38800	-1.08	-0.12	-1.17	0.283508	0.421476	-6.54
250138_at	DRH1 DEAD box protein - like DRH	---	AT5G14610	-1.09	-0.13	-1.17	0.283522	0.421476	-6.54
263094_at	hypothetical protein predicted by ge	---	AT2G16020	-1.10	-0.14	-1.16	0.283701	0.421716	-6.54
249761_at	acetyl-CoA:benzylalcohol acetyltran	---	AT5G23970	-1.12	-0.17	-1.16	0.28374	0.421746	-6.54
259000_at	hypothetical protein predicted by ge	---	AT3G01860	1.22	0.29	1.16	0.283935	0.422008	-6.54
266733_at	similar to axi 1 protein from Nicotiar	---	AT2G03280	-1.10	-0.13	-1.16	0.283976	0.422018	-6.54
246645_at	putative protein ;supported by full-l	---	AT5G35080	-1.12	-0.16	-1.16	0.283981	0.422018	-6.54
262274_at	deaminase, putative similar to cytid	TADA	AT1G68720	-1.08	-0.11	-1.16	0.283997	0.422018	-6.54
265836_at	Mutator-like transposase similar to	---	---	-1.10	-0.13	-1.16	0.284087	0.422085	-6.54
266429_at	hypothetical protein predicted by ge	---	AT2G07190	-1.10	-0.14	-1.16	0.284098	0.422085	-6.54
257311_at	phosphate transporter, putative cor	PHT2;1	AT3G26570	-1.10	-0.14	-1.16	0.284098	0.422085	-6.54
254093_at	putative protein extensin, Catharan	AtMC2	AT4G25110	-1.29	-0.36	-1.16	0.284193	0.422199	-6.54
247113_at	unknown protein ;supported by full-	---	AT5G65960	-1.10	-0.13	-1.16	0.284238	0.422238	-6.54
249366_at	dihydroxyacetone 3-phosphate redu	---	AT5G40610	1.30	0.37	1.16	0.284291	0.422289	-6.54
260206_at	putative protein kinase contains Pfa	---	AT1G70740	-1.24	-0.31	-1.16	0.284351	0.422351	-6.54
263772_at	Expressed protein ; supported by fu	ELF4-L3	AT2G06255	-1.18	-0.24	-1.16	0.284379	0.422365	-6.54
254203_at	putative protein various predicted p	AtGRF8	AT4G24150	1.09	0.12	1.16	0.284404	0.422374	-6.54
247986_at	unknown protein	---	AT5G56880	-1.12	-0.16	-1.16	0.284464	0.422436	-6.55
252230_at	putative protein various predicted p	---	AT3G49810	1.36	0.44	1.16	0.284617	0.422636	-6.55
258450_at	hypothetical protein predicted by ge	---	---	-1.09	-0.13	-1.16	0.284726	0.42277	-6.55
250615_at	putative protein ankyrins - different	---	AT5G07270	-1.12	-0.16	-1.16	0.284865	0.422919	-6.55

249726_at	unknown protein	---	AT5G35480	1.18	0.24	1.16	0.284874	0.422919	-6.55
255410_at	putative rac GTPase activating pro	---	AT4G03100	-1.10	-0.13	-1.16	0.284894	0.422919	-6.55
265687_s_a	hypothetical protein predicted by ge	---	AT2G24470	-1.09	-0.12	-1.16	0.2849	0.422919	-6.55
245754_at	hypothetical protein predicted by ge	---	AT1G35183	1.08	0.12	1.16	0.284922	0.422924	-6.55
249215_at	dihydroflavonol 4-reductase	DFR	AT5G42800	-1.16	-0.21	-1.16	0.284982	0.422985	-6.55
258668_at	hypothetical protein predicted by ge	---	AT3G08780	-1.09	-0.12	-1.16	0.285019	0.423013	-6.55
266625_at	putative peroxidase	---	AT2G35380	-1.10	-0.13	-1.16	0.285044	0.42302	-6.55
264626_at	unknown protein	AS2	AT1G65620	-1.16	-0.21	-1.16	0.285063	0.42302	-6.55
255320_at	putative transposon protein	---	---	-1.08	-0.11	-1.16	0.285098	0.42302	-6.55
251753_at	putative protein predicted protein, /	---	AT3G55760	-1.25	-0.33	-1.16	0.285101	0.42302	-6.55
248665_at	Expressed protein ; supported by fu	---	AT5G48655	1.08	0.11	1.16	0.285117	0.42302	-6.55
261359_at	hypothetical protein predicted by ge	---	---	-1.10	-0.14	-1.16	0.285191	0.423103	-6.55
247913_at	unknown protein	---	AT5G57510	1.67	0.74	1.16	0.285295	0.423229	-6.55
249202_at	cytochrome P450	CYP705A1	AT5G42580	-1.09	-0.12	-1.16	0.28536	0.423274	-6.55
255738_at	hypothetical protein predicted by ge	---	AT1G25570	-1.09	-0.12	-1.16	0.285368	0.423274	-6.55
264304_at	Expressed protein ; supported by fu	---	AT1G78895	1.12	0.16	1.16	0.285381	0.423274	-6.55
248567_at	putative protein contains similarity t	---	AT5G49750	-1.09	-0.12	-1.16	0.285477	0.42339	-6.55
264998_at	unknown protein similar to hypothet	---	AT1G67330	1.38	0.47	1.16	0.28554	0.423455	-6.55
249332_at	putative protein similar to unknown	---	AT5G40980	-1.12	-0.16	-1.16	0.285569	0.423471	-6.55
261399_at	receptor protein kinase, putative si	---	AT1G79620	-1.12	-0.16	-1.16	0.285588	0.423471	-6.55
245652_at	F-box protein family, putative	---	AT4G13960	-1.11	-0.15	-1.16	0.285642	0.423524	-6.55
258917_at	unknown protein C-terminal portion	---	AT3G10630	1.07	0.10	1.16	0.28568	0.423553	-6.55
262085_at	hypothetical protein predicted by ge	---	AT1G56060	1.53	0.61	1.16	0.285711	0.423571	-6.55
249663_s_a	unknown protein	---	---	-1.12	-0.16	-1.16	0.285765	0.423624	-6.55
249664_at	putative protein histone stem-loop	---	AT5G36722	-1.09	-0.13	-1.16	0.285815	0.423665	-6.55
261001_at	hypothetical protein similar to hypot	---	AT1G26530	1.12	0.16	1.16	0.28583	0.423665	-6.55
245495_at	hypothetical protein	---	AT4G16400	-1.07	-0.10	-1.16	0.285902	0.423745	-6.55
262723_at	hypothetical protein predicted by ge	---	AT1G43630	-1.13	-0.18	-1.16	0.285923	0.423748	-6.55
248596_at	putative protein contains similarity t	AtMYB111	AT5G49330	-1.24	-0.31	-1.16	0.286009	0.423848	-6.55
253437_at	putative protein mRNAs from Ricin	---	AT4G32460	-1.29	-0.37	-1.16	0.286053	0.423885	-6.55
245232_at	actin depolymerizing factor-like pro	ADF7	AT4G25590	-1.10	-0.14	-1.16	0.286117	0.423953	-6.55
249024_at	putative protein strong similarity to u	---	AT5G44860	1.09	0.13	1.16	0.28619	0.424034	-6.55
247195_at	putative protein contains similarity t	---	AT5G65500	-1.12	-0.16	-1.16	0.286262	0.424112	-6.55
254388_at	Expressed protein ; supported by fu	MSRB2	AT4G21860	1.10	0.13	1.16	0.286346	0.424205	-6.55
251302_at	RAV-like protein DNA-binding prote	NGA2	AT3G61970	-1.07	-0.10	-1.16	0.286362	0.424205	-6.55

246998_at	putative protein similar to unknown	---	AT5G67370	1.19	0.26	1.16	0.286395	0.424227	-6.55
259156_at	hypothetical protein predicted by ge	SEC8	AT3G10380	-1.09	-0.13	-1.16	0.286535	0.424407	-6.55
248233_at	F-box protein family, AtFBL13 cont	---	AT5G53840	-1.10	-0.14	-1.16	0.28657	0.424431	-6.55
257331_at	hypothetical protein	---	---	-1.12	-0.16	-1.16	0.286677	0.424561	-6.55
254723_at	ammonium transport protein (AMT1	AMT1;1	AT4G13510	1.14	0.18	1.16	0.286948	0.424906	-6.55
255381_at	RMA1 RING zinc finger protein ider	RMA1	AT4G03510	-1.20	-0.26	-1.16	0.287013	0.424906	-6.55
250304_at	elongation factor 1B alpha-subunit (	---	AT5G12110	-1.33	-0.41	-1.16	0.287016	0.424906	-6.55
255304_at	putative potassium transporter sim	KEA3	AT4G04850	-1.17	-0.23	-1.16	0.28702	0.424906	-6.55
263632_at	Expressed protein ; supported by cl	---	AT2G04795	-1.28	-0.36	-1.16	0.287046	0.424906	-6.55
252897_at	cytochrome P450 - like protein cyto	CYP96A9	AT4G39480	-1.10	-0.13	-1.16	0.287051	0.424906	-6.55
260487_at	RNA-binding protein, putative simil	Y14	AT1G51510	1.09	0.13	1.16	0.287056	0.424906	-6.55
249590_at	putative protein SIAH1, Brassica na	---	AT5G37870	-1.08	-0.12	-1.16	0.287058	0.424906	-6.55
262106_at	hypothetical protein similar to Wee1	WEE1	AT1G02970	-1.12	-0.16	-1.16	0.287213	0.425107	-6.55
264063_at	unknown protein	---	AT2G27900	-1.09	-0.13	-1.16	0.287292	0.425197	-6.55
259494_at	mitotic checkpoint protein, putative	---	AT1G15850	-1.12	-0.16	-1.16	0.287368	0.425282	-6.55
260131_at	unknown protein contains Pfam pro	---	AT1G66310	1.09	0.13	1.16	0.287402	0.425305	-6.55
252599_at	receptor-like protein kinase recepto	---	AT3G45390	-1.13	-0.18	-1.15	0.28745	0.425348	-6.55
251211_s_a	putative protein several hypothetica	---	AT3G62470	1.22	0.28	1.15	0.287551	0.425469	-6.55
247462_at	A9 protein precursor - like A9 prote	---	AT5G62080	-1.11	-0.15	-1.15	0.287616	0.425521	-6.55
254899_at	serine/threonine kinase-like protein	---	AT4G11490	-1.13	-0.17	-1.15	0.287623	0.425521	-6.55
259888_at	hypothetical protein similar to nodul	---	AT1G76350	1.27	0.35	1.15	0.287645	0.425526	-6.55
262498_at	8-oxoguanine DNA glycosylase-like	OGG1	AT1G21710	1.10	0.14	1.15	0.287746	0.425647	-6.55
253870_at	putative protein	---	AT4G27530	-1.10	-0.14	-1.15	0.287921	0.425879	-6.56
267330_at	unknown protein	---	AT2G19270	1.07	0.10	1.15	0.288012	0.425966	-6.56
252399_at	putative cyclic nucleotide-gated cha	CNGC16	AT3G48010	-1.08	-0.12	-1.15	0.288017	0.425966	-6.56
255418_at	predicted protein of unknown functio	---	AT4G03200	1.12	0.17	1.15	0.288084	0.426037	-6.56
249686_at	putative protein contains similarity t	CYP716A2	AT5G36140	-1.07	-0.10	-1.15	0.288253	0.42626	-6.56
259009_at	thioglucosidase 3D precursor identi	PYK10	AT3G09260	-1.17	-0.22	-1.15	0.28834	0.426361	-6.56
247060_at	succinate dehydrogenase flavoprote	SDH1-1	AT5G66760	1.33	0.41	1.15	0.288369	0.426376	-6.56
251439_at	putative protein unknown protein A	---	AT3G59950	1.11	0.15	1.15	0.288419	0.426387	-6.56
248002_at	Expressed protein ; supported by fu	ATARP8	AT5G56180	1.14	0.19	1.15	0.288465	0.426387	-6.56
253650_at	EST GB:N37377 spans last intron a	---	AT4G30020	-1.68	-0.75	-1.15	0.288468	0.426387	-6.56
258068_at	putative DNA-binding protein, GT-1	---	AT3G25990	1.09	0.12	1.15	0.288468	0.426387	-6.56
251363_at	putative transcription factor (MYB17	AtMYB17	AT3G61250	1.17	0.22	1.15	0.28847	0.426387	-6.56
248201_at	putative protein similar to unknown	PTAC15	AT5G54180	1.21	0.28	1.15	0.288537	0.426458	-6.56

258336_at	putative ethylene-inducible protein s	PDX1.2	AT3G16050	1.21	0.28	1.15	0.288716	0.426694	-6.56
245069_at	putative glucosyltransferase	UGT84B2	AT2G23250	-1.10	-0.13	-1.15	0.288788	0.426774	-6.56
261778_at	hypothetical protein predicted by ge	---	AT1G76220	-1.08	-0.10	-1.15	0.288811	0.42678	-6.56
267359_at	hypothetical protein predicted by ge	---	AT2G40020	1.13	0.18	1.15	0.2889	0.426884	-6.56
254876_at	putative phosphoribosylanthranilate	---	AT4G11610	-1.12	-0.17	-1.15	0.28892	0.426885	-6.56
255712_at	hypothetical protein similar to A. tha	---	AT4G00280	1.09	0.13	1.15	0.288952	0.426906	-6.56
248001_at	calcineurin B-like protein 2 (gb|AAC	CBL2	AT5G55990	1.13	0.17	1.15	0.288998	0.426945	-6.56
251181_at	putative protein pectinesterase hor	---	AT3G62820	-1.13	-0.17	-1.15	0.289078	0.427016	-6.56
263861_at	hypothetical protein	---	AT2G04560	-1.15	-0.20	-1.15	0.289085	0.427016	-6.56
258677_at	putative ribosomal-protein S6 kinas	PK1	AT3G08730	-1.11	-0.15	-1.15	0.289102	0.427016	-6.56
258852_at	unknown protein similar to C-termin	AT-P4H-2	AT3G06300	1.14	0.19	1.15	0.289196	0.427127	-6.56
252752_s_a	putative protein hypothetical protei	---	AT3G43470	-1.08	-0.12	-1.15	0.289312	0.427271	-6.56
260384_at	putative 2-isopropylmalate synthase	IMS1	AT1G74040	1.10	0.13	1.15	0.289446	0.427442	-6.56
266042_s_a	hypothetical protein	---	AT2G07734	1.15	0.21	1.15	0.289465	0.427442	-6.56
262069_at	hypothetical protein similar to SNF4	---	AT1G80090	-1.11	-0.15	-1.15	0.289546	0.427511	-6.56
256584_at	unknown protein	---	AT3G28750	-1.09	-0.12	-1.15	0.289549	0.427511	-6.56
252683_at	putative protein predicted proteins,	---	AT3G44380	-1.12	-0.17	-1.15	0.289686	0.427685	-6.56
264129_at	hypothetical protein predicted by ge	---	AT1G79170	-1.11	-0.15	-1.15	0.289739	0.427736	-6.56
255803_at	putative protein vesicle-associated	---	AT4G10170	1.09	0.12	1.15	0.289861	0.42788	-6.56
249981_at	putative protein predicted proteins,	---	AT5G18510	-1.12	-0.17	-1.15	0.289885	0.42788	-6.56
260524_at	unknown protein	---	AT2G47230	-1.09	-0.13	-1.15	0.289909	0.42788	-6.56
255844_at	putative protein kinase contains a p	---	AT2G33580	1.22	0.29	1.15	0.289912	0.42788	-6.56
248723_at	acetyl-CoA:benzylalcohol acetyltrar	---	AT5G47950	-1.10	-0.14	-1.15	0.289944	0.4279	-6.56
260958_at	unknown protein identical to unknov	---	AT1G06060	-1.11	-0.15	-1.15	0.290184	0.428227	-6.56
250712_at	human RAN binding protein 16-like	---	AT5G06120	1.11	0.15	1.15	0.290334	0.42842	-6.56
245720_at	RNA binding protein - like	---	AT5G04210	-1.09	-0.12	-1.15	0.290393	0.428479	-6.56
259638_at	coatomer complex subunit, putative	---	AT1G52360	-1.11	-0.16	-1.15	0.290455	0.428543	-6.56
246915_at	malate dehydrogenase - like protei	ATNADP-M	AT5G25880	1.18	0.24	1.15	0.290477	0.428548	-6.56
251949_at	receptor protein kinase -like protein	---	AT3G53590	-1.12	-0.16	-1.15	0.290638	0.428758	-6.56
245554_at	cytochrome P450 like protein	CYP705A4	AT4G15380	-1.07	-0.10	-1.15	0.290736	0.428874	-6.56
255254_at	hypothetical protein	---	AT4G05030	-1.13	-0.17	-1.15	0.290798	0.428938	-6.56
255671_at	Expressed protein ; supported by fu	---	AT4G00355	1.11	0.15	1.15	0.290834	0.428964	-6.56
266445_at	unknown protein	---	AT2G43070	-1.13	-0.17	-1.15	0.290902	0.429037	-6.56
253735_at	putative protein SNF7 protein - Sac	SNF7.1	AT4G29160	1.09	0.13	1.15	0.290966	0.429103	-6.56
245882_at	mitochondrial carrier-like protein m	DIC3	AT5G09470	1.11	0.15	1.15	0.291056	0.429206	-6.56

267366_at	unknown protein ;supported by full-	---	AT2G44310	1.09	0.13	1.15	0.291073	0.429206	-6.56
247655_at	zinc finger protein Zat12 ;supportec	RHL41	AT5G59820	1.44	0.52	1.15	0.291113	0.429236	-6.56
245553_at	lupeol synthase like protein	BARS1	AT4G15370	-1.08	-0.11	-1.15	0.291182	0.429311	-6.56
259949_at	hypothetical protein predicted by ge	---	AT1G71420	1.10	0.14	1.14	0.291312	0.429474	-6.56
249764_at	septum site-determining MinD (dbj|I	MIND	AT5G24020	1.12	0.17	1.14	0.291379	0.429526	-6.56
265079_at	hypothetical protein contains similar	---	AT1G55460	1.08	0.12	1.14	0.291384	0.429526	-6.56
265786_at	putative replication protein A1	---	---	1.07	0.10	1.14	0.29142	0.42955	-6.57
264184_at	early nodule-specific protein, putati	---	AT1G54790	-1.10	-0.13	-1.14	0.291529	0.429683	-6.57
263945_at	hypothetical protein predicted by ge	---	AT2G36030	-1.13	-0.17	-1.14	0.291615	0.429782	-6.57
248903_at	3-oxoacyl-[acyl-carrier-protein] synt	KAS I	AT5G46290	-1.18	-0.24	-1.14	0.291648	0.429804	-6.57
256490_at	unknown protein ;supported by full-	---	AT1G31460	1.14	0.19	1.14	0.291763	0.429945	-6.57
248323_at	unknown protein	---	AT5G52770	-1.07	-0.10	-1.14	0.291857	0.430054	-6.57
254130_at	MADS-box protein AGL24 ; suppor	AGL24	AT4G24540	1.17	0.23	1.14	0.291875	0.430054	-6.57
247033_at	SKP1 interacting partner 2 (SKIP2)	SKIP2	AT5G67250	-1.11	-0.15	-1.14	0.291932	0.43011	-6.57
254713_at	hypothetical protein	---	AT4G18090	-1.14	-0.18	-1.14	0.292051	0.430257	-6.57
264428_at	hypothetical protein predicted by ge	---	AT1G61790	1.15	0.20	1.14	0.292112	0.430294	-6.57
261184_at	pol polyprotein, putative similar to r	---	---	-1.11	-0.15	-1.14	0.292113	0.430294	-6.57
249541_at	anthranilate N-benzoyltransferase	---	AT5G38130	-1.10	-0.14	-1.14	0.292197	0.430321	-6.57
265476_at	putative thioredoxin M ; supported	ATHM3	AT2G15570	1.08	0.11	1.14	0.292225	0.430321	-6.57
256339_at	hypothetical protein	---	AT1G72080	-1.09	-0.12	-1.14	0.292258	0.430321	-6.57
255145_at	hypothetical protein	---	---	-1.11	-0.15	-1.14	0.29226	0.430321	-6.57
253800_at	putative protein probable cell wall p	---	AT4G28160	-1.11	-0.15	-1.14	0.292267	0.430321	-6.57
252753_at	hypothetical protein	---	AT3G43500	-1.10	-0.14	-1.14	0.292272	0.430321	-6.57
253151_at	putative polygalacturonase polygal	---	AT4G35670	-1.08	-0.11	-1.14	0.292295	0.430321	-6.57
265034_at	unknown protein ;supported by full-	---	AT1G61660	1.11	0.15	1.14	0.292323	0.430321	-6.57
258760_at	putative membrane protein similar	---	AT3G10780	1.19	0.25	1.14	0.292349	0.430321	-6.57
260885_at	protein kinase PK4, putative simila	CIPK18	AT1G29230	-1.12	-0.16	-1.14	0.292349	0.430321	-6.57
246054_at	putative protein predicted proteins,	---	AT5G08360	-1.09	-0.12	-1.14	0.292352	0.430321	-6.57
265393_at	unknown protein ; supported by cD	---	AT2G20830	-1.17	-0.23	-1.14	0.292358	0.430321	-6.57
263008_at	unknown protein	RPP27	AT1G54470	-1.08	-0.11	-1.14	0.2924	0.430355	-6.57
263360_at	unknown protein	---	AT2G03830	1.10	0.13	1.14	0.292451	0.430403	-6.57
258674_at	putative elongation factor P (EF-P)	---	AT3G08740	-1.16	-0.21	-1.14	0.292502	0.43045	-6.57
257560_at	galactosyltransferase, putative con	---	AT3G14960	1.08	0.11	1.14	0.292568	0.430519	-6.57
262773_at	putative nuclear matrix constituent p	LINC2	AT1G13220	-1.11	-0.15	-1.14	0.29262	0.430568	-6.57
265745_at	hypothetical protein predicted by ge	---	---	-1.08	-0.11	-1.14	0.292712	0.430676	-6.57

254888_at	putative protein predicted protein, A	---	AT4G11780	-1.09	-0.13	-1.14	0.292737	0.430684	-6.57
257094_at	hypothetical protein similar to tetraa	---	AT3G20480	1.15	0.20	1.14	0.292911	0.430907	-6.57
253352_at	pathogenesis-related protein 1 pred	---	AT4G33710	-1.11	-0.15	-1.14	0.292926	0.430907	-6.57
262077_at	dynamin-like protein similar to dyna	ADL3	AT1G59610	1.09	0.12	1.14	0.293051	0.431063	-6.57
263500_s_a	hypothetical protein	---	AT2G07672	1.11	0.15	1.14	0.293284	0.431377	-6.57
250915_at	homeodomain -like protein homeod	HB51	AT5G03790	-1.16	-0.21	-1.14	0.29337	0.431457	-6.57
252224_at	putative protein various ADP-RIBOS	ATARLA1B	AT3G49860	-1.11	-0.15	-1.14	0.293376	0.431457	-6.57
255281_at	contains similarity to glucan synthas	GSL1	AT4G04970	1.07	0.10	1.14	0.293398	0.431462	-6.57
267443_at	hypothetical protein predicted by ge	---	AT2G19000	-1.07	-0.10	-1.14	0.293428	0.431478	-6.57
257256_at	hypothetical protein contains Pfam	---	AT3G21970	-1.07	-0.10	-1.14	0.293459	0.431496	-6.57
255350_at	putative transposon protein similar t	---	---	1.08	0.11	1.14	0.293522	0.431561	-6.57
246615_at	putative protein similar to unknown	---	---	-1.10	-0.14	-1.14	0.293705	0.431789	-6.57
247629_at	putative protein	SIZ1	AT5G60410	-1.11	-0.15	-1.14	0.293715	0.431789	-6.57
247577_at	putative protein flavin-containing m	---	AT5G61290	1.10	0.14	1.14	0.293789	0.43187	-6.57
264710_at	putative phytochelatin synthetase s	COBL6	AT1G09790	-1.09	-0.13	-1.14	0.293888	0.431988	-6.57
246329_at	putative protein gamma-tubulin inte	---	AT3G43610	-1.11	-0.15	-1.14	0.294025	0.432161	-6.57
265935_at	putative senescence-associated pro	TET2	AT2G19580	-1.16	-0.22	-1.14	0.294159	0.432323	-6.57
264166_at	unknown protein ; supported by cDN	---	AT1G65370	-1.10	-0.14	-1.14	0.294173	0.432323	-6.57
262363_at	disease resistance protein, putative	---	AT1G72850	-1.09	-0.13	-1.14	0.29422	0.432365	-6.57
262099_s_a	auxin-regulated protein GH3, putat	GH3.4	AT1G59500	1.66	0.73	1.14	0.294249	0.43238	-6.57
250005_at	putative protein predicted proteins,	---	AT5G18760	1.08	0.12	1.14	0.2943	0.432427	-6.57
250814_at	E2, ubiquitin-conjugating enzyme,	UBC22	AT5G05080	1.11	0.15	1.14	0.294375	0.432508	-6.57
259061_at	putative GTP-binding protein simila	AtRABA5b	AT3G07410	1.20	0.26	1.14	0.29447	0.432599	-6.57
252988_at	putative cold-regulated protein cold	---	AT4G38410	-1.19	-0.25	-1.14	0.294475	0.432599	-6.57
258825_at	unknown protein	---	AT3G07180	1.08	0.11	1.14	0.294501	0.432609	-6.57
253346_at	hypothetical protein	---	AT4G33600	-1.09	-0.12	-1.14	0.29476	0.432962	-6.57
249638_at	acetyl-CoA synthetase ; supported	---	AT5G36880	1.26	0.33	1.14	0.294822	0.433011	-6.57
265632_at	hypothetical protein predicted by ge	---	AT2G14290	1.67	0.74	1.14	0.294831	0.433011	-6.57
248262_at	Avr9 elicitor response protein-like ;	---	AT5G53340	-1.13	-0.18	-1.14	0.294892	0.433073	-6.57
265869_at	putative two-component response r	ARR14	AT2G01760	-1.13	-0.17	-1.14	0.294929	0.433099	-6.57
252487_at	glucosyltransferase-like protein UD	UGT76E12	AT3G46660	1.51	0.60	1.14	0.294968	0.433128	-6.58
249514_at	14-3-3 protein GF14psi (grf3/RCI1)	GRF3	AT5G38480	1.11	0.14	1.14	0.295017	0.433173	-6.58
258932_at	hypothetical protein predicted by ge	PAP16	AT3G10150	1.09	0.12	1.14	0.295146	0.433334	-6.58
245875_at	hypothetical protein predicted by ge	---	AT1G26240	-1.12	-0.16	-1.14	0.295167	0.433336	-6.58
247943_at	light-inducible protein ATLS1-like ;s	---	AT5G57170	1.17	0.23	1.13	0.295293	0.433478	-6.58

266886_at	Expressed protein ; supported by fu	---	AT2G44745	-1.11	-0.15	-1.13	0.295334	0.433478	-6.58
259301_at	hypothetical protein predicted by ge	---	AT3G05110	1.13	0.18	1.13	0.295344	0.433478	-6.58
248808_at	putative protein contains similarity t	---	AT5G47510	-1.07	-0.10	-1.13	0.295357	0.433478	-6.58
265752_at	putative Athila retroelement ORF1 p	---	---	-1.09	-0.12	-1.13	0.295358	0.433478	-6.58
267019_at	hypothetical protein predicted by ge	---	AT2G39130	-1.11	-0.15	-1.13	0.295494	0.433649	-6.58
263284_at	unknown protein	---	AT2G36100	-1.09	-0.13	-1.13	0.295622	0.43381	-6.58
266031_at	hypothetical protein predicted by ge	---	---	-1.09	-0.12	-1.13	0.29569	0.433881	-6.58
259445_at	dioxygenase, putative similar to dio	GA2OX6	AT1G02400	-1.70	-0.77	-1.13	0.295761	0.433948	-6.58
252029_s_a	putative RNA binding protein hetero	---	AT3G52660	1.08	0.11	1.13	0.295773	0.433948	-6.58
261056_at	hypothetical protein similar to hypot	---	AT1G01360	1.14	0.19	1.13	0.295871	0.434048	-6.58
260161_at	hypothetical protein similar to putati	ROPGEF12	AT1G79860	1.08	0.11	1.13	0.29588	0.434048	-6.58
246500_at	putative protein HR21spA protein i	SYN4	AT5G16270	1.10	0.14	1.13	0.295938	0.434087	-6.58
251300_at	hypothetical protein ;supported by f	---	AT3G61980	-1.14	-0.18	-1.13	0.295944	0.434087	-6.58
264570_at	unknown protein Similar to Caenorh	---	AT1G05350	-1.11	-0.15	-1.13	0.296169	0.434389	-6.58
263544_at	putative ADP-glucose pyrophospho	APL4	AT2G21590	-1.11	-0.15	-1.13	0.296293	0.434543	-6.58
267190_at	putative N-myristoyltransferase ; su	NMT2	AT2G44170	-1.09	-0.13	-1.13	0.29632	0.434555	-6.58
255862_at	hypothetical protein predicted by ge	---	AT2G30300	-1.10	-0.13	-1.13	0.296371	0.434601	-6.58
255765_at	protein kinase, putative contains Pf	---	AT1G16760	-1.07	-0.10	-1.13	0.296446	0.434684	-6.58
246713_at	mRNA capping enzyme - like capp	---	AT5G28210	-1.12	-0.16	-1.13	0.296493	0.434725	-6.58
265859_at	hypothetical protein predicted by ge	---	---	-1.09	-0.13	-1.13	0.296549	0.434779	-6.58
249695_at	putative protein similar to unknown	---	---	1.09	0.12	1.13	0.296724	0.435007	-6.58
246273_at	globulin-like protein	---	AT4G36700	-1.08	-0.11	-1.13	0.296767	0.435041	-6.58
259480_at	hypothetical protein predicted by ge	---	AT1G19010	1.13	0.18	1.13	0.296877	0.435175	-6.58
249126_at	Expressed protein ; supported by cl	TOPP6	AT5G43380	1.22	0.29	1.13	0.296963	0.435274	-6.58
258224_at	LEA76 homologue type2 identical to	---	AT3G15670	-1.08	-0.12	-1.13	0.297017	0.435325	-6.58
255625_at	GBF2, G-box binding factor ;suppo	GBF2	AT4G01120	1.16	0.21	1.13	0.297043	0.435327	-6.58
259808_at	hypothetical protein contains Pfam	---	AT1G49790	-1.08	-0.11	-1.13	0.297057	0.435327	-6.58
246448_at	putative protein putative N-acetlytra	---	AT5G16800	-1.10	-0.14	-1.13	0.297159	0.435419	-6.58
258510_at	hypothetical protein predicted by ge	---	AT3G06600	-1.10	-0.14	-1.13	0.297164	0.435419	-6.58
265839_at	putative serpin	---	AT2G14540	-1.10	-0.13	-1.13	0.297177	0.435419	-6.58
256903_at	hypothetical protein	---	AT3G23960	-1.09	-0.13	-1.13	0.297253	0.435503	-6.58
255987_s_a	putative protein predicted proteins,	---	AT5G35050	1.08	0.11	1.13	0.297426	0.435729	-6.58
263077_at	putative mitotic control protein dis3	EMB2763	AT2G17510	1.08	0.11	1.13	0.297545	0.435875	-6.58
249756_at	Expressed protein ; supported by fu	---	AT5G24313	-1.08	-0.12	-1.13	0.297671	0.436031	-6.58
250534_at	NADH dehydrogenase ;supported	CI51	AT5G08530	-1.09	-0.12	-1.13	0.297735	0.436096	-6.58

253141_at	putative protein hypothetical protei	---	AT4G35430	1.11	0.15	1.13	0.29778	0.436122	-6.58
252342_at	endo-polygalacturonase - like prote	---	AT3G48950	-1.07	-0.10	-1.13	0.2978	0.436122	-6.58
251702_at	transcription factor-like protein mot	BZIP49	AT3G56660	-1.13	-0.17	-1.13	0.29782	0.436122	-6.58
251111_at	putative protein different hypothetica	---	AT5G01310	1.10	0.14	1.13	0.29784	0.436122	-6.58
249868_at	senescence-associated protein 5-lik	TET12	AT5G23030	-1.10	-0.13	-1.13	0.297848	0.436122	-6.58
254914_at	peroxidase ATP19a	---	AT4G11290	-1.51	-0.60	-1.13	0.297955	0.43625	-6.58
246228_at	peroxidase like protein	---	AT4G36430	1.43	0.51	1.13	0.298265	0.436677	-6.58
257929_at	RNA Polymerase II subunit 14.5 kD	NRPB9A	AT3G16980	1.10	0.14	1.13	0.298379	0.436816	-6.58
267023_at	putative protein transport protein SE	---	AT2G34250	-1.11	-0.16	-1.13	0.29845	0.436892	-6.58
257803_at	unknown protein	---	AT3G18790	1.09	0.12	1.13	0.298483	0.436902	-6.58
252238_at	peroxidase ATP21a	---	AT3G49960	-1.10	-0.13	-1.13	0.298496	0.436902	-6.58
251120_at	chloroplast ribosomal L1 - like prote	---	AT3G63490	-1.12	-0.16	-1.13	0.298564	0.436974	-6.58
AFFX-Athal-	Arabidopsis thaliana /REF=U84969	---	---	1.10	0.14	1.13	0.298785	0.43727	-6.59
258909_at	putative DnaJ protein Pfam HMM h	---	AT3G06340	1.08	0.10	1.13	0.298986	0.437536	-6.59
249399_at	putative protein intracellular protein	---	AT5G40290	-1.07	-0.10	-1.13	0.299036	0.43756	-6.59
248178_at	root cap protein 2-like protein	---	AT5G54370	-1.12	-0.17	-1.13	0.299041	0.43756	-6.59
245762_at	ATP-dependent DNA helicase Rec	---	AT1G27880	-1.07	-0.10	-1.12	0.299118	0.437644	-6.59
245633_at	unknown protein similar to putative	AtTLP10	AT1G25280	-1.07	-0.10	-1.12	0.299247	0.437782	-6.59
248051_at	Atmyb103 (gb|AAD40692.1)	ATMYB103	AT5G56110	-1.10	-0.13	-1.12	0.299264	0.437782	-6.59
258271_at	hypothetical protein predicted by ge	---	AT3G15605	-1.10	-0.14	-1.12	0.299269	0.437782	-6.59
246285_at	putative protein	---	AT4G36980	1.08	0.11	1.12	0.299351	0.437862	-6.59
250500_at	periaxin - like protein periaxin, Ratt	---	AT5G09530	-2.44	-1.28	-1.12	0.299362	0.437862	-6.59
262792_at	hypothetical protein predicted by ge	---	AT1G10710	-1.09	-0.13	-1.12	0.299434	0.437939	-6.59
258443_at	unknown protein	---	AT3G22290	1.11	0.15	1.12	0.299549	0.438043	-6.59
259680_at	putative AUX1-like permease simila	LAX3	AT1G77690	-1.50	-0.58	-1.12	0.299559	0.438043	-6.59
256562_at	Ty1/Copia-type retroelement polypr	---	---	1.09	0.12	1.12	0.299563	0.438043	-6.59
251571_at	putative protein several hypothetica	---	AT3G58330	-1.08	-0.12	-1.12	0.299615	0.438068	-6.59
265401_at	hypothetical protein predicted by ge	---	AT2G10970	-1.09	-0.12	-1.12	0.299618	0.438068	-6.59
260947_at	fructokinase, putative similar to fruc	---	AT1G06020	-1.07	-0.10	-1.12	0.29968	0.43813	-6.59
256775_at	hypothetical protein predicted by ge	---	AT3G13770	1.16	0.21	1.12	0.299705	0.438139	-6.59
257549_at	hypothetical protein predicted by ge	agl103	AT3G18650	-1.12	-0.16	-1.12	0.299751	0.438178	-6.59
251203_at	putative protein putative transcriptio	---	AT3G63070	1.08	0.11	1.12	0.299824	0.438256	-6.59
265655_at	unknown protein contains weak PH	---	AT2G13920	-1.09	-0.13	-1.12	0.29987	0.438296	-6.59
246247_at	putative protein	---	AT4G36640	1.32	0.40	1.12	0.299911	0.438327	-6.59
254342_at	receptor like protein (fragment) ES	---	AT4G22140	-1.08	-0.11	-1.12	0.299967	0.438381	-6.59

262389_at	hypothetical protein contains similar	---	AT1G49270	-1.09	-0.12	-1.12	0.300038	0.438447	-6.59
254491_at	putative protein hypothetical protein	---	AT4G20300	-1.14	-0.18	-1.12	0.300069	0.438447	-6.59
266742_at	predicted by genefinder and gensca	---	AT2G03050	-1.09	-0.12	-1.12	0.30007	0.438447	-6.59
250580_at	glutamate dehydrogenase 2 ; supp	GDH2	AT5G07440	-1.23	-0.30	-1.12	0.300176	0.438575	-6.59
255363_x_a	hypothetical protein	---	---	-1.14	-0.19	-1.12	0.30026	0.438668	-6.59
245549_at	cellulose synthase like protein	ATCSLB06	AT4G15320	-1.09	-0.13	-1.12	0.300318	0.438726	-6.59
252259_at	putative protein acidic ribosomal pr	---	AT3G49460	-1.14	-0.19	-1.12	0.300382	0.438784	-6.59
261290_at	hypothetical protein predicted by ge	---	AT1G36990	1.14	0.19	1.12	0.300397	0.438784	-6.59
253634_at	putative protein ENOD20 gene pro	---	AT4G30590	-1.07	-0.10	-1.12	0.300419	0.438789	-6.59
258795_at	hypothetical protein similar to putat	---	AT3G04570	-1.12	-0.16	-1.12	0.300494	0.43887	-6.59
257096_at	hypothetical protein	---	AT3G30820	-1.09	-0.12	-1.12	0.300773	0.439225	-6.59
247347_at	putative protein similar to unknown	SHA1	AT5G63780	-1.24	-0.31	-1.12	0.300775	0.439225	-6.59
253784_at	putative protein WREBP-1, Nicotia	PHR1	AT4G28610	1.10	0.14	1.12	0.300832	0.439279	-6.59
262725_at	hypothetical protein predicted by ge	---	AT1G43580	1.18	0.24	1.12	0.300872	0.43931	-6.59
245535_at	cell wall protein like	---	AT4G15160	-1.11	-0.15	-1.12	0.300915	0.439343	-6.59
246357_x_a	hypothetical protein	---	---	-1.10	-0.13	-1.12	0.300985	0.439418	-6.59
261616_at	NAC domain containing protein, pu	ANAC014	AT1G33060	1.07	0.10	1.12	0.301033	0.43946	-6.59
260234_at	putative lipase/acylhydrolase contai	---	AT1G74460	-1.13	-0.18	-1.12	0.301063	0.439475	-6.59
254609_at	Expressed protein ; supported by fu	---	AT4G18970	-1.26	-0.34	-1.12	0.301111	0.439518	-6.59
264841_at	putative protein kinase similar to (Z	---	AT1G03740	1.23	0.29	1.12	0.301168	0.439564	-6.59
263894_at	putative cytochrome P450	CYP96A5	AT2G21910	-1.10	-0.14	-1.12	0.301181	0.439564	-6.59
245279_at	putative protein ; supported by cDN	---	AT4G17270	-1.09	-0.12	-1.12	0.301214	0.439584	-6.59
265804_at	putative peptide methionine sulfoxid	---	AT2G18030	-1.08	-0.11	-1.12	0.301267	0.439633	-6.59
260333_at	putative polygalacturonase similar t	---	AT1G70500	-1.15	-0.20	-1.12	0.301373	0.439744	-6.59
246289_at	putative protein predicted protein A	---	AT3G56880	-1.18	-0.24	-1.12	0.301419	0.439744	-6.59
267073_at	unknown protein	---	AT2G41160	1.12	0.17	1.12	0.30143	0.439744	-6.59
251757_at	Ca-dependent solute carrier - like p	---	AT3G55640	-1.15	-0.20	-1.12	0.301437	0.439744	-6.59
245682_at	Expressed protein ; supported by cl	---	AT5G08750	-1.10	-0.13	-1.12	0.30144	0.439744	-6.59
247683_at	serine/threonine-specific protein kir	---	AT5G59660	-1.11	-0.15	-1.12	0.301468	0.439757	-6.59
253177_s_a	O-methyltransferase - like protein c	---	AT4G35150	-1.15	-0.20	-1.12	0.301519	0.439794	-6.59
254416_at	receptor-like serine/threonine protei	ARK3	AT4G21380	1.34	0.42	1.12	0.301532	0.439794	-6.59
266242_at	hypothetical protein predicted by ge	---	AT2G27790	1.09	0.12	1.12	0.301644	0.43993	-6.59
253642_at	hypothetical protein ;supported by f	---	AT4G29960	1.12	0.16	1.12	0.301837	0.44018	-6.59
258315_at	Expressed protein ; supported by fu	---	AT3G16175	1.10	0.14	1.12	0.301854	0.44018	-6.59
252570_at	isovaleryl-CoA-dehydrogenase pred	IVD	AT3G45300	1.22	0.28	1.12	0.301963	0.44031	-6.59

264741_at	aspartic protease, putative similar t	---	AT1G62290	-1.33	-0.41	-1.12	0.301999	0.440334	-6.59
267516_at	unknown protein	RPT2	AT2G30520	-1.11	-0.15	-1.12	0.302032	0.440341	-6.59
247475_at	IRE (root hair elongation)	IRE	AT5G62310	-1.09	-0.12	-1.12	0.302045	0.440341	-6.59
261508_at	unknown protein	---	AT1G71730	-1.10	-0.14	-1.12	0.302061	0.440341	-6.59
255643_at	hypothetical protein	---	AT4G00840	-1.09	-0.12	-1.12	0.302173	0.440477	-6.59
263683_at	putative NAM protein similar to GB	FEZ	AT1G26870	-1.10	-0.13	-1.12	0.302215	0.440508	-6.59
262885_at	alpha-tubulin, putative similar to alp	TUA1	AT1G64740	-1.11	-0.15	-1.12	0.302268	0.440558	-6.60
257649_at	predicted protein	ARR1	AT3G16857	-1.09	-0.12	-1.12	0.302289	0.440561	-6.60
263392_at	putative PttA-like transposon protei	---	---	-1.08	-0.11	-1.12	0.302317	0.440572	-6.60
265349_at	putative kinesin heavy chain	---	AT2G22610	-1.11	-0.15	-1.12	0.302339	0.440577	-6.60
259697_at	putative DNA-binding protein simila	---	AT1G68920	1.09	0.12	1.12	0.302476	0.440749	-6.60
258895_at	putative epoxide hydrolase similar t	---	AT3G05600	-1.08	-0.10	-1.12	0.302511	0.440771	-6.60
266409_at	hypothetical protein predicted by gr	---	AT2G38510	-1.11	-0.15	-1.12	0.302626	0.440911	-6.60
250993_at	putative protein HSPC172, Homo s	---	AT5G02280	-1.10	-0.14	-1.12	0.302665	0.44094	-6.60
261792_at	cinnamoyl CoA reductase, puitative	CCR1	AT1G15950	-1.11	-0.15	-1.12	0.302724	0.440988	-6.60
249736_at	unknown protein	---	AT5G24460	-1.07	-0.10	-1.12	0.302737	0.440988	-6.60
263264_at	histone H2A ; supported by full-len	HTA8	AT2G38810	1.17	0.23	1.12	0.302872	0.441156	-6.60
254280_at	Expressed protein ; supported by fu	SMO1-2	AT4G22756	1.14	0.19	1.12	0.302997	0.44131	-6.60
249688_at	putative protein tyrosine aminotrans	---	AT5G36160	-1.12	-0.17	-1.11	0.303088	0.441411	-6.60
251954_at	putative protein predicted protein, A	CPuORF51	AT3G53668	-1.22	-0.29	-1.11	0.303105	0.441411	-6.60
251780_s_a	beta-ketoacyl-ACP reductase - like	---	AT3G55290	-1.16	-0.22	-1.11	0.303149	0.441448	-6.60
252987_at	putative growth regulator protein au	---	AT4G38390	-1.11	-0.15	-1.11	0.303179	0.441463	-6.60
254459_at	gibberellin 20-oxidase - like protein	GA2OX8	AT4G21200	-1.08	-0.11	-1.11	0.303201	0.441467	-6.60
246799_at	putative protein various predicted b	---	AT5G26940	-1.13	-0.18	-1.11	0.303223	0.441471	-6.60
263444_at	putative MADS-box protein	AGL46	AT2G28700	-1.11	-0.15	-1.11	0.303302	0.441557	-6.60
258032_at	hypothetical protein predicted by ge	---	AT3G21170	-1.13	-0.18	-1.11	0.303346	0.441593	-6.60
246113_at	putative protein predicted proteins -	---	---	-1.08	-0.12	-1.11	0.303508	0.441801	-6.60
253869_at	hypothetical protein	---	AT4G27510	-1.12	-0.16	-1.11	0.303558	0.441845	-6.60
250224_at	putative protein similar to unknown	---	AT5G14150	-1.13	-0.17	-1.11	0.303714	0.442044	-6.60
260631_at	hypothetical protein predicted by ge	---	AT1G62350	1.11	0.15	1.11	0.303806	0.44215	-6.60
257665_at	unknown protein	---	AT3G20430	1.09	0.12	1.11	0.303889	0.442243	-6.60
267231_at	putative inositol polyphosphate 5'-p	---	AT2G43900	1.09	0.13	1.11	0.303986	0.442356	-6.60
264905_at	putative Mlo protein	MLO7	AT2G17430	-1.09	-0.12	-1.11	0.304021	0.442378	-6.60
266162_at	putative bHLH transcription factor	FRU	AT2G28160	-1.10	-0.13	-1.11	0.304156	0.442504	-6.60
263241_at	arginine decarboxylase identical to	ADC1	AT2G16500	1.33	0.41	1.11	0.304163	0.442504	-6.60

247084_at	NAM (no apical meristem)-like prote	NAC105	AT5G66300	1.09	0.12	1.11	0.304166	0.442504	-6.60
262650_at	lipoamide dehydrogenase, putative	---	AT1G14090	1.07	0.09	1.11	0.304203	0.44253	-6.60
260108_at	hypothetical protein similar to putati	---	AT1G63280	-1.10	-0.14	-1.11	0.304237	0.442551	-6.60
257748_at	hypothetical protein predicted by ge	PUB29	AT3G18710	1.77	0.83	1.11	0.304402	0.442763	-6.60
260142_at	unknown protein ; supported by cD	---	AT1G71900	-1.11	-0.15	-1.11	0.304485	0.442855	-6.60
265281_at	unknown protein ; supported by ful	---	AT2G28370	-1.11	-0.16	-1.11	0.304618	0.44302	-6.60
263292_at	putative Ta11-like non-LTR retroele	---	---	-1.09	-0.13	-1.11	0.304888	0.443386	-6.60
252428_at	putative protein rjs - Mus musculus	---	AT3G47660	-1.11	-0.14	-1.11	0.304947	0.443443	-6.60
252509_at	putative protein serine/threonine-sp	---	AT3G46240	-1.07	-0.10	-1.11	0.30498	0.443463	-6.60
250360_at	putative protein probable serine/thr	---	AT5G11360	-1.09	-0.12	-1.11	0.305212	0.443771	-6.60
252906_at	putative gamma-glutamyltransferas	GGT1	AT4G39640	-1.18	-0.24	-1.11	0.305255	0.443806	-6.60
255234_at	contains similarity to Medicago trun	---	AT4G05500	-1.09	-0.13	-1.11	0.305415	0.44401	-6.60
245680_at	hypothetical protein contains similar	---	AT1G56570	1.09	0.12	1.11	0.3057	0.444396	-6.60
248013_at	putative protein contains similarity t	ACA8	AT5G56330	-1.13	-0.18	-1.11	0.305793	0.444485	-6.60
263139_at	hypothetical protein similar to hypot	---	AT1G65110	-1.07	-0.10	-1.11	0.3058	0.444485	-6.60
253192_at	putative protein nuclear phosphopr	---	AT4G35370	-1.08	-0.11	-1.11	0.305874	0.444564	-6.60
255193_at	score=10.1, E=1.2, N=1 contains s	VFB3	AT4G07400	1.10	0.14	1.11	0.306148	0.444934	-6.61
259826_at	arm repeat-containing protein, puta	PUB17	AT1G29340	-1.15	-0.21	-1.11	0.306172	0.44494	-6.61
252800_at	putative protein	---	---	-1.09	-0.12	-1.11	0.306194	0.444944	-6.61
246963_at	putative protein nucleoid DNA-bind	---	AT5G24820	-1.11	-0.15	-1.11	0.3063	0.44507	-6.61
246828_at	putative protein hypothetical protei	---	---	-1.09	-0.12	-1.11	0.30632	0.44507	-6.61
250882_at	putative protein ;supported by full-l	---	AT5G04000	-1.09	-0.12	-1.11	0.306368	0.445111	-6.61
260709_at	hypothetical protein predicted by ge	ATNAP6	AT1G32500	-1.10	-0.14	-1.11	0.3064	0.445112	-6.61
248155_at	putative protein 3 (2'), 5'-bisphosph	AHL	AT5G54390	-1.11	-0.15	-1.11	0.306408	0.445112	-6.61
259767_s_a	unknown protein	---	AT1G29350	1.09	0.12	1.11	0.306426	0.445112	-6.61
245375_at	NAK like protein kinase	---	AT4G17660	-1.10	-0.14	-1.11	0.306479	0.44516	-6.61
258258_at	transcriptional regulator (FUSCA3)	FUS3	AT3G26790	-1.08	-0.11	-1.11	0.306534	0.445211	-6.61
245763_at	transposon protein, putative similar	---	AT1G27850	-1.08	-0.11	-1.11	0.306606	0.445288	-6.61
260536_at	putative electron transfer flavoprote	ETFQO	AT2G43400	1.17	0.23	1.11	0.306748	0.445441	-6.61
258890_at	putative transcription factor similar t	NF-YA2	AT3G05690	1.17	0.22	1.11	0.306751	0.445441	-6.61
256334_at	oligopeptide transporter, putative s	---	AT1G72120	-1.14	-0.18	-1.11	0.306837	0.445538	-6.61
265876_at	putative receptor-like protein kinase	---	AT2G42290	1.08	0.11	1.11	0.306894	0.445574	-6.61
254849_at	putative protein RNA splicing-relate	---	AT4G11970	1.07	0.10	1.11	0.306901	0.445574	-6.61
256565_at	hypothetical protein	AT3G1951	---	-1.09	-0.12	-1.11	0.307072	0.445793	-6.61
266035_at	enoyl-ACP reductase (enr-A)	MOD1	AT2G05990	-1.19	-0.25	-1.10	0.307105	0.445813	-6.61

267091_at	Expressed protein ; supported by fu	---	AT2G38185	1.15	0.20	1.10	0.307236	0.44595	-6.61
264393_at	unknown protein Contains similarity	---	AT1G11880	-1.09	-0.13	-1.10	0.307238	0.44595	-6.61
244961_at	hypothetical protein	---	---	1.08	0.11	1.10	0.307306	0.44602	-6.61
262627_at	hypothetical protein	---	AT1G06580	-1.10	-0.14	-1.10	0.307343	0.446045	-6.61
264116_at	putative poly (ADP-ribose) polymera	PARP2	AT2G31320	1.18	0.24	1.10	0.307473	0.446205	-6.61
259064_at	putative calmodulin similar to calmo	AGD11	AT3G07490	-1.10	-0.14	-1.10	0.307598	0.446359	-6.61
260828_at	unknown protein	---	AT1G06750	1.09	0.12	1.10	0.307834	0.446672	-6.61
261180_at	hypothetical protein predicted by ge	---	---	-1.09	-0.12	-1.10	0.307864	0.446674	-6.61
265117_at	putative proline-rich cell wall protei	---	AT1G62500	-1.14	-0.19	-1.10	0.307874	0.446674	-6.61
263964_at	putative CCHC-type zinc finger prot	---	AT2G12880	-1.10	-0.14	-1.10	0.307905	0.44669	-6.61
252079_at	MAP kinase ; supported by cDNA:	CPuORF44	AT3G51630	1.08	0.11	1.10	0.308013	0.446818	-6.61
260887_at	ascorbate oxidase promoter-bindin	---	AT1G29160	-1.34	-0.42	-1.10	0.30818	0.447032	-6.61
263111_s_a	hypothetical protein contains simila	---	AT1G65190	1.13	0.18	1.10	0.308285	0.447139	-6.61
246735_at	histone H2A-like protein histone H2	HTA7	AT5G27670	-1.10	-0.14	-1.10	0.308292	0.447139	-6.61
247356_at	beta-galactosidase (emb|CAB6474	MUM2	AT5G63800	1.19	0.25	1.10	0.308335	0.447171	-6.61
265734_at	predicted by genscan and genefind	---	AT2G01260	1.09	0.12	1.10	0.308375	0.447185	-6.61
245545_at	UTP-glucose glucosyltransferase	UGT71B5	AT4G15280	1.12	0.16	1.10	0.308383	0.447185	-6.61
259972_at	unknown protein N-term similar to	CUC3	AT1G76420	-1.15	-0.20	-1.10	0.308552	0.447401	-6.61
267087_at	putative MYB family transcription fa	MYB101	AT2G32460	1.14	0.19	1.10	0.308648	0.447489	-6.61
250008_at	triacylglycerol lipase-like protein tri	---	AT5G18630	1.20	0.26	1.10	0.308652	0.447489	-6.61
249337_at	putative protein contains similarity t	---	AT5G41080	1.40	0.49	1.10	0.308676	0.447496	-6.61
250271_at	putative protein predicted protein,	CLE40	AT5G12990	-1.08	-0.11	-1.10	0.308706	0.447511	-6.61
256893_x_a	unknown protein	---	---	1.07	0.10	1.10	0.30874	0.44752	-6.61
259151_at	kinesin-like protein similar to carbo	---	AT3G10310	-1.12	-0.16	-1.10	0.308751	0.44752	-6.61
248538_at	putative protein contains similarity t	---	AT5G50110	1.12	0.17	1.10	0.308835	0.447613	-6.61
255376_x_a	putative athila-like protein	---	---	-1.13	-0.17	-1.10	0.30891	0.447693	-6.61
254530_at	small GTP-binding protein - like GT	ARA7	AT4G19640	1.08	0.11	1.10	0.308965	0.447745	-6.61
254364_at	glycine-rich protein glycine-rich cel	---	AT4G22020	-1.11	-0.15	-1.10	0.308998	0.447764	-6.61
246938_at	cyclin 3a	CYCA2;1	AT5G25380	-1.11	-0.15	-1.10	0.309188	0.448011	-6.61
255900_at	hypothetical protein predicted by ge	---	AT1G17830	1.28	0.36	1.10	0.309279	0.448114	-6.61
254510_at	cadinene synthase like protein (+)-	---	AT4G20210	-1.12	-0.16	-1.10	0.309418	0.448287	-6.61
259831_at	hypothetical protein predicted by ge	ZFHD1	AT1G69600	-1.08	-0.12	-1.10	0.309493	0.448366	-6.61
259108_at	putative translationally controlled tu	---	AT3G05540	-1.08	-0.11	-1.10	0.309543	0.448411	-6.61
262724_s_a	PC-MYB2, putative similar to PC-M	---	AT1G43330	-1.08	-0.11	-1.10	0.309759	0.448696	-6.61
264812_at	putative thaumatin-like pathogenesi	---	AT2G17860	1.08	0.11	1.10	0.309845	0.448768	-6.62

252430_at	CHLOROPHYLL A-B BINDING PR	LHCA4	AT3G47470	-1.11	-0.16	-1.10	0.309848	0.448768	-6.62
266043_at	hypothetical protein	---	AT2G07724	1.12	0.16	1.10	0.309872	0.448773	-6.62
265767_at	putative exonuclease contains zinc-	REF4	AT2G48110	1.10	0.14	1.10	0.310021	0.448942	-6.62
267085_at	putative cellulose synthase	ATCSLB02	AT2G32620	1.07	0.10	1.10	0.310028	0.448942	-6.62
250943_at	putative protein	---	AT5G03360	-1.08	-0.11	-1.10	0.310209	0.449151	-6.62
254558_at	Expressed protein ; supported by fu	---	AT4G19185	-1.08	-0.11	-1.10	0.310212	0.449151	-6.62
245313_at	UFD1 like protein ; supported by ful	---	AT4G15420	-1.11	-0.15	-1.10	0.31028	0.449222	-6.62
259065_at	unknown protein similar to putative	GLR1.4	AT3G07520	1.17	0.22	1.10	0.31031	0.449237	-6.62
257370_at	hypothetical protein predicted by ge	---	AT2G35080	-1.09	-0.13	-1.10	0.310427	0.449378	-6.62
247510_at	putative protein predicted proteins,	---	AT5G62030	1.11	0.16	1.10	0.31048	0.449426	-6.62
261099_at	expansin At-EXP6, putative similar	ATEXPA18	AT1G62980	-1.12	-0.16	-1.10	0.310522	0.449458	-6.62
253645_at	putative protein G protein beta sub	VIP3	AT4G29830	1.09	0.12	1.10	0.310621	0.449572	-6.62
258889_at	putative pectinesterase similar to pe	---	AT3G05610	-1.13	-0.17	-1.10	0.310658	0.449598	-6.62
257341_at	pseudogene, Mutator-like transpos	---	---	-1.08	-0.11	-1.10	0.310757	0.449712	-6.62
249884_at	Na+/H+ antiporter-like protein	CHX9	AT5G22910	-1.09	-0.13	-1.10	0.310819	0.449774	-6.62
263752_at	putative retroelement pol polyprotei	---	---	-1.12	-0.16	-1.10	0.3109	0.449862	-6.62
246267_at	Acyl CoA binding protein, putative	ACBP6	AT1G31812	1.08	0.11	1.10	0.311063	0.450053	-6.62
256170_at	receptor protein kinase, putative co	---	AT1G51790	-1.16	-0.22	-1.10	0.311071	0.450053	-6.62
262406_at	myb-related protein, putative simila	AtMYB93	AT1G34670	-1.11	-0.15	-1.09	0.311301	0.450357	-6.62
262422_at	hypothetical protein similar to hypot	---	AT1G23440	1.11	0.15	1.09	0.311364	0.450419	-6.62
255936_at	calcium dependent protein kinase,	---	AT1G12680	1.09	0.12	1.09	0.311403	0.450447	-6.62
248314_at	putative protein similar to unknown	---	AT5G52620	1.07	0.10	1.09	0.311471	0.450517	-6.62
257777_x_a	hypothetical protein	---	---	-1.10	-0.14	-1.09	0.311535	0.450552	-6.62
246578_at	MADS-box protein, putative similar	AGL86	AT1G31630	-1.08	-0.11	-1.09	0.311537	0.450552	-6.62
251970_at	glucosyltransferase - like protein gl	UGT73D1	AT3G53150	1.16	0.22	1.09	0.311554	0.450552	-6.62
266066_at	putative xyloglucan endo-transglyco	XTH21	AT2G18800	-1.13	-0.17	-1.09	0.31158	0.45056	-6.62
255921_at	hypothetical protein contains Pfam	APUM8	AT1G22240	1.09	0.13	1.09	0.311672	0.450665	-6.62
254383_at	hypothetical protein	---	AT4G21950	-1.09	-0.12	-1.09	0.311753	0.450754	-6.62
252311_at	calcium dependent protein kinase -	---	AT3G49370	1.23	0.30	1.09	0.311783	0.450762	-6.62
247034_at	cyclin D3-like protein ;supported by	CYCD3;2	AT5G67260	1.13	0.18	1.09	0.311826	0.450762	-6.62
246129_at	putative protein predicted protein,	---	---	-1.07	-0.10	-1.09	0.311835	0.450762	-6.62
254866_at	putative protein other predicted pro	---	AT4G12220	1.07	0.10	1.09	0.311839	0.450762	-6.62
245259_at	kinesin like protein ; supported by c	PAKRP1	AT4G14150	-1.08	-0.11	-1.09	0.311858	0.450762	-6.62
248498_at	selenium-binding protein-like	---	AT5G50390	-1.09	-0.12	-1.09	0.312015	0.45096	-6.62
253225_at	Rho1Ps homolog Rac-like protein ;	ARAC3	AT4G35020	-1.10	-0.14	-1.09	0.312047	0.450978	-6.62

265378_at	putative GATA-type zinc finger tran	---	AT2G18380	-1.09	-0.12	-1.09	0.312084	0.451004	-6.62
260990_at	hypothetical protein predicted by ge	---	AT1G12180	-1.12	-0.16	-1.09	0.312296	0.451265	-6.62
246854_at	mitochondrial carrier - like protein r	---	AT5G26200	-1.10	-0.14	-1.09	0.312351	0.451265	-6.62
251848_at	Expressed protein ; supported by cl	BZIP25	AT3G54620	1.10	0.13	1.09	0.312356	0.451265	-6.62
247950_at	unknown protein	---	AT5G57230	1.09	0.12	1.09	0.312361	0.451265	-6.62
265213_at	clathrin protein family Similar to cla	---	AT1G05020	-1.13	-0.18	-1.09	0.312364	0.451265	-6.62
263587_at	putative synaptobrevin	ATVAMP71	AT2G25340	1.10	0.13	1.09	0.312422	0.45132	-6.62
245600_at	hypothetical protein	---	AT4G14230	-1.12	-0.16	-1.09	0.312493	0.451395	-6.62
266953_at	hypothetical protein predicted by ge	---	AT2G34540	-1.09	-0.12	-1.09	0.312551	0.45145	-6.62
258387_at	hypothetical protein predicted by ge	---	AT3G15550	-1.10	-0.14	-1.09	0.312753	0.451713	-6.62
250346_at	lysine decarboxylase - like protein	---	AT5G11950	1.27	0.34	1.09	0.312793	0.451742	-6.62
248363_at	nodulin-like protein	---	AT5G52480	-1.08	-0.11	-1.09	0.312917	0.451893	-6.62
254437_s_a	putative protein	---	AT3G06433	-1.15	-0.20	-1.09	0.313015	0.452005	-6.62
256128_at	laccase, putative similar to high-pI	LAC1	AT1G18140	-1.10	-0.14	-1.09	0.313113	0.452091	-6.62
245058_at	hypothetical protein predicted by ge	---	AT2G39790	-1.09	-0.12	-1.09	0.313114	0.452091	-6.62
250853_s_a	putative protein prtedicted prteins,	---	AT5G04680	-1.07	-0.10	-1.09	0.313136	0.452094	-6.62
254691_at	hypothetical protein ; supported by	---	AT4G17840	1.15	0.21	1.09	0.313163	0.452105	-6.62
250652_at	putative protein similar to unknown	FLA21	AT5G06920	-1.09	-0.12	-1.09	0.313268	0.452227	-6.62
266277_at	putative tropinone reductase	---	AT2G29310	1.10	0.14	1.09	0.313332	0.452291	-6.62
245001_at	PSII low MW protein	---	---	1.10	0.14	1.09	0.313362	0.452306	-6.62
259851_at	kinesin, putative	---	AT1G72250	-1.08	-0.11	-1.09	0.313616	0.452643	-6.63
250556_at	diacylglycerol kinase (ATDGK1) ; su	DGK1	AT5G07920	1.20	0.27	1.09	0.313659	0.452658	-6.63
252813_at	putative protein several hypothetica	---	---	-1.08	-0.12	-1.09	0.313666	0.452658	-6.63
252801_at	putative protein various predicted H	---	---	-1.08	-0.11	-1.09	0.313695	0.452672	-6.63
246753_at	putative protein	---	---	-1.11	-0.14	-1.09	0.313817	0.45282	-6.63
257859_at	Expressed protein ; supported by fu	---	AT3G12955	-1.10	-0.14	-1.09	0.313889	0.452894	-6.63
245369_at	Expressed protein ; supported by fu	---	AT4G15975	1.18	0.24	1.09	0.313947	0.452922	-6.63
257303_at	hypothetical protein predicted by ge	---	AT3G30230	-1.09	-0.12	-1.09	0.313948	0.452922	-6.63
249689_at	putative protein similar to unknown	---	AT5G36190	-1.07	-0.10	-1.09	0.314126	0.45315	-6.63
260099_at	unknown protein ;supported by full-	---	AT1G73180	1.08	0.11	1.09	0.314185	0.453182	-6.63
252296_at	putative protein GMFP7 isoprenyla	---	AT3G48970	-1.16	-0.22	-1.09	0.314188	0.453182	-6.63
248428_at	protein phosphatase-2C; PP2C-like	AHG1	AT5G51760	-1.08	-0.11	-1.09	0.314266	0.453266	-6.63
258019_at	hypothetical protein predicted by ge	---	AT3G19470	-1.08	-0.12	-1.09	0.314338	0.453342	-6.63
246902_at	putative protein	---	AT5G25640	-1.08	-0.11	-1.09	0.314432	0.453448	-6.63
250590_at	exonuclease-like protein	---	AT5G07710	-1.08	-0.12	-1.09	0.314474	0.45348	-6.63

257326_s_a	hypothetical protein	---	---	-1.11	-0.15	-1.09	0.314664	0.453725	-6.63
252274_at	branched-chain-amino-acid transar	BCAT3	AT3G49680	-1.09	-0.12	-1.09	0.314831	0.453921	-6.63
267139_s_a	hypothetical protein predicted by ge	---	AT2G38195 ,	-1.08	-0.10	-1.09	0.314839	0.453921	-6.63
254246_at	protein kinase - like protein recepto	EMB1290	AT4G23250	-1.08	-0.11	-1.09	0.314923	0.454013	-6.63
250870_at	hypothetical protein	---	AT5G03920	-1.09	-0.12	-1.09	0.315061	0.454183	-6.63
246687_at	putative protein proline-rich protein	---	AT5G33370	-1.12	-0.16	-1.09	0.315081	0.454183	-6.63
263790_at	hypothetical protein predicted by ge	---	AT2G24530	1.12	0.16	1.09	0.31511	0.454196	-6.63
263016_at	ubiquitin extension protein, putative	---	AT1G23410	1.10	0.14	1.08	0.315261	0.454336	-6.63
250211_at	unknown protein ; supported by cD	---	AT5G13880	-1.09	-0.12	-1.08	0.315261	0.454336	-6.63
249107_at	unknown protein	---	AT5G43680	1.09	0.12	1.08	0.315267	0.454336	-6.63
254467_at	receptor protein kinase - like protei	---	AT4G20450	-1.10	-0.14	-1.08	0.315326	0.454393	-6.63
256291_at	protein kinase, putative contains Pf	AtNek7	AT3G12200	1.10	0.14	1.08	0.315358	0.45441	-6.63
253741_at	putative protein various hypothetica	---	AT4G28890	1.08	0.11	1.08	0.315439	0.454471	-6.63
254158_at	putative protein dihydrofolate reduc	---	AT4G24380	-1.45	-0.53	-1.08	0.315455	0.454471	-6.63
246202_at	methionyl aminopeptidase-like prote	MAP1D	AT4G37040	-1.13	-0.18	-1.08	0.315477	0.454471	-6.63
267203_at	hypothetical protein predicted by ge	---	AT2G31035	1.07	0.09	1.08	0.31548	0.454471	-6.63
253411_at	homeobox gene ATH1	ATH1	AT4G32980	1.19	0.25	1.08	0.315512	0.454488	-6.63
259669_at	short chain alcohol dehydrogenase	ABA2	AT1G52340	1.11	0.15	1.08	0.315549	0.454513	-6.63
262582_at	hypothetical protein Is a member of	---	AT1G15410	-1.27	-0.35	-1.08	0.315579	0.454528	-6.63
254756_at	putative protein late embryogenesi	EDA21	AT4G13235	-1.09	-0.12	-1.08	0.315698	0.454671	-6.63
247070_at	Expressed protein ; supported by fu	---	AT5G66815	-1.10	-0.13	-1.08	0.315739	0.454681	-6.63
247304_at	amino acid transporter AAP4 (pir||S	AAP4	AT5G63850	-1.14	-0.19	-1.08	0.315745	0.454681	-6.63
248922_at	putative protein contains similarity t	WOX8	AT5G45980	-1.08	-0.11	-1.08	0.315766	0.454683	-6.63
246690_at	hypothetical protein	---	---	-1.09	-0.13	-1.08	0.31585	0.454774	-6.63
257230_at	DegP protease contains similarity to	DegP11	AT3G16540	-1.07	-0.10	-1.08	0.315921	0.454848	-6.63
264940_at	galactinol synthase, putative simila	AtGolS4	AT1G60470	1.32	0.40	1.08	0.316033	0.454981	-6.63
249189_at	putative protein similar to unknown	AtHB27	AT5G42780	-1.14	-0.18	-1.08	0.316078	0.45499	-6.63
256017_at	unknown protein ;supported by full-	JAZ1	AT1G19180	-1.32	-0.40	-1.08	0.31608	0.45499	-6.63
265503_at	putative non-LTR retroelement reve	---	---	-1.12	-0.16	-1.08	0.316141	0.45503	-6.63
251241_s_a	putative protein hypothetical protein	---	AT3G62460 ,	-1.13	-0.17	-1.08	0.316147	0.45503	-6.63
247105_at	putative protein similar to unknown	DAR5	AT5G66630	1.12	0.17	1.08	0.316297	0.455217	-6.63
245798_at	hypothetical protein predicted by ge	---	AT1G45545	1.07	0.10	1.08	0.316569	0.455579	-6.63
249603_at	putative protein predicted proteins,	---	AT5G37210	-1.08	-0.12	-1.08	0.316693	0.455703	-6.63
256906_at	hypothetical protein contains Pfam	---	AT3G24000	1.11	0.15	1.08	0.316694	0.455703	-6.63
248992_at	disease resistance protein RPS4	---	AT5G45230	-1.09	-0.13	-1.08	0.316784	0.455803	-6.63

250014_at	anthranilate phosphoribosyltransfe	TRP1	AT5G17990	-1.36	-0.44	-1.08	0.316919	0.455968	-6.63
261320_at	unknown protein ; supported by cDI	---	AT1G53120	1.08	0.11	1.08	0.316942	0.455972	-6.63
250275_at	putative protein mRNA, Moritella m	---	AT5G13030	-1.12	-0.17	-1.08	0.316979	0.455997	-6.63
263368_at	putative protein kinase contains a p	---	AT2G20470	-1.11	-0.15	-1.08	0.317044	0.456049	-6.63
249797_at	putative protein contains similarity t	---	AT5G23750	1.12	0.16	1.08	0.317055	0.456049	-6.63
256954_at	E2, ubiquitin-conjugating enzyme,	FUS9	AT3G13550	-1.10	-0.14	-1.08	0.317291	0.456352	-6.63
264444_at	putative squamosa-promoter bindin	---	AT1G27360	1.09	0.12	1.08	0.317306	0.456352	-6.63
265158_at	hypothetical protein predicted by ge	---	AT1G31040	-1.07	-0.10	-1.08	0.317341	0.456375	-6.63
265239_s_a	hypothetical protein	---	AT2G07692	-1.10	-0.14	-1.08	0.317422	0.456461	-6.63
245972_at	putative protein predicted proteins,	---	AT5G20680	-1.12	-0.16	-1.08	0.317529	0.456586	-6.63
249241_at	serine carboxypeptidase-II like	scpl41	AT5G42230	-1.08	-0.11	-1.08	0.317671	0.456755	-6.64
251269_at	putative protein Pm5 protein - Hom	---	AT3G62360	-1.11	-0.15	-1.08	0.317686	0.456755	-6.64
253750_at	putative transcription factor leghem	---	AT4G29000	1.12	0.17	1.08	0.317751	0.45682	-6.64
246626_at	hypothetical protein contains similar	---	AT1G48870	-1.13	-0.18	-1.08	0.317837	0.456896	-6.64
254474_at	putative protein predicted proteins,	---	AT4G20390	-1.13	-0.18	-1.08	0.317844	0.456896	-6.64
259310_s_a	Expressed protein ; supported by cl	---	AT3G05160	1.11	0.16	1.08	0.317913	0.456966	-6.64
267232_at	hypothetical protein predicted by ge	EDE1	AT2G44190	-1.09	-0.13	-1.08	0.318004	0.457068	-6.64
259988_at	Athila ORF 1, putative simlar to GI:	---	---	-1.07	-0.10	-1.08	0.318045	0.457098	-6.64
245718_at	putative protein	---	AT5G04110	1.11	0.15	1.08	0.318114	0.457143	-6.64
251338_at	putative protein VAP27, Nicotiana	VAP	AT3G60600	1.08	0.11	1.08	0.31812	0.457143	-6.64
258129_at	hypothetical protein predicted by ge	ROPGEF8	AT3G24620	-1.09	-0.13	-1.08	0.318163	0.457143	-6.64
251708_at	Expressed protein ; supported by cl	---	AT3G56580	-1.12	-0.16	-1.08	0.318167	0.457143	-6.64
246297_at	putative protein hypothetical protei	---	AT3G51760	1.13	0.18	1.08	0.318177	0.457143	-6.64
266550_s_a	Expressed protein ; supported by cl	---	AT2G35070	1.34	0.43	1.08	0.318253	0.457224	-6.64
253997_at	disease resistance protein RPS2 ;	RPS2	AT4G26090	1.31	0.39	1.08	0.318347	0.457331	-6.64
251693_at	putative protein several hypothetica	---	AT3G56550	1.10	0.13	1.08	0.318393	0.457367	-6.64
264079_at	putative seed storage protein (vicilir	---	AT2G28490	-1.09	-0.12	-1.08	0.318441	0.457408	-6.64
259879_at	putative calmodulin similar to calmo	CML38	AT1G76650	-1.48	-0.56	-1.08	0.318471	0.457422	-6.64
256666_at	histone H2A, putative similar to his	HTA13	AT3G20670	1.21	0.27	1.08	0.318681	0.457695	-6.64
248004_at	putative protein similar to unknown	PRA1.G2	AT5G56230	-1.19	-0.25	-1.08	0.318935	0.458031	-6.64
260724_at	unknown protein ;supported by full-	---	AT1G48140	1.09	0.12	1.08	0.319057	0.458177	-6.64
262265_at	hypothetical protein predicted by ge	---	---	-1.13	-0.17	-1.08	0.319241	0.458413	-6.64
261510_at	hypothetical protein predicted by ge	---	AT1G71760	-1.09	-0.12	-1.08	0.31927	0.458426	-6.64
253532_at	putative protein centromere protein	---	AT4G31570	-1.09	-0.12	-1.08	0.319346	0.458506	-6.64
259298_at	putative disease resistance protein	AtRLP31	AT3G05370	1.43	0.52	1.07	0.319517	0.458723	-6.64

254472_at	putative protein predicted proteins,	---	---	-1.07	-0.10	-1.07	0.319747	0.459007	-6.64
266747_at	predicted by genefinder and gensca	---	AT2G02870	1.11	0.15	1.07	0.319756	0.459007	-6.64
255811_at	heat shock protein 22.0 ; supported	ATHSP22.0	AT4G10250	-1.11	-0.15	-1.07	0.31996	0.459244	-6.64
264890_at	unknown protein	---	AT1G23180	1.13	0.17	1.07	0.319961	0.459244	-6.64
257192_at	unknown protein ;supported by full-	EMB2769	AT3G13200	1.10	0.14	1.07	0.320004	0.459262	-6.64
261934_at	UDP-glucose glucosyltransferase,	UGT85A1	AT1G22400	1.29	0.37	1.07	0.320013	0.459262	-6.64
245777_at	unknown protein contains similarity	atnudt21	AT1G73540	1.47	0.55	1.07	0.320038	0.459269	-6.64
252841_at	putative protein replication protein	---	---	-1.11	-0.15	-1.07	0.320101	0.45933	-6.64
263812_at	putative replication protein A1	---	---	-1.07	-0.10	-1.07	0.320123	0.459332	-6.64
247308_at	receptor-like protein kinase	---	AT5G63930	1.09	0.12	1.07	0.320199	0.459398	-6.64
248512_at	protein translocation complex Sec6	SIG2	AT1G08540	1.19	0.25	1.07	0.320209	0.459398	-6.64
265035_at	hypothetical protein contains Pfam	---	AT1G61620	1.10	0.13	1.07	0.320278	0.459468	-6.64
256802_at	cytochrome P450, putative similar	CYP705A3	AT3G20950	-1.08	-0.11	-1.07	0.320306	0.459479	-6.64
257486_at	unknown protein	---	AT1G63590	1.08	0.11	1.07	0.320548	0.459768	-6.64
263649_at	putative 1-aminocyclopropane-1-ca	---	AT1G04380	-1.07	-0.10	-1.07	0.320566	0.459768	-6.64
258731_at	unknown protein ; supported by cD	---	AT3G11880	1.09	0.12	1.07	0.320568	0.459768	-6.64
252371_at	putative protein finger protein SIG1	---	AT3G48070	1.10	0.14	1.07	0.320609	0.459798	-6.64
263748_at	putative protein kinase contains a p	---	AT2G21480	-1.09	-0.13	-1.07	0.320768	0.459968	-6.64
266214_at	putative retroelement integrase	---	---	-1.07	-0.10	-1.07	0.320785	0.459968	-6.64
255179_at	putative transposon protein	---	---	-1.07	-0.09	-1.07	0.320808	0.459968	-6.64
259699_at	hypothetical protein predicted by ge	---	AT1G68940	-1.08	-0.10	-1.07	0.320808	0.459968	-6.64
252190_at	putative protein various predicted g	---	AT3G50170	-1.08	-0.11	-1.07	0.320874	0.460033	-6.64
266118_at	protease inhibitor II ; supported by	LCR68	AT2G02130	-1.25	-0.32	-1.07	0.32093	0.460059	-6.64
247737_at	putative protein various predicted p	---	AT5G59200	1.07	0.09	1.07	0.320974	0.460059	-6.64
256082_at	hypothetical protein predicted by ge	---	AT1G20720	-1.16	-0.22	-1.07	0.320986	0.460059	-6.64
253681_at	cytidine deaminase 3 (CDA3)	---	AT4G29630	-1.09	-0.13	-1.07	0.320992	0.460059	-6.64
261389_s_a	hypothetical protein identical to hyp	---	---	1.07	0.10	1.07	0.320993	0.460059	-6.64
255415_at	hypothetical protein	---	AT4G03160	1.07	0.10	1.07	0.32102	0.46007	-6.64
265234_at	hypothetical protein	---	AT2G07721	-1.10	-0.14	-1.07	0.321102	0.460129	-6.64
254347_at	putative protein DNA-binding prote	WRKY31	AT4G22070	1.11	0.15	1.07	0.321102	0.460129	-6.64
264966_at	hypothetical protein predicted by ge	---	AT1G60570	-1.12	-0.17	-1.07	0.321197	0.460223	-6.64
254278_at	Expressed protein ; supported by fu	---	AT4G22740	-1.10	-0.13	-1.07	0.321216	0.460223	-6.64
263152_at	hypothetical protein predicted by ge	ASIL1	AT1G54060	-1.13	-0.18	-1.07	0.321228	0.460223	-6.64
248251_at	unknown protein	---	AT5G53220	1.08	0.11	1.07	0.321255	0.460233	-6.64
250783_at	cytochrome P450 ; supported by cl	CYP79A2	AT5G05260	-1.10	-0.14	-1.07	0.321309	0.460281	-6.64

266029_at	putative serine carboxypeptidase II	scpl38	AT2G05850	-1.09	-0.13	-1.07	0.321403	0.460366	-6.64
266740_at	hypothetical protein predicted by ge	---	AT2G46840	-1.09	-0.13	-1.07	0.321409	0.460366	-6.64
255328_at	putative leucyl tRNA synthetase	EMB2369	AT4G04350	-1.12	-0.17	-1.07	0.321481	0.460442	-6.64
246680_at	putative protein predicted proteins -	---	---	-1.10	-0.14	-1.07	0.321521	0.460469	-6.65
262535_at	trehalose-6-phosphate synthase, p	ATTPS3	AT1G17000	-1.07	-0.09	-1.07	0.321543	0.460473	-6.65
256218_at	hypothetical protein predicted by ge	---	AT1G56270	-1.11	-0.15	-1.07	0.321597	0.46052	-6.65
254935_at	putative disease resistance protein	---	AT4G10780	-1.11	-0.14	-1.07	0.321631	0.46054	-6.65
256051_at	hypothetical protein similar to putati	CHX14	AT1G06970	-1.11	-0.15	-1.07	0.321668	0.46056	-6.65
253999_at	1-aminocyclopropane-1-carboxylat	ACS7	AT4G26200	-1.20	-0.27	-1.07	0.321685	0.46056	-6.65
256867_at	hypothetical protein predicted by ge	---	AT3G26390	-1.08	-0.10	-1.07	0.321905	0.460835	-6.65
265526_x_a	putative Athila retroelement ORF1 p	---	---	-1.07	-0.10	-1.07	0.321918	0.460835	-6.65
248909_at	serine threonine protein kinase ; su	CIPK19	AT5G45810	-1.13	-0.17	-1.07	0.321954	0.460858	-6.65
249186_at	CHP-rich zinc finger protein-like	---	AT5G43040	-1.08	-0.11	-1.07	0.322021	0.460901	-6.65
259194_at	RNA helicase, DRH1 identical to R	DRH1	AT3G01540	1.07	0.10	1.07	0.322024	0.460901	-6.65
256548_at	hypothetical protein contains similar	---	AT3G14770	-1.57	-0.65	-1.07	0.322134	0.461023	-6.65
259203_at	hypothetical protein predicted by ge	---	AT3G09130	-1.09	-0.13	-1.07	0.32215	0.461023	-6.65
267288_at	similar to cold acclimation protein W	---	AT2G23680	1.32	0.40	1.07	0.32222	0.461093	-6.65
253568_at	putative protein receptor-like kinase	---	AT4G31230	1.09	0.13	1.07	0.322281	0.461153	-6.65
247354_at	flavonol synthase ;supported by ful	FLS3	AT5G63590	-1.07	-0.10	-1.07	0.322346	0.461216	-6.65
250911_at	SERINE/THREONINE-PROTEIN K	CTR1	AT5G03730	1.12	0.16	1.07	0.322371	0.461216	-6.65
252855_at	putative protein alpha-amino-epsilo	AGT2	AT4G39660	1.11	0.15	1.07	0.322386	0.461216	-6.65
252933_at	putative receptor-like protein kinase	---	AT4G39110	-1.10	-0.14	-1.07	0.322433	0.461253	-6.65
256917_at	unknown protein contains Pfam pro	---	AT3G18930	1.08	0.11	1.07	0.322479	0.461291	-6.65
259550_at	hypothetical protein ;supported by f	AGP5	AT1G35230	1.41	0.49	1.07	0.322516	0.461314	-6.65
249725_at	unknown protein	---	AT5G35470	1.08	0.11	1.07	0.322543	0.461325	-6.65
265525_at	putative Ty3-gypsy-like retroelemen	---	---	-1.10	-0.13	-1.07	0.322576	0.461343	-6.65
245858_at	CER1-like protein CER1-like, A.tha	---	AT5G28280	1.10	0.13	1.07	0.322639	0.461376	-6.65
266523_at	putative transportin	TRN1	AT2G16950	1.08	0.11	1.07	0.32264	0.461376	-6.65
247369_at	unknown protein	---	AT5G63340	-1.10	-0.14	-1.07	0.322718	0.461459	-6.65
251921_at	40S ribosomal protein S21 homolo	---	AT3G53890	1.23	0.29	1.07	0.322772	0.461508	-6.65
246006_at	putative protein DIM1 protein, Hom	YLS8	AT5G08290	1.08	0.12	1.07	0.322984	0.461782	-6.65
248161_at	unknown protein	---	AT5G54480	-1.09	-0.13	-1.07	0.323035	0.461799	-6.65
254208_at	Expressed protein ; supported by fu	---	AT4G24175	1.10	0.14	1.07	0.323036	0.461799	-6.65
244946_at	hypothetical protein	---	---	1.08	0.10	1.07	0.323079	0.461831	-6.65
259865_at	putative casein kinase I similar to ca	CKL2	AT1G72710	1.12	0.16	1.07	0.323104	0.461838	-6.65

266134_s_a	putative beta-expansin	---	AT1G65680	-1.09	-0.12	-1.07	0.323162	0.461892	-6.65
258594_at	hypothetical protein predicted by ge	---	AT3G04490	-1.09	-0.12	-1.07	0.323276	0.462025	-6.65
255166_at	putative protein putative centromer	---	---	-1.08	-0.11	-1.07	0.3233	0.462031	-6.65
245942_at	putative protein transcription regula	---	AT5G19490	1.07	0.10	1.07	0.32343	0.462187	-6.65
249213_at	unknown protein	---	AT5G42700	1.08	0.11	1.07	0.323462	0.46219	-6.65
252983_at	cinnamyl-alcohol dehydrogenase E	ELI3-1	AT4G37980	-1.17	-0.22	-1.07	0.323472	0.46219	-6.65
244927_at	hypothetical protein	---	AT2G07776	-1.11	-0.16	-1.06	0.323598	0.462341	-6.65
245857_at	putative protein various predicted p	---	---	-1.12	-0.16	-1.06	0.32362	0.462344	-6.65
249557_at	receptor serine/threonine kinase PF	PR5K	AT5G38280	1.17	0.22	1.06	0.323666	0.462379	-6.65
264603_at	hypothetical protein predicted by ge	---	AT1G04670	-1.07	-0.10	-1.06	0.323698	0.462396	-6.65
256312_x_a	hypothetical protein	---	AT1G35820	-1.08	-0.11	-1.06	0.32372	0.462399	-6.65
262079_at	unknown protein ; supported by cDI	ZCW7	AT1G59600	-1.13	-0.18	-1.06	0.323741	0.462401	-6.65
266502_at	hypothetical protein predicted by ge	---	AT2G47720	-1.08	-0.11	-1.06	0.323781	0.462429	-6.65
261506_at	choline kinase, putative similar to C	ATCK1	AT1G71697	-1.18	-0.24	-1.06	0.323813	0.462431	-6.65
247381_at	auxin-independent growth promoter	---	AT5G63390	-1.09	-0.13	-1.06	0.323823	0.462431	-6.65
263154_at	Expressed protein ; supported by fu	---	AT1G54110	1.11	0.15	1.06	0.323886	0.462491	-6.65
245810_at	axi 1 protein, putative similar to axi	---	AT1G38065	-1.10	-0.13	-1.06	0.324163	0.462858	-6.65
258121_s_a	putative geranylgeranyl pyrophosph	GGPS3	AT3G14530	1.10	0.14	1.06	0.32429	0.462984	-6.65
266159_at	putative chloroplast nucleoid DNA b	---	AT2G28030	-1.07	-0.10	-1.06	0.324292	0.462984	-6.65
250941_at	putative protein	---	AT5G03320	1.11	0.15	1.06	0.324377	0.463076	-6.65
256536_at	peptide chain release factor, putati	---	AT1G33330	1.08	0.11	1.06	0.324534	0.463272	-6.65
267146_at	hypothetical protein predicted by ge	---	AT2G38160	-1.08	-0.11	-1.06	0.324618	0.463363	-6.65
249602_at	porin -like protein porin, Prunus arr	---	AT5G37610	-1.09	-0.12	-1.06	0.324662	0.463397	-6.65
259698_at	hypothetical protein similar to hypo	---	AT1G68930	1.11	0.15	1.06	0.324941	0.463765	-6.65
257433_at	hypothetical protein predicted by ge	---	AT2G21990	1.08	0.11	1.06	0.325007	0.463831	-6.65
249371_at	hypothetical protein	---	AT5G40750	-1.07	-0.09	-1.06	0.325113	0.463953	-6.65
256610_at	hypothetical protein predicted by ge	---	AT3G30845	1.07	0.10	1.06	0.325163	0.463996	-6.65
253884_at	heat shock protein 21	HSP21	AT4G27670	-1.10	-0.14	-1.06	0.32521	0.464033	-6.65
260482_at	endomembrane protein EMP70 pre	---	AT1G10950	-1.09	-0.12	-1.06	0.325275	0.464071	-6.65
263081_at	putative homeodomain transcription	BLH5	AT2G27220	-1.07	-0.10	-1.06	0.325277	0.464071	-6.65
265259_at	putative WD-40 repeat protein simil	---	AT2G20330	1.09	0.13	1.06	0.325317	0.464079	-6.65
256740_at	hypothetical protein predicted by ge	---	AT3G29330	1.07	0.10	1.06	0.325323	0.464079	-6.65
265478_at	unknown protein ;supported by full-	MEE14	AT2G15890	1.08	0.12	1.06	0.325412	0.464176	-6.65
253991_at	putative nucleic acid binding protei	PEP	AT4G26000	-1.09	-0.12	-1.06	0.325466	0.464225	-6.65
247868_at	Myb-related transcription factor-like	MYB36	AT5G57620	-1.09	-0.12	-1.06	0.325507	0.464234	-6.65

258036_at	unknown protein	---	AT3G21210	-1.07	-0.10	-1.06	0.325513	0.464234	-6.65
254150_at	putative protein storage protein - P	---	AT4G24350	1.55	0.64	1.06	0.325593	0.464319	-6.66
265277_at	unknown protein ;supported by full-	---	AT2G28410	-1.27	-0.35	-1.06	0.325759	0.464526	-6.66
266849_at	putative vacuolar processing enzym	ALPHA-VP	AT2G25940	1.08	0.12	1.06	0.32584	0.464612	-6.66
246684_at	putative protein nucleoid DNA-bind	CDR1	AT5G33340	-1.12	-0.16	-1.06	0.32593	0.464692	-6.66
255528_at	hypothetical protein	---	AT4G02090	-1.08	-0.11	-1.06	0.325936	0.464692	-6.66
250179_at	putative protein	---	AT5G14440	1.10	0.14	1.06	0.326154	0.464962	-6.66
249879_at	serine/threonine protein kinase-like	---	AT5G23170	-1.09	-0.12	-1.06	0.326166	0.464962	-6.66
250157_at	prx10 peroxidase - like protein prx1	---	AT5G15180	-1.09	-0.12	-1.06	0.326286	0.465104	-6.66
253502_at	Cytochrome P450-like protein cyto	CYP82C4	AT4G31940	1.08	0.11	1.06	0.326308	0.465105	-6.66
254380_at	putative protein	---	AT4G21865	1.32	0.40	1.06	0.326328	0.465105	-6.66
255306_at	putative calcium dependent protein	CPK23	AT4G04740	1.13	0.18	1.06	0.326357	0.465117	-6.66
263969_at	putative VSF-1-like b-ZIP transcript	UNE4	AT2G12940	-1.09	-0.13	-1.06	0.326443	0.465204	-6.66
260090_at	putative serine carboxypeptidase s	scpl4	AT1G73310	1.07	0.10	1.06	0.326458	0.465204	-6.66
246531_at	MADS box protein AGL2 ; supporte	υɒo-01	AT5G15800	-1.11	-0.15	-1.06	0.326655	0.465454	-6.66
265674_at	unknown protein ; supported by ful	---	AT2G32190	1.31	0.39	1.06	0.326716	0.465487	-6.66
246649_at	putative protein predicted proteins,	---	---	-1.08	-0.11	-1.06	0.326718	0.465487	-6.66
261573_at	hypothetical protein identical to hyo	---	AT1G01180	-1.08	-0.11	-1.06	0.326743	0.465493	-6.66
252904_at	putative protein crp1 protein, Zea n	EMB2453	AT4G39620	-1.10	-0.13	-1.06	0.326849	0.465615	-6.66
252817_at	putative protein hypothetical protein	---	---	-1.11	-0.14	-1.06	0.326928	0.465678	-6.66
247276_at	putative protein contains similarity t	HEN4	AT5G64390	-1.15	-0.20	-1.06	0.326934	0.465678	-6.66
263310_s_a	hypothetical protein	---	---	-1.07	-0.10	-1.06	0.327	0.465743	-6.66
245717_at	Expressed protein ; supported by cl	---	AT5G33390	1.09	0.13	1.06	0.327078	0.465805	-6.66
251314_at	putative protein several hypothetica	---	AT3G61180	-1.07	-0.09	-1.06	0.327094	0.465805	-6.66
259827_at	hypothetical protein	---	AT1G72270	-1.08	-0.11	-1.06	0.327105	0.465805	-6.66
252994_at	putative protein gene PC326 protei	---	AT4G38480	-1.23	-0.29	-1.06	0.327211	0.465927	-6.66
254864_at	putative reverse transcriptase other	---	---	-1.06	-0.09	-1.06	0.327346	0.466091	-6.66
256139_at	Nt-gh3 deduced protein, putative s	---	AT1G48660	-1.10	-0.14	-1.06	0.327462	0.466211	-6.66
256202_s_a	hypothetical protein	---	AT1G48150	-1.08	-0.11	-1.06	0.327472	0.466211	-6.66
258740_at	Lon protease, putative similar to Lo	LON3	AT3G05780	-1.12	-0.17	-1.06	0.327686	0.466487	-6.66
247140_at	putative protein similar to unknown	---	AT5G66250	1.08	0.11	1.05	0.327835	0.46667	-6.66
258251_at	unknown protein ; supported by ful	---	AT3G15810	1.09	0.12	1.05	0.32791	0.466747	-6.66
248569_at	receptor protein kinase-like	---	AT5G49770	-1.10	-0.14	-1.05	0.327982	0.46682	-6.66
264235_at	myosin, putative similar to myosin (	XIE	AT1G54560	-1.10	-0.14	-1.05	0.328037	0.46687	-6.66
246093_at	ethylene-forming-enzyme-like diox	---	AT5G20550	1.09	0.12	1.05	0.328063	0.466878	-6.66

247252_at	unknown protein	---	AT5G64770	1.39	0.48	1.05	0.328105	0.466908	-6.66
247490_at	LFY floral meristem identity control	LFY	AT5G61850	-1.08	-0.11	-1.05	0.328128	0.466911	-6.66
258954_at	unknown protein contains Pfam pro	---	AT3G01400	-1.07	-0.09	-1.05	0.328216	0.467006	-6.66
252104_at	putative protein KIAA0851 protein,	RHD4	AT3G51460	1.11	0.15	1.05	0.328235	0.467006	-6.66
260192_at	putative DNA polymerase alpha sub	POLA2	AT1G67630	-1.27	-0.35	-1.05	0.328455	0.467289	-6.66
244996_at	ribosomal protein S2	---	---	-1.10	-0.14	-1.05	0.328543	0.467387	-6.66
261953_at	UDP-galactose 4-epimerase, putat	RHD1	AT1G64440	1.18	0.23	1.05	0.328656	0.467489	-6.66
253500_at	predicted protein RegA gene, Dicty	ARR10	AT4G31920	1.14	0.19	1.05	0.328668	0.467489	-6.66
252257_at	putative protein several hypothetica	---	AT3G49440	-1.07	-0.10	-1.05	0.328694	0.467489	-6.66
263695_at	putative phosphoribosylglycinamide	---	AT1G31220	1.08	0.12	1.05	0.328698	0.467489	-6.66
250989_at	cytochrome P450 - like protein cyto	CYP96A13	AT5G02900	-1.07	-0.09	-1.05	0.328761	0.467516	-6.66
254762_at	Expressed protein ; supported by fu	---	AT4G13230	-1.09	-0.12	-1.05	0.328765	0.467516	-6.66
265638_at	similar to PIG-L	---	AT2G27340	1.08	0.11	1.05	0.328778	0.467516	-6.66
262350_at	putative glutathione peroxidase ;su	ATGPX4	AT2G48150	-1.08	-0.11	-1.05	0.32881	0.467533	-6.66
256621_at	unknown protein ; supported by ful	---	AT3G24450	-1.09	-0.13	-1.05	0.328894	0.467623	-6.66
256096_at	unknown protein	---	AT1G13650	-1.21	-0.27	-1.05	0.329003	0.467749	-6.66
251680_at	putative protein 13S condensin XC	---	AT3G57060	-1.08	-0.11	-1.05	0.329046	0.467781	-6.66
264619_at	putative chloroplast nucleoid DNA-b	---	AT2G17760	1.10	0.13	1.05	0.329138	0.467882	-6.66
264935_at	hypothetical protein predicted by ge	---	AT1G61150	1.14	0.19	1.05	0.329183	0.467917	-6.66
265672_at	putative cysteine proteinase inhibito	---	AT2G31980	1.39	0.47	1.05	0.329204	0.467917	-6.66
262465_at	hypothetical protein similar to hypot	---	AT1G50270	-1.07	-0.10	-1.05	0.329239	0.467932	-6.66
258287_at	putative sulfate transporter similar t	SULTR3;4	AT3G15990	1.25	0.33	1.05	0.329255	0.467932	-6.66
264949_at	unknown protein contains similarity	MAF1	AT1G77080	-1.07	-0.10	-1.05	0.329338	0.468021	-6.66
248857_at	putative protein contains similarity t	---	AT5G46640	1.18	0.24	1.05	0.32942	0.468098	-6.66
252329_at	putative protein several hypothetica	---	AT3G48760	-1.08	-0.11	-1.05	0.329437	0.468098	-6.66
253287_at	putative protein dJ69E11.3, Homo	---	AT4G34270	1.07	0.10	1.05	0.329454	0.468098	-6.66
253906_at	putative protein disease resistance	---	AT4G27220	-1.12	-0.17	-1.05	0.329529	0.468176	-6.66
261307_at	unknown protein similar to Glu-tRNA	GATB	AT1G48520	-1.08	-0.11	-1.05	0.329565	0.468198	-6.66
252486_at	arm repeat containing protein homo	PUB13	AT3G46510	-1.09	-0.13	-1.05	0.329784	0.468458	-6.67
264544_s_a	unknown protein	---	AT1G55790	-1.08	-0.11	-1.05	0.32979	0.468458	-6.67
258738_at	hypothetical protein similar to hypot	---	AT3G05750	1.11	0.15	1.05	0.329828	0.468483	-6.67
256949_at	hypothetical protein predicted by ge	---	AT3G19070	-1.07	-0.10	-1.05	0.329881	0.46853	-6.67
255379_at	putative M-type thioredoxin similar	ATHM2	AT4G03520	1.07	0.10	1.05	0.329958	0.46861	-6.67
257415_at	hypothetical protein similar to hypot	---	AT1G70040	-1.10	-0.13	-1.05	0.330014	0.468646	-6.67
258420_at	putative RNA polymerase similar to	---	AT3G16680	-1.08	-0.11	-1.05	0.330024	0.468646	-6.67

245423_at	putative protein	---	AT4G17483	1.10	0.14	1.05	0.330166	0.468817	-6.67
259374_at	putative AP2 domain transcription fa	---	AT3G16280	1.12	0.17	1.05	0.330272	0.468938	-6.67
263622_at	hypothetical protein predicted by ge	---	AT2G04870	-1.08	-0.12	-1.05	0.330407	0.469069	-6.67
266654_at	putative oleosin protein	---	AT2G25890	-1.12	-0.16	-1.05	0.33044	0.469069	-6.67
265743_s_a	En/Spm-like transposon protein rela	---	---	-1.08	-0.10	-1.05	0.330442	0.469069	-6.67
248930_at	unknown protein	---	AT5G46010	-1.09	-0.12	-1.05	0.330446	0.469069	-6.67
259222_at	putative phosphoribosylanthranilate	---	AT3G03680	1.11	0.15	1.05	0.330484	0.469094	-6.67
245390_at	putative protein ;supported by full-le	---	AT4G17650	1.18	0.24	1.05	0.330524	0.469122	-6.67
250807_at	helicase-like transcription factor-like	---	AT5G05130	1.11	0.15	1.05	0.330683	0.469317	-6.67
247404_at	permease 1 - like protein permease	---	AT5G62890	-1.19	-0.26	-1.05	0.330703	0.469317	-6.67
247167_at	unknown protein	---	AT5G65850	-1.11	-0.15	-1.05	0.330727	0.469322	-6.67
266627_at	putative pre-mRNA splicing factor R	MEE29	AT2G35340	-1.09	-0.13	-1.05	0.330808	0.469408	-6.67
255912_at	2,3-oxidosqualene-triterpenoid cyc	---	AT1G66960	-1.11	-0.16	-1.05	0.330874	0.469467	-6.67
264771_at	phosphomethylpyrimidine kinase p	TH1	AT1G22940	-1.07	-0.10	-1.05	0.330891	0.469467	-6.67
252060_at	putative protein other hypothetical p	PAD4	AT3G52430	1.34	0.42	1.05	0.331465	0.470253	-6.67
253501_at	predicted protein predicted protein o	HSL1	AT4G32010	1.09	0.12	1.05	0.331532	0.470319	-6.67
264127_at	serine/threonine protein kinase, pu	AGC1.7	AT1G79250	-1.07	-0.09	-1.05	0.331778	0.470633	-6.67
249060_at	putative protein similar to unknown	VPS2.2	AT5G44560	-1.07	-0.10	-1.05	0.331815	0.470633	-6.67
257517_at	unknown protein	---	AT3G16330	-1.34	-0.42	-1.05	0.331822	0.470633	-6.67
256849_at	hypothetical protein similar to hypot	---	---	-1.10	-0.13	-1.05	0.331836	0.470633	-6.67
264483_at	unknown protein	---	AT1G77230	1.11	0.15	1.05	0.331882	0.470668	-6.67
263071_at	putative retroelement pol polyprotei	---	---	-1.08	-0.11	-1.05	0.331919	0.470691	-6.67
261251_at	hypothetical protein predicted by ge	---	AT1G05920	-1.14	-0.18	-1.05	0.331968	0.470731	-6.67
257737_at	uridine kinase/uracil phosphoribosy	---	AT3G27440	1.08	0.12	1.05	0.331999	0.470747	-6.67
257491_at	hypothetical protein predicted by ge	---	AT1G06170	-1.07	-0.09	-1.05	0.332057	0.470799	-6.67
252735_at	putative protein predicted proteins,	---	AT3G43170	1.08	0.11	1.04	0.332161	0.470918	-6.67
246142_at	putative protein	---	AT5G19970	1.07	0.10	1.04	0.332225	0.470979	-6.67
247893_at	RNA polymerase I, II and III 24.3 k	RPB5C	AT5G57980	-1.11	-0.15	-1.04	0.332386	0.471178	-6.67
265603_at	hypothetical protein predicted by ge	---	---	1.09	0.13	1.04	0.332508	0.471321	-6.67
245831_at	unknown protein	---	AT1G48840	-1.09	-0.13	-1.04	0.332679	0.471534	-6.67
262201_at	unknown protein	ORC4	AT2G01120	1.08	0.11	1.04	0.332711	0.471551	-6.67
254622_at	hypothetical protein contains similar	---	AT4G18375	1.15	0.20	1.04	0.332815	0.47165	-6.67
253354_at	putative protein RNA helicase DBP	---	AT4G33370	1.16	0.21	1.04	0.332848	0.47165	-6.67
257715_at	putative zinc transporter identical to	ZIP1	AT3G12750	1.18	0.23	1.04	0.332857	0.47165	-6.67
260515_at	ATP-dependent transmembrane tra	---	AT1G51460	-1.10	-0.13	-1.04	0.332864	0.47165	-6.67

256727_at	hypothetical protein	---	AT3G52240	-1.10	-0.14	-1.04	0.332899	0.471671	-6.67
255223_at	putative protein predicted proteins,	---	AT4G05370	-1.07	-0.10	-1.04	0.332995	0.471778	-6.67
255565_at	putative CHP-rich zinc finger protei	---	AT4G01760	-1.07	-0.10	-1.04	0.333114	0.471916	-6.67
263985_at	unknown protein ;supported by full-	---	AT2G42750	-1.15	-0.20	-1.04	0.333198	0.471999	-6.67
257473_at	unknown protein	---	AT1G33840	-1.12	-0.16	-1.04	0.333214	0.471999	-6.67
256439_at	putative [Mn] superoxide dismutase	MSD1	AT3G10920	1.10	0.14	1.04	0.333283	0.472068	-6.67
259401_at	hypothetical protein contains similar	CSTF77	AT1G17760	1.15	0.21	1.04	0.333373	0.472166	-6.67
253120_at	putative protein phospholipase D, A	PLDDELTA	AT4G35790	1.12	0.16	1.04	0.333404	0.472181	-6.67
247402_at	putative protein predicted proteins,	---	AT5G62750	-1.08	-0.11	-1.04	0.33352	0.472316	-6.67
248918_at	senescence-specific cysteine protea	SAG12	AT5G45890	-1.12	-0.16	-1.04	0.333604	0.472406	-6.67
267223_at	hypothetical protein predicted by ge	AtITPK4	AT2G43980	1.09	0.13	1.04	0.333649	0.472439	-6.67
249110_at	disease resistance protein	---	AT5G43730	1.07	0.10	1.04	0.333726	0.47252	-6.67
254204_at	putative protein CGI-58 protein - H	---	AT4G24160	-1.20	-0.26	-1.04	0.333758	0.472536	-6.67
256768_at	hypothetical protein predicted by ge	LDL2	AT3G13682	1.07	0.10	1.04	0.333805	0.472573	-6.67
252794_at	putative protein predicted proteins,	---	---	-1.07	-0.10	-1.04	0.33388	0.47265	-6.68
264980_at	receptor kinase, putative similar to	---	AT1G27190	-1.10	-0.14	-1.04	0.333915	0.47267	-6.68
250996_at	putative protein BcDNA.LD24793,	---	AT5G02530	1.08	0.11	1.04	0.33399	0.472747	-6.68
259263_at	putative UDP-glucose 6-dehydroge	---	AT3G01010	-1.08	-0.12	-1.04	0.334054	0.472807	-6.68
261862_at	DNA-binding protein, putative simil	---	AT1G50410	1.11	0.15	1.04	0.334075	0.472809	-6.68
261549_at	DNA-binding protein, putative conta	---	AT1G63470	-1.11	-0.15	-1.04	0.334284	0.473074	-6.68
262972_at	unknown protein	---	AT1G75620	-1.09	-0.13	-1.04	0.334328	0.473108	-6.68
258035_at	putative Ca2+-transporting ATPase	ACA9	AT3G21180	-1.10	-0.13	-1.04	0.334401	0.473182	-6.68
251898_at	floral homeotic protein APETALA3 (	AP3	AT3G54340	1.07	0.10	1.04	0.334482	0.473267	-6.68
261115_at	hypothetical protein predicted by ge	---	AT1G75360	-1.07	-0.10	-1.04	0.334619	0.473412	-6.68
254027_at	putative mitochondrial protein Hom	---	AT4G25835	1.14	0.19	1.04	0.334626	0.473412	-6.68
249891_at	unknown protein	---	AT5G22590	1.06	0.09	1.04	0.334671	0.473446	-6.68
251268_at	putative protein hypothetical protein	---	AT3G62350	-1.09	-0.13	-1.04	0.334695	0.473451	-6.68
262506_at	unknown protein Similar to gb|X842	NADK2	AT1G21640	1.13	0.17	1.04	0.334719	0.473455	-6.68
254716_at	putative protein LEA protein , Cicer	UNE15	AT4G13560	-1.13	-0.18	-1.04	0.334804	0.473546	-6.68
256764_at	hypothetical protein predicted by ge	---	AT3G29310	1.11	0.15	1.04	0.334828	0.47355	-6.68
251145_at	putative protein predicted protein, A	---	AT3G63500	1.10	0.14	1.04	0.334848	0.47355	-6.68
266318_at	Mutator-like transposase similar to	FRS3	AT2G27110	1.06	0.09	1.04	0.334899	0.473592	-6.68
247518_at	putative protein many predicted pro	---	AT5G61800	1.10	0.13	1.04	0.335058	0.473788	-6.68
261246_at	unknown protein	SYTB	AT1G20080	-1.09	-0.12	-1.04	0.335141	0.473876	-6.68
263271_s_a	hypothetical protein predicted by ge	---	---	1.07	0.09	1.04	0.33538	0.474184	-6.68

247163_at	Expressed protein ; supported by cl	---	AT5G65685	1.10	0.14	1.04	0.335455	0.474261	-6.68
264386_at	putative pyrophosphate-fructose-6-	---	AT1G12000	1.19	0.25	1.04	0.335566	0.474389	-6.68
247239_at	pectin methylesterase-like protein s	---	AT5G64640	-1.09	-0.13	-1.04	0.335663	0.474497	-6.68
259230_at	unknown protein ; supported by cD	OBE1	AT3G07780	-1.11	-0.15	-1.04	0.335706	0.474528	-6.68
261176_at	hypothetical protein predicted by ge	---	AT1G04780	1.07	0.10	1.04	0.335835	0.474674	-6.68
266233_at	putative phloem-specific lectin	AtPP2-B8	AT2G02340	-1.07	-0.10	-1.04	0.335851	0.474674	-6.68
251147_at	kinesin heavy chain - like protein ki	---	AT3G63480	-1.09	-0.12	-1.04	0.33591	0.474728	-6.68
258247_at	hypothetical protein predicted by ge	---	AT3G29130	-1.06	-0.09	-1.04	0.33604	0.474882	-6.68
264241_at	hypothetical protein contains similar	---	AT1G54840	-1.07	-0.09	-1.04	0.336067	0.47489	-6.68
249250_at	arm repeat containing protein	---	AT5G42340	-1.07	-0.10	-1.04	0.33623	0.47505	-6.68
267431_at	unknown protein ;supported by full-	MEE26	AT2G34870	-1.09	-0.13	-1.04	0.336236	0.47505	-6.68
258115_at	hypothetical protein predicted by ge	---	AT3G14670	-1.09	-0.12	-1.04	0.336242	0.47505	-6.68
247760_at	subtilisin-like serine protease conta	---	AT5G59130	-1.40	-0.49	-1.04	0.336322	0.475133	-6.68
245404_at	putative protein	---	AT4G17610	1.11	0.15	1.04	0.336397	0.475204	-6.68
267452_at	auxin response transcription factor	ETT	AT2G33860	-1.09	-0.13	-1.04	0.336414	0.475204	-6.68
249820_at	putative protein contains similarity t	---	AT5G23650	-1.14	-0.19	-1.03	0.336531	0.47529	-6.68
256937_at	hypothetical protein predicted by ge	---	AT3G22620	-1.75	-0.80	-1.03	0.336543	0.47529	-6.68
252437_at	putative protein pectinesterase hor	---	AT3G47380	1.26	0.34	1.03	0.336549	0.47529	-6.68
256830_at	eukaryotic translation initiation fact	TIF3C2	AT3G22860	-1.10	-0.14	-1.03	0.336574	0.47529	-6.68
251939_at	putative protein MICRONUCLEAR	---	AT3G53440	-1.13	-0.18	-1.03	0.336578	0.47529	-6.68
262335_at	hypothetical protein predicted by ge	SPO11-2	AT1G63990	-1.07	-0.09	-1.03	0.336789	0.47554	-6.68
263944_at	hypothetical protein predicted by ge	---	---	-1.07	-0.10	-1.03	0.336797	0.47554	-6.68
264809_at	superoxidase dismutase identical to	CSD1	AT1G08830	1.31	0.39	1.03	0.336825	0.47555	-6.68
250206_at	mitochondrial phosphate translocato	---	AT5G14040	1.17	0.22	1.03	0.336947	0.475692	-6.68
247809_at	unknown protein	---	AT5G58280	-1.09	-0.13	-1.03	0.33699	0.475724	-6.68
252632_s_a	disease resistance protein -like dis	RPP1	AT3G44480	-1.09	-0.13	-1.03	0.337166	0.475942	-6.68
246455_at	putative protein predicted protein F	---	AT5G16790	1.09	0.13	1.03	0.337203	0.475953	-6.68
261194_at	unknown protein contains Pfam pro	---	AT1G12770	1.06	0.09	1.03	0.337215	0.475953	-6.68
256019_at	cytochrome P-450, putative similar	---	AT1G58260	-1.08	-0.11	-1.03	0.337262	0.475971	-6.68
265790_at	putative amino acid or GABA perme	BAT1	AT2G01170	-1.12	-0.16	-1.03	0.33727	0.475971	-6.68
262873_at	hypothetical protein predicted by ge	---	AT1G64700	-1.09	-0.12	-1.03	0.337295	0.475977	-6.68
256832_at	meiotic recombination protein (AtD	DMC1	AT3G22880	-1.14	-0.19	-1.03	0.337346	0.476019	-6.68
256427_at	hypothetical protein predicted by ge	LBD21	AT3G11090	1.18	0.23	1.03	0.337414	0.476064	-6.68
258221_at	Snf1-related protein kinase KIN11 (	AKIN11	AT3G29160	1.16	0.22	1.03	0.337419	0.476064	-6.68
258028_at	hypothetical protein	---	AT3G27473	-1.06	-0.09	-1.03	0.337452	0.476081	-6.68

247117_at	3-hydroxyisobutyryl-coenzyme A hy	CHY1	AT5G65940	1.12	0.16	1.03	0.337494	0.476112	-6.68
253023_at	hypothetical protein	---	AT4G38070	-1.12	-0.16	-1.03	0.337572	0.476188	-6.68
256476_at	TNP2-like transposon protein simila	---	---	-1.08	-0.11	-1.03	0.337591	0.476188	-6.68
245343_at	hypothetical protein ;supported by f	---	AT4G15830	-1.07	-0.10	-1.03	0.33787	0.476547	-6.68
248131_at	unknown protein	---	AT5G54830	1.18	0.24	1.03	0.337887	0.476547	-6.68
256800_at	hypothetical protein predicted by ge	---	AT3G20900	-1.09	-0.13	-1.03	0.337955	0.476596	-6.68
258014_at	hypothetical protein predicted by ge	---	AT3G19330	1.13	0.18	1.03	0.337972	0.476596	-6.68
246281_at	hypothetical protein	NAPRT1	AT4G36940	-1.14	-0.19	-1.03	0.337984	0.476596	-6.68
265273_at	unknown protein	---	AT2G28320	1.09	0.13	1.03	0.338089	0.476703	-6.68
255438_at	putative oxidoreductase	AOP1	AT4G03070	-1.21	-0.27	-1.03	0.338102	0.476703	-6.68
254401_at	putative protein various predicted p	---	AT4G21300	1.08	0.11	1.03	0.33814	0.476728	-6.69
247583_at	putative protein predicted protein, S	---	AT5G60750	1.08	0.11	1.03	0.338161	0.476728	-6.69
262709_at	unknown protein ESTs gb|F15498,	SYP51	AT1G16240	1.10	0.14	1.03	0.338199	0.476752	-6.69
261874_at	ethylene responsive element bindin	ERF3	AT1G50640	1.10	0.14	1.03	0.338317	0.476838	-6.69
263088_at	putative replication protein A1	---	---	1.08	0.11	1.03	0.33833	0.476838	-6.69
249139_at	Cys2/His2-type zinc finger protein 3	AZF3	AT5G43170	1.20	0.26	1.03	0.338357	0.476838	-6.69
245135_at	putative non-LTR retroelement reve	---	---	-1.10	-0.13	-1.03	0.338358	0.476838	-6.69
253833_at	putative calcium binding protein tai	---	AT4G27790	-1.14	-0.19	-1.03	0.338364	0.476838	-6.69
256656_at	hypothetical protein similar to hypot	---	AT3G18900	-1.08	-0.11	-1.03	0.338439	0.476913	-6.69
254746_at	putative protein hypothetical protei	---	AT4G12980	-1.17	-0.23	-1.03	0.338597	0.477101	-6.69
247937_at	Ca2+-transporting ATPase-like prot	ACA8	AT5G57110	1.17	0.22	1.03	0.338631	0.477101	-6.69
252482_at	glucosyltransferase-like protein UD	UGT76E11	AT3G46670	-1.33	-0.41	-1.03	0.338635	0.477101	-6.69
250686_at	gamma-tubulin interacting protein-li	SPC98	AT5G06680	1.13	0.17	1.03	0.338751	0.477235	-6.69
247565_at	putative protein Atu, Drosophila me	VIP4	AT5G61150	1.08	0.11	1.03	0.338847	0.47734	-6.69
266019_at	putative calmodulin-binding protein	---	AT2G18750	1.07	0.10	1.03	0.338922	0.477417	-6.69
247710_at	receptor-like protein kinase recepto	---	AT5G59260	-1.07	-0.09	-1.03	0.339032	0.477489	-6.69
260842_at	19S proteosome subunit 9, putative	ATS9	AT1G29150	1.09	0.12	1.03	0.339034	0.477489	-6.69
264639_at	unknown protein similar to ubiquitin	UCH2	AT1G65650	1.10	0.14	1.03	0.339036	0.477489	-6.69
260613_at	unknown protein	---	AT1G53380	1.09	0.13	1.03	0.339193	0.477681	-6.69
256755_at	calmodulin, putative similar to GB:F	---	AT3G25600	-1.18	-0.23	-1.03	0.339315	0.477823	-6.69
248137_at	putative protein contains similarity t	---	AT5G54950	-1.09	-0.12	-1.03	0.33949	0.478026	-6.69
257488_s_a	putative RING-finger protein	---	AT1G36950	1.06	0.09	1.03	0.339501	0.478026	-6.69
253153_at	hypothetical protein	---	AT4G35700	-1.08	-0.11	-1.03	0.339535	0.478044	-6.69
257382_at	hypothetical protein predicted by ge	WRKY54	AT2G40750	-1.76	-0.81	-1.03	0.339589	0.47809	-6.69
248426_at	putative protein contains similarity t	---	AT5G51740	1.07	0.10	1.03	0.339712	0.478235	-6.69

257575_at	hypothetical protein similar to hypot	---	---	-1.09	-0.12	-1.03	0.339914	0.478489	-6.69
267069_at	unknown protein	ATCAMBP2	AT2G41010	-1.31	-0.39	-1.03	0.339951	0.478501	-6.69
267053_s_a	peroxidase	---	AT2G38380 ,	-1.19	-0.25	-1.03	0.339964	0.478501	-6.69
265810_at	unknown protein	---	AT2G18090	-1.14	-0.19	-1.03	0.340023	0.478535	-6.69
245353_at	hypothetical protein ;supported by f	---	AT4G16000	1.17	0.23	1.03	0.340031	0.478535	-6.69
249733_at	6-phosphogluconolactonase-like pr	emb2024	AT5G24400	1.08	0.12	1.03	0.340134	0.478624	-6.69
250631_at	pectin methyl-esterase-like protein	---	AT5G07430	-1.07	-0.10	-1.03	0.340136	0.478624	-6.69
258340_at	hypothetical protein predicted by ge	---	AT3G22770	-1.07	-0.10	-1.03	0.34018	0.478657	-6.69
257320_at	hypothetical protein	---	AT2G07749	1.10	0.13	1.03	0.340206	0.478663	-6.69
253687_at	putative protein ;supported by full-l	---	AT4G29520	1.46	0.54	1.03	0.340254	0.478692	-6.69
266402_at	hypothetical protein predicted by ge	---	AT2G38780	-1.14	-0.19	-1.03	0.340268	0.478692	-6.69
254698_at	NAM (no apical meristem) - like pro	anac071	AT4G17980	-1.06	-0.09	-1.03	0.340363	0.478796	-6.69
267451_at	putative AP2 domain transcription fa	---	AT2G33710	1.54	0.63	1.03	0.34042	0.478847	-6.69
249003_at	putative protein contains similarity t	---	AT5G44500	-1.09	-0.13	-1.03	0.340518	0.478954	-6.69
246784_at	signal peptidase ; supported by cD	---	AT5G27430	1.07	0.10	1.03	0.340613	0.479059	-6.69
250605_at	glycine/proline-rich protein flagellifo	---	AT5G07570	-1.09	-0.13	-1.03	0.340776	0.479259	-6.69
251135_at	putative protein proteophosphoglyc	---	AT5G01280	1.08	0.11	1.02	0.340819	0.479261	-6.69
258306_at	putative transcription factor similar t	ATBZIP42	AT3G30530	-1.06	-0.09	-1.02	0.34082	0.479261	-6.69
244918_at	hypothetical protein	AT2G0774	---	-1.09	-0.12	-1.02	0.340905	0.479351	-6.69
252791_at	transposase - like protein putative t	---	---	1.08	0.10	1.02	0.341008	0.479467	-6.69
261260_at	hypothetical protein predicted by ge	---	AT1G26680	-1.08	-0.11	-1.02	0.341032	0.479471	-6.69
259190_at	unknown protein est hit, predicted l	TPLATE	AT3G01780	-1.12	-0.16	-1.02	0.341106	0.479546	-6.69
250763_at	short chain alcohol dehydrogenase-	---	AT5G06060	-1.10	-0.13	-1.02	0.341164	0.479598	-6.69
266103_at	putative VAMP-associated protein	PVA12	AT2G45140	-1.09	-0.12	-1.02	0.341259	0.479702	-6.69
264419_s_a	hypothetical protein contains similar	---	AT1G43310 ,	1.24	0.31	1.02	0.341298	0.479728	-6.69
263387_at	unknown protein ; supported by cD	---	AT2G40160	-1.09	-0.13	-1.02	0.34133	0.479743	-6.69
265822_at	putative SEC1 family transport prot	ATSLY1	AT2G17980	-1.08	-0.12	-1.02	0.341402	0.479814	-6.69
246466_at	sugar transporter - like protein D-X	---	AT5G17010	1.13	0.17	1.02	0.341451	0.479842	-6.69
249760_at	(+)-delta-cadinene synthase (d-cad	TPS21	AT5G23960	-1.07	-0.10	-1.02	0.341464	0.479842	-6.69
263095_at	Ac-like transposase related to Ac/D	---	AT2G16040	-1.08	-0.10	-1.02	0.341492	0.479852	-6.69
265435_s_a	putative major intrinsic (channel) pr	---	AT1G31885 ,	-1.11	-0.15	-1.02	0.341633	0.480021	-6.69
246271_at	photosystem II oxygen-evolving cor	---	AT4G37230	-1.09	-0.12	-1.02	0.341727	0.480123	-6.69
254544_at	putative chitinase chitinase (EC 3.2	---	AT4G19820	-1.10	-0.14	-1.02	0.341785	0.480136	-6.69
255098_at	hypothetical protein	---	AT4G08640	-1.07	-0.09	-1.02	0.341785	0.480136	-6.69
258969_at	hypothetical protein predicted by ge	---	AT3G10680	-1.07	-0.10	-1.02	0.341819	0.480136	-6.69

254412_at	putative protein chloroplast DNA-bi	B160	AT4G21430	-1.14	-0.19	-1.02	0.341821	0.480136	-6.69
265180_at	putative OBP32pep protein ;suppo	---	AT1G23590	-1.06	-0.09	-1.02	0.341913	0.480236	-6.69
257557_at	terpene synthase-related protein, p	---	AT3G14490	-1.11	-0.15	-1.02	0.341947	0.480254	-6.69
245163_at	putative flavin-containing monooxyg	---	AT2G33230	-1.11	-0.15	-1.02	0.341989	0.480283	-6.69
246242_at	putative protein	---	AT4G36600	-1.12	-0.17	-1.02	0.342022	0.480301	-6.69
253355_at	putative protein	---	AT4G33380	1.07	0.10	1.02	0.342195	0.480514	-6.69
248539_at	ribitol dehydrogenase-like ; suppor	---	AT5G50130	1.11	0.15	1.02	0.34233	0.480674	-6.69
256134_at	aminoalcoholphosphotransferase id	AAPT1	AT1G13560	-1.08	-0.11	-1.02	0.342512	0.480899	-6.70
266928_at	putative phosphomannomutase ;su	PMM	AT2G45790	-1.12	-0.17	-1.02	0.342604	0.481	-6.70
256500_at	Ty1/copia-element polyprotein	---	---	-1.09	-0.12	-1.02	0.342626	0.481	-6.70
247673_at	tRNA intron endonuclease - like pr	SEN2	AT5G60230	-1.07	-0.09	-1.02	0.342715	0.481083	-6.70
256247_at	SRF-type transcription factor contai	AGL91	AT3G66656	-1.07	-0.10	-1.02	0.342727	0.481083	-6.70
266954_at	hypothetical protein predicted by ge	---	AT2G34530	-1.08	-0.10	-1.02	0.342779	0.481127	-6.70
252011_at	carbonic anhydrase (CAH1) ; supp	ACA1	AT3G52720	-1.12	-0.17	-1.02	0.34281	0.48114	-6.70
253887_at	putative protein isp4 protein, Schiz	OPT6	AT4G27730	-1.22	-0.28	-1.02	0.342893	0.481227	-6.70
251063_at	protein kinase ATN1-like protein pr	---	AT5G01850	1.08	0.12	1.02	0.342946	0.481233	-6.70
255405_s_a	hypothetical protein similar to A. tha	---	AT4G03440 ,	-1.11	-0.15	-1.02	0.342964	0.481233	-6.70
262905_at	thioredoxin, putative similar to thior	ATH7	AT1G59730	-1.07	-0.10	-1.02	0.343002	0.481233	-6.70
264159_at	unknown protein ;supported by full-	---	AT1G65270	1.07	0.10	1.02	0.343002	0.481233	-6.70
264187_at	unknown protein ;supported by full-	---	AT1G54860	-1.07	-0.09	-1.02	0.343003	0.481233	-6.70
251577_at	putative protein several hypothetica	---	AT3G58350	-1.11	-0.15	-1.02	0.343027	0.481237	-6.70
265971_at	putative retroelement pol polyprotei	---	---	-1.08	-0.11	-1.02	0.343209	0.481463	-6.70
249474_s_a	germin-like protein (GLP2a) copy2	GER2	AT5G39130 ,	1.10	0.14	1.02	0.34339	0.481688	-6.70
255562_at	G/T DNA mismatch repair enzyme s	MSH6	AT4G02070	1.11	0.15	1.02	0.343413	0.48169	-6.70
254385_s_a	putative protein CGI-131 protein, H	ATMSRB7	AT4G21830 ,	1.95	0.97	1.02	0.343652	0.481996	-6.70
245828_at	transcription factor, putative similar	VIM1	AT1G57820	-1.10	-0.14	-1.02	0.343867	0.482269	-6.70
259101_at	hypothetical protein predicted by ge	---	AT3G11640	-1.07	-0.10	-1.02	0.344083	0.482526	-6.70
245988_at	putative protein predicted protein, F	---	AT5G20610	1.11	0.16	1.02	0.344093	0.482526	-6.70
257069_at	hypothetical protein predicted by ge	---	AT3G28170	-1.08	-0.11	-1.02	0.344161	0.482591	-6.70
248648_at	putative protein similar to unknown	---	AT5G49220	1.09	0.13	1.02	0.344346	0.482821	-6.70
259449_at	hypothetical protein similar to putati	---	AT1G13800	1.10	0.14	1.02	0.344369	0.482824	-6.70
256821_at	hypothetical protein predicted by ge	---	AT3G22180	1.13	0.18	1.02	0.344414	0.482857	-6.70
250219_at	putative protein similar to unknown	MPL1	AT5G14180	1.22	0.28	1.02	0.344494	0.48294	-6.70
251438_s_a	putative protein hypothetical protei	---	AT3G59930 ,	1.53	0.61	1.02	0.344534	0.482966	-6.70
264081_at	putative RING zinc finger transcripti	---	AT2G28540	1.10	0.14	1.02	0.34462	0.483029	-6.70

257321_at	hypothetical protein	---	---	1.17	0.23	1.02	0.344638	0.483029	-6.70
249976_at	Serine/arginine rich protein - like S	SCL28	AT5G18810	-1.09	-0.12	-1.02	0.344643	0.483029	-6.70
249407_at	putative protein predicted proteins,	---	AT5G40320	-1.08	-0.10	-1.02	0.344667	0.483034	-6.70
251133_at	LAX1 / AUX1 -like permease	---	AT5G01240	1.13	0.18	1.02	0.344719	0.483076	-6.70
247903_at	unknown protein ; supported by cD	---	AT5G57340	1.11	0.15	1.02	0.344842	0.48322	-6.70
265288_at	putative glutaredoxin ;supported by	---	AT2G20270	-1.11	-0.15	-1.02	0.344949	0.483339	-6.70
249429_at	putative protein	---	AT5G39880	-1.06	-0.09	-1.02	0.345163	0.48361	-6.70
258261_at	putative RING zinc finger protein c	---	AT3G15740	1.15	0.21	1.01	0.345327	0.48381	-6.70
248368_at	mandelonitrile lyase-like protein ; s	---	AT5G51950	-1.08	-0.11	-1.01	0.345353	0.483816	-6.70
260778_at	splicing factor, putative similar to h	---	AT1G14640	-1.11	-0.15	-1.01	0.345388	0.483836	-6.70
258572_at	hypothetical protein	---	AT3G04270	1.07	0.09	1.01	0.34543	0.483865	-6.70
258903_at	hypothetical protein similar to zinc	---	AT3G06410	1.13	0.18	1.01	0.345583	0.48405	-6.70
250236_at	delta subunit of mitochondrial F1-A	---	AT5G13450	1.10	0.13	1.01	0.345609	0.484056	-6.70
263498_at	unknown protein ; supported by cD	LSH10	AT2G42610	-1.47	-0.56	-1.01	0.345685	0.484133	-6.70
264193_at	CRK1 protein, putative similar to C	---	AT1G54610	1.07	0.10	1.01	0.345761	0.48421	-6.70
261371_at	ubiquitin-conjugating enzyme, puta	UBC26	AT1G53020	-1.07	-0.10	-1.01	0.345914	0.484394	-6.70
258185_at	hypothetical protein similar to hypot	---	AT3G21580	-1.11	-0.15	-1.01	0.345941	0.484403	-6.70
265082_at	hypothetical protein predicted by ge	---	AT1G03830	1.08	0.11	1.01	0.346035	0.484504	-6.70
245013_at	ATPase epsilon subunit	---	---	-1.07	-0.09	-1.01	0.346162	0.484652	-6.70
253933_at	hypothetical protein	---	AT4G26820	-1.10	-0.13	-1.01	0.34622	0.484704	-6.70
251829_at	putative protein KIAA0882 protein,	---	AT3G55020	-1.20	-0.26	-1.01	0.346677	0.485314	-6.70
248801_at	homeobox-leucine zipper protein-lik	HAT2	AT5G47370	-1.28	-0.35	-1.01	0.346766	0.485396	-6.70
262881_at	unknown protein ; supported by cD	---	AT1G64890	-1.16	-0.21	-1.01	0.346778	0.485396	-6.70
262420_at	hypothetical protein contains simila	TAF15	AT1G50300	-1.07	-0.09	-1.01	0.346862	0.485484	-6.71
258220_at	DnaJ, putative similar to DnaJ GB:	---	AT3G17830	1.12	0.16	1.01	0.346933	0.485554	-6.71
254217_at	putative protein other hypothetical	---	AT4G23720	-1.07	-0.10	-1.01	0.347055	0.485694	-6.71
265704_at	hypothetical protein predicted by ge	---	AT2G03420	-1.13	-0.17	-1.01	0.347087	0.485709	-6.71
248695_at	putative protein similar to unknown	---	AT5G48350	-1.12	-0.17	-1.01	0.347193	0.485828	-6.71
266360_at	Mutator-like transposase similar to	FRS2	AT2G32250	-1.19	-0.25	-1.01	0.347246	0.485852	-6.71
250204_at	leucine zipper protein-like	ATEXO70C	AT5G13990	-1.09	-0.13	-1.01	0.347253	0.485852	-6.71
249815_at	60S ribosomal protein L13 ; suppo	---	AT5G23900	1.13	0.18	1.01	0.347569	0.486263	-6.71
260782_at	unknown protein ; supported by cD	---	AT1G14570	1.07	0.10	1.01	0.34768	0.486389	-6.71
254896_at	MADS-box protein AGL14	AGL14	AT4G11880	-1.07	-0.10	-1.01	0.347761	0.486449	-6.71
259153_at	unknown protein ;supported by full-	---	AT3G10250	1.10	0.14	1.01	0.347765	0.486449	-6.71
256805_at	unknown protein contains similarity	---	AT3G20930	1.19	0.25	1.01	0.347841	0.486507	-6.71

253737_at	Expressed protein ; supported by fu	---	AT4G28703	1.26	0.34	1.01	0.34785	0.486507	-6.71
247888_at	phytocyanin/early nodulin-like prote	---	AT5G57920	1.09	0.12	1.01	0.347895	0.486514	-6.71
246532_at	putative protein beta-glucan-elicitor	---	AT5G15870	-1.38	-0.47	-1.01	0.347897	0.486514	-6.71
246980_at	peptidylprolyl isomerase (cyclophilin	---	AT5G67530	1.08	0.11	1.01	0.347993	0.486619	-6.71
252209_at	putative protein RXF26, Arabidops	---	AT3G50400	-1.16	-0.22	-1.01	0.348063	0.486686	-6.71
262507_at	receptor-like protein kinase, putativ	---	AT1G11330	-1.10	-0.14	-1.01	0.348089	0.486693	-6.71
251694_s_a	putative protein NAM (no apical me	anac063	AT3G55210	-1.07	-0.10	-1.01	0.348175	0.486783	-6.71
248168_at	beta-glucosidase	BGLU41	AT5G54570	-1.09	-0.12	-1.01	0.348238	0.486818	-6.71
247096_at	S-adenosyl-L-methionine:salicylic a	---	AT5G66430	1.06	0.09	1.01	0.348243	0.486818	-6.71
260233_at	putative cytochrome P450 similar to	CYP98A9	AT1G74550	-1.14	-0.19	-1.01	0.348308	0.48688	-6.71
255189_at	hypothetical protein	---	AT4G07350	-1.07	-0.10	-1.01	0.348407	0.486988	-6.71
257710_at	unknown protein ;supported by full-	---	AT3G27350	1.09	0.13	1.01	0.348446	0.487013	-6.71
246102_at	putative protein various predicted p	---	AT5G32590	-1.07	-0.10	-1.01	0.348605	0.487205	-6.71
245846_at	P-type transporting ATPase, putati	---	AT1G26130	1.11	0.15	1.01	0.348663	0.487239	-6.71
263261_at	putative pre-mRNA splicing factor s	---	AT1G10580	1.06	0.09	1.01	0.348699	0.487239	-6.71
248018_at	putative protein strong similarity to u	---	AT5G56470	-1.10	-0.14	-1.01	0.348707	0.487239	-6.71
266174_at	hypothetical protein predicted by ge	---	AT2G02440	-1.06	-0.09	-1.01	0.348714	0.487239	-6.71
247452_at	H-protein promoter binding factor-lik	CDF1	AT5G62430	-1.13	-0.18	-1.01	0.348852	0.487353	-6.71
249184_at	receptor kinase-like protein	---	AT5G43020	-1.14	-0.19	-1.01	0.348856	0.487353	-6.71
256391_at	hypothetical protein predicted by ge	---	AT3G06090	-1.06	-0.09	-1.01	0.34886	0.487353	-6.71
256022_at	amino acid permease I identical to a	AAP1	AT1G58360	-1.24	-0.31	-1.01	0.348933	0.487425	-6.71
259050_at	hypothetical protein similar to GB:A	---	AT3G03356	-1.06	-0.09	-1.01	0.348961	0.487434	-6.71
262788_at	unknown protein ESTs gb|T75618 a	---	AT1G10690	1.12	0.16	1.01	0.349066	0.487551	-6.71
263366_at	unknown protein	---	AT2G20440	-1.09	-0.13	-1.01	0.349177	0.487677	-6.71
247948_at	putative protein strong similarity to u	---	AT5G57130	-1.10	-0.14	-1.01	0.349404	0.487963	-6.71
258150_at	hypothetical protein ;supported by f	---	AT3G18160	1.17	0.23	1.01	0.349578	0.488177	-6.71
260870_at	GTP-binding protein(RAB1Y), puta	ATRAB18	AT1G43890	-1.10	-0.13	-1.00	0.349707	0.488326	-6.71
[truncated: 567,648 more chars]
